# Risk of major depressive increases with increasing frequency of alcohol drinking: a bidirectional two-sample Mendelian randomization analysis

**DOI:** 10.3389/fpubh.2024.1372758

**Published:** 2024-06-05

**Authors:** Weiyu Feng, Bing Zhang, Pengyu Duan, Yong-hong Bi, Zhehao Jin, Xiaoyan Li, Xiangcheng Zhao, Kun Zuo

**Affiliations:** Department of Anesthesiology, The Key Laboratory of Anesthesiology and Intensive Care Research of Heilongjiang Province, Second Affiliated Hospital of Harbin Medical University, Harbin Medical University, Harbin, China

**Keywords:** major depression (MDD), alcohol consumer, alcohol intake frequency, fat percentage, BMI

## Abstract

**Introduction:**

A growing body of evidence suggests that alcohol use disorders coexist with depression. However, the causal relationship between alcohol consumption and depression remains a topic of controversy.

**Methods:**

We conducted a two-sample two-way Mendelian randomization analysis using genetic variants associated with alcohol use and major depressive disorder from a genome-wide association study.

**Results:**

Our research indicates that drinking alcohol can reduce the risk of major depression (odds ratio: 0.71, 95% confidence interval: 0.54~0.93, p = 0.01), while increasing the frequency of drinking can increase the risk of major depression (odds ratio: 1.09, 95% confidence interval: 1.00~1.18, p = 0.04). Furthermore, our multivariate MR analysis demonstrated that even after accounting for different types of drinking, the promoting effect of drinking frequency on the likelihood of developing major depression still persists (odds ratio: 1.13, 95% confidence interval: 1.04~1.23, p = 0.005). Additionally, mediation analysis using a two-step MR approach revealed that this effect is partially mediated by the adiposity index, with a mediated proportion of 37.5% (95% confidence interval: 0.22 to 0.38).

**Discussion:**

In this study, we found that alcohol consumption can alleviate major depression, while alcohol intake frequency can aggravate it.These findings have important implications for the development of prevention and intervention strategies targeting alcohol-related depression.

## Introduction

Depression is a widespread mental disorder that impacts individuals globally and has emerged as the sixth primary burden of disease on a global scale (1, 2). The prevalence of depression has escalated over time, with a 50% surge in worldwide instances recorded between 1990 and 2017 (3). Consequently, comprehending the underlying causes of depression is imperative for its prevention. Extensive evidence has verified that several risk factors are associated with depression, encompassing family history, stress, and specific social elements (4). Alcohol, renowned for fostering social interaction, sexual conduct, and stress alleviation across the globe (5), is also employed as a self-treatment remedy for patients enduring inherent or secondary mental disorders (6). Consequently, investigating the correspondence between alcohol consumption and depression remains a captivating subject (7).

Drinking and depression frequently coexist, and the connection between the two is intricate. The precise causal relationship between them remains uncertain (8). Some investigations have suggested that alcohol dependence is linked to an elevated risk of depression. For instance, a study of 3,967 adolescents in an ongoing cohort discovered that consuming alcohol was an autonomous factor contributing to depression (9). Another cohort study based on the US adult population also disclosed that alcohol use disorder substantially heightens the chance of subsequent depression (10). These investigations propose that alcohol use disorder can instigate depression. In contrast, there are also studies indicating that depression can result in increased alcohol consumption (11). Turner et al. (12) conducted a comprehensive review from 1997 to 2018 encompassing self-medication, mood disorders, and anxiety, and determined that mood disorders and anxiety elevate the risk of substance use disorders. Additionally, Jin-Seok Lee et al. (13) verified in mice that depression attributable to social isolation can be intensified by neuroinflammation caused by microglia, leading to augmented alcohol intake. Hence, it is evident that the emergence of alcohol use disorder and depression is a reciprocal and reinforcing relationship. On the contrary, certain investigations have displayed that moderate drinking might diminish the likelihood of depression (10, 14, 15). Alcohol has been found to normalize the sphingomyelin and monoamine functions of the nucleus accumbens in depressed mice, thereby alleviating depressive behavior (16). Furthermore, other studies have substantiated that drinking or excessive drinking does not amplify the risk of depression (17). Therefore, additional research is required to ascertain the causal association between drinking and depression, as well as comprehend the bidirectional nature of this connection.

To confirm the reliability of RCT study results in terms of causal inference, additional research methods are necessary due to the time-consuming nature of RCT studies (18). Mendelian randomization research is a particular approach that aims to infer potential causal associations between exposure factors and outcomes by utilizing genetic variation as an instrumental variable (19). This research method leverages the fact that genetic variation is randomly assigned during meiosis and fertilization, making it unaffected by self-selection and predetermined before the onset of diseases. Consequently, this minimizes the impact of confounding factors and issues related to reverse causation (20). To evaluate the possible causal relationship between depression and drinking (including frequency and type), a two-sample Mendelian randomization analysis was performed in this particular investigation. Furthermore, a two-step Mendelian randomization analysis was employed to explore the potential mediator between depression and drinking.

## Methods

### Two-sample study design

The study utilized two-sample Mendelian randomization (MR) to analyze the relationship between alcohol consumption (frequency and type) and major depression. The researchers conducted univariate and multivariate analyses using summary genetic data from GWAS and UK Biobank. Mendelian randomization is a method that leverages genetic variation to estimate the causal effects of risk factors on disease outcomes. To ensure the validity of MR studies, three assumptions must be met: (1) genetic variants are associated with risk factors (correlation assumption), (2) there are no confounding factors influencing the associations between genetic variants and outcomes (independence assumption), and (3) the restrictive assumption is excluded (21).

### Date sources

Severe depression data were obtained from the depression meta-analysis conducted by Howard et al. (22), which examined various depression phenotypes in participants from 23andMe, PGC, and UK Biobank, including 170,756 patients with depression and 329,443 controls. However, participant data from 23andMe were not included in the publicly available data. The alcohol consumption data were sourced from the GWAS study conducted by Clarke et al. (23), which analyzed the weekly and monthly drinking volume as well as drinking type of 112,117 participants from UK Biobank. Other data, such as drinking frequency and drinking type, were extracted from published GWAS studies from UK Biobank. Additionally, there are several risk factors associated with alcohol consumption and depression, including inflammation levels [inflammatory biomarkers IL-6 (24) and C-reactive protein (25)], acid sphingomyelinase (16), body mass index (26), body fat percentage (BFP) (27), and diet-related metabolites (28). These data were obtained from the IEU Open GWAS database summary website.^1^ The study aimed to assess whether these factors mediate the causal relationship between alcohol use and major depression. The relevant data are organized in Supplementary Table 1.

### Selection of the genetic instrumental variables

To investigate the causal relationship between alcohol consumption and major depression, we conducted a study using genome-wide association studies (GWAS). We identified a total of 450 SNPs for alcohol consumption, 8,460 SNPs for alcohol intake frequency, and 4,606 SNPs for major depression that reached genome-wide significance (*p* < 5 × 10^−8^; Supplementary Data Sheet 1). To ensure accuracy, we used linkage disequilibrium statistics (LD) to screen for significant SNPs and excluded any overlap between genetic sites (r^2^ < 0.001, kb = 10,000). Additionally, we accounted for potential confounding factors by examining each SNP in the PhenoScanner GWAS database (29, 30) and eliminating SNPs that were influenced by factors such as smoking, anxiety, mental stress, and pain.

### Testing instrument strength and statistical power

Instrument strength is determined by the magnitude and precision of the association of genetic instruments with risk factors, which is represented by the *F* value. The F value is calculated based on the proportion of variance in the phenotype (R^2^), sample size (N), and number of instruments (k), using the formula F = R^2^ (N − k − 1)/k (1 − R^2^) (31). R_i_^2^ for instrument i can be calculated using the approximation R_i_^2^ = 2 × EAF_i_ × (1 − EAF_i_) × β_i_^2^, where EAF_i_ represents the effect allele frequency and β_i_ is the estimated genetic effect of exposure (32). An F statistic ≥10 indicates that the risk of incorporating weak correlation instrument bias in the MR analysis is relatively low (33).

### Statistical analyses

#### Univariate Mendelian randomization analysis

Univariate Mendelian randomization analysis was conducted using the inverse-variance weighted (IVW) method under a multiplicative random effects model (31). This method combines Wald ratio estimates for each SNP into a single causal estimate for each risk factor, where each estimate is obtained by dividing the SNP-outcome association by the SNP-exposure association (33). To address potential bias introduced by pleiotropic instrumental variables, sensitivity analysis was performed to resolve heterogeneity in causal estimates. In fixed effects variance weighted analysis, Cochran’s Q value was calculated to quantify the heterogeneity produced by different genetic variants, with *p* ≤ 0.05 indicating the presence of heterogeneity (34, 35). If heterogeneity was detected, a random-effects IVW MR analysis was employed. MR-Egger regression, based on the intercept term, was used to evaluate the presence of horizontal pleiotropy, with pleiotropy bias considered to exist when the deviation *p* < 0.05 (36). Additionally, sensitivity analyses were conducted using the Weighted median (37), Weighted mode (38), MR-Egger regression (39), and Simple mode methods. Briefly, the weighted median method estimates the causal effect based on the median of the weighted empirical density function of individual SNP effect estimates. This method remains applicable even in cases of horizontal pleiotropy and partial violation of the Mendelian randomization assumption (37). The weighted mode method clusters SNPs based on the similarity of causal effects and estimates causal effects based on the cluster with the largest number of SNPs, thereby providing unbiased estimates (38). Additionally, we used MR-PRESSO to assess the presence of outlier SNPs. MR-PRESSO compares the distance of all genetic instruments to the regression line (sum of squared residuals) to the distance expected under the null hypothesis of no horizontal pleiotropy (40). Furthermore, we performed a leave-one-out SNP analysis to evaluate the impact of individual variants on the observed causal effects (41).

#### Multivariate Mendelian randomization analysis

In order to investigate the relationship between drinking and severe depression, we conducted a multivariate Mendelian randomization analysis (42) that considered the frequency and type of drinking. This analysis allowed us to simultaneously estimate the effects of drinking frequency and the coexistence of different types of alcohol (beer/cider, fortified wine, red wine, white wine/champagne, spirits, other alcohol) on depression severity. To ensure the accuracy of our results, we used genetic tools from relevant GWAS and employed linkage disequilibrium detection (r^2^ = 0.001, kb = 10,000) to prevent SNP overlap. Additionally, we utilized LASSO analysis to eliminate exposure factors with collinearity.

#### Mediation analysis

Mediation analysis was conducted to evaluate the causal effects of potential mediator exposures on major depression. Firstly, genetic tools were used to estimate the effects of alcohol use and alcohol frequency on the mediators. Secondly, genetic tools for the identified mediators were used to assess their causal effects on major depression. The “coefficient product” method (43) was employed to determine the indirect effect of drinking and drinking frequency on the risk of major depression through each potential mediator, if evidence supported the influence of these factors. Standard errors for indirect effects were obtained using the delta method (44).

## Results

### Univariate Mendelian randomization analysis

**Figure 1:**
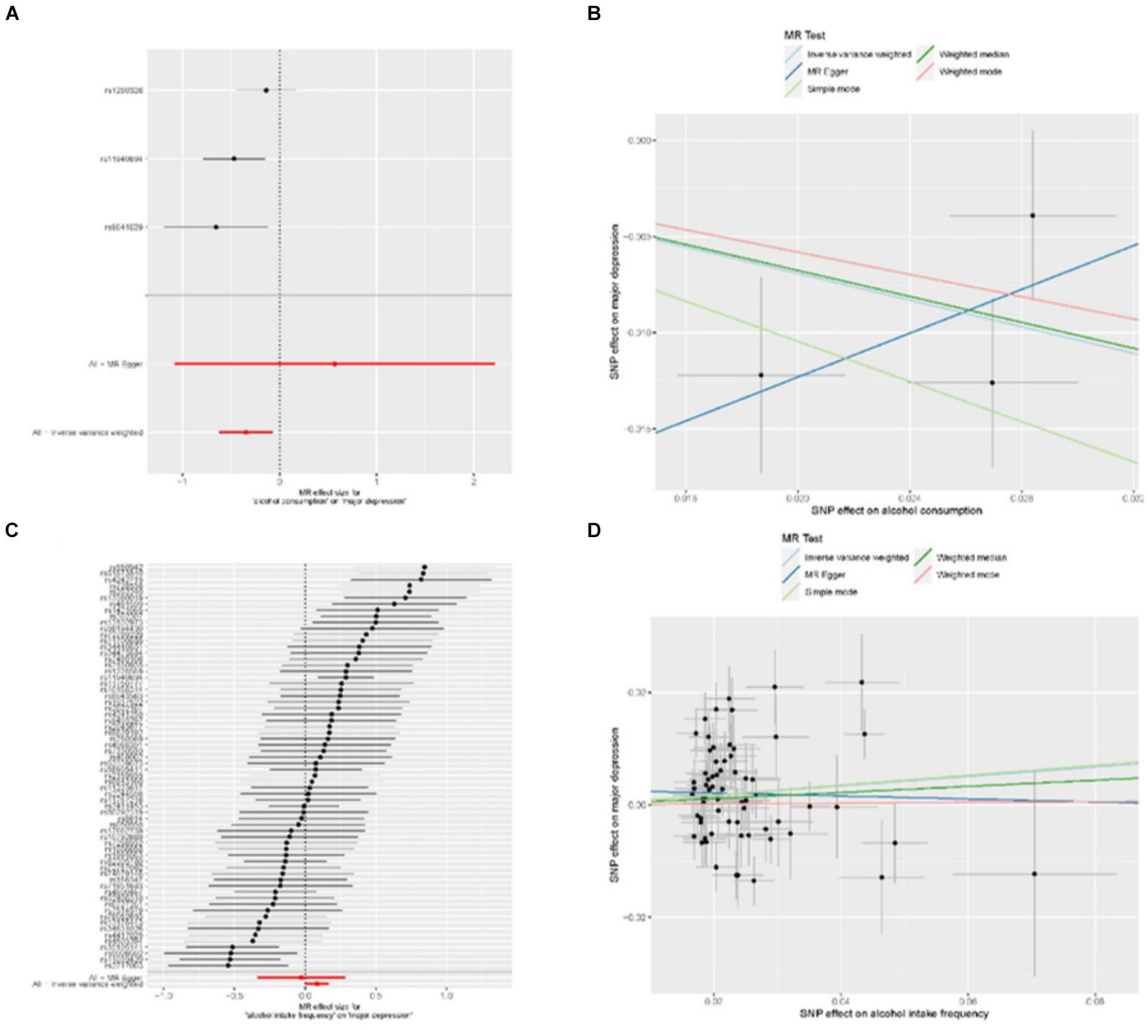
MR results for the relationship between alcohol and major depression. **(A)** forest plot of individual and combined SNP MR-estimated effect sizes. The effect estimates represent the log odds for major depression increase in alcohol consumption, and the error bars represent 95% CIs. **(B)** Scatter plot of SNP effects on relative alcohol consumption vs. major depression, with the slope of each line corresponding to the estimated MR effect per method. The data are expressed as raw β values with 95% CIs. **(C)** Forest plot of individual and combined SNP MR-estimated effect sizes, that is alcohol intake frequency and major depression. **(D)** Scatter plot of SNP effects on relative alcohol intake frequency vs. major depression.

To investigate the correlation between alcohol intake and major depressive disorder, we performed a two-sample Mendelian randomization analysis. The analysis included all SNPs listed in Supplementary Data Sheet 2. Our univariate MR analysis revealed a causal relationship, indicating that alcohol consumption is a protective factor for major depression. The IVW odds ratio (OR) was 0.71 with a 95% confidence interval (CI) of 0.54 to 0.93, and a *p-*value of 0.01 (Table 1). The absence of weak correlation bias was supported by the *F* value (Table 1), and heterogeneity was not detected according to Cochran’s Q value (Q = 3.63, *p* = 0.16; Supplementary Data Sheet 3). No potential outliers were identified by MR-PRESSO. Additionally, MR-Egger intercept analysis provided no evidence of directional pleiotropy (*p* = 0.47; Supplementary Data Sheet 3). The weighted median analysis yielded consistent results with the IVW method (OR = 0.71; 95% CI 0.56 to 0.91; *p* = 0.01), further supporting the protective effect of alcohol consumption on the risk of major depression. The forest plot in Figure 1A displays the estimates of the effect of SNPs associated with alcohol consumption on the risk of major depression. Additionally, displayed in Figure 1B is a scatterplot which showcases the correlation amid the consumption of alcohol and the likelihood of encountering significant depression. The slopes of various regression studies are denoted by distinctively colored lines. A negative causal relationship exists between the frequency of alcohol intake and major depression. As the frequency of drinking increases, the probability of suffering from major depression also increases. The IVW OR value is 1.09 with a 95% CI of 1.00 to 1.18, and a *p* value of 0.04 (Table 1). The *F* value indicates no presence of weak correlation bias (Table 1). The inclusion of SNPs shows heterogeneity, as indicated by Cochran’s Q value of 160 and a *p* value of 4.41 × 10^−10^ (Supplementary Data Sheet 4). However, the IVW method is not affected by heterogeneity, ensuring the credibility of the results. MR-PRESSO analysis did not detect any outliers. Additionally, MR-Egger intercept analysis found no evidence of directional pleiotropy with a *p* value of 0.47 (Supplementary Data Sheet 4). The forest plot in Figure 1C presents the estimates of the effect of SNPs associated with alcohol intake frequency on the risk of major depression. Furthermore, Figure 1D shows a scatter plot illustrating the association between alcohol intake frequency and the risk of major depression.

**Table 1:** MR results for the relationship between alcohol and depression.

Method	Number of SNPs	F	OR (95%CI)	*P*
**Alcohol consumption on major depression**				
IVW	3	96	0.71 (0.54–0.93)	0.01
Weighted median			0.71 (0.56–0.91)	0.01
Weighted mode			0.75 (0.52–1.08)	0.26
Simple mode			0.59 (0.38–0.91)	0.14
MR-Egger			1.77 (0.34–9.22)	0.62
**Alcohol intake frequency on major depression**				
IVW	66	6,140	1.09 (1.00–1.18)	0.04
Weighted median			1.06 (0.96–1.16)	0.24
Weighted mode			1.00 (0.80–1.27)	0.95
Simple mode			1.09 (0.85–1.40)	0.50
MR-Egger			0.97 (0.72–1.32)	0.86

Regarding the protective effect of alcohol consumption on major depression, we investigated the relationship between different types of drinking and this effect. We conducted separate single-factor Mendelian analyses to examine the effects of intake of beer/cider, fortified wine, red wine, white wine/champagne, spirits, and other alcohol on major depression. However, we found that there were too few strongly correlated SNPs when extracting SNPs, so we adjusted the *p*-value to 5 × 10^−6^ (Supplementary Data Sheet 5). The included SNPs can be found in Supplementary Data Sheet 6. The results obtained using the IVW method indicated that there was no significant causal relationship between the type of drinking and the occurrence of major depression. OR values and corresponding 95% CI for each type of alcohol were as follows: beer/cider (OR value 1.00, 95% CI 0.91~1.09, *p* = 0.95), fortified wine (OR value 0.99, 95% CI 0.76~1.28, *p* = 0.93), red wine (OR value 0.97, 95% CI 0.88~1.11, *p* = 0.81), white wine/champagne (OR value 0.98, 95% CI 0.86~1.11, *p* = 0.73), spirits (OR value 1.13, 95% CI 1.00~1.29, *p* = 0.06), and other alcohol (OR value 1.02, 95% CI 0.82~1.28, *p* = 0.84; Supplementary Table 2).

### Multivariate Mendelian randomization analysis

To investigate the effects of alcohol consumption and alcohol intake frequency on major depression, we conducted a multivariate Mendelian randomization analysis. We specifically examined the affect of different types of alcohol on major depression. The findings from our analysis revealed that when considering multiple types of drinking, alcohol consumption no longer showed a causal effect on major depression (OR value 1.05, 95% CI 0.79~1.39, *p* = 0.76). However, alcohol intake frequency remained a significant contributing factor for major depression (OR value 1.13, 95% CI 1.04~1.23, *p* = 0.005), as presented in Table 2.

**Table 2:** Multivariable MR analysis estimating the effect of relative alcohol on MDD, conditioning on different alcohol.

Method	Number of SNPs	OR (95%CI)	*P*
**Alcohol consumption**			
IVW	4	1.05 (0.79–1.39)	0.76
**Alcohol intake frequency**			
IVW	79	1.13 (1.04–1.23)	0.51 × 10^−2^

### Causal inference of major depression on alcohol consumption and alcohol intake frequency

In order to examine the potential causal effect of major depression on alcohol consumption and alcohol intake frequency, we used major depression as an exposure factor and performed a univariate Mendelian randomization analysis. The SNPs used in the analysis are shown in Supplementary Data Sheet 7. The results of the analysis are presented in Table 3. Our findings indicate that major depression does not have a causal effect on alcohol consumption (OR value 1.00, 95% CI 0.96 ~ 1.03, *p* = 0.82). Regarding the causal analysis of major depression on alcohol intake frequency, the weighted median method suggests that major depression is involved with an increase in alcohol intake frequency (OR value 1.09, 95% CI 1.02~1.17, *p* = 0.007). However, according to the IVW method, major depression does not have an effect on alcohol intake frequency (OR value 1.08, 95% CI 1.00~1.16, *p* = 0.07). It is important to consider that the data analyzed in this study exhibits strong heterogeneity (Cochran’s Q value is 38, *p* = 6.93 × 10^−21^; Supplementary Data Sheet 8). Therefore, it is concluded that major depression does not have a causal relationship with alcohol intake frequency.

**Table 3:** Bidirectional MR results for the relationship between major depression and alcohol.

Method	Number of SNPs	F	OR (95%CI)	P
**Major depression on alcohol consumption**				
IVW	42	7,401	1.00 (0.96−1.03)	0.81
Weighted median			0.99 (0.94−1.03)	0.56
Weighted mode			0.95 (0.88−1.03)	0.25
Simple mode			0.93 (0.83−1.03)	0.17
MR-Egger			1.02 (0.89−1.18)	0.74
**Major depression on alcohol intake frequency**				
IVW	39	6,860	1.08 (1.00−1.16)	0.07
Weighted median			1.09 (1.02−1.17)	0.007
Weighted mode			1.11 (0.97−1.28)	0.14
Simple mode			1.12 (0.96−1.31)	0.15
MR-Egger			0.96 (0.65−1.44)	0.86

### Mediation analysis

**Figure 2:**
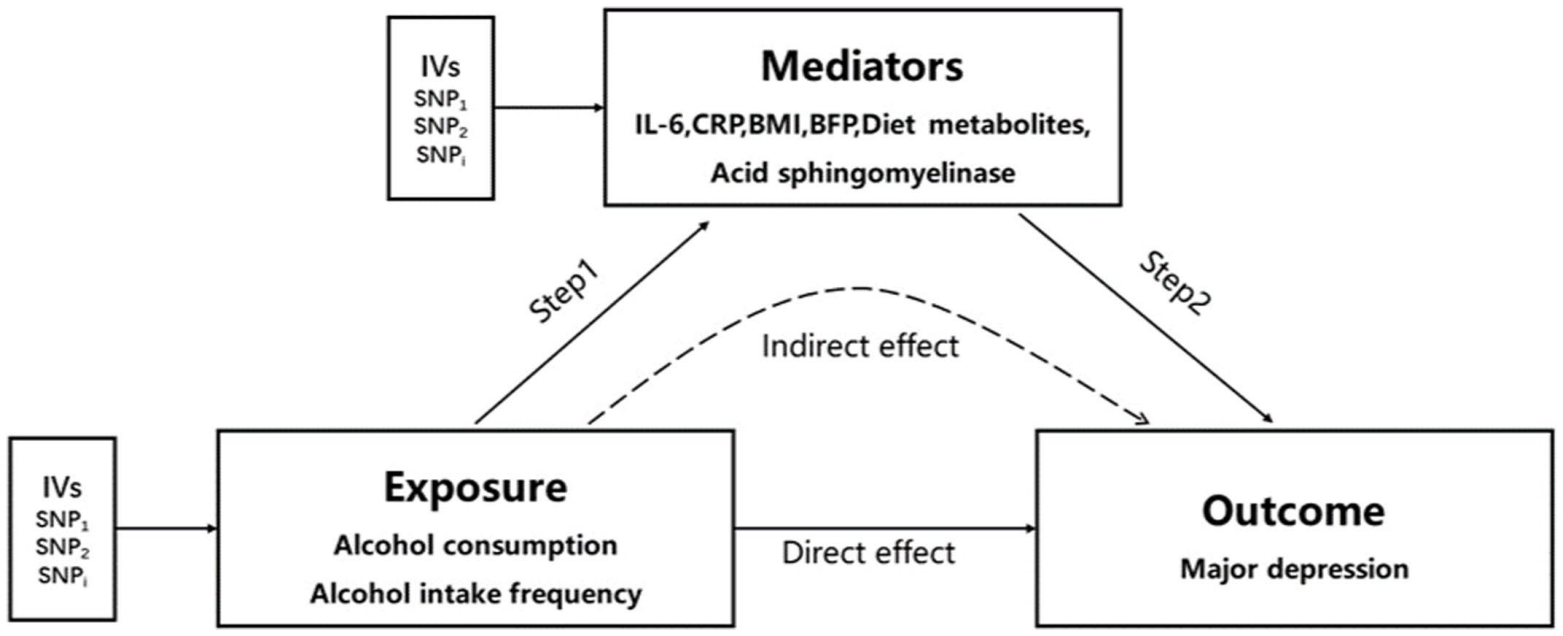
Two-step MR analysis framework. Step 1 estimated the causal effect of the exposure on the potential mediators, and step 2 assessed the causal effect of the mediators on major depression. “Direct effect” indicates the effect of exposure on major depression. “Indirect effect” indicates the effect of exposure on major depression through the mediator. IVs, instrumental variables.

Considering the potential impact of alcohol consumption on depression-related metabolites (45), we performed a two-step Mendelian randomization study, incorporating inflammation levels [including IL-6 (24) and CRP (25)], diet-related metabolites (28) (such as vitamin A, mannitol, and hippuric acid), acid sphingomyelinase (16), body mass index (26), and BFP (27) as mediating factors. Figure 2 (see Supplementary Data Sheet 9 for included SNPs) illustrates the study design. In the first step, we conducted univariate MR analyses to assess the causal effects of alcohol consumption and alcohol intake frequency on the aforementioned mediating factors. Our findings indicated a causal relationship between alcohol consumption and alcohol intake frequency with BMI, showing that alcohol consumption increases BMI (IVW method β = 0.33, 95% CI 0.08 to 0.59, *p* = 0.97 × 10^−2^), and alcohol intake frequency also leads to an increase in BMI (IVW method β = 0.21, 95% CI 0.09 to 0.32, *p* = 3.5 × 10^−4^). (Please refer to Table 4 for details.) Moreover, we observed a causal relationship only between alcohol intake frequency and the BFP. Alcohol intake frequency was found to be a contributing factor to the increase in the BFP (IVW method β = 0.14, 95% CI 0.07 to 0.32, *p* = 6.94 × 10^−5^), while alcohol consumption did not exhibit this effect (IVW method β = 0.09, 95% CI −0.10 to 0.28, *p* = 0.33), as shown in Table 4. The remaining factors showed no causal effect on alcohol consumption and alcohol intake frequency (Supplementary Tables 3, 4). Subsequently, we conducted a multivariate MR analysis involving BMI, BFP, and major depression. The results revealed that only the BFP had a causal effect on major depression when combined with BMI. An increase in the BFP was associated with an elevated risk of major depression (β = 0.21, 95% CI 0.03 to 0.38, *p* = 0.02), as shown in Table 4. Therefore, we examined the impact of alcohol consumption frequency on major depression by considering the BFP as a mediator. Our findings revealed that the BFP had a mediation effect value of 0.03 (95% CI: 0.01–0.03, *p* = 2.30 × 10^−4^), with a mediation ratio of 37.5% (95% CI, 0.22–0.38), as presented in Table 5.

**Table 4:** Two-step MR results for the relationship between major depression and alcohol.

Method	Number of SNPs	OR (95%CI)	β (95%CI)	*P*
**Step1. Alcohol consumption on BMI**				
IVW	3	1.40 (1.08–1.80)	0.33 (0.08–0.59)	0.97 × 10^−2^
**Step1. Alcohol consumption on BFP**				
IVW	3	1.10 (0.91–1.33)	0.09 (−0.10–1.28)	0.33
**Step1. Alcohol intake frequency on BMI**				
IVW	81	1.23 (1.10–1.38)	0.21 (0.09–0.32)	0.35 × 10^-3^
**Step1. Alcohol intake frequency on BFP**				
IVW	81	1.15 (1.07–1.24)	0.14 (0.07–0.21)	0.69 × 10^−6^
**Step2. BMI on major depression, conditioning on BFP**				
IVW	353	0.99 (0.88–1.12)	−0.008 (−0.13–0.11)	0.90
**Step2. BFP on major depression, conditioning on BMI**				
IVW	286	1.23 (1.03–1.47)	0.21 (0.03–0.38)	0.02

**Table 5:** The mediation effect of alcohol intake frequency on major depression via BFP.

Mediator	Total effect	Direct effect A	Direct effect B	Mediator effect	*P*	Mediated proportion
	β (95%CI)	β (95%CI)	β (95%CI)	β (95%CI)		β (95%CI)
BFP	0.08 (0.002–0.17)	0.14 (0.07–0.21)	0.21 (0.03–0.38)	0.03 (0.01–0.03)	2.30 × 10^−4^	37.5% (0.22–0.38)

## Discussion

In this study, we conducted an analysis on the causal effects of alcohol consumption and alcohol intake frequency on major depression. We found that alcohol consumption can alleviate major depression without considering different types of drinking, while alcohol intake frequency can aggravate it. However, when we comprehensively considered different types of drinking and possible mediating factors, we concluded that alcohol intake frequency is the main cause of aggravating major depression. One of the reasons for this is that alcohol intake frequency worsens the BFP, which in turn aggravates major depression.

In analyzing the causal effects of alcohol consumption and alcohol intake frequency on major depression, our study emphasizes the importance of the amount of alcohol consumed. The data for alcohol consumption was obtained from the GWAS study conducted by Clarke et al. (23). The average age of the participants in this study was 59.6 years old, with an average weekly alcohol consumption of 121.04 g, which is below the theoretical minimum risk drinking amount of 130.90 g/week (46). Therefore, the participants in this study can be classified as moderate drinkers. Our research findings suggest that moderate drinking can alleviate major depression. However, as alcohol intake frequency increases and the amount of drinking exceeds the theoretical minimum drinking amount, alcohol consumption becomes a factor that increasing the risk of major depression. This viewpoint is partially supported by the study conducted by Hammerton et al. (47). In their investigation, encompassing a sample of 3,902 adolescents, they discovered a direct correlation between alcohol dependency during the age of 18 and the onset of depression at the age of 24. However, no substantiating proof was identified to support the notion of a connection between alcohol usage and depression. Another study by Li et al. (48) examined the correlation between alcohol intake, alcohol use disorder, and the risk of depression. Their findings revealed that moderate drinking (0 g~84 g/week) reduces the risk of depression, while alcohol use disorder significantly increases the risk of subsequent depression (relative risk 1.57, 95% CI 1.41~1.76). This indicates that excessive drinking to the extent of alcohol use disorder can indeed contribute to depression.

A variety of biological mechanisms can provide evidence for the causal relationship between alcohol consumption and depression, such as the serotonin hypothesis. Serotonin (5-hydroxytryptamine), a neurotransmitter, is known to be related to the pathophysiology and treatment of depression (49). The synthesis of serotonin in the brain depends on the level of its precursor, tryptophan, in the plasma (50). During short-term or long-term alcohol abuse, the activity of hepatic tryptophan pyrrolase that responsible for tryptophan degradation increases, resulting in a decrease in plasma tryptophan levels. This reduction in tryptophan levels leads to a decrease in serotonin synthesis in the brain (51). The mediating effect of obesity is also related to tryptophan metabolism. Obesity is considered a chronic inflammatory state, with adipocytes secreting inflammatory cytokines such as IFN-γ, TNFα, and IL-1β (52). Alcohol exacerbates this inflammatory state, which in turn stimulates indoleamine 2,3-dioxygenase (IDO) and leads to the catabolism of tryptophan. This further reduces plasma tryptophan levels and hampers the synthesis of serotonin in the brain (53). In addition to reducing plasma tryptophan content, alcohol can directly decrease the number of serotonin neurons in the dorsal raphe nucleus through inflammatory reactions (54), contributing to the development of depression. The presence of obesity can intensify these inflammatory reactions (53). Another theory on the pathogenesis of depression involves the hyperfunction of the hypothalamic–pituitary–adrenal (HPA) axis, characterized by increased activity of corticotropin-releasing factor, reduced negative feedback function, and elevated cortisol levels (55). Alcohol not only affects the HPA axis of the fetus through maternal intake during early life, increasing the risk of subsequent depression (56), but it can also directly stimulate the HPA axis in adults, promoting the onset of depression (57). Cortisol has been found to facilitate the conversion of preadipocytes into mature adipocytes, and an elevation in cortisol levels is associated with an increase in fat accumulation (58). However, the precise mechanism through which alcohol consumption exacerbates depression by affecting adiposity requires further investigation through rigorous and high-quality studies.

For the reduction of depression risk through moderate alcohol consumption, it appears to be associated with the enhancement of dopaminergic and GABAergic signaling (59). *In vivo* microdialysis studies conducted on awake and freely moving rodents have demonstrated that alcohol increases dopamine levels in the nAc, thereby suggesting a decrease in depressive symptoms (60, 61). Additionally, alcohol can enhance the function of GABAergic transmission in the amygdala, which is also linked to resistance against depressive symptoms (62, 63). It is important to note that enhanced dopaminergic and GABAergic signaling is also associated with addictive behaviors (64). Therefore, moderate alcohol consumption can reduce the risk of depression, while excessive alcohol consumption can lead to addictive behaviors, which may also be indicative of depression. The influence of social factors may contribute to the diminished visibility of the causal connection between major depression and alcohol consumption when taking into account the collective impact of various alcoholic beverages. Research indicates that individuals who prefer red wine or white wine/champagne tend to have higher education levels and greater economic income compared with those who prefer beer/cider. This may explain why the consumption of red wine and white wine/champagne is linked to a lower risk of depression (65–68). As the population’s income and education levels continue to rise, it will be necessary to reevaluate the impact of different drinking preferences on major depression in the future.

In this research, a comprehensive two-sample Mendelian randomization study was conducted, utilizing a substantial number of stable and persistent genetic variants extracted from the GWAS summary. The primary objective of this investigation was to delve into the potential causal relationship between alcohol consumption, alcohol intake frequency and major depression, specifically focusing on major depression and normal drinking. Notably, the study exclusively focused on individuals of European descent to ensure the absence of ethnic bias. The decision to employ MR analysis instead of retrospective studies was made due to its ability to eliminate confounding variables and its immunity to reverse causation. Furthermore, MR studies offer larger sample sizes and a closer approximation to random assignment in comparison to randomized controlled trials. Moreover, our study utilized instrumental variables that were considerably more detailed, comprehensive, and reliable when compared to previous research endeavors. However, it is crucial to acknowledge certain limitations within this study. Firstly, the biological significance of the genetic tools employed in this research remains unknown, thus we cannot completely dismiss the possibility of violations of the independence and exclusion restriction assumptions, particularly regarding pleiotropy. Nonetheless, we incorporated various methods, such as sensitivity analysis employing the Cochran Q statistic, MR-PRESSO, weighted median, weighted mode, and MR-Egger, to determine trustworthy causal estimates. Secondly, the SNPs associated with alcohol consumption predominantly originated from the UK Biobank study, which predominantly consisted of individuals with a mean age of 59.6 years. Conversely, the sample used in the major depressive disorder GWAS encompassed a broader age range. Consequently, it is plausible that alcohol usage among younger individuals could be influenced by other variants with distinct associations with major depression, However, it should be duly noted that relevant GWAS data pertaining to this phenomenon is not yet accessible. Thirdly, it is important to note that we cannot confirm whether the individuals in the study received psychotherapy as part of their treatment. The inclusion of psychotherapy may have some influence on the study’s results. This is because, if the participants had received psychotherapy, it is likely that it would have helped them reduce their alcohol usage and alleviate their significant depression. Therefore, these factors must be considered when interpreting the study’s conclusions. Lastly, while MR can effectively serve as an alternative to randomized controlled trials when assessing causality, it is crucial to acknowledge that genetic variation is inherently influenced by innate factors and may not entirely reflect the impact of a particular intervention on the outcomes. To obtain definitive confirmation of causal relationships, it may be necessary to conduct randomized controlled trials (RCTs) on preventive interventions. Additionally, it should be noted that the depression genome-wide association studies (GWAS) utilized in our analysis did not account for the diversity of major depressive disorder (MDD), specifically atypical depression and melancholic depression.

To explore the potential correlation between depression and alcohol consumption, a bidirectional two-sample MR analysis was executed in this research. The results show strong genetic evidence supporting a causal connection between increased frequency of drinking and heightened susceptibility to major depression. Furthermore, it was observed that the impact of augmented alcohol intake on depression risk is partially influenced by the BFP. These findings have important therapeutic implications for people with alcohol use disorder and MDD. Individuals with comorbid drinking disorders and MDD may benefit from a progressive reduction in alcohol use and weight management during treatment rather than abrupt alcohol abstinence. This approach can help align patients’ psychological and physiological elements with treatment goals, potentially reducing psychological stress associated with alcohol withdrawal and alleviating depression symptoms. A less rigorous treatment strategy may also increase patient compliance and overall therapy efficacy. It is advisable to carry out a robust randomized clinical trial in the future to substantiate the significance of these discoveries.

## Data availability statement

The original contributions presented in the study are included in the article/Supplementary material, further inquiries can be directed to the corresponding author.

## Ethics statement

Ethical approval was not required for the study involving humans in accordance with the local legislation and institutional requirements. Written informed consent to participate in this study was not required from the participants or the participants’ legal guardians/next of kin in accordance with the national legislation and the institutional requirements.

## Author contributions

WF: Writing – original draft, Writing – review & editing, Data curation, Investigation. BZ: Writing – review & editing, Funding acquisition, Project administration, Supervision, Validation. PD: Data curation, Writing – review & editing. Y-hB: Methodology, Writing – review & editing. ZJ: Validation, Writing – review & editing. XL: Investigation, Writing – review & editing. XZ: Software, Writing – review & editing. KZ: Data curation, Writing – review & editing.

## Footnotes

^1^ https://gwas.mrcieu.ac.uk/
