# Supplementary material for: Risk of major depressive increases with increasing frequency of alcohol drinking: a bidirectional two-sample Mendelian randomization analysis
Source: Front Public Health. 2024 Jun 5;12:1372758. doi: 10.3389/fpubh.2024.1372758 (PMC11186411; doi:10.3389/fpubh.2024.1372758)
Supplement: Supplementary file 1 [file Table_1.pdf]

Supplementary table 1. Summary of the GWAS included in this two-sample MR study

Variable	ID	Sample size	Number of SNPs	Consortium	Population	Sex	Year
Alcohol consumption	ieu-a-1283	112,117	12,935,359	UK Biobank	European	Males and Females	2017
Alcohol intake frequency	ukb-b-5779	462,346	9,851,867	MRC-IEU	European	Males and Females	2018
Major depression	ieu-b-102	500,199	--	PGC	European	Males and Females	2019
Beer/cider intake	ukb-b-4961	64,949	9,851,867	MRC-IEU	European	Males and Females	2018
Fortifide wine intake	ukb-b-9624	64,942	9,851,867	MRC-IEU	European	Males and Females	2018
Red wine intake	ukb-b-13556	64,949	9,851,867	MRC-IEU	European	Males and Females	2018
White wine/champagne intake	ukb-b-311	64,949	9,851,867	MRC-IEU	European	Males and Females	2018
Spirtis intake	ukb-b-3751	64,949	9,851,867	MRC-IEU	European	Males and Females	2018
Other alcohol intake	ukb-b-12901	64,944	9,851,867	MRC-IEU	European	Males and Females	2018
Hydroxyhippurate	met-a-703	4,368	2,543,571	--	European	Males and Females	2014
Acid phosphodiesterase	prot-a-2781	3,301	10,534,735	--	European	Males and Females	2018
BMI	ukb-b-19953	461,460	9,851,867	MRC-IEU	European	Males and Females	2018
Body fat percentage	ukb-b-8909	454,633	9,851,867	MRC-IEU	European	Males and Females	2018
CRP	ieu-b-4764	61,308	8,036,590	Within family GWAS consortium	European	Males and Females	2021
IL-6	ebi-a-GCST004446	8,189	9,790,590	--	European	Males and Females	2016
Mannitol	met-a-376	5,917	2,545,531	--	European	Males and Females	2014
Vitamin A	ukb-b-9596	460,351	9,851,867	MRC-IEU	European	Males and Females	2018

Supplementary table 2. MR results for the relationship between different alcohol and major depression

Method	Number of SNPs	OR（95%CI）	β (95%CI)	P
**Beer/cider intake on major depression**				
IVW	11	1.00（0.91-1.09）	-0.003（-0.09-0.08）	0.95
**Fortified wine intake on major depression**				
IVW	20	0.99（0.76-1.28）	-0.01（-0.27-0.25）	0.93
**Red wine intake on major depression**				
IVW	8	0.99（0.88-1.11）	-0.01（-0.13-0.10）	0.81
**White wine/champange intake on major depression**				
IVW	12	0.98（0.86-1.11）	-0.02（-0.15-0.11）	0.73
**Spirits intake on major depression**				
IVW	20	1.13（1.00-1.29）	0.13（-0.004-0.26）	0.06
**Other alcohol intake on major depression**				
IVW	37	1.02（0.82-1.28）	0.02（-0.20-0.24）	0.84

Supplementary table 3. MR results for the relationship between alcohol consumption and mediators

Method	Number of SNPs	OR（95%CI）	β (95%CI)	P
**Alcohol consumption on IL-6**				
IVW	3	1.22（0.57-2.61）	0.20（-0.56-0.96）	0.60
**Alcohol consumption on CRP**				
IVW	3	0.40（0.08-2.00）	-0.93（-2.55-0.69）	0.26
**Alcohol consumption on BMI**				
IVW	3	1.40（1.08-1.80）	0.33（0.08-0.59）	0.97×10^{-2}
**Alcohol consumption on body fat percentage**				
IVW	3	1.10（0.92-1.33）	0.09（-0.10-0.28）	0.33
**Alcohol consumption on hydroxyhippurate**				
IVW	2	0.97（0.60-1.58）	-0.03（-0.52-0.46）	0.91
**Alcohol consumption on acid phosphodiesterase**				
IVW	5	0.52（0.13-2.07）	-0.65（-2.02-0.73）	0.36
**Alcohol consumption on minnitol**				
IVW	2	0.99（0.55-1.77）	-0.009（-0.59-0.57）	0.97
**Alcohol consumption on Vitamin A**				
IVW	3	1.00（0.99-1.02）	0.001（-0.01-0.15）	0.84

Supplementary table 4. MR results for the relationship between alcohol intake frequency and mediators

Method	Number of SNPs	OR（95%CI）	β (95%CI)	P
**Alcohol intake frequency on IL-6**				
IVW	85	1.05（0.89-1.25）	0.05（-0.12-0.22）	0.54
**Alcohol intake frequency on CRP**				
IVW	63	1.08（0.99-1.17）	0.07（-0.01-0.16）	0.09
**Alcohol intake frequency on BMI**				
IVW	81	1.23（1.10-1.38）	0.21（0.09-0.32）	0.35×10^{-3}
**Alcohol intake frequency on body fat percentage**				
IVW	81	1.15（1.07-1.24）	0.14（0.07-0.21）	0.69×10^{-6}
**Alcohol intake frequency on hydroxyhippurate**				
IVW	36	0.99（0.87-1.14）	-0.007（-0.14-0.13）	0.92
**Alcohol intake frequency on acid phosphodiesterase**				
IVW	93	1.14（0.90-1.44）	0.13（-0.10-0.37）	0.27
**Alcohol intake frequency on minnitol**				
IVW	36	0.91（0.78-1.07）	-0.08（-0.25-0.07）	0.27
**Alcohol intake frequency on Vitamin A**				
IVW	91	1.001（0.999-1.005）	0.002（-0.001-0.005）	0.27
