# Supplementary material for: Risk of major depressive increases with increasing frequency of alcohol drinking: a bidirectional two-sample Mendelian randomization analysis
Source: Front Public Health. 2024 Jun 5;12:1372758. doi: 10.3389/fpubh.2024.1372758 (PMC11186411; doi:10.3389/fpubh.2024.1372758)
Supplement: Supplementary file 2 [file Data_Sheet_1.PDF]

Alcohol consumption SNPs

chr.exposu	pos.exposu	other_allel	effect_allel	beta.expos	se.exposur	pval.expos	eaf.exposu	samplesize	ncase.expo	SNP	ncontrol.e	exposure	mr_keep.e	pval_origir	id.exposure
2	27556721	G	A	0.01633	0.002992	4.85E-08	0.4381	110852	NA	rs3739095	NA	ieu-a-128	TRUE	reported	hp48Sr
2	27568871	T	C	0.01638	0.002988	4.26E-08	0.4385	111137	NA	rs1020559	NA	ieu-a-128	TRUE	reported	hp48Sr
2	27582035	A	G	0.0172	0.002997	9.63E-09	0.4438	110458	NA	rs9678013	NA	ieu-a-128	TRUE	reported	hp48Sr
2	27583156	G	A	0.01688	0.002993	1.72E-08	0.4433	110823	NA	rs6729709	NA	ieu-a-128	TRUE	reported	hp48Sr
2	27584666	A	G	0.01693	0.002991	1.52E-08	0.4437	110965	NA	rs7586601	NA	ieu-a-128	TRUE	reported	hp48Sr
2	27598097	T	C	0.02708	0.003023	3.41E-19	0.605	108388	NA	rs4665972	NA	ieu-a-128	TRUE	reported	hp48Sr
2	27619049	C	G	0.0174	0.002989	5.84E-09	0.4468	111069	NA	rs1083864	NA	ieu-a-128	TRUE	reported	hp48Sr
2	27625661	A	G	0.01747	0.002985	4.82E-09	0.4474	111370	NA	rs1260338	NA	ieu-a-128	TRUE	reported	hp48Sr
2	27635463	A	G	0.02058	0.002973	4.50E-12	0.72	112176	NA	rs1728918	NA	ieu-a-128	TRUE	reported	hp48Sr
2	27648726	T	C	0.01785	0.002984	2.20E-09	0.4473	111442	NA	rs4665978	NA	ieu-a-128	TRUE	reported	hp48Sr
2	27649929	T	G	0.01741	0.002983	5.35E-09	0.4466	111542	NA	rs1728911	NA	ieu-a-128	TRUE	reported	hp48Sr
2	27651375	C	T	0.01713	0.002989	9.98E-09	0.4427	111068	NA	rs2303370	NA	ieu-a-128	TRUE	reported	hp48Sr
2	27667520	T	TTTTG	0.01747	0.002976	4.39E-09	0.4474	112066	NA	rs1063508	NA	ieu-a-128	TRUE	reported	hp48Sr
2	27678471	A	G	0.01772	0.002978	2.71E-09	0.446	111878	NA	rs780105	NA	ieu-a-128	TRUE	reported	hp48Sr
2	27684957	T	C	0.01755	0.002975	3.68E-09	0.4471	112150	NA	rs780108	NA	ieu-a-128	TRUE	reported	hp48Sr
2	27685388	G	A	0.01757	0.002974	3.48E-09	0.4468	112176	NA	rs780110	NA	ieu-a-128	TRUE	reported	hp48Sr
2	27711893	A	G	0.01665	0.002975	2.19E-08	0.4671	112101	NA	rs1260327	NA	ieu-a-128	TRUE	reported	hp48Sr
2	27730940	T	C	0.02839	0.002973	1.34E-21	0.6066	112176	NA	rs1260326	NA	ieu-a-128	TRUE	reported	hp48Sr
2	27734972	G	A	0.02449	0.002983	2.25E-16	0.5569	111477	NA	rs6547692	NA	ieu-a-128	TRUE	reported	hp48Sr
2	27741072	C	G	0.02465	0.002994	1.83E-16	0.5698	110688	NA	rs780096	NA	ieu-a-128	TRUE	reported	hp48Sr
2	27741105	A	G	0.02467	0.002993	1.72E-16	0.5699	110707	NA	rs780095	NA	ieu-a-128	TRUE	reported	hp48Sr
2	27741237	T	C	0.02786	0.002973	7.27E-21	0.6183	112176	NA	rs780094	NA	ieu-a-128	TRUE	reported	hp48Sr
2	27742603	T	C	0.02763	0.002973	1.52E-20	0.6181	112176	NA	rs780093	NA	ieu-a-128	TRUE	reported	hp48Sr
2	27748597	C	A	0.02394	0.002976	8.78E-16	0.5669	112019	NA	rs1260334	NA	ieu-a-128	TRUE	reported	hp48Sr
2	27748624	A	G	0.02385	0.002974	1.08E-15	0.5668	112176	NA	rs1260333	NA	ieu-a-128	TRUE	reported	hp48Sr
2	27748904	G	A	0.02389	0.002994	1.49E-15	0.5672	110623	NA	rs1313566	NA	ieu-a-128	TRUE	reported	hp48Sr
2	27748992	AT	A	0.02807	0.003003	9.09E-21	0.6189	109946	NA	rs1133684	NA	ieu-a-128	TRUE	reported	hp48Sr
2	27750545	A	G	0.02437	0.003075	2.32E-15	0.5576	104378	NA	rs2950835	NA	ieu-a-128	TRUE	reported	hp48Sr
2	27750546	T	A	0.02428	0.003075	2.96E-15	0.5575	104366	NA	rs2911711	NA	ieu-a-128	TRUE	reported	hp48Sr
2	27752463	G	A	0.02895	0.003095	8.54E-21	0.6211	102757	NA	rs1112704	NA	ieu-a-128	TRUE	reported	hp48Sr
2	27752871	C	T	0.02292	0.00303	3.98E-14	0.5688	107865	NA	rs6753534	NA	ieu-a-128	TRUE	reported	hp48Sr
2	27753878	A	C	-0.02216	0.003089	7.36E-13	0.2604	103306	NA	rs4665985	NA	ieu-a-128	TRUE	reported	hp48Sr
2	27755825	G	A	-0.0223	0.003004	1.14E-13	0.2521	109910	NA	rs4665987	NA	ieu-a-128	TRUE	reported	hp48Sr
2	27761436	C	A	-0.0221	0.003006	1.98E-13	0.2514	109725	NA	rs4665988	NA	ieu-a-128	TRUE	reported	hp48Sr
2	27764199	C	T	-0.02144	0.003029	1.48E-12	0.2451	108013	NA	rs7720071	NA	ieu-a-128	TRUE	reported	hp48Sr
2	27765587	T	C	-0.02216	0.003004	1.63E-13	0.2521	109916	NA	rs1247313	NA	ieu-a-128	TRUE	reported	hp48Sr
2	27766284	G	A	-0.02222	0.003004	1.41E-13	0.2521	109916	NA	rs4665991	NA	ieu-a-128	TRUE	reported	hp48Sr
2	27767488	GC	G	-0.02227	0.003011	1.41E-13	0.2529	109360	NA	rs6708099	NA	ieu-a-128	TRUE	reported	hp48Sr
2	27776631	AT	A	-0.02221	0.00301	1.62E-13	0.2491	109433	NA	rs2021036	NA	ieu-a-128	TRUE	reported	hp48Sr
2	27778167	A	C	-0.02217	0.003004	1.58E-13	0.2518	109939	NA	rs1299546	NA	ieu-a-128	TRUE	reported	hp48Sr
2	27778560	A	G	-0.02234	0.003009	1.15E-13	0.2502	109525	NA	rs1016906	NA	ieu-a-128	TRUE	reported	hp48Sr
2	27778572	G	A	-0.02234	0.003009	1.15E-13	0.2502	109525	NA	rs1017987	NA	ieu-a-128	TRUE	reported	hp48Sr
2	27783801	T	C	-0.02198	0.002974	1.48E-13	0.2539	112151	NA	rs4665382	NA	ieu-a-128	TRUE	reported	hp48Sr
2	27784658	C	T	-0.02193	0.002975	1.70E-13	0.2537	112076	NA	rs7570476	NA	ieu-a-128	TRUE	reported	hp48Sr
2	27786188	A	T	-0.02194	0.002974	1.64E-13	0.2539	112139	NA	rs1020852	NA	ieu-a-128	TRUE	reported	hp48Sr
2	27791555	C	G	-0.02193	0.002974	1.66E-13	0.2539	112154	NA	rs4665383	NA	ieu-a-128	TRUE	reported	hp48Sr
2	27793921	G	T	-0.02184	0.002974	2.09E-13	0.2539	112158	NA	rs7577311	NA	ieu-a-128	TRUE	reported	hp48Sr
2	27801493	T	C	-0.0219	0.002974	1.78E-13	0.2539	112176	NA	rs1919127	NA	ieu-a-128	TRUE	reported	hp48Sr
2	27801759	A	G	-0.02189	0.002974	1.85E-13	0.2539	112176	NA	rs1919128	NA	ieu-a-128	TRUE	reported	hp48Sr
2	27811722	A	G	-0.02178	0.002978	2.60E-13	0.2532	111885	NA	rs1247884	NA	ieu-a-128	TRUE	reported	hp48Sr
2	27812252	G	A	-0.02184	0.002974	2.11E-13	0.2539	112162	NA	rs6760250	NA	ieu-a-128	TRUE	reported	hp48Sr
2	27815510	A	C	-0.02169	0.002974	3.01E-13	0.2542	112176	NA	rs1302287	NA	ieu-a-128	TRUE	reported	hp48Sr
2	27817817	G	A	-0.02199	0.002976	1.49E-13	0.2539	112032	NA	rs1301058	NA	ieu-a-128	TRUE	reported	hp48Sr
2	27821482	C	T	-0.02188	0.002975	1.94E-13	0.2534	112050	NA	rs1298967	NA	ieu-a-128	TRUE	reported	hp48Sr
2	27825715	T	C	-0.02195	0.002975	1.62E-13	0.2536	112112	NA	rs1246747	NA	ieu-a-128	TRUE	reported	hp48Sr
2	27827092	C	T	-0.0223	0.002975	6.69E-14	0.2534	112077	NA	rs1246461	NA	ieu-a-128	TRUE	reported	hp48Sr
2	27832055	A	G	-0.02202	0.002974	1.34E-13	0.2537	112155	NA	rs2384656	NA	ieu-a-128	TRUE	reported	hp48Sr
2	27833502	T	C	-0.02202	0.002975	1.35E-13	0.2534	112090	NA	rs7417506	NA	ieu-a-128	TRUE	reported	hp48Sr
2	27833687	C	T	-0.02181	0.002976	2.34E-13	0.2534	112001	NA	rs3401699	NA	ieu-a-128	TRUE	reported	hp48Sr
2	27834152	T	C	-0.02194	0.002974	1.61E-13	0.2535	112176	NA	rs1298705	NA	ieu-a-128	TRUE	reported	hp48Sr
2	27836605	C	T	-0.02212	0.002978	1.12E-13	0.2526	111854	NA	rs1302094	NA	ieu-a-128	TRUE	reported	hp48Sr
2	27839369	T	C	-0.02086	0.002976	2.44E-12	0.268	111999	NA	rs4666000	NA	ieu-a-128	TRUE	reported	hp48Sr
2	27839539	T	C	-0.02081	0.00298	2.87E-12	0.2671	111723	NA	rs2068834	NA	ieu-a-128	TRUE	reported	hp48Sr
2	27840640	G	C	-0.02222	0.002976	8.25E-14	0.2535	111998	NA	rs4666002	NA	ieu-a-128	TRUE	reported	hp48Sr
2	27851918	G	A	-0.02157	0.002974	4.14E-13	0.2495	112176	NA	rs3749147	NA	ieu-a-128	TRUE	reported	hp48Sr
2	27853245	C	G	-0.0218	0.002977	2.45E-13	0.2494	111967	NA	rs1300285	NA	ieu-a-128	TRUE	reported	hp48Sr
2	27854524	G	A	-0.0217	0.002976	3.07E-13	0.25	111993	NA	rs3450205	NA	ieu-a-128	TRUE	reported	hp48Sr
2	27859351	T	A	-0.01995	0.002987	2.40E-11	0.2648	111194	NA	rs6719175	NA	ieu-a-128	TRUE	reported	hp48Sr
2	27867573	GT	G	0.01898	0.003006	2.72E-10	0.8055	109751	NA	rs3501216	NA	ieu-a-128	TRUE	reported	hp48Sr
2	27892023	A	T	-0.01934	0.00298	8.72E-11	0.2001	111732	NA	rs2272406	NA	ieu-a-128	TRUE	reported	hp48Sr
2	27901728	C	T	-0.0193	0.002978	9.04E-11	0.2008	111930	NA	rs1302120	NA	ieu-a-128	TRUE	reported	hp48Sr
2	27910706	A	C	-0.01853	0.002987	5.57E-10	0.1982	111225	NA	rs1302309	NA	ieu-a-128	TRUE	reported	hp48Sr
2	27913188	T	C	-0.01849	0.002985	5.93E-10	0.1988	111352	NA	rs1299877	NA	ieu-a-128	TRUE	reported	hp48Sr
2	27928797	T	C	-0.01916	0.002984	1.36E-10	0.202	111478	NA	rs1303097	NA	ieu-a-128	TRUE	reported	hp48Sr
2	27932587	A	G	-0.01927	0.002984	1.06E-10	0.2019	111464	NA	rs6727388	NA	ieu-a-128	TRUE	reported	hp48Sr
2	27933642	C	T	-0.01922	0.002984	1.19E-10	0.202	111484	NA	rs4616435	NA	ieu-a-128	TRUE	reported	hp48Sr
2	27934731	G	A	-0.01925	0.002985	1.12E-10	0.2017	111381	NA	rs6727215	NA	ieu-a-128	TRUE	reported	hp48Sr
2	27950837	G	A	-0.01797	0.002997	2.03E-09	0.2018	110453	NA	rs1043270	NA	ieu-a-128	TRUE	reported	hp48Sr
2	27967260	G	C	-0.01726	0.003008	9.64E-09	0.1642	109602	NA	rs1302319	NA	ieu-a-128	TRUE	reported	hp48Sr
2	27987221	C	G	-0.01721	0.003017	1.17E-08	0.164	108886	NA	rs1300648	NA	ieu-a-128	TRUE	reported	hp48Sr
2	28003174	G	T	-0.01703	0.003	1.36E-08	0.165	110251	NA	rs1303034	NA	ieu-a-128	TRUE	reported	hp48Sr
3	85024021	T	A	0.01694	0.003	1.63E-08	0.3717	110094	NA	rs1307587	NA	ieu-a-128	TRUE	reported	hp48Sr
3	85067018	A	G	0.01676	0.003029	3.14E-08	0.4708	107975	NA	rs9816652	NA	ieu-a-128	TRUE	reported	hp48Sr
3	85236425	G	A	0.01798	0.00299	1.85E-09	0.3158	110858	NA	rs1376935	NA	ieu-a-128	TRUE	reported	hp48Sr
3	85264266	G	A	0.01853	0.002982	5.17E-10	0.3116	111539	NA	rs1307838	NA	ieu-a-128	TRUE	reported	hp48Sr
3	85370081	A	AT	0.01771	0.002985	2.98E-09	0.3399	111278	NA	rs7110827	NA	ieu-a-128	TRUE	reported	hp48Sr
3	85370873	C	T	0.01827	0.002981	8.89E-10	0.3049	111597	NA	rs9831374	NA	ieu-a-128	TRUE	reported	hp48Sr
3	85371838	A	G	0.0183	0.002976	7.91E-10	0.3051	111929	NA	rs1171927	NA	ieu-a-128	TRUE	reported	hp48Sr
3	85372128	T	A	0.01796	0.002976	1.62E-09	0.3413	111921	NA	rs1171568	NA	ieu-a-128	TRUE	reported	hp48Sr
3	85391672	G	A	0.01832	0.002973	7.18E-10	0.3164	112176	NA	rs9811546	NA	ieu-a-128	TRUE	reported	hp48Sr
3	85391799	CCTT	C	0.01817	0.003013	1.62E-09	0.3153	109181	NA	rs6703367	NA	ieu-a-128	TRUE	reported	hp48Sr
3	85405501	T	C	0.01807	0.002985	1.41E-09	0.2244	111268	NA	rs9822731	NA	ieu-a-128	TRUE	reported	hp48Sr
3	85406367	C	T	0.01807	0.002985	1.43E-09	0.2353	111296	NA	rs1249801	NA	ieu-a-128	TRUE	reported	hp48Sr
3	85407980	G	T	0.01837	0.002973	6.54E-10	0.2363	112176	NA	rs9814516	NA	ieu-a-128	TRUE	reported	hp48Sr
3	85409299	C	T	0.01827	0.002997	1.09E-09	0.2208	110385	NA	rs1015486	NA	ieu-a-128	TRUE	reported	hp48Sr
3	85409313	G	A	-0.01641	0.003003	4.67E-08	0.7325	109938	NA	rs9824178	NA	ieu-a-128	TRUE	reported	hp48Sr
3	85409353	A	G	-0.01636	0.002997	4.82E-08	0.7313	110395	NA	rs9861451	NA	ieu-a-128	TRUE	reported	hp48Sr
3	85409884	C	T	0.01864	0.002977	3.84E-10	0.2351	111879	NA	rs1307663	NA	ieu-a-128	TRUE	reported	hp48Sr
3	85409979	C	T	0.01822	0.002975	9.16E-10	0.2248	112040	NA	rs1307683	NA	ieu-a-128	TRUE	reported	hp48Sr
3	85410088	T	A	0.01824	0.002974	8.76E-10	0.2358	112093	NA	rs1309975	NA	ieu-a-128	TRUE	reported	hp48Sr
3	85413937	C	A	0.0181	0.002978	1.22E-09	0.2247	111810	NA	rs9846211	NA	ieu-a-128	TRUE	reported	hp48Sr
3	85413995	T	C	0.01743	0.00298	4.95E-09	0.2435	111660	NA	rs9866089	NA	ieu-a-128	TRUE	reported	hp48Sr
3	85414856	G	A	0.0184	0.002986	7.25E-10	0.2337	111211	NA	rs1112788	NA	ieu-a-128	TRUE	reported	hp48Sr

3	85416384	A	G	0.01819	0.002978	1.00E-09	0.2247	111790	NA	rs9870448	NA	ieu-a-128	TRUE	reported	hp48Sr
3	85420724	C	A	0.01823	0.002978	9.23E-10	0.2247	111785	NA	rs9854869	NA	ieu-a-128	TRUE	reported	hp48Sr
3	85421737	C	G	0.01798	0.002979	1.58E-09	0.2444	111736	NA	rs4279114	NA	ieu-a-128	TRUE	reported	hp48Sr
3	85422587	C	T	0.01793	0.002978	1.74E-09	0.2445	111761	NA	rs9865191	NA	ieu-a-128	TRUE	reported	hp48Sr
3	85428963	C	T	0.01797	0.002979	1.60E-09	0.2442	111746	NA	rs1433711	NA	ieu-a-128	TRUE	reported	hp48Sr
3	85429172	C	A	0.01809	0.00298	1.29E-09	0.2446	111644	NA	rs1308517	NA	ieu-a-128	TRUE	reported	hp48Sr
3	85430137	T	C	0.01813	0.002978	1.15E-09	0.2443	111762	NA	rs9819830	NA	ieu-a-128	TRUE	reported	hp48Sr
3	85432315	G	C	0.01814	0.002978	1.13E-09	0.2443	111773	NA	rs9814919	NA	ieu-a-128	TRUE	reported	hp48Sr
3	85434357	T	A	0.01803	0.002978	1.43E-09	0.2442	111764	NA	rs2326313	NA	ieu-a-128	TRUE	reported	hp48Sr
3	85436125	C	T	0.01809	0.002978	1.25E-09	0.2444	111818	NA	rs9879025	NA	ieu-a-128	TRUE	reported	hp48Sr
3	85438564	A	G	0.01815	0.002978	1.10E-09	0.2442	111792	NA	rs1821349	NA	ieu-a-128	TRUE	reported	hp48Sr
3	85438709	A	G	0.01815	0.002978	1.09E-09	0.2443	111818	NA	rs1368743	NA	ieu-a-128	TRUE	reported	hp48Sr
3	85442709	C	T	0.01826	0.002978	8.64E-10	0.2443	111800	NA	rs9309977	NA	ieu-a-128	TRUE	reported	hp48Sr
3	85448493	T	C	0.01828	0.002977	8.33E-10	0.2446	111835	NA	rs9863620	NA	ieu-a-128	TRUE	reported	hp48Sr
3	85449777	G	T	0.01838	0.002982	7.09E-10	0.2434	111507	NA	rs7162688	NA	ieu-a-128	TRUE	reported	hp48Sr
3	85451804	C	A	0.01823	0.002981	9.68E-10	0.2448	111562	NA	rs7650834	NA	ieu-a-128	TRUE	reported	hp48Sr
3	85458683	C	A	0.01835	0.002977	7.23E-10	0.2445	111841	NA	rs2117151	NA	ieu-a-128	TRUE	reported	hp48Sr
3	85458861	T	C	0.0183	0.002977	8.03E-10	0.2446	111841	NA	rs1030721	NA	ieu-a-128	TRUE	reported	hp48Sr
3	85461302	G	A	0.01841	0.002977	6.30E-10	0.2437	111831	NA	rs9880919	NA	ieu-a-128	TRUE	reported	hp48Sr
3	85462587	T	C	0.01829	0.00298	8.34E-10	0.2241	111625	NA	rs6791236	NA	ieu-a-128	TRUE	reported	hp48Sr
3	85466452	T	C	0.018	0.002974	1.44E-09	0.2449	112095	NA	rs6549024	NA	ieu-a-128	TRUE	reported	hp48Sr
3	85468919	A	G	0.01802	0.002974	1.39E-09	0.2449	112100	NA	rs1249346	NA	ieu-a-128	TRUE	reported	hp48Sr
3	85470642	T	C	0.01761	0.002997	4.23E-09	0.219	110380	NA	rs2196096	NA	ieu-a-128	TRUE	reported	hp48Sr
3	85470645	C	T	0.01765	0.002996	3.85E-09	0.2191	110389	NA	rs2196097	NA	ieu-a-128	TRUE	reported	hp48Sr
3	85471818	G	A	0.01791	0.002974	1.73E-09	0.2449	112097	NA	rs1043349	NA	ieu-a-128	TRUE	reported	hp48Sr
3	85473317	A	G	0.01801	0.002975	1.42E-09	0.2449	112046	NA	rs6772991	NA	ieu-a-128	TRUE	reported	hp48Sr
3	85473932	T	A	0.01797	0.002975	1.55E-09	0.2448	112046	NA	rs9865745	NA	ieu-a-128	TRUE	reported	hp48Sr
3	85475597	T	A	0.01725	0.002989	7.79E-09	0.2412	111009	NA	rs744580	NA	ieu-a-128	TRUE	reported	hp48Sr
3	85476094	G	A	0.01784	0.002976	2.03E-09	0.2447	111937	NA	rs1308123	NA	ieu-a-128	TRUE	reported	hp48Sr
3	85476219	C	G	0.01783	0.002976	2.10E-09	0.2447	111939	NA	rs6807456	NA	ieu-a-128	TRUE	reported	hp48Sr
3	85476898	C	T	0.01811	0.002982	1.26E-09	0.245	111494	NA	rs6808159	NA	ieu-a-128	TRUE	reported	hp48Sr
3	85479827	A	AGAGAAA	0.0179	0.002978	1.86E-09	0.2444	111810	NA	rs1480219	NA	ieu-a-128	TRUE	reported	hp48Sr
3	85480184	C	T	0.01783	0.002976	2.10E-09	0.2447	111939	NA	rs1025533	NA	ieu-a-128	TRUE	reported	hp48Sr
3	85481659	C	T	0.0178	0.002977	2.26E-09	0.225	111892	NA	rs1171623	NA	ieu-a-128	TRUE	reported	hp48Sr
3	85485110	C	T	0.01786	0.002981	2.11E-09	0.244	111549	NA	rs1309262	NA	ieu-a-128	TRUE	reported	hp48Sr
3	85486209	GT	G	0.01791	0.002976	1.77E-09	0.2252	111949	NA	rs3548931	NA	ieu-a-128	TRUE	reported	hp48Sr
3	85486931	T	A	0.0175	0.002984	4.50E-09	0.2428	111373	NA	rs1025531	NA	ieu-a-128	TRUE	reported	hp48Sr
3	85488462	C	T	0.01788	0.002976	1.90E-09	0.2447	111934	NA	rs9868427	NA	ieu-a-128	TRUE	reported	hp48Sr
3	85488646	T	G	0.01796	0.002976	1.59E-09	0.2453	111925	NA	rs9813512	NA	ieu-a-128	TRUE	reported	hp48Sr
3	85489878	AT	A	0.01783	0.002976	2.10E-09	0.2447	111939	NA	rs1129390	NA	ieu-a-128	TRUE	reported	hp48Sr
3	85489906	G	A	0.01755	0.002998	4.86E-09	0.2437	110276	NA	rs9874740	NA	ieu-a-128	TRUE	reported	hp48Sr
3	85489907	C	T	0.0179	0.002976	1.80E-09	0.2252	111960	NA	rs9874491	NA	ieu-a-128	TRUE	reported	hp48Sr
3	85491756	A	G	0.01677	0.003009	2.47E-08	0.2162	109479	NA	rs5514140	NA	ieu-a-128	TRUE	reported	hp48Sr
3	85491757	T	A	0.01677	0.003009	2.47E-08	0.2162	109479	NA	rs5698220	NA	ieu-a-128	TRUE	reported	hp48Sr
3	85491764	A	G	0.01677	0.003009	2.47E-08	0.2162	109479	NA	rs5309494	NA	ieu-a-128	TRUE	reported	hp48Sr
3	85491765	T	A	0.01677	0.003009	2.47E-08	0.2162	109479	NA	rs5499428	NA	ieu-a-128	TRUE	reported	hp48Sr
3	85491766	A	G	0.01676	0.003009	2.55E-08	0.2162	109474	NA	rs5681877	NA	ieu-a-128	TRUE	reported	hp48Sr
3	85493753	C	T	0.01795	0.002978	1.65E-09	0.2248	111828	NA	rs9820228	NA	ieu-a-128	TRUE	reported	hp48Sr
3	85494267	C	T	0.01792	0.002978	1.76E-09	0.2445	111834	NA	rs9821041	NA	ieu-a-128	TRUE	reported	hp48Sr
3	85496631	C	T	0.01789	0.002977	1.85E-09	0.2446	111886	NA	rs1112789	NA	ieu-a-128	TRUE	reported	hp48Sr
3	85497198	T	C	0.01814	0.002976	1.10E-09	0.2251	111918	NA	rs9828679	NA	ieu-a-128	TRUE	reported	hp48Sr
3	85502083	G	A	0.01785	0.002977	2.03E-09	0.2447	111885	NA	rs9835441	NA	ieu-a-128	TRUE	reported	hp48Sr
3	85505246	A	G	0.01808	0.002976	1.24E-09	0.2252	111929	NA	rs2196098	NA	ieu-a-128	TRUE	reported	hp48Sr
3	85505874	A	G	0.01791	0.002977	1.80E-09	0.2447	111891	NA	rs9309980	NA	ieu-a-128	TRUE	reported	hp48Sr
3	85505944	A	G	0.01806	0.002977	1.33E-09	0.2444	111839	NA	rs9309981	NA	ieu-a-128	TRUE	reported	hp48Sr
3	85507231	T	C	0.01803	0.002978	1.41E-09	0.2452	111817	NA	rs9309982	NA	ieu-a-128	TRUE	reported	hp48Sr
3	85508499	A	G	0.01841	0.002997	8.24E-10	0.2489	110337	NA	rs1368740	NA	ieu-a-128	TRUE	reported	hp48Sr
3	85508582	T	C	0.01791	0.002977	1.78E-09	0.2447	111885	NA	rs1368739	NA	ieu-a-128	TRUE	reported	hp48Sr
3	85508814	A	G	0.0181	0.002977	1.21E-09	0.2252	111908	NA	rs1306843	NA	ieu-a-128	TRUE	reported	hp48Sr
3	85509064	AT	A	0.01795	0.002977	1.65E-09	0.2447	111867	NA	rs3426652	NA	ieu-a-128	TRUE	reported	hp48Sr
3	85509429	GA	G	-0.01817	0.002995	1.30E-09	0.7599	110513	NA	rs7110829	NA	ieu-a-128	TRUE	reported	hp48Sr
3	85509991	T	C	0.01829	0.00298	8.51E-10	0.245	111613	NA	rs1263376	NA	ieu-a-128	TRUE	reported	hp48Sr
3	85510183	T	C	0.01856	0.002979	4.68E-10	0.245	111702	NA	rs1112789	NA	ieu-a-128	TRUE	reported	hp48Sr
3	85512824	C	A	0.0185	0.002977	5.14E-10	0.2452	111894	NA	rs9838811	NA	ieu-a-128	TRUE	reported	hp48Sr
3	85517507	T	A	0.0182	0.00299	1.15E-09	0.2292	110887	NA	rs1307016	NA	ieu-a-128	TRUE	reported	hp48Sr
3	85531199	A	T	0.01836	0.002974	6.74E-10	0.227	112073	NA	rs9841144	NA	ieu-a-128	TRUE	reported	hp48Sr
3	85537665	A	C	0.01833	0.002974	7.20E-10	0.227	112068	NA	rs9849399	NA	ieu-a-128	TRUE	reported	hp48Sr
3	85544070	C	A	0.01825	0.002975	8.61E-10	0.2271	111997	NA	rs9844512	NA	ieu-a-128	TRUE	reported	hp48Sr
3	85553096	C	A	0.01842	0.002975	5.96E-10	0.2268	112019	NA	rs6809805	NA	ieu-a-128	TRUE	reported	hp48Sr
3	85553994	C	G	0.01798	0.002986	1.72E-09	0.2251	111224	NA	rs1308453	NA	ieu-a-128	TRUE	reported	hp48Sr
3	85569361	T	G	0.01869	0.002976	3.36E-10	0.2269	111977	NA	rs9841829	NA	ieu-a-128	TRUE	reported	hp48Sr
3	85576706	T	C	0.01852	0.002989	5.72E-10	0.2286	111000	NA	rs9851444	NA	ieu-a-128	TRUE	reported	hp48Sr
3	85590097	G	A	0.01846	0.002977	5.62E-10	0.2267	111897	NA	rs9873400	NA	ieu-a-128	TRUE	reported	hp48Sr
3	85594169	C	T	0.01839	0.002981	6.88E-10	0.2261	111566	NA	rs7628948	NA	ieu-a-128	TRUE	reported	hp48Sr
3	85594359	C	G	0.01838	0.002981	6.99E-10	0.2261	111600	NA	rs1449404	NA	ieu-a-128	TRUE	reported	hp48Sr
3	85595726	A	T	0.01835	0.002981	7.53E-10	0.2261	111575	NA	rs1309243	NA	ieu-a-128	TRUE	reported	hp48Sr
3	85596665	A	C	0.0178	0.002998	2.91E-09	0.2215	110313	NA	rs1171291	NA	ieu-a-128	TRUE	reported	hp48Sr
3	85598424	T	C	0.01841	0.002981	6.60E-10	0.2261	111593	NA	rs1271462	NA	ieu-a-128	TRUE	reported	hp48Sr
3	85604425	C	T	0.01841	0.002984	6.89E-10	0.2252	111334	NA	rs1171390	NA	ieu-a-128	TRUE	reported	hp48Sr
3	85610382	C	A	0.01835	0.002982	7.60E-10	0.226	111531	NA	rs9825916	NA	ieu-a-128	TRUE	reported	hp48Sr
3	85615144	A	G	0.01834	0.002982	7.80E-10	0.2259	111520	NA	rs9810724	NA	ieu-a-128	TRUE	reported	hp48Sr
3	85616260	A	C	0.01827	0.002982	9.07E-10	0.2259	111504	NA	rs1307766	NA	ieu-a-128	TRUE	reported	hp48Sr
3	85618445	T	C	0.01733	0.003004	8.05E-09	0.2189	109806	NA	rs1375553	NA	ieu-a-128	TRUE	reported	hp48Sr
3	85618447	G	T	0.01779	0.002994	2.80E-09	0.2217	110597	NA	rs1375552	NA	ieu-a-128	TRUE	reported	hp48Sr
3	85621395	C	T	0.01825	0.002983	9.59E-10	0.2261	111413	NA	rs1335347	NA	ieu-a-128	TRUE	reported	hp48Sr
3	85633021	A	C	0.0187	0.002983	3.66E-10	0.2265	111453	NA	rs3534446	NA	ieu-a-128	TRUE	reported	hp48Sr
3	85640815	C	G	0.01857	0.002983	4.83E-10	0.2265	111454	NA	rs9826458	NA	ieu-a-128	TRUE	reported	hp48Sr
3	85648957	G	T	0.01755	0.003008	5.38E-09	0.2249	109502	NA	rs3887138	NA	ieu-a-128	TRUE	reported	hp48Sr
3	85650004	T	C	0.01731	0.003017	9.56E-09	0.2235	108873	NA	rs9866322	NA	ieu-a-128	TRUE	reported	hp48Sr
3	85650217	T	C	0.01739	0.003016	8.10E-09	0.2235	108927	NA	rs9866761	NA	ieu-a-128	TRUE	reported	hp48Sr
3	85656222	A	C	0.01684	0.003012	2.26E-08	0.2446	109256	NA	rs9812845	NA	ieu-a-128	TRUE	reported	hp48Sr
3	85657089	G	A	0.0168	0.003011	2.44E-08	0.2445	109282	NA	rs1263568	NA	ieu-a-128	TRUE	reported	hp48Sr
3	85657402	G	C	0.01678	0.003012	2.57E-08	0.2442	109190	NA	rs9309985	NA	ieu-a-128	TRUE	reported	hp48Sr
3	85662558	C	G	0.01669	0.002986	2.27E-08	0.2465	111236	NA	rs1306168	NA	ieu-a-128	TRUE	reported	hp48Sr
3	85662925	G	T	0.01644	0.002987	3.70E-08	0.2472	111104	NA	rs1112790	NA	ieu-a-128	TRUE	reported	hp48Sr
3	85666617	C	T	0.01634	0.00299	4.66E-08	0.2462	110880	NA	rs9880010	NA	ieu-a-128	TRUE	reported	hp48Sr
3	85667586	G	A	0.01666	0.002986	2.40E-08	0.2475	111200	NA	rs1449396	NA	ieu-a-128	TRUE	reported	hp48Sr
3	85667683	C	A	0.01668	0.002986	2.32E-08	0.2471	111186	NA	rs1449395	NA	ieu-a-128	TRUE	reported	hp48Sr
3	85672935	C	A	0.0166	0.002997	3.04E-08	0.2462	110402	NA	rs9836967	NA	ieu-a-128	TRUE	reported	hp48Sr
3	85674372	G	A	0.01653	0.002997	3.50E-08	0.2463	110389	NA	rs7643091	NA	ieu-a-128	TRUE	reported	hp48Sr
3	85674658	T	C	0.01656	0.002997	3.31E-08	0.2465	110390	NA	rs1308218	NA	ieu-a-128	TRUE	reported	hp48Sr
3	85677837	A	G	0.01678	0.002998	2.17E-08	0.2461	110292	NA	rs3532668	NA	ieu-a-128	TRUE	reported	hp48Sr
3	85680716	A	G	0.01645	0.002996	4.04E-08	0.2419	110458	NA	rs9813436	NA	ieu-a-128	TRUE	reported	hp48Sr
3	85682273	A	C	0.01641	0.002996	4.33E-08	0.2418	110426	NA	rs9819476	NA	ieu-a-128	TRUE	reported	hp48Sr
3	85687785	T	G	0.01632	0.002984	4.53E-08	0.2398	111349	NA	rs9880272	NA	ieu-a-128	TRUE	reported	hp48Sr
3	85690918	T	C	0.01653	0.002991	3.26E-08	0.2405	110843	NA	rs2847548	NA	ieu-a-128	TRUE	reported	hp48Sr
3	85691434	A	G	0.01638	0.002988	4.23E-08	0.2392	111031	NA	rs1309786	NA	ieu-a-128	TRUE	reported	hp48Sr

3	85702021	G	T	0.01628	0.002984	4.91E-08	0.2398	111368	NA	rs1004910	NA	ieu-a-128	TRUE	reported	hp48Sr
3	85702336	T	C	0.01628	0.002984	4.91E-08	0.2398	111368	NA	rs9309986	NA	ieu-a-128	TRUE	reported	hp48Sr
3	85706554	A	C	0.01632	0.002982	4.44E-08	0.2404	111531	NA	rs9840636	NA	ieu-a-128	TRUE	reported	hp48Sr
3	85731444	A	G	0.01626	0.002974	4.57E-08	0.2406	112073	NA	rs1309628	NA	ieu-a-128	TRUE	reported	hp48Sr
3	85739485	CTT	C	0.01631	0.002976	4.25E-08	0.2407	111940	NA	rs1054765	NA	ieu-a-128	TRUE	reported	hp48Sr
4	39393801	C	T	0.01783	0.003023	3.68E-09	0.5692	108791	NA	rs6842066	NA	ieu-a-128	TRUE	reported	hp48Sr
4	39395570	T	C	0.01658	0.003009	3.58E-08	0.3606	109541	NA	rs4975011	NA	ieu-a-128	TRUE	reported	hp48Sr
4	39395630	T	A	0.01843	0.003009	9.09E-10	0.5549	109790	NA	rs2381376	NA	ieu-a-128	TRUE	reported	hp48Sr
4	39397439	CTTCT	C	0.01805	0.003006	1.92E-09	0.5549	110056	NA	rs1454789	NA	ieu-a-128	TRUE	reported	hp48Sr
4	39397670	G	A	-0.01764	0.003003	4.31E-09	0.427	110256	NA	rs7662509	NA	ieu-a-128	TRUE	reported	hp48Sr
4	39398625	T	C	0.01841	0.003	8.49E-10	0.554	110490	NA	rs6857705	NA	ieu-a-128	TRUE	reported	hp48Sr
4	39399404	T	C	0.01837	0.003002	9.50E-10	0.5546	110329	NA	rs1264368	NA	ieu-a-128	TRUE	reported	hp48Sr
4	39400998	G	A	0.01784	0.002986	2.32E-09	0.5545	111538	NA	rs1311079	NA	ieu-a-128	TRUE	reported	hp48Sr
4	39402525	C	T	0.01768	0.002983	3.05E-09	0.5526	111838	NA	rs7661596	NA	ieu-a-128	TRUE	reported	hp48Sr
4	39402745	A	G	0.01776	0.002984	2.68E-09	0.5527	111733	NA	rs7686103	NA	ieu-a-128	TRUE	reported	hp48Sr
4	39403243	G	A	0.0164	0.002991	4.19E-08	0.3607	110923	NA	rs5923257	NA	ieu-a-128	TRUE	reported	hp48Sr
4	39403655	TATATACA	T	0.01735	0.002987	6.24E-09	0.5516	111532	NA	rs2017184	NA	ieu-a-128	TRUE	reported	hp48Sr
4	39403731	C	T	0.01809	0.002978	1.24E-09	0.5532	112176	NA	rs3396816	NA	ieu-a-128	TRUE	reported	hp48Sr
4	39404023	G	T	0.01777	0.002981	2.52E-09	0.5526	111952	NA	rs1193753	NA	ieu-a-128	TRUE	reported	hp48Sr
4	39404306	C	T	0.0174	0.002984	5.52E-09	0.5524	111734	NA	rs2381379	NA	ieu-a-128	TRUE	reported	hp48Sr
4	39404575	G	A	0.01752	0.002984	4.33E-09	0.5519	111703	NA	rs6715298	NA	ieu-a-128	TRUE	reported	hp48Sr
4	39404872	A	G	0.01769	0.002985	3.07E-09	0.5521	111669	NA	rs1312940	NA	ieu-a-128	TRUE	reported	hp48Sr
4	39405151	T	G	0.01776	0.002981	2.58E-09	0.5526	111941	NA	rs1313543	NA	ieu-a-128	TRUE	reported	hp48Sr
4	39405240	A	T	0.01777	0.002981	2.52E-09	0.5526	111942	NA	rs1313010	NA	ieu-a-128	TRUE	reported	hp48Sr
4	39406370	G	A	0.01713	0.003003	1.17E-08	0.558	110285	NA	rs7686419	NA	ieu-a-128	TRUE	reported	hp48Sr
4	39406417	C	G	0.01736	0.003001	7.27E-09	0.5545	110456	NA	rs7687811	NA	ieu-a-128	TRUE	reported	hp48Sr
4	39406490	G	A	0.01703	0.002991	1.25E-08	0.5545	111245	NA	rs7686610	NA	ieu-a-128	TRUE	reported	hp48Sr
4	39406987	T	C	0.01808	0.002984	1.37E-09	0.5536	111742	NA	rs1314951	NA	ieu-a-128	TRUE	reported	hp48Sr
4	39410374	A	AT	-0.0178	0.003	2.99E-09	0.4268	110511	NA	rs3570401	NA	ieu-a-128	TRUE	reported	hp48Sr
4	39411407	C	T	-0.01819	0.003069	3.08E-09	0.2033	104942	NA	rs6231081	NA	ieu-a-128	TRUE	reported	hp48Sr
4	39412384	C	A	0.01761	0.003034	6.50E-09	0.3466	107716	NA	rs7323858	NA	ieu-a-128	TRUE	reported	hp48Sr
4	39412910	C	T	0.01713	0.003039	1.73E-08	0.3374	107316	NA	rs1223368	NA	ieu-a-128	TRUE	reported	hp48Sr
4	39413650	A	T	0.02152	0.00304	1.46E-12	0.3071	107184	NA	rs5801537	NA	ieu-a-128	TRUE	reported	hp48Sr
4	39413780	G	A	0.02674	0.003046	1.71E-18	0.6059	107019	NA	rs2871282	NA	ieu-a-128	TRUE	reported	hp48Sr
4	39413861	A	C	0.01914	0.00303	2.67E-10	0.2499	107951	NA	rs2046330	NA	ieu-a-128	TRUE	reported	hp48Sr
4	39414993	A	G	0.02694	0.003042	8.43E-19	0.6063	107311	NA	rs1194069	NA	ieu-a-128	TRUE	reported	hp48Sr
4	39418192	G	A	0.01708	0.002987	1.07E-08	0.2725	111306	NA	rs6836420	NA	ieu-a-128	TRUE	reported	hp48Sr
4	39418301	GA	G	-0.02593	0.003019	8.98E-18	0.3705	109127	NA	rs3551097	NA	ieu-a-128	TRUE	reported	hp48Sr
4	39418965	G	A	-0.02559	0.003018	2.32E-17	0.371	109174	NA	rs3553805	NA	ieu-a-128	TRUE	reported	hp48Sr
4	39419229	A	T	0.01719	0.002985	8.45E-09	0.2844	111439	NA	rs1003038	NA	ieu-a-128	TRUE	reported	hp48Sr
4	39419409	T	G	0.01734	0.002978	5.84E-09	0.2834	111985	NA	rs7674434	NA	ieu-a-128	TRUE	reported	hp48Sr
4	39420981	A	G	-0.0257	0.003016	1.60E-17	0.3711	109346	NA	rs1263994	NA	ieu-a-128	TRUE	reported	hp48Sr
4	39420994	A	G	0.01753	0.002975	3.80E-09	0.284	112176	NA	rs9991733	NA	ieu-a-128	TRUE	reported	hp48Sr
4	39422242	T	C	-0.02577	0.003015	1.27E-17	0.3712	109404	NA	rs1313079	NA	ieu-a-128	TRUE	reported	hp48Sr
4	39422324	A	G	-0.02573	0.003015	1.44E-17	0.3712	109409	NA	rs1312544	NA	ieu-a-128	TRUE	reported	hp48Sr
4	39423118	C	T	0.01675	0.002991	2.14E-08	0.2704	110984	NA	rs7678241	NA	ieu-a-128	TRUE	reported	hp48Sr
4	39423789	G	A	-0.02541	0.003017	3.68E-17	0.3706	109282	NA	rs4975013	NA	ieu-a-128	TRUE	reported	hp48Sr
4	39424895	T	TA	-0.02542	0.00299	1.89E-17	0.3714	111285	NA	rs3419364	NA	ieu-a-128	TRUE	reported	hp48Sr
4	39425248	A	G	-0.02542	0.002979	1.44E-17	0.3716	112176	NA	rs1314690	NA	ieu-a-128	TRUE	reported	hp48Sr
4	39426325	C	T	-0.01752	0.003213	4.97E-08	0.458	93430	NA	rs6531716	NA	ieu-a-128	TRUE	reported	hp48Sr
4	98373818	C	T	-0.01672	0.002986	2.14E-08	0.003142	111243	NA	rs1434752	NA	ieu-a-128	TRUE	reported	hp48Sr
4	98381878	CA	C	-0.01692	0.002986	1.46E-08	0.003137	111247	NA	rs5505025	NA	ieu-a-128	TRUE	reported	hp48Sr
4	98394739	C	T	-0.01692	0.002987	1.47E-08	0.00314	111158	NA	rs1382837	NA	ieu-a-128	TRUE	reported	hp48Sr
4	98452892	T	A	-0.017	0.002986	1.25E-08	0.003272	111258	NA	rs1445028	NA	ieu-a-128	TRUE	reported	hp48Sr
4	98528709	A	G	-0.0174	0.002986	5.68E-09	0.003313	111236	NA	rs1453296	NA	ieu-a-128	TRUE	reported	hp48Sr
4	98594635	T	C	-0.01666	0.002987	2.42E-08	0.003286	111217	NA	rs1483570	NA	ieu-a-128	TRUE	reported	hp48Sr
4	98744985	T	A	-0.01731	0.002986	6.77E-09	0.003295	111224	NA	rs1509558	NA	ieu-a-128	TRUE	reported	hp48Sr
4	98748279	C	G	-0.01731	0.002986	6.76E-09	0.003295	111222	NA	rs1382946	NA	ieu-a-128	TRUE	reported	hp48Sr
4	98775282	TC	T	-0.01714	0.002987	9.63E-09	0.003286	111214	NA	rs2018458	NA	ieu-a-128	TRUE	reported	hp48Sr
4	98960542	T	C	-0.01724	0.002985	7.64E-09	0.003243	111331	NA	rs1420385	NA	ieu-a-128	TRUE	reported	hp48Sr
4	98984061	T	A	-0.017	0.002985	1.23E-08	0.003243	111310	NA	rs1409749	NA	ieu-a-128	TRUE	reported	hp48Sr
4	99029109	T	C	-0.01712	0.002985	9.84E-09	0.003328	111323	NA	rs1380813	NA	ieu-a-128	TRUE	reported	hp48Sr
4	99101007	T	G	-0.01751	0.002985	4.42E-09	0.003287	111361	NA	rs1491273	NA	ieu-a-128	TRUE	reported	hp48Sr
4	99161352	A	G	-0.01785	0.002981	2.13E-09	0.003094	111657	NA	rs1401847	NA	ieu-a-128	TRUE	reported	hp48Sr
4	99267157	A	G	-0.01701	0.00298	1.15E-08	0.003188	111675	NA	rs1480375	NA	ieu-a-128	TRUE	reported	hp48Sr
4	99300438	A	C	-0.01664	0.002981	2.36E-08	0.003161	111674	NA	rs1413008	NA	ieu-a-128	TRUE	reported	hp48Sr
4	99441375	C	T	-0.01641	0.00298	3.66E-08	0.002216	111708	NA	rs1826544	NA	ieu-a-128	TRUE	reported	hp48Sr
4	99465096	A	G	-0.01959	0.002983	5.22E-11	0.003987	111493	NA	rs1430929	NA	ieu-a-128	TRUE	reported	hp48Sr
4	99567918	T	C	-0.03079	0.002998	1.01E-24	0.005011	110262	NA	rs1140262	NA	ieu-a-128	TRUE	reported	hp48Sr
4	99572723	G	T	0.01799	0.002982	1.61E-09	0.996473	111576	NA	rs4699663	NA	ieu-a-128	TRUE	reported	hp48Sr
4	99630017	C	T	-0.03105	0.002996	3.79E-25	0.00712	110458	NA	rs1930992	NA	ieu-a-128	TRUE	reported	hp48Sr
4	99644166	C	T	-0.01783	0.00298	2.17E-09	0.007253	111745	NA	rs6232547	NA	ieu-a-128	TRUE	reported	hp48Sr
4	99704167	G	A	-0.02221	0.002978	8.86E-14	0.005277	111894	NA	rs2141284	NA	ieu-a-128	TRUE	reported	hp48Sr
4	99713350	C	T	-0.02967	0.002991	3.50E-23	0.008294	110861	NA	rs1441987	NA	ieu-a-128	TRUE	reported	hp48Sr
4	99751794	A	G	-0.02966	0.002992	3.65E-23	0.007671	110809	NA	rs1454412	NA	ieu-a-128	TRUE	reported	hp48Sr
4	99941138	A	G	-0.02845	0.002993	2.05E-21	0.01341	110683	NA	rs1467880	NA	ieu-a-128	TRUE	reported	hp48Sr
4	99994405	T	C	-0.02636	0.002981	9.58E-19	0.006239	111643	NA	rs2900157	NA	ieu-a-128	TRUE	reported	hp48Sr
4	1E+08	A	C	-0.03183	0.002984	1.48E-26	0.008842	111404	NA	rs5516762	NA	ieu-a-128	TRUE	reported	hp48Sr
4	1E+08	T	C	-0.02125	0.002994	1.28E-12	0.01608	110630	NA	rs1162034	NA	ieu-a-128	TRUE	reported	hp48Sr
4	1E+08	G	A	-0.02567	0.002978	6.93E-18	0.006199	111865	NA	rs1173369	NA	ieu-a-128	TRUE	reported	hp48Sr
4	1E+08	G	C	-0.03427	0.002976	1.15E-30	0.009985	112071	NA	rs1454527	NA	ieu-a-128	TRUE	reported	hp48Sr
4	1E+08	G	A	-0.03391	0.002978	5.08E-30	0.009684	111883	NA	rs1384959	NA	ieu-a-128	TRUE	reported	hp48Sr
4	1E+08	G	A	0.01741	0.002978	5.03E-09	0.4189	112069	NA	rs1789892	NA	ieu-a-128	TRUE	reported	hp48Sr
4	1E+08	G	T	-0.01686	0.002976	1.47E-08	0.05992	112034	NA	rs1136590	NA	ieu-a-128	TRUE	reported	hp48Sr
4	1E+08	T	C	0.02036	0.002976	7.98E-12	0.8692	111953	NA	rs3114045	NA	ieu-a-128	TRUE	reported	hp48Sr
4	1E+08	A	G	0.01756	0.002977	3.69E-09	0.4188	112151	NA	rs1154433	NA	ieu-a-128	TRUE	reported	hp48Sr
4	1E+08	T	C	0.01748	0.002977	4.31E-09	0.4183	112158	NA	rs1229978	NA	ieu-a-128	TRUE	reported	hp48Sr
4	1E+08	A	G	0.01748	0.002977	4.32E-09	0.4183	112159	NA	rs1662031	NA	ieu-a-128	TRUE	reported	hp48Sr
4	1E+08	T	C	0.01748	0.002977	4.32E-09	0.4183	112159	NA	rs1612735	NA	ieu-a-128	TRUE	reported	hp48Sr
4	1E+08	A	C	0.01748	0.002977	4.32E-09	0.4183	112159	NA	rs1789898	NA	ieu-a-128	TRUE	reported	hp48Sr
4	1E+08	T	C	0.01671	0.003008	2.79E-08	0.4134	109826	NA	rs1442479	NA	ieu-a-128	TRUE	reported	hp48Sr
4	1E+08	A	C	0.01651	0.003009	4.08E-08	0.4131	109722	NA	rs1442480	NA	ieu-a-128	TRUE	reported	hp48Sr
4	1E+08	A	T	0.01745	0.002985	5.00E-09	0.416	111595	NA	rs1789900	NA	ieu-a-128	TRUE	reported	hp48Sr
4	1E+08	A	G	0.01736	0.002981	5.76E-09	0.4181	111921	NA	rs1442481	NA	ieu-a-128	TRUE	reported	hp48Sr
4	1E+08	G	A	0.01735	0.003016	8.70E-09	0.4099	109226	NA	rs1693456	NA	ieu-a-128	TRUE	reported	hp48Sr
4	1E+08	T	C	0.01756	0.00298	3.79E-09	0.4181	111958	NA	rs2851299	NA	ieu-a-128	TRUE	reported	hp48Sr
4	1E+08	T	C	0.01755	0.002978	3.79E-09	0.4181	112090	NA	rs1662060	NA	ieu-a-128	TRUE	reported	hp48Sr
4	1E+08	C	T	0.01748	0.002977	4.37E-09	0.4183	112160	NA	rs1789902	NA	ieu-a-128	TRUE	reported	hp48Sr
4	1E+08	T	C	0.01749	0.002977	4.29E-09	0.4182	112149	NA	rs1693476	NA	ieu-a-128	TRUE	reported	hp48Sr
4	1E+08	A	C	0.0175	0.002977	4.14E-09	0.4184	112150	NA	rs1662059	NA	ieu-a-128	TRUE	reported	hp48Sr
4	1E+08	G	C	0.01649	0.002989	3.42E-08	0.4165	111317	NA	rs2903199	NA	ieu-a-128	TRUE	reported	hp48Sr
4	1E+08	C	T	-0.01722	0.002978	7.42E-09	0.06164	111885	NA	rs5612228	NA	ieu-a-128	TRUE	reported	hp48Sr
4	1E+08	T	C	0.01746	0.002977	4.48E-09	0.4183	112176	NA	rs698	NA	ieu-a-128	TRUE	reported	hp48Sr
4	1E+08	C	G	0.01793	0.003004	2.40E-09	0.413	110134	NA	rs2298755	NA	ieu-a-128	TRUE	reported	hp48Sr
4	1E+08	C	CA	0.01795	0.003006	2.35E-09	0.4129	110021	NA	rs3216150	NA	ieu-a-128	TRUE	reported	hp48Sr
4	1E+08	G	A	0.01756	0.002977	3.66E-09	0.4183	112173	NA	rs3114048	NA	ieu-a-128	TRUE	reported	hp48Sr
4	1E+08	G	A	0.01757	0.002977	3.60E-09	0.4183	112167	NA	rs1693471	NA	ieu-a-128	TRUE	reported	hp48Sr

4	1E+08	C	G	0.01759	0.002977	3.48E-09	0.4179	112160	NA	rs1789903	NA	ieu-a-128	TRUE	reported	hp48Sr
4	1E+08	C	T	-0.03364	0.002976	1.34E-29	0.009713	112016	NA	rs1419739	NA	ieu-a-128	TRUE	reported	hp48Sr
4	1E+08	T	C	0.01756	0.002977	3.66E-09	0.4183	112173	NA	rs1662058	NA	ieu-a-128	TRUE	reported	hp48Sr
4	1E+08	C	T	0.01756	0.002977	3.72E-09	0.4183	112157	NA	rs1693477	NA	ieu-a-128	TRUE	reported	hp48Sr
4	1E+08	T	A	0.01765	0.003039	6.31E-09	0.4129	107514	NA	rs1789906	NA	ieu-a-128	TRUE	reported	hp48Sr
4	1E+08	T	C	0.01765	0.003039	6.30E-09	0.4128	107478	NA	rs904093	NA	ieu-a-128	TRUE	reported	hp48Sr
4	1E+08	A	G	0.01787	0.003043	4.34E-09	0.4129	107166	NA	rs904094	NA	ieu-a-128	TRUE	reported	hp48Sr
4	1E+08	A	G	0.0174	0.00302	8.32E-09	0.4094	108994	NA	rs904095	NA	ieu-a-128	TRUE	reported	hp48Sr
4	1E+08	T	C	0.01786	0.003022	3.41E-09	0.4087	108829	NA	rs1662057	NA	ieu-a-128	TRUE	reported	hp48Sr
4	1E+08	A	T	0.01767	0.003021	4.93E-09	0.4083	108855	NA	rs1789908	NA	ieu-a-128	TRUE	reported	hp48Sr
4	1E+08	T	G	0.01748	0.002981	4.56E-09	0.4186	111869	NA	rs904096	NA	ieu-a-128	TRUE	reported	hp48Sr
4	1E+08	A	G	0.01749	0.00298	4.43E-09	0.4179	111945	NA	rs1789910	NA	ieu-a-128	TRUE	reported	hp48Sr
4	1E+08	G	C	0.01762	0.002992	3.94E-09	0.416	111042	NA	rs1693480	NA	ieu-a-128	TRUE	reported	hp48Sr
4	1E+08	T	C	0.01772	0.002991	3.13E-09	0.4161	111126	NA	rs1789911	NA	ieu-a-128	TRUE	reported	hp48Sr
4	1E+08	C	T	0.0174	0.002985	5.59E-09	0.4177	111596	NA	rs1693481	NA	ieu-a-128	TRUE	reported	hp48Sr
4	1E+08	C	T	0.01775	0.003008	3.65E-09	0.4148	109842	NA	rs1789912	NA	ieu-a-128	TRUE	reported	hp48Sr
4	1E+08	C	T	0.01769	0.003009	4.16E-09	0.4146	109792	NA	rs1693482	NA	ieu-a-128	TRUE	reported	hp48Sr
4	1E+08	G	C	0.01763	0.002979	3.29E-09	0.4185	112026	NA	rs1631460	NA	ieu-a-128	TRUE	reported	hp48Sr
4	1E+08	T	C	0.01724	0.002979	7.23E-09	0.4179	112027	NA	rs1693483	NA	ieu-a-128	TRUE	reported	hp48Sr
4	1E+08	C	G	0.01724	0.002979	7.23E-09	0.4179	112027	NA	rs1693424	NA	ieu-a-128	TRUE	reported	hp48Sr
4	1E+08	C	A	0.01744	0.002983	5.06E-09	0.4175	111700	NA	rs1625439	NA	ieu-a-128	TRUE	reported	hp48Sr
4	1E+08	A	G	0.01733	0.002985	6.46E-09	0.4172	111591	NA	rs1789913	NA	ieu-a-128	TRUE	reported	hp48Sr
4	1E+08	G	A	0.0174	0.002978	5.12E-09	0.4181	112095	NA	rs6230729	NA	ieu-a-128	TRUE	reported	hp48Sr
4	1E+08	C	G	0.0174	0.002978	5.12E-09	0.4181	112095	NA	rs6230729	NA	ieu-a-128	TRUE	reported	hp48Sr
4	1E+08	G	T	0.01713	0.002988	9.84E-09	0.4158	111391	NA	rs1789914	NA	ieu-a-128	TRUE	reported	hp48Sr
4	1E+08	T	TAACTC	0.01756	0.002985	3.99E-09	0.417	111614	NA	rs3455178	NA	ieu-a-128	TRUE	reported	hp48Sr
4	1E+08	C	T	0.01744	0.002978	4.78E-09	0.4181	112092	NA	rs1693425	NA	ieu-a-128	TRUE	reported	hp48Sr
4	1E+08	A	G	0.01749	0.002979	4.35E-09	0.4181	112017	NA	rs1693426	NA	ieu-a-128	TRUE	reported	hp48Sr
4	1E+08	A	C	0.01739	0.002978	5.27E-09	0.418	112079	NA	rs1662053	NA	ieu-a-128	TRUE	reported	hp48Sr
4	1E+08	A	G	0.01736	0.002982	5.77E-09	0.4179	111822	NA	rs1662052	NA	ieu-a-128	TRUE	reported	hp48Sr
4	1E+08	G	A	0.01723	0.002983	7.73E-09	0.4184	111725	NA	rs1789916	NA	ieu-a-128	TRUE	reported	hp48Sr
4	1E+08	T	C	0.01752	0.002979	4.04E-09	0.418	112068	NA	rs1693427	NA	ieu-a-128	TRUE	reported	hp48Sr
4	1E+08	A	T	0.01744	0.002978	4.70E-09	0.4181	112107	NA	rs1693428	NA	ieu-a-128	TRUE	reported	hp48Sr
4	1E+08	A	G	-0.01765	0.002975	3.00E-09	0.06057	112083	NA	rs6532815	NA	ieu-a-128	TRUE	reported	hp48Sr
4	1E+08	G	A	0.01709	0.002982	9.97E-09	0.4173	111825	NA	rs1789917	NA	ieu-a-128	TRUE	reported	hp48Sr
4	1E+08	C	A	0.01752	0.002979	4.07E-09	0.418	112043	NA	rs1662051	NA	ieu-a-128	TRUE	reported	hp48Sr
4	1E+08	C	T	0.01745	0.002978	4.68E-09	0.4181	112107	NA	rs1693430	NA	ieu-a-128	TRUE	reported	hp48Sr
4	1E+08	A	G	0.01757	0.002987	4.07E-09	0.4146	111449	NA	rs1789919	NA	ieu-a-128	TRUE	reported	hp48Sr
4	1E+08	T	G	0.01735	0.00298	5.79E-09	0.4177	111951	NA	rs1693431	NA	ieu-a-128	TRUE	reported	hp48Sr
4	1E+08	A	C	0.02118	0.002978	1.14E-12	0.99195	111862	NA	rs283413	NA	ieu-a-128	TRUE	reported	hp48Sr
4	1E+08	A	AATTTAT	0.01747	0.002982	4.71E-09	0.4172	111802	NA	rs3473603	NA	ieu-a-128	TRUE	reported	hp48Sr
4	1E+08	A	G	0.0178	0.003004	3.14E-09	0.4247	110114	NA	rs1312541	NA	ieu-a-128	TRUE	reported	hp48Sr
4	1E+08	C	CTG	0.01787	0.002986	2.15E-09	0.4179	111532	NA	rs3508195	NA	ieu-a-128	TRUE	reported	hp48Sr
4	1E+08	TA	T	0.01741	0.003003	6.81E-09	0.4126	110196	NA	rs3592070	NA	ieu-a-128	TRUE	reported	hp48Sr
4	1E+08	A	G	0.01712	0.00298	9.12E-09	0.4173	111964	NA	rs1629270	NA	ieu-a-128	TRUE	reported	hp48Sr
4	1E+08	C	T	0.01685	0.002977	1.51E-08	0.4188	112170	NA	rs1662049	NA	ieu-a-128	TRUE	reported	hp48Sr
4	1E+08	T	C	0.01707	0.002986	1.09E-08	0.4166	111485	NA	rs1662048	NA	ieu-a-128	TRUE	reported	hp48Sr
4	1E+08	G	A	-0.0176	0.002974	3.30E-09	0.06062	112137	NA	rs3476149	NA	ieu-a-128	TRUE	reported	hp48Sr
4	1E+08	G	GACTTA	0.01687	0.002979	1.50E-08	0.4185	112011	NA	rs3522193	NA	ieu-a-128	TRUE	reported	hp48Sr
4	1E+08	C	T	0.01684	0.002977	1.55E-08	0.4188	112176	NA	rs1789924	NA	ieu-a-128	TRUE	reported	hp48Sr
4	1E+08	G	A	0.01681	0.002977	1.65E-08	0.4187	112148	NA	rs1629838	NA	ieu-a-128	TRUE	reported	hp48Sr
4	1E+08	C	G	0.01679	0.002981	1.80E-08	0.4178	111871	NA	rs2584453	NA	ieu-a-128	TRUE	reported	hp48Sr
4	1E+08	G	GAGAT	0.01671	0.002981	2.10E-08	0.4191	111843	NA	rs3547891	NA	ieu-a-128	TRUE	reported	hp48Sr
4	1E+08	T	C	0.01693	0.002978	1.33E-08	0.4183	112073	NA	rs1789925	NA	ieu-a-128	TRUE	reported	hp48Sr
4	1E+08	G	A	0.01702	0.002982	1.14E-08	0.4175	111825	NA	rs1693469	NA	ieu-a-128	TRUE	reported	hp48Sr
4	1E+08	G	A	0.01682	0.002977	1.61E-08	0.4187	112144	NA	rs1149982	NA	ieu-a-128	TRUE	reported	hp48Sr
4	1E+08	T	C	0.01681	0.002977	1.65E-08	0.4187	112148	NA	rs4093924	NA	ieu-a-128	TRUE	reported	hp48Sr
4	1E+08	C	T	0.01701	0.002982	1.17E-08	0.4195	111829	NA	rs2453980	NA	ieu-a-128	TRUE	reported	hp48Sr
4	1E+08	G	GAGA	0.01663	0.002978	2.37E-08	0.419	112072	NA	rs3602084	NA	ieu-a-128	TRUE	reported	hp48Sr
4	1E+08	T	C	-0.01757	0.002974	3.49E-09	0.06073	112176	NA	rs4699741	NA	ieu-a-128	TRUE	reported	hp48Sr
4	1E+08	C	A	0.01645	0.002982	3.48E-08	0.4189	111781	NA	rs980972	NA	ieu-a-128	TRUE	reported	hp48Sr
4	1E+08	G	A	0.01681	0.002981	1.73E-08	0.4185	111881	NA	rs1693470	NA	ieu-a-128	TRUE	reported	hp48Sr
4	1E+08	G	A	0.01669	0.00298	2.13E-08	0.4181	111932	NA	rs1662039	NA	ieu-a-128	TRUE	reported	hp48Sr
4	1E+08	C	T	0.01665	0.002983	2.39E-08	0.417	111730	NA	rs2851300	NA	ieu-a-128	TRUE	reported	hp48Sr
4	1E+08	G	C	0.01674	0.00298	1.93E-08	0.4183	111970	NA	rs1234584	NA	ieu-a-128	TRUE	reported	hp48Sr
4	1E+08	T	C	0.01678	0.003014	2.61E-08	0.4108	109345	NA	rs1662021	NA	ieu-a-128	TRUE	reported	hp48Sr
4	1E+08	T	C	0.01676	0.003014	2.68E-08	0.4109	109412	NA	rs1662020	NA	ieu-a-128	TRUE	reported	hp48Sr
4	1E+08	C	T	-0.01764	0.002974	3.03E-09	0.0606	112144	NA	rs1139097	NA	ieu-a-128	TRUE	reported	hp48Sr
4	1E+08	C	T	-0.01763	0.002974	3.07E-09	0.06062	112156	NA	rs1151799	NA	ieu-a-128	TRUE	reported	hp48Sr
4	1E+08	C	A	-0.01741	0.002975	4.91E-09	0.06131	112068	NA	rs1114059	NA	ieu-a-128	TRUE	reported	hp48Sr
4	1E+08	A	G	0.01675	0.002979	1.91E-08	0.4183	112005	NA	rs1229858	NA	ieu-a-128	TRUE	reported	hp48Sr
4	1E+08	C	T	0.01667	0.00298	2.23E-08	0.4182	111990	NA	rs1229857	NA	ieu-a-128	TRUE	reported	hp48Sr
4	1E+08	G	C	0.01665	0.002979	2.30E-08	0.4184	112045	NA	rs1229856	NA	ieu-a-128	TRUE	reported	hp48Sr
4	1E+08	G	A	-0.01751	0.002975	4.00E-09	0.06126	112055	NA	rs4699743	NA	ieu-a-128	TRUE	reported	hp48Sr
4	1E+08	G	A	-0.01764	0.002975	3.02E-09	0.06061	112107	NA	rs1478511	NA	ieu-a-128	TRUE	reported	hp48Sr
4	1E+08	A	G	0.01678	0.002984	1.89E-08	0.4174	111683	NA	rs1229855	NA	ieu-a-128	TRUE	reported	hp48Sr
4	1E+08	C	T	0.01651	0.002981	3.10E-08	0.4182	111853	NA	rs1229854	NA	ieu-a-128	TRUE	reported	hp48Sr
4	1E+08	C	T	0.01666	0.002979	2.25E-08	0.4184	112042	NA	rs1229851	NA	ieu-a-128	TRUE	reported	hp48Sr
4	1E+08	AT	A	-0.0174	0.002975	4.99E-09	0.06131	112071	NA	rs1119999	NA	ieu-a-128	TRUE	reported	hp48Sr
4	1E+08	G	A	0.01659	0.002979	2.54E-08	0.4183	112041	NA	rs1154435	NA	ieu-a-128	TRUE	reported	hp48Sr
4	1E+08	G	A	0.01719	0.002987	8.77E-09	0.417	111432	NA	rs1154438	NA	ieu-a-128	TRUE	reported	hp48Sr
4	1E+08	C	T	0.01656	0.002987	3.00E-08	0.418	111403	NA	rs1154439	NA	ieu-a-128	TRUE	reported	hp48Sr
4	1E+08	G	A	0.01682	0.002989	1.84E-08	0.4183	111253	NA	rs1154440	NA	ieu-a-128	TRUE	reported	hp48Sr
4	1E+08	C	T	-0.01713	0.002982	9.15E-09	0.05997	111582	NA	rs7692041	NA	ieu-a-128	TRUE	reported	hp48Sr
4	1E+08	C	T	0.01719	0.003013	1.16E-08	0.4183	109454	NA	rs1229973	NA	ieu-a-128	TRUE	reported	hp48Sr
4	1E+08	C	G	0.01777	0.003047	5.44E-09	0.412	106900	NA	rs1583974	NA	ieu-a-128	TRUE	reported	hp48Sr
4	1E+08	C	T	0.01789	0.003044	4.21E-09	0.4134	107103	NA	rs1154442	NA	ieu-a-128	TRUE	reported	hp48Sr
4	1E+08	T	A	0.01719	0.003041	1.58E-08	0.4172	107296	NA	rs1154444	NA	ieu-a-128	TRUE	reported	hp48Sr
4	1E+08	T	G	0.01714	0.003047	1.87E-08	0.4166	106893	NA	rs1154445	NA	ieu-a-128	TRUE	reported	hp48Sr
4	1E+08	G	T	0.01726	0.003054	1.59E-08	0.4164	106355	NA	rs1154447	NA	ieu-a-128	TRUE	reported	hp48Sr
4	1E+08	C	A	-0.01632	0.00299	4.83E-08	0.05807	110938	NA	rs1837934	NA	ieu-a-128	TRUE	reported	hp48Sr
4	1E+08	C	A	-0.01642	0.00299	4.00E-08	0.05823	110956	NA	rs7268192	NA	ieu-a-128	TRUE	reported	hp48Sr
4	1E+08	GT	G	0.0226	0.002987	3.85E-14	0.994122	111266	NA	rs2001911	NA	ieu-a-128	TRUE	reported	hp48Sr
4	1E+08	C	T	-0.02236	0.002976	5.77E-14	0.006442	112076	NA	rs1887262	NA	ieu-a-128	TRUE	reported	hp48Sr
4	1E+08	T	G	-0.02229	0.002976	6.98E-14	0.00646	112080	NA	rs5436693	NA	ieu-a-128	TRUE	reported	hp48Sr
4	1E+08	A	C	-0.02229	0.002976	6.98E-14	0.00646	112080	NA	rs1478253	NA	ieu-a-128	TRUE	reported	hp48Sr
4	1.01E+08	G	T	-0.01629	0.002977	4.45E-08	0.01986	111959	NA	rs1510949	NA	ieu-a-128	TRUE	reported	hp48Sr
4	1.01E+08	TC	T	-0.01646	0.002977	3.21E-08	0.01969	111959	NA	rs2013489	NA	ieu-a-128	TRUE	reported	hp48Sr
4	1.01E+08	A	G	-0.01646	0.002977	3.21E-08	0.01969	111959	NA	rs1134337	NA	ieu-a-128	TRUE	reported	hp48Sr
4	1.01E+08	A	T	-0.01646	0.002977	3.21E-08	0.01969	111959	NA	rs7966077	NA	ieu-a-128	TRUE	reported	hp48Sr
4	1.01E+08	C	A	-0.01633	0.002977	4.14E-08	0.01943	111931	NA	rs1120421	NA	ieu-a-128	TRUE	reported	hp48Sr
4	1.01E+08	G	A	-0.01625	0.002977	4.79E-08	0.01961	111961	NA	rs7912165	NA	ieu-a-128	TRUE	reported	hp48Sr
4	1.01E+08	C	A	-0.01628	0.002977	4.54E-08	0.01961	111957	NA	rs1496038	NA	ieu-a-128	TRUE	reported	hp48Sr
4	1.01E+08	G	A	-0.01628	0.002977	4.54E-08	0.01961	111957	NA	rs1118118	NA	ieu-a-128	TRUE	reported	hp48Sr
4	1.01E+08	C	G	-0.01628	0.002977	4.54E-08	0.01961	111957	NA	rs1120372	NA	ieu-a-128	TRUE	reported	hp48Sr
4	1.01E+08	T	C	-0.01628	0.002977	4.54E-08	0.01961	111957	NA	rs8025152	NA	ieu-a-128	TRUE	reported	hp48Sr
4	1.01E+08	A	G	-0.01628	0.002977	4.54E-08	0.01961	111957	NA	rs1130464	NA	ieu-a-128	TRUE	reported	hp48Sr

4	1.01E+08	G	A	-0.01623	0.002977	4.99E-08	0.01962	111961	NA	rs1121441	NA	ieu-a-128	TRUE	reported	hp48Sr
4	1.01E+08	C	T	-0.01623	0.002977	4.99E-08	0.01962	111961	NA	rs1138368	NA	ieu-a-128	TRUE	reported	hp48Sr
4	1.01E+08	C	T	-0.0165	0.002977	2.97E-08	0.0195	111950	NA	rs1137954	NA	ieu-a-128	TRUE	reported	hp48Sr
4	1.01E+08	TTTTTA	T	-0.01634	0.002979	4.10E-08	0.01869	111799	NA	rs2015018	NA	ieu-a-128	TRUE	reported	hp48Sr
4	1.01E+08	G	A	-0.01632	0.002977	4.23E-08	0.0194	111958	NA	rs1126731	NA	ieu-a-128	TRUE	reported	hp48Sr
4	1.01E+08	T	TA	-0.01632	0.002977	4.23E-08	0.0194	111958	NA	rs2006861	NA	ieu-a-128	TRUE	reported	hp48Sr
4	1.01E+08	G	A	-0.02271	0.002976	2.36E-14	0.006363	112057	NA	rs1489603	NA	ieu-a-128	TRUE	reported	hp48Sr
4	1.01E+08	G	A	-0.01637	0.002978	3.89E-08	0.01948	111840	NA	rs1124162	NA	ieu-a-128	TRUE	reported	hp48Sr
4	1.01E+08	A	G	-0.01632	0.002977	4.23E-08	0.0194	111958	NA	rs1127715	NA	ieu-a-128	TRUE	reported	hp48Sr
4	1.01E+08	T	C	-0.01639	0.002975	3.65E-08	0.01933	112054	NA	rs1131420	NA	ieu-a-128	TRUE	reported	hp48Sr
4	1.01E+08	G	A	-0.02311	0.002975	7.96E-15	0.006229	112144	NA	rs1508487	NA	ieu-a-128	TRUE	reported	hp48Sr
4	1.01E+08	G	A	-0.01639	0.002975	3.65E-08	0.01933	112054	NA	rs1131369	NA	ieu-a-128	TRUE	reported	hp48Sr
4	1.01E+08	C	T	-0.01634	0.002975	3.96E-08	0.01947	112118	NA	rs1126263	NA	ieu-a-128	TRUE	reported	hp48Sr
4	1.01E+08	G	A	-0.01638	0.002975	3.69E-08	0.01944	112115	NA	rs1127901	NA	ieu-a-128	TRUE	reported	hp48Sr
4	1.01E+08	CT	C	-0.01634	0.002975	3.96E-08	0.01947	112118	NA	rs2020076	NA	ieu-a-128	TRUE	reported	hp48Sr
4	1.01E+08	A	T	-0.01697	0.002976	1.19E-08	0.01888	112011	NA	rs7476423	NA	ieu-a-128	TRUE	reported	hp48Sr
4	1.01E+08	T	G	-0.01642	0.002975	3.41E-08	0.01946	112118	NA	rs1129663	NA	ieu-a-128	TRUE	reported	hp48Sr
4	1.01E+08	A	G	-0.01634	0.002976	4.01E-08	0.01895	112004	NA	rs1451173	NA	ieu-a-128	TRUE	reported	hp48Sr
4	1.01E+08	TAAAC	T	-0.01654	0.002974	2.69E-08	0.01945	112128	NA	rs1468967	NA	ieu-a-128	TRUE	reported	hp48Sr
4	1.01E+08	T	C	-0.01668	0.002974	2.06E-08	0.01947	112130	NA	rs1139527	NA	ieu-a-128	TRUE	reported	hp48Sr
4	1.01E+08	A	T	-0.01668	0.002974	2.06E-08	0.01947	112130	NA	rs1130130	NA	ieu-a-128	TRUE	reported	hp48Sr
4	1.01E+08	C	CT	-0.01911	0.002977	1.36E-10	0.00467	111994	NA	rs2005765	NA	ieu-a-128	TRUE	reported	hp48Sr
4	1.01E+08	C	A	-0.0198	0.002976	2.89E-11	0.004641	112032	NA	rs1402801	NA	ieu-a-128	TRUE	reported	hp48Sr
4	1.01E+08	C	T	-0.01967	0.002976	3.87E-11	0.004651	112028	NA	rs1502083	NA	ieu-a-128	TRUE	reported	hp48Sr
4	1.01E+08	C	T	-0.0194	0.002977	7.30E-11	0.004783	111966	NA	rs1483821	NA	ieu-a-128	TRUE	reported	hp48Sr
4	1.01E+08	C	T	-0.01937	0.002977	7.75E-11	0.004506	111961	NA	rs1500214	NA	ieu-a-128	TRUE	reported	hp48Sr

Alcohol intake frequency SNPs

chr.exposu	pos.exposu	other_allel	effect_allel	beta.expos	se.exposur	pval.expos	eaf.exposu	samplesize	ncase.expo	SNP	ncontrol.e	exposure	mr_keep.e	pval_origir	id.exposure
1	930533	C	G	-0.01722	0.00312	3.40E-08	0.386045	462346	NA	rs3128110	NA	UKB-b-57	TRUE	reported	8KoqAI
1	930567	A	G	-0.01716	0.003121	3.80E-08	0.386088	462346	NA	rs3121574	NA	UKB-b-57	TRUE	reported	8KoqAI
1	930751	C	G	-0.01734	0.003122	2.80E-08	0.385044	462346	NA	rs3128111	NA	UKB-b-57	TRUE	reported	8KoqAI
1	931166	A	G	-0.01714	0.003121	3.90E-08	0.386729	462346	NA	rs2710880	NA	UKB-b-57	TRUE	reported	8KoqAI
1	931362	G	A	-0.01747	0.003123	2.20E-08	0.385536	462346	NA	rs2799060	NA	UKB-b-57	TRUE	reported	8KoqAI
1	933790	G	A	0.017322	0.00308	1.90E-08	0.578644	462346	NA	rs9442392	NA	UKB-b-57	TRUE	reported	8KoqAI
1	936111	C	T	0.017265	0.003079	2.10E-08	0.573581	462346	NA	rs1936360	NA	UKB-b-57	TRUE	reported	8KoqAI
1	940005	A	G	-0.01825	0.00311	4.40E-09	0.399238	462346	NA	rs2799056	NA	UKB-b-57	TRUE	reported	8KoqAI
1	940096	C	T	0.018148	0.00307	3.40E-09	0.565333	462346	NA	rs4503294	NA	UKB-b-57	TRUE	reported	8KoqAI
1	941284	C	T	-0.01819	0.00311	4.90E-09	0.397373	462346	NA	rs3128116	NA	UKB-b-57	TRUE	reported	8KoqAI
1	941334	G	A	-0.01824	0.003111	4.50E-09	0.397376	462346	NA	rs5768359	NA	UKB-b-57	TRUE	reported	8KoqAI
1	941539	C	T	-0.01823	0.003111	4.60E-09	0.399568	462346	NA	rs9778087	NA	UKB-b-57	TRUE	reported	8KoqAI
1	943687	G	C	-0.01766	0.003112	1.40E-08	0.395193	462346	NA	rs2465140	NA	UKB-b-57	TRUE	reported	8KoqAI
1	943968	C	T	0.016834	0.003072	4.30E-08	0.5697	462346	NA	rs1330331	NA	UKB-b-57	TRUE	reported	8KoqAI
1	944564	T	C	-0.01782	0.003107	9.80E-09	0.396425	462346	NA	rs3128117	NA	UKB-b-57	TRUE	reported	8KoqAI
1	949608	G	A	-0.01742	0.003098	1.90E-08	0.394755	462346	NA	rs1921	NA	UKB-b-57	TRUE	reported	8KoqAI
1	950243	A	C	-0.01809	0.003105	5.70E-09	0.391689	462346	NA	rs1891906	NA	UKB-b-57	TRUE	reported	8KoqAI
1	950677	T	C	0.017522	0.003066	1.10E-08	0.571154	462346	NA	rs9331223	NA	UKB-b-57	TRUE	reported	8KoqAI
1	951564	A	G	-0.01823	0.003108	4.50E-09	0.391942	462346	NA	rs2465141	NA	UKB-b-57	TRUE	reported	8KoqAI
1	952003	G	A	-0.01822	0.003112	4.80E-09	0.389888	462346	NA	rs3128118	NA	UKB-b-57	TRUE	reported	8KoqAI
1	962891	C	T	0.017468	0.003069	1.30E-08	0.578605	462346	NA	rs4970394	NA	UKB-b-57	TRUE	reported	8KoqAI
1	970215	G	C	0.016793	0.003065	4.30E-08	0.577049	462346	NA	rs9442364	NA	UKB-b-57	TRUE	reported	8KoqAI
1	972180	G	A	0.016843	0.003068	4.00E-08	0.577449	462346	NA	rs4970350	NA	UKB-b-57	TRUE	reported	8KoqAI
1	4569436	T	A	0.019803	0.003365	4.00E-09	0.70882	462346	NA	rs780569	NA	UKB-b-57	TRUE	reported	8KoqAI
1	4571946	T	A	0.019361	0.003367	8.90E-09	0.709125	462346	NA	rs1762825	NA	UKB-b-57	TRUE	reported	8KoqAI
1	4573099	A	G	0.018384	0.003357	4.40E-08	0.706022	462346	NA	rs4480324	NA	UKB-b-57	TRUE	reported	8KoqAI
1	35356674	G	A	-0.01809	0.003155	9.90E-09	0.360077	462346	NA	rs6177635	NA	UKB-b-57	TRUE	reported	8KoqAI
1	35360078	A	G	-0.01842	0.003148	4.90E-09	0.361321	462346	NA	rs6662980	NA	UKB-b-57	TRUE	reported	8KoqAI
1	35361487	A	G	-0.01839	0.003153	5.50E-09	0.360512	462346	NA	rs5592144	NA	UKB-b-57	TRUE	reported	8KoqAI
1	35361639	C	A	-0.01847	0.003152	4.60E-09	0.360715	462346	NA	rs6177635	NA	UKB-b-57	TRUE	reported	8KoqAI
1	35363422	A	G	-0.01857	0.003164	4.40E-09	0.360457	462346	NA	rs2184187	NA	UKB-b-57	TRUE	reported	8KoqAI
1	35363679	G	A	-0.01884	0.003165	2.70E-09	0.361153	462346	NA	rs4417025	NA	UKB-b-57	TRUE	reported	8KoqAI
1	51159347	C	T	0.017203	0.003079	2.30E-08	0.406527	462346	NA	rs7515597	NA	UKB-b-57	TRUE	reported	8KoqAI
1	51181099	T	C	0.017438	0.003078	1.50E-08	0.406757	462346	NA	rs6693790	NA	UKB-b-57	TRUE	reported	8KoqAI
1	51183257	T	C	0.017411	0.003078	1.60E-08	0.40676	462346	NA	rs1120576	NA	UKB-b-57	TRUE	reported	8KoqAI
1	51188674	G	C	0.017255	0.003079	2.10E-08	0.406666	462346	NA	rs6680846	NA	UKB-b-57	TRUE	reported	8KoqAI
1	51193555	A	G	0.017396	0.003078	1.60E-08	0.406846	462346	NA	rs6668519	NA	UKB-b-57	TRUE	reported	8KoqAI
1	51199116	T	G	0.017232	0.003079	2.20E-08	0.406552	462346	NA	rs1208547	NA	UKB-b-57	TRUE	reported	8KoqAI
1	51199193	C	T	0.017252	0.003079	2.10E-08	0.406707	462346	NA	rs1012753	NA	UKB-b-57	TRUE	reported	8KoqAI
1	51216616	C	T	0.017231	0.003079	2.20E-08	0.406711	462346	NA	rs1256537	NA	UKB-b-57	TRUE	reported	8KoqAI
1	51217065	A	G	0.01738	0.003078	1.60E-08	0.406933	462346	NA	rs1138994	NA	UKB-b-57	TRUE	reported	8KoqAI
1	51218695	G	A	0.017811	0.003085	7.70E-09	0.40423	462346	NA	rs2878710	NA	UKB-b-57	TRUE	reported	8KoqAI
1	51220127	T	C	0.017348	0.003079	1.70E-08	0.406957	462346	NA	rs1120576	NA	UKB-b-57	TRUE	reported	8KoqAI
1	51223480	C	T	0.017211	0.003079	2.30E-08	0.406721	462346	NA	rs1120576	NA	UKB-b-57	TRUE	reported	8KoqAI
1	51243374	G	C	0.017011	0.00308	3.30E-08	0.410588	462346	NA	rs1416685	NA	UKB-b-57	TRUE	reported	8KoqAI
1	51245247	T	C	0.017293	0.003088	2.20E-08	0.406042	462346	NA	rs1840536	NA	UKB-b-57	TRUE	reported	8KoqAI
1	94051350	A	C	0.019667	0.003598	4.60E-08	0.232457	462346	NA	rs7514579	NA	UKB-b-57	TRUE	reported	8KoqAI
1	2.1E+08	T	G	-0.01675	0.00307	4.90E-08	0.419448	462346	NA	rs1207843	NA	UKB-b-57	TRUE	reported	8KoqAI
1	2.17E+08	T	C	-0.01838	0.003119	3.80E-09	0.605114	462346	NA	rs2244598	NA	UKB-b-57	TRUE	reported	8KoqAI
2	2191758	C	G	-0.02283	0.004166	4.30E-08	0.156521	462346	NA	rs6759325	NA	UKB-b-57	TRUE	reported	8KoqAI
2	2192750	G	A	-0.02275	0.004166	4.70E-08	0.15642	462346	NA	rs6709211	NA	UKB-b-57	TRUE	reported	8KoqAI
2	2195519	G	A	-0.02275	0.00417	4.90E-08	0.156038	462346	NA	rs1724732	NA	UKB-b-57	TRUE	reported	8KoqAI
2	2198307	C	T	-0.02274	0.00417	4.90E-08	0.156043	462346	NA	rs1247006	NA	UKB-b-57	TRUE	reported	8KoqAI
2	2202864	G	T	-0.02273	0.004167	4.90E-08	0.156267	462346	NA	rs1246764	NA	UKB-b-57	TRUE	reported	8KoqAI
2	2204378	C	T	-0.02277	0.004168	4.70E-08	0.156212	462346	NA	rs7276922	NA	UKB-b-57	TRUE	reported	8KoqAI
2	2216320	T	G	-0.02286	0.004175	4.40E-08	0.155823	462346	NA	rs1582415	NA	UKB-b-57	TRUE	reported	8KoqAI
2	2216368	G	T	-0.02286	0.004175	4.40E-08	0.155822	462346	NA	rs1582414	NA	UKB-b-57	TRUE	reported	8KoqAI
2	2217132	C	T	-0.02286	0.004175	4.40E-08	0.155843	462346	NA	rs5827596	NA	UKB-b-57	TRUE	reported	8KoqAI
2	2218209	G	A	-0.02302	0.004184	3.70E-08	0.155007	462346	NA	rs1247861	NA	UKB-b-57	TRUE	reported	8KoqAI
2	2218788	A	T	-0.02302	0.004184	3.70E-08	0.155001	462346	NA	rs1086553	NA	UKB-b-57	TRUE	reported	8KoqAI
2	2219212	C	G	-0.02273	0.00417	5.00E-08	0.15625	462346	NA	rs1592732	NA	UKB-b-57	TRUE	reported	8KoqAI
2	2220160	T	C	-0.02276	0.004172	4.90E-08	0.156264	462346	NA	rs6168837	NA	UKB-b-57	TRUE	reported	8KoqAI
2	2220237	T	G	-0.02303	0.004186	3.80E-08	0.154965	462346	NA	rs7276922	NA	UKB-b-57	TRUE	reported	8KoqAI
2	2220795	A	T	-0.02314	0.004192	3.40E-08	0.154942	462346	NA	rs7276922	NA	UKB-b-57	TRUE	reported	8KoqAI
2	2222451	G	A	-0.02309	0.004215	4.30E-08	0.155388	462346	NA	rs7420242	NA	UKB-b-57	TRUE	reported	8KoqAI
2	23833943	C	G	0.018964	0.00344	3.50E-08	0.265413	462346	NA	rs672643	NA	UKB-b-57	TRUE	reported	8KoqAI
2	23879268	A	C	0.024599	0.003729	4.20E-11	0.211099	462346	NA	rs3795936	NA	UKB-b-57	TRUE	reported	8KoqAI
2	23879304	A	G	0.024589	0.00373	4.30E-11	0.211034	462346	NA	rs6698366	NA	UKB-b-57	TRUE	reported	8KoqAI
2	23880823	A	G	0.024957	0.003731	2.20E-11	0.210574	462346	NA	rs6652386	NA	UKB-b-57	TRUE	reported	8KoqAI
2	23884746	T	C	-0.01779	0.003131	1.30E-08	0.619282	462346	NA	rs4665627	NA	UKB-b-57	TRUE	reported	8KoqAI
2	23884929	A	G	0.025	0.003722	1.90E-11	0.212035	462346	NA	rs1078345	NA	UKB-b-57	TRUE	reported	8KoqAI
2	23886149	T	C	-0.02137	0.003288	8.00E-11	0.689613	462346	NA	rs1862901	NA	UKB-b-57	TRUE	reported	8KoqAI
2	23887437	C	T	-0.02239	0.003258	6.30E-12	0.679886	462346	NA	rs1991083	NA	UKB-b-57	TRUE	reported	8KoqAI
2	23888471	C	T	-0.02159	0.003241	2.70E-11	0.673816	462346	NA	rs6733007	NA	UKB-b-57	TRUE	reported	8KoqAI
2	23889134	C	G	-0.02187	0.003247	1.60E-11	0.676208	462346	NA	rs7586023	NA	UKB-b-57	TRUE	reported	8KoqAI
2	23889287	G	C	-0.01771	0.003047	6.10E-09	0.458734	462346	NA	rs1007194	NA	UKB-b-57	TRUE	reported	8KoqAI
2	23891759	G	A	-0.02112	0.003235	6.70E-11	0.67271	462346	NA	rs1112516	NA	UKB-b-57	TRUE	reported	8KoqAI
2	23892552	T	A	0.044417	0.007962	2.40E-08	0.037649	462346	NA	rs1442409	NA	UKB-b-57	TRUE	reported	8KoqAI
2	23895107	C	T	0.044486	0.007945	2.20E-08	0.037765	462346	NA	rs1156679	NA	UKB-b-57	TRUE	reported	8KoqAI
2	23895414	C	T	-0.0196	0.003212	1.10E-09	0.665204	462346	NA	rs7588944	NA	UKB-b-57	TRUE	reported	8KoqAI
2	23895597	T	C	0.023292	0.003748	5.20E-10	0.206296	462346	NA	rs5583622	NA	UKB-b-57	TRUE	reported	8KoqAI
2	23896049	A	G	-0.02087	0.003391	7.50E-10	0.724954	462346	NA	rs1271300	NA	UKB-b-57	TRUE	reported	8KoqAI
2	23897255	T	C	0.045006	0.00797	1.60E-08	0.037424	462346	NA	rs1153282	NA	UKB-b-57	TRUE	reported	8KoqAI
2	23897996	C	T	-0.02012	0.003215	3.90E-10	0.666777	462346	NA	rs889862	NA	UKB-b-57	TRUE	reported	8KoqAI
2	23898149	A	G	0.022678	0.003903	6.20E-09	0.184214	462346	NA	rs7279429	NA	UKB-b-57	TRUE	reported	8KoqAI
2	23898765	T	G	-0.01774	0.003105	1.10E-08	0.609772	462346	NA	rs1223303	NA	UKB-b-57	TRUE	reported	8KoqAI
2	23898966	T	C	-0.01711	0.003044	1.90E-08	0.451848	462346	NA	rs1112516	NA	UKB-b-57	TRUE	reported	8KoqAI
2	23899095	T	C	0.022781	0.003839	3.00E-09	0.192331	462346	NA	rs7567043	NA	UKB-b-57	TRUE	reported	8KoqAI
2	23899807	A	G	0.023823	0.003912	1.10E-09	0.182758	462346	NA	rs6713865	NA	UKB-b-57	TRUE	reported	8KoqAI
2	23899876	A	G	0.023822	0.003912	1.10E-09	0.182751	462346	NA	rs6713979	NA	UKB-b-57	TRUE	reported	8KoqAI
2	23899948	A	G	0.02383	0.003912	1.10E-09	0.182736	462346	NA	rs6714016	NA	UKB-b-57	TRUE	reported	8KoqAI
2	23900628	G	A	0.045257	0.007968	1.30E-08	0.037413	462346	NA	rs1152629	NA	UKB-b-57	TRUE	reported	8KoqAI
2	23900938	T	C	0.023829	0.003916	1.20E-09	0.182418	462346	NA	rs5598738	NA	UKB-b-57	TRUE	reported	8KoqAI
2	23901362	A	C	0.025235	0.004051	4.70E-10	0.167408	462346	NA	rs7279610	NA	UKB-b-57	TRUE	reported	8KoqAI
2	23901766	G	A	0.045188	0.007965	1.40E-08	0.037427	462346	NA	rs1422260	NA	UKB-b-57	TRUE	reported	8KoqAI
2	23902314	T	C	0.044502	0.007961	2.30E-08	0.03747	462346	NA	rs7766954	NA	UKB-b-57	TRUE	reported	8KoqAI
2	23903085	A	C	0.044954	0.007975	1.70E-08	0.037363	462346	NA	rs1141874	NA	UKB-b-57	TRUE	reported	8KoqAI
2	23903353	G	A	0.044281	0.008034	3.60E-08	0.037	462346	NA	rs1164570	NA	UKB-b-57	TRUE	reported	8KoqAI
2	23903382	G	A	0.04498	0.007997	1.90E-08	0.037251	462346	NA	rs1494468	NA	UKB-b-57	TRUE	reported	8KoqAI
2	23903395	A	G	0.044128	0.008038	4.00E-08	0.036967	462346	NA	rs1473870	NA	UKB-b-57	TRUE	reported	8KoqAI
2	23903812	C	T	0.044986	0.007972	1.70E-08	0.037377	462346	NA	rs1386424	NA	UKB-b-57	TRUE	reported	8KoqAI
2	23904703	G	A	0.044842	0.00797	1.80E-08	0.037478	462346	NA	rs1210543	NA	UKB-b-57	TRUE	reported	8KoqAI
2	23905531	C	T	0.045051	0.007974	1.60E-08	0.037367	462346	NA	rs1495019	NA	UKB-b-57	TRUE	reported	8KoqAI

2	23905550	A	G	0.045278	0.007969	1.30E-08	0.037473	462346	NA	rs1400231	NA	UKB-b-57	TRUE	reported	8KoqAI
2	23906397	G	T	0.045195	0.007974	1.40E-08	0.037396	462346	NA	rs751313	NA	UKB-b-57	TRUE	reported	8KoqAI
2	23906551	G	C	0.044932	0.007975	1.80E-08	0.037371	462346	NA	rs751314	NA	UKB-b-57	TRUE	reported	8KoqAI
2	23907679	C	T	0.044966	0.00798	1.80E-08	0.037356	462346	NA	rs1157638	NA	UKB-b-57	TRUE	reported	8KoqAI
2	23907963	C	T	0.044919	0.00798	1.80E-08	0.037353	462346	NA	rs1146894	NA	UKB-b-57	TRUE	reported	8KoqAI
2	23909413	C	T	0.044919	0.007983	1.80E-08	0.037354	462346	NA	rs1141291	NA	UKB-b-57	TRUE	reported	8KoqAI
2	23910779	C	T	0.045125	0.008112	2.70E-08	0.036506	462346	NA	rs1704580	NA	UKB-b-57	TRUE	reported	8KoqAI
2	23912401	C	T	-0.0206	0.003475	3.10E-09	0.745	462346	NA	rs2551347	NA	UKB-b-57	TRUE	reported	8KoqAI
2	23916605	C	T	0.044964	0.007984	1.80E-08	0.037338	462346	NA	rs1704581	NA	UKB-b-57	TRUE	reported	8KoqAI
2	23918891	C	T	0.044924	0.007983	1.80E-08	0.037331	462346	NA	rs1166995	NA	UKB-b-57	TRUE	reported	8KoqAI
2	23919207	G	A	0.044892	0.007983	1.90E-08	0.03733	462346	NA	rs6174425	NA	UKB-b-57	TRUE	reported	8KoqAI
2	23919557	G	A	0.044964	0.007983	1.80E-08	0.03734	462346	NA	rs1147811	NA	UKB-b-57	TRUE	reported	8KoqAI
2	23920230	C	A	0.044851	0.007983	1.90E-08	0.037324	462346	NA	rs7995134	NA	UKB-b-57	TRUE	reported	8KoqAI
2	23923115	G	A	0.044794	0.007982	2.00E-08	0.037326	462346	NA	rs8000984	NA	UKB-b-57	TRUE	reported	8KoqAI
2	23924400	T	C	0.044891	0.007981	1.90E-08	0.037322	462346	NA	rs1434301	NA	UKB-b-57	TRUE	reported	8KoqAI
2	23927497	C	T	0.044846	0.007981	1.90E-08	0.037307	462346	NA	rs1471540	NA	UKB-b-57	TRUE	reported	8KoqAI
2	23930126	G	A	0.044822	0.007982	2.00E-08	0.037317	462346	NA	rs1161876	NA	UKB-b-57	TRUE	reported	8KoqAI
2	23931607	T	C	0.044512	0.007983	2.50E-08	0.037298	462346	NA	rs1167071	NA	UKB-b-57	TRUE	reported	8KoqAI
2	23934946	T	A	0.044283	0.007998	3.10E-08	0.037166	462346	NA	rs1704585	NA	UKB-b-57	TRUE	reported	8KoqAI
2	23935965	C	T	0.044271	0.007998	3.10E-08	0.037167	462346	NA	rs1143550	NA	UKB-b-57	TRUE	reported	8KoqAI
2	23937400	G	A	0.043947	0.007997	3.90E-08	0.037216	462346	NA	rs1494060	NA	UKB-b-57	TRUE	reported	8KoqAI
2	23938371	G	A	0.044378	0.007983	2.70E-08	0.037291	462346	NA	rs1844271	NA	UKB-b-57	TRUE	reported	8KoqAI
2	23955385	A	C	0.043118	0.007882	4.50E-08	0.038646	462346	NA	rs1420963	NA	UKB-b-57	TRUE	reported	8KoqAI
2	23998332	T	G	0.043814	0.008014	4.60E-08	0.036979	462346	NA	rs1818230	NA	UKB-b-57	TRUE	reported	8KoqAI
2	24013953	A	G	0.043973	0.008026	4.30E-08	0.036889	462346	NA	rs1165931	NA	UKB-b-57	TRUE	reported	8KoqAI
2	24022165	G	A	0.043971	0.008027	4.30E-08	0.036888	462346	NA	rs1155405	NA	UKB-b-57	TRUE	reported	8KoqAI
2	24037690	C	A	0.044568	0.008026	2.80E-08	0.036893	462346	NA	rs1413755	NA	UKB-b-57	TRUE	reported	8KoqAI
2	24106445	C	T	0.051994	0.009346	2.70E-08	0.028479	462346	NA	rs1508213	NA	UKB-b-57	TRUE	reported	8KoqAI
2	24119404	G	T	0.044734	0.008019	2.40E-08	0.036953	462346	NA	rs1144609	NA	UKB-b-57	TRUE	reported	8KoqAI
2	24171689	G	A	0.044332	0.008015	3.20E-08	0.036992	462346	NA	rs7663642	NA	UKB-b-57	TRUE	reported	8KoqAI
2	24184246	G	A	0.044236	0.008013	3.40E-08	0.03705	462346	NA	rs1494415	NA	UKB-b-57	TRUE	reported	8KoqAI
2	24202512	C	A	-0.01727	0.003148	4.10E-08	0.368679	462346	NA	rs1043263	NA	UKB-b-57	TRUE	reported	8KoqAI
2	24217341	T	C	0.044376	0.008023	3.20E-08	0.036998	462346	NA	rs8018843	NA	UKB-b-57	TRUE	reported	8KoqAI
2	24221655	G	A	0.044633	0.008111	3.70E-08	0.03668	462346	NA	rs8028387	NA	UKB-b-57	TRUE	reported	8KoqAI
2	24224048	C	T	0.044559	0.008032	2.90E-08	0.037007	462346	NA	rs1493341	NA	UKB-b-57	TRUE	reported	8KoqAI
2	24225489	T	A	0.044489	0.008032	3.00E-08	0.037012	462346	NA	rs1399776	NA	UKB-b-57	TRUE	reported	8KoqAI
2	27384710	C	T	-0.0196	0.003394	7.70E-09	0.277881	462346	NA	rs1659684	NA	UKB-b-57	TRUE	reported	8KoqAI
2	27386087	T	C	-0.0167	0.003059	4.80E-08	0.498442	462346	NA	rs2580757	NA	UKB-b-57	TRUE	reported	8KoqAI
2	27386726	A	C	0.017974	0.00324	2.90E-08	0.660042	462346	NA	rs7596471	NA	UKB-b-57	TRUE	reported	8KoqAI
2	27386799	T	C	0.019084	0.00328	5.90E-09	0.66736	462346	NA	rs1275539	NA	UKB-b-57	TRUE	reported	8KoqAI
2	27387429	T	C	-0.02132	0.003353	2.00E-10	0.29126	462346	NA	rs2384575	NA	UKB-b-57	TRUE	reported	8KoqAI
2	27389195	A	G	0.022152	0.003312	2.20E-11	0.696763	462346	NA	rs1275541	NA	UKB-b-57	TRUE	reported	8KoqAI
2	27393030	A	G	0.022348	0.003307	1.40E-11	0.696527	462346	NA	rs1659689	NA	UKB-b-57	TRUE	reported	8KoqAI
2	27398196	T	A	0.022541	0.003298	8.20E-12	0.696662	462346	NA	rs2580754	NA	UKB-b-57	TRUE	reported	8KoqAI
2	27399294	C	T	0.022603	0.003296	7.00E-12	0.696802	462346	NA	rs1659676	NA	UKB-b-57	TRUE	reported	8KoqAI
2	27404674	C	G	0.029938	0.005281	1.40E-08	0.091253	462346	NA	rs6212874	NA	UKB-b-57	TRUE	reported	8KoqAI
2	27405835	A	G	0.021994	0.003319	3.40E-11	0.706063	462346	NA	rs1821986	NA	UKB-b-57	TRUE	reported	8KoqAI
2	27406944	T	A	0.022039	0.003318	3.10E-11	0.705895	462346	NA	rs1659695	NA	UKB-b-57	TRUE	reported	8KoqAI
2	27409140	T	A	0.022023	0.003317	3.20E-11	0.705555	462346	NA	rs2439074	NA	UKB-b-57	TRUE	reported	8KoqAI
2	27412263	A	G	0.021862	0.003319	4.50E-11	0.705817	462346	NA	rs1659699	NA	UKB-b-57	TRUE	reported	8KoqAI
2	27414877	T	C	0.021766	0.003319	5.40E-11	0.706105	462346	NA	rs1659701	NA	UKB-b-57	TRUE	reported	8KoqAI
2	27416702	A	T	0.021895	0.003306	3.50E-11	0.695258	462346	NA	rs1275504	NA	UKB-b-57	TRUE	reported	8KoqAI
2	27417051	G	A	0.021827	0.003319	4.80E-11	0.706198	462346	NA	rs6055578	NA	UKB-b-57	TRUE	reported	8KoqAI
2	27419282	T	G	0.021739	0.003319	5.70E-11	0.706155	462346	NA	rs1275501	NA	UKB-b-57	TRUE	reported	8KoqAI
2	27419804	A	T	0.029518	0.00527	2.10E-08	0.091614	462346	NA	rs6213067	NA	UKB-b-57	TRUE	reported	8KoqAI
2	27420605	A	G	0.029642	0.00527	1.90E-08	0.091585	462346	NA	rs6213067	NA	UKB-b-57	TRUE	reported	8KoqAI
2	27420690	C	T	0.021795	0.003318	5.10E-11	0.70596	462346	NA	rs1659685	NA	UKB-b-57	TRUE	reported	8KoqAI
2	27424636	G	A	0.022385	0.003284	9.30E-12	0.695827	462346	NA	rs1395	NA	UKB-b-57	TRUE	reported	8KoqAI
2	27426878	C	T	0.021794	0.003318	5.10E-11	0.706114	462346	NA	rs1275522	NA	UKB-b-57	TRUE	reported	8KoqAI
2	27430781	A	G	0.021769	0.003319	5.40E-11	0.706227	462346	NA	rs1275528	NA	UKB-b-57	TRUE	reported	8KoqAI
2	27432547	A	G	0.02186	0.003319	4.50E-11	0.706041	462346	NA	rs1275530	NA	UKB-b-57	TRUE	reported	8KoqAI
2	27435374	G	A	0.021331	0.003313	1.20E-10	0.702414	462346	NA	rs11608	NA	UKB-b-57	TRUE	reported	8KoqAI
2	27437496	C	T	0.021968	0.003319	3.60E-11	0.706252	462346	NA	rs1275535	NA	UKB-b-57	TRUE	reported	8KoqAI
2	27441302	C	G	0.021972	0.003319	3.60E-11	0.706248	462346	NA	rs1275537	NA	UKB-b-57	TRUE	reported	8KoqAI
2	27441397	A	G	0.022002	0.003319	3.40E-11	0.706242	462346	NA	rs1275538	NA	UKB-b-57	TRUE	reported	8KoqAI
2	27443196	A	G	-0.02184	0.003319	4.70E-11	0.293765	462346	NA	rs4665958	NA	UKB-b-57	TRUE	reported	8KoqAI
2	27446230	T	C	-0.02185	0.003319	4.60E-11	0.29376	462346	NA	rs9309604	NA	UKB-b-57	TRUE	reported	8KoqAI
2	27449293	C	G	-0.02192	0.003319	4.00E-11	0.293855	462346	NA	rs7593448	NA	UKB-b-57	TRUE	reported	8KoqAI
2	27450724	G	A	-0.02196	0.003319	3.70E-11	0.293832	462346	NA	rs3769143	NA	UKB-b-57	TRUE	reported	8KoqAI
2	27451429	T	C	-0.02196	0.003319	3.70E-11	0.293785	462346	NA	rs5636413	NA	UKB-b-57	TRUE	reported	8KoqAI
2	27452784	T	C	-0.022	0.003319	3.40E-11	0.293781	462346	NA	rs6717980	NA	UKB-b-57	TRUE	reported	8KoqAI
2	27460968	A	G	-0.022	0.00332	3.40E-11	0.293912	462346	NA	rs1141313	NA	UKB-b-57	TRUE	reported	8KoqAI
2	27462405	T	A	-0.02207	0.00332	3.00E-11	0.293752	462346	NA	rs2014252	NA	UKB-b-57	TRUE	reported	8KoqAI
2	27471025	C	T	-0.02252	0.003338	1.50E-11	0.288642	462346	NA	rs1561535	NA	UKB-b-57	TRUE	reported	8KoqAI
2	27482090	T	C	-0.02256	0.003336	1.40E-11	0.288324	462346	NA	rs1018778	NA	UKB-b-57	TRUE	reported	8KoqAI
2	27484620	A	G	-0.02202	0.003317	3.10E-11	0.294102	462346	NA	rs1169305	NA	UKB-b-57	TRUE	reported	8KoqAI
2	27486134	G	A	0.033904	0.005015	1.40E-11	0.102676	462346	NA	rs1134072	NA	UKB-b-57	TRUE	reported	8KoqAI
2	27488432	G	C	-0.02236	0.003321	1.70E-11	0.29289	462346	NA	rs6547521	NA	UKB-b-57	TRUE	reported	8KoqAI
2	27497548	A	G	-0.02096	0.003741	2.10E-08	0.207	462346	NA	rs7281753	NA	UKB-b-57	TRUE	reported	8KoqAI
2	27497815	T	G	-0.02082	0.003722	2.20E-08	0.208396	462346	NA	rs6063162	NA	UKB-b-57	TRUE	reported	8KoqAI
2	27498844	G	A	0.029134	0.005201	2.10E-08	0.093278	462346	NA	rs7281753	NA	UKB-b-57	TRUE	reported	8KoqAI
2	27499037	A	G	-0.02194	0.003643	1.70E-09	0.221494	462346	NA	rs6213070	NA	UKB-b-57	TRUE	reported	8KoqAI
2	27500381	T	C	-0.02498	0.003451	4.60E-13	0.259222	462346	NA	rs4665961	NA	UKB-b-57	TRUE	reported	8KoqAI
2	27502445	A	G	-0.02067	0.003721	2.80E-08	0.208759	462346	NA	rs1112695	NA	UKB-b-57	TRUE	reported	8KoqAI
2	27504312	A	G	-0.0216	0.003643	3.10E-09	0.221338	462346	NA	rs1992291	NA	UKB-b-57	TRUE	reported	8KoqAI
2	27505782	C	T	-0.02054	0.003721	3.40E-08	0.208748	462346	NA	rs6213070	NA	UKB-b-57	TRUE	reported	8KoqAI
2	27508710	C	T	-0.02062	0.003722	3.10E-08	0.208531	462346	NA	rs1168613	NA	UKB-b-57	TRUE	reported	8KoqAI
2	27509032	T	C	-0.02491	0.003451	5.20E-13	0.25924	462346	NA	rs4665376	NA	UKB-b-57	TRUE	reported	8KoqAI
2	27512878	T	G	-0.02062	0.003722	3.00E-08	0.208538	462346	NA	rs9710901	NA	UKB-b-57	TRUE	reported	8KoqAI
2	27514116	A	G	-0.02056	0.003721	3.30E-08	0.208739	462346	NA	rs1205339	NA	UKB-b-57	TRUE	reported	8KoqAI
2	27515105	A	G	-0.02061	0.003722	3.10E-08	0.208537	462346	NA	rs4665962	NA	UKB-b-57	TRUE	reported	8KoqAI
2	27515527	C	T	-0.02489	0.003451	5.50E-13	0.25918	462346	NA	rs1189811	NA	UKB-b-57	TRUE	reported	8KoqAI
2	27517683	A	G	-0.02138	0.003643	4.30E-09	0.221813	462346	NA	rs6213071	NA	UKB-b-57	TRUE	reported	8KoqAI
2	27519153	G	T	-0.02486	0.003451	5.80E-13	0.259163	462346	NA	rs1340432	NA	UKB-b-57	TRUE	reported	8KoqAI
2	27519254	G	A	-0.02483	0.00345	6.10E-13	0.259191	462346	NA	rs1340444	NA	UKB-b-57	TRUE	reported	8KoqAI
2	27523089	G	A	0.029094	0.005231	2.70E-08	0.09254	462346	NA	rs6213071	NA	UKB-b-57	TRUE	reported	8KoqAI
2	27525263	A	G	-0.0237	0.003473	8.90E-12	0.255235	462346	NA	rs6760868	NA	UKB-b-57	TRUE	reported	8KoqAI
2	27525350	G	A	-0.02381	0.003475	7.20E-12	0.254501	462346	NA	rs6716850	NA	UKB-b-57	TRUE	reported	8KoqAI
2	27527321	G	A	-0.02302	0.00348	3.70E-11	0.253975	462346	NA	rs7575245	NA	UKB-b-57	TRUE	reported	8KoqAI
2	27528692	T	C	-0.02463	0.003452	9.60E-13	0.259697	462346	NA	rs4665963	NA	UKB-b-57	TRUE	reported	8KoqAI
2	27532870	A	G	-0.02061	0.003722	3.10E-08	0.209114	462346	NA	rs6213071	NA	UKB-b-57	TRUE	reported	8KoqAI
2	27536380	T	C	-0.02462	0.003459	1.10E-12	0.258224	462346	NA	rs4665965	NA	UKB-b-57	TRUE	reported	8KoqAI
2	27541053	C	G	-0.02457	0.003461	1.30E-12	0.258248	462346	NA	rs4665966	NA	UKB-b-57	TRUE	reported	8KoqAI
2	27544285	C	T	-0.02038	0.003735	4.90E-08	0.207999	462346	NA	rs1122227	NA	UKB-b-57	TRUE	reported	8KoqAI
2	27548038	A	C	-0.0274	0.003089	7.20E-19	0.398165	462346	NA	rs4665378	NA	UKB-b-57	TRUE	reported	8KoqAI
2	27550967	A	G	-0.02729	0.003087	9.40E-19	0.398106	462346	NA	rs1049817	NA	UKB-b-57	TRUE	reported	8KoqAI
2	27556721	G	A	-0.03113	0.003049	1.80E-24	0.436378	462346	NA	rs3739095	NA	UKB-b-57	TRUE	reported	8KoqAI

2	27558252	A	G	-0.02668	0.003104	8.20E-18	0.43238	462346	NA	rs1168413	NA	UKB-b-57	TRUE	reported	8KoqAI
2	27566520	T	A	-0.02354	0.003366	2.70E-12	0.281166	462346	NA	rs1168980	NA	UKB-b-57	TRUE	reported	8KoqAI
2	27566989	T	C	-0.02718	0.003091	1.40E-18	0.396962	462346	NA	rs1112697	NA	UKB-b-57	TRUE	reported	8KoqAI
2	27567407	G	T	-0.02717	0.003091	1.50E-18	0.397431	462346	NA	rs6743819	NA	UKB-b-57	TRUE	reported	8KoqAI
2	27568565	T	C	-0.02721	0.003091	1.30E-18	0.397439	462346	NA	rs1020521	NA	UKB-b-57	TRUE	reported	8KoqAI
2	27571848	C	A	-0.02725	0.003092	1.20E-18	0.397112	462346	NA	rs1127964	NA	UKB-b-57	TRUE	reported	8KoqAI
2	27574159	T	C	-0.0235	0.003366	2.90E-12	0.281147	462346	NA	rs1169395	NA	UKB-b-57	TRUE	reported	8KoqAI
2	27574953	T	C	-0.02725	0.003092	1.20E-18	0.397174	462346	NA	rs4665969	NA	UKB-b-57	TRUE	reported	8KoqAI
2	27579231	T	C	-0.02724	0.003091	1.20E-18	0.397677	462346	NA	rs6760828	NA	UKB-b-57	TRUE	reported	8KoqAI
2	27583273	A	G	-0.02705	0.003102	2.80E-18	0.38975	462346	NA	rs6716894	NA	UKB-b-57	TRUE	reported	8KoqAI
2	27584666	A	G	-0.03113	0.003047	1.70E-24	0.442667	462346	NA	rs7586601	NA	UKB-b-57	TRUE	reported	8KoqAI
2	27586155	A	C	-0.02624	0.003119	3.90E-17	0.377914	462346	NA	rs1169554	NA	UKB-b-57	TRUE	reported	8KoqAI
2	27589810	T	C	-0.02698	0.003099	3.20E-18	0.390088	462346	NA	rs2280737	NA	UKB-b-57	TRUE	reported	8KoqAI
2	27591804	T	C	-0.02609	0.003235	7.40E-16	0.32171	462346	NA	rs8005181	NA	UKB-b-57	TRUE	reported	8KoqAI
2	27592423	C	T	-0.02621	0.003102	2.90E-17	0.390203	462346	NA	rs7602534	NA	UKB-b-57	TRUE	reported	8KoqAI
2	27595756	G	C	-0.02533	0.003096	2.80E-16	0.394107	462346	NA	rs1528533	NA	UKB-b-57	TRUE	reported	8KoqAI
2	27596395	C	A	-0.02523	0.003095	3.70E-16	0.39436	462346	NA	rs6547589	NA	UKB-b-57	TRUE	reported	8KoqAI
2	27598097	T	C	-0.04866	0.003108	3.10E-55	0.604481	462346	NA	rs4665972	NA	UKB-b-57	TRUE	reported	8KoqAI
2	27599132	G	A	-0.02594	0.003091	4.70E-17	0.398856	462346	NA	rs1247542	NA	UKB-b-57	TRUE	reported	8KoqAI
2	27599875	T	G	-0.02527	0.003096	3.30E-16	0.394297	462346	NA	rs1104	NA	UKB-b-57	TRUE	reported	8KoqAI
2	27600239	G	A	-0.02615	0.003102	3.50E-17	0.389952	462346	NA	rs13472	NA	UKB-b-57	TRUE	reported	8KoqAI
2	27604279	A	G	-0.02517	0.003097	4.30E-16	0.394196	462346	NA	rs4582	NA	UKB-b-57	TRUE	reported	8KoqAI
2	27605762	G	A	-0.0262	0.003102	3.10E-17	0.390021	462346	NA	rs3434182	NA	UKB-b-57	TRUE	reported	8KoqAI
2	27608115	C	T	-0.0253	0.003096	3.00E-16	0.394368	462346	NA	rs1647284	NA	UKB-b-57	TRUE	reported	8KoqAI
2	27610917	A	G	-0.02529	0.003096	3.20E-16	0.394369	462346	NA	rs1115955	NA	UKB-b-57	TRUE	reported	8KoqAI
2	27611469	A	G	-0.02532	0.003096	2.90E-16	0.39442	462346	NA	rs7594812	NA	UKB-b-57	TRUE	reported	8KoqAI
2	27613031	C	A	-0.02628	0.003103	2.40E-17	0.390075	462346	NA	rs1247670	NA	UKB-b-57	TRUE	reported	8KoqAI
2	27614449	G	A	-0.02523	0.003122	6.40E-16	0.377878	462346	NA	rs7281951	NA	UKB-b-57	TRUE	reported	8KoqAI
2	27615294	C	T	-0.02534	0.003097	2.70E-16	0.39441	462346	NA	rs1271144	NA	UKB-b-57	TRUE	reported	8KoqAI
2	27615389	C	T	-0.02627	0.003103	2.50E-17	0.390069	462346	NA	rs1189755	NA	UKB-b-57	TRUE	reported	8KoqAI
2	27616790	T	C	-0.02537	0.003097	2.60E-16	0.394399	462346	NA	rs809058	NA	UKB-b-57	TRUE	reported	8KoqAI
2	27619049	C	G	-0.0314	0.003048	6.90E-25	0.445006	462346	NA	rs1083864	NA	UKB-b-57	TRUE	reported	8KoqAI
2	27620827	A	G	-0.02532	0.003097	3.00E-16	0.394311	462346	NA	rs1083865	NA	UKB-b-57	TRUE	reported	8KoqAI
2	27625005	G	T	-0.04144	0.007569	4.40E-08	0.085618	462346	NA	rs7585589	NA	UKB-b-57	TRUE	reported	8KoqAI
2	27625661	A	G	-0.03132	0.003046	8.60E-25	0.445579	462346	NA	rs1260338	NA	UKB-b-57	TRUE	reported	8KoqAI
2	27626017	A	T	-0.02615	0.003105	3.70E-17	0.38973	462346	NA	rs4665975	NA	UKB-b-57	TRUE	reported	8KoqAI
2	27626945	A	T	-0.02534	0.003098	2.90E-16	0.394247	462346	NA	rs2911712	NA	UKB-b-57	TRUE	reported	8KoqAI
2	27627366	G	A	-0.02538	0.003098	2.60E-16	0.394231	462346	NA	rs2950834	NA	UKB-b-57	TRUE	reported	8KoqAI
2	27631191	C	T	-0.02537	0.003098	2.70E-16	0.394208	462346	NA	rs7563162	NA	UKB-b-57	TRUE	reported	8KoqAI
2	27635463	A	G	-0.0443	0.003404	1.00E-38	0.721786	462346	NA	rs1728918	NA	UKB-b-57	TRUE	reported	8KoqAI
2	27635582	A	G	-0.02539	0.003098	2.50E-16	0.394178	462346	NA	rs1060525	NA	UKB-b-57	TRUE	reported	8KoqAI
2	27636458	C	T	-0.02638	0.003684	8.00E-13	0.299101	462346	NA	rs1128823	NA	UKB-b-57	TRUE	reported	8KoqAI
2	27637235	C	T	-0.02634	0.003105	2.10E-17	0.389949	462346	NA	rs2010087	NA	UKB-b-57	TRUE	reported	8KoqAI
2	27640325	A	G	-0.02541	0.003098	2.40E-16	0.394164	462346	NA	rs4665976	NA	UKB-b-57	TRUE	reported	8KoqAI
2	27640980	G	A	-0.02542	0.003098	2.30E-16	0.394159	462346	NA	rs2911713	NA	UKB-b-57	TRUE	reported	8KoqAI
2	27641522	C	T	-0.02638	0.003105	2.00E-17	0.389929	462346	NA	rs1169026	NA	UKB-b-57	TRUE	reported	8KoqAI
2	27643002	A	G	-0.02628	0.003105	2.60E-17	0.389827	462346	NA	rs1167556	NA	UKB-b-57	TRUE	reported	8KoqAI
2	27644464	A	C	-0.02538	0.003098	2.60E-16	0.394173	462346	NA	rs1728922	NA	UKB-b-57	TRUE	reported	8KoqAI
2	27646083	A	G	-0.02461	0.003136	4.20E-15	0.380025	462346	NA	rs5964439	NA	UKB-b-57	TRUE	reported	8KoqAI
2	27646770	C	T	-0.02538	0.003098	2.60E-16	0.394162	462346	NA	rs6547626	NA	UKB-b-57	TRUE	reported	8KoqAI
2	27647152	A	G	-0.02536	0.003098	2.70E-16	0.394119	462346	NA	rs1625123	NA	UKB-b-57	TRUE	reported	8KoqAI
2	27647576	C	T	-0.02536	0.003097	2.70E-16	0.394067	462346	NA	rs1169143	NA	UKB-b-57	TRUE	reported	8KoqAI
2	27648284	A	T	-0.02626	0.003102	2.60E-17	0.389751	462346	NA	rs1338815	NA	UKB-b-57	TRUE	reported	8KoqAI
2	27648590	A	G	-0.02538	0.003095	2.30E-16	0.394045	462346	NA	rs4665977	NA	UKB-b-57	TRUE	reported	8KoqAI
2	27648726	T	C	-0.03136	0.003042	6.40E-25	0.445236	462346	NA	rs4665978	NA	UKB-b-57	TRUE	reported	8KoqAI
2	27649375	A	G	-0.02611	0.003101	3.70E-17	0.389231	462346	NA	rs1339183	NA	UKB-b-57	TRUE	reported	8KoqAI
2	27649929	T	G	-0.03116	0.003042	1.30E-24	0.444782	462346	NA	rs1728911	NA	UKB-b-57	TRUE	reported	8KoqAI
2	27651054	A	T	-0.02468	0.003101	1.70E-15	0.389309	462346	NA	rs1647267	NA	UKB-b-57	TRUE	reported	8KoqAI
2	27651375	C	T	-0.03065	0.003046	8.10E-24	0.440638	462346	NA	rs2303370	NA	UKB-b-57	TRUE	reported	8KoqAI
2	27651693	T	C	-0.02472	0.003101	1.60E-15	0.389376	462346	NA	rs1018293	NA	UKB-b-57	TRUE	reported	8KoqAI
2	27652153	G	T	-0.02469	0.003101	1.70E-15	0.389326	462346	NA	rs780100	NA	UKB-b-57	TRUE	reported	8KoqAI
2	27655447	T	G	-0.02599	0.003101	5.20E-17	0.389276	462346	NA	rs3845686	NA	UKB-b-57	TRUE	reported	8KoqAI
2	27656036	A	G	-0.02519	0.003094	4.00E-16	0.393492	462346	NA	rs809673	NA	UKB-b-57	TRUE	reported	8KoqAI
2	27657167	T	C	-0.02519	0.003094	3.90E-16	0.393489	462346	NA	rs704791	NA	UKB-b-57	TRUE	reported	8KoqAI
2	27659491	T	C	-0.02507	0.003095	5.40E-16	0.393601	462346	NA	rs780102	NA	UKB-b-57	TRUE	reported	8KoqAI
2	27661020	T	C	-0.02519	0.003094	3.90E-16	0.393491	462346	NA	rs780103	NA	UKB-b-57	TRUE	reported	8KoqAI
2	27663215	T	A	-0.02519	0.003094	4.00E-16	0.393492	462346	NA	rs1260341	NA	UKB-b-57	TRUE	reported	8KoqAI
2	27663416	G	T	-0.02521	0.003095	3.70E-16	0.393464	462346	NA	rs1260342	NA	UKB-b-57	TRUE	reported	8KoqAI
2	27665361	C	G	-0.02536	0.003095	2.50E-16	0.393451	462346	NA	rs780112	NA	UKB-b-57	TRUE	reported	8KoqAI
2	27667297	A	G	-0.02539	0.003095	2.30E-16	0.393326	462346	NA	rs4803	NA	UKB-b-57	TRUE	reported	8KoqAI
2	27670307	G	A	-0.02602	0.003101	4.90E-17	0.388805	462346	NA	rs1112699	NA	UKB-b-57	TRUE	reported	8KoqAI
2	27671255	T	C	-0.02538	0.003095	2.40E-16	0.393266	462346	NA	rs7597145	NA	UKB-b-57	TRUE	reported	8KoqAI
2	27671257	C	T	-0.02539	0.003095	2.30E-16	0.393267	462346	NA	rs7580759	NA	UKB-b-57	TRUE	reported	8KoqAI
2	27673798	T	C	-0.02556	0.003095	1.50E-16	0.39354	462346	NA	rs1262430	NA	UKB-b-57	TRUE	reported	8KoqAI
2	27676287	A	T	-0.02621	0.003101	2.90E-17	0.38905	462346	NA	rs5607682	NA	UKB-b-57	TRUE	reported	8KoqAI
2	27677691	G	A	-0.02526	0.003095	3.20E-16	0.393227	462346	NA	rs780104	NA	UKB-b-57	TRUE	reported	8KoqAI
2	27678471	A	G	-0.03104	0.003042	1.90E-24	0.445233	462346	NA	rs780105	NA	UKB-b-57	TRUE	reported	8KoqAI
2	27679919	A	G	-0.02606	0.003101	4.30E-17	0.388947	462346	NA	rs1188778	NA	UKB-b-57	TRUE	reported	8KoqAI
2	27681598	A	C	-0.02535	0.003095	2.60E-16	0.393329	462346	NA	rs780106	NA	UKB-b-57	TRUE	reported	8KoqAI
2	27682850	G	T	-0.0262	0.003101	3.00E-17	0.388951	462346	NA	rs7281953	NA	UKB-b-57	TRUE	reported	8KoqAI
2	27684734	A	G	-0.02543	0.003095	2.10E-16	0.39347	462346	NA	rs780107	NA	UKB-b-57	TRUE	reported	8KoqAI
2	27684957	T	C	-0.03096	0.003042	2.50E-24	0.44531	462346	NA	rs780108	NA	UKB-b-57	TRUE	reported	8KoqAI
2	27685014	C	T	-0.02543	0.003095	2.10E-16	0.393466	462346	NA	rs780109	NA	UKB-b-57	TRUE	reported	8KoqAI
2	27685388	G	A	-0.03112	0.003042	1.40E-24	0.444914	462346	NA	rs780110	NA	UKB-b-57	TRUE	reported	8KoqAI
2	27686480	A	G	0.035	0.004028	3.70E-18	0.174253	462346	NA	rs6708657	NA	UKB-b-57	TRUE	reported	8KoqAI
2	27688601	C	T	-0.02534	0.003095	2.60E-16	0.393332	462346	NA	rs1647276	NA	UKB-b-57	TRUE	reported	8KoqAI
2	27689700	A	G	-0.02518	0.003095	4.10E-16	0.393312	462346	NA	rs1406295	NA	UKB-b-57	TRUE	reported	8KoqAI
2	27689899	T	C	-0.02617	0.003101	3.20E-17	0.389211	462346	NA	rs3845687	NA	UKB-b-57	TRUE	reported	8KoqAI
2	27690991	C	T	-0.02602	0.003101	4.90E-17	0.388941	462346	NA	rs1190477	NA	UKB-b-57	TRUE	reported	8KoqAI
2	27692214	T	C	-0.02533	0.003095	2.70E-16	0.393362	462346	NA	rs1728912	NA	UKB-b-57	TRUE	reported	8KoqAI
2	27692261	A	G	-0.02535	0.003095	2.60E-16	0.393332	462346	NA	rs1647265	NA	UKB-b-57	TRUE	reported	8KoqAI
2	27692631	A	G	-0.03791	0.005562	9.40E-12	0.918373	462346	NA	rs7562296	NA	UKB-b-57	TRUE	reported	8KoqAI
2	27692973	A	G	-0.0262	0.003101	3.00E-17	0.389105	462346	NA	rs1112701	NA	UKB-b-57	TRUE	reported	8KoqAI
2	27693485	T	C	-0.02535	0.003095	2.60E-16	0.393332	462346	NA	rs1647266	NA	UKB-b-57	TRUE	reported	8KoqAI
2	27696207	A	C	-0.02617	0.003102	3.20E-17	0.389263	462346	NA	rs1139549	NA	UKB-b-57	TRUE	reported	8KoqAI
2	27696737	C	T	-0.02629	0.003101	2.30E-17	0.389488	462346	NA	rs7596856	NA	UKB-b-57	TRUE	reported	8KoqAI
2	27698343	C	G	-0.02529	0.003095	3.00E-16	0.393144	462346	NA	rs780117	NA	UKB-b-57	TRUE	reported	8KoqAI
2	27703495	A	G	-0.02347	0.00307	2.10E-14	0.413542	462346	NA	rs1260345	NA	UKB-b-57	TRUE	reported	8KoqAI
2	27705369	T	C	-0.02346	0.00307	2.20E-14	0.413271	462346	NA	rs1260330	NA	UKB-b-57	TRUE	reported	8KoqAI
2	27705437	T	C	-0.02346	0.00307	2.20E-14	0.413271	462346	NA	rs1260329	NA	UKB-b-57	TRUE	reported	8KoqAI
2	27706640	T	C	-0.02422	0.003076	3.40E-15	0.408987	462346	NA	rs2272417	NA	UKB-b-57	TRUE	reported	8KoqAI
2	27709965	A	G	-0.0235	0.00307	2.00E-14	0.413247	462346	NA	rs809059	NA	UKB-b-57	TRUE	reported	8KoqAI
2	27710421	C	T	0.035758	0.006487	3.50E-08	0.059165	462346	NA	rs1300126	NA	UKB-b-57	TRUE	reported	8KoqAI
2	27711631	T	C	-0.02338	0.003071	2.60E-14	0.413798	462346	NA	rs1260328	NA	UKB-b-57	TRUE	reported	8KoqAI
2	27711893	A	G	-0.02945	0.003032	2.70E-22	0.465614	462346	NA	rs1260327	NA	UKB-b-57	TRUE	reported	8KoqAI
2	27713634	G	A	-0.02634	0.003101	2.00E-17	0.389084	462346	NA	rs1018110	NA	UKB-b-57	TRUE	reported	8KoqAI
2	27714511	A	C	-0.02554	0.003103	1.90E-16	0.391573	462346	NA	rs1647285	NA	UKB-b-57	TRUE	reported	8KoqAI

2	27714517	A	T	-0.02551	0.003103	2.00E-16	0.391519	462346	NA	rs1647286	NA	UKB-b-57	TRUE	reported	8KoqAI
2	27714526	T	C	-0.02626	0.003109	3.00E-17	0.38742	462346	NA	rs2288155	NA	UKB-b-57	TRUE	reported	8KoqAI
2	27715207	T	A	-0.02636	0.003101	1.90E-17	0.389059	462346	NA	rs8395	NA	UKB-b-57	TRUE	reported	8KoqAI
2	27715416	C	T	-0.02638	0.003102	1.80E-17	0.388883	462346	NA	rs2303369	NA	UKB-b-57	TRUE	reported	8KoqAI
2	27716004	T	C	-0.02547	0.003095	1.90E-16	0.39309	462346	NA	rs780088	NA	UKB-b-57	TRUE	reported	8KoqAI
2	27716494	G	A	-0.02545	0.003096	2.00E-16	0.392993	462346	NA	rs704795	NA	UKB-b-57	TRUE	reported	8KoqAI
2	27718474	T	C	-0.03812	0.005562	7.20E-12	0.918209	462346	NA	rs780090	NA	UKB-b-57	TRUE	reported	8KoqAI
2	27721971	T	C	-0.0255	0.003094	1.70E-16	0.394358	462346	NA	rs813592	NA	UKB-b-57	TRUE	reported	8KoqAI
2	27722416	G	A	-0.02551	0.003094	1.70E-16	0.394087	462346	NA	rs1260320	NA	UKB-b-57	TRUE	reported	8KoqAI
2	27725572	C	A	-0.0255	0.003094	1.70E-16	0.393996	462346	NA	rs715325	NA	UKB-b-57	TRUE	reported	8KoqAI
2	27725761	C	T	-0.02624	0.0031	2.50E-17	0.390118	462346	NA	rs715326	NA	UKB-b-57	TRUE	reported	8KoqAI
2	27727314	C	T	-0.02564	0.003097	1.30E-16	0.394059	462346	NA	rs1016926	NA	UKB-b-57	TRUE	reported	8KoqAI
2	27728777	C	G	-0.02655	0.003105	1.20E-17	0.389705	462346	NA	rs2293572	NA	UKB-b-57	TRUE	reported	8KoqAI
2	27729480	G	A	-0.02657	0.003105	1.10E-17	0.389776	462346	NA	rs2293571	NA	UKB-b-57	TRUE	reported	8KoqAI
2	27730940	T	C	-0.05057	0.00309	3.30E-60	0.604262	462346	NA	rs1260326	NA	UKB-b-57	TRUE	reported	8KoqAI
2	27731212	T	C	-0.03354	0.003846	2.70E-18	0.195183	462346	NA	rs3817588	NA	UKB-b-57	TRUE	reported	8KoqAI
2	27731376	C	T	-0.02623	0.003103	2.80E-17	0.389594	462346	NA	rs3507376	NA	UKB-b-57	TRUE	reported	8KoqAI
2	27733452	G	A	-0.02633	0.003104	2.20E-17	0.389724	462346	NA	rs4425043	NA	UKB-b-57	TRUE	reported	8KoqAI
2	27734972	G	A	-0.04302	0.003043	2.20E-45	0.55373	462346	NA	rs6547692	NA	UKB-b-57	TRUE	reported	8KoqAI
2	27735033	G	A	0.03586	0.006553	4.40E-08	0.058153	462346	NA	rs1414287	NA	UKB-b-57	TRUE	reported	8KoqAI
2	27737181	A	G	-0.02628	0.003103	2.50E-17	0.389808	462346	NA	rs8179225	NA	UKB-b-57	TRUE	reported	8KoqAI
2	27737900	A	T	-0.0262	0.003103	3.10E-17	0.389688	462346	NA	rs8179229	NA	UKB-b-57	TRUE	reported	8KoqAI
2	27738854	C	T	-0.02616	0.003103	3.40E-17	0.389679	462346	NA	rs1247170	NA	UKB-b-57	TRUE	reported	8KoqAI
2	27740404	C	G	-0.02618	0.003102	3.20E-17	0.389561	462346	NA	rs1112704	NA	UKB-b-57	TRUE	reported	8KoqAI
2	27741072	C	G	-0.04275	0.003048	1.10E-44	0.563824	462346	NA	rs780096	NA	UKB-b-57	TRUE	reported	8KoqAI
2	27741105	A	G	-0.04281	0.003048	8.40E-45	0.564015	462346	NA	rs780095	NA	UKB-b-57	TRUE	reported	8KoqAI
2	27741237	T	C	-0.05099	0.003105	1.30E-60	0.615206	462346	NA	rs780094	NA	UKB-b-57	TRUE	reported	8KoqAI
2	27742603	T	C	-0.05096	0.003105	1.60E-60	0.615203	462346	NA	rs780093	NA	UKB-b-57	TRUE	reported	8KoqAI
2	27743154	A	G	-0.03455	0.004118	4.80E-17	0.160324	462346	NA	rs780092	NA	UKB-b-57	TRUE	reported	8KoqAI
2	27743215	A	G	-0.0326	0.00408	1.30E-15	0.164064	462346	NA	rs814295	NA	UKB-b-57	TRUE	reported	8KoqAI
2	27743423	G	A	-0.02495	0.003087	6.30E-16	0.399128	462346	NA	rs1168135	NA	UKB-b-57	TRUE	reported	8KoqAI
2	27743630	C	T	-0.02499	0.003088	5.80E-16	0.399124	462346	NA	rs1302265	NA	UKB-b-57	TRUE	reported	8KoqAI
2	27744089	A	G	-0.03469	0.004124	4.00E-17	0.159885	462346	NA	rs7492890	NA	UKB-b-57	TRUE	reported	8KoqAI
2	27745373	G	A	0.10173	0.018227	2.40E-08	0.006923	462346	NA	rs8179249	NA	UKB-b-57	TRUE	reported	8KoqAI
2	27745946	A	G	-0.02498	0.003088	6.10E-16	0.399169	462346	NA	rs7589217	NA	UKB-b-57	TRUE	reported	8KoqAI
2	27746832	A	C	-0.02498	0.003089	6.10E-16	0.399294	462346	NA	rs8179252	NA	UKB-b-57	TRUE	reported	8KoqAI
2	27748597	C	A	-0.04271	0.003046	1.20E-44	0.563317	462346	NA	rs1260334	NA	UKB-b-57	TRUE	reported	8KoqAI
2	27748624	A	G	-0.0427	0.003046	1.20E-44	0.563303	462346	NA	rs1260333	NA	UKB-b-57	TRUE	reported	8KoqAI
2	27748782	G	C	-0.02153	0.003341	1.20E-10	0.29277	462346	NA	rs5582821	NA	UKB-b-57	TRUE	reported	8KoqAI
2	27748904	G	A	-0.04272	0.003051	1.50E-44	0.563343	462346	NA	rs1313566	NA	UKB-b-57	TRUE	reported	8KoqAI
2	27749669	G	A	-0.02507	0.0031	6.10E-16	0.399576	462346	NA	rs1168262	NA	UKB-b-57	TRUE	reported	8KoqAI
2	27750139	C	T	-0.03425	0.004152	1.60E-16	0.159297	462346	NA	rs6744393	NA	UKB-b-57	TRUE	reported	8KoqAI
2	27750545	A	G	-0.04211	0.003061	4.70E-43	0.563546	462346	NA	rs2950835	NA	UKB-b-57	TRUE	reported	8KoqAI
2	27750546	T	A	-0.04211	0.003061	4.70E-43	0.563539	462346	NA	rs2911711	NA	UKB-b-57	TRUE	reported	8KoqAI
2	27751562	A	C	-0.03396	0.004158	3.20E-16	0.158508	462346	NA	rs7868077	NA	UKB-b-57	TRUE	reported	8KoqAI
2	27752296	A	G	-0.02535	0.003102	3.00E-16	0.400439	462346	NA	rs1168208	NA	UKB-b-57	TRUE	reported	8KoqAI
2	27752871	C	T	-0.04187	0.00307	2.30E-42	0.564383	462346	NA	rs6753534	NA	UKB-b-57	TRUE	reported	8KoqAI
2	27753878	A	C	0.039717	0.003428	4.90E-31	0.273303	462346	NA	rs4665985	NA	UKB-b-57	TRUE	reported	8KoqAI
2	27755112	T	G	-0.02803	0.0037	3.60E-14	0.211974	462346	NA	rs6213187	NA	UKB-b-57	TRUE	reported	8KoqAI
2	27755825	G	A	0.04001	0.003463	7.10E-31	0.256812	462346	NA	rs4665987	NA	UKB-b-57	TRUE	reported	8KoqAI
2	27757343	A	G	-0.02813	0.003699	2.90E-14	0.211923	462346	NA	rs1018134	NA	UKB-b-57	TRUE	reported	8KoqAI
2	27761436	C	A	0.040033	0.003463	6.60E-31	0.25681	462346	NA	rs4665988	NA	UKB-b-57	TRUE	reported	8KoqAI
2	27764781	G	T	-0.02809	0.0037	3.20E-14	0.211546	462346	NA	rs6213188	NA	UKB-b-57	TRUE	reported	8KoqAI
2	27765480	T	C	-0.02809	0.0037	3.20E-14	0.211547	462346	NA	rs6213188	NA	UKB-b-57	TRUE	reported	8KoqAI
2	27765587	T	C	0.040076	0.003462	5.50E-31	0.256948	462346	NA	rs1247313	NA	UKB-b-57	TRUE	reported	8KoqAI
2	27766284	G	A	0.040125	0.003463	4.80E-31	0.256805	462346	NA	rs4665991	NA	UKB-b-57	TRUE	reported	8KoqAI
2	27768381	C	T	-0.02808	0.0037	3.20E-14	0.211538	462346	NA	rs6214127	NA	UKB-b-57	TRUE	reported	8KoqAI
2	27771415	C	T	-0.028	0.0037	3.80E-14	0.2115	462346	NA	rs6214127	NA	UKB-b-57	TRUE	reported	8KoqAI
2	27774449	G	A	-0.028	0.0037	3.80E-14	0.211503	462346	NA	rs2384627	NA	UKB-b-57	TRUE	reported	8KoqAI
2	27777651	G	T	-0.02794	0.003699	4.30E-14	0.211625	462346	NA	rs6214128	NA	UKB-b-57	TRUE	reported	8KoqAI
2	27778016	T	C	-0.02793	0.003699	4.30E-14	0.211622	462346	NA	rs6751087	NA	UKB-b-57	TRUE	reported	8KoqAI
2	27778167	A	C	0.040047	0.003464	6.40E-31	0.256473	462346	NA	rs1299546	NA	UKB-b-57	TRUE	reported	8KoqAI
2	27778560	A	G	0.039985	0.003463	7.70E-31	0.256616	462346	NA	rs1016906	NA	UKB-b-57	TRUE	reported	8KoqAI
2	27778572	G	A	0.039994	0.003463	7.50E-31	0.256612	462346	NA	rs1017987	NA	UKB-b-57	TRUE	reported	8KoqAI
2	27783392	T	C	-0.02845	0.003701	1.50E-14	0.211235	462346	NA	rs6214129	NA	UKB-b-57	TRUE	reported	8KoqAI
2	27783801	T	C	0.040107	0.003463	5.20E-31	0.256383	462346	NA	rs4665382	NA	UKB-b-57	TRUE	reported	8KoqAI
2	27784034	C	T	-0.02821	0.003699	2.50E-14	0.211459	462346	NA	rs6214129	NA	UKB-b-57	TRUE	reported	8KoqAI
2	27784300	A	C	-0.02834	0.003698	1.80E-14	0.211684	462346	NA	rs6214129	NA	UKB-b-57	TRUE	reported	8KoqAI
2	27784658	C	T	0.040047	0.003463	6.20E-31	0.256522	462346	NA	rs7570476	NA	UKB-b-57	TRUE	reported	8KoqAI
2	27786188	A	T	0.040024	0.003463	6.70E-31	0.256519	462346	NA	rs1020852	NA	UKB-b-57	TRUE	reported	8KoqAI
2	27786929	T	C	-0.0282	0.003699	2.50E-14	0.211463	462346	NA	rs6085466	NA	UKB-b-57	TRUE	reported	8KoqAI
2	27789861	T	A	-0.02835	0.003698	1.80E-14	0.211727	462346	NA	rs965813	NA	UKB-b-57	TRUE	reported	8KoqAI
2	27791555	C	G	0.040076	0.003463	5.70E-31	0.256382	462346	NA	rs4665383	NA	UKB-b-57	TRUE	reported	8KoqAI
2	27792323	A	C	-0.02824	0.003698	2.20E-14	0.211549	462346	NA	rs5987613	NA	UKB-b-57	TRUE	reported	8KoqAI
2	27793921	G	T	0.04001	0.003463	7.00E-31	0.256516	462346	NA	rs7577311	NA	UKB-b-57	TRUE	reported	8KoqAI
2	27796927	A	G	-0.02818	0.003697	2.50E-14	0.211733	462346	NA	rs6704596	NA	UKB-b-57	TRUE	reported	8KoqAI
2	27801493	T	C	0.03989	0.003463	1.00E-30	0.256472	462346	NA	rs1919127	NA	UKB-b-57	TRUE	reported	8KoqAI
2	27801759	A	G	0.040057	0.003462	5.90E-31	0.256403	462346	NA	rs1919128	NA	UKB-b-57	TRUE	reported	8KoqAI
2	27802805	A	G	-0.02838	0.0037	1.70E-14	0.211206	462346	NA	rs3811644	NA	UKB-b-57	TRUE	reported	8KoqAI
2	27808154	T	C	-0.0282	0.003697	2.40E-14	0.211676	462346	NA	rs6734059	NA	UKB-b-57	TRUE	reported	8KoqAI
2	27811722	A	G	0.039108	0.003477	2.40E-29	0.2545	462346	NA	rs1247884	NA	UKB-b-57	TRUE	reported	8KoqAI
2	27812252	G	A	0.039865	0.003462	1.10E-30	0.256469	462346	NA	rs6760250	NA	UKB-b-57	TRUE	reported	8KoqAI
2	27814374	C	T	-0.02806	0.003702	3.50E-14	0.211206	462346	NA	rs6213896	NA	UKB-b-57	TRUE	reported	8KoqAI
2	27814438	C	T	-0.02806	0.003702	3.40E-14	0.211212	462346	NA	rs6213896	NA	UKB-b-57	TRUE	reported	8KoqAI
2	27815510	A	C	0.039805	0.003464	1.50E-30	0.256916	462346	NA	rs1302287	NA	UKB-b-57	TRUE	reported	8KoqAI
2	27816315	G	A	-0.02831	0.0037	2.00E-14	0.211191	462346	NA	rs7601344	NA	UKB-b-57	TRUE	reported	8KoqAI
2	27816593	T	C	-0.02822	0.003699	2.40E-14	0.211691	462346	NA	rs6213896	NA	UKB-b-57	TRUE	reported	8KoqAI
2	27817817	G	A	0.039958	0.003463	8.50E-31	0.256341	462346	NA	rs1301058	NA	UKB-b-57	TRUE	reported	8KoqAI
2	27818721	C	G	-0.0283	0.003694	1.90E-14	0.212201	462346	NA	rs1919129	NA	UKB-b-57	TRUE	reported	8KoqAI
2	27819172	G	A	-0.02815	0.003698	2.70E-14	0.211605	462346	NA	rs6213896	NA	UKB-b-57	TRUE	reported	8KoqAI
2	27821482	C	T	0.04009	0.003463	5.40E-31	0.256411	462346	NA	rs1298967	NA	UKB-b-57	TRUE	reported	8KoqAI
2	27824168	G	C	-0.0282	0.003698	2.40E-14	0.211584	462346	NA	rs5672535	NA	UKB-b-57	TRUE	reported	8KoqAI
2	27824485	C	G	-0.02821	0.003698	2.40E-14	0.211563	462346	NA	rs6213896	NA	UKB-b-57	TRUE	reported	8KoqAI
2	27825715	T	C	0.040203	0.003466	4.10E-31	0.255932	462346	NA	rs1246747	NA	UKB-b-57	TRUE	reported	8KoqAI
2	27827092	C	T	0.040246	0.003465	3.50E-31	0.255881	462346	NA	rs1246461	NA	UKB-b-57	TRUE	reported	8KoqAI
2	27827196	G	T	-0.02844	0.003694	1.40E-14	0.212308	462346	NA	rs2141372	NA	UKB-b-57	TRUE	reported	8KoqAI
2	27830067	A	G	-0.02846	0.003693	1.30E-14	0.212468	462346	NA	rs6719960	NA	UKB-b-57	TRUE	reported	8KoqAI
2	27831607	T	C	-0.02847	0.003693	1.30E-14	0.212463	462346	NA	rs6547735	NA	UKB-b-57	TRUE	reported	8KoqAI
2	27832055	A	G	0.040102	0.003465	5.60E-31	0.256081	462346	NA	rs2384656	NA	UKB-b-57	TRUE	reported	8KoqAI
2	27832285	A	G	-0.02684	0.003598	8.70E-14	0.230094	462346	NA	rs1881397	NA	UKB-b-57	TRUE	reported	8KoqAI
2	27833502	T	C	0.040186	0.003465	4.20E-31	0.256145	462346	NA	rs7417506	NA	UKB-b-57	TRUE	reported	8KoqAI
2	27833687	C	T	0.040216	0.003464	3.70E-31	0.256105	462346	NA	rs3401699	NA	UKB-b-57	TRUE	reported	8KoqAI
2	27834152	T	C	0.040219	0.003464	3.70E-31	0.256099	462346	NA	rs1298705	NA	UKB-b-57	TRUE	reported	8KoqAI
2	27834615	A	C	-0.02838	0.003695	1.60E-14	0.212157	462346	NA	rs1340576	NA	UKB-b-57	TRUE	reported	8KoqAI
2	27835082	G	A	-0.02835	0.003701	1.80E-14	0.211025	462346	NA	rs7817028	NA	UKB-b-57	TRUE	reported	8KoqAI
2	27836605	C	T	0.040237	0.003464	3.40E-31	0.256156	462346	NA	rs1302094	NA	UKB-b-57	TRUE	reported	8KoqAI
2	27838549	G	A	-0.02829	0.003698	2.00E-14	0.211784	462346	NA	rs1881395	NA	UKB-b-57	TRUE	reported	8KoqAI

2	27839369	T	C	0.037556	0.003402	2.40E-28	0.270825	462346	NA	rs4666000	NA	UKB-b-57	TRUE	reported	8KoqAI
2	27839539	T	C	0.037537	0.003402	2.60E-28	0.27081	462346	NA	rs2068834	NA	UKB-b-57	TRUE	reported	8KoqAI
2	27839832	C	G	-0.02845	0.003695	1.40E-14	0.212205	462346	NA	rs7571558	NA	UKB-b-57	TRUE	reported	8KoqAI
2	27840640	G	C	0.040187	0.003465	4.20E-31	0.256116	462346	NA	rs4666002	NA	UKB-b-57	TRUE	reported	8KoqAI
2	27840754	G	A	-0.02827	0.003698	2.10E-14	0.211698	462346	NA	rs6213897	NA	UKB-b-57	TRUE	reported	8KoqAI
2	27844601	T	G	-0.02908	0.003689	3.20E-15	0.212849	462346	NA	rs1881396	NA	UKB-b-57	TRUE	reported	8KoqAI
2	27851918	G	A	0.039614	0.003483	5.70E-30	0.251827	462346	NA	rs3749147	NA	UKB-b-57	TRUE	reported	8KoqAI
2	27853245	C	G	0.039573	0.003483	6.60E-30	0.25209	462346	NA	rs1300285	NA	UKB-b-57	TRUE	reported	8KoqAI
2	27854524	G	A	0.039569	0.003483	6.50E-30	0.252175	462346	NA	rs3450205	NA	UKB-b-57	TRUE	reported	8KoqAI
2	27855397	A	T	-0.02682	0.003667	2.60E-13	0.216402	462346	NA	rs1017372	NA	UKB-b-57	TRUE	reported	8KoqAI
2	27859351	T	A	0.036743	0.003417	5.70E-27	0.269295	462346	NA	rs6719175	NA	UKB-b-57	TRUE	reported	8KoqAI
2	27860258	G	A	0.027534	0.003378	3.60E-16	0.695927	462346	NA	rs2141371	NA	UKB-b-57	TRUE	reported	8KoqAI
2	27866139	A	G	-0.02672	0.003671	3.40E-13	0.215918	462346	NA	rs7735036	NA	UKB-b-57	TRUE	reported	8KoqAI
2	27871566	G	A	-0.02285	0.003286	3.50E-12	0.317231	462346	NA	rs1131602	NA	UKB-b-57	TRUE	reported	8KoqAI
2	27871593	C	T	-0.02662	0.00367	4.10E-13	0.215985	462346	NA	rs1247458	NA	UKB-b-57	TRUE	reported	8KoqAI
2	27873326	C	G	-0.02684	0.003669	2.60E-13	0.215983	462346	NA	rs8731	NA	UKB-b-57	TRUE	reported	8KoqAI
2	27878686	T	C	-0.02699	0.00367	1.90E-13	0.215918	462346	NA	rs4666010	NA	UKB-b-57	TRUE	reported	8KoqAI
2	27892023	A	T	0.03862	0.003756	8.50E-25	0.203621	462346	NA	rs2272406	NA	UKB-b-57	TRUE	reported	8KoqAI
2	27895073	C	T	0.032242	0.004552	1.40E-12	0.128929	462346	NA	rs2178198	NA	UKB-b-57	TRUE	reported	8KoqAI
2	27901728	C	T	0.038759	0.003757	5.90E-25	0.203595	462346	NA	rs1302120	NA	UKB-b-57	TRUE	reported	8KoqAI
2	27910706	A	C	0.03858	0.003756	9.40E-25	0.203808	462346	NA	rs1302309	NA	UKB-b-57	TRUE	reported	8KoqAI
2	27913188	T	C	0.038692	0.003757	7.20E-25	0.203641	462346	NA	rs1299877	NA	UKB-b-57	TRUE	reported	8KoqAI
2	27928797	T	C	0.038024	0.003744	3.10E-24	0.205836	462346	NA	rs1303097	NA	UKB-b-57	TRUE	reported	8KoqAI
2	27932587	A	G	0.037942	0.003745	4.00E-24	0.205686	462346	NA	rs6727388	NA	UKB-b-57	TRUE	reported	8KoqAI
2	27933642	C	T	0.03788	0.003745	4.80E-24	0.205699	462346	NA	rs4616435	NA	UKB-b-57	TRUE	reported	8KoqAI
2	27934524	T	C	-0.02759	0.003425	7.80E-16	0.263832	462346	NA	rs5819997	NA	UKB-b-57	TRUE	reported	8KoqAI
2	27934731	G	A	0.037915	0.003745	4.40E-24	0.2056	462346	NA	rs6727215	NA	UKB-b-57	TRUE	reported	8KoqAI
2	27935352	C	A	-0.02595	0.00429	1.50E-09	0.145988	462346	NA	rs4665386	NA	UKB-b-57	TRUE	reported	8KoqAI
2	27941888	A	G	0.026899	0.003673	2.40E-13	0.783926	462346	NA	rs6742571	NA	UKB-b-57	TRUE	reported	8KoqAI
2	27950837	G	A	0.037505	0.003743	1.20E-23	0.207276	462346	NA	rs1043270	NA	UKB-b-57	TRUE	reported	8KoqAI
2	27951658	T	C	0.026665	0.003608	1.50E-13	0.772236	462346	NA	rs867282	NA	UKB-b-57	TRUE	reported	8KoqAI
2	27952235	A	T	0.026815	0.003573	6.10E-14	0.765656	462346	NA	rs2384655	NA	UKB-b-57	TRUE	reported	8KoqAI
2	27952406	C	T	0.026756	0.003572	6.90E-14	0.765523	462346	NA	rs1299408	NA	UKB-b-57	TRUE	reported	8KoqAI
2	27952767	G	C	0.02675	0.003573	7.00E-14	0.765599	462346	NA	rs1299337	NA	UKB-b-57	TRUE	reported	8KoqAI
2	27956481	C	T	-0.02651	0.00361	2.10E-13	0.227461	462346	NA	rs1262414	NA	UKB-b-57	TRUE	reported	8KoqAI
2	27958855	C	G	0.026463	0.003572	1.30E-13	0.765504	462346	NA	rs6753736	NA	UKB-b-57	TRUE	reported	8KoqAI
2	27960776	C	T	0.029392	0.004569	1.20E-10	0.125289	462346	NA	rs6213901	NA	UKB-b-57	TRUE	reported	8KoqAI
2	27961344	G	C	0.020734	0.003475	2.40E-09	0.25929	462346	NA	rs1317580	NA	UKB-b-57	TRUE	reported	8KoqAI
2	27961614	T	C	-0.02212	0.003925	1.70E-08	0.180544	462346	NA	rs7542981	NA	UKB-b-57	TRUE	reported	8KoqAI
2	27963133	C	T	-0.02436	0.00351	3.90E-12	0.246358	462346	NA	rs1119386	NA	UKB-b-57	TRUE	reported	8KoqAI
2	27963330	G	A	0.024317	0.003476	2.60E-12	0.745955	462346	NA	rs1271422	NA	UKB-b-57	TRUE	reported	8KoqAI
2	27964328	G	C	-0.02459	0.003478	1.60E-12	0.253564	462346	NA	rs9678824	NA	UKB-b-57	TRUE	reported	8KoqAI
2	27967260	G	C	0.036831	0.004034	6.80E-20	0.171088	462346	NA	rs1302319	NA	UKB-b-57	TRUE	reported	8KoqAI
2	27968454	A	C	0.024258	0.003479	3.10E-12	0.746818	462346	NA	rs9967838	NA	UKB-b-57	TRUE	reported	8KoqAI
2	27972445	G	A	0.024287	0.003475	2.80E-12	0.746038	462346	NA	rs9677663	NA	UKB-b-57	TRUE	reported	8KoqAI
2	27972833	A	G	0.029583	0.004665	2.30E-10	0.122053	462346	NA	rs1210444	NA	UKB-b-57	TRUE	reported	8KoqAI
2	27974260	C	G	-0.02458	0.003478	1.60E-12	0.253399	462346	NA	rs5723888	NA	UKB-b-57	TRUE	reported	8KoqAI
2	27974971	A	G	0.024303	0.003475	2.70E-12	0.746092	462346	NA	rs1020124	NA	UKB-b-57	TRUE	reported	8KoqAI
2	27975394	T	C	0.024274	0.003475	2.80E-12	0.74607	462346	NA	rs6547796	NA	UKB-b-57	TRUE	reported	8KoqAI
2	27980912	G	C	-0.0246	0.003509	2.40E-12	0.246277	462346	NA	rs4666011	NA	UKB-b-57	TRUE	reported	8KoqAI
2	27980944	C	T	0.024594	0.003478	1.50E-12	0.746975	462346	NA	rs1020902	NA	UKB-b-57	TRUE	reported	8KoqAI
2	27981655	G	C	-0.02458	0.003509	2.50E-12	0.246267	462346	NA	rs1247134	NA	UKB-b-57	TRUE	reported	8KoqAI
2	27982119	A	G	-0.02478	0.00348	1.10E-12	0.252701	462346	NA	rs7715438	NA	UKB-b-57	TRUE	reported	8KoqAI
2	27987046	A	C	0.024244	0.00343	1.60E-12	0.73646	462346	NA	rs1247790	NA	UKB-b-57	TRUE	reported	8KoqAI
2	27987221	C	G	0.037995	0.004027	3.90E-21	0.172204	462346	NA	rs1300648	NA	UKB-b-57	TRUE	reported	8KoqAI
2	27988821	G	A	0.02551	0.003399	6.20E-14	0.727135	462346	NA	rs1301348	NA	UKB-b-57	TRUE	reported	8KoqAI
2	27989909	C	T	-0.02463	0.003458	1.10E-12	0.256908	462346	NA	rs4665390	NA	UKB-b-57	TRUE	reported	8KoqAI
2	27995781	A	C	0.024434	0.003424	9.60E-13	0.735291	462346	NA	rs3736594	NA	UKB-b-57	TRUE	reported	8KoqAI
2	27995931	G	A	0.024616	0.00345	9.70E-13	0.741422	462346	NA	rs3792252	NA	UKB-b-57	TRUE	reported	8KoqAI
2	28003174	G	T	0.036532	0.004038	1.50E-19	0.171361	462346	NA	rs1303034	NA	UKB-b-57	TRUE	reported	8KoqAI
2	28017792	A	G	-0.02091	0.003704	1.70E-08	0.210949	462346	NA	rs7593136	NA	UKB-b-57	TRUE	reported	8KoqAI
2	28018025	A	C	-0.02119	0.003707	1.10E-08	0.210444	462346	NA	rs6744348	NA	UKB-b-57	TRUE	reported	8KoqAI
2	28018131	T	G	-0.02128	0.003751	1.40E-08	0.203768	462346	NA	rs3603582	NA	UKB-b-57	TRUE	reported	8KoqAI
2	28018293	A	G	-0.02155	0.003749	9.10E-09	0.203984	462346	NA	rs4666012	NA	UKB-b-57	TRUE	reported	8KoqAI
2	28019106	C	T	-0.02115	0.003706	1.20E-08	0.210447	462346	NA	rs4666013	NA	UKB-b-57	TRUE	reported	8KoqAI
2	28019175	G	A	-0.02092	0.003703	1.60E-08	0.210994	462346	NA	rs4666014	NA	UKB-b-57	TRUE	reported	8KoqAI
2	28019367	G	A	-0.0212	0.003707	1.10E-08	0.210471	462346	NA	rs4666015	NA	UKB-b-57	TRUE	reported	8KoqAI
2	28019856	T	C	-0.02112	0.003707	1.20E-08	0.210564	462346	NA	rs3418167	NA	UKB-b-57	TRUE	reported	8KoqAI
2	28019966	T	C	-0.02138	0.00375	1.20E-08	0.204629	462346	NA	rs3567950	NA	UKB-b-57	TRUE	reported	8KoqAI
2	28020157	C	T	-0.0211	0.003706	1.20E-08	0.210425	462346	NA	rs1262317	NA	UKB-b-57	TRUE	reported	8KoqAI
2	28020966	T	A	-0.02096	0.003752	2.30E-08	0.20348	462346	NA	rs3935148	NA	UKB-b-57	TRUE	reported	8KoqAI
2	28021043	G	T	-0.02059	0.003706	2.70E-08	0.210418	462346	NA	rs1302742	NA	UKB-b-57	TRUE	reported	8KoqAI
2	28022675	G	A	-0.02074	0.003703	2.10E-08	0.210638	462346	NA	rs7570730	NA	UKB-b-57	TRUE	reported	8KoqAI
2	28023120	T	C	-0.02066	0.003702	2.40E-08	0.210807	462346	NA	rs1112712	NA	UKB-b-57	TRUE	reported	8KoqAI
2	28023284	C	T	-0.02047	0.0037	3.10E-08	0.211175	462346	NA	rs1112712	NA	UKB-b-57	TRUE	reported	8KoqAI
2	28023797	G	A	-0.02091	0.00375	2.40E-08	0.203664	462346	NA	rs1247334	NA	UKB-b-57	TRUE	reported	8KoqAI
2	28024024	G	T	-0.02071	0.003704	2.20E-08	0.210647	462346	NA	rs1247340	NA	UKB-b-57	TRUE	reported	8KoqAI
2	28024642	C	T	-0.02091	0.00375	2.40E-08	0.203671	462346	NA	rs1301903	NA	UKB-b-57	TRUE	reported	8KoqAI
2	28024984	T	C	-0.02077	0.003704	2.10E-08	0.210652	462346	NA	rs4632296	NA	UKB-b-57	TRUE	reported	8KoqAI
2	28025061	T	C	-0.02033	0.003715	4.40E-08	0.209738	462346	NA	rs1300106	NA	UKB-b-57	TRUE	reported	8KoqAI
2	28025066	A	C	-0.02032	0.003715	4.50E-08	0.209738	462346	NA	rs1299586	NA	UKB-b-57	TRUE	reported	8KoqAI
2	28025580	G	C	-0.02048	0.003701	3.20E-08	0.211185	462346	NA	rs1189176	NA	UKB-b-57	TRUE	reported	8KoqAI
2	28028191	C	T	-0.02049	0.003702	3.10E-08	0.211192	462346	NA	rs1246501	NA	UKB-b-57	TRUE	reported	8KoqAI
2	28029649	A	G	-0.0204	0.003701	3.60E-08	0.211334	462346	NA	rs4665392	NA	UKB-b-57	TRUE	reported	8KoqAI
2	28030437	A	G	-0.02039	0.003702	3.60E-08	0.211353	462346	NA	rs1298698	NA	UKB-b-57	TRUE	reported	8KoqAI
2	28032137	G	C	-0.02056	0.003751	4.20E-08	0.204075	462346	NA	rs1302300	NA	UKB-b-57	TRUE	reported	8KoqAI
2	28066122	T	C	0.033133	0.003974	7.70E-17	0.178029	462346	NA	rs7444611	NA	UKB-b-57	TRUE	reported	8KoqAI
2	28085544	C	A	0.032535	0.003978	2.90E-16	0.177038	462346	NA	rs7735356	NA	UKB-b-57	TRUE	reported	8KoqAI
2	28113911	A	G	0.032508	0.00398	3.10E-16	0.176749	462346	NA	rs2305929	NA	UKB-b-57	TRUE	reported	8KoqAI
2	28244926	G	C	0.030563	0.004715	9.10E-11	0.116475	462346	NA	rs6214039	NA	UKB-b-57	TRUE	reported	8KoqAI
2	28246841	G	A	0.031004	0.004718	5.00E-11	0.116282	462346	NA	rs6214039	NA	UKB-b-57	TRUE	reported	8KoqAI
2	45121466	G	A	-0.01903	0.003068	5.60E-10	0.575058	462346	NA	rs7604640	NA	UKB-b-57	TRUE	reported	8KoqAI
2	45124291	C	G	-0.01871	0.003072	1.10E-09	0.578512	462346	NA	rs4953143	NA	UKB-b-57	TRUE	reported	8KoqAI
2	45124563	C	A	-0.01883	0.003069	8.40E-10	0.575491	462346	NA	rs4143310	NA	UKB-b-57	TRUE	reported	8KoqAI
2	45128493	A	G	-0.01829	0.003074	2.70E-09	0.579114	462346	NA	rs7584428	NA	UKB-b-57	TRUE	reported	8KoqAI
2	45130405	G	A	-0.0195	0.003095	3.00E-10	0.598872	462346	NA	rs512942	NA	UKB-b-57	TRUE	reported	8KoqAI
2	45130410	C	A	-0.01981	0.003111	1.90E-10	0.39308	462346	NA	rs528483	NA	UKB-b-57	TRUE	reported	8KoqAI
2	45131643	G	A	-0.01962	0.003087	2.10E-10	0.590785	462346	NA	rs163521	NA	UKB-b-57	TRUE	reported	8KoqAI
2	45132047	G	A	-0.01975	0.003086	1.60E-10	0.591619	462346	NA	rs163522	NA	UKB-b-57	TRUE	reported	8KoqAI
2	45132465	T	A	-0.01957	0.003088	2.30E-10	0.59343	462346	NA	rs340517	NA	UKB-b-57	TRUE	reported	8KoqAI
2	45134871	G	C	-0.02134	0.00305	2.60E-12	0.555673	462346	NA	rs340496	NA	UKB-b-57	TRUE	reported	8KoqAI
2	45134900	G	C	-0.02142	0.003049	2.10E-12	0.554666	462346	NA	rs340497	NA	UKB-b-57	TRUE	reported	8KoqAI
2	45135885	A	C	-0.02121	0.003051	3.60E-12	0.556471	462346	NA	rs340498	NA	UKB-b-57	TRUE	reported	8KoqAI
2	45136693	G	C	-0.02126	0.003048	3.10E-12	0.555826	462346	NA	rs564906	NA	UKB-b-57	TRUE	reported	8KoqAI
2	45136742	T	C	-0.02131	0.003049	2.70E-12	0.557673	462346	NA	rs564207	NA	UKB-b-57	TRUE	reported	8KoqAI
2	45137115	A	G	-0.01908	0.003033	3.20E-10	0.509779	462346	NA	rs544911	NA	UKB-b-57	TRUE	reported	8KoqAI
2	45138179	G	A	-0.01817	0.003149	8.00E-09	0.36707	462346	NA	rs1629802	NA	UKB-b-57	TRUE	reported	8KoqAI

2	45138325	A	G	-0.0193	0.003032	1.90E-10	0.507839	462346	NA	rs539447	NA	UKB-b-57	TRUE	reported	8KoqAI
2	45138444	A	C	-0.01847	0.003103	2.60E-09	0.395785	462346	NA	rs540395	NA	UKB-b-57	TRUE	reported	8KoqAI
2	45138507	A	G	-0.02149	0.003045	1.70E-12	0.555078	462346	NA	rs540606	NA	UKB-b-57	TRUE	reported	8KoqAI
2	45138941	C	T	-0.01769	0.003196	3.10E-08	0.344725	462346	NA	rs501964	NA	UKB-b-57	TRUE	reported	8KoqAI
2	45139093	A	G	-0.02136	0.003044	2.30E-12	0.555612	462346	NA	rs567047	NA	UKB-b-57	TRUE	reported	8KoqAI
2	45139153	A	G	-0.01883	0.003098	1.20E-09	0.398528	462346	NA	rs567897	NA	UKB-b-57	TRUE	reported	8KoqAI
2	45139779	C	T	-0.02174	0.003043	9.10E-13	0.557689	462346	NA	rs473098	NA	UKB-b-57	TRUE	reported	8KoqAI
2	45139904	C	T	-0.02164	0.003043	1.20E-12	0.558787	462346	NA	rs472140	NA	UKB-b-57	TRUE	reported	8KoqAI
2	45141180	T	C	-0.01909	0.003103	7.60E-10	0.394826	462346	NA	rs494904	NA	UKB-b-57	TRUE	reported	8KoqAI
2	45141218	G	A	-0.02161	0.003049	1.40E-12	0.560366	462346	NA	rs518853	NA	UKB-b-57	TRUE	reported	8KoqAI
2	45142673	C	T	-0.02146	0.00305	2.00E-12	0.559145	462346	NA	rs570436	NA	UKB-b-57	TRUE	reported	8KoqAI
2	45143175	A	T	-0.02117	0.003052	4.00E-12	0.561024	462346	NA	rs578584	NA	UKB-b-57	TRUE	reported	8KoqAI
2	45143382	G	C	-0.02124	0.003052	3.40E-12	0.561501	462346	NA	rs542883	NA	UKB-b-57	TRUE	reported	8KoqAI
2	45151600	C	T	-0.01981	0.00324	9.70E-10	0.326902	462346	NA	rs7279410	NA	UKB-b-57	TRUE	reported	8KoqAI
2	45151616	C	T	-0.02121	0.003047	3.40E-12	0.551073	462346	NA	rs490340	NA	UKB-b-57	TRUE	reported	8KoqAI
2	45152522	A	T	-0.02028	0.003311	9.00E-10	0.301581	462346	NA	rs4953148	NA	UKB-b-57	TRUE	reported	8KoqAI
2	45153287	G	C	-0.01988	0.003229	7.40E-10	0.329296	462346	NA	rs538543	NA	UKB-b-57	TRUE	reported	8KoqAI
2	45153508	C	T	-0.01974	0.003229	9.70E-10	0.32916	462346	NA	rs549814	NA	UKB-b-57	TRUE	reported	8KoqAI
2	45154229	A	G	-0.01985	0.003287	1.50E-09	0.308156	462346	NA	rs7568987	NA	UKB-b-57	TRUE	reported	8KoqAI
2	45154418	A	C	-0.02011	0.003278	8.50E-10	0.311284	462346	NA	rs7569203	NA	UKB-b-57	TRUE	reported	8KoqAI
2	45154662	C	T	-0.02003	0.003217	4.80E-10	0.333399	462346	NA	rs504764	NA	UKB-b-57	TRUE	reported	8KoqAI
2	45154689	C	T	-0.02025	0.003172	1.70E-10	0.352612	462346	NA	rs504675	NA	UKB-b-57	TRUE	reported	8KoqAI
2	45154908	G	A	-0.02114	0.003041	3.60E-12	0.554845	462346	NA	rs528301	NA	UKB-b-57	TRUE	reported	8KoqAI
2	45155276	C	T	-0.02035	0.003284	5.80E-10	0.309849	462346	NA	rs1338303	NA	UKB-b-57	TRUE	reported	8KoqAI
2	45156324	G	A	-0.0198	0.003276	1.50E-09	0.314806	462346	NA	rs1338686	NA	UKB-b-57	TRUE	reported	8KoqAI
2	45157307	T	C	-0.02062	0.003201	1.20E-10	0.344779	462346	NA	rs4953149	NA	UKB-b-57	TRUE	reported	8KoqAI
2	45157336	C	T	-0.02047	0.003201	1.60E-10	0.344632	462346	NA	rs4953150	NA	UKB-b-57	TRUE	reported	8KoqAI
2	45159091	G	A	-0.02223	0.003045	2.90E-13	0.532943	462346	NA	rs1004787	NA	UKB-b-57	TRUE	reported	8KoqAI
2	45165045	A	C	-0.01848	0.003051	1.40E-09	0.563594	462346	NA	rs895632	NA	UKB-b-57	TRUE	reported	8KoqAI
2	45165389	C	T	-0.01736	0.003043	1.20E-08	0.549364	462346	NA	rs1561227	NA	UKB-b-57	TRUE	reported	8KoqAI
2	45167570	G	T	-0.01912	0.003071	4.80E-10	0.58176	462346	NA	rs83995	NA	UKB-b-57	TRUE	reported	8KoqAI
2	45167886	T	A	-0.0172	0.003048	1.70E-08	0.549668	462346	NA	rs741813	NA	UKB-b-57	TRUE	reported	8KoqAI
2	45170153	G	A	-0.02002	0.003269	9.10E-10	0.310371	462346	NA	rs4953152	NA	UKB-b-57	TRUE	reported	8KoqAI
2	45175289	C	G	-0.01785	0.003103	8.90E-09	0.584422	462346	NA	rs338070	NA	UKB-b-57	TRUE	reported	8KoqAI
2	45175521	A	G	-0.01793	0.003086	6.30E-09	0.565104	462346	NA	rs534567	NA	UKB-b-57	TRUE	reported	8KoqAI
2	45175585	G	A	-0.01774	0.003101	1.10E-08	0.581659	462346	NA	rs173076	NA	UKB-b-57	TRUE	reported	8KoqAI
2	57942987	T	C	-0.02002	0.003152	2.10E-10	0.633094	462346	NA	rs1049607	NA	UKB-b-57	TRUE	reported	8KoqAI
2	57943567	T	C	-0.01785	0.003053	4.90E-09	0.55257	462346	NA	rs2125924	NA	UKB-b-57	TRUE	reported	8KoqAI
2	57945522	A	G	-0.01996	0.003153	2.40E-10	0.633716	462346	NA	rs6720888	NA	UKB-b-57	TRUE	reported	8KoqAI
2	57947440	T	C	-0.02018	0.00314	1.30E-10	0.628122	462346	NA	rs1340233	NA	UKB-b-57	TRUE	reported	8KoqAI
2	57948635	A	G	-0.02012	0.00314	1.50E-10	0.628227	462346	NA	rs1341801	NA	UKB-b-57	TRUE	reported	8KoqAI
2	57950346	G	A	-0.01755	0.003053	8.90E-09	0.552581	462346	NA	rs4671319	NA	UKB-b-57	TRUE	reported	8KoqAI
2	57951208	T	G	-0.01996	0.003153	2.40E-10	0.63386	462346	NA	rs1019637	NA	UKB-b-57	TRUE	reported	8KoqAI
2	57952870	A	C	-0.02	0.00314	1.90E-10	0.628165	462346	NA	rs2290867	NA	UKB-b-57	TRUE	reported	8KoqAI
2	57956088	G	A	-0.01724	0.003122	3.30E-08	0.384314	462346	NA	rs1302512	NA	UKB-b-57	TRUE	reported	8KoqAI
2	57957572	G	A	-0.01983	0.003138	2.70E-10	0.627442	462346	NA	rs1018679	NA	UKB-b-57	TRUE	reported	8KoqAI
2	57967563	A	G	-0.01957	0.003144	4.80E-10	0.63148	462346	NA	rs6709720	NA	UKB-b-57	TRUE	reported	8KoqAI
2	57971343	A	G	-0.01962	0.003141	4.30E-10	0.631641	462346	NA	rs2125925	NA	UKB-b-57	TRUE	reported	8KoqAI
2	57972834	C	A	-0.01913	0.00314	1.10E-09	0.63051	462346	NA	rs1300204	NA	UKB-b-57	TRUE	reported	8KoqAI
2	57973344	A	C	-0.01909	0.00314	1.20E-09	0.630748	462346	NA	rs3886275	NA	UKB-b-57	TRUE	reported	8KoqAI
2	57973452	C	T	-0.01907	0.00314	1.20E-09	0.630735	462346	NA	rs717817	NA	UKB-b-57	TRUE	reported	8KoqAI
2	57974429	A	T	-0.01913	0.00314	1.10E-09	0.630949	462346	NA	rs7567497	NA	UKB-b-57	TRUE	reported	8KoqAI
2	57975170	T	C	-0.01915	0.00314	1.10E-09	0.6311	462346	NA	rs1112572	NA	UKB-b-57	TRUE	reported	8KoqAI
2	57975524	T	G	-0.01939	0.00314	6.70E-10	0.631388	462346	NA	rs1167936	NA	UKB-b-57	TRUE	reported	8KoqAI
2	57980955	A	T	-0.01941	0.00314	6.30E-10	0.63127	462346	NA	rs1020461	NA	UKB-b-57	TRUE	reported	8KoqAI
2	57987201	C	G	-0.01955	0.003136	4.60E-10	0.62924	462346	NA	rs1299722	NA	UKB-b-57	TRUE	reported	8KoqAI
2	57992112	G	A	-0.01744	0.003046	1.00E-08	0.553562	462346	NA	rs1303242	NA	UKB-b-57	TRUE	reported	8KoqAI
2	57993568	G	A	-0.01941	0.003138	6.20E-10	0.631156	462346	NA	rs1299079	NA	UKB-b-57	TRUE	reported	8KoqAI
2	57993643	G	A	-0.01915	0.003144	1.10E-09	0.6332	462346	NA	rs1049607	NA	UKB-b-57	TRUE	reported	8KoqAI
2	57995586	C	T	-0.01975	0.003135	3.00E-10	0.629232	462346	NA	rs7561842	NA	UKB-b-57	TRUE	reported	8KoqAI
2	57995852	A	G	-0.01957	0.003139	4.50E-10	0.631637	462346	NA	rs7588492	NA	UKB-b-57	TRUE	reported	8KoqAI
2	57997136	G	C	-0.0197	0.003133	3.20E-10	0.629217	462346	NA	rs1049607	NA	UKB-b-57	TRUE	reported	8KoqAI
2	58002818	G	T	-0.01975	0.003131	2.80E-10	0.628719	462346	NA	rs1086530	NA	UKB-b-57	TRUE	reported	8KoqAI
2	58005792	G	A	-0.01968	0.00313	3.20E-10	0.628293	462346	NA	rs6730037	NA	UKB-b-57	TRUE	reported	8KoqAI
2	58006624	G	A	-0.01965	0.00313	3.40E-10	0.628192	462346	NA	rs1189885	NA	UKB-b-57	TRUE	reported	8KoqAI
2	58007475	A	T	-0.01966	0.00313	3.40E-10	0.628271	462346	NA	rs1271335	NA	UKB-b-57	TRUE	reported	8KoqAI
2	58007759	G	A	-0.0197	0.00313	3.10E-10	0.628495	462346	NA	rs6737913	NA	UKB-b-57	TRUE	reported	8KoqAI
2	58008155	T	C	-0.01943	0.003135	5.70E-10	0.630393	462346	NA	rs6756221	NA	UKB-b-57	TRUE	reported	8KoqAI
2	58008872	A	C	-0.01968	0.00313	3.20E-10	0.628438	462346	NA	rs4296411	NA	UKB-b-57	TRUE	reported	8KoqAI
2	58010040	T	A	-0.01974	0.00313	2.80E-10	0.628363	462346	NA	rs6761347	NA	UKB-b-57	TRUE	reported	8KoqAI
2	58011697	A	G	-0.01981	0.00313	2.50E-10	0.628653	462346	NA	rs1568450	NA	UKB-b-57	TRUE	reported	8KoqAI
2	58014723	C	T	-0.01965	0.003129	3.40E-10	0.62828	462346	NA	rs1302661	NA	UKB-b-57	TRUE	reported	8KoqAI
2	58014831	C	T	-0.01961	0.003127	3.60E-10	0.627877	462346	NA	rs1302683	NA	UKB-b-57	TRUE	reported	8KoqAI
2	58016412	G	A	-0.01985	0.00313	2.30E-10	0.628451	462346	NA	rs1016648	NA	UKB-b-57	TRUE	reported	8KoqAI
2	58016879	C	T	-0.01948	0.003129	4.80E-10	0.627632	462346	NA	rs1299132	NA	UKB-b-57	TRUE	reported	8KoqAI
2	58017224	C	T	-0.01967	0.003129	3.30E-10	0.62812	462346	NA	rs1167605	NA	UKB-b-57	TRUE	reported	8KoqAI
2	58019903	T	C	-0.01964	0.00313	3.50E-10	0.62855	462346	NA	rs1589407	NA	UKB-b-57	TRUE	reported	8KoqAI
2	58021528	G	A	-0.01945	0.003129	5.10E-10	0.627611	462346	NA	rs2090793	NA	UKB-b-57	TRUE	reported	8KoqAI
2	58021882	C	A	-0.01976	0.003129	2.70E-10	0.62802	462346	NA	rs4672223	NA	UKB-b-57	TRUE	reported	8KoqAI
2	58023009	A	T	-0.01948	0.003129	4.70E-10	0.627702	462346	NA	rs6545663	NA	UKB-b-57	TRUE	reported	8KoqAI
2	58023134	C	T	-0.01943	0.003129	5.30E-10	0.627513	462346	NA	rs6545664	NA	UKB-b-57	TRUE	reported	8KoqAI
2	58023218	G	A	-0.01956	0.003129	4.10E-10	0.627999	462346	NA	rs6545665	NA	UKB-b-57	TRUE	reported	8KoqAI
2	58025937	C	T	-0.0195	0.00313	4.70E-10	0.628015	462346	NA	rs2717048	NA	UKB-b-57	TRUE	reported	8KoqAI
2	58026312	G	A	-0.01947	0.00313	5.00E-10	0.628071	462346	NA	rs2683614	NA	UKB-b-57	TRUE	reported	8KoqAI
2	58027337	C	T	-0.01971	0.00313	3.00E-10	0.628163	462346	NA	rs954593	NA	UKB-b-57	TRUE	reported	8KoqAI
2	58027493	C	A	-0.01959	0.00313	3.90E-10	0.628099	462346	NA	rs2683615	NA	UKB-b-57	TRUE	reported	8KoqAI
2	58030962	G	A	-0.01963	0.00313	3.60E-10	0.628282	462346	NA	rs2717071	NA	UKB-b-57	TRUE	reported	8KoqAI
2	58032029	C	G	-0.01963	0.003131	3.60E-10	0.628509	462346	NA	rs1474215	NA	UKB-b-57	TRUE	reported	8KoqAI
2	58032530	G	C	-0.01964	0.003131	3.50E-10	0.628338	462346	NA	rs2717075	NA	UKB-b-57	TRUE	reported	8KoqAI
2	58032553	A	T	-0.01964	0.003131	3.50E-10	0.627946	462346	NA	rs2683620	NA	UKB-b-57	TRUE	reported	8KoqAI
2	58032759	C	T	-0.01974	0.003131	2.90E-10	0.628345	462346	NA	rs2683619	NA	UKB-b-57	TRUE	reported	8KoqAI
2	58033361	A	G	-0.01758	0.003046	7.90E-09	0.554759	462346	NA	rs2683618	NA	UKB-b-57	TRUE	reported	8KoqAI
2	58035491	C	T	-0.01974	0.003131	2.90E-10	0.628358	462346	NA	rs2717018	NA	UKB-b-57	TRUE	reported	8KoqAI
2	58035555	G	A	-0.01968	0.003131	3.20E-10	0.628084	462346	NA	rs2683616	NA	UKB-b-57	TRUE	reported	8KoqAI
2	58036044	A	G	-0.01972	0.003132	3.00E-10	0.62873	462346	NA	rs2683628	NA	UKB-b-57	TRUE	reported	8KoqAI
2	58037369	A	G	-0.0197	0.003132	3.10E-10	0.628728	462346	NA	rs2683629	NA	UKB-b-57	TRUE	reported	8KoqAI
2	58039357	T	C	-0.01974	0.003132	2.90E-10	0.628624	462346	NA	rs2953439	NA	UKB-b-57	TRUE	reported	8KoqAI
2	58040729	C	T	-0.01966	0.003133	3.50E-10	0.628531	462346	NA	rs7598169	NA	UKB-b-57	TRUE	reported	8KoqAI
2	58041482	C	T	-0.01962	0.003133	3.80E-10	0.628416	462346	NA	rs2953441	NA	UKB-b-57	TRUE	reported	8KoqAI
2	58042241	G	A	-0.01959	0.003132	4.00E-10	0.628243	462346	NA	rs1402398	NA	UKB-b-57	TRUE	reported	8KoqAI
2	58044220	G	A	-0.01966	0.003132	3.40E-10	0.628247	462346	NA	rs2717055	NA	UKB-b-57	TRUE	reported	8KoqAI
2	58045395	T	C	-0.01978	0.003133	2.70E-10	0.628719	462346	NA	rs1402399	NA	UKB-b-57	TRUE	reported	8KoqAI
2	58046683	G	A	-0.01972	0.003132	3.10E-10	0.628287	462346	NA	rs2717054	NA	UKB-b-57	TRUE	reported	8KoqAI
2	58046995	A	G	-0.01975	0.003132	2.90E-10	0.628262	462346	NA	rs2683621	NA	UKB-b-57	TRUE	reported	8KoqAI
2	58047101	G	C	-0.01981	0.003132	2.60E-10	0.628314	462346	NA	rs2717053	NA	UKB-b-57	TRUE	reported	8KoqAI
2	58047263	C	A	-0.01987	0.003133	2.30E-10	0.628679	462346	NA	rs2717052	NA	UKB-b-57	TRUE	reported	8KoqAI
2	58048221	T	C	-0.01949	0.003348	5.90E-09	0.709539	462346	NA	rs1915459	NA	UKB-b-57	TRUE	reported	8KoqAI

2	58049605	C	T	-0.01954	0.003347	5.30E-09	0.709447	462346	NA	rs2717050	NA	UKB-b-57	TRUE	reported	8KoqAI
2	58049901	T	C	-0.01954	0.003348	5.30E-09	0.709559	462346	NA	rs2717049	NA	UKB-b-57	TRUE	reported	8KoqAI
2	58050936	G	T	-0.01967	0.003347	4.20E-09	0.709372	462346	NA	rs1949613	NA	UKB-b-57	TRUE	reported	8KoqAI
2	58051437	T	A	-0.01958	0.003346	4.90E-09	0.709016	462346	NA	rs2717047	NA	UKB-b-57	TRUE	reported	8KoqAI
2	58051769	C	A	-0.01946	0.003346	6.00E-09	0.708971	462346	NA	rs1218564	NA	UKB-b-57	TRUE	reported	8KoqAI
2	58052499	G	A	-0.01946	0.003347	6.00E-09	0.709174	462346	NA	rs2683625	NA	UKB-b-57	TRUE	reported	8KoqAI
2	58053121	G	A	-0.01945	0.003346	6.20E-09	0.708974	462346	NA	rs2947348	NA	UKB-b-57	TRUE	reported	8KoqAI
2	58053706	C	T	-0.01933	0.003346	7.70E-09	0.708992	462346	NA	rs2953443	NA	UKB-b-57	TRUE	reported	8KoqAI
2	58058069	C	G	-0.01968	0.003134	3.40E-10	0.62913	462346	NA	rs2683630	NA	UKB-b-57	TRUE	reported	8KoqAI
2	58061127	C	T	-0.01945	0.003133	5.30E-10	0.628011	462346	NA	rs2717076	NA	UKB-b-57	TRUE	reported	8KoqAI
2	58063748	C	A	-0.01938	0.003345	6.80E-09	0.708686	462346	NA	rs1520965	NA	UKB-b-57	TRUE	reported	8KoqAI
2	58065936	T	A	-0.017	0.003108	4.50E-08	0.389768	462346	NA	rs2717079	NA	UKB-b-57	TRUE	reported	8KoqAI
2	58069464	A	T	-0.01889	0.003343	1.60E-08	0.707764	462346	NA	rs1106302	NA	UKB-b-57	TRUE	reported	8KoqAI
2	58071224	C	T	-0.01883	0.003341	1.70E-08	0.707358	462346	NA	rs2947350	NA	UKB-b-57	TRUE	reported	8KoqAI
2	58073603	A	C	-0.01925	0.003345	8.60E-09	0.708782	462346	NA	rs1607889	NA	UKB-b-57	TRUE	reported	8KoqAI
2	58074053	G	A	-0.01896	0.00334	1.40E-08	0.707363	462346	NA	rs2204019	NA	UKB-b-57	TRUE	reported	8KoqAI
2	58075743	T	C	-0.01886	0.003347	1.80E-08	0.708654	462346	NA	rs2716999	NA	UKB-b-57	TRUE	reported	8KoqAI
2	58076229	T	C	-0.01916	0.003345	1.00E-08	0.708654	462346	NA	rs2683622	NA	UKB-b-57	TRUE	reported	8KoqAI
2	58076796	G	T	-0.0191	0.003344	1.10E-08	0.708224	462346	NA	rs2947351	NA	UKB-b-57	TRUE	reported	8KoqAI
2	58076951	T	C	-0.01917	0.003346	1.00E-08	0.708778	462346	NA	rs2953437	NA	UKB-b-57	TRUE	reported	8KoqAI
2	58077827	A	G	-0.01921	0.003346	9.50E-09	0.708891	462346	NA	rs2953438	NA	UKB-b-57	TRUE	reported	8KoqAI
2	58083420	C	T	-0.01873	0.003338	2.00E-08	0.706726	462346	NA	rs2953442	NA	UKB-b-57	TRUE	reported	8KoqAI
2	58087409	G	C	-0.01915	0.003343	1.00E-08	0.708638	462346	NA	rs2717074	NA	UKB-b-57	TRUE	reported	8KoqAI
2	58087596	A	G	-0.019	0.003344	1.30E-08	0.708945	462346	NA	rs2717073	NA	UKB-b-57	TRUE	reported	8KoqAI
2	58087739	T	C	-0.01903	0.003342	1.20E-08	0.708226	462346	NA	rs2683626	NA	UKB-b-57	TRUE	reported	8KoqAI
2	58089124	C	T	-0.01906	0.003342	1.20E-08	0.707869	462346	NA	rs2717072	NA	UKB-b-57	TRUE	reported	8KoqAI
2	58092395	T	C	-0.01907	0.003342	1.20E-08	0.708326	462346	NA	rs2717070	NA	UKB-b-57	TRUE	reported	8KoqAI
2	58093903	A	T	-0.01881	0.003343	1.80E-08	0.708796	462346	NA	rs1044589	NA	UKB-b-57	TRUE	reported	8KoqAI
2	58094873	A	C	-0.01836	0.003102	3.30E-09	0.611665	462346	NA	rs2717068	NA	UKB-b-57	TRUE	reported	8KoqAI
2	58110969	C	A	-0.02037	0.003085	4.00E-11	0.585731	462346	NA	rs2717063	NA	UKB-b-57	TRUE	reported	8KoqAI
2	58111064	A	G	-0.01716	0.003111	3.50E-08	0.383964	462346	NA	rs6720288	NA	UKB-b-57	TRUE	reported	8KoqAI
2	58111922	T	G	-0.01709	0.003112	4.00E-08	0.383555	462346	NA	rs1533725	NA	UKB-b-57	TRUE	reported	8KoqAI
2	58113995	T	C	-0.01854	0.003087	1.90E-09	0.599868	462346	NA	rs2683634	NA	UKB-b-57	TRUE	reported	8KoqAI
2	58115072	G	C	-0.01793	0.00311	8.20E-09	0.383931	462346	NA	rs2678911	NA	UKB-b-57	TRUE	reported	8KoqAI
2	58116640	T	C	-0.01787	0.003106	8.80E-09	0.385755	462346	NA	rs2683632	NA	UKB-b-57	TRUE	reported	8KoqAI
2	58117095	T	C	-0.01741	0.003106	2.10E-08	0.386398	462346	NA	rs2678913	NA	UKB-b-57	TRUE	reported	8KoqAI
2	58117926	C	G	-0.01722	0.003107	3.00E-08	0.38593	462346	NA	rs2678914	NA	UKB-b-57	TRUE	reported	8KoqAI
2	58119122	A	G	-0.01746	0.003107	1.90E-08	0.386243	462346	NA	rs2717060	NA	UKB-b-57	TRUE	reported	8KoqAI
2	58119574	C	T	-0.01853	0.003088	2.00E-09	0.600378	462346	NA	rs2678915	NA	UKB-b-57	TRUE	reported	8KoqAI
2	58119582	G	T	-0.01776	0.003108	1.10E-08	0.384933	462346	NA	rs2678916	NA	UKB-b-57	TRUE	reported	8KoqAI
2	58119657	C	G	-0.0177	0.003107	1.20E-08	0.385638	462346	NA	rs2717059	NA	UKB-b-57	TRUE	reported	8KoqAI
2	58120553	T	A	-0.01752	0.003107	1.70E-08	0.386225	462346	NA	rs2678917	NA	UKB-b-57	TRUE	reported	8KoqAI
2	58121391	C	T	-0.01774	0.003109	1.10E-08	0.384559	462346	NA	rs2678918	NA	UKB-b-57	TRUE	reported	8KoqAI
2	58123724	T	C	-0.01845	0.003088	2.30E-09	0.59974	462346	NA	rs1356544	NA	UKB-b-57	TRUE	reported	8KoqAI
2	58123798	T	C	-0.01848	0.003088	2.20E-09	0.599917	462346	NA	rs1356545	NA	UKB-b-57	TRUE	reported	8KoqAI
2	58127647	A	G	-0.01824	0.003135	6.00E-09	0.379001	462346	NA	rs2678922	NA	UKB-b-57	TRUE	reported	8KoqAI
2	58127648	A	C	-0.01823	0.003135	6.10E-09	0.378995	462346	NA	rs2678923	NA	UKB-b-57	TRUE	reported	8KoqAI
2	58127907	C	T	-0.01807	0.00311	6.20E-09	0.384112	462346	NA	rs2717057	NA	UKB-b-57	TRUE	reported	8KoqAI
2	58128804	C	T	-0.01884	0.003087	1.00E-09	0.599374	462346	NA	rs2717056	NA	UKB-b-57	TRUE	reported	8KoqAI
2	58130327	C	T	-0.01884	0.003087	1.00E-09	0.599429	462346	NA	rs2678908	NA	UKB-b-57	TRUE	reported	8KoqAI
2	58133522	A	G	-0.01816	0.003112	5.30E-09	0.383592	462346	NA	rs2678907	NA	UKB-b-57	TRUE	reported	8KoqAI
2	58134048	T	A	-0.01887	0.003086	9.70E-10	0.599371	462346	NA	rs2678906	NA	UKB-b-57	TRUE	reported	8KoqAI
2	58135023	T	C	-0.01827	0.003111	4.30E-09	0.383614	462346	NA	rs867740	NA	UKB-b-57	TRUE	reported	8KoqAI
2	58135872	A	G	-0.01889	0.003086	9.40E-10	0.599707	462346	NA	rs2678905	NA	UKB-b-57	TRUE	reported	8KoqAI
2	58136220	C	G	-0.01818	0.003112	5.20E-09	0.383487	462346	NA	rs2717000	NA	UKB-b-57	TRUE	reported	8KoqAI
2	58137618	C	T	-0.01881	0.003086	1.10E-09	0.599329	462346	NA	rs2717001	NA	UKB-b-57	TRUE	reported	8KoqAI
2	58137755	G	T	-0.01882	0.003087	1.10E-09	0.599137	462346	NA	rs2678904	NA	UKB-b-57	TRUE	reported	8KoqAI
2	58137930	A	G	-0.01883	0.003085	1.00E-09	0.599449	462346	NA	rs2678903	NA	UKB-b-57	TRUE	reported	8KoqAI
2	58138581	G	A	-0.01819	0.003112	5.00E-09	0.383435	462346	NA	rs2678902	NA	UKB-b-57	TRUE	reported	8KoqAI
2	58138651	A	G	-0.01885	0.003086	1.00E-09	0.599634	462346	NA	rs2678901	NA	UKB-b-57	TRUE	reported	8KoqAI
2	58139593	A	G	-0.01882	0.003086	1.10E-09	0.599646	462346	NA	rs1040225	NA	UKB-b-57	TRUE	reported	8KoqAI
2	58139865	G	C	-0.01888	0.003086	9.50E-10	0.599625	462346	NA	rs1040224	NA	UKB-b-57	TRUE	reported	8KoqAI
2	58139906	A	T	-0.01839	0.003112	3.40E-09	0.383172	462346	NA	rs2678895	NA	UKB-b-57	TRUE	reported	8KoqAI
2	58140977	G	A	-0.01884	0.003085	1.00E-09	0.599535	462346	NA	rs2678881	NA	UKB-b-57	TRUE	reported	8KoqAI
2	58142647	T	G	-0.01888	0.003086	9.40E-10	0.59956	462346	NA	rs2717002	NA	UKB-b-57	TRUE	reported	8KoqAI
2	58143438	A	G	-0.01899	0.003087	7.60E-10	0.599867	462346	NA	rs2717003	NA	UKB-b-57	TRUE	reported	8KoqAI
2	58143854	C	A	-0.01886	0.003086	9.80E-10	0.599373	462346	NA	rs2717004	NA	UKB-b-57	TRUE	reported	8KoqAI
2	58144542	C	A	-0.01832	0.003113	4.00E-09	0.383368	462346	NA	rs951965	NA	UKB-b-57	TRUE	reported	8KoqAI
2	58145060	A	G	-0.01837	0.003113	3.60E-09	0.383176	462346	NA	rs2717005	NA	UKB-b-57	TRUE	reported	8KoqAI
2	58148180	G	A	-0.01898	0.003086	7.70E-10	0.599859	462346	NA	rs2465804	NA	UKB-b-57	TRUE	reported	8KoqAI
2	58148349	C	T	-0.01823	0.003113	4.80E-09	0.383028	462346	NA	rs2465805	NA	UKB-b-57	TRUE	reported	8KoqAI
2	58149116	G	A	-0.01898	0.003086	7.70E-10	0.599873	462346	NA	rs2717007	NA	UKB-b-57	TRUE	reported	8KoqAI
2	58149158	T	C	-0.01832	0.003113	4.00E-09	0.383422	462346	NA	rs2717008	NA	UKB-b-57	TRUE	reported	8KoqAI
2	58150941	A	C	-0.01885	0.003086	1.00E-09	0.599646	462346	NA	rs2459982	NA	UKB-b-57	TRUE	reported	8KoqAI
2	58151817	A	G	-0.01897	0.003087	8.00E-10	0.599997	462346	NA	rs2717010	NA	UKB-b-57	TRUE	reported	8KoqAI
2	58152075	T	C	-0.01894	0.003087	8.50E-10	0.599775	462346	NA	rs2717011	NA	UKB-b-57	TRUE	reported	8KoqAI
2	58152280	C	A	-0.01852	0.003114	2.70E-09	0.383008	462346	NA	rs2717012	NA	UKB-b-57	TRUE	reported	8KoqAI
2	58152537	G	A	-0.0184	0.003113	3.40E-09	0.383282	462346	NA	rs2717013	NA	UKB-b-57	TRUE	reported	8KoqAI
2	58154249	T	C	-0.0189	0.003085	9.10E-10	0.599491	462346	NA	rs2717014	NA	UKB-b-57	TRUE	reported	8KoqAI
2	58154601	G	A	-0.01899	0.003086	7.50E-10	0.599763	462346	NA	rs2678873	NA	UKB-b-57	TRUE	reported	8KoqAI
2	58154915	T	A	-0.01813	0.00312	6.20E-09	0.378704	462346	NA	rs2678874	NA	UKB-b-57	TRUE	reported	8KoqAI
2	58155253	T	C	-0.01902	0.003086	7.20E-10	0.599848	462346	NA	rs2717015	NA	UKB-b-57	TRUE	reported	8KoqAI
2	58155358	A	T	-0.01901	0.003086	7.20E-10	0.599843	462346	NA	rs2312141	NA	UKB-b-57	TRUE	reported	8KoqAI
2	58155829	G	A	-0.01818	0.003121	5.70E-09	0.378124	462346	NA	rs2312143	NA	UKB-b-57	TRUE	reported	8KoqAI
2	58156583	A	C	-0.01905	0.003086	6.70E-10	0.59976	462346	NA	rs2139054	NA	UKB-b-57	TRUE	reported	8KoqAI
2	58156772	G	C	-0.01708	0.003129	4.80E-08	0.617565	462346	NA	rs2176546	NA	UKB-b-57	TRUE	reported	8KoqAI
2	58158152	A	G	-0.01852	0.003091	2.10E-09	0.603894	462346	NA	rs2678879	NA	UKB-b-57	TRUE	reported	8KoqAI
2	58158283	C	T	-0.01895	0.003085	8.10E-10	0.59928	462346	NA	rs2678880	NA	UKB-b-57	TRUE	reported	8KoqAI
2	58159362	T	A	-0.0185	0.003091	2.20E-09	0.603558	462346	NA	rs2678882	NA	UKB-b-57	TRUE	reported	8KoqAI
2	58159473	T	G	-0.01847	0.003091	2.30E-09	0.604026	462346	NA	rs2717019	NA	UKB-b-57	TRUE	reported	8KoqAI
2	58159670	A	G	-0.01843	0.003091	2.50E-09	0.604031	462346	NA	rs2678883	NA	UKB-b-57	TRUE	reported	8KoqAI
2	58159852	T	C	-0.01829	0.00312	4.60E-09	0.378652	462346	NA	rs2717020	NA	UKB-b-57	TRUE	reported	8KoqAI
2	58160138	T	G	-0.0184	0.003121	3.80E-09	0.377988	462346	NA	rs2678885	NA	UKB-b-57	TRUE	reported	8KoqAI
2	58160605	G	A	-0.0182	0.003121	5.50E-09	0.378119	462346	NA	rs2717021	NA	UKB-b-57	TRUE	reported	8KoqAI
2	58160639	G	C	-0.01847	0.003091	2.30E-09	0.603868	462346	NA	rs2717022	NA	UKB-b-57	TRUE	reported	8KoqAI
2	58161221	A	G	-0.01849	0.003091	2.20E-09	0.603965	462346	NA	rs2678886	NA	UKB-b-57	TRUE	reported	8KoqAI
2	58161355	C	T	-0.01848	0.003091	2.30E-09	0.604083	462346	NA	rs2717023	NA	UKB-b-57	TRUE	reported	8KoqAI
2	58161397	C	G	-0.01845	0.003091	2.40E-09	0.60411	462346	NA	rs2717024	NA	UKB-b-57	TRUE	reported	8KoqAI
2	58161752	C	A	-0.01852	0.003091	2.10E-09	0.603814	462346	NA	rs2678887	NA	UKB-b-57	TRUE	reported	8KoqAI
2	58162191	A	G	-0.01845	0.003091	2.40E-09	0.603842	462346	NA	rs2678888	NA	UKB-b-57	TRUE	reported	8KoqAI
2	58163146	G	T	-0.01826	0.003122	4.90E-09	0.377923	462346	NA	rs2717025	NA	UKB-b-57	TRUE	reported	8KoqAI
2	58163178	A	G	-0.01826	0.003122	4.90E-09	0.377918	462346	NA	rs2717026	NA	UKB-b-57	TRUE	reported	8KoqAI
2	58164223	A	G	-0.01836	0.00309	2.80E-09	0.603593	462346	NA	rs2678889	NA	UKB-b-57	TRUE	reported	8KoqAI
2	58164435	C	T	-0.01835	0.003089	2.80E-09	0.604138	462346	NA	rs2678890	NA	UKB-b-57	TRUE	reported	8KoqAI
2	58165595	G	T	-0.01834	0.003121	4.20E-09	0.377911	462346	NA	rs2717029	NA	UKB-b-57	TRUE	reported	8KoqAI
2	58165694	T	C	-0.01822	0.003121	5.30E-09	0.378146	462346	NA	rs2717030	NA	UKB-b-57	TRUE	reported	8KoqAI
2	58166181	G	A	-0.01844	0.00309	2.40E-09	0.603595	462346	NA	rs970941	NA	UKB-b-57	TRUE	reported	8KoqAI

2	58166468	T	C	-0.01841	0.00309	2.60E-09	0.60359	462346	NA	rs2717031	NA	UKB-b-57	TRUE	reported	8KoqAI
2	58166643	T	G	-0.01843	0.00309	2.50E-09	0.603617	462346	NA	rs2678891	NA	UKB-b-57	TRUE	reported	8KoqAI
2	58166760	T	C	-0.01839	0.003095	2.80E-09	0.60456	462346	NA	rs2717032	NA	UKB-b-57	TRUE	reported	8KoqAI
2	58166769	T	C	-0.01852	0.003095	2.20E-09	0.604824	462346	NA	rs2717033	NA	UKB-b-57	TRUE	reported	8KoqAI
2	58167258	T	G	-0.01797	0.003098	6.60E-09	0.600731	462346	NA	rs2717034	NA	UKB-b-57	TRUE	reported	8KoqAI
2	58167260	G	C	-0.01801	0.003099	6.20E-09	0.60052	462346	NA	rs2678894	NA	UKB-b-57	TRUE	reported	8KoqAI
2	58167440	C	T	-0.01801	0.00309	5.60E-09	0.602315	462346	NA	rs2717035	NA	UKB-b-57	TRUE	reported	8KoqAI
2	58168098	T	C	-0.0183	0.003092	3.20E-09	0.603757	462346	NA	rs2717036	NA	UKB-b-57	TRUE	reported	8KoqAI
2	58168831	A	G	-0.0179	0.003124	1.00E-08	0.376983	462346	NA	rs2717038	NA	UKB-b-57	TRUE	reported	8KoqAI
2	58168856	G	T	-0.01795	0.003091	6.30E-09	0.602263	462346	NA	rs1401100	NA	UKB-b-57	TRUE	reported	8KoqAI
2	58169166	G	T	-0.01816	0.003124	6.10E-09	0.377148	462346	NA	rs2717039	NA	UKB-b-57	TRUE	reported	8KoqAI
2	58169388	A	G	-0.01806	0.003091	5.20E-09	0.6026	462346	NA	rs2678896	NA	UKB-b-57	TRUE	reported	8KoqAI
2	58169418	G	A	-0.01851	0.003107	2.60E-09	0.607447	462346	NA	rs2678897	NA	UKB-b-57	TRUE	reported	8KoqAI
2	58169494	T	C	-0.01812	0.003091	4.60E-09	0.602615	462346	NA	rs2717040	NA	UKB-b-57	TRUE	reported	8KoqAI
2	58170004	T	C	-0.01854	0.003093	2.00E-09	0.604024	462346	NA	rs1568253	NA	UKB-b-57	TRUE	reported	8KoqAI
2	58170161	C	A	-0.01862	0.003093	1.70E-09	0.603275	462346	NA	rs1568254	NA	UKB-b-57	TRUE	reported	8KoqAI
2	58171220	A	C	-0.01791	0.003137	1.10E-08	0.614852	462346	NA	rs1518393	NA	UKB-b-57	TRUE	reported	8KoqAI
2	58171287	A	G	-0.0182	0.003096	4.20E-09	0.605539	462346	NA	rs1518394	NA	UKB-b-57	TRUE	reported	8KoqAI
2	58172107	C	T	-0.01843	0.003083	2.30E-09	0.594445	462346	NA	rs2717041	NA	UKB-b-57	TRUE	reported	8KoqAI
2	58177683	T	G	-0.01882	0.003063	8.00E-10	0.427958	462346	NA	rs2678900	NA	UKB-b-57	TRUE	reported	8KoqAI
2	58196110	A	G	-0.01931	0.003076	3.50E-10	0.417045	462346	NA	rs2030237	NA	UKB-b-57	TRUE	reported	8KoqAI
2	58200748	A	G	-0.01738	0.003108	2.30E-08	0.61004	462346	NA	rs1271337	NA	UKB-b-57	TRUE	reported	8KoqAI
2	58201202	G	A	-0.01906	0.003077	5.90E-10	0.416165	462346	NA	rs1017229	NA	UKB-b-57	TRUE	reported	8KoqAI
2	58203057	C	T	-0.01869	0.00308	1.30E-09	0.414561	462346	NA	rs4672226	NA	UKB-b-57	TRUE	reported	8KoqAI
2	58208074	A	G	-0.01749	0.00311	1.90E-08	0.61085	462346	NA	rs1518395	NA	UKB-b-57	TRUE	reported	8KoqAI
2	58210239	T	C	-0.01729	0.003108	2.70E-08	0.610395	462346	NA	rs6732310	NA	UKB-b-57	TRUE	reported	8KoqAI
2	58210522	C	A	-0.01921	0.003076	4.20E-10	0.41722	462346	NA	rs6717480	NA	UKB-b-57	TRUE	reported	8KoqAI
2	58229428	A	C	-0.01768	0.003055	7.10E-09	0.440226	462346	NA	rs7573056	NA	UKB-b-57	TRUE	reported	8KoqAI
2	58233464	C	T	-0.01717	0.00308	2.50E-08	0.415477	462346	NA	rs7561688	NA	UKB-b-57	TRUE	reported	8KoqAI
2	58233754	A	T	-0.01755	0.003078	1.20E-08	0.416872	462346	NA	rs6214000	NA	UKB-b-57	TRUE	reported	8KoqAI
2	58237405	T	C	-0.01736	0.003114	2.50E-08	0.388994	462346	NA	rs4672228	NA	UKB-b-57	TRUE	reported	8KoqAI
2	58240442	G	C	-0.01715	0.003081	2.60E-08	0.416241	462346	NA	rs7568266	NA	UKB-b-57	TRUE	reported	8KoqAI
2	58243332	T	C	-0.0176	0.003077	1.10E-08	0.417859	462346	NA	rs2858310	NA	UKB-b-57	TRUE	reported	8KoqAI
2	58246649	T	C	-0.01758	0.00308	1.10E-08	0.416308	462346	NA	rs1704930	NA	UKB-b-57	TRUE	reported	8KoqAI
2	58268373	C	T	-0.01726	0.003071	1.90E-08	0.425403	462346	NA	rs1301461	NA	UKB-b-57	TRUE	reported	8KoqAI
2	58373205	C	T	-0.01737	0.003156	3.70E-08	0.362416	462346	NA	rs6727746	NA	UKB-b-57	TRUE	reported	8KoqAI
2	58385353	T	C	0.017457	0.003159	3.30E-08	0.639918	462346	NA	rs848292	NA	UKB-b-57	TRUE	reported	8KoqAI
2	58408687	C	T	0.017237	0.003151	4.50E-08	0.636349	462346	NA	rs848282	NA	UKB-b-57	TRUE	reported	8KoqAI
2	58412472	T	C	0.017204	0.003156	5.00E-08	0.639444	462346	NA	rs848280	NA	UKB-b-57	TRUE	reported	8KoqAI
2	58416115	C	A	0.017632	0.003147	2.10E-08	0.635851	462346	NA	rs1100188	NA	UKB-b-57	TRUE	reported	8KoqAI
2	58419649	C	A	0.017342	0.003148	3.60E-08	0.636046	462346	NA	rs848279	NA	UKB-b-57	TRUE	reported	8KoqAI
2	58420885	A	G	0.017847	0.003192	2.30E-08	0.655319	462346	NA	rs848278	NA	UKB-b-57	TRUE	reported	8KoqAI
2	58444610	C	G	0.017575	0.003155	2.50E-08	0.63966	462346	NA	rs6709460	NA	UKB-b-57	TRUE	reported	8KoqAI
2	65556341	G	A	-0.02381	0.003968	2.00E-09	0.179947	462346	NA	rs6546147	NA	UKB-b-57	TRUE	reported	8KoqAI
2	65558588	C	T	-0.02432	0.00392	5.50E-10	0.184023	462346	NA	rs6727281	NA	UKB-b-57	TRUE	reported	8KoqAI
2	65567312	C	T	-0.0242	0.003914	6.30E-10	0.184497	462346	NA	rs7590888	NA	UKB-b-57	TRUE	reported	8KoqAI
2	65570009	G	C	-0.02425	0.003913	5.70E-10	0.184396	462346	NA	rs1016516	NA	UKB-b-57	TRUE	reported	8KoqAI
2	74226102	C	T	0.025064	0.004403	1.30E-08	0.13763	462346	NA	rs4241258	NA	UKB-b-57	TRUE	reported	8KoqAI
2	74228241	T	C	0.025349	0.004604	3.70E-08	0.123708	462346	NA	rs3525979	NA	UKB-b-57	TRUE	reported	8KoqAI
2	97598001	T	C	0.024399	0.004387	2.70E-08	0.137997	462346	NA	rs6062186	NA	UKB-b-57	TRUE	reported	8KoqAI
2	97598899	A	G	0.024354	0.004385	2.80E-08	0.138018	462346	NA	rs1339339	NA	UKB-b-57	TRUE	reported	8KoqAI
2	97599618	T	C	0.025491	0.004413	7.60E-09	0.135696	462346	NA	rs7574488	NA	UKB-b-57	TRUE	reported	8KoqAI
2	97600626	G	T	0.025375	0.00441	8.70E-09	0.135668	462346	NA	rs1320147	NA	UKB-b-57	TRUE	reported	8KoqAI
2	97600827	T	C	0.025395	0.004409	8.40E-09	0.135676	462346	NA	rs1340322	NA	UKB-b-57	TRUE	reported	8KoqAI
2	97600916	C	A	0.025422	0.004409	8.10E-09	0.135669	462346	NA	rs1338756	NA	UKB-b-57	TRUE	reported	8KoqAI
2	97601024	A	G	0.0256	0.004405	6.20E-09	0.13592	462346	NA	rs2872686	NA	UKB-b-57	TRUE	reported	8KoqAI
2	97797680	T	C	0.029612	0.004492	4.30E-11	0.134041	462346	NA	rs1339001	NA	UKB-b-57	TRUE	reported	8KoqAI
2	1.94E+08	T	C	0.030135	0.00546	3.40E-08	0.089115	462346	NA	rs1766275	NA	UKB-b-57	TRUE	reported	8KoqAI
2	2.12E+08	G	T	-0.01676	0.003048	3.80E-08	0.446306	462346	NA	rs6750325	NA	UKB-b-57	TRUE	reported	8KoqAI
2	2.15E+08	A	T	-0.0167	0.003037	3.80E-08	0.536249	462346	NA	rs4673826	NA	UKB-b-57	TRUE	reported	8KoqAI
2	2.15E+08	A	G	-0.01748	0.003077	1.40E-08	0.583175	462346	NA	rs7563065	NA	UKB-b-57	TRUE	reported	8KoqAI
2	2.15E+08	T	C	-0.01756	0.003031	6.80E-09	0.478244	462346	NA	rs7558283	NA	UKB-b-57	TRUE	reported	8KoqAI
2	2.15E+08	T	C	-0.01659	0.003036	4.60E-08	0.537081	462346	NA	rs6711051	NA	UKB-b-57	TRUE	reported	8KoqAI
2	2.15E+08	C	T	-0.01723	0.003031	1.30E-08	0.523857	462346	NA	rs7599588	NA	UKB-b-57	TRUE	reported	8KoqAI
2	2.15E+08	G	T	0.018032	0.00304	3.00E-09	0.456316	462346	NA	rs3526529	NA	UKB-b-57	TRUE	reported	8KoqAI
2	2.15E+08	G	A	0.017973	0.003037	3.20E-09	0.462101	462346	NA	rs6071327	NA	UKB-b-57	TRUE	reported	8KoqAI
2	2.15E+08	G	A	0.017954	0.003037	3.40E-09	0.463877	462346	NA	rs3528697	NA	UKB-b-57	TRUE	reported	8KoqAI
2	2.15E+08	A	G	0.018091	0.003037	2.60E-09	0.465604	462346	NA	rs6760280	NA	UKB-b-57	TRUE	reported	8KoqAI
2	2.15E+08	G	C	0.017871	0.003028	3.60E-09	0.481879	462346	NA	rs7424055	NA	UKB-b-57	TRUE	reported	8KoqAI
2	2.15E+08	C	T	0.01783	0.003027	3.90E-09	0.481796	462346	NA	rs6219932	NA	UKB-b-57	TRUE	reported	8KoqAI
2	2.15E+08	G	T	0.01803	0.003033	2.80E-09	0.46086	462346	NA	rs6219932	NA	UKB-b-57	TRUE	reported	8KoqAI
2	2.15E+08	C	T	0.017808	0.003024	3.90E-09	0.476571	462346	NA	rs7602370	NA	UKB-b-57	TRUE	reported	8KoqAI
2	2.15E+08	G	A	0.017937	0.003025	3.00E-09	0.476511	462346	NA	rs3431785	NA	UKB-b-57	TRUE	reported	8KoqAI
2	2.15E+08	T	C	0.017629	0.003025	5.60E-09	0.479367	462346	NA	rs3607984	NA	UKB-b-57	TRUE	reported	8KoqAI
2	2.15E+08	A	G	0.018033	0.003031	2.70E-09	0.462896	462346	NA	rs1302306	NA	UKB-b-57	TRUE	reported	8KoqAI
2	2.15E+08	T	C	-0.0189	0.003027	4.30E-10	0.494466	462346	NA	rs1093253	NA	UKB-b-57	TRUE	reported	8KoqAI
2	2.15E+08	T	G	0.01828	0.003033	1.70E-09	0.461287	462346	NA	rs4273218	NA	UKB-b-57	TRUE	reported	8KoqAI
2	2.15E+08	C	A	0.018274	0.003034	1.70E-09	0.459604	462346	NA	rs3505825	NA	UKB-b-57	TRUE	reported	8KoqAI
2	2.15E+08	G	A	0.018498	0.003034	1.10E-09	0.459839	462346	NA	rs3475218	NA	UKB-b-57	TRUE	reported	8KoqAI
2	2.15E+08	G	A	0.018465	0.003037	1.20E-09	0.459238	462346	NA	rs3401545	NA	UKB-b-57	TRUE	reported	8KoqAI
2	2.15E+08	A	C	0.018453	0.003037	1.20E-09	0.459329	462346	NA	rs3577537	NA	UKB-b-57	TRUE	reported	8KoqAI
2	2.15E+08	C	T	0.016943	0.003104	4.80E-08	0.396584	462346	NA	rs3534489	NA	UKB-b-57	TRUE	reported	8KoqAI
2	2.15E+08	G	A	0.01814	0.003029	2.10E-09	0.480051	462346	NA	rs1302950	NA	UKB-b-57	TRUE	reported	8KoqAI
2	2.15E+08	G	C	0.017635	0.00303	5.90E-09	0.48662	462346	NA	rs3518745	NA	UKB-b-57	TRUE	reported	8KoqAI
2	2.15E+08	C	A	0.017858	0.003028	3.70E-09	0.485873	462346	NA	rs6219988	NA	UKB-b-57	TRUE	reported	8KoqAI
2	2.15E+08	A	T	-0.01724	0.003028	1.30E-08	0.510528	462346	NA	rs4416211	NA	UKB-b-57	TRUE	reported	8KoqAI
2	2.15E+08	T	C	-0.01701	0.003028	2.00E-08	0.512255	462346	NA	rs1018723	NA	UKB-b-57	TRUE	reported	8KoqAI
2	2.15E+08	T	G	0.01768	0.003029	5.30E-09	0.476653	462346	NA	rs6219988	NA	UKB-b-57	TRUE	reported	8KoqAI
2	2.15E+08	C	T	0.01755	0.003029	6.90E-09	0.476468	462346	NA	rs3413066	NA	UKB-b-57	TRUE	reported	8KoqAI
2	2.15E+08	A	G	-0.01756	0.003028	6.70E-09	0.522881	462346	NA	rs4673851	NA	UKB-b-57	TRUE	reported	8KoqAI
2	2.15E+08	C	T	0.017701	0.003029	5.10E-09	0.475162	462346	NA	rs1301335	NA	UKB-b-57	TRUE	reported	8KoqAI
2	2.15E+08	T	G	-0.01722	0.003026	1.30E-08	0.518738	462346	NA	rs1004875	NA	UKB-b-57	TRUE	reported	8KoqAI
2	2.15E+08	G	A	-0.01674	0.003025	3.10E-08	0.519742	462346	NA	rs1261752	NA	UKB-b-57	TRUE	reported	8KoqAI
2	2.15E+08	A	C	-0.01711	0.003026	1.50E-08	0.505655	462346	NA	rs1018998	NA	UKB-b-57	TRUE	reported	8KoqAI
2	2.15E+08	A	T	-0.01704	0.003026	1.80E-08	0.505969	462346	NA	rs1019245	NA	UKB-b-57	TRUE	reported	8KoqAI
2	2.15E+08	T	C	-0.01778	0.003027	4.30E-09	0.514486	462346	NA	rs1022185	NA	UKB-b-57	TRUE	reported	8KoqAI
2	2.15E+08	T	C	-0.01768	0.003029	5.30E-09	0.506663	462346	NA	rs1169117	NA	UKB-b-57	TRUE	reported	8KoqAI
2	2.15E+08	T	C	-0.01771	0.003029	5.10E-09	0.506596	462346	NA	rs1020209	NA	UKB-b-57	TRUE	reported	8KoqAI
2	2.15E+08	T	C	-0.0178	0.003029	4.20E-09	0.506327	462346	NA	rs1020511	NA	UKB-b-57	TRUE	reported	8KoqAI
2	2.15E+08	T	G	-0.01767	0.003032	5.60E-09	0.490806	462346	NA	rs4672712	NA	UKB-b-57	TRUE	reported	8KoqAI
2	2.15E+08	C	T	-0.01979	0.003036	7.20E-11	0.470852	462346	NA	rs1018831	NA	UKB-b-57	TRUE	reported	8KoqAI
2	2.15E+08	T	G	-0.01943	0.003037	1.60E-10	0.470584	462346	NA	rs4480977	NA	UKB-b-57	TRUE	reported	8KoqAI
3	38545349	C	T	0.016683	0.003052	4.60E-08	0.433553	462346	NA	rs6599206	NA	UKB-b-57	TRUE	reported	8KoqAI
3	38552250	A	G	0.016885	0.00305	3.10E-08	0.435454	462346	NA	rs7630012	NA	UKB-b-57	TRUE	reported	8KoqAI
3	38553639	C	T	0.016903	0.00305	3.00E-08	0.43495	462346	NA	rs2154778	NA	UKB-b-57	TRUE	reported	8KoqAI
3	38557161	T	C	0.016882	0.00305	3.10E-08	0.435455	462346	NA	rs2284819	NA	UKB-b-57	TRUE	reported	8KoqAI
3	38557921	T	C	0.016838	0.00305	3.40E-08	0.435415	462346	NA	rs6805386	NA	UKB-b-57	TRUE	reported	8KoqAI

3	38558197	A	G	0.016838	0.00305	3.40E-08	0.435415	462346	NA	rs6599209	NA	UKB-b-57	TRUE	reported	8KoqAI
3	38558906	T	C	0.016839	0.00305	3.40E-08	0.435415	462346	NA	rs6599212	NA	UKB-b-57	TRUE	reported	8KoqAI
3	38560345	A	G	0.016844	0.00305	3.30E-08	0.4354	462346	NA	rs2051215	NA	UKB-b-57	TRUE	reported	8KoqAI
3	38562288	C	T	0.016877	0.00305	3.10E-08	0.435205	462346	NA	rs2051216	NA	UKB-b-57	TRUE	reported	8KoqAI
3	38562579	C	T	0.016876	0.00305	3.10E-08	0.435206	462346	NA	rs2284820	NA	UKB-b-57	TRUE	reported	8KoqAI
3	38566480	G	A	0.016792	0.00305	3.70E-08	0.43519	462346	NA	rs1065800	NA	UKB-b-57	TRUE	reported	8KoqAI
3	38567771	C	A	0.016877	0.00305	3.10E-08	0.435204	462346	NA	rs6796845	NA	UKB-b-57	TRUE	reported	8KoqAI
3	38568828	T	C	0.016769	0.00305	3.80E-08	0.435421	462346	NA	rs3762791	NA	UKB-b-57	TRUE	reported	8KoqAI
3	38569463	G	T	0.016932	0.00305	2.80E-08	0.435133	462346	NA	rs9829192	NA	UKB-b-57	TRUE	reported	8KoqAI
3	38574237	C	T	0.016701	0.003063	5.00E-08	0.434918	462346	NA	rs6802071	NA	UKB-b-57	TRUE	reported	8KoqAI
3	47103282	A	T	0.060567	0.011086	4.70E-08	0.020815	462346	NA	rs1161288	NA	UKB-b-57	TRUE	reported	8KoqAI
3	48610900	A	T	0.060271	0.010543	1.10E-08	0.02207	462346	NA	rs1410635	NA	UKB-b-57	TRUE	reported	8KoqAI
3	48731450	C	T	0.042558	0.007025	1.40E-09	0.050601	462346	NA	rs7639878	NA	UKB-b-57	TRUE	reported	8KoqAI
3	48866313	C	G	0.034757	0.005347	8.00E-11	0.088915	462346	NA	rs1445192	NA	UKB-b-57	TRUE	reported	8KoqAI
3	48879153	C	T	0.04526	0.00668	1.20E-11	0.054404	462346	NA	rs7541133	NA	UKB-b-57	TRUE	reported	8KoqAI
3	48926161	C	T	0.04713	0.006993	1.60E-11	0.050887	462346	NA	rs1405026	NA	UKB-b-57	TRUE	reported	8KoqAI
3	48938167	G	A	0.045979	0.006665	5.20E-12	0.054496	462346	NA	rs1860863	NA	UKB-b-57	TRUE	reported	8KoqAI
3	48940334	G	C	0.046014	0.006663	5.00E-12	0.054516	462346	NA	rs1384120	NA	UKB-b-57	TRUE	reported	8KoqAI
3	48947188	G	A	0.035634	0.005343	2.60E-11	0.088897	462346	NA	rs1881469	NA	UKB-b-57	TRUE	reported	8KoqAI
3	48965078	A	C	0.043133	0.006555	4.70E-11	0.056429	462346	NA	rs3422164	NA	UKB-b-57	TRUE	reported	8KoqAI
3	49021111	T	C	0.045326	0.006642	8.80E-12	0.054872	462346	NA	rs1116743	NA	UKB-b-57	TRUE	reported	8KoqAI
3	49029468	C	T	0.046427	0.006687	3.80E-12	0.054327	462346	NA	rs7608265	NA	UKB-b-57	TRUE	reported	8KoqAI
3	49120006	T	C	0.034179	0.005229	6.30E-11	0.092372	462346	NA	rs1114948	NA	UKB-b-57	TRUE	reported	8KoqAI
3	49220504	A	C	0.048284	0.006992	5.00E-12	0.050832	462346	NA	rs1379203	NA	UKB-b-57	TRUE	reported	8KoqAI
3	49250007	C	T	0.036713	0.00534	6.20E-12	0.089885	462346	NA	rs1130111	NA	UKB-b-57	TRUE	reported	8KoqAI
3	49425004	G	A	-0.0357	0.006361	2.00E-08	0.060508	462346	NA	rs3407173	NA	UKB-b-57	TRUE	reported	8KoqAI
3	49435706	C	T	-0.03641	0.006522	2.40E-08	0.058059	462346	NA	rs1438678	NA	UKB-b-57	TRUE	reported	8KoqAI
3	49495682	T	C	0.049594	0.007445	2.70E-11	0.046111	462346	NA	rs1129782	NA	UKB-b-57	TRUE	reported	8KoqAI
3	49575831	C	T	-0.03484	0.006383	4.80E-08	0.060403	462346	NA	rs3451988	NA	UKB-b-57	TRUE	reported	8KoqAI
3	49585241	C	G	0.035759	0.005397	3.50E-11	0.088537	462346	NA	rs7447323	NA	UKB-b-57	TRUE	reported	8KoqAI
3	49634594	T	C	-0.03545	0.006335	2.20E-08	0.060807	462346	NA	rs1307108	NA	UKB-b-57	TRUE	reported	8KoqAI
3	49641049	G	A	-0.03591	0.006397	2.00E-08	0.059602	462346	NA	rs1310079	NA	UKB-b-57	TRUE	reported	8KoqAI
3	49657209	G	C	0.035135	0.005257	2.30E-11	0.091058	462346	NA	rs7619491	NA	UKB-b-57	TRUE	reported	8KoqAI
3	49706699	A	G	-0.03556	0.006403	2.80E-08	0.059511	462346	NA	rs3611607	NA	UKB-b-57	TRUE	reported	8KoqAI
3	49709147	A	T	-0.03558	0.006403	2.80E-08	0.059508	462346	NA	rs7132498	NA	UKB-b-57	TRUE	reported	8KoqAI
3	49734040	G	T	0.018101	0.003248	2.50E-08	0.319062	462346	NA	rs1249622	NA	UKB-b-57	TRUE	reported	8KoqAI
3	49735746	C	G	0.017855	0.003231	3.30E-08	0.322557	462346	NA	rs1113021	NA	UKB-b-57	TRUE	reported	8KoqAI
3	49736172	C	A	0.017857	0.003231	3.30E-08	0.322355	462346	NA	rs1113021	NA	UKB-b-57	TRUE	reported	8KoqAI
3	49737954	G	A	-0.03627	0.00639	1.40E-08	0.05969	462346	NA	rs3562024	NA	UKB-b-57	TRUE	reported	8KoqAI
3	49738816	G	A	0.046049	0.006744	8.60E-12	0.053092	462346	NA	rs7958155	NA	UKB-b-57	TRUE	reported	8KoqAI
3	49738945	C	T	0.017855	0.003231	3.30E-08	0.322353	462346	NA	rs6804655	NA	UKB-b-57	TRUE	reported	8KoqAI
3	49747560	C	T	-0.03632	0.006413	1.50E-08	0.059447	462346	NA	rs1306115	NA	UKB-b-57	TRUE	reported	8KoqAI
3	49751585	C	T	0.017841	0.003231	3.30E-08	0.322598	462346	NA	rs2291542	NA	UKB-b-57	TRUE	reported	8KoqAI
3	49751900	C	A	0.017837	0.003231	3.40E-08	0.322605	462346	NA	rs1171067	NA	UKB-b-57	TRUE	reported	8KoqAI
3	49758764	A	G	0.01794	0.003231	2.80E-08	0.322543	462346	NA	rs4768	NA	UKB-b-57	TRUE	reported	8KoqAI
3	49765483	C	G	0.017863	0.003231	3.20E-08	0.322161	462346	NA	rs2307021	NA	UKB-b-57	TRUE	reported	8KoqAI
3	49770032	G	A	0.017727	0.00323	4.10E-08	0.32301	462346	NA	rs3749237	NA	UKB-b-57	TRUE	reported	8KoqAI
3	49776178	C	T	0.017983	0.003234	2.70E-08	0.321485	462346	NA	rs1171495	NA	UKB-b-57	TRUE	reported	8KoqAI
3	49779764	C	T	0.017789	0.003231	3.70E-08	0.322314	462346	NA	rs1308613	NA	UKB-b-57	TRUE	reported	8KoqAI
3	49779906	C	T	0.017992	0.003235	2.70E-08	0.321446	462346	NA	rs1308646	NA	UKB-b-57	TRUE	reported	8KoqAI
3	49808007	A	G	0.044702	0.006926	1.10E-10	0.051469	462346	NA	rs1155065	NA	UKB-b-57	TRUE	reported	8KoqAI
3	49808190	G	A	0.018123	0.003226	1.90E-08	0.324005	462346	NA	rs7431078	NA	UKB-b-57	TRUE	reported	8KoqAI
3	49808517	A	G	0.018051	0.003227	2.20E-08	0.324357	462346	NA	rs3452227	NA	UKB-b-57	TRUE	reported	8KoqAI
3	49808981	C	T	0.01822	0.003227	1.60E-08	0.323855	462346	NA	rs1171746	NA	UKB-b-57	TRUE	reported	8KoqAI
3	49812606	C	T	0.018104	0.003226	2.00E-08	0.323994	462346	NA	rs3521017	NA	UKB-b-57	TRUE	reported	8KoqAI
3	49819779	T	G	0.017868	0.003231	3.20E-08	0.322598	462346	NA	rs3547440	NA	UKB-b-57	TRUE	reported	8KoqAI
3	49821878	G	C	0.017881	0.003232	3.10E-08	0.322181	462346	NA	rs1308424	NA	UKB-b-57	TRUE	reported	8KoqAI
3	49823024	G	C	0.017893	0.003232	3.10E-08	0.322161	462346	NA	rs1192090	NA	UKB-b-57	TRUE	reported	8KoqAI
3	49829326	G	A	0.017832	0.003234	3.50E-08	0.321806	462346	NA	rs6226071	NA	UKB-b-57	TRUE	reported	8KoqAI
3	49831698	G	A	0.017769	0.003233	3.90E-08	0.321866	462346	NA	rs3473533	NA	UKB-b-57	TRUE	reported	8KoqAI
3	49834767	A	C	0.01808	0.003238	2.40E-08	0.319881	462346	NA	rs7132499	NA	UKB-b-57	TRUE	reported	8KoqAI
3	49846041	G	A	0.018153	0.003227	1.90E-08	0.323873	462346	NA	rs2234385	NA	UKB-b-57	TRUE	reported	8KoqAI
3	49847623	A	G	0.018189	0.003228	1.80E-08	0.323928	462346	NA	rs1799845	NA	UKB-b-57	TRUE	reported	8KoqAI
3	49849723	G	A	0.018101	0.003229	2.10E-08	0.323747	462346	NA	rs1799843	NA	UKB-b-57	TRUE	reported	8KoqAI
3	49852419	G	A	0.01797	0.003233	2.70E-08	0.322066	462346	NA	rs6226071	NA	UKB-b-57	TRUE	reported	8KoqAI
3	49860678	C	T	0.017638	0.00323	4.80E-08	0.323141	462346	NA	rs9850465	NA	UKB-b-57	TRUE	reported	8KoqAI
3	49871641	C	T	0.017842	0.003232	3.40E-08	0.322457	462346	NA	rs2352967	NA	UKB-b-57	TRUE	reported	8KoqAI
3	49878652	G	A	0.017985	0.003238	2.80E-08	0.320392	462346	NA	rs1317140	NA	UKB-b-57	TRUE	reported	8KoqAI
3	49884261	C	T	0.018089	0.003245	2.50E-08	0.320155	462346	NA	rs9819511	NA	UKB-b-57	TRUE	reported	8KoqAI
3	49884913	C	T	0.018162	0.003245	2.20E-08	0.320062	462346	NA	rs1331606	NA	UKB-b-57	TRUE	reported	8KoqAI
3	49889919	T	C	0.018192	0.003242	2.00E-08	0.322503	462346	NA	rs6226072	NA	UKB-b-57	TRUE	reported	8KoqAI
3	49891002	G	C	0.018089	0.003244	2.40E-08	0.322537	462346	NA	rs1086595	NA	UKB-b-57	TRUE	reported	8KoqAI
3	49891885	T	C	0.017826	0.003242	3.80E-08	0.324676	462346	NA	rs2352975	NA	UKB-b-57	TRUE	reported	8KoqAI
3	49892896	A	G	0.017962	0.003249	3.20E-08	0.324135	462346	NA	rs1004941	NA	UKB-b-57	TRUE	reported	8KoqAI
3	49894030	T	C	0.018095	0.003252	2.60E-08	0.324572	462346	NA	rs2276864	NA	UKB-b-57	TRUE	reported	8KoqAI
3	49897830	C	T	0.017463	0.00303	8.20E-09	0.510088	462346	NA	rs2681780	NA	UKB-b-57	TRUE	reported	8KoqAI
3	49898000	A	G	0.017467	0.00303	8.20E-09	0.510134	462346	NA	rs2777888	NA	UKB-b-57	TRUE	reported	8KoqAI
3	49898273	A	G	0.017479	0.00303	8.00E-09	0.510151	462346	NA	rs2681781	NA	UKB-b-57	TRUE	reported	8KoqAI
3	49902544	A	G	0.017146	0.003024	1.40E-08	0.50511	462346	NA	rs9821675	NA	UKB-b-57	TRUE	reported	8KoqAI
3	49907111	C	A	0.017769	0.003026	4.30E-09	0.511118	462346	NA	rs6446187	NA	UKB-b-57	TRUE	reported	8KoqAI
3	49910084	T	C	0.017577	0.003026	6.30E-09	0.51171	462346	NA	rs1317154	NA	UKB-b-57	TRUE	reported	8KoqAI
3	49913705	T	C	0.020662	0.00377	4.20E-08	0.204104	462346	NA	rs3602237	NA	UKB-b-57	TRUE	reported	8KoqAI
3	49920571	T	C	0.017481	0.00303	7.90E-09	0.511674	462346	NA	rs9843653	NA	UKB-b-57	TRUE	reported	8KoqAI
3	49924424	G	A	0.017858	0.003026	3.60E-09	0.514807	462346	NA	rs1171319	NA	UKB-b-57	TRUE	reported	8KoqAI
3	49924940	T	C	0.018077	0.003029	2.40E-09	0.515937	462346	NA	rs1062633	NA	UKB-b-57	TRUE	reported	8KoqAI
3	49930215	C	T	0.017976	0.003028	2.90E-09	0.515106	462346	NA	rs6795703	NA	UKB-b-57	TRUE	reported	8KoqAI
3	49930316	C	T	0.027873	0.00484	8.50E-09	0.112148	462346	NA	rs1118737	NA	UKB-b-57	TRUE	reported	8KoqAI
3	49936102	T	C	0.018069	0.003029	2.40E-09	0.515725	462346	NA	rs2230590	NA	UKB-b-57	TRUE	reported	8KoqAI
3	49936910	T	A	0.017998	0.003026	2.70E-09	0.514773	462346	NA	rs1113022	NA	UKB-b-57	TRUE	reported	8KoqAI
3	49938227	C	G	0.018106	0.003027	2.20E-09	0.5159	462346	NA	rs3774758	NA	UKB-b-57	TRUE	reported	8KoqAI
3	49939587	G	A	0.018103	0.003027	2.20E-09	0.515925	462346	NA	rs3733135	NA	UKB-b-57	TRUE	reported	8KoqAI
3	49941436	G	A	0.017365	0.003025	9.50E-09	0.506155	462346	NA	rs2280406	NA	UKB-b-57	TRUE	reported	8KoqAI
3	49949834	A	T	0.018009	0.003027	2.70E-09	0.515249	462346	NA	rs7634084	NA	UKB-b-57	TRUE	reported	8KoqAI
3	49951316	T	C	0.018035	0.003027	2.50E-09	0.516047	462346	NA	rs9848497	NA	UKB-b-57	TRUE	reported	8KoqAI
3	49955296	A	G	0.040822	0.006659	8.80E-10	0.056459	462346	NA	rs1506283	NA	UKB-b-57	TRUE	reported	8KoqAI
3	49960388	T	C	0.018003	0.003027	2.70E-09	0.515194	462346	NA	rs6226209	NA	UKB-b-57	TRUE	reported	8KoqAI
3	49968572	T	C	0.018284	0.003031	1.60E-09	0.530816	462346	NA	rs6771546	NA	UKB-b-57	TRUE	reported	8KoqAI
3	49971514	C	A	0.018285	0.003044	1.90E-09	0.545999	462346	NA	rs7613875	NA	UKB-b-57	TRUE	reported	8KoqAI
3	49972101	A	G	0.018338	0.003031	1.40E-09	0.530804	462346	NA	rs5575173	NA	UKB-b-57	TRUE	reported	8KoqAI
3	49975334	A	G	0.018336	0.003031	1.50E-09	0.530809	462346	NA	rs7621026	NA	UKB-b-57	TRUE	reported	8KoqAI
3	49975654	C	T	0.018469	0.003031	1.10E-09	0.531257	462346	NA	rs7609971	NA	UKB-b-57	TRUE	reported	8KoqAI
3	49978069	C	T	0.018444	0.003031	1.20E-09	0.531302	462346	NA	rs6772095	NA	UKB-b-57	TRUE	reported	8KoqAI
3	49986118	T	C	0.018436	0.003031	1.20E-09	0.531303	462346	NA	rs7294233	NA	UKB-b-57	TRUE	reported	8KoqAI
3	49993191	T	G	0.018332	0.003031	1.50E-09	0.530812	462346	NA	rs1113022	NA	UKB-b-57	TRUE	reported	8KoqAI
3	49993319	A	G	0.018212	0.003096	4.00E-09	0.513534	462346	NA	rs1113022	NA	UKB-b-57	TRUE	reported	8KoqAI
3	49995518	T	C	0.018268	0.003031	1.70E-09	0.530868	462346	NA	rs6792892	NA	UKB-b-57	TRUE	reported	8KoqAI
3	49997963	G	A	0.018371	0.003031	1.30E-09	0.530788	462346	NA	rs2624843	NA	UKB-b-57	TRUE	reported	8KoqAI

3	49998282	C	T	0.018464	0.003031	1.10E-09	0.531258	462346	NA	rs2883057	NA	UKB-b-57	TRUE	reported	8KoqAI
3	50002245	T	C	0.018339	0.003031	1.40E-09	0.530806	462346	NA	rs1249697	NA	UKB-b-57	TRUE	reported	8KoqAI
3	50003246	C	T	0.018339	0.003031	1.40E-09	0.530806	462346	NA	rs1113022	NA	UKB-b-57	TRUE	reported	8KoqAI
3	50004209	G	A	0.018373	0.003031	1.30E-09	0.530789	462346	NA	rs2252833	NA	UKB-b-57	TRUE	reported	8KoqAI
3	50008566	A	G	0.018439	0.003031	1.20E-09	0.531302	462346	NA	rs6446189	NA	UKB-b-57	TRUE	reported	8KoqAI
3	50009176	T	C	0.018373	0.003031	1.30E-09	0.530793	462346	NA	rs3506572	NA	UKB-b-57	TRUE	reported	8KoqAI
3	50011095	T	C	0.018338	0.003031	1.40E-09	0.530811	462346	NA	rs2681778	NA	UKB-b-57	TRUE	reported	8KoqAI
3	50011540	T	C	0.018333	0.003031	1.50E-09	0.530812	462346	NA	rs1113022	NA	UKB-b-57	TRUE	reported	8KoqAI
3	50017478	A	G	0.018459	0.003031	1.10E-09	0.531266	462346	NA	rs6780731	NA	UKB-b-57	TRUE	reported	8KoqAI
3	50018585	T	G	0.018327	0.003031	1.50E-09	0.530823	462346	NA	rs1113023	NA	UKB-b-57	TRUE	reported	8KoqAI
3	50022292	G	A	0.018465	0.003031	1.10E-09	0.531261	462346	NA	rs4688690	NA	UKB-b-57	TRUE	reported	8KoqAI
3	50022926	T	C	0.018438	0.003031	1.20E-09	0.531307	462346	NA	rs9853222	NA	UKB-b-57	TRUE	reported	8KoqAI
3	50025028	A	G	0.018328	0.003031	1.50E-09	0.53089	462346	NA	rs1306012	NA	UKB-b-57	TRUE	reported	8KoqAI
3	50025530	A	G	0.018424	0.003032	1.20E-09	0.531387	462346	NA	rs1248560	NA	UKB-b-57	TRUE	reported	8KoqAI
3	50026029	A	C	0.018432	0.003032	1.20E-09	0.531382	462346	NA	rs3408057	NA	UKB-b-57	TRUE	reported	8KoqAI
3	50027774	C	T	0.018325	0.003031	1.50E-09	0.530904	462346	NA	rs2353579	NA	UKB-b-57	TRUE	reported	8KoqAI
3	50028385	G	A	0.018452	0.003032	1.20E-09	0.531346	462346	NA	rs4688758	NA	UKB-b-57	TRUE	reported	8KoqAI
3	50035323	T	C	0.018446	0.003031	1.20E-09	0.531287	462346	NA	rs4688757	NA	UKB-b-57	TRUE	reported	8KoqAI
3	50035542	C	T	0.01847	0.003031	1.10E-09	0.531239	462346	NA	rs4688689	NA	UKB-b-57	TRUE	reported	8KoqAI
3	50037123	C	G	0.018471	0.003031	1.10E-09	0.531249	462346	NA	rs3774733	NA	UKB-b-57	TRUE	reported	8KoqAI
3	50037940	A	G	0.018471	0.003031	1.10E-09	0.531251	462346	NA	rs6767381	NA	UKB-b-57	TRUE	reported	8KoqAI
3	50038664	G	A	0.018468	0.003031	1.10E-09	0.530723	462346	NA	rs1192519	NA	UKB-b-57	TRUE	reported	8KoqAI
3	50039474	G	A	0.018446	0.003031	1.20E-09	0.531295	462346	NA	rs7628058	NA	UKB-b-57	TRUE	reported	8KoqAI
3	50040194	T	A	0.018459	0.003031	1.10E-09	0.530672	462346	NA	rs4688756	NA	UKB-b-57	TRUE	reported	8KoqAI
3	50041313	C	T	-0.01833	0.003033	1.50E-09	0.472988	462346	NA	rs6765484	NA	UKB-b-57	TRUE	reported	8KoqAI
3	50046046	G	T	-0.01842	0.003032	1.20E-09	0.468709	462346	NA	rs2353586	NA	UKB-b-57	TRUE	reported	8KoqAI
3	50046064	G	A	-0.01845	0.003032	1.20E-09	0.46876	462346	NA	rs2353587	NA	UKB-b-57	TRUE	reported	8KoqAI
3	50047404	C	T	-0.01846	0.003031	1.10E-09	0.468719	462346	NA	rs9881008	NA	UKB-b-57	TRUE	reported	8KoqAI
3	50049299	T	G	-0.01844	0.003031	1.20E-09	0.468699	462346	NA	rs7634917	NA	UKB-b-57	TRUE	reported	8KoqAI
3	50052729	C	G	-0.01832	0.003031	1.50E-09	0.469182	462346	NA	rs6446190	NA	UKB-b-57	TRUE	reported	8KoqAI
3	50059339	T	C	-0.01838	0.003031	1.30E-09	0.469227	462346	NA	rs9861216	NA	UKB-b-57	TRUE	reported	8KoqAI
3	50059758	T	C	-0.01838	0.003031	1.30E-09	0.469227	462346	NA	rs6446193	NA	UKB-b-57	TRUE	reported	8KoqAI
3	50059866	T	C	-0.01838	0.003031	1.30E-09	0.469235	462346	NA	rs7650214	NA	UKB-b-57	TRUE	reported	8KoqAI
3	50060332	G	C	-0.01833	0.003031	1.50E-09	0.469188	462346	NA	rs1171356	NA	UKB-b-57	TRUE	reported	8KoqAI
3	50064424	G	A	-0.01843	0.003031	1.20E-09	0.468719	462346	NA	rs4688755	NA	UKB-b-57	TRUE	reported	8KoqAI
3	50064867	T	C	-0.01845	0.003031	1.20E-09	0.468702	462346	NA	rs2526751	NA	UKB-b-57	TRUE	reported	8KoqAI
3	50069452	G	T	-0.01843	0.003031	1.20E-09	0.468668	462346	NA	rs9866695	NA	UKB-b-57	TRUE	reported	8KoqAI
3	50070763	A	C	-0.01842	0.003031	1.20E-09	0.468755	462346	NA	rs7621352	NA	UKB-b-57	TRUE	reported	8KoqAI
3	50073046	C	T	-0.01829	0.003031	1.60E-09	0.469251	462346	NA	rs6685163	NA	UKB-b-57	TRUE	reported	8KoqAI
3	50075494	A	G	0.047067	0.007414	2.20E-10	0.047424	462346	NA	rs1126343	NA	UKB-b-57	TRUE	reported	8KoqAI
3	50078541	C	T	-0.01831	0.003031	1.50E-09	0.469256	462346	NA	rs9814664	NA	UKB-b-57	TRUE	reported	8KoqAI
3	50080439	A	G	-0.01835	0.003031	1.40E-09	0.469283	462346	NA	rs2526748	NA	UKB-b-57	TRUE	reported	8KoqAI
3	50088266	T	C	-0.01816	0.003027	2.00E-09	0.477777	462346	NA	rs2624824	NA	UKB-b-57	TRUE	reported	8KoqAI
3	50089690	G	T	-0.01823	0.003027	1.70E-09	0.47724	462346	NA	rs2624823	NA	UKB-b-57	TRUE	reported	8KoqAI
3	50092226	G	A	-0.01842	0.003031	1.20E-09	0.468797	462346	NA	rs9311446	NA	UKB-b-57	TRUE	reported	8KoqAI
3	50097319	C	A	-0.01842	0.003031	1.20E-09	0.468806	462346	NA	rs2245365	NA	UKB-b-57	TRUE	reported	8KoqAI
3	50107512	G	A	-0.01829	0.003031	1.60E-09	0.469247	462346	NA	rs2240329	NA	UKB-b-57	TRUE	reported	8KoqAI
3	50113034	A	G	-0.01826	0.003027	1.60E-09	0.477319	462346	NA	rs2240327	NA	UKB-b-57	TRUE	reported	8KoqAI
3	50115245	G	A	-0.01816	0.003027	2.00E-09	0.477748	462346	NA	rs2526743	NA	UKB-b-57	TRUE	reported	8KoqAI
3	50117312	T	G	-0.0182	0.003027	1.80E-09	0.477739	462346	NA	rs2624816	NA	UKB-b-57	TRUE	reported	8KoqAI
3	50118748	T	C	-0.01819	0.003027	1.80E-09	0.477796	462346	NA	rs3483171	NA	UKB-b-57	TRUE	reported	8KoqAI
3	50123417	A	G	-0.01826	0.003027	1.60E-09	0.477262	462346	NA	rs1004908	NA	UKB-b-57	TRUE	reported	8KoqAI
3	50125996	G	A	-0.01814	0.003027	2.00E-09	0.477753	462346	NA	rs2526754	NA	UKB-b-57	TRUE	reported	8KoqAI
3	50128386	G	A	-0.01826	0.003027	1.60E-09	0.477246	462346	NA	rs2240326	NA	UKB-b-57	TRUE	reported	8KoqAI
3	50128893	A	G	-0.01817	0.003027	1.90E-09	0.477775	462346	NA	rs2624853	NA	UKB-b-57	TRUE	reported	8KoqAI
3	50129399	C	T	-0.01833	0.003027	1.40E-09	0.47771	462346	NA	rs2013208	NA	UKB-b-57	TRUE	reported	8KoqAI
3	50139142	A	G	-0.0184	0.003031	1.30E-09	0.469318	462346	NA	rs2856234	NA	UKB-b-57	TRUE	reported	8KoqAI
3	50146094	T	A	-0.01849	0.003031	1.10E-09	0.468766	462346	NA	rs3733133	NA	UKB-b-57	TRUE	reported	8KoqAI
3	50148305	C	G	-0.01811	0.003027	2.20E-09	0.477767	462346	NA	rs2301166	NA	UKB-b-57	TRUE	reported	8KoqAI
3	50154989	G	A	-0.01833	0.003031	1.50E-09	0.469372	462346	NA	rs2624825	NA	UKB-b-57	TRUE	reported	8KoqAI
3	50159844	T	C	-0.01819	0.003027	1.90E-09	0.478017	462346	NA	rs2071207	NA	UKB-b-57	TRUE	reported	8KoqAI
3	50160109	G	A	-0.01843	0.003031	1.20E-09	0.469558	462346	NA	rs2071206	NA	UKB-b-57	TRUE	reported	8KoqAI
3	50162291	T	G	-0.01839	0.003028	1.30E-09	0.47741	462346	NA	rs2008877	NA	UKB-b-57	TRUE	reported	8KoqAI
3	50169715	C	T	-0.01892	0.003032	4.30E-10	0.47361	462346	NA	rs4688686	NA	UKB-b-57	TRUE	reported	8KoqAI
3	50174184	C	T	-0.01981	0.00303	6.30E-11	0.488308	462346	NA	rs7428430	NA	UKB-b-57	TRUE	reported	8KoqAI
3	50174197	G	T	-0.01966	0.003458	1.30E-08	0.74183	462346	NA	rs2624847	NA	UKB-b-57	TRUE	reported	8KoqAI
3	50174848	G	A	-0.01906	0.003031	3.20E-10	0.475462	462346	NA	rs7642934	NA	UKB-b-57	TRUE	reported	8KoqAI
3	50185967	G	A	0.046834	0.008324	1.80E-08	0.03641	462346	NA	rs1122696	NA	UKB-b-57	TRUE	reported	8KoqAI
3	50204745	C	T	-0.01687	0.003041	2.90E-08	0.462599	462346	NA	rs3774745	NA	UKB-b-57	TRUE	reported	8KoqAI
3	50207227	C	T	-0.01716	0.003033	1.50E-08	0.464388	462346	NA	rs3774749	NA	UKB-b-57	TRUE	reported	8KoqAI
3	50208406	C	G	-0.01671	0.003034	3.60E-08	0.464917	462346	NA	rs3774750	NA	UKB-b-57	TRUE	reported	8KoqAI
3	68387830	G	A	-0.01665	0.003035	4.10E-08	0.471416	462346	NA	rs1491747	NA	UKB-b-57	TRUE	reported	8KoqAI
3	68406773	T	C	-0.01688	0.003034	2.60E-08	0.473559	462346	NA	rs262245	NA	UKB-b-57	TRUE	reported	8KoqAI
3	68406779	G	C	-0.01689	0.003034	2.60E-08	0.473487	462346	NA	rs262244	NA	UKB-b-57	TRUE	reported	8KoqAI
3	68407029	A	G	-0.01686	0.00303	2.60E-08	0.473335	462346	NA	rs262243	NA	UKB-b-57	TRUE	reported	8KoqAI
3	68407054	G	A	-0.01694	0.00303	2.20E-08	0.473271	462346	NA	rs262242	NA	UKB-b-57	TRUE	reported	8KoqAI
3	68407323	A	G	-0.01692	0.00303	2.40E-08	0.473287	462346	NA	rs262241	NA	UKB-b-57	TRUE	reported	8KoqAI
3	68407880	T	C	-0.01695	0.00303	2.20E-08	0.47329	462346	NA	rs63108	NA	UKB-b-57	TRUE	reported	8KoqAI
3	68408109	C	T	-0.01721	0.003035	1.40E-08	0.468553	462346	NA	rs262240	NA	UKB-b-57	TRUE	reported	8KoqAI
3	68408117	G	A	-0.01713	0.003034	1.60E-08	0.468696	462346	NA	rs262239	NA	UKB-b-57	TRUE	reported	8KoqAI
3	68408322	T	A	-0.01691	0.003031	2.40E-08	0.47429	462346	NA	rs262238	NA	UKB-b-57	TRUE	reported	8KoqAI
3	68408550	T	C	-0.01677	0.003031	3.20E-08	0.474598	462346	NA	rs262237	NA	UKB-b-57	TRUE	reported	8KoqAI
3	68408564	A	G	-0.01677	0.003031	3.20E-08	0.474596	462346	NA	rs262236	NA	UKB-b-57	TRUE	reported	8KoqAI
3	68409374	C	T	-0.01683	0.003032	2.90E-08	0.474262	462346	NA	rs262234	NA	UKB-b-57	TRUE	reported	8KoqAI
3	68413184	G	T	-0.01664	0.003032	4.00E-08	0.473909	462346	NA	rs262227	NA	UKB-b-57	TRUE	reported	8KoqAI
3	68413287	G	A	-0.01668	0.003032	3.80E-08	0.473917	462346	NA	rs262226	NA	UKB-b-57	TRUE	reported	8KoqAI
3	71498561	T	C	-0.02214	0.003065	5.00E-13	0.428486	462346	NA	rs5571689	NA	UKB-b-57	TRUE	reported	8KoqAI
3	71503479	T	G	-0.02241	0.003065	2.60E-13	0.433509	462346	NA	rs1170789	NA	UKB-b-57	TRUE	reported	8KoqAI
3	71516857	T	G	-0.01857	0.003036	9.50E-10	0.478631	462346	NA	rs1306042	NA	UKB-b-57	TRUE	reported	8KoqAI
3	71517527	G	T	-0.01853	0.003036	1.00E-09	0.478488	462346	NA	rs1170892	NA	UKB-b-57	TRUE	reported	8KoqAI
3	71517643	T	C	-0.01855	0.003036	9.90E-10	0.478613	462346	NA	rs1170627	NA	UKB-b-57	TRUE	reported	8KoqAI
3	71520890	A	C	-0.02267	0.00306	1.30E-13	0.431614	462346	NA	rs5720504	NA	UKB-b-57	TRUE	reported	8KoqAI
3	71522178	A	G	-0.01912	0.003036	3.00E-10	0.477457	462346	NA	rs3554015	NA	UKB-b-57	TRUE	reported	8KoqAI
3	71527191	T	C	-0.01934	0.003034	1.80E-10	0.481062	462346	NA	rs3426444	NA	UKB-b-57	TRUE	reported	8KoqAI
3	71527785	T	C	-0.01933	0.003034	1.90E-10	0.481066	462346	NA	rs9836630	NA	UKB-b-57	TRUE	reported	8KoqAI
3	71531170	A	G	-0.01942	0.003034	1.50E-10	0.481027	462346	NA	rs9853081	NA	UKB-b-57	TRUE	reported	8KoqAI
3	71531467	T	C	-0.0194	0.003034	1.60E-10	0.47741	462346	NA	rs3548336	NA	UKB-b-57	TRUE	reported	8KoqAI
3	71531708	T	C	-0.01945	0.003033	1.40E-10	0.481019	462346	NA	rs1171582	NA	UKB-b-57	TRUE	reported	8KoqAI
3	71532113	T	C	-0.0194	0.003034	1.60E-10	0.477439	462346	NA	rs6805189	NA	UKB-b-57	TRUE	reported	8KoqAI
3	71532621	C	T	-0.02299	0.003058	5.50E-14	0.431424	462346	NA	rs6224716	NA	UKB-b-57	TRUE	reported	8KoqAI
3	71532738	C	T	-0.01954	0.003034	1.20E-10	0.477288	462346	NA	rs6224716	NA	UKB-b-57	TRUE	reported	8KoqAI
3	71533378	G	C	-0.01939	0.003035	1.70E-10	0.479765	462346	NA	rs1332733	NA	UKB-b-57	TRUE	reported	8KoqAI
3	71534763	T	C	-0.02307	0.003058	4.50E-14	0.431463	462346	NA	rs1876245	NA	UKB-b-57	TRUE	reported	8KoqAI
3	71535338	T	G	-0.0196	0.003033	1.00E-10	0.480942	462346	NA	rs9878602	NA	UKB-b-57	TRUE	reported	8KoqAI
3	71539405	T	C	-0.02315	0.003056	3.60E-14	0.431473	462346	NA	rs3449279	NA	UKB-b-57	TRUE	reported	8KoqAI
3	71542279	C	A	-0.02339	0.003056	2.00E-14	0.431618	462346	NA	rs7309344	NA	UKB-b-57	TRUE	reported	8KoqAI
3	71543758	G	T	-0.0234	0.00306	2.10E-14	0.429512	462346	NA	rs7632921	NA	UKB-b-57	TRUE	reported	8KoqAI

3	71544252	T	C	-0.02308	0.003056	4.30E-14	0.431639	462346	NA	rs3576860	NA	UKB-b-57	TRUE	reported	8KoqAI
3	71544614	A	G	-0.01966	0.003032	8.90E-11	0.481376	462346	NA	rs7623765	NA	UKB-b-57	TRUE	reported	8KoqAI
3	71545170	C	A	-0.02313	0.00306	4.10E-14	0.430312	462346	NA	rs1172052	NA	UKB-b-57	TRUE	reported	8KoqAI
3	71547590	A	G	-0.02368	0.003058	9.70E-15	0.431803	462346	NA	rs6224486	NA	UKB-b-57	TRUE	reported	8KoqAI
3	71549639	T	C	-0.02334	0.003058	2.30E-14	0.432585	462346	NA	rs6779258	NA	UKB-b-57	TRUE	reported	8KoqAI
3	71550670	T	A	-0.02342	0.003059	1.90E-14	0.432318	462346	NA	rs6224486	NA	UKB-b-57	TRUE	reported	8KoqAI
3	71550774	T	C	-0.02335	0.003059	2.30E-14	0.432582	462346	NA	rs6224486	NA	UKB-b-57	TRUE	reported	8KoqAI
3	71551971	A	G	-0.02348	0.003058	1.60E-14	0.433507	462346	NA	rs9832796	NA	UKB-b-57	TRUE	reported	8KoqAI
3	71552119	G	A	-0.02347	0.003058	1.70E-14	0.433495	462346	NA	rs1392226	NA	UKB-b-57	TRUE	reported	8KoqAI
3	71552366	T	C	-0.02347	0.003058	1.70E-14	0.433507	462346	NA	rs9837383	NA	UKB-b-57	TRUE	reported	8KoqAI
3	71553132	T	G	-0.02368	0.003059	9.90E-15	0.433274	462346	NA	rs9842406	NA	UKB-b-57	TRUE	reported	8KoqAI
3	71554185	C	T	-0.02323	0.003066	3.60E-14	0.430556	462346	NA	rs6780776	NA	UKB-b-57	TRUE	reported	8KoqAI
3	71556471	A	G	-0.02357	0.003061	1.40E-14	0.432542	462346	NA	rs6075693	NA	UKB-b-57	TRUE	reported	8KoqAI
3	71557945	C	A	-0.02372	0.003069	1.10E-14	0.428123	462346	NA	rs3434664	NA	UKB-b-57	TRUE	reported	8KoqAI
3	71563561	C	T	-0.0231	0.00307	5.30E-14	0.428257	462346	NA	rs5792957	NA	UKB-b-57	TRUE	reported	8KoqAI
3	71563777	G	T	-0.02294	0.00307	7.90E-14	0.428399	462346	NA	rs6013520	NA	UKB-b-57	TRUE	reported	8KoqAI
3	71564943	G	A	-0.02319	0.003065	3.80E-14	0.430727	462346	NA	rs1766232	NA	UKB-b-57	TRUE	reported	8KoqAI
3	71569678	G	T	-0.02312	0.003069	4.90E-14	0.429134	462346	NA	rs1432611	NA	UKB-b-57	TRUE	reported	8KoqAI
3	71571345	C	T	-0.02308	0.003054	4.20E-14	0.441835	462346	NA	rs6803008	NA	UKB-b-57	TRUE	reported	8KoqAI
3	71571386	G	A	-0.02314	0.003054	3.60E-14	0.441597	462346	NA	rs6778026	NA	UKB-b-57	TRUE	reported	8KoqAI
3	71571667	T	C	-0.02302	0.003055	4.90E-14	0.441499	462346	NA	rs1499895	NA	UKB-b-57	TRUE	reported	8KoqAI
3	71571696	C	T	-0.02304	0.003059	5.00E-14	0.440171	462346	NA	rs1499894	NA	UKB-b-57	TRUE	reported	8KoqAI
3	71572338	C	G	-0.0229	0.003057	6.80E-14	0.440545	462346	NA	rs7649993	NA	UKB-b-57	TRUE	reported	8KoqAI
3	71572406	G	A	-0.02295	0.003057	6.10E-14	0.440559	462346	NA	rs5634379	NA	UKB-b-57	TRUE	reported	8KoqAI
3	71574051	C	T	-0.02323	0.003071	3.90E-14	0.428703	462346	NA	rs6764416	NA	UKB-b-57	TRUE	reported	8KoqAI
3	71576058	C	T	-0.02324	0.003071	3.80E-14	0.428743	462346	NA	rs1112429	NA	UKB-b-57	TRUE	reported	8KoqAI
3	71578737	T	A	-0.02346	0.003062	1.90E-14	0.437602	462346	NA	rs7624274	NA	UKB-b-57	TRUE	reported	8KoqAI
3	71579022	C	A	-0.02386	0.00307	7.70E-15	0.429053	462346	NA	rs7610856	NA	UKB-b-57	TRUE	reported	8KoqAI
3	71582521	G	A	-0.02372	0.003068	1.10E-14	0.430263	462346	NA	rs1171433	NA	UKB-b-57	TRUE	reported	8KoqAI
3	71583177	A	G	-0.02324	0.003059	3.00E-14	0.4396	462346	NA	rs3548056	NA	UKB-b-57	TRUE	reported	8KoqAI
3	71583383	G	A	-0.02379	0.003067	8.70E-15	0.431141	462346	NA	rs6224488	NA	UKB-b-57	TRUE	reported	8KoqAI
3	71585144	G	A	0.020017	0.003616	3.10E-08	0.228494	462346	NA	rs9866090	NA	UKB-b-57	TRUE	reported	8KoqAI
3	71586293	C	G	-0.02258	0.003093	2.90E-13	0.403657	462346	NA	rs5573631	NA	UKB-b-57	TRUE	reported	8KoqAI
3	71587392	C	G	-0.02321	0.003102	7.30E-14	0.395842	462346	NA	rs6224488	NA	UKB-b-57	TRUE	reported	8KoqAI
3	71589755	G	T	-0.02249	0.003096	3.80E-13	0.401699	462346	NA	rs2036281	NA	UKB-b-57	TRUE	reported	8KoqAI
3	71592594	C	T	-0.02302	0.003107	1.30E-13	0.393506	462346	NA	rs6790644	NA	UKB-b-57	TRUE	reported	8KoqAI
3	71594837	T	C	-0.02295	0.003093	1.20E-13	0.40403	462346	NA	rs1171997	NA	UKB-b-57	TRUE	reported	8KoqAI
3	71595258	T	C	-0.0221	0.003092	9.00E-13	0.405516	462346	NA	rs1172012	NA	UKB-b-57	TRUE	reported	8KoqAI
3	71599571	C	G	-0.02287	0.0031	1.60E-13	0.395292	462346	NA	rs7640614	NA	UKB-b-57	TRUE	reported	8KoqAI
3	71603361	C	T	-0.02298	0.0031	1.20E-13	0.39491	462346	NA	rs7309490	NA	UKB-b-57	TRUE	reported	8KoqAI
3	71603774	T	C	-0.02259	0.003067	1.80E-13	0.422029	462346	NA	rs6224488	NA	UKB-b-57	TRUE	reported	8KoqAI
3	71606466	T	G	-0.02268	0.003065	1.40E-13	0.423299	462346	NA	rs6549400	NA	UKB-b-57	TRUE	reported	8KoqAI
3	71607159	G	C	-0.02244	0.003065	2.50E-13	0.423151	462346	NA	rs1171887	NA	UKB-b-57	TRUE	reported	8KoqAI
3	71609007	G	A	-0.02229	0.003064	3.40E-13	0.424402	462346	NA	rs6224488	NA	UKB-b-57	TRUE	reported	8KoqAI
3	71609276	C	T	0.018436	0.003032	1.20E-09	0.494666	462346	NA	rs830605	NA	UKB-b-57	TRUE	reported	8KoqAI
3	71611630	T	C	-0.02314	0.003074	5.20E-14	0.414477	462346	NA	rs6224489	NA	UKB-b-57	TRUE	reported	8KoqAI
3	71640776	A	G	-0.02012	0.0031	8.50E-11	0.407559	462346	NA	rs1529675	NA	UKB-b-57	TRUE	reported	8KoqAI
3	71643296	G	A	-0.01952	0.003086	2.50E-10	0.412811	462346	NA	rs1170990	NA	UKB-b-57	TRUE	reported	8KoqAI
3	71643451	T	C	-0.01969	0.003085	1.70E-10	0.413706	462346	NA	rs830663	NA	UKB-b-57	TRUE	reported	8KoqAI
3	71645511	T	C	-0.01961	0.003084	2.00E-10	0.413791	462346	NA	rs830651	NA	UKB-b-57	TRUE	reported	8KoqAI
3	71648868	T	A	-0.01935	0.003083	3.40E-10	0.413088	462346	NA	rs853866	NA	UKB-b-57	TRUE	reported	8KoqAI
3	71650885	C	T	-0.0193	0.003083	3.80E-10	0.412538	462346	NA	rs830654	NA	UKB-b-57	TRUE	reported	8KoqAI
3	71656045	C	T	-0.01797	0.003078	5.30E-09	0.415427	462346	NA	rs844215	NA	UKB-b-57	TRUE	reported	8KoqAI
3	71667063	G	A	-0.01925	0.00308	4.10E-10	0.413283	462346	NA	rs830643	NA	UKB-b-57	TRUE	reported	8KoqAI
3	71668037	T	C	-0.01891	0.003077	8.00E-10	0.414819	462346	NA	rs1374915	NA	UKB-b-57	TRUE	reported	8KoqAI
3	71668055	G	A	-0.01909	0.003078	5.60E-10	0.415322	462346	NA	rs830642	NA	UKB-b-57	TRUE	reported	8KoqAI
3	71668528	G	T	-0.01898	0.003077	6.90E-10	0.415748	462346	NA	rs830641	NA	UKB-b-57	TRUE	reported	8KoqAI
3	71668564	G	A	-0.01903	0.003077	6.30E-10	0.415683	462346	NA	rs830640	NA	UKB-b-57	TRUE	reported	8KoqAI
3	71669656	G	A	-0.01903	0.003077	6.20E-10	0.417182	462346	NA	rs704255	NA	UKB-b-57	TRUE	reported	8KoqAI
3	71672666	T	C	-0.01779	0.003078	7.60E-09	0.415011	462346	NA	rs844219	NA	UKB-b-57	TRUE	reported	8KoqAI
3	71672825	C	T	-0.01745	0.003078	1.40E-08	0.413807	462346	NA	rs1766294	NA	UKB-b-57	TRUE	reported	8KoqAI
3	71673281	G	T	-0.01742	0.003077	1.50E-08	0.413947	462346	NA	rs830634	NA	UKB-b-57	TRUE	reported	8KoqAI
3	71673337	G	A	-0.01752	0.003077	1.20E-08	0.414008	462346	NA	rs830633	NA	UKB-b-57	TRUE	reported	8KoqAI
3	71673353	G	A	-0.01732	0.003077	1.80E-08	0.414538	462346	NA	rs830632	NA	UKB-b-57	TRUE	reported	8KoqAI
3	71673602	C	T	-0.0174	0.003077	1.60E-08	0.414338	462346	NA	rs830630	NA	UKB-b-57	TRUE	reported	8KoqAI
3	71675423	G	A	-0.01718	0.003076	2.30E-08	0.41374	462346	NA	rs862687	NA	UKB-b-57	TRUE	reported	8KoqAI
3	71676158	A	C	-0.01689	0.003074	3.90E-08	0.415134	462346	NA	rs6782815	NA	UKB-b-57	TRUE	reported	8KoqAI
3	71677457	G	A	-0.01718	0.003073	2.30E-08	0.413979	462346	NA	rs6224492	NA	UKB-b-57	TRUE	reported	8KoqAI
3	71677490	A	G	-0.01703	0.003072	3.00E-08	0.415484	462346	NA	rs5616743	NA	UKB-b-57	TRUE	reported	8KoqAI
3	71677981	C	A	-0.01699	0.003073	3.20E-08	0.413189	462346	NA	rs1766316	NA	UKB-b-57	TRUE	reported	8KoqAI
3	71679148	C	T	-0.01724	0.003069	1.90E-08	0.415634	462346	NA	rs830620	NA	UKB-b-57	TRUE	reported	8KoqAI
3	85015779	G	A	-0.01724	0.003032	1.30E-08	0.515376	462346	NA	rs1248860	NA	UKB-b-57	TRUE	reported	8KoqAI
3	85018612	G	A	-0.01731	0.00303	1.10E-08	0.515406	462346	NA	rs1248857	NA	UKB-b-57	TRUE	reported	8KoqAI
3	85035279	A	G	-0.01664	0.003034	4.10E-08	0.475493	462346	NA	rs9834688	NA	UKB-b-57	TRUE	reported	8KoqAI
3	85052150	C	T	-0.01677	0.003036	3.30E-08	0.475747	462346	NA	rs9831123	NA	UKB-b-57	TRUE	reported	8KoqAI
3	85053425	T	A	-0.01673	0.003037	3.60E-08	0.475661	462346	NA	rs1872553	NA	UKB-b-57	TRUE	reported	8KoqAI
3	85057281	C	T	-0.01671	0.003036	3.70E-08	0.475679	462346	NA	rs4301023	NA	UKB-b-57	TRUE	reported	8KoqAI
3	85057349	G	T	-0.01672	0.003037	3.70E-08	0.475717	462346	NA	rs7651996	NA	UKB-b-57	TRUE	reported	8KoqAI
3	85067018	A	G	-0.01683	0.003042	3.20E-08	0.475766	462346	NA	rs9816652	NA	UKB-b-57	TRUE	reported	8KoqAI
3	85236425	G	A	-0.01822	0.003296	3.20E-08	0.314267	462346	NA	rs1376935	NA	UKB-b-57	TRUE	reported	8KoqAI
3	85264266	G	A	-0.0187	0.003272	1.10E-08	0.311666	462346	NA	rs1307838	NA	UKB-b-57	TRUE	reported	8KoqAI
3	85370873	C	T	-0.01882	0.003288	1.00E-08	0.30637	462346	NA	rs9831374	NA	UKB-b-57	TRUE	reported	8KoqAI
3	85371838	A	G	-0.01882	0.003288	1.00E-08	0.306384	462346	NA	rs1171927	NA	UKB-b-57	TRUE	reported	8KoqAI
3	85387310	A	T	-0.01907	0.003166	1.70E-09	0.378188	462346	NA	rs9309973	NA	UKB-b-57	TRUE	reported	8KoqAI
3	85391672	G	A	-0.02093	0.003255	1.30E-10	0.317083	462346	NA	rs9811546	NA	UKB-b-57	TRUE	reported	8KoqAI
3	85397006	G	A	-0.01945	0.003382	8.90E-09	0.28755	462346	NA	rs9810508	NA	UKB-b-57	TRUE	reported	8KoqAI
3	85399603	G	T	-0.01749	0.003135	2.40E-08	0.372785	462346	NA	rs3887151	NA	UKB-b-57	TRUE	reported	8KoqAI
3	85400801	T	C	0.018708	0.003228	6.80E-09	0.672419	462346	NA	rs6225308	NA	UKB-b-57	TRUE	reported	8KoqAI
3	85401822	T	G	-0.01738	0.003136	3.00E-08	0.372049	462346	NA	rs1249342	NA	UKB-b-57	TRUE	reported	8KoqAI
3	85401867	T	G	-0.01744	0.003134	2.60E-08	0.372941	462346	NA	rs986035	NA	UKB-b-57	TRUE	reported	8KoqAI
3	85402469	T	C	-0.01744	0.003133	2.60E-08	0.374088	462346	NA	rs9861237	NA	UKB-b-57	TRUE	reported	8KoqAI
3	85403892	A	G	0.02352	0.00344	8.10E-12	0.736905	462346	NA	rs2873237	NA	UKB-b-57	TRUE	reported	8KoqAI
3	85404030	T	G	0.023383	0.003394	5.60E-12	0.725309	462346	NA	rs6225310	NA	UKB-b-57	TRUE	reported	8KoqAI
3	85404783	G	T	0.018638	0.003179	4.50E-09	0.6525	462346	NA	rs2117152	NA	UKB-b-57	TRUE	reported	8KoqAI
3	85404960	G	A	-0.01912	0.003269	5.00E-09	0.31056	462346	NA	rs2163972	NA	UKB-b-57	TRUE	reported	8KoqAI
3	85405501	T	C	-0.02529	0.003623	2.90E-12	0.224925	462346	NA	rs9822731	NA	UKB-b-57	TRUE	reported	8KoqAI
3	85406367	C	T	-0.02476	0.003556	3.40E-12	0.236962	462346	NA	rs1249801	NA	UKB-b-57	TRUE	reported	8KoqAI
3	85406735	A	G	0.018581	0.003178	5.00E-09	0.652637	462346	NA	rs4856269	NA	UKB-b-57	TRUE	reported	8KoqAI
3	85406931	G	C	-0.01915	0.00327	4.80E-09	0.310254	462346	NA	rs9809258	NA	UKB-b-57	TRUE	reported	8KoqAI
3	85406938	G	T	-0.01917	0.00327	4.60E-09	0.310329	462346	NA	rs1170577	NA	UKB-b-57	TRUE	reported	8KoqAI
3	85407544	A	T	-0.0186	0.003179	4.90E-09	0.347402	462346	NA	rs1263494	NA	UKB-b-57	TRUE	reported	8KoqAI
3	85407546	T	A	0.018482	0.003178	6.10E-09	0.650273	462346	NA	rs7261572	NA	UKB-b-57	TRUE	reported	8KoqAI
3	85407980	G	T	-0.02511	0.003556	1.60E-12	0.237423	462346	NA	rs9814516	NA	UKB-b-57	TRUE	reported	8KoqAI
3	85409299	C	T	-0.02531	0.003623	2.80E-12	0.224943	462346	NA	rs1015486	NA	UKB-b-57	TRUE	reported	8KoqAI
3	85409313	G	A	0.023204	0.003392	7.90E-12	0.72613	462346	NA	rs9824178	NA	UKB-b-57	TRUE	reported	8KoqAI
3	85409353	A	G	0.023192	0.003392	8.10E-12	0.726139	462346	NA	rs9861451	NA	UKB-b-57	TRUE	reported	8KoqAI
3	85409470	G	A	0.023311	0.003387	5.80E-12	0.724643	462346	NA	rs1191749	NA	UKB-b-57	TRUE	reported	8KoqAI

3	85409884	C	T	-0.02475	0.003556	3.40E-12	0.236942	462346	NA	rs1307663	NA	UKB-b-57	TRUE	reported	8KoqAI
3	85409979	C	T	-0.02531	0.003623	2.80E-12	0.224968	462346	NA	rs1307683	NA	UKB-b-57	TRUE	reported	8KoqAI
3	85410088	T	A	-0.02476	0.003556	3.30E-12	0.237022	462346	NA	rs1309975	NA	UKB-b-57	TRUE	reported	8KoqAI
3	85411012	G	A	-0.01903	0.003267	5.70E-09	0.310679	462346	NA	rs1271462	NA	UKB-b-57	TRUE	reported	8KoqAI
3	85411193	G	T	0.018486	0.003158	4.80E-09	0.643711	462346	NA	rs9990096	NA	UKB-b-57	TRUE	reported	8KoqAI
3	85412330	A	G	0.018634	0.003173	4.30E-09	0.650865	462346	NA	rs1368748	NA	UKB-b-57	TRUE	reported	8KoqAI
3	85412537	A	G	-0.01909	0.003266	5.10E-09	0.310897	462346	NA	rs1309410	NA	UKB-b-57	TRUE	reported	8KoqAI
3	85413180	T	C	-0.01895	0.003241	5.00E-09	0.319474	462346	NA	rs1249424	NA	UKB-b-57	TRUE	reported	8KoqAI
3	85413937	C	A	-0.02522	0.003621	3.30E-12	0.225269	462346	NA	rs9846211	NA	UKB-b-57	TRUE	reported	8KoqAI
3	85413995	T	C	-0.0243	0.003512	4.50E-12	0.24577	462346	NA	rs9866089	NA	UKB-b-57	TRUE	reported	8KoqAI
3	85414291	T	C	0.023091	0.003391	9.80E-12	0.725952	462346	NA	rs7627971	NA	UKB-b-57	TRUE	reported	8KoqAI
3	85414856	G	A	-0.02475	0.003555	3.40E-12	0.237182	462346	NA	rs1112788	NA	UKB-b-57	TRUE	reported	8KoqAI
3	85416384	A	G	-0.02517	0.003622	3.70E-12	0.225161	462346	NA	rs9870448	NA	UKB-b-57	TRUE	reported	8KoqAI
3	85420724	C	A	-0.02518	0.003622	3.60E-12	0.225169	462346	NA	rs9854869	NA	UKB-b-57	TRUE	reported	8KoqAI
3	85421737	C	G	-0.02425	0.003512	5.10E-12	0.245804	462346	NA	rs4279114	NA	UKB-b-57	TRUE	reported	8KoqAI
3	85422587	C	T	-0.02424	0.003513	5.20E-12	0.245738	462346	NA	rs9865191	NA	UKB-b-57	TRUE	reported	8KoqAI
3	85426840	G	A	0.022947	0.003355	7.90E-12	0.71575	462346	NA	rs6776114	NA	UKB-b-57	TRUE	reported	8KoqAI
3	85428726	T	C	0.019415	0.003055	2.10E-10	0.550706	462346	NA	rs2163971	NA	UKB-b-57	TRUE	reported	8KoqAI
3	85428963	C	T	-0.02425	0.003515	5.20E-12	0.245508	462346	NA	rs1433711	NA	UKB-b-57	TRUE	reported	8KoqAI
3	85429172	C	A	-0.02429	0.003515	4.80E-12	0.245548	462346	NA	rs1308517	NA	UKB-b-57	TRUE	reported	8KoqAI
3	85430137	T	C	-0.0242	0.003515	5.80E-12	0.245394	462346	NA	rs9819830	NA	UKB-b-57	TRUE	reported	8KoqAI
3	85432315	G	C	-0.02428	0.003515	5.00E-12	0.245346	462346	NA	rs9814919	NA	UKB-b-57	TRUE	reported	8KoqAI
3	85434260	T	C	0.020142	0.003097	7.80E-11	0.599678	462346	NA	rs1192101	NA	UKB-b-57	TRUE	reported	8KoqAI
3	85434357	T	A	-0.02427	0.003515	5.00E-12	0.245402	462346	NA	rs2326313	NA	UKB-b-57	TRUE	reported	8KoqAI
3	85436125	C	T	-0.0244	0.003514	3.80E-12	0.245585	462346	NA	rs9879025	NA	UKB-b-57	TRUE	reported	8KoqAI
3	85438564	A	G	-0.02423	0.003515	5.40E-12	0.245397	462346	NA	rs1821349	NA	UKB-b-57	TRUE	reported	8KoqAI
3	85438709	A	G	-0.02423	0.003515	5.40E-12	0.245397	462346	NA	rs1368743	NA	UKB-b-57	TRUE	reported	8KoqAI
3	85439136	A	G	0.020464	0.003115	5.10E-11	0.618622	462346	NA	rs1051108	NA	UKB-b-57	TRUE	reported	8KoqAI
3	85442208	A	G	0.020625	0.003115	3.50E-11	0.618328	462346	NA	rs1368742	NA	UKB-b-57	TRUE	reported	8KoqAI
3	85442709	C	T	-0.02426	0.003515	5.10E-12	0.245388	462346	NA	rs9309977	NA	UKB-b-57	TRUE	reported	8KoqAI
3	85443793	G	A	0.020447	0.003115	5.20E-11	0.618497	462346	NA	rs1991872	NA	UKB-b-57	TRUE	reported	8KoqAI
3	85446558	G	A	-0.02422	0.003515	5.60E-12	0.245535	462346	NA	rs9833314	NA	UKB-b-57	TRUE	reported	8KoqAI
3	85446776	A	G	0.020422	0.003115	5.50E-11	0.618537	462346	NA	rs1263779	NA	UKB-b-57	TRUE	reported	8KoqAI
3	85448493	T	C	-0.02412	0.003515	6.70E-12	0.245558	462346	NA	rs9863620	NA	UKB-b-57	TRUE	reported	8KoqAI
3	85449777	G	T	-0.02418	0.003515	6.00E-12	0.24553	462346	NA	rs7162688	NA	UKB-b-57	TRUE	reported	8KoqAI
3	85451804	C	A	-0.02413	0.003515	6.70E-12	0.245486	462346	NA	rs7650834	NA	UKB-b-57	TRUE	reported	8KoqAI
3	85454815	C	T	0.020302	0.003114	7.00E-11	0.618103	462346	NA	rs1263848	NA	UKB-b-57	TRUE	reported	8KoqAI
3	85457240	A	G	0.020667	0.003113	3.20E-11	0.618047	462346	NA	rs6225068	NA	UKB-b-57	TRUE	reported	8KoqAI
3	85458577	A	G	0.020267	0.003114	7.60E-11	0.618143	462346	NA	rs1191889	NA	UKB-b-57	TRUE	reported	8KoqAI
3	85458683	C	A	-0.02391	0.003513	1.00E-11	0.245749	462346	NA	rs2117151	NA	UKB-b-57	TRUE	reported	8KoqAI
3	85458861	T	C	-0.02389	0.003514	1.10E-11	0.245812	462346	NA	rs1030721	NA	UKB-b-57	TRUE	reported	8KoqAI
3	85460131	C	T	0.020485	0.003113	4.70E-11	0.617782	462346	NA	rs1549979	NA	UKB-b-57	TRUE	reported	8KoqAI
3	85461302	G	A	-0.02402	0.003518	8.70E-12	0.244737	462346	NA	rs9880919	NA	UKB-b-57	TRUE	reported	8KoqAI
3	85462587	T	C	-0.02505	0.003621	4.50E-12	0.225642	462346	NA	rs6791236	NA	UKB-b-57	TRUE	reported	8KoqAI
3	85464125	A	G	0.020283	0.003113	7.30E-11	0.618119	462346	NA	rs4856569	NA	UKB-b-57	TRUE	reported	8KoqAI
3	85466452	T	C	-0.02388	0.003513	1.10E-11	0.24581	462346	NA	rs6549024	NA	UKB-b-57	TRUE	reported	8KoqAI
3	85467972	C	A	0.020129	0.003113	1.00E-10	0.618412	462346	NA	rs2053108	NA	UKB-b-57	TRUE	reported	8KoqAI
3	85468059	T	C	0.020151	0.003114	9.70E-11	0.618424	462346	NA	rs6808400	NA	UKB-b-57	TRUE	reported	8KoqAI
3	85468919	A	G	-0.02397	0.003513	8.80E-12	0.245807	462346	NA	rs1249346	NA	UKB-b-57	TRUE	reported	8KoqAI
3	85469041	C	G	0.020145	0.003114	9.80E-11	0.618462	462346	NA	rs6780968	NA	UKB-b-57	TRUE	reported	8KoqAI
3	85469353	T	A	0.020101	0.003114	1.10E-10	0.618286	462346	NA	rs6225068	NA	UKB-b-57	TRUE	reported	8KoqAI
3	85470642	T	C	-0.02502	0.003632	5.70E-12	0.224704	462346	NA	rs2196096	NA	UKB-b-57	TRUE	reported	8KoqAI
3	85470645	C	T	-0.025	0.003631	5.80E-12	0.224711	462346	NA	rs2196097	NA	UKB-b-57	TRUE	reported	8KoqAI
3	85470687	T	G	0.020375	0.003113	5.90E-11	0.617965	462346	NA	rs6031153	NA	UKB-b-57	TRUE	reported	8KoqAI
3	85471818	G	A	-0.02364	0.003513	1.70E-11	0.245621	462346	NA	rs1043349	NA	UKB-b-57	TRUE	reported	8KoqAI
3	85472033	C	T	0.019988	0.003113	1.40E-10	0.618575	462346	NA	rs7636243	NA	UKB-b-57	TRUE	reported	8KoqAI
3	85472227	T	C	0.019972	0.003113	1.40E-10	0.618573	462346	NA	rs7650284	NA	UKB-b-57	TRUE	reported	8KoqAI
3	85472462	G	A	0.020102	0.003113	1.10E-10	0.618469	462346	NA	rs7638953	NA	UKB-b-57	TRUE	reported	8KoqAI
3	85473317	A	G	-0.0237	0.003512	1.50E-11	0.245638	462346	NA	rs6772991	NA	UKB-b-57	TRUE	reported	8KoqAI
3	85473932	T	A	-0.02364	0.003513	1.70E-11	0.245597	462346	NA	rs9865745	NA	UKB-b-57	TRUE	reported	8KoqAI
3	85474868	G	C	0.020316	0.003113	6.70E-11	0.617845	462346	NA	rs4856571	NA	UKB-b-57	TRUE	reported	8KoqAI
3	85475597	T	A	-0.02363	0.003516	1.80E-11	0.245403	462346	NA	rs744580	NA	UKB-b-57	TRUE	reported	8KoqAI
3	85475647	T	C	0.020098	0.003114	1.10E-10	0.618222	462346	NA	rs3589454	NA	UKB-b-57	TRUE	reported	8KoqAI
3	85476094	G	A	-0.02362	0.003516	1.80E-11	0.245457	462346	NA	rs1308123	NA	UKB-b-57	TRUE	reported	8KoqAI
3	85476898	C	T	-0.02367	0.003516	1.70E-11	0.245446	462346	NA	rs6808159	NA	UKB-b-57	TRUE	reported	8KoqAI
3	85477017	T	G	0.020025	0.003116	1.30E-10	0.618703	462346	NA	rs2326309	NA	UKB-b-57	TRUE	reported	8KoqAI
3	85477032	G	A	0.019994	0.003116	1.40E-10	0.618694	462346	NA	rs2326310	NA	UKB-b-57	TRUE	reported	8KoqAI
3	85480184	C	T	-0.02373	0.003514	1.40E-11	0.245658	462346	NA	rs1025533	NA	UKB-b-57	TRUE	reported	8KoqAI
3	85481412	T	C	0.020338	0.003113	6.50E-11	0.618133	462346	NA	rs1865250	NA	UKB-b-57	TRUE	reported	8KoqAI
3	85481659	C	T	-0.02488	0.003621	6.40E-12	0.225622	462346	NA	rs1171623	NA	UKB-b-57	TRUE	reported	8KoqAI
3	85481979	T	C	0.020031	0.003114	1.30E-10	0.618792	462346	NA	rs1433708	NA	UKB-b-57	TRUE	reported	8KoqAI
3	85482287	C	T	0.019989	0.003115	1.40E-10	0.618777	462346	NA	rs9860769	NA	UKB-b-57	TRUE	reported	8KoqAI
3	85482595	G	A	0.020099	0.003114	1.10E-10	0.618544	462346	NA	rs7609594	NA	UKB-b-57	TRUE	reported	8KoqAI
3	85482661	G	A	0.020142	0.003114	9.90E-11	0.61848	462346	NA	rs3582724	NA	UKB-b-57	TRUE	reported	8KoqAI
3	85485087	G	C	0.020038	0.003114	1.20E-10	0.618797	462346	NA	rs2196099	NA	UKB-b-57	TRUE	reported	8KoqAI
3	85485110	C	T	-0.02369	0.003514	1.60E-11	0.245611	462346	NA	rs1309262	NA	UKB-b-57	TRUE	reported	8KoqAI
3	85485230	G	T	0.020028	0.003114	1.30E-10	0.618798	462346	NA	rs2217720	NA	UKB-b-57	TRUE	reported	8KoqAI
3	85486931	T	A	-0.02375	0.003514	1.40E-11	0.245602	462346	NA	rs1025531	NA	UKB-b-57	TRUE	reported	8KoqAI
3	85487465	G	A	0.020029	0.003114	1.30E-10	0.618797	462346	NA	rs9863488	NA	UKB-b-57	TRUE	reported	8KoqAI
3	85487482	T	C	0.020336	0.003113	6.50E-11	0.618129	462346	NA	rs1192952	NA	UKB-b-57	TRUE	reported	8KoqAI
3	85488325	C	T	0.020029	0.003114	1.30E-10	0.618798	462346	NA	rs1821351	NA	UKB-b-57	TRUE	reported	8KoqAI
3	85488353	G	T	0.020151	0.003114	9.70E-11	0.618471	462346	NA	rs1263746	NA	UKB-b-57	TRUE	reported	8KoqAI
3	85488462	C	T	-0.02374	0.003514	1.40E-11	0.245608	462346	NA	rs9868427	NA	UKB-b-57	TRUE	reported	8KoqAI
3	85488646	T	G	-0.02374	0.003514	1.40E-11	0.245606	462346	NA	rs9813512	NA	UKB-b-57	TRUE	reported	8KoqAI
3	85488740	A	G	0.020028	0.003115	1.30E-10	0.618797	462346	NA	rs1865252	NA	UKB-b-57	TRUE	reported	8KoqAI
3	85489069	A	C	0.020153	0.003114	9.70E-11	0.618446	462346	NA	rs1865251	NA	UKB-b-57	TRUE	reported	8KoqAI
3	85489304	A	T	0.020387	0.003113	5.80E-11	0.618114	462346	NA	rs1897699	NA	UKB-b-57	TRUE	reported	8KoqAI
3	85489906	G	A	-0.02378	0.003514	1.30E-11	0.245616	462346	NA	rs9874740	NA	UKB-b-57	TRUE	reported	8KoqAI
3	85490947	G	A	0.020379	0.003113	5.90E-11	0.618113	462346	NA	rs2082556	NA	UKB-b-57	TRUE	reported	8KoqAI
3	85493225	G	A	0.020066	0.003114	1.20E-10	0.618496	462346	NA	rs7627044	NA	UKB-b-57	TRUE	reported	8KoqAI
3	85493753	C	T	-0.02484	0.003622	7.00E-12	0.225578	462346	NA	rs9820228	NA	UKB-b-57	TRUE	reported	8KoqAI
3	85494267	C	T	-0.0238	0.003514	1.20E-11	0.245619	462346	NA	rs9821041	NA	UKB-b-57	TRUE	reported	8KoqAI
3	85494419	T	C	0.020195	0.003114	8.80E-11	0.618455	462346	NA	rs4856572	NA	UKB-b-57	TRUE	reported	8KoqAI
3	85496631	C	T	-0.0237	0.003514	1.60E-11	0.245576	462346	NA	rs1112789	NA	UKB-b-57	TRUE	reported	8KoqAI
3	85496758	A	C	0.020112	0.003114	1.10E-10	0.618494	462346	NA	rs2033526	NA	UKB-b-57	TRUE	reported	8KoqAI
3	85497198	T	C	-0.02513	0.003624	4.10E-12	0.22538	462346	NA	rs9828679	NA	UKB-b-57	TRUE	reported	8KoqAI
3	85497807	A	G	0.019917	0.003115	1.60E-10	0.619076	462346	NA	rs1192295	NA	UKB-b-57	TRUE	reported	8KoqAI
3	85499035	C	T	0.020256	0.003114	7.80E-11	0.618226	462346	NA	rs993137	NA	UKB-b-57	TRUE	reported	8KoqAI
3	85499333	T	C	0.020026	0.003115	1.30E-10	0.618738	462346	NA	rs993136	NA	UKB-b-57	TRUE	reported	8KoqAI
3	85500389	A	G	0.020069	0.003115	1.20E-10	0.618779	462346	NA	rs6762695	NA	UKB-b-57	TRUE	reported	8KoqAI
3	85502083	G	A	-0.0238	0.003515	1.30E-11	0.245527	462346	NA	rs9835441	NA	UKB-b-57	TRUE	reported	8KoqAI
3	85502591	C	T	0.020031	0.003115	1.30E-10	0.618734	462346	NA	rs993716	NA	UKB-b-57	TRUE	reported	8KoqAI
3	85504452	G	A	0.019978	0.003115	1.40E-10	0.618651	462346	NA	rs2117153	NA	UKB-b-57	TRUE	reported	8KoqAI
3	85504949	T	G	0.020057	0.003115	1.20E-10	0.618731	462346	NA	rs1433714	NA	UKB-b-57	TRUE	reported	8KoqAI
3	85505246	A	G	-0.02502	0.003622	5.00E-12	0.225467	462346	NA	rs2196098	NA	UKB-b-57	TRUE	reported	8KoqAI
3	85505874	A	G	-0.0238	0.003515	1.30E-11	0.245531	462346	NA	rs9309980	NA	UKB-b-57	TRUE	reported	8KoqAI
3	85505944	A	G	-0.0238	0.003515	1.30E-11	0.245531	462346	NA	rs9309981	NA	UKB-b-57	TRUE	reported	8KoqAI

3	85507231	T	C	-0.0238	0.003515	1.30E-11	0.245524	462346	NA	rs9309982	NA	UKB-b-57	TRUE	reported	8KoqAI
3	85508499	A	G	-0.0232	0.003492	3.00E-11	0.252012	462346	NA	rs1368740	NA	UKB-b-57	TRUE	reported	8KoqAI
3	85508582	T	C	-0.02381	0.003515	1.30E-11	0.245524	462346	NA	rs1368739	NA	UKB-b-57	TRUE	reported	8KoqAI
3	85508814	A	G	-0.02495	0.003622	5.70E-12	0.225531	462346	NA	rs1306843	NA	UKB-b-57	TRUE	reported	8KoqAI
3	85508953	G	A	0.020005	0.003115	1.30E-10	0.61873	462346	NA	rs4856573	NA	UKB-b-57	TRUE	reported	8KoqAI
3	85509991	T	C	-0.02395	0.003513	9.20E-12	0.246026	462346	NA	rs1263376	NA	UKB-b-57	TRUE	reported	8KoqAI
3	85510183	T	C	-0.02395	0.003512	9.20E-12	0.246033	462346	NA	rs1112789	NA	UKB-b-57	TRUE	reported	8KoqAI
3	85510543	A	T	0.020126	0.003114	1.00E-10	0.618239	462346	NA	rs6225071	NA	UKB-b-57	TRUE	reported	8KoqAI
3	85512824	C	A	-0.02396	0.003513	9.00E-12	0.246028	462346	NA	rs9838811	NA	UKB-b-57	TRUE	reported	8KoqAI
3	85513000	T	C	0.020157	0.003114	9.60E-11	0.618222	462346	NA	rs6774985	NA	UKB-b-57	TRUE	reported	8KoqAI
3	85513115	C	A	0.020399	0.003113	5.70E-11	0.617663	462346	NA	rs6762267	NA	UKB-b-57	TRUE	reported	8KoqAI
3	85513716	C	T	0.020441	0.003114	5.20E-11	0.617522	462346	NA	rs6225071	NA	UKB-b-57	TRUE	reported	8KoqAI
3	85513793	A	G	0.020497	0.003145	7.20E-11	0.636072	462346	NA	rs6225071	NA	UKB-b-57	TRUE	reported	8KoqAI
3	85515776	G	A	0.020315	0.003128	8.30E-11	0.626996	462346	NA	rs6225071	NA	UKB-b-57	TRUE	reported	8KoqAI
3	85515996	G	A	0.0203	0.003127	8.50E-11	0.626974	462346	NA	rs6225071	NA	UKB-b-57	TRUE	reported	8KoqAI
3	85517507	T	A	-0.0248	0.003606	6.20E-12	0.228598	462346	NA	rs1307016	NA	UKB-b-57	TRUE	reported	8KoqAI
3	85518580	A	G	0.020537	0.003165	8.60E-11	0.646976	462346	NA	rs2875907	NA	UKB-b-57	TRUE	reported	8KoqAI
3	85519411	T	C	0.020399	0.003125	6.70E-11	0.626398	462346	NA	rs1368750	NA	UKB-b-57	TRUE	reported	8KoqAI
3	85521017	T	C	0.020398	0.003125	6.70E-11	0.626398	462346	NA	rs1155666	NA	UKB-b-57	TRUE	reported	8KoqAI
3	85522794	T	G	0.020399	0.003125	6.70E-11	0.626443	462346	NA	rs2069123	NA	UKB-b-57	TRUE	reported	8KoqAI
3	85524170	C	T	0.020584	0.003164	7.80E-11	0.647099	462346	NA	rs1112789	NA	UKB-b-57	TRUE	reported	8KoqAI
3	85524474	T	A	0.020487	0.003125	5.50E-11	0.626426	462346	NA	rs6733664	NA	UKB-b-57	TRUE	reported	8KoqAI
3	85525323	T	G	0.020377	0.003165	1.20E-10	0.64735	462346	NA	rs1263779	NA	UKB-b-57	TRUE	reported	8KoqAI
3	85526866	C	G	0.020543	0.003125	4.90E-11	0.626351	462346	NA	rs7621381	NA	UKB-b-57	TRUE	reported	8KoqAI
3	85527334	G	A	0.020543	0.003125	4.90E-11	0.626351	462346	NA	rs1542248	NA	UKB-b-57	TRUE	reported	8KoqAI
3	85529628	G	A	0.020498	0.003125	5.40E-11	0.626422	462346	NA	rs7614148	NA	UKB-b-57	TRUE	reported	8KoqAI
3	85531199	A	T	-0.02497	0.003612	4.70E-12	0.22678	462346	NA	rs9841144	NA	UKB-b-57	TRUE	reported	8KoqAI
3	85531850	G	T	0.020483	0.003126	5.60E-11	0.6263	462346	NA	rs6225071	NA	UKB-b-57	TRUE	reported	8KoqAI
3	85531887	C	A	0.020483	0.003126	5.60E-11	0.6263	462346	NA	rs1051108	NA	UKB-b-57	TRUE	reported	8KoqAI
3	85532119	G	A	0.020498	0.003125	5.40E-11	0.626419	462346	NA	rs1751519	NA	UKB-b-57	TRUE	reported	8KoqAI
3	85534767	A	G	0.020612	0.003164	7.30E-11	0.647096	462346	NA	rs1813696	NA	UKB-b-57	TRUE	reported	8KoqAI
3	85536314	C	T	0.02061	0.003164	7.40E-11	0.647044	462346	NA	rs2077839	NA	UKB-b-57	TRUE	reported	8KoqAI
3	85537665	A	C	-0.02497	0.003612	4.80E-12	0.226826	462346	NA	rs9849399	NA	UKB-b-57	TRUE	reported	8KoqAI
3	85539078	A	G	0.020569	0.003165	8.00E-11	0.647041	462346	NA	rs7647981	NA	UKB-b-57	TRUE	reported	8KoqAI
3	85539234	T	C	0.020621	0.003164	7.20E-11	0.647044	462346	NA	rs6741640	NA	UKB-b-57	TRUE	reported	8KoqAI
3	85539810	T	G	0.020531	0.003125	5.00E-11	0.62636	462346	NA	rs1112789	NA	UKB-b-57	TRUE	reported	8KoqAI
3	85539816	C	T	0.020532	0.003125	5.00E-11	0.626364	462346	NA	rs1112789	NA	UKB-b-57	TRUE	reported	8KoqAI
3	85540764	A	C	0.02062	0.003164	7.20E-11	0.647046	462346	NA	rs5775542	NA	UKB-b-57	TRUE	reported	8KoqAI
3	85544070	C	A	-0.02495	0.003613	5.00E-12	0.226939	462346	NA	rs9844512	NA	UKB-b-57	TRUE	reported	8KoqAI
3	85544210	G	T	0.020597	0.003126	4.40E-11	0.626323	462346	NA	rs1551043	NA	UKB-b-57	TRUE	reported	8KoqAI
3	85544283	T	C	0.020619	0.003165	7.30E-11	0.646947	462346	NA	rs1551044	NA	UKB-b-57	TRUE	reported	8KoqAI
3	85545179	T	A	0.020622	0.003164	7.20E-11	0.64702	462346	NA	rs7614552	NA	UKB-b-57	TRUE	reported	8KoqAI
3	85545866	T	C	0.020621	0.003165	7.30E-11	0.646952	462346	NA	rs1043352	NA	UKB-b-57	TRUE	reported	8KoqAI
3	85546701	C	T	0.020617	0.003165	7.30E-11	0.646952	462346	NA	rs1043349	NA	UKB-b-57	TRUE	reported	8KoqAI
3	85546798	G	A	0.020531	0.00315	7.10E-11	0.634439	462346	NA	rs1043350	NA	UKB-b-57	TRUE	reported	8KoqAI
3	85548686	A	G	0.020686	0.003165	6.30E-11	0.646796	462346	NA	rs7618124	NA	UKB-b-57	TRUE	reported	8KoqAI
3	85549020	A	G	0.020521	0.003126	5.20E-11	0.626254	462346	NA	rs7618429	NA	UKB-b-57	TRUE	reported	8KoqAI
3	85550080	T	C	0.020624	0.003165	7.20E-11	0.646949	462346	NA	rs6549030	NA	UKB-b-57	TRUE	reported	8KoqAI
3	85550969	T	C	0.020551	0.003125	4.80E-11	0.626332	462346	NA	rs6225075	NA	UKB-b-57	TRUE	reported	8KoqAI
3	85551403	C	T	0.020427	0.003169	1.10E-10	0.648632	462346	NA	rs726610	NA	UKB-b-57	TRUE	reported	8KoqAI
3	85551820	T	C	0.020649	0.003164	6.80E-11	0.64702	462346	NA	rs1263879	NA	UKB-b-57	TRUE	reported	8KoqAI
3	85552787	C	T	0.020417	0.003166	1.10E-10	0.647299	462346	NA	rs4637303	NA	UKB-b-57	TRUE	reported	8KoqAI
3	85553096	C	A	-0.02502	0.003612	4.30E-12	0.226848	462346	NA	rs6809805	NA	UKB-b-57	TRUE	reported	8KoqAI
3	85553994	C	G	-0.02502	0.003613	4.40E-12	0.226954	462346	NA	rs1308453	NA	UKB-b-57	TRUE	reported	8KoqAI
3	85554582	G	T	0.020516	0.003126	5.30E-11	0.626202	462346	NA	rs1449391	NA	UKB-b-57	TRUE	reported	8KoqAI
3	85557571	G	T	0.020558	0.003125	4.70E-11	0.626244	462346	NA	rs6225075	NA	UKB-b-57	TRUE	reported	8KoqAI
3	85561808	T	C	0.020652	0.003165	6.80E-11	0.646856	462346	NA	rs1112789	NA	UKB-b-57	TRUE	reported	8KoqAI
3	85563846	A	C	0.020582	0.003125	4.50E-11	0.626182	462346	NA	rs6804845	NA	UKB-b-57	TRUE	reported	8KoqAI
3	85564863	G	T	0.020575	0.003124	4.50E-11	0.626281	462346	NA	rs6225075	NA	UKB-b-57	TRUE	reported	8KoqAI
3	85565494	T	C	0.020653	0.003164	6.70E-11	0.646959	462346	NA	rs1745599	NA	UKB-b-57	TRUE	reported	8KoqAI
3	85565728	G	T	0.020579	0.003124	4.50E-11	0.626268	462346	NA	rs1375560	NA	UKB-b-57	TRUE	reported	8KoqAI
3	85569068	G	A	0.020604	0.003165	7.50E-11	0.646961	462346	NA	rs1992967	NA	UKB-b-57	TRUE	reported	8KoqAI
3	85569172	A	G	0.020529	0.003125	5.10E-11	0.62627	462346	NA	rs1751558	NA	UKB-b-57	TRUE	reported	8KoqAI
3	85569361	T	G	-0.02486	0.003611	5.80E-12	0.226996	462346	NA	rs9841829	NA	UKB-b-57	TRUE	reported	8KoqAI
3	85570959	A	G	0.020532	0.003125	5.00E-11	0.62627	462346	NA	rs1051108	NA	UKB-b-57	TRUE	reported	8KoqAI
3	85571253	A	C	0.020568	0.003124	4.60E-11	0.626264	462346	NA	rs6764988	NA	UKB-b-57	TRUE	reported	8KoqAI
3	85571883	C	T	0.020549	0.003125	4.90E-11	0.626277	462346	NA	rs1868532	NA	UKB-b-57	TRUE	reported	8KoqAI
3	85572549	C	T	0.020549	0.003125	4.90E-11	0.626277	462346	NA	rs1449393	NA	UKB-b-57	TRUE	reported	8KoqAI
3	85572912	C	T	0.020585	0.003124	4.40E-11	0.626266	462346	NA	rs1449394	NA	UKB-b-57	TRUE	reported	8KoqAI
3	85573086	T	C	0.020549	0.003125	4.90E-11	0.626277	462346	NA	rs4856579	NA	UKB-b-57	TRUE	reported	8KoqAI
3	85573333	T	A	0.020549	0.003125	4.90E-11	0.626277	462346	NA	rs4856580	NA	UKB-b-57	TRUE	reported	8KoqAI
3	85574072	T	C	0.020598	0.003124	4.30E-11	0.626269	462346	NA	rs5882894	NA	UKB-b-57	TRUE	reported	8KoqAI
3	85574282	G	A	0.02055	0.003125	4.80E-11	0.626279	462346	NA	rs5982572	NA	UKB-b-57	TRUE	reported	8KoqAI
3	85574420	G	A	0.020553	0.003124	4.80E-11	0.62623	462346	NA	rs6225246	NA	UKB-b-57	TRUE	reported	8KoqAI
3	85574559	G	A	0.020598	0.003124	4.30E-11	0.626269	462346	NA	rs6225246	NA	UKB-b-57	TRUE	reported	8KoqAI
3	85574694	A	G	0.020609	0.003124	4.20E-11	0.626201	462346	NA	rs6225246	NA	UKB-b-57	TRUE	reported	8KoqAI
3	85574699	C	T	0.02059	0.003124	4.40E-11	0.626266	462346	NA	rs6225246	NA	UKB-b-57	TRUE	reported	8KoqAI
3	85574742	A	G	0.02059	0.003124	4.40E-11	0.626266	462346	NA	rs6225246	NA	UKB-b-57	TRUE	reported	8KoqAI
3	85574780	G	C	0.02059	0.003124	4.40E-11	0.626266	462346	NA	rs6225246	NA	UKB-b-57	TRUE	reported	8KoqAI
3	85575365	C	T	0.020524	0.003126	5.20E-11	0.626217	462346	NA	rs1470635	NA	UKB-b-57	TRUE	reported	8KoqAI
3	85575441	A	C	0.020583	0.003124	4.40E-11	0.626266	462346	NA	rs1470636	NA	UKB-b-57	TRUE	reported	8KoqAI
3	85575519	T	C	0.020517	0.003126	5.30E-11	0.626208	462346	NA	rs7427346	NA	UKB-b-57	TRUE	reported	8KoqAI
3	85575690	T	G	0.020583	0.003124	4.40E-11	0.626266	462346	NA	rs3573854	NA	UKB-b-57	TRUE	reported	8KoqAI
3	85575723	C	A	0.020583	0.003124	4.50E-11	0.626266	462346	NA	rs1745626	NA	UKB-b-57	TRUE	reported	8KoqAI
3	85575775	T	C	0.020424	0.003126	6.40E-11	0.625795	462346	NA	rs3570142	NA	UKB-b-57	TRUE	reported	8KoqAI
3	85575797	T	C	0.020657	0.003164	6.60E-11	0.646957	462346	NA	rs1249172	NA	UKB-b-57	TRUE	reported	8KoqAI
3	85575844	A	G	0.020517	0.003126	5.30E-11	0.626217	462346	NA	rs5996723	NA	UKB-b-57	TRUE	reported	8KoqAI
3	85575986	A	G	0.020589	0.003124	4.40E-11	0.626267	462346	NA	rs5922586	NA	UKB-b-57	TRUE	reported	8KoqAI
3	85576091	T	C	0.020588	0.003124	4.40E-11	0.626266	462346	NA	rs6042779	NA	UKB-b-57	TRUE	reported	8KoqAI
3	85576104	C	T	0.020589	0.003124	4.40E-11	0.626267	462346	NA	rs5677891	NA	UKB-b-57	TRUE	reported	8KoqAI
3	85576458	A	C	0.020612	0.003124	4.20E-11	0.626244	462346	NA	rs6650510	NA	UKB-b-57	TRUE	reported	8KoqAI
3	85576706	T	C	-0.02513	0.003612	3.40E-12	0.226809	462346	NA	rs9851444	NA	UKB-b-57	TRUE	reported	8KoqAI
3	85577006	G	A	0.020603	0.003124	4.30E-11	0.626269	462346	NA	rs5677787	NA	UKB-b-57	TRUE	reported	8KoqAI
3	85577166	G	A	0.020571	0.003126	4.70E-11	0.626226	462346	NA	rs4856581	NA	UKB-b-57	TRUE	reported	8KoqAI
3	85577269	A	G	0.0206	0.003124	4.30E-11	0.626265	462346	NA	rs4856583	NA	UKB-b-57	TRUE	reported	8KoqAI
3	85577537	A	G	0.020588	0.003126	4.50E-11	0.626227	462346	NA	rs1449389	NA	UKB-b-57	TRUE	reported	8KoqAI
3	85577691	C	G	0.02062	0.003124	4.10E-11	0.626261	462346	NA	rs5767124	NA	UKB-b-57	TRUE	reported	8KoqAI
3	85577703	G	A	0.020651	0.003125	3.90E-11	0.626071	462346	NA	rs1449390	NA	UKB-b-57	TRUE	reported	8KoqAI
3	85578035	G	A	0.020659	0.003165	6.70E-11	0.646918	462346	NA	rs1051107	NA	UKB-b-57	TRUE	reported	8KoqAI
3	85578077	G	A	0.020585	0.003126	4.50E-11	0.626226	462346	NA	rs5593974	NA	UKB-b-57	TRUE	reported	8KoqAI
3	85578129	G	A	0.020606	0.003124	4.20E-11	0.626295	462346	NA	rs6225249	NA	UKB-b-57	TRUE	reported	8KoqAI
3	85578180	G	A	0.020593	0.003126	4.40E-11	0.626227	462346	NA	rs6225249	NA	UKB-b-57	TRUE	reported	8KoqAI
3	85578463	C	T	0.0206	0.003124	4.30E-11	0.626297	462346	NA	rs6225249	NA	UKB-b-57	TRUE	reported	8KoqAI
3	85578725	C	A	0.020627	0.003124	4.00E-11	0.62627	462346	NA	rs5618792	NA	UKB-b-57	TRUE	reported	8KoqAI
3	85578841	C	A	0.020679	0.003165	6.40E-11	0.646925	462346	NA	rs1263365	NA	UKB-b-57	TRUE	reported	8KoqAI
3	85580328	A	T	0.020474	0.003166	1.00E-10	0.647281	462346	NA	rs4856584	NA	UKB-b-57	TRUE	reported	8KoqAI
3	85580588	G	A	0.0206	0.003126	4.40E-11	0.626343	462346	NA	rs1751625	NA	UKB-b-57	TRUE	reported	8KoqAI

3	85581099	G	A	0.020406	0.003126	6.60E-11	0.626777	462346	NA	rs1192352	NA	UKB-b-57	TRUE	reported	8KoqAI
3	85581105	T	A	0.020387	0.003127	7.10E-11	0.626747	462346	NA	rs3560869	NA	UKB-b-57	TRUE	reported	8KoqAI
3	85581231	G	A	0.020638	0.003126	4.00E-11	0.626253	462346	NA	rs7765712	NA	UKB-b-57	TRUE	reported	8KoqAI
3	85581386	G	A	0.020653	0.003124	3.80E-11	0.62628	462346	NA	rs1751628	NA	UKB-b-57	TRUE	reported	8KoqAI
3	85581758	G	C	0.020639	0.003126	4.00E-11	0.626239	462346	NA	rs6225250	NA	UKB-b-57	TRUE	reported	8KoqAI
3	85581779	C	T	0.020638	0.003126	4.00E-11	0.626238	462346	NA	rs6225250	NA	UKB-b-57	TRUE	reported	8KoqAI
3	85581991	A	C	0.020641	0.003124	3.90E-11	0.626294	462346	NA	rs1133512	NA	UKB-b-57	TRUE	reported	8KoqAI
3	85582026	T	C	0.020641	0.003124	3.90E-11	0.626294	462346	NA	rs6225250	NA	UKB-b-57	TRUE	reported	8KoqAI
3	85582078	C	T	0.020657	0.003124	3.80E-11	0.62628	462346	NA	rs6225250	NA	UKB-b-57	TRUE	reported	8KoqAI
3	85582231	G	A	0.020666	0.003166	6.70E-11	0.647224	462346	NA	rs6225250	NA	UKB-b-57	TRUE	reported	8KoqAI
3	85582245	C	T	0.020639	0.003126	4.00E-11	0.62624	462346	NA	rs6225250	NA	UKB-b-57	TRUE	reported	8KoqAI
3	85582643	C	T	0.020628	0.003126	4.10E-11	0.626215	462346	NA	rs1745676	NA	UKB-b-57	TRUE	reported	8KoqAI
3	85582680	A	G	0.02063	0.003124	4.00E-11	0.626271	462346	NA	rs1751634	NA	UKB-b-57	TRUE	reported	8KoqAI
3	85582713	G	A	0.020628	0.003126	4.10E-11	0.626215	462346	NA	rs6701842	NA	UKB-b-57	TRUE	reported	8KoqAI
3	85582939	T	A	0.020627	0.003126	4.10E-11	0.626215	462346	NA	rs1745682	NA	UKB-b-57	TRUE	reported	8KoqAI
3	85583193	A	G	0.020646	0.003124	3.90E-11	0.626256	462346	NA	rs1051107	NA	UKB-b-57	TRUE	reported	8KoqAI
3	85583403	G	A	0.020646	0.003124	3.90E-11	0.626256	462346	NA	rs1051107	NA	UKB-b-57	TRUE	reported	8KoqAI
3	85583471	G	A	0.020628	0.003126	4.10E-11	0.626216	462346	NA	rs5891984	NA	UKB-b-57	TRUE	reported	8KoqAI
3	85583506	C	G	0.020628	0.003126	4.10E-11	0.626216	462346	NA	rs5949187	NA	UKB-b-57	TRUE	reported	8KoqAI
3	85583542	A	G	0.020645	0.003124	3.90E-11	0.626256	462346	NA	rs5977097	NA	UKB-b-57	TRUE	reported	8KoqAI
3	85583608	T	C	0.020473	0.003168	1.00E-10	0.648926	462346	NA	rs1262960	NA	UKB-b-57	TRUE	reported	8KoqAI
3	85583745	A	G	0.020644	0.003124	3.90E-11	0.626262	462346	NA	rs5677921	NA	UKB-b-57	TRUE	reported	8KoqAI
3	85583882	C	G	0.020635	0.003127	4.10E-11	0.626372	462346	NA	rs1751647	NA	UKB-b-57	TRUE	reported	8KoqAI
3	85583927	T	A	0.020643	0.003124	3.90E-11	0.626262	462346	NA	rs5775656	NA	UKB-b-57	TRUE	reported	8KoqAI
3	85583945	T	C	0.020635	0.003127	4.10E-11	0.626372	462346	NA	rs1751650	NA	UKB-b-57	TRUE	reported	8KoqAI
3	85584478	A	T	0.020641	0.003127	4.10E-11	0.626396	462346	NA	rs6225251	NA	UKB-b-57	TRUE	reported	8KoqAI
3	85585091	A	G	0.020653	0.003124	3.80E-11	0.626293	462346	NA	rs6662517	NA	UKB-b-57	TRUE	reported	8KoqAI
3	85585144	G	C	0.020633	0.003125	4.00E-11	0.626356	462346	NA	rs1751658	NA	UKB-b-57	TRUE	reported	8KoqAI
3	85585277	C	T	0.020646	0.003124	3.90E-11	0.626287	462346	NA	rs1745705	NA	UKB-b-57	TRUE	reported	8KoqAI
3	85585340	A	T	0.020646	0.003124	3.90E-11	0.626287	462346	NA	rs6754460	NA	UKB-b-57	TRUE	reported	8KoqAI
3	85585431	G	A	0.020651	0.003126	4.00E-11	0.626331	462346	NA	rs1751668	NA	UKB-b-57	TRUE	reported	8KoqAI
3	85585646	A	G	0.020646	0.003124	3.90E-11	0.626287	462346	NA	rs6678179	NA	UKB-b-57	TRUE	reported	8KoqAI
3	85585860	G	A	0.020647	0.003124	3.90E-11	0.626287	462346	NA	rs1745718	NA	UKB-b-57	TRUE	reported	8KoqAI
3	85586521	A	T	0.020647	0.003124	3.90E-11	0.626287	462346	NA	rs1745721	NA	UKB-b-57	TRUE	reported	8KoqAI
3	85586835	A	T	0.020646	0.003124	3.90E-11	0.626287	462346	NA	rs2167047	NA	UKB-b-57	TRUE	reported	8KoqAI
3	85586921	C	T	0.020682	0.003124	3.60E-11	0.626289	462346	NA	rs2122235	NA	UKB-b-57	TRUE	reported	8KoqAI
3	85587537	C	T	0.020688	0.003124	3.50E-11	0.626286	462346	NA	rs6225251	NA	UKB-b-57	TRUE	reported	8KoqAI
3	85587710	A	C	0.0207	0.003124	3.40E-11	0.626261	462346	NA	rs1751685	NA	UKB-b-57	TRUE	reported	8KoqAI
3	85587729	C	G	0.02052	0.003165	8.90E-11	0.647603	462346	NA	rs1112789	NA	UKB-b-57	TRUE	reported	8KoqAI
3	85588205	T	C	0.020748	0.003126	3.20E-11	0.626425	462346	NA	rs3543871	NA	UKB-b-57	TRUE	reported	8KoqAI
3	85588375	T	G	0.020687	0.003124	3.50E-11	0.626286	462346	NA	rs1745737	NA	UKB-b-57	TRUE	reported	8KoqAI
3	85588494	C	T	0.020643	0.003127	4.10E-11	0.626389	462346	NA	rs6225251	NA	UKB-b-57	TRUE	reported	8KoqAI
3	85588962	A	C	0.020606	0.003127	4.40E-11	0.626406	462346	NA	rs1745742	NA	UKB-b-57	TRUE	reported	8KoqAI
3	85589010	T	C	0.020603	0.003127	4.40E-11	0.626405	462346	NA	rs1745745	NA	UKB-b-57	TRUE	reported	8KoqAI
3	85589022	A	T	0.020679	0.003124	3.60E-11	0.626289	462346	NA	rs6225251	NA	UKB-b-57	TRUE	reported	8KoqAI
3	85589246	T	A	0.020678	0.003124	3.60E-11	0.62629	462346	NA	rs6225251	NA	UKB-b-57	TRUE	reported	8KoqAI
3	85589275	G	A	0.020639	0.003124	3.90E-11	0.626296	462346	NA	rs6766493	NA	UKB-b-57	TRUE	reported	8KoqAI
3	85589580	T	C	0.020576	0.003124	4.50E-11	0.626218	462346	NA	rs6779501	NA	UKB-b-57	TRUE	reported	8KoqAI
3	85589897	A	G	0.020632	0.003124	4.00E-11	0.626291	462346	NA	rs4856271	NA	UKB-b-57	TRUE	reported	8KoqAI
3	85590097	G	A	-0.02509	0.003618	4.00E-12	0.22641	462346	NA	rs9873400	NA	UKB-b-57	TRUE	reported	8KoqAI
3	85590444	A	T	0.020574	0.003127	4.70E-11	0.626412	462346	NA	rs6158660	NA	UKB-b-57	TRUE	reported	8KoqAI
3	85590701	C	G	0.020641	0.003124	3.90E-11	0.626296	462346	NA	rs1751708	NA	UKB-b-57	TRUE	reported	8KoqAI
3	85591108	G	A	0.020574	0.003127	4.70E-11	0.626412	462346	NA	rs1751712	NA	UKB-b-57	TRUE	reported	8KoqAI
3	85591229	A	C	0.020641	0.003124	3.90E-11	0.626296	462346	NA	rs1751714	NA	UKB-b-57	TRUE	reported	8KoqAI
3	85591363	C	T	0.020574	0.003127	4.70E-11	0.626413	462346	NA	rs1745764	NA	UKB-b-57	TRUE	reported	8KoqAI
3	85591451	T	C	0.020574	0.003127	4.70E-11	0.626412	462346	NA	rs1745766	NA	UKB-b-57	TRUE	reported	8KoqAI
3	85591586	G	C	0.020641	0.003124	3.90E-11	0.626296	462346	NA	rs1751723	NA	UKB-b-57	TRUE	reported	8KoqAI
3	85591785	A	T	0.020641	0.003124	3.90E-11	0.626296	462346	NA	rs6692268	NA	UKB-b-57	TRUE	reported	8KoqAI
3	85591981	C	T	0.020574	0.003127	4.70E-11	0.626412	462346	NA	rs7620313	NA	UKB-b-57	TRUE	reported	8KoqAI
3	85592342	C	A	0.020659	0.003124	3.80E-11	0.626246	462346	NA	rs1900918	NA	UKB-b-57	TRUE	reported	8KoqAI
3	85592750	T	A	0.020641	0.003124	3.90E-11	0.626296	462346	NA	rs6790090	NA	UKB-b-57	TRUE	reported	8KoqAI
3	85592983	G	T	0.020637	0.003124	4.00E-11	0.626318	462346	NA	rs6777456	NA	UKB-b-57	TRUE	reported	8KoqAI
3	85592988	G	A	0.020584	0.003127	4.60E-11	0.626435	462346	NA	rs6777458	NA	UKB-b-57	TRUE	reported	8KoqAI
3	85593146	A	C	0.020589	0.003127	4.60E-11	0.626411	462346	NA	rs1751727	NA	UKB-b-57	TRUE	reported	8KoqAI
3	85593584	C	T	0.021219	0.003122	1.10E-11	0.620989	462346	NA	rs6780346	NA	UKB-b-57	TRUE	reported	8KoqAI
3	85593673	A	G	0.020653	0.003125	3.90E-11	0.626276	462346	NA	rs6804626	NA	UKB-b-57	TRUE	reported	8KoqAI
3	85594169	C	T	-0.02518	0.003614	3.20E-12	0.226511	462346	NA	rs7628948	NA	UKB-b-57	TRUE	reported	8KoqAI
3	85594359	C	G	-0.02513	0.003618	3.70E-12	0.226394	462346	NA	rs1449404	NA	UKB-b-57	TRUE	reported	8KoqAI
3	85594428	A	C	0.020625	0.003167	7.40E-11	0.64736	462346	NA	rs6807666	NA	UKB-b-57	TRUE	reported	8KoqAI
3	85594562	A	G	0.0206	0.003127	4.50E-11	0.626408	462346	NA	rs1112789	NA	UKB-b-57	TRUE	reported	8KoqAI
3	85594599	A	G	0.020597	0.003127	4.50E-11	0.626411	462346	NA	rs1112790	NA	UKB-b-57	TRUE	reported	8KoqAI
3	85594695	A	G	0.020558	0.003127	4.90E-11	0.626533	462346	NA	rs1086561	NA	UKB-b-57	TRUE	reported	8KoqAI
3	85594846	A	C	0.020623	0.003124	4.10E-11	0.626337	462346	NA	rs1551045	NA	UKB-b-57	TRUE	reported	8KoqAI
3	85594864	A	T	0.020598	0.003127	4.50E-11	0.626411	462346	NA	rs1463203	NA	UKB-b-57	TRUE	reported	8KoqAI
3	85595133	T	C	0.020598	0.003127	4.50E-11	0.626411	462346	NA	rs1463204	NA	UKB-b-57	TRUE	reported	8KoqAI
3	85595397	G	C	0.020598	0.003127	4.50E-11	0.626411	462346	NA	rs1551046	NA	UKB-b-57	TRUE	reported	8KoqAI
3	85595726	A	T	-0.02513	0.003618	3.80E-12	0.226397	462346	NA	rs1309243	NA	UKB-b-57	TRUE	reported	8KoqAI
3	85595857	G	T	0.020625	0.003124	4.10E-11	0.626337	462346	NA	rs1551047	NA	UKB-b-57	TRUE	reported	8KoqAI
3	85595945	G	T	0.020625	0.003124	4.10E-11	0.626337	462346	NA	rs1551048	NA	UKB-b-57	TRUE	reported	8KoqAI
3	85596219	A	G	0.020598	0.003127	4.50E-11	0.626412	462346	NA	rs6765881	NA	UKB-b-57	TRUE	reported	8KoqAI
3	85596323	A	G	0.020598	0.003127	4.50E-11	0.626412	462346	NA	rs6765991	NA	UKB-b-57	TRUE	reported	8KoqAI
3	85596432	G	A	0.020626	0.003124	4.10E-11	0.626337	462346	NA	rs1463205	NA	UKB-b-57	TRUE	reported	8KoqAI
3	85596479	C	T	0.020598	0.003127	4.50E-11	0.626412	462346	NA	rs4502590	NA	UKB-b-57	TRUE	reported	8KoqAI
3	85596597	T	A	0.020625	0.003124	4.10E-11	0.626337	462346	NA	rs1463206	NA	UKB-b-57	TRUE	reported	8KoqAI
3	85596665	A	C	-0.02511	0.003618	3.90E-12	0.226398	462346	NA	rs1171291	NA	UKB-b-57	TRUE	reported	8KoqAI
3	85596704	C	T	0.020625	0.003124	4.10E-11	0.626337	462346	NA	rs1463207	NA	UKB-b-57	TRUE	reported	8KoqAI
3	85596712	C	A	0.020616	0.003124	4.10E-11	0.626334	462346	NA	rs1463208	NA	UKB-b-57	TRUE	reported	8KoqAI
3	85596855	T	C	0.020616	0.003124	4.10E-11	0.626333	462346	NA	rs4603966	NA	UKB-b-57	TRUE	reported	8KoqAI
3	85597009	C	T	0.020616	0.003124	4.10E-11	0.626333	462346	NA	rs6225396	NA	UKB-b-57	TRUE	reported	8KoqAI
3	85597014	G	A	0.020622	0.003124	4.10E-11	0.62634	462346	NA	rs6225396	NA	UKB-b-57	TRUE	reported	8KoqAI
3	85597135	G	A	0.020668	0.003164	6.50E-11	0.647308	462346	NA	rs2326319	NA	UKB-b-57	TRUE	reported	8KoqAI
3	85598244	T	A	0.020614	0.003124	4.20E-11	0.626334	462346	NA	rs4856585	NA	UKB-b-57	TRUE	reported	8KoqAI
3	85598260	G	A	0.020614	0.003124	4.20E-11	0.626334	462346	NA	rs4856273	NA	UKB-b-57	TRUE	reported	8KoqAI
3	85598424	T	C	-0.02511	0.003617	3.90E-12	0.226434	462346	NA	rs1271462	NA	UKB-b-57	TRUE	reported	8KoqAI
3	85598481	T	C	0.020593	0.003127	4.50E-11	0.626371	462346	NA	rs6792295	NA	UKB-b-57	TRUE	reported	8KoqAI
3	85599014	A	T	0.020622	0.003124	4.10E-11	0.62634	462346	NA	rs6549033	NA	UKB-b-57	TRUE	reported	8KoqAI
3	85599067	T	C	0.02067	0.003164	6.50E-11	0.647305	462346	NA	rs6549035	NA	UKB-b-57	TRUE	reported	8KoqAI
3	85599325	G	A	0.020595	0.003127	4.50E-11	0.626373	462346	NA	rs6225396	NA	UKB-b-57	TRUE	reported	8KoqAI
3	85599391	G	T	0.020588	0.003127	4.60E-11	0.626362	462346	NA	rs4301022	NA	UKB-b-57	TRUE	reported	8KoqAI
3	85599588	G	A	0.020643	0.003167	7.10E-11	0.647336	462346	NA	rs6225396	NA	UKB-b-57	TRUE	reported	8KoqAI
3	85599601	T	C	0.020643	0.003167	7.10E-11	0.647336	462346	NA	rs6225396	NA	UKB-b-57	TRUE	reported	8KoqAI
3	85599711	G	A	0.020667	0.003124	3.70E-11	0.626187	462346	NA	rs1745850	NA	UKB-b-57	TRUE	reported	8KoqAI
3	85599792	G	A	0.020608	0.003125	4.30E-11	0.626129	462346	NA	rs6225396	NA	UKB-b-57	TRUE	reported	8KoqAI
3	85599825	C	T	0.020618	0.003125	4.20E-11	0.626121	462346	NA	rs1751808	NA	UKB-b-57	TRUE	reported	8KoqAI
3	85599868	T	G	0.020608	0.003125	4.30E-11	0.626048	462346	NA	rs1745857	NA	UKB-b-57	TRUE	reported	8KoqAI
3	85599918	C	T	0.020705	0.003125	3.50E-11	0.625871	462346	NA	rs6783137	NA	UKB-b-57	TRUE	reported	8KoqAI
3	85599920	C	G	0.020699	0.003125	3.50E-11	0.625841	462346	NA	rs6783138	NA	UKB-b-57	TRUE	reported	8KoqAI

3	85599977	C	T	0.020572	0.003128	4.80E-11	0.626107	462346	NA	rs1745860	NA	UKB-b-57	TRUE	reported	8KoqAI
3	85600077	T	C	0.020603	0.003124	4.30E-11	0.626304	462346	NA	rs1449405	NA	UKB-b-57	TRUE	reported	8KoqAI
3	85600212	A	C	0.02055	0.003127	5.00E-11	0.626392	462346	NA	rs1449406	NA	UKB-b-57	TRUE	reported	8KoqAI
3	85600384	A	G	0.020604	0.003127	4.40E-11	0.626335	462346	NA	rs1449407	NA	UKB-b-57	TRUE	reported	8KoqAI
3	85600590	G	T	0.02057	0.003127	4.70E-11	0.626408	462346	NA	rs6549036	NA	UKB-b-57	TRUE	reported	8KoqAI
3	85600708	G	A	0.020574	0.003127	4.70E-11	0.626399	462346	NA	rs6549037	NA	UKB-b-57	TRUE	reported	8KoqAI
3	85600771	G	C	0.020569	0.003127	4.80E-11	0.626403	462346	NA	rs6225397	NA	UKB-b-57	TRUE	reported	8KoqAI
3	85600794	A	G	0.020594	0.003124	4.30E-11	0.626295	462346	NA	rs6225397	NA	UKB-b-57	TRUE	reported	8KoqAI
3	85600940	G	C	0.020569	0.003127	4.80E-11	0.6264	462346	NA	rs1449408	NA	UKB-b-57	TRUE	reported	8KoqAI
3	85601440	T	A	0.020588	0.003164	7.70E-11	0.647334	462346	NA	rs1249275	NA	UKB-b-57	TRUE	reported	8KoqAI
3	85602746	C	A	0.020567	0.003129	4.90E-11	0.626354	462346	NA	rs2029132	NA	UKB-b-57	TRUE	reported	8KoqAI
3	85602808	G	A	0.020567	0.003129	4.90E-11	0.626354	462346	NA	rs2029133	NA	UKB-b-57	TRUE	reported	8KoqAI
3	85603219	C	G	0.020567	0.003129	4.90E-11	0.626354	462346	NA	rs1449410	NA	UKB-b-57	TRUE	reported	8KoqAI
3	85603262	T	G	0.020625	0.003169	7.60E-11	0.647334	462346	NA	rs2029134	NA	UKB-b-57	TRUE	reported	8KoqAI
3	85603309	A	G	0.020513	0.003165	9.10E-11	0.64761	462346	NA	rs7618494	NA	UKB-b-57	TRUE	reported	8KoqAI
3	85603371	G	A	0.020627	0.003169	7.60E-11	0.647331	462346	NA	rs7640828	NA	UKB-b-57	TRUE	reported	8KoqAI
3	85603462	T	C	0.020505	0.003168	9.60E-11	0.64896	462346	NA	rs7652683	NA	UKB-b-57	TRUE	reported	8KoqAI
3	85603643	T	G	0.020486	0.003168	1.00E-10	0.648945	462346	NA	rs7652808	NA	UKB-b-57	TRUE	reported	8KoqAI
3	85603872	T	G	0.020619	0.003125	4.10E-11	0.626296	462346	NA	rs6225046	NA	UKB-b-57	TRUE	reported	8KoqAI
3	85604158	A	G	0.020585	0.003125	4.50E-11	0.626321	462346	NA	rs1375544	NA	UKB-b-57	TRUE	reported	8KoqAI
3	85604320	G	T	0.02041	0.003132	7.20E-11	0.626523	462346	NA	rs1375545	NA	UKB-b-57	TRUE	reported	8KoqAI
3	85604405	G	A	0.020491	0.003126	5.60E-11	0.626485	462346	NA	rs1375546	NA	UKB-b-57	TRUE	reported	8KoqAI
3	85604425	C	T	-0.02533	0.003617	2.50E-12	0.22647	462346	NA	rs1171390	NA	UKB-b-57	TRUE	reported	8KoqAI
3	85604765	G	A	0.020506	0.003126	5.40E-11	0.62645	462346	NA	rs7261572	NA	UKB-b-57	TRUE	reported	8KoqAI
3	85604923	C	T	0.020399	0.003131	7.30E-11	0.62647	462346	NA	rs1751858	NA	UKB-b-57	TRUE	reported	8KoqAI
3	85604963	T	A	0.020588	0.003165	7.80E-11	0.647275	462346	NA	rs6758446	NA	UKB-b-57	TRUE	reported	8KoqAI
3	85605046	G	A	0.020397	0.003131	7.30E-11	0.626466	462346	NA	rs6663101	NA	UKB-b-57	TRUE	reported	8KoqAI
3	85605061	C	T	0.020397	0.003131	7.30E-11	0.626466	462346	NA	rs6804927	NA	UKB-b-57	TRUE	reported	8KoqAI
3	85605180	T	C	0.020563	0.003126	4.80E-11	0.626146	462346	NA	rs7313379	NA	UKB-b-57	TRUE	reported	8KoqAI
3	85605183	C	T	0.020463	0.003132	6.40E-11	0.6263	462346	NA	rs7313380	NA	UKB-b-57	TRUE	reported	8KoqAI
3	85605206	G	T	0.02055	0.003126	4.90E-11	0.626191	462346	NA	rs6756800	NA	UKB-b-57	TRUE	reported	8KoqAI
3	85605437	C	T	0.020405	0.003131	7.20E-11	0.62649	462346	NA	rs6225046	NA	UKB-b-57	TRUE	reported	8KoqAI
3	85606003	A	G	0.020534	0.003125	5.00E-11	0.62633	462346	NA	rs6549039	NA	UKB-b-57	TRUE	reported	8KoqAI
3	85606061	G	T	0.020416	0.003131	7.00E-11	0.626457	462346	NA	rs6549040	NA	UKB-b-57	TRUE	reported	8KoqAI
3	85606462	C	G	0.020495	0.003171	1.00E-10	0.647414	462346	NA	rs4856274	NA	UKB-b-57	TRUE	reported	8KoqAI
3	85606747	A	G	0.020534	0.003125	5.00E-11	0.626312	462346	NA	rs4856277	NA	UKB-b-57	TRUE	reported	8KoqAI
3	85606962	C	A	0.020412	0.003131	7.10E-11	0.626438	462346	NA	rs6727727	NA	UKB-b-57	TRUE	reported	8KoqAI
3	85607003	T	C	0.020412	0.003131	7.10E-11	0.626438	462346	NA	rs4543024	NA	UKB-b-57	TRUE	reported	8KoqAI
3	85607010	A	G	0.020531	0.003125	5.00E-11	0.626313	462346	NA	rs6802850	NA	UKB-b-57	TRUE	reported	8KoqAI
3	85607142	A	G	0.020531	0.003125	5.00E-11	0.626313	462346	NA	rs4441668	NA	UKB-b-57	TRUE	reported	8KoqAI
3	85607161	C	T	0.020412	0.003131	7.10E-11	0.626438	462346	NA	rs1449370	NA	UKB-b-57	TRUE	reported	8KoqAI
3	85607285	A	G	0.020413	0.003131	7.00E-11	0.626457	462346	NA	rs6649908	NA	UKB-b-57	TRUE	reported	8KoqAI
3	85607323	T	C	0.020413	0.003131	7.00E-11	0.626457	462346	NA	rs1449371	NA	UKB-b-57	TRUE	reported	8KoqAI
3	85607393	G	A	0.020545	0.003125	4.90E-11	0.626329	462346	NA	rs6654451	NA	UKB-b-57	TRUE	reported	8KoqAI
3	85607605	G	T	0.020417	0.003131	7.00E-11	0.626457	462346	NA	rs7653790	NA	UKB-b-57	TRUE	reported	8KoqAI
3	85608137	G	A	-0.02518	0.00369	8.90E-12	0.217188	462346	NA	rs1375547	NA	UKB-b-57	TRUE	reported	8KoqAI
3	85608139	A	C	0.020419	0.003131	6.90E-11	0.62646	462346	NA	rs724304	NA	UKB-b-57	TRUE	reported	8KoqAI
3	85608199	T	G	0.020539	0.003125	5.00E-11	0.626326	462346	NA	rs1449372	NA	UKB-b-57	TRUE	reported	8KoqAI
3	85608461	G	A	0.020417	0.003131	7.00E-11	0.626457	462346	NA	rs1449373	NA	UKB-b-57	TRUE	reported	8KoqAI
3	85608487	C	T	0.020417	0.003131	7.00E-11	0.626457	462346	NA	rs1449374	NA	UKB-b-57	TRUE	reported	8KoqAI
3	85608568	G	T	0.020545	0.003125	4.90E-11	0.626329	462346	NA	rs1449375	NA	UKB-b-57	TRUE	reported	8KoqAI
3	85609072	T	C	0.020407	0.003131	7.10E-11	0.626424	462346	NA	rs2167043	NA	UKB-b-57	TRUE	reported	8KoqAI
3	85609095	C	T	0.020406	0.003131	7.10E-11	0.626427	462346	NA	rs2167044	NA	UKB-b-57	TRUE	reported	8KoqAI
3	85609289	A	G	0.020407	0.003131	7.10E-11	0.626426	462346	NA	rs5907310	NA	UKB-b-57	TRUE	reported	8KoqAI
3	85609303	T	C	0.020407	0.003131	7.10E-11	0.626426	462346	NA	rs6225046	NA	UKB-b-57	TRUE	reported	8KoqAI
3	85609365	G	A	0.020412	0.003131	7.00E-11	0.62643	462346	NA	rs1745956	NA	UKB-b-57	TRUE	reported	8KoqAI
3	85609795	C	T	0.020402	0.003131	7.20E-11	0.626465	462346	NA	rs1449378	NA	UKB-b-57	TRUE	reported	8KoqAI
3	85609879	T	C	0.020522	0.003125	5.10E-11	0.626304	462346	NA	rs1449379	NA	UKB-b-57	TRUE	reported	8KoqAI
3	85610083	A	G	0.020417	0.003131	7.00E-11	0.626457	462346	NA	rs1530738	NA	UKB-b-57	TRUE	reported	8KoqAI
3	85610114	T	A	0.020527	0.003125	5.10E-11	0.626331	462346	NA	rs1530739	NA	UKB-b-57	TRUE	reported	8KoqAI
3	85610487	G	A	0.020418	0.003131	7.00E-11	0.626456	462346	NA	rs1530740	NA	UKB-b-57	TRUE	reported	8KoqAI
3	85610649	C	T	0.020416	0.003131	7.00E-11	0.626457	462346	NA	rs1751924	NA	UKB-b-57	TRUE	reported	8KoqAI
3	85610695	C	T	0.020526	0.003125	5.10E-11	0.626331	462346	NA	rs6225046	NA	UKB-b-57	TRUE	reported	8KoqAI
3	85610725	A	G	0.020524	0.003125	5.10E-11	0.626303	462346	NA	rs1751926	NA	UKB-b-57	TRUE	reported	8KoqAI
3	85610855	A	G	0.020495	0.003171	1.00E-10	0.64739	462346	NA	rs6054136	NA	UKB-b-57	TRUE	reported	8KoqAI
3	85610976	G	A	0.020528	0.003125	5.10E-11	0.62633	462346	NA	rs2167045	NA	UKB-b-57	TRUE	reported	8KoqAI
3	85611068	G	A	0.020418	0.003131	7.00E-11	0.626456	462346	NA	rs2122233	NA	UKB-b-57	TRUE	reported	8KoqAI
3	85611139	G	A	0.020528	0.003125	5.10E-11	0.62633	462346	NA	rs2122234	NA	UKB-b-57	TRUE	reported	8KoqAI
3	85611420	G	A	0.020418	0.003131	7.00E-11	0.626456	462346	NA	rs4856588	NA	UKB-b-57	TRUE	reported	8KoqAI
3	85611560	A	G	0.020418	0.003131	7.00E-11	0.626456	462346	NA	rs4856589	NA	UKB-b-57	TRUE	reported	8KoqAI
3	85611791	G	A	0.020418	0.003131	7.00E-11	0.626456	462346	NA	rs1900914	NA	UKB-b-57	TRUE	reported	8KoqAI
3	85611903	C	T	0.020418	0.003131	7.00E-11	0.626456	462346	NA	rs1449380	NA	UKB-b-57	TRUE	reported	8KoqAI
3	85612042	A	G	0.020563	0.003125	4.70E-11	0.626345	462346	NA	rs1449381	NA	UKB-b-57	TRUE	reported	8KoqAI
3	85612053	G	A	0.020433	0.003131	6.70E-11	0.626468	462346	NA	rs1449382	NA	UKB-b-57	TRUE	reported	8KoqAI
3	85612528	G	A	0.020428	0.003131	6.80E-11	0.626431	462346	NA	rs4856590	NA	UKB-b-57	TRUE	reported	8KoqAI
3	85612700	T	C	0.020588	0.003125	4.50E-11	0.626335	462346	NA	rs1745990	NA	UKB-b-57	TRUE	reported	8KoqAI
3	85612748	C	A	0.020433	0.003131	6.70E-11	0.626468	462346	NA	rs6225047	NA	UKB-b-57	TRUE	reported	8KoqAI
3	85612767	T	C	0.020433	0.003131	6.70E-11	0.626468	462346	NA	rs4856593	NA	UKB-b-57	TRUE	reported	8KoqAI
3	85613422	G	C	0.020563	0.003125	4.70E-11	0.626345	462346	NA	rs1900915	NA	UKB-b-57	TRUE	reported	8KoqAI
3	85613473	C	T	0.020433	0.003131	6.70E-11	0.626468	462346	NA	rs2326320	NA	UKB-b-57	TRUE	reported	8KoqAI
3	85613501	T	C	0.020557	0.003125	4.80E-11	0.626333	462346	NA	rs1375551	NA	UKB-b-57	TRUE	reported	8KoqAI
3	85613831	A	G	0.020433	0.003131	6.70E-11	0.626468	462346	NA	rs1449383	NA	UKB-b-57	TRUE	reported	8KoqAI
3	85613855	T	A	0.020563	0.003125	4.70E-11	0.626345	462346	NA	rs1449384	NA	UKB-b-57	TRUE	reported	8KoqAI
3	85613991	T	C	0.020566	0.003125	4.70E-11	0.626344	462346	NA	rs1449385	NA	UKB-b-57	TRUE	reported	8KoqAI
3	85614034	C	T	0.020649	0.003165	6.80E-11	0.647305	462346	NA	rs5603142	NA	UKB-b-57	TRUE	reported	8KoqAI
3	85614272	A	G	0.020517	0.003171	9.80E-11	0.647426	462346	NA	rs1702301	NA	UKB-b-57	TRUE	reported	8KoqAI
3	85614670	T	A	0.020437	0.003131	6.70E-11	0.626466	462346	NA	rs6799924	NA	UKB-b-57	TRUE	reported	8KoqAI
3	85614746	C	A	0.020578	0.003125	4.60E-11	0.626339	462346	NA	rs6225048	NA	UKB-b-57	TRUE	reported	8KoqAI
3	85614934	C	T	0.020578	0.003125	4.60E-11	0.626339	462346	NA	rs6225049	NA	UKB-b-57	TRUE	reported	8KoqAI
3	85615144	A	G	-0.02521	0.003626	3.50E-12	0.226374	462346	NA	rs9810724	NA	UKB-b-57	TRUE	reported	8KoqAI
3	85615183	C	T	0.020564	0.003125	4.70E-11	0.626345	462346	NA	rs7617306	NA	UKB-b-57	TRUE	reported	8KoqAI
3	85615298	T	G	0.020437	0.003131	6.70E-11	0.626466	462346	NA	rs7629091	NA	UKB-b-57	TRUE	reported	8KoqAI
3	85615981	C	G	0.020706	0.003125	3.50E-11	0.62617	462346	NA	rs1051108	NA	UKB-b-57	TRUE	reported	8KoqAI
3	85616009	G	T	0.020577	0.003131	4.90E-11	0.626292	462346	NA	rs6225049	NA	UKB-b-57	TRUE	reported	8KoqAI
3	85616014	T	C	0.020711	0.003125	3.40E-11	0.6262	462346	NA	rs6225049	NA	UKB-b-57	TRUE	reported	8KoqAI
3	85616118	A	C	0.020683	0.003125	3.60E-11	0.626186	462346	NA	rs1751994	NA	UKB-b-57	TRUE	reported	8KoqAI
3	85616260	A	C	-0.02521	0.003626	3.60E-12	0.226377	462346	NA	rs1307766	NA	UKB-b-57	TRUE	reported	8KoqAI
3	85616809	C	T	0.020556	0.003125	4.80E-11	0.626353	462346	NA	rs1597315	NA	UKB-b-57	TRUE	reported	8KoqAI
3	85617287	A	T	0.020556	0.003125	4.80E-11	0.626353	462346	NA	rs6801823	NA	UKB-b-57	TRUE	reported	8KoqAI
3	85618112	G	T	0.020483	0.00313	6.00E-11	0.625034	462346	NA	rs1746054	NA	UKB-b-57	TRUE	reported	8KoqAI
3	85618308	T	C	0.020556	0.003125	4.80E-11	0.626353	462346	NA	rs1375555	NA	UKB-b-57	TRUE	reported	8KoqAI
3	85618419	A	G	0.020557	0.003125	4.80E-11	0.626354	462346	NA	rs1375554	NA	UKB-b-57	TRUE	reported	8KoqAI
3	85618447	G	T	-0.02536	0.003617	2.30E-12	0.226501	462346	NA	rs1375552	NA	UKB-b-57	TRUE	reported	8KoqAI
3	85619079	T	G	0.020323	0.003135	9.00E-11	0.6282	462346	NA	rs1449386	NA	UKB-b-57	TRUE	reported	8KoqAI
3	85619644	T	C	0.020432	0.003131	6.80E-11	0.626474	462346	NA	rs5921108	NA	UKB-b-57	TRUE	reported	8KoqAI
3	85619783	A	G	0.020541	0.003134	5.60E-11	0.627822	462346	NA	rs1263956	NA	UKB-b-57	TRUE	reported	8KoqAI
3	85619857	C	T	0.020556	0.003125	4.80E-11	0.626353	462346	NA	rs6131659	NA	UKB-b-57	TRUE	reported	8KoqAI

3	85620481	T	G	0.020554	0.003125	4.80E-11	0.626354	462346	NA	rs5941725	NA	UKB-b-57	TRUE	reported	8KoqAI
3	85620621	G	T	0.020429	0.003131	6.80E-11	0.626475	462346	NA	rs6225049	NA	UKB-b-57	TRUE	reported	8KoqAI
3	85621010	A	G	0.02054	0.003125	5.00E-11	0.626343	462346	NA	rs5727624	NA	UKB-b-57	TRUE	reported	8KoqAI
3	85621205	A	G	0.020414	0.003131	7.00E-11	0.626463	462346	NA	rs5983502	NA	UKB-b-57	TRUE	reported	8KoqAI
3	85621395	C	T	-0.02522	0.003626	3.50E-12	0.226374	462346	NA	rs1335347	NA	UKB-b-57	TRUE	reported	8KoqAI
3	85621931	T	C	0.020539	0.003125	5.00E-11	0.626347	462346	NA	rs7616458	NA	UKB-b-57	TRUE	reported	8KoqAI
3	85622242	C	A	0.020613	0.003128	4.40E-11	0.627824	462346	NA	rs1702303	NA	UKB-b-57	TRUE	reported	8KoqAI
3	85622663	G	A	0.020652	0.003128	4.00E-11	0.627688	462346	NA	rs1249444	NA	UKB-b-57	TRUE	reported	8KoqAI
3	85622699	T	A	0.020616	0.003131	4.50E-11	0.626279	462346	NA	rs5888949	NA	UKB-b-57	TRUE	reported	8KoqAI
3	85623271	C	T	0.020532	0.003125	5.00E-11	0.626331	462346	NA	rs1746070	NA	UKB-b-57	TRUE	reported	8KoqAI
3	85624131	A	T	0.020597	0.003126	4.40E-11	0.626632	462346	NA	rs6788098	NA	UKB-b-57	TRUE	reported	8KoqAI
3	85624189	A	G	0.020687	0.003127	3.70E-11	0.62681	462346	NA	rs6790699	NA	UKB-b-57	TRUE	reported	8KoqAI
3	85624386	G	T	0.020499	0.003131	5.80E-11	0.626242	462346	NA	rs4856596	NA	UKB-b-57	TRUE	reported	8KoqAI
3	85624561	C	T	0.020549	0.003125	4.90E-11	0.626147	462346	NA	rs6766690	NA	UKB-b-57	TRUE	reported	8KoqAI
3	85624773	C	G	0.02062	0.003125	4.20E-11	0.626106	462346	NA	rs1263804	NA	UKB-b-57	TRUE	reported	8KoqAI
3	85625231	A	G	0.020483	0.003131	6.00E-11	0.626214	462346	NA	rs6790415	NA	UKB-b-57	TRUE	reported	8KoqAI
3	85625852	G	T	0.020743	0.003128	3.30E-11	0.627441	462346	NA	rs7617323	NA	UKB-b-57	TRUE	reported	8KoqAI
3	85626054	C	T	0.020623	0.003125	4.10E-11	0.626097	462346	NA	rs7617356	NA	UKB-b-57	TRUE	reported	8KoqAI
3	85627691	C	T	0.020489	0.003131	6.00E-11	0.626208	462346	NA	rs6225050	NA	UKB-b-57	TRUE	reported	8KoqAI
3	85628688	G	A	0.020495	0.003131	5.90E-11	0.626207	462346	NA	rs6762535	NA	UKB-b-57	TRUE	reported	8KoqAI
3	85628865	G	A	0.020489	0.003131	6.00E-11	0.62621	462346	NA	rs6762733	NA	UKB-b-57	TRUE	reported	8KoqAI
3	85629183	C	G	0.020484	0.003131	6.00E-11	0.626208	462346	NA	rs6549041	NA	UKB-b-57	TRUE	reported	8KoqAI
3	85629255	A	G	0.020484	0.003131	6.00E-11	0.626208	462346	NA	rs6549042	NA	UKB-b-57	TRUE	reported	8KoqAI
3	85630551	A	C	0.020771	0.003165	5.30E-11	0.646981	462346	NA	rs1551042	NA	UKB-b-57	TRUE	reported	8KoqAI
3	85631142	A	T	0.020374	0.003174	1.40E-10	0.648872	462346	NA	rs1972994	NA	UKB-b-57	TRUE	reported	8KoqAI
3	85631845	G	C	0.020368	0.003131	7.70E-11	0.626071	462346	NA	rs6772956	NA	UKB-b-57	TRUE	reported	8KoqAI
3	85632126	A	T	0.020531	0.003125	5.10E-11	0.626148	462346	NA	rs6549043	NA	UKB-b-57	TRUE	reported	8KoqAI
3	85632432	A	G	0.020526	0.003125	5.10E-11	0.626143	462346	NA	rs6549044	NA	UKB-b-57	TRUE	reported	8KoqAI
3	85633021	A	C	-0.02522	0.003623	3.40E-12	0.22694	462346	NA	rs3534446	NA	UKB-b-57	TRUE	reported	8KoqAI
3	85634216	C	G	0.020638	0.003128	4.20E-11	0.627487	462346	NA	rs6225050	NA	UKB-b-57	TRUE	reported	8KoqAI
3	85634410	T	A	0.020527	0.003125	5.10E-11	0.626142	462346	NA	rs6225050	NA	UKB-b-57	TRUE	reported	8KoqAI
3	85635126	G	T	0.020408	0.003131	7.10E-11	0.626244	462346	NA	rs1375557	NA	UKB-b-57	TRUE	reported	8KoqAI
3	85635190	A	G	0.020399	0.003131	7.30E-11	0.626252	462346	NA	rs1375558	NA	UKB-b-57	TRUE	reported	8KoqAI
3	85636421	G	A	0.020394	0.003131	7.30E-11	0.62625	462346	NA	rs7634761	NA	UKB-b-57	TRUE	reported	8KoqAI
3	85636628	A	G	0.020522	0.003125	5.20E-11	0.626145	462346	NA	rs4508796	NA	UKB-b-57	TRUE	reported	8KoqAI
3	85638325	C	G	0.020531	0.003171	9.50E-11	0.64725	462346	NA	rs6225053	NA	UKB-b-57	TRUE	reported	8KoqAI
3	85639548	T	A	0.020394	0.003131	7.30E-11	0.62625	462346	NA	rs6794866	NA	UKB-b-57	TRUE	reported	8KoqAI
3	85639672	G	A	0.020589	0.003166	7.80E-11	0.647151	462346	NA	rs6782190	NA	UKB-b-57	TRUE	reported	8KoqAI
3	85640136	C	G	0.020396	0.003131	7.30E-11	0.62625	462346	NA	rs6795539	NA	UKB-b-57	TRUE	reported	8KoqAI
3	85640418	A	G	0.020524	0.003125	5.10E-11	0.626144	462346	NA	rs6800104	NA	UKB-b-57	TRUE	reported	8KoqAI
3	85640815	C	G	-0.02525	0.003623	3.20E-12	0.226898	462346	NA	rs9826458	NA	UKB-b-57	TRUE	reported	8KoqAI
3	85641825	G	T	0.020385	0.003128	7.20E-11	0.626027	462346	NA	rs1191909	NA	UKB-b-57	TRUE	reported	8KoqAI
3	85642479	G	A	0.020387	0.003136	8.00E-11	0.627462	462346	NA	rs1375566	NA	UKB-b-57	TRUE	reported	8KoqAI
3	85642881	A	G	0.02036	0.003136	8.50E-11	0.626266	462346	NA	rs1375565	NA	UKB-b-57	TRUE	reported	8KoqAI
3	85644047	T	A	0.020467	0.003138	6.90E-11	0.626261	462346	NA	rs2029130	NA	UKB-b-57	TRUE	reported	8KoqAI
3	85644366	G	C	0.020612	0.003178	8.80E-11	0.647195	462346	NA	rs1086561	NA	UKB-b-57	TRUE	reported	8KoqAI
3	85644911	T	C	0.020442	0.003184	1.40E-10	0.647225	462346	NA	rs1205432	NA	UKB-b-57	TRUE	reported	8KoqAI
3	85645057	C	T	0.020309	0.003144	1.00E-10	0.626291	462346	NA	rs6225054	NA	UKB-b-57	TRUE	reported	8KoqAI
3	85645873	C	T	0.020061	0.003187	3.10E-10	0.648759	462346	NA	rs2044725	NA	UKB-b-57	TRUE	reported	8KoqAI
3	85646002	A	G	0.02031	0.003184	1.80E-10	0.647091	462346	NA	rs2044723	NA	UKB-b-57	TRUE	reported	8KoqAI
3	85646426	T	G	0.020508	0.003178	1.10E-10	0.647056	462346	NA	rs1900916	NA	UKB-b-57	TRUE	reported	8KoqAI
3	85646525	T	A	0.020366	0.003138	8.60E-11	0.626099	462346	NA	rs1947221	NA	UKB-b-57	TRUE	reported	8KoqAI
3	85646828	G	A	0.020384	0.003185	1.60E-10	0.647289	462346	NA	rs1449402	NA	UKB-b-57	TRUE	reported	8KoqAI
3	85646850	C	T	0.020398	0.003185	1.50E-10	0.647262	462346	NA	rs1449401	NA	UKB-b-57	TRUE	reported	8KoqAI
3	85647614	G	T	0.020248	0.003148	1.30E-10	0.626399	462346	NA	rs6226391	NA	UKB-b-57	TRUE	reported	8KoqAI
3	85648246	G	A	0.020237	0.00315	1.30E-10	0.626397	462346	NA	rs6226391	NA	UKB-b-57	TRUE	reported	8KoqAI
3	85648957	G	T	-0.02555	0.003638	2.20E-12	0.226372	462346	NA	rs3887138	NA	UKB-b-57	TRUE	reported	8KoqAI
3	85649198	T	G	0.020321	0.003153	1.20E-10	0.626557	462346	NA	rs956281	NA	UKB-b-57	TRUE	reported	8KoqAI
3	85650004	T	C	-0.02556	0.003641	2.20E-12	0.226348	462346	NA	rs9866322	NA	UKB-b-57	TRUE	reported	8KoqAI
3	85650217	T	C	-0.0255	0.003641	2.50E-12	0.226489	462346	NA	rs9866761	NA	UKB-b-57	TRUE	reported	8KoqAI
3	85650790	A	C	0.020365	0.003162	1.20E-10	0.627636	462346	NA	rs4856598	NA	UKB-b-57	TRUE	reported	8KoqAI
3	85651136	A	G	0.020177	0.003159	1.70E-10	0.626477	462346	NA	rs3449510	NA	UKB-b-57	TRUE	reported	8KoqAI
3	1.32E+08	T	G	0.019101	0.003481	4.10E-08	0.259648	462346	NA	rs922505	NA	UKB-b-57	TRUE	reported	8KoqAI
3	1.32E+08	C	T	0.018509	0.003387	4.60E-08	0.278863	462346	NA	rs9850253	NA	UKB-b-57	TRUE	reported	8KoqAI
3	1.32E+08	A	G	0.018456	0.003387	5.00E-08	0.278764	462346	NA	rs1170940	NA	UKB-b-57	TRUE	reported	8KoqAI
3	1.32E+08	A	G	0.018482	0.003386	4.80E-08	0.278857	462346	NA	rs1170923	NA	UKB-b-57	TRUE	reported	8KoqAI
3	1.32E+08	G	C	0.018511	0.003386	4.60E-08	0.278824	462346	NA	rs750049	NA	UKB-b-57	TRUE	reported	8KoqAI
3	1.32E+08	T	G	0.018519	0.003386	4.50E-08	0.278771	462346	NA	rs9289399	NA	UKB-b-57	TRUE	reported	8KoqAI
3	1.32E+08	C	T	0.018495	0.003389	4.90E-08	0.277808	462346	NA	rs1308351	NA	UKB-b-57	TRUE	reported	8KoqAI
3	1.32E+08	A	T	0.018501	0.003389	4.80E-08	0.277806	462346	NA	rs1308355	NA	UKB-b-57	TRUE	reported	8KoqAI
3	1.32E+08	T	C	0.018506	0.00339	4.80E-08	0.277808	462346	NA	rs1306393	NA	UKB-b-57	TRUE	reported	8KoqAI
3	1.32E+08	T	G	0.018497	0.003389	4.80E-08	0.277808	462346	NA	rs1306397	NA	UKB-b-57	TRUE	reported	8KoqAI
3	1.32E+08	G	A	0.020422	0.003534	7.60E-09	0.244726	462346	NA	rs9850646	NA	UKB-b-57	TRUE	reported	8KoqAI
3	1.32E+08	G	C	0.020541	0.003745	4.10E-08	0.213017	462346	NA	rs9838144	NA	UKB-b-57	TRUE	reported	8KoqAI
3	1.32E+08	G	A	0.020525	0.003531	6.10E-09	0.244633	462346	NA	rs9858200	NA	UKB-b-57	TRUE	reported	8KoqAI
3	1.32E+08	T	G	0.018573	0.003382	4.00E-08	0.277788	462346	NA	rs6801798	NA	UKB-b-57	TRUE	reported	8KoqAI
3	1.32E+08	C	T	0.020533	0.003528	5.90E-09	0.244881	462346	NA	rs6787662	NA	UKB-b-57	TRUE	reported	8KoqAI
3	1.32E+08	C	T	0.020545	0.003528	5.80E-09	0.244898	462346	NA	rs6787869	NA	UKB-b-57	TRUE	reported	8KoqAI
3	1.32E+08	G	C	0.020534	0.003528	5.90E-09	0.244875	462346	NA	rs1249801	NA	UKB-b-57	TRUE	reported	8KoqAI
3	1.32E+08	C	T	0.018682	0.003384	3.40E-08	0.277338	462346	NA	rs9855089	NA	UKB-b-57	TRUE	reported	8KoqAI
3	1.32E+08	T	A	0.018663	0.003384	3.50E-08	0.277325	462346	NA	rs9875979	NA	UKB-b-57	TRUE	reported	8KoqAI
3	1.32E+08	C	G	0.018711	0.003384	3.20E-08	0.277408	462346	NA	rs9837417	NA	UKB-b-57	TRUE	reported	8KoqAI
3	1.32E+08	A	G	0.018752	0.003384	3.00E-08	0.277441	462346	NA	rs9876011	NA	UKB-b-57	TRUE	reported	8KoqAI
3	1.32E+08	A	G	0.018703	0.003384	3.20E-08	0.277507	462346	NA	rs9816797	NA	UKB-b-57	TRUE	reported	8KoqAI
3	1.32E+08	C	A	0.018557	0.003385	4.20E-08	0.277154	462346	NA	rs1841536	NA	UKB-b-57	TRUE	reported	8KoqAI
3	1.32E+08	C	T	0.018618	0.003388	3.90E-08	0.276903	462346	NA	rs9847672	NA	UKB-b-57	TRUE	reported	8KoqAI
3	1.32E+08	G	A	0.018526	0.003388	4.50E-08	0.276927	462346	NA	rs1841534	NA	UKB-b-57	TRUE	reported	8KoqAI
3	1.32E+08	T	C	0.018743	0.003388	3.20E-08	0.276946	462346	NA	rs9872712	NA	UKB-b-57	TRUE	reported	8KoqAI
3	1.32E+08	G	T	0.018898	0.003388	2.40E-08	0.276992	462346	NA	rs9869417	NA	UKB-b-57	TRUE	reported	8KoqAI
3	1.32E+08	A	C	0.018897	0.003387	2.40E-08	0.277288	462346	NA	rs9835574	NA	UKB-b-57	TRUE	reported	8KoqAI
3	1.32E+08	G	A	0.018896	0.003386	2.40E-08	0.277164	462346	NA	rs9874137	NA	UKB-b-57	TRUE	reported	8KoqAI
3	1.32E+08	G	C	0.01892	0.003385	2.30E-08	0.277125	462346	NA	rs9874767	NA	UKB-b-57	TRUE	reported	8KoqAI
3	1.32E+08	G	A	0.018857	0.003384	2.50E-08	0.277136	462346	NA	rs3749262	NA	UKB-b-57	TRUE	reported	8KoqAI
3	1.32E+08	T	C	0.019124	0.003383	1.60E-08	0.277902	462346	NA	rs1225004	NA	UKB-b-57	TRUE	reported	8KoqAI
3	1.32E+08	T	G	0.019132	0.003382	1.50E-08	0.27802	462346	NA	rs733344	NA	UKB-b-57	TRUE	reported	8KoqAI
3	1.32E+08	G	A	0.018739	0.003382	3.00E-08	0.277379	462346	NA	rs747503	NA	UKB-b-57	TRUE	reported	8KoqAI
3	1.32E+08	C	T	0.018788	0.003379	2.70E-08	0.278148	462346	NA	rs957919	NA	UKB-b-57	TRUE	reported	8KoqAI
3	1.32E+08	C	T	0.019087	0.003384	1.70E-08	0.278667	462346	NA	rs1224997	NA	UKB-b-57	TRUE	reported	8KoqAI
3	1.32E+08	C	T	0.019878	0.003437	7.30E-09	0.265218	462346	NA	rs1228588	NA	UKB-b-57	TRUE	reported	8KoqAI
3	1.32E+08	G	A	0.02107	0.003528	2.30E-09	0.246133	462346	NA	rs1228589	NA	UKB-b-57	TRUE	reported	8KoqAI
3	1.32E+08	G	A	0.018987	0.003388	2.10E-08	0.278035	462346	NA	rs1225060	NA	UKB-b-57	TRUE	reported	8KoqAI
3	1.32E+08	T	A	0.018954	0.003387	2.20E-08	0.278199	462346	NA	rs1225057	NA	UKB-b-57	TRUE	reported	8KoqAI
3	1.32E+08	C	A	0.019746	0.00344	9.50E-09	0.264813	462346	NA	rs1225056	NA	UKB-b-57	TRUE	reported	8KoqAI
3	1.32E+08	T	C	0.019802	0.003441	8.70E-09	0.264742	462346	NA	rs1225053	NA	UKB-b-57	TRUE	reported	8KoqAI
3	1.32E+08	C	T	0.019806	0.003444	8.90E-09	0.26509	462346	NA	rs1225049	NA	UKB-b-57	TRUE	reported	8KoqAI
3	1.32E+08	C	G	0.019729	0.003445	1.00E-08	0.264981	462346	NA	rs1225046	NA	UKB-b-57	TRUE	reported	8KoqAI
3	1.74E+08	A	C	0.017212	0.003143	4.30E-08	0.36783	462346	NA	rs7620744	NA	UKB-b-57	TRUE	reported	8KoqAI

3	1.74E+08	A	G	0.017619	0.003192	3.40E-08	0.351356	462346	NA	rs7643292	NA	UKB-b-57	TRUE	reported	8KoqAI
3	1.74E+08	T	C	0.01766	0.003192	3.20E-08	0.35131	462346	NA	rs7645708	NA	UKB-b-57	TRUE	reported	8KoqAI
3	1.74E+08	C	T	0.017278	0.003169	5.00E-08	0.356488	462346	NA	rs1093679	NA	UKB-b-57	TRUE	reported	8KoqAI
3	1.74E+08	G	A	0.018094	0.003113	6.10E-09	0.384022	462346	NA	rs1249434	NA	UKB-b-57	TRUE	reported	8KoqAI
3	1.74E+08	C	T	0.018083	0.003112	6.20E-09	0.38395	462346	NA	rs1093679	NA	UKB-b-57	TRUE	reported	8KoqAI
3	1.74E+08	G	A	0.0179	0.003113	9.00E-09	0.383728	462346	NA	rs938441	NA	UKB-b-57	TRUE	reported	8KoqAI
3	1.74E+08	G	A	0.017998	0.003113	7.40E-09	0.383903	462346	NA	rs1684813	NA	UKB-b-57	TRUE	reported	8KoqAI
3	1.74E+08	T	G	0.01823	0.003116	4.90E-09	0.383186	462346	NA	rs1515591	NA	UKB-b-57	TRUE	reported	8KoqAI
3	1.74E+08	C	T	0.018245	0.003116	4.80E-09	0.383245	462346	NA	rs1515590	NA	UKB-b-57	TRUE	reported	8KoqAI
3	1.74E+08	T	C	0.01807	0.003113	6.50E-09	0.384082	462346	NA	rs1515589	NA	UKB-b-57	TRUE	reported	8KoqAI
3	1.74E+08	C	A	0.018042	0.003113	6.80E-09	0.384129	462346	NA	rs1093680	NA	UKB-b-57	TRUE	reported	8KoqAI
3	1.74E+08	C	T	0.018035	0.003113	6.90E-09	0.38413	462346	NA	rs1093680	NA	UKB-b-57	TRUE	reported	8KoqAI
3	1.74E+08	A	G	0.017857	0.003118	1.00E-08	0.383436	462346	NA	rs1515588	NA	UKB-b-57	TRUE	reported	8KoqAI
3	1.74E+08	C	T	0.01796	0.003118	8.40E-09	0.383197	462346	NA	rs1515587	NA	UKB-b-57	TRUE	reported	8KoqAI
3	1.74E+08	G	A	0.018028	0.003114	7.10E-09	0.38416	462346	NA	rs6674095	NA	UKB-b-57	TRUE	reported	8KoqAI
4	2468696	C	T	0.064072	0.01149	2.50E-08	0.019578	462346	NA	rs1154006	NA	UKB-b-57	TRUE	reported	8KoqAI
4	2935618	C	G	0.047152	0.006963	1.30E-11	0.052008	462346	NA	rs1785125	NA	UKB-b-57	TRUE	reported	8KoqAI
4	3078937	A	G	-0.01759	0.003085	1.20E-08	0.411037	462346	NA	rs3905238	NA	UKB-b-57	TRUE	reported	8KoqAI
4	3080173	G	A	-0.01741	0.003077	1.50E-08	0.420375	462346	NA	rs3856973	NA	UKB-b-57	TRUE	reported	8KoqAI
4	3085477	G	A	-0.01786	0.003073	6.20E-09	0.421344	462346	NA	rs2886743	NA	UKB-b-57	TRUE	reported	8KoqAI
4	3089259	A	G	-0.0174	0.00307	1.40E-08	0.424572	462346	NA	rs2285086	NA	UKB-b-57	TRUE	reported	8KoqAI
4	3089564	C	T	-0.01764	0.003105	1.30E-08	0.393613	462346	NA	rs6134820	NA	UKB-b-57	TRUE	reported	8KoqAI
4	3097495	G	A	0.017827	0.003126	1.20E-08	0.378516	462346	NA	rs2882009	NA	UKB-b-57	TRUE	reported	8KoqAI
4	3109442	A	G	0.01906	0.00311	8.90E-10	0.383514	462346	NA	rs1001597	NA	UKB-b-57	TRUE	reported	8KoqAI
4	3111103	T	C	-0.01738	0.003068	1.50E-08	0.42248	462346	NA	rs6446722	NA	UKB-b-57	TRUE	reported	8KoqAI
4	3111410	G	A	-0.01778	0.00307	6.90E-09	0.42113	462346	NA	rs7691627	NA	UKB-b-57	TRUE	reported	8KoqAI
4	3117579	T	C	-0.01817	0.003071	3.20E-09	0.420817	462346	NA	rs1778079	NA	UKB-b-57	TRUE	reported	8KoqAI
4	3118116	T	G	-0.01818	0.003071	3.20E-09	0.420762	462346	NA	rs2071655	NA	UKB-b-57	TRUE	reported	8KoqAI
4	3122507	T	G	-0.01814	0.003071	3.50E-09	0.421265	462346	NA	rs4690072	NA	UKB-b-57	TRUE	reported	8KoqAI
4	3126813	T	C	-0.01791	0.003068	5.30E-09	0.423167	462346	NA	rs6446723	NA	UKB-b-57	TRUE	reported	8KoqAI
4	3141488	G	A	-0.01781	0.003076	7.10E-09	0.414859	462346	NA	rs363063	NA	UKB-b-57	TRUE	reported	8KoqAI
4	3151813	C	T	0.020227	0.003202	2.70E-10	0.339862	462346	NA	rs1173123	NA	UKB-b-57	TRUE	reported	8KoqAI
4	3160150	G	A	-0.01843	0.003068	1.90E-09	0.422467	462346	NA	rs4690073	NA	UKB-b-57	TRUE	reported	8KoqAI
4	3166250	G	A	0.018158	0.003143	7.60E-09	0.367163	462346	NA	rs9884693	NA	UKB-b-57	TRUE	reported	8KoqAI
4	3171370	A	G	-0.01828	0.003067	2.50E-09	0.423107	462346	NA	rs4690011	NA	UKB-b-57	TRUE	reported	8KoqAI
4	3172439	C	T	-0.01829	0.003069	2.60E-09	0.421697	462346	NA	rs2840321	NA	UKB-b-57	TRUE	reported	8KoqAI
4	3180021	T	C	-0.02025	0.003066	3.90E-11	0.575152	462346	NA	rs363096	NA	UKB-b-57	TRUE	reported	8KoqAI
4	3190486	T	C	-0.0183	0.003067	2.40E-09	0.422023	462346	NA	rs6844859	NA	UKB-b-57	TRUE	reported	8KoqAI
4	3196029	A	C	0.018185	0.003072	3.20E-09	0.581743	462346	NA	rs363092	NA	UKB-b-57	TRUE	reported	8KoqAI
4	3200079	A	G	-0.018	0.003071	4.60E-09	0.418639	462346	NA	rs2298971	NA	UKB-b-57	TRUE	reported	8KoqAI
4	3207142	A	G	-0.01788	0.003066	5.50E-09	0.422692	462346	NA	rs7685686	NA	UKB-b-57	TRUE	reported	8KoqAI
4	3215835	T	C	-0.0177	0.003064	7.60E-09	0.422874	462346	NA	rs362331	NA	UKB-b-57	TRUE	reported	8KoqAI
4	3216815	C	G	-0.01806	0.00307	4.00E-09	0.418263	462346	NA	rs916171	NA	UKB-b-57	TRUE	reported	8KoqAI
4	3223891	T	C	-0.01814	0.003066	3.30E-09	0.423141	462346	NA	rs2071662	NA	UKB-b-57	TRUE	reported	8KoqAI
4	3224933	T	G	-0.01831	0.003065	2.30E-09	0.422457	462346	NA	rs362319	NA	UKB-b-57	TRUE	reported	8KoqAI
4	3225389	A	G	-0.01837	0.003065	2.10E-09	0.422616	462346	NA	rs82333	NA	UKB-b-57	TRUE	reported	8KoqAI
4	3225478	T	C	-0.01817	0.003065	3.10E-09	0.422586	462346	NA	rs110501	NA	UKB-b-57	TRUE	reported	8KoqAI
4	3227592	T	A	-0.01734	0.003064	1.50E-08	0.427475	462346	NA	rs2276882	NA	UKB-b-57	TRUE	reported	8KoqAI
4	3228492	A	G	-0.01794	0.003068	5.00E-09	0.420559	462346	NA	rs2237006	NA	UKB-b-57	TRUE	reported	8KoqAI
4	3230804	A	C	0.017577	0.003092	1.30E-08	0.59688	462346	NA	rs2530598	NA	UKB-b-57	TRUE	reported	8KoqAI
4	3234828	G	T	-0.01796	0.003073	5.10E-09	0.415476	462346	NA	rs2269478	NA	UKB-b-57	TRUE	reported	8KoqAI
4	3235241	A	G	-0.01883	0.003072	8.80E-10	0.417616	462346	NA	rs362314	NA	UKB-b-57	TRUE	reported	8KoqAI
4	3235450	T	C	-0.01891	0.003066	6.90E-10	0.423303	462346	NA	rs2237007	NA	UKB-b-57	TRUE	reported	8KoqAI
4	3237644	T	C	-0.01802	0.00307	4.40E-09	0.424067	462346	NA	rs362312	NA	UKB-b-57	TRUE	reported	8KoqAI
4	3240159	C	T	-0.01942	0.003072	2.60E-10	0.420536	462346	NA	rs2071704	NA	UKB-b-57	TRUE	reported	8KoqAI
4	3240915	G	C	-0.01857	0.003075	1.50E-09	0.417988	462346	NA	rs362309	NA	UKB-b-57	TRUE	reported	8KoqAI
4	3241845	C	T	0.043305	0.005802	8.40E-14	0.074582	462346	NA	rs362307	NA	UKB-b-57	TRUE	reported	8KoqAI
4	3249828	T	C	0.018885	0.003217	4.30E-09	0.345963	462346	NA	rs1264680	NA	UKB-b-57	TRUE	reported	8KoqAI
4	3253183	A	G	-0.01855	0.003066	1.40E-09	0.445326	462346	NA	rs3095075	NA	UKB-b-57	TRUE	reported	8KoqAI
4	3254767	T	A	-0.01769	0.003078	9.00E-09	0.437261	462346	NA	rs3129321	NA	UKB-b-57	TRUE	reported	8KoqAI
4	3256805	A	G	-0.01909	0.003073	5.30E-10	0.430887	462346	NA	rs3135174	NA	UKB-b-57	TRUE	reported	8KoqAI
4	3258236	C	G	-0.01905	0.003069	5.40E-10	0.430976	462346	NA	rs108850	NA	UKB-b-57	TRUE	reported	8KoqAI
4	3259065	T	C	-0.01898	0.003067	6.10E-10	0.431292	462346	NA	rs2079642	NA	UKB-b-57	TRUE	reported	8KoqAI
4	3259632	G	C	-0.0171	0.003119	4.10E-08	0.382961	462346	NA	rs362266	NA	UKB-b-57	TRUE	reported	8KoqAI
4	3260057	A	G	-0.02014	0.003059	4.60E-11	0.437629	462346	NA	rs362280	NA	UKB-b-57	TRUE	reported	8KoqAI
4	3260072	G	A	-0.01717	0.003119	3.70E-08	0.38228	462346	NA	rs362279	NA	UKB-b-57	TRUE	reported	8KoqAI
4	3261127	A	G	-0.01732	0.0031	2.30E-08	0.393625	462346	NA	rs3135171	NA	UKB-b-57	TRUE	reported	8KoqAI
4	3261152	G	A	-0.01732	0.003099	2.30E-08	0.393747	462346	NA	rs3095074	NA	UKB-b-57	TRUE	reported	8KoqAI
4	3262105	T	G	-0.01751	0.00311	1.80E-08	0.384368	462346	NA	rs4690081	NA	UKB-b-57	TRUE	reported	8KoqAI
4	3262323	T	C	-0.01786	0.003073	6.20E-09	0.41348	462346	NA	rs3135170	NA	UKB-b-57	TRUE	reported	8KoqAI
4	3262374	C	T	-0.0176	0.00311	1.50E-08	0.383991	462346	NA	rs3135169	NA	UKB-b-57	TRUE	reported	8KoqAI
4	3262699	T	C	-0.01771	0.003109	1.20E-08	0.384189	462346	NA	rs3135168	NA	UKB-b-57	TRUE	reported	8KoqAI
4	3263138	G	A	-0.01733	0.003111	2.50E-08	0.383015	462346	NA	rs3095073	NA	UKB-b-57	TRUE	reported	8KoqAI
4	3263325	G	A	-0.0171	0.003114	4.00E-08	0.381981	462346	NA	rs3095072	NA	UKB-b-57	TRUE	reported	8KoqAI
4	3263499	G	A	-0.01714	0.003112	3.60E-08	0.382977	462346	NA	rs3095083	NA	UKB-b-57	TRUE	reported	8KoqAI
4	3263617	C	A	-0.01739	0.00311	2.30E-08	0.383851	462346	NA	rs3095082	NA	UKB-b-57	TRUE	reported	8KoqAI
4	3263776	C	T	-0.01703	0.003113	4.50E-08	0.382191	462346	NA	rs3095081	NA	UKB-b-57	TRUE	reported	8KoqAI
4	3264210	T	C	-0.01754	0.003111	1.70E-08	0.384163	462346	NA	rs3121420	NA	UKB-b-57	TRUE	reported	8KoqAI
4	3265130	G	C	-0.01825	0.003065	2.60E-09	0.422495	462346	NA	rs3129320	NA	UKB-b-57	TRUE	reported	8KoqAI
4	3266860	C	T	-0.01806	0.003061	3.60E-09	0.567988	462346	NA	rs2108753	NA	UKB-b-57	TRUE	reported	8KoqAI
4	3267342	G	A	-0.01812	0.003061	3.20E-09	0.568936	462346	NA	rs2798225	NA	UKB-b-57	TRUE	reported	8KoqAI
4	3267668	G	A	-0.01787	0.00306	5.20E-09	0.568347	462346	NA	rs2798224	NA	UKB-b-57	TRUE	reported	8KoqAI
4	3268646	A	G	-0.01803	0.003067	4.10E-09	0.57823	462346	NA	rs2858089	NA	UKB-b-57	TRUE	reported	8KoqAI
4	3268710	A	G	-0.02061	0.003119	3.90E-11	0.61771	462346	NA	rs2858088	NA	UKB-b-57	TRUE	reported	8KoqAI
4	3268716	T	C	-0.01859	0.00307	1.40E-09	0.579365	462346	NA	rs2798222	NA	UKB-b-57	TRUE	reported	8KoqAI
4	3268892	T	A	-0.01782	0.003085	7.50E-09	0.597143	462346	NA	rs2858087	NA	UKB-b-57	TRUE	reported	8KoqAI
4	3269787	C	G	-0.01824	0.003084	3.30E-09	0.596615	462346	NA	rs2071671	NA	UKB-b-57	TRUE	reported	8KoqAI
4	3269905	A	G	-0.01798	0.003088	5.80E-09	0.599483	462346	NA	rs2071668	NA	UKB-b-57	TRUE	reported	8KoqAI
4	3269926	C	A	-0.01826	0.003085	3.30E-09	0.597471	462346	NA	rs2071667	NA	UKB-b-57	TRUE	reported	8KoqAI
4	3270488	T	C	-0.01813	0.003089	4.40E-09	0.596784	462346	NA	rs2798221	NA	UKB-b-57	TRUE	reported	8KoqAI
4	3270611	A	G	-0.01835	0.003085	2.70E-09	0.597884	462346	NA	rs2858086	NA	UKB-b-57	TRUE	reported	8KoqAI
4	3270913	C	G	-0.01826	0.003086	3.30E-09	0.597677	462346	NA	rs2157093	NA	UKB-b-57	TRUE	reported	8KoqAI
4	3271226	T	G	-0.01859	0.003087	1.70E-09	0.598246	462346	NA	rs2858085	NA	UKB-b-57	TRUE	reported	8KoqAI
4	3272118	A	C	0.018409	0.003109	3.20E-09	0.386574	462346	NA	rs2858084	NA	UKB-b-57	TRUE	reported	8KoqAI
4	3272331	T	A	0.018318	0.003109	3.80E-09	0.386379	462346	NA	rs2858083	NA	UKB-b-57	TRUE	reported	8KoqAI
4	3272378	C	G	0.018329	0.003109	3.70E-09	0.386371	462346	NA	rs2530601	NA	UKB-b-57	TRUE	reported	8KoqAI
4	3272782	G	A	0.04119	0.005717	5.80E-13	0.077021	462346	NA	rs7603478	NA	UKB-b-57	TRUE	reported	8KoqAI
4	3272930	C	G	0.018496	0.003109	2.70E-09	0.386334	462346	NA	rs2530597	NA	UKB-b-57	TRUE	reported	8KoqAI
4	3273092	G	C	0.018753	0.00312	1.80E-09	0.383434	462346	NA	rs2157092	NA	UKB-b-57	TRUE	reported	8KoqAI
4	3273099	G	A	0.018727	0.00312	1.90E-09	0.383435	462346	NA	rs2157091	NA	UKB-b-57	TRUE	reported	8KoqAI
4	3273110	C	T	0.018579	0.003122	2.70E-09	0.381958	462346	NA	rs2157090	NA	UKB-b-57	TRUE	reported	8KoqAI
4	3273538	C	G	0.018812	0.00311	1.50E-09	0.385663	462346	NA	rs7674472	NA	UKB-b-57	TRUE	reported	8KoqAI
4	3273625	G	C	0.018842	0.00311	1.40E-09	0.385651	462346	NA	rs7656026	NA	UKB-b-57	TRUE	reported	8KoqAI
4	3273908	C	T	0.021163	0.003182	2.90E-11	0.347123	462346	NA	rs7675084	NA	UKB-b-57	TRUE	reported	8KoqAI
4	3274214	T	C	0.018925	0.003112	1.20E-09	0.385145	462346	NA	rs6446726	NA	UKB-b-57	TRUE	reported	8KoqAI
4	3274488	T	A	0.01843	0.003109	3.10E-09	0.386217	462346	NA	rs6446727	NA	UKB-b-57	TRUE	reported	8KoqAI
4	3275035	A	G	0.019001	0.003112	1.00E-09	0.384985	462346	NA	rs1080500	NA	UKB-b-57	TRUE	reported	8KoqAI

4	3275484	C	T	0.021258	0.003182	2.40E-11	0.347392	462346	NA	rs9996868	NA	UKB-b-57	TRUE	reported	8KoqAI
4	3275650	A	G	0.019047	0.003112	9.30E-10	0.384964	462346	NA	rs6446728	NA	UKB-b-57	TRUE	reported	8KoqAI
4	3276491	C	T	0.021165	0.003182	2.90E-11	0.347245	462346	NA	rs1093792	NA	UKB-b-57	TRUE	reported	8KoqAI
4	3280106	G	A	0.018265	0.003108	4.20E-09	0.385822	462346	NA	rs7661630	NA	UKB-b-57	TRUE	reported	8KoqAI
4	3281369	A	G	0.031676	0.004508	2.10E-12	0.129866	462346	NA	rs1120538	NA	UKB-b-57	TRUE	reported	8KoqAI
4	3281526	G	A	0.031779	0.004509	1.80E-12	0.12978	462346	NA	rs7319333	NA	UKB-b-57	TRUE	reported	8KoqAI
4	3281799	A	G	0.031645	0.004441	1.00E-12	0.135125	462346	NA	rs7319334	NA	UKB-b-57	TRUE	reported	8KoqAI
4	3281856	C	T	0.031519	0.004605	7.70E-12	0.125934	462346	NA	rs7887187	NA	UKB-b-57	TRUE	reported	8KoqAI
4	3281868	T	C	0.031518	0.004606	7.80E-12	0.125953	462346	NA	rs7857828	NA	UKB-b-57	TRUE	reported	8KoqAI
4	3281869	G	A	0.031541	0.004607	7.60E-12	0.125874	462346	NA	rs1142986	NA	UKB-b-57	TRUE	reported	8KoqAI
4	3282181	C	T	0.031727	0.004507	1.90E-12	0.129826	462346	NA	rs7319334	NA	UKB-b-57	TRUE	reported	8KoqAI
4	3282774	T	A	0.031649	0.004506	2.20E-12	0.129888	462346	NA	rs1251048	NA	UKB-b-57	TRUE	reported	8KoqAI
4	3282848	C	T	0.031649	0.004505	2.10E-12	0.129889	462346	NA	rs1250800	NA	UKB-b-57	TRUE	reported	8KoqAI
4	3283339	C	T	0.031595	0.004504	2.30E-12	0.129909	462346	NA	rs7658412	NA	UKB-b-57	TRUE	reported	8KoqAI
4	3283422	C	T	0.028403	0.004021	1.60E-12	0.172021	462346	NA	rs7658462	NA	UKB-b-57	TRUE	reported	8KoqAI
4	3283996	T	G	0.03161	0.004502	2.20E-12	0.129919	462346	NA	rs5701088	NA	UKB-b-57	TRUE	reported	8KoqAI
4	3284186	A	T	0.031598	0.004501	2.20E-12	0.129926	462346	NA	rs1250498	NA	UKB-b-57	TRUE	reported	8KoqAI
4	3284305	A	G	0.031603	0.004501	2.20E-12	0.129937	462346	NA	rs1250501	NA	UKB-b-57	TRUE	reported	8KoqAI
4	3284340	G	A	0.031492	0.004498	2.50E-12	0.130147	462346	NA	rs1250045	NA	UKB-b-57	TRUE	reported	8KoqAI
4	3284560	T	C	0.031531	0.004501	2.50E-12	0.129963	462346	NA	rs1250682	NA	UKB-b-57	TRUE	reported	8KoqAI
4	3284751	G	A	0.019393	0.003354	7.40E-09	0.28597	462346	NA	rs6446731	NA	UKB-b-57	TRUE	reported	8KoqAI
4	3285477	T	A	0.031387	0.004507	3.30E-12	0.129879	462346	NA	rs7319334	NA	UKB-b-57	TRUE	reported	8KoqAI
4	3286505	G	A	0.031433	0.004511	3.20E-12	0.129891	462346	NA	rs7319334	NA	UKB-b-57	TRUE	reported	8KoqAI
4	3297743	C	T	0.019773	0.003484	1.40E-08	0.254925	462346	NA	rs7695631	NA	UKB-b-57	TRUE	reported	8KoqAI
4	3298800	T	C	0.030734	0.004477	6.70E-12	0.132574	462346	NA	rs2051559	NA	UKB-b-57	TRUE	reported	8KoqAI
4	3300083	A	G	0.022467	0.003617	5.20E-10	0.227947	462346	NA	rs6829279	NA	UKB-b-57	TRUE	reported	8KoqAI
4	3305116	A	G	0.019166	0.003468	3.30E-08	0.257113	462346	NA	rs743699	NA	UKB-b-57	TRUE	reported	8KoqAI
4	3319271	C	T	0.01987	0.00348	1.10E-08	0.253144	462346	NA	rs1000636	NA	UKB-b-57	TRUE	reported	8KoqAI
4	3325181	T	C	0.019648	0.003478	1.60E-08	0.253474	462346	NA	rs7679872	NA	UKB-b-57	TRUE	reported	8KoqAI
4	3330248	C	T	0.020232	0.00349	6.70E-09	0.251626	462346	NA	rs2860004	NA	UKB-b-57	TRUE	reported	8KoqAI
4	3334614	C	T	0.019378	0.003479	2.60E-08	0.253606	462346	NA	rs6852922	NA	UKB-b-57	TRUE	reported	8KoqAI
4	3339038	G	T	0.02193	0.00362	1.40E-09	0.226307	462346	NA	rs7668784	NA	UKB-b-57	TRUE	reported	8KoqAI
4	3341230	C	G	0.022723	0.003633	4.00E-10	0.224222	462346	NA	rs1250052	NA	UKB-b-57	TRUE	reported	8KoqAI
4	3347872	C	T	0.020314	0.003471	4.80E-09	0.255829	462346	NA	rs5959442	NA	UKB-b-57	TRUE	reported	8KoqAI
4	3367258	G	C	0.020478	0.003468	3.50E-09	0.256371	462346	NA	rs3549458	NA	UKB-b-57	TRUE	reported	8KoqAI
4	3368332	C	T	0.018205	0.003233	1.80E-08	0.324196	462346	NA	rs6843312	NA	UKB-b-57	TRUE	reported	8KoqAI
4	3368573	G	A	0.018454	0.003249	1.40E-08	0.319844	462346	NA	rs1002657	NA	UKB-b-57	TRUE	reported	8KoqAI
4	3368996	G	A	0.020716	0.003462	2.20E-09	0.258498	462346	NA	rs5898271	NA	UKB-b-57	TRUE	reported	8KoqAI
4	3369099	C	T	0.020571	0.003463	2.80E-09	0.257811	462346	NA	rs1684421	NA	UKB-b-57	TRUE	reported	8KoqAI
4	3372263	A	G	0.018725	0.003267	1.00E-08	0.310579	462346	NA	rs6826720	NA	UKB-b-57	TRUE	reported	8KoqAI
4	3373499	T	C	0.018802	0.003267	8.70E-09	0.310437	462346	NA	rs1311591	NA	UKB-b-57	TRUE	reported	8KoqAI
4	3374162	C	T	0.020535	0.003359	9.70E-10	0.283261	462346	NA	rs1315217	NA	UKB-b-57	TRUE	reported	8KoqAI
4	3375722	C	T	0.018576	0.003269	1.30E-08	0.310465	462346	NA	rs6855619	NA	UKB-b-57	TRUE	reported	8KoqAI
4	3376302	A	G	0.018487	0.003268	1.50E-08	0.310935	462346	NA	rs1021286	NA	UKB-b-57	TRUE	reported	8KoqAI
4	3380088	T	C	0.031456	0.004518	3.40E-12	0.131356	462346	NA	rs6851060	NA	UKB-b-57	TRUE	reported	8KoqAI
4	3381990	G	A	0.020731	0.003416	1.30E-09	0.282084	462346	NA	rs1313839	NA	UKB-b-57	TRUE	reported	8KoqAI
4	3383552	C	T	0.030531	0.004765	1.50E-10	0.119574	462346	NA	rs963695	NA	UKB-b-57	TRUE	reported	8KoqAI
4	3383553	A	G	0.01893	0.003467	4.70E-08	0.272145	462346	NA	rs2343800	NA	UKB-b-57	TRUE	reported	8KoqAI
4	3385176	A	T	0.033763	0.00553	1.00E-09	0.082864	462346	NA	rs1001279	NA	UKB-b-57	TRUE	reported	8KoqAI
4	3386876	G	A	0.077848	0.014241	4.60E-08	0.013644	462346	NA	rs1382475	NA	UKB-b-57	TRUE	reported	8KoqAI
4	3390146	T	C	0.030995	0.004906	2.70E-10	0.104754	462346	NA	rs2269496	NA	UKB-b-57	TRUE	reported	8KoqAI
4	3392274	C	T	0.028712	0.005172	2.80E-08	0.095468	462346	NA	rs7319338	NA	UKB-b-57	TRUE	reported	8KoqAI
4	3414654	G	A	0.03053	0.004996	9.90E-10	0.102223	462346	NA	rs1002792	NA	UKB-b-57	TRUE	reported	8KoqAI
4	3418084	A	G	0.03143	0.004969	2.50E-10	0.103564	462346	NA	rs1719095	NA	UKB-b-57	TRUE	reported	8KoqAI
4	3419840	G	A	0.03065	0.004979	7.50E-10	0.102773	462346	NA	rs1264198	NA	UKB-b-57	TRUE	reported	8KoqAI
4	3420271	G	A	0.030824	0.004984	6.20E-10	0.102744	462346	NA	rs3748037	NA	UKB-b-57	TRUE	reported	8KoqAI
4	3437308	C	T	0.030816	0.005237	4.00E-09	0.094496	462346	NA	rs2854818	NA	UKB-b-57	TRUE	reported	8KoqAI
4	3442011	G	C	0.019297	0.00354	5.00E-08	0.247474	462346	NA	rs762861	NA	UKB-b-57	TRUE	reported	8KoqAI
4	3443931	A	G	-0.01836	0.003134	4.70E-09	0.614833	462346	NA	rs1310821	NA	UKB-b-57	TRUE	reported	8KoqAI
4	3446091	G	T	0.024604	0.004337	1.40E-08	0.141036	462346	NA	rs3748034	NA	UKB-b-57	TRUE	reported	8KoqAI
4	3452345	G	A	0.018024	0.003254	3.10E-08	0.331962	462346	NA	rs5995028	NA	UKB-b-57	TRUE	reported	8KoqAI
4	25342606	A	G	-0.02016	0.003656	3.50E-08	0.233746	462346	NA	rs5620371	NA	UKB-b-57	TRUE	reported	8KoqAI
4	25408838	G	A	-0.02018	0.003593	1.90E-08	0.230728	462346	NA	rs3481147	NA	UKB-b-57	TRUE	reported	8KoqAI
4	38602202	C	G	-0.01731	0.003128	3.10E-08	0.37379	462346	NA	rs337633	NA	UKB-b-57	TRUE	reported	8KoqAI
4	38602945	A	T	-0.0173	0.003128	3.20E-08	0.373628	462346	NA	rs337634	NA	UKB-b-57	TRUE	reported	8KoqAI
4	38604470	G	A	-0.01728	0.003126	3.30E-08	0.373665	462346	NA	rs337637	NA	UKB-b-57	TRUE	reported	8KoqAI
4	38604807	T	C	-0.01722	0.003125	3.60E-08	0.374029	462346	NA	rs337638	NA	UKB-b-57	TRUE	reported	8KoqAI
4	38613107	C	G	-0.01853	0.003173	5.20E-09	0.349452	462346	NA	rs3449485	NA	UKB-b-57	TRUE	reported	8KoqAI
4	38625501	G	C	-0.01743	0.003169	3.80E-08	0.352196	462346	NA	rs1314483	NA	UKB-b-57	TRUE	reported	8KoqAI
4	38626522	C	T	-0.01746	0.003169	3.60E-08	0.3522	462346	NA	rs1310209	NA	UKB-b-57	TRUE	reported	8KoqAI
4	38632166	T	C	-0.01743	0.003172	3.90E-08	0.351972	462346	NA	rs3607140	NA	UKB-b-57	TRUE	reported	8KoqAI
4	38634704	T	C	-0.01749	0.003177	3.70E-08	0.351458	462346	NA	rs3554742	NA	UKB-b-57	TRUE	reported	8KoqAI
4	38659594	G	A	-0.01739	0.003171	4.10E-08	0.35333	462346	NA	rs3472200	NA	UKB-b-57	TRUE	reported	8KoqAI
4	38680015	A	G	-0.01739	0.003168	4.10E-08	0.360207	462346	NA	rs1313285	NA	UKB-b-57	TRUE	reported	8KoqAI
4	39061656	C	A	0.022358	0.003749	2.50E-09	0.212856	462346	NA	rs1250624	NA	UKB-b-57	TRUE	reported	8KoqAI
4	39080572	G	A	0.023463	0.003943	2.70E-09	0.179904	462346	NA	rs3567631	NA	UKB-b-57	TRUE	reported	8KoqAI
4	39224297	G	A	0.01676	0.003053	4.00E-08	0.559459	462346	NA	rs9997015	NA	UKB-b-57	TRUE	reported	8KoqAI
4	39227548	C	A	0.016682	0.003053	4.60E-08	0.559488	462346	NA	rs4974996	NA	UKB-b-57	TRUE	reported	8KoqAI
4	39234408	A	G	0.01698	0.003053	2.70E-08	0.560738	462346	NA	rs2060805	NA	UKB-b-57	TRUE	reported	8KoqAI
4	39238456	A	C	0.031528	0.003647	5.40E-18	0.239402	462346	NA	rs4974930	NA	UKB-b-57	TRUE	reported	8KoqAI
4	39240188	C	T	0.016856	0.003053	3.40E-08	0.560787	462346	NA	rs7666632	NA	UKB-b-57	TRUE	reported	8KoqAI
4	39242277	A	G	0.016914	0.00306	3.20E-08	0.560679	462346	NA	rs1001035	NA	UKB-b-57	TRUE	reported	8KoqAI
4	39249265	C	T	0.016947	0.003053	2.80E-08	0.561501	462346	NA	rs4974932	NA	UKB-b-57	TRUE	reported	8KoqAI
4	39253915	G	A	0.016998	0.003053	2.60E-08	0.561407	462346	NA	rs3473496	NA	UKB-b-57	TRUE	reported	8KoqAI
4	39256324	G	A	0.017051	0.003053	2.30E-08	0.561354	462346	NA	rs1173679	NA	UKB-b-57	TRUE	reported	8KoqAI
4	39258771	G	A	0.017007	0.003053	2.50E-08	0.561314	462346	NA	rs1451818	NA	UKB-b-57	TRUE	reported	8KoqAI
4	39259900	G	A	0.016983	0.003053	2.70E-08	0.561344	462346	NA	rs1250132	NA	UKB-b-57	TRUE	reported	8KoqAI
4	39265701	G	A	0.01704	0.003053	2.40E-08	0.561366	462346	NA	rs1109698	NA	UKB-b-57	TRUE	reported	8KoqAI
4	39266464	T	C	-0.01717	0.003054	1.90E-08	0.438989	462346	NA	rs4975001	NA	UKB-b-57	TRUE	reported	8KoqAI
4	39268005	A	C	-0.01701	0.003055	2.60E-08	0.439025	462346	NA	rs2008679	NA	UKB-b-57	TRUE	reported	8KoqAI
4	39268436	T	G	-0.01709	0.003054	2.20E-08	0.438422	462346	NA	rs1809112	NA	UKB-b-57	TRUE	reported	8KoqAI
4	39270885	T	G	-0.0171	0.003055	2.10E-08	0.438448	462346	NA	rs6531700	NA	UKB-b-57	TRUE	reported	8KoqAI
4	39271913	T	C	-0.01707	0.003055	2.30E-08	0.438442	462346	NA	rs3733281	NA	UKB-b-57	TRUE	reported	8KoqAI
4	39271995	G	C	-0.01952	0.003052	1.60E-10	0.469563	462346	NA	rs1264341	NA	UKB-b-57	TRUE	reported	8KoqAI
4	39273034	A	G	0.017243	0.003055	1.70E-08	0.56139	462346	NA	rs2381371	NA	UKB-b-57	TRUE	reported	8KoqAI
4	39273091	A	C	0.017189	0.003055	1.80E-08	0.561438	462346	NA	rs2381372	NA	UKB-b-57	TRUE	reported	8KoqAI
4	39273584	C	T	0.01721	0.003055	1.80E-08	0.561438	462346	NA	rs1265121	NA	UKB-b-57	TRUE	reported	8KoqAI
4	39275394	T	C	-0.01722	0.003055	1.70E-08	0.438432	462346	NA	rs6531702	NA	UKB-b-57	TRUE	reported	8KoqAI
4	39276990	A	G	0.030112	0.003826	3.50E-15	0.208286	462346	NA	rs6230832	NA	UKB-b-57	TRUE	reported	8KoqAI
4	39277296	T	C	-0.01717	0.003055	1.90E-08	0.438335	462346	NA	rs1743135	NA	UKB-b-57	TRUE	reported	8KoqAI
4	39279724	T	C	-0.0169	0.003055	3.10E-08	0.438418	462346	NA	rs1264808	NA	UKB-b-57	TRUE	reported	8KoqAI
4	39279907	A	G	0.01685	0.003055	3.50E-08	0.561461	462346	NA	rs2276888	NA	UKB-b-57	TRUE	reported	8KoqAI
4	39280655	A	G	-0.0169	0.003056	3.20E-08	0.438246	462346	NA	rs746678	NA	UKB-b-57	TRUE	reported	8KoqAI
4	39281262	G	T	-0.01678	0.003056	4.00E-08	0.438628	462346	NA	rs745642	NA	UKB-b-57	TRUE	reported	8KoqAI
4	39282106	A	G	-0.01696	0.003056	2.90E-08	0.438235	462346	NA	rs2086752	NA	UKB-b-57	TRUE	reported	8KoqAI
4	39282303	A	C	-0.01693	0.003056	3.00E-08	0.438279	462346	NA	rs2381373	NA	UKB-b-57	TRUE	reported	8KoqAI
4	39282563	A	C	0.01689	0.003056	3.20E-08	0.561426	462346	NA	rs6829660	NA	UKB-b-57	TRUE	reported	8KoqAI

4	39283146	G	T	-0.01695	0.003057	2.90E-08	0.438298	462346	NA	rs6852967	NA	UKB-b-57	TRUE	reported	8KoqAI
4	39283406	G	T	-0.01708	0.003057	2.30E-08	0.437952	462346	NA	rs6531703	NA	UKB-b-57	TRUE	reported	8KoqAI
4	39283746	T	C	-0.01694	0.003057	3.00E-08	0.438333	462346	NA	rs6531704	NA	UKB-b-57	TRUE	reported	8KoqAI
4	39283984	G	C	-0.01695	0.003058	3.00E-08	0.438213	462346	NA	rs7654501	NA	UKB-b-57	TRUE	reported	8KoqAI
4	39284265	A	G	-0.01695	0.003058	3.00E-08	0.439025	462346	NA	rs7680452	NA	UKB-b-57	TRUE	reported	8KoqAI
4	39284435	G	A	-0.01681	0.003057	3.80E-08	0.438336	462346	NA	rs7659894	NA	UKB-b-57	TRUE	reported	8KoqAI
4	39284821	A	G	-0.01728	0.003059	1.60E-08	0.441106	462346	NA	rs7686161	NA	UKB-b-57	TRUE	reported	8KoqAI
4	39285224	C	T	-0.01687	0.003062	3.60E-08	0.435844	462346	NA	rs7662880	NA	UKB-b-57	TRUE	reported	8KoqAI
4	39286093	T	C	-0.01676	0.00306	4.30E-08	0.436537	462346	NA	rs1264879	NA	UKB-b-57	TRUE	reported	8KoqAI
4	39286766	G	T	-0.01674	0.00306	4.50E-08	0.436583	462346	NA	rs2062229	NA	UKB-b-57	TRUE	reported	8KoqAI
4	39287322	C	T	-0.01675	0.00306	4.40E-08	0.436475	462346	NA	rs7564	NA	UKB-b-57	TRUE	reported	8KoqAI
4	39288353	T	G	-0.0167	0.003063	4.90E-08	0.435595	462346	NA	rs4975002	NA	UKB-b-57	TRUE	reported	8KoqAI
4	39288747	C	T	-0.0167	0.003062	4.90E-08	0.435607	462346	NA	rs1001959	NA	UKB-b-57	TRUE	reported	8KoqAI
4	39289308	G	C	0.01676	0.003066	4.60E-08	0.565958	462346	NA	rs17754	NA	UKB-b-57	TRUE	reported	8KoqAI
4	39289473	A	G	-0.01686	0.003063	3.70E-08	0.436227	462346	NA	rs1057807	NA	UKB-b-57	TRUE	reported	8KoqAI
4	39297200	G	A	-0.0208	0.003801	4.40E-08	0.202254	462346	NA	rs2306597	NA	UKB-b-57	TRUE	reported	8KoqAI
4	39302249	T	G	-0.01675	0.003065	4.60E-08	0.435583	462346	NA	rs6531709	NA	UKB-b-57	TRUE	reported	8KoqAI
4	39302462	C	T	-0.01688	0.003065	3.60E-08	0.434932	462346	NA	rs7663694	NA	UKB-b-57	TRUE	reported	8KoqAI
4	39303101	T	C	0.016831	0.003065	4.00E-08	0.564749	462346	NA	rs7693837	NA	UKB-b-57	TRUE	reported	8KoqAI
4	39312524	C	T	0.019526	0.00303	1.20E-10	0.520098	462346	NA	rs1250144	NA	UKB-b-57	TRUE	reported	8KoqAI
4	39313470	G	C	-0.01975	0.00303	7.20E-11	0.479126	462346	NA	rs4975003	NA	UKB-b-57	TRUE	reported	8KoqAI
4	39313700	T	C	-0.01975	0.00303	7.10E-11	0.479137	462346	NA	rs4975004	NA	UKB-b-57	TRUE	reported	8KoqAI
4	39313829	T	C	-0.01983	0.003031	6.10E-11	0.478686	462346	NA	rs2381375	NA	UKB-b-57	TRUE	reported	8KoqAI
4	39316791	T	C	-0.01983	0.003031	6.00E-11	0.474463	462346	NA	rs6531711	NA	UKB-b-57	TRUE	reported	8KoqAI
4	39317791	C	T	-0.0201	0.003031	3.30E-11	0.473797	462346	NA	rs1173087	NA	UKB-b-57	TRUE	reported	8KoqAI
4	39317825	G	A	-0.0199	0.003031	5.10E-11	0.474257	462346	NA	rs1172238	NA	UKB-b-57	TRUE	reported	8KoqAI
4	39319764	G	A	0.019868	0.003031	5.60E-11	0.524192	462346	NA	rs1733502	NA	UKB-b-57	TRUE	reported	8KoqAI
4	39327019	G	A	-0.0199	0.003031	5.20E-11	0.473947	462346	NA	rs6815219	NA	UKB-b-57	TRUE	reported	8KoqAI
4	39327344	G	A	-0.01987	0.003031	5.50E-11	0.473981	462346	NA	rs6815859	NA	UKB-b-57	TRUE	reported	8KoqAI
4	39329102	G	A	-0.01988	0.003031	5.40E-11	0.473993	462346	NA	rs1109699	NA	UKB-b-57	TRUE	reported	8KoqAI
4	39330296	A	T	0.019801	0.003029	6.30E-11	0.524971	462346	NA	rs6531712	NA	UKB-b-57	TRUE	reported	8KoqAI
4	39333969	C	T	-0.02043	0.003038	1.80E-11	0.473359	462346	NA	rs2859130	NA	UKB-b-57	TRUE	reported	8KoqAI
4	39334387	T	C	0.025625	0.00305	4.40E-17	0.455228	462346	NA	rs7658917	NA	UKB-b-57	TRUE	reported	8KoqAI
4	39335475	G	A	0.019836	0.003028	5.70E-11	0.524937	462346	NA	rs3796515	NA	UKB-b-57	TRUE	reported	8KoqAI
4	39337634	T	C	-0.0198	0.003028	6.10E-11	0.474429	462346	NA	rs1193971	NA	UKB-b-57	TRUE	reported	8KoqAI
4	39339400	C	A	0.019816	0.003029	6.00E-11	0.525189	462346	NA	rs1172750	NA	UKB-b-57	TRUE	reported	8KoqAI
4	39339933	T	C	0.019794	0.003029	6.40E-11	0.524964	462346	NA	rs958633	NA	UKB-b-57	TRUE	reported	8KoqAI
4	39340664	G	A	0.019837	0.003028	5.70E-11	0.52509	462346	NA	rs1109699	NA	UKB-b-57	TRUE	reported	8KoqAI
4	39343617	C	A	0.01975	0.003028	6.90E-11	0.52497	462346	NA	rs1531850	NA	UKB-b-57	TRUE	reported	8KoqAI
4	39343940	C	A	0.019823	0.003028	5.90E-11	0.524891	462346	NA	rs2306596	NA	UKB-b-57	TRUE	reported	8KoqAI
4	39344961	G	C	0.019777	0.003028	6.50E-11	0.525008	462346	NA	rs1313790	NA	UKB-b-57	TRUE	reported	8KoqAI
4	39347498	A	G	0.0198	0.003028	6.20E-11	0.525248	462346	NA	rs1173502	NA	UKB-b-57	TRUE	reported	8KoqAI
4	39351868	C	T	0.019744	0.003025	6.70E-11	0.524837	462346	NA	rs1311558	NA	UKB-b-57	TRUE	reported	8KoqAI
4	39352820	G	A	0.019902	0.003025	4.70E-11	0.524992	462346	NA	rs1109699	NA	UKB-b-57	TRUE	reported	8KoqAI
4	39355429	C	T	-0.02575	0.003049	3.00E-17	0.544771	462346	NA	rs1314357	NA	UKB-b-57	TRUE	reported	8KoqAI
4	39355434	T	C	-0.02551	0.003049	6.00E-17	0.545309	462346	NA	rs1312427	NA	UKB-b-57	TRUE	reported	8KoqAI
4	39355835	A	G	0.025485	0.003047	6.10E-17	0.454655	462346	NA	rs3542613	NA	UKB-b-57	TRUE	reported	8KoqAI
4	39361069	A	G	0.02573	0.003049	3.20E-17	0.455243	462346	NA	rs6835111	NA	UKB-b-57	TRUE	reported	8KoqAI
4	39361977	G	T	-0.02595	0.003049	1.70E-17	0.543603	462346	NA	rs4974938	NA	UKB-b-57	TRUE	reported	8KoqAI
4	39366590	T	C	0.025809	0.003054	2.90E-17	0.455499	462346	NA	rs6844176	NA	UKB-b-57	TRUE	reported	8KoqAI
4	39367654	T	C	-0.02255	0.003266	5.10E-12	0.326243	462346	NA	rs6851075	NA	UKB-b-57	TRUE	reported	8KoqAI
4	39368083	C	T	-0.0298	0.003035	9.40E-23	0.511833	462346	NA	rs3736168	NA	UKB-b-57	TRUE	reported	8KoqAI
4	39371096	C	T	0.028876	0.003035	1.80E-21	0.471796	462346	NA	rs6231077	NA	UKB-b-57	TRUE	reported	8KoqAI
4	39375329	C	T	0.028923	0.00303	1.40E-21	0.471798	462346	NA	rs6231077	NA	UKB-b-57	TRUE	reported	8KoqAI
4	39377405	C	A	0.0284	0.003027	6.40E-21	0.47276	462346	NA	rs1312378	NA	UKB-b-57	TRUE	reported	8KoqAI
4	39378458	C	T	0.028625	0.003029	3.40E-21	0.472639	462346	NA	rs6531714	NA	UKB-b-57	TRUE	reported	8KoqAI
4	39383354	T	G	0.029713	0.003034	1.20E-22	0.48122	462346	NA	rs6834187	NA	UKB-b-57	TRUE	reported	8KoqAI
4	39383810	T	C	-0.02451	0.003263	5.80E-14	0.319036	462346	NA	rs1135512	NA	UKB-b-57	TRUE	reported	8KoqAI
4	39384439	T	C	-0.02465	0.003264	4.30E-14	0.318458	462346	NA	rs1130473	NA	UKB-b-57	TRUE	reported	8KoqAI
4	39385821	C	A	0.029348	0.003035	4.10E-22	0.480465	462346	NA	rs6823497	NA	UKB-b-57	TRUE	reported	8KoqAI
4	39391940	G	A	0.029277	0.003037	5.40E-22	0.480781	462346	NA	rs1003301	NA	UKB-b-57	TRUE	reported	8KoqAI
4	39393801	C	T	-0.03112	0.003068	3.50E-24	0.568684	462346	NA	rs6842066	NA	UKB-b-57	TRUE	reported	8KoqAI
4	39395570	T	C	-0.02639	0.00316	6.80E-17	0.361494	462346	NA	rs4975011	NA	UKB-b-57	TRUE	reported	8KoqAI
4	39395630	T	A	-0.03148	0.00305	5.60E-25	0.553552	462346	NA	rs2381376	NA	UKB-b-57	TRUE	reported	8KoqAI
4	39397670	G	A	0.03096	0.003065	5.40E-24	0.427844	462346	NA	rs7662509	NA	UKB-b-57	TRUE	reported	8KoqAI
4	39398625	T	C	-0.03134	0.003046	8.00E-25	0.552366	462346	NA	rs6857705	NA	UKB-b-57	TRUE	reported	8KoqAI
4	39399404	T	C	-0.03149	0.003048	5.10E-25	0.553327	462346	NA	rs1264368	NA	UKB-b-57	TRUE	reported	8KoqAI
4	39400998	G	A	-0.03135	0.003045	7.40E-25	0.553227	462346	NA	rs1311079	NA	UKB-b-57	TRUE	reported	8KoqAI
4	39402525	C	T	-0.03115	0.003042	1.30E-24	0.551565	462346	NA	rs7661596	NA	UKB-b-57	TRUE	reported	8KoqAI
4	39402745	A	G	-0.03114	0.003042	1.30E-24	0.55156	462346	NA	rs7686103	NA	UKB-b-57	TRUE	reported	8KoqAI
4	39403243	G	A	-0.02642	0.003156	5.70E-17	0.360799	462346	NA	rs5923257	NA	UKB-b-57	TRUE	reported	8KoqAI
4	39403731	C	T	-0.03117	0.003041	1.20E-24	0.552441	462346	NA	rs3396816	NA	UKB-b-57	TRUE	reported	8KoqAI
4	39404306	C	T	-0.03112	0.003041	1.40E-24	0.551573	462346	NA	rs2381379	NA	UKB-b-57	TRUE	reported	8KoqAI
4	39404575	G	A	-0.03113	0.003041	1.40E-24	0.551562	462346	NA	rs6715298	NA	UKB-b-57	TRUE	reported	8KoqAI
4	39404872	A	G	-0.03113	0.003042	1.40E-24	0.551571	462346	NA	rs1312940	NA	UKB-b-57	TRUE	reported	8KoqAI
4	39405151	T	G	-0.03114	0.003042	1.30E-24	0.551612	462346	NA	rs1313543	NA	UKB-b-57	TRUE	reported	8KoqAI
4	39405240	A	T	-0.03117	0.003042	1.20E-24	0.551592	462346	NA	rs1313010	NA	UKB-b-57	TRUE	reported	8KoqAI
4	39406254	C	T	0.029136	0.004303	1.30E-11	0.213233	462346	NA	rs7682824	NA	UKB-b-57	TRUE	reported	8KoqAI
4	39406365	C	T	-0.02648	0.003159	5.20E-17	0.361548	462346	NA	rs1114088	NA	UKB-b-57	TRUE	reported	8KoqAI
4	39406370	G	A	-0.03115	0.003046	1.50E-24	0.5522	462346	NA	rs7686419	NA	UKB-b-57	TRUE	reported	8KoqAI
4	39406417	C	G	-0.03083	0.003044	4.20E-24	0.550013	462346	NA	rs7687811	NA	UKB-b-57	TRUE	reported	8KoqAI
4	39406490	G	A	-0.03123	0.003043	1.00E-24	0.551584	462346	NA	rs7686610	NA	UKB-b-57	TRUE	reported	8KoqAI
4	39406987	T	C	-0.03134	0.003044	7.50E-25	0.552455	462346	NA	rs1314951	NA	UKB-b-57	TRUE	reported	8KoqAI
4	39411407	C	T	0.039865	0.003776	4.60E-26	0.21185	462346	NA	rs6231081	NA	UKB-b-57	TRUE	reported	8KoqAI
4	39412384	C	A	-0.02769	0.003194	4.40E-18	0.349729	462346	NA	rs7323858	NA	UKB-b-57	TRUE	reported	8KoqAI
4	39412910	C	T	-0.02632	0.003215	2.70E-16	0.341322	462346	NA	rs1223368	NA	UKB-b-57	TRUE	reported	8KoqAI
4	39413260	C	T	0.032127	0.005129	3.80E-10	0.104481	462346	NA	rs1428035	NA	UKB-b-57	TRUE	reported	8KoqAI
4	39413650	A	T	-0.02987	0.003293	1.20E-19	0.311288	462346	NA	rs5801537	NA	UKB-b-57	TRUE	reported	8KoqAI
4	39413780	G	A	-0.04321	0.003116	1.00E-43	0.604284	462346	NA	rs2871282	NA	UKB-b-57	TRUE	reported	8KoqAI
4	39413861	A	C	-0.02784	0.003496	1.70E-15	0.256271	462346	NA	rs2046330	NA	UKB-b-57	TRUE	reported	8KoqAI
4	39414993	A	G	-0.04371	0.003116	1.00E-44	0.604193	462346	NA	rs1194069	NA	UKB-b-57	TRUE	reported	8KoqAI
4	39415355	A	T	-0.02056	0.003352	8.60E-10	0.296875	462346	NA	rs6842549	NA	UKB-b-57	TRUE	reported	8KoqAI
4	39418192	G	A	-0.0221	0.003398	7.80E-11	0.275373	462346	NA	rs6836420	NA	UKB-b-57	TRUE	reported	8KoqAI
4	39418965	G	A	0.042956	0.003142	1.50E-42	0.372521	462346	NA	rs3553805	NA	UKB-b-57	TRUE	reported	8KoqAI
4	39419229	A	T	-0.02217	0.003352	3.70E-11	0.287093	462346	NA	rs1003038	NA	UKB-b-57	TRUE	reported	8KoqAI
4	39419322	T	C	-0.02052	0.003407	1.70E-09	0.275129	462346	NA	rs1314747	NA	UKB-b-57	TRUE	reported	8KoqAI
4	39419409	T	G	-0.02265	0.003354	1.50E-11	0.285469	462346	NA	rs7674434	NA	UKB-b-57	TRUE	reported	8KoqAI
4	39420981	A	G	0.042998	0.00314	1.10E-42	0.372787	462346	NA	rs1263994	NA	UKB-b-57	TRUE	reported	8KoqAI
4	39420994	A	G	-0.02248	0.003352	2.00E-11	0.285672	462346	NA	rs9991733	NA	UKB-b-57	TRUE	reported	8KoqAI
4	39421819	G	T	-0.0218	0.003655	2.40E-09	0.223128	462346	NA	rs1215270	NA	UKB-b-57	TRUE	reported	8KoqAI
4	39422242	T	C	0.043023	0.00314	1.00E-42	0.372562	462346	NA	rs1313079	NA	UKB-b-57	TRUE	reported	8KoqAI
4	39422324	A	G	0.042978	0.003141	1.30E-42	0.372543	462346	NA	rs1312544	NA	UKB-b-57	TRUE	reported	8KoqAI
4	39423118	C	T	-0.02111	0.0034	5.30E-10	0.275017	462346	NA	rs7678241	NA	UKB-b-57	TRUE	reported	8KoqAI
4	39423512	G	A	-0.02142	0.003659	4.80E-09	0.223055	462346	NA	rs4975012	NA	UKB-b-57	TRUE	reported	8KoqAI
4	39423789	G	A	0.042819	0.003141	2.60E-42	0.371955	462346	NA	rs4975013	NA	UKB-b-57	TRUE	reported	8KoqAI
4	39424679	T	G	0.020381	0.003417	2.50E-09	0.724794	462346	NA	rs2608846	NA	UKB-b-57	TRUE	reported	8KoqAI
4	39425195	C	A	-0.02057	0.00342	1.80E-09	0.274787	462346	NA	rs7160616	NA	UKB-b-57	TRUE	reported	8KoqAI

4	39425248	A	G	0.0429	0.003127	7.80E-43	0.372034	462346	NA	rs1314690	NA	UKB-b-57	TRUE	reported	8KoqAI
4	39426325	C	T	0.029848	0.003136	1.80E-21	0.456003	462346	NA	rs6531716	NA	UKB-b-57	TRUE	reported	8KoqAI
4	39427608	C	G	-0.02557	0.004542	1.80E-08	0.128519	462346	NA	rs1135353	NA	UKB-b-57	TRUE	reported	8KoqAI
4	39428893	A	G	-0.02579	0.004536	1.30E-08	0.128394	462346	NA	rs1493486	NA	UKB-b-57	TRUE	reported	8KoqAI
4	39428974	G	A	-0.02569	0.004535	1.50E-08	0.12844	462346	NA	rs1486920	NA	UKB-b-57	TRUE	reported	8KoqAI
4	39431631	C	T	-0.02639	0.004476	3.70E-09	0.131691	462346	NA	rs1743189	NA	UKB-b-57	TRUE	reported	8KoqAI
4	39438149	G	A	0.020523	0.003364	1.10E-09	0.716811	462346	NA	rs2687963	NA	UKB-b-57	TRUE	reported	8KoqAI
4	39438504	A	G	0.019602	0.003461	1.50E-08	0.740238	462346	NA	rs2608820	NA	UKB-b-57	TRUE	reported	8KoqAI
4	39439264	T	C	-0.0244	0.003808	1.50E-10	0.19948	462346	NA	rs4975015	NA	UKB-b-57	TRUE	reported	8KoqAI
4	39439719	C	T	-0.02462	0.003813	1.10E-10	0.199215	462346	NA	rs4975016	NA	UKB-b-57	TRUE	reported	8KoqAI
4	39441490	G	A	0.01812	0.003045	2.70E-09	0.508051	462346	NA	rs1250741	NA	UKB-b-57	TRUE	reported	8KoqAI
4	39446337	T	A	0.0186	0.003231	8.60E-09	0.324703	462346	NA	rs6854452	NA	UKB-b-57	TRUE	reported	8KoqAI
4	39449406	T	C	0.01855	0.003229	9.20E-09	0.324564	462346	NA	rs1251334	NA	UKB-b-57	TRUE	reported	8KoqAI
4	39450229	C	A	0.017784	0.003227	3.60E-08	0.324793	462346	NA	rs4975017	NA	UKB-b-57	TRUE	reported	8KoqAI
4	39452124	G	A	0.018385	0.003228	1.20E-08	0.324617	462346	NA	rs1311214	NA	UKB-b-57	TRUE	reported	8KoqAI
4	39453790	A	G	0.018353	0.003226	1.30E-08	0.324669	462346	NA	rs1051752	NA	UKB-b-57	TRUE	reported	8KoqAI
4	39454293	G	A	0.018196	0.003226	1.70E-08	0.324635	462346	NA	rs6855868	NA	UKB-b-57	TRUE	reported	8KoqAI
4	39454308	G	A	0.018342	0.003226	1.30E-08	0.324722	462346	NA	rs6855886	NA	UKB-b-57	TRUE	reported	8KoqAI
4	39457617	G	A	0.018369	0.003226	1.20E-08	0.324718	462346	NA	rs1310302	NA	UKB-b-57	TRUE	reported	8KoqAI
4	39460490	C	G	0.018331	0.003228	1.40E-08	0.324704	462346	NA	rs2276890	NA	UKB-b-57	TRUE	reported	8KoqAI
4	39461973	G	A	0.017703	0.003151	1.90E-08	0.359492	462346	NA	rs3775076	NA	UKB-b-57	TRUE	reported	8KoqAI
4	39472366	A	T	0.017901	0.00315	1.30E-08	0.359759	462346	NA	rs1109699	NA	UKB-b-57	TRUE	reported	8KoqAI
4	39472842	T	A	0.017856	0.00315	1.40E-08	0.359735	462346	NA	rs2125314	NA	UKB-b-57	TRUE	reported	8KoqAI
4	39473704	C	T	-0.01803	0.00313	8.40E-09	0.374179	462346	NA	rs6805802	NA	UKB-b-57	TRUE	reported	8KoqAI
4	39473716	T	C	0.017806	0.003152	1.60E-08	0.359268	462346	NA	rs1250163	NA	UKB-b-57	TRUE	reported	8KoqAI
4	39475815	C	T	0.017947	0.00315	1.20E-08	0.359756	462346	NA	rs6837580	NA	UKB-b-57	TRUE	reported	8KoqAI
4	39478164	C	T	0.017894	0.00315	1.30E-08	0.359776	462346	NA	rs1761836	NA	UKB-b-57	TRUE	reported	8KoqAI
4	39478946	C	T	0.018018	0.00315	1.10E-08	0.359344	462346	NA	rs1193481	NA	UKB-b-57	TRUE	reported	8KoqAI
4	39481662	T	C	0.01827	0.003153	6.80E-09	0.358057	462346	NA	rs1314446	NA	UKB-b-57	TRUE	reported	8KoqAI
4	39528939	C	T	0.016775	0.003051	3.80E-08	0.445204	462346	NA	rs1314648	NA	UKB-b-57	TRUE	reported	8KoqAI
4	39533948	C	T	0.016739	0.003065	4.70E-08	0.445519	462346	NA	rs1251220	NA	UKB-b-57	TRUE	reported	8KoqAI
4	46738897	A	G	0.101376	0.018299	3.00E-08	0.007702	462346	NA	rs1404688	NA	UKB-b-57	TRUE	reported	8KoqAI
4	55088093	C	T	-0.01862	0.003368	3.20E-08	0.280364	462346	NA	rs2862222	NA	UKB-b-57	TRUE	reported	8KoqAI
4	55092626	T	C	-0.01856	0.003367	3.50E-08	0.280513	462346	NA	rs2114039	NA	UKB-b-57	TRUE	reported	8KoqAI
4	55093955	G	A	-0.01855	0.003368	3.60E-08	0.280317	462346	NA	rs6554162	NA	UKB-b-57	TRUE	reported	8KoqAI
4	55096270	A	T	-0.01853	0.003368	3.70E-08	0.28032	462346	NA	rs7668190	NA	UKB-b-57	TRUE	reported	8KoqAI
4	55097835	G	C	-0.01847	0.003368	4.10E-08	0.280307	462346	NA	rs4864504	NA	UKB-b-57	TRUE	reported	8KoqAI
4	55486128	G	A	-0.01722	0.003088	2.50E-08	0.404137	462346	NA	rs2703456	NA	UKB-b-57	TRUE	reported	8KoqAI
4	55486182	A	G	-0.01725	0.003087	2.30E-08	0.403951	462346	NA	rs7675203	NA	UKB-b-57	TRUE	reported	8KoqAI
4	55486267	C	T	-0.01753	0.003085	1.30E-08	0.404053	462346	NA	rs7697799	NA	UKB-b-57	TRUE	reported	8KoqAI
4	55486791	G	T	-0.01748	0.003082	1.40E-08	0.404026	462346	NA	rs1251289	NA	UKB-b-57	TRUE	reported	8KoqAI
4	55487511	T	C	-0.01747	0.003082	1.40E-08	0.40404	462346	NA	rs1313856	NA	UKB-b-57	TRUE	reported	8KoqAI
4	55487538	G	A	-0.01716	0.003078	2.50E-08	0.584983	462346	NA	rs2537861	NA	UKB-b-57	TRUE	reported	8KoqAI
4	55487718	C	T	-0.01683	0.003045	3.30E-08	0.550243	462346	NA	rs6815651	NA	UKB-b-57	TRUE	reported	8KoqAI
4	55487740	C	T	-0.01748	0.003082	1.40E-08	0.403954	462346	NA	rs1173049	NA	UKB-b-57	TRUE	reported	8KoqAI
4	55488643	G	A	-0.01734	0.003082	1.80E-08	0.403842	462346	NA	rs2215726	NA	UKB-b-57	TRUE	reported	8KoqAI
4	55488987	A	G	-0.0175	0.003082	1.40E-08	0.403905	462346	NA	rs1173601	NA	UKB-b-57	TRUE	reported	8KoqAI
4	55489587	T	C	-0.01752	0.003082	1.30E-08	0.403907	462346	NA	rs1249819	NA	UKB-b-57	TRUE	reported	8KoqAI
4	55491904	T	G	-0.01754	0.003081	1.20E-08	0.404174	462346	NA	rs759082	NA	UKB-b-57	TRUE	reported	8KoqAI
4	55492667	C	T	-0.01783	0.003081	7.20E-09	0.403765	462346	NA	rs7656845	NA	UKB-b-57	TRUE	reported	8KoqAI
4	55492690	C	T	-0.01741	0.003082	1.60E-08	0.403686	462346	NA	rs7656858	NA	UKB-b-57	TRUE	reported	8KoqAI
4	55492761	G	A	-0.01774	0.003081	8.40E-09	0.403733	462346	NA	rs7655556	NA	UKB-b-57	TRUE	reported	8KoqAI
4	55492816	G	T	-0.0176	0.003081	1.10E-08	0.403583	462346	NA	rs7655588	NA	UKB-b-57	TRUE	reported	8KoqAI
4	55493237	A	C	-0.01786	0.003081	6.80E-09	0.404219	462346	NA	rs7159964	NA	UKB-b-57	TRUE	reported	8KoqAI
4	55494332	A	G	-0.01757	0.003083	1.20E-08	0.403772	462346	NA	rs6851498	NA	UKB-b-57	TRUE	reported	8KoqAI
4	55495793	T	A	-0.01735	0.003047	1.20E-08	0.55583	462346	NA	rs6815910	NA	UKB-b-57	TRUE	reported	8KoqAI
4	55495948	T	G	-0.01761	0.003046	7.50E-09	0.555921	462346	NA	rs3477206	NA	UKB-b-57	TRUE	reported	8KoqAI
4	55496262	G	T	-0.0171	0.00312	4.20E-08	0.379716	462346	NA	rs1782776	NA	UKB-b-57	TRUE	reported	8KoqAI
4	55497167	C	T	-0.0174	0.003045	1.10E-08	0.555221	462346	NA	rs1314007	NA	UKB-b-57	TRUE	reported	8KoqAI
4	55497446	T	A	-0.01715	0.003116	3.70E-08	0.380582	462346	NA	rs1312117	NA	UKB-b-57	TRUE	reported	8KoqAI
4	55497921	C	G	-0.0175	0.003045	9.10E-09	0.555411	462346	NA	rs1173607	NA	UKB-b-57	TRUE	reported	8KoqAI
4	55498515	G	T	-0.01808	0.003026	2.30E-09	0.486849	462346	NA	rs7694444	NA	UKB-b-57	TRUE	reported	8KoqAI
4	55499090	T	C	-0.01783	0.003074	6.60E-09	0.585626	462346	NA	rs2537863	NA	UKB-b-57	TRUE	reported	8KoqAI
4	55499386	C	G	-0.01809	0.003025	2.20E-09	0.486953	462346	NA	rs7655709	NA	UKB-b-57	TRUE	reported	8KoqAI
4	55499537	C	T	-0.0181	0.003025	2.20E-09	0.486973	462346	NA	rs7656088	NA	UKB-b-57	TRUE	reported	8KoqAI
4	55499751	T	C	-0.01747	0.003044	9.40E-09	0.555809	462346	NA	rs6554193	NA	UKB-b-57	TRUE	reported	8KoqAI
4	55499810	T	G	-0.01747	0.003044	9.40E-09	0.555838	462346	NA	rs6554194	NA	UKB-b-57	TRUE	reported	8KoqAI
4	55500714	G	T	-0.018	0.003026	2.70E-09	0.486064	462346	NA	rs6554195	NA	UKB-b-57	TRUE	reported	8KoqAI
4	55501109	C	G	-0.01713	0.003113	3.70E-08	0.380968	462346	NA	rs6554196	NA	UKB-b-57	TRUE	reported	8KoqAI
4	55501195	C	T	-0.01797	0.003071	4.90E-09	0.585019	462346	NA	rs6554197	NA	UKB-b-57	TRUE	reported	8KoqAI
4	55501728	A	G	-0.01804	0.003023	2.40E-09	0.487508	462346	NA	rs1310683	NA	UKB-b-57	TRUE	reported	8KoqAI
4	55501921	A	C	-0.01803	0.003071	4.40E-09	0.585219	462346	NA	rs1172232	NA	UKB-b-57	TRUE	reported	8KoqAI
4	55502503	T	C	-0.01779	0.003069	6.80E-09	0.582818	462346	NA	rs4864913	NA	UKB-b-57	TRUE	reported	8KoqAI
4	55503194	A	G	-0.018	0.003024	2.60E-09	0.485046	462346	NA	rs723585	NA	UKB-b-57	TRUE	reported	8KoqAI
4	55503813	G	A	-0.0171	0.003114	4.00E-08	0.380537	462346	NA	rs1782797	NA	UKB-b-57	TRUE	reported	8KoqAI
4	55505360	A	G	-0.01734	0.003041	1.20E-08	0.553282	462346	NA	rs2192158	NA	UKB-b-57	TRUE	reported	8KoqAI
4	55505529	C	G	-0.01739	0.003041	1.10E-08	0.553061	462346	NA	rs2192159	NA	UKB-b-57	TRUE	reported	8KoqAI
4	55505911	C	T	-0.01718	0.003041	1.60E-08	0.552742	462346	NA	rs1249936	NA	UKB-b-57	TRUE	reported	8KoqAI
4	55505933	T	A	-0.01721	0.003042	1.50E-08	0.552741	462346	NA	rs1250413	NA	UKB-b-57	TRUE	reported	8KoqAI
4	55508664	G	A	-0.0179	0.003074	5.80E-09	0.586863	462346	NA	rs7679314	NA	UKB-b-57	TRUE	reported	8KoqAI
4	55509189	G	A	-0.01812	0.003026	2.10E-09	0.488792	462346	NA	rs7684939	NA	UKB-b-57	TRUE	reported	8KoqAI
4	55509748	C	T	-0.0174	0.003044	1.10E-08	0.55716	462346	NA	rs1172727	NA	UKB-b-57	TRUE	reported	8KoqAI
4	55510862	C	T	-0.01744	0.003044	1.00E-08	0.558308	462346	NA	rs1048884	NA	UKB-b-57	TRUE	reported	8KoqAI
4	55511498	G	A	-0.01687	0.003042	2.90E-08	0.556254	462346	NA	rs1194073	NA	UKB-b-57	TRUE	reported	8KoqAI
4	55512066	T	C	-0.01688	0.003042	2.90E-08	0.556547	462346	NA	rs7695300	NA	UKB-b-57	TRUE	reported	8KoqAI
4	55515210	A	G	-0.01712	0.003043	1.90E-08	0.556966	462346	NA	rs6825293	NA	UKB-b-57	TRUE	reported	8KoqAI
4	55517506	G	T	-0.01713	0.003108	3.60E-08	0.392018	462346	NA	rs3544140	NA	UKB-b-57	TRUE	reported	8KoqAI
4	55518877	T	A	-0.01719	0.003111	3.30E-08	0.385719	462346	NA	rs7694422	NA	UKB-b-57	TRUE	reported	8KoqAI
4	55518878	C	A	-0.01719	0.003111	3.30E-08	0.385719	462346	NA	rs7665611	NA	UKB-b-57	TRUE	reported	8KoqAI
4	55521017	G	A	-0.01857	0.003026	8.30E-10	0.490369	462346	NA	rs2159935	NA	UKB-b-57	TRUE	reported	8KoqAI
4	55522160	G	A	-0.01792	0.003074	5.60E-09	0.588212	462346	NA	rs6554198	NA	UKB-b-57	TRUE	reported	8KoqAI
4	55522488	G	T	-0.01775	0.003044	5.50E-09	0.558496	462346	NA	rs6554199	NA	UKB-b-57	TRUE	reported	8KoqAI
4	55523938	G	A	-0.01736	0.003109	2.40E-08	0.386023	462346	NA	rs1005438	NA	UKB-b-57	TRUE	reported	8KoqAI
4	55524304	T	C	-0.01776	0.003044	5.40E-09	0.558953	462346	NA	rs999020	NA	UKB-b-57	TRUE	reported	8KoqAI
4	55524533	C	G	-0.01765	0.003045	6.80E-09	0.559049	462346	NA	rs999021	NA	UKB-b-57	TRUE	reported	8KoqAI
4	55524733	G	T	-0.01738	0.003109	2.30E-08	0.386029	462346	NA	rs3822213	NA	UKB-b-57	TRUE	reported	8KoqAI
4	55525080	C	T	-0.01734	0.003109	2.40E-08	0.38609	462346	NA	rs3756197	NA	UKB-b-57	TRUE	reported	8KoqAI
4	55525153	A	G	-0.01782	0.003075	6.80E-09	0.588642	462346	NA	rs2855778	NA	UKB-b-57	TRUE	reported	8KoqAI
4	55525833	T	C	-0.01774	0.003044	5.60E-09	0.558836	462346	NA	rs2237038	NA	UKB-b-57	TRUE	reported	8KoqAI
4	55525930	A	C	-0.01776	0.003043	5.30E-09	0.558843	462346	NA	rs2237037	NA	UKB-b-57	TRUE	reported	8KoqAI
4	55526251	G	T	-0.01744	0.003109	2.00E-08	0.386047	462346	NA	rs2237035	NA	UKB-b-57	TRUE	reported	8KoqAI
4	55526615	C	T	-0.01772	0.003043	5.80E-09	0.559034	462346	NA	rs3819393	NA	UKB-b-57	TRUE	reported	8KoqAI
4	55526694	G	A	-0.01718	0.003108	3.20E-08	0.386482	462346	NA	rs3819392	NA	UKB-b-57	TRUE	reported	8KoqAI
4	55526702	A	G	-0.01773	0.003044	5.70E-09	0.559069	462346	NA	rs3819391	NA	UKB-b-57	TRUE	reported	8KoqAI
4	55526920	G	T	-0.0172	0.003108	3.20E-08	0.386469	462346	NA	rs3819389	NA	UKB-b-57	TRUE	reported	8KoqAI
4	55526962	G	T	-0.0177	0.003043	6.00E-09	0.559033	462346	NA	rs981959	NA	UKB-b-57	TRUE	reported	8KoqAI
4	55527065	A	G	-0.01722	0.003108	3.00E-08	0.386452	462346	NA	rs3819387	NA	UKB-b-57	TRUE	reported	8KoqAI

4	55527374	T	C	-0.01774	0.003044	5.60E-09	0.559022	462346	NA	rs3819386	NA	UKB-b-57	TRUE	reported	8KoqAI
4	55529409	G	C	-0.0177	0.003046	6.20E-09	0.559191	462346	NA	rs6554200	NA	UKB-b-57	TRUE	reported	8KoqAI
4	55530881	C	T	-0.01728	0.003112	2.80E-08	0.386679	462346	NA	rs1173335	NA	UKB-b-57	TRUE	reported	8KoqAI
4	55531680	C	T	-0.01728	0.003113	2.90E-08	0.386671	462346	NA	rs4864915	NA	UKB-b-57	TRUE	reported	8KoqAI
4	55532448	T	A	-0.01736	0.003115	2.50E-08	0.386642	462346	NA	rs2237031	NA	UKB-b-57	TRUE	reported	8KoqAI
4	55532548	T	A	-0.01778	0.003051	5.60E-09	0.558982	462346	NA	rs2237030	NA	UKB-b-57	TRUE	reported	8KoqAI
4	55533116	C	T	-0.01736	0.003116	2.50E-08	0.386637	462346	NA	rs2282781	NA	UKB-b-57	TRUE	reported	8KoqAI
4	55533551	T	G	-0.0175	0.003117	2.00E-08	0.386852	462346	NA	rs2282780	NA	UKB-b-57	TRUE	reported	8KoqAI
4	55533583	G	A	-0.01754	0.003117	1.80E-08	0.386844	462346	NA	rs2282779	NA	UKB-b-57	TRUE	reported	8KoqAI
4	55533621	A	T	-0.01753	0.003117	1.90E-08	0.386852	462346	NA	rs2282778	NA	UKB-b-57	TRUE	reported	8KoqAI
4	55534933	A	G	-0.01742	0.003118	2.30E-08	0.388468	462346	NA	rs3428486	NA	UKB-b-57	TRUE	reported	8KoqAI
4	55535316	A	T	-0.01731	0.003085	2.00E-08	0.418855	462346	NA	rs7684000	NA	UKB-b-57	TRUE	reported	8KoqAI
4	55535336	G	A	-0.01761	0.003107	1.50E-08	0.601413	462346	NA	rs2237029	NA	UKB-b-57	TRUE	reported	8KoqAI
4	55536375	T	G	-0.0181	0.00307	3.70E-09	0.439695	462346	NA	rs2237028	NA	UKB-b-57	TRUE	reported	8KoqAI
4	55537014	T	C	-0.0178	0.003056	5.80E-09	0.469412	462346	NA	rs2017472	NA	UKB-b-57	TRUE	reported	8KoqAI
4	55537481	C	G	-0.01786	0.003123	1.10E-08	0.390521	462346	NA	rs759083	NA	UKB-b-57	TRUE	reported	8KoqAI
4	55538347	G	C	-0.01796	0.003125	9.00E-09	0.390461	462346	NA	rs1312885	NA	UKB-b-57	TRUE	reported	8KoqAI
4	55538880	T	C	-0.0178	0.003075	7.00E-09	0.439438	462346	NA	rs7663318	NA	UKB-b-57	TRUE	reported	8KoqAI
4	55539253	A	G	-0.01741	0.003068	1.40E-08	0.453859	462346	NA	rs2237026	NA	UKB-b-57	TRUE	reported	8KoqAI
4	55539264	C	T	-0.01747	0.003068	1.20E-08	0.45416	462346	NA	rs1250346	NA	UKB-b-57	TRUE	reported	8KoqAI
4	55541879	T	C	-0.0185	0.003072	1.70E-09	0.555476	462346	NA	rs2237025	NA	UKB-b-57	TRUE	reported	8KoqAI
4	55546998	A	G	-0.01773	0.003052	6.30E-09	0.551011	462346	NA	rs2537866	NA	UKB-b-57	TRUE	reported	8KoqAI
4	55547380	T	C	-0.01781	0.003052	5.40E-09	0.55118	462346	NA	rs2855770	NA	UKB-b-57	TRUE	reported	8KoqAI
4	55548416	T	C	-0.01807	0.003051	3.10E-09	0.551396	462346	NA	rs2703464	NA	UKB-b-57	TRUE	reported	8KoqAI
4	55549124	A	G	-0.01802	0.00305	3.50E-09	0.55098	462346	NA	rs2537867	NA	UKB-b-57	TRUE	reported	8KoqAI
4	55554155	G	A	-0.01696	0.003025	2.10E-08	0.487979	462346	NA	rs2855777	NA	UKB-b-57	TRUE	reported	8KoqAI
4	55554331	T	C	-0.01663	0.003025	3.80E-08	0.487683	462346	NA	rs2703488	NA	UKB-b-57	TRUE	reported	8KoqAI
4	55556040	A	C	-0.01813	0.003044	2.60E-09	0.551135	462346	NA	rs2703475	NA	UKB-b-57	TRUE	reported	8KoqAI
4	55557984	A	G	-0.01805	0.003045	3.10E-09	0.551055	462346	NA	rs2237023	NA	UKB-b-57	TRUE	reported	8KoqAI
4	55559314	T	C	-0.01807	0.003045	3.00E-09	0.551094	462346	NA	rs2703461	NA	UKB-b-57	TRUE	reported	8KoqAI
4	55559563	G	C	-0.01812	0.003046	2.70E-09	0.550978	462346	NA	rs6810872	NA	UKB-b-57	TRUE	reported	8KoqAI
4	55561067	T	C	-0.0177	0.00303	5.10E-09	0.500041	462346	NA	rs2237021	NA	UKB-b-57	TRUE	reported	8KoqAI
4	55563462	A	G	-0.01769	0.00303	5.30E-09	0.501189	462346	NA	rs2282775	NA	UKB-b-57	TRUE	reported	8KoqAI
4	55563683	T	G	-0.01769	0.00303	5.30E-09	0.501204	462346	NA	rs2872966	NA	UKB-b-57	TRUE	reported	8KoqAI
4	55564049	C	T	-0.01686	0.003032	2.70E-08	0.486005	462346	NA	rs3433276	NA	UKB-b-57	TRUE	reported	8KoqAI
4	55566266	G	A	0.016572	0.003034	4.70E-08	0.516608	462346	NA	rs3134884	NA	UKB-b-57	TRUE	reported	8KoqAI
4	55567888	A	C	0.018333	0.003057	2.00E-09	0.439775	462346	NA	rs3134885	NA	UKB-b-57	TRUE	reported	8KoqAI
4	55568229	A	G	0.016889	0.003034	2.60E-08	0.513275	462346	NA	rs3111795	NA	UKB-b-57	TRUE	reported	8KoqAI
4	55568539	G	T	-0.01694	0.003034	2.40E-08	0.485732	462346	NA	rs5629503	NA	UKB-b-57	TRUE	reported	8KoqAI
4	55569146	T	C	0.018314	0.003057	2.10E-09	0.439794	462346	NA	rs3020825	NA	UKB-b-57	TRUE	reported	8KoqAI
4	55571441	C	T	0.018347	0.003058	2.00E-09	0.439477	462346	NA	rs3111800	NA	UKB-b-57	TRUE	reported	8KoqAI
4	55573693	G	A	-0.01775	0.003036	5.10E-09	0.499862	462346	NA	rs1724005	NA	UKB-b-57	TRUE	reported	8KoqAI
4	55573935	G	A	-0.01782	0.003037	4.40E-09	0.49991	462346	NA	rs2237020	NA	UKB-b-57	TRUE	reported	8KoqAI
4	55574182	C	T	0.017749	0.003037	5.10E-09	0.499032	462346	NA	rs3020826	NA	UKB-b-57	TRUE	reported	8KoqAI
4	55574930	G	A	0.018226	0.003062	2.70E-09	0.439212	462346	NA	rs2237019	NA	UKB-b-57	TRUE	reported	8KoqAI
4	55579081	G	A	0.017865	0.003072	6.00E-09	0.440088	462346	NA	rs3020827	NA	UKB-b-57	TRUE	reported	8KoqAI
4	55579484	T	A	-0.01774	0.003072	7.80E-09	0.559138	462346	NA	rs4864919	NA	UKB-b-57	TRUE	reported	8KoqAI
4	97187643	G	A	0.196158	0.031756	6.50E-10	0.00237	462346	NA	rs1891857	NA	UKB-b-57	TRUE	reported	8KoqAI
4	97191460	G	A	0.195999	0.031645	5.90E-10	0.002378	462346	NA	rs1490050	NA	UKB-b-57	TRUE	reported	8KoqAI
4	97264066	C	G	0.199828	0.031433	2.10E-10	0.002447	462346	NA	rs1907072	NA	UKB-b-57	TRUE	reported	8KoqAI
4	97497119	G	A	0.21302	0.033285	1.60E-10	0.002236	462346	NA	rs1810041	NA	UKB-b-57	TRUE	reported	8KoqAI
4	98373818	C	T	0.159901	0.022696	1.80E-12	0.004617	462346	NA	rs1434752	NA	UKB-b-57	TRUE	reported	8KoqAI
4	98394739	C	T	0.185283	0.024585	4.80E-14	0.003995	462346	NA	rs1382837	NA	UKB-b-57	TRUE	reported	8KoqAI
4	98452892	T	A	0.180246	0.025216	8.80E-13	0.003913	462346	NA	rs1445028	NA	UKB-b-57	TRUE	reported	8KoqAI
4	98744985	T	A	0.180137	0.024826	4.00E-13	0.004005	462346	NA	rs1509558	NA	UKB-b-57	TRUE	reported	8KoqAI
4	98748279	C	G	0.171091	0.023289	2.00E-13	0.004459	462346	NA	rs1382946	NA	UKB-b-57	TRUE	reported	8KoqAI
4	98979582	C	G	0.22236	0.03803	5.00E-09	0.001747	462346	NA	rs1727284	NA	UKB-b-57	TRUE	reported	8KoqAI
4	99161352	A	G	0.187043	0.025162	1.10E-13	0.003911	462346	NA	rs1401847	NA	UKB-b-57	TRUE	reported	8KoqAI
4	99465096	A	G	0.185671	0.021975	2.90E-17	0.005398	462346	NA	rs1430929	NA	UKB-b-57	TRUE	reported	8KoqAI
4	99557766	T	A	-0.20823	0.018783	1.50E-28	0.992548	462346	NA	rs846004	NA	UKB-b-57	TRUE	reported	8KoqAI
4	99567226	C	T	-0.20709	0.018544	5.90E-29	0.99236	462346	NA	rs1919208	NA	UKB-b-57	TRUE	reported	8KoqAI
4	99567918	T	C	0.222367	0.017667	2.50E-36	0.009037	462346	NA	rs1140262	NA	UKB-b-57	TRUE	reported	8KoqAI
4	99572723	G	T	-0.26238	0.02024	2.00E-38	0.993953	462346	NA	rs4699663	NA	UKB-b-57	TRUE	reported	8KoqAI
4	99610593	T	A	0.065583	0.008965	2.60E-13	0.030357	462346	NA	rs6232546	NA	UKB-b-57	TRUE	reported	8KoqAI
4	99630189	G	C	0.048941	0.008462	7.30E-09	0.034925	462346	NA	rs6232546	NA	UKB-b-57	TRUE	reported	8KoqAI
4	99644166	C	T	0.217044	0.016858	6.30E-38	0.008873	462346	NA	rs6232547	NA	UKB-b-57	TRUE	reported	8KoqAI
4	99653424	T	A	0.047655	0.008441	1.60E-08	0.035072	462346	NA	rs2866019	NA	UKB-b-57	TRUE	reported	8KoqAI
4	99686494	A	G	0.046573	0.007871	3.30E-09	0.03898	462346	NA	rs2178126	NA	UKB-b-57	TRUE	reported	8KoqAI
4	99687738	A	C	0.043086	0.007349	4.60E-09	0.044459	462346	NA	rs1406034	NA	UKB-b-57	TRUE	reported	8KoqAI
4	99689210	C	T	0.043433	0.007303	2.70E-09	0.044989	462346	NA	rs3467374	NA	UKB-b-57	TRUE	reported	8KoqAI
4	99689987	T	C	0.043395	0.007303	2.80E-09	0.044985	462346	NA	rs1852627	NA	UKB-b-57	TRUE	reported	8KoqAI
4	99690466	T	C	0.043494	0.007303	2.60E-09	0.044984	462346	NA	rs3402016	NA	UKB-b-57	TRUE	reported	8KoqAI
4	99691047	G	A	0.052746	0.006659	2.30E-15	0.059643	462346	NA	rs7161265	NA	UKB-b-57	TRUE	reported	8KoqAI
4	99691805	T	C	0.042273	0.007301	7.00E-09	0.045017	462346	NA	rs3410848	NA	UKB-b-57	TRUE	reported	8KoqAI
4	99694550	G	A	0.043591	0.007304	2.40E-09	0.044994	462346	NA	rs3524844	NA	UKB-b-57	TRUE	reported	8KoqAI
4	99696147	T	C	0.042566	0.007314	5.90E-09	0.04517	462346	NA	rs3441763	NA	UKB-b-57	TRUE	reported	8KoqAI
4	99698611	G	A	0.044586	0.007293	9.80E-10	0.045179	462346	NA	rs2866025	NA	UKB-b-57	TRUE	reported	8KoqAI
4	99699536	G	A	0.044722	0.007293	8.70E-10	0.045195	462346	NA	rs3413993	NA	UKB-b-57	TRUE	reported	8KoqAI
4	99702014	C	T	0.048748	0.007842	5.10E-10	0.039207	462346	NA	rs2866027	NA	UKB-b-57	TRUE	reported	8KoqAI
4	99703130	A	G	0.044271	0.007366	1.90E-09	0.044539	462346	NA	rs3519707	NA	UKB-b-57	TRUE	reported	8KoqAI
4	99704167	G	A	0.260946	0.018426	1.60E-45	0.007078	462346	NA	rs2141284	NA	UKB-b-57	TRUE	reported	8KoqAI
4	99712384	A	G	0.084484	0.009888	1.30E-17	0.024253	462346	NA	rs3420060	NA	UKB-b-57	TRUE	reported	8KoqAI
4	99713350	C	T	0.25317	0.014869	5.20E-65	0.011535	462346	NA	rs1441987	NA	UKB-b-57	TRUE	reported	8KoqAI
4	99713468	G	A	0.060351	0.007521	1.00E-15	0.042995	462346	NA	rs1702811	NA	UKB-b-57	TRUE	reported	8KoqAI
4	99714547	C	G	0.061314	0.007629	9.20E-16	0.041573	462346	NA	rs1822529	NA	UKB-b-57	TRUE	reported	8KoqAI
4	99714600	C	A	0.060108	0.00753	1.40E-15	0.042849	462346	NA	rs1373245	NA	UKB-b-57	TRUE	reported	8KoqAI
4	99717473	C	T	0.061363	0.007572	5.30E-16	0.042312	462346	NA	rs6835829	NA	UKB-b-57	TRUE	reported	8KoqAI
4	99717970	A	G	0.05991	0.00752	1.60E-15	0.042915	462346	NA	rs6836719	NA	UKB-b-57	TRUE	reported	8KoqAI
4	99718844	C	T	0.060969	0.007573	8.20E-16	0.042209	462346	NA	rs3519712	NA	UKB-b-57	TRUE	reported	8KoqAI
4	99719961	T	C	0.060423	0.007526	9.90E-16	0.042678	462346	NA	rs1867343	NA	UKB-b-57	TRUE	reported	8KoqAI
4	99721136	T	A	0.062751	0.007617	1.70E-16	0.041352	462346	NA	rs3568003	NA	UKB-b-57	TRUE	reported	8KoqAI
4	99721336	T	A	0.062562	0.007583	1.60E-16	0.042223	462346	NA	rs3406714	NA	UKB-b-57	TRUE	reported	8KoqAI
4	99721406	T	C	0.061818	0.007601	4.20E-16	0.041512	462346	NA	rs6232369	NA	UKB-b-57	TRUE	reported	8KoqAI
4	99721909	C	T	0.061904	0.007605	3.90E-16	0.041467	462346	NA	rs1373244	NA	UKB-b-57	TRUE	reported	8KoqAI
4	99723696	T	C	0.061984	0.007604	3.60E-16	0.041463	462346	NA	rs1264163	NA	UKB-b-57	TRUE	reported	8KoqAI
4	99726315	C	T	0.06225	0.007621	3.10E-16	0.041262	462346	NA	rs1918741	NA	UKB-b-57	TRUE	reported	8KoqAI
4	99727222	C	A	0.062322	0.007621	2.90E-16	0.041266	462346	NA	rs1436538	NA	UKB-b-57	TRUE	reported	8KoqAI
4	99728276	G	A	0.062226	0.007606	2.80E-16	0.041426	462346	NA	rs1264957	NA	UKB-b-57	TRUE	reported	8KoqAI
4	99728473	A	G	0.062228	0.007606	2.80E-16	0.041426	462346	NA	rs3470022	NA	UKB-b-57	TRUE	reported	8KoqAI
4	99729736	G	A	0.062256	0.007606	2.70E-16	0.041416	462346	NA	rs6232370	NA	UKB-b-57	TRUE	reported	8KoqAI
4	99730024	C	G	0.062236	0.007617	3.10E-16	0.041285	462346	NA	rs5603170	NA	UKB-b-57	TRUE	reported	8KoqAI
4	99731866	G	C	0.062333	0.007616	2.70E-16	0.041293	462346	NA	rs1264079	NA	UKB-b-57	TRUE	reported	8KoqAI
4	99734532	C	A	0.062368	0.007604	2.40E-16	0.04143	462346	NA	rs3537445	NA	UKB-b-57	TRUE	reported	8KoqAI
4	99737453	T	G	-0.06234	0.007607	2.50E-16	0.958623	462346	NA	rs1596416	NA	UKB-b-57	TRUE	reported	8KoqAI
4	99737796	T	A	-0.06223	0.007595	2.50E-16	0.958485	462346	NA	rs1838231	NA	UKB-b-57	TRUE	reported	8KoqAI
4	99739125	T	C	-0.06222	0.007595	2.60E-16	0.958485	462346	NA	rs1596415	NA	UKB-b-57	TRUE	reported	8KoqAI
4	99739151	C	T	-0.06241	0.007608	2.30E-16	0.958622	462346	NA	rs1838230	NA	UKB-b-57	TRUE	reported	8KoqAI

4	99743185	C	T	-0.0631	0.007588	9.10E-17	0.958388	462346	NA	rs1109763	NA	UKB-b-57	TRUE	reported	8KoqAI
4	99743255	C	T	-0.06265	0.007591	1.50E-16	0.95844	462346	NA	rs1109763	NA	UKB-b-57	TRUE	reported	8KoqAI
4	99743545	A	G	-0.06216	0.007594	2.70E-16	0.958498	462346	NA	rs1233101	NA	UKB-b-57	TRUE	reported	8KoqAI
4	99743724	A	T	-0.06216	0.007595	2.70E-16	0.958518	462346	NA	rs1562035	NA	UKB-b-57	TRUE	reported	8KoqAI
4	99745975	T	C	-0.06227	0.007594	2.40E-16	0.958422	462346	NA	rs4235431	NA	UKB-b-57	TRUE	reported	8KoqAI
4	99748968	G	T	-0.06245	0.007608	2.20E-16	0.958759	462346	NA	rs6843891	NA	UKB-b-57	TRUE	reported	8KoqAI
4	99750252	G	A	-0.06261	0.007601	1.80E-16	0.958712	462346	NA	rs1596414	NA	UKB-b-57	TRUE	reported	8KoqAI
4	99751794	A	G	0.259467	0.015713	2.90E-61	0.010642	462346	NA	rs1454412	NA	UKB-b-57	TRUE	reported	8KoqAI
4	99759132	G	A	-0.06268	0.007624	2.00E-16	0.958843	462346	NA	rs4699680	NA	UKB-b-57	TRUE	reported	8KoqAI
4	99773191	T	G	-0.04684	0.006437	3.40E-13	0.940804	462346	NA	rs1160489	NA	UKB-b-57	TRUE	reported	8KoqAI
4	99775406	G	A	-0.04653	0.006441	5.10E-13	0.940788	462346	NA	rs7665186	NA	UKB-b-57	TRUE	reported	8KoqAI
4	99779875	C	G	-0.04764	0.006456	1.60E-13	0.940592	462346	NA	rs961757	NA	UKB-b-57	TRUE	reported	8KoqAI
4	99780391	C	T	-0.0475	0.006468	2.10E-13	0.940771	462346	NA	rs6532769	NA	UKB-b-57	TRUE	reported	8KoqAI
4	99781025	T	C	-0.04723	0.006488	3.40E-13	0.941212	462346	NA	rs7677046	NA	UKB-b-57	TRUE	reported	8KoqAI
4	99808693	T	C	-0.05426	0.008008	1.20E-11	0.961034	462346	NA	rs1813867	NA	UKB-b-57	TRUE	reported	8KoqAI
4	99941138	A	G	0.180684	0.011811	7.80E-53	0.017786	462346	NA	rs1467880	NA	UKB-b-57	TRUE	reported	8KoqAI
4	99994405	T	C	0.295431	0.018362	3.00E-58	0.007626	462346	NA	rs2900157	NA	UKB-b-57	TRUE	reported	8KoqAI
4	1E+08	G	C	0.048512	0.007363	4.40E-11	0.047216	462346	NA	rs2900120	NA	UKB-b-57	TRUE	reported	8KoqAI
4	1E+08	G	A	0.252391	0.046129	4.50E-08	0.00118	462346	NA	rs1880407	NA	UKB-b-57	TRUE	reported	8KoqAI
4	1E+08	C	T	-0.07744	0.013929	2.70E-08	0.013176	462346	NA	rs1847501	NA	UKB-b-57	TRUE	reported	8KoqAI
4	1E+08	T	C	0.149613	0.011047	8.70E-42	0.021322	462346	NA	rs1162034	NA	UKB-b-57	TRUE	reported	8KoqAI
4	1E+08	A	T	0.240574	0.044136	5.00E-08	0.001246	462346	NA	rs1483296	NA	UKB-b-57	TRUE	reported	8KoqAI
4	1E+08	G	A	0.287557	0.017201	9.70E-63	0.00816	462346	NA	rs1173369	NA	UKB-b-57	TRUE	reported	8KoqAI
4	1E+08	A	G	0.054372	0.007378	1.70E-13	0.045534	462346	NA	rs1154431	NA	UKB-b-57	TRUE	reported	8KoqAI
4	1E+08	T	C	-0.26171	0.009185	1.40E-178	0.97277	462346	NA	rs1229984	NA	UKB-b-57	TRUE	reported	8KoqAI
4	1E+08	A	G	-0.02986	0.003085	3.70E-22	0.448157	462346	NA	rs3811802	NA	UKB-b-57	TRUE	reported	8KoqAI
4	1E+08	C	A	-0.03908	0.004005	1.70E-22	0.172909	462346	NA	rs2213041	NA	UKB-b-57	TRUE	reported	8KoqAI
4	1E+08	A	G	-0.04649	0.005103	8.30E-20	0.097735	462346	NA	rs7834655	NA	UKB-b-57	TRUE	reported	8KoqAI
4	1E+08	G	A	-0.03903	0.004005	1.90E-22	0.173004	462346	NA	rs1789889	NA	UKB-b-57	TRUE	reported	8KoqAI
4	1E+08	G	C	0.260632	0.013608	9.20E-82	0.012444	462346	NA	rs1454527	NA	UKB-b-57	TRUE	reported	8KoqAI
4	1E+08	A	T	-0.03904	0.004004	1.90E-22	0.172903	462346	NA	rs2226898	NA	UKB-b-57	TRUE	reported	8KoqAI
4	1E+08	G	A	0.260654	0.013619	1.20E-81	0.012543	462346	NA	rs1384959	NA	UKB-b-57	TRUE	reported	8KoqAI
4	1E+08	G	A	-0.039	0.004004	2.00E-22	0.172894	462346	NA	rs1789890	NA	UKB-b-57	TRUE	reported	8KoqAI
4	1E+08	C	A	-0.03898	0.004004	2.10E-22	0.172875	462346	NA	rs1789891	NA	UKB-b-57	TRUE	reported	8KoqAI
4	1E+08	G	A	-0.03273	0.003064	1.20E-26	0.416053	462346	NA	rs1789892	NA	UKB-b-57	TRUE	reported	8KoqAI
4	1E+08	C	A	0.023401	0.003635	1.20E-10	0.226374	462346	NA	rs2173201	NA	UKB-b-57	TRUE	reported	8KoqAI
4	1E+08	G	T	0.062588	0.006199	5.70E-24	0.063525	462346	NA	rs1136590	NA	UKB-b-57	TRUE	reported	8KoqAI
4	1E+08	T	A	-0.03913	0.004004	1.50E-22	0.172917	462346	NA	rs1229863	NA	UKB-b-57	TRUE	reported	8KoqAI
4	1E+08	T	C	-0.0464	0.004432	1.20E-25	0.865652	462346	NA	rs3114045	NA	UKB-b-57	TRUE	reported	8KoqAI
4	1E+08	G	A	-0.03913	0.004004	1.50E-22	0.172928	462346	NA	rs1154432	NA	UKB-b-57	TRUE	reported	8KoqAI
4	1E+08	A	G	-0.03269	0.003063	1.40E-26	0.416154	462346	NA	rs1154433	NA	UKB-b-57	TRUE	reported	8KoqAI
4	1E+08	G	A	-0.03916	0.004004	1.40E-22	0.172923	462346	NA	rs1154430	NA	UKB-b-57	TRUE	reported	8KoqAI
4	1E+08	C	T	-0.01788	0.003246	3.60E-08	0.317931	462346	NA	rs1229979	NA	UKB-b-57	TRUE	reported	8KoqAI
4	1E+08	T	C	-0.03273	0.003064	1.20E-26	0.415547	462346	NA	rs1229978	NA	UKB-b-57	TRUE	reported	8KoqAI
4	1E+08	C	G	-0.01789	0.003246	3.60E-08	0.317933	462346	NA	rs1789895	NA	UKB-b-57	TRUE	reported	8KoqAI
4	1E+08	A	G	-0.03274	0.003064	1.20E-26	0.41555	462346	NA	rs1662031	NA	UKB-b-57	TRUE	reported	8KoqAI
4	1E+08	G	A	0.025197	0.003022	7.60E-17	0.513313	462346	NA	rs1789896	NA	UKB-b-57	TRUE	reported	8KoqAI
4	1E+08	T	C	-0.04664	0.005103	6.30E-20	0.0976	462346	NA	rs2298753	NA	UKB-b-57	TRUE	reported	8KoqAI
4	1E+08	T	C	-0.03273	0.003064	1.30E-26	0.415503	462346	NA	rs1612735	NA	UKB-b-57	TRUE	reported	8KoqAI
4	1E+08	A	C	-0.03273	0.003064	1.20E-26	0.415523	462346	NA	rs1789898	NA	UKB-b-57	TRUE	reported	8KoqAI
4	1E+08	T	G	-0.01786	0.003246	3.70E-08	0.317922	462346	NA	rs1662033	NA	UKB-b-57	TRUE	reported	8KoqAI
4	1E+08	T	C	-0.01786	0.003246	3.70E-08	0.317927	462346	NA	rs1693454	NA	UKB-b-57	TRUE	reported	8KoqAI
4	1E+08	T	G	-0.04664	0.005104	6.40E-20	0.097582	462346	NA	rs3910713	NA	UKB-b-57	TRUE	reported	8KoqAI
4	1E+08	T	C	-0.03272	0.003064	1.30E-26	0.415445	462346	NA	rs1442479	NA	UKB-b-57	TRUE	reported	8KoqAI
4	1E+08	A	C	-0.03272	0.003064	1.30E-26	0.415445	462346	NA	rs1442480	NA	UKB-b-57	TRUE	reported	8KoqAI
4	1E+08	A	T	-0.03273	0.003064	1.30E-26	0.41544	462346	NA	rs1789900	NA	UKB-b-57	TRUE	reported	8KoqAI
4	1E+08	A	G	-0.0327	0.003064	1.40E-26	0.41546	462346	NA	rs1442481	NA	UKB-b-57	TRUE	reported	8KoqAI
4	1E+08	T	C	0.022739	0.003633	3.90E-10	0.226308	462346	NA	rs3846449	NA	UKB-b-57	TRUE	reported	8KoqAI
4	1E+08	G	A	-0.0327	0.003064	1.40E-26	0.41546	462346	NA	rs1693456	NA	UKB-b-57	TRUE	reported	8KoqAI
4	1E+08	T	C	-0.01783	0.003247	4.00E-08	0.317859	462346	NA	rs1789901	NA	UKB-b-57	TRUE	reported	8KoqAI
4	1E+08	T	C	-0.03273	0.003064	1.30E-26	0.415499	462346	NA	rs1662060	NA	UKB-b-57	TRUE	reported	8KoqAI
4	1E+08	C	T	-0.03269	0.003064	1.40E-26	0.415496	462346	NA	rs1789902	NA	UKB-b-57	TRUE	reported	8KoqAI
4	1E+08	T	C	-0.03269	0.003064	1.40E-26	0.415496	462346	NA	rs1693476	NA	UKB-b-57	TRUE	reported	8KoqAI
4	1E+08	A	C	-0.03267	0.003064	1.50E-26	0.415493	462346	NA	rs1662059	NA	UKB-b-57	TRUE	reported	8KoqAI
4	1E+08	C	A	-0.04664	0.005103	6.20E-20	0.0976	462346	NA	rs6230729	NA	UKB-b-57	TRUE	reported	8KoqAI
4	1E+08	T	C	-0.03266	0.003064	1.60E-26	0.415498	462346	NA	rs698	NA	UKB-b-57	TRUE	reported	8KoqAI
4	1E+08	C	G	-0.03267	0.003064	1.50E-26	0.415493	462346	NA	rs2298755	NA	UKB-b-57	TRUE	reported	8KoqAI
4	1E+08	G	A	-0.03267	0.003064	1.50E-26	0.415493	462346	NA	rs3114048	NA	UKB-b-57	TRUE	reported	8KoqAI
4	1E+08	C	A	-0.04669	0.005103	5.80E-20	0.097597	462346	NA	rs3518976	NA	UKB-b-57	TRUE	reported	8KoqAI
4	1E+08	G	A	-0.03267	0.003064	1.50E-26	0.415509	462346	NA	rs1693471	NA	UKB-b-57	TRUE	reported	8KoqAI
4	1E+08	C	G	-0.03267	0.003064	1.50E-26	0.415493	462346	NA	rs1789903	NA	UKB-b-57	TRUE	reported	8KoqAI
4	1E+08	C	T	0.26084	0.013611	7.50E-82	0.012449	462346	NA	rs1419739	NA	UKB-b-57	TRUE	reported	8KoqAI
4	1E+08	G	A	-0.01777	0.003246	4.40E-08	0.317888	462346	NA	rs1789904	NA	UKB-b-57	TRUE	reported	8KoqAI
4	1E+08	A	G	0.022756	0.003633	3.80E-10	0.22613	462346	NA	rs2009181	NA	UKB-b-57	TRUE	reported	8KoqAI
4	1E+08	T	C	-0.03267	0.003064	1.50E-26	0.415468	462346	NA	rs1662058	NA	UKB-b-57	TRUE	reported	8KoqAI
4	1E+08	C	T	-0.03267	0.003064	1.50E-26	0.415492	462346	NA	rs1693477	NA	UKB-b-57	TRUE	reported	8KoqAI
4	1E+08	T	A	-0.03256	0.003064	2.30E-26	0.415517	462346	NA	rs1789906	NA	UKB-b-57	TRUE	reported	8KoqAI
4	1E+08	T	C	-0.03263	0.003064	1.80E-26	0.415402	462346	NA	rs904093	NA	UKB-b-57	TRUE	reported	8KoqAI
4	1E+08	A	G	-0.03255	0.003064	2.30E-26	0.415505	462346	NA	rs904094	NA	UKB-b-57	TRUE	reported	8KoqAI
4	1E+08	A	G	-0.03255	0.003064	2.30E-26	0.415499	462346	NA	rs904095	NA	UKB-b-57	TRUE	reported	8KoqAI
4	1E+08	T	C	-0.03255	0.003064	2.30E-26	0.415499	462346	NA	rs1662057	NA	UKB-b-57	TRUE	reported	8KoqAI
4	1E+08	A	T	-0.03255	0.003064	2.30E-26	0.415499	462346	NA	rs1789908	NA	UKB-b-57	TRUE	reported	8KoqAI
4	1E+08	T	G	-0.03254	0.003064	2.40E-26	0.415546	462346	NA	rs904096	NA	UKB-b-57	TRUE	reported	8KoqAI
4	1E+08	A	T	-0.04676	0.005104	5.10E-20	0.097603	462346	NA	rs2866153	NA	UKB-b-57	TRUE	reported	8KoqAI
4	1E+08	A	G	-0.03255	0.003064	2.30E-26	0.415517	462346	NA	rs1789910	NA	UKB-b-57	TRUE	reported	8KoqAI
4	1E+08	G	C	-0.03255	0.003064	2.30E-26	0.415517	462346	NA	rs1693480	NA	UKB-b-57	TRUE	reported	8KoqAI
4	1E+08	T	C	-0.03255	0.003064	2.30E-26	0.415517	462346	NA	rs1789911	NA	UKB-b-57	TRUE	reported	8KoqAI
4	1E+08	C	T	-0.03223	0.003064	7.00E-26	0.415715	462346	NA	rs1693481	NA	UKB-b-57	TRUE	reported	8KoqAI
4	1E+08	C	T	-0.03236	0.003066	4.70E-26	0.415148	462346	NA	rs1789912	NA	UKB-b-57	TRUE	reported	8KoqAI
4	1E+08	C	T	-0.03254	0.003064	2.40E-26	0.415502	462346	NA	rs1693482	NA	UKB-b-57	TRUE	reported	8KoqAI
4	1E+08	G	C	-0.03256	0.003064	2.30E-26	0.415472	462346	NA	rs1631460	NA	UKB-b-57	TRUE	reported	8KoqAI
4	1E+08	T	C	-0.03264	0.003064	1.70E-26	0.415444	462346	NA	rs1693483	NA	UKB-b-57	TRUE	reported	8KoqAI
4	1E+08	C	G	-0.03263	0.003064	1.80E-26	0.415441	462346	NA	rs1693424	NA	UKB-b-57	TRUE	reported	8KoqAI
4	1E+08	C	A	-0.03263	0.003064	1.70E-26	0.415414	462346	NA	rs1625439	NA	UKB-b-57	TRUE	reported	8KoqAI
4	1E+08	A	G	-0.03263	0.003064	1.70E-26	0.415414	462346	NA	rs1789913	NA	UKB-b-57	TRUE	reported	8KoqAI
4	1E+08	G	A	-0.03266	0.003064	1.60E-26	0.415389	462346	NA	rs6230729	NA	UKB-b-57	TRUE	reported	8KoqAI
4	1E+08	A	C	-0.01842	0.003349	3.80E-08	0.290081	462346	NA	rs1662056	NA	UKB-b-57	TRUE	reported	8KoqAI
4	1E+08	G	C	-0.0184	0.003348	3.90E-08	0.290276	462346	NA	rs1662055	NA	UKB-b-57	TRUE	reported	8KoqAI
4	1E+08	C	T	-0.03265	0.003064	1.60E-26	0.415388	462346	NA	rs1693425	NA	UKB-b-57	TRUE	reported	8KoqAI
4	1E+08	T	C	0.022738	0.003633	3.90E-10	0.226156	462346	NA	rs2241894	NA	UKB-b-57	TRUE	reported	8KoqAI
4	1E+08	A	G	-0.03268	0.003064	1.50E-26	0.415484	462346	NA	rs1693426	NA	UKB-b-57	TRUE	reported	8KoqAI
4	1E+08	A	G	-0.01775	0.003247	4.50E-08	0.317872	462346	NA	rs1789915	NA	UKB-b-57	TRUE	reported	8KoqAI
4	1E+08	A	C	-0.03266	0.003064	1.60E-26	0.415472	462346	NA	rs1662053	NA	UKB-b-57	TRUE	reported	8KoqAI
4	1E+08	A	G	-0.03246	0.003064	3.20E-26	0.415593	462346	NA	rs1662052	NA	UKB-b-57	TRUE	reported	8KoqAI
4	1E+08	G	A	-0.03246	0.003064	3.20E-26	0.415598	462346	NA	rs1789916	NA	UKB-b-57	TRUE	reported	8KoqAI
4	1E+08	T	C	-0.03267	0.003064	1.60E-26	0.415465	462346	NA	rs1693427	NA	UKB-b-57	TRUE	reported	8KoqAI
4	1E+08	A	T	-0.03269	0.003064	1.50E-26	0.415456	462346	NA	rs1693428	NA	UKB-b-57	TRUE	reported	8KoqAI

4	1E+08	G	A	-0.04675	0.005104	5.20E-20	0.097605	462346	NA	rs6230731	NA	UKB-b-57	TRUE	reported	8KoqAI
4	1E+08	A	G	0.062401	0.006192	6.90E-24	0.063775	462346	NA	rs6532815	NA	UKB-b-57	TRUE	reported	8KoqAI
4	1E+08	G	A	-0.03268	0.003064	1.50E-26	0.415448	462346	NA	rs1789917	NA	UKB-b-57	TRUE	reported	8KoqAI
4	1E+08	C	A	-0.03269	0.003064	1.40E-26	0.415442	462346	NA	rs1662051	NA	UKB-b-57	TRUE	reported	8KoqAI
4	1E+08	C	T	0.02271	0.003632	4.00E-10	0.226181	462346	NA	rs1263983	NA	UKB-b-57	TRUE	reported	8KoqAI
4	1E+08	A	G	-0.07319	0.009888	1.30E-13	0.975816	462346	NA	rs283412	NA	UKB-b-57	TRUE	reported	8KoqAI
4	1E+08	A	G	-0.0327	0.003064	1.40E-26	0.415423	462346	NA	rs1789919	NA	UKB-b-57	TRUE	reported	8KoqAI
4	1E+08	T	G	-0.03269	0.003064	1.40E-26	0.415442	462346	NA	rs1693431	NA	UKB-b-57	TRUE	reported	8KoqAI
4	1E+08	C	T	0.019848	0.003411	5.90E-09	0.272756	462346	NA	rs3762896	NA	UKB-b-57	TRUE	reported	8KoqAI
4	1E+08	A	C	-0.04686	0.005103	4.20E-20	0.097645	462346	NA	rs3398819	NA	UKB-b-57	TRUE	reported	8KoqAI
4	1E+08	A	C	-0.18989	0.015709	1.20E-33	0.990681	462346	NA	rs283413	NA	UKB-b-57	TRUE	reported	8KoqAI
4	1E+08	T	C	0.019829	0.003411	6.10E-09	0.272773	462346	NA	rs4147542	NA	UKB-b-57	TRUE	reported	8KoqAI
4	1E+08	A	C	-0.04693	0.005109	4.10E-20	0.097525	462346	NA	rs3417805	NA	UKB-b-57	TRUE	reported	8KoqAI
4	1E+08	A	C	-0.01777	0.003247	4.40E-08	0.317868	462346	NA	rs1789920	NA	UKB-b-57	TRUE	reported	8KoqAI
4	1E+08	G	A	-0.04677	0.005104	5.10E-20	0.097604	462346	NA	rs1752659	NA	UKB-b-57	TRUE	reported	8KoqAI
4	1E+08	G	A	-0.01777	0.003247	4.50E-08	0.317867	462346	NA	rs1789921	NA	UKB-b-57	TRUE	reported	8KoqAI
4	1E+08	C	T	0.025367	0.003045	8.10E-17	0.560078	462346	NA	rs283415	NA	UKB-b-57	TRUE	reported	8KoqAI
4	1E+08	C	G	-0.01778	0.003247	4.40E-08	0.317839	462346	NA	rs3133158	NA	UKB-b-57	TRUE	reported	8KoqAI
4	1E+08	A	G	-0.03268	0.003064	1.50E-26	0.415437	462346	NA	rs1629270	NA	UKB-b-57	TRUE	reported	8KoqAI
4	1E+08	C	T	-0.01778	0.003247	4.30E-08	0.317817	462346	NA	rs1789923	NA	UKB-b-57	TRUE	reported	8KoqAI
4	1E+08	C	T	-0.03151	0.003064	8.20E-25	0.416246	462346	NA	rs1662049	NA	UKB-b-57	TRUE	reported	8KoqAI
4	1E+08	T	C	-0.03152	0.003064	8.00E-25	0.416248	462346	NA	rs1662048	NA	UKB-b-57	TRUE	reported	8KoqAI
4	1E+08	T	C	0.01984	0.003412	6.10E-09	0.272716	462346	NA	rs6846835	NA	UKB-b-57	TRUE	reported	8KoqAI
4	1E+08	A	G	-0.04674	0.005104	5.30E-20	0.097617	462346	NA	rs1758602	NA	UKB-b-57	TRUE	reported	8KoqAI
4	1E+08	G	A	0.062208	0.006186	8.60E-24	0.063926	462346	NA	rs3476149	NA	UKB-b-57	TRUE	reported	8KoqAI
4	1E+08	C	G	0.019821	0.003412	6.30E-09	0.272699	462346	NA	rs1193686	NA	UKB-b-57	TRUE	reported	8KoqAI
4	1E+08	G	C	0.019896	0.003411	5.40E-09	0.272859	462346	NA	rs4147541	NA	UKB-b-57	TRUE	reported	8KoqAI
4	1E+08	C	T	-0.03161	0.003064	5.90E-25	0.416183	462346	NA	rs1789924	NA	UKB-b-57	TRUE	reported	8KoqAI
4	1E+08	A	T	-0.04673	0.005104	5.40E-20	0.097618	462346	NA	rs3477468	NA	UKB-b-57	TRUE	reported	8KoqAI
4	1E+08	T	C	-0.04662	0.005103	6.40E-20	0.097787	462346	NA	rs1758616	NA	UKB-b-57	TRUE	reported	8KoqAI
4	1E+08	A	G	-0.04673	0.005104	5.40E-20	0.097618	462346	NA	rs1149982	NA	UKB-b-57	TRUE	reported	8KoqAI
4	1E+08	G	A	-0.03162	0.003064	5.70E-25	0.416163	462346	NA	rs1629838	NA	UKB-b-57	TRUE	reported	8KoqAI
4	1E+08	C	T	0.020014	0.003411	4.40E-09	0.27292	462346	NA	rs1149982	NA	UKB-b-57	TRUE	reported	8KoqAI
4	1E+08	C	G	-0.03163	0.003064	5.50E-25	0.416148	462346	NA	rs2584453	NA	UKB-b-57	TRUE	reported	8KoqAI
4	1E+08	T	C	-0.03163	0.003064	5.50E-25	0.416148	462346	NA	rs1789925	NA	UKB-b-57	TRUE	reported	8KoqAI
4	1E+08	G	A	-0.03163	0.003064	5.50E-25	0.416148	462346	NA	rs1693469	NA	UKB-b-57	TRUE	reported	8KoqAI
4	1E+08	G	C	-0.06365	0.010059	2.50E-10	0.976612	462346	NA	rs166893	NA	UKB-b-57	TRUE	reported	8KoqAI
4	1E+08	T	C	-0.06362	0.010059	2.50E-10	0.976548	462346	NA	rs283407	NA	UKB-b-57	TRUE	reported	8KoqAI
4	1E+08	G	A	-0.04678	0.005104	5.00E-20	0.097609	462346	NA	rs7874440	NA	UKB-b-57	TRUE	reported	8KoqAI
4	1E+08	A	G	-0.06364	0.010059	2.50E-10	0.976613	462346	NA	rs172222	NA	UKB-b-57	TRUE	reported	8KoqAI
4	1E+08	G	T	0.025399	0.003045	7.40E-17	0.560055	462346	NA	rs283408	NA	UKB-b-57	TRUE	reported	8KoqAI
4	1E+08	G	A	0.019886	0.003411	5.50E-09	0.272849	462346	NA	rs1000654	NA	UKB-b-57	TRUE	reported	8KoqAI
4	1E+08	C	T	-0.03164	0.003064	5.30E-25	0.416167	462346	NA	rs2453980	NA	UKB-b-57	TRUE	reported	8KoqAI
4	1E+08	T	C	-0.04677	0.005104	5.00E-20	0.097603	462346	NA	rs1149982	NA	UKB-b-57	TRUE	reported	8KoqAI
4	1E+08	T	C	0.061558	0.006186	2.50E-23	0.063702	462346	NA	rs4699741	NA	UKB-b-57	TRUE	reported	8KoqAI
4	1E+08	C	A	-0.03162	0.003064	5.60E-25	0.41614	462346	NA	rs980972	NA	UKB-b-57	TRUE	reported	8KoqAI
4	1E+08	T	C	-0.04671	0.004997	9.00E-21	0.10247	462346	NA	rs6230732	NA	UKB-b-57	TRUE	reported	8KoqAI
4	1E+08	G	A	-0.03163	0.003064	5.50E-25	0.416148	462346	NA	rs1693470	NA	UKB-b-57	TRUE	reported	8KoqAI
4	1E+08	G	A	-0.03162	0.003064	5.60E-25	0.416141	462346	NA	rs1662039	NA	UKB-b-57	TRUE	reported	8KoqAI
4	1E+08	C	T	-0.03161	0.003064	5.80E-25	0.416188	462346	NA	rs2851300	NA	UKB-b-57	TRUE	reported	8KoqAI
4	1E+08	G	A	-0.04666	0.004995	9.60E-21	0.102518	462346	NA	rs7823415	NA	UKB-b-57	TRUE	reported	8KoqAI
4	1E+08	G	C	-0.03162	0.003064	5.60E-25	0.416141	462346	NA	rs1234584	NA	UKB-b-57	TRUE	reported	8KoqAI
4	1E+08	T	C	-0.03162	0.003064	5.80E-25	0.416096	462346	NA	rs1662021	NA	UKB-b-57	TRUE	reported	8KoqAI
4	1E+08	T	C	-0.03162	0.003064	5.60E-25	0.41613	462346	NA	rs1662020	NA	UKB-b-57	TRUE	reported	8KoqAI
4	1E+08	G	A	-0.04666	0.004995	9.50E-21	0.102517	462346	NA	rs6230732	NA	UKB-b-57	TRUE	reported	8KoqAI
4	1E+08	C	T	0.062197	0.006188	9.10E-24	0.063669	462346	NA	rs1139097	NA	UKB-b-57	TRUE	reported	8KoqAI
4	1E+08	T	C	0.01988	0.003417	6.00E-09	0.271183	462346	NA	rs1264980	NA	UKB-b-57	TRUE	reported	8KoqAI
4	1E+08	T	C	-0.03166	0.003064	5.10E-25	0.415986	462346	NA	rs1238015	NA	UKB-b-57	TRUE	reported	8KoqAI
4	1E+08	C	T	0.061982	0.00619	1.30E-23	0.063607	462346	NA	rs1151799	NA	UKB-b-57	TRUE	reported	8KoqAI
4	1E+08	A	G	-0.06364	0.010059	2.50E-10	0.976611	462346	NA	rs283405	NA	UKB-b-57	TRUE	reported	8KoqAI
4	1E+08	A	G	-0.06431	0.010075	1.70E-10	0.976693	462346	NA	rs3098807	NA	UKB-b-57	TRUE	reported	8KoqAI
4	1E+08	C	A	0.061693	0.006135	8.60E-24	0.0649	462346	NA	rs1114059	NA	UKB-b-57	TRUE	reported	8KoqAI
4	1E+08	A	G	-0.03163	0.003064	5.60E-25	0.41617	462346	NA	rs1229858	NA	UKB-b-57	TRUE	reported	8KoqAI
4	1E+08	C	T	-0.03163	0.003064	5.40E-25	0.416147	462346	NA	rs1229857	NA	UKB-b-57	TRUE	reported	8KoqAI
4	1E+08	T	C	0.019872	0.003411	5.70E-09	0.272832	462346	NA	rs1003116	NA	UKB-b-57	TRUE	reported	8KoqAI
4	1E+08	G	C	-0.03163	0.003064	5.40E-25	0.416147	462346	NA	rs1229856	NA	UKB-b-57	TRUE	reported	8KoqAI
4	1E+08	G	A	0.061689	0.006135	8.80E-24	0.06489	462346	NA	rs4699743	NA	UKB-b-57	TRUE	reported	8KoqAI
4	1E+08	C	T	-0.04679	0.004994	7.30E-21	0.102574	462346	NA	rs6230732	NA	UKB-b-57	TRUE	reported	8KoqAI
4	1E+08	G	A	0.061994	0.006191	1.30E-23	0.063603	462346	NA	rs1478511	NA	UKB-b-57	TRUE	reported	8KoqAI
4	1E+08	A	G	-0.03165	0.003064	5.20E-25	0.416044	462346	NA	rs1229855	NA	UKB-b-57	TRUE	reported	8KoqAI
4	1E+08	C	T	-0.03164	0.003064	5.30E-25	0.416126	462346	NA	rs1229854	NA	UKB-b-57	TRUE	reported	8KoqAI
4	1E+08	C	G	-0.0467	0.004995	8.90E-21	0.102536	462346	NA	rs6230732	NA	UKB-b-57	TRUE	reported	8KoqAI
4	1E+08	C	T	-0.03164	0.003064	5.30E-25	0.416126	462346	NA	rs1229851	NA	UKB-b-57	TRUE	reported	8KoqAI
4	1E+08	A	G	-0.04681	0.004999	7.70E-21	0.102455	462346	NA	rs5610971	NA	UKB-b-57	TRUE	reported	8KoqAI
4	1E+08	G	A	-0.03172	0.003066	4.30E-25	0.416077	462346	NA	rs1154435	NA	UKB-b-57	TRUE	reported	8KoqAI
4	1E+08	T	C	-0.06371	0.01007	2.50E-10	0.976617	462346	NA	rs283423	NA	UKB-b-57	TRUE	reported	8KoqAI
4	1E+08	A	T	-0.0472	0.005007	4.20E-21	0.102447	462346	NA	rs5600968	NA	UKB-b-57	TRUE	reported	8KoqAI
4	1E+08	G	A	-0.03183	0.003072	3.80E-25	0.415781	462346	NA	rs1154438	NA	UKB-b-57	TRUE	reported	8KoqAI
4	1E+08	G	A	-0.04741	0.005016	3.30E-21	0.102334	462346	NA	rs2165670	NA	UKB-b-57	TRUE	reported	8KoqAI
4	1E+08	G	T	-0.06319	0.010068	3.50E-10	0.976471	462346	NA	rs192550	NA	UKB-b-57	TRUE	reported	8KoqAI
4	1E+08	C	T	-0.03188	0.003073	3.20E-25	0.416101	462346	NA	rs1154439	NA	UKB-b-57	TRUE	reported	8KoqAI
4	1E+08	G	A	-0.03187	0.003074	3.40E-25	0.416156	462346	NA	rs1154440	NA	UKB-b-57	TRUE	reported	8KoqAI
4	1E+08	C	T	0.061902	0.006163	9.70E-24	0.064824	462346	NA	rs7692041	NA	UKB-b-57	TRUE	reported	8KoqAI
4	1E+08	G	A	-0.04756	0.00502	2.70E-21	0.102407	462346	NA	rs6230576	NA	UKB-b-57	TRUE	reported	8KoqAI
4	1E+08	C	T	-0.03197	0.003079	3.00E-25	0.41616	462346	NA	rs1229973	NA	UKB-b-57	TRUE	reported	8KoqAI
4	1E+08	T	C	-0.04757	0.005027	3.00E-21	0.102406	462346	NA	rs6230576	NA	UKB-b-57	TRUE	reported	8KoqAI
4	1E+08	C	G	-0.03223	0.003089	1.80E-25	0.415192	462346	NA	rs1583974	NA	UKB-b-57	TRUE	reported	8KoqAI
4	1E+08	C	T	-0.03217	0.003089	2.20E-25	0.415432	462346	NA	rs1154442	NA	UKB-b-57	TRUE	reported	8KoqAI
4	1E+08	C	T	-0.04763	0.005037	3.20E-21	0.102329	462346	NA	rs1583973	NA	UKB-b-57	TRUE	reported	8KoqAI
4	1E+08	T	A	-0.03221	0.003086	1.70E-25	0.415996	462346	NA	rs1154444	NA	UKB-b-57	TRUE	reported	8KoqAI
4	1E+08	C	G	-0.04778	0.00504	2.60E-21	0.102342	462346	NA	rs7833610	NA	UKB-b-57	TRUE	reported	8KoqAI
4	1E+08	T	G	-0.03204	0.003088	3.20E-25	0.416244	462346	NA	rs1154445	NA	UKB-b-57	TRUE	reported	8KoqAI
4	1E+08	G	T	-0.03212	0.003092	2.80E-25	0.416157	462346	NA	rs1154447	NA	UKB-b-57	TRUE	reported	8KoqAI
4	1E+08	C	T	-0.04789	0.005049	2.40E-21	0.102808	462346	NA	rs6230576	NA	UKB-b-57	TRUE	reported	8KoqAI
4	1E+08	T	G	-0.04825	0.005053	1.30E-21	0.102423	462346	NA	rs6230576	NA	UKB-b-57	TRUE	reported	8KoqAI
4	1E+08	C	A	0.062165	0.006299	5.70E-23	0.062876	462346	NA	rs1837934	NA	UKB-b-57	TRUE	reported	8KoqAI
4	1E+08	C	A	0.061809	0.006283	7.70E-23	0.063127	462346	NA	rs7268192	NA	UKB-b-57	TRUE	reported	8KoqAI
4	1E+08	A	T	-0.04835	0.005057	1.20E-21	0.102391	462346	NA	rs5639036	NA	UKB-b-57	TRUE	reported	8KoqAI
4	1E+08	A	T	0.020522	0.00344	2.40E-09	0.270212	462346	NA	rs1264438	NA	UKB-b-57	TRUE	reported	8KoqAI
4	1E+08	C	G	-0.04844	0.005062	1.10E-21	0.102313	462346	NA	rs5569552	NA	UKB-b-57	TRUE	reported	8KoqAI
4	1E+08	C	G	-0.04852	0.005066	9.90E-22	0.102253	462346	NA	rs6230578	NA	UKB-b-57	TRUE	reported	8KoqAI
4	1E+08	C	A	-0.03809	0.003866	6.70E-23	0.196337	462346	NA	rs1302808	NA	UKB-b-57	TRUE	reported	8KoqAI
4	1E+08	A	G	-0.06612	0.010206	9.30E-11	0.976688	462346	NA	rs283419	NA	UKB-b-57	TRUE	reported	8KoqAI
4	1E+08	G	C	-0.02773	0.004027	5.70E-12	0.187017	462346	NA	rs6843522	NA	UKB-b-57	TRUE	reported	8KoqAI
4	1E+08	C	T	-0.0422	0.004858	3.70E-18	0.115118	462346	NA	rs6230578	NA	UKB-b-57	TRUE	reported	8KoqAI
4	1E+08	G	A	0.026081	0.003802	6.90E-12	0.201312	462346	NA	rs1814125	NA	UKB-b-57	TRUE	reported	8KoqAI
4	1E+08	C	T	-0.0453	0.00526	7.20E-18	0.095747	462346	NA	rs6230578	NA	UKB-b-57	TRUE	reported	8KoqAI

4	1E+08	T	A	-0.04041	0.004629	2.60E-18	0.1278	462346	NA	rs3511157	NA	UKB-b-57	TRUE	reported	8KoqAI
4	1E+08	G	A	-0.04048	0.004629	2.30E-18	0.127797	462346	NA	rs3607632	NA	UKB-b-57	TRUE	reported	8KoqAI
4	1E+08	G	T	-0.0405	0.004627	2.10E-18	0.127912	462346	NA	rs3419239	NA	UKB-b-57	TRUE	reported	8KoqAI
4	1E+08	G	A	-0.04123	0.004621	4.50E-19	0.128279	462346	NA	rs3407694	NA	UKB-b-57	TRUE	reported	8KoqAI
4	1E+08	A	G	-0.04088	0.004621	9.10E-19	0.128355	462346	NA	rs3604191	NA	UKB-b-57	TRUE	reported	8KoqAI
4	1E+08	C	T	-0.04092	0.004623	8.60E-19	0.128292	462346	NA	rs3500210	NA	UKB-b-57	TRUE	reported	8KoqAI
4	1E+08	C	T	-0.0413	0.004621	4.00E-19	0.128273	462346	NA	rs3414418	NA	UKB-b-57	TRUE	reported	8KoqAI
4	1E+08	T	C	-0.04131	0.004612	3.30E-19	0.128685	462346	NA	rs3457613	NA	UKB-b-57	TRUE	reported	8KoqAI
4	1E+08	C	T	-0.0414	0.004611	2.70E-19	0.128713	462346	NA	rs3530292	NA	UKB-b-57	TRUE	reported	8KoqAI
4	1E+08	T	G	-0.04139	0.004609	2.70E-19	0.128741	462346	NA	rs3475077	NA	UKB-b-57	TRUE	reported	8KoqAI
4	1E+08	A	G	0.025391	0.00379	2.10E-11	0.20271	462346	NA	rs2880841	NA	UKB-b-57	TRUE	reported	8KoqAI
4	1E+08	C	T	0.025488	0.003829	2.80E-11	0.200189	462346	NA	rs2887202	NA	UKB-b-57	TRUE	reported	8KoqAI
4	1E+08	T	G	0.025482	0.003829	2.80E-11	0.200119	462346	NA	rs2884729	NA	UKB-b-57	TRUE	reported	8KoqAI
4	1E+08	G	A	0.025304	0.003807	3.00E-11	0.201546	462346	NA	rs2881469	NA	UKB-b-57	TRUE	reported	8KoqAI
4	1E+08	T	C	0.025301	0.003805	2.90E-11	0.201661	462346	NA	rs2880118	NA	UKB-b-57	TRUE	reported	8KoqAI
4	1E+08	T	C	0.025372	0.003796	2.30E-11	0.20226	462346	NA	rs2881367	NA	UKB-b-57	TRUE	reported	8KoqAI
4	1E+08	C	G	0.025453	0.003791	1.90E-11	0.202528	462346	NA	rs2885569	NA	UKB-b-57	TRUE	reported	8KoqAI
4	1E+08	C	T	0.025403	0.003783	1.90E-11	0.20308	462346	NA	rs6672877	NA	UKB-b-57	TRUE	reported	8KoqAI
4	1E+08	A	C	0.025353	0.003781	2.00E-11	0.203178	462346	NA	rs2877659	NA	UKB-b-57	TRUE	reported	8KoqAI
4	1E+08	T	C	0.025723	0.003794	1.20E-11	0.202089	462346	NA	rs6834433	NA	UKB-b-57	TRUE	reported	8KoqAI
4	1E+08	A	G	0.025739	0.003797	1.20E-11	0.20191	462346	NA	rs6857143	NA	UKB-b-57	TRUE	reported	8KoqAI
4	1E+08	T	G	0.025633	0.003797	1.50E-11	0.201912	462346	NA	rs6834464	NA	UKB-b-57	TRUE	reported	8KoqAI
4	1E+08	C	T	-0.04179	0.004604	1.10E-19	0.12862	462346	NA	rs1312146	NA	UKB-b-57	TRUE	reported	8KoqAI
4	1E+08	T	A	-0.0441	0.004959	6.00E-19	0.108441	462346	NA	rs5627950	NA	UKB-b-57	TRUE	reported	8KoqAI
4	1E+08	T	C	0.025462	0.003788	1.80E-11	0.202278	462346	NA	rs1121534	NA	UKB-b-57	TRUE	reported	8KoqAI
4	1E+08	G	A	-0.04181	0.004599	9.80E-20	0.128642	462346	NA	rs6827898	NA	UKB-b-57	TRUE	reported	8KoqAI
4	1E+08	G	A	0.025219	0.00378	2.50E-11	0.202501	462346	NA	rs1003290	NA	UKB-b-57	TRUE	reported	8KoqAI
4	1E+08	T	C	0.025166	0.003779	2.80E-11	0.202559	462346	NA	rs9999976	NA	UKB-b-57	TRUE	reported	8KoqAI
4	1E+08	T	A	-0.04182	0.004599	9.70E-20	0.128582	462346	NA	rs3498088	NA	UKB-b-57	TRUE	reported	8KoqAI
4	1E+08	T	C	0.025206	0.003772	2.40E-11	0.202985	462346	NA	rs1000006	NA	UKB-b-57	TRUE	reported	8KoqAI
4	1E+08	C	A	0.025251	0.003773	2.20E-11	0.202837	462346	NA	rs7608813	NA	UKB-b-57	TRUE	reported	8KoqAI
4	1E+08	C	T	0.025113	0.00377	2.70E-11	0.202897	462346	NA	rs1265116	NA	UKB-b-57	TRUE	reported	8KoqAI
4	1E+08	C	T	0.025149	0.003766	2.40E-11	0.203049	462346	NA	rs6650352	NA	UKB-b-57	TRUE	reported	8KoqAI
4	1E+08	T	C	0.025285	0.003764	1.80E-11	0.20309	462346	NA	rs2881822	NA	UKB-b-57	TRUE	reported	8KoqAI
4	1E+08	G	T	0.025301	0.003761	1.70E-11	0.203174	462346	NA	rs9991913	NA	UKB-b-57	TRUE	reported	8KoqAI
4	1E+08	G	C	0.025248	0.00376	1.90E-11	0.203141	462346	NA	rs9992022	NA	UKB-b-57	TRUE	reported	8KoqAI
4	1E+08	A	G	-0.04193	0.004587	6.10E-20	0.12852	462346	NA	rs1311029	NA	UKB-b-57	TRUE	reported	8KoqAI
4	1E+08	G	T	-0.04183	0.004588	7.70E-20	0.128444	462346	NA	rs5689950	NA	UKB-b-57	TRUE	reported	8KoqAI
4	1E+08	G	A	0.025212	0.003759	2.00E-11	0.203098	462346	NA	rs9994612	NA	UKB-b-57	TRUE	reported	8KoqAI
4	1E+08	A	C	0.025276	0.003758	1.70E-11	0.203141	462346	NA	rs1001713	NA	UKB-b-57	TRUE	reported	8KoqAI
4	1E+08	T	C	0.025295	0.003758	1.70E-11	0.203088	462346	NA	rs1908963	NA	UKB-b-57	TRUE	reported	8KoqAI
4	1E+08	A	G	0.025302	0.003758	1.70E-11	0.203076	462346	NA	rs1837931	NA	UKB-b-57	TRUE	reported	8KoqAI
4	1E+08	G	A	0.025229	0.003757	1.90E-11	0.203137	462346	NA	rs1837930	NA	UKB-b-57	TRUE	reported	8KoqAI
4	1E+08	G	C	0.025412	0.003758	1.40E-11	0.203062	462346	NA	rs2882420	NA	UKB-b-57	TRUE	reported	8KoqAI
4	1E+08	T	C	0.027093	0.004687	7.50E-09	0.121991	462346	NA	rs1250567	NA	UKB-b-57	TRUE	reported	8KoqAI
4	1E+08	C	A	0.027102	0.004687	7.40E-09	0.121979	462346	NA	rs1250172	NA	UKB-b-57	TRUE	reported	8KoqAI
4	1E+08	T	C	0.025217	0.003757	1.90E-11	0.203133	462346	NA	rs1264563	NA	UKB-b-57	TRUE	reported	8KoqAI
4	1E+08	G	A	0.02523	0.003757	1.90E-11	0.203147	462346	NA	rs1264252	NA	UKB-b-57	TRUE	reported	8KoqAI
4	1E+08	G	T	-0.04187	0.004581	6.30E-20	0.128604	462346	NA	rs1579752	NA	UKB-b-57	TRUE	reported	8KoqAI
4	1E+08	T	A	0.025272	0.003757	1.70E-11	0.203136	462346	NA	rs1264574	NA	UKB-b-57	TRUE	reported	8KoqAI
4	1E+08	A	C	-0.04186	0.004581	6.30E-20	0.128618	462346	NA	rs1826907	NA	UKB-b-57	TRUE	reported	8KoqAI
4	1E+08	C	A	0.025263	0.003757	1.80E-11	0.20322	462346	NA	rs1264263	NA	UKB-b-57	TRUE	reported	8KoqAI
4	1E+08	T	C	-0.04447	0.004959	3.00E-19	0.10745	462346	NA	rs1826908	NA	UKB-b-57	TRUE	reported	8KoqAI
4	1E+08	T	A	0.025259	0.003757	1.80E-11	0.203113	462346	NA	rs1264641	NA	UKB-b-57	TRUE	reported	8KoqAI
4	1E+08	T	A	0.025266	0.003757	1.80E-11	0.20313	462346	NA	rs1264646	NA	UKB-b-57	TRUE	reported	8KoqAI
4	1E+08	T	C	0.027016	0.004687	8.20E-09	0.122005	462346	NA	rs7268193	NA	UKB-b-57	TRUE	reported	8KoqAI
4	1E+08	A	C	0.025267	0.003757	1.80E-11	0.203132	462346	NA	rs1264936	NA	UKB-b-57	TRUE	reported	8KoqAI
4	1E+08	T	G	-0.04186	0.004581	6.30E-20	0.128618	462346	NA	rs3586450	NA	UKB-b-57	TRUE	reported	8KoqAI
4	1E+08	G	A	0.025271	0.003757	1.70E-11	0.20313	462346	NA	rs1264341	NA	UKB-b-57	TRUE	reported	8KoqAI
4	1E+08	T	C	0.025253	0.003757	1.80E-11	0.203193	462346	NA	rs6734156	NA	UKB-b-57	TRUE	reported	8KoqAI
4	1E+08	A	T	-0.04359	0.005105	1.40E-17	0.101094	462346	NA	rs1702889	NA	UKB-b-57	TRUE	reported	8KoqAI
4	1E+08	T	C	0.025175	0.003757	2.10E-11	0.203154	462346	NA	rs1264731	NA	UKB-b-57	TRUE	reported	8KoqAI
4	1E+08	G	A	0.025097	0.003758	2.40E-11	0.203011	462346	NA	rs1264419	NA	UKB-b-57	TRUE	reported	8KoqAI
4	1E+08	T	G	0.02509	0.003758	2.50E-11	0.203023	462346	NA	rs1264735	NA	UKB-b-57	TRUE	reported	8KoqAI
4	1E+08	A	T	0.025098	0.003758	2.40E-11	0.203012	462346	NA	rs1265018	NA	UKB-b-57	TRUE	reported	8KoqAI
4	1E+08	T	A	0.025098	0.003758	2.40E-11	0.203012	462346	NA	rs1264737	NA	UKB-b-57	TRUE	reported	8KoqAI
4	1E+08	T	C	0.025153	0.003757	2.20E-11	0.203063	462346	NA	rs1264744	NA	UKB-b-57	TRUE	reported	8KoqAI
4	1E+08	G	A	-0.02704	0.004686	8.00E-09	0.87799	462346	NA	rs2584456	NA	UKB-b-57	TRUE	reported	8KoqAI
4	1E+08	T	C	0.025174	0.003757	2.10E-11	0.20308	462346	NA	rs1264751	NA	UKB-b-57	TRUE	reported	8KoqAI
4	1E+08	G	C	0.025247	0.003756	1.80E-11	0.203138	462346	NA	rs1051643	NA	UKB-b-57	TRUE	reported	8KoqAI
4	1E+08	A	C	0.025155	0.003757	2.20E-11	0.203065	462346	NA	rs1702891	NA	UKB-b-57	TRUE	reported	8KoqAI
4	1E+08	G	T	-0.02703	0.004686	8.10E-09	0.877989	462346	NA	rs2851292	NA	UKB-b-57	TRUE	reported	8KoqAI
4	1E+08	C	T	-0.02696	0.004687	8.70E-09	0.877924	462346	NA	rs2646008	NA	UKB-b-57	TRUE	reported	8KoqAI
4	1E+08	T	A	-0.02702	0.004685	8.10E-09	0.877993	462346	NA	rs2584457	NA	UKB-b-57	TRUE	reported	8KoqAI
4	1E+08	T	C	0.025149	0.003755	2.10E-11	0.20315	462346	NA	rs1264844	NA	UKB-b-57	TRUE	reported	8KoqAI
4	1E+08	C	T	-0.04463	0.004954	2.10E-19	0.107571	462346	NA	rs1372679	NA	UKB-b-57	TRUE	reported	8KoqAI
4	1E+08	C	A	-0.04413	0.004936	3.90E-19	0.108473	462346	NA	rs1372680	NA	UKB-b-57	TRUE	reported	8KoqAI
4	1E+08	A	G	-0.04178	0.004578	7.10E-20	0.128647	462346	NA	rs1051644	NA	UKB-b-57	TRUE	reported	8KoqAI
4	1E+08	A	G	-0.02702	0.004685	8.10E-09	0.878004	462346	NA	rs2584458	NA	UKB-b-57	TRUE	reported	8KoqAI
4	1E+08	A	T	-0.04183	0.004578	6.40E-20	0.128646	462346	NA	rs5691205	NA	UKB-b-57	TRUE	reported	8KoqAI
4	1E+08	T	C	0.025221	0.003757	1.90E-11	0.203117	462346	NA	rs1442484	NA	UKB-b-57	TRUE	reported	8KoqAI
4	1E+08	G	C	0.02522	0.003757	1.90E-11	0.203122	462346	NA	rs1702894	NA	UKB-b-57	TRUE	reported	8KoqAI
4	1E+08	C	T	0.02521	0.003758	2.00E-11	0.20308	462346	NA	rs6658838	NA	UKB-b-57	TRUE	reported	8KoqAI
4	1E+08	C	T	-0.02695	0.004688	9.00E-09	0.878149	462346	NA	rs2851293	NA	UKB-b-57	TRUE	reported	8KoqAI
4	1E+08	A	G	-0.04394	0.004936	5.50E-19	0.108533	462346	NA	rs7383280	NA	UKB-b-57	TRUE	reported	8KoqAI
4	1E+08	C	T	-0.0269	0.004685	9.30E-09	0.878003	462346	NA	rs2646013	NA	UKB-b-57	TRUE	reported	8KoqAI
4	1E+08	G	C	-0.02695	0.004685	8.80E-09	0.878035	462346	NA	rs2646012	NA	UKB-b-57	TRUE	reported	8KoqAI
4	1E+08	G	A	0.025404	0.003758	1.40E-11	0.203027	462346	NA	rs6840700	NA	UKB-b-57	TRUE	reported	8KoqAI
4	1E+08	T	C	0.025218	0.003757	1.90E-11	0.203112	462346	NA	rs6088307	NA	UKB-b-57	TRUE	reported	8KoqAI
4	1E+08	G	A	-0.04401	0.004936	4.80E-19	0.108469	462346	NA	rs7383280	NA	UKB-b-57	TRUE	reported	8KoqAI
4	1E+08	C	G	0.025402	0.003758	1.40E-11	0.203025	462346	NA	rs2877082	NA	UKB-b-57	TRUE	reported	8KoqAI
4	1E+08	C	A	-0.04178	0.004578	7.10E-20	0.128634	462346	NA	rs6812790	NA	UKB-b-57	TRUE	reported	8KoqAI
4	1E+08	G	C	-0.04185	0.004579	6.20E-20	0.128578	462346	NA	rs3436142	NA	UKB-b-57	TRUE	reported	8KoqAI
4	1E+08	C	A	-0.0419	0.004579	5.70E-20	0.128591	462346	NA	rs1313890	NA	UKB-b-57	TRUE	reported	8KoqAI
4	1E+08	A	G	0.025394	0.003758	1.40E-11	0.203023	462346	NA	rs2839124	NA	UKB-b-57	TRUE	reported	8KoqAI
4	1E+08	T	C	-0.02668	0.004698	1.40E-08	0.878972	462346	NA	rs2851294	NA	UKB-b-57	TRUE	reported	8KoqAI
4	1E+08	A	G	0.025197	0.003758	2.00E-11	0.203098	462346	NA	rs378624	NA	UKB-b-57	TRUE	reported	8KoqAI
4	1E+08	C	T	-0.02662	0.004696	1.40E-08	0.878818	462346	NA	rs2851017	NA	UKB-b-57	TRUE	reported	8KoqAI
4	1E+08	C	T	0.025148	0.003758	2.20E-11	0.203059	462346	NA	rs2032350	NA	UKB-b-57	TRUE	reported	8KoqAI
4	1E+08	A	T	-0.02671	0.004698	1.30E-08	0.87897	462346	NA	rs6857127	NA	UKB-b-57	TRUE	reported	8KoqAI
4	1E+08	T	C	-0.02682	0.004697	1.10E-08	0.87886	462346	NA	rs6835524	NA	UKB-b-57	TRUE	reported	8KoqAI
4	1E+08	A	T	-0.04173	0.004578	7.90E-20	0.128585	462346	NA	rs1250892	NA	UKB-b-57	TRUE	reported	8KoqAI
4	1E+08	A	G	0.02506	0.003757	2.60E-11	0.203464	462346	NA	rs1264820	NA	UKB-b-57	TRUE	reported	8KoqAI
4	1E+08	G	A	-0.02659	0.004696	1.50E-08	0.878805	462346	NA	rs2584459	NA	UKB-b-57	TRUE	reported	8KoqAI
4	1E+08	C	T	0.025133	0.003759	2.30E-11	0.203085	462346	NA	rs7660346	NA	UKB-b-57	TRUE	reported	8KoqAI
4	1E+08	A	T	-0.04173	0.004578	7.90E-20	0.128587	462346	NA	rs1251005	NA	UKB-b-57	TRUE	reported	8KoqAI
4	1E+08	C	T	-0.04175	0.004578	7.60E-20	0.128575	462346	NA	rs7667079	NA	UKB-b-57	TRUE	reported	8KoqAI

4	1E+08	C	T	-0.04175	0.004578	7.60E-20	0.128575	462346	NA	rs7667212	NA	UKB-b-57	TRUE	reported	8KoqAI
4	1E+08	C	T	-0.04175	0.004578	7.60E-20	0.128575	462346	NA	rs6532816	NA	UKB-b-57	TRUE	reported	8KoqAI
4	1E+08	G	A	0.025154	0.00376	2.20E-11	0.20313	462346	NA	rs1604759	NA	UKB-b-57	TRUE	reported	8KoqAI
4	1E+08	C	G	-0.02619	0.004701	2.60E-08	0.8792	462346	NA	rs2197147	NA	UKB-b-57	TRUE	reported	8KoqAI
4	1E+08	A	G	0.025106	0.003759	2.40E-11	0.203448	462346	NA	rs6124815	NA	UKB-b-57	TRUE	reported	8KoqAI
4	1E+08	T	A	-0.04175	0.004578	7.50E-20	0.128595	462346	NA	rs1583977	NA	UKB-b-57	TRUE	reported	8KoqAI
4	1E+08	G	A	-0.02636	0.004686	1.90E-08	0.8781	462346	NA	rs2654834	NA	UKB-b-57	TRUE	reported	8KoqAI
4	1E+08	C	G	-0.02574	0.004716	4.80E-08	0.879746	462346	NA	rs2851016	NA	UKB-b-57	TRUE	reported	8KoqAI
4	1E+08	G	A	0.025572	0.003764	1.10E-11	0.203184	462346	NA	rs1442493	NA	UKB-b-57	TRUE	reported	8KoqAI
4	1E+08	G	A	-0.04156	0.004579	1.10E-19	0.128686	462346	NA	rs4699748	NA	UKB-b-57	TRUE	reported	8KoqAI
4	1E+08	A	G	0.025404	0.003763	1.50E-11	0.203324	462346	NA	rs7692081	NA	UKB-b-57	TRUE	reported	8KoqAI
4	1E+08	T	C	-0.0265	0.004719	1.90E-08	0.879809	462346	NA	rs2584461	NA	UKB-b-57	TRUE	reported	8KoqAI
4	1E+08	A	G	0.025383	0.003763	1.50E-11	0.203331	462346	NA	rs1442492	NA	UKB-b-57	TRUE	reported	8KoqAI
4	1E+08	C	T	0.026593	0.00472	1.80E-08	0.120058	462346	NA	rs1702896	NA	UKB-b-57	TRUE	reported	8KoqAI
4	1E+08	T	C	0.027175	0.003946	5.70E-12	0.188672	462346	NA	rs1702897	NA	UKB-b-57	TRUE	reported	8KoqAI
4	1E+08	T	C	-0.02921	0.005341	4.50E-08	0.089015	462346	NA	rs284788	NA	UKB-b-57	TRUE	reported	8KoqAI
4	1E+08	G	A	0.033885	0.005798	5.10E-09	0.073915	462346	NA	rs7290500	NA	UKB-b-57	TRUE	reported	8KoqAI
4	1E+08	A	C	0.085561	0.010361	1.50E-16	0.021762	462346	NA	rs1489154	NA	UKB-b-57	TRUE	reported	8KoqAI
4	1E+08	T	C	0.0856	0.01036	1.40E-16	0.021752	462346	NA	rs1460221	NA	UKB-b-57	TRUE	reported	8KoqAI
4	1E+08	C	T	0.035582	0.005752	6.20E-10	0.074974	462346	NA	rs1264574	NA	UKB-b-57	TRUE	reported	8KoqAI
4	1E+08	T	A	0.085397	0.010348	1.50E-16	0.021777	462346	NA	rs1134453	NA	UKB-b-57	TRUE	reported	8KoqAI
4	1E+08	T	A	0.027183	0.004906	3.00E-08	0.106409	462346	NA	rs1583971	NA	UKB-b-57	TRUE	reported	8KoqAI
4	1E+08	G	A	0.047816	0.00644	1.10E-13	0.058583	462346	NA	rs1119865	NA	UKB-b-57	TRUE	reported	8KoqAI
4	1E+08	G	T	0.085491	0.010349	1.40E-16	0.02177	462346	NA	rs1115018	NA	UKB-b-57	TRUE	reported	8KoqAI
4	1E+08	A	C	0.047894	0.00644	1.00E-13	0.05859	462346	NA	rs1969097	NA	UKB-b-57	TRUE	reported	8KoqAI
4	1E+08	C	T	0.085512	0.010346	1.40E-16	0.021787	462346	NA	rs1114660	NA	UKB-b-57	TRUE	reported	8KoqAI
4	1E+08	A	G	0.047785	0.006439	1.20E-13	0.0586	462346	NA	rs1147192	NA	UKB-b-57	TRUE	reported	8KoqAI
4	1E+08	C	A	0.036145	0.005738	3.00E-10	0.075403	462346	NA	rs7693958	NA	UKB-b-57	TRUE	reported	8KoqAI
4	1E+08	C	T	0.047858	0.00644	1.10E-13	0.058573	462346	NA	rs1162456	NA	UKB-b-57	TRUE	reported	8KoqAI
4	1E+08	A	C	0.047846	0.00644	1.10E-13	0.058574	462346	NA	rs1153377	NA	UKB-b-57	TRUE	reported	8KoqAI
4	1E+08	A	C	0.08674	0.010736	6.50E-16	0.020844	462346	NA	rs1811636	NA	UKB-b-57	TRUE	reported	8KoqAI
4	1E+08	A	G	0.08674	0.010736	6.50E-16	0.020844	462346	NA	rs1846265	NA	UKB-b-57	TRUE	reported	8KoqAI
4	1E+08	A	G	0.085412	0.010347	1.50E-16	0.021768	462346	NA	rs1888951	NA	UKB-b-57	TRUE	reported	8KoqAI
4	1E+08	A	T	0.036025	0.005739	3.40E-10	0.075306	462346	NA	rs1122840	NA	UKB-b-57	TRUE	reported	8KoqAI
4	1E+08	G	T	0.085424	0.010347	1.50E-16	0.021767	462346	NA	rs1509189	NA	UKB-b-57	TRUE	reported	8KoqAI
4	1E+08	A	C	0.085424	0.010347	1.50E-16	0.021767	462346	NA	rs1500524	NA	UKB-b-57	TRUE	reported	8KoqAI
4	1E+08	G	A	0.085416	0.010347	1.50E-16	0.021767	462346	NA	rs1906530	NA	UKB-b-57	TRUE	reported	8KoqAI
4	1E+08	C	T	0.085422	0.010347	1.50E-16	0.021767	462346	NA	rs1467487	NA	UKB-b-57	TRUE	reported	8KoqAI
4	1E+08	A	G	0.047659	0.006439	1.30E-13	0.058596	462346	NA	rs1160407	NA	UKB-b-57	TRUE	reported	8KoqAI
4	1E+08	A	G	0.085418	0.010347	1.50E-16	0.021767	462346	NA	rs1437227	NA	UKB-b-57	TRUE	reported	8KoqAI
4	1E+08	G	A	0.035528	0.00575	6.50E-10	0.074968	462346	NA	rs2878231	NA	UKB-b-57	TRUE	reported	8KoqAI
4	1E+08	T	C	0.085133	0.010344	1.90E-16	0.021773	462346	NA	rs1452347	NA	UKB-b-57	TRUE	reported	8KoqAI
4	1E+08	G	A	0.047728	0.006438	1.20E-13	0.058578	462346	NA	rs1117417	NA	UKB-b-57	TRUE	reported	8KoqAI
4	1E+08	T	C	0.085391	0.010345	1.50E-16	0.02177	462346	NA	rs1115694	NA	UKB-b-57	TRUE	reported	8KoqAI
4	1E+08	C	T	0.035372	0.005749	7.60E-10	0.074957	462346	NA	rs7811128	NA	UKB-b-57	TRUE	reported	8KoqAI
4	1E+08	A	G	0.047397	0.006437	1.80E-13	0.058575	462346	NA	rs1125519	NA	UKB-b-57	TRUE	reported	8KoqAI
4	1E+08	A	G	0.044323	0.006263	1.50E-12	0.062091	462346	NA	rs1553432	NA	UKB-b-57	TRUE	reported	8KoqAI
4	1E+08	G	A	0.072285	0.009609	5.40E-14	0.025341	462346	NA	rs1553433	NA	UKB-b-57	TRUE	reported	8KoqAI
4	1E+08	C	T	0.045314	0.006259	4.50E-13	0.062213	462346	NA	rs7962382	NA	UKB-b-57	TRUE	reported	8KoqAI
4	1E+08	T	C	0.03413	0.005621	1.30E-09	0.078693	462346	NA	rs2083685	NA	UKB-b-57	TRUE	reported	8KoqAI
4	1E+08	T	G	0.042646	0.006213	6.70E-12	0.063227	462346	NA	rs7290674	NA	UKB-b-57	TRUE	reported	8KoqAI
4	1E+08	T	C	0.073507	0.009561	1.50E-14	0.025624	462346	NA	rs7572009	NA	UKB-b-57	TRUE	reported	8KoqAI
4	1E+08	A	T	0.044287	0.006266	1.60E-12	0.062046	462346	NA	rs1129533	NA	UKB-b-57	TRUE	reported	8KoqAI
4	1E+08	C	A	0.072174	0.009609	5.90E-14	0.025344	462346	NA	rs1136682	NA	UKB-b-57	TRUE	reported	8KoqAI
4	1E+08	A	G	0.044301	0.006266	1.60E-12	0.062044	462346	NA	rs1127001	NA	UKB-b-57	TRUE	reported	8KoqAI
4	1E+08	G	A	0.042237	0.006228	1.20E-11	0.06291	462346	NA	rs7615638	NA	UKB-b-57	TRUE	reported	8KoqAI
4	1E+08	T	G	0.072186	0.009609	5.80E-14	0.025344	462346	NA	rs1115103	NA	UKB-b-57	TRUE	reported	8KoqAI
4	1E+08	G	C	0.042165	0.006228	1.30E-11	0.062916	462346	NA	rs7290674	NA	UKB-b-57	TRUE	reported	8KoqAI
4	1E+08	G	C	0.034169	0.005623	1.20E-09	0.078653	462346	NA	rs1250926	NA	UKB-b-57	TRUE	reported	8KoqAI
4	1E+08	A	G	0.044373	0.006268	1.40E-12	0.062037	462346	NA	rs7647897	NA	UKB-b-57	TRUE	reported	8KoqAI
4	1E+08	A	G	0.031931	0.005658	1.70E-08	0.077603	462346	NA	rs7862575	NA	UKB-b-57	TRUE	reported	8KoqAI
4	1E+08	G	A	0.04438	0.006268	1.40E-12	0.062034	462346	NA	rs7747191	NA	UKB-b-57	TRUE	reported	8KoqAI
4	1E+08	C	T	0.042767	0.006234	6.90E-12	0.062802	462346	NA	rs7290674	NA	UKB-b-57	TRUE	reported	8KoqAI
4	1E+08	G	A	0.044341	0.006267	1.50E-12	0.062041	462346	NA	rs1139029	NA	UKB-b-57	TRUE	reported	8KoqAI
4	1E+08	C	T	0.042268	0.006229	1.20E-11	0.062906	462346	NA	rs7290674	NA	UKB-b-57	TRUE	reported	8KoqAI
4	1E+08	G	A	0.034118	0.005624	1.30E-09	0.078649	462346	NA	rs1503775	NA	UKB-b-57	TRUE	reported	8KoqAI
4	1E+08	A	G	0.072222	0.00961	5.70E-14	0.025344	462346	NA	rs1125311	NA	UKB-b-57	TRUE	reported	8KoqAI
4	1E+08	T	C	0.042324	0.00623	1.10E-11	0.062902	462346	NA	rs7290675	NA	UKB-b-57	TRUE	reported	8KoqAI
4	1E+08	G	A	0.034125	0.005623	1.30E-09	0.078661	462346	NA	rs7669241	NA	UKB-b-57	TRUE	reported	8KoqAI
4	1E+08	T	C	0.042276	0.00623	1.20E-11	0.062904	462346	NA	rs7567010	NA	UKB-b-57	TRUE	reported	8KoqAI
4	1E+08	T	C	0.072235	0.00961	5.60E-14	0.025345	462346	NA	rs7890872	NA	UKB-b-57	TRUE	reported	8KoqAI
4	1E+08	A	T	0.072237	0.00961	5.60E-14	0.025345	462346	NA	rs7710788	NA	UKB-b-57	TRUE	reported	8KoqAI
4	1E+08	T	C	0.042282	0.00623	1.10E-11	0.062904	462346	NA	rs7290675	NA	UKB-b-57	TRUE	reported	8KoqAI
4	1E+08	T	C	0.072252	0.00961	5.50E-14	0.025345	462346	NA	rs1127902	NA	UKB-b-57	TRUE	reported	8KoqAI
4	1E+08	C	G	0.044387	0.006268	1.40E-12	0.062035	462346	NA	rs1132275	NA	UKB-b-57	TRUE	reported	8KoqAI
4	1E+08	A	T	0.044396	0.006269	1.40E-12	0.062033	462346	NA	rs7482660	NA	UKB-b-57	TRUE	reported	8KoqAI
4	1E+08	G	A	0.044399	0.006269	1.40E-12	0.062033	462346	NA	rs7567462	NA	UKB-b-57	TRUE	reported	8KoqAI
4	1E+08	A	C	0.044403	0.006269	1.40E-12	0.062032	462346	NA	rs7851074	NA	UKB-b-57	TRUE	reported	8KoqAI
4	1E+08	A	G	0.072281	0.00961	5.40E-14	0.025345	462346	NA	rs1124301	NA	UKB-b-57	TRUE	reported	8KoqAI
4	1E+08	T	A	0.072274	0.00961	5.50E-14	0.025345	462346	NA	rs1407021	NA	UKB-b-57	TRUE	reported	8KoqAI
4	1E+08	G	A	0.042849	0.006228	6.00E-12	0.062962	462346	NA	rs1847370	NA	UKB-b-57	TRUE	reported	8KoqAI
4	1E+08	T	C	0.042853	0.006228	6.00E-12	0.062959	462346	NA	rs1847371	NA	UKB-b-57	TRUE	reported	8KoqAI
4	1E+08	C	T	0.042855	0.006228	5.90E-12	0.06296	462346	NA	rs1847372	NA	UKB-b-57	TRUE	reported	8KoqAI
4	1E+08	T	A	0.042858	0.006228	5.90E-12	0.062957	462346	NA	rs1847373	NA	UKB-b-57	TRUE	reported	8KoqAI
4	1E+08	T	C	0.042327	0.006232	1.10E-11	0.062879	462346	NA	rs7290676	NA	UKB-b-57	TRUE	reported	8KoqAI
4	1E+08	A	G	0.04232	0.006232	1.10E-11	0.062873	462346	NA	rs7290676	NA	UKB-b-57	TRUE	reported	8KoqAI
4	1E+08	G	A	0.071965	0.009609	6.90E-14	0.025351	462346	NA	rs1121020	NA	UKB-b-57	TRUE	reported	8KoqAI
4	1E+08	A	G	0.071307	0.009606	1.10E-13	0.025373	462346	NA	rs1123032	NA	UKB-b-57	TRUE	reported	8KoqAI
4	1E+08	G	A	0.070756	0.009612	1.80E-13	0.025352	462346	NA	rs1127242	NA	UKB-b-57	TRUE	reported	8KoqAI
4	1E+08	T	C	0.069467	0.00957	3.90E-13	0.025634	462346	NA	rs1119392	NA	UKB-b-57	TRUE	reported	8KoqAI
4	1E+08	T	C	0.078442	0.010068	6.60E-15	0.023091	462346	NA	rs7290677	NA	UKB-b-57	TRUE	reported	8KoqAI
4	1E+08	C	T	0.082272	0.010226	8.60E-16	0.022321	462346	NA	rs1121144	NA	UKB-b-57	TRUE	reported	8KoqAI
4	1E+08	G	C	0.082207	0.010225	9.00E-16	0.022318	462346	NA	rs1133896	NA	UKB-b-57	TRUE	reported	8KoqAI
4	1E+08	A	G	0.082079	0.010225	9.90E-16	0.022316	462346	NA	rs4127570	NA	UKB-b-57	TRUE	reported	8KoqAI
4	1E+08	C	T	0.074701	0.009876	3.90E-14	0.024116	462346	NA	rs1123527	NA	UKB-b-57	TRUE	reported	8KoqAI
4	1E+08	C	T	0.214284	0.016469	1.10E-38	0.008454	462346	NA	rs1887262	NA	UKB-b-57	TRUE	reported	8KoqAI
4	1E+08	T	C	0.082129	0.010225	9.50E-16	0.022315	462346	NA	rs1113071	NA	UKB-b-57	TRUE	reported	8KoqAI
4	1E+08	A	G	0.082129	0.010225	9.60E-16	0.022315	462346	NA	rs1125274	NA	UKB-b-57	TRUE	reported	8KoqAI
4	1E+08	A	G	0.082129	0.010224	9.50E-16	0.022314	462346	NA	rs4127570	NA	UKB-b-57	TRUE	reported	8KoqAI
4	1E+08	T	C	0.082129	0.010224	9.50E-16	0.022314	462346	NA	rs1450140	NA	UKB-b-57	TRUE	reported	8KoqAI
4	1E+08	C	T	0.082126	0.010224	9.60E-16	0.022314	462346	NA	rs1130193	NA	UKB-b-57	TRUE	reported	8KoqAI
4	1E+08	T	G	0.214655	0.01648	8.80E-39	0.008438	462346	NA	rs5436693	NA	UKB-b-57	TRUE	reported	8KoqAI
4	1E+08	T	G	0.082098	0.010225	9.80E-16	0.022315	462346	NA	rs1129547	NA	UKB-b-57	TRUE	reported	8KoqAI
4	1E+08	A	C	0.215016	0.016474	6.20E-39	0.008449	462346	NA	rs1478253	NA	UKB-b-57	TRUE	reported	8KoqAI
4	1E+08	T	G	0.082092	0.010225	9.80E-16	0.022316	462346	NA	rs1116943	NA	UKB-b-57	TRUE	reported	8KoqAI
4	1E+08	A	G	0.082092	0.010225	9.80E-16	0.022316	462346	NA	rs1125763	NA	UKB-b-57	TRUE	reported	8KoqAI
4	1E+08	A	T	0.08209	0.010225	9.90E-16	0.022316	462346	NA	rs7586008	NA	UKB-b-57	TRUE	reported	8KoqAI

4	1E+08	T	C	0.082206	0.010224	9.00E-16	0.022323	462346	NA	rs1912538	NA	UKB-b-57	TRUE	reported	8KoqAI
4	1E+08	G	A	0.081965	0.010225	1.10E-15	0.022315	462346	NA	rs1130341	NA	UKB-b-57	TRUE	reported	8KoqAI
4	1E+08	A	G	0.081963	0.010225	1.10E-15	0.022315	462346	NA	rs1117872	NA	UKB-b-57	TRUE	reported	8KoqAI
4	1E+08	T	C	0.081709	0.010223	1.30E-15	0.022324	462346	NA	rs7600614	NA	UKB-b-57	TRUE	reported	8KoqAI
4	1.01E+08	G	A	0.081904	0.010221	1.10E-15	0.022318	462346	NA	rs1512537	NA	UKB-b-57	TRUE	reported	8KoqAI
4	1.01E+08	A	G	0.081831	0.010224	1.20E-15	0.022308	462346	NA	rs1132874	NA	UKB-b-57	TRUE	reported	8KoqAI
4	1.01E+08	T	C	0.08172	0.010216	1.30E-15	0.022324	462346	NA	rs1135574	NA	UKB-b-57	TRUE	reported	8KoqAI
4	1.01E+08	G	A	0.081712	0.010215	1.30E-15	0.022326	462346	NA	rs1443195	NA	UKB-b-57	TRUE	reported	8KoqAI
4	1.01E+08	C	T	0.081558	0.010217	1.40E-15	0.022369	462346	NA	rs1487660	NA	UKB-b-57	TRUE	reported	8KoqAI
4	1.01E+08	C	T	0.081711	0.010215	1.30E-15	0.022326	462346	NA	rs1508499	NA	UKB-b-57	TRUE	reported	8KoqAI
4	1.01E+08	T	A	0.081687	0.010216	1.30E-15	0.022324	462346	NA	rs2130906	NA	UKB-b-57	TRUE	reported	8KoqAI
4	1.01E+08	C	A	0.081707	0.010216	1.30E-15	0.022323	462346	NA	rs2130907	NA	UKB-b-57	TRUE	reported	8KoqAI
4	1.01E+08	T	G	0.081566	0.010218	1.40E-15	0.022318	462346	NA	rs7729080	NA	UKB-b-57	TRUE	reported	8KoqAI
4	1.01E+08	A	G	0.081629	0.010217	1.40E-15	0.022318	462346	NA	rs2035813	NA	UKB-b-57	TRUE	reported	8KoqAI
4	1.01E+08	G	A	0.081735	0.010216	1.20E-15	0.022322	462346	NA	rs1458556	NA	UKB-b-57	TRUE	reported	8KoqAI
4	1.01E+08	C	T	0.081912	0.010221	1.10E-15	0.022293	462346	NA	rs1121258	NA	UKB-b-57	TRUE	reported	8KoqAI
4	1.01E+08	C	T	0.082017	0.01022	1.00E-15	0.022297	462346	NA	rs1130057	NA	UKB-b-57	TRUE	reported	8KoqAI
4	1.01E+08	A	G	0.082003	0.01022	1.00E-15	0.022298	462346	NA	rs1115365	NA	UKB-b-57	TRUE	reported	8KoqAI
4	1.01E+08	C	T	0.08188	0.01022	1.10E-15	0.022294	462346	NA	rs1125689	NA	UKB-b-57	TRUE	reported	8KoqAI
4	1.01E+08	G	A	0.080666	0.010203	2.70E-15	0.022365	462346	NA	rs1133379	NA	UKB-b-57	TRUE	reported	8KoqAI
4	1.01E+08	C	T	0.081836	0.01022	1.20E-15	0.022302	462346	NA	rs7818909	NA	UKB-b-57	TRUE	reported	8KoqAI
4	1.01E+08	C	T	0.08187	0.01022	1.10E-15	0.022303	462346	NA	rs7914982	NA	UKB-b-57	TRUE	reported	8KoqAI
4	1.01E+08	G	A	0.08205	0.010218	9.80E-16	0.02231	462346	NA	rs1122053	NA	UKB-b-57	TRUE	reported	8KoqAI
4	1.01E+08	A	G	0.082084	0.01022	9.60E-16	0.02231	462346	NA	rs1138802	NA	UKB-b-57	TRUE	reported	8KoqAI
4	1.01E+08	G	C	0.082192	0.010221	8.90E-16	0.022312	462346	NA	rs1124076	NA	UKB-b-57	TRUE	reported	8KoqAI
4	1.01E+08	G	T	0.082218	0.010222	8.80E-16	0.022309	462346	NA	rs1129250	NA	UKB-b-57	TRUE	reported	8KoqAI
4	1.01E+08	G	T	0.08227	0.010223	8.50E-16	0.022308	462346	NA	rs1510949	NA	UKB-b-57	TRUE	reported	8KoqAI
4	1.01E+08	A	G	0.084927	0.01028	1.40E-16	0.022088	462346	NA	rs1134337	NA	UKB-b-57	TRUE	reported	8KoqAI
4	1.01E+08	A	T	0.084962	0.010279	1.40E-16	0.022089	462346	NA	rs7966077	NA	UKB-b-57	TRUE	reported	8KoqAI
4	1.01E+08	C	A	0.08387	0.010298	3.80E-16	0.022005	462346	NA	rs1120421	NA	UKB-b-57	TRUE	reported	8KoqAI
4	1.01E+08	G	A	0.085029	0.010281	1.30E-16	0.022087	462346	NA	rs8014766	NA	UKB-b-57	TRUE	reported	8KoqAI
4	1.01E+08	G	A	0.087121	0.010361	4.10E-17	0.02181	462346	NA	rs7912165	NA	UKB-b-57	TRUE	reported	8KoqAI
4	1.01E+08	C	A	0.086716	0.010367	6.00E-17	0.021783	462346	NA	rs1496038	NA	UKB-b-57	TRUE	reported	8KoqAI
4	1.01E+08	A	G	0.086734	0.010367	5.90E-17	0.021783	462346	NA	rs1134991	NA	UKB-b-57	TRUE	reported	8KoqAI
4	1.01E+08	G	A	0.087397	0.010372	3.60E-17	0.02176	462346	NA	rs1118118	NA	UKB-b-57	TRUE	reported	8KoqAI
4	1.01E+08	C	G	0.087426	0.010372	3.50E-17	0.021759	462346	NA	rs1120372	NA	UKB-b-57	TRUE	reported	8KoqAI
4	1.01E+08	T	C	0.087438	0.010373	3.50E-17	0.021757	462346	NA	rs8025152	NA	UKB-b-57	TRUE	reported	8KoqAI
4	1.01E+08	A	G	0.087741	0.010371	2.70E-17	0.021797	462346	NA	rs1130464	NA	UKB-b-57	TRUE	reported	8KoqAI
4	1.01E+08	G	A	0.088505	0.010336	1.10E-17	0.021905	462346	NA	rs1121441	NA	UKB-b-57	TRUE	reported	8KoqAI
4	1.01E+08	C	T	0.08861	0.010335	1.00E-17	0.021904	462346	NA	rs1138368	NA	UKB-b-57	TRUE	reported	8KoqAI
4	1.01E+08	G	T	0.084584	0.010196	1.10E-16	0.022519	462346	NA	rs7290875	NA	UKB-b-57	TRUE	reported	8KoqAI
4	1.01E+08	T	G	0.084129	0.010222	1.90E-16	0.022423	462346	NA	rs7290876	NA	UKB-b-57	TRUE	reported	8KoqAI
4	1.01E+08	C	T	0.088742	0.010364	1.10E-17	0.021777	462346	NA	rs1137954	NA	UKB-b-57	TRUE	reported	8KoqAI
4	1.01E+08	G	A	0.088949	0.01038	1.00E-17	0.021696	462346	NA	rs1126731	NA	UKB-b-57	TRUE	reported	8KoqAI
4	1.01E+08	G	A	0.218925	0.016604	1.10E-39	0.008274	462346	NA	rs1489603	NA	UKB-b-57	TRUE	reported	8KoqAI
4	1.01E+08	G	A	0.090549	0.010481	5.70E-18	0.021537	462346	NA	rs1124162	NA	UKB-b-57	TRUE	reported	8KoqAI
4	1.01E+08	A	G	0.089139	0.010379	8.80E-18	0.021698	462346	NA	rs1127715	NA	UKB-b-57	TRUE	reported	8KoqAI
4	1.01E+08	A	C	0.083801	0.01025	2.90E-16	0.022259	462346	NA	rs7291062	NA	UKB-b-57	TRUE	reported	8KoqAI
4	1.01E+08	T	C	0.088257	0.010393	2.00E-17	0.021628	462346	NA	rs1131420	NA	UKB-b-57	TRUE	reported	8KoqAI
4	1.01E+08	T	A	0.088292	0.010392	2.00E-17	0.021629	462346	NA	rs1424291	NA	UKB-b-57	TRUE	reported	8KoqAI
4	1.01E+08	G	A	0.218238	0.016644	2.80E-39	0.008207	462346	NA	rs1508487	NA	UKB-b-57	TRUE	reported	8KoqAI
4	1.01E+08	G	A	0.088401	0.010391	1.80E-17	0.021625	462346	NA	rs1131369	NA	UKB-b-57	TRUE	reported	8KoqAI
4	1.01E+08	C	T	0.088368	0.010391	1.80E-17	0.02163	462346	NA	rs1126263	NA	UKB-b-57	TRUE	reported	8KoqAI
4	1.01E+08	G	A	0.088368	0.01039	1.80E-17	0.02163	462346	NA	rs1127901	NA	UKB-b-57	TRUE	reported	8KoqAI
4	1.01E+08	A	T	0.088491	0.010391	1.60E-17	0.021626	462346	NA	rs7476423	NA	UKB-b-57	TRUE	reported	8KoqAI
4	1.01E+08	T	G	0.088436	0.01039	1.70E-17	0.02163	462346	NA	rs1129663	NA	UKB-b-57	TRUE	reported	8KoqAI
4	1.01E+08	A	G	0.088534	0.01039	1.60E-17	0.021626	462346	NA	rs1451173	NA	UKB-b-57	TRUE	reported	8KoqAI
4	1.01E+08	T	C	0.08877	0.010386	1.30E-17	0.021628	462346	NA	rs1139527	NA	UKB-b-57	TRUE	reported	8KoqAI
4	1.01E+08	A	T	0.088663	0.010389	1.40E-17	0.02162	462346	NA	rs1130130	NA	UKB-b-57	TRUE	reported	8KoqAI
4	1.01E+08	G	A	0.088961	0.010397	1.20E-17	0.021606	462346	NA	rs7954802	NA	UKB-b-57	TRUE	reported	8KoqAI
4	1.01E+08	C	A	0.195078	0.019182	2.70E-24	0.006325	462346	NA	rs1402801	NA	UKB-b-57	TRUE	reported	8KoqAI
4	1.01E+08	C	T	0.191215	0.018986	7.40E-24	0.006416	462346	NA	rs1502083	NA	UKB-b-57	TRUE	reported	8KoqAI
4	1.01E+08	C	T	0.187636	0.018786	1.70E-23	0.006581	462346	NA	rs1483821	NA	UKB-b-57	TRUE	reported	8KoqAI
4	1.01E+08	C	T	0.198027	0.019626	6.10E-24	0.006174	462346	NA	rs1500214	NA	UKB-b-57	TRUE	reported	8KoqAI
4	1.01E+08	C	T	0.206718	0.021072	1.00E-22	0.005509	462346	NA	rs1827893	NA	UKB-b-57	TRUE	reported	8KoqAI
4	1.01E+08	T	C	0.203977	0.021153	5.30E-22	0.005485	462346	NA	rs1495804	NA	UKB-b-57	TRUE	reported	8KoqAI
4	1.01E+08	G	C	0.191637	0.022682	2.90E-17	0.004627	462346	NA	rs1885143	NA	UKB-b-57	TRUE	reported	8KoqAI
4	1.01E+08	T	A	0.174982	0.027366	1.60E-10	0.003289	462346	NA	rs1855276	NA	UKB-b-57	TRUE	reported	8KoqAI
4	1.01E+08	C	T	0.165147	0.029012	1.30E-08	0.002821	462346	NA	rs1927038	NA	UKB-b-57	TRUE	reported	8KoqAI
4	1.03E+08	G	T	0.039239	0.006535	1.90E-09	0.060452	462346	NA	rs1813006	NA	UKB-b-57	TRUE	reported	8KoqAI
4	1.03E+08	A	G	0.03597	0.005753	4.10E-10	0.081097	462346	NA	rs6855246	NA	UKB-b-57	TRUE	reported	8KoqAI
4	1.03E+08	A	C	0.041457	0.005639	2.00E-13	0.083292	462346	NA	rs3522520	NA	UKB-b-57	TRUE	reported	8KoqAI
4	1.03E+08	A	T	0.042014	0.005629	8.40E-14	0.08346	462346	NA	rs3551836	NA	UKB-b-57	TRUE	reported	8KoqAI
4	1.03E+08	C	T	0.044804	0.005754	6.90E-15	0.074923	462346	NA	rs1310732	NA	UKB-b-57	TRUE	reported	8KoqAI
4	1.03E+08	A	G	0.043834	0.005499	1.60E-15	0.083483	462346	NA	rs1313509	NA	UKB-b-57	TRUE	reported	8KoqAI
4	1.03E+08	C	T	0.039483	0.005657	3.00E-12	0.079424	462346	NA	rs1310558	NA	UKB-b-57	TRUE	reported	8KoqAI
4	1.03E+08	A	G	0.039167	0.00589	2.90E-11	0.071118	462346	NA	rs3433316	NA	UKB-b-57	TRUE	reported	8KoqAI
4	1.03E+08	C	T	0.036582	0.005549	4.30E-11	0.081286	462346	NA	rs1311446	NA	UKB-b-57	TRUE	reported	8KoqAI
4	1.03E+08	C	T	0.03664	0.005549	4.00E-11	0.081308	462346	NA	rs1311473	NA	UKB-b-57	TRUE	reported	8KoqAI
4	1.03E+08	G	C	0.036586	0.005549	4.30E-11	0.081287	462346	NA	rs1313568	NA	UKB-b-57	TRUE	reported	8KoqAI
4	1.03E+08	G	A	0.036646	0.005549	4.00E-11	0.081309	462346	NA	rs1314323	NA	UKB-b-57	TRUE	reported	8KoqAI
4	1.03E+08	T	C	0.03794	0.005751	4.20E-11	0.075373	462346	NA	rs1310927	NA	UKB-b-57	TRUE	reported	8KoqAI
4	1.03E+08	C	T	0.036268	0.005593	8.90E-11	0.080232	462346	NA	rs7162162	NA	UKB-b-57	TRUE	reported	8KoqAI
4	1.03E+08	G	A	0.034983	0.005359	6.70E-11	0.08823	462346	NA	rs1314003	NA	UKB-b-57	TRUE	reported	8KoqAI
4	1.03E+08	G	A	0.034854	0.005379	9.20E-11	0.088183	462346	NA	rs1311638	NA	UKB-b-57	TRUE	reported	8KoqAI
4	1.03E+08	G	A	0.035636	0.005435	5.50E-11	0.086328	462346	NA	rs1312995	NA	UKB-b-57	TRUE	reported	8KoqAI
4	1.03E+08	G	A	0.03474	0.005383	1.10E-10	0.088253	462346	NA	rs1585606	NA	UKB-b-57	TRUE	reported	8KoqAI
4	1.03E+08	T	C	0.035547	0.005439	6.30E-11	0.086383	462346	NA	rs1310751	NA	UKB-b-57	TRUE	reported	8KoqAI
4	1.03E+08	C	T	0.035113	0.005437	1.10E-10	0.088052	462346	NA	rs3431688	NA	UKB-b-57	TRUE	reported	8KoqAI
4	1.03E+08	T	C	0.032155	0.005186	5.60E-10	0.10878	462346	NA	rs230489	NA	UKB-b-57	TRUE	reported	8KoqAI
4	1.36E+08	T	C	-0.01763	0.003137	1.90E-08	0.616766	462346	NA	rs1311293	NA	UKB-b-57	TRUE	reported	8KoqAI
4	1.36E+08	C	T	-0.01774	0.003122	1.30E-08	0.616927	462346	NA	rs1311285	NA	UKB-b-57	TRUE	reported	8KoqAI
4	1.36E+08	A	G	-0.01783	0.00312	1.10E-08	0.616847	462346	NA	rs1043406	NA	UKB-b-57	TRUE	reported	8KoqAI
4	1.36E+08	C	T	-0.01809	0.003109	5.90E-09	0.613361	462346	NA	rs1310371	NA	UKB-b-57	TRUE	reported	8KoqAI
4	1.36E+08	A	G	-0.01795	0.003109	7.70E-09	0.613455	462346	NA	rs1310395	NA	UKB-b-57	TRUE	reported	8KoqAI
4	1.36E+08	T	C	-0.01793	0.003109	8.00E-09	0.613416	462346	NA	rs1315199	NA	UKB-b-57	TRUE	reported	8KoqAI
4	1.36E+08	G	C	-0.01797	0.003109	7.50E-09	0.613565	462346	NA	rs1250184	NA	UKB-b-57	TRUE	reported	8KoqAI
4	1.36E+08	T	G	-0.01801	0.003109	6.90E-09	0.613426	462346	NA	rs1250660	NA	UKB-b-57	TRUE	reported	8KoqAI
4	1.36E+08	A	G	-0.018	0.003109	7.10E-09	0.613455	462346	NA	rs1109929	NA	UKB-b-57	TRUE	reported	8KoqAI
4	1.36E+08	G	C	-0.01804	0.003109	6.50E-09	0.61348	462346	NA	rs1109929	NA	UKB-b-57	TRUE	reported	8KoqAI
4	1.36E+08	C	T	-0.01804	0.003109	6.50E-09	0.61348	462346	NA	rs1250380	NA	UKB-b-57	TRUE	reported	8KoqAI
4	1.36E+08	G	A	-0.01805	0.003109	6.40E-09	0.613468	462346	NA	rs1314087	NA	UKB-b-57	TRUE	reported	8KoqAI
4	1.36E+08	T	C	-0.01809	0.00311	6.00E-09	0.613407	462346	NA	rs4561977	NA	UKB-b-57	TRUE	reported	8KoqAI
4	1.36E+08	C	G	-0.01809	0.003109	6.00E-09	0.613477	462346	NA	rs4331828	NA	UKB-b-57	TRUE	reported	8KoqAI
4	1.36E+08	A	G	-0.01814	0.003109	5.40E-09	0.6135	462346	NA	rs4395564	NA	UKB-b-57	TRUE	reported	8KoqAI
4	1.36E+08	G	T	-0.01803	0.003114	7.00E-09	0.611904	462346	NA	rs4640720	NA	UKB-b-57	TRUE	reported	8KoqAI
4	1.36E+08	C	T	-0.01801	0.003114	7.20E-09	0.611787	462346	NA	rs4469136	NA	UKB-b-57	TRUE	reported	8KoqAI

4	1.36E+08	G	T	-0.01812	0.003109	5.70E-09	0.613458	462346	NA	rs1173102	NA	UKB-b-57	TRUE	reported	8KoqAl
4	1.36E+08	T	C	-0.01813	0.003109	5.50E-09	0.613457	462346	NA	rs1173538	NA	UKB-b-57	TRUE	reported	8KoqAl
4	1.36E+08	A	T	-0.01813	0.003109	5.50E-09	0.613461	462346	NA	rs1172274	NA	UKB-b-57	TRUE	reported	8KoqAl
4	1.36E+08	G	A	-0.01848	0.003109	2.70E-09	0.613067	462346	NA	rs7656273	NA	UKB-b-57	TRUE	reported	8KoqAl
4	1.36E+08	T	C	-0.0185	0.003108	2.60E-09	0.613057	462346	NA	rs1313419	NA	UKB-b-57	TRUE	reported	8KoqAl
4	1.36E+08	A	T	-0.01851	0.003108	2.60E-09	0.613066	462346	NA	rs1310662	NA	UKB-b-57	TRUE	reported	8KoqAl
4	1.36E+08	T	A	-0.01855	0.003108	2.40E-09	0.613206	462346	NA	rs6822196	NA	UKB-b-57	TRUE	reported	8KoqAl
4	1.36E+08	C	T	-0.01863	0.003108	2.00E-09	0.613132	462346	NA	rs7693757	NA	UKB-b-57	TRUE	reported	8KoqAl
4	1.36E+08	T	C	0.01832	0.003112	3.90E-09	0.385453	462346	NA	rs4864240	NA	UKB-b-57	TRUE	reported	8KoqAl
4	1.36E+08	T	C	-0.01865	0.003108	2.00E-09	0.613156	462346	NA	rs4280794	NA	UKB-b-57	TRUE	reported	8KoqAl
4	1.36E+08	T	A	-0.01863	0.003108	2.00E-09	0.613198	462346	NA	rs4280795	NA	UKB-b-57	TRUE	reported	8KoqAl
4	1.36E+08	A	C	-0.01864	0.003108	2.00E-09	0.613142	462346	NA	rs1312917	NA	UKB-b-57	TRUE	reported	8KoqAl
4	1.36E+08	A	G	-0.01866	0.003108	1.90E-09	0.613166	462346	NA	rs1312920	NA	UKB-b-57	TRUE	reported	8KoqAl
4	1.36E+08	A	G	0.01833	0.003111	3.80E-09	0.385355	462346	NA	rs1264382	NA	UKB-b-57	TRUE	reported	8KoqAl
4	1.36E+08	G	A	-0.01859	0.003108	2.20E-09	0.613098	462346	NA	rs5596758	NA	UKB-b-57	TRUE	reported	8KoqAl
4	1.36E+08	T	C	-0.01865	0.003108	1.90E-09	0.61313	462346	NA	rs1312624	NA	UKB-b-57	TRUE	reported	8KoqAl
4	1.36E+08	A	T	-0.0187	0.003108	1.80E-09	0.613159	462346	NA	rs4535381	NA	UKB-b-57	TRUE	reported	8KoqAl
4	1.36E+08	C	T	0.018349	0.003112	3.70E-09	0.385366	462346	NA	rs4437307	NA	UKB-b-57	TRUE	reported	8KoqAl
4	1.36E+08	A	G	-0.01866	0.003108	1.90E-09	0.613135	462346	NA	rs1173031	NA	UKB-b-57	TRUE	reported	8KoqAl
4	1.36E+08	C	T	0.018318	0.003111	3.90E-09	0.385621	462346	NA	rs4597893	NA	UKB-b-57	TRUE	reported	8KoqAl
4	1.36E+08	C	T	0.018313	0.003112	4.00E-09	0.385546	462346	NA	rs6858552	NA	UKB-b-57	TRUE	reported	8KoqAl
4	1.36E+08	A	C	-0.01872	0.003108	1.70E-09	0.612983	462346	NA	rs1314938	NA	UKB-b-57	TRUE	reported	8KoqAl
4	1.36E+08	G	A	0.018371	0.003112	3.60E-09	0.385534	462346	NA	rs4864244	NA	UKB-b-57	TRUE	reported	8KoqAl
4	1.36E+08	C	A	0.018592	0.003115	2.40E-09	0.386561	462346	NA	rs2887413	NA	UKB-b-57	TRUE	reported	8KoqAl
4	1.36E+08	A	G	0.018556	0.003115	2.60E-09	0.386584	462346	NA	rs2879992	NA	UKB-b-57	TRUE	reported	8KoqAl
4	1.36E+08	T	C	-0.01867	0.003108	1.90E-09	0.612957	462346	NA	rs4479760	NA	UKB-b-57	TRUE	reported	8KoqAl
4	1.36E+08	C	T	0.018372	0.003112	3.50E-09	0.385526	462346	NA	rs1043407	NA	UKB-b-57	TRUE	reported	8KoqAl
4	1.36E+08	C	T	-0.01864	0.003108	2.00E-09	0.613076	462346	NA	rs1249985	NA	UKB-b-57	TRUE	reported	8KoqAl
4	1.36E+08	G	A	0.018387	0.003112	3.40E-09	0.385516	462346	NA	rs4328967	NA	UKB-b-57	TRUE	reported	8KoqAl
4	1.36E+08	G	A	0.018398	0.003112	3.40E-09	0.385855	462346	NA	rs9998499	NA	UKB-b-57	TRUE	reported	8KoqAl
4	1.36E+08	T	G	0.018387	0.003112	3.40E-09	0.385516	462346	NA	rs4130840	NA	UKB-b-57	TRUE	reported	8KoqAl
4	1.36E+08	T	C	0.0184	0.003112	3.40E-09	0.385521	462346	NA	rs4130841	NA	UKB-b-57	TRUE	reported	8KoqAl
4	1.36E+08	C	T	0.018294	0.003112	4.10E-09	0.385555	462346	NA	rs7656631	NA	UKB-b-57	TRUE	reported	8KoqAl
4	1.36E+08	C	T	0.01843	0.003112	3.20E-09	0.3855	462346	NA	rs4629509	NA	UKB-b-57	TRUE	reported	8KoqAl
4	1.36E+08	G	T	-0.01864	0.003108	2.00E-09	0.612921	462346	NA	rs1314277	NA	UKB-b-57	TRUE	reported	8KoqAl
4	1.36E+08	A	C	0.018438	0.003112	3.10E-09	0.385487	462346	NA	rs6535050	NA	UKB-b-57	TRUE	reported	8KoqAl
4	1.36E+08	G	A	0.0184	0.003112	3.40E-09	0.385454	462346	NA	rs6535051	NA	UKB-b-57	TRUE	reported	8KoqAl
4	1.36E+08	G	A	-0.01855	0.003109	2.40E-09	0.612972	462346	NA	rs7656209	NA	UKB-b-57	TRUE	reported	8KoqAl
4	1.36E+08	T	C	0.018339	0.003113	3.80E-09	0.386003	462346	NA	rs6838419	NA	UKB-b-57	TRUE	reported	8KoqAl
4	1.36E+08	G	A	-0.01862	0.003109	2.10E-09	0.612522	462346	NA	rs7656595	NA	UKB-b-57	TRUE	reported	8KoqAl
4	1.36E+08	A	G	0.018417	0.003113	3.30E-09	0.385745	462346	NA	rs2880146	NA	UKB-b-57	TRUE	reported	8KoqAl
4	1.36E+08	T	A	0.018418	0.003113	3.30E-09	0.385831	462346	NA	rs2882693	NA	UKB-b-57	TRUE	reported	8KoqAl
4	1.36E+08	T	C	0.018209	0.003112	4.90E-09	0.385624	462346	NA	rs2876928	NA	UKB-b-57	TRUE	reported	8KoqAl
4	1.36E+08	G	T	-0.01871	0.003108	1.70E-09	0.612866	462346	NA	rs1109930	NA	UKB-b-57	TRUE	reported	8KoqAl
4	1.36E+08	A	T	-0.01873	0.003108	1.70E-09	0.612958	462346	NA	rs6853276	NA	UKB-b-57	TRUE	reported	8KoqAl
4	1.36E+08	G	T	-0.01883	0.003107	1.40E-09	0.612836	462346	NA	rs1313145	NA	UKB-b-57	TRUE	reported	8KoqAl
4	1.36E+08	G	T	-0.01879	0.003203	4.50E-09	0.663246	462346	NA	rs4429776	NA	UKB-b-57	TRUE	reported	8KoqAl
4	1.36E+08	C	T	-0.01864	0.003107	2.00E-09	0.612977	462346	NA	rs1310803	NA	UKB-b-57	TRUE	reported	8KoqAl
4	1.36E+08	A	G	-0.01867	0.00311	1.90E-09	0.612953	462346	NA	rs1312876	NA	UKB-b-57	TRUE	reported	8KoqAl
4	1.36E+08	C	G	-0.01857	0.003108	2.30E-09	0.613078	462346	NA	rs1109930	NA	UKB-b-57	TRUE	reported	8KoqAl
4	1.36E+08	A	G	-0.01856	0.003108	2.30E-09	0.612964	462346	NA	rs1109930	NA	UKB-b-57	TRUE	reported	8KoqAl
4	1.36E+08	T	C	-0.01846	0.003203	8.30E-09	0.663366	462346	NA	rs1250160	NA	UKB-b-57	TRUE	reported	8KoqAl
4	1.36E+08	G	A	0.018579	0.003111	2.30E-09	0.386196	462346	NA	rs7439938	NA	UKB-b-57	TRUE	reported	8KoqAl
4	1.36E+08	T	C	-0.01868	0.003207	5.70E-09	0.664295	462346	NA	rs5595111	NA	UKB-b-57	TRUE	reported	8KoqAl
4	1.36E+08	C	A	-0.01863	0.003109	2.10E-09	0.612208	462346	NA	rs1311266	NA	UKB-b-57	TRUE	reported	8KoqAl
4	1.36E+08	G	A	-0.0186	0.003205	6.50E-09	0.66315	462346	NA	rs7697346	NA	UKB-b-57	TRUE	reported	8KoqAl
4	1.36E+08	C	A	-0.01748	0.003099	1.70E-08	0.5879	462346	NA	rs1311922	NA	UKB-b-57	TRUE	reported	8KoqAl
4	1.36E+08	C	A	0.018645	0.003208	6.20E-09	0.33518	462346	NA	rs7442176	NA	UKB-b-57	TRUE	reported	8KoqAl
4	1.36E+08	A	G	0.018559	0.003206	7.10E-09	0.335971	462346	NA	rs7442212	NA	UKB-b-57	TRUE	reported	8KoqAl
4	1.36E+08	G	T	0.018582	0.003206	6.80E-09	0.33599	462346	NA	rs1021282	NA	UKB-b-57	TRUE	reported	8KoqAl
4	1.36E+08	G	A	-0.01875	0.003204	4.90E-09	0.66334	462346	NA	rs1312675	NA	UKB-b-57	TRUE	reported	8KoqAl
4	1.36E+08	C	T	0.018577	0.003206	6.90E-09	0.335976	462346	NA	rs17496	NA	UKB-b-57	TRUE	reported	8KoqAl
4	1.36E+08	C	T	-0.01871	0.003204	5.20E-09	0.66347	462346	NA	rs1312662	NA	UKB-b-57	TRUE	reported	8KoqAl
4	1.36E+08	T	C	0.018527	0.003206	7.50E-09	0.335871	462346	NA	rs1021313	NA	UKB-b-57	TRUE	reported	8KoqAl
4	1.36E+08	G	A	-0.01869	0.003203	5.40E-09	0.663486	462346	NA	rs1312765	NA	UKB-b-57	TRUE	reported	8KoqAl
4	1.36E+08	T	C	-0.01941	0.003119	4.90E-10	0.61881	462346	NA	rs1310297	NA	UKB-b-57	TRUE	reported	8KoqAl
4	1.36E+08	C	A	-0.01866	0.003204	5.70E-09	0.663532	462346	NA	rs1312756	NA	UKB-b-57	TRUE	reported	8KoqAl
4	1.36E+08	C	A	-0.01937	0.003119	5.30E-10	0.618876	462346	NA	rs1312757	NA	UKB-b-57	TRUE	reported	8KoqAl
4	1.36E+08	C	T	-0.01871	0.003203	5.20E-09	0.663481	462346	NA	rs7435952	NA	UKB-b-57	TRUE	reported	8KoqAl
4	1.36E+08	G	T	-0.01884	0.003203	4.00E-09	0.663455	462346	NA	rs4541577	NA	UKB-b-57	TRUE	reported	8KoqAl
4	1.36E+08	A	G	-0.01872	0.003205	5.20E-09	0.664226	462346	NA	rs4643872	NA	UKB-b-57	TRUE	reported	8KoqAl
4	1.36E+08	A	G	-0.01873	0.003205	5.10E-09	0.664178	462346	NA	rs1173589	NA	UKB-b-57	TRUE	reported	8KoqAl
4	1.36E+08	G	A	-0.01873	0.003205	5.10E-09	0.664183	462346	NA	rs4546304	NA	UKB-b-57	TRUE	reported	8KoqAl
4	1.36E+08	C	T	-0.0193	0.003121	6.30E-10	0.62026	462346	NA	rs4611981	NA	UKB-b-57	TRUE	reported	8KoqAl
4	1.36E+08	G	A	-0.01872	0.003207	5.30E-09	0.664284	462346	NA	rs1314780	NA	UKB-b-57	TRUE	reported	8KoqAl
4	1.36E+08	G	A	-0.01863	0.003204	6.10E-09	0.663396	462346	NA	rs1310453	NA	UKB-b-57	TRUE	reported	8KoqAl
4	1.36E+08	C	T	-0.01866	0.003204	5.80E-09	0.662899	462346	NA	rs1310466	NA	UKB-b-57	TRUE	reported	8KoqAl
4	1.36E+08	A	C	-0.01923	0.003122	7.30E-10	0.620349	462346	NA	rs4569802	NA	UKB-b-57	TRUE	reported	8KoqAl
4	1.36E+08	A	C	-0.01868	0.003207	5.70E-09	0.664235	462346	NA	rs4453983	NA	UKB-b-57	TRUE	reported	8KoqAl
4	1.36E+08	T	C	-0.01875	0.003207	5.00E-09	0.664306	462346	NA	rs1312264	NA	UKB-b-57	TRUE	reported	8KoqAl
4	1.36E+08	G	A	-0.01879	0.003208	4.70E-09	0.664187	462346	NA	rs4543187	NA	UKB-b-57	TRUE	reported	8KoqAl
4	1.36E+08	T	G	-0.01877	0.003208	4.90E-09	0.664315	462346	NA	rs4521398	NA	UKB-b-57	TRUE	reported	8KoqAl
4	1.36E+08	A	C	-0.01873	0.00321	5.30E-09	0.663701	462346	NA	rs1310349	NA	UKB-b-57	TRUE	reported	8KoqAl
4	1.36E+08	G	T	-0.01863	0.00321	6.50E-09	0.66303	462346	NA	rs1172269	NA	UKB-b-57	TRUE	reported	8KoqAl
4	1.36E+08	T	G	-0.0192	0.003126	8.20E-10	0.619133	462346	NA	rs1172699	NA	UKB-b-57	TRUE	reported	8KoqAl
4	1.36E+08	T	C	-0.01874	0.003211	5.30E-09	0.663552	462346	NA	rs1109930	NA	UKB-b-57	TRUE	reported	8KoqAl
4	1.36E+08	G	C	-0.01878	0.003212	5.10E-09	0.66313	462346	NA	rs1109930	NA	UKB-b-57	TRUE	reported	8KoqAl
4	1.36E+08	A	G	-0.01938	0.00313	6.00E-10	0.620409	462346	NA	rs9685153	NA	UKB-b-57	TRUE	reported	8KoqAl
4	1.36E+08	G	T	-0.01872	0.003215	5.80E-09	0.66311	462346	NA	rs1311907	NA	UKB-b-57	TRUE	reported	8KoqAl
4	1.36E+08	T	A	-0.01878	0.003215	5.20E-09	0.662987	462346	NA	rs7438900	NA	UKB-b-57	TRUE	reported	8KoqAl
4	1.36E+08	T	G	-0.01817	0.003328	4.70E-08	0.70542	462346	NA	rs1002198	NA	UKB-b-57	TRUE	reported	8KoqAl
4	1.85E+08	C	T	0.017547	0.003174	3.20E-08	0.631421	462346	NA	rs6815422	NA	UKB-b-57	TRUE	reported	8KoqAl
4	1.85E+08	A	T	0.017883	0.003171	1.70E-08	0.631802	462346	NA	rs3611573	NA	UKB-b-57	TRUE	reported	8KoqAl
4	1.85E+08	C	A	0.018294	0.003154	6.60E-09	0.626705	462346	NA	rs6233967	NA	UKB-b-57	TRUE	reported	8KoqAl
5	67824690	C	T	0.02254	0.004071	3.10E-08	0.16931	462346	NA	rs5619443	NA	UKB-b-57	TRUE	reported	8KoqAl
5	87176970	T	A	-0.01916	0.003502	4.50E-08	0.249792	462346	NA	rs710348	NA	UKB-b-57	TRUE	reported	8KoqAl
5	87190925	T	G	-0.01911	0.003501	4.80E-08	0.249776	462346	NA	rs6867687	NA	UKB-b-57	TRUE	reported	8KoqAl
5	87192117	G	T	-0.0191	0.003501	4.90E-08	0.249773	462346	NA	rs1350437	NA	UKB-b-57	TRUE	reported	8KoqAl
5	87237371	G	A	0.019473	0.003565	4.70E-08	0.762757	462346	NA	rs2037850	NA	UKB-b-57	TRUE	reported	8KoqAl
5	87237428	G	C	0.019509	0.003565	4.50E-08	0.762691	462346	NA	rs2037851	NA	UKB-b-57	TRUE	reported	8KoqAl
5	87237448	T	C	0.019511	0.003565	4.40E-08	0.762691	462346	NA	rs2037852	NA	UKB-b-57	TRUE	reported	8KoqAl
5	87238345	A	G	0.019463	0.003565	4.80E-08	0.762762	462346	NA	rs1534603	NA	UKB-b-57	TRUE	reported	8KoqAl
5	87239340	T	A	0.019462	0.003565	4.80E-08	0.762763	462346	NA	rs1997322	NA	UKB-b-57	TRUE	reported	8KoqAl
5	87239366	T	C	0.019462	0.003565	4.80E-08	0.762763	462346	NA	rs1997321	NA	UKB-b-57	TRUE	reported	8KoqAl
5	87242987	C	T	0.019441	0.003565	5.00E-08	0.762747	462346	NA	rs7722762	NA	UKB-b-57	TRUE	reported	8KoqAl
5	87243338	A	G	0.019448	0.003565	4.90E-08	0.762739	462346	NA	rs1406407	NA	UKB-b-57	TRUE	reported	8KoqAl
5	87244902	C	T	0.019431	0.003565	5.00E-08	0.762735	462346	NA	rs1046246	NA	UKB-b-57	TRUE	reported	8KoqAl

5	87245390	A	G	0.019475	0.003565	4.70E-08	0.762741	462346	NA	rs4916772	NA	UKB-b-57	TRUE	reported	8KoqAI
5	87245644	G	A	0.019468	0.003565	4.70E-08	0.762412	462346	NA	rs1720748	NA	UKB-b-57	TRUE	reported	8KoqAI
5	87253181	T	C	0.020393	0.003547	9.00E-09	0.759673	462346	NA	rs3422877	NA	UKB-b-57	TRUE	reported	8KoqAI
5	87254187	C	T	0.020489	0.003548	7.70E-09	0.759784	462346	NA	rs6875790	NA	UKB-b-57	TRUE	reported	8KoqAI
5	87256406	A	G	0.020487	0.003548	7.80E-09	0.759767	462346	NA	rs1569186	NA	UKB-b-57	TRUE	reported	8KoqAI
5	87258291	G	C	0.020692	0.003548	5.50E-09	0.759646	462346	NA	rs6868844	NA	UKB-b-57	TRUE	reported	8KoqAI
5	87259822	C	T	0.020479	0.003549	7.90E-09	0.75975	462346	NA	rs7715258	NA	UKB-b-57	TRUE	reported	8KoqAI
5	87268129	G	A	0.020489	0.003549	7.80E-09	0.759735	462346	NA	rs1252314	NA	UKB-b-57	TRUE	reported	8KoqAI
5	87277404	C	T	0.020694	0.003549	5.50E-09	0.7596	462346	NA	rs2410759	NA	UKB-b-57	TRUE	reported	8KoqAI
5	87281774	C	T	0.02164	0.003444	3.30E-10	0.735572	462346	NA	rs1046246	NA	UKB-b-57	TRUE	reported	8KoqAI
5	87284024	T	C	0.020698	0.00355	5.50E-09	0.759586	462346	NA	rs7735528	NA	UKB-b-57	TRUE	reported	8KoqAI
5	87284972	C	T	0.020492	0.00355	7.80E-09	0.759685	462346	NA	rs7720682	NA	UKB-b-57	TRUE	reported	8KoqAI
5	87287010	C	T	0.020366	0.00355	9.60E-09	0.759573	462346	NA	rs3988251	NA	UKB-b-57	TRUE	reported	8KoqAI
5	87289586	A	T	-0.02111	0.003842	3.90E-08	0.193193	462346	NA	rs7445186	NA	UKB-b-57	TRUE	reported	8KoqAI
5	87290221	C	A	-0.02336	0.004257	4.10E-08	0.149367	462346	NA	rs9986279	NA	UKB-b-57	TRUE	reported	8KoqAI
5	87291976	A	T	0.021364	0.003566	2.10E-09	0.762749	462346	NA	rs4296831	NA	UKB-b-57	TRUE	reported	8KoqAI
5	87292070	A	G	0.021385	0.003566	2.00E-09	0.762741	462346	NA	rs4295422	NA	UKB-b-57	TRUE	reported	8KoqAI
5	87297330	T	A	-0.0212	0.003843	3.40E-08	0.193084	462346	NA	rs6236951	NA	UKB-b-57	TRUE	reported	8KoqAI
5	87299472	A	T	-0.02119	0.003843	3.50E-08	0.193091	462346	NA	rs1004086	NA	UKB-b-57	TRUE	reported	8KoqAI
5	87299915	A	G	0.021386	0.003566	2.00E-09	0.762677	462346	NA	rs4529233	NA	UKB-b-57	TRUE	reported	8KoqAI
5	87301956	G	C	-0.02167	0.003955	4.30E-08	0.179777	462346	NA	rs3471297	NA	UKB-b-57	TRUE	reported	8KoqAI
5	87305744	T	A	0.021602	0.003576	1.50E-09	0.762772	462346	NA	rs7955379	NA	UKB-b-57	TRUE	reported	8KoqAI
5	87310165	G	A	0.021548	0.003575	1.70E-09	0.76254	462346	NA	rs9686201	NA	UKB-b-57	TRUE	reported	8KoqAI
5	87311113	G	A	0.021734	0.003574	1.20E-09	0.76245	462346	NA	rs1251486	NA	UKB-b-57	TRUE	reported	8KoqAI
5	87311560	C	T	0.021558	0.003575	1.60E-09	0.762541	462346	NA	rs1316405	NA	UKB-b-57	TRUE	reported	8KoqAI
5	87313988	T	G	-0.02142	0.003854	2.70E-08	0.193305	462346	NA	rs6236808	NA	UKB-b-57	TRUE	reported	8KoqAI
5	87316821	C	T	-0.02144	0.003854	2.60E-08	0.193325	462346	NA	rs1195452	NA	UKB-b-57	TRUE	reported	8KoqAI
5	87318587	A	T	-0.02145	0.003854	2.60E-08	0.193308	462346	NA	rs1195795	NA	UKB-b-57	TRUE	reported	8KoqAI
5	87319502	C	T	-0.02146	0.003854	2.60E-08	0.193304	462346	NA	rs6236808	NA	UKB-b-57	TRUE	reported	8KoqAI
5	87320219	G	A	0.021535	0.003576	1.70E-09	0.762524	462346	NA	rs3523249	NA	UKB-b-57	TRUE	reported	8KoqAI
5	87322568	T	C	0.021549	0.003576	1.70E-09	0.762528	462346	NA	rs1080585	NA	UKB-b-57	TRUE	reported	8KoqAI
5	87325202	T	C	0.021575	0.003576	1.60E-09	0.762554	462346	NA	rs3910029	NA	UKB-b-57	TRUE	reported	8KoqAI
5	87326916	A	G	0.018172	0.003329	4.80E-08	0.700983	462346	NA	rs3463953	NA	UKB-b-57	TRUE	reported	8KoqAI
5	87327401	T	G	0.021515	0.003577	1.80E-09	0.762581	462346	NA	rs1316552	NA	UKB-b-57	TRUE	reported	8KoqAI
5	87327886	C	T	-0.02134	0.003856	3.10E-08	0.193292	462346	NA	rs1006811	NA	UKB-b-57	TRUE	reported	8KoqAI
5	87328220	C	T	-0.02124	0.003855	3.60E-08	0.193512	462346	NA	rs6236809	NA	UKB-b-57	TRUE	reported	8KoqAI
5	87329176	A	G	-0.02181	0.003858	1.60E-08	0.19446	462346	NA	rs2866771	NA	UKB-b-57	TRUE	reported	8KoqAI
5	87329636	A	G	0.022781	0.003586	2.10E-10	0.763446	462346	NA	rs4916802	NA	UKB-b-57	TRUE	reported	8KoqAI
5	87333181	T	C	-0.02336	0.003986	4.60E-09	0.178103	462346	NA	rs1005670	NA	UKB-b-57	TRUE	reported	8KoqAI
5	87335745	C	T	-0.02339	0.003985	4.40E-09	0.178216	462346	NA	rs1005335	NA	UKB-b-57	TRUE	reported	8KoqAI
5	87338662	G	A	0.022739	0.003583	2.20E-10	0.763213	462346	NA	rs919451	NA	UKB-b-57	TRUE	reported	8KoqAI
5	87339384	G	A	-0.02332	0.003986	4.90E-09	0.178093	462346	NA	rs7700770	NA	UKB-b-57	TRUE	reported	8KoqAI
5	87339693	A	G	-0.02336	0.003986	4.60E-09	0.178214	462346	NA	rs7700867	NA	UKB-b-57	TRUE	reported	8KoqAI
5	87341486	G	A	-0.02322	0.00402	7.60E-09	0.17621	462346	NA	rs1144038	NA	UKB-b-57	TRUE	reported	8KoqAI
5	87343557	A	G	-0.02316	0.003986	6.30E-09	0.178202	462346	NA	rs2848458	NA	UKB-b-57	TRUE	reported	8KoqAI
5	87346722	G	C	0.022589	0.003583	2.90E-10	0.763101	462346	NA	rs7700887	NA	UKB-b-57	TRUE	reported	8KoqAI
5	87348352	C	T	0.01914	0.003396	1.70E-08	0.720129	462346	NA	rs4099928	NA	UKB-b-57	TRUE	reported	8KoqAI
5	87354220	C	G	-0.02298	0.003986	8.20E-09	0.178084	462346	NA	rs1007093	NA	UKB-b-57	TRUE	reported	8KoqAI
5	87355444	G	A	0.019294	0.003396	1.30E-08	0.720167	462346	NA	rs3866898	NA	UKB-b-57	TRUE	reported	8KoqAI
5	87357286	T	C	-0.02291	0.003987	9.20E-09	0.178022	462346	NA	rs737021	NA	UKB-b-57	TRUE	reported	8KoqAI
5	87362109	A	C	-0.02287	0.003987	9.70E-09	0.178105	462346	NA	rs1006151	NA	UKB-b-57	TRUE	reported	8KoqAI
5	87366829	G	A	-0.02496	0.004359	1.00E-08	0.143454	462346	NA	rs1005555	NA	UKB-b-57	TRUE	reported	8KoqAI
5	87368073	A	C	0.022724	0.003584	2.30E-10	0.76311	462346	NA	rs1835240	NA	UKB-b-57	TRUE	reported	8KoqAI
5	87371222	G	A	-0.02509	0.004362	8.90E-09	0.143328	462346	NA	rs1896892	NA	UKB-b-57	TRUE	reported	8KoqAI
5	87372106	A	G	0.022452	0.003584	3.70E-10	0.763166	462346	NA	rs1265757	NA	UKB-b-57	TRUE	reported	8KoqAI
5	87374361	G	A	-0.02285	0.003987	1.00E-08	0.178102	462346	NA	rs1044067	NA	UKB-b-57	TRUE	reported	8KoqAI
5	87378625	A	G	-0.02291	0.003989	9.30E-09	0.17797	462346	NA	rs4305667	NA	UKB-b-57	TRUE	reported	8KoqAI
5	87378699	C	G	-0.02292	0.003989	9.10E-09	0.177976	462346	NA	rs4579285	NA	UKB-b-57	TRUE	reported	8KoqAI
5	87384441	G	C	0.022497	0.003589	3.70E-10	0.76331	462346	NA	rs4916714	NA	UKB-b-57	TRUE	reported	8KoqAI
5	87385664	A	G	-0.02273	0.003981	1.10E-08	0.179249	462346	NA	rs1003795	NA	UKB-b-57	TRUE	reported	8KoqAI
5	87387291	A	G	0.019883	0.003337	2.50E-09	0.702221	462346	NA	rs1427921	NA	UKB-b-57	TRUE	reported	8KoqAI
5	87389057	G	C	-0.02311	0.003928	4.00E-09	0.182161	462346	NA	rs6452770	NA	UKB-b-57	TRUE	reported	8KoqAI
5	87391462	C	T	-0.02305	0.003923	4.20E-09	0.182393	462346	NA	rs2195270	NA	UKB-b-57	TRUE	reported	8KoqAI
5	87393746	A	G	-0.02207	0.003919	1.80E-08	0.184494	462346	NA	rs1814149	NA	UKB-b-57	TRUE	reported	8KoqAI
5	87399002	C	T	-0.02352	0.003933	2.20E-09	0.182027	462346	NA	rs1007998	NA	UKB-b-57	TRUE	reported	8KoqAI
5	87399503	T	C	-0.02361	0.003932	1.90E-09	0.182387	462346	NA	rs1346443	NA	UKB-b-57	TRUE	reported	8KoqAI
5	87399904	G	T	-0.02402	0.003984	1.60E-09	0.178759	462346	NA	rs6864178	NA	UKB-b-57	TRUE	reported	8KoqAI
5	87399919	A	G	-0.02392	0.003979	1.80E-09	0.179073	462346	NA	rs6863807	NA	UKB-b-57	TRUE	reported	8KoqAI
5	87401353	T	C	-0.02351	0.003934	2.30E-09	0.181949	462346	NA	rs6452772	NA	UKB-b-57	TRUE	reported	8KoqAI
5	87405162	G	A	-0.02356	0.003935	2.10E-09	0.181964	462346	NA	rs1335753	NA	UKB-b-57	TRUE	reported	8KoqAI
5	87406916	A	G	-0.02359	0.003935	2.00E-09	0.18203	462346	NA	rs6863354	NA	UKB-b-57	TRUE	reported	8KoqAI
5	87407636	C	T	-0.0236	0.003935	2.00E-09	0.182028	462346	NA	rs1834908	NA	UKB-b-57	TRUE	reported	8KoqAI
5	87411057	A	C	-0.02363	0.003936	1.90E-09	0.182024	462346	NA	rs4916888	NA	UKB-b-57	TRUE	reported	8KoqAI
5	87415177	A	G	-0.02363	0.003937	2.00E-09	0.182015	462346	NA	rs6452773	NA	UKB-b-57	TRUE	reported	8KoqAI
5	87416963	G	A	-0.02361	0.003938	2.00E-09	0.181987	462346	NA	rs1834912	NA	UKB-b-57	TRUE	reported	8KoqAI
5	87417211	C	T	-0.02363	0.003938	2.00E-09	0.181985	462346	NA	rs1837637	NA	UKB-b-57	TRUE	reported	8KoqAI
5	87417311	A	G	-0.02363	0.003938	2.00E-09	0.181987	462346	NA	rs1005169	NA	UKB-b-57	TRUE	reported	8KoqAI
5	87417635	C	T	-0.02365	0.003939	1.90E-09	0.181873	462346	NA	rs1006700	NA	UKB-b-57	TRUE	reported	8KoqAI
5	87420231	C	T	-0.02362	0.003938	2.00E-09	0.181979	462346	NA	rs7729079	NA	UKB-b-57	TRUE	reported	8KoqAI
5	87420907	A	G	-0.02359	0.003942	2.20E-09	0.18174	462346	NA	rs1007854	NA	UKB-b-57	TRUE	reported	8KoqAI
5	87421345	G	A	-0.02361	0.003939	2.00E-09	0.181903	462346	NA	rs1004013	NA	UKB-b-57	TRUE	reported	8KoqAI
5	87428897	T	C	-0.02352	0.003977	3.30E-09	0.179649	462346	NA	rs7448105	NA	UKB-b-57	TRUE	reported	8KoqAI
5	87429920	T	A	-0.02297	0.003941	5.60E-09	0.182028	462346	NA	rs6897265	NA	UKB-b-57	TRUE	reported	8KoqAI
5	87430302	A	G	-0.02564	0.004354	3.90E-09	0.142456	462346	NA	rs1728605	NA	UKB-b-57	TRUE	reported	8KoqAI
5	87430946	G	A	-0.02408	0.003959	1.20E-09	0.179883	462346	NA	rs1021425	NA	UKB-b-57	TRUE	reported	8KoqAI
5	87432547	G	A	-0.02407	0.003958	1.20E-09	0.180013	462346	NA	rs1332820	NA	UKB-b-57	TRUE	reported	8KoqAI
5	87433204	T	G	-0.02427	0.003967	9.40E-10	0.179589	462346	NA	rs1005601	NA	UKB-b-57	TRUE	reported	8KoqAI
5	87434054	A	G	-0.0241	0.003961	1.20E-09	0.179853	462346	NA	rs1816739	NA	UKB-b-57	TRUE	reported	8KoqAI
5	87434708	A	C	-0.02408	0.003962	1.20E-09	0.179838	462346	NA	rs1006558	NA	UKB-b-57	TRUE	reported	8KoqAI
5	87435486	T	C	-0.02404	0.003962	1.30E-09	0.179812	462346	NA	rs1051429	NA	UKB-b-57	TRUE	reported	8KoqAI
5	87435633	C	T	-0.02401	0.003962	1.40E-09	0.179917	462346	NA	rs1005262	NA	UKB-b-57	TRUE	reported	8KoqAI
5	87437079	G	A	0.020174	0.003225	4.00E-10	0.666752	462346	NA	rs1316878	NA	UKB-b-57	TRUE	reported	8KoqAI
5	87439691	C	T	0.018955	0.003279	7.50E-09	0.688181	462346	NA	rs4916889	NA	UKB-b-57	TRUE	reported	8KoqAI
5	87441181	G	C	-0.0192	0.003506	4.30E-08	0.249502	462346	NA	rs1005393	NA	UKB-b-57	TRUE	reported	8KoqAI
5	87442528	T	C	0.02251	0.003341	1.60E-11	0.707593	462346	NA	rs3555940	NA	UKB-b-57	TRUE	reported	8KoqAI
5	87443958	A	T	-0.02376	0.003604	4.40E-11	0.230365	462346	NA	rs6884911	NA	UKB-b-57	TRUE	reported	8KoqAI
5	87445490	A	G	0.018226	0.003278	2.70E-08	0.688825	462346	NA	rs1252267	NA	UKB-b-57	TRUE	reported	8KoqAI
5	87447687	T	A	0.018225	0.003278	2.70E-08	0.688828	462346	NA	rs1252179	NA	UKB-b-57	TRUE	reported	8KoqAI
5	87449984	A	G	-0.02372	0.003602	4.50E-11	0.230376	462346	NA	rs1004474	NA	UKB-b-57	TRUE	reported	8KoqAI
5	87454270	T	C	-0.02374	0.003602	4.40E-11	0.23034	462346	NA	rs1047427	NA	UKB-b-57	TRUE	reported	8KoqAI
5	87454387	G	C	-0.02374	0.003602	4.40E-11	0.23037	462346	NA	rs3936337	NA	UKB-b-57	TRUE	reported	8KoqAI
5	87454464	C	T	-0.02372	0.003602	4.50E-11	0.230334	462346	NA	rs1047427	NA	UKB-b-57	TRUE	reported	8KoqAI
5	87454999	C	T	0.020571	0.003131	5.00E-11	0.623256	462346	NA	rs5570422	NA	UKB-b-57	TRUE	reported	8KoqAI
5	87455013	G	T	0.020595	0.003131	4.70E-11	0.623326	462346	NA	rs5636871	NA	UKB-b-57	TRUE	reported	8KoqAI
5	87455655	A	G	0.02056	0.003129	5.00E-11	0.62411	462346	NA	rs1317097	NA	UKB-b-57	TRUE	reported	8KoqAI
5	87457198	A	G	-0.02365	0.003603	5.30E-11	0.230238	462346	NA	rs1335710	NA	UKB-b-57	TRUE	reported	8KoqAI
5	87458220	T	A	-0.02365	0.003603	5.30E-11	0.230237	462346	NA	rs1007536	NA	UKB-b-57	TRUE	reported	8KoqAI

5	87459042	C	T	0.020468	0.00313	6.20E-11	0.624346	462346	NA	rs1251998	NA	UKB-b-57	TRUE	reported	8KoqAI
5	87461867	G	A	0.020455	0.00313	6.40E-11	0.624347	462346	NA	rs1317539	NA	UKB-b-57	TRUE	reported	8KoqAI
5	87466769	G	T	-0.0237	0.003602	4.70E-11	0.230332	462346	NA	rs1335514	NA	UKB-b-57	TRUE	reported	8KoqAI
5	87467351	T	G	-0.02434	0.004383	2.80E-08	0.140066	462346	NA	rs2410760	NA	UKB-b-57	TRUE	reported	8KoqAI
5	87467586	G	A	0.020568	0.003129	4.90E-11	0.6241	462346	NA	rs1251568	NA	UKB-b-57	TRUE	reported	8KoqAI
5	87468095	C	T	-0.0237	0.003602	4.70E-11	0.230331	462346	NA	rs6891381	NA	UKB-b-57	TRUE	reported	8KoqAI
5	87469449	C	A	-0.02357	0.003649	1.10E-10	0.222637	462346	NA	rs1004589	NA	UKB-b-57	TRUE	reported	8KoqAI
5	87471365	T	C	0.02052	0.003129	5.40E-11	0.624088	462346	NA	rs4916891	NA	UKB-b-57	TRUE	reported	8KoqAI
5	87472298	G	A	-0.02363	0.003602	5.30E-11	0.230329	462346	NA	rs2165380	NA	UKB-b-57	TRUE	reported	8KoqAI
5	87472669	G	A	-0.02366	0.003602	5.10E-11	0.230414	462346	NA	rs2114970	NA	UKB-b-57	TRUE	reported	8KoqAI
5	87472679	C	T	0.020397	0.003128	7.00E-11	0.623743	462346	NA	rs1174053	NA	UKB-b-57	TRUE	reported	8KoqAI
5	87472977	G	A	-0.02364	0.003602	5.30E-11	0.230338	462346	NA	rs2410761	NA	UKB-b-57	TRUE	reported	8KoqAI
5	87473289	G	A	0.020393	0.003128	7.10E-11	0.623677	462346	NA	rs1174134	NA	UKB-b-57	TRUE	reported	8KoqAI
5	87477748	T	C	-0.02358	0.003596	5.50E-11	0.230261	462346	NA	rs1007476	NA	UKB-b-57	TRUE	reported	8KoqAI
5	87479450	C	T	0.020416	0.003125	6.40E-11	0.624036	462346	NA	rs6884089	NA	UKB-b-57	TRUE	reported	8KoqAI
5	87481574	T	C	-0.02364	0.003596	4.90E-11	0.230283	462346	NA	rs1336020	NA	UKB-b-57	TRUE	reported	8KoqAI
5	87484007	C	T	0.020394	0.003124	6.70E-11	0.624447	462346	NA	rs7712097	NA	UKB-b-57	TRUE	reported	8KoqAI
5	87484965	T	C	-0.02364	0.003596	4.90E-11	0.230287	462346	NA	rs1007996	NA	UKB-b-57	TRUE	reported	8KoqAI
5	87486538	T	C	0.020556	0.003124	4.70E-11	0.624187	462346	NA	rs168569	NA	UKB-b-57	TRUE	reported	8KoqAI
5	87487942	T	C	0.020612	0.003124	4.20E-11	0.624156	462346	NA	rs357509	NA	UKB-b-57	TRUE	reported	8KoqAI
5	87490664	G	A	-0.02371	0.003595	4.30E-11	0.230327	462346	NA	rs33702	NA	UKB-b-57	TRUE	reported	8KoqAI
5	87490776	T	C	-0.02371	0.003595	4.20E-11	0.23034	462346	NA	rs33701	NA	UKB-b-57	TRUE	reported	8KoqAI
5	87490908	T	C	-0.02369	0.003595	4.40E-11	0.230339	462346	NA	rs33700	NA	UKB-b-57	TRUE	reported	8KoqAI
5	87491545	T	C	0.020612	0.003124	4.20E-11	0.624162	462346	NA	rs33699	NA	UKB-b-57	TRUE	reported	8KoqAI
5	87493757	C	G	-0.02369	0.003595	4.40E-11	0.230306	462346	NA	rs358295	NA	UKB-b-57	TRUE	reported	8KoqAI
5	87495337	C	T	-0.02343	0.003595	7.20E-11	0.230228	462346	NA	rs358287	NA	UKB-b-57	TRUE	reported	8KoqAI
5	87495929	T	C	0.020482	0.003123	5.50E-11	0.623819	462346	NA	rs376400	NA	UKB-b-57	TRUE	reported	8KoqAI
5	87497612	T	C	-0.02215	0.004008	3.20E-08	0.173667	462346	NA	rs2850522	NA	UKB-b-57	TRUE	reported	8KoqAI
5	87498027	G	A	0.020553	0.003124	4.70E-11	0.62397	462346	NA	rs3099436	NA	UKB-b-57	TRUE	reported	8KoqAI
5	87498069	C	T	0.020552	0.003124	4.70E-11	0.62397	462346	NA	rs3098676	NA	UKB-b-57	TRUE	reported	8KoqAI
5	87498128	T	A	0.020569	0.003124	4.60E-11	0.623978	462346	NA	rs3099435	NA	UKB-b-57	TRUE	reported	8KoqAI
5	87498849	G	A	0.020681	0.003123	3.50E-11	0.623148	462346	NA	rs3097146	NA	UKB-b-57	TRUE	reported	8KoqAI
5	87500678	A	G	-0.02388	0.003592	3.00E-11	0.23101	462346	NA	rs152229	NA	UKB-b-57	TRUE	reported	8KoqAI
5	87504285	T	C	0.024309	0.003158	1.40E-14	0.641874	462346	NA	rs33705	NA	UKB-b-57	TRUE	reported	8KoqAI
5	87505164	C	G	-0.02387	0.003592	3.00E-11	0.231027	462346	NA	rs33706	NA	UKB-b-57	TRUE	reported	8KoqAI
5	87505229	G	C	-0.02388	0.003592	3.00E-11	0.231027	462346	NA	rs33707	NA	UKB-b-57	TRUE	reported	8KoqAI
5	87507137	A	G	-0.02362	0.003595	5.00E-11	0.230551	462346	NA	rs33708	NA	UKB-b-57	TRUE	reported	8KoqAI
5	87507440	C	T	-0.02363	0.003595	4.90E-11	0.230534	462346	NA	rs42298	NA	UKB-b-57	TRUE	reported	8KoqAI
5	87509260	C	T	-0.02396	0.003593	2.60E-11	0.230942	462346	NA	rs33710	NA	UKB-b-57	TRUE	reported	8KoqAI
5	87511832	T	G	0.023931	0.003223	1.10E-13	0.665308	462346	NA	rs33711	NA	UKB-b-57	TRUE	reported	8KoqAI
5	87512030	A	C	-0.02412	0.003601	2.10E-11	0.231832	462346	NA	rs33712	NA	UKB-b-57	TRUE	reported	8KoqAI
5	87512314	C	T	0.024634	0.00317	7.70E-15	0.641615	462346	NA	rs33713	NA	UKB-b-57	TRUE	reported	8KoqAI
5	87512532	G	A	-0.02412	0.003612	2.40E-11	0.231423	462346	NA	rs33714	NA	UKB-b-57	TRUE	reported	8KoqAI
5	87520595	C	G	0.021259	0.003101	7.10E-12	0.525517	462346	NA	rs185065	NA	UKB-b-57	TRUE	reported	8KoqAI
5	87530253	A	C	-0.02504	0.00374	2.10E-11	0.210125	462346	NA	rs247916	NA	UKB-b-57	TRUE	reported	8KoqAI
5	87532258	G	A	-0.02507	0.00374	2.10E-11	0.21002	462346	NA	rs193707	NA	UKB-b-57	TRUE	reported	8KoqAI
5	87545318	C	T	0.021277	0.003053	3.20E-12	0.536007	462346	NA	rs3099439	NA	UKB-b-57	TRUE	reported	8KoqAI
5	87557207	A	G	0.021491	0.003053	1.90E-12	0.53648	462346	NA	rs3099438	NA	UKB-b-57	TRUE	reported	8KoqAI
5	87560034	C	T	-0.02513	0.003742	1.80E-11	0.209748	462346	NA	rs247915	NA	UKB-b-57	TRUE	reported	8KoqAI
5	87561765	T	C	-0.02506	0.00374	2.10E-11	0.209926	462346	NA	rs33704	NA	UKB-b-57	TRUE	reported	8KoqAI
5	87566731	C	T	-0.02513	0.003742	1.90E-11	0.209743	462346	NA	rs33703	NA	UKB-b-57	TRUE	reported	8KoqAI
5	87581913	T	C	-0.02165	0.003052	1.30E-12	0.462123	462346	NA	rs2565726	NA	UKB-b-57	TRUE	reported	8KoqAI
5	87592024	C	T	-0.02185	0.003052	8.10E-13	0.461761	462346	NA	rs247909	NA	UKB-b-57	TRUE	reported	8KoqAI
5	87596675	T	G	-0.02171	0.003051	1.10E-12	0.462098	462346	NA	rs2582045	NA	UKB-b-57	TRUE	reported	8KoqAI
5	87598818	G	A	-0.02547	0.003748	1.10E-11	0.208431	462346	NA	rs2115885	NA	UKB-b-57	TRUE	reported	8KoqAI
5	87601508	G	A	-0.02171	0.003051	1.10E-12	0.462083	462346	NA	rs2565727	NA	UKB-b-57	TRUE	reported	8KoqAI
5	87602093	T	C	-0.02171	0.003051	1.10E-12	0.4621	462346	NA	rs2582044	NA	UKB-b-57	TRUE	reported	8KoqAI
5	87610059	A	C	-0.02181	0.003043	7.60E-13	0.4621	462346	NA	rs3110021	NA	UKB-b-57	TRUE	reported	8KoqAI
5	87614019	C	G	-0.02553	0.003739	8.60E-12	0.208446	462346	NA	rs2728786	NA	UKB-b-57	TRUE	reported	8KoqAI
5	87618728	G	C	-0.0219	0.003043	6.10E-13	0.461713	462346	NA	rs247913	NA	UKB-b-57	TRUE	reported	8KoqAI
5	87620249	T	G	-0.02189	0.003043	6.30E-13	0.461713	462346	NA	rs247914	NA	UKB-b-57	TRUE	reported	8KoqAI
5	87624837	A	G	-0.02546	0.003736	9.50E-12	0.208419	462346	NA	rs2728784	NA	UKB-b-57	TRUE	reported	8KoqAI
5	87625244	G	A	-0.02182	0.003041	7.30E-13	0.462058	462346	NA	rs2582042	NA	UKB-b-57	TRUE	reported	8KoqAI
5	87633758	A	G	-0.02191	0.003041	5.80E-13	0.46174	462346	NA	rs247911	NA	UKB-b-57	TRUE	reported	8KoqAI
5	87634289	A	G	-0.02543	0.003736	1.00E-11	0.208435	462346	NA	rs193706	NA	UKB-b-57	TRUE	reported	8KoqAI
5	87634692	C	G	-0.02196	0.003041	5.10E-13	0.461781	462346	NA	rs247912	NA	UKB-b-57	TRUE	reported	8KoqAI
5	87637449	C	T	-0.0218	0.003041	7.60E-13	0.461606	462346	NA	rs3099437	NA	UKB-b-57	TRUE	reported	8KoqAI
5	87644310	G	A	0.021949	0.00304	5.20E-13	0.53826	462346	NA	rs2195613	NA	UKB-b-57	TRUE	reported	8KoqAI
5	87647584	T	C	0.021796	0.00304	7.50E-13	0.537955	462346	NA	rs1094252	NA	UKB-b-57	TRUE	reported	8KoqAI
5	87662320	T	C	0.02564	0.003732	6.40E-12	0.791331	462346	NA	rs1315715	NA	UKB-b-57	TRUE	reported	8KoqAI
5	87666641	G	T	0.025619	0.003732	6.70E-12	0.791315	462346	NA	rs2410763	NA	UKB-b-57	TRUE	reported	8KoqAI
5	87677848	C	T	-0.0217	0.00304	9.40E-13	0.461948	462346	NA	rs4916720	NA	UKB-b-57	TRUE	reported	8KoqAI
5	87678585	C	T	-0.02185	0.00304	6.60E-13	0.461502	462346	NA	rs1430205	NA	UKB-b-57	TRUE	reported	8KoqAI
5	87678632	T	C	-0.0255	0.003732	8.40E-12	0.20866	462346	NA	rs1430204	NA	UKB-b-57	TRUE	reported	8KoqAI
5	87679052	T	C	-0.02176	0.00304	8.10E-13	0.461882	462346	NA	rs4916908	NA	UKB-b-57	TRUE	reported	8KoqAI
5	87679325	C	A	-0.02554	0.003733	7.80E-12	0.208619	462346	NA	rs1004334	NA	UKB-b-57	TRUE	reported	8KoqAI
5	87680994	A	G	-0.02178	0.003038	7.50E-13	0.466891	462346	NA	rs6452784	NA	UKB-b-57	TRUE	reported	8KoqAI
5	87682030	G	A	-0.0216	0.003038	1.20E-12	0.467244	462346	NA	rs6414945	NA	UKB-b-57	TRUE	reported	8KoqAI
5	87682877	C	G	-0.02497	0.003697	1.50E-11	0.214016	462346	NA	rs7442885	NA	UKB-b-57	TRUE	reported	8KoqAI
5	87685500	C	T	-0.02176	0.003038	8.00E-13	0.466923	462346	NA	rs6452785	NA	UKB-b-57	TRUE	reported	8KoqAI
5	87693637	G	C	-0.0216	0.003037	1.10E-12	0.467263	462346	NA	rs2898558	NA	UKB-b-57	TRUE	reported	8KoqAI
5	87696843	G	A	-0.0216	0.003037	1.10E-12	0.467275	462346	NA	rs4916910	NA	UKB-b-57	TRUE	reported	8KoqAI
5	87697533	C	T	-0.02503	0.003696	1.30E-11	0.214003	462346	NA	rs6870983	NA	UKB-b-57	TRUE	reported	8KoqAI
5	87703099	C	G	-0.02506	0.003697	1.20E-11	0.213956	462346	NA	rs7445169	NA	UKB-b-57	TRUE	reported	8KoqAI
5	87705268	C	G	-0.02507	0.003696	1.20E-11	0.213921	462346	NA	rs2410767	NA	UKB-b-57	TRUE	reported	8KoqAI
5	87712519	C	A	-0.0213	0.003034	2.20E-12	0.467673	462346	NA	rs7708715	NA	UKB-b-57	TRUE	reported	8KoqAI
5	87712831	A	G	-0.02149	0.003035	1.40E-12	0.466926	462346	NA	rs6452787	NA	UKB-b-57	TRUE	reported	8KoqAI
5	87712913	G	A	-0.02524	0.003545	1.10E-12	0.240354	462346	NA	rs6452788	NA	UKB-b-57	TRUE	reported	8KoqAI
5	87714704	A	G	-0.02608	0.004735	3.60E-08	0.11763	462346	NA	rs1047427	NA	UKB-b-57	TRUE	reported	8KoqAI
5	87715465	A	G	-0.02525	0.00356	1.30E-12	0.237291	462346	NA	rs6884773	NA	UKB-b-57	TRUE	reported	8KoqAI
5	87729711	A	C	-0.02129	0.003033	2.20E-12	0.467579	462346	NA	rs6414946	NA	UKB-b-57	TRUE	reported	8KoqAI
5	87730027	A	G	-0.02519	0.003559	1.50E-12	0.237258	462346	NA	rs7444298	NA	UKB-b-57	TRUE	reported	8KoqAI
5	87740423	T	G	-0.0249	0.003593	4.20E-12	0.231497	462346	NA	rs7717613	NA	UKB-b-57	TRUE	reported	8KoqAI
5	87740893	T	C	-0.02089	0.003039	6.20E-12	0.460859	462346	NA	rs7722095	NA	UKB-b-57	TRUE	reported	8KoqAI
5	87744977	G	A	-0.02491	0.003593	4.10E-12	0.231541	462346	NA	rs1047391	NA	UKB-b-57	TRUE	reported	8KoqAI
5	87745382	C	T	-0.0249	0.003594	4.20E-12	0.231365	462346	NA	rs1007487	NA	UKB-b-57	TRUE	reported	8KoqAI
5	87751752	G	A	-0.02082	0.003041	7.50E-12	0.461398	462346	NA	rs7728883	NA	UKB-b-57	TRUE	reported	8KoqAI
5	87752695	A	G	-0.021	0.003041	5.10E-12	0.460513	462346	NA	rs7448716	NA	UKB-b-57	TRUE	reported	8KoqAI
5	87754805	C	T	-0.02082	0.003041	7.60E-12	0.461345	462346	NA	rs6866315	NA	UKB-b-57	TRUE	reported	8KoqAI
5	87755411	A	G	-0.02482	0.003596	5.10E-12	0.231526	462346	NA	rs6236912	NA	UKB-b-57	TRUE	reported	8KoqAI
5	87756708	G	A	-0.02482	0.003596	5.10E-12	0.231494	462346	NA	rs6452790	NA	UKB-b-57	TRUE	reported	8KoqAI
5	87756821	C	T	-0.02103	0.003042	4.70E-12	0.460386	462346	NA	rs4352629	NA	UKB-b-57	TRUE	reported	8KoqAI
5	87756918	C	T	-0.02099	0.003042	5.20E-12	0.460485	462346	NA	rs4571506	NA	UKB-b-57	TRUE	reported	8KoqAI
5	87763468	C	A	-0.02109	0.003043	4.20E-12	0.458078	462346	NA	rs1006072	NA	UKB-b-57	TRUE	reported	8KoqAI
5	87763516	G	A	-0.02129	0.003044	2.70E-12	0.457229	462346	NA	rs1195247	NA	UKB-b-57	TRUE	reported	8KoqAI
5	87764347	T	A	-0.02485	0.003596	4.90E-12	0.231517	462346	NA	rs1021417	NA	UKB-b-57	TRUE	reported	8KoqAI
5	87764581	C	T	-0.02129	0.003044	2.70E-12	0.457235	462346	NA	rs4293953	NA	UKB-b-57	TRUE	reported	8KoqAI

5	87764618	G	A	-0.02122	0.003043	3.10E-12	0.45768	462346	NA	rs4244212	NA	UKB-b-57	TRUE	reported	8KoqAI
5	87765771	T	C	-0.02131	0.003044	2.60E-12	0.457156	462346	NA	rs9293500	NA	UKB-b-57	TRUE	reported	8KoqAI
5	87769693	C	T	-0.02112	0.003043	4.00E-12	0.457978	462346	NA	rs6891239	NA	UKB-b-57	TRUE	reported	8KoqAI
5	87771093	A	C	-0.02133	0.003044	2.50E-12	0.457162	462346	NA	rs6452791	NA	UKB-b-57	TRUE	reported	8KoqAI
5	87773655	A	G	-0.0213	0.003044	2.60E-12	0.457155	462346	NA	rs6873449	NA	UKB-b-57	TRUE	reported	8KoqAI
5	87773729	G	T	-0.02111	0.003044	4.00E-12	0.457982	462346	NA	rs6874021	NA	UKB-b-57	TRUE	reported	8KoqAI
5	87775691	T	C	-0.02154	0.003056	1.80E-12	0.440475	462346	NA	rs7706932	NA	UKB-b-57	TRUE	reported	8KoqAI
5	87781168	C	T	-0.0218	0.003067	1.20E-12	0.435757	462346	NA	rs1004461	NA	UKB-b-57	TRUE	reported	8KoqAI
5	87783713	T	C	-0.0182	0.003236	1.80E-08	0.33051	462346	NA	rs2067919	NA	UKB-b-57	TRUE	reported	8KoqAI
5	87792844	G	A	-0.0222	0.003062	4.20E-13	0.516574	462346	NA	rs6452792	NA	UKB-b-57	TRUE	reported	8KoqAI
5	87795525	G	A	-0.02328	0.00362	1.30E-10	0.230638	462346	NA	rs7737179	NA	UKB-b-57	TRUE	reported	8KoqAI
5	87802144	C	T	-0.02329	0.003631	1.40E-10	0.228474	462346	NA	rs1690319	NA	UKB-b-57	TRUE	reported	8KoqAI
5	87809696	T	G	-0.02324	0.003632	1.60E-10	0.228193	462346	NA	rs6870741	NA	UKB-b-57	TRUE	reported	8KoqAI
5	87818174	G	T	-0.02305	0.003631	2.20E-10	0.228217	462346	NA	rs6452793	NA	UKB-b-57	TRUE	reported	8KoqAI
5	87822672	T	A	0.022697	0.003052	1.00E-13	0.485103	462346	NA	rs2194027	NA	UKB-b-57	TRUE	reported	8KoqAI
5	87823299	C	T	-0.02307	0.003631	2.10E-10	0.228436	462346	NA	rs6452794	NA	UKB-b-57	TRUE	reported	8KoqAI
5	87825490	C	T	-0.01873	0.003267	9.80E-09	0.317926	462346	NA	rs2194028	NA	UKB-b-57	TRUE	reported	8KoqAI
5	87830090	T	C	-0.02313	0.00363	1.90E-10	0.228454	462346	NA	rs6021696	NA	UKB-b-57	TRUE	reported	8KoqAI
5	87831034	T	C	-0.02309	0.00363	2.00E-10	0.228498	462346	NA	rs6452795	NA	UKB-b-57	TRUE	reported	8KoqAI
5	87847273	T	A	0.023342	0.003078	3.40E-14	0.564994	462346	NA	rs1265339	NA	UKB-b-57	TRUE	reported	8KoqAI
5	87854395	A	C	0.023948	0.0031	1.10E-14	0.420617	462346	NA	rs4916723	NA	UKB-b-57	TRUE	reported	8KoqAI
5	87858930	T	G	0.022612	0.00313	5.00E-13	0.381478	462346	NA	rs1742206	NA	UKB-b-57	TRUE	reported	8KoqAI
5	87863610	C	T	0.018301	0.003063	2.30E-09	0.428814	462346	NA	rs1659071	NA	UKB-b-57	TRUE	reported	8KoqAI
5	87865788	A	G	0.018265	0.003063	2.50E-09	0.428765	462346	NA	rs1644047	NA	UKB-b-57	TRUE	reported	8KoqAI
5	87870213	G	A	0.016764	0.003038	3.40E-08	0.476324	462346	NA	rs1006358	NA	UKB-b-57	TRUE	reported	8KoqAI
5	87870248	T	C	0.01831	0.003063	2.30E-09	0.428815	462346	NA	rs1007145	NA	UKB-b-57	TRUE	reported	8KoqAI
5	87873623	C	T	0.018363	0.003063	2.00E-09	0.428798	462346	NA	rs918429	NA	UKB-b-57	TRUE	reported	8KoqAI
5	87875379	C	T	0.018317	0.003063	2.20E-09	0.428809	462346	NA	rs7722396	NA	UKB-b-57	TRUE	reported	8KoqAI
5	87880572	A	G	0.018308	0.003063	2.30E-09	0.428846	462346	NA	rs7721028	NA	UKB-b-57	TRUE	reported	8KoqAI
5	87882179	A	G	0.018279	0.003063	2.40E-09	0.428835	462346	NA	rs1080255	NA	UKB-b-57	TRUE	reported	8KoqAI
5	87883503	T	C	0.022395	0.003124	7.60E-13	0.381748	462346	NA	rs6236915	NA	UKB-b-57	TRUE	reported	8KoqAI
5	87883838	G	C	0.022391	0.003124	7.70E-13	0.381748	462346	NA	rs5602265	NA	UKB-b-57	TRUE	reported	8KoqAI
5	87896602	C	T	0.022218	0.003125	1.20E-12	0.381012	462346	NA	rs324886	NA	UKB-b-57	TRUE	reported	8KoqAI
5	87897271	A	C	0.016713	0.003038	3.80E-08	0.475534	462346	NA	rs324885	NA	UKB-b-57	TRUE	reported	8KoqAI
5	87901163	G	A	0.020271	0.003151	1.20E-10	0.362987	462346	NA	rs6859545	NA	UKB-b-57	TRUE	reported	8KoqAI
5	87913831	G	A	0.020275	0.003151	1.20E-10	0.362867	462346	NA	rs324895	NA	UKB-b-57	TRUE	reported	8KoqAI
5	87914264	A	G	0.020248	0.003151	1.30E-10	0.362866	462346	NA	rs324896	NA	UKB-b-57	TRUE	reported	8KoqAI
5	87915481	G	T	0.02026	0.003151	1.30E-10	0.362811	462346	NA	rs1046233	NA	UKB-b-57	TRUE	reported	8KoqAI
5	87920306	C	A	0.020253	0.003151	1.30E-10	0.362808	462346	NA	rs1644042	NA	UKB-b-57	TRUE	reported	8KoqAI
5	87923882	T	G	0.020219	0.003152	1.40E-10	0.362796	462346	NA	rs1644044	NA	UKB-b-57	TRUE	reported	8KoqAI
5	87924821	C	T	0.020206	0.003152	1.50E-10	0.362669	462346	NA	rs1644045	NA	UKB-b-57	TRUE	reported	8KoqAI
5	87926463	G	A	0.020187	0.003152	1.50E-10	0.362742	462346	NA	rs1174035	NA	UKB-b-57	TRUE	reported	8KoqAI
5	87928966	G	T	0.020166	0.003152	1.60E-10	0.362741	462346	NA	rs1628250	NA	UKB-b-57	TRUE	reported	8KoqAI
5	87932809	G	T	0.02017	0.003155	1.60E-10	0.362688	462346	NA	rs4916661	NA	UKB-b-57	TRUE	reported	8KoqAI
5	87968864	A	G	-0.02143	0.003449	5.20E-10	0.268013	462346	NA	rs6882046	NA	UKB-b-57	TRUE	reported	8KoqAI
5	87974682	G	T	0.037212	0.006344	4.50E-09	0.06493	462346	NA	rs7277186	NA	UKB-b-57	TRUE	reported	8KoqAI
5	87985295	G	C	0.018344	0.003109	3.60E-09	0.586191	462346	NA	rs26579	NA	UKB-b-57	TRUE	reported	8KoqAI
5	87992576	A	G	0.018382	0.003105	3.20E-09	0.593226	462346	NA	rs27732	NA	UKB-b-57	TRUE	reported	8KoqAI
5	87993198	A	G	0.017738	0.003097	1.00E-08	0.587905	462346	NA	rs410671	NA	UKB-b-57	TRUE	reported	8KoqAI
5	87993365	C	T	0.01768	0.003097	1.10E-08	0.58781	462346	NA	rs34338	NA	UKB-b-57	TRUE	reported	8KoqAI
5	88000636	C	T	0.017342	0.003078	1.80E-08	0.581401	462346	NA	rs254781	NA	UKB-b-57	TRUE	reported	8KoqAI
5	88001186	A	G	0.017346	0.003078	1.70E-08	0.581427	462346	NA	rs194225	NA	UKB-b-57	TRUE	reported	8KoqAI
5	88001969	A	G	0.017323	0.003077	1.80E-08	0.581399	462346	NA	rs639725	NA	UKB-b-57	TRUE	reported	8KoqAI
5	88002084	T	A	0.017322	0.003077	1.80E-08	0.581398	462346	NA	rs640177	NA	UKB-b-57	TRUE	reported	8KoqAI
5	88002653	T	C	0.017352	0.003077	1.70E-08	0.581353	462346	NA	rs447801	NA	UKB-b-57	TRUE	reported	8KoqAI
5	88003403	C	T	0.017377	0.003076	1.60E-08	0.581348	462346	NA	rs454214	NA	UKB-b-57	TRUE	reported	8KoqAI
5	88004616	C	T	0.017443	0.003076	1.40E-08	0.581615	462346	NA	rs1081158	NA	UKB-b-57	TRUE	reported	8KoqAI
5	88005827	T	C	0.017447	0.003076	1.40E-08	0.581608	462346	NA	rs446219	NA	UKB-b-57	TRUE	reported	8KoqAI
5	88006764	T	C	0.017468	0.003076	1.40E-08	0.581595	462346	NA	rs185291	NA	UKB-b-57	TRUE	reported	8KoqAI
5	88011139	G	C	0.017589	0.003074	1.10E-08	0.581665	462346	NA	rs40504	NA	UKB-b-57	TRUE	reported	8KoqAI
5	88015545	A	C	0.017871	0.003079	6.50E-09	0.580522	462346	NA	rs34316	NA	UKB-b-57	TRUE	reported	8KoqAI
5	88019474	G	A	0.018183	0.003078	3.50E-09	0.586674	462346	NA	rs42850	NA	UKB-b-57	TRUE	reported	8KoqAI
5	88019629	T	C	0.018112	0.003078	4.00E-09	0.586676	462346	NA	rs254779	NA	UKB-b-57	TRUE	reported	8KoqAI
5	88019747	G	C	0.018153	0.003077	3.70E-09	0.586629	462346	NA	rs254780	NA	UKB-b-57	TRUE	reported	8KoqAI
5	88020319	C	G	0.018165	0.003077	3.60E-09	0.586637	462346	NA	rs34321	NA	UKB-b-57	TRUE	reported	8KoqAI
5	88021527	T	G	0.018114	0.003077	3.90E-09	0.586716	462346	NA	rs34320	NA	UKB-b-57	TRUE	reported	8KoqAI
5	88023349	A	T	0.018147	0.003077	3.70E-09	0.586694	462346	NA	rs34319	NA	UKB-b-57	TRUE	reported	8KoqAI
5	88026870	C	T	0.017721	0.003075	8.30E-09	0.585509	462346	NA	rs187270	NA	UKB-b-57	TRUE	reported	8KoqAI
5	88026902	C	T	0.020028	0.003336	1.90E-09	0.710901	462346	NA	rs190438	NA	UKB-b-57	TRUE	reported	8KoqAI
5	88027149	G	A	0.01774	0.003075	8.00E-09	0.585476	462346	NA	rs34318	NA	UKB-b-57	TRUE	reported	8KoqAI
5	88030670	C	T	0.017812	0.003075	6.90E-09	0.585848	462346	NA	rs1315824	NA	UKB-b-57	TRUE	reported	8KoqAI
5	88031820	T	C	0.017894	0.003075	5.90E-09	0.58575	462346	NA	rs601942	NA	UKB-b-57	TRUE	reported	8KoqAI
5	88032522	C	T	0.017843	0.003075	6.50E-09	0.585783	462346	NA	rs587490	NA	UKB-b-57	TRUE	reported	8KoqAI
5	88032651	G	T	0.017815	0.003075	6.90E-09	0.585794	462346	NA	rs588282	NA	UKB-b-57	TRUE	reported	8KoqAI
5	88035887	C	T	0.017814	0.003074	6.90E-09	0.585779	462346	NA	rs676096	NA	UKB-b-57	TRUE	reported	8KoqAI
5	88036486	C	T	0.017824	0.003074	6.70E-09	0.585802	462346	NA	rs663046	NA	UKB-b-57	TRUE	reported	8KoqAI
5	88038390	A	G	0.017837	0.003074	6.60E-09	0.585804	462346	NA	rs618298	NA	UKB-b-57	TRUE	reported	8KoqAI
5	88038845	C	T	0.017854	0.003074	6.30E-09	0.585798	462346	NA	rs616391	NA	UKB-b-57	TRUE	reported	8KoqAI
5	88039660	T	A	0.017851	0.003074	6.40E-09	0.585798	462346	NA	rs2457979	NA	UKB-b-57	TRUE	reported	8KoqAI
5	88040316	C	G	0.017846	0.003074	6.40E-09	0.585804	462346	NA	rs589088	NA	UKB-b-57	TRUE	reported	8KoqAI
5	88041781	G	A	0.01783	0.003074	6.60E-09	0.585789	462346	NA	rs628158	NA	UKB-b-57	TRUE	reported	8KoqAI
5	88042296	T	C	0.017817	0.003074	6.80E-09	0.5858	462346	NA	rs625970	NA	UKB-b-57	TRUE	reported	8KoqAI
5	88043656	G	C	0.017832	0.003074	6.60E-09	0.585803	462346	NA	rs599402	NA	UKB-b-57	TRUE	reported	8KoqAI
5	88045575	C	T	0.017802	0.003074	7.00E-09	0.585518	462346	NA	rs677679	NA	UKB-b-57	TRUE	reported	8KoqAI
5	88046158	C	A	0.017791	0.003074	7.10E-09	0.58551	462346	NA	rs3514876	NA	UKB-b-57	TRUE	reported	8KoqAI
5	88046590	C	T	0.017795	0.003074	7.10E-09	0.585507	462346	NA	rs2431391	NA	UKB-b-57	TRUE	reported	8KoqAI
5	88047999	T	C	0.01779	0.003074	7.10E-09	0.585505	462346	NA	rs2247885	NA	UKB-b-57	TRUE	reported	8KoqAI
5	88048071	T	C	0.017794	0.003074	7.10E-09	0.585508	462346	NA	rs647983	NA	UKB-b-57	TRUE	reported	8KoqAI
5	88048457	C	T	0.017785	0.003074	7.20E-09	0.585512	462346	NA	rs1705565	NA	UKB-b-57	TRUE	reported	8KoqAI
5	88048767	A	G	0.017797	0.003073	6.90E-09	0.585514	462346	NA	rs651666	NA	UKB-b-57	TRUE	reported	8KoqAI
5	88052687	A	C	0.017798	0.003073	6.90E-09	0.585514	462346	NA	rs601499	NA	UKB-b-57	TRUE	reported	8KoqAI
5	88054292	A	G	0.017813	0.003073	6.70E-09	0.585551	462346	NA	rs681446	NA	UKB-b-57	TRUE	reported	8KoqAI
5	88054763	C	A	0.017805	0.003073	6.80E-09	0.585551	462346	NA	rs679232	NA	UKB-b-57	TRUE	reported	8KoqAI
5	88055135	A	G	0.017827	0.003073	6.50E-09	0.585553	462346	NA	rs667070	NA	UKB-b-57	TRUE	reported	8KoqAI
5	88055223	C	T	0.017827	0.003073	6.60E-09	0.585553	462346	NA	rs650228	NA	UKB-b-57	TRUE	reported	8KoqAI
5	88055313	G	A	0.017805	0.003073	6.80E-09	0.585549	462346	NA	rs650702	NA	UKB-b-57	TRUE	reported	8KoqAI
5	88055517	T	C	0.017827	0.003072	6.50E-09	0.585553	462346	NA	rs665324	NA	UKB-b-57	TRUE	reported	8KoqAI
5	88055761	A	G	0.020072	0.003333	1.70E-09	0.711029	462346	NA	rs664366	NA	UKB-b-57	TRUE	reported	8KoqAI
5	88057915	T	C	0.017491	0.003062	1.10E-08	0.575013	462346	NA	rs617944	NA	UKB-b-57	TRUE	reported	8KoqAI
5	88058163	T	C	0.017492	0.003062	1.10E-08	0.575005	462346	NA	rs606393	NA	UKB-b-57	TRUE	reported	8KoqAI
5	88064012	C	T	0.017406	0.003061	1.30E-08	0.57522	462346	NA	rs618741	NA	UKB-b-57	TRUE	reported	8KoqAI
5	88064462	T	C	0.017421	0.003062	1.30E-08	0.575275	462346	NA	rs589544	NA	UKB-b-57	TRUE	reported	8KoqAI
5	88064809	G	A	0.01746	0.003063	1.20E-08	0.575352	462346	NA	rs591228	NA	UKB-b-57	TRUE	reported	8KoqAI
5	88065263	G	A	0.017393	0.003067	1.40E-08	0.575273	462346	NA	rs610217	NA	UKB-b-57	TRUE	reported	8KoqAI
5	88065628	A	G	0.017289	0.003076	1.90E-08	0.574474	462346	NA	rs1174991	NA	UKB-b-57	TRUE	reported	8KoqAI
5	88065637	G	T	0.017304	0.003075	1.80E-08	0.574393	462346	NA	rs1174344	NA	UKB-b-57	TRUE	reported	8KoqAI
5	1.44E+08	A	G	-0.0171	0.003033	1.70E-08	0.522309	462346	NA	rs1315312	NA	UKB-b-57	TRUE	reported	8KoqAI

5	1.44E+08	C	T	-0.01705	0.003033	1.90E-08	0.522341	462346	NA	rs3853463	NA	UKB-b-57	TRUE	reported	8KoqAI
5	1.44E+08	T	C	-0.01709	0.003033	1.70E-08	0.522375	462346	NA	rs1265689	NA	UKB-b-57	TRUE	reported	8KoqAI
5	1.44E+08	G	A	-0.01709	0.003033	1.70E-08	0.522376	462346	NA	rs7730888	NA	UKB-b-57	TRUE	reported	8KoqAI
5	1.44E+08	G	A	-0.01709	0.003033	1.70E-08	0.522312	462346	NA	rs1265837	NA	UKB-b-57	TRUE	reported	8KoqAI
5	1.44E+08	C	G	-0.0171	0.003033	1.70E-08	0.522358	462346	NA	rs1195313	NA	UKB-b-57	TRUE	reported	8KoqAI
5	1.44E+08	T	C	-0.01711	0.003033	1.70E-08	0.522339	462346	NA	rs1195721	NA	UKB-b-57	TRUE	reported	8KoqAI
5	1.44E+08	T	G	-0.01888	0.00304	5.20E-10	0.462161	462346	NA	rs7728602	NA	UKB-b-57	TRUE	reported	8KoqAI
5	1.44E+08	T	C	-0.01707	0.003033	1.80E-08	0.52239	462346	NA	rs1004441	NA	UKB-b-57	TRUE	reported	8KoqAI
5	1.44E+08	T	A	-0.01715	0.003033	1.60E-08	0.522374	462346	NA	rs1004449	NA	UKB-b-57	TRUE	reported	8KoqAI
5	1.44E+08	T	C	-0.01714	0.003033	1.60E-08	0.522377	462346	NA	rs1271792	NA	UKB-b-57	TRUE	reported	8KoqAI
5	1.44E+08	A	T	-0.01711	0.003033	1.70E-08	0.522357	462346	NA	rs7736261	NA	UKB-b-57	TRUE	reported	8KoqAI
5	1.44E+08	G	A	-0.01707	0.003033	1.80E-08	0.522323	462346	NA	rs9324970	NA	UKB-b-57	TRUE	reported	8KoqAI
5	1.44E+08	C	G	-0.01707	0.003033	1.80E-08	0.522323	462346	NA	rs9324971	NA	UKB-b-57	TRUE	reported	8KoqAI
5	1.44E+08	T	A	-0.0172	0.003033	1.40E-08	0.522462	462346	NA	rs7721465	NA	UKB-b-57	TRUE	reported	8KoqAI
5	1.44E+08	C	A	-0.01707	0.003033	1.80E-08	0.522323	462346	NA	rs7735888	NA	UKB-b-57	TRUE	reported	8KoqAI
5	1.44E+08	C	T	-0.01721	0.003033	1.40E-08	0.522475	462346	NA	rs3853465	NA	UKB-b-57	TRUE	reported	8KoqAI
5	1.44E+08	C	T	-0.01721	0.003033	1.40E-08	0.522475	462346	NA	rs6863157	NA	UKB-b-57	TRUE	reported	8KoqAI
5	1.44E+08	T	C	-0.01721	0.003033	1.40E-08	0.522475	462346	NA	rs6888070	NA	UKB-b-57	TRUE	reported	8KoqAI
5	1.44E+08	T	C	-0.0172	0.003033	1.40E-08	0.522499	462346	NA	rs4912992	NA	UKB-b-57	TRUE	reported	8KoqAI
5	1.44E+08	G	T	-0.0172	0.003033	1.40E-08	0.522499	462346	NA	rs4244035	NA	UKB-b-57	TRUE	reported	8KoqAI
5	1.44E+08	T	C	-0.01721	0.003033	1.40E-08	0.522504	462346	NA	rs4912996	NA	UKB-b-57	TRUE	reported	8KoqAI
5	1.44E+08	C	T	-0.01722	0.003033	1.40E-08	0.522469	462346	NA	rs1004222	NA	UKB-b-57	TRUE	reported	8KoqAI
5	1.44E+08	G	A	-0.01722	0.003033	1.40E-08	0.522466	462346	NA	rs1003652	NA	UKB-b-57	TRUE	reported	8KoqAI
5	1.44E+08	G	T	-0.01708	0.003033	1.80E-08	0.522329	462346	NA	rs6878682	NA	UKB-b-57	TRUE	reported	8KoqAI
5	1.44E+08	G	A	-0.01721	0.003033	1.40E-08	0.522435	462346	NA	rs1047725	NA	UKB-b-57	TRUE	reported	8KoqAI
5	1.44E+08	T	C	-0.01722	0.003033	1.40E-08	0.522434	462346	NA	rs1047725	NA	UKB-b-57	TRUE	reported	8KoqAI
5	1.44E+08	G	A	-0.01721	0.003033	1.40E-08	0.522469	462346	NA	rs3853466	NA	UKB-b-57	TRUE	reported	8KoqAI
5	1.44E+08	C	G	-0.01721	0.003033	1.40E-08	0.522468	462346	NA	rs1010349	NA	UKB-b-57	TRUE	reported	8KoqAI
5	1.44E+08	A	C	-0.01919	0.00304	2.70E-10	0.462259	462346	NA	rs461599	NA	UKB-b-57	TRUE	reported	8KoqAI
5	1.44E+08	G	A	-0.01724	0.003033	1.30E-08	0.52246	462346	NA	rs392659	NA	UKB-b-57	TRUE	reported	8KoqAI
5	1.44E+08	A	T	-0.0172	0.003033	1.40E-08	0.522455	462346	NA	rs455193	NA	UKB-b-57	TRUE	reported	8KoqAI
5	1.44E+08	T	A	-0.01716	0.003033	1.50E-08	0.522468	462346	NA	rs457077	NA	UKB-b-57	TRUE	reported	8KoqAI
5	1.44E+08	G	A	-0.01689	0.00304	2.80E-08	0.541109	462346	NA	rs466795	NA	UKB-b-57	TRUE	reported	8KoqAI
5	1.44E+08	A	G	-0.01691	0.00304	2.60E-08	0.541121	462346	NA	rs459807	NA	UKB-b-57	TRUE	reported	8KoqAI
5	1.44E+08	A	G	-0.01702	0.00304	2.10E-08	0.541015	462346	NA	rs458366	NA	UKB-b-57	TRUE	reported	8KoqAI
5	1.44E+08	G	T	-0.01682	0.003031	2.90E-08	0.51575	462346	NA	rs374554	NA	UKB-b-57	TRUE	reported	8KoqAI
5	1.44E+08	C	T	-0.01678	0.003039	3.40E-08	0.539273	462346	NA	rs3853467	NA	UKB-b-57	TRUE	reported	8KoqAI
5	1.44E+08	A	G	-0.01673	0.003039	3.70E-08	0.539209	462346	NA	rs3853468	NA	UKB-b-57	TRUE	reported	8KoqAI
5	1.44E+08	T	C	-0.01674	0.003039	3.60E-08	0.539221	462346	NA	rs3853469	NA	UKB-b-57	TRUE	reported	8KoqAI
5	1.44E+08	A	C	-0.01676	0.003039	3.50E-08	0.539272	462346	NA	rs6893570	NA	UKB-b-57	TRUE	reported	8KoqAI
5	1.44E+08	T	C	-0.01806	0.003029	2.50E-09	0.505548	462346	NA	rs4912997	NA	UKB-b-57	TRUE	reported	8KoqAI
5	1.44E+08	A	T	-0.01674	0.003039	3.60E-08	0.539275	462346	NA	rs4349753	NA	UKB-b-57	TRUE	reported	8KoqAI
5	1.44E+08	A	G	-0.01671	0.003039	3.80E-08	0.539282	462346	NA	rs4912998	NA	UKB-b-57	TRUE	reported	8KoqAI
5	1.44E+08	G	A	-0.01808	0.003029	2.40E-09	0.50569	462346	NA	rs4912682	NA	UKB-b-57	TRUE	reported	8KoqAI
5	1.44E+08	C	A	-0.01674	0.003039	3.60E-08	0.539267	462346	NA	rs171586	NA	UKB-b-57	TRUE	reported	8KoqAI
5	1.44E+08	G	A	-0.01678	0.003039	3.30E-08	0.539264	462346	NA	rs3866871	NA	UKB-b-57	TRUE	reported	8KoqAI
5	1.44E+08	T	A	-0.01677	0.003039	3.40E-08	0.539269	462346	NA	rs248587	NA	UKB-b-57	TRUE	reported	8KoqAI
5	1.44E+08	T	C	-0.01675	0.003039	3.60E-08	0.539292	462346	NA	rs122533	NA	UKB-b-57	TRUE	reported	8KoqAI
5	1.44E+08	G	T	-0.01674	0.003039	3.60E-08	0.539296	462346	NA	rs122531	NA	UKB-b-57	TRUE	reported	8KoqAI
5	1.44E+08	C	T	-0.01683	0.003039	3.00E-08	0.53912	462346	NA	rs122530	NA	UKB-b-57	TRUE	reported	8KoqAI
5	1.44E+08	G	A	-0.01673	0.003039	3.70E-08	0.539294	462346	NA	rs122529	NA	UKB-b-57	TRUE	reported	8KoqAI
5	1.44E+08	C	T	-0.01674	0.003039	3.60E-08	0.539268	462346	NA	rs247570	NA	UKB-b-57	TRUE	reported	8KoqAI
5	1.44E+08	G	A	-0.01809	0.003029	2.40E-09	0.505677	462346	NA	rs247571	NA	UKB-b-57	TRUE	reported	8KoqAI
5	1.44E+08	T	C	-0.01676	0.003039	3.50E-08	0.53929	462346	NA	rs247572	NA	UKB-b-57	TRUE	reported	8KoqAI
5	1.44E+08	A	G	-0.01672	0.003038	3.70E-08	0.539453	462346	NA	rs247573	NA	UKB-b-57	TRUE	reported	8KoqAI
5	1.44E+08	A	G	-0.01671	0.003038	3.80E-08	0.539425	462346	NA	rs247574	NA	UKB-b-57	TRUE	reported	8KoqAI
5	1.44E+08	A	G	-0.01669	0.003038	3.90E-08	0.539455	462346	NA	rs171585	NA	UKB-b-57	TRUE	reported	8KoqAI
5	1.44E+08	G	A	-0.01672	0.003038	3.80E-08	0.539549	462346	NA	rs247565	NA	UKB-b-57	TRUE	reported	8KoqAI
5	1.44E+08	T	C	-0.01794	0.003029	3.20E-09	0.505814	462346	NA	rs247566	NA	UKB-b-57	TRUE	reported	8KoqAI
5	1.44E+08	G	C	-0.01665	0.003038	4.30E-08	0.539399	462346	NA	rs456670	NA	UKB-b-57	TRUE	reported	8KoqAI
5	1.44E+08	G	C	-0.01665	0.003038	4.20E-08	0.539424	462346	NA	rs118783	NA	UKB-b-57	TRUE	reported	8KoqAI
5	1.44E+08	T	A	-0.018	0.003029	2.80E-09	0.50567	462346	NA	rs118784	NA	UKB-b-57	TRUE	reported	8KoqAI
5	1.44E+08	T	G	-0.01697	0.003038	2.30E-08	0.538932	462346	NA	rs4913000	NA	UKB-b-57	TRUE	reported	8KoqAI
5	1.44E+08	A	T	-0.01699	0.003038	2.20E-08	0.538961	462346	NA	rs1316074	NA	UKB-b-57	TRUE	reported	8KoqAI
5	1.44E+08	C	G	-0.01689	0.003038	2.70E-08	0.539178	462346	NA	rs3902641	NA	UKB-b-57	TRUE	reported	8KoqAI
5	1.44E+08	G	A	-0.01695	0.003038	2.40E-08	0.539059	462346	NA	rs3909840	NA	UKB-b-57	TRUE	reported	8KoqAI
5	1.44E+08	G	C	-0.01684	0.003037	2.90E-08	0.539151	462346	NA	rs248560	NA	UKB-b-57	TRUE	reported	8KoqAI
5	1.44E+08	T	G	-0.018	0.003028	2.80E-09	0.505503	462346	NA	rs248558	NA	UKB-b-57	TRUE	reported	8KoqAI
5	1.44E+08	G	A	-0.01686	0.003037	2.90E-08	0.539151	462346	NA	rs248557	NA	UKB-b-57	TRUE	reported	8KoqAI
5	1.44E+08	A	G	-0.01683	0.003037	3.00E-08	0.539153	462346	NA	rs248556	NA	UKB-b-57	TRUE	reported	8KoqAI
5	1.44E+08	G	A	-0.0168	0.003037	3.20E-08	0.538998	462346	NA	rs693443	NA	UKB-b-57	TRUE	reported	8KoqAI
5	1.44E+08	C	T	-0.01676	0.003037	3.40E-08	0.539108	462346	NA	rs378442	NA	UKB-b-57	TRUE	reported	8KoqAI
5	1.44E+08	T	C	-0.01674	0.003038	3.60E-08	0.53873	462346	NA	rs389997	NA	UKB-b-57	TRUE	reported	8KoqAI
5	1.44E+08	C	G	-0.0168	0.003037	3.20E-08	0.538972	462346	NA	rs173572	NA	UKB-b-57	TRUE	reported	8KoqAI
5	1.44E+08	T	A	-0.0168	0.003037	3.20E-08	0.538981	462346	NA	rs248568	NA	UKB-b-57	TRUE	reported	8KoqAI
5	1.44E+08	T	A	-0.01679	0.003037	3.20E-08	0.538982	462346	NA	rs248567	NA	UKB-b-57	TRUE	reported	8KoqAI
5	1.44E+08	T	C	-0.01678	0.003037	3.30E-08	0.538962	462346	NA	rs248566	NA	UKB-b-57	TRUE	reported	8KoqAI
5	1.44E+08	A	T	-0.01682	0.003037	3.10E-08	0.538978	462346	NA	rs248565	NA	UKB-b-57	TRUE	reported	8KoqAI
5	1.44E+08	C	G	-0.01682	0.003037	3.10E-08	0.538982	462346	NA	rs248564	NA	UKB-b-57	TRUE	reported	8KoqAI
5	1.44E+08	C	T	-0.01681	0.003037	3.10E-08	0.538988	462346	NA	rs248563	NA	UKB-b-57	TRUE	reported	8KoqAI
5	1.44E+08	T	A	-0.01676	0.003036	3.40E-08	0.539041	462346	NA	rs1981611	NA	UKB-b-57	TRUE	reported	8KoqAI
5	1.44E+08	C	T	-0.01678	0.003037	3.20E-08	0.538911	462346	NA	rs482011	NA	UKB-b-57	TRUE	reported	8KoqAI
5	1.44E+08	T	G	-0.01678	0.003037	3.30E-08	0.538922	462346	NA	rs446327	NA	UKB-b-57	TRUE	reported	8KoqAI
5	1.44E+08	G	A	-0.01671	0.003035	3.70E-08	0.538838	462346	NA	rs423327	NA	UKB-b-57	TRUE	reported	8KoqAI
5	1.44E+08	T	A	-0.01675	0.003037	3.50E-08	0.539002	462346	NA	rs990701	NA	UKB-b-57	TRUE	reported	8KoqAI
5	1.44E+08	A	G	-0.01688	0.003038	2.70E-08	0.538805	462346	NA	rs248578	NA	UKB-b-57	TRUE	reported	8KoqAI
5	1.44E+08	T	C	-0.0169	0.003039	2.70E-08	0.539093	462346	NA	rs248577	NA	UKB-b-57	TRUE	reported	8KoqAI
5	1.44E+08	A	G	0.017	0.00304	2.20E-08	0.461204	462346	NA	rs1004556	NA	UKB-b-57	TRUE	reported	8KoqAI
5	1.44E+08	A	C	0.016955	0.003039	2.40E-08	0.461259	462346	NA	rs1005073	NA	UKB-b-57	TRUE	reported	8KoqAI
5	1.44E+08	G	A	0.016875	0.003039	2.80E-08	0.461225	462346	NA	rs1432747	NA	UKB-b-57	TRUE	reported	8KoqAI
5	1.44E+08	A	C	0.016938	0.00304	2.50E-08	0.461454	462346	NA	rs1432746	NA	UKB-b-57	TRUE	reported	8KoqAI
5	1.44E+08	T	C	0.016919	0.003039	2.60E-08	0.461243	462346	NA	rs1006091	NA	UKB-b-57	TRUE	reported	8KoqAI
5	1.44E+08	C	T	0.016975	0.00304	2.40E-08	0.460711	462346	NA	rs2163748	NA	UKB-b-57	TRUE	reported	8KoqAI
5	1.44E+08	C	T	0.016926	0.00304	2.60E-08	0.460413	462346	NA	rs6240370	NA	UKB-b-57	TRUE	reported	8KoqAI
5	1.44E+08	A	G	0.017401	0.003032	9.60E-09	0.514916	462346	NA	rs1745416	NA	UKB-b-57	TRUE	reported	8KoqAI
5	1.44E+08	C	A	0.017363	0.003038	1.10E-08	0.503958	462346	NA	rs2190794	NA	UKB-b-57	TRUE	reported	8KoqAI
5	1.44E+08	C	T	0.017462	0.003039	9.10E-09	0.503802	462346	NA	rs1087561	NA	UKB-b-57	TRUE	reported	8KoqAI
5	1.44E+08	G	T	-0.01788	0.003041	4.10E-09	0.495142	462346	NA	rs2190789	NA	UKB-b-57	TRUE	reported	8KoqAI
5	1.46E+08	T	C	-0.01865	0.003405	4.40E-08	0.272468	462346	NA	rs6237383	NA	UKB-b-57	TRUE	reported	8KoqAI
5	1.46E+08	T	A	-0.01864	0.003402	4.30E-08	0.272895	462346	NA	rs3514254	NA	UKB-b-57	TRUE	reported	8KoqAI
5	1.46E+08	C	T	-0.01865	0.00339	3.80E-08	0.276349	462346	NA	rs1317844	NA	UKB-b-57	TRUE	reported	8KoqAI
5	1.46E+08	T	G	-0.01861	0.00339	4.00E-08	0.27636	462346	NA	rs1051556	NA	UKB-b-57	TRUE	reported	8KoqAI
5	1.46E+08	T	C	-0.01862	0.00339	4.00E-08	0.276497	462346	NA	rs6237276	NA	UKB-b-57	TRUE	reported	8KoqAI
5	1.67E+08	G	A	-0.02049	0.003726	3.80E-08	0.209454	462346	NA	rs1175077	NA	UKB-b-57	TRUE	reported	8KoqAI
6	12873775	A	G	-0.01723	0.003022	1.20E-08	0.506067	462346	NA	rs9381462	NA	UKB-b-57	TRUE	reported	8KoqAI
6	12874309	G	A	-0.01747	0.003023	7.50E-09	0.504279	462346	NA	rs1571997	NA	UKB-b-57	TRUE	reported	8KoqAI
6	12879101	A	T	-0.0176	0.003025	6.00E-09	0.504774	462346	NA	rs9472790	NA	UKB-b-57	TRUE	reported	8KoqAI

6	12903957	A	G	-0.01935	0.003082	3.50E-10	0.405493	462346	NA	rs9349379	NA	UKB-b-57	TRUE	reported	8KoqAI
6	31328518	G	A	0.026544	0.004518	4.20E-09	0.128736	462346	NA	rs5618923	NA	UKB-b-57	TRUE	reported	8KoqAI
6	31936057	G	T	0.034832	0.006038	8.00E-09	0.067269	462346	NA	rs3424110	NA	UKB-b-57	TRUE	reported	8KoqAI
6	32009487	T	C	0.033655	0.005978	1.80E-08	0.071856	462346	NA	rs6920540	NA	UKB-b-57	TRUE	reported	8KoqAI
6	32074804	T	C	0.029444	0.004935	2.40E-09	0.10497	462346	NA	rs1215385	NA	UKB-b-57	TRUE	reported	8KoqAI
6	32081196	C	A	0.029232	0.004939	3.30E-09	0.104774	462346	NA	rs2873217	NA	UKB-b-57	TRUE	reported	8KoqAI
6	32091843	G	A	0.029371	0.004939	2.70E-09	0.104702	462346	NA	rs1221510	NA	UKB-b-57	TRUE	reported	8KoqAI
6	32097290	C	T	0.02926	0.004938	3.10E-09	0.104763	462346	NA	rs2873217	NA	UKB-b-57	TRUE	reported	8KoqAI
6	32097983	G	A	0.029141	0.004939	3.60E-09	0.104719	462346	NA	rs9391734	NA	UKB-b-57	TRUE	reported	8KoqAI
6	32102680	A	C	0.028361	0.00492	8.20E-09	0.105821	462346	NA	rs1321131	NA	UKB-b-57	TRUE	reported	8KoqAI
6	32106014	C	A	0.028847	0.004932	4.90E-09	0.105161	462346	NA	rs6904786	NA	UKB-b-57	TRUE	reported	8KoqAI
6	32107161	T	C	0.029339	0.00494	2.90E-09	0.104804	462346	NA	rs1221385	NA	UKB-b-57	TRUE	reported	8KoqAI
6	98233195	C	T	-0.02056	0.003081	2.50E-11	0.432974	462346	NA	rs2388148	NA	UKB-b-57	TRUE	reported	8KoqAI
6	98297832	T	C	-0.02299	0.003165	3.80E-13	0.366039	462346	NA	rs1933720	NA	UKB-b-57	TRUE	reported	8KoqAI
6	98310291	T	G	-0.01999	0.003044	5.10E-11	0.46607	462346	NA	rs9320747	NA	UKB-b-57	TRUE	reported	8KoqAI
6	98310484	C	T	-0.01972	0.003044	9.20E-11	0.465983	462346	NA	rs9374896	NA	UKB-b-57	TRUE	reported	8KoqAI
6	98310571	G	A	-0.01974	0.003044	8.90E-11	0.465951	462346	NA	rs9374897	NA	UKB-b-57	TRUE	reported	8KoqAI
6	98312143	T	G	0.019772	0.003042	8.10E-11	0.533717	462346	NA	rs1338549	NA	UKB-b-57	TRUE	reported	8KoqAI
6	98313223	C	G	-0.0197	0.003041	9.20E-11	0.465944	462346	NA	rs1933721	NA	UKB-b-57	TRUE	reported	8KoqAI
6	98314912	A	G	-0.01956	0.003038	1.20E-10	0.466063	462346	NA	rs2039722	NA	UKB-b-57	TRUE	reported	8KoqAI
6	98316447	C	T	-0.0195	0.003038	1.40E-10	0.46546	462346	NA	rs9401250	NA	UKB-b-57	TRUE	reported	8KoqAI
6	98322282	T	C	-0.01955	0.003035	1.20E-10	0.465533	462346	NA	rs4142530	NA	UKB-b-57	TRUE	reported	8KoqAI
6	98322872	G	A	-0.0189	0.003033	4.60E-10	0.476117	462346	NA	rs4580876	NA	UKB-b-57	TRUE	reported	8KoqAI
6	98324132	C	T	-0.0195	0.003035	1.30E-10	0.465559	462346	NA	rs1220577	NA	UKB-b-57	TRUE	reported	8KoqAI
6	98324943	C	A	-0.0195	0.003034	1.30E-10	0.465552	462346	NA	rs9401278	NA	UKB-b-57	TRUE	reported	8KoqAI
6	98325000	C	A	-0.0195	0.003034	1.30E-10	0.46555	462346	NA	rs9387823	NA	UKB-b-57	TRUE	reported	8KoqAI
6	98325541	A	G	-0.01952	0.003034	1.20E-10	0.465545	462346	NA	rs9489980	NA	UKB-b-57	TRUE	reported	8KoqAI
6	98325884	G	A	-0.01952	0.003034	1.20E-10	0.465544	462346	NA	rs4839927	NA	UKB-b-57	TRUE	reported	8KoqAI
6	98326012	A	G	-0.01952	0.003034	1.20E-10	0.465545	462346	NA	rs4839928	NA	UKB-b-57	TRUE	reported	8KoqAI
6	98326080	G	C	-0.01952	0.003035	1.30E-10	0.46563	462346	NA	rs4839929	NA	UKB-b-57	TRUE	reported	8KoqAI
6	98326863	G	A	-0.0195	0.003034	1.30E-10	0.465559	462346	NA	rs2039719	NA	UKB-b-57	TRUE	reported	8KoqAI
6	98328963	C	A	-0.01949	0.003034	1.30E-10	0.465563	462346	NA	rs9387845	NA	UKB-b-57	TRUE	reported	8KoqAI
6	98329165	G	A	-0.01949	0.003034	1.30E-10	0.465567	462346	NA	rs1266259	NA	UKB-b-57	TRUE	reported	8KoqAI
6	98329264	G	C	-0.01954	0.003035	1.20E-10	0.465393	462346	NA	rs1266260	NA	UKB-b-57	TRUE	reported	8KoqAI
6	98330025	G	A	-0.01955	0.003034	1.20E-10	0.465428	462346	NA	rs9372620	NA	UKB-b-57	TRUE	reported	8KoqAI
6	98330225	G	A	-0.01953	0.003034	1.20E-10	0.465437	462346	NA	rs7756023	NA	UKB-b-57	TRUE	reported	8KoqAI
6	98330626	C	T	-0.01952	0.003034	1.30E-10	0.465466	462346	NA	rs7744474	NA	UKB-b-57	TRUE	reported	8KoqAI
6	98331085	C	G	-0.01953	0.003034	1.20E-10	0.465441	462346	NA	rs9490000	NA	UKB-b-57	TRUE	reported	8KoqAI
6	98331133	T	C	-0.01953	0.003034	1.20E-10	0.465438	462346	NA	rs9490001	NA	UKB-b-57	TRUE	reported	8KoqAI
6	98331316	A	G	-0.01953	0.003034	1.20E-10	0.465447	462346	NA	rs9490003	NA	UKB-b-57	TRUE	reported	8KoqAI
6	98331357	A	G	-0.01953	0.003034	1.20E-10	0.465444	462346	NA	rs9320759	NA	UKB-b-57	TRUE	reported	8KoqAI
6	98331937	A	C	-0.01941	0.003035	1.60E-10	0.465446	462346	NA	rs9401305	NA	UKB-b-57	TRUE	reported	8KoqAI
6	98332567	G	A	-0.01956	0.003035	1.10E-10	0.465385	462346	NA	rs1048486	NA	UKB-b-57	TRUE	reported	8KoqAI
6	98332622	C	T	-0.01956	0.003035	1.10E-10	0.465385	462346	NA	rs1048486	NA	UKB-b-57	TRUE	reported	8KoqAI
6	98332930	A	G	-0.01956	0.003035	1.20E-10	0.465379	462346	NA	rs7775492	NA	UKB-b-57	TRUE	reported	8KoqAI
6	98333409	G	A	-0.01967	0.003036	9.20E-11	0.464674	462346	NA	rs1218967	NA	UKB-b-57	TRUE	reported	8KoqAI
6	98344031	G	A	-0.02556	0.003125	2.90E-16	0.381706	462346	NA	rs9372625	NA	UKB-b-57	TRUE	reported	8KoqAI
6	98345853	T	A	-0.02	0.003052	5.70E-11	0.46899	462346	NA	rs6906737	NA	UKB-b-57	TRUE	reported	8KoqAI
6	98346633	T	C	0.019058	0.003034	3.30E-10	0.490776	462346	NA	rs1338552	NA	UKB-b-57	TRUE	reported	8KoqAI
6	98346704	T	A	0.019074	0.003034	3.30E-10	0.490898	462346	NA	rs1338553	NA	UKB-b-57	TRUE	reported	8KoqAI
6	98346801	A	G	0.019283	0.003033	2.00E-10	0.499419	462346	NA	rs1338554	NA	UKB-b-57	TRUE	reported	8KoqAI
6	98348985	A	C	0.01765	0.003031	5.70E-09	0.50698	462346	NA	rs1115396	NA	UKB-b-57	TRUE	reported	8KoqAI
6	98349364	T	C	0.017298	0.003031	1.10E-08	0.498446	462346	NA	rs7761722	NA	UKB-b-57	TRUE	reported	8KoqAI
6	98349496	A	G	0.017603	0.003031	6.30E-09	0.506976	462346	NA	rs7757867	NA	UKB-b-57	TRUE	reported	8KoqAI
6	98349530	T	G	0.017542	0.003031	7.10E-09	0.507126	462346	NA	rs7761915	NA	UKB-b-57	TRUE	reported	8KoqAI
6	98350319	A	C	0.017629	0.003031	6.00E-09	0.506965	462346	NA	rs6927038	NA	UKB-b-57	TRUE	reported	8KoqAI
6	98351457	C	T	0.017626	0.00303	6.00E-09	0.507037	462346	NA	rs9482077	NA	UKB-b-57	TRUE	reported	8KoqAI
6	98353622	T	C	0.017591	0.00303	6.40E-09	0.506854	462346	NA	rs6569161	NA	UKB-b-57	TRUE	reported	8KoqAI
6	98355187	G	T	0.017293	0.003029	1.10E-08	0.498234	462346	NA	rs6901729	NA	UKB-b-57	TRUE	reported	8KoqAI
6	98355221	A	C	0.01762	0.003029	6.00E-09	0.506838	462346	NA	rs6925043	NA	UKB-b-57	TRUE	reported	8KoqAI
6	98355661	A	G	0.017634	0.003029	5.80E-09	0.506842	462346	NA	rs1115396	NA	UKB-b-57	TRUE	reported	8KoqAI
6	98356183	G	C	0.017579	0.003029	6.50E-09	0.506841	462346	NA	rs6907873	NA	UKB-b-57	TRUE	reported	8KoqAI
6	98356211	C	T	0.017316	0.003029	1.10E-08	0.49823	462346	NA	rs6909175	NA	UKB-b-57	TRUE	reported	8KoqAI
6	98356503	T	A	0.017588	0.003028	6.30E-09	0.506825	462346	NA	rs1087221	NA	UKB-b-57	TRUE	reported	8KoqAI
6	98358083	C	T	0.017561	0.003027	6.60E-09	0.506799	462346	NA	rs6920927	NA	UKB-b-57	TRUE	reported	8KoqAI
6	98358220	T	C	0.022949	0.003135	2.50E-13	0.628577	462346	NA	rs1115396	NA	UKB-b-57	TRUE	reported	8KoqAI
6	98358575	G	A	0.017569	0.003027	6.50E-09	0.506893	462346	NA	rs6924808	NA	UKB-b-57	TRUE	reported	8KoqAI
6	98358618	G	A	0.017315	0.003028	1.10E-08	0.497982	462346	NA	rs6924833	NA	UKB-b-57	TRUE	reported	8KoqAI
6	98359053	C	T	0.017305	0.003027	1.10E-08	0.498267	462346	NA	rs6927014	NA	UKB-b-57	TRUE	reported	8KoqAI
6	98360211	T	C	0.017595	0.003027	6.10E-09	0.506869	462346	NA	rs4839930	NA	UKB-b-57	TRUE	reported	8KoqAI
6	98361406	A	G	0.017495	0.003026	7.40E-09	0.506864	462346	NA	rs2388151	NA	UKB-b-57	TRUE	reported	8KoqAI
6	98361770	T	C	0.017328	0.003027	1.00E-08	0.498257	462346	NA	rs6939508	NA	UKB-b-57	TRUE	reported	8KoqAI
6	98361795	A	C	0.017591	0.003027	6.20E-09	0.506731	462346	NA	rs6934784	NA	UKB-b-57	TRUE	reported	8KoqAI
6	98361813	A	G	0.017325	0.003027	1.00E-08	0.49823	462346	NA	rs6934796	NA	UKB-b-57	TRUE	reported	8KoqAI
6	98364895	A	G	0.022914	0.003134	2.70E-13	0.628445	462346	NA	rs9482094	NA	UKB-b-57	TRUE	reported	8KoqAI
6	98365289	G	A	-0.02285	0.003135	3.10E-13	0.370879	462346	NA	rs4839715	NA	UKB-b-57	TRUE	reported	8KoqAI
6	98366713	T	C	0.017316	0.003027	1.10E-08	0.498363	462346	NA	rs1219228	NA	UKB-b-57	TRUE	reported	8KoqAI
6	98368639	A	T	0.017513	0.003027	7.30E-09	0.506946	462346	NA	rs1115397	NA	UKB-b-57	TRUE	reported	8KoqAI
6	98369230	T	G	0.022927	0.003135	2.60E-13	0.62869	462346	NA	rs4339469	NA	UKB-b-57	TRUE	reported	8KoqAI
6	98370615	A	T	0.01751	0.003028	7.30E-09	0.506889	462346	NA	rs1115398	NA	UKB-b-57	TRUE	reported	8KoqAI
6	98373765	T	C	0.022835	0.003135	3.30E-13	0.628347	462346	NA	rs6937215	NA	UKB-b-57	TRUE	reported	8KoqAI
6	98392667	C	A	0.019997	0.003101	1.10E-10	0.602001	462346	NA	rs9482120	NA	UKB-b-57	TRUE	reported	8KoqAI
6	98399208	C	G	-0.02004	0.003101	1.00E-10	0.398445	462346	NA	rs3512139	NA	UKB-b-57	TRUE	reported	8KoqAI
6	98399521	A	G	0.019945	0.0031	1.30E-10	0.601197	462346	NA	rs4548017	NA	UKB-b-57	TRUE	reported	8KoqAI
6	98411631	A	G	0.019905	0.003098	1.30E-10	0.601721	462346	NA	rs6905544	NA	UKB-b-57	TRUE	reported	8KoqAI
6	98421721	T	C	0.019966	0.003095	1.10E-10	0.601514	462346	NA	rs6938973	NA	UKB-b-57	TRUE	reported	8KoqAI
6	98429337	T	C	0.019803	0.003096	1.60E-10	0.602848	462346	NA	rs9320823	NA	UKB-b-57	TRUE	reported	8KoqAI
6	98435125	G	T	0.019812	0.003096	1.60E-10	0.602822	462346	NA	rs1087222	NA	UKB-b-57	TRUE	reported	8KoqAI
6	98452680	T	C	-0.02053	0.0031	3.50E-11	0.393861	462346	NA	rs9401452	NA	UKB-b-57	TRUE	reported	8KoqAI
6	98458340	C	T	-0.02051	0.003099	3.60E-11	0.393971	462346	NA	rs9401458	NA	UKB-b-57	TRUE	reported	8KoqAI
6	98475160	A	G	-0.01693	0.003052	2.90E-08	0.43733	462346	NA	rs7772104	NA	UKB-b-57	TRUE	reported	8KoqAI
6	98481755	C	T	-0.01692	0.003052	3.00E-08	0.43733	462346	NA	rs7760876	NA	UKB-b-57	TRUE	reported	8KoqAI
6	98487056	G	C	-0.01688	0.003052	3.20E-08	0.437412	462346	NA	rs9375104	NA	UKB-b-57	TRUE	reported	8KoqAI
6	98488641	C	A	-0.01689	0.003052	3.10E-08	0.43741	462346	NA	rs1266333	NA	UKB-b-57	TRUE	reported	8KoqAI
6	98489561	T	C	-0.0169	0.003052	3.10E-08	0.437422	462346	NA	rs9398662	NA	UKB-b-57	TRUE	reported	8KoqAI
6	98491210	G	A	-0.01689	0.003053	3.20E-08	0.437578	462346	NA	rs9388046	NA	UKB-b-57	TRUE	reported	8KoqAI
6	98494218	C	T	-0.01703	0.003053	2.40E-08	0.437231	462346	NA	rs1319425	NA	UKB-b-57	TRUE	reported	8KoqAI
6	98500712	T	C	-0.0207	0.003099	2.40E-11	0.393349	462346	NA	rs9401501	NA	UKB-b-57	TRUE	reported	8KoqAI
6	98506764	T	C	-0.02069	0.003099	2.50E-11	0.393326	462346	NA	rs1321522	NA	UKB-b-57	TRUE	reported	8KoqAI
6	98507430	C	T	-0.02076	0.003099	2.10E-11	0.393342	462346	NA	rs4839938	NA	UKB-b-57	TRUE	reported	8KoqAI
6	98507832	A	C	-0.02074	0.003099	2.20E-11	0.393346	462346	NA	rs4839939	NA	UKB-b-57	TRUE	reported	8KoqAI
6	98509656	T	G	-0.02074	0.0031	2.20E-11	0.393302	462346	NA	rs4576255	NA	UKB-b-57	TRUE	reported	8KoqAI
6	98513032	A	G	0.020427	0.003089	3.80E-11	0.598737	462346	NA	rs3004179	NA	UKB-b-57	TRUE	reported	8KoqAI
6	98514302	G	A	-0.0207	0.003099	2.40E-11	0.39328	462346	NA	rs5573174	NA	UKB-b-57	TRUE	reported	8KoqAI
6	98519911	T	G	-0.02076	0.003099	2.10E-11	0.393363	462346	NA	rs4401671	NA	UKB-b-57	TRUE	reported	8KoqAI
6	98522436	C	G	-0.02068	0.0031	2.50E-11	0.393249	462346	NA	rs1321607	NA	UKB-b-57	TRUE	reported	8KoqAI
6	98522698	T	C	-0.02073	0.003099	2.20E-11	0.393326	462346	NA	rs3506245	NA	UKB-b-57	TRUE	reported	8KoqAI

6	98525726	C	T	-0.0207	0.003099	2.40E-11	0.393274	462346	NA	rs9388083	NA	UKB-b-57	TRUE	reported	8KoqAI
6	98526943	C	T	-0.02064	0.0031	2.80E-11	0.393264	462346	NA	rs9398676	NA	UKB-b-57	TRUE	reported	8KoqAI
6	98527087	C	T	-0.02065	0.0031	2.70E-11	0.393253	462346	NA	rs9388090	NA	UKB-b-57	TRUE	reported	8KoqAI
6	98528141	T	C	-0.02073	0.003105	2.40E-11	0.392298	462346	NA	rs7766486	NA	UKB-b-57	TRUE	reported	8KoqAI
6	98528143	C	T	-0.02104	0.003106	1.20E-11	0.392771	462346	NA	rs7742854	NA	UKB-b-57	TRUE	reported	8KoqAI
6	98528404	G	A	-0.02069	0.0031	2.50E-11	0.393262	462346	NA	rs4458695	NA	UKB-b-57	TRUE	reported	8KoqAI
6	98529166	C	T	-0.02067	0.0031	2.60E-11	0.393244	462346	NA	rs1217631	NA	UKB-b-57	TRUE	reported	8KoqAI
6	98539377	C	G	-0.02075	0.003101	2.20E-11	0.393031	462346	NA	rs901628	NA	UKB-b-57	TRUE	reported	8KoqAI
6	98539379	C	T	-0.02075	0.003101	2.20E-11	0.393007	462346	NA	rs901629	NA	UKB-b-57	TRUE	reported	8KoqAI
6	98539519	C	T	-0.02066	0.0031	2.60E-11	0.393223	462346	NA	rs901630	NA	UKB-b-57	TRUE	reported	8KoqAI
6	98545611	G	T	-0.02085	0.003101	1.80E-11	0.392926	462346	NA	rs6242266	NA	UKB-b-57	TRUE	reported	8KoqAI
6	98546547	G	T	-0.0195	0.003135	4.90E-10	0.376878	462346	NA	rs3509976	NA	UKB-b-57	TRUE	reported	8KoqAI
6	98549801	A	C	-0.0179	0.003042	4.00E-09	0.484718	462346	NA	rs9401593	NA	UKB-b-57	TRUE	reported	8KoqAI
6	98550289	C	A	-0.01793	0.003041	3.70E-09	0.484394	462346	NA	rs1906252	NA	UKB-b-57	TRUE	reported	8KoqAI
6	98553894	G	A	-0.01801	0.003039	3.10E-09	0.484285	462346	NA	rs1487441	NA	UKB-b-57	TRUE	reported	8KoqAI
6	98555272	C	T	-0.01803	0.003039	3.00E-09	0.484261	462346	NA	rs9375188	NA	UKB-b-57	TRUE	reported	8KoqAI
6	98565211	C	T	-0.01829	0.003035	1.70E-09	0.482111	462346	NA	rs1487445	NA	UKB-b-57	TRUE	reported	8KoqAI
6	98574560	C	T	-0.01855	0.003034	9.70E-10	0.482394	462346	NA	rs968050	NA	UKB-b-57	TRUE	reported	8KoqAI
6	98576223	G	A	-0.01849	0.003035	1.10E-09	0.48242	462346	NA	rs1220296	NA	UKB-b-57	TRUE	reported	8KoqAI
6	98577689	C	T	-0.01853	0.003034	1.00E-09	0.48239	462346	NA	rs9372734	NA	UKB-b-57	TRUE	reported	8KoqAI
6	98582900	G	A	-0.01856	0.003034	9.60E-10	0.482434	462346	NA	rs1220608	NA	UKB-b-57	TRUE	reported	8KoqAI
6	98591622	A	G	-0.0185	0.003036	1.10E-09	0.482452	462346	NA	rs2388334	NA	UKB-b-57	TRUE	reported	8KoqAI
6	1.41E+08	G	A	0.01764	0.003153	2.20E-08	0.37227	462346	NA	rs1452991	NA	UKB-b-57	TRUE	reported	8KoqAI
6	1.41E+08	T	C	0.018506	0.00314	3.80E-09	0.36804	462346	NA	rs7298512	NA	UKB-b-57	TRUE	reported	8KoqAI
6	1.41E+08	G	T	0.017912	0.003144	1.20E-08	0.365429	462346	NA	rs6242988	NA	UKB-b-57	TRUE	reported	8KoqAI
6	1.41E+08	T	C	0.01815	0.003142	7.60E-09	0.366839	462346	NA	rs6916409	NA	UKB-b-57	TRUE	reported	8KoqAI
6	1.41E+08	T	C	0.017869	0.003144	1.30E-08	0.365446	462346	NA	rs9321801	NA	UKB-b-57	TRUE	reported	8KoqAI
6	1.41E+08	C	T	0.01784	0.003144	1.40E-08	0.365435	462346	NA	rs5641210	NA	UKB-b-57	TRUE	reported	8KoqAI
6	1.41E+08	C	T	0.018274	0.00314	5.90E-09	0.367805	462346	NA	rs1175425	NA	UKB-b-57	TRUE	reported	8KoqAI
6	1.41E+08	T	C	0.018475	0.003138	3.90E-09	0.36787	462346	NA	rs9403253	NA	UKB-b-57	TRUE	reported	8KoqAI
6	1.41E+08	C	T	0.01798	0.003143	1.10E-08	0.365944	462346	NA	rs6916229	NA	UKB-b-57	TRUE	reported	8KoqAI
6	1.41E+08	G	A	0.01849	0.003139	3.80E-09	0.367822	462346	NA	rs1115516	NA	UKB-b-57	TRUE	reported	8KoqAI
6	1.42E+08	G	A	0.017868	0.003144	1.30E-08	0.36538	462346	NA	rs2328190	NA	UKB-b-57	TRUE	reported	8KoqAI
6	1.42E+08	C	A	0.018489	0.003139	3.80E-09	0.367822	462346	NA	rs2328191	NA	UKB-b-57	TRUE	reported	8KoqAI
6	1.42E+08	A	G	0.018385	0.00314	4.80E-09	0.367109	462346	NA	rs1196826	NA	UKB-b-57	TRUE	reported	8KoqAI
6	1.42E+08	C	A	0.018489	0.003139	3.80E-09	0.367829	462346	NA	rs6932051	NA	UKB-b-57	TRUE	reported	8KoqAI
6	1.42E+08	G	A	0.018491	0.003139	3.80E-09	0.367822	462346	NA	rs9385939	NA	UKB-b-57	TRUE	reported	8KoqAI
6	1.42E+08	T	C	0.018473	0.003139	4.00E-09	0.367828	462346	NA	rs6925531	NA	UKB-b-57	TRUE	reported	8KoqAI
6	1.42E+08	C	G	0.018476	0.003139	3.90E-09	0.367829	462346	NA	rs2328193	NA	UKB-b-57	TRUE	reported	8KoqAI
6	1.42E+08	T	C	0.018324	0.00314	5.40E-09	0.367192	462346	NA	rs2167761	NA	UKB-b-57	TRUE	reported	8KoqAI
6	1.42E+08	G	T	0.018465	0.003139	4.00E-09	0.367867	462346	NA	rs7762337	NA	UKB-b-57	TRUE	reported	8KoqAI
6	1.42E+08	C	T	0.018428	0.003139	4.40E-09	0.367873	462346	NA	rs6900746	NA	UKB-b-57	TRUE	reported	8KoqAI
6	1.42E+08	C	G	0.018426	0.00314	4.40E-09	0.36781	462346	NA	rs1196519	NA	UKB-b-57	TRUE	reported	8KoqAI
6	1.42E+08	T	C	0.01841	0.00314	4.50E-09	0.367804	462346	NA	rs9389884	NA	UKB-b-57	TRUE	reported	8KoqAI
6	1.42E+08	A	C	0.017539	0.003146	2.50E-08	0.36556	462346	NA	rs9373298	NA	UKB-b-57	TRUE	reported	8KoqAI
6	1.42E+08	C	T	0.018739	0.003144	2.50E-09	0.367608	462346	NA	rs9389886	NA	UKB-b-57	TRUE	reported	8KoqAI
6	1.42E+08	G	A	0.01874	0.003144	2.50E-09	0.367608	462346	NA	rs3911276	NA	UKB-b-57	TRUE	reported	8KoqAI
6	1.42E+08	G	T	0.01874	0.003144	2.50E-09	0.367608	462346	NA	rs9389887	NA	UKB-b-57	TRUE	reported	8KoqAI
6	1.42E+08	C	T	0.01874	0.003144	2.50E-09	0.367609	462346	NA	rs1782972	NA	UKB-b-57	TRUE	reported	8KoqAI
6	1.42E+08	G	C	0.01874	0.003144	2.50E-09	0.367609	462346	NA	rs7299109	NA	UKB-b-57	TRUE	reported	8KoqAI
6	1.42E+08	A	G	0.018894	0.00315	2.00E-09	0.366189	462346	NA	rs9389888	NA	UKB-b-57	TRUE	reported	8KoqAI
6	1.42E+08	C	T	0.018889	0.00315	2.00E-09	0.366183	462346	NA	rs9403261	NA	UKB-b-57	TRUE	reported	8KoqAI
6	1.42E+08	A	C	0.018697	0.003144	2.70E-09	0.367668	462346	NA	rs6941096	NA	UKB-b-57	TRUE	reported	8KoqAI
6	1.42E+08	A	G	0.01871	0.003149	2.80E-09	0.366713	462346	NA	rs6941145	NA	UKB-b-57	TRUE	reported	8KoqAI
6	1.42E+08	G	A	0.018731	0.003149	2.70E-09	0.366729	462346	NA	rs6941722	NA	UKB-b-57	TRUE	reported	8KoqAI
6	1.42E+08	A	T	0.01878	0.003147	2.40E-09	0.367071	462346	NA	rs6941306	NA	UKB-b-57	TRUE	reported	8KoqAI
6	1.42E+08	T	C	0.018735	0.003143	2.50E-09	0.367744	462346	NA	rs6925481	NA	UKB-b-57	TRUE	reported	8KoqAI
6	1.42E+08	G	A	0.018735	0.003143	2.50E-09	0.367744	462346	NA	rs7741796	NA	UKB-b-57	TRUE	reported	8KoqAI
6	1.42E+08	T	C	0.018717	0.003143	2.60E-09	0.367752	462346	NA	rs7762315	NA	UKB-b-57	TRUE	reported	8KoqAI
6	1.42E+08	C	T	0.01873	0.003144	2.60E-09	0.367974	462346	NA	rs7742222	NA	UKB-b-57	TRUE	reported	8KoqAI
6	1.42E+08	G	A	0.018732	0.003143	2.50E-09	0.367755	462346	NA	rs7742882	NA	UKB-b-57	TRUE	reported	8KoqAI
6	1.42E+08	G	C	0.018735	0.003143	2.50E-09	0.367748	462346	NA	rs7746946	NA	UKB-b-57	TRUE	reported	8KoqAI
6	1.42E+08	C	T	0.018734	0.003143	2.50E-09	0.367774	462346	NA	rs4146326	NA	UKB-b-57	TRUE	reported	8KoqAI
6	1.42E+08	T	C	0.018739	0.003143	2.50E-09	0.367756	462346	NA	rs1776799	NA	UKB-b-57	TRUE	reported	8KoqAI
6	1.42E+08	A	T	0.018738	0.003143	2.50E-09	0.367752	462346	NA	rs1776804	NA	UKB-b-57	TRUE	reported	8KoqAI
6	1.42E+08	C	T	0.018745	0.003143	2.50E-09	0.367754	462346	NA	rs4114765	NA	UKB-b-57	TRUE	reported	8KoqAI
6	1.42E+08	A	T	0.018767	0.003145	2.40E-09	0.367297	462346	NA	rs9389889	NA	UKB-b-57	TRUE	reported	8KoqAI
6	1.42E+08	C	T	0.018728	0.003144	2.60E-09	0.36773	462346	NA	rs6916170	NA	UKB-b-57	TRUE	reported	8KoqAI
6	1.42E+08	G	C	0.018766	0.003143	2.40E-09	0.367726	462346	NA	rs718176	NA	UKB-b-57	TRUE	reported	8KoqAI
6	1.42E+08	A	T	0.018756	0.003143	2.40E-09	0.367725	462346	NA	rs7299273	NA	UKB-b-57	TRUE	reported	8KoqAI
6	1.42E+08	G	T	0.018741	0.003143	2.50E-09	0.367748	462346	NA	rs9385942	NA	UKB-b-57	TRUE	reported	8KoqAI
6	1.42E+08	A	G	0.018738	0.003143	2.50E-09	0.367746	462346	NA	rs6243050	NA	UKB-b-57	TRUE	reported	8KoqAI
6	1.42E+08	G	A	0.018738	0.003143	2.50E-09	0.367746	462346	NA	rs7297602	NA	UKB-b-57	TRUE	reported	8KoqAI
6	1.42E+08	G	C	0.017796	0.003148	1.60E-08	0.365512	462346	NA	rs6243050	NA	UKB-b-57	TRUE	reported	8KoqAI
6	1.42E+08	A	T	0.018722	0.003144	2.60E-09	0.367632	462346	NA	rs9389890	NA	UKB-b-57	TRUE	reported	8KoqAI
6	1.42E+08	C	T	0.018738	0.003143	2.50E-09	0.367746	462346	NA	rs9403263	NA	UKB-b-57	TRUE	reported	8KoqAI
6	1.42E+08	G	C	0.018728	0.003143	2.60E-09	0.367761	462346	NA	rs9403264	NA	UKB-b-57	TRUE	reported	8KoqAI
6	1.42E+08	A	T	0.018737	0.003143	2.50E-09	0.367746	462346	NA	rs3966689	NA	UKB-b-57	TRUE	reported	8KoqAI
6	1.42E+08	G	A	0.018737	0.003143	2.50E-09	0.367746	462346	NA	rs7762366	NA	UKB-b-57	TRUE	reported	8KoqAI
6	1.42E+08	G	A	0.018661	0.003143	2.90E-09	0.367832	462346	NA	rs7762818	NA	UKB-b-57	TRUE	reported	8KoqAI
6	1.42E+08	C	G	0.018738	0.003143	2.50E-09	0.367746	462346	NA	rs7766708	NA	UKB-b-57	TRUE	reported	8KoqAI
6	1.42E+08	T	C	0.018738	0.003143	2.50E-09	0.367746	462346	NA	rs7749146	NA	UKB-b-57	TRUE	reported	8KoqAI
6	1.42E+08	A	G	0.018739	0.003143	2.50E-09	0.367746	462346	NA	rs9403268	NA	UKB-b-57	TRUE	reported	8KoqAI
6	1.42E+08	G	C	0.018739	0.003143	2.50E-09	0.367746	462346	NA	rs9389893	NA	UKB-b-57	TRUE	reported	8KoqAI
6	1.42E+08	G	T	0.01874	0.003143	2.50E-09	0.367742	462346	NA	rs9376587	NA	UKB-b-57	TRUE	reported	8KoqAI
6	1.42E+08	G	A	0.018741	0.003143	2.50E-09	0.367742	462346	NA	rs9403269	NA	UKB-b-57	TRUE	reported	8KoqAI
6	1.42E+08	C	T	0.018745	0.003143	2.50E-09	0.367747	462346	NA	rs9376588	NA	UKB-b-57	TRUE	reported	8KoqAI
6	1.42E+08	C	T	0.018743	0.003143	2.50E-09	0.367747	462346	NA	rs9376589	NA	UKB-b-57	TRUE	reported	8KoqAI
6	1.42E+08	G	T	0.01874	0.003143	2.50E-09	0.367739	462346	NA	rs6243051	NA	UKB-b-57	TRUE	reported	8KoqAI
6	1.42E+08	G	A	0.01874	0.003143	2.50E-09	0.36774	462346	NA	rs6570451	NA	UKB-b-57	TRUE	reported	8KoqAI
6	1.42E+08	A	G	0.018756	0.003143	2.40E-09	0.367723	462346	NA	rs6570452	NA	UKB-b-57	TRUE	reported	8KoqAI
6	1.42E+08	T	C	0.018756	0.003143	2.40E-09	0.367723	462346	NA	rs6570453	NA	UKB-b-57	TRUE	reported	8KoqAI
6	1.42E+08	C	T	0.018674	0.003142	2.80E-09	0.368062	462346	NA	rs6923390	NA	UKB-b-57	TRUE	reported	8KoqAI
6	1.42E+08	A	G	0.01874	0.003143	2.50E-09	0.367745	462346	NA	rs9403270	NA	UKB-b-57	TRUE	reported	8KoqAI
6	1.42E+08	T	C	0.018739	0.003143	2.50E-09	0.367745	462346	NA	rs9403271	NA	UKB-b-57	TRUE	reported	8KoqAI
6	1.42E+08	G	A	0.018715	0.003143	2.60E-09	0.367738	462346	NA	rs9403272	NA	UKB-b-57	TRUE	reported	8KoqAI
6	1.42E+08	C	T	0.018746	0.003143	2.50E-09	0.367749	462346	NA	rs7744055	NA	UKB-b-57	TRUE	reported	8KoqAI
6	1.42E+08	A	G	0.018748	0.003143	2.50E-09	0.36775	462346	NA	rs6570454	NA	UKB-b-57	TRUE	reported	8KoqAI
6	1.42E+08	C	T	0.01875	0.003148	2.60E-09	0.366909	462346	NA	rs4425611	NA	UKB-b-57	TRUE	reported	8KoqAI
6	1.42E+08	G	T	0.01875	0.003148	2.60E-09	0.366909	462346	NA	rs4568472	NA	UKB-b-57	TRUE	reported	8KoqAI
6	1.42E+08	A	G	0.018679	0.003143	2.80E-09	0.367719	462346	NA	rs4329137	NA	UKB-b-57	TRUE	reported	8KoqAI
6	1.42E+08	G	T	0.01868	0.003143	2.80E-09	0.367719	462346	NA	rs4416687	NA	UKB-b-57	TRUE	reported	8KoqAI
6	1.42E+08	C	T	0.018683	0.003143	2.80E-09	0.367718	462346	NA	rs4467796	NA	UKB-b-57	TRUE	reported	8KoqAI
6	1.42E+08	T	C	0.018676	0.003143	2.80E-09	0.367708	462346	NA	rs4565326	NA	UKB-b-57	TRUE	reported	8KoqAI
6	1.42E+08	T	C	0.018681	0.003143	2.80E-09	0.367687	462346	NA	rs9376590	NA	UKB-b-57	TRUE	reported	8KoqAI
6	1.42E+08	C	A	0.018482	0.003167	5.40E-09	0.360574	462346	NA	rs9389895	NA	UKB-b-57	TRUE	reported	8KoqAI
6	1.42E+08	T	C	0.018658	0.003144	2.90E-09	0.367647	462346	NA	rs9376591	NA	UKB-b-57	TRUE	reported	8KoqAI

6	1.42E+08	C	G	0.018669	0.003143	2.90E-09	0.367726	462346	NA	rs9385943	NA	UKB-b-57	TRUE	reported	8KoqAI
6	1.42E+08	G	A	0.018686	0.003143	2.80E-09	0.367684	462346	NA	rs9389896	NA	UKB-b-57	TRUE	reported	8KoqAI
6	1.42E+08	C	T	0.018698	0.003143	2.70E-09	0.367691	462346	NA	rs6243051	NA	UKB-b-57	TRUE	reported	8KoqAI
6	1.42E+08	G	T	0.018701	0.003143	2.70E-09	0.367693	462346	NA	rs1776820	NA	UKB-b-57	TRUE	reported	8KoqAI
6	1.42E+08	C	G	0.018693	0.003143	2.70E-09	0.367702	462346	NA	rs7298011	NA	UKB-b-57	TRUE	reported	8KoqAI
6	1.42E+08	T	C	0.018497	0.003143	4.00E-09	0.367481	462346	NA	rs6243051	NA	UKB-b-57	TRUE	reported	8KoqAI
6	1.42E+08	G	C	0.018694	0.003143	2.70E-09	0.367696	462346	NA	rs6243051	NA	UKB-b-57	TRUE	reported	8KoqAI
6	1.42E+08	A	G	0.018507	0.003143	3.90E-09	0.367442	462346	NA	rs1776820	NA	UKB-b-57	TRUE	reported	8KoqAI
6	1.42E+08	A	C	0.018587	0.003144	3.40E-09	0.367276	462346	NA	rs7298012	NA	UKB-b-57	TRUE	reported	8KoqAI
6	1.42E+08	A	C	0.017772	0.003147	1.60E-08	0.365655	462346	NA	rs4280976	NA	UKB-b-57	TRUE	reported	8KoqAI
6	1.42E+08	C	G	0.018463	0.003144	4.30E-09	0.367207	462346	NA	rs1377329	NA	UKB-b-57	TRUE	reported	8KoqAI
6	1.42E+08	G	A	0.018503	0.003144	4.00E-09	0.367169	462346	NA	rs1377328	NA	UKB-b-57	TRUE	reported	8KoqAI
6	1.42E+08	A	G	0.018515	0.003144	3.90E-09	0.367272	462346	NA	rs1377327	NA	UKB-b-57	TRUE	reported	8KoqAI
6	1.42E+08	C	T	0.018553	0.003144	3.60E-09	0.367252	462346	NA	rs1377326	NA	UKB-b-57	TRUE	reported	8KoqAI
6	1.42E+08	G	A	0.018863	0.003147	2.00E-09	0.366069	462346	NA	rs6928489	NA	UKB-b-57	TRUE	reported	8KoqAI
6	1.42E+08	G	A	0.018854	0.003147	2.10E-09	0.366117	462346	NA	rs9376592	NA	UKB-b-57	TRUE	reported	8KoqAI
6	1.42E+08	C	T	0.018874	0.003146	2.00E-09	0.366385	462346	NA	rs9389897	NA	UKB-b-57	TRUE	reported	8KoqAI
6	1.42E+08	T	C	0.018137	0.00316	9.50E-09	0.358593	462346	NA	rs1542311	NA	UKB-b-57	TRUE	reported	8KoqAI
6	1.42E+08	T	C	0.018025	0.003159	1.20E-08	0.359005	462346	NA	rs2845553	NA	UKB-b-57	TRUE	reported	8KoqAI
6	1.42E+08	T	G	0.018497	0.003154	4.50E-09	0.3608	462346	NA	rs7753346	NA	UKB-b-57	TRUE	reported	8KoqAI
6	1.42E+08	T	C	0.016925	0.003072	3.60E-08	0.418469	462346	NA	rs9495937	NA	UKB-b-57	TRUE	reported	8KoqAI
6	1.42E+08	C	A	0.018424	0.00312	3.50E-09	0.381062	462346	NA	rs9389901	NA	UKB-b-57	TRUE	reported	8KoqAI
6	1.42E+08	C	T	0.017368	0.003166	4.10E-08	0.356076	462346	NA	rs6243052	NA	UKB-b-57	TRUE	reported	8KoqAI
6	1.42E+08	T	C	0.017938	0.003124	9.30E-09	0.379361	462346	NA	rs9403279	NA	UKB-b-57	TRUE	reported	8KoqAI
6	1.42E+08	T	C	0.01787	0.003121	1.00E-08	0.380428	462346	NA	rs1530925	NA	UKB-b-57	TRUE	reported	8KoqAI
6	1.42E+08	C	A	0.018049	0.003124	7.60E-09	0.379082	462346	NA	rs1037442	NA	UKB-b-57	TRUE	reported	8KoqAI
6	1.42E+08	C	A	0.018198	0.003121	5.50E-09	0.380254	462346	NA	rs719196	NA	UKB-b-57	TRUE	reported	8KoqAI
6	1.42E+08	C	T	0.017868	0.003123	1.10E-08	0.379315	462346	NA	rs9403284	NA	UKB-b-57	TRUE	reported	8KoqAI
6	1.42E+08	G	A	0.01778	0.003122	1.20E-08	0.379384	462346	NA	rs6936611	NA	UKB-b-57	TRUE	reported	8KoqAI
6	1.42E+08	A	T	0.018284	0.003121	4.70E-09	0.380313	462346	NA	rs1335306	NA	UKB-b-57	TRUE	reported	8KoqAI
6	1.42E+08	T	A	0.018176	0.00312	5.70E-09	0.380629	462346	NA	rs1335303	NA	UKB-b-57	TRUE	reported	8KoqAI
6	1.42E+08	G	A	0.018127	0.003121	6.30E-09	0.380508	462346	NA	rs1217351	NA	UKB-b-57	TRUE	reported	8KoqAI
6	1.42E+08	T	A	0.01787	0.003123	1.10E-08	0.37942	462346	NA	rs1335302	NA	UKB-b-57	TRUE	reported	8KoqAI
6	1.42E+08	C	T	0.018357	0.003121	4.10E-09	0.380332	462346	NA	rs9385949	NA	UKB-b-57	TRUE	reported	8KoqAI
6	1.42E+08	T	C	0.017901	0.003123	9.90E-09	0.379641	462346	NA	rs9389905	NA	UKB-b-57	TRUE	reported	8KoqAI
6	1.42E+08	G	A	0.01823	0.003121	5.20E-09	0.380666	462346	NA	rs5594940	NA	UKB-b-57	TRUE	reported	8KoqAI
6	1.42E+08	A	C	0.018329	0.003121	4.30E-09	0.380919	462346	NA	rs7760560	NA	UKB-b-57	TRUE	reported	8KoqAI
6	1.42E+08	A	G	0.01807	0.003144	9.10E-09	0.366694	462346	NA	rs1335308	NA	UKB-b-57	TRUE	reported	8KoqAI
6	1.42E+08	C	G	0.018263	0.00312	4.80E-09	0.380753	462346	NA	rs1932191	NA	UKB-b-57	TRUE	reported	8KoqAI
6	1.42E+08	G	A	0.018257	0.003122	5.00E-09	0.380321	462346	NA	rs4270804	NA	UKB-b-57	TRUE	reported	8KoqAI
6	1.42E+08	A	G	0.018177	0.003134	6.60E-09	0.371671	462346	NA	rs9376607	NA	UKB-b-57	TRUE	reported	8KoqAI
6	1.42E+08	T	A	0.018408	0.003131	4.10E-09	0.37314	462346	NA	rs9495962	NA	UKB-b-57	TRUE	reported	8KoqAI
6	1.42E+08	G	A	0.018425	0.003131	4.00E-09	0.372846	462346	NA	rs9376609	NA	UKB-b-57	TRUE	reported	8KoqAI
6	1.42E+08	G	T	0.018573	0.003132	3.00E-09	0.372671	462346	NA	rs1175565	NA	UKB-b-57	TRUE	reported	8KoqAI
6	1.42E+08	T	C	0.018223	0.003156	7.80E-09	0.35932	462346	NA	rs9403288	NA	UKB-b-57	TRUE	reported	8KoqAI
6	1.42E+08	T	C	0.018557	0.003132	3.10E-09	0.372664	462346	NA	rs9385951	NA	UKB-b-57	TRUE	reported	8KoqAI
6	1.42E+08	A	T	0.018834	0.003159	2.50E-09	0.368245	462346	NA	rs9376610	NA	UKB-b-57	TRUE	reported	8KoqAI
6	1.42E+08	C	T	0.018291	0.003156	6.80E-09	0.35926	462346	NA	rs9399363	NA	UKB-b-57	TRUE	reported	8KoqAI
6	1.42E+08	C	G	0.018468	0.003131	3.70E-09	0.372897	462346	NA	rs9385953	NA	UKB-b-57	TRUE	reported	8KoqAI
6	1.42E+08	T	A	0.018245	0.003133	5.80E-09	0.371608	462346	NA	rs1176013	NA	UKB-b-57	TRUE	reported	8KoqAI
6	1.42E+08	C	G	0.018499	0.003131	3.40E-09	0.372892	462346	NA	rs9403292	NA	UKB-b-57	TRUE	reported	8KoqAI
6	1.42E+08	C	T	0.017379	0.003079	1.70E-08	0.408463	462346	NA	rs9389913	NA	UKB-b-57	TRUE	reported	8KoqAI
6	1.42E+08	C	A	0.018158	0.003141	7.40E-09	0.368906	462346	NA	rs9376611	NA	UKB-b-57	TRUE	reported	8KoqAI
6	1.42E+08	T	C	0.01863	0.003131	2.70E-09	0.372705	462346	NA	rs9399364	NA	UKB-b-57	TRUE	reported	8KoqAI
6	1.42E+08	C	T	0.017024	0.003079	3.20E-08	0.407466	462346	NA	rs9376613	NA	UKB-b-57	TRUE	reported	8KoqAI
6	1.42E+08	T	C	0.017349	0.003079	1.80E-08	0.408226	462346	NA	rs1776980	NA	UKB-b-57	TRUE	reported	8KoqAI
6	1.42E+08	A	G	0.017401	0.003079	1.60E-08	0.408552	462346	NA	rs9495988	NA	UKB-b-57	TRUE	reported	8KoqAI
6	1.42E+08	G	A	0.017335	0.003079	1.80E-08	0.408416	462346	NA	rs9389914	NA	UKB-b-57	TRUE	reported	8KoqAI
6	1.42E+08	G	T	0.016974	0.00309	4.00E-08	0.403702	462346	NA	rs9385954	NA	UKB-b-57	TRUE	reported	8KoqAI
6	1.42E+08	G	A	0.018609	0.003131	2.80E-09	0.3727	462346	NA	rs9385955	NA	UKB-b-57	TRUE	reported	8KoqAI
6	1.42E+08	T	C	0.017398	0.003079	1.60E-08	0.408521	462346	NA	rs9321812	NA	UKB-b-57	TRUE	reported	8KoqAI
6	1.42E+08	T	C	0.018316	0.003131	4.90E-09	0.373046	462346	NA	rs1175401	NA	UKB-b-57	TRUE	reported	8KoqAI
6	1.42E+08	C	T	0.017378	0.003079	1.70E-08	0.408625	462346	NA	rs2039563	NA	UKB-b-57	TRUE	reported	8KoqAI
6	1.42E+08	C	T	0.017391	0.003079	1.60E-08	0.40858	462346	NA	rs1335300	NA	UKB-b-57	TRUE	reported	8KoqAI
6	1.42E+08	G	A	0.017407	0.003079	1.60E-08	0.408574	462346	NA	rs1335298	NA	UKB-b-57	TRUE	reported	8KoqAI
6	1.42E+08	C	A	0.018621	0.003131	2.70E-09	0.372867	462346	NA	rs7744875	NA	UKB-b-57	TRUE	reported	8KoqAI
6	1.42E+08	G	A	0.017359	0.003079	1.70E-08	0.40828	462346	NA	rs9403295	NA	UKB-b-57	TRUE	reported	8KoqAI
6	1.42E+08	C	T	0.017404	0.003078	1.60E-08	0.408597	462346	NA	rs9403296	NA	UKB-b-57	TRUE	reported	8KoqAI
6	1.42E+08	G	A	0.018823	0.00313	1.80E-09	0.372967	462346	NA	rs9403297	NA	UKB-b-57	TRUE	reported	8KoqAI
6	1.42E+08	G	C	0.018689	0.00313	2.40E-09	0.372779	462346	NA	rs9403298	NA	UKB-b-57	TRUE	reported	8KoqAI
6	1.42E+08	T	A	0.018404	0.003154	5.40E-09	0.360461	462346	NA	rs9403300	NA	UKB-b-57	TRUE	reported	8KoqAI
6	1.42E+08	A	G	0.017442	0.003077	1.40E-08	0.410076	462346	NA	rs9389917	NA	UKB-b-57	TRUE	reported	8KoqAI
6	1.42E+08	G	A	0.017498	0.003078	1.30E-08	0.409948	462346	NA	rs9385956	NA	UKB-b-57	TRUE	reported	8KoqAI
6	1.42E+08	T	C	0.01732	0.003079	1.90E-08	0.408758	462346	NA	rs7767774	NA	UKB-b-57	TRUE	reported	8KoqAI
6	1.42E+08	A	T	0.017355	0.003079	1.70E-08	0.408619	462346	NA	rs9484499	NA	UKB-b-57	TRUE	reported	8KoqAI
6	1.42E+08	C	T	0.017385	0.003088	1.80E-08	0.408429	462346	NA	rs7763498	NA	UKB-b-57	TRUE	reported	8KoqAI
7	1852753	C	T	-0.02512	0.004138	1.30E-09	0.160144	462346	NA	rs1176063	NA	UKB-b-57	TRUE	reported	8KoqAI
7	1853964	G	A	-0.02539	0.004133	8.00E-10	0.160795	462346	NA	rs1176819	NA	UKB-b-57	TRUE	reported	8KoqAI
7	1854159	C	G	-0.02495	0.004139	1.70E-09	0.161084	462346	NA	rs7304631	NA	UKB-b-57	TRUE	reported	8KoqAI
7	1855531	G	A	-0.02552	0.004472	1.20E-08	0.132908	462346	NA	rs7680	NA	UKB-b-57	TRUE	reported	8KoqAI
7	1856273	C	T	-0.02491	0.004166	2.20E-09	0.156502	462346	NA	rs1177223	NA	UKB-b-57	TRUE	reported	8KoqAI
7	1858651	C	T	-0.02455	0.004373	2.00E-08	0.136974	462346	NA	rs1176125	NA	UKB-b-57	TRUE	reported	8KoqAI
7	1860980	G	A	-0.02316	0.004208	3.70E-08	0.153114	462346	NA	rs1176986	NA	UKB-b-57	TRUE	reported	8KoqAI
7	1861695	C	T	-0.02318	0.004208	3.60E-08	0.153107	462346	NA	rs1176415	NA	UKB-b-57	TRUE	reported	8KoqAI
7	1862183	C	T	-0.02315	0.004189	3.30E-08	0.154783	462346	NA	rs5599589	NA	UKB-b-57	TRUE	reported	8KoqAI
7	1896690	C	T	-0.02187	0.003926	2.50E-08	0.18425	462346	NA	rs1176381	NA	UKB-b-57	TRUE	reported	8KoqAI
7	1899447	A	G	-0.02445	0.004015	1.10E-09	0.173838	462346	NA	rs1177014	NA	UKB-b-57	TRUE	reported	8KoqAI
7	1911458	A	C	-0.02457	0.004088	1.90E-09	0.165548	462346	NA	rs1198245	NA	UKB-b-57	TRUE	reported	8KoqAI
7	1913270	G	A	-0.0246	0.004088	1.80E-09	0.165401	462346	NA	rs1197311	NA	UKB-b-57	TRUE	reported	8KoqAI
7	1920356	G	A	-0.02503	0.004066	7.50E-10	0.166954	462346	NA	rs3404019	NA	UKB-b-57	TRUE	reported	8KoqAI
7	1926636	G	A	-0.02556	0.004049	2.70E-10	0.168654	462346	NA	rs6243666	NA	UKB-b-57	TRUE	reported	8KoqAI
7	1929019	G	T	-0.02494	0.004067	8.70E-10	0.166742	462346	NA	rs7304810	NA	UKB-b-57	TRUE	reported	8KoqAI
7	1932936	G	A	-0.02536	0.004296	3.50E-09	0.154439	462346	NA	rs1211363	NA	UKB-b-57	TRUE	reported	8KoqAI
7	1935598	T	C	-0.02524	0.004009	3.10E-10	0.172557	462346	NA	rs7791299	NA	UKB-b-57	TRUE	reported	8KoqAI
7	1935988	T	C	-0.02502	0.00402	4.80E-10	0.171386	462346	NA	rs7791899	NA	UKB-b-57	TRUE	reported	8KoqAI
7	1936938	G	A	-0.02513	0.004013	3.80E-10	0.172169	462346	NA	rs6244289	NA	UKB-b-57	TRUE	reported	8KoqAI
7	1937261	G	A	-0.02522	0.00403	3.90E-10	0.170832	462346	NA	rs1026759	NA	UKB-b-57	TRUE	reported	8KoqAI
7	1939021	T	C	-0.02524	0.004012	3.20E-10	0.172329	462346	NA	rs7807014	NA	UKB-b-57	TRUE	reported	8KoqAI
7	1941003	T	C	-0.02529	0.004016	3.00E-10	0.17199	462346	NA	rs1177182	NA	UKB-b-57	TRUE	reported	8KoqAI
7	1941051	C	T	-0.02517	0.004022	3.90E-10	0.171381	462346	NA	rs1176751	NA	UKB-b-57	TRUE	reported	8KoqAI
7	1945442	C	G	-0.0251	0.004038	5.10E-10	0.169939	462346	NA	rs1176703	NA	UKB-b-57	TRUE	reported	8KoqAI
7	1945468	C	T	-0.02505	0.004036	5.50E-10	0.170133	462346	NA	rs1176704	NA	UKB-b-57	TRUE	reported	8KoqAI
7	1946130	C	G	-0.02537	0.004024	2.90E-10	0.171193	462346	NA	rs5640396	NA	UKB-b-57	TRUE	reported	8KoqAI
7	1948454	C	T	-0.0245	0.003988	8.00E-10	0.174946	462346	NA	rs6244289	NA	UKB-b-57	TRUE	reported	8KoqAI
7	1950337	G	A	-0.02423	0.003975	1.10E-09	0.176173	462346	NA	rs6947019	NA	UKB-b-57	TRUE	reported	8KoqAI
7	1950809	C	T	-0.02416	0.003979	1.30E-09	0.17587	462346	NA	rs4332037	NA	UKB-b-57	TRUE	reported	8KoqAI
7	1952139	T	C	0.023591	0.003973	2.90E-09	0.823196	462346	NA	rs6461009	NA	UKB-b-57	TRUE	reported	8KoqAI

7	1953416	G	A	-0.0218	0.00382	1.10E-08	0.195849	462346	NA	rs6244290	NA	UKB-b-57	TRUE	reported	8KoqAI
7	1953615	G	A	0.02138	0.003816	2.10E-08	0.803819	462346	NA	rs4721188	NA	UKB-b-57	TRUE	reported	8KoqAI
7	1961814	C	A	-0.02583	0.00409	2.70E-10	0.164541	462346	NA	rs2872830	NA	UKB-b-57	TRUE	reported	8KoqAI
7	1961882	C	A	-0.026	0.004091	2.10E-10	0.164488	462346	NA	rs7305012	NA	UKB-b-57	TRUE	reported	8KoqAI
7	1962163	G	T	0.025346	0.004087	5.60E-10	0.835196	462346	NA	rs1026860	NA	UKB-b-57	TRUE	reported	8KoqAI
7	1964357	A	T	0.025405	0.004086	5.10E-10	0.835157	462346	NA	rs1403175	NA	UKB-b-57	TRUE	reported	8KoqAI
7	1970229	C	T	-0.02093	0.003782	3.10E-08	0.200993	462346	NA	rs7844346	NA	UKB-b-57	TRUE	reported	8KoqAI
7	1970649	G	A	-0.02093	0.003782	3.10E-08	0.200962	462346	NA	rs6244291	NA	UKB-b-57	TRUE	reported	8KoqAI
7	1971226	G	A	-0.02103	0.003792	2.90E-08	0.199376	462346	NA	rs6140992	NA	UKB-b-57	TRUE	reported	8KoqAI
7	1973970	C	T	-0.02265	0.003892	5.90E-09	0.186014	462346	NA	rs5609313	NA	UKB-b-57	TRUE	reported	8KoqAI
7	1978002	T	C	-0.02159	0.003879	2.60E-08	0.187371	462346	NA	rs5625910	NA	UKB-b-57	TRUE	reported	8KoqAI
7	1989976	C	T	-0.02249	0.003834	4.50E-09	0.194235	462346	NA	rs6244292	NA	UKB-b-57	TRUE	reported	8KoqAI
7	2002733	T	C	-0.02273	0.003827	2.90E-09	0.194517	462346	NA	rs1177362	NA	UKB-b-57	TRUE	reported	8KoqAI
7	2015047	T	G	-0.02215	0.003882	1.20E-08	0.187335	462346	NA	rs6244294	NA	UKB-b-57	TRUE	reported	8KoqAI
7	2020904	G	C	-0.02246	0.00389	7.70E-09	0.186177	462346	NA	rs1176478	NA	UKB-b-57	TRUE	reported	8KoqAI
7	2021248	C	T	-0.02232	0.003895	1.00E-08	0.185827	462346	NA	rs5579076	NA	UKB-b-57	TRUE	reported	8KoqAI
7	2025001	G	C	-0.02247	0.003889	7.60E-09	0.186189	462346	NA	rs5714837	NA	UKB-b-57	TRUE	reported	8KoqAI
7	2028968	G	T	-0.02208	0.003887	1.30E-08	0.186211	462346	NA	rs3480971	NA	UKB-b-57	TRUE	reported	8KoqAI
7	2032803	C	T	-0.02244	0.003888	7.80E-09	0.186151	462346	NA	rs1176459	NA	UKB-b-57	TRUE	reported	8KoqAI
7	2033780	G	C	-0.02228	0.003891	1.00E-08	0.186008	462346	NA	rs868754	NA	UKB-b-57	TRUE	reported	8KoqAI
7	2048706	A	G	-0.02213	0.003916	1.60E-08	0.18292	462346	NA	rs5630529	NA	UKB-b-57	TRUE	reported	8KoqAI
7	2050747	C	T	-0.022	0.003916	1.90E-08	0.18314	462346	NA	rs5618048	NA	UKB-b-57	TRUE	reported	8KoqAI
7	2051503	C	G	-0.02221	0.003929	1.60E-08	0.18154	462346	NA	rs1151473	NA	UKB-b-57	TRUE	reported	8KoqAI
7	2052318	C	T	-0.02229	0.003929	1.40E-08	0.18144	462346	NA	rs6244488	NA	UKB-b-57	TRUE	reported	8KoqAI
7	2059761	G	A	-0.02333	0.003925	2.80E-09	0.182036	462346	NA	rs5598845	NA	UKB-b-57	TRUE	reported	8KoqAI
7	2060126	C	T	-0.02327	0.003925	3.00E-09	0.182059	462346	NA	rs5601634	NA	UKB-b-57	TRUE	reported	8KoqAI
7	2060775	C	T	-0.0233	0.003924	2.90E-09	0.182165	462346	NA	rs1176254	NA	UKB-b-57	TRUE	reported	8KoqAI
7	2061111	C	A	-0.02328	0.003924	3.00E-09	0.182166	462346	NA	rs1176263	NA	UKB-b-57	TRUE	reported	8KoqAI
7	2070554	T	C	-0.02291	0.003922	5.20E-09	0.182294	462346	NA	rs5577313	NA	UKB-b-57	TRUE	reported	8KoqAI
7	2070680	G	A	-0.02291	0.003922	5.20E-09	0.182296	462346	NA	rs5595273	NA	UKB-b-57	TRUE	reported	8KoqAI
7	2071132	A	G	-0.02297	0.003881	3.20E-09	0.187392	462346	NA	rs5599324	NA	UKB-b-57	TRUE	reported	8KoqAI
7	2071723	C	T	-0.02301	0.003922	4.40E-09	0.182249	462346	NA	rs4470910	NA	UKB-b-57	TRUE	reported	8KoqAI
7	2074122	A	G	-0.02322	0.003881	2.20E-09	0.187287	462346	NA	rs2852899	NA	UKB-b-57	TRUE	reported	8KoqAI
7	2074290	C	T	-0.02321	0.00388	2.20E-09	0.187301	462346	NA	rs1533827	NA	UKB-b-57	TRUE	reported	8KoqAI
7	2078855	G	C	-0.02304	0.003881	2.90E-09	0.187106	462346	NA	rs5568321	NA	UKB-b-57	TRUE	reported	8KoqAI
7	2080114	G	A	-0.0232	0.003882	2.30E-09	0.186939	462346	NA	rs1176375	NA	UKB-b-57	TRUE	reported	8KoqAI
7	2106928	C	T	-0.02217	0.003931	1.70E-08	0.182094	462346	NA	rs3473105	NA	UKB-b-57	TRUE	reported	8KoqAI
7	2109499	C	T	-0.02153	0.003943	4.80E-08	0.18097	462346	NA	rs6244491	NA	UKB-b-57	TRUE	reported	8KoqAI
7	2109821	G	C	-0.02205	0.003932	2.00E-08	0.182081	462346	NA	rs1177262	NA	UKB-b-57	TRUE	reported	8KoqAI
7	2110346	T	C	-0.02175	0.003935	3.30E-08	0.181928	462346	NA	rs3823624	NA	UKB-b-57	TRUE	reported	8KoqAI
7	11500372	C	T	0.035798	0.006382	2.00E-08	0.059921	462346	NA	rs1181348	NA	UKB-b-57	TRUE	reported	8KoqAI
7	39304027	C	G	0.016568	0.00304	5.00E-08	0.48491	462346	NA	rs4316059	NA	UKB-b-57	TRUE	reported	8KoqAI
7	39325539	T	C	0.016839	0.003029	2.70E-08	0.510247	462346	NA	rs6949878	NA	UKB-b-57	TRUE	reported	8KoqAI
7	39325802	G	A	0.01686	0.003029	2.60E-08	0.510245	462346	NA	rs9648478	NA	UKB-b-57	TRUE	reported	8KoqAI
7	39326147	G	A	0.016847	0.003029	2.70E-08	0.510238	462346	NA	rs1270171	NA	UKB-b-57	TRUE	reported	8KoqAI
7	39327468	G	T	0.016839	0.003028	2.70E-08	0.510239	462346	NA	rs4345472	NA	UKB-b-57	TRUE	reported	8KoqAI
7	39327538	A	G	0.016834	0.003028	2.70E-08	0.510232	462346	NA	rs4551233	NA	UKB-b-57	TRUE	reported	8KoqAI
7	39327704	T	C	0.016835	0.003028	2.70E-08	0.51023	462346	NA	rs4336506	NA	UKB-b-57	TRUE	reported	8KoqAI
7	39328581	C	A	0.016706	0.003028	3.40E-08	0.510402	462346	NA	rs6955599	NA	UKB-b-57	TRUE	reported	8KoqAI
7	39329539	G	A	0.016804	0.003027	2.90E-08	0.510053	462346	NA	rs4723841	NA	UKB-b-57	TRUE	reported	8KoqAI
7	72850178	T	C	-0.02092	0.003769	2.80E-08	0.203951	462346	NA	rs1178947	NA	UKB-b-57	TRUE	reported	8KoqAI
7	72856430	T	C	-0.02175	0.003809	1.10E-08	0.195902	462346	NA	rs1178979	NA	UKB-b-57	TRUE	reported	8KoqAI
7	72857049	A	G	-0.02178	0.00381	1.10E-08	0.19586	462346	NA	rs1178977	NA	UKB-b-57	TRUE	reported	8KoqAI
7	72868522	A	G	-0.02168	0.00381	1.30E-08	0.196024	462346	NA	rs1306476	NA	UKB-b-57	TRUE	reported	8KoqAI
7	72883106	T	C	-0.02113	0.003806	2.80E-08	0.197199	462346	NA	rs6246514	NA	UKB-b-57	TRUE	reported	8KoqAI
7	72885810	G	A	-0.02163	0.003813	1.40E-08	0.195718	462346	NA	rs6976930	NA	UKB-b-57	TRUE	reported	8KoqAI
7	72904810	C	T	-0.02162	0.00381	1.40E-08	0.195848	462346	NA	rs1714571	NA	UKB-b-57	TRUE	reported	8KoqAI
7	72923338	A	G	-0.02156	0.00381	1.50E-08	0.195934	462346	NA	rs3443782	NA	UKB-b-57	TRUE	reported	8KoqAI
7	72934510	A	G	-0.02172	0.003812	1.20E-08	0.195899	462346	NA	rs7811265	NA	UKB-b-57	TRUE	reported	8KoqAI
7	72938718	T	C	-0.02179	0.003814	1.10E-08	0.195604	462346	NA	rs6246626	NA	UKB-b-57	TRUE	reported	8KoqAI
7	72939244	G	C	-0.0217	0.003815	1.30E-08	0.19556	462346	NA	rs1198399	NA	UKB-b-57	TRUE	reported	8KoqAI
7	72971531	C	G	-0.01881	0.003363	2.20E-08	0.282037	462346	NA	rs7793710	NA	UKB-b-57	TRUE	reported	8KoqAI
7	72973854	C	A	-0.01838	0.003361	4.60E-08	0.282587	462346	NA	rs1324461	NA	UKB-b-57	TRUE	reported	8KoqAI
7	72976018	C	T	-0.01873	0.003363	2.50E-08	0.281932	462346	NA	rs1322533	NA	UKB-b-57	TRUE	reported	8KoqAI
7	72976048	C	T	-0.01871	0.003363	2.70E-08	0.281877	462346	NA	rs1322534	NA	UKB-b-57	TRUE	reported	8KoqAI
7	72977249	C	T	-0.02266	0.003815	2.90E-09	0.195602	462346	NA	rs3459443	NA	UKB-b-57	TRUE	reported	8KoqAI
7	72977637	T	A	-0.01872	0.003363	2.60E-08	0.281939	462346	NA	rs6946689	NA	UKB-b-57	TRUE	reported	8KoqAI
7	72977898	A	G	-0.01869	0.003363	2.70E-08	0.281922	462346	NA	rs1253931	NA	UKB-b-57	TRUE	reported	8KoqAI
7	72978112	A	G	-0.01872	0.003363	2.60E-08	0.281921	462346	NA	rs1253935	NA	UKB-b-57	TRUE	reported	8KoqAI
7	72988069	C	T	-0.02258	0.003812	3.20E-09	0.195634	462346	NA	rs3565912	NA	UKB-b-57	TRUE	reported	8KoqAI
7	72989390	A	G	-0.02255	0.003812	3.30E-09	0.195761	462346	NA	rs1197440	NA	UKB-b-57	TRUE	reported	8KoqAI
7	72998952	A	G	-0.02247	0.003812	3.80E-09	0.195669	462346	NA	rs9638180	NA	UKB-b-57	TRUE	reported	8KoqAI
7	72999105	T	G	-0.02243	0.003812	4.00E-09	0.195788	462346	NA	rs9638182	NA	UKB-b-57	TRUE	reported	8KoqAI
7	73005776	C	T	-0.02256	0.003804	3.00E-09	0.196759	462346	NA	rs3586650	NA	UKB-b-57	TRUE	reported	8KoqAI
7	73006388	C	T	-0.02256	0.003804	3.00E-09	0.196762	462346	NA	rs1322566	NA	UKB-b-57	TRUE	reported	8KoqAI
7	73007943	G	A	-0.0226	0.003804	2.80E-09	0.196776	462346	NA	rs1051921	NA	UKB-b-57	TRUE	reported	8KoqAI
7	73010442	C	T	-0.02248	0.003806	3.50E-09	0.197165	462346	NA	rs1324787	NA	UKB-b-57	TRUE	reported	8KoqAI
7	73013269	T	C	-0.01829	0.003354	5.00E-08	0.284337	462346	NA	rs3573291	NA	UKB-b-57	TRUE	reported	8KoqAI
7	73013901	C	T	-0.02489	0.004515	3.50E-08	0.128626	462346	NA	rs1323554	NA	UKB-b-57	TRUE	reported	8KoqAI
7	73015369	G	A	-0.02479	0.004519	4.10E-08	0.128437	462346	NA	rs1324006	NA	UKB-b-57	TRUE	reported	8KoqAI
7	73016181	T	C	-0.02342	0.003798	6.90E-10	0.197779	462346	NA	rs3434632	NA	UKB-b-57	TRUE	reported	8KoqAI
7	73016400	A	G	-0.0228	0.003785	1.70E-09	0.200607	462346	NA	rs5638378	NA	UKB-b-57	TRUE	reported	8KoqAI
7	73016862	T	C	-0.02346	0.003798	6.50E-10	0.197781	462346	NA	rs1324099	NA	UKB-b-57	TRUE	reported	8KoqAI
7	73017005	A	G	-0.02336	0.003796	7.60E-10	0.198023	462346	NA	rs1322665	NA	UKB-b-57	TRUE	reported	8KoqAI
7	73017657	C	T	-0.02344	0.003788	6.00E-10	0.198014	462346	NA	rs3536820	NA	UKB-b-57	TRUE	reported	8KoqAI
7	73018524	T	C	-0.01878	0.003354	2.10E-08	0.284418	462346	NA	rs3416676	NA	UKB-b-57	TRUE	reported	8KoqAI
7	73019975	A	G	-0.01917	0.003359	1.20E-08	0.282904	462346	NA	rs6101070	NA	UKB-b-57	TRUE	reported	8KoqAI
7	73020337	C	G	-0.02544	0.004524	1.90E-08	0.128023	462346	NA	rs3812316	NA	UKB-b-57	TRUE	reported	8KoqAI
7	73020676	G	A	-0.01908	0.003359	1.30E-08	0.282787	462346	NA	rs6968170	NA	UKB-b-57	TRUE	reported	8KoqAI
7	73022746	G	A	-0.02561	0.004521	1.50E-08	0.128273	462346	NA	rs1324699	NA	UKB-b-57	TRUE	reported	8KoqAI
7	73025975	A	G	-0.02551	0.004522	1.70E-08	0.128177	462346	NA	rs1323413	NA	UKB-b-57	TRUE	reported	8KoqAI
7	73026151	A	T	-0.02544	0.004522	1.80E-08	0.128187	462346	NA	rs1323437	NA	UKB-b-57	TRUE	reported	8KoqAI
7	73026378	C	T	-0.02281	0.004117	3.00E-08	0.160775	462346	NA	rs1714575	NA	UKB-b-57	TRUE	reported	8KoqAI
7	73027450	C	G	-0.01928	0.003358	9.40E-09	0.283641	462346	NA	rs7808877	NA	UKB-b-57	TRUE	reported	8KoqAI
7	73029437	C	T	-0.02551	0.004503	1.50E-08	0.129759	462346	NA	rs3395198	NA	UKB-b-57	TRUE	reported	8KoqAI
7	73030103	A	C	-0.0194	0.003354	7.30E-09	0.284972	462346	NA	rs2074768	NA	UKB-b-57	TRUE	reported	8KoqAI
7	73030175	G	A	-0.02559	0.004513	1.40E-08	0.129037	462346	NA	rs1322961	NA	UKB-b-57	TRUE	reported	8KoqAI
7	73032265	G	C	-0.01938	0.003355	7.70E-09	0.284951	462346	NA	rs7798357	NA	UKB-b-57	TRUE	reported	8KoqAI
7	73032835	T	C	-0.01938	0.003355	7.70E-09	0.284967	462346	NA	rs7785479	NA	UKB-b-57	TRUE	reported	8KoqAI
7	73033559	G	A	-0.02554	0.004515	1.50E-08	0.129035	462346	NA	rs3406258	NA	UKB-b-57	TRUE	reported	8KoqAI
7	73034030	A	G	-0.01934	0.003359	8.60E-09	0.283875	462346	NA	rs6975495	NA	UKB-b-57	TRUE	reported	8KoqAI
7	73034929	C	T	-0.02544	0.004499	1.60E-08	0.130165	462346	NA	rs7155673	NA	UKB-b-57	TRUE	reported	8KoqAI
7	73035857	T	C	-0.01934	0.003355	8.30E-09	0.284967	462346	NA	rs7800944	NA	UKB-b-57	TRUE	reported	8KoqAI
7	73037366	G	A	-0.02494	0.003774	3.90E-11	0.202206	462346	NA	rs5574770	NA	UKB-b-57	TRUE	reported	8KoqAI
7	73037956	A	G	-0.02651	0.004454	2.60E-09	0.133492	462346	NA	rs3406047	NA	UKB-b-57	TRUE	reported	8KoqAI
7	73039406	C	G	-0.02489	0.003774	4.20E-11	0.202238	462346	NA	rs3549386	NA	UKB-b-57	TRUE	reported	8KoqAI
7	73042085	C	T	-0.02549	0.003774	1.40E-11	0.202827	462346	NA	rs6246631	NA	UKB-b-57	TRUE	reported	8KoqAI

7	73042614	A	G	-0.02107	0.003392	5.20E-10	0.277054	462346	NA	rs7786376	NA	UKB-b-57	TRUE	reported	8KoqAI
7	73043101	G	A	-0.02476	0.004503	3.80E-08	0.12959	462346	NA	rs3495819	NA	UKB-b-57	TRUE	reported	8KoqAI
7	73043665	T	C	-0.02496	0.004508	3.10E-08	0.129273	462346	NA	rs3443094	NA	UKB-b-57	TRUE	reported	8KoqAI
7	73045483	T	C	-0.0249	0.004509	3.30E-08	0.129258	462346	NA	rs7784668	NA	UKB-b-57	TRUE	reported	8KoqAI
7	73045938	T	C	-0.02468	0.004514	4.60E-08	0.128981	462346	NA	rs7881580	NA	UKB-b-57	TRUE	reported	8KoqAI
7	73055461	A	C	-0.02439	0.004475	5.00E-08	0.133393	462346	NA	rs3581400	NA	UKB-b-57	TRUE	reported	8KoqAI
7	73056750	T	C	-0.02286	0.003779	1.50E-09	0.202997	462346	NA	rs6957745	NA	UKB-b-57	TRUE	reported	8KoqAI
7	74283220	C	T	0.020563	0.003388	1.30E-09	0.285382	462346	NA	rs801076	NA	UKB-b-57	TRUE	reported	8KoqAI
7	74358919	C	T	0.022716	0.003496	8.20E-11	0.257128	462346	NA	rs2529287	NA	UKB-b-57	TRUE	reported	8KoqAI
7	74360784	C	G	0.020796	0.003374	7.10E-10	0.289124	462346	NA	rs1637366	NA	UKB-b-57	TRUE	reported	8KoqAI
7	74402206	G	T	0.021876	0.003844	1.30E-08	0.207413	462346	NA	rs3552540	NA	UKB-b-57	TRUE	reported	8KoqAI
7	74844193	C	A	0.021965	0.00376	5.10E-09	0.221435	462346	NA	rs8689755	NA	UKB-b-57	TRUE	reported	8KoqAI
7	99872071	T	C	0.020627	0.003728	3.10E-08	0.208646	462346	NA	rs6943160	NA	UKB-b-57	TRUE	reported	8KoqAI
7	1.33E+08	C	A	0.019321	0.003487	3.00E-08	0.252582	462346	NA	rs4731905	NA	UKB-b-57	TRUE	reported	8KoqAI
7	1.33E+08	G	A	0.019424	0.003488	2.60E-08	0.25258	462346	NA	rs9649581	NA	UKB-b-57	TRUE	reported	8KoqAI
7	1.33E+08	C	T	0.019037	0.003493	5.00E-08	0.251321	462346	NA	rs1266684	NA	UKB-b-57	TRUE	reported	8KoqAI
7	1.33E+08	C	G	0.019112	0.003505	4.90E-08	0.250328	462346	NA	rs4142405	NA	UKB-b-57	TRUE	reported	8KoqAI
7	1.33E+08	C	T	0.019232	0.003505	4.10E-08	0.250373	462346	NA	rs4142404	NA	UKB-b-57	TRUE	reported	8KoqAI
7	1.33E+08	G	A	0.019003	0.003482	4.80E-08	0.254176	462346	NA	rs6952177	NA	UKB-b-57	TRUE	reported	8KoqAI
7	1.33E+08	C	T	0.019062	0.003495	4.90E-08	0.251082	462346	NA	rs6967574	NA	UKB-b-57	TRUE	reported	8KoqAI
7	1.42E+08	G	T	0.021761	0.003102	2.30E-12	0.400576	462346	NA	rs4726481	NA	UKB-b-57	TRUE	reported	8KoqAI
7	1.42E+08	T	C	0.020349	0.00304	2.20E-11	0.447854	462346	NA	rs1024693	NA	UKB-b-57	TRUE	reported	8KoqAI
7	1.42E+08	G	A	-0.02048	0.00304	1.60E-11	0.552066	462346	NA	rs1726866	NA	UKB-b-57	TRUE	reported	8KoqAI
7	1.42E+08	C	G	0.021396	0.003084	4.00E-12	0.402085	462346	NA	rs713598	NA	UKB-b-57	TRUE	reported	8KoqAI
7	1.53E+08	C	T	-0.01724	0.00303	1.30E-08	0.477874	462346	NA	rs2533149	NA	UKB-b-57	TRUE	reported	8KoqAI
7	1.53E+08	T	C	-0.01707	0.003028	1.70E-08	0.490009	462346	NA	rs2533148	NA	UKB-b-57	TRUE	reported	8KoqAI
7	1.53E+08	T	A	-0.01825	0.00306	2.50E-09	0.435103	462346	NA	rs2533147	NA	UKB-b-57	TRUE	reported	8KoqAI
7	1.53E+08	A	T	-0.01734	0.00303	1.00E-08	0.478012	462346	NA	rs2533146	NA	UKB-b-57	TRUE	reported	8KoqAI
7	1.53E+08	T	G	-0.01684	0.003028	2.70E-08	0.489916	462346	NA	rs2533145	NA	UKB-b-57	TRUE	reported	8KoqAI
7	1.53E+08	T	C	-0.01733	0.00303	1.10E-08	0.477989	462346	NA	rs2533141	NA	UKB-b-57	TRUE	reported	8KoqAI
7	1.53E+08	G	C	-0.01692	0.00303	2.40E-08	0.487639	462346	NA	rs2622217	NA	UKB-b-57	TRUE	reported	8KoqAI
7	1.53E+08	G	C	-0.01735	0.00303	1.00E-08	0.477967	462346	NA	rs2622216	NA	UKB-b-57	TRUE	reported	8KoqAI
7	1.53E+08	G	A	-0.01828	0.003061	2.30E-09	0.435118	462346	NA	rs7348818	NA	UKB-b-57	TRUE	reported	8KoqAI
7	1.53E+08	A	G	-0.01695	0.003028	2.20E-08	0.489782	462346	NA	rs7348819	NA	UKB-b-57	TRUE	reported	8KoqAI
7	1.53E+08	A	T	-0.01694	0.003029	2.20E-08	0.489828	462346	NA	rs1024693	NA	UKB-b-57	TRUE	reported	8KoqAI
7	1.53E+08	A	G	-0.01693	0.003029	2.30E-08	0.489865	462346	NA	rs1024719	NA	UKB-b-57	TRUE	reported	8KoqAI
7	1.53E+08	C	G	-0.01836	0.003062	2.00E-09	0.435072	462346	NA	rs2928844	NA	UKB-b-57	TRUE	reported	8KoqAI
7	1.53E+08	G	A	-0.01743	0.003031	9.00E-09	0.477923	462346	NA	rs2928845	NA	UKB-b-57	TRUE	reported	8KoqAI
7	1.53E+08	A	G	-0.01698	0.00303	2.10E-08	0.489846	462346	NA	rs2907674	NA	UKB-b-57	TRUE	reported	8KoqAI
7	1.53E+08	T	C	-0.01747	0.003032	8.30E-09	0.47793	462346	NA	rs2928843	NA	UKB-b-57	TRUE	reported	8KoqAI
7	1.53E+08	A	G	-0.01698	0.00303	2.10E-08	0.4898	462346	NA	rs2533128	NA	UKB-b-57	TRUE	reported	8KoqAI
7	1.53E+08	A	C	-0.01748	0.003032	8.20E-09	0.477938	462346	NA	rs2533127	NA	UKB-b-57	TRUE	reported	8KoqAI
7	1.53E+08	C	T	-0.01813	0.003065	3.30E-09	0.434616	462346	NA	rs2622186	NA	UKB-b-57	TRUE	reported	8KoqAI
7	1.53E+08	A	T	-0.01715	0.003034	1.60E-08	0.477249	462346	NA	rs2622187	NA	UKB-b-57	TRUE	reported	8KoqAI
7	1.53E+08	G	A	-0.01701	0.00303	2.00E-08	0.489772	462346	NA	rs2533126	NA	UKB-b-57	TRUE	reported	8KoqAI
7	1.53E+08	A	G	-0.01698	0.00303	2.10E-08	0.489779	462346	NA	rs2533125	NA	UKB-b-57	TRUE	reported	8KoqAI
7	1.53E+08	G	A	-0.01744	0.003032	8.80E-09	0.477887	462346	NA	rs2533124	NA	UKB-b-57	TRUE	reported	8KoqAI
7	1.53E+08	C	T	-0.01836	0.003064	2.10E-09	0.435166	462346	NA	rs2622129	NA	UKB-b-57	TRUE	reported	8KoqAI
7	1.53E+08	G	T	-0.01672	0.003032	3.50E-08	0.489438	462346	NA	rs987053	NA	UKB-b-57	TRUE	reported	8KoqAI
7	1.53E+08	T	G	-0.01839	0.003064	2.00E-09	0.435235	462346	NA	rs980266	NA	UKB-b-57	TRUE	reported	8KoqAI
7	1.53E+08	A	G	-0.01697	0.003032	2.20E-08	0.491206	462346	NA	rs2533123	NA	UKB-b-57	TRUE	reported	8KoqAI
7	1.53E+08	C	A	-0.01683	0.003038	3.00E-08	0.484414	462346	NA	rs2533273	NA	UKB-b-57	TRUE	reported	8KoqAI
7	1.53E+08	T	C	-0.01712	0.003029	1.60E-08	0.47905	462346	NA	rs1026840	NA	UKB-b-57	TRUE	reported	8KoqAI
7	1.53E+08	A	G	-0.01697	0.003031	2.10E-08	0.479939	462346	NA	rs2622171	NA	UKB-b-57	TRUE	reported	8KoqAI
7	1.53E+08	T	A	-0.01713	0.003029	1.60E-08	0.479054	462346	NA	rs2622170	NA	UKB-b-57	TRUE	reported	8KoqAI
7	1.53E+08	G	A	-0.01912	0.003067	4.60E-10	0.428653	462346	NA	rs2622167	NA	UKB-b-57	TRUE	reported	8KoqAI
7	1.53E+08	G	A	-0.01714	0.003028	1.50E-08	0.479005	462346	NA	rs2622166	NA	UKB-b-57	TRUE	reported	8KoqAI
7	1.53E+08	T	C	-0.01711	0.003028	1.60E-08	0.479018	462346	NA	rs2622165	NA	UKB-b-57	TRUE	reported	8KoqAI
7	1.53E+08	C	T	-0.01706	0.003027	1.70E-08	0.478937	462346	NA	rs6958769	NA	UKB-b-57	TRUE	reported	8KoqAI
7	1.53E+08	G	A	-0.01717	0.003027	1.40E-08	0.479067	462346	NA	rs2098112	NA	UKB-b-57	TRUE	reported	8KoqAI
7	1.53E+08	C	T	-0.01907	0.003066	5.00E-10	0.428827	462346	NA	rs2079822	NA	UKB-b-57	TRUE	reported	8KoqAI
7	1.53E+08	A	G	-0.017	0.003028	2.00E-08	0.478713	462346	NA	rs2622238	NA	UKB-b-57	TRUE	reported	8KoqAI
7	1.53E+08	A	G	-0.01906	0.003083	6.20E-10	0.424234	462346	NA	rs2622237	NA	UKB-b-57	TRUE	reported	8KoqAI
7	1.53E+08	G	A	-0.01716	0.003062	2.10E-08	0.467959	462346	NA	rs6969458	NA	UKB-b-57	TRUE	reported	8KoqAI
7	1.53E+08	T	C	-0.0171	0.003062	2.30E-08	0.468014	462346	NA	rs6951574	NA	UKB-b-57	TRUE	reported	8KoqAI
7	1.53E+08	A	G	-0.01701	0.00303	2.00E-08	0.478749	462346	NA	rs2622226	NA	UKB-b-57	TRUE	reported	8KoqAI
7	1.53E+08	A	G	-0.01701	0.003031	2.00E-08	0.478693	462346	NA	rs2622225	NA	UKB-b-57	TRUE	reported	8KoqAI
7	1.53E+08	T	A	-0.01898	0.003069	6.20E-10	0.428546	462346	NA	rs2622224	NA	UKB-b-57	TRUE	reported	8KoqAI
7	1.53E+08	C	T	-0.01706	0.003031	1.80E-08	0.478645	462346	NA	rs1028226	NA	UKB-b-57	TRUE	reported	8KoqAI
7	1.53E+08	A	G	-0.01905	0.003101	8.10E-10	0.42802	462346	NA	rs2622099	NA	UKB-b-57	TRUE	reported	8KoqAI
7	1.53E+08	G	C	-0.01908	0.003101	7.60E-10	0.428121	462346	NA	rs2622100	NA	UKB-b-57	TRUE	reported	8KoqAI
7	1.53E+08	G	A	-0.01699	0.003067	3.00E-08	0.478462	462346	NA	rs2622101	NA	UKB-b-57	TRUE	reported	8KoqAI
8	30822094	G	A	-0.01723	0.003046	1.50E-08	0.558386	462346	NA	rs6985028	NA	UKB-b-57	TRUE	reported	8KoqAI
8	30825004	A	C	-0.01728	0.003046	1.40E-08	0.558887	462346	NA	rs5906602	NA	UKB-b-57	TRUE	reported	8KoqAI
8	30825080	A	G	-0.01729	0.003046	1.40E-08	0.558884	462346	NA	rs6054358	NA	UKB-b-57	TRUE	reported	8KoqAI
8	30825518	C	T	-0.01717	0.003046	1.70E-08	0.558608	462346	NA	rs9297164	NA	UKB-b-57	TRUE	reported	8KoqAI
8	30828375	G	A	-0.0172	0.003046	1.60E-08	0.558529	462346	NA	rs2725366	NA	UKB-b-57	TRUE	reported	8KoqAI
8	30828427	G	A	-0.01717	0.003047	1.70E-08	0.558698	462346	NA	rs2725367	NA	UKB-b-57	TRUE	reported	8KoqAI
8	30835535	A	G	-0.01865	0.003093	1.60E-09	0.604464	462346	NA	rs2042073	NA	UKB-b-57	TRUE	reported	8KoqAI
8	30837144	C	G	-0.01806	0.003114	6.70E-09	0.61734	462346	NA	rs2543617	NA	UKB-b-57	TRUE	reported	8KoqAI
8	30837459	T	C	-0.01803	0.003115	7.10E-09	0.617368	462346	NA	rs2543616	NA	UKB-b-57	TRUE	reported	8KoqAI
8	30840591	G	A	-0.01867	0.003091	1.50E-09	0.604158	462346	NA	rs2111861	NA	UKB-b-57	TRUE	reported	8KoqAI
8	30840651	C	T	-0.01872	0.003091	1.40E-09	0.604293	462346	NA	rs2160935	NA	UKB-b-57	TRUE	reported	8KoqAI
8	30841331	C	T	-0.01869	0.003091	1.50E-09	0.604252	462346	NA	rs2080720	NA	UKB-b-57	TRUE	reported	8KoqAI
8	30845178	T	C	-0.01869	0.003091	1.50E-09	0.604314	462346	NA	rs2430958	NA	UKB-b-57	TRUE	reported	8KoqAI
8	30847558	A	C	-0.01866	0.003091	1.60E-09	0.604299	462346	NA	rs2543604	NA	UKB-b-57	TRUE	reported	8KoqAI
8	30847760	C	T	-0.0184	0.003089	2.60E-09	0.604081	462346	NA	rs2042074	NA	UKB-b-57	TRUE	reported	8KoqAI
8	30848776	T	C	-0.01858	0.003091	1.80E-09	0.604229	462346	NA	rs2543601	NA	UKB-b-57	TRUE	reported	8KoqAI
8	30851024	G	C	-0.01847	0.003091	2.30E-09	0.604193	462346	NA	rs2725369	NA	UKB-b-57	TRUE	reported	8KoqAI
8	30851260	C	A	-0.01778	0.003089	8.60E-09	0.601503	462346	NA	rs1421333	NA	UKB-b-57	TRUE	reported	8KoqAI
8	30856092	C	A	-0.0185	0.003093	2.20E-09	0.604247	462346	NA	rs2543598	NA	UKB-b-57	TRUE	reported	8KoqAI
8	30856943	A	G	-0.01807	0.003081	4.50E-09	0.594211	462346	NA	rs4733067	NA	UKB-b-57	TRUE	reported	8KoqAI
8	30858054	C	T	-0.01848	0.003093	2.30E-09	0.60391	462346	NA	rs1125841	NA	UKB-b-57	TRUE	reported	8KoqAI
8	30859050	C	T	-0.01807	0.003081	4.40E-09	0.594267	462346	NA	rs1095477	NA	UKB-b-57	TRUE	reported	8KoqAI
8	30863722	G	A	-0.01786	0.003088	7.30E-09	0.593593	462346	NA	rs1008724	NA	UKB-b-57	TRUE	reported	8KoqAI
8	87212302	A	G	-0.02264	0.004149	4.90E-08	0.157333	462346	NA	rs6981293	NA	UKB-b-57	TRUE	reported	8KoqAI
8	87214346	C	T	-0.02268	0.004151	4.60E-08	0.157151	462346	NA	rs3444085	NA	UKB-b-57	TRUE	reported	8KoqAI
8	87216432	T	C	-0.02262	0.00415	5.00E-08	0.157223	462346	NA	rs1177537	NA	UKB-b-57	TRUE	reported	8KoqAI
8	87216600	G	A	-0.02267	0.00415	4.70E-08	0.157169	462346	NA	rs1178425	NA	UKB-b-57	TRUE	reported	8KoqAI
8	87216851	G	C	-0.02267	0.00415	4.70E-08	0.157172	462346	NA	rs3409234	NA	UKB-b-57	TRUE	reported	8KoqAI
8	1.42E+08	T	C	-0.02097	0.003838	4.70E-08	0.192081	462346	NA	rs2235117	NA	UKB-b-57	TRUE	reported	8KoqAI
8	1.42E+08	T	C	-0.02135	0.003844	2.80E-08	0.192176	462346	NA	rs2977453	NA	UKB-b-57	TRUE	reported	8KoqAI
8	1.42E+08	C	G	-0.02592	0.004599	1.70E-08	0.124072	462346	NA	rs2977454	NA	UKB-b-57	TRUE	reported	8KoqAI
8	1.42E+08	C	T	-0.02168	0.003889	2.50E-08	0.188046	462346	NA	rs2977464	NA	UKB-b-57	TRUE	reported	8KoqAI
8	1.42E+08	C	G	-0.0217	0.00389	2.40E-08	0.187728	462346	NA	rs2944772	NA	UKB-b-57	TRUE	reported	8KoqAI
8	1.43E+08	C	T	0.022212	0.003209	4.40E-12	0.388717	462346	NA	rs1326626	NA	UKB-b-57	TRUE	reported	8KoqAI
8	1.43E+08	G	A	-0.01821	0.003296	3.30E-08	0.32596	462346	NA	rs3569262	NA	UKB-b-57	TRUE	reported	8KoqAI

8	1.43E+08	C	T	0.024416	0.003201	2.40E-14	0.369127	462346	NA	rs1178721	NA	UKB-b-57	TRUE	reported	8KoqAI
8	1.43E+08	T	C	-0.01836	0.003308	2.90E-08	0.318621	462346	NA	rs1326241	NA	UKB-b-57	TRUE	reported	8KoqAI
8	1.43E+08	G	T	0.022442	0.003163	1.30E-12	0.383687	462346	NA	rs1178207	NA	UKB-b-57	TRUE	reported	8KoqAI
8	1.43E+08	C	G	0.023604	0.003177	1.10E-13	0.3751	462346	NA	rs746839	NA	UKB-b-57	TRUE	reported	8KoqAI
8	1.43E+08	C	T	-0.01827	0.003299	3.10E-08	0.3174	462346	NA	rs751100	NA	UKB-b-57	TRUE	reported	8KoqAI
8	1.43E+08	G	A	0.022072	0.003152	2.50E-12	0.38407	462346	NA	rs1106761	NA	UKB-b-57	TRUE	reported	8KoqAI
8	1.43E+08	C	T	0.022088	0.003151	2.40E-12	0.383906	462346	NA	rs1078141	NA	UKB-b-57	TRUE	reported	8KoqAI
8	1.43E+08	C	T	-0.01854	0.003294	1.80E-08	0.316359	462346	NA	rs6251343	NA	UKB-b-57	TRUE	reported	8KoqAI
8	1.43E+08	C	T	-0.01854	0.003291	1.80E-08	0.316454	462346	NA	rs7011675	NA	UKB-b-57	TRUE	reported	8KoqAI
8	1.43E+08	A	G	-0.0181	0.003289	3.70E-08	0.317214	462346	NA	rs307426	NA	UKB-b-57	TRUE	reported	8KoqAI
8	1.43E+08	G	A	-0.01861	0.003289	1.50E-08	0.316144	462346	NA	rs898631	NA	UKB-b-57	TRUE	reported	8KoqAI
8	1.43E+08	C	T	-0.01892	0.003245	5.60E-09	0.332152	462346	NA	rs754583	NA	UKB-b-57	TRUE	reported	8KoqAI
8	1.43E+08	G	A	-0.0189	0.003244	5.70E-09	0.331989	462346	NA	rs4907360	NA	UKB-b-57	TRUE	reported	8KoqAI
8	1.43E+08	C	T	-0.01909	0.003245	4.00E-09	0.331832	462346	NA	rs4907324	NA	UKB-b-57	TRUE	reported	8KoqAI
8	1.43E+08	G	T	-0.02257	0.003065	1.80E-13	0.546241	462346	NA	rs1566085	NA	UKB-b-57	TRUE	reported	8KoqAI
8	1.43E+08	A	G	-0.01867	0.003231	7.60E-09	0.330552	462346	NA	rs4907361	NA	UKB-b-57	TRUE	reported	8KoqAI
8	1.43E+08	G	A	-0.01869	0.00323	7.20E-09	0.329724	462346	NA	rs3524343	NA	UKB-b-57	TRUE	reported	8KoqAI
8	1.43E+08	A	C	0.021265	0.003105	7.50E-12	0.397184	462346	NA	rs1178266	NA	UKB-b-57	TRUE	reported	8KoqAI
8	1.43E+08	G	A	-0.01873	0.003224	6.30E-09	0.329612	462346	NA	rs6251344	NA	UKB-b-57	TRUE	reported	8KoqAI
8	1.43E+08	A	G	-0.01873	0.003224	6.20E-09	0.329638	462346	NA	rs6251344	NA	UKB-b-57	TRUE	reported	8KoqAI
8	1.43E+08	A	G	0.021232	0.0031	7.50E-12	0.397206	462346	NA	rs7357604	NA	UKB-b-57	TRUE	reported	8KoqAI
8	1.43E+08	T	A	0.021263	0.0031	6.90E-12	0.397269	462346	NA	rs903959	NA	UKB-b-57	TRUE	reported	8KoqAI
8	1.43E+08	A	G	-0.01885	0.00322	4.80E-09	0.329881	462346	NA	rs3417827	NA	UKB-b-57	TRUE	reported	8KoqAI
8	1.43E+08	C	T	0.020149	0.00312	1.10E-10	0.38648	462346	NA	rs2874460	NA	UKB-b-57	TRUE	reported	8KoqAI
8	1.43E+08	C	T	-0.0186	0.003222	7.80E-09	0.330347	462346	NA	rs4907369	NA	UKB-b-57	TRUE	reported	8KoqAI
8	1.43E+08	C	T	0.019961	0.003107	1.30E-10	0.391821	462346	NA	rs1254968	NA	UKB-b-57	TRUE	reported	8KoqAI
8	1.43E+08	C	T	-0.01869	0.003222	6.60E-09	0.330469	462346	NA	rs4289850	NA	UKB-b-57	TRUE	reported	8KoqAI
8	1.43E+08	G	A	-0.01867	0.003223	6.80E-09	0.330293	462346	NA	rs3457225	NA	UKB-b-57	TRUE	reported	8KoqAI
8	1.43E+08	T	C	0.020123	0.003117	1.10E-10	0.385511	462346	NA	rs1177533	NA	UKB-b-57	TRUE	reported	8KoqAI
8	1.43E+08	C	A	-0.01828	0.003275	2.40E-08	0.311873	462346	NA	rs1327322	NA	UKB-b-57	TRUE	reported	8KoqAI
8	1.43E+08	G	A	0.019713	0.003122	2.70E-10	0.382786	462346	NA	rs7846428	NA	UKB-b-57	TRUE	reported	8KoqAI
8	1.43E+08	G	T	-0.01774	0.003224	3.80E-08	0.330687	462346	NA	rs1328063	NA	UKB-b-57	TRUE	reported	8KoqAI
8	1.43E+08	A	C	-0.01775	0.003224	3.70E-08	0.330819	462346	NA	rs1324902	NA	UKB-b-57	TRUE	reported	8KoqAI
8	1.43E+08	G	A	0.019688	0.003122	2.90E-10	0.382901	462346	NA	rs2379268	NA	UKB-b-57	TRUE	reported	8KoqAI
8	1.43E+08	C	T	0.019653	0.003056	1.30E-10	0.451189	462346	NA	rs1008813	NA	UKB-b-57	TRUE	reported	8KoqAI
8	1.43E+08	G	A	0.020019	0.003131	1.60E-10	0.382262	462346	NA	rs1178221	NA	UKB-b-57	TRUE	reported	8KoqAI
8	1.43E+08	C	T	-0.01813	0.003233	2.00E-08	0.328617	462346	NA	rs2860433	NA	UKB-b-57	TRUE	reported	8KoqAI
8	1.43E+08	G	C	0.019812	0.003202	6.10E-10	0.343361	462346	NA	rs9693779	NA	UKB-b-57	TRUE	reported	8KoqAI
8	1.43E+08	T	G	0.018016	0.003117	7.50E-09	0.387455	462346	NA	rs2869098	NA	UKB-b-57	TRUE	reported	8KoqAI
8	1.43E+08	T	C	0.018196	0.003115	5.20E-09	0.389128	462346	NA	rs5963656	NA	UKB-b-57	TRUE	reported	8KoqAI
8	1.43E+08	G	A	0.019969	0.003231	6.40E-10	0.331364	462346	NA	rs1178608	NA	UKB-b-57	TRUE	reported	8KoqAI
8	1.43E+08	G	A	-0.01831	0.003115	4.20E-09	0.612032	462346	NA	rs1178699	NA	UKB-b-57	TRUE	reported	8KoqAI
8	1.43E+08	T	A	0.020004	0.003138	1.80E-10	0.369862	462346	NA	rs903961	NA	UKB-b-57	TRUE	reported	8KoqAI
8	1.43E+08	C	T	0.020028	0.003137	1.70E-10	0.36935	462346	NA	rs4382507	NA	UKB-b-57	TRUE	reported	8KoqAI
8	1.43E+08	T	C	0.019945	0.003138	2.10E-10	0.368885	462346	NA	rs1178646	NA	UKB-b-57	TRUE	reported	8KoqAI
8	1.43E+08	G	A	-0.01828	0.003115	4.40E-09	0.612994	462346	NA	rs4342639	NA	UKB-b-57	TRUE	reported	8KoqAI
8	1.43E+08	G	A	0.020058	0.003135	1.60E-10	0.369378	462346	NA	rs6994100	NA	UKB-b-57	TRUE	reported	8KoqAI
8	1.43E+08	G	A	0.019971	0.003137	1.90E-10	0.369219	462346	NA	rs7371341	NA	UKB-b-57	TRUE	reported	8KoqAI
8	1.43E+08	C	T	0.019554	0.003126	4.00E-10	0.37845	462346	NA	rs7005575	NA	UKB-b-57	TRUE	reported	8KoqAI
8	1.43E+08	C	T	-0.01949	0.003127	4.60E-10	0.621078	462346	NA	rs7006068	NA	UKB-b-57	TRUE	reported	8KoqAI
8	1.43E+08	T	G	0.019712	0.003151	3.90E-10	0.365763	462346	NA	rs7371341	NA	UKB-b-57	TRUE	reported	8KoqAI
8	1.43E+08	A	G	0.019697	0.003151	4.10E-10	0.365841	462346	NA	rs5720903	NA	UKB-b-57	TRUE	reported	8KoqAI
8	1.43E+08	C	G	0.019643	0.003139	3.90E-10	0.374837	462346	NA	rs5780026	NA	UKB-b-57	TRUE	reported	8KoqAI
8	1.43E+08	T	C	-0.01907	0.003145	1.40E-09	0.626886	462346	NA	rs1008843	NA	UKB-b-57	TRUE	reported	8KoqAI
8	1.43E+08	G	A	0.022501	0.004009	2.00E-08	0.827822	462346	NA	rs9802050	NA	UKB-b-57	TRUE	reported	8KoqAI
8	1.43E+08	G	C	0.022548	0.004008	1.80E-08	0.827961	462346	NA	rs1009213	NA	UKB-b-57	TRUE	reported	8KoqAI
8	1.43E+08	T	C	0.022492	0.004006	2.00E-08	0.827973	462346	NA	rs4977027	NA	UKB-b-57	TRUE	reported	8KoqAI
8	1.43E+08	T	G	0.022589	0.004005	1.70E-08	0.827962	462346	NA	rs4301486	NA	UKB-b-57	TRUE	reported	8KoqAI
8	1.43E+08	G	C	0.022642	0.004005	1.60E-08	0.827974	462346	NA	rs7462663	NA	UKB-b-57	TRUE	reported	8KoqAI
8	1.43E+08	G	A	0.022418	0.004008	2.20E-08	0.828047	462346	NA	rs7463430	NA	UKB-b-57	TRUE	reported	8KoqAI
8	1.43E+08	C	T	0.022361	0.004007	2.40E-08	0.827977	462346	NA	rs4567083	NA	UKB-b-57	TRUE	reported	8KoqAI
8	1.43E+08	T	C	0.022368	0.004007	2.40E-08	0.828049	462346	NA	rs4509378	NA	UKB-b-57	TRUE	reported	8KoqAI
8	1.43E+08	A	C	0.022369	0.004007	2.40E-08	0.828052	462346	NA	rs4509379	NA	UKB-b-57	TRUE	reported	8KoqAI
8	1.43E+08	G	A	0.022394	0.004007	2.30E-08	0.828032	462346	NA	rs4291309	NA	UKB-b-57	TRUE	reported	8KoqAI
8	1.43E+08	T	C	0.022369	0.004007	2.40E-08	0.828051	462346	NA	rs4398970	NA	UKB-b-57	TRUE	reported	8KoqAI
8	1.43E+08	G	T	0.022368	0.004007	2.40E-08	0.828051	462346	NA	rs7834984	NA	UKB-b-57	TRUE	reported	8KoqAI
8	1.43E+08	T	C	0.022364	0.004008	2.40E-08	0.828066	462346	NA	rs7829227	NA	UKB-b-57	TRUE	reported	8KoqAI
8	1.43E+08	C	T	0.022364	0.004008	2.40E-08	0.828066	462346	NA	rs7386438	NA	UKB-b-57	TRUE	reported	8KoqAI
8	1.43E+08	T	C	0.022437	0.004008	2.20E-08	0.828037	462346	NA	rs7387341	NA	UKB-b-57	TRUE	reported	8KoqAI
8	1.43E+08	G	A	0.022435	0.004007	2.20E-08	0.827898	462346	NA	rs7010666	NA	UKB-b-57	TRUE	reported	8KoqAI
8	1.43E+08	C	T	0.022418	0.004006	2.20E-08	0.827885	462346	NA	rs6991858	NA	UKB-b-57	TRUE	reported	8KoqAI
8	1.43E+08	T	C	0.022417	0.004008	2.20E-08	0.828039	462346	NA	rs7004392	NA	UKB-b-57	TRUE	reported	8KoqAI
8	1.43E+08	G	A	0.022404	0.004008	2.30E-08	0.828038	462346	NA	rs7844419	NA	UKB-b-57	TRUE	reported	8KoqAI
8	1.43E+08	C	A	0.022411	0.004008	2.20E-08	0.828002	462346	NA	rs7386893	NA	UKB-b-57	TRUE	reported	8KoqAI
8	1.43E+08	A	G	0.022376	0.004008	2.40E-08	0.828059	462346	NA	rs7387419	NA	UKB-b-57	TRUE	reported	8KoqAI
8	1.43E+08	G	C	0.022315	0.004008	2.60E-08	0.827986	462346	NA	rs7388480	NA	UKB-b-57	TRUE	reported	8KoqAI
8	1.43E+08	T	C	0.022314	0.004008	2.60E-08	0.827986	462346	NA	rs7387733	NA	UKB-b-57	TRUE	reported	8KoqAI
8	1.43E+08	C	G	0.022331	0.004008	2.50E-08	0.827927	462346	NA	rs7827501	NA	UKB-b-57	TRUE	reported	8KoqAI
8	1.43E+08	A	G	0.022403	0.004008	2.30E-08	0.827895	462346	NA	rs7834320	NA	UKB-b-57	TRUE	reported	8KoqAI
8	1.43E+08	C	T	0.022369	0.004008	2.40E-08	0.827939	462346	NA	rs7827631	NA	UKB-b-57	TRUE	reported	8KoqAI
8	1.43E+08	C	T	0.022178	0.004008	3.10E-08	0.82766	462346	NA	rs1326861	NA	UKB-b-57	TRUE	reported	8KoqAI
8	1.43E+08	C	A	0.022106	0.004008	3.50E-08	0.827644	462346	NA	rs1326114	NA	UKB-b-57	TRUE	reported	8KoqAI
8	1.43E+08	A	C	0.022402	0.004009	2.30E-08	0.828042	462346	NA	rs1009397	NA	UKB-b-57	TRUE	reported	8KoqAI
8	1.43E+08	A	G	0.021979	0.004006	4.10E-08	0.826923	462346	NA	rs1009342	NA	UKB-b-57	TRUE	reported	8KoqAI
8	1.43E+08	C	A	0.022428	0.004009	2.20E-08	0.828037	462346	NA	rs1010873	NA	UKB-b-57	TRUE	reported	8KoqAI
8	1.43E+08	A	T	0.022357	0.004009	2.40E-08	0.828019	462346	NA	rs7388074	NA	UKB-b-57	TRUE	reported	8KoqAI
8	1.43E+08	C	T	0.022376	0.00401	2.40E-08	0.828047	462346	NA	rs3576708	NA	UKB-b-57	TRUE	reported	8KoqAI
8	1.43E+08	A	C	0.022374	0.00401	2.40E-08	0.828056	462346	NA	rs6251142	NA	UKB-b-57	TRUE	reported	8KoqAI
8	1.43E+08	T	C	0.022303	0.00401	2.70E-08	0.828069	462346	NA	rs3504288	NA	UKB-b-57	TRUE	reported	8KoqAI
8	1.43E+08	C	A	0.022297	0.00401	2.70E-08	0.828043	462346	NA	rs4928695	NA	UKB-b-57	TRUE	reported	8KoqAI
8	1.43E+08	T	C	0.022255	0.004011	2.90E-08	0.828032	462346	NA	rs6583626	NA	UKB-b-57	TRUE	reported	8KoqAI
8	1.43E+08	T	C	0.022368	0.00401	2.40E-08	0.827827	462346	NA	rs6583627	NA	UKB-b-57	TRUE	reported	8KoqAI
8	1.43E+08	T	C	0.02218	0.004008	3.10E-08	0.827559	462346	NA	rs6583628	NA	UKB-b-57	TRUE	reported	8KoqAI
8	1.43E+08	C	G	0.022301	0.004009	2.70E-08	0.827803	462346	NA	rs7386423	NA	UKB-b-57	TRUE	reported	8KoqAI
8	1.43E+08	A	G	0.022162	0.004011	3.30E-08	0.828064	462346	NA	rs6583630	NA	UKB-b-57	TRUE	reported	8KoqAI
8	1.43E+08	T	G	0.022264	0.004011	2.90E-08	0.828075	462346	NA	rs7465024	NA	UKB-b-57	TRUE	reported	8KoqAI
8	1.43E+08	A	G	0.022592	0.004014	1.80E-08	0.828341	462346	NA	rs3577322	NA	UKB-b-57	TRUE	reported	8KoqAI
8	1.43E+08	T	C	0.022572	0.004014	1.90E-08	0.828345	462346	NA	rs6251143	NA	UKB-b-57	TRUE	reported	8KoqAI
8	1.43E+08	T	C	0.022614	0.004014	1.80E-08	0.828348	462346	NA	rs9720657	NA	UKB-b-57	TRUE	reported	8KoqAI
8	1.43E+08	C	T	0.022541	0.004014	2.00E-08	0.828335	462346	NA	rs9692995	NA	UKB-b-57	TRUE	reported	8KoqAI
8	1.44E+08	A	G	-0.01947	0.003356	6.60E-09	0.292723	462346	NA	rs1325688	NA	UKB-b-57	TRUE	reported	8KoqAI
8	1.44E+08	C	T	-0.01991	0.003349	2.80E-09	0.295753	462346	NA	rs1326394	NA	UKB-b-57	TRUE	reported	8KoqAI
8	1.44E+08	T	C	-0.02171	0.003619	2.00E-09	0.23562	462346	NA	rs7460106	NA	UKB-b-57	TRUE	reported	8KoqAI
9	16287769	T	C	-0.03207	0.005758	2.50E-08	0.074515	462346	NA	rs7467914	NA	UKB-b-57	TRUE	reported	8KoqAI
10	99715744	G	A	0.01665	0.003053	4.90E-08	0.437454	462346	NA	rs489062	NA	UKB-b-57	TRUE	reported	8KoqAI
10	1.03E+08	G	T	0.020374	0.003303	6.90E-10	0.32785	462346	NA	rs6187351	NA	UKB-b-57	TRUE	reported	8KoqAI
10	1.34E+08	C	T	-0.01963	0.003505	2.10E-08	0.250749	462346	NA	rs1276269	NA	UKB-b-57	TRUE	reported	8KoqAI

10	1.34E+08	C	A	-0.01936	0.003497	3.10E-08	0.251829	462346	NA	rs1159186	NA	UKB-b-57	TRUE	reported	8KoqAI
10	1.34E+08	C	T	-0.02031	0.003511	7.20E-09	0.248546	462346	NA	rs1114613	NA	UKB-b-57	TRUE	reported	8KoqAI
10	1.34E+08	T	C	-0.01952	0.00349	2.20E-08	0.252107	462346	NA	rs5814297	NA	UKB-b-57	TRUE	reported	8KoqAI
10	1.34E+08	G	A	-0.01905	0.003491	4.80E-08	0.25169	462346	NA	rs1277197	NA	UKB-b-57	TRUE	reported	8KoqAI
10	1.34E+08	T	A	-0.0199	0.0035	1.30E-08	0.24924	462346	NA	rs3453691	NA	UKB-b-57	TRUE	reported	8KoqAI
10	1.34E+08	G	C	-0.0195	0.003494	2.40E-08	0.250714	462346	NA	rs1181308	NA	UKB-b-57	TRUE	reported	8KoqAI
10	1.34E+08	G	A	-0.02036	0.003503	6.20E-09	0.24819	462346	NA	rs3447388	NA	UKB-b-57	TRUE	reported	8KoqAI
10	1.34E+08	G	A	-0.01929	0.003486	3.10E-08	0.252548	462346	NA	rs7921305	NA	UKB-b-57	TRUE	reported	8KoqAI
10	1.34E+08	G	A	-0.01928	0.003486	3.20E-08	0.252542	462346	NA	rs7921418	NA	UKB-b-57	TRUE	reported	8KoqAI
10	1.34E+08	C	T	-0.0203	0.003498	6.50E-09	0.249881	462346	NA	rs1276176	NA	UKB-b-57	TRUE	reported	8KoqAI
10	1.34E+08	G	T	-0.01935	0.003488	2.90E-08	0.251975	462346	NA	rs1241137	NA	UKB-b-57	TRUE	reported	8KoqAI
10	1.34E+08	G	A	-0.01905	0.003479	4.30E-08	0.253978	462346	NA	rs1276470	NA	UKB-b-57	TRUE	reported	8KoqAI
10	1.34E+08	G	C	-0.01976	0.003473	1.30E-08	0.257279	462346	NA	rs5577171	NA	UKB-b-57	TRUE	reported	8KoqAI
10	1.34E+08	C	T	-0.01959	0.003471	1.70E-08	0.257728	462346	NA	rs5611855	NA	UKB-b-57	TRUE	reported	8KoqAI
10	1.34E+08	G	A	-0.01865	0.003248	9.30E-09	0.680585	462346	NA	rs4242715	NA	UKB-b-57	TRUE	reported	8KoqAI
10	1.34E+08	C	T	-0.01807	0.003216	1.90E-08	0.669344	462346	NA	rs2814162	NA	UKB-b-57	TRUE	reported	8KoqAI
10	1.34E+08	C	T	-0.01809	0.003232	2.20E-08	0.67233	462346	NA	rs2814158	NA	UKB-b-57	TRUE	reported	8KoqAI
10	1.34E+08	G	A	-0.01808	0.003221	2.00E-08	0.667239	462346	NA	rs2637629	NA	UKB-b-57	TRUE	reported	8KoqAI
11	47359706	C	A	0.018349	0.003324	3.40E-08	0.295886	462346	NA	rs1157009	NA	UKB-b-57	TRUE	reported	8KoqAI
11	47365199	G	A	0.018577	0.003331	2.40E-08	0.297891	462346	NA	rs2856650	NA	UKB-b-57	TRUE	reported	8KoqAI
11	47370957	A	C	0.020064	0.003348	2.10E-09	0.307865	462346	NA	rs2071305	NA	UKB-b-57	TRUE	reported	8KoqAI
11	47372377	G	C	0.020937	0.003263	1.40E-10	0.31862	462346	NA	rs2071304	NA	UKB-b-57	TRUE	reported	8KoqAI
11	47374998	A	G	-0.02127	0.003254	6.30E-11	0.681986	462346	NA	rs2856661	NA	UKB-b-57	TRUE	reported	8KoqAI
11	47375193	G	A	0.021185	0.003257	7.80E-11	0.316497	462346	NA	rs1103919	NA	UKB-b-57	TRUE	reported	8KoqAI
11	47376363	A	G	-0.02088	0.003248	1.30E-10	0.679712	462346	NA	rs3740686	NA	UKB-b-57	TRUE	reported	8KoqAI
11	47376448	G	A	-0.02095	0.003245	1.10E-10	0.679614	462346	NA	rs1057233	NA	UKB-b-57	TRUE	reported	8KoqAI
11	47379212	G	C	0.021398	0.003255	4.90E-11	0.316466	462346	NA	rs1103919	NA	UKB-b-57	TRUE	reported	8KoqAI
11	47379407	C	T	-0.02103	0.003243	9.00E-11	0.678891	462346	NA	rs2084946	NA	UKB-b-57	TRUE	reported	8KoqAI
11	47379615	G	A	-0.02106	0.003243	8.40E-11	0.679511	462346	NA	rs1542321	NA	UKB-b-57	TRUE	reported	8KoqAI
11	47379646	A	C	0.021444	0.003254	4.40E-11	0.31649	462346	NA	rs1103920	NA	UKB-b-57	TRUE	reported	8KoqAI
11	47380061	A	C	-0.02111	0.003243	7.70E-11	0.679661	462346	NA	rs1073455	NA	UKB-b-57	TRUE	reported	8KoqAI
11	47382426	A	T	0.021625	0.003237	2.40E-11	0.323229	462346	NA	rs1103920	NA	UKB-b-57	TRUE	reported	8KoqAI
11	47383275	T	C	-0.02136	0.003231	3.80E-11	0.675045	462346	NA	rs7105851	NA	UKB-b-57	TRUE	reported	8KoqAI
11	47384597	A	G	0.021667	0.003236	2.10E-11	0.323275	462346	NA	rs1103920	NA	UKB-b-57	TRUE	reported	8KoqAI
11	47385194	G	A	0.021696	0.003235	2.00E-11	0.323333	462346	NA	rs6711645	NA	UKB-b-57	TRUE	reported	8KoqAI
11	47385923	G	A	-0.02148	0.003228	2.90E-11	0.674595	462346	NA	rs1083869	NA	UKB-b-57	TRUE	reported	8KoqAI
11	47386073	T	C	-0.02148	0.003228	2.90E-11	0.674677	462346	NA	rs1083869	NA	UKB-b-57	TRUE	reported	8KoqAI
11	47387599	C	T	-0.02089	0.003223	9.20E-11	0.673932	462346	NA	rs935914	NA	UKB-b-57	TRUE	reported	8KoqAI
11	47389223	A	G	0.021687	0.00323	1.90E-11	0.323769	462346	NA	rs3599635	NA	UKB-b-57	TRUE	reported	8KoqAI
11	47389676	C	G	-0.02158	0.003227	2.30E-11	0.674412	462346	NA	rs4752827	NA	UKB-b-57	TRUE	reported	8KoqAI
11	47390692	A	G	-0.0215	0.003226	2.60E-11	0.674644	462346	NA	rs4752987	NA	UKB-b-57	TRUE	reported	8KoqAI
11	47391039	C	T	-0.0215	0.003226	2.70E-11	0.674642	462346	NA	rs1076925	NA	UKB-b-57	TRUE	reported	8KoqAI
11	47391745	G	A	0.021831	0.003233	1.50E-11	0.323625	462346	NA	rs6747207	NA	UKB-b-57	TRUE	reported	8KoqAI
11	47391879	G	A	-0.0215	0.003226	2.70E-11	0.674614	462346	NA	rs7928163	NA	UKB-b-57	TRUE	reported	8KoqAI
11	47392114	G	A	-0.02069	0.003217	1.30E-10	0.671002	462346	NA	rs7928419	NA	UKB-b-57	TRUE	reported	8KoqAI
11	47393303	A	T	0.021706	0.003231	1.80E-11	0.323776	462346	NA	rs1103921	NA	UKB-b-57	TRUE	reported	8KoqAI
11	47393638	T	C	-0.02155	0.003227	2.50E-11	0.674149	462346	NA	rs4992357	NA	UKB-b-57	TRUE	reported	8KoqAI
11	47394338	T	C	-0.02162	0.003228	2.10E-11	0.674396	462346	NA	rs896816	NA	UKB-b-57	TRUE	reported	8KoqAI
11	47395240	G	A	-0.02161	0.003228	2.10E-11	0.674386	462346	NA	rs7940536	NA	UKB-b-57	TRUE	reported	8KoqAI
11	47397353	G	A	0.022049	0.003239	9.90E-12	0.321795	462346	NA	rs5603082	NA	UKB-b-57	TRUE	reported	8KoqAI
11	47397714	C	G	0.0218	0.003234	1.60E-11	0.324017	462346	NA	rs5567708	NA	UKB-b-57	TRUE	reported	8KoqAI
11	47398963	G	A	0.022055	0.003239	9.80E-12	0.321351	462346	NA	rs1214656	NA	UKB-b-57	TRUE	reported	8KoqAI
11	47401058	A	G	0.021676	0.003236	2.10E-11	0.323154	462346	NA	rs1160117	NA	UKB-b-57	TRUE	reported	8KoqAI
11	47401060	T	G	0.021711	0.003237	2.00E-11	0.323147	462346	NA	rs1160798	NA	UKB-b-57	TRUE	reported	8KoqAI
11	47401171	A	C	0.021714	0.003234	1.90E-11	0.323436	462346	NA	rs1160160	NA	UKB-b-57	TRUE	reported	8KoqAI
11	47406592	C	T	-0.01955	0.003037	1.20E-10	0.53296	462346	NA	rs1103921	NA	UKB-b-57	TRUE	reported	8KoqAI
11	47409212	C	T	-0.02169	0.00323	1.90E-11	0.675501	462346	NA	rs7111957	NA	UKB-b-57	TRUE	reported	8KoqAI
11	47410200	C	T	-0.02167	0.00323	1.90E-11	0.675503	462346	NA	rs6485753	NA	UKB-b-57	TRUE	reported	8KoqAI
11	47410393	G	A	0.021935	0.003237	1.20E-11	0.322668	462346	NA	rs4752990	NA	UKB-b-57	TRUE	reported	8KoqAI
11	47410951	T	C	-0.02168	0.00323	1.90E-11	0.675514	462346	NA	rs4752993	NA	UKB-b-57	TRUE	reported	8KoqAI
11	47411065	A	T	-0.02168	0.00323	1.90E-11	0.675517	462346	NA	rs4752994	NA	UKB-b-57	TRUE	reported	8KoqAI
11	47411581	G	A	-0.02167	0.00323	2.00E-11	0.675524	462346	NA	rs1076926	NA	UKB-b-57	TRUE	reported	8KoqAI
11	47413475	G	A	0.021824	0.003236	1.50E-11	0.322645	462346	NA	rs1280352	NA	UKB-b-57	TRUE	reported	8KoqAI
11	47413733	A	G	0.021786	0.003235	1.70E-11	0.322747	462346	NA	rs1160567	NA	UKB-b-57	TRUE	reported	8KoqAI
11	47416636	G	A	0.021845	0.003235	1.50E-11	0.322644	462346	NA	rs5587615	NA	UKB-b-57	TRUE	reported	8KoqAI
11	47418306	T	C	0.021609	0.003231	2.30E-11	0.32454	462346	NA	rs1280227	NA	UKB-b-57	TRUE	reported	8KoqAI
11	47418465	C	G	0.021832	0.003236	1.50E-11	0.322726	462346	NA	rs5896562	NA	UKB-b-57	TRUE	reported	8KoqAI
11	47419129	A	G	-0.02168	0.00323	1.90E-11	0.675364	462346	NA	rs7924485	NA	UKB-b-57	TRUE	reported	8KoqAI
11	47419301	T	G	-0.02149	0.00323	2.90E-11	0.675231	462346	NA	rs896815	NA	UKB-b-57	TRUE	reported	8KoqAI
11	47419757	G	T	0.02162	0.003235	2.40E-11	0.322886	462346	NA	rs1317164	NA	UKB-b-57	TRUE	reported	8KoqAI
11	47423553	A	G	-0.0214	0.003229	3.40E-11	0.674962	462346	NA	rs4752832	NA	UKB-b-57	TRUE	reported	8KoqAI
11	47424400	C	T	0.021784	0.003234	1.60E-11	0.322747	462346	NA	rs1103921	NA	UKB-b-57	TRUE	reported	8KoqAI
11	47424717	T	C	0.022016	0.00324	1.10E-11	0.320961	462346	NA	rs1083870	NA	UKB-b-57	TRUE	reported	8KoqAI
11	47426956	G	A	0.021805	0.003235	1.60E-11	0.322864	462346	NA	rs1120606	NA	UKB-b-57	TRUE	reported	8KoqAI
11	47427825	T	C	-0.02135	0.003228	3.80E-11	0.675006	462346	NA	rs1083870	NA	UKB-b-57	TRUE	reported	8KoqAI
11	47428565	C	T	0.02176	0.003234	1.70E-11	0.322885	462346	NA	rs4752999	NA	UKB-b-57	TRUE	reported	8KoqAI
11	47429245	T	A	0.021782	0.003234	1.60E-11	0.322707	462346	NA	rs7107726	NA	UKB-b-57	TRUE	reported	8KoqAI
11	47430458	T	C	-0.02136	0.003228	3.70E-11	0.674932	462346	NA	rs7937331	NA	UKB-b-57	TRUE	reported	8KoqAI
11	47431966	C	T	0.021879	0.003246	1.60E-11	0.320788	462346	NA	rs755555	NA	UKB-b-57	TRUE	reported	8KoqAI
11	47432034	G	C	-0.0214	0.003232	3.60E-11	0.675481	462346	NA	rs755554	NA	UKB-b-57	TRUE	reported	8KoqAI
11	47432303	G	A	-0.02134	0.003227	3.80E-11	0.674972	462346	NA	rs755553	NA	UKB-b-57	TRUE	reported	8KoqAI
11	47434777	G	A	-0.02131	0.003228	4.00E-11	0.675029	462346	NA	rs6485756	NA	UKB-b-57	TRUE	reported	8KoqAI
11	47437202	C	T	-0.0213	0.003228	4.20E-11	0.674989	462346	NA	rs2293577	NA	UKB-b-57	TRUE	reported	8KoqAI
11	47437403	C	T	0.021889	0.003239	1.40E-11	0.321063	462346	NA	rs2293578	NA	UKB-b-57	TRUE	reported	8KoqAI
11	47439444	A	G	0.022006	0.003224	8.70E-12	0.328011	462346	NA	rs2053979	NA	UKB-b-57	TRUE	reported	8KoqAI
11	47439938	A	G	-0.02126	0.003227	4.50E-11	0.674983	462346	NA	rs1074280	NA	UKB-b-57	TRUE	reported	8KoqAI
11	47440283	T	A	-0.02134	0.003227	3.70E-11	0.675077	462346	NA	rs2293580	NA	UKB-b-57	TRUE	reported	8KoqAI
11	47441472	T	A	-0.02315	0.003331	3.70E-12	0.697992	462346	NA	rs3781627	NA	UKB-b-57	TRUE	reported	8KoqAI
11	47442893	T	A	-0.02113	0.003226	5.80E-11	0.675027	462346	NA	rs3781626	NA	UKB-b-57	TRUE	reported	8KoqAI
11	47443080	T	C	0.021527	0.003232	2.70E-11	0.322822	462346	NA	rs3781625	NA	UKB-b-57	TRUE	reported	8KoqAI
11	47446292	A	T	0.021502	0.003232	2.90E-11	0.322815	462346	NA	rs5592779	NA	UKB-b-57	TRUE	reported	8KoqAI
11	47448497	T	C	0.021468	0.003222	2.70E-11	0.325968	462346	NA	rs1160058	NA	UKB-b-57	TRUE	reported	8KoqAI
11	47449101	T	G	0.021325	0.003226	3.80E-11	0.324695	462346	NA	rs6007562	NA	UKB-b-57	TRUE	reported	8KoqAI
11	47450338	C	T	0.021471	0.003232	3.10E-11	0.322821	462346	NA	rs3549653	NA	UKB-b-57	TRUE	reported	8KoqAI
11	47454972	C	G	0.021373	0.003226	3.50E-11	0.324644	462346	NA	rs2868459	NA	UKB-b-57	TRUE	reported	8KoqAI
11	47457539	A	G	0.021845	0.003238	1.50E-11	0.320737	462346	NA	rs1160468	NA	UKB-b-57	TRUE	reported	8KoqAI
11	47458765	T	C	0.021366	0.003227	3.60E-11	0.324585	462346	NA	rs7106956	NA	UKB-b-57	TRUE	reported	8KoqAI
11	47459963	T	A	0.021517	0.003233	2.80E-11	0.322727	462346	NA	rs3570502	NA	UKB-b-57	TRUE	reported	8KoqAI
11	47460901	T	C	0.021798	0.003238	1.70E-11	0.320816	462346	NA	rs1103923	NA	UKB-b-57	TRUE	reported	8KoqAI
11	47466006	G	A	0.021434	0.003228	3.10E-11	0.324413	462346	NA	rs4752838	NA	UKB-b-57	TRUE	reported	8KoqAI
11	47466790	A	T	0.021252	0.003225	4.40E-11	0.32645	462346	NA	rs7925389	NA	UKB-b-57	TRUE	reported	8KoqAI
11	47475189	G	T	0.020767	0.003165	5.30E-11	0.351239	462346	NA	rs3518477	NA	UKB-b-57	TRUE	reported	8KoqAI
11	47481533	T	C	-0.02232	0.00304	2.10E-13	0.446536	462346	NA	rs7950696	NA	UKB-b-57	TRUE	reported	8KoqAI
11	47486885	C	T	0.021286	0.003167	1.80E-11	0.350133	462346	NA	rs1317149	NA	UKB-b-57	TRUE	reported	8KoqAI
11	47495746	G	T	0.020914	0.003169	4.10E-11	0.350136	462346	NA	rs1103925	NA	UKB-b-57	TRUE	reported	8KoqAI
11	47500267	T	C	-0.02284	0.003039	5.60E-14	0.446816	462346	NA	rs2242081	NA	UKB-b-57	TRUE	reported	8KoqAI
11	47504075	G	T	-0.02229	0.003047	2.60E-13	0.448298	462346	NA	rs3740699	NA	UKB-b-57	TRUE	reported	8KoqAI

11	47527052	G	T	0.023111	0.003179	3.60E-13	0.367756	462346	NA	rs1083872	NA	UKB-b-57	TRUE	reported	8KoqAI
11	47529947	C	A	0.022412	0.003072	3.00E-13	0.408308	462346	NA	rs7124681	NA	UKB-b-57	TRUE	reported	8KoqAI
11	47534964	T	C	-0.02262	0.003039	9.80E-14	0.446685	462346	NA	rs2203712	NA	UKB-b-57	TRUE	reported	8KoqAI
11	47539697	T	C	0.02136	0.003167	1.50E-11	0.350021	462346	NA	rs4752845	NA	UKB-b-57	TRUE	reported	8KoqAI
11	47547046	T	C	0.021221	0.003166	2.00E-11	0.350194	462346	NA	rs3495898	NA	UKB-b-57	TRUE	reported	8KoqAI
11	47554337	G	A	-0.0227	0.00304	8.20E-14	0.446601	462346	NA	rs1074281	NA	UKB-b-57	TRUE	reported	8KoqAI
11	47558858	G	A	-0.02262	0.00304	1.00E-13	0.446746	462346	NA	rs1103928	NA	UKB-b-57	TRUE	reported	8KoqAI
11	47566352	T	C	0.022697	0.003071	1.50E-13	0.408806	462346	NA	rs7928842	NA	UKB-b-57	TRUE	reported	8KoqAI
11	47568074	C	T	0.021326	0.003167	1.60E-11	0.350073	462346	NA	rs6674940	NA	UKB-b-57	TRUE	reported	8KoqAI
11	47571808	T	C	-0.02264	0.003039	9.50E-14	0.446653	462346	NA	rs1222659	NA	UKB-b-57	TRUE	reported	8KoqAI
11	47581443	G	A	-0.01868	0.003294	1.40E-08	0.302395	462346	NA	rs1103929	NA	UKB-b-57	TRUE	reported	8KoqAI
11	47583121	C	T	0.021231	0.003166	2.00E-11	0.35012	462346	NA	rs1279834	NA	UKB-b-57	TRUE	reported	8KoqAI
11	47585348	G	T	-0.02374	0.003067	9.80E-15	0.425817	462346	NA	rs7126378	NA	UKB-b-57	TRUE	reported	8KoqAI
11	47586376	T	G	0.021094	0.003166	2.70E-11	0.350247	462346	NA	rs5640041	NA	UKB-b-57	TRUE	reported	8KoqAI
11	47589707	C	T	0.021055	0.003166	2.90E-11	0.350306	462346	NA	rs7945473	NA	UKB-b-57	TRUE	reported	8KoqAI
11	47602729	C	T	0.021037	0.003166	3.00E-11	0.35027	462346	NA	rs2030166	NA	UKB-b-57	TRUE	reported	8KoqAI
11	47604639	C	T	0.022543	0.003073	2.20E-13	0.408548	462346	NA	rs1279802	NA	UKB-b-57	TRUE	reported	8KoqAI
11	47606483	G	A	0.020679	0.003176	7.50E-11	0.349792	462346	NA	rs1160534	NA	UKB-b-57	TRUE	reported	8KoqAI
11	47607940	C	G	-0.02261	0.003039	1.00E-13	0.446728	462346	NA	rs1083873	NA	UKB-b-57	TRUE	reported	8KoqAI
11	47611152	C	T	0.022666	0.003072	1.60E-13	0.408656	462346	NA	rs1103930	NA	UKB-b-57	TRUE	reported	8KoqAI
11	47615798	G	C	0.021085	0.003167	2.80E-11	0.349911	462346	NA	rs3562499	NA	UKB-b-57	TRUE	reported	8KoqAI
11	47622139	G	A	0.020952	0.003167	3.70E-11	0.349784	462346	NA	rs1279962	NA	UKB-b-57	TRUE	reported	8KoqAI
11	47622412	G	A	0.02257	0.003072	2.00E-13	0.40849	462346	NA	rs1103930	NA	UKB-b-57	TRUE	reported	8KoqAI
11	47623890	C	T	0.021279	0.003182	2.30E-11	0.348467	462346	NA	rs1236323	NA	UKB-b-57	TRUE	reported	8KoqAI
11	47624714	C	A	0.021107	0.003167	2.60E-11	0.349952	462346	NA	rs1241969	NA	UKB-b-57	TRUE	reported	8KoqAI
11	47627302	C	T	-0.02255	0.003041	1.20E-13	0.446464	462346	NA	rs6485763	NA	UKB-b-57	TRUE	reported	8KoqAI
11	47640429	G	C	0.02092	0.003169	4.10E-11	0.34886	462346	NA	rs1064608	NA	UKB-b-57	TRUE	reported	8KoqAI
11	47643891	T	A	0.02093	0.003168	4.00E-11	0.348977	462346	NA	rs3817335	NA	UKB-b-57	TRUE	reported	8KoqAI
11	47648042	G	A	0.021041	0.003168	3.10E-11	0.349044	462346	NA	rs4752856	NA	UKB-b-57	TRUE	reported	8KoqAI
11	47650993	C	T	0.022299	0.003073	4.00E-13	0.407521	462346	NA	rs3817334	NA	UKB-b-57	TRUE	reported	8KoqAI
11	47655380	C	T	-0.02253	0.003039	1.20E-13	0.446866	462346	NA	rs7945312	NA	UKB-b-57	TRUE	reported	8KoqAI
11	47655752	C	T	0.021018	0.003167	3.20E-11	0.349838	462346	NA	rs4752857	NA	UKB-b-57	TRUE	reported	8KoqAI
11	47658314	C	T	0.021043	0.003167	3.00E-11	0.349835	462346	NA	rs1279457	NA	UKB-b-57	TRUE	reported	8KoqAI
11	47662932	T	C	-0.01801	0.003295	4.60E-08	0.301644	462346	NA	rs7120548	NA	UKB-b-57	TRUE	reported	8KoqAI
11	47663049	A	G	0.021021	0.003166	3.20E-11	0.350093	462346	NA	rs1083873	NA	UKB-b-57	TRUE	reported	8KoqAI
11	47665686	G	A	0.023148	0.00308	5.60E-14	0.403916	462346	NA	rs1103932	NA	UKB-b-57	TRUE	reported	8KoqAI
11	47667167	G	C	-0.02226	0.003038	2.40E-13	0.448675	462346	NA	rs7129797	NA	UKB-b-57	TRUE	reported	8KoqAI
11	47669175	T	C	0.023156	0.003079	5.50E-14	0.40432	462346	NA	rs5936079	NA	UKB-b-57	TRUE	reported	8KoqAI
11	47669249	A	C	-0.02224	0.003038	2.40E-13	0.450563	462346	NA	rs7107792	NA	UKB-b-57	TRUE	reported	8KoqAI
11	47669289	C	A	-0.02218	0.003038	2.80E-13	0.448747	462346	NA	rs7107922	NA	UKB-b-57	TRUE	reported	8KoqAI
11	47669619	G	A	0.023141	0.003079	5.60E-14	0.40413	462346	NA	rs1241950	NA	UKB-b-57	TRUE	reported	8KoqAI
11	47669665	T	C	0.021614	0.003177	1.00E-11	0.345399	462346	NA	rs1278764	NA	UKB-b-57	TRUE	reported	8KoqAI
11	47672743	C	T	-0.02231	0.003037	2.10E-13	0.450658	462346	NA	rs1083873	NA	UKB-b-57	TRUE	reported	8KoqAI
11	47674084	A	T	0.021555	0.003178	1.20E-11	0.345389	462346	NA	rs1103932	NA	UKB-b-57	TRUE	reported	8KoqAI
11	47674101	G	A	0.02229	0.003039	2.20E-13	0.550227	462346	NA	rs2869024	NA	UKB-b-57	TRUE	reported	8KoqAI
11	47674934	G	A	-0.02223	0.003038	2.50E-13	0.449703	462346	NA	rs7103013	NA	UKB-b-57	TRUE	reported	8KoqAI
11	47675316	T	C	-0.02217	0.003038	2.90E-13	0.449783	462346	NA	rs7119517	NA	UKB-b-57	TRUE	reported	8KoqAI
11	47676085	G	A	-0.02212	0.003039	3.40E-13	0.447966	462346	NA	rs7107535	NA	UKB-b-57	TRUE	reported	8KoqAI
11	47676170	A	G	-0.01916	0.003024	2.40E-10	0.50674	462346	NA	rs7107356	NA	UKB-b-57	TRUE	reported	8KoqAI
11	47679980	A	G	-0.02223	0.003039	2.50E-13	0.447913	462346	NA	rs1083874	NA	UKB-b-57	TRUE	reported	8KoqAI
11	47681370	T	G	-0.02205	0.003039	4.00E-13	0.451035	462346	NA	rs1056387	NA	UKB-b-57	TRUE	reported	8KoqAI
11	47682402	C	T	-0.02222	0.003039	2.60E-13	0.447496	462346	NA	rs7933184	NA	UKB-b-57	TRUE	reported	8KoqAI
11	47682671	T	C	-0.02228	0.003038	2.20E-13	0.449297	462346	NA	rs1236231	NA	UKB-b-57	TRUE	reported	8KoqAI
11	47683765	A	G	-0.02229	0.003038	2.20E-13	0.449288	462346	NA	rs1083874	NA	UKB-b-57	TRUE	reported	8KoqAI
11	47684908	T	C	-0.02224	0.003039	2.50E-13	0.447485	462346	NA	rs1103932	NA	UKB-b-57	TRUE	reported	8KoqAI
11	47685354	C	T	0.022359	0.003038	1.80E-13	0.550303	462346	NA	rs4543938	NA	UKB-b-57	TRUE	reported	8KoqAI
11	47685829	C	T	0.022363	0.003038	1.80E-13	0.550298	462346	NA	rs4752858	NA	UKB-b-57	TRUE	reported	8KoqAI
11	47687147	A	G	0.021116	0.003172	2.80E-11	0.347795	462346	NA	rs1278711	NA	UKB-b-57	TRUE	reported	8KoqAI
11	47689343	C	T	-0.02222	0.003039	2.60E-13	0.447469	462346	NA	rs1074281	NA	UKB-b-57	TRUE	reported	8KoqAI
11	47694699	C	T	0.022378	0.003038	1.80E-13	0.550344	462346	NA	rs4539273	NA	UKB-b-57	TRUE	reported	8KoqAI
11	47695508	G	A	-0.02223	0.003039	2.60E-13	0.447408	462346	NA	rs7116970	NA	UKB-b-57	TRUE	reported	8KoqAI
11	47696214	A	T	-0.0223	0.003038	2.10E-13	0.449244	462346	NA	rs7460815	NA	UKB-b-57	TRUE	reported	8KoqAI
11	47701528	C	A	0.022372	0.00304	1.90E-13	0.550033	462346	NA	rs1228672	NA	UKB-b-57	TRUE	reported	8KoqAI
11	47701819	T	C	-0.02231	0.003038	2.10E-13	0.449232	462346	NA	rs7105791	NA	UKB-b-57	TRUE	reported	8KoqAI
11	47703943	T	C	-0.02266	0.003038	8.80E-14	0.448108	462346	NA	rs3758655	NA	UKB-b-57	TRUE	reported	8KoqAI
11	47704200	T	G	-0.02272	0.003039	7.60E-14	0.448038	462346	NA	rs7936948	NA	UKB-b-57	TRUE	reported	8KoqAI
11	47705015	C	A	0.022837	0.003037	5.50E-14	0.549701	462346	NA	rs4752860	NA	UKB-b-57	TRUE	reported	8KoqAI
11	47706086	C	T	-0.02274	0.003038	7.20E-14	0.448086	462346	NA	rs1083874	NA	UKB-b-57	TRUE	reported	8KoqAI
11	47706141	A	G	0.022867	0.003038	5.10E-14	0.549684	462346	NA	rs1103933	NA	UKB-b-57	TRUE	reported	8KoqAI
11	47706291	A	G	-0.02272	0.003038	7.60E-14	0.44807	462346	NA	rs1083874	NA	UKB-b-57	TRUE	reported	8KoqAI
11	47707156	T	C	-0.02272	0.003038	7.60E-14	0.44806	462346	NA	rs1103933	NA	UKB-b-57	TRUE	reported	8KoqAI
11	47709212	C	T	-0.02273	0.003038	7.30E-14	0.448075	462346	NA	rs1076928	NA	UKB-b-57	TRUE	reported	8KoqAI
11	47715524	T	C	-0.0227	0.003037	7.90E-14	0.449195	462346	NA	rs7943213	NA	UKB-b-57	TRUE	reported	8KoqAI
11	47716324	G	C	0.021531	0.003175	1.20E-11	0.346147	462346	NA	rs1083874	NA	UKB-b-57	TRUE	reported	8KoqAI
11	47716381	T	G	0.02162	0.003175	9.90E-12	0.346129	462346	NA	rs1083874	NA	UKB-b-57	TRUE	reported	8KoqAI
11	47716975	C	A	0.021555	0.003176	1.10E-11	0.346074	462346	NA	rs1103934	NA	UKB-b-57	TRUE	reported	8KoqAI
11	47720228	A	G	-0.02313	0.003036	2.50E-14	0.454448	462346	NA	rs1103934	NA	UKB-b-57	TRUE	reported	8KoqAI
11	47720509	T	C	-0.0231	0.003037	2.80E-14	0.452638	462346	NA	rs7942074	NA	UKB-b-57	TRUE	reported	8KoqAI
11	47722798	G	A	-0.0231	0.003044	3.20E-14	0.452195	462346	NA	rs7935521	NA	UKB-b-57	TRUE	reported	8KoqAI
11	47722819	G	A	-0.02317	0.003045	2.70E-14	0.451969	462346	NA	rs7935528	NA	UKB-b-57	TRUE	reported	8KoqAI
11	47722823	C	G	-0.02318	0.003044	2.70E-14	0.451894	462346	NA	rs7935344	NA	UKB-b-57	TRUE	reported	8KoqAI
11	47723938	A	G	0.023255	0.003036	1.90E-14	0.545099	462346	NA	rs7939420	NA	UKB-b-57	TRUE	reported	8KoqAI
11	47725306	C	T	0.021454	0.00319	1.80E-11	0.342131	462346	NA	rs1160482	NA	UKB-b-57	TRUE	reported	8KoqAI
11	47726977	C	G	0.021707	0.003177	8.30E-12	0.346129	462346	NA	rs1160239	NA	UKB-b-57	TRUE	reported	8KoqAI
11	47728617	G	A	0.021915	0.003179	5.40E-12	0.348281	462346	NA	rs1103934	NA	UKB-b-57	TRUE	reported	8KoqAI
11	47729180	G	A	-0.01958	0.003123	3.70E-10	0.394214	462346	NA	rs1076928	NA	UKB-b-57	TRUE	reported	8KoqAI
11	47729770	C	T	-0.0231	0.003037	2.80E-14	0.452667	462346	NA	rs1278594	NA	UKB-b-57	TRUE	reported	8KoqAI
11	47734444	G	A	-0.02311	0.003037	2.70E-14	0.452662	462346	NA	rs7111509	NA	UKB-b-57	TRUE	reported	8KoqAI
11	47735154	T	A	0.023153	0.003037	2.50E-14	0.544934	462346	NA	rs1103935	NA	UKB-b-57	TRUE	reported	8KoqAI
11	47740662	G	A	-0.02312	0.003037	2.70E-14	0.452671	462346	NA	rs2869534	NA	UKB-b-57	TRUE	reported	8KoqAI
11	47744394	C	A	-0.02309	0.003037	2.90E-14	0.452728	462346	NA	rs1083875	NA	UKB-b-57	TRUE	reported	8KoqAI
11	47745374	C	T	-0.02306	0.003037	3.10E-14	0.452675	462346	NA	rs7102205	NA	UKB-b-57	TRUE	reported	8KoqAI
11	47749960	C	T	0.021709	0.003175	8.00E-12	0.346474	462346	NA	rs7130758	NA	UKB-b-57	TRUE	reported	8KoqAI
11	47752775	A	G	0.021699	0.003174	8.10E-12	0.346629	462346	NA	rs1778893	NA	UKB-b-57	TRUE	reported	8KoqAI
11	47753370	C	T	0.023203	0.003035	2.10E-14	0.544919	462346	NA	rs2290851	NA	UKB-b-57	TRUE	reported	8KoqAI
11	47756475	A	C	0.021877	0.003177	5.70E-12	0.348034	462346	NA	rs1280319	NA	UKB-b-57	TRUE	reported	8KoqAI
11	47756568	C	T	-0.02309	0.003036	2.80E-14	0.452765	462346	NA	rs1563821	NA	UKB-b-57	TRUE	reported	8KoqAI
11	47758449	T	C	0.02177	0.003174	7.00E-12	0.346539	462346	NA	rs3491002	NA	UKB-b-57	TRUE	reported	8KoqAI
11	47758531	A	G	-0.02311	0.003037	2.80E-14	0.454331	462346	NA	rs1083875	NA	UKB-b-57	TRUE	reported	8KoqAI
11	47758532	A	C	-0.02313	0.003036	2.50E-14	0.454577	462346	NA	rs1083875	NA	UKB-b-57	TRUE	reported	8KoqAI
11	47758675	G	A	0.0232	0.003035	2.10E-14	0.544915	462346	NA	rs7116320	NA	UKB-b-57	TRUE	reported	8KoqAI
11	47759841	G	A	0.023213	0.003036	2.10E-14	0.545002	462346	NA	rs4752865	NA	UKB-b-57	TRUE	reported	8KoqAI
11	47761471	C	T	0.021737	0.003174	7.50E-12	0.346544	462346	NA	rs1160233	NA	UKB-b-57	TRUE	reported	8KoqAI
11	47763016	T	C	0.021796	0.003174	6.60E-12	0.346715	462346	NA	rs1083875	NA	UKB-b-57	TRUE	reported	8KoqAI
11	47763509	A	G	-0.02309	0.003036	2.90E-14	0.452723	462346	NA	rs6485773	NA	UKB-b-57	TRUE	reported	8KoqAI
11	47769564	G	A	0.021714	0.003174	7.90E-12	0.346439	462346	NA	rs3492339	NA	UKB-b-57	TRUE	reported	8KoqAI
11	47769936	A	C	-0.02309	0.003036	2.80E-14	0.452768	462346	NA	rs7932703	NA	UKB-b-57	TRUE	reported	8KoqAI

11	47771476	T	C	-0.0231	0.003036	2.80E-14	0.452773	462346	NA	rs1083875	NA	UKB-b-57	TRUE	reported	8KoqAI
11	47772208	T	G	0.023182	0.003035	2.20E-14	0.544908	462346	NA	rs6083325	NA	UKB-b-57	TRUE	reported	8KoqAI
11	47773518	A	G	0.023245	0.003036	1.90E-14	0.545076	462346	NA	rs6485775	NA	UKB-b-57	TRUE	reported	8KoqAI
11	47776283	C	T	-0.02309	0.003036	2.90E-14	0.452773	462346	NA	rs7929014	NA	UKB-b-57	TRUE	reported	8KoqAI
11	47777920	G	C	0.02318	0.003036	2.20E-14	0.54491	462346	NA	rs7122217	NA	UKB-b-57	TRUE	reported	8KoqAI
11	47779045	T	C	-0.02312	0.003036	2.60E-14	0.454594	462346	NA	rs1236064	NA	UKB-b-57	TRUE	reported	8KoqAI
11	47779586	C	T	0.021687	0.003174	8.30E-12	0.346497	462346	NA	rs1242121	NA	UKB-b-57	TRUE	reported	8KoqAI
11	47780222	G	A	0.023181	0.003035	2.20E-14	0.54491	462346	NA	rs1242156	NA	UKB-b-57	TRUE	reported	8KoqAI
11	47781057	C	T	0.023179	0.003035	2.20E-14	0.54491	462346	NA	rs1074282	NA	UKB-b-57	TRUE	reported	8KoqAI
11	47781306	A	G	0.021752	0.003174	7.20E-12	0.346602	462346	NA	rs7927771	NA	UKB-b-57	TRUE	reported	8KoqAI
11	47781460	G	T	-0.02307	0.003036	3.00E-14	0.452775	462346	NA	rs7108479	NA	UKB-b-57	TRUE	reported	8KoqAI
11	47782076	C	T	-0.02308	0.003036	2.90E-14	0.452773	462346	NA	rs6485778	NA	UKB-b-57	TRUE	reported	8KoqAI
11	47783076	G	A	0.021629	0.003174	9.40E-12	0.346837	462346	NA	rs1236103	NA	UKB-b-57	TRUE	reported	8KoqAI
11	47785285	G	C	-0.02311	0.003036	2.70E-14	0.454594	462346	NA	rs1103937	NA	UKB-b-57	TRUE	reported	8KoqAI
11	47785347	T	C	-0.02311	0.003036	2.70E-14	0.45459	462346	NA	rs1083875	NA	UKB-b-57	TRUE	reported	8KoqAI
11	47786184	C	T	0.021726	0.003174	7.60E-12	0.346641	462346	NA	rs7947730	NA	UKB-b-57	TRUE	reported	8KoqAI
11	47786414	A	G	-0.02309	0.003036	2.90E-14	0.452773	462346	NA	rs1083876	NA	UKB-b-57	TRUE	reported	8KoqAI
11	47787445	G	A	-0.02308	0.003036	2.90E-14	0.452767	462346	NA	rs7111576	NA	UKB-b-57	TRUE	reported	8KoqAI
11	47787538	G	A	-0.02308	0.003036	2.90E-14	0.452773	462346	NA	rs7111606	NA	UKB-b-57	TRUE	reported	8KoqAI
11	47789549	G	T	-0.02315	0.003037	2.40E-14	0.452861	462346	NA	rs1103937	NA	UKB-b-57	TRUE	reported	8KoqAI
11	47789776	C	T	-0.02319	0.003036	2.20E-14	0.454674	462346	NA	rs1103937	NA	UKB-b-57	TRUE	reported	8KoqAI
11	47791003	C	T	-0.02316	0.003037	2.40E-14	0.452862	462346	NA	rs1103937	NA	UKB-b-57	TRUE	reported	8KoqAI
11	47791429	C	T	0.023283	0.003035	1.70E-14	0.544922	462346	NA	rs2869541	NA	UKB-b-57	TRUE	reported	8KoqAI
11	47791870	G	A	-0.02316	0.003037	2.40E-14	0.452859	462346	NA	rs7129237	NA	UKB-b-57	TRUE	reported	8KoqAI
11	47794538	G	C	-0.02315	0.003037	2.50E-14	0.45287	462346	NA	rs6189576	NA	UKB-b-57	TRUE	reported	8KoqAI
11	47794802	C	T	-0.02315	0.003037	2.50E-14	0.45287	462346	NA	rs1083876	NA	UKB-b-57	TRUE	reported	8KoqAI
11	47795169	C	T	0.021704	0.003174	8.00E-12	0.346574	462346	NA	rs3590210	NA	UKB-b-57	TRUE	reported	8KoqAI
11	47796062	T	C	0.021622	0.003169	9.00E-12	0.348399	462346	NA	rs1103938	NA	UKB-b-57	TRUE	reported	8KoqAI
11	47796295	C	G	0.021746	0.003174	7.30E-12	0.346614	462346	NA	rs1103939	NA	UKB-b-57	TRUE	reported	8KoqAI
11	47796662	G	A	0.021687	0.003175	8.40E-12	0.346466	462346	NA	rs1103939	NA	UKB-b-57	TRUE	reported	8KoqAI
11	47797917	A	T	-0.02317	0.003036	2.30E-14	0.45469	462346	NA	rs7101597	NA	UKB-b-57	TRUE	reported	8KoqAI
11	47798753	A	G	-0.02316	0.003036	2.40E-14	0.454724	462346	NA	rs1103939	NA	UKB-b-57	TRUE	reported	8KoqAI
11	47799775	C	G	0.021721	0.003174	7.80E-12	0.346462	462346	NA	rs9909	NA	UKB-b-57	TRUE	reported	8KoqAI
11	47801101	A	G	-0.02314	0.003037	2.50E-14	0.45288	462346	NA	rs7114813	NA	UKB-b-57	TRUE	reported	8KoqAI
11	47805418	G	C	0.02316	0.003036	2.40E-14	0.544989	462346	NA	rs7925211	NA	UKB-b-57	TRUE	reported	8KoqAI
11	47805478	T	C	0.02179	0.003175	6.80E-12	0.346638	462346	NA	rs3580582	NA	UKB-b-57	TRUE	reported	8KoqAI
11	47805614	C	A	0.021815	0.003174	6.30E-12	0.346962	462346	NA	rs7105282	NA	UKB-b-57	TRUE	reported	8KoqAI
11	47805856	G	A	-0.02314	0.003037	2.50E-14	0.452893	462346	NA	rs1222643	NA	UKB-b-57	TRUE	reported	8KoqAI
11	47807774	T	C	0.021444	0.003173	1.40E-11	0.346875	462346	NA	rs2290850	NA	UKB-b-57	TRUE	reported	8KoqAI
11	47808836	C	G	0.023245	0.003035	1.90E-14	0.54486	462346	NA	rs1083876	NA	UKB-b-57	TRUE	reported	8KoqAI
11	47810617	C	T	-0.02317	0.003036	2.30E-14	0.452826	462346	NA	rs1083876	NA	UKB-b-57	TRUE	reported	8KoqAI
11	47811933	A	G	-0.02316	0.003035	2.30E-14	0.454682	462346	NA	rs2125838	NA	UKB-b-57	TRUE	reported	8KoqAI
11	47812311	C	T	0.021713	0.003174	7.90E-12	0.346529	462346	NA	rs1278733	NA	UKB-b-57	TRUE	reported	8KoqAI
11	47814280	A	G	-0.02314	0.003036	2.50E-14	0.452825	462346	NA	rs754434	NA	UKB-b-57	TRUE	reported	8KoqAI
11	47815702	G	A	0.023317	0.003035	1.60E-14	0.544458	462346	NA	rs4752792	NA	UKB-b-57	TRUE	reported	8KoqAI
11	47816052	G	A	-0.02318	0.003036	2.20E-14	0.452825	462346	NA	rs1083876	NA	UKB-b-57	TRUE	reported	8KoqAI
11	47817010	T	G	-0.0232	0.003036	2.10E-14	0.453196	462346	NA	rs3886262	NA	UKB-b-57	TRUE	reported	8KoqAI
11	47817290	A	G	-0.02322	0.003036	2.00E-14	0.453191	462346	NA	rs2904127	NA	UKB-b-57	TRUE	reported	8KoqAI
11	47817441	C	T	0.021639	0.003175	9.40E-12	0.346066	462346	NA	rs7120333	NA	UKB-b-57	TRUE	reported	8KoqAI
11	47823056	C	T	-0.02323	0.003036	2.00E-14	0.453172	462346	NA	rs7112248	NA	UKB-b-57	TRUE	reported	8KoqAI
11	47825333	A	G	0.023306	0.003035	1.60E-14	0.544541	462346	NA	rs1083876	NA	UKB-b-57	TRUE	reported	8KoqAI
11	47825590	G	A	-0.02327	0.003036	1.80E-14	0.453229	462346	NA	rs1103940	NA	UKB-b-57	TRUE	reported	8KoqAI
11	47826090	A	C	-0.02327	0.003036	1.80E-14	0.453174	462346	NA	rs1103940	NA	UKB-b-57	TRUE	reported	8KoqAI
11	47828988	C	T	0.023324	0.003035	1.50E-14	0.544546	462346	NA	rs7115228	NA	UKB-b-57	TRUE	reported	8KoqAI
11	47830590	T	C	-0.02324	0.003036	1.90E-14	0.453188	462346	NA	rs1222585	NA	UKB-b-57	TRUE	reported	8KoqAI
11	47831332	A	G	0.023368	0.003035	1.40E-14	0.544536	462346	NA	rs1083877	NA	UKB-b-57	TRUE	reported	8KoqAI
11	47832793	G	A	0.021726	0.003175	7.80E-12	0.346058	462346	NA	rs1160577	NA	UKB-b-57	TRUE	reported	8KoqAI
11	47833398	A	T	0.023333	0.003035	1.50E-14	0.5445	462346	NA	rs1563820	NA	UKB-b-57	TRUE	reported	8KoqAI
11	47834346	G	A	-0.02293	0.00305	5.60E-14	0.44974	462346	NA	rs1083877	NA	UKB-b-57	TRUE	reported	8KoqAI
11	47836062	T	C	0.02169	0.003175	8.50E-12	0.346103	462346	NA	rs1278583	NA	UKB-b-57	TRUE	reported	8KoqAI
11	47837408	G	C	-0.02324	0.003036	1.90E-14	0.453188	462346	NA	rs6485786	NA	UKB-b-57	TRUE	reported	8KoqAI
11	47840622	T	C	0.023302	0.003035	1.60E-14	0.544491	462346	NA	rs2861730	NA	UKB-b-57	TRUE	reported	8KoqAI
11	47841581	G	A	0.021701	0.003176	8.30E-12	0.346087	462346	NA	rs1103941	NA	UKB-b-57	TRUE	reported	8KoqAI
11	47841862	A	G	0.023304	0.003035	1.60E-14	0.544491	462346	NA	rs4752796	NA	UKB-b-57	TRUE	reported	8KoqAI
11	47843133	A	C	0.0233	0.003035	1.60E-14	0.544465	462346	NA	rs7122182	NA	UKB-b-57	TRUE	reported	8KoqAI
11	47844368	G	A	0.023294	0.003035	1.70E-14	0.544485	462346	NA	rs7948468	NA	UKB-b-57	TRUE	reported	8KoqAI
11	47845814	A	G	-0.02322	0.003036	2.00E-14	0.453193	462346	NA	rs1103941	NA	UKB-b-57	TRUE	reported	8KoqAI
11	47846711	G	A	0.021668	0.003176	8.90E-12	0.346008	462346	NA	rs1103941	NA	UKB-b-57	TRUE	reported	8KoqAI
11	47847428	G	A	0.021665	0.003175	8.90E-12	0.346038	462346	NA	rs3598550	NA	UKB-b-57	TRUE	reported	8KoqAI
11	47848442	T	G	-0.02323	0.003036	2.00E-14	0.453209	462346	NA	rs7110300	NA	UKB-b-57	TRUE	reported	8KoqAI
11	47849470	T	G	-0.02323	0.003036	2.00E-14	0.453184	462346	NA	rs9633830	NA	UKB-b-57	TRUE	reported	8KoqAI
11	47851354	C	T	-0.02323	0.003036	2.00E-14	0.453195	462346	NA	rs1103941	NA	UKB-b-57	TRUE	reported	8KoqAI
11	47851376	C	T	0.021749	0.003175	7.40E-12	0.346083	462346	NA	rs3412897	NA	UKB-b-57	TRUE	reported	8KoqAI
11	47852057	T	C	-0.02327	0.003035	1.80E-14	0.45501	462346	NA	rs7123952	NA	UKB-b-57	TRUE	reported	8KoqAI
11	47856114	A	G	-0.02324	0.003036	1.90E-14	0.453205	462346	NA	rs1083877	NA	UKB-b-57	TRUE	reported	8KoqAI
11	47856382	C	G	-0.02327	0.003035	1.80E-14	0.45501	462346	NA	rs1083877	NA	UKB-b-57	TRUE	reported	8KoqAI
11	47857253	T	C	-0.0232	0.003036	2.10E-14	0.455088	462346	NA	rs3816605	NA	UKB-b-57	TRUE	reported	8KoqAI
11	47857520	C	T	0.021685	0.003175	8.50E-12	0.346205	462346	NA	rs4752873	NA	UKB-b-57	TRUE	reported	8KoqAI
11	47860193	C	T	-0.02321	0.003036	2.10E-14	0.453232	462346	NA	rs1083877	NA	UKB-b-57	TRUE	reported	8KoqAI
11	47860954	A	C	-0.02289	0.003044	5.50E-14	0.454811	462346	NA	rs1083877	NA	UKB-b-57	TRUE	reported	8KoqAI
11	47863119	G	A	0.021703	0.003175	8.20E-12	0.346081	462346	NA	rs1103942	NA	UKB-b-57	TRUE	reported	8KoqAI
11	47864392	T	A	-0.0232	0.003036	2.10E-14	0.453233	462346	NA	rs1045891	NA	UKB-b-57	TRUE	reported	8KoqAI
11	47864842	C	T	-0.02322	0.003036	2.10E-14	0.453227	462346	NA	rs1074282	NA	UKB-b-57	TRUE	reported	8KoqAI
11	47865160	G	T	-0.02324	0.003036	1.90E-14	0.455045	462346	NA	rs1076929	NA	UKB-b-57	TRUE	reported	8KoqAI
11	47865560	C	T	-0.02321	0.003036	2.10E-14	0.453228	462346	NA	rs1076930	NA	UKB-b-57	TRUE	reported	8KoqAI
11	47867059	C	T	-0.02356	0.003037	8.70E-15	0.454624	462346	NA	rs1103942	NA	UKB-b-57	TRUE	reported	8KoqAI
11	47868853	C	G	0.021779	0.003175	6.90E-12	0.346082	462346	NA	rs1241885	NA	UKB-b-57	TRUE	reported	8KoqAI
11	47871017	T	C	-0.02326	0.003036	1.80E-14	0.4551	462346	NA	rs7950674	NA	UKB-b-57	TRUE	reported	8KoqAI
11	47871368	G	C	0.021761	0.003175	7.20E-12	0.346065	462346	NA	rs1139454	NA	UKB-b-57	TRUE	reported	8KoqAI
11	47872012	G	T	-0.02323	0.003036	2.00E-14	0.453294	462346	NA	rs1103943	NA	UKB-b-57	TRUE	reported	8KoqAI
11	47873327	G	A	-0.02323	0.003036	2.00E-14	0.453282	462346	NA	rs1103943	NA	UKB-b-57	TRUE	reported	8KoqAI
11	47874364	G	A	0.021589	0.003176	1.10E-11	0.345846	462346	NA	rs4752797	NA	UKB-b-57	TRUE	reported	8KoqAI
11	47875910	T	C	-0.02336	0.003036	1.40E-14	0.453679	462346	NA	rs2170815	NA	UKB-b-57	TRUE	reported	8KoqAI
11	47877493	C	T	0.021671	0.003176	8.90E-12	0.345949	462346	NA	rs1103943	NA	UKB-b-57	TRUE	reported	8KoqAI
11	47877800	A	G	-0.02338	0.003036	1.40E-14	0.455496	462346	NA	rs1083878	NA	UKB-b-57	TRUE	reported	8KoqAI
11	47878264	G	A	-0.02335	0.003036	1.40E-14	0.453679	462346	NA	rs1257693	NA	UKB-b-57	TRUE	reported	8KoqAI
11	47881740	A	G	-0.02336	0.003036	1.40E-14	0.453694	462346	NA	rs7929725	NA	UKB-b-57	TRUE	reported	8KoqAI
11	47882130	C	A	-0.02327	0.003037	1.80E-14	0.453686	462346	NA	rs1236624	NA	UKB-b-57	TRUE	reported	8KoqAI
11	47882341	G	A	-0.02322	0.003037	2.10E-14	0.453732	462346	NA	rs1083878	NA	UKB-b-57	TRUE	reported	8KoqAI
11	47882782	C	T	-0.02335	0.003037	1.50E-14	0.453494	462346	NA	rs7933896	NA	UKB-b-57	TRUE	reported	8KoqAI
11	47883880	G	A	0.023321	0.003036	1.60E-14	0.544025	462346	NA	rs7116346	NA	UKB-b-57	TRUE	reported	8KoqAI
11	47884260	T	C	-0.02323	0.003037	2.00E-14	0.453692	462346	NA	rs1103943	NA	UKB-b-57	TRUE	reported	8KoqAI
11	47886364	T	C	0.02334	0.003036	1.50E-14	0.543991	462346	NA	rs4752798	NA	UKB-b-57	TRUE	reported	8KoqAI
11	47886639	T	A	-0.02326	0.003037	1.90E-14	0.453686	462346	NA	rs7943464	NA	UKB-b-57	TRUE	reported	8KoqAI
11	47887087	T	C	-0.02329	0.003036	1.70E-14	0.455505	462346	NA	rs7944084	NA	UKB-b-57	TRUE	reported	8KoqAI
11	47889545	C	T	0.023301	0.003037	1.70E-14	0.543969	462346	NA	rs7118747	NA	UKB-b-57	TRUE	reported	8KoqAI
11	47889850	C	T	0.021538	0.003177	1.20E-11	0.346312	462346	NA	rs1279810	NA	UKB-b-57	TRUE	reported	8KoqAI

11	47890421	A	T	-0.02326	0.003037	1.90E-14	0.453705	462346	NA	rs7941399	NA	UKB-b-57	TRUE	reported	8KoqAI
11	47898535	A	G	0.021262	0.003183	2.40E-11	0.345875	462346	NA	rs4752881	NA	UKB-b-57	TRUE	reported	8KoqAI
11	47899030	C	T	-0.02308	0.003044	3.40E-14	0.453544	462346	NA	rs1483988	NA	UKB-b-57	TRUE	reported	8KoqAI
11	47902883	G	A	0.021156	0.003185	3.10E-11	0.345808	462346	NA	rs1236443	NA	UKB-b-57	TRUE	reported	8KoqAI
11	47904086	G	A	-0.02154	0.003048	1.60E-12	0.460958	462346	NA	rs1083878	NA	UKB-b-57	TRUE	reported	8KoqAI
11	47907641	A	G	0.019421	0.003146	6.70E-10	0.365521	462346	NA	rs4752801	NA	UKB-b-57	TRUE	reported	8KoqAI
11	47909313	C	T	-0.02123	0.003052	3.50E-12	0.436184	462346	NA	rs2046377	NA	UKB-b-57	TRUE	reported	8KoqAI
11	47910823	T	G	0.019334	0.003146	7.90E-10	0.365461	462346	NA	rs1236125	NA	UKB-b-57	TRUE	reported	8KoqAI
11	47912150	G	A	0.021393	0.003049	2.30E-12	0.559909	462346	NA	rs1471712	NA	UKB-b-57	TRUE	reported	8KoqAI
11	47913097	C	T	0.021392	0.003049	2.30E-12	0.559901	462346	NA	rs7121038	NA	UKB-b-57	TRUE	reported	8KoqAI
11	47914311	C	G	-0.02132	0.003049	2.70E-12	0.439785	462346	NA	rs6485794	NA	UKB-b-57	TRUE	reported	8KoqAI
11	47914941	C	G	0.021206	0.003053	3.80E-12	0.560805	462346	NA	rs2849882	NA	UKB-b-57	TRUE	reported	8KoqAI
11	47915299	A	G	0.019272	0.003173	1.20E-09	0.361101	462346	NA	rs3446793	NA	UKB-b-57	TRUE	reported	8KoqAI
11	47917631	G	A	0.021438	0.003048	2.00E-12	0.559782	462346	NA	rs1103944	NA	UKB-b-57	TRUE	reported	8KoqAI
11	47918416	G	T	0.021341	0.003048	2.50E-12	0.559904	462346	NA	rs1103944	NA	UKB-b-57	TRUE	reported	8KoqAI
11	47932666	G	A	0.019473	0.003145	5.90E-10	0.366984	462346	NA	rs1280224	NA	UKB-b-57	TRUE	reported	8KoqAI
11	47946836	G	A	0.018738	0.003157	2.90E-09	0.363263	462346	NA	rs2930191	NA	UKB-b-57	TRUE	reported	8KoqAI
11	58394154	C	T	0.022401	0.003989	2.00E-08	0.823865	462346	NA	rs550942	NA	UKB-b-57	TRUE	reported	8KoqAI
11	58415508	T	A	0.022877	0.004106	2.50E-08	0.834725	462346	NA	rs629950	NA	UKB-b-57	TRUE	reported	8KoqAI
11	82651993	G	A	0.017283	0.00307	1.80E-08	0.574433	462346	NA	rs7933350	NA	UKB-b-57	TRUE	reported	8KoqAI
11	82675578	A	G	0.016612	0.003041	4.70E-08	0.52864	462346	NA	rs1079266	NA	UKB-b-57	TRUE	reported	8KoqAI
11	82686151	T	C	0.017356	0.003041	1.10E-08	0.504762	462346	NA	rs1060298	NA	UKB-b-57	TRUE	reported	8KoqAI
11	82686632	C	T	0.01737	0.003041	1.10E-08	0.504588	462346	NA	rs1060296	NA	UKB-b-57	TRUE	reported	8KoqAI
11	82688356	A	G	0.017432	0.003041	9.90E-09	0.505254	462346	NA	rs1079266	NA	UKB-b-57	TRUE	reported	8KoqAI
11	82691952	A	G	0.017115	0.003041	1.80E-08	0.507302	462346	NA	rs1123339	NA	UKB-b-57	TRUE	reported	8KoqAI
11	82694032	C	T	0.016573	0.003038	4.90E-08	0.509651	462346	NA	rs7931613	NA	UKB-b-57	TRUE	reported	8KoqAI
11	82710889	A	C	0.017042	0.003038	2.00E-08	0.513417	462346	NA	rs1089806	NA	UKB-b-57	TRUE	reported	8KoqAI
11	82711209	G	A	0.016986	0.003038	2.30E-08	0.513362	462346	NA	rs1089806	NA	UKB-b-57	TRUE	reported	8KoqAI
11	85736436	G	T	0.018725	0.003428	4.70E-08	0.265558	462346	NA	rs6190730	NA	UKB-b-57	TRUE	reported	8KoqAI
11	85749858	T	C	0.018703	0.003428	4.90E-08	0.265741	462346	NA	rs2509606	NA	UKB-b-57	TRUE	reported	8KoqAI
11	85767985	A	C	0.018687	0.003428	5.00E-08	0.265796	462346	NA	rs590055	NA	UKB-b-57	TRUE	reported	8KoqAI
11	1.13E+08	G	C	0.017461	0.003139	2.70E-08	0.384082	462346	NA	rs1760161	NA	UKB-b-57	TRUE	reported	8KoqAI
11	1.13E+08	A	C	0.017671	0.003129	1.60E-08	0.379215	462346	NA	rs7948028	NA	UKB-b-57	TRUE	reported	8KoqAI
11	1.13E+08	C	T	0.017414	0.003129	2.60E-08	0.379035	462346	NA	rs4337071	NA	UKB-b-57	TRUE	reported	8KoqAI
11	1.13E+08	G	A	0.017448	0.003129	2.50E-08	0.378853	462346	NA	rs4630328	NA	UKB-b-57	TRUE	reported	8KoqAI
11	1.13E+08	G	A	0.01718	0.003122	3.70E-08	0.379576	462346	NA	rs1160105	NA	UKB-b-57	TRUE	reported	8KoqAI
11	1.13E+08	C	A	0.017228	0.003122	3.40E-08	0.379569	462346	NA	rs6190278	NA	UKB-b-57	TRUE	reported	8KoqAI
11	1.13E+08	T	C	0.01712	0.00312	4.10E-08	0.379552	462346	NA	rs6190280	NA	UKB-b-57	TRUE	reported	8KoqAI
11	1.13E+08	C	A	0.017149	0.003119	3.80E-08	0.379587	462346	NA	rs7110440	NA	UKB-b-57	TRUE	reported	8KoqAI
11	1.13E+08	C	G	0.017178	0.003119	3.60E-08	0.379616	462346	NA	rs3527707	NA	UKB-b-57	TRUE	reported	8KoqAI
11	1.13E+08	T	C	-0.0172	0.003118	3.50E-08	0.620787	462346	NA	rs7121986	NA	UKB-b-57	TRUE	reported	8KoqAI
11	1.13E+08	G	A	-0.0172	0.003117	3.40E-08	0.620789	462346	NA	rs6589377	NA	UKB-b-57	TRUE	reported	8KoqAI
11	1.13E+08	C	T	-0.0172	0.003117	3.50E-08	0.62079	462346	NA	rs7929453	NA	UKB-b-57	TRUE	reported	8KoqAI
11	1.13E+08	T	C	0.017071	0.003117	4.30E-08	0.378999	462346	NA	rs1760203	NA	UKB-b-57	TRUE	reported	8KoqAI
11	1.13E+08	C	T	-0.01699	0.003117	5.00E-08	0.620965	462346	NA	rs4938021	NA	UKB-b-57	TRUE	reported	8KoqAI
11	1.13E+08	C	T	-0.01699	0.003117	5.00E-08	0.620967	462346	NA	rs4936275	NA	UKB-b-57	TRUE	reported	8KoqAI
11	1.13E+08	G	C	-0.01699	0.003117	5.00E-08	0.620968	462346	NA	rs4936276	NA	UKB-b-57	TRUE	reported	8KoqAI
11	1.22E+08	G	A	0.01784	0.003098	8.50E-09	0.394777	462346	NA	rs1622638	NA	UKB-b-57	TRUE	reported	8KoqAI
11	1.22E+08	T	C	0.017967	0.003099	6.70E-09	0.392206	462346	NA	rs1666658	NA	UKB-b-57	TRUE	reported	8KoqAI
11	1.24E+08	T	C	0.017357	0.003177	4.70E-08	0.349547	462346	NA	rs1121950	NA	UKB-b-57	TRUE	reported	8KoqAI
11	1.34E+08	T	C	0.0205	0.003631	1.60E-08	0.226914	462346	NA	rs1089476	NA	UKB-b-57	TRUE	reported	8KoqAI
11	1.34E+08	G	A	0.025091	0.003754	2.30E-11	0.206155	462346	NA	rs1122361	NA	UKB-b-57	TRUE	reported	8KoqAI
11	1.34E+08	T	C	0.024976	0.003757	3.00E-11	0.206528	462346	NA	rs748919	NA	UKB-b-57	TRUE	reported	8KoqAI
12	23065782	A	G	-0.01858	0.003383	4.00E-08	0.279524	462346	NA	rs2453875	NA	UKB-b-57	TRUE	reported	8KoqAI
12	23727301	A	G	-0.02372	0.004312	3.80E-08	0.147849	462346	NA	rs7298932	NA	UKB-b-57	TRUE	reported	8KoqAI
12	54595546	A	G	-0.0191	0.003079	5.50E-10	0.466837	462346	NA	rs1183478	NA	UKB-b-57	TRUE	reported	8KoqAI
12	54600543	G	C	-0.02006	0.003048	4.60E-11	0.489851	462346	NA	rs1317996	NA	UKB-b-57	TRUE	reported	8KoqAI
12	54604666	C	T	-0.01737	0.00315	3.50E-08	0.371122	462346	NA	rs1087654	NA	UKB-b-57	TRUE	reported	8KoqAI
12	54608556	G	T	-0.01736	0.00318	4.80E-08	0.34744	462346	NA	rs5616231	NA	UKB-b-57	TRUE	reported	8KoqAI
12	54611925	T	C	-0.01739	0.00318	4.50E-08	0.347078	462346	NA	rs1161484	NA	UKB-b-57	TRUE	reported	8KoqAI
12	54612774	C	T	-0.01737	0.00318	4.70E-08	0.347032	462346	NA	rs7294533	NA	UKB-b-57	TRUE	reported	8KoqAI
12	54612871	G	A	-0.01734	0.00318	4.90E-08	0.346817	462346	NA	rs1242553	NA	UKB-b-57	TRUE	reported	8KoqAI
12	54613265	G	A	-0.01736	0.00318	4.80E-08	0.346795	462346	NA	rs2046517	NA	UKB-b-57	TRUE	reported	8KoqAI
12	54613331	T	A	-0.01737	0.00318	4.70E-08	0.347029	462346	NA	rs2046518	NA	UKB-b-57	TRUE	reported	8KoqAI
12	54614253	T	G	-0.01743	0.003181	4.20E-08	0.34701	462346	NA	rs5598084	NA	UKB-b-57	TRUE	reported	8KoqAI
12	54614391	G	A	-0.0175	0.003181	3.80E-08	0.346731	462346	NA	rs5590272	NA	UKB-b-57	TRUE	reported	8KoqAI
12	54615062	A	C	-0.01746	0.003181	4.10E-08	0.346676	462346	NA	rs1710931	NA	UKB-b-57	TRUE	reported	8KoqAI
12	54616729	G	A	-0.01764	0.003182	3.00E-08	0.347293	462346	NA	rs1161259	NA	UKB-b-57	TRUE	reported	8KoqAI
12	54617195	C	G	-0.01765	0.003182	2.90E-08	0.34718	462346	NA	rs1161264	NA	UKB-b-57	TRUE	reported	8KoqAI
12	54619994	G	A	-0.01794	0.0032	2.10E-08	0.340526	462346	NA	rs7967589	NA	UKB-b-57	TRUE	reported	8KoqAI
12	54620593	C	A	-0.02189	0.003038	5.80E-13	0.485517	462346	NA	rs1486339	NA	UKB-b-57	TRUE	reported	8KoqAI
12	54623132	G	A	-0.02663	0.003078	5.10E-18	0.410052	462346	NA	rs5890541	NA	UKB-b-57	TRUE	reported	8KoqAI
12	54623650	C	T	-0.02462	0.00342	6.10E-13	0.268041	462346	NA	rs1242735	NA	UKB-b-57	TRUE	reported	8KoqAI
12	54626752	T	C	-0.02659	0.003076	5.40E-18	0.41002	462346	NA	rs1056010	NA	UKB-b-57	TRUE	reported	8KoqAI
12	54627090	C	T	-0.02462	0.003419	6.00E-13	0.267887	462346	NA	rs2272470	NA	UKB-b-57	TRUE	reported	8KoqAI
12	54627857	A	G	-0.02646	0.003075	7.80E-18	0.410494	462346	NA	rs6012732	NA	UKB-b-57	TRUE	reported	8KoqAI
12	54634666	T	C	-0.02785	0.004083	9.00E-12	0.164836	462346	NA	rs1242561	NA	UKB-b-57	TRUE	reported	8KoqAI
12	54635846	G	T	-0.02654	0.003073	5.80E-18	0.410243	462346	NA	rs2293602	NA	UKB-b-57	TRUE	reported	8KoqAI
12	54636783	T	C	-0.02651	0.003074	6.30E-18	0.410102	462346	NA	rs4759349	NA	UKB-b-57	TRUE	reported	8KoqAI
12	54646921	C	G	-0.02644	0.003073	7.70E-18	0.410259	462346	NA	rs6580979	NA	UKB-b-57	TRUE	reported	8KoqAI
12	54646996	C	T	-0.02979	0.005336	2.40E-08	0.090914	462346	NA	rs8000978	NA	UKB-b-57	TRUE	reported	8KoqAI
12	54651471	A	G	-0.0264	0.003073	8.60E-18	0.410485	462346	NA	rs1140681	NA	UKB-b-57	TRUE	reported	8KoqAI
12	54652654	G	A	0.025251	0.003075	2.20E-16	0.55009	462346	NA	rs1021973	NA	UKB-b-57	TRUE	reported	8KoqAI
12	54653258	G	A	-0.02654	0.003074	5.90E-18	0.409859	462346	NA	rs4759073	NA	UKB-b-57	TRUE	reported	8KoqAI
12	54660427	G	A	-0.02653	0.003072	5.70E-18	0.410205	462346	NA	rs5728106	NA	UKB-b-57	TRUE	reported	8KoqAI
12	54661102	T	C	-0.02646	0.003072	7.10E-18	0.410263	462346	NA	rs1710990	NA	UKB-b-57	TRUE	reported	8KoqAI
12	54664097	C	T	-0.02647	0.003072	7.10E-18	0.410259	462346	NA	rs4759074	NA	UKB-b-57	TRUE	reported	8KoqAI
12	54667285	T	C	-0.02641	0.003074	8.60E-18	0.410178	462346	NA	rs4759075	NA	UKB-b-57	TRUE	reported	8KoqAI
12	54668471	T	C	-0.01851	0.003193	6.70E-09	0.652609	462346	NA	rs7964426	NA	UKB-b-57	TRUE	reported	8KoqAI
12	54668908	T	C	-0.02583	0.003106	8.90E-17	0.388477	462346	NA	rs1711010	NA	UKB-b-57	TRUE	reported	8KoqAI
12	54670055	T	C	-0.02632	0.003075	1.10E-17	0.410017	462346	NA	rs1920046	NA	UKB-b-57	TRUE	reported	8KoqAI
12	54670398	C	T	-0.02644	0.003075	8.10E-18	0.410262	462346	NA	rs1920045	NA	UKB-b-57	TRUE	reported	8KoqAI
12	54670880	C	G	-0.02639	0.003075	9.30E-18	0.410256	462346	NA	rs4237798	NA	UKB-b-57	TRUE	reported	8KoqAI
12	54674235	A	G	-0.02657	0.00308	6.30E-18	0.407726	462346	NA	rs3809162	NA	UKB-b-57	TRUE	reported	8KoqAI
12	54677980	C	T	-0.01787	0.003187	2.10E-08	0.652559	462346	NA	rs7967622	NA	UKB-b-57	TRUE	reported	8KoqAI
12	54683639	T	A	-0.02551	0.003085	1.30E-16	0.410072	462346	NA	rs5594715	NA	UKB-b-57	TRUE	reported	8KoqAI
12	54684955	T	C	-0.01775	0.003184	2.50E-08	0.652527	462346	NA	rs1182925	NA	UKB-b-57	TRUE	reported	8KoqAI
12	54687232	A	C	-0.0176	0.00315	2.30E-08	0.639684	462346	NA	rs1050632	NA	UKB-b-57	TRUE	reported	8KoqAI
12	54699586	T	A	-0.02149	0.003157	1.00E-11	0.367918	462346	NA	rs1117086	NA	UKB-b-57	TRUE	reported	8KoqAI
12	54701896	A	G	-0.02164	0.003159	7.30E-12	0.367129	462346	NA	rs1087654	NA	UKB-b-57	TRUE	reported	8KoqAI
12	54702519	A	G	-0.02135	0.003156	1.30E-11	0.368494	462346	NA	rs7299922	NA	UKB-b-57	TRUE	reported	8KoqAI
12	54702868	C	T	-0.02793	0.004581	1.10E-09	0.127518	462346	NA	rs6192143	NA	UKB-b-57	TRUE	reported	8KoqAI
12	54703302	G	A	-0.02812	0.004578	8.10E-10	0.12781	462346	NA	rs1183172	NA	UKB-b-57	TRUE	reported	8KoqAI
12	56396768	G	C	-0.01756	0.0032	4.10E-08	0.338509	462346	NA	rs1689510	NA	UKB-b-57	TRUE	reported	8KoqAI
12	56403577	C	T	-0.01776	0.003192	2.60E-08	0.340841	462346	NA	rs772921	NA	UKB-b-57	TRUE	reported	8KoqAI
12	56412487	T	G	-0.01757	0.003189	3.60E-08	0.341347	462346	NA	rs1701704	NA	UKB-b-57	TRUE	reported	8KoqAI
12	56416928	A	C	-0.01758	0.003189	3.50E-08	0.341406	462346	NA	rs2456973	NA	UKB-b-57	TRUE	reported	8KoqAI

12	56427808	C	T	-0.01792	0.003194	2.00E-08	0.340676	462346	NA	rs1702877	NA	UKB-b-57	TRUE	reported	8KoqAI
12	56435412	G	A	-0.01783	0.003193	2.30E-08	0.34131	462346	NA	rs705704	NA	UKB-b-57	TRUE	reported	8KoqAI
12	56444632	C	T	-0.01827	0.003194	1.10E-08	0.341479	462346	NA	rs3441553	NA	UKB-b-57	TRUE	reported	8KoqAI
12	56445366	G	A	-0.01828	0.003194	1.00E-08	0.34147	462346	NA	rs1087686	NA	UKB-b-57	TRUE	reported	8KoqAI
12	56446766	C	T	-0.01823	0.003194	1.10E-08	0.341453	462346	NA	rs6193896	NA	UKB-b-57	TRUE	reported	8KoqAI
12	56446804	C	T	-0.01825	0.003194	1.10E-08	0.341459	462346	NA	rs6193896	NA	UKB-b-57	TRUE	reported	8KoqAI
12	56449435	G	A	-0.01842	0.003198	8.40E-09	0.339998	462346	NA	rs7302200	NA	UKB-b-57	TRUE	reported	8KoqAI
12	56455294	C	T	-0.01816	0.003196	1.30E-08	0.341247	462346	NA	rs2640564	NA	UKB-b-57	TRUE	reported	8KoqAI
12	56474379	T	C	0.01771	0.003173	2.40E-08	0.649484	462346	NA	rs3741499	NA	UKB-b-57	TRUE	reported	8KoqAI
12	56474480	A	G	0.017712	0.003173	2.40E-08	0.649533	462346	NA	rs4759229	NA	UKB-b-57	TRUE	reported	8KoqAI
12	56485269	G	A	-0.01673	0.003035	3.50E-08	0.473219	462346	NA	rs798829	NA	UKB-b-57	TRUE	reported	8KoqAI
12	56486159	G	A	-0.01677	0.003034	3.30E-08	0.473245	462346	NA	rs773116	NA	UKB-b-57	TRUE	reported	8KoqAI
12	57369910	G	A	-0.01766	0.003088	1.10E-08	0.584259	462346	NA	rs1098740	NA	UKB-b-57	TRUE	reported	8KoqAI
12	57502981	C	T	-0.01779	0.003044	5.00E-09	0.448569	462346	NA	rs324012	NA	UKB-b-57	TRUE	reported	8KoqAI
12	57503775	C	T	-0.01731	0.003089	2.10E-08	0.39733	462346	NA	rs167769	NA	UKB-b-57	TRUE	reported	8KoqAI
12	57508649	G	T	-0.01753	0.003099	1.50E-08	0.400948	462346	NA	rs3001427	NA	UKB-b-57	TRUE	reported	8KoqAI
12	57509055	T	C	-0.01767	0.003052	7.00E-09	0.45266	462346	NA	rs3001426	NA	UKB-b-57	TRUE	reported	8KoqAI
12	57509102	C	T	-0.01752	0.003099	1.60E-08	0.401039	462346	NA	rs3122929	NA	UKB-b-57	TRUE	reported	8KoqAI
12	57509569	C	T	-0.01743	0.0031	1.90E-08	0.401512	462346	NA	rs3001425	NA	UKB-b-57	TRUE	reported	8KoqAI
12	57510309	T	C	-0.01748	0.003051	1.00E-08	0.452556	462346	NA	rs324014	NA	UKB-b-57	TRUE	reported	8KoqAI
12	57510511	C	G	-0.01776	0.003043	5.30E-09	0.520319	462346	NA	rs2122692	NA	UKB-b-57	TRUE	reported	8KoqAI
12	57510661	C	T	-0.01769	0.003043	6.10E-09	0.520354	462346	NA	rs324013	NA	UKB-b-57	TRUE	reported	8KoqAI
12	57511734	T	C	-0.01768	0.00305	6.80E-09	0.451772	462346	NA	rs1231269	NA	UKB-b-57	TRUE	reported	8KoqAI
12	57512875	C	G	-0.01761	0.00305	7.70E-09	0.451698	462346	NA	rs1117210	NA	UKB-b-57	TRUE	reported	8KoqAI
12	57525756	G	A	-0.01674	0.003061	4.60E-08	0.42332	462346	NA	rs4759275	NA	UKB-b-57	TRUE	reported	8KoqAI
12	57527283	T	C	-0.01678	0.003075	4.80E-08	0.412	462346	NA	rs1117211	NA	UKB-b-57	TRUE	reported	8KoqAI
12	1.24E+08	G	C	0.019436	0.003516	3.20E-08	0.753767	462346	NA	rs2851447	NA	UKB-b-57	TRUE	reported	8KoqAI
12	1.24E+08	C	T	0.019397	0.003516	3.50E-08	0.753762	462346	NA	rs1790135	NA	UKB-b-57	TRUE	reported	8KoqAI
12	1.24E+08	G	A	0.019386	0.003516	3.50E-08	0.753757	462346	NA	rs2102949	NA	UKB-b-57	TRUE	reported	8KoqAI
12	1.24E+08	A	G	0.019376	0.003516	3.60E-08	0.753759	462346	NA	rs4460848	NA	UKB-b-57	TRUE	reported	8KoqAI
12	1.24E+08	G	A	0.019399	0.003516	3.40E-08	0.753761	462346	NA	rs1716180	NA	UKB-b-57	TRUE	reported	8KoqAI
12	1.24E+08	T	G	0.019386	0.003516	3.50E-08	0.753781	462346	NA	rs1105718	NA	UKB-b-57	TRUE	reported	8KoqAI
12	1.24E+08	C	T	0.019284	0.003515	4.10E-08	0.753723	462346	NA	rs2851443	NA	UKB-b-57	TRUE	reported	8KoqAI
12	1.24E+08	C	T	0.019349	0.003516	3.70E-08	0.753791	462346	NA	rs3018099	NA	UKB-b-57	TRUE	reported	8KoqAI
12	1.24E+08	T	C	0.019353	0.003514	3.60E-08	0.753347	462346	NA	rs1727319	NA	UKB-b-57	TRUE	reported	8KoqAI
12	1.24E+08	C	T	0.019786	0.003519	1.90E-08	0.753368	462346	NA	rs1727332	NA	UKB-b-57	TRUE	reported	8KoqAI
12	1.24E+08	C	G	0.019737	0.003516	2.00E-08	0.752298	462346	NA	rs1727331	NA	UKB-b-57	TRUE	reported	8KoqAI
12	1.24E+08	G	A	0.019567	0.003514	2.60E-08	0.752391	462346	NA	rs7304782	NA	UKB-b-57	TRUE	reported	8KoqAI
12	1.24E+08	C	A	0.019619	0.003514	2.40E-08	0.752365	462346	NA	rs1077299	NA	UKB-b-57	TRUE	reported	8KoqAI
12	1.24E+08	G	T	0.019558	0.003513	2.60E-08	0.752388	462346	NA	rs1105720	NA	UKB-b-57	TRUE	reported	8KoqAI
12	1.24E+08	A	C	0.01952	0.003513	2.70E-08	0.752371	462346	NA	rs1074415	NA	UKB-b-57	TRUE	reported	8KoqAI
12	1.24E+08	G	C	0.019551	0.003513	2.60E-08	0.752412	462346	NA	rs1074415	NA	UKB-b-57	TRUE	reported	8KoqAI
12	1.24E+08	C	T	-0.02065	0.003784	4.90E-08	0.200966	462346	NA	rs5880947	NA	UKB-b-57	TRUE	reported	8KoqAI
12	1.24E+08	C	T	-0.02073	0.003788	4.40E-08	0.200447	462346	NA	rs1282945	NA	UKB-b-57	TRUE	reported	8KoqAI
12	1.24E+08	G	A	0.019532	0.003512	2.70E-08	0.752413	462346	NA	rs1105720	NA	UKB-b-57	TRUE	reported	8KoqAI
12	1.24E+08	G	A	0.019456	0.003512	3.00E-08	0.752371	462346	NA	rs1077299	NA	UKB-b-57	TRUE	reported	8KoqAI
12	1.24E+08	G	A	-0.02071	0.003788	4.50E-08	0.20035	462346	NA	rs1281789	NA	UKB-b-57	TRUE	reported	8KoqAI
12	1.24E+08	G	A	-0.02063	0.003784	5.00E-08	0.200888	462346	NA	rs1281806	NA	UKB-b-57	TRUE	reported	8KoqAI
12	1.24E+08	C	T	0.019431	0.003512	3.20E-08	0.752416	462346	NA	rs7137286	NA	UKB-b-57	TRUE	reported	8KoqAI
12	1.24E+08	G	A	-0.02071	0.003784	4.40E-08	0.200822	462346	NA	rs3434146	NA	UKB-b-57	TRUE	reported	8KoqAI
12	1.24E+08	T	G	0.01946	0.003511	3.00E-08	0.752365	462346	NA	rs1105720	NA	UKB-b-57	TRUE	reported	8KoqAI
12	1.24E+08	A	G	0.01938	0.003511	3.40E-08	0.752234	462346	NA	rs1153232	NA	UKB-b-57	TRUE	reported	8KoqAI
12	1.24E+08	A	G	0.019347	0.00351	3.50E-08	0.752286	462346	NA	rs1105720	NA	UKB-b-57	TRUE	reported	8KoqAI
12	1.24E+08	G	A	0.019511	0.003511	2.70E-08	0.752283	462346	NA	rs1609520	NA	UKB-b-57	TRUE	reported	8KoqAI
12	1.24E+08	A	G	0.019365	0.003509	3.40E-08	0.752291	462346	NA	rs1568427	NA	UKB-b-57	TRUE	reported	8KoqAI
12	1.24E+08	G	A	-0.02081	0.003785	3.80E-08	0.200527	462346	NA	rs1879380	NA	UKB-b-57	TRUE	reported	8KoqAI
12	1.24E+08	G	A	0.019427	0.003508	3.10E-08	0.752248	462346	NA	rs1969355	NA	UKB-b-57	TRUE	reported	8KoqAI
12	1.24E+08	G	A	0.01959	0.003516	2.50E-08	0.752874	462346	NA	rs1073490	NA	UKB-b-57	TRUE	reported	8KoqAI
12	1.24E+08	G	A	0.019586	0.003516	2.50E-08	0.752873	462346	NA	rs1073490	NA	UKB-b-57	TRUE	reported	8KoqAI
12	1.24E+08	G	C	0.019356	0.003508	3.40E-08	0.752114	462346	NA	rs1073257	NA	UKB-b-57	TRUE	reported	8KoqAI
12	1.24E+08	G	A	0.01935	0.003508	3.50E-08	0.752002	462346	NA	rs1980251	NA	UKB-b-57	TRUE	reported	8KoqAI
12	1.24E+08	G	T	-0.02065	0.003784	4.80E-08	0.200673	462346	NA	rs6633	NA	UKB-b-57	TRUE	reported	8KoqAI
12	1.24E+08	A	T	0.019379	0.003509	3.30E-08	0.752129	462346	NA	rs1077300	NA	UKB-b-57	TRUE	reported	8KoqAI
12	1.24E+08	T	C	-0.02073	0.003784	4.30E-08	0.20067	462346	NA	rs1282495	NA	UKB-b-57	TRUE	reported	8KoqAI
12	1.24E+08	C	G	-0.02091	0.003779	3.10E-08	0.201159	462346	NA	rs7953929	NA	UKB-b-57	TRUE	reported	8KoqAI
12	1.24E+08	G	A	-0.02085	0.003765	3.00E-08	0.203605	462346	NA	rs7306755	NA	UKB-b-57	TRUE	reported	8KoqAI
12	1.24E+08	G	A	-0.02083	0.003769	3.30E-08	0.203346	462346	NA	rs7144457	NA	UKB-b-57	TRUE	reported	8KoqAI
12	1.24E+08	A	G	0.020824	0.00376	3.00E-08	0.79544	462346	NA	rs9739070	NA	UKB-b-57	TRUE	reported	8KoqAI
12	1.24E+08	C	T	-0.02082	0.003765	3.20E-08	0.203655	462346	NA	rs1282143	NA	UKB-b-57	TRUE	reported	8KoqAI
12	1.24E+08	C	T	-0.02063	0.003762	4.10E-08	0.203635	462346	NA	rs1060105	NA	UKB-b-57	TRUE	reported	8KoqAI
12	1.24E+08	G	A	-0.02057	0.003763	4.60E-08	0.204166	462346	NA	rs2858383	NA	UKB-b-57	TRUE	reported	8KoqAI
12	1.24E+08	A	G	-0.0207	0.003761	3.70E-08	0.204925	462346	NA	rs2837257	NA	UKB-b-57	TRUE	reported	8KoqAI
12	1.24E+08	T	C	0.0207	0.003552	5.60E-09	0.759525	462346	NA	rs2876812	NA	UKB-b-57	TRUE	reported	8KoqAI
12	1.24E+08	T	C	0.021254	0.00384	3.10E-08	0.805984	462346	NA	rs2841089	NA	UKB-b-57	TRUE	reported	8KoqAI
12	1.24E+08	G	A	0.021349	0.00384	2.70E-08	0.806279	462346	NA	rs2848186	NA	UKB-b-57	TRUE	reported	8KoqAI
12	1.24E+08	C	T	-0.02159	0.00385	2.00E-08	0.192477	462346	NA	rs2871549	NA	UKB-b-57	TRUE	reported	8KoqAI
12	1.24E+08	C	T	-0.02172	0.003841	1.60E-08	0.193231	462346	NA	rs2867649	NA	UKB-b-57	TRUE	reported	8KoqAI
12	1.24E+08	G	A	-0.02165	0.003837	1.70E-08	0.193626	462346	NA	rs2862501	NA	UKB-b-57	TRUE	reported	8KoqAI
12	1.24E+08	C	T	-0.02189	0.00384	1.20E-08	0.193428	462346	NA	rs2870669	NA	UKB-b-57	TRUE	reported	8KoqAI
12	1.24E+08	G	A	-0.02144	0.003904	4.00E-08	0.186178	462346	NA	rs4930719	NA	UKB-b-57	TRUE	reported	8KoqAI
13	49888131	C	A	0.016799	0.00305	3.60E-08	0.468759	462346	NA	rs1161980	NA	UKB-b-57	TRUE	reported	8KoqAI
13	49915391	T	C	0.020657	0.003481	3.00E-09	0.257922	462346	NA	rs6561520	NA	UKB-b-57	TRUE	reported	8KoqAI
13	49925365	A	C	0.020798	0.003425	1.30E-09	0.271646	462346	NA	rs7991335	NA	UKB-b-57	TRUE	reported	8KoqAI
13	49927732	T	C	0.020561	0.003424	1.90E-09	0.271231	462346	NA	rs9568188	NA	UKB-b-57	TRUE	reported	8KoqAI
13	49936877	A	G	0.020297	0.003421	3.00E-09	0.271463	462346	NA	rs7326978	NA	UKB-b-57	TRUE	reported	8KoqAI
13	49939903	C	T	0.020701	0.003477	2.60E-09	0.257272	462346	NA	rs9535211	NA	UKB-b-57	TRUE	reported	8KoqAI
13	49970938	G	A	-0.02122	0.003404	4.60E-10	0.7204	462346	NA	rs6420299	NA	UKB-b-57	TRUE	reported	8KoqAI
13	49971400	C	T	-0.02133	0.003405	3.70E-10	0.720352	462346	NA	rs7330939	NA	UKB-b-57	TRUE	reported	8KoqAI
13	49974650	C	T	-0.02098	0.003409	7.50E-10	0.72157	462346	NA	rs9596096	NA	UKB-b-57	TRUE	reported	8KoqAI
13	49979661	A	G	-0.02065	0.003409	1.40E-09	0.72204	462346	NA	rs7993917	NA	UKB-b-57	TRUE	reported	8KoqAI
13	49981064	G	T	-0.02077	0.003406	1.10E-09	0.721852	462346	NA	rs7338471	NA	UKB-b-57	TRUE	reported	8KoqAI
13	49982096	C	T	0.021744	0.003563	1.00E-09	0.242342	462346	NA	rs1707286	NA	UKB-b-57	TRUE	reported	8KoqAI
13	49983055	C	A	-0.02078	0.003409	1.10E-09	0.721894	462346	NA	rs9568196	NA	UKB-b-57	TRUE	reported	8KoqAI
13	49985272	T	C	-0.02076	0.003408	1.10E-09	0.721325	462346	NA	rs4942828	NA	UKB-b-57	TRUE	reported	8KoqAI
13	49986118	A	G	-0.02103	0.00338	4.80E-10	0.713022	462346	NA	rs2181328	NA	UKB-b-57	TRUE	reported	8KoqAI
13	49986602	A	G	-0.02088	0.003381	6.60E-10	0.713414	462346	NA	rs1218458	NA	UKB-b-57	TRUE	reported	8KoqAI
13	68023159	C	T	0.016607	0.003032	4.30E-08	0.498613	462346	NA	rs4619273	NA	UKB-b-57	TRUE	reported	8KoqAI
13	68026719	G	C	0.016641	0.003031	4.00E-08	0.498641	462346	NA	rs7336878	NA	UKB-b-57	TRUE	reported	8KoqAI
13	68027308	A	G	0.016658	0.003031	3.90E-08	0.498668	462346	NA	rs9564398	NA	UKB-b-57	TRUE	reported	8KoqAI
13	68027699	G	A	0.016651	0.003031	3.90E-08	0.498668	462346	NA	rs9564399	NA	UKB-b-57	TRUE	reported	8KoqAI
13	68029895	G	T	0.01664	0.00303	4.00E-08	0.498678	462346	NA	rs9599228	NA	UKB-b-57	TRUE	reported	8KoqAI
13	68033258	T	G	0.016668	0.00303	3.80E-08	0.49882	462346	NA	rs7331794	NA	UKB-b-57	TRUE	reported	8KoqAI
13	68037909	A	C	0.016728	0.003029	3.30E-08	0.497849	462346	NA	rs4451847	NA	UKB-b-57	TRUE	reported	8KoqAI
13	68039150	T	C	0.016723	0.003029	3.40E-08	0.497885	462346	NA	rs7336348	NA	UKB-b-57	TRUE	reported	8KoqAI
13	68039549	C	T	0.016741	0.003029	3.30E-08	0.497863	462346	NA	rs7317130	NA	UKB-b-57	TRUE	reported	8KoqAI
13	68053176	C	A	0.016602	0.003029	4.20E-08	0.498335	462346	NA	rs1258442	NA	UKB-b-57	TRUE	reported	8KoqAI
13	68055556	T	A	0.01662	0.003029	4.10E-08	0.498133	462346	NA	rs6196006	NA	UKB-b-57	TRUE	reported	8KoqAI

13	68061403	G	T	0.016529	0.003028	4.80E-08	0.498271	462346	NA	rs9571790	NA	UKB-b-57	TRUE	reported	8KoqAI
13	68080817	A	G	0.016898	0.003032	2.50E-08	0.528054	462346	NA	rs1937522	NA	UKB-b-57	TRUE	reported	8KoqAI
13	68084026	T	C	0.016661	0.003031	3.80E-08	0.527739	462346	NA	rs6562493	NA	UKB-b-57	TRUE	reported	8KoqAI
13	68084237	C	T	0.016695	0.00303	3.60E-08	0.52774	462346	NA	rs6562494	NA	UKB-b-57	TRUE	reported	8KoqAI
13	68085344	A	T	0.016774	0.00303	3.10E-08	0.527988	462346	NA	rs9571794	NA	UKB-b-57	TRUE	reported	8KoqAI
13	68085885	A	C	0.01673	0.00303	3.40E-08	0.52785	462346	NA	rs1937519	NA	UKB-b-57	TRUE	reported	8KoqAI
13	68086971	T	C	0.016723	0.00303	3.40E-08	0.527849	462346	NA	rs8000979	NA	UKB-b-57	TRUE	reported	8KoqAI
13	68087479	T	A	0.01671	0.00303	3.50E-08	0.52778	462346	NA	rs1937518	NA	UKB-b-57	TRUE	reported	8KoqAI
13	68089045	T	C	0.01672	0.00303	3.40E-08	0.527785	462346	NA	rs1286981	NA	UKB-b-57	TRUE	reported	8KoqAI
13	68122525	A	T	0.016605	0.003038	4.60E-08	0.527314	462346	NA	rs4559806	NA	UKB-b-57	TRUE	reported	8KoqAI
14	59063842	G	A	-0.01857	0.003328	2.40E-08	0.300812	462346	NA	rs7155669	NA	UKB-b-57	TRUE	reported	8KoqAI
14	59068284	T	G	0.017093	0.003083	3.00E-08	0.422572	462346	NA	rs221325	NA	UKB-b-57	TRUE	reported	8KoqAI
14	59068964	T	C	0.017173	0.003084	2.60E-08	0.422312	462346	NA	rs468167	NA	UKB-b-57	TRUE	reported	8KoqAI
14	59069053	G	T	0.017191	0.003055	1.80E-08	0.459419	462346	NA	rs221326	NA	UKB-b-57	TRUE	reported	8KoqAI
14	59072144	T	C	0.017918	0.003077	5.80E-09	0.426418	462346	NA	rs1078243	NA	UKB-b-57	TRUE	reported	8KoqAI
14	59072226	G	T	0.017949	0.003051	4.00E-09	0.463343	462346	NA	rs186347	NA	UKB-b-57	TRUE	reported	8KoqAI
14	59074136	A	C	0.017542	0.003037	7.70E-09	0.463562	462346	NA	rs160459	NA	UKB-b-57	TRUE	reported	8KoqAI
14	73480209	C	G	0.018417	0.003311	2.70E-08	0.299074	462346	NA	rs2333020	NA	UKB-b-57	TRUE	reported	8KoqAI
14	73520136	C	A	-0.01873	0.003169	3.40E-09	0.354702	462346	NA	rs1130041	NA	UKB-b-57	TRUE	reported	8KoqAI
14	73523162	C	T	-0.01885	0.003168	2.70E-09	0.354749	462346	NA	rs2535911	NA	UKB-b-57	TRUE	reported	8KoqAI
14	73525265	A	T	-0.01856	0.003175	5.00E-09	0.352191	462346	NA	rs2535923	NA	UKB-b-57	TRUE	reported	8KoqAI
14	73526613	G	A	-0.01852	0.003169	5.10E-09	0.353457	462346	NA	rs2806049	NA	UKB-b-57	TRUE	reported	8KoqAI
14	73529094	A	G	-0.01836	0.003181	7.80E-09	0.380623	462346	NA	rs2806048	NA	UKB-b-57	TRUE	reported	8KoqAI
14	73532676	A	G	-0.01847	0.003169	5.60E-09	0.353469	462346	NA	rs2806047	NA	UKB-b-57	TRUE	reported	8KoqAI
14	73577012	G	C	0.018592	0.003169	4.40E-09	0.646353	462346	NA	rs214290	NA	UKB-b-57	TRUE	reported	8KoqAI
14	73581736	G	A	0.018641	0.003168	4.00E-09	0.646186	462346	NA	rs165937	NA	UKB-b-57	TRUE	reported	8KoqAI
14	73586076	T	C	0.018469	0.003173	5.90E-09	0.647373	462346	NA	rs214287	NA	UKB-b-57	TRUE	reported	8KoqAI
14	73604578	G	C	0.01814	0.003174	1.10E-08	0.647652	462346	NA	rs214279	NA	UKB-b-57	TRUE	reported	8KoqAI
14	73615610	C	G	0.018206	0.003173	9.60E-09	0.647422	462346	NA	rs214274	NA	UKB-b-57	TRUE	reported	8KoqAI
14	73616124	A	G	0.018241	0.003173	9.00E-09	0.647425	462346	NA	rs214273	NA	UKB-b-57	TRUE	reported	8KoqAI
14	73632670	G	A	0.018024	0.00317	1.30E-08	0.645981	462346	NA	rs214271	NA	UKB-b-57	TRUE	reported	8KoqAI
14	73640638	A	G	0.018149	0.003171	1.00E-08	0.646517	462346	NA	rs214269	NA	UKB-b-57	TRUE	reported	8KoqAI
14	73662165	A	G	0.017899	0.003177	1.80E-08	0.646933	462346	NA	rs214262	NA	UKB-b-57	TRUE	reported	8KoqAI
14	73662469	T	C	0.017912	0.003177	1.70E-08	0.646782	462346	NA	rs214261	NA	UKB-b-57	TRUE	reported	8KoqAI
14	73683194	T	C	0.018204	0.003182	1.10E-08	0.648001	462346	NA	rs177413	NA	UKB-b-57	TRUE	reported	8KoqAI
14	73882705	C	T	0.016722	0.00304	3.80E-08	0.452745	462346	NA	rs2215061	NA	UKB-b-57	TRUE	reported	8KoqAI
14	73884540	A	G	0.016594	0.003029	4.30E-08	0.48275	462346	NA	rs1014235	NA	UKB-b-57	TRUE	reported	8KoqAI
15	34644985	C	T	0.016678	0.003057	4.90E-08	0.541632	462346	NA	rs7272251	NA	UKB-b-57	TRUE	reported	8KoqAI
15	34659517	G	C	-0.01967	0.00332	3.10E-09	0.336989	462346	NA	rs1177994	NA	UKB-b-57	TRUE	reported	8KoqAI
15	76467799	C	T	-0.03769	0.006626	1.30E-08	0.056084	462346	NA	rs7703115	NA	UKB-b-57	TRUE	reported	8KoqAI
15	76476361	G	A	-0.02845	0.005221	5.00E-08	0.093046	462346	NA	rs1745657	NA	UKB-b-57	TRUE	reported	8KoqAI
15	76508632	T	C	-0.03937	0.006496	1.40E-09	0.057704	462346	NA	rs8029231	NA	UKB-b-57	TRUE	reported	8KoqAI
15	76667396	C	T	-0.03377	0.006189	4.80E-08	0.064016	462346	NA	rs1131345	NA	UKB-b-57	TRUE	reported	8KoqAI
15	76773830	C	T	-0.03901	0.00658	3.10E-09	0.056713	462346	NA	rs1135527	NA	UKB-b-57	TRUE	reported	8KoqAI
15	76900257	G	A	-0.04018	0.006882	5.30E-09	0.053362	462346	NA	rs1429888	NA	UKB-b-57	TRUE	reported	8KoqAI
15	76908519	C	A	-0.03892	0.006548	2.80E-09	0.056843	462346	NA	rs7629320	NA	UKB-b-57	TRUE	reported	8KoqAI
15	76920197	G	A	-0.04018	0.006879	5.20E-09	0.053351	462346	NA	rs1115192	NA	UKB-b-57	TRUE	reported	8KoqAI
15	77164686	T	C	-0.04111	0.00681	1.60E-09	0.054665	462346	NA	rs1181886	NA	UKB-b-57	TRUE	reported	8KoqAI
15	77164688	T	A	-0.04111	0.006809	1.60E-09	0.054676	462346	NA	rs1171740	NA	UKB-b-57	TRUE	reported	8KoqAI
16	6172126	C	T	-0.01691	0.003048	2.90E-08	0.446061	462346	NA	rs3463102	NA	UKB-b-57	TRUE	reported	8KoqAI
16	6174251	A	C	-0.01663	0.003045	4.70E-08	0.447466	462346	NA	rs1386089	NA	UKB-b-57	TRUE	reported	8KoqAI
16	6178072	C	G	-0.01663	0.003051	5.00E-08	0.448423	462346	NA	rs5607360	NA	UKB-b-57	TRUE	reported	8KoqAI
16	13746582	C	T	0.018795	0.003432	4.30E-08	0.734048	462346	NA	rs1164090	NA	UKB-b-57	TRUE	reported	8KoqAI
16	13747803	C	G	0.021091	0.003626	6.00E-09	0.774542	462346	NA	rs1076884	NA	UKB-b-57	TRUE	reported	8KoqAI
16	13748916	G	C	0.020948	0.003622	7.30E-09	0.773353	462346	NA	rs7499362	NA	UKB-b-57	TRUE	reported	8KoqAI
16	13749265	A	C	0.021022	0.003626	6.70E-09	0.774629	462346	NA	rs7499750	NA	UKB-b-57	TRUE	reported	8KoqAI
16	13749859	T	C	0.020969	0.003626	7.30E-09	0.774599	462346	NA	rs7405404	NA	UKB-b-57	TRUE	reported	8KoqAI
16	13750219	T	C	0.020832	0.003626	9.20E-09	0.774676	462346	NA	rs7403809	NA	UKB-b-57	TRUE	reported	8KoqAI
16	13750257	T	G	0.018719	0.003434	5.00E-08	0.735454	462346	NA	rs7403810	NA	UKB-b-57	TRUE	reported	8KoqAI
16	13750694	T	C	0.02051	0.003617	1.40E-08	0.772869	462346	NA	rs7185124	NA	UKB-b-57	TRUE	reported	8KoqAI
16	13750724	C	G	0.018734	0.003434	4.90E-08	0.735464	462346	NA	rs7202384	NA	UKB-b-57	TRUE	reported	8KoqAI
16	13751825	A	G	-0.02106	0.003625	6.20E-09	0.225388	462346	NA	rs1186488	NA	UKB-b-57	TRUE	reported	8KoqAI
16	13752356	C	T	-0.02068	0.003626	1.20E-08	0.225334	462346	NA	rs1292587	NA	UKB-b-57	TRUE	reported	8KoqAI
16	13753384	G	A	-0.02099	0.003624	6.90E-09	0.225321	462346	NA	rs8055219	NA	UKB-b-57	TRUE	reported	8KoqAI
16	13760217	G	A	-0.01875	0.003434	4.70E-08	0.26535	462346	NA	rs4456522	NA	UKB-b-57	TRUE	reported	8KoqAI
16	19982353	G	C	0.023365	0.003471	1.70E-11	0.737192	462346	NA	rs8043563	NA	UKB-b-57	TRUE	reported	8KoqAI
16	19982528	A	C	0.022872	0.003527	8.90E-11	0.750642	462346	NA	rs8064142	NA	UKB-b-57	TRUE	reported	8KoqAI
16	19982685	C	T	-0.02746	0.003699	1.10E-13	0.216495	462346	NA	rs1107442	NA	UKB-b-57	TRUE	reported	8KoqAI
16	19983753	G	A	-0.02566	0.003652	2.10E-12	0.223122	462346	NA	rs1858986	NA	UKB-b-57	TRUE	reported	8KoqAI
16	19983820	A	G	-0.02538	0.00365	3.60E-12	0.223564	462346	NA	rs7972570	NA	UKB-b-57	TRUE	reported	8KoqAI
16	19984427	G	T	-0.02566	0.003649	2.00E-12	0.223181	462346	NA	rs4780833	NA	UKB-b-57	TRUE	reported	8KoqAI
16	19984726	G	C	-0.02568	0.003648	1.90E-12	0.223161	462346	NA	rs4780834	NA	UKB-b-57	TRUE	reported	8KoqAI
16	19984847	G	A	-0.02566	0.003647	2.00E-12	0.22316	462346	NA	rs9938146	NA	UKB-b-57	TRUE	reported	8KoqAI
16	19985123	C	A	-0.02572	0.003647	1.80E-12	0.223165	462346	NA	rs7277106	NA	UKB-b-57	TRUE	reported	8KoqAI
16	19985393	A	C	-0.02356	0.003592	5.50E-11	0.233005	462346	NA	rs4780836	NA	UKB-b-57	TRUE	reported	8KoqAI
16	19985516	A	G	-0.02544	0.003643	2.90E-12	0.223489	462346	NA	rs4780271	NA	UKB-b-57	TRUE	reported	8KoqAI
16	19986891	A	G	-0.02563	0.003635	1.80E-12	0.223606	462346	NA	rs9923407	NA	UKB-b-57	TRUE	reported	8KoqAI
16	19987636	A	G	-0.02657	0.003678	5.00E-13	0.217075	462346	NA	rs2865110	NA	UKB-b-57	TRUE	reported	8KoqAI
16	19988661	T	C	-0.02585	0.003642	1.30E-12	0.223248	462346	NA	rs5667777	NA	UKB-b-57	TRUE	reported	8KoqAI
16	19988852	A	G	-0.02568	0.003641	1.70E-12	0.223644	462346	NA	rs9940317	NA	UKB-b-57	TRUE	reported	8KoqAI
16	19989840	C	T	-0.02673	0.003681	3.80E-13	0.217741	462346	NA	rs5685936	NA	UKB-b-57	TRUE	reported	8KoqAI
16	19990080	A	G	-0.02578	0.003642	1.40E-12	0.224131	462346	NA	rs7277106	NA	UKB-b-57	TRUE	reported	8KoqAI
16	19990219	A	G	-0.02595	0.003643	1.10E-12	0.223932	462346	NA	rs7277106	NA	UKB-b-57	TRUE	reported	8KoqAI
16	19990613	A	G	-0.02585	0.003643	1.30E-12	0.22408	462346	NA	rs7277106	NA	UKB-b-57	TRUE	reported	8KoqAI
16	19992121	G	A	-0.02676	0.003679	3.50E-13	0.219038	462346	NA	rs4365294	NA	UKB-b-57	TRUE	reported	8KoqAI
16	19992184	G	A	-0.02679	0.003679	3.20E-13	0.219067	462346	NA	rs2883661	NA	UKB-b-57	TRUE	reported	8KoqAI
16	19992816	A	T	-0.02916	0.003758	8.60E-15	0.207277	462346	NA	rs7277106	NA	UKB-b-57	TRUE	reported	8KoqAI
16	19992996	G	A	0.023196	0.003383	7.10E-12	0.71605	462346	NA	rs1011939	NA	UKB-b-57	TRUE	reported	8KoqAI
16	19993015	T	C	-0.02481	0.003543	2.50E-12	0.245753	462346	NA	rs2841560	NA	UKB-b-57	TRUE	reported	8KoqAI
16	19993750	C	T	-0.02659	0.003386	4.10E-15	0.282915	462346	NA	rs7277107	NA	UKB-b-57	TRUE	reported	8KoqAI
16	19994743	C	G	-0.02312	0.003293	2.20E-12	0.310338	462346	NA	rs2355371	NA	UKB-b-57	TRUE	reported	8KoqAI
16	19998311	C	G	-0.02339	0.003282	1.00E-12	0.312139	462346	NA	rs2846156	NA	UKB-b-57	TRUE	reported	8KoqAI
16	19998585	G	C	-0.02327	0.003286	1.40E-12	0.310571	462346	NA	rs7457528	NA	UKB-b-57	TRUE	reported	8KoqAI
16	19998923	G	A	-0.02336	0.003281	1.10E-12	0.311982	462346	NA	rs7277107	NA	UKB-b-57	TRUE	reported	8KoqAI
16	19999561	A	T	-0.0231	0.003217	7.00E-13	0.335858	462346	NA	rs1107442	NA	UKB-b-57	TRUE	reported	8KoqAI
16	20002345	A	C	-0.02173	0.003361	1.00E-10	0.284974	462346	NA	rs5630863	NA	UKB-b-57	TRUE	reported	8KoqAI
16	20002523	A	C	-0.02678	0.00351	2.40E-14	0.248549	462346	NA	rs7277107	NA	UKB-b-57	TRUE	reported	8KoqAI
16	20006745	C	G	-0.02608	0.003472	5.80E-14	0.25652	462346	NA	rs6750135	NA	UKB-b-57	TRUE	reported	8KoqAI
16	20006986	A	G	-0.02533	0.003457	2.30E-13	0.260374	462346	NA	rs5779005	NA	UKB-b-57	TRUE	reported	8KoqAI
16	20008346	C	T	-0.02609	0.00368	1.40E-12	0.216784	462346	NA	rs8027890	NA	UKB-b-57	TRUE	reported	8KoqAI
16	20008417	G	A	-0.02568	0.003464	1.20E-13	0.258405	462346	NA	rs7203942	NA	UKB-b-57	TRUE	reported	8KoqAI
16	20009247	T	C	-0.02519	0.003296	2.10E-14	0.303691	462346	NA	rs1858988	NA	UKB-b-57	TRUE	reported	8KoqAI
16	20011100	A	G	-0.02564	0.003673	2.90E-12	0.217565	462346	NA	rs1338064	NA	UKB-b-57	TRUE	reported	8KoqAI
16	20013793	G	A	-0.02559	0.003306	9.80E-15	0.298863	462346	NA	rs2764771	NA	UKB-b-57	TRUE	reported	8KoqAI
16	20016510	C	A	-0.02499	0.003667	9.40E-12	0.217868	462346	NA	rs7277107	NA	UKB-b-57	TRUE	reported	8KoqAI
16	20016650	C	T	-0.02544	0.003672	4.30E-12	0.216799	462346	NA	rs7277107	NA	UKB-b-57	TRUE	reported	8KoqAI
16	20017233	C	G	-0.02553	0.003464	1.70E-13	0.256333	462346	NA	rs2097694	NA	UKB-b-57	TRUE	reported	8KoqAI

16	20017935	T	C	-0.02556	0.003464	1.60E-13	0.256393	462346	NA	rs2521480	NA	UKB-b-57	TRUE	reported	8KoqAI
16	20018751	C	T	-0.02576	0.003459	9.60E-14	0.257349	462346	NA	rs2764791	NA	UKB-b-57	TRUE	reported	8KoqAI
16	20019342	G	C	-0.02574	0.003459	9.90E-14	0.257368	462346	NA	rs2521482	NA	UKB-b-57	TRUE	reported	8KoqAI
16	20019541	A	G	-0.02574	0.003459	9.90E-14	0.257371	462346	NA	rs1925485	NA	UKB-b-57	TRUE	reported	8KoqAI
16	20019804	T	C	-0.02574	0.003459	9.90E-14	0.257371	462346	NA	rs1925486	NA	UKB-b-57	TRUE	reported	8KoqAI
16	20020013	T	G	-0.02558	0.003464	1.50E-13	0.256407	462346	NA	rs2521483	NA	UKB-b-57	TRUE	reported	8KoqAI
16	20020901	T	C	-0.02574	0.003459	1.00E-13	0.257381	462346	NA	rs2764735	NA	UKB-b-57	TRUE	reported	8KoqAI
16	20021383	C	T	-0.02663	0.003706	6.70E-13	0.212071	462346	NA	rs7277108	NA	UKB-b-57	TRUE	reported	8KoqAI
16	20021535	A	G	-0.02577	0.003463	1.00E-13	0.25652	462346	NA	rs1925487	NA	UKB-b-57	TRUE	reported	8KoqAI
16	20021884	A	G	-0.02574	0.003459	9.90E-14	0.257375	462346	NA	rs1925488	NA	UKB-b-57	TRUE	reported	8KoqAI
16	20022062	C	T	-0.02568	0.003665	2.50E-12	0.217799	462346	NA	rs4780272	NA	UKB-b-57	TRUE	reported	8KoqAI
16	20022107	A	G	-0.02575	0.003459	9.70E-14	0.257368	462346	NA	rs2521485	NA	UKB-b-57	TRUE	reported	8KoqAI
16	20022576	T	C	-0.02568	0.003665	2.50E-12	0.217799	462346	NA	rs7277108	NA	UKB-b-57	TRUE	reported	8KoqAI
16	20023601	A	G	-0.02567	0.003665	2.50E-12	0.217804	462346	NA	rs7277108	NA	UKB-b-57	TRUE	reported	8KoqAI
16	20023954	A	G	-0.02578	0.003459	9.10E-14	0.257344	462346	NA	rs2521486	NA	UKB-b-57	TRUE	reported	8KoqAI
16	20023974	C	T	-0.02552	0.003298	1.00E-14	0.300772	462346	NA	rs2764740	NA	UKB-b-57	TRUE	reported	8KoqAI
16	20025347	G	C	-0.02562	0.003464	1.40E-13	0.256381	462346	NA	rs1925493	NA	UKB-b-57	TRUE	reported	8KoqAI
16	20027213	G	A	-0.02559	0.003463	1.50E-13	0.256448	462346	NA	rs1902812	NA	UKB-b-57	TRUE	reported	8KoqAI
16	20027295	A	G	-0.02554	0.003672	3.50E-12	0.216873	462346	NA	rs1696941	NA	UKB-b-57	TRUE	reported	8KoqAI
16	20028013	T	C	-0.02303	0.00326	1.60E-12	0.318213	462346	NA	rs2521477	NA	UKB-b-57	TRUE	reported	8KoqAI
16	20028998	T	C	-0.02585	0.003468	9.10E-14	0.257988	462346	NA	rs2521478	NA	UKB-b-57	TRUE	reported	8KoqAI
16	20029359	C	A	-0.02536	0.003301	1.60E-14	0.302781	462346	NA	rs2477625	NA	UKB-b-57	TRUE	reported	8KoqAI
16	20030598	G	T	-0.02586	0.003468	8.90E-14	0.257984	462346	NA	rs1379576	NA	UKB-b-57	TRUE	reported	8KoqAI
16	20030832	C	T	-0.0252	0.003304	2.40E-14	0.301836	462346	NA	rs2764742	NA	UKB-b-57	TRUE	reported	8KoqAI
16	20031016	A	G	-0.02573	0.00367	2.40E-12	0.219133	462346	NA	rs7277108	NA	UKB-b-57	TRUE	reported	8KoqAI
16	20032436	T	C	-0.02529	0.003307	2.00E-14	0.301227	462346	NA	rs7191132	NA	UKB-b-57	TRUE	reported	8KoqAI
16	20034247	C	T	-0.02597	0.003472	7.40E-14	0.257362	462346	NA	rs1379577	NA	UKB-b-57	TRUE	reported	8KoqAI
16	20034418	G	A	-0.02597	0.003472	7.40E-14	0.257361	462346	NA	rs1379578	NA	UKB-b-57	TRUE	reported	8KoqAI
16	20034996	T	C	-0.02589	0.003675	1.90E-12	0.218515	462346	NA	rs1350380	NA	UKB-b-57	TRUE	reported	8KoqAI
16	20036619	G	A	-0.02602	0.003473	6.70E-14	0.257255	462346	NA	rs2764744	NA	UKB-b-57	TRUE	reported	8KoqAI
16	20037123	G	A	-0.02583	0.003676	2.10E-12	0.218493	462346	NA	rs5770553	NA	UKB-b-57	TRUE	reported	8KoqAI
16	20037731	G	T	-0.02532	0.003307	1.90E-14	0.301228	462346	NA	rs2764745	NA	UKB-b-57	TRUE	reported	8KoqAI
16	20037965	C	T	0.025419	0.003305	1.50E-14	0.698129	462346	NA	rs4780273	NA	UKB-b-57	TRUE	reported	8KoqAI
16	20038412	G	T	-0.02594	0.003472	8.00E-14	0.257343	462346	NA	rs2182155	NA	UKB-b-57	TRUE	reported	8KoqAI
16	20039229	C	G	-0.02604	0.003473	6.40E-14	0.257243	462346	NA	rs1902811	NA	UKB-b-57	TRUE	reported	8KoqAI
16	20040042	C	T	-0.02595	0.003472	7.80E-14	0.25737	462346	NA	rs989426	NA	UKB-b-57	TRUE	reported	8KoqAI
16	20040458	A	G	-0.02531	0.003307	2.00E-14	0.30125	462346	NA	rs2125148	NA	UKB-b-57	TRUE	reported	8KoqAI
16	20041133	A	G	-0.02584	0.003675	2.10E-12	0.218604	462346	NA	rs1031634	NA	UKB-b-57	TRUE	reported	8KoqAI
16	20041289	C	T	0.025393	0.003305	1.60E-14	0.698102	462346	NA	rs2279022	NA	UKB-b-57	TRUE	reported	8KoqAI
16	20043330	T	C	-0.0254	0.003307	1.60E-14	0.301548	462346	NA	rs2147865	NA	UKB-b-57	TRUE	reported	8KoqAI
16	20045026	A	T	0.024937	0.003303	4.30E-14	0.696548	462346	NA	rs2045458	NA	UKB-b-57	TRUE	reported	8KoqAI
16	20045399	G	A	-0.02554	0.003668	3.30E-12	0.220414	462346	NA	rs7277109	NA	UKB-b-57	TRUE	reported	8KoqAI
16	20045668	A	G	-0.02513	0.003666	7.10E-12	0.220759	462346	NA	rs727590	NA	UKB-b-57	TRUE	reported	8KoqAI
16	20046082	C	A	-0.02546	0.003668	3.90E-12	0.220328	462346	NA	rs7655479	NA	UKB-b-57	TRUE	reported	8KoqAI
16	20046115	G	A	0.025114	0.003302	2.80E-14	0.696384	462346	NA	rs2045457	NA	UKB-b-57	TRUE	reported	8KoqAI
16	20047093	A	G	-0.02563	0.0037	4.30E-12	0.216521	462346	NA	rs4780837	NA	UKB-b-57	TRUE	reported	8KoqAI
16	20049222	G	A	-0.02589	0.003695	2.40E-12	0.218051	462346	NA	rs7277109	NA	UKB-b-57	TRUE	reported	8KoqAI
16	20050466	C	G	-0.02765	0.003575	1.00E-14	0.239571	462346	NA	rs868554	NA	UKB-b-57	TRUE	reported	8KoqAI
16	20050658	C	T	0.021132	0.003237	6.60E-11	0.669316	462346	NA	rs889203	NA	UKB-b-57	TRUE	reported	8KoqAI
16	20052123	G	A	-0.02773	0.003577	9.00E-15	0.239762	462346	NA	rs1379575	NA	UKB-b-57	TRUE	reported	8KoqAI
16	20054371	A	G	-0.02779	0.003578	8.10E-15	0.242376	462346	NA	rs889201	NA	UKB-b-57	TRUE	reported	8KoqAI
16	24693048	A	G	0.037224	0.00656	1.40E-08	0.05671	462346	NA	rs7276862	NA	UKB-b-57	TRUE	reported	8KoqAI
16	28298418	A	C	0.028671	0.00357	9.70E-16	0.243037	462346	NA	rs1122231	NA	UKB-b-57	TRUE	reported	8KoqAI
16	28299132	G	A	-0.0199	0.003039	5.90E-11	0.470688	462346	NA	rs2870996	NA	UKB-b-57	TRUE	reported	8KoqAI
16	28301487	T	C	-0.01977	0.003026	6.40E-11	0.470913	462346	NA	rs9939450	NA	UKB-b-57	TRUE	reported	8KoqAI
16	28304997	T	C	-0.02028	0.00304	2.50E-11	0.451898	462346	NA	rs8052258	NA	UKB-b-57	TRUE	reported	8KoqAI
16	28307940	C	T	-0.02347	0.003057	1.60E-14	0.425094	462346	NA	rs3395752	NA	UKB-b-57	TRUE	reported	8KoqAI
16	28318440	A	G	-0.02468	0.003078	1.10E-15	0.4171	462346	NA	rs2650494	NA	UKB-b-57	TRUE	reported	8KoqAI
16	28318992	C	T	-0.02475	0.003079	9.10E-16	0.417081	462346	NA	rs3425658	NA	UKB-b-57	TRUE	reported	8KoqAI
16	28327463	G	A	-0.02578	0.003105	1.00E-16	0.402745	462346	NA	rs2726039	NA	UKB-b-57	TRUE	reported	8KoqAI
16	28328226	T	A	-0.02582	0.003104	8.80E-17	0.402734	462346	NA	rs2055390	NA	UKB-b-57	TRUE	reported	8KoqAI
16	28330790	C	G	-0.0258	0.003099	8.30E-17	0.404933	462346	NA	rs2726040	NA	UKB-b-57	TRUE	reported	8KoqAI
16	28333411	G	A	0.03268	0.003325	8.40E-23	0.296686	462346	NA	rs2650492	NA	UKB-b-57	TRUE	reported	8KoqAI
16	28336882	T	C	0.032075	0.003236	3.70E-23	0.324113	462346	NA	rs2726034	NA	UKB-b-57	TRUE	reported	8KoqAI
16	28338039	A	G	0.033559	0.003063	6.20E-28	0.422413	462346	NA	rs2726033	NA	UKB-b-57	TRUE	reported	8KoqAI
16	28338043	T	C	0.033753	0.003062	3.00E-28	0.422066	462346	NA	rs2726032	NA	UKB-b-57	TRUE	reported	8KoqAI
16	28340945	A	G	0.034024	0.003071	1.60E-28	0.418455	462346	NA	rs1259835	NA	UKB-b-57	TRUE	reported	8KoqAI
16	28347140	A	C	0.034937	0.003099	1.70E-29	0.398214	462346	NA	rs2726036	NA	UKB-b-57	TRUE	reported	8KoqAI
16	28350730	G	A	0.037955	0.004566	9.30E-17	0.204217	462346	NA	rs1405123	NA	UKB-b-57	TRUE	reported	8KoqAI
16	28379443	A	G	0.033707	0.003122	3.50E-27	0.413756	462346	NA	rs865719	NA	UKB-b-57	TRUE	reported	8KoqAI
16	28383243	C	T	0.034722	0.003114	7.20E-29	0.412267	462346	NA	rs6203256	NA	UKB-b-57	TRUE	reported	8KoqAI
16	28409696	G	A	0.03054	0.004282	9.90E-13	0.741681	462346	NA	rs2856637	NA	UKB-b-57	TRUE	reported	8KoqAI
16	28414300	A	G	0.037273	0.006797	4.20E-08	0.098426	462346	NA	rs1886588	NA	UKB-b-57	TRUE	reported	8KoqAI
16	28417203	T	G	0.033327	0.005683	4.50E-09	0.139712	462346	NA	rs2521334	NA	UKB-b-57	TRUE	reported	8KoqAI
16	28485141	T	C	0.03342	0.003079	1.90E-27	0.408138	462346	NA	rs149299	NA	UKB-b-57	TRUE	reported	8KoqAI
16	28487056	A	G	0.03371	0.003078	6.40E-28	0.408876	462346	NA	rs151179	NA	UKB-b-57	TRUE	reported	8KoqAI
16	28487213	G	T	0.0335	0.00311	4.70E-27	0.43025	462346	NA	rs151180	NA	UKB-b-57	TRUE	reported	8KoqAI
16	28488369	A	T	0.033374	0.003078	2.10E-27	0.408147	462346	NA	rs181195	NA	UKB-b-57	TRUE	reported	8KoqAI
16	28489342	G	A	-0.02671	0.003137	1.70E-17	0.412639	462346	NA	rs2837451	NA	UKB-b-57	TRUE	reported	8KoqAI
16	28489346	T	C	0.032581	0.003045	1.00E-26	0.446203	462346	NA	rs34839	NA	UKB-b-57	TRUE	reported	8KoqAI
16	28490517	T	C	0.033488	0.003076	1.30E-27	0.409689	462346	NA	rs151181	NA	UKB-b-57	TRUE	reported	8KoqAI
16	28492510	C	A	0.033456	0.003077	1.60E-27	0.408242	462346	NA	rs151303	NA	UKB-b-57	TRUE	reported	8KoqAI
16	28494350	A	G	0.032499	0.003045	1.40E-26	0.446345	462346	NA	rs34838	NA	UKB-b-57	TRUE	reported	8KoqAI
16	28494421	A	G	0.032676	0.003046	7.50E-27	0.446554	462346	NA	rs42861	NA	UKB-b-57	TRUE	reported	8KoqAI
16	28494823	A	G	0.032584	0.003045	1.00E-26	0.4465	462346	NA	rs34836	NA	UKB-b-57	TRUE	reported	8KoqAI
16	28495752	C	T	0.033606	0.003078	9.30E-28	0.408564	462346	NA	rs151182	NA	UKB-b-57	TRUE	reported	8KoqAI
16	28497059	T	A	0.033456	0.003077	1.60E-27	0.408321	462346	NA	rs151301	NA	UKB-b-57	TRUE	reported	8KoqAI
16	28499291	A	G	0.034167	0.003076	1.10E-28	0.415833	462346	NA	rs34835	NA	UKB-b-57	TRUE	reported	8KoqAI
16	28504181	G	A	0.034165	0.003073	1.00E-28	0.415068	462346	NA	rs27741	NA	UKB-b-57	TRUE	reported	8KoqAI
16	28506872	A	G	0.033545	0.003077	1.10E-27	0.408765	462346	NA	rs149271	NA	UKB-b-57	TRUE	reported	8KoqAI
16	28507644	C	G	0.033156	0.003078	4.70E-27	0.409012	462346	NA	rs180743	NA	UKB-b-57	TRUE	reported	8KoqAI
16	28508048	A	G	0.03364	0.003078	8.20E-28	0.409126	462346	NA	rs180744	NA	UKB-b-57	TRUE	reported	8KoqAI
16	28508069	C	T	0.033261	0.003079	3.30E-27	0.40803	462346	NA	rs151174	NA	UKB-b-57	TRUE	reported	8KoqAI
16	28508447	A	G	0.034096	0.00307	1.20E-28	0.427823	462346	NA	rs40831	NA	UKB-b-57	TRUE	reported	8KoqAI
16	28510044	C	G	0.024457	0.003031	7.10E-16	0.522644	462346	NA	rs40833	NA	UKB-b-57	TRUE	reported	8KoqAI
16	28510393	C	T	0.028728	0.003038	3.20E-21	0.460692	462346	NA	rs40834	NA	UKB-b-57	TRUE	reported	8KoqAI
16	28510537	A	G	0.028744	0.003036	2.90E-21	0.460839	462346	NA	rs40836	NA	UKB-b-57	TRUE	reported	8KoqAI
16	28510845	A	G	0.028681	0.003036	3.50E-21	0.46069	462346	NA	rs40837	NA	UKB-b-57	TRUE	reported	8KoqAI
16	28512371	A	C	0.028988	0.003218	2.10E-19	0.333765	462346	NA	rs181203	NA	UKB-b-57	TRUE	reported	8KoqAI
16	28513129	T	C	0.028412	0.003226	1.30E-18	0.346012	462346	NA	rs181205	NA	UKB-b-57	TRUE	reported	8KoqAI
16	28513403	A	G	0.028801	0.003228	4.60E-19	0.331901	462346	NA	rs181206	NA	UKB-b-57	TRUE	reported	8KoqAI
16	28513530	C	T	0.029449	0.00322	5.90E-20	0.3338	462346	NA	rs181207	NA	UKB-b-57	TRUE	reported	8KoqAI
16	28514854	G	T	0.029158	0.003219	1.30E-19	0.333277	462346	NA	rs181209	NA	UKB-b-57	TRUE	reported	8KoqAI
16	28517709	T	C	0.0289	0.003037	1.80E-21	0.46072	462346	NA	rs26528	NA	UKB-b-57	TRUE	reported	8KoqAI
16	28519096	T	C	0.028885	0.003037	1.90E-21	0.46075	462346	NA	rs153109	NA	UKB-b-57	TRUE	reported	8KoqAI
16	28523144	T	C	0.029039	0.003198	1.10E-19	0.339794	462346	NA	rs5635490	NA	UKB-b-57	TRUE	reported	8KoqAI
16	28524612	C	G	0.028966	0.003036	1.40E-21	0.461211	462346	NA	rs240707	NA	UKB-b-57	TRUE	reported	8KoqAI

16	28526897	T	C	0.034251	0.003068	6.30E-29	0.417003	462346	NA	rs153106	NA	UKB-b-57	TRUE	reported	8KoqAI
16	28527221	C	T	0.029234	0.003196	5.90E-20	0.340276	462346	NA	rs6203431	NA	UKB-b-57	TRUE	reported	8KoqAI
16	28527326	G	A	0.029236	0.003196	5.80E-20	0.340265	462346	NA	rs7904649	NA	UKB-b-57	TRUE	reported	8KoqAI
16	28528623	T	C	-0.02274	0.003121	3.20E-13	0.403649	462346	NA	rs153103	NA	UKB-b-57	TRUE	reported	8KoqAI
16	28528781	G	A	0.028902	0.0032	1.70E-19	0.341876	462346	NA	rs2844995	NA	UKB-b-57	TRUE	reported	8KoqAI
16	28529716	C	T	0.034262	0.003068	5.90E-29	0.417016	462346	NA	rs240705	NA	UKB-b-57	TRUE	reported	8KoqAI
16	28529949	T	C	0.03432	0.003069	4.90E-29	0.417562	462346	NA	rs240704	NA	UKB-b-57	TRUE	reported	8KoqAI
16	28531287	A	G	0.028617	0.00306	8.60E-21	0.358975	462346	NA	rs4787458	NA	UKB-b-57	TRUE	reported	8KoqAI
16	28531353	C	T	0.034241	0.003068	6.30E-29	0.416991	462346	NA	rs240702	NA	UKB-b-57	TRUE	reported	8KoqAI
16	28532188	T	G	0.034119	0.00307	1.10E-28	0.417064	462346	NA	rs6203431	NA	UKB-b-57	TRUE	reported	8KoqAI
16	28533071	C	T	0.033999	0.003069	1.60E-28	0.416218	462346	NA	rs2869866	NA	UKB-b-57	TRUE	reported	8KoqAI
16	28534038	C	T	0.033929	0.003069	2.10E-28	0.41614	462346	NA	rs6203432	NA	UKB-b-57	TRUE	reported	8KoqAI
16	28535003	A	G	0.034033	0.003069	1.40E-28	0.416292	462346	NA	rs4788085	NA	UKB-b-57	TRUE	reported	8KoqAI
16	28535305	A	G	0.033984	0.003069	1.70E-28	0.41627	462346	NA	rs2877295	NA	UKB-b-57	TRUE	reported	8KoqAI
16	28535834	G	A	0.031637	0.003115	3.20E-24	0.379885	462346	NA	rs6203432	NA	UKB-b-57	TRUE	reported	8KoqAI
16	28536473	C	T	0.033906	0.003069	2.20E-28	0.416101	462346	NA	rs6203432	NA	UKB-b-57	TRUE	reported	8KoqAI
16	28537971	T	C	0.033965	0.003069	1.80E-28	0.416373	462346	NA	rs2106480	NA	UKB-b-57	TRUE	reported	8KoqAI
16	28538640	A	G	0.031326	0.003117	9.10E-24	0.379474	462346	NA	rs6203432	NA	UKB-b-57	TRUE	reported	8KoqAI
16	28539848	C	T	0.033804	0.003069	3.20E-28	0.416114	462346	NA	rs4788084	NA	UKB-b-57	TRUE	reported	8KoqAI
16	28542172	T	C	0.025307	0.003044	9.40E-17	0.540983	462346	NA	rs231977	NA	UKB-b-57	TRUE	reported	8KoqAI
16	28543381	G	A	0.033878	0.003069	2.40E-28	0.416073	462346	NA	rs1244655	NA	UKB-b-57	TRUE	reported	8KoqAI
16	28545449	A	G	0.03397	0.003069	1.80E-28	0.416251	462346	NA	rs4788083	NA	UKB-b-57	TRUE	reported	8KoqAI
16	28545587	G	C	0.025012	0.003042	2.00E-16	0.541723	462346	NA	rs231976	NA	UKB-b-57	TRUE	reported	8KoqAI
16	28549823	A	C	0.025142	0.00304	1.30E-16	0.539629	462346	NA	rs151226	NA	UKB-b-57	TRUE	reported	8KoqAI
16	28550667	C	T	0.03208	0.003125	1.00E-24	0.368522	462346	NA	rs3785354	NA	UKB-b-57	TRUE	reported	8KoqAI
16	28554108	G	A	0.032008	0.003126	1.30E-24	0.368371	462346	NA	rs1074631	NA	UKB-b-57	TRUE	reported	8KoqAI
16	28555400	A	G	0.032069	0.003125	1.00E-24	0.36872	462346	NA	rs4787457	NA	UKB-b-57	TRUE	reported	8KoqAI
16	28555590	C	T	0.025126	0.00304	1.40E-16	0.539526	462346	NA	rs151321	NA	UKB-b-57	TRUE	reported	8KoqAI
16	28555706	T	A	0.034031	0.003072	1.60E-28	0.41386	462346	NA	rs9926245	NA	UKB-b-57	TRUE	reported	8KoqAI
16	28556147	T	C	0.032009	0.003125	1.30E-24	0.368439	462346	NA	rs4788081	NA	UKB-b-57	TRUE	reported	8KoqAI
16	28558081	C	T	0.032058	0.003125	1.10E-24	0.368737	462346	NA	rs4788080	NA	UKB-b-57	TRUE	reported	8KoqAI
16	28559363	C	A	0.032029	0.003125	1.20E-24	0.368743	462346	NA	rs4788079	NA	UKB-b-57	TRUE	reported	8KoqAI
16	28559573	C	T	0.03205	0.003125	1.10E-24	0.368744	462346	NA	rs4787456	NA	UKB-b-57	TRUE	reported	8KoqAI
16	28559981	G	A	0.032028	0.003125	1.20E-24	0.368767	462346	NA	rs4788078	NA	UKB-b-57	TRUE	reported	8KoqAI
16	28561581	C	T	0.024871	0.003049	3.40E-16	0.545324	462346	NA	rs149278	NA	UKB-b-57	TRUE	reported	8KoqAI
16	28561982	T	C	0.034046	0.003071	1.50E-28	0.413956	462346	NA	rs1333117	NA	UKB-b-57	TRUE	reported	8KoqAI
16	28562256	A	G	0.032032	0.003124	1.10E-24	0.368814	462346	NA	rs6203435	NA	UKB-b-57	TRUE	reported	8KoqAI
16	28563026	A	G	0.025033	0.003039	1.80E-16	0.539742	462346	NA	rs151228	NA	UKB-b-57	TRUE	reported	8KoqAI
16	28565489	G	A	0.031831	0.003125	2.30E-24	0.368529	462346	NA	rs6203435	NA	UKB-b-57	TRUE	reported	8KoqAI
16	28565667	C	G	0.034077	0.003072	1.40E-28	0.413926	462346	NA	rs7191618	NA	UKB-b-57	TRUE	reported	8KoqAI
16	28565953	A	G	0.032205	0.003122	5.90E-25	0.369985	462346	NA	rs7187604	NA	UKB-b-57	TRUE	reported	8KoqAI
16	28566158	G	T	0.034021	0.003072	1.60E-28	0.413938	462346	NA	rs3859172	NA	UKB-b-57	TRUE	reported	8KoqAI
16	28569764	G	C	0.032051	0.003122	1.00E-24	0.369449	462346	NA	rs4788077	NA	UKB-b-57	TRUE	reported	8KoqAI
16	28570005	C	T	0.032268	0.003124	5.20E-25	0.369412	462346	NA	rs4788076	NA	UKB-b-57	TRUE	reported	8KoqAI
16	28574615	A	G	0.024987	0.003038	1.90E-16	0.53975	462346	NA	rs480400	NA	UKB-b-57	TRUE	reported	8KoqAI
16	28577931	T	C	-0.01937	0.00318	1.10E-09	0.351849	462346	NA	rs497523	NA	UKB-b-57	TRUE	reported	8KoqAI
16	28580333	T	C	0.024996	0.00304	2.00E-16	0.539855	462346	NA	rs151232	NA	UKB-b-57	TRUE	reported	8KoqAI
16	28580685	A	G	0.031838	0.00312	1.90E-24	0.369746	462346	NA	rs5627220	NA	UKB-b-57	TRUE	reported	8KoqAI
16	28580829	C	T	0.031953	0.003119	1.30E-24	0.370135	462346	NA	rs5639627	NA	UKB-b-57	TRUE	reported	8KoqAI
16	28581038	G	C	0.031846	0.00312	1.90E-24	0.369745	462346	NA	rs6203435	NA	UKB-b-57	TRUE	reported	8KoqAI
16	28582142	G	T	-0.02511	0.003043	1.60E-16	0.459751	462346	NA	rs193628	NA	UKB-b-57	TRUE	reported	8KoqAI
16	28582849	A	G	0.025088	0.003041	1.60E-16	0.539766	462346	NA	rs151231	NA	UKB-b-57	TRUE	reported	8KoqAI
16	28582941	C	A	0.032322	0.003117	3.40E-25	0.379709	462346	NA	rs1244746	NA	UKB-b-57	TRUE	reported	8KoqAI
16	28583362	A	G	0.032325	0.003117	3.40E-25	0.379742	462346	NA	rs1121910	NA	UKB-b-57	TRUE	reported	8KoqAI
16	28583610	C	T	0.032326	0.003117	3.40E-25	0.379742	462346	NA	rs1116935	NA	UKB-b-57	TRUE	reported	8KoqAI
16	28583865	C	T	0.032326	0.003117	3.40E-25	0.379743	462346	NA	rs1107491	NA	UKB-b-57	TRUE	reported	8KoqAI
16	28585636	C	T	0.032352	0.003117	3.10E-25	0.379633	462346	NA	rs5620919	NA	UKB-b-57	TRUE	reported	8KoqAI
16	28586127	T	C	0.031716	0.003098	1.30E-24	0.372148	462346	NA	rs7193402	NA	UKB-b-57	TRUE	reported	8KoqAI
16	28587389	T	C	0.032322	0.003117	3.40E-25	0.379736	462346	NA	rs7186573	NA	UKB-b-57	TRUE	reported	8KoqAI
16	28587597	G	T	0.031998	0.003119	1.10E-24	0.370132	462346	NA	rs6203435	NA	UKB-b-57	TRUE	reported	8KoqAI
16	28588049	A	C	0.032004	0.003119	1.10E-24	0.370151	462346	NA	rs1244582	NA	UKB-b-57	TRUE	reported	8KoqAI
16	28588395	A	C	0.03199	0.003119	1.10E-24	0.370142	462346	NA	rs1244574	NA	UKB-b-57	TRUE	reported	8KoqAI
16	28588443	C	T	0.032454	0.003119	2.40E-25	0.379544	462346	NA	rs1244842	NA	UKB-b-57	TRUE	reported	8KoqAI
16	28589455	G	A	0.032004	0.003119	1.10E-24	0.370128	462346	NA	rs4787455	NA	UKB-b-57	TRUE	reported	8KoqAI
16	28590862	C	T	0.032306	0.003117	3.60E-25	0.379725	462346	NA	rs1120477	NA	UKB-b-57	TRUE	reported	8KoqAI
16	28590989	C	T	0.031902	0.00312	1.50E-24	0.369744	462346	NA	rs1128317	NA	UKB-b-57	TRUE	reported	8KoqAI
16	28593347	T	C	0.03216	0.003117	5.90E-25	0.379538	462346	NA	rs1770730	NA	UKB-b-57	TRUE	reported	8KoqAI
16	28593597	G	A	0.031973	0.003119	1.20E-24	0.370097	462346	NA	rs4788074	NA	UKB-b-57	TRUE	reported	8KoqAI
16	28594549	A	G	0.032304	0.003117	3.60E-25	0.379733	462346	NA	rs4788073	NA	UKB-b-57	TRUE	reported	8KoqAI
16	28595700	A	G	0.032327	0.003117	3.40E-25	0.379691	462346	NA	rs1764000	NA	UKB-b-57	TRUE	reported	8KoqAI
16	28595810	C	G	0.032334	0.003118	3.30E-25	0.379677	462346	NA	rs7553955	NA	UKB-b-57	TRUE	reported	8KoqAI
16	28599411	A	G	0.032343	0.003118	3.30E-25	0.379708	462346	NA	rs5579203	NA	UKB-b-57	TRUE	reported	8KoqAI
16	28600091	C	T	0.032052	0.003121	9.60E-25	0.370001	462346	NA	rs6203435	NA	UKB-b-57	TRUE	reported	8KoqAI
16	28603168	T	A	0.032086	0.003124	9.40E-25	0.369975	462346	NA	rs2077031	NA	UKB-b-57	TRUE	reported	8KoqAI
16	28603335	T	C	0.032027	0.003125	1.20E-24	0.369551	462346	NA	rs762634	NA	UKB-b-57	TRUE	reported	8KoqAI
16	28603342	A	G	0.032126	0.003126	8.80E-25	0.369796	462346	NA	rs710410	NA	UKB-b-57	TRUE	reported	8KoqAI
16	28603655	T	G	0.032065	0.003124	1.00E-24	0.370002	462346	NA	rs1059491	NA	UKB-b-57	TRUE	reported	8KoqAI
16	28604686	A	G	0.025362	0.003052	9.60E-17	0.539641	462346	NA	rs3743963	NA	UKB-b-57	TRUE	reported	8KoqAI
16	28605915	T	A	0.032352	0.003122	3.70E-25	0.379679	462346	NA	rs6203156	NA	UKB-b-57	TRUE	reported	8KoqAI
16	28606160	G	T	0.032333	0.003122	3.90E-25	0.379669	462346	NA	rs1132083	NA	UKB-b-57	TRUE	reported	8KoqAI
16	28607070	A	G	0.032345	0.003123	3.90E-25	0.379613	462346	NA	rs4149406	NA	UKB-b-57	TRUE	reported	8KoqAI
16	28607532	G	A	0.030905	0.003194	3.80E-22	0.34778	462346	NA	rs4115668	NA	UKB-b-57	TRUE	reported	8KoqAI
16	28608230	G	A	0.032355	0.003123	3.80E-25	0.379688	462346	NA	rs743590	NA	UKB-b-57	TRUE	reported	8KoqAI
16	28608341	T	C	0.032143	0.003126	8.50E-25	0.369976	462346	NA	rs762633	NA	UKB-b-57	TRUE	reported	8KoqAI
16	28608746	A	C	0.032159	0.003127	8.20E-25	0.370115	462346	NA	rs3517581	NA	UKB-b-57	TRUE	reported	8KoqAI
16	28608938	C	G	0.032186	0.003127	7.60E-25	0.370111	462346	NA	rs4149398	NA	UKB-b-57	TRUE	reported	8KoqAI
16	28609329	A	T	0.031596	0.003136	7.10E-24	0.375558	462346	NA	rs6203156	NA	UKB-b-57	TRUE	reported	8KoqAI
16	28609963	C	G	-0.01766	0.003229	4.50E-08	0.520123	462346	NA	rs4787453	NA	UKB-b-57	TRUE	reported	8KoqAI
16	28613965	A	G	0.033927	0.003165	8.10E-27	0.392082	462346	NA	rs1107490	NA	UKB-b-57	TRUE	reported	8KoqAI
16	28614488	C	T	0.031825	0.003236	8.10E-23	0.3447	462346	NA	rs7192601	NA	UKB-b-57	TRUE	reported	8KoqAI
16	28614734	T	C	0.033928	0.003161	7.10E-27	0.392904	462346	NA	rs7191548	NA	UKB-b-57	TRUE	reported	8KoqAI
16	28616259	T	G	0.03408	0.003162	4.30E-27	0.393051	462346	NA	rs6498091	NA	UKB-b-57	TRUE	reported	8KoqAI
16	28616399	G	A	0.033935	0.003162	7.10E-27	0.392877	462346	NA	rs4788070	NA	UKB-b-57	TRUE	reported	8KoqAI
16	28616665	C	A	0.034048	0.003165	5.50E-27	0.392203	462346	NA	rs4788069	NA	UKB-b-57	TRUE	reported	8KoqAI
16	28616723	C	T	0.03409	0.003165	4.70E-27	0.392221	462346	NA	rs4788068	NA	UKB-b-57	TRUE	reported	8KoqAI
16	28617057	G	A	0.034027	0.003166	6.10E-27	0.39204	462346	NA	rs1042157	NA	UKB-b-57	TRUE	reported	8KoqAI
16	28617128	T	C	0.031333	0.003229	2.90E-22	0.354349	462346	NA	rs6839	NA	UKB-b-57	TRUE	reported	8KoqAI
16	28617514	C	T	0.031635	0.00325	2.20E-22	0.342271	462346	NA	rs1042028	NA	UKB-b-57	TRUE	reported	8KoqAI
16	28617552	C	G	0.034495	0.003181	2.10E-27	0.391532	462346	NA	rs3176926	NA	UKB-b-57	TRUE	reported	8KoqAI
16	28617574	C	T	0.033966	0.003179	1.20E-26	0.388976	462346	NA	rs4149394	NA	UKB-b-57	TRUE	reported	8KoqAI
16	28617795	G	A	0.034168	0.003178	5.90E-27	0.389372	462346	NA	rs7903969	NA	UKB-b-57	TRUE	reported	8KoqAI
16	28617813	A	G	0.03403	0.003179	9.70E-27	0.389148	462346	NA	rs4149392	NA	UKB-b-57	TRUE	reported	8KoqAI
16	28617885	G	A	0.033487	0.003253	7.60E-25	0.371676	462346	NA	rs7530164	NA	UKB-b-57	TRUE	reported	8KoqAI
16	28617890	A	G	0.033933	0.003254	1.80E-25	0.371373	462346	NA	rs1168405	NA	UKB-b-57	TRUE	reported	8KoqAI
16	28617934	T	C	0.03452	0.003334	4.10E-25	0.354042	462346	NA	rs1113841	NA	UKB-b-57	TRUE	reported	8KoqAI
16	28617971	T	C	0.034588	0.003827	1.60E-19	0.277306	462346	NA	rs4149391	NA	UKB-b-57	TRUE	reported	8KoqAI
16	28617993	C	A	0.03542	0.004168	1.90E-17	0.239206	462346	NA	rs4127815	NA	UKB-b-57	TRUE	reported	8KoqAI

16	28618047	A	T	0.030733	0.005409	1.30E-08	0.148166	462346	NA	rs4149388	NA	UKB-b-57	TRUE	reported	8KoqAI
16	28618068	G	A	0.032486	0.00387	4.70E-17	0.270354	462346	NA	rs7475211	NA	UKB-b-57	TRUE	reported	8KoqAI
16	28618237	T	C	0.034225	0.003169	3.40E-27	0.391417	462346	NA	rs9282862	NA	UKB-b-57	TRUE	reported	8KoqAI
16	28618545	G	T	0.031839	0.003231	6.50E-23	0.354428	462346	NA	rs2925624	NA	UKB-b-57	TRUE	reported	8KoqAI
16	28618636	A	G	0.034584	0.003168	9.50E-28	0.392039	462346	NA	rs3020804	NA	UKB-b-57	TRUE	reported	8KoqAI
16	28618708	C	T	-0.02702	0.003176	1.80E-17	0.43573	462346	NA	rs1164819	NA	UKB-b-57	TRUE	reported	8KoqAI
16	28618914	T	A	0.0343	0.003168	2.60E-27	0.391589	462346	NA	rs3020803	NA	UKB-b-57	TRUE	reported	8KoqAI
16	28619076	T	C	0.034493	0.003168	1.30E-27	0.39175	462346	NA	rs2925629	NA	UKB-b-57	TRUE	reported	8KoqAI
16	28619132	T	C	0.034432	0.003168	1.60E-27	0.391673	462346	NA	rs2925630	NA	UKB-b-57	TRUE	reported	8KoqAI
16	28619911	T	C	0.034473	0.003168	1.40E-27	0.391975	462346	NA	rs1126447	NA	UKB-b-57	TRUE	reported	8KoqAI
16	28620572	C	T	0.027947	0.003133	4.70E-19	0.482683	462346	NA	rs750155	NA	UKB-b-57	TRUE	reported	8KoqAI
16	28620800	C	G	-0.02498	0.003229	1.00E-14	0.395658	462346	NA	rs3760091	NA	UKB-b-57	TRUE	reported	8KoqAI
16	28621311	T	C	0.022878	0.003543	1.10E-10	0.699325	462346	NA	rs2077412	NA	UKB-b-57	TRUE	reported	8KoqAI
16	28622154	C	G	-0.02413	0.003262	1.40E-13	0.395509	462346	NA	rs9922110	NA	UKB-b-57	TRUE	reported	8KoqAI
16	28622993	A	G	-0.03343	0.003213	2.40E-25	0.602929	462346	NA	rs2925635	NA	UKB-b-57	TRUE	reported	8KoqAI
16	28626966	T	C	-0.03341	0.003124	1.10E-26	0.624081	462346	NA	rs2872918	NA	UKB-b-57	TRUE	reported	8KoqAI
16	28627004	T	C	-0.03341	0.003124	1.10E-26	0.624073	462346	NA	rs2848036	NA	UKB-b-57	TRUE	reported	8KoqAI
16	28627063	C	T	-0.03345	0.003124	9.50E-27	0.624088	462346	NA	rs2867683	NA	UKB-b-57	TRUE	reported	8KoqAI
16	28629300	C	T	-0.03339	0.003123	1.10E-26	0.624137	462346	NA	rs6498089	NA	UKB-b-57	TRUE	reported	8KoqAI
16	28631360	A	T	-0.0335	0.003131	1.00E-26	0.62877	462346	NA	rs2841008	NA	UKB-b-57	TRUE	reported	8KoqAI
16	28631530	A	T	0.033149	0.003133	3.70E-26	0.370128	462346	NA	rs6203160	NA	UKB-b-57	TRUE	reported	8KoqAI
16	28631585	T	G	-0.03349	0.003129	1.00E-26	0.62878	462346	NA	rs1968752	NA	UKB-b-57	TRUE	reported	8KoqAI
16	28631759	T	C	-0.03369	0.003131	5.20E-27	0.628854	462346	NA	rs1968751	NA	UKB-b-57	TRUE	reported	8KoqAI
16	28631856	G	T	0.029333	0.004669	3.30E-10	0.132204	462346	NA	rs1487381	NA	UKB-b-57	TRUE	reported	8KoqAI
16	28632021	T	G	-0.03508	0.00309	7.30E-30	0.597353	462346	NA	rs2411453	NA	UKB-b-57	TRUE	reported	8KoqAI
16	28632288	G	C	-0.0338	0.003132	3.80E-27	0.628905	462346	NA	rs8049739	NA	UKB-b-57	TRUE	reported	8KoqAI
16	28643300	T	G	0.032242	0.003374	1.20E-21	0.282834	462346	NA	rs4788061	NA	UKB-b-57	TRUE	reported	8KoqAI
16	28644459	C	T	0.032212	0.003375	1.40E-21	0.282664	462346	NA	rs1244600	NA	UKB-b-57	TRUE	reported	8KoqAI
16	28644663	A	T	0.021538	0.003391	2.10E-10	0.702009	462346	NA	rs7193413	NA	UKB-b-57	TRUE	reported	8KoqAI
16	28644980	G	A	0.032515	0.00337	5.00E-22	0.284216	462346	NA	rs9929198	NA	UKB-b-57	TRUE	reported	8KoqAI
16	28645127	C	T	0.032268	0.003389	1.70E-21	0.280225	462346	NA	rs1118203	NA	UKB-b-57	TRUE	reported	8KoqAI
16	28647568	T	C	0.032945	0.003377	1.70E-22	0.282281	462346	NA	rs3987654	NA	UKB-b-57	TRUE	reported	8KoqAI
16	28649651	C	A	0.034484	0.003084	5.10E-29	0.405508	462346	NA	rs3562651	NA	UKB-b-57	TRUE	reported	8KoqAI
16	28651732	C	T	0.032233	0.003366	1.00E-21	0.281705	462346	NA	rs7279380	NA	UKB-b-57	TRUE	reported	8KoqAI
16	28718755	A	G	0.033176	0.003408	2.10E-22	0.338101	462346	NA	rs1882370	NA	UKB-b-57	TRUE	reported	8KoqAI
16	28754684	G	A	0.033566	0.00309	1.80E-27	0.415198	462346	NA	rs378421	NA	UKB-b-57	TRUE	reported	8KoqAI
16	28823097	A	G	0.032404	0.003088	9.20E-26	0.400044	462346	NA	rs6203661	NA	UKB-b-57	TRUE	reported	8KoqAI
16	28823755	G	A	-0.01874	0.00334	2.00E-08	0.299909	462346	NA	rs4533290	NA	UKB-b-57	TRUE	reported	8KoqAI
16	28824685	T	C	0.032448	0.003087	7.80E-26	0.399995	462346	NA	rs6203661	NA	UKB-b-57	TRUE	reported	8KoqAI
16	28825100	T	C	0.032463	0.003086	7.10E-26	0.400954	462346	NA	rs1130299	NA	UKB-b-57	TRUE	reported	8KoqAI
16	28825420	C	T	0.03245	0.003086	7.40E-26	0.400976	462346	NA	rs1186051	NA	UKB-b-57	TRUE	reported	8KoqAI
16	28825605	G	A	0.032451	0.003086	7.30E-26	0.400982	462346	NA	rs2008514	NA	UKB-b-57	TRUE	reported	8KoqAI
16	28825777	C	T	0.032452	0.003086	7.30E-26	0.400988	462346	NA	rs1987472	NA	UKB-b-57	TRUE	reported	8KoqAI
16	28825866	T	G	0.032457	0.003086	7.20E-26	0.400985	462346	NA	rs1987471	NA	UKB-b-57	TRUE	reported	8KoqAI
16	28825953	A	G	0.032539	0.003085	5.30E-26	0.400922	462346	NA	rs5618613	NA	UKB-b-57	TRUE	reported	8KoqAI
16	28826049	C	T	0.0197	0.003357	4.40E-09	0.704174	462346	NA	rs2847231	NA	UKB-b-57	TRUE	reported	8KoqAI
16	28826307	A	G	0.032433	0.003087	8.20E-26	0.400048	462346	NA	rs6203661	NA	UKB-b-57	TRUE	reported	8KoqAI
16	28827498	T	C	0.032489	0.003086	6.50E-26	0.400967	462346	NA	rs6203661	NA	UKB-b-57	TRUE	reported	8KoqAI
16	28828834	A	C	0.032509	0.003086	6.10E-26	0.400942	462346	NA	rs6203661	NA	UKB-b-57	TRUE	reported	8KoqAI
16	28829228	A	G	0.021941	0.003177	5.00E-12	0.649682	462346	NA	rs2888876	NA	UKB-b-57	TRUE	reported	8KoqAI
16	28831359	T	C	0.032546	0.003087	5.40E-26	0.4009	462346	NA	rs4788095	NA	UKB-b-57	TRUE	reported	8KoqAI
16	28832382	C	T	0.032634	0.003087	4.00E-26	0.40091	462346	NA	rs7279380	NA	UKB-b-57	TRUE	reported	8KoqAI
16	28833097	A	G	0.032561	0.003087	5.20E-26	0.40088	462346	NA	rs6203662	NA	UKB-b-57	TRUE	reported	8KoqAI
16	28833299	C	A	0.032559	0.003087	5.20E-26	0.400879	462346	NA	rs6203662	NA	UKB-b-57	TRUE	reported	8KoqAI
16	28836205	T	C	0.032506	0.003087	6.30E-26	0.400882	462346	NA	rs4451951	NA	UKB-b-57	TRUE	reported	8KoqAI
16	28837203	T	G	0.032555	0.003088	5.60E-26	0.400019	462346	NA	rs6203662	NA	UKB-b-57	TRUE	reported	8KoqAI
16	28837515	T	C	0.032823	0.003085	1.90E-26	0.402461	462346	NA	rs8049439	NA	UKB-b-57	TRUE	reported	8KoqAI
16	28837906	A	G	0.032207	0.003089	1.90E-25	0.398716	462346	NA	rs8062405	NA	UKB-b-57	TRUE	reported	8KoqAI
16	28838073	A	G	0.021911	0.003221	1.00E-11	0.652891	462346	NA	rs1244848	NA	UKB-b-57	TRUE	reported	8KoqAI
16	28839392	G	A	0.03268	0.00309	3.90E-26	0.39777	462346	NA	rs7279381	NA	UKB-b-57	TRUE	reported	8KoqAI
16	28839425	T	C	0.032634	0.003089	4.30E-26	0.398731	462346	NA	rs7861323	NA	UKB-b-57	TRUE	reported	8KoqAI
16	28839735	C	G	0.032659	0.00309	4.20E-26	0.397794	462346	NA	rs6203662	NA	UKB-b-57	TRUE	reported	8KoqAI
16	28839930	G	T	0.032643	0.003089	4.20E-26	0.398737	462346	NA	rs6203662	NA	UKB-b-57	TRUE	reported	8KoqAI
16	28840381	A	G	0.032582	0.003088	5.10E-26	0.399905	462346	NA	rs7279381	NA	UKB-b-57	TRUE	reported	8KoqAI
16	28841777	C	T	0.032721	0.00309	3.40E-26	0.397632	462346	NA	rs1244388	NA	UKB-b-57	TRUE	reported	8KoqAI
16	28843025	C	T	0.032779	0.003089	2.60E-26	0.398551	462346	NA	rs5599157	NA	UKB-b-57	TRUE	reported	8KoqAI
16	28843118	A	G	0.032779	0.003089	2.60E-26	0.398553	462346	NA	rs5635868	NA	UKB-b-57	TRUE	reported	8KoqAI
16	28844068	T	A	0.02246	0.003171	1.40E-12	0.648771	462346	NA	rs7498491	NA	UKB-b-57	TRUE	reported	8KoqAI
16	28844284	A	G	0.032789	0.003089	2.50E-26	0.398553	462346	NA	rs6203662	NA	UKB-b-57	TRUE	reported	8KoqAI
16	28844365	G	A	0.032789	0.003089	2.60E-26	0.398553	462346	NA	rs6203665	NA	UKB-b-57	TRUE	reported	8KoqAI
16	28845251	G	A	0.032794	0.003089	2.50E-26	0.39853	462346	NA	rs1244417	NA	UKB-b-57	TRUE	reported	8KoqAI
16	28846840	T	G	0.032807	0.003089	2.40E-26	0.398532	462346	NA	rs5640491	NA	UKB-b-57	TRUE	reported	8KoqAI
16	28846866	G	A	0.032794	0.003089	2.50E-26	0.398517	462346	NA	rs5571989	NA	UKB-b-57	TRUE	reported	8KoqAI
16	28847149	G	C	0.032783	0.003089	2.60E-26	0.398543	462346	NA	rs5583074	NA	UKB-b-57	TRUE	reported	8KoqAI
16	28847246	T	C	0.03255	0.003082	4.50E-26	0.407004	462346	NA	rs1292840	NA	UKB-b-57	TRUE	reported	8KoqAI
16	28848668	T	C	0.032821	0.00309	2.40E-26	0.397621	462346	NA	rs1232511	NA	UKB-b-57	TRUE	reported	8KoqAI
16	28848818	A	G	0.03273	0.003089	3.20E-26	0.398487	462346	NA	rs1232527	NA	UKB-b-57	TRUE	reported	8KoqAI
16	28850371	T	G	0.032575	0.003087	5.00E-26	0.402364	462346	NA	rs6203665	NA	UKB-b-57	TRUE	reported	8KoqAI
16	28853996	A	C	0.032814	0.003089	2.30E-26	0.398657	462346	NA	rs3088215	NA	UKB-b-57	TRUE	reported	8KoqAI
16	28854769	A	G	0.032784	0.003089	2.60E-26	0.398573	462346	NA	rs2840362	NA	UKB-b-57	TRUE	reported	8KoqAI
16	28855522	G	C	0.032784	0.003089	2.60E-26	0.398573	462346	NA	rs6173756	NA	UKB-b-57	TRUE	reported	8KoqAI
16	28855727	A	G	0.032784	0.003089	2.60E-26	0.398573	462346	NA	rs4788099	NA	UKB-b-57	TRUE	reported	8KoqAI
16	28857645	A	G	0.032841	0.003085	1.90E-26	0.402132	462346	NA	rs7187776	NA	UKB-b-57	TRUE	reported	8KoqAI
16	28859802	A	G	0.032764	0.003089	2.80E-26	0.398565	462346	NA	rs9972693	NA	UKB-b-57	TRUE	reported	8KoqAI
16	28860183	G	A	0.032807	0.003089	2.40E-26	0.399157	462346	NA	rs1130797	NA	UKB-b-57	TRUE	reported	8KoqAI
16	28860237	C	G	0.032778	0.003089	2.60E-26	0.39903	462346	NA	rs1232501	NA	UKB-b-57	TRUE	reported	8KoqAI
16	28861734	A	C	0.032753	0.003089	2.90E-26	0.398562	462346	NA	rs9972768	NA	UKB-b-57	TRUE	reported	8KoqAI
16	28861881	C	T	0.03275	0.003089	2.90E-26	0.398549	462346	NA	rs5604078	NA	UKB-b-57	TRUE	reported	8KoqAI
16	28863451	G	A	0.032753	0.003089	2.90E-26	0.398562	462346	NA	rs7279381	NA	UKB-b-57	TRUE	reported	8KoqAI
16	28864673	T	C	0.032709	0.003089	3.40E-26	0.398554	462346	NA	rs4788100	NA	UKB-b-57	TRUE	reported	8KoqAI
16	28865042	T	C	0.032694	0.003089	3.50E-26	0.398578	462346	NA	rs6203736	NA	UKB-b-57	TRUE	reported	8KoqAI
16	28865892	C	T	0.032705	0.003089	3.40E-26	0.398499	462346	NA	rs7205323	NA	UKB-b-57	TRUE	reported	8KoqAI
16	28865916	G	A	0.032723	0.003091	3.40E-26	0.397526	462346	NA	rs7187333	NA	UKB-b-57	TRUE	reported	8KoqAI
16	28867804	C	T	0.032732	0.003089	3.10E-26	0.398518	462346	NA	rs4788101	NA	UKB-b-57	TRUE	reported	8KoqAI
16	28868695	G	A	0.032356	0.003092	1.20E-25	0.403753	462346	NA	rs6203736	NA	UKB-b-57	TRUE	reported	8KoqAI
16	28868962	C	G	0.032595	0.003091	5.30E-26	0.39817	462346	NA	rs6203736	NA	UKB-b-57	TRUE	reported	8KoqAI
16	28870596	A	G	0.032743	0.003089	3.00E-26	0.398595	462346	NA	rs1115060	NA	UKB-b-57	TRUE	reported	8KoqAI
16	28870962	G	A	0.032712	0.00309	3.50E-26	0.397699	462346	NA	rs1244658	NA	UKB-b-57	TRUE	reported	8KoqAI
16	28871191	C	G	0.032711	0.0031	5.00E-26	0.39553	462346	NA	rs1244890	NA	UKB-b-57	TRUE	reported	8KoqAI
16	28871657	C	A	0.032757	0.00309	3.00E-26	0.397647	462346	NA	rs6203736	NA	UKB-b-57	TRUE	reported	8KoqAI
16	28871853	C	T	0.032734	0.003089	3.10E-26	0.398581	462346	NA	rs1185951	NA	UKB-b-57	TRUE	reported	8KoqAI
16	28871860	A	G	0.032743	0.003089	3.00E-26	0.398595	462346	NA	rs1186113	NA	UKB-b-57	TRUE	reported	8KoqAI
16	28871989	A	G	0.032743	0.003089	3.00E-26	0.398595	462346	NA	rs1186117	NA	UKB-b-57	TRUE	reported	8KoqAI
16	28872354	G	C	0.032759	0.00309	3.00E-26	0.397643	462346	NA	rs7499337	NA	UKB-b-57	TRUE	reported	8KoqAI
16	28872598	T	C	0.032743	0.003089	3.00E-26	0.398595	462346	NA	rs6565229	NA	UKB-b-57	TRUE	reported	8KoqAI
16	28873398	G	A	0.032807	0.003089	2.40E-26	0.398577	462346	NA	rs4788102	NA	UKB-b-57	TRUE	reported	8KoqAI
16	28874547	C	G	0.032725	0.003089	3.20E-26	0.398572	462346	NA	rs6203736	NA	UKB-b-57	TRUE	reported	8KoqAI

16	28875122	T	G	0.032744	0.003089	3.00E-26	0.398595	462346	NA	rs7198606	NA	UKB-b-57	TRUE	reported	8KoqAI
16	28875204	A	T	0.032744	0.003089	3.00E-26	0.398595	462346	NA	rs1186475	NA	UKB-b-57	TRUE	reported	8KoqAI
16	28875482	A	G	0.032744	0.003089	3.00E-26	0.398595	462346	NA	rs7193733	NA	UKB-b-57	TRUE	reported	8KoqAI
16	28879484	A	G	0.032749	0.003091	3.10E-26	0.398382	462346	NA	rs1186337	NA	UKB-b-57	TRUE	reported	8KoqAI
16	28881001	G	T	0.022361	0.003163	1.50E-12	0.644862	462346	NA	rs1115062	NA	UKB-b-57	TRUE	reported	8KoqAI
16	28881202	A	C	0.03274	0.003089	3.00E-26	0.398594	462346	NA	rs8055982	NA	UKB-b-57	TRUE	reported	8KoqAI
16	28882424	T	C	0.032709	0.003089	3.40E-26	0.398617	462346	NA	rs7498603	NA	UKB-b-57	TRUE	reported	8KoqAI
16	28883241	A	G	0.03308	0.003093	1.10E-26	0.399688	462346	NA	rs7498665	NA	UKB-b-57	TRUE	reported	8KoqAI
16	28883327	T	C	0.022108	0.003162	2.70E-12	0.644084	462346	NA	rs2843334	NA	UKB-b-57	TRUE	reported	8KoqAI
16	28883841	C	T	0.032779	0.00309	2.80E-26	0.397619	462346	NA	rs6203736	NA	UKB-b-57	TRUE	reported	8KoqAI
16	28885659	C	T	0.032813	0.00309	2.50E-26	0.397548	462346	NA	rs7359397	NA	UKB-b-57	TRUE	reported	8KoqAI
16	28885931	T	C	0.032738	0.003089	3.10E-26	0.398593	462346	NA	rs1186410	NA	UKB-b-57	TRUE	reported	8KoqAI
16	28889486	C	A	0.032725	0.003089	3.20E-26	0.398625	462346	NA	rs3888190	NA	UKB-b-57	TRUE	reported	8KoqAI
16	28890131	C	A	0.032733	0.003089	3.10E-26	0.398621	462346	NA	rs6203737	NA	UKB-b-57	TRUE	reported	8KoqAI
16	28891465	C	T	0.032748	0.00309	3.00E-26	0.398607	462346	NA	rs8055138	NA	UKB-b-57	TRUE	reported	8KoqAI
16	28893295	C	G	0.022167	0.003164	2.40E-12	0.644032	462346	NA	rs6203737	NA	UKB-b-57	TRUE	reported	8KoqAI
16	28893532	A	G	0.0328	0.003091	2.60E-26	0.398608	462346	NA	rs1164121	NA	UKB-b-57	TRUE	reported	8KoqAI
16	28893571	T	C	0.032827	0.003092	2.50E-26	0.39764	462346	NA	rs7498555	NA	UKB-b-57	TRUE	reported	8KoqAI
16	28895130	A	G	0.032899	0.003091	1.90E-26	0.398636	462346	NA	rs8061590	NA	UKB-b-57	TRUE	reported	8KoqAI
16	28896015	C	T	0.032404	0.003166	1.40E-24	0.354569	462346	NA	rs1164244	NA	UKB-b-57	TRUE	reported	8KoqAI
16	28897452	G	A	0.03247	0.003166	1.10E-24	0.354667	462346	NA	rs8056890	NA	UKB-b-57	TRUE	reported	8KoqAI
16	28898793	T	C	0.031956	0.003159	4.60E-24	0.365831	462346	NA	rs6565259	NA	UKB-b-57	TRUE	reported	8KoqAI
16	28899411	C	T	-0.01978	0.003306	2.20E-09	0.319029	462346	NA	rs7501203	NA	UKB-b-57	TRUE	reported	8KoqAI
16	28903188	T	C	-0.02244	0.003165	1.40E-12	0.356101	462346	NA	rs9746702	NA	UKB-b-57	TRUE	reported	8KoqAI
16	28906084	G	C	-0.03251	0.003167	1.00E-24	0.646349	462346	NA	rs9931989	NA	UKB-b-57	TRUE	reported	8KoqAI
16	28913787	T	C	-0.0328	0.003165	3.60E-25	0.64702	462346	NA	rs7189927	NA	UKB-b-57	TRUE	reported	8KoqAI
16	28915217	G	A	-0.03263	0.003167	6.80E-25	0.647495	462346	NA	rs8046545	NA	UKB-b-57	TRUE	reported	8KoqAI
16	28915433	A	C	-0.0223	0.003175	2.10E-12	0.354434	462346	NA	rs2071342	NA	UKB-b-57	TRUE	reported	8KoqAI
16	28915527	G	A	-0.03252	0.003167	9.70E-25	0.647618	462346	NA	rs10499	NA	UKB-b-57	TRUE	reported	8KoqAI
16	28917644	T	C	-0.03265	0.003166	6.20E-25	0.646859	462346	NA	rs7188071	NA	UKB-b-57	TRUE	reported	8KoqAI
16	28917746	G	A	-0.02228	0.003164	1.90E-12	0.357275	462346	NA	rs6203741	NA	UKB-b-57	TRUE	reported	8KoqAI
16	28919341	A	G	-0.03262	0.003168	7.30E-25	0.647741	462346	NA	rs7206214	NA	UKB-b-57	TRUE	reported	8KoqAI
16	28921809	T	C	-0.03082	0.003214	9.00E-22	0.668709	462346	NA	rs7184597	NA	UKB-b-57	TRUE	reported	8KoqAI
16	28922149	C	T	-0.03066	0.003211	1.30E-21	0.667459	462346	NA	rs1164665	NA	UKB-b-57	TRUE	reported	8KoqAI
16	28922743	A	G	-0.03091	0.003215	7.10E-22	0.668721	462346	NA	rs9708958	NA	UKB-b-57	TRUE	reported	8KoqAI
16	28925978	T	C	-0.03088	0.003221	9.10E-22	0.669152	462346	NA	rs3924376	NA	UKB-b-57	TRUE	reported	8KoqAI
16	28927818	C	A	-0.03088	0.00321	6.50E-22	0.66312	462346	NA	rs1291905	NA	UKB-b-57	TRUE	reported	8KoqAI
16	28933248	C	T	-0.03001	0.003214	9.80E-21	0.664427	462346	NA	rs6565293	NA	UKB-b-57	TRUE	reported	8KoqAI
16	28933468	C	T	-0.02991	0.003211	1.20E-20	0.663273	462346	NA	rs7185232	NA	UKB-b-57	TRUE	reported	8KoqAI
16	28937259	T	C	-0.02997	0.00321	9.80E-21	0.663028	462346	NA	rs4072402	NA	UKB-b-57	TRUE	reported	8KoqAI
16	28940916	T	G	-0.0214	0.003147	1.10E-11	0.367464	462346	NA	rs5693381	NA	UKB-b-57	TRUE	reported	8KoqAI
16	28944396	C	G	-0.02916	0.003233	1.90E-19	0.675078	462346	NA	rs2904880	NA	UKB-b-57	TRUE	reported	8KoqAI
16	28944700	G	T	-0.02251	0.003162	1.10E-12	0.357064	462346	NA	rs3597929	NA	UKB-b-57	TRUE	reported	8KoqAI
16	28950432	C	T	-0.02227	0.003157	1.70E-12	0.360239	462346	NA	rs2070962	NA	UKB-b-57	TRUE	reported	8KoqAI
16	28951457	C	T	-0.02317	0.004012	7.70E-09	0.182718	462346	NA	rs1164530	NA	UKB-b-57	TRUE	reported	8KoqAI
16	28952638	T	C	-0.02992	0.003243	2.80E-20	0.67755	462346	NA	rs1163989	NA	UKB-b-57	TRUE	reported	8KoqAI
16	28954408	C	T	-0.02229	0.003156	1.60E-12	0.360323	462346	NA	rs6203742	NA	UKB-b-57	TRUE	reported	8KoqAI
16	28956464	T	C	-0.0215	0.003137	7.20E-12	0.370394	462346	NA	rs7188755	NA	UKB-b-57	TRUE	reported	8KoqAI
16	28958754	A	G	-0.02189	0.003137	3.00E-12	0.370084	462346	NA	rs2081199	NA	UKB-b-57	TRUE	reported	8KoqAI
16	28962755	G	A	-0.02247	0.003154	1.10E-12	0.359884	462346	NA	rs1244927	NA	UKB-b-57	TRUE	reported	8KoqAI
16	28963012	T	C	-0.02266	0.003152	6.50E-13	0.360771	462346	NA	rs6203742	NA	UKB-b-57	TRUE	reported	8KoqAI
16	28964716	A	G	-0.02204	0.003157	3.00E-12	0.358589	462346	NA	rs6203742	NA	UKB-b-57	TRUE	reported	8KoqAI
16	28969480	A	G	-0.02128	0.00314	1.20E-11	0.368166	462346	NA	rs9937676	NA	UKB-b-57	TRUE	reported	8KoqAI
16	28973268	A	G	-0.02854	0.003309	6.30E-18	0.698151	462346	NA	rs1164329	NA	UKB-b-57	TRUE	reported	8KoqAI
16	28974240	C	A	-0.02182	0.003157	4.80E-12	0.358586	462346	NA	rs1164272	NA	UKB-b-57	TRUE	reported	8KoqAI
16	28977020	G	A	-0.02856	0.003309	6.10E-18	0.698429	462346	NA	rs7500321	NA	UKB-b-57	TRUE	reported	8KoqAI
16	28977713	T	C	-0.02194	0.003157	3.60E-12	0.358723	462346	NA	rs1137843	NA	UKB-b-57	TRUE	reported	8KoqAI
16	28980531	G	A	-0.02184	0.003157	4.60E-12	0.358578	462346	NA	rs1115067	NA	UKB-b-57	TRUE	reported	8KoqAI
16	28980693	T	G	-0.02186	0.003157	4.40E-12	0.358603	462346	NA	rs4788111	NA	UKB-b-57	TRUE	reported	8KoqAI
16	28981511	G	C	-0.02848	0.003308	7.40E-18	0.698079	462346	NA	rs7192056	NA	UKB-b-57	TRUE	reported	8KoqAI
16	28983936	G	C	-0.02853	0.003309	6.50E-18	0.698454	462346	NA	rs1164530	NA	UKB-b-57	TRUE	reported	8KoqAI
16	28984945	A	C	-0.02185	0.003155	4.40E-12	0.358661	462346	NA	rs1164263	NA	UKB-b-57	TRUE	reported	8KoqAI
16	28987518	G	A	-0.0218	0.003156	4.90E-12	0.358572	462346	NA	rs4788112	NA	UKB-b-57	TRUE	reported	8KoqAI
16	28988269	C	T	-0.02851	0.003308	6.70E-18	0.69813	462346	NA	rs8045689	NA	UKB-b-57	TRUE	reported	8KoqAI
16	28990101	C	T	-0.02852	0.003308	6.60E-18	0.698125	462346	NA	rs7187575	NA	UKB-b-57	TRUE	reported	8KoqAI
16	28992000	G	A	-0.02182	0.003156	4.80E-12	0.358459	462346	NA	rs4788113	NA	UKB-b-57	TRUE	reported	8KoqAI
16	28992646	G	A	-0.02122	0.003138	1.40E-11	0.368044	462346	NA	rs3922668	NA	UKB-b-57	TRUE	reported	8KoqAI
16	28993049	A	C	-0.0286	0.003308	5.40E-18	0.698436	462346	NA	rs6565300	NA	UKB-b-57	TRUE	reported	8KoqAI
16	28995757	C	A	-0.02875	0.00331	3.80E-18	0.698299	462346	NA	rs7140	NA	UKB-b-57	TRUE	reported	8KoqAI
16	29000446	G	A	-0.02211	0.003166	2.90E-12	0.358453	462346	NA	rs1156167	NA	UKB-b-57	TRUE	reported	8KoqAI
16	29001460	A	C	-0.02846	0.003275	3.60E-18	0.690566	462346	NA	rs9929088	NA	UKB-b-57	TRUE	reported	8KoqAI
16	29002706	G	A	-0.02206	0.003169	3.40E-12	0.358995	462346	NA	rs6203742	NA	UKB-b-57	TRUE	reported	8KoqAI
16	29007489	C	T	-0.02022	0.003202	2.70E-10	0.365976	462346	NA	rs1118506	NA	UKB-b-57	TRUE	reported	8KoqAI
16	29008079	C	T	-0.02118	0.003195	3.40E-11	0.360143	462346	NA	rs6203742	NA	UKB-b-57	TRUE	reported	8KoqAI
16	29011438	A	C	-0.02426	0.00386	3.30E-10	0.804119	462346	NA	rs4788116	NA	UKB-b-57	TRUE	reported	8KoqAI
16	29011464	T	C	-0.02466	0.003854	1.60E-10	0.803275	462346	NA	rs4788117	NA	UKB-b-57	TRUE	reported	8KoqAI
16	29012393	T	C	-0.02407	0.003856	4.30E-10	0.804138	462346	NA	rs8057063	NA	UKB-b-57	TRUE	reported	8KoqAI
16	29014309	T	A	-0.02415	0.003848	3.50E-10	0.803988	462346	NA	rs1210294	NA	UKB-b-57	TRUE	reported	8KoqAI
16	29015149	C	A	-0.02348	0.003869	1.30E-09	0.807503	462346	NA	rs9929456	NA	UKB-b-57	TRUE	reported	8KoqAI
16	29018610	A	G	-0.02278	0.003845	3.10E-09	0.807081	462346	NA	rs7186784	NA	UKB-b-57	TRUE	reported	8KoqAI
16	29019356	G	T	-0.02275	0.003837	3.00E-09	0.806764	462346	NA	rs4788119	NA	UKB-b-57	TRUE	reported	8KoqAI
16	29820480	C	T	0.025008	0.004296	5.80E-09	0.145155	462346	NA	rs3428659	NA	UKB-b-57	TRUE	reported	8KoqAI
16	29826365	G	T	0.02471	0.00429	8.40E-09	0.14565	462346	NA	rs1045968	NA	UKB-b-57	TRUE	reported	8KoqAI
16	29826538	T	C	-0.02487	0.004275	6.00E-09	0.853093	462346	NA	rs10204	NA	UKB-b-57	TRUE	reported	8KoqAI
16	29828710	C	G	0.024494	0.004289	1.10E-08	0.145767	462346	NA	rs8058824	NA	UKB-b-57	TRUE	reported	8KoqAI
16	29829615	T	C	0.024697	0.00429	8.50E-09	0.145669	462346	NA	rs3590155	NA	UKB-b-57	TRUE	reported	8KoqAI
16	29830829	T	C	0.024818	0.004288	7.10E-09	0.145856	462346	NA	rs1186418	NA	UKB-b-57	TRUE	reported	8KoqAI
16	29832138	G	A	0.024744	0.004289	8.00E-09	0.145686	462346	NA	rs9923649	NA	UKB-b-57	TRUE	reported	8KoqAI
16	29833414	T	G	0.024875	0.004288	6.60E-09	0.146057	462346	NA	rs8061634	NA	UKB-b-57	TRUE	reported	8KoqAI
16	29833488	G	T	0.024982	0.004276	5.10E-09	0.146806	462346	NA	rs1057451	NA	UKB-b-57	TRUE	reported	8KoqAI
16	29833714	G	A	0.025042	0.004293	5.50E-09	0.145394	462346	NA	rs1057452	NA	UKB-b-57	TRUE	reported	8KoqAI
16	29834954	C	T	0.024677	0.00429	8.80E-09	0.145669	462346	NA	rs6205637	NA	UKB-b-57	TRUE	reported	8KoqAI
16	29835440	A	G	0.025002	0.004274	4.90E-09	0.146927	462346	NA	rs1185987	NA	UKB-b-57	TRUE	reported	8KoqAI
16	29835588	G	C	0.024681	0.004289	8.70E-09	0.145696	462346	NA	rs1186374	NA	UKB-b-57	TRUE	reported	8KoqAI
16	29837108	C	T	0.024745	0.004316	9.90E-09	0.144222	462346	NA	rs9938630	NA	UKB-b-57	TRUE	reported	8KoqAI
16	29839449	T	C	0.024358	0.004316	1.70E-08	0.143734	462346	NA	rs9921783	NA	UKB-b-57	TRUE	reported	8KoqAI
16	29839893	G	C	0.024338	0.004316	1.70E-08	0.14373	462346	NA	rs4788185	NA	UKB-b-57	TRUE	reported	8KoqAI
16	29842547	G	A	0.024333	0.004317	1.70E-08	0.143601	462346	NA	rs3471440	NA	UKB-b-57	TRUE	reported	8KoqAI
16	29842632	G	A	0.02441	0.004316	1.60E-08	0.143621	462346	NA	rs7138942	NA	UKB-b-57	TRUE	reported	8KoqAI
16	29842635	C	T	0.024427	0.004317	1.50E-08	0.143599	462346	NA	rs7138942	NA	UKB-b-57	TRUE	reported	8KoqAI
16	29842891	C	T	0.024428	0.004317	1.50E-08	0.143622	462346	NA	rs3412722	NA	UKB-b-57	TRUE	reported	8KoqAI
16	29844279	G	A	0.024385	0.004316	1.60E-08	0.143647	462346	NA	rs1292076	NA	UKB-b-57	TRUE	reported	8KoqAI
16	29845685	T	C	0.024612	0.004297	1.00E-08	0.144964	462346	NA	rs4788187	NA	UKB-b-57	TRUE	reported	8KoqAI
16	29846729	C	A	0.024555	0.004318	1.30E-08	0.143416	462346	NA	rs1293269	NA	UKB-b-57	TRUE	reported	8KoqAI
16	29848417	A	G	0.024284	0.004313	1.80E-08	0.143749	462346	NA	rs1333173	NA	UKB-b-57	TRUE	reported	8KoqAI
16	29848932	A	G	0.024246	0.004317	1.90E-08	0.143697	462346	NA	rs1186373	NA	UKB-b-57	TRUE	reported	8KoqAI
16	29848946	A	G	0.02445	0.004301	1.30E-08	0.144954	462346	NA	rs1186373	NA	UKB-b-57	TRUE	reported	8KoqAI

16	29850705	C	T	0.023903	0.004318	3.10E-08	0.144013	462346	NA	rs1333207	NA	UKB-b-57	TRUE	reported	8KoqAI
16	29852860	C	T	0.023919	0.004289	2.40E-08	0.14566	462346	NA	rs3815824	NA	UKB-b-57	TRUE	reported	8KoqAI
16	29855731	G	A	0.024248	0.004285	1.50E-08	0.145946	462346	NA	rs9939514	NA	UKB-b-57	TRUE	reported	8KoqAI
16	29857316	G	A	0.024147	0.004285	1.70E-08	0.145932	462346	NA	rs4787480	NA	UKB-b-57	TRUE	reported	8KoqAI
16	29857901	G	A	0.024503	0.004289	1.10E-08	0.145621	462346	NA	rs6205637	NA	UKB-b-57	TRUE	reported	8KoqAI
16	29859627	C	T	0.024244	0.004287	1.60E-08	0.145799	462346	NA	rs3422285	NA	UKB-b-57	TRUE	reported	8KoqAI
16	29859977	A	G	0.024263	0.004285	1.50E-08	0.145941	462346	NA	rs7201519	NA	UKB-b-57	TRUE	reported	8KoqAI
16	29860685	A	C	0.024351	0.004285	1.30E-08	0.145921	462346	NA	rs2859968	NA	UKB-b-57	TRUE	reported	8KoqAI
16	29860863	G	A	0.024625	0.004272	8.20E-09	0.147049	462346	NA	rs4788188	NA	UKB-b-57	TRUE	reported	8KoqAI
16	29861447	G	A	0.024284	0.004287	1.50E-08	0.145947	462346	NA	rs3976455	NA	UKB-b-57	TRUE	reported	8KoqAI
16	29865673	G	A	0.024345	0.0043	1.50E-08	0.145781	462346	NA	rs9932653	NA	UKB-b-57	TRUE	reported	8KoqAI
16	29869176	C	T	0.02311	0.003412	1.30E-11	0.281659	462346	NA	rs3424872	NA	UKB-b-57	TRUE	reported	8KoqAI
16	29874935	T	C	0.022443	0.003366	2.60E-11	0.292562	462346	NA	rs889695	NA	UKB-b-57	TRUE	reported	8KoqAI
16	29875241	A	G	0.022339	0.003366	3.20E-11	0.292878	462346	NA	rs747973	NA	UKB-b-57	TRUE	reported	8KoqAI
16	29878161	A	T	0.023107	0.003274	1.70E-12	0.319414	462346	NA	rs4474709	NA	UKB-b-57	TRUE	reported	8KoqAI
16	29878249	G	T	0.022941	0.003277	2.60E-12	0.318092	462346	NA	rs4787482	NA	UKB-b-57	TRUE	reported	8KoqAI
16	29878468	T	C	0.02327	0.003275	1.20E-12	0.319295	462346	NA	rs7198022	NA	UKB-b-57	TRUE	reported	8KoqAI
16	29880874	C	T	0.022979	0.003272	2.20E-12	0.317821	462346	NA	rs3533097	NA	UKB-b-57	TRUE	reported	8KoqAI
16	29881482	A	G	0.023312	0.003268	9.80E-13	0.319341	462346	NA	rs6044788	NA	UKB-b-57	TRUE	reported	8KoqAI
16	29882490	C	T	0.022816	0.003238	1.80E-12	0.321591	462346	NA	rs1291771	NA	UKB-b-57	TRUE	reported	8KoqAI
16	29885447	A	G	0.024106	0.003202	5.10E-14	0.336368	462346	NA	rs4787483	NA	UKB-b-57	TRUE	reported	8KoqAI
16	29885698	A	G	-0.02048	0.00308	2.90E-11	0.438537	462346	NA	rs1164404	NA	UKB-b-57	TRUE	reported	8KoqAI
16	29887215	T	C	0.024216	0.003204	4.10E-14	0.336317	462346	NA	rs6681872	NA	UKB-b-57	TRUE	reported	8KoqAI
16	29892184	G	A	0.027448	0.003287	6.80E-17	0.306959	462346	NA	rs1134437	NA	UKB-b-57	TRUE	reported	8KoqAI
16	29895303	G	C	0.02386	0.003177	6.00E-14	0.347605	462346	NA	rs1291961	NA	UKB-b-57	TRUE	reported	8KoqAI
16	29896390	G	A	0.024109	0.00318	3.40E-14	0.34723	462346	NA	rs8051833	NA	UKB-b-57	TRUE	reported	8KoqAI
16	29902414	C	T	0.024585	0.003183	1.10E-14	0.345649	462346	NA	rs7359380	NA	UKB-b-57	TRUE	reported	8KoqAI
16	29906546	C	T	0.02466	0.003181	9.00E-15	0.345976	462346	NA	rs4787484	NA	UKB-b-57	TRUE	reported	8KoqAI
16	29908777	C	G	0.024289	0.003176	2.00E-14	0.349077	462346	NA	rs1293023	NA	UKB-b-57	TRUE	reported	8KoqAI
16	29910518	C	G	0.024039	0.003176	3.80E-14	0.348715	462346	NA	rs11344	NA	UKB-b-57	TRUE	reported	8KoqAI
16	29910725	A	G	0.024213	0.003174	2.40E-14	0.349465	462346	NA	rs7498372	NA	UKB-b-57	TRUE	reported	8KoqAI
16	29911260	G	A	0.024078	0.003176	3.40E-14	0.348495	462346	NA	rs7189550	NA	UKB-b-57	TRUE	reported	8KoqAI
16	29911416	G	A	0.024279	0.003176	2.10E-14	0.348505	462346	NA	rs7189750	NA	UKB-b-57	TRUE	reported	8KoqAI
16	29924422	A	T	0.0174	0.003038	1.00E-08	0.548586	462346	NA	rs1244497	NA	UKB-b-57	TRUE	reported	8KoqAI
16	29924905	A	G	0.016859	0.003045	3.10E-08	0.557487	462346	NA	rs7201384	NA	UKB-b-57	TRUE	reported	8KoqAI
16	29925445	C	T	0.017817	0.003037	4.50E-09	0.547113	462346	NA	rs8059619	NA	UKB-b-57	TRUE	reported	8KoqAI
16	29927702	G	A	0.017433	0.003038	9.60E-09	0.548543	462346	NA	rs1008373	NA	UKB-b-57	TRUE	reported	8KoqAI
16	29929728	C	T	0.017451	0.003038	9.20E-09	0.548173	462346	NA	rs8052502	NA	UKB-b-57	TRUE	reported	8KoqAI
16	29931899	G	A	0.017539	0.003038	7.80E-09	0.548302	462346	NA	rs4407079	NA	UKB-b-57	TRUE	reported	8KoqAI
16	29932064	C	T	0.017569	0.003038	7.40E-09	0.548881	462346	NA	rs4609871	NA	UKB-b-57	TRUE	reported	8KoqAI
16	29932691	T	C	0.01753	0.003038	7.90E-09	0.548709	462346	NA	rs9936474	NA	UKB-b-57	TRUE	reported	8KoqAI
16	29934050	A	G	0.016906	0.003052	3.00E-08	0.554981	462346	NA	rs1293240	NA	UKB-b-57	TRUE	reported	8KoqAI
16	29934754	T	A	0.017542	0.003038	7.80E-09	0.54879	462346	NA	rs1259604	NA	UKB-b-57	TRUE	reported	8KoqAI
16	29934819	C	T	0.017549	0.003038	7.60E-09	0.548746	462346	NA	rs1115057	NA	UKB-b-57	TRUE	reported	8KoqAI
16	29935066	G	C	0.016816	0.003044	3.30E-08	0.5566	462346	NA	rs1115057	NA	UKB-b-57	TRUE	reported	8KoqAI
16	29936654	A	G	0.01691	0.003046	2.80E-08	0.558199	462346	NA	rs1271697	NA	UKB-b-57	TRUE	reported	8KoqAI
16	29937652	G	A	0.016945	0.003045	2.60E-08	0.556978	462346	NA	rs1271697	NA	UKB-b-57	TRUE	reported	8KoqAI
16	29938820	A	G	0.017209	0.003046	1.60E-08	0.558505	462346	NA	rs4420550	NA	UKB-b-57	TRUE	reported	8KoqAI
16	29939293	T	C	0.017176	0.003047	1.70E-08	0.558702	462346	NA	rs4424923	NA	UKB-b-57	TRUE	reported	8KoqAI
16	29939877	A	G	0.017355	0.003047	1.20E-08	0.558548	462346	NA	rs1269130	NA	UKB-b-57	TRUE	reported	8KoqAI
16	29943459	C	A	0.017872	0.003039	4.10E-09	0.549513	462346	NA	rs1293349	NA	UKB-b-57	TRUE	reported	8KoqAI
16	29944832	G	A	0.017928	0.003042	3.80E-09	0.548964	462346	NA	rs1271697	NA	UKB-b-57	TRUE	reported	8KoqAI
16	29946670	T	C	0.017868	0.00304	4.20E-09	0.549501	462346	NA	rs1164961	NA	UKB-b-57	TRUE	reported	8KoqAI
16	29946708	G	A	0.017943	0.00304	3.60E-09	0.549504	462346	NA	rs1164293	NA	UKB-b-57	TRUE	reported	8KoqAI
16	29946945	T	C	0.017976	0.003041	3.40E-09	0.550161	462346	NA	rs4527039	NA	UKB-b-57	TRUE	reported	8KoqAI
16	29948401	G	A	0.017867	0.00304	4.20E-09	0.551263	462346	NA	rs4788190	NA	UKB-b-57	TRUE	reported	8KoqAI
16	29950829	C	T	0.017799	0.003041	4.80E-09	0.550348	462346	NA	rs1271697	NA	UKB-b-57	TRUE	reported	8KoqAI
16	29954654	T	G	0.017985	0.003043	3.40E-09	0.551312	462346	NA	rs4402589	NA	UKB-b-57	TRUE	reported	8KoqAI
16	29954955	A	G	0.017874	0.003043	4.30E-09	0.551312	462346	NA	rs3924855	NA	UKB-b-57	TRUE	reported	8KoqAI
16	29958216	G	A	0.021418	0.003032	1.60E-12	0.467152	462346	NA	rs8054556	NA	UKB-b-57	TRUE	reported	8KoqAI
16	29960468	G	A	0.021914	0.003037	5.40E-13	0.4561	462346	NA	rs8056535	NA	UKB-b-57	TRUE	reported	8KoqAI
16	29960593	T	C	0.021915	0.003037	5.40E-13	0.45611	462346	NA	rs4788195	NA	UKB-b-57	TRUE	reported	8KoqAI
16	29962352	A	G	0.021962	0.003037	4.70E-13	0.456279	462346	NA	rs7190185	NA	UKB-b-57	TRUE	reported	8KoqAI
16	29962802	G	A	0.021909	0.003037	5.40E-13	0.455707	462346	NA	rs7193532	NA	UKB-b-57	TRUE	reported	8KoqAI
16	29964357	T	C	0.021932	0.003036	5.00E-13	0.456378	462346	NA	rs1291865	NA	UKB-b-57	TRUE	reported	8KoqAI
16	29965496	C	T	0.021807	0.003036	6.90E-13	0.455785	462346	NA	rs1115057	NA	UKB-b-57	TRUE	reported	8KoqAI
16	29966277	G	C	0.021976	0.003035	4.50E-13	0.456447	462346	NA	rs1164612	NA	UKB-b-57	TRUE	reported	8KoqAI
16	29967434	A	G	0.021979	0.003036	4.50E-13	0.456414	462346	NA	rs4788196	NA	UKB-b-57	TRUE	reported	8KoqAI
16	29968015	C	T	0.022063	0.003036	3.70E-13	0.456006	462346	NA	rs7204797	NA	UKB-b-57	TRUE	reported	8KoqAI
16	29970834	G	A	0.02203	0.003037	4.00E-13	0.456079	462346	NA	rs4788197	NA	UKB-b-57	TRUE	reported	8KoqAI
16	29970857	C	G	0.018298	0.003032	1.60E-09	0.523799	462346	NA	rs4788199	NA	UKB-b-57	TRUE	reported	8KoqAI
16	29971265	G	T	0.021957	0.003037	4.80E-13	0.455973	462346	NA	rs4788200	NA	UKB-b-57	TRUE	reported	8KoqAI
16	29972690	C	T	0.021966	0.003037	4.70E-13	0.455963	462346	NA	rs4788201	NA	UKB-b-57	TRUE	reported	8KoqAI
16	29974167	G	A	0.022014	0.003037	4.20E-13	0.455914	462346	NA	rs6565173	NA	UKB-b-57	TRUE	reported	8KoqAI
16	29977562	A	T	0.022169	0.003037	2.90E-13	0.45599	462346	NA	rs4787486	NA	UKB-b-57	TRUE	reported	8KoqAI
16	29978003	C	T	0.022193	0.003037	2.70E-13	0.455978	462346	NA	rs4788202	NA	UKB-b-57	TRUE	reported	8KoqAI
16	29978827	G	A	0.020044	0.003064	6.10E-11	0.433685	462346	NA	rs4788203	NA	UKB-b-57	TRUE	reported	8KoqAI
16	29982961	C	T	0.022364	0.003036	1.80E-13	0.455762	462346	NA	rs9932702	NA	UKB-b-57	TRUE	reported	8KoqAI
16	29983119	G	T	0.022353	0.003036	1.80E-13	0.45597	462346	NA	rs9932196	NA	UKB-b-57	TRUE	reported	8KoqAI
16	29983148	T	C	0.022352	0.003036	1.80E-13	0.455747	462346	NA	rs9925102	NA	UKB-b-57	TRUE	reported	8KoqAI
16	29984070	C	G	0.022243	0.003036	2.40E-13	0.45555	462346	NA	rs11901	NA	UKB-b-57	TRUE	reported	8KoqAI
16	29984839	C	G	0.02221	0.003036	2.60E-13	0.455535	462346	NA	rs4318227	NA	UKB-b-57	TRUE	reported	8KoqAI
16	29986205	C	G	0.022168	0.003035	2.80E-13	0.45607	462346	NA	rs1087145	NA	UKB-b-57	TRUE	reported	8KoqAI
16	29986525	G	A	0.022248	0.003035	2.30E-13	0.455423	462346	NA	rs1115057	NA	UKB-b-57	TRUE	reported	8KoqAI
16	29988349	G	A	0.022291	0.003035	2.10E-13	0.455962	462346	NA	rs4283241	NA	UKB-b-57	TRUE	reported	8KoqAI
16	29988941	A	G	0.022191	0.003036	2.70E-13	0.455312	462346	NA	rs4583255	NA	UKB-b-57	TRUE	reported	8KoqAI
16	29993108	C	G	0.022128	0.003036	3.10E-13	0.456057	462346	NA	rs1164204	NA	UKB-b-57	TRUE	reported	8KoqAI
16	29993686	G	C	0.022171	0.003035	2.80E-13	0.45601	462346	NA	rs9925915	NA	UKB-b-57	TRUE	reported	8KoqAI
16	29994736	T	A	0.022275	0.003035	2.20E-13	0.455431	462346	NA	rs3814884	NA	UKB-b-57	TRUE	reported	8KoqAI
16	29994922	C	T	0.020791	0.003039	7.80E-12	0.4824	462346	NA	rs3814883	NA	UKB-b-57	TRUE	reported	8KoqAI
16	29995218	G	A	0.022229	0.003036	2.40E-13	0.455879	462346	NA	rs4788204	NA	UKB-b-57	TRUE	reported	8KoqAI
16	29995803	A	C	0.018386	0.003029	1.30E-09	0.523536	462346	NA	rs4787488	NA	UKB-b-57	TRUE	reported	8KoqAI
16	29995880	G	A	0.022275	0.003036	2.20E-13	0.455577	462346	NA	rs4787489	NA	UKB-b-57	TRUE	reported	8KoqAI
16	29998200	A	G	0.018453	0.003028	1.10E-09	0.523714	462346	NA	rs4077410	NA	UKB-b-57	TRUE	reported	8KoqAI
16	30000330	T	G	0.022289	0.003036	2.10E-13	0.455759	462346	NA	rs1115057	NA	UKB-b-57	TRUE	reported	8KoqAI
16	30000901	G	A	0.022266	0.003036	2.20E-13	0.455753	462346	NA	rs3814881	NA	UKB-b-57	TRUE	reported	8KoqAI
16	30003076	G	A	0.022286	0.003035	2.10E-13	0.455569	462346	NA	rs9924686	NA	UKB-b-57	TRUE	reported	8KoqAI
16	30008644	C	T	0.018351	0.003028	1.40E-09	0.52353	462346	NA	rs1164914	NA	UKB-b-57	TRUE	reported	8KoqAI
16	30011618	C	T	0.022326	0.003036	1.90E-13	0.455551	462346	NA	rs1292199	NA	UKB-b-57	TRUE	reported	8KoqAI
16	30013066	A	G	0.018425	0.00303	1.20E-09	0.524024	462346	NA	rs1293195	NA	UKB-b-57	TRUE	reported	8KoqAI
16	30013266	C	T	0.022227	0.003036	2.50E-13	0.455613	462346	NA	rs4788209	NA	UKB-b-57	TRUE	reported	8KoqAI
16	30013674	C	G	0.022309	0.003036	2.00E-13	0.455636	462346	NA	rs4788210	NA	UKB-b-57	TRUE	reported	8KoqAI
16	30014564	C	T	0.022256	0.003036	2.30E-13	0.455794	462346	NA	rs1115058	NA	UKB-b-57	TRUE	reported	8KoqAI
16	30015337	A	G	0.018076	0.003034	2.60E-09	0.535147	462346	NA	rs4787491	NA	UKB-b-57	TRUE	reported	8KoqAI
16	30015565	C	T	0.018431	0.00303	1.20E-09	0.523819	462346	NA	rs4788211	NA	UKB-b-57	TRUE	reported	8KoqAI
16	30016022	C	T	0.018685	0.003032	7.20E-10	0.520786	462346	NA	rs1292861	NA	UKB-b-57	TRUE	reported	8KoqAI
16	30017895	T	C	0.022325	0.003036	1.90E-13	0.455861	462346	NA	rs3814880	NA	UKB-b-57	TRUE	reported	8KoqAI

16	30018500	C	T	0.022223	0.003037	2.50E-13	0.455629	462346	NA	rs3935873	NA	UKB-b-57	TRUE	reported	8KoqAI
16	30018720	C	T	0.025889	0.00309	5.40E-17	0.399438	462346	NA	rs1292175	NA	UKB-b-57	TRUE	reported	8KoqAI
16	30018982	C	G	0.018327	0.003035	1.60E-09	0.521904	462346	NA	rs9972866	NA	UKB-b-57	TRUE	reported	8KoqAI
16	30019375	A	C	0.018263	0.00303	1.70E-09	0.52395	462346	NA	rs1186317	NA	UKB-b-57	TRUE	reported	8KoqAI
16	30022564	A	G	0.021165	0.003034	3.10E-12	0.468655	462346	NA	rs1293357	NA	UKB-b-57	TRUE	reported	8KoqAI
16	30023157	T	C	0.021232	0.003034	2.60E-12	0.468643	462346	NA	rs7191849	NA	UKB-b-57	TRUE	reported	8KoqAI
16	30023786	C	G	0.025937	0.003086	4.30E-17	0.40057	462346	NA	rs1232540	NA	UKB-b-57	TRUE	reported	8KoqAI
16	30026469	G	A	0.025963	0.003087	4.10E-17	0.401036	462346	NA	rs1164927	NA	UKB-b-57	TRUE	reported	8KoqAI
16	30026748	C	T	0.025955	0.003087	4.20E-17	0.401176	462346	NA	rs1293570	NA	UKB-b-57	TRUE	reported	8KoqAI
16	30027694	G	A	0.026024	0.003088	3.50E-17	0.401792	462346	NA	rs1244410	NA	UKB-b-57	TRUE	reported	8KoqAI
16	30029135	A	G	0.021299	0.003034	2.20E-12	0.468902	462346	NA	rs1259654	NA	UKB-b-57	TRUE	reported	8KoqAI
16	30029548	C	T	0.025747	0.003087	7.50E-17	0.400823	462346	NA	rs1259885	NA	UKB-b-57	TRUE	reported	8KoqAI
16	30030195	A	C	0.025951	0.003086	4.20E-17	0.401242	462346	NA	rs1164261	NA	UKB-b-57	TRUE	reported	8KoqAI
16	30030699	T	C	0.025973	0.003086	3.90E-17	0.401237	462346	NA	rs1115058	NA	UKB-b-57	TRUE	reported	8KoqAI
16	30033428	A	G	0.02148	0.003034	1.40E-12	0.468148	462346	NA	rs3560501	NA	UKB-b-57	TRUE	reported	8KoqAI
16	30033527	G	C	0.022272	0.003172	2.20E-12	0.640835	462346	NA	rs7193065	NA	UKB-b-57	TRUE	reported	8KoqAI
16	30033633	T	C	0.026061	0.003086	3.00E-17	0.401196	462346	NA	rs1232553	NA	UKB-b-57	TRUE	reported	8KoqAI
16	30034189	A	G	0.021385	0.003033	1.80E-12	0.467981	462346	NA	rs1115058	NA	UKB-b-57	TRUE	reported	8KoqAI
16	30034469	T	G	0.021395	0.003033	1.70E-12	0.46798	462346	NA	rs4788212	NA	UKB-b-57	TRUE	reported	8KoqAI
16	30034524	T	C	0.021388	0.003033	1.80E-12	0.46798	462346	NA	rs4788213	NA	UKB-b-57	TRUE	reported	8KoqAI
16	30037128	A	C	0.021362	0.003033	1.90E-12	0.467643	462346	NA	rs1154432	NA	UKB-b-57	TRUE	reported	8KoqAI
16	30039678	C	A	0.02111	0.003036	3.50E-12	0.465539	462346	NA	rs1115058	NA	UKB-b-57	TRUE	reported	8KoqAI
16	30040178	C	G	0.021415	0.003033	1.70E-12	0.467669	462346	NA	rs7204852	NA	UKB-b-57	TRUE	reported	8KoqAI
16	30041966	A	G	0.021574	0.003033	1.10E-12	0.468591	462346	NA	rs3814878	NA	UKB-b-57	TRUE	reported	8KoqAI
16	30042677	G	T	0.026119	0.003086	2.60E-17	0.401624	462346	NA	rs3814877	NA	UKB-b-57	TRUE	reported	8KoqAI
16	30043110	T	G	0.021415	0.003033	1.70E-12	0.467661	462346	NA	rs1244637	NA	UKB-b-57	TRUE	reported	8KoqAI
16	30044581	G	A	0.021412	0.003033	1.70E-12	0.46765	462346	NA	rs8043883	NA	UKB-b-57	TRUE	reported	8KoqAI
16	30045789	C	G	0.0261	0.003086	2.70E-17	0.401091	462346	NA	rs1164243	NA	UKB-b-57	TRUE	reported	8KoqAI
16	30046042	G	A	0.021357	0.003033	1.90E-12	0.467601	462346	NA	rs8054507	NA	UKB-b-57	TRUE	reported	8KoqAI
16	30047930	G	A	0.021532	0.003033	1.30E-12	0.467847	462346	NA	rs4788215	NA	UKB-b-57	TRUE	reported	8KoqAI
16	30048077	C	G	0.021514	0.003033	1.30E-12	0.467751	462346	NA	rs4788216	NA	UKB-b-57	TRUE	reported	8KoqAI
16	30048553	C	T	0.026058	0.003088	3.20E-17	0.400556	462346	NA	rs3546792	NA	UKB-b-57	TRUE	reported	8KoqAI
16	30050200	A	C	0.02105	0.003197	4.60E-11	0.649935	462346	NA	rs7190269	NA	UKB-b-57	TRUE	reported	8KoqAI
16	30050494	G	A	0.021605	0.003036	1.10E-12	0.467519	462346	NA	rs6646744	NA	UKB-b-57	TRUE	reported	8KoqAI
16	30050505	C	T	0.021609	0.003034	1.10E-12	0.467837	462346	NA	rs6721815	NA	UKB-b-57	TRUE	reported	8KoqAI
16	30051312	A	G	0.021607	0.003034	1.10E-12	0.467788	462346	NA	rs4788217	NA	UKB-b-57	TRUE	reported	8KoqAI
16	30055549	T	C	0.021778	0.003035	7.20E-13	0.46792	462346	NA	rs4787492	NA	UKB-b-57	TRUE	reported	8KoqAI
16	30055750	T	C	0.026165	0.00309	2.50E-17	0.40073	462346	NA	rs4788218	NA	UKB-b-57	TRUE	reported	8KoqAI
16	30057148	C	T	0.026345	0.003088	1.40E-17	0.401541	462346	NA	rs3510514	NA	UKB-b-57	TRUE	reported	8KoqAI
16	30059593	T	G	0.021853	0.003035	6.00E-13	0.467757	462346	NA	rs1164239	NA	UKB-b-57	TRUE	reported	8KoqAI
16	30060655	A	G	0.026084	0.00309	3.10E-17	0.401023	462346	NA	rs1164274	NA	UKB-b-57	TRUE	reported	8KoqAI
16	30063646	A	G	0.021853	0.003035	6.00E-13	0.467486	462346	NA	rs4788219	NA	UKB-b-57	TRUE	reported	8KoqAI
16	30063780	A	G	0.021845	0.003035	6.10E-13	0.467481	462346	NA	rs4788220	NA	UKB-b-57	TRUE	reported	8KoqAI
16	30068354	C	T	0.026189	0.003085	2.10E-17	0.403426	462346	NA	rs9939774	NA	UKB-b-57	TRUE	reported	8KoqAI
16	30068469	T	G	0.021807	0.003033	6.50E-13	0.469579	462346	NA	rs9932193	NA	UKB-b-57	TRUE	reported	8KoqAI
16	30072530	T	C	0.021824	0.003032	6.20E-13	0.469539	462346	NA	rs9928448	NA	UKB-b-57	TRUE	reported	8KoqAI
16	30078492	G	C	0.021711	0.003031	7.80E-13	0.501981	462346	NA	rs9783783	NA	UKB-b-57	TRUE	reported	8KoqAI
16	30082508	G	T	0.021829	0.003029	5.70E-13	0.480133	462346	NA	rs7201518	NA	UKB-b-57	TRUE	reported	8KoqAI
16	30089908	C	T	0.021918	0.003033	4.90E-13	0.469417	462346	NA	rs1244791	NA	UKB-b-57	TRUE	reported	8KoqAI
16	30089911	T	C	0.021841	0.003033	5.90E-13	0.469435	462346	NA	rs1244576	NA	UKB-b-57	TRUE	reported	8KoqAI
16	30092048	A	G	0.021853	0.003029	5.40E-13	0.480255	462346	NA	rs9932605	NA	UKB-b-57	TRUE	reported	8KoqAI
16	30093779	C	G	0.026176	0.003084	2.10E-17	0.403179	462346	NA	rs2278557	NA	UKB-b-57	TRUE	reported	8KoqAI
16	30095236	C	T	0.021742	0.003033	7.60E-13	0.469451	462346	NA	rs1164445	NA	UKB-b-57	TRUE	reported	8KoqAI
16	30101596	C	A	0.021943	0.003029	4.40E-13	0.479456	462346	NA	rs8060511	NA	UKB-b-57	TRUE	reported	8KoqAI
16	30107285	A	T	0.021454	0.003031	1.50E-12	0.479487	462346	NA	rs1244497	NA	UKB-b-57	TRUE	reported	8KoqAI
16	30107781	A	G	0.021455	0.003031	1.50E-12	0.477866	462346	NA	rs7353017	NA	UKB-b-57	TRUE	reported	8KoqAI
16	30110467	T	C	0.021338	0.003031	1.90E-12	0.477572	462346	NA	rs9922041	NA	UKB-b-57	TRUE	reported	8KoqAI
16	30114124	C	T	0.021068	0.003029	3.50E-12	0.477339	462346	NA	rs7205802	NA	UKB-b-57	TRUE	reported	8KoqAI
16	30117253	G	A	0.024969	0.003096	7.30E-16	0.399994	462346	NA	rs6205775	NA	UKB-b-57	TRUE	reported	8KoqAI
16	30118345	T	C	0.021401	0.003036	1.80E-12	0.478014	462346	NA	rs1244441	NA	UKB-b-57	TRUE	reported	8KoqAI
16	30120372	C	T	-0.01847	0.003268	1.60E-08	0.349517	462346	NA	rs5607992	NA	UKB-b-57	TRUE	reported	8KoqAI
16	30125840	G	C	-0.01925	0.003048	2.70E-10	0.442127	462346	NA	rs7542	NA	UKB-b-57	TRUE	reported	8KoqAI
16	30130493	C	A	-0.02144	0.003759	1.20E-08	0.310558	462346	NA	rs1186508	NA	UKB-b-57	TRUE	reported	8KoqAI
16	30147265	C	T	-0.0172	0.003048	1.70E-08	0.444067	462346	NA	rs7201780	NA	UKB-b-57	TRUE	reported	8KoqAI
16	30164488	A	G	-0.01654	0.003024	4.50E-08	0.505169	462346	NA	rs1186571	NA	UKB-b-57	TRUE	reported	8KoqAI
16	30169393	C	G	-0.01658	0.003025	4.30E-08	0.514165	462346	NA	rs1292719	NA	UKB-b-57	TRUE	reported	8KoqAI
16	30172627	A	G	-0.01649	0.003025	5.00E-08	0.514091	462346	NA	rs1333181	NA	UKB-b-57	TRUE	reported	8KoqAI
16	30174926	T	C	-0.01653	0.003024	4.50E-08	0.513921	462346	NA	rs6565176	NA	UKB-b-57	TRUE	reported	8KoqAI
16	30178516	A	C	-0.01668	0.003026	3.50E-08	0.521591	462346	NA	rs1210232	NA	UKB-b-57	TRUE	reported	8KoqAI
16	30182541	T	C	-0.01664	0.003026	3.80E-08	0.521619	462346	NA	rs8057425	NA	UKB-b-57	TRUE	reported	8KoqAI
16	30194840	A	G	-0.01678	0.003029	3.10E-08	0.522759	462346	NA	rs930392	NA	UKB-b-57	TRUE	reported	8KoqAI
16	30198151	A	G	-0.01658	0.003031	4.50E-08	0.519621	462346	NA	rs1132812	NA	UKB-b-57	TRUE	reported	8KoqAI
16	30320307	G	T	0.016548	0.003033	4.90E-08	0.490643	462346	NA	rs4471699	NA	UKB-b-57	TRUE	reported	8KoqAI
16	30323380	G	A	0.016559	0.003033	4.80E-08	0.490664	462346	NA	rs1442089	NA	UKB-b-57	TRUE	reported	8KoqAI
16	30376691	G	A	0.030103	0.004476	1.70E-11	0.131391	462346	NA	rs4500754	NA	UKB-b-57	TRUE	reported	8KoqAI
16	30406168	C	G	0.03254	0.005576	5.30E-09	0.084039	462346	NA	rs2858810	NA	UKB-b-57	TRUE	reported	8KoqAI
16	30448522	C	T	0.029637	0.004403	1.70E-11	0.141059	462346	NA	rs1116888	NA	UKB-b-57	TRUE	reported	8KoqAI
16	30468890	T	C	0.027677	0.004234	6.30E-11	0.152779	462346	NA	rs6757558	NA	UKB-b-57	TRUE	reported	8KoqAI
16	30469957	C	T	0.027633	0.004232	6.60E-11	0.152823	462346	NA	rs7195241	NA	UKB-b-57	TRUE	reported	8KoqAI
16	30474856	G	A	0.028383	0.004346	6.50E-11	0.141221	462346	NA	rs9934579	NA	UKB-b-57	TRUE	reported	8KoqAI
16	30475160	G	A	0.028289	0.004355	8.20E-11	0.140709	462346	NA	rs1115556	NA	UKB-b-57	TRUE	reported	8KoqAI
16	30478891	C	T	0.027768	0.004343	1.60E-10	0.141786	462346	NA	rs1112302	NA	UKB-b-57	TRUE	reported	8KoqAI
16	30490854	G	A	0.027344	0.00434	3.00E-10	0.141772	462346	NA	rs1186259	NA	UKB-b-57	TRUE	reported	8KoqAI
16	30491164	T	G	0.027809	0.004237	5.30E-11	0.150847	462346	NA	rs1136027	NA	UKB-b-57	TRUE	reported	8KoqAI
16	30491783	T	C	0.027558	0.004349	2.40E-10	0.140968	462346	NA	rs1783954	NA	UKB-b-57	TRUE	reported	8KoqAI
16	30492690	A	C	0.027819	0.004243	5.50E-11	0.150372	462346	NA	rs1087145	NA	UKB-b-57	TRUE	reported	8KoqAI
16	30502558	T	C	0.024803	0.004449	2.50E-08	0.134386	462346	NA	rs3506926	NA	UKB-b-57	TRUE	reported	8KoqAI
16	30519628	A	G	0.017041	0.003038	2.00E-08	0.521044	462346	NA	rs9746755	NA	UKB-b-57	TRUE	reported	8KoqAI
16	30585535	T	C	0.021193	0.003064	4.60E-12	0.4692	462346	NA	rs6565192	NA	UKB-b-57	TRUE	reported	8KoqAI
16	30611451	A	C	0.01918	0.003173	1.50E-09	0.356255	462346	NA	rs5587568	NA	UKB-b-57	TRUE	reported	8KoqAI
16	30623979	T	G	0.020186	0.003043	3.30E-11	0.477738	462346	NA	rs6769328	NA	UKB-b-57	TRUE	reported	8KoqAI
16	30630226	G	A	0.018035	0.003157	1.10E-08	0.359349	462346	NA	rs2884111	NA	UKB-b-57	TRUE	reported	8KoqAI
16	30634300	A	G	0.019851	0.003039	6.50E-11	0.476369	462346	NA	rs9927137	NA	UKB-b-57	TRUE	reported	8KoqAI
16	30635659	A	G	0.018224	0.003152	7.40E-09	0.359219	462346	NA	rs7186852	NA	UKB-b-57	TRUE	reported	8KoqAI
16	30636611	G	A	0.018224	0.003156	7.70E-09	0.357575	462346	NA	rs4485386	NA	UKB-b-57	TRUE	reported	8KoqAI
16	30636737	G	C	0.018274	0.003156	7.10E-09	0.357558	462346	NA	rs7191389	NA	UKB-b-57	TRUE	reported	8KoqAI
16	30636777	C	T	0.018149	0.003155	8.80E-09	0.358144	462346	NA	rs7499339	NA	UKB-b-57	TRUE	reported	8KoqAI
16	30636808	T	C	0.01823	0.003156	7.70E-09	0.357572	462346	NA	rs1130244	NA	UKB-b-57	TRUE	reported	8KoqAI
16	30636893	T	C	0.017631	0.003191	3.30E-08	0.345	462346	NA	rs1116391	NA	UKB-b-57	TRUE	reported	8KoqAI
16	30639710	G	A	0.018611	0.003155	3.70E-09	0.358221	462346	NA	rs7187802	NA	UKB-b-57	TRUE	reported	8KoqAI
16	30640185	G	A	0.018217	0.003153	7.60E-09	0.359306	462346	NA	rs7188789	NA	UKB-b-57	TRUE	reported	8KoqAI
16	30640550	C	T	0.018103	0.003152	9.30E-09	0.359946	462346	NA	rs7184055	NA	UKB-b-57	TRUE	reported	8KoqAI
16	30642867	C	T	0.017766	0.003143	1.60E-08	0.363507	462346	NA	rs7197475	NA	UKB-b-57	TRUE	reported	8KoqAI
16	30644216	C	T	0.017629	0.003144	2.00E-08	0.363686	462346	NA	rs7204632	NA	UKB-b-57	TRUE	reported	8KoqAI
16	30644567	C	T	0.017678	0.003144	1.90E-08	0.363461	462346	NA	rs7187071	NA	UKB-b-57	TRUE	reported	8KoqAI
16	30644899	T	C	0.0177	0.003144	1.80E-08	0.363535	462346	NA	rs9925025	NA	UKB-b-57	TRUE	reported	8KoqAI
16	30645283	G	A	0.017683	0.003144	1.90E-08	0.363464	462346	NA	rs2872833	NA	UKB-b-57	TRUE	reported	8KoqAI

16	30645416	G	C	0.017708	0.003145	1.80E-08	0.363426	462346	NA	rs7191761	NA	UKB-b-57	TRUE	reported	8KoqAI
16	30645644	A	T	0.017707	0.003145	1.80E-08	0.363522	462346	NA	rs7194347	NA	UKB-b-57	TRUE	reported	8KoqAI
16	30646496	G	A	0.017739	0.003146	1.70E-08	0.36352	462346	NA	rs5752636	NA	UKB-b-57	TRUE	reported	8KoqAI
16	30646970	T	C	0.017734	0.003145	1.70E-08	0.363553	462346	NA	rs3606456	NA	UKB-b-57	TRUE	reported	8KoqAI
16	30648260	A	G	0.017692	0.003146	1.90E-08	0.363513	462346	NA	rs7185620	NA	UKB-b-57	TRUE	reported	8KoqAI
16	30648547	G	A	0.017673	0.003146	1.90E-08	0.363714	462346	NA	rs6565195	NA	UKB-b-57	TRUE	reported	8KoqAI
16	30656594	T	A	0.018151	0.003147	8.00E-09	0.365072	462346	NA	rs6565196	NA	UKB-b-57	TRUE	reported	8KoqAI
16	30660700	G	A	0.018047	0.003145	9.60E-09	0.365807	462346	NA	rs2919426	NA	UKB-b-57	TRUE	reported	8KoqAI
16	30820866	T	C	-0.01843	0.003101	2.80E-09	0.390759	462346	NA	rs6205723	NA	UKB-b-57	TRUE	reported	8KoqAI
16	30823047	G	T	-0.01842	0.003101	2.80E-09	0.390866	462346	NA	rs4889490	NA	UKB-b-57	TRUE	reported	8KoqAI
16	30833246	C	T	-0.01815	0.003103	4.90E-09	0.391953	462346	NA	rs9319587	NA	UKB-b-57	TRUE	reported	8KoqAI
16	30846134	G	A	0.018177	0.003123	5.90E-09	0.612469	462346	NA	rs1186405	NA	UKB-b-57	TRUE	reported	8KoqAI
16	30878863	C	T	-0.01933	0.003132	6.80E-10	0.382303	462346	NA	rs4889634	NA	UKB-b-57	TRUE	reported	8KoqAI
16	30916233	G	A	-0.01882	0.003113	1.50E-09	0.381517	462346	NA	rs8046707	NA	UKB-b-57	TRUE	reported	8KoqAI
16	30918487	C	G	-0.01876	0.003105	1.50E-09	0.382057	462346	NA	rs1164965	NA	UKB-b-57	TRUE	reported	8KoqAI
16	30945887	G	A	-0.0199	0.003082	1.10E-10	0.405768	462346	NA	rs3573374	NA	UKB-b-57	TRUE	reported	8KoqAI
16	30948397	A	G	-0.01983	0.003082	1.20E-10	0.405877	462346	NA	rs1291772	NA	UKB-b-57	TRUE	reported	8KoqAI
16	30951566	C	G	-0.01987	0.003082	1.10E-10	0.405958	462346	NA	rs1333634	NA	UKB-b-57	TRUE	reported	8KoqAI
16	30979818	C	T	-0.0205	0.003096	3.60E-11	0.395399	462346	NA	rs1164096	NA	UKB-b-57	TRUE	reported	8KoqAI
16	30982225	A	G	-0.02045	0.003095	3.90E-11	0.396036	462346	NA	rs4889603	NA	UKB-b-57	TRUE	reported	8KoqAI
16	31004169	T	C	0.02101	0.003524	2.50E-09	0.297802	462346	NA	rs1785512	NA	UKB-b-57	TRUE	reported	8KoqAI
16	31011183	A	G	-0.02001	0.003131	1.60E-10	0.378187	462346	NA	rs4889606	NA	UKB-b-57	TRUE	reported	8KoqAI
16	31011821	T	G	-0.02013	0.003146	1.60E-10	0.375902	462346	NA	rs1271697	NA	UKB-b-57	TRUE	reported	8KoqAI
16	31014179	C	T	-0.01979	0.003132	2.60E-10	0.377847	462346	NA	rs9926533	NA	UKB-b-57	TRUE	reported	8KoqAI
16	31025641	C	T	-0.02014	0.003124	1.10E-10	0.377868	462346	NA	rs3489853	NA	UKB-b-57	TRUE	reported	8KoqAI
16	31037020	A	T	-0.02035	0.003122	7.00E-11	0.377082	462346	NA	rs1115060	NA	UKB-b-57	TRUE	reported	8KoqAI
16	31038687	T	A	-0.02041	0.003122	6.20E-11	0.376956	462346	NA	rs9939558	NA	UKB-b-57	TRUE	reported	8KoqAI
16	31042299	G	A	-0.02013	0.003122	1.10E-10	0.376576	462346	NA	rs1293441	NA	UKB-b-57	TRUE	reported	8KoqAI
16	31048079	C	T	-0.02004	0.003121	1.40E-10	0.376874	462346	NA	rs1087145	NA	UKB-b-57	TRUE	reported	8KoqAI
16	31048280	C	T	-0.02021	0.003121	9.60E-11	0.376682	462346	NA	rs1244832	NA	UKB-b-57	TRUE	reported	8KoqAI
16	31053467	C	T	-0.02011	0.003121	1.20E-10	0.376824	462346	NA	rs9939417	NA	UKB-b-57	TRUE	reported	8KoqAI
16	31054040	G	C	-0.01925	0.003113	6.20E-10	0.381683	462346	NA	rs2288004	NA	UKB-b-57	TRUE	reported	8KoqAI
16	31062701	C	A	-0.01917	0.003115	7.60E-10	0.382068	462346	NA	rs9929899	NA	UKB-b-57	TRUE	reported	8KoqAI
16	31065918	T	A	-0.01902	0.003114	1.00E-09	0.381893	462346	NA	rs4889530	NA	UKB-b-57	TRUE	reported	8KoqAI
16	31069854	G	A	-0.0197	0.003124	2.80E-10	0.375669	462346	NA	rs3535101	NA	UKB-b-57	TRUE	reported	8KoqAI
16	31070481	A	T	-0.01955	0.003125	4.00E-10	0.37561	462346	NA	rs5681353	NA	UKB-b-57	TRUE	reported	8KoqAI
16	31075175	G	A	-0.01969	0.003123	2.90E-10	0.375198	462346	NA	rs2303223	NA	UKB-b-57	TRUE	reported	8KoqAI
16	31079371	G	T	-0.01966	0.003124	3.10E-10	0.37537	462346	NA	rs881929	NA	UKB-b-57	TRUE	reported	8KoqAI
16	31081585	A	G	-0.01983	0.003123	2.20E-10	0.375599	462346	NA	rs9796794	NA	UKB-b-57	TRUE	reported	8KoqAI
16	31085470	T	C	-0.01895	0.003115	1.20E-09	0.380422	462346	NA	rs2303222	NA	UKB-b-57	TRUE	reported	8KoqAI
16	31088347	G	A	-0.01925	0.003129	7.60E-10	0.372287	462346	NA	rs749671	NA	UKB-b-57	TRUE	reported	8KoqAI
16	31088625	A	G	-0.01968	0.003124	3.00E-10	0.375781	462346	NA	rs749670	NA	UKB-b-57	TRUE	reported	8KoqAI
16	31103796	A	G	0.019494	0.003123	4.30E-10	0.624208	462346	NA	rs2359612	NA	UKB-b-57	TRUE	reported	8KoqAI
16	31104509	C	G	-0.01887	0.003115	1.40E-09	0.380212	462346	NA	rs8050894	NA	UKB-b-57	TRUE	reported	8KoqAI
16	31104878	G	A	-0.01969	0.003123	2.90E-10	0.375416	462346	NA	rs9934438	NA	UKB-b-57	TRUE	reported	8KoqAI
16	31105554	A	C	-0.01973	0.003468	1.30E-08	0.254972	462346	NA	rs2884737	NA	UKB-b-57	TRUE	reported	8KoqAI
16	31107689	C	T	-0.01985	0.003123	2.10E-10	0.375269	462346	NA	rs9923231	NA	UKB-b-57	TRUE	reported	8KoqAI
16	31110981	G	A	0.017568	0.003139	2.20E-08	0.633354	462346	NA	rs7196161	NA	UKB-b-57	TRUE	reported	8KoqAI
16	31114234	A	G	0.017634	0.003138	1.90E-08	0.63255	462346	NA	rs6116204	NA	UKB-b-57	TRUE	reported	8KoqAI
16	31117389	T	C	0.017352	0.003144	3.40E-08	0.635316	462346	NA	rs5631440	NA	UKB-b-57	TRUE	reported	8KoqAI
16	31117413	C	T	-0.01818	0.003153	8.10E-09	0.35974	462346	NA	rs2032915	NA	UKB-b-57	TRUE	reported	8KoqAI
16	31118024	C	A	-0.0174	0.003144	3.10E-08	0.364573	462346	NA	rs1271698	NA	UKB-b-57	TRUE	reported	8KoqAI
16	31121793	G	A	-0.01819	0.003153	8.00E-09	0.359761	462346	NA	rs14235	NA	UKB-b-57	TRUE	reported	8KoqAI
16	31124407	A	G	-0.01744	0.003145	2.90E-08	0.364778	462346	NA	rs749767	NA	UKB-b-57	TRUE	reported	8KoqAI
16	31125776	T	C	0.017409	0.003143	3.00E-08	0.634995	462346	NA	rs1164744	NA	UKB-b-57	TRUE	reported	8KoqAI
16	31128615	C	T	-0.01747	0.003144	2.70E-08	0.364574	462346	NA	rs4889619	NA	UKB-b-57	TRUE	reported	8KoqAI
16	31128668	C	A	-0.01748	0.003144	2.70E-08	0.364577	462346	NA	rs1271698	NA	UKB-b-57	TRUE	reported	8KoqAI
16	31129895	A	G	-0.01823	0.003153	7.40E-09	0.35978	462346	NA	rs9925964	NA	UKB-b-57	TRUE	reported	8KoqAI
16	31129942	C	T	0.017357	0.003143	3.30E-08	0.635021	462346	NA	rs1978487	NA	UKB-b-57	TRUE	reported	8KoqAI
16	31131121	C	T	0.017363	0.003143	3.30E-08	0.635006	462346	NA	rs1978485	NA	UKB-b-57	TRUE	reported	8KoqAI
16	31131174	G	A	-0.01822	0.003153	7.50E-09	0.359808	462346	NA	rs4889620	NA	UKB-b-57	TRUE	reported	8KoqAI
16	31131614	A	G	0.018143	0.003152	8.60E-09	0.639824	462346	NA	rs4527034	NA	UKB-b-57	TRUE	reported	8KoqAI
16	31137712	C	T	-0.01823	0.003153	7.40E-09	0.359732	462346	NA	rs889548	NA	UKB-b-57	TRUE	reported	8KoqAI
16	31140799	G	T	-0.01832	0.003154	6.30E-09	0.359581	462346	NA	rs9936329	NA	UKB-b-57	TRUE	reported	8KoqAI
16	31141993	C	T	-0.01837	0.003159	6.00E-09	0.357739	462346	NA	rs1549293	NA	UKB-b-57	TRUE	reported	8KoqAI
16	31145219	C	T	-0.01846	0.003162	5.30E-09	0.357402	462346	NA	rs1259751	NA	UKB-b-57	TRUE	reported	8KoqAI
16	31147548	G	A	0.018297	0.003156	6.70E-09	0.638621	462346	NA	rs2855475	NA	UKB-b-57	TRUE	reported	8KoqAI
16	31149142	A	G	0.018124	0.003176	1.20E-08	0.643891	462346	NA	rs9972727	NA	UKB-b-57	TRUE	reported	8KoqAI
16	51205819	T	G	0.022875	0.004063	1.80E-08	0.168868	462346	NA	rs728538	NA	UKB-b-57	TRUE	reported	8KoqAI
16	51210495	T	C	-0.02176	0.003789	9.30E-09	0.797617	462346	NA	rs2193098	NA	UKB-b-57	TRUE	reported	8KoqAI
16	53798622	G	T	0.017928	0.003054	4.30E-09	0.439503	462346	NA	rs8047587	NA	UKB-b-57	TRUE	reported	8KoqAI
16	53799507	G	A	0.018913	0.003062	6.60E-10	0.424321	462346	NA	rs9937053	NA	UKB-b-57	TRUE	reported	8KoqAI
16	53799847	G	A	0.018895	0.003063	6.80E-10	0.424274	462346	NA	rs9937354	NA	UKB-b-57	TRUE	reported	8KoqAI
16	53799905	A	G	0.018882	0.003063	7.00E-10	0.424278	462346	NA	rs9928094	NA	UKB-b-57	TRUE	reported	8KoqAI
16	53799977	T	G	0.018702	0.003063	1.00E-09	0.423455	462346	NA	rs9930333	NA	UKB-b-57	TRUE	reported	8KoqAI
16	53799985	T	A	0.018702	0.003063	1.00E-09	0.423456	462346	NA	rs9930397	NA	UKB-b-57	TRUE	reported	8KoqAI
16	53800200	C	T	0.018718	0.003063	9.90E-10	0.42344	462346	NA	rs9940278	NA	UKB-b-57	TRUE	reported	8KoqAI
16	53800568	G	A	0.018718	0.003063	9.90E-10	0.423454	462346	NA	rs9939973	NA	UKB-b-57	TRUE	reported	8KoqAI
16	53800629	C	G	0.01871	0.003063	1.00E-09	0.423457	462346	NA	rs9940646	NA	UKB-b-57	TRUE	reported	8KoqAI
16	53800754	G	A	0.018667	0.003063	1.10E-09	0.423552	462346	NA	rs9940128	NA	UKB-b-57	TRUE	reported	8KoqAI
16	53800954	T	C	0.019939	0.003085	1.00E-10	0.403447	462346	NA	rs1421085	NA	UKB-b-57	TRUE	reported	8KoqAI
16	53801343	C	A	0.018719	0.003063	9.90E-10	0.423465	462346	NA	rs1421086	NA	UKB-b-57	TRUE	reported	8KoqAI
16	53801549	C	T	0.018711	0.003063	1.00E-09	0.423468	462346	NA	rs9923147	NA	UKB-b-57	TRUE	reported	8KoqAI
16	53801985	C	T	0.018641	0.003063	1.20E-09	0.423649	462346	NA	rs9923544	NA	UKB-b-57	TRUE	reported	8KoqAI
16	53802494	C	T	0.019731	0.003085	1.60E-10	0.403658	462346	NA	rs1164201	NA	UKB-b-57	TRUE	reported	8KoqAI
16	53803187	A	T	0.018569	0.003063	1.30E-09	0.423678	462346	NA	rs1558901	NA	UKB-b-57	TRUE	reported	8KoqAI
16	53803223	G	A	0.019704	0.003085	1.70E-10	0.403638	462346	NA	rs6204840	NA	UKB-b-57	TRUE	reported	8KoqAI
16	53803574	T	A	0.019696	0.003085	1.70E-10	0.403654	462346	NA	rs1558902	NA	UKB-b-57	TRUE	reported	8KoqAI
16	53805207	C	A	0.018636	0.003062	1.20E-09	0.424671	462346	NA	rs1107598	NA	UKB-b-57	TRUE	reported	8KoqAI
16	53806453	A	G	0.019745	0.003084	1.50E-10	0.404628	462346	NA	rs5609464	NA	UKB-b-57	TRUE	reported	8KoqAI
16	53809123	C	T	0.019352	0.003085	3.50E-10	0.403647	462346	NA	rs5587272	NA	UKB-b-57	TRUE	reported	8KoqAI
16	53809247	G	A	0.018388	0.003062	1.90E-09	0.424017	462346	NA	rs1121980	NA	UKB-b-57	TRUE	reported	8KoqAI
16	53810546	C	A	0.018042	0.003098	5.70E-09	0.39467	462346	NA	rs7187250	NA	UKB-b-57	TRUE	reported	8KoqAI
16	53810686	T	C	0.01814	0.003097	4.70E-09	0.3951	462346	NA	rs7193144	NA	UKB-b-57	TRUE	reported	8KoqAI
16	53810943	C	T	0.018049	0.003097	5.60E-09	0.394992	462346	NA	rs6203339	NA	UKB-b-57	TRUE	reported	8KoqAI
16	53811788	A	G	0.018288	0.003097	3.50E-09	0.394818	462346	NA	rs6203340	NA	UKB-b-57	TRUE	reported	8KoqAI
16	53812433	T	C	0.01829	0.003095	3.40E-09	0.396254	462346	NA	rs8063057	NA	UKB-b-57	TRUE	reported	8KoqAI
16	53813367	T	G	0.017838	0.003095	8.30E-09	0.3953	462346	NA	rs1781744	NA	UKB-b-57	TRUE	reported	8KoqAI
16	53813450	A	T	0.018069	0.003095	5.30E-09	0.395398	462346	NA	rs8043757	NA	UKB-b-57	TRUE	reported	8KoqAI
16	53814363	G	T	0.018609	0.003095	1.80E-09	0.399605	462346	NA	rs9972653	NA	UKB-b-57	TRUE	reported	8KoqAI
16	53815435	T	C	0.017971	0.003098	6.60E-09	0.394149	462346	NA	rs1781749	NA	UKB-b-57	TRUE	reported	8KoqAI
16	53816275	C	A	0.018093	0.003096	5.10E-09	0.395095	462346	NA	rs8050136	NA	UKB-b-57	TRUE	reported	8KoqAI
16	53816752	A	G	0.017976	0.003096	6.40E-09	0.395141	462346	NA	rs8051591	NA	UKB-b-57	TRUE	reported	8KoqAI
16	53816838	G	A	0.017899	0.003096	7.40E-09	0.394803	462346	NA	rs9935401	NA	UKB-b-57	TRUE	reported	8KoqAI
16	53818167	T	C	0.016946	0.003072	3.50E-08	0.414082	462346	NA	rs9933509	NA	UKB-b-57	TRUE	reported	8KoqAI
16	53818460	G	T	0.017895	0.003098	7.60E-09	0.393606	462346	NA	rs3751812	NA	UKB-b-57	TRUE	reported	8KoqAI
16	53818708	G	T	0.017007	0.00304	2.20E-08	0.544614	462346	NA	rs3751813	NA	UKB-b-57	TRUE	reported	8KoqAI

16	53818724	G	A	0.01791	0.003096	7.30E-09	0.394709	462346	NA	rs3751814	NA	UKB-b-57	TRUE	reported	8KoqAI
16	53818813	A	G	0.016954	0.003072	3.40E-08	0.414079	462346	NA	rs9931900	NA	UKB-b-57	TRUE	reported	8KoqAI
16	53818834	A	G	0.017893	0.003096	7.50E-09	0.394668	462346	NA	rs5631353	NA	UKB-b-57	TRUE	reported	8KoqAI
16	53819169	T	C	0.017923	0.003097	7.10E-09	0.394662	462346	NA	rs9936385	NA	UKB-b-57	TRUE	reported	8KoqAI
16	53819198	G	C	0.017924	0.003097	7.10E-09	0.394661	462346	NA	rs9923233	NA	UKB-b-57	TRUE	reported	8KoqAI
16	53819367	G	A	0.017926	0.003096	7.10E-09	0.394683	462346	NA	rs9923312	NA	UKB-b-57	TRUE	reported	8KoqAI
16	53819771	G	A	0.017958	0.003096	6.60E-09	0.394673	462346	NA	rs1107598	NA	UKB-b-57	TRUE	reported	8KoqAI
16	53819877	C	T	0.017947	0.003096	6.80E-09	0.394653	462346	NA	rs1107598	NA	UKB-b-57	TRUE	reported	8KoqAI
16	53819893	A	G	0.017947	0.003096	6.80E-09	0.394653	462346	NA	rs1107599	NA	UKB-b-57	TRUE	reported	8KoqAI
16	53819937	A	T	0.017946	0.003096	6.80E-09	0.394649	462346	NA	rs1107599	NA	UKB-b-57	TRUE	reported	8KoqAI
16	53820066	T	C	0.017977	0.003096	6.40E-09	0.394654	462346	NA	rs1107599	NA	UKB-b-57	TRUE	reported	8KoqAI
16	53820503	G	A	0.017988	0.003096	6.30E-09	0.39465	462346	NA	rs9926289	NA	UKB-b-57	TRUE	reported	8KoqAI
16	53820527	T	A	0.018049	0.003096	5.60E-09	0.394667	462346	NA	rs9939609	NA	UKB-b-57	TRUE	reported	8KoqAI
16	53820813	A	G	0.01703	0.003072	3.00E-08	0.414038	462346	NA	rs9937709	NA	UKB-b-57	TRUE	reported	8KoqAI
16	53820996	C	G	0.017981	0.003098	6.50E-09	0.393907	462346	NA	rs9927317	NA	UKB-b-57	TRUE	reported	8KoqAI
16	53821125	A	G	0.017954	0.003098	6.80E-09	0.393467	462346	NA	rs1781771	NA	UKB-b-57	TRUE	reported	8KoqAI
16	53821297	T	C	0.017969	0.003096	6.50E-09	0.394649	462346	NA	rs7206410	NA	UKB-b-57	TRUE	reported	8KoqAI
16	53821413	T	C	0.017023	0.003072	3.00E-08	0.413997	462346	NA	rs7206629	NA	UKB-b-57	TRUE	reported	8KoqAI
16	53821615	A	G	0.017964	0.003096	6.60E-09	0.394711	462346	NA	rs7202116	NA	UKB-b-57	TRUE	reported	8KoqAI
16	53821690	A	G	0.018002	0.003096	6.10E-09	0.394706	462346	NA	rs7202296	NA	UKB-b-57	TRUE	reported	8KoqAI
16	53821862	C	T	0.017025	0.003072	3.00E-08	0.413965	462346	NA	rs7201850	NA	UKB-b-57	TRUE	reported	8KoqAI
16	53822142	C	A	0.017955	0.003097	6.70E-09	0.394587	462346	NA	rs6690803	NA	UKB-b-57	TRUE	reported	8KoqAI
16	53822183	C	T	0.017958	0.003097	6.70E-09	0.394597	462346	NA	rs7280369	NA	UKB-b-57	TRUE	reported	8KoqAI
16	53822237	A	G	0.017951	0.003097	6.80E-09	0.394589	462346	NA	rs6203340	NA	UKB-b-57	TRUE	reported	8KoqAI
16	53822239	A	G	0.017955	0.003097	6.70E-09	0.394596	462346	NA	rs6203340	NA	UKB-b-57	TRUE	reported	8KoqAI
16	53822387	C	T	0.017934	0.003098	7.10E-09	0.393464	462346	NA	rs6203340	NA	UKB-b-57	TRUE	reported	8KoqAI
16	53822440	G	A	0.017961	0.003097	6.60E-09	0.394565	462346	NA	rs7206122	NA	UKB-b-57	TRUE	reported	8KoqAI
16	53822502	T	G	0.017952	0.003097	6.70E-09	0.394574	462346	NA	rs7190396	NA	UKB-b-57	TRUE	reported	8KoqAI
16	53822651	A	G	0.017938	0.003097	6.90E-09	0.394591	462346	NA	rs7185735	NA	UKB-b-57	TRUE	reported	8KoqAI
16	53823727	T	C	0.017909	0.003098	7.40E-09	0.393451	462346	NA	rs7999496	NA	UKB-b-57	TRUE	reported	8KoqAI
16	53824007	G	A	0.017596	0.003033	6.50E-09	0.524096	462346	NA	rs1164671	NA	UKB-b-57	TRUE	reported	8KoqAI
16	53825488	C	T	0.016845	0.003073	4.20E-08	0.412721	462346	NA	rs9941349	NA	UKB-b-57	TRUE	reported	8KoqAI
16	53826028	T	C	0.017109	0.003073	2.60E-08	0.412988	462346	NA	rs2856772	NA	UKB-b-57	TRUE	reported	8KoqAI
16	53827179	C	G	0.01699	0.003074	3.20E-08	0.412801	462346	NA	rs9931494	NA	UKB-b-57	TRUE	reported	8KoqAI
16	53827479	A	G	0.017947	0.003099	6.90E-09	0.393446	462346	NA	rs1046828	NA	UKB-b-57	TRUE	reported	8KoqAI
16	53827962	A	G	0.017949	0.003099	6.90E-09	0.393457	462346	NA	rs6203340	NA	UKB-b-57	TRUE	reported	8KoqAI
16	53828066	C	T	0.017916	0.003099	7.40E-09	0.39338	462346	NA	rs1781796	NA	UKB-b-57	TRUE	reported	8KoqAI
16	53830055	C	G	0.017231	0.003075	2.10E-08	0.412913	462346	NA	rs6203341	NA	UKB-b-57	TRUE	reported	8KoqAI
16	53830465	A	G	0.017011	0.003053	2.50E-08	0.433358	462346	NA	rs9930506	NA	UKB-b-57	TRUE	reported	8KoqAI
16	53830491	T	C	0.016655	0.003054	4.90E-08	0.433454	462346	NA	rs9932754	NA	UKB-b-57	TRUE	reported	8KoqAI
16	53830867	T	A	0.016652	0.003054	4.90E-08	0.433546	462346	NA	rs9933040	NA	UKB-b-57	TRUE	reported	8KoqAI
16	53831354	C	T	0.017171	0.003074	2.30E-08	0.412916	462346	NA	rs7280561	NA	UKB-b-57	TRUE	reported	8KoqAI
16	53834607	T	C	0.017036	0.003073	3.00E-08	0.41492	462346	NA	rs7188250	NA	UKB-b-57	TRUE	reported	8KoqAI
16	53834608	G	A	0.017193	0.003075	2.30E-08	0.412798	462346	NA	rs7280561	NA	UKB-b-57	TRUE	reported	8KoqAI
16	53837144	G	T	0.017072	0.003074	2.80E-08	0.412768	462346	NA	rs1107599	NA	UKB-b-57	TRUE	reported	8KoqAI
16	53837342	A	G	0.017169	0.003074	2.30E-08	0.413121	462346	NA	rs7280561	NA	UKB-b-57	TRUE	reported	8KoqAI
16	53840525	C	G	0.017118	0.003073	2.60E-08	0.413024	462346	NA	rs1214957	NA	UKB-b-57	TRUE	reported	8KoqAI
16	53842908	G	A	0.017001	0.003073	3.20E-08	0.41249	462346	NA	rs1214983	NA	UKB-b-57	TRUE	reported	8KoqAI
16	64938505	C	T	0.021691	0.003922	3.20E-08	0.183691	462346	NA	rs1259962	NA	UKB-b-57	TRUE	reported	8KoqAI
16	64938709	T	A	0.021768	0.003922	2.90E-08	0.183562	462346	NA	rs1333437	NA	UKB-b-57	TRUE	reported	8KoqAI
16	64943026	T	A	0.021329	0.003891	4.20E-08	0.186937	462346	NA	rs1050050	NA	UKB-b-57	TRUE	reported	8KoqAI
16	64945230	C	G	0.021277	0.003892	4.60E-08	0.186879	462346	NA	rs1259610	NA	UKB-b-57	TRUE	reported	8KoqAI
16	64945284	A	G	0.021339	0.003892	4.20E-08	0.186868	462346	NA	rs1896754	NA	UKB-b-57	TRUE	reported	8KoqAI
16	64946215	C	T	0.021356	0.003891	4.00E-08	0.187033	462346	NA	rs1107555	NA	UKB-b-57	TRUE	reported	8KoqAI
16	64948718	C	A	0.021406	0.003891	3.80E-08	0.186982	462346	NA	rs1107555	NA	UKB-b-57	TRUE	reported	8KoqAI
16	64955205	G	A	0.021564	0.003892	3.00E-08	0.186794	462346	NA	rs1390641	NA	UKB-b-57	TRUE	reported	8KoqAI
16	64956309	G	A	0.021655	0.003891	2.60E-08	0.186815	462346	NA	rs5824512	NA	UKB-b-57	TRUE	reported	8KoqAI
16	64956443	G	C	0.02156	0.003891	3.00E-08	0.18677	462346	NA	rs5787984	NA	UKB-b-57	TRUE	reported	8KoqAI
16	64960624	G	A	0.021547	0.003888	3.00E-08	0.186893	462346	NA	rs1214974	NA	UKB-b-57	TRUE	reported	8KoqAI
16	64961353	A	T	0.021486	0.003889	3.30E-08	0.186845	462346	NA	rs5825945	NA	UKB-b-57	TRUE	reported	8KoqAI
16	64965198	A	G	0.021516	0.003888	3.10E-08	0.18686	462346	NA	rs5828356	NA	UKB-b-57	TRUE	reported	8KoqAI
16	64972773	C	T	0.021471	0.003887	3.30E-08	0.187032	462346	NA	rs1496397	NA	UKB-b-57	TRUE	reported	8KoqAI
16	64973894	T	C	0.021481	0.003886	3.20E-08	0.187214	462346	NA	rs1696817	NA	UKB-b-57	TRUE	reported	8KoqAI
16	64977883	C	G	0.021423	0.003886	3.50E-08	0.187177	462346	NA	rs1695648	NA	UKB-b-57	TRUE	reported	8KoqAI
16	64979976	G	T	0.021486	0.003886	3.20E-08	0.187218	462346	NA	rs1520238	NA	UKB-b-57	TRUE	reported	8KoqAI
16	69548788	T	C	-0.02752	0.004331	2.10E-10	0.852181	462346	NA	rs7200764	NA	UKB-b-57	TRUE	reported	8KoqAI
16	69559696	C	T	-0.02673	0.004265	3.70E-10	0.850458	462346	NA	rs4783720	NA	UKB-b-57	TRUE	reported	8KoqAI
16	69563890	C	T	-0.02647	0.004262	5.30E-10	0.850421	462346	NA	rs7193778	NA	UKB-b-57	TRUE	reported	8KoqAI
16	69578482	A	G	-0.02593	0.004227	8.50E-10	0.848207	462346	NA	rs8062941	NA	UKB-b-57	TRUE	reported	8KoqAI
16	69590366	A	G	-0.02619	0.004224	5.70E-10	0.848001	462346	NA	rs1549287	NA	UKB-b-57	TRUE	reported	8KoqAI
16	69591167	T	C	-0.02619	0.004222	5.50E-10	0.847934	462346	NA	rs1164499	NA	UKB-b-57	TRUE	reported	8KoqAI
16	69591283	C	A	-0.0262	0.004222	5.50E-10	0.847933	462346	NA	rs1972658	NA	UKB-b-57	TRUE	reported	8KoqAI
16	69591580	A	G	-0.02629	0.004223	4.80E-10	0.847974	462346	NA	rs1465451	NA	UKB-b-57	TRUE	reported	8KoqAI
16	69599803	A	G	-0.02627	0.004225	5.10E-10	0.847994	462346	NA	rs8048032	NA	UKB-b-57	TRUE	reported	8KoqAI
16	69610002	A	G	-0.02622	0.004242	6.40E-10	0.848807	462346	NA	rs244423	NA	UKB-b-57	TRUE	reported	8KoqAI
16	69617513	C	G	-0.02626	0.004238	5.80E-10	0.848654	462346	NA	rs244422	NA	UKB-b-57	TRUE	reported	8KoqAI
16	69619388	T	G	-0.02623	0.004242	6.30E-10	0.848807	462346	NA	rs168637	NA	UKB-b-57	TRUE	reported	8KoqAI
16	69631748	A	G	-0.02634	0.004242	5.30E-10	0.848841	462346	NA	rs33060	NA	UKB-b-57	TRUE	reported	8KoqAI
16	69633245	T	C	-0.02634	0.004239	5.20E-10	0.848616	462346	NA	rs33061	NA	UKB-b-57	TRUE	reported	8KoqAI
16	69640217	A	G	-0.02633	0.004242	5.40E-10	0.848857	462346	NA	rs33063	NA	UKB-b-57	TRUE	reported	8KoqAI
16	69653696	C	G	-0.02687	0.004225	2.00E-10	0.84761	462346	NA	rs39999	NA	UKB-b-57	TRUE	reported	8KoqAI
16	69656889	G	A	-0.0268	0.004226	2.30E-10	0.847747	462346	NA	rs190282	NA	UKB-b-57	TRUE	reported	8KoqAI
16	69657656	T	C	-0.02677	0.004241	2.70E-10	0.848592	462346	NA	rs244419	NA	UKB-b-57	TRUE	reported	8KoqAI
16	69665208	T	C	-0.02668	0.004242	3.20E-10	0.848675	462346	NA	rs244416	NA	UKB-b-57	TRUE	reported	8KoqAI
16	69695643	G	A	0.026495	0.004233	3.90E-10	0.151948	462346	NA	rs7278311	NA	UKB-b-57	TRUE	reported	8KoqAI
16	69697615	A	C	0.026739	0.004223	2.40E-10	0.152401	462346	NA	rs7278311	NA	UKB-b-57	TRUE	reported	8KoqAI
16	69701487	G	C	0.026696	0.004226	2.70E-10	0.152206	462346	NA	rs1729806	NA	UKB-b-57	TRUE	reported	8KoqAI
16	69711888	T	G	0.026645	0.004224	2.80E-10	0.152343	462346	NA	rs6204997	NA	UKB-b-57	TRUE	reported	8KoqAI
16	69737468	C	G	0.026739	0.004231	2.60E-10	0.152052	462346	NA	rs9980	NA	UKB-b-57	TRUE	reported	8KoqAI
16	69737908	C	T	0.026747	0.004231	2.60E-10	0.152059	462346	NA	rs1043470	NA	UKB-b-57	TRUE	reported	8KoqAI
16	69742387	A	G	0.026746	0.004224	2.40E-10	0.152421	462346	NA	rs5612599	NA	UKB-b-57	TRUE	reported	8KoqAI
16	69763280	C	T	0.026789	0.00405	3.70E-11	0.169432	462346	NA	rs1134410	NA	UKB-b-57	TRUE	reported	8KoqAI
16	69765524	C	T	0.026685	0.00408	6.10E-11	0.166421	462346	NA	rs1214922	NA	UKB-b-57	TRUE	reported	8KoqAI
16	69770032	G	A	0.027223	0.004076	2.40E-11	0.166584	462346	NA	rs1389932	NA	UKB-b-57	TRUE	reported	8KoqAI
16	69775500	C	T	0.027083	0.004076	3.00E-11	0.166787	462346	NA	rs1729947	NA	UKB-b-57	TRUE	reported	8KoqAI
16	69789196	C	T	0.02694	0.004065	3.40E-11	0.167129	462346	NA	rs5625987	NA	UKB-b-57	TRUE	reported	8KoqAI
16	69795323	A	T	0.022769	0.003928	6.80E-09	0.184105	462346	NA	rs5614006	NA	UKB-b-57	TRUE	reported	8KoqAI
16	69802865	A	T	0.023126	0.003886	2.70E-09	0.186871	462346	NA	rs6205003	NA	UKB-b-57	TRUE	reported	8KoqAI
16	69818983	A	G	0.022894	0.003887	3.90E-09	0.186849	462346	NA	rs1134206	NA	UKB-b-57	TRUE	reported	8KoqAI
16	71861143	A	T	-0.01984	0.003394	5.10E-09	0.275756	462346	NA	rs7185027	NA	UKB-b-57	TRUE	reported	8KoqAI
16	71864108	T	C	-0.01986	0.003393	4.80E-09	0.27597	462346	NA	rs2878494	NA	UKB-b-57	TRUE	reported	8KoqAI
16	71866460	G	A	-0.02001	0.003394	3.70E-09	0.275624	462346	NA	rs8053906	NA	UKB-b-57	TRUE	reported	8KoqAI
16	71870208	G	C	-0.01961	0.003391	7.30E-09	0.275317	462346	NA	rs8046044	NA	UKB-b-57	TRUE	reported	8KoqAI
16	71876064	G	C	-0.01981	0.003391	5.20E-09	0.275082	462346	NA	rs3559514	NA	UKB-b-57	TRUE	reported	8KoqAI
16	71879162	C	T	-0.01968	0.003391	6.50E-09	0.27523	462346	NA	rs3520900	NA	UKB-b-57	TRUE	reported	8KoqAI
16	71879672	G	A	-0.01972	0.003391	6.10E-09	0.275273	462346	NA	rs3502875	NA	UKB-b-57	TRUE	reported	8KoqAI
16	71880586	C	G	-0.01951	0.00339	8.60E-09	0.275606	462346	NA	rs1333017	NA	UKB-b-57	TRUE	reported	8KoqAI

16	71881030	A	G	-0.01984	0.003391	4.90E-09	0.275106	462346	NA	rs1164013	NA	UKB-b-57	TRUE	reported	8KoqAI
16	71881337	A	T	-0.01887	0.0034	2.90E-08	0.273245	462346	NA	rs1085250	NA	UKB-b-57	TRUE	reported	8KoqAI
16	71890550	G	A	-0.01911	0.003397	1.80E-08	0.274233	462346	NA	rs3088285	NA	UKB-b-57	TRUE	reported	8KoqAI
16	71891966	T	G	-0.01948	0.003395	9.60E-09	0.274646	462346	NA	rs8063657	NA	UKB-b-57	TRUE	reported	8KoqAI
16	71898479	C	T	-0.01918	0.003392	1.60E-08	0.274543	462346	NA	rs7198834	NA	UKB-b-57	TRUE	reported	8KoqAI
16	71899189	G	A	-0.01892	0.003411	2.90E-08	0.270489	462346	NA	rs8052358	NA	UKB-b-57	TRUE	reported	8KoqAI
16	71901648	T	C	-0.01995	0.00339	4.00E-09	0.275116	462346	NA	rs1735742	NA	UKB-b-57	TRUE	reported	8KoqAI
16	71904178	A	G	-0.01964	0.003388	6.80E-09	0.275583	462346	NA	rs1050056	NA	UKB-b-57	TRUE	reported	8KoqAI
16	71904877	G	A	-0.01961	0.003395	7.60E-09	0.27394	462346	NA	rs1214991	NA	UKB-b-57	TRUE	reported	8KoqAI
16	71905892	G	A	-0.01944	0.003394	1.00E-08	0.274012	462346	NA	rs1292145	NA	UKB-b-57	TRUE	reported	8KoqAI
16	71909230	G	A	-0.01956	0.003388	7.70E-09	0.275567	462346	NA	rs1244808	NA	UKB-b-57	TRUE	reported	8KoqAI
16	71918675	T	C	-0.01916	0.003391	1.60E-08	0.274598	462346	NA	rs1107590	NA	UKB-b-57	TRUE	reported	8KoqAI
16	71921969	G	A	-0.01949	0.003393	9.20E-09	0.274165	462346	NA	rs3466483	NA	UKB-b-57	TRUE	reported	8KoqAI
16	71928693	C	T	-0.01944	0.003393	1.00E-08	0.274034	462346	NA	rs7138693	NA	UKB-b-57	TRUE	reported	8KoqAI
16	71941259	C	T	-0.01949	0.003387	8.70E-09	0.275642	462346	NA	rs3553552	NA	UKB-b-57	TRUE	reported	8KoqAI
16	71943309	C	T	-0.01985	0.003389	4.70E-09	0.275205	462346	NA	rs1244556	NA	UKB-b-57	TRUE	reported	8KoqAI
16	71945278	A	G	-0.01984	0.003389	4.70E-09	0.275196	462346	NA	rs7138694	NA	UKB-b-57	TRUE	reported	8KoqAI
16	71945847	G	C	-0.01954	0.003388	8.00E-09	0.275613	462346	NA	rs3540479	NA	UKB-b-57	TRUE	reported	8KoqAI
16	71947734	G	A	-0.01984	0.003389	4.80E-09	0.275198	462346	NA	rs4528577	NA	UKB-b-57	TRUE	reported	8KoqAI
16	71950293	G	A	-0.01929	0.003412	1.60E-08	0.271146	462346	NA	rs1292127	NA	UKB-b-57	TRUE	reported	8KoqAI
16	71953022	G	A	-0.0197	0.003387	6.00E-09	0.275657	462346	NA	rs7188591	NA	UKB-b-57	TRUE	reported	8KoqAI
16	71958052	A	G	-0.0197	0.003388	6.00E-09	0.275655	462346	NA	rs7197486	NA	UKB-b-57	TRUE	reported	8KoqAI
16	71959078	G	C	-0.01991	0.00339	4.30E-09	0.275153	462346	NA	rs7138478	NA	UKB-b-57	TRUE	reported	8KoqAI
16	71961906	A	G	-0.01983	0.003389	4.80E-09	0.275773	462346	NA	rs3751820	NA	UKB-b-57	TRUE	reported	8KoqAI
16	71963186	C	T	-0.0199	0.00339	4.40E-09	0.27508	462346	NA	rs7195988	NA	UKB-b-57	TRUE	reported	8KoqAI
16	71963924	C	T	-0.01988	0.00339	4.50E-09	0.275055	462346	NA	rs3402991	NA	UKB-b-57	TRUE	reported	8KoqAI
16	71964805	C	T	-0.01925	0.003427	1.90E-08	0.266002	462346	NA	rs9940234	NA	UKB-b-57	TRUE	reported	8KoqAI
16	71965230	C	G	-0.0195	0.003428	1.30E-08	0.26553	462346	NA	rs1293451	NA	UKB-b-57	TRUE	reported	8KoqAI
16	71967783	C	T	-0.0195	0.003428	1.30E-08	0.265596	462346	NA	rs1244564	NA	UKB-b-57	TRUE	reported	8KoqAI
16	71967927	C	T	-0.01971	0.003427	8.80E-09	0.265663	462346	NA	rs1735840	NA	UKB-b-57	TRUE	reported	8KoqAI
16	71968091	A	C	-0.0195	0.003428	1.30E-08	0.265523	462346	NA	rs1214930	NA	UKB-b-57	TRUE	reported	8KoqAI
16	71968147	G	C	-0.01949	0.003428	1.30E-08	0.265562	462346	NA	rs1214932	NA	UKB-b-57	TRUE	reported	8KoqAI
16	71969497	C	T	-0.01933	0.003426	1.70E-08	0.26598	462346	NA	rs1186545	NA	UKB-b-57	TRUE	reported	8KoqAI
16	71971091	T	C	-0.0189	0.003425	3.40E-08	0.266304	462346	NA	rs7191848	NA	UKB-b-57	TRUE	reported	8KoqAI
16	71971900	G	A	-0.01949	0.003428	1.30E-08	0.265739	462346	NA	rs1085250	NA	UKB-b-57	TRUE	reported	8KoqAI
16	71978313	C	T	-0.01933	0.003426	1.70E-08	0.26638	462346	NA	rs8049508	NA	UKB-b-57	TRUE	reported	8KoqAI
16	71979120	T	A	-0.01954	0.003427	1.20E-08	0.266508	462346	NA	rs1292769	NA	UKB-b-57	TRUE	reported	8KoqAI
16	71981374	T	G	-0.01945	0.003427	1.40E-08	0.266362	462346	NA	rs8050058	NA	UKB-b-57	TRUE	reported	8KoqAI
16	71983929	A	T	-0.02022	0.003448	4.50E-09	0.262071	462346	NA	rs3537766	NA	UKB-b-57	TRUE	reported	8KoqAI
16	71988383	G	A	-0.02023	0.003436	3.90E-09	0.263724	462346	NA	rs1163984	NA	UKB-b-57	TRUE	reported	8KoqAI
16	71992584	G	A	-0.02234	0.003417	6.20E-11	0.268855	462346	NA	rs3618299	NA	UKB-b-57	TRUE	reported	8KoqAI
16	71992817	C	A	-0.02237	0.003417	6.00E-11	0.268809	462346	NA	rs7138694	NA	UKB-b-57	TRUE	reported	8KoqAI
16	71995989	A	T	-0.02227	0.00342	7.40E-11	0.268748	462346	NA	rs3410168	NA	UKB-b-57	TRUE	reported	8KoqAI
16	72052034	C	G	-0.02727	0.004266	1.60E-10	0.148056	462346	NA	rs1164788	NA	UKB-b-57	TRUE	reported	8KoqAI
16	72054052	G	A	-0.0272	0.004291	2.30E-10	0.147015	462346	NA	rs1121286	NA	UKB-b-57	TRUE	reported	8KoqAI
16	72054066	T	G	-0.02718	0.004291	2.40E-10	0.146983	462346	NA	rs1413057	NA	UKB-b-57	TRUE	reported	8KoqAI
16	72055336	C	A	-0.02763	0.004285	1.10E-10	0.147497	462346	NA	rs5638729	NA	UKB-b-57	TRUE	reported	8KoqAI
16	72058667	G	A	-0.0276	0.004266	9.80E-11	0.147883	462346	NA	rs1133657	NA	UKB-b-57	TRUE	reported	8KoqAI
16	72061041	G	A	-0.02758	0.004265	1.00E-10	0.147888	462346	NA	rs1131912	NA	UKB-b-57	TRUE	reported	8KoqAI
16	72064905	A	G	-0.02661	0.004243	3.60E-10	0.149686	462346	NA	rs5582490	NA	UKB-b-57	TRUE	reported	8KoqAI
16	72066102	A	C	-0.02241	0.003941	1.30E-08	0.179873	462346	NA	rs7278703	NA	UKB-b-57	TRUE	reported	8KoqAI
16	72066879	C	T	-0.02763	0.004264	9.10E-11	0.147867	462346	NA	rs1164142	NA	UKB-b-57	TRUE	reported	8KoqAI
16	72067522	A	G	-0.02769	0.004264	8.40E-11	0.14787	462346	NA	rs7278704	NA	UKB-b-57	TRUE	reported	8KoqAI
16	72071501	C	T	-0.02683	0.004242	2.50E-10	0.149854	462346	NA	rs7499467	NA	UKB-b-57	TRUE	reported	8KoqAI
16	72078907	G	A	-0.02276	0.003958	8.90E-09	0.178303	462346	NA	rs1424241	NA	UKB-b-57	TRUE	reported	8KoqAI
16	72079342	A	G	-0.02623	0.004239	6.10E-10	0.150129	462346	NA	rs7278704	NA	UKB-b-57	TRUE	reported	8KoqAI
16	72093134	C	A	-0.02612	0.004237	7.10E-10	0.152215	462346	NA	rs3852780	NA	UKB-b-57	TRUE	reported	8KoqAI
16	72098260	C	G	-0.02248	0.003941	1.20E-08	0.180188	462346	NA	rs5612924	NA	UKB-b-57	TRUE	reported	8KoqAI
16	72099694	C	T	-0.027	0.004246	2.00E-10	0.149609	462346	NA	rs7278705	NA	UKB-b-57	TRUE	reported	8KoqAI
16	72102813	G	C	-0.02207	0.003936	2.00E-08	0.180942	462346	NA	rs6499558	NA	UKB-b-57	TRUE	reported	8KoqAI
16	72105084	C	T	-0.02281	0.003943	7.30E-09	0.179677	462346	NA	rs7278705	NA	UKB-b-57	TRUE	reported	8KoqAI
16	72105371	T	C	-0.02697	0.004247	2.10E-10	0.149513	462346	NA	rs7278706	NA	UKB-b-57	TRUE	reported	8KoqAI
16	72105844	G	A	-0.02819	0.004103	6.40E-12	0.162767	462346	NA	rs7278706	NA	UKB-b-57	TRUE	reported	8KoqAI
16	72106024	C	T	-0.0227	0.003941	8.40E-09	0.179977	462346	NA	rs8047930	NA	UKB-b-57	TRUE	reported	8KoqAI
16	72106906	T	C	-0.02261	0.003956	1.10E-08	0.178456	462346	NA	rs1107591	NA	UKB-b-57	TRUE	reported	8KoqAI
16	72108687	A	T	-0.02692	0.004246	2.30E-10	0.149466	462346	NA	rs1164636	NA	UKB-b-57	TRUE	reported	8KoqAI
16	72151101	T	C	-0.02387	0.004126	7.20E-09	0.160853	462346	NA	rs7278707	NA	UKB-b-57	TRUE	reported	8KoqAI
16	72153125	C	G	-0.02464	0.004153	3.00E-09	0.157772	462346	NA	rs4788459	NA	UKB-b-57	TRUE	reported	8KoqAI
16	72154682	G	T	-0.02479	0.004142	2.20E-09	0.158821	462346	NA	rs4788605	NA	UKB-b-57	TRUE	reported	8KoqAI
16	72155241	G	A	-0.02382	0.00415	9.60E-09	0.158187	462346	NA	rs1164172	NA	UKB-b-57	TRUE	reported	8KoqAI
16	72156674	C	T	-0.02368	0.004147	1.10E-08	0.158266	462346	NA	rs7278707	NA	UKB-b-57	TRUE	reported	8KoqAI
16	72158493	C	T	-0.02452	0.004156	3.70E-09	0.157413	462346	NA	rs7278707	NA	UKB-b-57	TRUE	reported	8KoqAI
16	72162966	G	T	-0.02327	0.004148	2.00E-08	0.158228	462346	NA	rs3483258	NA	UKB-b-57	TRUE	reported	8KoqAI
16	72169002	C	A	-0.02387	0.004147	8.60E-09	0.15825	462346	NA	rs1760393	NA	UKB-b-57	TRUE	reported	8KoqAI
16	72172300	C	T	-0.02364	0.004169	1.40E-08	0.156627	462346	NA	rs1164072	NA	UKB-b-57	TRUE	reported	8KoqAI
16	72175256	A	G	-0.02393	0.004146	7.90E-09	0.158302	462346	NA	rs4788611	NA	UKB-b-57	TRUE	reported	8KoqAI
16	72182564	C	T	-0.02359	0.004151	1.30E-08	0.157815	462346	NA	rs7278708	NA	UKB-b-57	TRUE	reported	8KoqAI
16	72184566	C	T	-0.02349	0.00415	1.50E-08	0.157807	462346	NA	rs3537063	NA	UKB-b-57	TRUE	reported	8KoqAI
16	72187957	A	T	-0.0245	0.004152	3.60E-09	0.158336	462346	NA	rs3812985	NA	UKB-b-57	TRUE	reported	8KoqAI
16	72188828	T	C	-0.02409	0.00415	6.40E-09	0.158433	462346	NA	rs4788614	NA	UKB-b-57	TRUE	reported	8KoqAI
16	72190184	C	T	-0.02392	0.00417	9.70E-09	0.156517	462346	NA	rs7278709	NA	UKB-b-57	TRUE	reported	8KoqAI
16	72190380	G	A	-0.02389	0.00417	1.00E-08	0.156539	462346	NA	rs1429798	NA	UKB-b-57	TRUE	reported	8KoqAI
16	72190766	C	T	-0.02392	0.00417	9.70E-09	0.156509	462346	NA	rs7278709	NA	UKB-b-57	TRUE	reported	8KoqAI
16	72192372	G	A	-0.02364	0.004171	1.40E-08	0.156598	462346	NA	rs4788615	NA	UKB-b-57	TRUE	reported	8KoqAI
16	72192613	C	A	-0.02365	0.004171	1.40E-08	0.15657	462346	NA	rs7278709	NA	UKB-b-57	TRUE	reported	8KoqAI
16	72192789	G	A	-0.02363	0.004171	1.50E-08	0.156616	462346	NA	rs1164847	NA	UKB-b-57	TRUE	reported	8KoqAI
16	72193906	A	G	-0.02466	0.004118	2.10E-09	0.161848	462346	NA	rs8047881	NA	UKB-b-57	TRUE	reported	8KoqAI
16	72196776	A	G	-0.02358	0.004151	1.30E-08	0.158613	462346	NA	rs2841907	NA	UKB-b-57	TRUE	reported	8KoqAI
16	72198642	G	A	-0.02402	0.004237	1.40E-08	0.151107	462346	NA	rs3852785	NA	UKB-b-57	TRUE	reported	8KoqAI
16	72204069	T	A	-0.02386	0.00419	1.20E-08	0.154909	462346	NA	rs7279111	NA	UKB-b-57	TRUE	reported	8KoqAI
16	72207315	T	C	-0.02457	0.004074	1.60E-09	0.166166	462346	NA	rs7279111	NA	UKB-b-57	TRUE	reported	8KoqAI
16	72213691	G	C	-0.02456	0.004151	3.30E-09	0.158691	462346	NA	rs5595572	NA	UKB-b-57	TRUE	reported	8KoqAI
16	72215147	A	T	-0.02339	0.004082	1.00E-08	0.16719	462346	NA	rs3574611	NA	UKB-b-57	TRUE	reported	8KoqAI
16	72217385	T	C	-0.02425	0.004052	2.20E-09	0.167947	462346	NA	rs7279111	NA	UKB-b-57	TRUE	reported	8KoqAI
16	72223716	G	T	-0.02338	0.00419	2.40E-08	0.154705	462346	NA	rs4788463	NA	UKB-b-57	TRUE	reported	8KoqAI
16	72224583	G	T	-0.02351	0.00419	2.00E-08	0.154692	462346	NA	rs7279112	NA	UKB-b-57	TRUE	reported	8KoqAI
16	72225294	C	T	-0.02269	0.004109	3.40E-08	0.161995	462346	NA	rs7279112	NA	UKB-b-57	TRUE	reported	8KoqAI
16	72229532	T	C	-0.02281	0.00405	1.80E-08	0.1678	462346	NA	rs1164589	NA	UKB-b-57	TRUE	reported	8KoqAI
16	72243277	T	C	-0.02288	0.004057	1.70E-08	0.168921	462346	NA	rs1164712	NA	UKB-b-57	TRUE	reported	8KoqAI
16	72258743	C	T	-0.02349	0.004209	2.40E-08	0.154124	462346	NA	rs6174747	NA	UKB-b-57	TRUE	reported	8KoqAI
16	72259718	G	C	-0.02329	0.004205	3.10E-08	0.154322	462346	NA	rs7279113	NA	UKB-b-57	TRUE	reported	8KoqAI
16	72338507	G	C	-0.02944	0.004897	1.80E-09	0.107761	462346	NA	rs7961669	NA	UKB-b-57	TRUE	reported	8KoqAI
16	72348272	A	T	-0.02937	0.004887	1.80E-09	0.108281	462346	NA	rs7847017	NA	UKB-b-57	TRUE	reported	8KoqAI
16	72356964	T	C	-0.0301	0.004901	8.20E-10	0.107904	462346	NA	rs1164857	NA	UKB-b-57	TRUE	reported	8KoqAI
16	72594387	C	T	-0.02307	0.003992	7.60E-09	0.176756	462346	NA	rs1050056	NA	UKB-b-57	TRUE	reported	8KoqAI
16	72628693	C	A	-0.02491	0.004112	1.40E-09	0.165764	462346	NA	rs7280318	NA	UKB-b-57	TRUE	reported	8KoqAI
16	73912588	G	C	0.017421	0.003088	1.70E-08	0.426338	462346	NA	rs1104608	NA	UKB-b-57	TRUE	reported	8KoqAI

17	7439014	A	G	-0.02135	0.003633	4.20E-09	0.774023	462346	NA	rs9892862	NA	UKB-b-57	TRUE	reported	8KoqAI
17	7440584	T	C	-0.02138	0.003634	4.00E-09	0.774488	462346	NA	rs8073177	NA	UKB-b-57	TRUE	reported	8KoqAI
17	7440984	C	T	-0.02137	0.003633	4.00E-09	0.774302	462346	NA	rs9902027	NA	UKB-b-57	TRUE	reported	8KoqAI
17	7446081	T	C	0.020941	0.003643	9.00E-09	0.222193	462346	NA	rs1165601	NA	UKB-b-57	TRUE	reported	8KoqAI
17	7447064	A	G	0.021046	0.003647	7.90E-09	0.222023	462346	NA	rs9900162	NA	UKB-b-57	TRUE	reported	8KoqAI
17	7447910	C	T	0.020993	0.003647	8.60E-09	0.222078	462346	NA	rs1165178	NA	UKB-b-57	TRUE	reported	8KoqAI
17	7448003	C	T	0.020933	0.003647	9.50E-09	0.222069	462346	NA	rs1107869	NA	UKB-b-57	TRUE	reported	8KoqAI
17	7452078	G	A	0.020923	0.003657	1.10E-08	0.221043	462346	NA	rs7729490	NA	UKB-b-57	TRUE	reported	8KoqAI
17	7599851	T	C	0.021571	0.003926	3.90E-08	0.185092	462346	NA	rs1295029	NA	UKB-b-57	TRUE	reported	8KoqAI
17	7600926	A	G	0.021714	0.003931	3.30E-08	0.184509	462346	NA	rs1165909	NA	UKB-b-57	TRUE	reported	8KoqAI
17	7604471	A	G	0.023964	0.004083	4.40E-09	0.16947	462346	NA	rs6081518	NA	UKB-b-57	TRUE	reported	8KoqAI
17	7606722	C	G	0.022095	0.003861	1.10E-08	0.198305	462346	NA	rs7640	NA	UKB-b-57	TRUE	reported	8KoqAI
17	7615286	C	T	0.023735	0.004085	6.20E-09	0.164625	462346	NA	rs9907011	NA	UKB-b-57	TRUE	reported	8KoqAI
17	7615745	G	A	0.023788	0.003962	1.90E-09	0.176998	462346	NA	rs9906502	NA	UKB-b-57	TRUE	reported	8KoqAI
17	27564013	C	T	0.024997	0.004093	1.00E-09	0.169072	462346	NA	rs1121780	NA	UKB-b-57	TRUE	reported	8KoqAI
17	27588806	C	A	0.024781	0.003925	2.70E-10	0.182509	462346	NA	rs8614	NA	UKB-b-57	TRUE	reported	8KoqAI
17	27693998	A	G	0.021627	0.003941	4.10E-08	0.180772	462346	NA	rs7262498	NA	UKB-b-57	TRUE	reported	8KoqAI
17	27726151	A	G	0.021509	0.003938	4.70E-08	0.181183	462346	NA	rs9907984	NA	UKB-b-57	TRUE	reported	8KoqAI
17	27732942	C	T	0.021482	0.003937	4.80E-08	0.181008	462346	NA	rs9900684	NA	UKB-b-57	TRUE	reported	8KoqAI
17	29681245	A	G	-0.017	0.003094	3.90E-08	0.602188	462346	NA	rs2525570	NA	UKB-b-57	TRUE	reported	8KoqAI
17	29730350	T	C	0.019867	0.003641	4.90E-08	0.232109	462346	NA	rs9891943	NA	UKB-b-57	TRUE	reported	8KoqAI
17	29735752	A	C	0.020589	0.00359	9.70E-09	0.239585	462346	NA	rs9912298	NA	UKB-b-57	TRUE	reported	8KoqAI
17	40698075	A	G	0.020601	0.003508	4.30E-09	0.248268	462346	NA	rs591939	NA	UKB-b-57	TRUE	reported	8KoqAI
17	40720632	G	A	0.018372	0.003355	4.40E-08	0.284587	462346	NA	rs646123	NA	UKB-b-57	TRUE	reported	8KoqAI
17	40721042	C	T	0.020736	0.003508	3.40E-09	0.247918	462346	NA	rs650558	NA	UKB-b-57	TRUE	reported	8KoqAI
17	40722029	A	G	0.018438	0.003357	3.90E-08	0.284851	462346	NA	rs665268	NA	UKB-b-57	TRUE	reported	8KoqAI
17	40731411	G	C	0.018432	0.00337	4.50E-08	0.280584	462346	NA	rs3485540	NA	UKB-b-57	TRUE	reported	8KoqAI
17	40731597	T	A	0.018517	0.003355	3.40E-08	0.284536	462346	NA	rs6963	NA	UKB-b-57	TRUE	reported	8KoqAI
17	40735641	C	T	0.018549	0.003356	3.20E-08	0.284488	462346	NA	rs1045408	NA	UKB-b-57	TRUE	reported	8KoqAI
17	40741013	T	C	0.018493	0.003357	3.60E-08	0.284174	462346	NA	rs1295163	NA	UKB-b-57	TRUE	reported	8KoqAI
17	40759448	G	A	0.018677	0.003355	2.60E-08	0.28457	462346	NA	rs6092981	NA	UKB-b-57	TRUE	reported	8KoqAI
17	40768629	C	T	0.018568	0.003354	3.10E-08	0.284646	462346	NA	rs7432239	NA	UKB-b-57	TRUE	reported	8KoqAI
17	40772288	T	C	0.018726	0.003355	2.40E-08	0.284676	462346	NA	rs3563595	NA	UKB-b-57	TRUE	reported	8KoqAI
17	40780847	C	T	0.018545	0.003355	3.20E-08	0.28464	462346	NA	rs1260145	NA	UKB-b-57	TRUE	reported	8KoqAI
17	40781561	G	T	0.018473	0.003371	4.20E-08	0.280625	462346	NA	rs3413814	NA	UKB-b-57	TRUE	reported	8KoqAI
17	40787764	C	G	0.018674	0.003357	2.70E-08	0.284649	462346	NA	rs1293890	NA	UKB-b-57	TRUE	reported	8KoqAI
17	43473307	C	G	0.021595	0.003908	3.30E-08	0.18454	462346	NA	rs6206459	NA	UKB-b-57	TRUE	reported	8KoqAI
17	43474668	C	G	0.021663	0.003902	2.80E-08	0.18523	462346	NA	rs6206459	NA	UKB-b-57	TRUE	reported	8KoqAI
17	43475929	G	A	0.021691	0.003904	2.80E-08	0.185081	462346	NA	rs2028078	NA	UKB-b-57	TRUE	reported	8KoqAI
17	43476807	A	G	0.021576	0.00391	3.40E-08	0.184348	462346	NA	rs5622038	NA	UKB-b-57	TRUE	reported	8KoqAI
17	43479748	T	C	0.021615	0.003902	3.00E-08	0.185468	462346	NA	rs5579350	NA	UKB-b-57	TRUE	reported	8KoqAI
17	43480701	G	A	0.021613	0.003902	3.10E-08	0.185456	462346	NA	rs6206460	NA	UKB-b-57	TRUE	reported	8KoqAI
17	43483551	C	T	0.021608	0.003903	3.10E-08	0.185515	462346	NA	rs5623691	NA	UKB-b-57	TRUE	reported	8KoqAI
17	43484496	C	T	0.021676	0.003902	2.80E-08	0.18564	462346	NA	rs6206460	NA	UKB-b-57	TRUE	reported	8KoqAI
17	43484598	G	T	0.021947	0.0039	1.80E-08	0.185755	462346	NA	rs7398439	NA	UKB-b-57	TRUE	reported	8KoqAI
17	43485551	T	G	0.021869	0.003902	2.10E-08	0.185607	462346	NA	rs6206463	NA	UKB-b-57	TRUE	reported	8KoqAI
17	43487217	G	A	0.022189	0.0039	1.30E-08	0.18573	462346	NA	rs3607891	NA	UKB-b-57	TRUE	reported	8KoqAI
17	43487424	T	C	0.022302	0.003898	1.10E-08	0.185877	462346	NA	rs3538931	NA	UKB-b-57	TRUE	reported	8KoqAI
17	43487574	C	A	0.022218	0.003906	1.30E-08	0.185097	462346	NA	rs3532713	NA	UKB-b-57	TRUE	reported	8KoqAI
17	43488382	C	T	0.022225	0.003899	1.20E-08	0.185827	462346	NA	rs8071011	NA	UKB-b-57	TRUE	reported	8KoqAI
17	43488792	T	C	0.021949	0.003894	1.70E-08	0.186493	462346	NA	rs6206464	NA	UKB-b-57	TRUE	reported	8KoqAI
17	43490853	G	A	0.022182	0.003902	1.30E-08	0.185157	462346	NA	rs6206464	NA	UKB-b-57	TRUE	reported	8KoqAI
17	43491003	G	A	0.022253	0.003896	1.10E-08	0.185921	462346	NA	rs3406361	NA	UKB-b-57	TRUE	reported	8KoqAI
17	43492357	C	T	0.022254	0.003895	1.10E-08	0.185924	462346	NA	rs1294079	NA	UKB-b-57	TRUE	reported	8KoqAI
17	43493101	G	A	0.022282	0.003895	1.10E-08	0.185905	462346	NA	rs1294771	NA	UKB-b-57	TRUE	reported	8KoqAI
17	43493504	C	T	0.022256	0.003895	1.10E-08	0.185929	462346	NA	rs7137356	NA	UKB-b-57	TRUE	reported	8KoqAI
17	43493560	C	G	0.022257	0.003895	1.10E-08	0.185929	462346	NA	rs3491510	NA	UKB-b-57	TRUE	reported	8KoqAI
17	43493835	T	C	0.022211	0.003895	1.20E-08	0.18594	462346	NA	rs1294295	NA	UKB-b-57	TRUE	reported	8KoqAI
17	43495216	G	A	0.022291	0.003894	1.00E-08	0.185938	462346	NA	rs3588442	NA	UKB-b-57	TRUE	reported	8KoqAI
17	43495852	A	G	0.022253	0.003894	1.10E-08	0.185937	462346	NA	rs3428692	NA	UKB-b-57	TRUE	reported	8KoqAI
17	43496465	T	C	0.022235	0.003893	1.10E-08	0.185969	462346	NA	rs5637863	NA	UKB-b-57	TRUE	reported	8KoqAI
17	43496828	C	T	0.022233	0.003893	1.10E-08	0.185971	462346	NA	rs1294672	NA	UKB-b-57	TRUE	reported	8KoqAI
17	43497210	G	A	0.022221	0.003893	1.10E-08	0.18596	462346	NA	rs1295276	NA	UKB-b-57	TRUE	reported	8KoqAI
17	43498180	C	T	0.022239	0.003892	1.10E-08	0.185977	462346	NA	rs3542058	NA	UKB-b-57	TRUE	reported	8KoqAI
17	43498181	A	G	0.022381	0.00391	1.00E-08	0.184867	462346	NA	rs3479254	NA	UKB-b-57	TRUE	reported	8KoqAI
17	43499328	T	C	0.022116	0.003891	1.30E-08	0.186098	462346	NA	rs3551990	NA	UKB-b-57	TRUE	reported	8KoqAI
17	43499839	A	G	0.022243	0.00389	1.10E-08	0.185988	462346	NA	rs1294266	NA	UKB-b-57	TRUE	reported	8KoqAI
17	43500477	G	A	0.022166	0.003896	1.30E-08	0.18522	462346	NA	rs6206464	NA	UKB-b-57	TRUE	reported	8KoqAI
17	43500587	G	A	0.022325	0.003889	9.40E-09	0.186143	462346	NA	rs6206464	NA	UKB-b-57	TRUE	reported	8KoqAI
17	43500621	G	A	0.022218	0.00389	1.10E-08	0.185988	462346	NA	rs6206464	NA	UKB-b-57	TRUE	reported	8KoqAI
17	43501442	T	C	0.022208	0.00389	1.10E-08	0.186005	462346	NA	rs7222389	NA	UKB-b-57	TRUE	reported	8KoqAI
17	43501591	T	C	0.02226	0.003888	1.00E-08	0.186152	462346	NA	rs5564832	NA	UKB-b-57	TRUE	reported	8KoqAI
17	43501940	G	A	0.022204	0.00389	1.10E-08	0.186004	462346	NA	rs5621210	NA	UKB-b-57	TRUE	reported	8KoqAI
17	43502111	A	G	0.022201	0.003889	1.10E-08	0.186003	462346	NA	rs6206464	NA	UKB-b-57	TRUE	reported	8KoqAI
17	43502241	T	C	0.022167	0.003889	1.20E-08	0.186011	462346	NA	rs3562671	NA	UKB-b-57	TRUE	reported	8KoqAI
17	43503000	G	A	0.022121	0.003895	1.40E-08	0.18524	462346	NA	rs6206465	NA	UKB-b-57	TRUE	reported	8KoqAI
17	43503284	G	A	0.021988	0.003902	1.80E-08	0.184928	462346	NA	rs7634412	NA	UKB-b-57	TRUE	reported	8KoqAI
17	43503294	A	C	0.022188	0.003896	1.20E-08	0.185489	462346	NA	rs7209501	NA	UKB-b-57	TRUE	reported	8KoqAI
17	43507297	C	T	0.022331	0.003894	9.70E-09	0.185383	462346	NA	rs1294925	NA	UKB-b-57	TRUE	reported	8KoqAI
17	43507403	G	A	0.022118	0.003896	1.40E-08	0.18516	462346	NA	rs7220206	NA	UKB-b-57	TRUE	reported	8KoqAI
17	43507649	A	G	0.022236	0.003894	1.10E-08	0.185282	462346	NA	rs7222444	NA	UKB-b-57	TRUE	reported	8KoqAI
17	43508223	G	T	0.02228	0.003896	1.10E-08	0.185193	462346	NA	rs1293918	NA	UKB-b-57	TRUE	reported	8KoqAI
17	43508303	A	G	0.022192	0.003894	1.20E-08	0.185277	462346	NA	rs3401894	NA	UKB-b-57	TRUE	reported	8KoqAI
17	43508616	G	T	0.022205	0.003894	1.20E-08	0.18529	462346	NA	rs3410435	NA	UKB-b-57	TRUE	reported	8KoqAI
17	43509310	A	G	0.0222	0.003894	1.20E-08	0.185292	462346	NA	rs6206465	NA	UKB-b-57	TRUE	reported	8KoqAI
17	43509316	C	T	0.022212	0.003894	1.20E-08	0.185299	462346	NA	rs6206465	NA	UKB-b-57	TRUE	reported	8KoqAI
17	43509778	C	T	0.022175	0.003895	1.20E-08	0.185277	462346	NA	rs5602083	NA	UKB-b-57	TRUE	reported	8KoqAI
17	43510187	G	C	0.022166	0.003895	1.30E-08	0.185276	462346	NA	rs5564294	NA	UKB-b-57	TRUE	reported	8KoqAI
17	43511435	C	A	0.022209	0.003895	1.20E-08	0.185323	462346	NA	rs3446544	NA	UKB-b-57	TRUE	reported	8KoqAI
17	43512206	A	G	0.022217	0.003896	1.20E-08	0.185335	462346	NA	rs1294690	NA	UKB-b-57	TRUE	reported	8KoqAI
17	43512318	G	A	0.022216	0.003896	1.20E-08	0.185337	462346	NA	rs5616893	NA	UKB-b-57	TRUE	reported	8KoqAI
17	43512439	G	C	0.02222	0.003896	1.20E-08	0.185336	462346	NA	rs5579040	NA	UKB-b-57	TRUE	reported	8KoqAI
17	43513441	C	T	0.02222	0.003896	1.20E-08	0.185363	462346	NA	rs11012	NA	UKB-b-57	TRUE	reported	8KoqAI
17	43513551	C	G	0.022237	0.003896	1.10E-08	0.185361	462346	NA	rs9730	NA	UKB-b-57	TRUE	reported	8KoqAI
17	43513896	C	T	0.022195	0.003896	1.20E-08	0.185334	462346	NA	rs6206465	NA	UKB-b-57	TRUE	reported	8KoqAI
17	43514954	G	A	0.022234	0.003896	1.10E-08	0.185371	462346	NA	rs6206465	NA	UKB-b-57	TRUE	reported	8KoqAI
17	43515885	A	G	0.022235	0.003896	1.10E-08	0.185372	462346	NA	rs3611499	NA	UKB-b-57	TRUE	reported	8KoqAI
17	43515927	C	T	0.022235	0.003896	1.10E-08	0.185372	462346	NA	rs3535451	NA	UKB-b-57	TRUE	reported	8KoqAI
17	43516402	A	G	0.022221	0.003896	1.20E-08	0.185349	462346	NA	rs1763130	NA	UKB-b-57	TRUE	reported	8KoqAI
17	43517054	G	A	0.02222	0.003896	1.20E-08	0.185354	462346	NA	rs6206465	NA	UKB-b-57	TRUE	reported	8KoqAI
17	43517252	T	C	0.022262	0.003896	1.10E-08	0.185405	462346	NA	rs3548931	NA	UKB-b-57	TRUE	reported	8KoqAI
17	43520118	A	G	0.023123	0.003958	5.20E-09	0.181254	462346	NA	rs2684641	NA	UKB-b-57	TRUE	reported	8KoqAI
17	43520272	G	A	0.022233	0.003897	1.20E-08	0.185358	462346	NA	rs6206537	NA	UKB-b-57	TRUE	reported	8KoqAI
17	43521161	C	G	0.022234	0.003896	1.20E-08	0.185381	462346	NA	rs6206537	NA	UKB-b-57	TRUE	reported	8KoqAI
17	43521193	A	G	0.022272	0.003896	1.10E-08	0.185393	462346	NA	rs6206537	NA	UKB-b-57	TRUE	reported	8KoqAI
17	43522361	T	C	0.022158	0.003896	1.30E-08	0.18551	462346	NA	rs6206537	NA	UKB-b-57	TRUE	reported	8KoqAI
17	43524526	C	T	0.022134	0.003897	1.30E-08	0.185504	462346	NA	rs1135750	NA	UKB-b-57	TRUE	reported	8KoqAI

17	43525022	G	A	0.022324	0.003897	1.00E-08	0.185407	462346	NA	rs7137357	NA	UKB-b-57	TRUE	reported	8KoqAI
17	43525365	C	T	0.022181	0.003897	1.30E-08	0.185489	462346	NA	rs6206537	NA	UKB-b-57	TRUE	reported	8KoqAI
17	43527323	C	T	0.022234	0.003897	1.20E-08	0.18552	462346	NA	rs1125384	NA	UKB-b-57	TRUE	reported	8KoqAI
17	43529293	G	A	0.022406	0.003897	8.90E-09	0.185403	462346	NA	rs2077606	NA	UKB-b-57	TRUE	reported	8KoqAI
17	43534353	T	C	0.021762	0.003878	2.00E-08	0.188025	462346	NA	rs2960000	NA	UKB-b-57	TRUE	reported	8KoqAI
17	43534694	C	T	0.022327	0.003897	1.00E-08	0.185457	462346	NA	rs6206538	NA	UKB-b-57	TRUE	reported	8KoqAI
17	43536408	C	T	0.022412	0.003897	8.90E-09	0.185433	462346	NA	rs5570388	NA	UKB-b-57	TRUE	reported	8KoqAI
17	43536743	C	T	0.022412	0.003897	8.90E-09	0.185438	462346	NA	rs5600571	NA	UKB-b-57	TRUE	reported	8KoqAI
17	43538523	G	A	0.022344	0.003898	9.90E-09	0.18539	462346	NA	rs1114236	NA	UKB-b-57	TRUE	reported	8KoqAI
17	43538807	T	G	0.02239	0.003897	9.20E-09	0.185409	462346	NA	rs6206538	NA	UKB-b-57	TRUE	reported	8KoqAI
17	43539968	G	A	0.022426	0.003897	8.70E-09	0.185429	462346	NA	rs4325608	NA	UKB-b-57	TRUE	reported	8KoqAI
17	43541627	C	A	0.022439	0.003897	8.50E-09	0.185435	462346	NA	rs2139890	NA	UKB-b-57	TRUE	reported	8KoqAI
17	43541656	C	T	0.022332	0.003897	1.00E-08	0.185463	462346	NA	rs3946526	NA	UKB-b-57	TRUE	reported	8KoqAI
17	43544379	G	A	0.022809	0.003894	4.70E-09	0.185191	462346	NA	rs5566379	NA	UKB-b-57	TRUE	reported	8KoqAI
17	43545893	T	C	0.02277	0.003894	5.00E-09	0.185136	462346	NA	rs1879581	NA	UKB-b-57	TRUE	reported	8KoqAI
17	43548424	A	G	0.02268	0.003895	5.80E-09	0.185173	462346	NA	rs5567131	NA	UKB-b-57	TRUE	reported	8KoqAI
17	43548481	A	G	0.022778	0.003895	5.00E-09	0.185127	462346	NA	rs5565215	NA	UKB-b-57	TRUE	reported	8KoqAI
17	43549526	A	G	0.022764	0.003895	5.10E-09	0.185184	462346	NA	rs1763167	NA	UKB-b-57	TRUE	reported	8KoqAI
17	43549608	G	A	0.022728	0.003895	5.40E-09	0.185152	462346	NA	rs1493664	NA	UKB-b-57	TRUE	reported	8KoqAI
17	43550107	A	G	0.022775	0.003895	5.00E-09	0.185182	462346	NA	rs2090847	NA	UKB-b-57	TRUE	reported	8KoqAI
17	43551083	C	T	0.022682	0.003897	5.90E-09	0.185007	462346	NA	rs5574686	NA	UKB-b-57	TRUE	reported	8KoqAI
17	43551151	C	G	0.022788	0.003895	4.90E-09	0.185181	462346	NA	rs1295096	NA	UKB-b-57	TRUE	reported	8KoqAI
17	43551321	G	C	0.022766	0.003895	5.00E-09	0.185206	462346	NA	rs5601579	NA	UKB-b-57	TRUE	reported	8KoqAI
17	43551613	C	T	0.02277	0.003895	5.00E-09	0.185206	462346	NA	rs6206540	NA	UKB-b-57	TRUE	reported	8KoqAI
17	43552537	G	A	0.022781	0.003895	4.90E-09	0.185205	462346	NA	rs7123884	NA	UKB-b-57	TRUE	reported	8KoqAI
17	43552717	G	C	0.022804	0.003895	4.80E-09	0.185171	462346	NA	rs1245207	NA	UKB-b-57	TRUE	reported	8KoqAI
17	43552921	C	T	0.022806	0.003895	4.80E-09	0.185153	462346	NA	rs1245227	NA	UKB-b-57	TRUE	reported	8KoqAI
17	43555253	A	G	0.022721	0.003895	5.40E-09	0.185262	462346	NA	rs5619275	NA	UKB-b-57	TRUE	reported	8KoqAI
17	43556652	G	A	0.022793	0.003896	4.90E-09	0.185108	462346	NA	rs6206543	NA	UKB-b-57	TRUE	reported	8KoqAI
17	43556807	T	C	0.022677	0.003889	5.50E-09	0.185997	462346	NA	rs5592554	NA	UKB-b-57	TRUE	reported	8KoqAI
17	43556982	G	A	0.022836	0.003895	4.60E-09	0.185145	462346	NA	rs5564351	NA	UKB-b-57	TRUE	reported	8KoqAI
17	43557612	G	A	0.022838	0.003896	4.60E-09	0.185138	462346	NA	rs6206543	NA	UKB-b-57	TRUE	reported	8KoqAI
17	43558092	C	T	0.022793	0.003896	4.90E-09	0.185104	462346	NA	rs6206543	NA	UKB-b-57	TRUE	reported	8KoqAI
17	43563894	T	C	0.022893	0.003896	4.20E-09	0.185158	462346	NA	rs6206544	NA	UKB-b-57	TRUE	reported	8KoqAI
17	43564222	T	G	0.02272	0.003896	5.50E-09	0.185223	462346	NA	rs9899111	NA	UKB-b-57	TRUE	reported	8KoqAI
17	43565840	G	A	0.02291	0.003897	4.10E-09	0.185196	462346	NA	rs6206544	NA	UKB-b-57	TRUE	reported	8KoqAI
17	43567175	T	G	0.022899	0.003899	4.30E-09	0.185	462346	NA	rs1879585	NA	UKB-b-57	TRUE	reported	8KoqAI
17	43569245	T	A	0.022802	0.003898	4.90E-09	0.185065	462346	NA	rs1133228	NA	UKB-b-57	TRUE	reported	8KoqAI
17	43569770	C	T	0.023127	0.003927	3.90E-09	0.183787	462346	NA	rs6206544	NA	UKB-b-57	TRUE	reported	8KoqAI
17	43569909	T	C	0.0226	0.003896	6.60E-09	0.185401	462346	NA	rs6206544	NA	UKB-b-57	TRUE	reported	8KoqAI
17	43570407	C	T	0.02243	0.003896	8.50E-09	0.185458	462346	NA	rs6206545	NA	UKB-b-57	TRUE	reported	8KoqAI
17	43570893	C	T	0.022605	0.003896	6.50E-09	0.185411	462346	NA	rs1879583	NA	UKB-b-57	TRUE	reported	8KoqAI
17	43572419	C	T	0.02373	0.003624	5.90E-11	0.226101	462346	NA	rs5632822	NA	UKB-b-57	TRUE	reported	8KoqAI
17	43573419	C	T	0.023876	0.003633	5.00E-11	0.225283	462346	NA	rs6206545	NA	UKB-b-57	TRUE	reported	8KoqAI
17	43581752	T	C	-0.02097	0.003729	1.90E-08	0.698492	462346	NA	rs1056009	NA	UKB-b-57	TRUE	reported	8KoqAI
17	43656627	T	C	0.023915	0.003831	4.30E-10	0.203156	462346	NA	rs2463518	NA	UKB-b-57	TRUE	reported	8KoqAI
17	43656895	G	A	0.024301	0.003845	2.60E-10	0.202104	462346	NA	rs2463519	NA	UKB-b-57	TRUE	reported	8KoqAI
17	43657257	C	T	0.023587	0.003809	5.90E-10	0.204875	462346	NA	rs7974262	NA	UKB-b-57	TRUE	reported	8KoqAI
17	43658516	C	T	0.023682	0.003849	7.60E-10	0.201841	462346	NA	rs1724433	NA	UKB-b-57	TRUE	reported	8KoqAI
17	43659975	T	C	0.022812	0.003692	6.50E-10	0.212748	462346	NA	rs2668680	NA	UKB-b-57	TRUE	reported	8KoqAI
17	43662020	A	G	0.02379	0.003842	6.00E-10	0.201678	462346	NA	rs1724429	NA	UKB-b-57	TRUE	reported	8KoqAI
17	43663247	C	A	0.023393	0.003617	1.00E-10	0.226462	462346	NA	rs1724390	NA	UKB-b-57	TRUE	reported	8KoqAI
17	43666906	G	C	0.02388	0.003625	4.40E-11	0.225242	462346	NA	rs2696425	NA	UKB-b-57	TRUE	reported	8KoqAI
17	43669931	T	C	0.023845	0.003605	3.70E-11	0.229147	462346	NA	rs1724411	NA	UKB-b-57	TRUE	reported	8KoqAI
17	43670696	G	C	0.024515	0.003722	4.50E-11	0.215905	462346	NA	rs1706731	NA	UKB-b-57	TRUE	reported	8KoqAI
17	43671099	C	A	0.024161	0.003635	3.00E-11	0.224121	462346	NA	rs2668643	NA	UKB-b-57	TRUE	reported	8KoqAI
17	43671436	A	G	0.03703	0.006577	1.80E-08	0.089981	462346	NA	rs2532373	NA	UKB-b-57	TRUE	reported	8KoqAI
17	43685826	A	G	0.024074	0.003634	3.50E-11	0.22427	462346	NA	rs413519	NA	UKB-b-57	TRUE	reported	8KoqAI
17	43686420	A	G	0.024556	0.00364	1.50E-11	0.22353	462346	NA	rs440778	NA	UKB-b-57	TRUE	reported	8KoqAI
17	43688317	A	G	0.024015	0.003617	3.10E-11	0.226304	462346	NA	rs2876897	NA	UKB-b-57	TRUE	reported	8KoqAI
17	43691377	T	C	0.02571	0.003974	9.80E-11	0.197407	462346	NA	rs382362	NA	UKB-b-57	TRUE	reported	8KoqAI
17	43692935	A	G	0.024039	0.003624	3.30E-11	0.224976	462346	NA	rs611351	NA	UKB-b-57	TRUE	reported	8KoqAI
17	43693538	G	T	0.024171	0.003624	2.50E-11	0.22497	462346	NA	rs418891	NA	UKB-b-57	TRUE	reported	8KoqAI
17	43705356	G	A	0.024145	0.003623	2.70E-11	0.224845	462346	NA	rs383241	NA	UKB-b-57	TRUE	reported	8KoqAI
17	43705518	A	G	0.024144	0.003623	2.70E-11	0.224845	462346	NA	rs376120	NA	UKB-b-57	TRUE	reported	8KoqAI
17	43705601	A	G	0.024001	0.003616	3.20E-11	0.226167	462346	NA	rs365825	NA	UKB-b-57	TRUE	reported	8KoqAI
17	43705756	G	C	0.024116	0.003616	2.60E-11	0.226091	462346	NA	rs393838	NA	UKB-b-57	TRUE	reported	8KoqAI
17	43707619	C	A	0.024147	0.003623	2.60E-11	0.224994	462346	NA	rs681485	NA	UKB-b-57	TRUE	reported	8KoqAI
17	43707620	G	T	0.024143	0.003623	2.70E-11	0.224846	462346	NA	rs594591	NA	UKB-b-57	TRUE	reported	8KoqAI
17	43707951	C	T	0.024125	0.003623	2.80E-11	0.224836	462346	NA	rs444071	NA	UKB-b-57	TRUE	reported	8KoqAI
17	43707955	C	A	0.024125	0.003623	2.80E-11	0.224836	462346	NA	rs396593	NA	UKB-b-57	TRUE	reported	8KoqAI
17	43709415	C	T	0.02412	0.003623	2.80E-11	0.224876	462346	NA	rs436667	NA	UKB-b-57	TRUE	reported	8KoqAI
17	43709867	T	G	0.024061	0.003623	3.10E-11	0.224825	462346	NA	rs650927	NA	UKB-b-57	TRUE	reported	8KoqAI
17	43709893	A	G	0.024061	0.003623	3.10E-11	0.224825	462346	NA	rs671740	NA	UKB-b-57	TRUE	reported	8KoqAI
17	43710127	T	A	0.024066	0.003624	3.10E-11	0.224814	462346	NA	rs81632	NA	UKB-b-57	TRUE	reported	8KoqAI
17	43710371	G	A	0.024087	0.003624	3.00E-11	0.224813	462346	NA	rs1694063	NA	UKB-b-57	TRUE	reported	8KoqAI
17	43710384	A	C	0.024075	0.003624	3.10E-11	0.224816	462346	NA	rs1768661	NA	UKB-b-57	TRUE	reported	8KoqAI
17	43711312	C	T	0.024061	0.003623	3.10E-11	0.224825	462346	NA	rs2942170	NA	UKB-b-57	TRUE	reported	8KoqAI
17	43711539	G	C	0.024102	0.003623	2.90E-11	0.224847	462346	NA	rs2942169	NA	UKB-b-57	TRUE	reported	8KoqAI
17	43712337	C	T	0.024101	0.003623	2.90E-11	0.224847	462346	NA	rs241045	NA	UKB-b-57	TRUE	reported	8KoqAI
17	43712464	G	A	0.024101	0.003623	2.90E-11	0.224847	462346	NA	rs241044	NA	UKB-b-57	TRUE	reported	8KoqAI
17	43712657	T	C	0.024101	0.003623	2.90E-11	0.224847	462346	NA	rs241042	NA	UKB-b-57	TRUE	reported	8KoqAI
17	43713925	A	G	0.024103	0.003623	2.90E-11	0.224847	462346	NA	rs241041	NA	UKB-b-57	TRUE	reported	8KoqAI
17	43714673	A	T	0.024103	0.003623	2.90E-11	0.224847	462346	NA	rs241039	NA	UKB-b-57	TRUE	reported	8KoqAI
17	43714850	G	A	0.024099	0.003623	2.90E-11	0.22485	462346	NA	rs2942168	NA	UKB-b-57	TRUE	reported	8KoqAI
17	43715018	T	C	0.024101	0.003623	2.90E-11	0.224847	462346	NA	rs2942167	NA	UKB-b-57	TRUE	reported	8KoqAI
17	43715427	T	C	0.02405	0.003622	3.10E-11	0.225081	462346	NA	rs2942166	NA	UKB-b-57	TRUE	reported	8KoqAI
17	43715619	G	C	0.024101	0.003623	2.90E-11	0.224847	462346	NA	rs2942165	NA	UKB-b-57	TRUE	reported	8KoqAI
17	43715924	A	G	0.024115	0.003623	2.80E-11	0.224848	462346	NA	rs1173681	NA	UKB-b-57	TRUE	reported	8KoqAI
17	43716885	A	G	0.024101	0.003623	2.90E-11	0.224847	462346	NA	rs413778	NA	UKB-b-57	TRUE	reported	8KoqAI
17	43717131	C	T	0.024103	0.003623	2.90E-11	0.224846	462346	NA	rs389217	NA	UKB-b-57	TRUE	reported	8KoqAI
17	43717415	A	G	0.023918	0.003623	4.00E-11	0.22499	462346	NA	rs389978	NA	UKB-b-57	TRUE	reported	8KoqAI
17	43717803	T	C	0.024161	0.003623	2.60E-11	0.224988	462346	NA	rs439558	NA	UKB-b-57	TRUE	reported	8KoqAI
17	43718336	T	C	0.024102	0.003616	2.60E-11	0.226069	462346	NA	rs434805	NA	UKB-b-57	TRUE	reported	8KoqAI
17	43719143	A	G	0.02421	0.003616	2.10E-11	0.226318	462346	NA	rs393152	NA	UKB-b-57	TRUE	reported	8KoqAI
17	43719870	G	A	0.024111	0.003623	2.80E-11	0.224847	462346	NA	rs3108317	NA	UKB-b-57	TRUE	reported	8KoqAI
17	43721283	G	C	0.024147	0.003623	2.70E-11	0.224898	462346	NA	rs2942164	NA	UKB-b-57	TRUE	reported	8KoqAI
17	43721457	C	T	0.024128	0.003623	2.80E-11	0.224846	462346	NA	rs1706746	NA	UKB-b-57	TRUE	reported	8KoqAI
17	43722509	C	G	0.024125	0.003623	2.80E-11	0.224896	462346	NA	rs1706748	NA	UKB-b-57	TRUE	reported	8KoqAI
17	43723189	G	A	0.024284	0.003623	2.00E-11	0.224942	462346	NA	rs413917	NA	UKB-b-57	TRUE	reported	8KoqAI
17	43723462	C	T	0.024129	0.003623	2.70E-11	0.224846	462346	NA	rs3418	NA	UKB-b-57	TRUE	reported	8KoqAI
17	43723605	T	C	0.024116	0.003623	2.80E-11	0.224844	462346	NA	rs393675	NA	UKB-b-57	TRUE	reported	8KoqAI
17	43724555	G	A	0.02413	0.003623	2.70E-11	0.224847	462346	NA	rs449501	NA	UKB-b-57	TRUE	reported	8KoqAI
17	43725212	G	A	0.024131	0.003623	2.70E-11	0.224845	462346	NA	rs448830	NA	UKB-b-57	TRUE	reported	8KoqAI
17	43725684	G	A	0.024131	0.003623	2.70E-11	0.224845	462346	NA	rs434428	NA	UKB-b-57	TRUE	reported	8KoqAI
17	43725762	G	A	0.024131	0.003623	2.70E-11	0.224845	462346	NA	rs434598	NA	UKB-b-57	TRUE	reported	8KoqAI
17	43725992	G	A	0.024139	0.003623	2.70E-11	0.224846	462346	NA	rs434971	NA	UKB-b-57	TRUE	reported	8KoqAI

17	43726125	A	C	0.024135	0.003623	2.70E-11	0.224846	462346	NA	rs385691	NA	UKB-b-57	TRUE	reported	8KoqAI
17	43726574	T	C	0.02413	0.003623	2.70E-11	0.224846	462346	NA	rs455028	NA	UKB-b-57	TRUE	reported	8KoqAI
17	43726588	C	T	0.024136	0.003623	2.70E-11	0.224834	462346	NA	rs366858	NA	UKB-b-57	TRUE	reported	8KoqAI
17	43726659	A	C	-0.02393	0.00346	4.60E-12	0.742382	462346	NA	rs439945	NA	UKB-b-57	TRUE	reported	8KoqAI
17	43727061	C	T	0.02411	0.003623	2.90E-11	0.224829	462346	NA	rs453997	NA	UKB-b-57	TRUE	reported	8KoqAI
17	43727887	A	T	0.024106	0.003623	2.90E-11	0.22484	462346	NA	rs424243	NA	UKB-b-57	TRUE	reported	8KoqAI
17	43728137	G	A	0.024101	0.003623	2.90E-11	0.224831	462346	NA	rs422112	NA	UKB-b-57	TRUE	reported	8KoqAI
17	43728376	G	A	-0.02383	0.003459	5.70E-12	0.742138	462346	NA	rs417968	NA	UKB-b-57	TRUE	reported	8KoqAI
17	43729384	G	A	0.024098	0.003623	2.90E-11	0.224831	462346	NA	rs413844	NA	UKB-b-57	TRUE	reported	8KoqAI
17	43729432	C	T	0.024099	0.003623	2.90E-11	0.224834	462346	NA	rs647483	NA	UKB-b-57	TRUE	reported	8KoqAI
17	43730598	G	A	0.024098	0.003623	2.90E-11	0.224832	462346	NA	rs241038	NA	UKB-b-57	TRUE	reported	8KoqAI
17	43730636	G	T	0.024098	0.003623	2.90E-11	0.224832	462346	NA	rs241037	NA	UKB-b-57	TRUE	reported	8KoqAI
17	43731719	A	C	0.024202	0.003623	2.40E-11	0.225283	462346	NA	rs241036	NA	UKB-b-57	TRUE	reported	8KoqAI
17	43731896	C	T	0.024113	0.003623	2.80E-11	0.224848	462346	NA	rs241035	NA	UKB-b-57	TRUE	reported	8KoqAI
17	43733048	A	T	0.024116	0.003623	2.80E-11	0.224848	462346	NA	rs241034	NA	UKB-b-57	TRUE	reported	8KoqAI
17	43733983	G	A	0.024151	0.003623	2.60E-11	0.224861	462346	NA	rs241033	NA	UKB-b-57	TRUE	reported	8KoqAI
17	43734145	T	C	0.024039	0.003623	3.30E-11	0.224944	462346	NA	rs241032	NA	UKB-b-57	TRUE	reported	8KoqAI
17	43734304	T	C	0.024119	0.003623	2.80E-11	0.224846	462346	NA	rs241031	NA	UKB-b-57	TRUE	reported	8KoqAI
17	43734503	A	G	0.024119	0.003623	2.80E-11	0.224847	462346	NA	rs241030	NA	UKB-b-57	TRUE	reported	8KoqAI
17	43734643	T	C	0.024118	0.003623	2.80E-11	0.224843	462346	NA	rs241029	NA	UKB-b-57	TRUE	reported	8KoqAI
17	43735276	C	T	0.024108	0.003623	2.90E-11	0.224843	462346	NA	rs241028	NA	UKB-b-57	TRUE	reported	8KoqAI
17	43735478	A	G	0.024125	0.003623	2.80E-11	0.224849	462346	NA	rs241027	NA	UKB-b-57	TRUE	reported	8KoqAI
17	43735555	G	A	0.024124	0.003623	2.80E-11	0.224846	462346	NA	rs241026	NA	UKB-b-57	TRUE	reported	8KoqAI
17	43736763	C	T	0.024127	0.003623	2.80E-11	0.224845	462346	NA	rs241024	NA	UKB-b-57	TRUE	reported	8KoqAI
17	43737040	G	A	0.024127	0.003623	2.80E-11	0.224845	462346	NA	rs241023	NA	UKB-b-57	TRUE	reported	8KoqAI
17	43737730	T	C	0.024131	0.003623	2.70E-11	0.224844	462346	NA	rs241022	NA	UKB-b-57	TRUE	reported	8KoqAI
17	43738627	T	G	0.024132	0.003623	2.70E-11	0.224845	462346	NA	rs241021	NA	UKB-b-57	TRUE	reported	8KoqAI
17	43738676	C	A	0.024132	0.003623	2.70E-11	0.224845	462346	NA	rs241020	NA	UKB-b-57	TRUE	reported	8KoqAI
17	43740565	G	T	-0.0241	0.003464	3.50E-12	0.743147	462346	NA	rs1724409	NA	UKB-b-57	TRUE	reported	8KoqAI
17	43740856	C	T	0.024148	0.003623	2.60E-11	0.224891	462346	NA	rs6205393	NA	UKB-b-57	TRUE	reported	8KoqAI
17	43740967	C	T	0.024137	0.003623	2.70E-11	0.224844	462346	NA	rs1112731	NA	UKB-b-57	TRUE	reported	8KoqAI
17	43741028	A	G	0.024137	0.003623	2.70E-11	0.224844	462346	NA	rs6205394	NA	UKB-b-57	TRUE	reported	8KoqAI
17	43741452	C	T	0.02414	0.003623	2.70E-11	0.224843	462346	NA	rs1776057	NA	UKB-b-57	TRUE	reported	8KoqAI
17	43743045	T	C	0.024149	0.003623	2.60E-11	0.224842	462346	NA	rs1776063	NA	UKB-b-57	TRUE	reported	8KoqAI
17	43744344	T	A	-0.02338	0.003505	2.50E-11	0.75629	462346	NA	rs1635298	NA	UKB-b-57	TRUE	reported	8KoqAI
17	43744990	C	T	0.024135	0.003624	2.80E-11	0.22479	462346	NA	rs1768746	NA	UKB-b-57	TRUE	reported	8KoqAI
17	43746276	G	T	0.024138	0.003623	2.70E-11	0.224841	462346	NA	rs1776073	NA	UKB-b-57	TRUE	reported	8KoqAI
17	43746418	T	A	0.024137	0.003623	2.70E-11	0.224841	462346	NA	rs7967510	NA	UKB-b-57	TRUE	reported	8KoqAI
17	43746721	A	G	0.024136	0.003623	2.70E-11	0.224841	462346	NA	rs1768750	NA	UKB-b-57	TRUE	reported	8KoqAI
17	43746839	T	C	0.024148	0.003623	2.70E-11	0.224843	462346	NA	rs6205394	NA	UKB-b-57	TRUE	reported	8KoqAI
17	43747615	C	T	-0.02309	0.003514	5.00E-11	0.754031	462346	NA	rs1724407	NA	UKB-b-57	TRUE	reported	8KoqAI
17	43747677	C	T	0.024134	0.003623	2.70E-11	0.224842	462346	NA	rs6205394	NA	UKB-b-57	TRUE	reported	8KoqAI
17	43747683	G	A	0.024134	0.003623	2.70E-11	0.224842	462346	NA	rs6205394	NA	UKB-b-57	TRUE	reported	8KoqAI
17	43748046	C	T	0.024131	0.003623	2.70E-11	0.22486	462346	NA	rs6205394	NA	UKB-b-57	TRUE	reported	8KoqAI
17	43748101	A	G	0.024133	0.003623	2.70E-11	0.224842	462346	NA	rs6205394	NA	UKB-b-57	TRUE	reported	8KoqAI
17	43748412	A	G	0.02413	0.003623	2.70E-11	0.224844	462346	NA	rs6205395	NA	UKB-b-57	TRUE	reported	8KoqAI
17	43749168	G	C	0.024131	0.003623	2.70E-11	0.224842	462346	NA	rs6205395	NA	UKB-b-57	TRUE	reported	8KoqAI
17	43749261	T	C	0.024143	0.003623	2.70E-11	0.224845	462346	NA	rs6205395	NA	UKB-b-57	TRUE	reported	8KoqAI
17	43749454	G	A	0.024133	0.003623	2.70E-11	0.224895	462346	NA	rs6205395	NA	UKB-b-57	TRUE	reported	8KoqAI
17	43749579	T	C	0.024142	0.003623	2.70E-11	0.224845	462346	NA	rs1768753	NA	UKB-b-57	TRUE	reported	8KoqAI
17	43749942	A	G	-0.02322	0.003514	3.90E-11	0.753915	462346	NA	rs1631850	NA	UKB-b-57	TRUE	reported	8KoqAI
17	43749949	G	A	0.02419	0.003623	2.50E-11	0.224855	462346	NA	rs6205395	NA	UKB-b-57	TRUE	reported	8KoqAI
17	43749980	T	C	0.024186	0.003623	2.50E-11	0.224862	462346	NA	rs6205395	NA	UKB-b-57	TRUE	reported	8KoqAI
17	43750010	G	A	0.024139	0.003623	2.70E-11	0.224846	462346	NA	rs1768757	NA	UKB-b-57	TRUE	reported	8KoqAI
17	43750137	G	C	0.024139	0.003623	2.70E-11	0.224847	462346	NA	rs1776081	NA	UKB-b-57	TRUE	reported	8KoqAI
17	43750172	G	A	-0.02312	0.003515	4.80E-11	0.753959	462346	NA	rs1630095	NA	UKB-b-57	TRUE	reported	8KoqAI
17	43750238	A	G	0.024186	0.003623	2.50E-11	0.224863	462346	NA	rs1768762	NA	UKB-b-57	TRUE	reported	8KoqAI
17	43750315	A	G	0.024176	0.003623	2.50E-11	0.22487	462346	NA	rs6205395	NA	UKB-b-57	TRUE	reported	8KoqAI
17	43750910	C	T	-0.02308	0.003513	5.00E-11	0.754006	462346	NA	rs4792824	NA	UKB-b-57	TRUE	reported	8KoqAI
17	43750939	T	C	0.02413	0.003623	2.70E-11	0.224854	462346	NA	rs6205395	NA	UKB-b-57	TRUE	reported	8KoqAI
17	43751913	G	A	-0.02281	0.003513	8.50E-11	0.753523	462346	NA	rs1635291	NA	UKB-b-57	TRUE	reported	8KoqAI
17	43752039	C	T	0.024129	0.003623	2.70E-11	0.224877	462346	NA	rs6205566	NA	UKB-b-57	TRUE	reported	8KoqAI
17	43752566	C	T	0.024143	0.003623	2.70E-11	0.224848	462346	NA	rs6205566	NA	UKB-b-57	TRUE	reported	8KoqAI
17	43753454	A	G	0.024143	0.003623	2.70E-11	0.224849	462346	NA	rs6205566	NA	UKB-b-57	TRUE	reported	8KoqAI
17	43753642	A	G	0.024143	0.003623	2.70E-11	0.224849	462346	NA	rs6205566	NA	UKB-b-57	TRUE	reported	8KoqAI
17	43754010	T	C	0.024142	0.003623	2.70E-11	0.224849	462346	NA	rs7671781	NA	UKB-b-57	TRUE	reported	8KoqAI
17	43754099	G	A	0.024142	0.003623	2.70E-11	0.224851	462346	NA	rs1768766	NA	UKB-b-57	TRUE	reported	8KoqAI
17	43754209	G	C	0.024142	0.003623	2.70E-11	0.22485	462346	NA	rs7907378	NA	UKB-b-57	TRUE	reported	8KoqAI
17	43754505	A	C	0.024141	0.003623	2.70E-11	0.224851	462346	NA	rs6205566	NA	UKB-b-57	TRUE	reported	8KoqAI
17	43754569	G	A	0.024151	0.003623	2.60E-11	0.22486	462346	NA	rs6205568	NA	UKB-b-57	TRUE	reported	8KoqAI
17	43754576	C	G	0.024142	0.003623	2.70E-11	0.224851	462346	NA	rs6205569	NA	UKB-b-57	TRUE	reported	8KoqAI
17	43754850	T	C	0.024142	0.003623	2.70E-11	0.224852	462346	NA	rs6205569	NA	UKB-b-57	TRUE	reported	8KoqAI
17	43755857	C	A	-0.02296	0.003513	6.30E-11	0.753957	462346	NA	rs1724401	NA	UKB-b-57	TRUE	reported	8KoqAI
17	43755871	A	C	-0.02296	0.003513	6.30E-11	0.753957	462346	NA	rs1724400	NA	UKB-b-57	TRUE	reported	8KoqAI
17	43756340	T	C	0.024128	0.003623	2.80E-11	0.224862	462346	NA	rs7439825	NA	UKB-b-57	TRUE	reported	8KoqAI
17	43756376	G	C	0.024139	0.003623	2.70E-11	0.224917	462346	NA	rs1768774	NA	UKB-b-57	TRUE	reported	8KoqAI
17	43756458	G	A	0.024291	0.003634	2.30E-11	0.223996	462346	NA	rs872972	NA	UKB-b-57	TRUE	reported	8KoqAI
17	43756466	T	C	0.024195	0.003633	2.70E-11	0.224085	462346	NA	rs757503	NA	UKB-b-57	TRUE	reported	8KoqAI
17	43756506	C	T	0.024166	0.003624	2.60E-11	0.224828	462346	NA	rs757502	NA	UKB-b-57	TRUE	reported	8KoqAI
17	43756685	A	G	0.024066	0.003624	3.10E-11	0.224822	462346	NA	rs757501	NA	UKB-b-57	TRUE	reported	8KoqAI
17	43756833	C	A	0.02412	0.003623	2.80E-11	0.224839	462346	NA	rs757500	NA	UKB-b-57	TRUE	reported	8KoqAI
17	43756910	T	C	0.024083	0.003624	3.00E-11	0.224821	462346	NA	rs6205569	NA	UKB-b-57	TRUE	reported	8KoqAI
17	43756969	A	G	-0.02298	0.003507	5.60E-11	0.752118	462346	NA	rs1635288	NA	UKB-b-57	TRUE	reported	8KoqAI
17	43757161	C	T	0.024114	0.003623	2.80E-11	0.224832	462346	NA	rs7848784	NA	UKB-b-57	TRUE	reported	8KoqAI
17	43757450	C	A	0.024006	0.003673	6.30E-11	0.21938	462346	NA	rs5597401	NA	UKB-b-57	TRUE	reported	8KoqAI
17	43757776	T	C	0.024088	0.003624	3.00E-11	0.224773	462346	NA	rs5610811	NA	UKB-b-57	TRUE	reported	8KoqAI
17	43757777	C	T	0.024082	0.003624	3.00E-11	0.224773	462346	NA	rs5637802	NA	UKB-b-57	TRUE	reported	8KoqAI
17	43758011	G	C	0.024098	0.003623	2.90E-11	0.22484	462346	NA	rs5565162	NA	UKB-b-57	TRUE	reported	8KoqAI
17	43758078	G	A	0.024107	0.003623	2.90E-11	0.224837	462346	NA	rs1121553	NA	UKB-b-57	TRUE	reported	8KoqAI
17	43758148	C	T	0.024102	0.003623	2.90E-11	0.224845	462346	NA	rs6205569	NA	UKB-b-57	TRUE	reported	8KoqAI
17	43758238	C	T	0.024063	0.003623	3.10E-11	0.224905	462346	NA	rs6205569	NA	UKB-b-57	TRUE	reported	8KoqAI
17	43758382	T	C	0.024074	0.003623	3.00E-11	0.224852	462346	NA	rs1776104	NA	UKB-b-57	TRUE	reported	8KoqAI
17	43758742	T	C	0.024067	0.003624	3.10E-11	0.224819	462346	NA	rs6205570	NA	UKB-b-57	TRUE	reported	8KoqAI
17	43758787	G	A	0.023909	0.003645	5.40E-11	0.222671	462346	NA	rs6205570	NA	UKB-b-57	TRUE	reported	8KoqAI
17	43758790	C	T	0.023922	0.003645	5.30E-11	0.222664	462346	NA	rs6205570	NA	UKB-b-57	TRUE	reported	8KoqAI
17	43758885	A	G	0.024091	0.003647	4.00E-11	0.223042	462346	NA	rs1124319	NA	UKB-b-57	TRUE	reported	8KoqAI
17	43758898	A	G	0.023005	0.003537	7.80E-11	0.243908	462346	NA	rs5722298	NA	UKB-b-57	TRUE	reported	8KoqAI
17	43759048	T	C	0.024099	0.003623	2.90E-11	0.224842	462346	NA	rs1768783	NA	UKB-b-57	TRUE	reported	8KoqAI
17	43759426	C	T	0.024103	0.003623	2.90E-11	0.224846	462346	NA	rs7633392	NA	UKB-b-57	TRUE	reported	8KoqAI
17	43759431	C	T	0.024116	0.003623	2.80E-11	0.224854	462346	NA	rs7645392	NA	UKB-b-57	TRUE	reported	8KoqAI
17	43759719	A	G	0.024094	0.003623	2.90E-11	0.224853	462346	NA	rs1768784	NA	UKB-b-57	TRUE	reported	8KoqAI
17	43760080	C	A	0.024022	0.003624	3.40E-11	0.22473	462346	NA	rs5640082	NA	UKB-b-57	TRUE	reported	8KoqAI
17	43760192	A	G	0.024091	0.003623	3.00E-11	0.224846	462346	NA	rs1121694	NA	UKB-b-57	TRUE	reported	8KoqAI
17	43760199	C	T	0.024098	0.003623	2.90E-11	0.224849	462346	NA	rs1137283	NA	UKB-b-57	TRUE	reported	8KoqAI
17	43760389	A	G	0.024103	0.003623	2.90E-11	0.224847	462346	NA	rs1776110	NA	UKB-b-57	TRUE	reported	8KoqAI
17	43760516	A	T	0.024102	0.003623	2.90E-11	0.224847	462346	NA	rs1776112	NA	UKB-b-57	TRUE	reported	8KoqAI
17	43760577	G	A	0.024103	0.003623	2.90E-11	0.224847	462346	NA	rs6205570	NA	UKB-b-57	TRUE	reported	8KoqAI

17	43760599	A	C	0.024102	0.003623	2.90E-11	0.224847	462346	NA	rs6205570	NA	UKB-b-57	TRUE	reported	8KoqAI
17	43760685	T	C	0.024101	0.003623	2.90E-11	0.224846	462346	NA	rs6205570	NA	UKB-b-57	TRUE	reported	8KoqAI
17	43761381	A	G	0.024101	0.003623	2.90E-11	0.224846	462346	NA	rs7511509	NA	UKB-b-57	TRUE	reported	8KoqAI
17	43761482	T	C	0.024101	0.003623	2.90E-11	0.224846	462346	NA	rs6205570	NA	UKB-b-57	TRUE	reported	8KoqAI
17	43761519	T	G	0.022987	0.003517	6.30E-11	0.245651	462346	NA	rs1694103	NA	UKB-b-57	TRUE	reported	8KoqAI
17	43761565	T	C	0.022971	0.003517	6.50E-11	0.245642	462346	NA	rs1694103	NA	UKB-b-57	TRUE	reported	8KoqAI
17	43761856	C	T	0.02409	0.003623	3.00E-11	0.224844	462346	NA	rs2049515	NA	UKB-b-57	TRUE	reported	8KoqAI
17	43761939	G	C	0.024098	0.003623	2.90E-11	0.22485	462346	NA	rs2158474	NA	UKB-b-57	TRUE	reported	8KoqAI
17	43761974	C	A	0.024087	0.003623	3.00E-11	0.224849	462346	NA	rs6205570	NA	UKB-b-57	TRUE	reported	8KoqAI
17	43761985	A	T	0.024088	0.003623	3.00E-11	0.224848	462346	NA	rs7692767	NA	UKB-b-57	TRUE	reported	8KoqAI
17	43762255	T	C	0.024098	0.003623	2.90E-11	0.22485	462346	NA	rs1776120	NA	UKB-b-57	TRUE	reported	8KoqAI
17	43762429	C	T	0.024092	0.003623	2.90E-11	0.224848	462346	NA	rs6205570	NA	UKB-b-57	TRUE	reported	8KoqAI
17	43762554	A	G	0.024099	0.003623	2.90E-11	0.224846	462346	NA	rs6205571	NA	UKB-b-57	TRUE	reported	8KoqAI
17	43762594	A	T	0.024099	0.003623	2.90E-11	0.224846	462346	NA	rs1768800	NA	UKB-b-57	TRUE	reported	8KoqAI
17	43762846	A	G	0.024099	0.003623	2.90E-11	0.224846	462346	NA	rs2040846	NA	UKB-b-57	TRUE	reported	8KoqAI
17	43763007	T	C	0.024099	0.003623	2.90E-11	0.224846	462346	NA	rs4486953	NA	UKB-b-57	TRUE	reported	8KoqAI
17	43763202	G	A	0.02403	0.003623	3.30E-11	0.224896	462346	NA	rs1768803	NA	UKB-b-57	TRUE	reported	8KoqAI
17	43763241	C	T	0.024088	0.003623	3.00E-11	0.224865	462346	NA	rs1768805	NA	UKB-b-57	TRUE	reported	8KoqAI
17	43763935	A	G	0.024091	0.003623	3.00E-11	0.224846	462346	NA	rs1768806	NA	UKB-b-57	TRUE	reported	8KoqAI
17	43764378	G	A	0.024042	0.003623	3.20E-11	0.224864	462346	NA	rs7722041	NA	UKB-b-57	TRUE	reported	8KoqAI
17	43764565	A	G	0.024087	0.003623	3.00E-11	0.224848	462346	NA	rs6205571	NA	UKB-b-57	TRUE	reported	8KoqAI
17	43764685	C	T	0.024087	0.003623	3.00E-11	0.224848	462346	NA	rs6205571	NA	UKB-b-57	TRUE	reported	8KoqAI
17	43764796	G	T	0.024078	0.003624	3.00E-11	0.224829	462346	NA	rs6205571	NA	UKB-b-57	TRUE	reported	8KoqAI
17	43764847	G	A	0.024078	0.003624	3.00E-11	0.224829	462346	NA	rs6205571	NA	UKB-b-57	TRUE	reported	8KoqAI
17	43764987	G	A	0.024088	0.003623	3.00E-11	0.224847	462346	NA	rs1768809	NA	UKB-b-57	TRUE	reported	8KoqAI
17	43765080	A	G	0.024125	0.003624	2.80E-11	0.224802	462346	NA	rs1768812	NA	UKB-b-57	TRUE	reported	8KoqAI
17	43765381	G	C	0.024078	0.003623	3.00E-11	0.224854	462346	NA	rs7731058	NA	UKB-b-57	TRUE	reported	8KoqAI
17	43765450	G	A	0.024097	0.003623	2.90E-11	0.224851	462346	NA	rs1776138	NA	UKB-b-57	TRUE	reported	8KoqAI
17	43765778	C	T	0.024088	0.003623	3.00E-11	0.224849	462346	NA	rs6205571	NA	UKB-b-57	TRUE	reported	8KoqAI
17	43765881	T	C	0.022909	0.003518	7.40E-11	0.245568	462346	NA	rs6503443	NA	UKB-b-57	TRUE	reported	8KoqAI
17	43766352	G	C	0.024076	0.003623	3.00E-11	0.224845	462346	NA	rs1768820	NA	UKB-b-57	TRUE	reported	8KoqAI
17	43766415	A	G	0.024481	0.003641	1.80E-11	0.223206	462346	NA	rs6205571	NA	UKB-b-57	TRUE	reported	8KoqAI
17	43766418	C	T	0.024447	0.003642	1.90E-11	0.223186	462346	NA	rs6205571	NA	UKB-b-57	TRUE	reported	8KoqAI
17	43766754	C	T	0.024087	0.003623	3.00E-11	0.224847	462346	NA	rs1768824	NA	UKB-b-57	TRUE	reported	8KoqAI
17	43767219	A	C	0.024101	0.003623	2.90E-11	0.224842	462346	NA	rs2040844	NA	UKB-b-57	TRUE	reported	8KoqAI
17	43767538	A	G	0.024089	0.003623	3.00E-11	0.224844	462346	NA	rs6205575	NA	UKB-b-57	TRUE	reported	8KoqAI
17	43767628	G	A	0.024026	0.003624	3.40E-11	0.224794	462346	NA	rs6205575	NA	UKB-b-57	TRUE	reported	8KoqAI
17	43767631	G	A	0.024088	0.003623	3.00E-11	0.224846	462346	NA	rs6205575	NA	UKB-b-57	TRUE	reported	8KoqAI
17	43767773	T	C	0.022927	0.003518	7.10E-11	0.245638	462346	NA	rs7215239	NA	UKB-b-57	TRUE	reported	8KoqAI
17	43767815	C	T	0.024092	0.003623	2.90E-11	0.224847	462346	NA	rs1768829	NA	UKB-b-57	TRUE	reported	8KoqAI
17	43768230	C	G	0.024088	0.003623	3.00E-11	0.224846	462346	NA	rs6205575	NA	UKB-b-57	TRUE	reported	8KoqAI
17	43768346	G	A	0.024502	0.00364	1.70E-11	0.223627	462346	NA	rs7847134	NA	UKB-b-57	TRUE	reported	8KoqAI
17	43768347	C	T	0.024554	0.00364	1.50E-11	0.223649	462346	NA	rs7805249	NA	UKB-b-57	TRUE	reported	8KoqAI
17	43768467	C	T	0.024267	0.003623	2.10E-11	0.224824	462346	NA	rs5610001	NA	UKB-b-57	TRUE	reported	8KoqAI
17	43768496	C	T	0.024181	0.003624	2.50E-11	0.224782	462346	NA	rs5598062	NA	UKB-b-57	TRUE	reported	8KoqAI
17	43768501	C	T	0.024206	0.003624	2.40E-11	0.224773	462346	NA	rs5566904	NA	UKB-b-57	TRUE	reported	8KoqAI
17	43768633	G	A	0.023988	0.003624	3.60E-11	0.224855	462346	NA	rs5632383	NA	UKB-b-57	TRUE	reported	8KoqAI
17	43768703	C	T	0.024094	0.003623	2.90E-11	0.224847	462346	NA	rs5597215	NA	UKB-b-57	TRUE	reported	8KoqAI
17	43768727	G	A	0.02421	0.003623	2.40E-11	0.224892	462346	NA	rs5611053	NA	UKB-b-57	TRUE	reported	8KoqAI
17	43769088	T	C	0.024088	0.003623	3.00E-11	0.224845	462346	NA	rs1776158	NA	UKB-b-57	TRUE	reported	8KoqAI
17	43769277	C	G	0.024102	0.003624	2.90E-11	0.224825	462346	NA	rs5584073	NA	UKB-b-57	TRUE	reported	8KoqAI
17	43769321	A	C	0.024088	0.003623	3.00E-11	0.224845	462346	NA	rs5628308	NA	UKB-b-57	TRUE	reported	8KoqAI
17	43769516	C	A	0.024088	0.003623	3.00E-11	0.224845	462346	NA	rs5631273	NA	UKB-b-57	TRUE	reported	8KoqAI
17	43770416	T	C	0.02409	0.003623	3.00E-11	0.224844	462346	NA	rs7476570	NA	UKB-b-57	TRUE	reported	8KoqAI
17	43770443	T	C	0.02409	0.003623	3.00E-11	0.224844	462346	NA	rs6205686	NA	UKB-b-57	TRUE	reported	8KoqAI
17	43770525	C	T	0.024098	0.003623	2.90E-11	0.224848	462346	NA	rs6205686	NA	UKB-b-57	TRUE	reported	8KoqAI
17	43770595	A	G	0.02409	0.003623	3.00E-11	0.224844	462346	NA	rs6205686	NA	UKB-b-57	TRUE	reported	8KoqAI
17	43770712	C	T	0.023019	0.00352	6.10E-11	0.245388	462346	NA	rs7207373	NA	UKB-b-57	TRUE	reported	8KoqAI
17	43770995	G	A	0.02409	0.003623	3.00E-11	0.224844	462346	NA	rs6205686	NA	UKB-b-57	TRUE	reported	8KoqAI
17	43771193	T	C	0.024082	0.003623	3.00E-11	0.224842	462346	NA	rs6205686	NA	UKB-b-57	TRUE	reported	8KoqAI
17	43771493	G	A	0.024082	0.003623	3.00E-11	0.224841	462346	NA	rs6205686	NA	UKB-b-57	TRUE	reported	8KoqAI
17	43771627	A	G	0.02409	0.003623	3.00E-11	0.224844	462346	NA	rs6205686	NA	UKB-b-57	TRUE	reported	8KoqAI
17	43772018	G	T	0.024091	0.003623	3.00E-11	0.224843	462346	NA	rs5620076	NA	UKB-b-57	TRUE	reported	8KoqAI
17	43772109	C	A	0.024091	0.003623	3.00E-11	0.224843	462346	NA	rs1768839	NA	UKB-b-57	TRUE	reported	8KoqAI
17	43772251	C	T	0.024079	0.003623	3.00E-11	0.224847	462346	NA	rs1768841	NA	UKB-b-57	TRUE	reported	8KoqAI
17	43772407	C	T	0.024091	0.003623	3.00E-11	0.224843	462346	NA	rs5592763	NA	UKB-b-57	TRUE	reported	8KoqAI
17	43772540	G	A	0.024093	0.003623	2.90E-11	0.224842	462346	NA	rs1768843	NA	UKB-b-57	TRUE	reported	8KoqAI
17	43773085	G	A	0.024093	0.003623	2.90E-11	0.224842	462346	NA	rs1768845	NA	UKB-b-57	TRUE	reported	8KoqAI
17	43773124	A	C	0.024093	0.003623	2.90E-11	0.224842	462346	NA	rs1049114	NA	UKB-b-57	TRUE	reported	8KoqAI
17	43773248	A	T	0.024094	0.003623	2.90E-11	0.224842	462346	NA	rs1049114	NA	UKB-b-57	TRUE	reported	8KoqAI
17	43773447	T	C	0.024085	0.003623	3.00E-11	0.22484	462346	NA	rs6205686	NA	UKB-b-57	TRUE	reported	8KoqAI
17	43773607	T	G	0.024094	0.003623	2.90E-11	0.224841	462346	NA	rs1768851	NA	UKB-b-57	TRUE	reported	8KoqAI
17	43773784	C	A	0.024109	0.003623	2.90E-11	0.224838	462346	NA	rs6205687	NA	UKB-b-57	TRUE	reported	8KoqAI
17	43773877	A	T	0.024095	0.003623	2.90E-11	0.224841	462346	NA	rs1768853	NA	UKB-b-57	TRUE	reported	8KoqAI
17	43773943	A	G	0.024095	0.003623	2.90E-11	0.224841	462346	NA	rs1768855	NA	UKB-b-57	TRUE	reported	8KoqAI
17	43774497	A	T	0.024085	0.003623	3.00E-11	0.224839	462346	NA	rs5616216	NA	UKB-b-57	TRUE	reported	8KoqAI
17	43774845	C	T	0.024084	0.003623	3.00E-11	0.224839	462346	NA	rs5639109	NA	UKB-b-57	TRUE	reported	8KoqAI
17	43774959	T	C	0.024085	0.003623	3.00E-11	0.224839	462346	NA	rs1776183	NA	UKB-b-57	TRUE	reported	8KoqAI
17	43775015	T	C	0.024095	0.003623	2.90E-11	0.22484	462346	NA	rs6205687	NA	UKB-b-57	TRUE	reported	8KoqAI
17	43775145	G	A	0.024114	0.003624	2.80E-11	0.224819	462346	NA	rs1215014	NA	UKB-b-57	TRUE	reported	8KoqAI
17	43775402	T	C	0.024107	0.003624	2.90E-11	0.224825	462346	NA	rs1215060	NA	UKB-b-57	TRUE	reported	8KoqAI
17	43775479	T	C	0.024019	0.003624	3.40E-11	0.224691	462346	NA	rs1215061	NA	UKB-b-57	TRUE	reported	8KoqAI
17	43775546	A	G	0.024088	0.003623	3.00E-11	0.224847	462346	NA	rs1215054	NA	UKB-b-57	TRUE	reported	8KoqAI
17	43775929	C	G	0.024088	0.003623	3.00E-11	0.224846	462346	NA	rs1768868	NA	UKB-b-57	TRUE	reported	8KoqAI
17	43776061	C	T	0.024108	0.003624	2.90E-11	0.224823	462346	NA	rs1215045	NA	UKB-b-57	TRUE	reported	8KoqAI
17	43776242	A	G	0.024115	0.003624	2.80E-11	0.224816	462346	NA	rs1215009	NA	UKB-b-57	TRUE	reported	8KoqAI
17	43776371	C	T	0.024115	0.003624	2.80E-11	0.224816	462346	NA	rs1215046	NA	UKB-b-57	TRUE	reported	8KoqAI
17	43776391	G	C	0.024115	0.003624	2.80E-11	0.224816	462346	NA	rs1215043	NA	UKB-b-57	TRUE	reported	8KoqAI
17	43776741	T	C	0.024224	0.003638	2.80E-11	0.223779	462346	NA	rs7676170	NA	UKB-b-57	TRUE	reported	8KoqAI
17	43776742	T	C	0.024226	0.003637	2.70E-11	0.22381	462346	NA	rs8014327	NA	UKB-b-57	TRUE	reported	8KoqAI
17	43776787	C	T	0.024097	0.003623	2.90E-11	0.224845	462346	NA	rs1776198	NA	UKB-b-57	TRUE	reported	8KoqAI
17	43776945	T	C	0.024096	0.003623	2.90E-11	0.224841	462346	NA	rs6205687	NA	UKB-b-57	TRUE	reported	8KoqAI
17	43777542	A	G	0.024094	0.003623	2.90E-11	0.224838	462346	NA	rs1768876	NA	UKB-b-57	TRUE	reported	8KoqAI
17	43777578	C	T	0.024094	0.003623	2.90E-11	0.224838	462346	NA	rs6205687	NA	UKB-b-57	TRUE	reported	8KoqAI
17	43777710	T	C	0.024095	0.003623	2.90E-11	0.224838	462346	NA	rs1768877	NA	UKB-b-57	TRUE	reported	8KoqAI
17	43777745	C	A	0.024086	0.003623	3.00E-11	0.224841	462346	NA	rs1776207	NA	UKB-b-57	TRUE	reported	8KoqAI
17	43778003	T	A	0.024095	0.003623	2.90E-11	0.224837	462346	NA	rs6205687	NA	UKB-b-57	TRUE	reported	8KoqAI
17	43778406	A	G	0.024166	0.003624	2.60E-11	0.224974	462346	NA	rs1768887	NA	UKB-b-57	TRUE	reported	8KoqAI
17	43778602	C	T	0.024099	0.003624	2.90E-11	0.224838	462346	NA	rs1776216	NA	UKB-b-57	TRUE	reported	8KoqAI
17	43778680	T	A	0.024013	0.003821	3.30E-10	0.204196	462346	NA	rs1768891	NA	UKB-b-57	TRUE	reported	8KoqAI
17	43778752	C	T	0.023861	0.003625	4.60E-11	0.224725	462346	NA	rs6205687	NA	UKB-b-57	TRUE	reported	8KoqAI
17	43778828	T	G	0.024163	0.003623	2.60E-11	0.225349	462346	NA	rs6205687	NA	UKB-b-57	TRUE	reported	8KoqAI
17	43779351	G	A	0.024193	0.003625	2.50E-11	0.224716	462346	NA	rs1768892	NA	UKB-b-57	TRUE	reported	8KoqAI
17	43779419	T	A	0.024169	0.003624	2.60E-11	0.224779	462346	NA	rs1768894	NA	UKB-b-57	TRUE	reported	8KoqAI
17	43779624	C	T	0.024185	0.003639	3.00E-11	0.223299	462346	NA	rs1526128	NA	UKB-b-57	TRUE	reported	8KoqAI
17	43779657	T	C	0.024177	0.003624	2.50E-11	0.224788	462346	NA	rs1526129	NA	UKB-b-57	TRUE	reported	8KoqAI
17	43780561	C	T	0.024192	0.003625	2.50E-11	0.22475	462346	NA	rs6205687	NA	UKB-b-57	TRUE	reported	8KoqAI

17	43780948	T	C	0.024215	0.003625	2.40E-11	0.224816	462346	NA	rs1776230	NA	UKB-b-57	TRUE	reported	8KoqAI
17	43781105	A	G	0.023941	0.003626	4.10E-11	0.224614	462346	NA	rs968028	NA	UKB-b-57	TRUE	reported	8KoqAI
17	43781250	C	T	0.024193	0.003627	2.60E-11	0.224449	462346	NA	rs968027	NA	UKB-b-57	TRUE	reported	8KoqAI
17	43781426	T	C	0.024283	0.003626	2.10E-11	0.224647	462346	NA	rs6205687	NA	UKB-b-57	TRUE	reported	8KoqAI
17	43781505	C	T	0.024261	0.003625	2.20E-11	0.224689	462346	NA	rs7989263	NA	UKB-b-57	TRUE	reported	8KoqAI
17	43781778	A	G	0.02421	0.003625	2.40E-11	0.224722	462346	NA	rs1776236	NA	UKB-b-57	TRUE	reported	8KoqAI
17	43782304	T	C	0.024172	0.003624	2.60E-11	0.224758	462346	NA	rs6205688	NA	UKB-b-57	TRUE	reported	8KoqAI
17	43782455	G	T	0.024315	0.003626	2.00E-11	0.224634	462346	NA	rs6205688	NA	UKB-b-57	TRUE	reported	8KoqAI
17	43782492	A	G	0.024289	0.003625	2.10E-11	0.224698	462346	NA	rs1768910	NA	UKB-b-57	TRUE	reported	8KoqAI
17	43782558	T	C	0.024266	0.003625	2.20E-11	0.224692	462346	NA	rs1768911	NA	UKB-b-57	TRUE	reported	8KoqAI
17	43782693	A	G	0.024181	0.003627	2.60E-11	0.225107	462346	NA	rs6205690	NA	UKB-b-57	TRUE	reported	8KoqAI
17	43782708	T	C	0.024179	0.003625	2.50E-11	0.224818	462346	NA	rs6205690	NA	UKB-b-57	TRUE	reported	8KoqAI
17	43783803	A	G	0.024307	0.003629	2.10E-11	0.225313	462346	NA	rs6205690	NA	UKB-b-57	TRUE	reported	8KoqAI
17	43784222	T	C	0.024201	0.003624	2.40E-11	0.224839	462346	NA	rs6205691	NA	UKB-b-57	TRUE	reported	8KoqAI
17	43784624	T	C	0.024194	0.003624	2.50E-11	0.224719	462346	NA	rs6205691	NA	UKB-b-57	TRUE	reported	8KoqAI
17	43784935	C	G	0.024221	0.003626	2.40E-11	0.224566	462346	NA	rs7856888	NA	UKB-b-57	TRUE	reported	8KoqAI
17	43784948	G	T	0.024204	0.003625	2.40E-11	0.224667	462346	NA	rs6205691	NA	UKB-b-57	TRUE	reported	8KoqAI
17	43784994	C	T	0.024278	0.003627	2.20E-11	0.224503	462346	NA	rs6205691	NA	UKB-b-57	TRUE	reported	8KoqAI
17	43785349	T	C	0.024166	0.003624	2.60E-11	0.224869	462346	NA	rs6166760	NA	UKB-b-57	TRUE	reported	8KoqAI
17	43785627	A	T	0.024173	0.003624	2.60E-11	0.224709	462346	NA	rs6205691	NA	UKB-b-57	TRUE	reported	8KoqAI
17	43785808	T	C	0.024179	0.003624	2.50E-11	0.224702	462346	NA	rs1768921	NA	UKB-b-57	TRUE	reported	8KoqAI
17	43785938	C	T	0.024172	0.003624	2.60E-11	0.224701	462346	NA	rs6205691	NA	UKB-b-57	TRUE	reported	8KoqAI
17	43786013	G	T	0.024172	0.003624	2.60E-11	0.2247	462346	NA	rs6205691	NA	UKB-b-57	TRUE	reported	8KoqAI
17	43786676	T	G	0.024263	0.003626	2.20E-11	0.22458	462346	NA	rs6205691	NA	UKB-b-57	TRUE	reported	8KoqAI
17	43786698	C	T	0.024252	0.003626	2.30E-11	0.224575	462346	NA	rs6205691	NA	UKB-b-57	TRUE	reported	8KoqAI
17	43787130	T	C	0.024215	0.003624	2.40E-11	0.224711	462346	NA	rs1776253	NA	UKB-b-57	TRUE	reported	8KoqAI
17	43787226	G	A	0.02423	0.003624	2.30E-11	0.224756	462346	NA	rs6205692	NA	UKB-b-57	TRUE	reported	8KoqAI
17	43787306	T	C	0.024236	0.003624	2.30E-11	0.22475	462346	NA	rs6205692	NA	UKB-b-57	TRUE	reported	8KoqAI
17	43787563	C	G	0.024213	0.003624	2.40E-11	0.224708	462346	NA	rs6205692	NA	UKB-b-57	TRUE	reported	8KoqAI
17	43788371	C	T	0.024203	0.003624	2.40E-11	0.224732	462346	NA	rs5629058	NA	UKB-b-57	TRUE	reported	8KoqAI
17	43788419	C	A	0.024214	0.003624	2.40E-11	0.224707	462346	NA	rs5639707	NA	UKB-b-57	TRUE	reported	8KoqAI
17	43788649	T	C	0.024217	0.003624	2.40E-11	0.224701	462346	NA	rs6205692	NA	UKB-b-57	TRUE	reported	8KoqAI
17	43788666	C	G	0.024217	0.003624	2.40E-11	0.224701	462346	NA	rs6205692	NA	UKB-b-57	TRUE	reported	8KoqAI
17	43788767	G	A	0.02421	0.003624	2.40E-11	0.22473	462346	NA	rs6205692	NA	UKB-b-57	TRUE	reported	8KoqAI
17	43788782	C	T	0.024209	0.003625	2.40E-11	0.224764	462346	NA	rs6205692	NA	UKB-b-57	TRUE	reported	8KoqAI
17	43789144	G	A	0.024212	0.003624	2.40E-11	0.224729	462346	NA	rs6205692	NA	UKB-b-57	TRUE	reported	8KoqAI
17	43789640	A	C	0.024124	0.003623	2.80E-11	0.22504	462346	NA	rs6205693	NA	UKB-b-57	TRUE	reported	8KoqAI
17	43789698	G	A	0.024561	0.003635	1.40E-11	0.222912	462346	NA	rs6205693	NA	UKB-b-57	TRUE	reported	8KoqAI
17	43789971	G	C	0.02408	0.00364	3.70E-11	0.22339	462346	NA	rs6205693	NA	UKB-b-57	TRUE	reported	8KoqAI
17	43789978	G	A	0.024099	0.00364	3.60E-11	0.22341	462346	NA	rs6205693	NA	UKB-b-57	TRUE	reported	8KoqAI
17	43790005	A	G	0.023987	0.003633	4.10E-11	0.223905	462346	NA	rs6205693	NA	UKB-b-57	TRUE	reported	8KoqAI
17	43790401	A	G	0.024222	0.003624	2.30E-11	0.224718	462346	NA	rs7932235	NA	UKB-b-57	TRUE	reported	8KoqAI
17	43790446	A	G	0.024218	0.003624	2.30E-11	0.224723	462346	NA	rs6205693	NA	UKB-b-57	TRUE	reported	8KoqAI
17	43790649	C	A	0.024218	0.003624	2.40E-11	0.224724	462346	NA	rs7507613	NA	UKB-b-57	TRUE	reported	8KoqAI
17	43790911	A	T	0.024218	0.003624	2.40E-11	0.224724	462346	NA	rs6205693	NA	UKB-b-57	TRUE	reported	8KoqAI
17	43791181	A	C	0.024216	0.003624	2.40E-11	0.224728	462346	NA	rs6205693	NA	UKB-b-57	TRUE	reported	8KoqAI
17	43791332	T	C	0.024244	0.003624	2.20E-11	0.224706	462346	NA	rs6205693	NA	UKB-b-57	TRUE	reported	8KoqAI
17	43791344	T	A	0.024244	0.003624	2.20E-11	0.224707	462346	NA	rs6205694	NA	UKB-b-57	TRUE	reported	8KoqAI
17	43791405	C	A	0.024244	0.003624	2.20E-11	0.224707	462346	NA	rs7880376	NA	UKB-b-57	TRUE	reported	8KoqAI
17	43791429	G	A	0.024244	0.003624	2.20E-11	0.224707	462346	NA	rs7738737	NA	UKB-b-57	TRUE	reported	8KoqAI
17	43791468	T	C	0.024244	0.003624	2.20E-11	0.224707	462346	NA	rs7599032	NA	UKB-b-57	TRUE	reported	8KoqAI
17	43791610	T	C	0.024215	0.003624	2.40E-11	0.22473	462346	NA	rs1136616	NA	UKB-b-57	TRUE	reported	8KoqAI
17	43791652	G	A	0.024215	0.003624	2.40E-11	0.22473	462346	NA	rs7457953	NA	UKB-b-57	TRUE	reported	8KoqAI
17	43792252	C	A	0.024252	0.003624	2.20E-11	0.224701	462346	NA	rs5597391	NA	UKB-b-57	TRUE	reported	8KoqAI
17	43792326	C	T	0.024314	0.003625	2.00E-11	0.224653	462346	NA	rs5622024	NA	UKB-b-57	TRUE	reported	8KoqAI
17	43792358	A	G	0.024303	0.003625	2.00E-11	0.22463	462346	NA	rs5616044	NA	UKB-b-57	TRUE	reported	8KoqAI
17	43792411	A	G	0.024359	0.00364	2.20E-11	0.223353	462346	NA	rs5616756	NA	UKB-b-57	TRUE	reported	8KoqAI
17	43792418	T	C	0.024349	0.00364	2.20E-11	0.223374	462346	NA	rs5584997	NA	UKB-b-57	TRUE	reported	8KoqAI
17	43792586	A	C	0.024286	0.003625	2.10E-11	0.224708	462346	NA	rs1880749	NA	UKB-b-57	TRUE	reported	8KoqAI
17	43792895	T	G	0.024272	0.003625	2.10E-11	0.224681	462346	NA	rs1568951	NA	UKB-b-57	TRUE	reported	8KoqAI
17	43792896	T	C	0.024272	0.003625	2.10E-11	0.224681	462346	NA	rs1568950	NA	UKB-b-57	TRUE	reported	8KoqAI
17	43792975	G	A	0.024216	0.003624	2.40E-11	0.224734	462346	NA	rs1568949	NA	UKB-b-57	TRUE	reported	8KoqAI
17	43793200	A	T	0.024214	0.003624	2.40E-11	0.224731	462346	NA	rs1105571	NA	UKB-b-57	TRUE	reported	8KoqAI
17	43793342	A	G	0.024216	0.003624	2.40E-11	0.224734	462346	NA	rs1105570	NA	UKB-b-57	TRUE	reported	8KoqAI
17	43793388	C	T	0.024216	0.003624	2.40E-11	0.224734	462346	NA	rs1105569	NA	UKB-b-57	TRUE	reported	8KoqAI
17	43793651	G	A	0.024193	0.003624	2.50E-11	0.224723	462346	NA	rs7502718	NA	UKB-b-57	TRUE	reported	8KoqAI
17	43793665	A	G	0.024215	0.003624	2.40E-11	0.224732	462346	NA	rs7502937	NA	UKB-b-57	TRUE	reported	8KoqAI
17	43793828	G	A	0.024218	0.003624	2.40E-11	0.224867	462346	NA	rs4479288	NA	UKB-b-57	TRUE	reported	8KoqAI
17	43793951	G	A	0.024199	0.003624	2.40E-11	0.224717	462346	NA	rs4479289	NA	UKB-b-57	TRUE	reported	8KoqAI
17	43794182	G	A	0.02421	0.003624	2.40E-11	0.224696	462346	NA	rs4609898	NA	UKB-b-57	TRUE	reported	8KoqAI
17	43794209	G	C	0.024198	0.003624	2.40E-11	0.224717	462346	NA	rs4605208	NA	UKB-b-57	TRUE	reported	8KoqAI
17	43794286	C	T	0.024212	0.003624	2.40E-11	0.224697	462346	NA	rs7531053	NA	UKB-b-57	TRUE	reported	8KoqAI
17	43795238	G	A	0.024197	0.003624	2.40E-11	0.224719	462346	NA	rs7600785	NA	UKB-b-57	TRUE	reported	8KoqAI
17	43795573	C	T	0.024197	0.003624	2.40E-11	0.22472	462346	NA	rs1114151	NA	UKB-b-57	TRUE	reported	8KoqAI
17	43795634	C	T	0.024197	0.003624	2.40E-11	0.22472	462346	NA	rs1139916	NA	UKB-b-57	TRUE	reported	8KoqAI
17	43795768	T	C	0.024197	0.003624	2.40E-11	0.22472	462346	NA	rs1129953	NA	UKB-b-57	TRUE	reported	8KoqAI
17	43796541	T	A	0.024196	0.003624	2.50E-11	0.22472	462346	NA	rs1880750	NA	UKB-b-57	TRUE	reported	8KoqAI
17	43797087	A	T	0.024198	0.003624	2.40E-11	0.224884	462346	NA	rs5616734	NA	UKB-b-57	TRUE	reported	8KoqAI
17	43797246	C	T	0.024206	0.003624	2.40E-11	0.224699	462346	NA	rs5604679	NA	UKB-b-57	TRUE	reported	8KoqAI
17	43798015	A	C	0.024194	0.003624	2.50E-11	0.224731	462346	NA	rs5632372	NA	UKB-b-57	TRUE	reported	8KoqAI
17	43798160	G	A	0.024215	0.003624	2.40E-11	0.224737	462346	NA	rs5598997	NA	UKB-b-57	TRUE	reported	8KoqAI
17	43798360	A	G	0.024215	0.003624	2.40E-11	0.224737	462346	NA	rs5593813	NA	UKB-b-57	TRUE	reported	8KoqAI
17	43798401	G	A	0.024217	0.003624	2.30E-11	0.22475	462346	NA	rs6205694	NA	UKB-b-57	TRUE	reported	8KoqAI
17	43798546	A	G	0.024216	0.003624	2.40E-11	0.224737	462346	NA	rs7540395	NA	UKB-b-57	TRUE	reported	8KoqAI
17	43798902	C	T	0.024353	0.003647	2.40E-11	0.222886	462346	NA	rs5583805	NA	UKB-b-57	TRUE	reported	8KoqAI
17	43798903	A	G	0.024353	0.003647	2.40E-11	0.222882	462346	NA	rs5606262	NA	UKB-b-57	TRUE	reported	8KoqAI
17	43799033	C	T	0.024231	0.003625	2.30E-11	0.22465	462346	NA	rs5596267	NA	UKB-b-57	TRUE	reported	8KoqAI
17	43799048	G	A	0.024338	0.003646	2.50E-11	0.222993	462346	NA	rs5589611	NA	UKB-b-57	TRUE	reported	8KoqAI
17	43799052	A	G	0.024334	0.003641	2.30E-11	0.223373	462346	NA	rs5578773	NA	UKB-b-57	TRUE	reported	8KoqAI
17	43799509	A	C	0.02424	0.003625	2.30E-11	0.224637	462346	NA	rs5565396	NA	UKB-b-57	TRUE	reported	8KoqAI
17	43799667	C	A	0.024235	0.003625	2.30E-11	0.224655	462346	NA	rs6205437	NA	UKB-b-57	TRUE	reported	8KoqAI
17	43799804	C	A	0.024234	0.003625	2.30E-11	0.224655	462346	NA	rs7781900	NA	UKB-b-57	TRUE	reported	8KoqAI
17	43799810	G	C	0.024229	0.003625	2.30E-11	0.224653	462346	NA	rs7666786	NA	UKB-b-57	TRUE	reported	8KoqAI
17	43800351	A	C	0.02422	0.003624	2.30E-11	0.224739	462346	NA	rs1756343	NA	UKB-b-57	TRUE	reported	8KoqAI
17	43800404	T	G	0.02422	0.003624	2.30E-11	0.224739	462346	NA	rs7737007	NA	UKB-b-57	TRUE	reported	8KoqAI
17	43800987	C	A	0.024275	0.003624	2.10E-11	0.224708	462346	NA	rs6205438	NA	UKB-b-57	TRUE	reported	8KoqAI
17	43801092	T	C	0.024221	0.003624	2.30E-11	0.224739	462346	NA	rs1125709	NA	UKB-b-57	TRUE	reported	8KoqAI
17	43801185	A	G	0.024161	0.003624	2.60E-11	0.224813	462346	NA	rs6205438	NA	UKB-b-57	TRUE	reported	8KoqAI
17	43801593	G	A	0.024222	0.003624	2.30E-11	0.224739	462346	NA	rs1764901	NA	UKB-b-57	TRUE	reported	8KoqAI
17	43801695	C	T	0.024276	0.003624	2.10E-11	0.224709	462346	NA	rs1756350	NA	UKB-b-57	TRUE	reported	8KoqAI
17	43802525	C	T	0.024279	0.003624	2.10E-11	0.224711	462346	NA	rs6205438	NA	UKB-b-57	TRUE	reported	8KoqAI
17	43802907	T	C	0.024278	0.003624	2.10E-11	0.224712	462346	NA	rs6205438	NA	UKB-b-57	TRUE	reported	8KoqAI
17	43802971	C	T	0.02428	0.003624	2.10E-11	0.224727	462346	NA	rs1051487	NA	UKB-b-57	TRUE	reported	8KoqAI
17	43803189	C	A	-0.02323	0.003407	9.10E-12	0.729589	462346	NA	rs1358071	NA	UKB-b-57	TRUE	reported	8KoqAI
17	43803788	T	C	0.024232	0.003624	2.30E-11	0.224742	462346	NA	rs5628022	NA	UKB-b-57	TRUE	reported	8KoqAI
17	43804306	T	C	0.024225	0.003625	2.30E-11	0.224696	462346	NA	rs6205438	NA	UKB-b-57	TRUE	reported	8KoqAI
17	43804317	A	G	-0.02426	0.003622	2.10E-11	0.77474	462346	NA	rs9897399	NA	UKB-b-57	TRUE	reported	8KoqAI

17	43804389	T	A	0.024216	0.003624	2.30E-11	0.224759	462346	NA	rs5620556	NA	UKB-b-57	TRUE	reported	8KoqAI
17	43804619	C	T	0.024182	0.003623	2.50E-11	0.224798	462346	NA	rs5638066	NA	UKB-b-57	TRUE	reported	8KoqAI
17	43805194	T	G	-0.01734	0.003037	1.10E-08	0.537748	462346	NA	rs9303521	NA	UKB-b-57	TRUE	reported	8KoqAI
17	43805525	A	C	0.024204	0.003624	2.40E-11	0.224774	462346	NA	rs6205438	NA	UKB-b-57	TRUE	reported	8KoqAI
17	43805590	C	T	0.024292	0.003625	2.10E-11	0.224709	462346	NA	rs6205438	NA	UKB-b-57	TRUE	reported	8KoqAI
17	43806015	G	A	0.024169	0.003624	2.60E-11	0.224804	462346	NA	rs4401083	NA	UKB-b-57	TRUE	reported	8KoqAI
17	43806264	C	T	0.024188	0.003624	2.50E-11	0.224782	462346	NA	rs1880752	NA	UKB-b-57	TRUE	reported	8KoqAI
17	43806451	C	T	0.024189	0.003624	2.50E-11	0.22479	462346	NA	rs4617909	NA	UKB-b-57	TRUE	reported	8KoqAI
17	43806925	G	A	0.024188	0.003624	2.50E-11	0.224782	462346	NA	rs2902662	NA	UKB-b-57	TRUE	reported	8KoqAI
17	43807063	C	T	0.024188	0.003624	2.50E-11	0.224782	462346	NA	rs2864087	NA	UKB-b-57	TRUE	reported	8KoqAI
17	43807099	G	A	0.024167	0.003624	2.60E-11	0.224879	462346	NA	rs2902661	NA	UKB-b-57	TRUE	reported	8KoqAI
17	43807113	C	T	0.02423	0.003626	2.30E-11	0.224619	462346	NA	rs4609899	NA	UKB-b-57	TRUE	reported	8KoqAI
17	43807134	T	C	0.024223	0.003626	2.40E-11	0.224605	462346	NA	rs4471726	NA	UKB-b-57	TRUE	reported	8KoqAI
17	43807485	T	C	0.024187	0.003624	2.50E-11	0.224782	462346	NA	rs7922687	NA	UKB-b-57	TRUE	reported	8KoqAI
17	43807955	A	C	0.024188	0.003624	2.50E-11	0.224782	462346	NA	rs1756359	NA	UKB-b-57	TRUE	reported	8KoqAI
17	43808067	G	A	0.024188	0.003624	2.50E-11	0.224782	462346	NA	rs1764913	NA	UKB-b-57	TRUE	reported	8KoqAI
17	43808321	G	A	0.024188	0.003624	2.50E-11	0.224782	462346	NA	rs6205438	NA	UKB-b-57	TRUE	reported	8KoqAI
17	43808443	A	C	0.024187	0.003624	2.50E-11	0.224782	462346	NA	rs6205439	NA	UKB-b-57	TRUE	reported	8KoqAI
17	43808763	A	G	0.024187	0.003624	2.50E-11	0.224782	462346	NA	rs5594721	NA	UKB-b-57	TRUE	reported	8KoqAI
17	43808945	C	T	0.024187	0.003624	2.50E-11	0.224782	462346	NA	rs6205439	NA	UKB-b-57	TRUE	reported	8KoqAI
17	43809016	C	T	0.024187	0.003624	2.50E-11	0.224782	462346	NA	rs4390635	NA	UKB-b-57	TRUE	reported	8KoqAI
17	43809264	T	G	0.024192	0.003624	2.50E-11	0.224781	462346	NA	rs6205439	NA	UKB-b-57	TRUE	reported	8KoqAI
17	43809328	A	G	0.024177	0.003624	2.50E-11	0.224782	462346	NA	rs7598634	NA	UKB-b-57	TRUE	reported	8KoqAI
17	43810371	G	C	0.024192	0.003624	2.50E-11	0.224781	462346	NA	rs1764916	NA	UKB-b-57	TRUE	reported	8KoqAI
17	43810608	T	A	0.024178	0.003624	2.50E-11	0.224782	462346	NA	rs6205439	NA	UKB-b-57	TRUE	reported	8KoqAI
17	43810782	G	C	0.024178	0.003624	2.50E-11	0.224782	462346	NA	rs1137909	NA	UKB-b-57	TRUE	reported	8KoqAI
17	43810851	G	C	0.024255	0.003651	3.10E-11	0.222425	462346	NA	rs7780745	NA	UKB-b-57	TRUE	reported	8KoqAI
17	43810873	A	T	0.024316	0.003665	3.20E-11	0.22124	462346	NA	rs7502233	NA	UKB-b-57	TRUE	reported	8KoqAI
17	43810896	C	T	0.023723	0.003652	8.30E-11	0.224916	462346	NA	rs7780406	NA	UKB-b-57	TRUE	reported	8KoqAI
17	43810902	A	G	0.024346	0.003675	3.50E-11	0.220263	462346	NA	rs7730184	NA	UKB-b-57	TRUE	reported	8KoqAI
17	43811060	T	G	0.024178	0.003624	2.50E-11	0.224782	462346	NA	rs6205439	NA	UKB-b-57	TRUE	reported	8KoqAI
17	43811072	A	G	0.024194	0.003624	2.40E-11	0.224766	462346	NA	rs1756368	NA	UKB-b-57	TRUE	reported	8KoqAI
17	43811510	T	C	0.024194	0.003624	2.40E-11	0.22478	462346	NA	rs6205439	NA	UKB-b-57	TRUE	reported	8KoqAI
17	43811673	C	T	0.024195	0.003624	2.40E-11	0.224779	462346	NA	rs1756371	NA	UKB-b-57	TRUE	reported	8KoqAI
17	43811792	C	T	0.02418	0.003624	2.50E-11	0.22478	462346	NA	rs6205439	NA	UKB-b-57	TRUE	reported	8KoqAI
17	43811950	A	C	0.02418	0.003624	2.50E-11	0.22478	462346	NA	rs4569335	NA	UKB-b-57	TRUE	reported	8KoqAI
17	43812177	C	T	0.02418	0.003624	2.50E-11	0.22478	462346	NA	rs1526125	NA	UKB-b-57	TRUE	reported	8KoqAI
17	43812218	C	T	0.024187	0.003625	2.50E-11	0.22471	462346	NA	rs1526126	NA	UKB-b-57	TRUE	reported	8KoqAI
17	43812398	C	G	0.024163	0.003624	2.60E-11	0.224787	462346	NA	rs1526127	NA	UKB-b-57	TRUE	reported	8KoqAI
17	43812599	A	G	0.024163	0.003624	2.60E-11	0.224787	462346	NA	rs6205439	NA	UKB-b-57	TRUE	reported	8KoqAI
17	43812624	G	A	0.024163	0.003624	2.60E-11	0.224787	462346	NA	rs6205439	NA	UKB-b-57	TRUE	reported	8KoqAI
17	43812649	T	C	0.024163	0.003624	2.60E-11	0.224787	462346	NA	rs6205440	NA	UKB-b-57	TRUE	reported	8KoqAI
17	43812684	A	G	0.024163	0.003624	2.60E-11	0.224787	462346	NA	rs6205441	NA	UKB-b-57	TRUE	reported	8KoqAI
17	43813002	G	A	0.024165	0.003624	2.60E-11	0.224787	462346	NA	rs6205441	NA	UKB-b-57	TRUE	reported	8KoqAI
17	43813252	C	G	0.024171	0.003624	2.60E-11	0.224788	462346	NA	rs1756378	NA	UKB-b-57	TRUE	reported	8KoqAI
17	43813370	G	C	0.024171	0.003624	2.60E-11	0.224788	462346	NA	rs6205441	NA	UKB-b-57	TRUE	reported	8KoqAI
17	43813415	G	A	0.024171	0.003624	2.60E-11	0.224788	462346	NA	rs6205441	NA	UKB-b-57	TRUE	reported	8KoqAI
17	43814020	T	C	0.023973	0.003622	3.60E-11	0.225252	462346	NA	rs1045128	NA	UKB-b-57	TRUE	reported	8KoqAI
17	43814427	T	C	0.024172	0.003624	2.50E-11	0.224788	462346	NA	rs7642181	NA	UKB-b-57	TRUE	reported	8KoqAI
17	43815141	A	G	0.024172	0.003624	2.50E-11	0.224788	462346	NA	rs5600624	NA	UKB-b-57	TRUE	reported	8KoqAI
17	43815336	A	G	0.024179	0.003624	2.50E-11	0.224787	462346	NA	rs5629811	NA	UKB-b-57	TRUE	reported	8KoqAI
17	43815713	C	T	0.024198	0.003624	2.40E-11	0.224845	462346	NA	rs1608955	NA	UKB-b-57	TRUE	reported	8KoqAI
17	43815820	T	A	0.024198	0.003624	2.40E-11	0.224845	462346	NA	rs1608956	NA	UKB-b-57	TRUE	reported	8KoqAI
17	43815859	A	C	0.024176	0.003624	2.50E-11	0.224788	462346	NA	rs6205441	NA	UKB-b-57	TRUE	reported	8KoqAI
17	43816052	C	T	0.024241	0.003624	2.20E-11	0.224844	462346	NA	rs7844027	NA	UKB-b-57	TRUE	reported	8KoqAI
17	43816106	C	A	0.024147	0.003624	2.70E-11	0.224736	462346	NA	rs7550094	NA	UKB-b-57	TRUE	reported	8KoqAI
17	43816306	G	A	0.024147	0.003624	2.70E-11	0.224736	462346	NA	rs6205441	NA	UKB-b-57	TRUE	reported	8KoqAI
17	43816605	A	C	0.024177	0.003624	2.50E-11	0.224787	462346	NA	rs6205441	NA	UKB-b-57	TRUE	reported	8KoqAI
17	43816655	C	G	0.024176	0.003624	2.50E-11	0.224788	462346	NA	rs6205442	NA	UKB-b-57	TRUE	reported	8KoqAI
17	43816657	G	A	0.024176	0.003624	2.50E-11	0.224788	462346	NA	rs6205442	NA	UKB-b-57	TRUE	reported	8KoqAI
17	43817459	C	T	0.024176	0.003624	2.50E-11	0.224788	462346	NA	rs1756380	NA	UKB-b-57	TRUE	reported	8KoqAI
17	43817557	C	T	0.024176	0.003624	2.50E-11	0.224788	462346	NA	rs5629599	NA	UKB-b-57	TRUE	reported	8KoqAI
17	43817608	G	A	0.024176	0.003624	2.50E-11	0.224788	462346	NA	rs5638496	NA	UKB-b-57	TRUE	reported	8KoqAI
17	43817832	G	A	0.024174	0.003624	2.50E-11	0.224784	462346	NA	rs5617823	NA	UKB-b-57	TRUE	reported	8KoqAI
17	43817898	G	A	0.024174	0.003624	2.50E-11	0.224784	462346	NA	rs8015755	NA	UKB-b-57	TRUE	reported	8KoqAI
17	43818160	A	C	0.02418	0.003624	2.50E-11	0.224791	462346	NA	rs7672863	NA	UKB-b-57	TRUE	reported	8KoqAI
17	43818222	C	A	0.024176	0.003624	2.50E-11	0.224788	462346	NA	rs1756382	NA	UKB-b-57	TRUE	reported	8KoqAI
17	43818611	G	A	0.02418	0.003624	2.50E-11	0.224789	462346	NA	rs8007242	NA	UKB-b-57	TRUE	reported	8KoqAI
17	43818657	T	C	0.024181	0.003624	2.50E-11	0.224789	462346	NA	rs7711462	NA	UKB-b-57	TRUE	reported	8KoqAI
17	43818690	G	T	0.024181	0.003624	2.50E-11	0.224789	462346	NA	rs7631142	NA	UKB-b-57	TRUE	reported	8KoqAI
17	43818906	A	G	0.024181	0.003624	2.50E-11	0.224789	462346	NA	rs1756386	NA	UKB-b-57	TRUE	reported	8KoqAI
17	43818946	A	T	0.02418	0.003624	2.50E-11	0.224789	462346	NA	rs1756388	NA	UKB-b-57	TRUE	reported	8KoqAI
17	43819384	G	A	0.024171	0.003624	2.60E-11	0.224797	462346	NA	rs6205442	NA	UKB-b-57	TRUE	reported	8KoqAI
17	43819450	G	A	0.02417	0.003624	2.60E-11	0.224798	462346	NA	rs1756392	NA	UKB-b-57	TRUE	reported	8KoqAI
17	43819480	C	T	0.024169	0.003624	2.60E-11	0.224798	462346	NA	rs6205442	NA	UKB-b-57	TRUE	reported	8KoqAI
17	43820741	A	T	0.024139	0.003624	2.70E-11	0.22482	462346	NA	rs6205442	NA	UKB-b-57	TRUE	reported	8KoqAI
17	43821062	A	G	0.024136	0.003624	2.70E-11	0.224822	462346	NA	rs6205442	NA	UKB-b-57	TRUE	reported	8KoqAI
17	43821685	C	A	0.024136	0.003624	2.70E-11	0.224822	462346	NA	rs7446499	NA	UKB-b-57	TRUE	reported	8KoqAI
17	43822398	T	C	0.024172	0.003624	2.50E-11	0.224829	462346	NA	rs2004260	NA	UKB-b-57	TRUE	reported	8KoqAI
17	43822511	G	A	0.0242	0.003624	2.40E-11	0.224816	462346	NA	rs6205442	NA	UKB-b-57	TRUE	reported	8KoqAI
17	43823125	G	A	0.024205	0.003624	2.40E-11	0.224825	462346	NA	rs6205442	NA	UKB-b-57	TRUE	reported	8KoqAI
17	43823229	T	C	0.023976	0.003623	3.60E-11	0.225288	462346	NA	rs7617114	NA	UKB-b-57	TRUE	reported	8KoqAI
17	43823326	A	G	0.024149	0.003624	2.70E-11	0.224833	462346	NA	rs7564324	NA	UKB-b-57	TRUE	reported	8KoqAI
17	43823880	C	T	0.02412	0.003624	2.80E-11	0.224824	462346	NA	rs6205443	NA	UKB-b-57	TRUE	reported	8KoqAI
17	43823889	C	A	0.02412	0.003624	2.80E-11	0.224824	462346	NA	rs6205443	NA	UKB-b-57	TRUE	reported	8KoqAI
17	43824201	G	A	0.02412	0.003624	2.80E-11	0.224823	462346	NA	rs1114970	NA	UKB-b-57	TRUE	reported	8KoqAI
17	43824348	T	C	0.02412	0.003624	2.80E-11	0.224823	462346	NA	rs6205443	NA	UKB-b-57	TRUE	reported	8KoqAI
17	43824360	T	C	0.023475	0.003405	5.40E-12	0.271346	462346	NA	rs7207400	NA	UKB-b-57	TRUE	reported	8KoqAI
17	43824382	T	G	0.02412	0.003624	2.80E-11	0.224823	462346	NA	rs6205443	NA	UKB-b-57	TRUE	reported	8KoqAI
17	43824848	A	G	0.02412	0.003624	2.80E-11	0.224823	462346	NA	rs1215068	NA	UKB-b-57	TRUE	reported	8KoqAI
17	43824908	C	T	0.02412	0.003624	2.80E-11	0.224823	462346	NA	rs1215033	NA	UKB-b-57	TRUE	reported	8KoqAI
17	43825339	C	G	0.02412	0.003624	2.80E-11	0.224815	462346	NA	rs6205443	NA	UKB-b-57	TRUE	reported	8KoqAI
17	43825478	G	A	0.024114	0.003624	2.80E-11	0.224824	462346	NA	rs7571519	NA	UKB-b-57	TRUE	reported	8KoqAI
17	43825568	C	T	0.024215	0.003637	2.80E-11	0.223587	462346	NA	rs6205443	NA	UKB-b-57	TRUE	reported	8KoqAI
17	43825576	T	C	0.024214	0.003637	2.80E-11	0.223583	462346	NA	rs6205443	NA	UKB-b-57	TRUE	reported	8KoqAI
17	43825711	A	C	0.024114	0.003624	2.80E-11	0.224824	462346	NA	rs6205443	NA	UKB-b-57	TRUE	reported	8KoqAI
17	43825725	G	T	0.024114	0.003624	2.80E-11	0.224824	462346	NA	rs6205443	NA	UKB-b-57	TRUE	reported	8KoqAI
17	43825912	A	G	0.024113	0.003624	2.80E-11	0.224824	462346	NA	rs1733479	NA	UKB-b-57	TRUE	reported	8KoqAI
17	43826305	G	A	0.024093	0.003624	3.00E-11	0.224844	462346	NA	rs1215065	NA	UKB-b-57	TRUE	reported	8KoqAI
17	43826351	A	G	0.024117	0.003624	2.80E-11	0.22482	462346	NA	rs1215036	NA	UKB-b-57	TRUE	reported	8KoqAI
17	43826637	G	A	0.024118	0.003624	2.80E-11	0.22482	462346	NA	rs1215067	NA	UKB-b-57	TRUE	reported	8KoqAI
17	43827209	G	A	0.024117	0.003624	2.80E-11	0.224821	462346	NA	rs1733489	NA	UKB-b-57	TRUE	reported	8KoqAI
17	43827244	C	T	0.024185	0.003621	2.40E-11	0.225484	462346	NA	rs1733492	NA	UKB-b-57	TRUE	reported	8KoqAI
17	43827391	G	C	0.024195	0.003624	2.40E-11	0.224894	462346	NA	rs6205444	NA	UKB-b-57	TRUE	reported	8KoqAI
17	43827431	A	G	0.024192	0.003624	2.50E-11	0.224896	462346	NA	rs1215045	NA	UKB-b-57	TRUE	reported	8KoqAI
17	43827471	G	C	0.024194	0.003624	2.50E-11	0.22488	462346	NA	rs1215004	NA	UKB-b-57	TRUE	reported	8KoqAI
17	43827508	A	G	0.024073	0.003624	3.10E-11	0.224863	462346	NA	rs1215045	NA	UKB-b-57	TRUE	reported	8KoqAI

17	43828221	T	A	0.024118	0.003624	2.80E-11	0.224818	462346	NA	rs1215060	NA	UKB-b-57	TRUE	reported	8KoqAI
17	43828617	G	A	0.024111	0.003624	2.90E-11	0.224815	462346	NA	rs1733494	NA	UKB-b-57	TRUE	reported	8KoqAI
17	43828698	C	T	0.024111	0.003624	2.90E-11	0.224818	462346	NA	rs1742606	NA	UKB-b-57	TRUE	reported	8KoqAI
17	43828764	G	A	0.024673	0.003901	2.50E-10	0.19126	462346	NA	rs7917280	NA	UKB-b-57	TRUE	reported	8KoqAI
17	43828935	G	C	0.024109	0.003624	2.90E-11	0.224816	462346	NA	rs1742610	NA	UKB-b-57	TRUE	reported	8KoqAI
17	43829353	A	G	0.024122	0.003624	2.80E-11	0.224926	462346	NA	rs6205444	NA	UKB-b-57	TRUE	reported	8KoqAI
17	43830382	G	A	0.024167	0.003625	2.60E-11	0.224731	462346	NA	rs3587908	NA	UKB-b-57	TRUE	reported	8KoqAI
17	43830640	C	T	0.024152	0.003624	2.70E-11	0.224745	462346	NA	rs6205586	NA	UKB-b-57	TRUE	reported	8KoqAI
17	43830685	G	A	0.024163	0.003624	2.60E-11	0.224736	462346	NA	rs6205586	NA	UKB-b-57	TRUE	reported	8KoqAI
17	43830938	G	C	0.024155	0.003624	2.70E-11	0.224743	462346	NA	rs1742617	NA	UKB-b-57	TRUE	reported	8KoqAI
17	43831112	G	A	0.024157	0.003624	2.60E-11	0.224744	462346	NA	rs6205587	NA	UKB-b-57	TRUE	reported	8KoqAI
17	43832115	A	G	0.024168	0.003624	2.60E-11	0.224761	462346	NA	rs5564541	NA	UKB-b-57	TRUE	reported	8KoqAI
17	43832337	A	G	0.024142	0.003628	2.80E-11	0.224422	462346	NA	rs3563166	NA	UKB-b-57	TRUE	reported	8KoqAI
17	43832367	G	A	0.02414	0.003628	2.80E-11	0.224446	462346	NA	rs1742619	NA	UKB-b-57	TRUE	reported	8KoqAI
17	43832618	G	A	0.024266	0.003625	2.20E-11	0.224681	462346	NA	rs3528250	NA	UKB-b-57	TRUE	reported	8KoqAI
17	43832864	T	C	0.024266	0.003625	2.20E-11	0.224681	462346	NA	rs6205587	NA	UKB-b-57	TRUE	reported	8KoqAI
17	43832940	A	G	0.024478	0.003631	1.60E-11	0.224118	462346	NA	rs6205587	NA	UKB-b-57	TRUE	reported	8KoqAI
17	43833824	G	C	0.024171	0.003624	2.60E-11	0.22476	462346	NA	rs3604769	NA	UKB-b-57	TRUE	reported	8KoqAI
17	43833921	G	A	0.024171	0.003624	2.60E-11	0.22476	462346	NA	rs3419556	NA	UKB-b-57	TRUE	reported	8KoqAI
17	43833981	A	G	0.024168	0.003625	2.60E-11	0.224713	462346	NA	rs3457927	NA	UKB-b-57	TRUE	reported	8KoqAI
17	43834074	C	G	0.024173	0.003624	2.60E-11	0.224754	462346	NA	rs3421125	NA	UKB-b-57	TRUE	reported	8KoqAI
17	43834314	A	G	0.024173	0.003624	2.60E-11	0.22476	462346	NA	rs3400851	NA	UKB-b-57	TRUE	reported	8KoqAI
17	43834482	G	A	0.024171	0.003624	2.60E-11	0.224759	462346	NA	rs1294308	NA	UKB-b-57	TRUE	reported	8KoqAI
17	43834970	C	T	0.024167	0.003624	2.60E-11	0.224762	462346	NA	rs7350923	NA	UKB-b-57	TRUE	reported	8KoqAI
17	43835351	T	C	0.024171	0.003624	2.60E-11	0.224771	462346	NA	rs1107971	NA	UKB-b-57	TRUE	reported	8KoqAI
17	43835854	C	A	0.024175	0.003624	2.60E-11	0.224757	462346	NA	rs6205587	NA	UKB-b-57	TRUE	reported	8KoqAI
17	43836051	T	A	0.024225	0.003624	2.30E-11	0.224819	462346	NA	rs7666942	NA	UKB-b-57	TRUE	reported	8KoqAI
17	43836191	G	A	0.024187	0.003625	2.50E-11	0.224745	462346	NA	rs7451426	NA	UKB-b-57	TRUE	reported	8KoqAI
17	43836673	C	T	0.024176	0.003625	2.60E-11	0.224751	462346	NA	rs1294821	NA	UKB-b-57	TRUE	reported	8KoqAI
17	43836920	T	C	0.024164	0.003625	2.60E-11	0.224702	462346	NA	rs5601390	NA	UKB-b-57	TRUE	reported	8KoqAI
17	43836953	A	C	0.02468	0.003839	1.30E-10	0.196383	462346	NA	rs5621451	NA	UKB-b-57	TRUE	reported	8KoqAI
17	43837091	T	C	0.024151	0.003626	2.70E-11	0.224677	462346	NA	rs5616890	NA	UKB-b-57	TRUE	reported	8KoqAI
17	43837115	A	G	0.024152	0.003625	2.70E-11	0.224712	462346	NA	rs5574744	NA	UKB-b-57	TRUE	reported	8KoqAI
17	43837237	A	G	0.024162	0.003625	2.60E-11	0.224723	462346	NA	rs5580135	NA	UKB-b-57	TRUE	reported	8KoqAI
17	43837446	C	G	0.024254	0.003626	2.20E-11	0.22465	462346	NA	rs6205588	NA	UKB-b-57	TRUE	reported	8KoqAI
17	43837567	C	T	0.024165	0.003625	2.60E-11	0.224734	462346	NA	rs6205588	NA	UKB-b-57	TRUE	reported	8KoqAI
17	43837782	A	G	0.024175	0.003625	2.60E-11	0.224721	462346	NA	rs7988250	NA	UKB-b-57	TRUE	reported	8KoqAI
17	43838014	A	G	0.024228	0.003626	2.30E-11	0.224684	462346	NA	rs6205588	NA	UKB-b-57	TRUE	reported	8KoqAI
17	43838071	C	T	0.024188	0.003625	2.50E-11	0.224738	462346	NA	rs2843927	NA	UKB-b-57	TRUE	reported	8KoqAI
17	43838482	G	T	0.024171	0.003625	2.60E-11	0.224733	462346	NA	rs1215062	NA	UKB-b-57	TRUE	reported	8KoqAI
17	43838678	G	A	0.024188	0.003629	2.60E-11	0.224422	462346	NA	rs6205588	NA	UKB-b-57	TRUE	reported	8KoqAI
17	43838710	T	C	0.024188	0.003629	2.60E-11	0.224419	462346	NA	rs6205588	NA	UKB-b-57	TRUE	reported	8KoqAI
17	43838720	C	T	0.02434	0.003654	2.70E-11	0.220834	462346	NA	rs6205588	NA	UKB-b-57	TRUE	reported	8KoqAI
17	43838919	G	A	0.024094	0.003626	3.00E-11	0.224631	462346	NA	rs6205588	NA	UKB-b-57	TRUE	reported	8KoqAI
17	43839253	G	A	0.024138	0.003629	2.90E-11	0.22437	462346	NA	rs7137531	NA	UKB-b-57	TRUE	reported	8KoqAI
17	43839951	A	T	0.024072	0.003638	3.70E-11	0.224178	462346	NA	rs1107971	NA	UKB-b-57	TRUE	reported	8KoqAI
17	43840006	T	G	0.023748	0.003653	8.00E-11	0.222855	462346	NA	rs1107971	NA	UKB-b-57	TRUE	reported	8KoqAI
17	43840016	G	A	0.023898	0.003654	6.10E-11	0.222342	462346	NA	rs1107972	NA	UKB-b-57	TRUE	reported	8KoqAI
17	43840107	C	A	0.023956	0.003627	4.00E-11	0.224734	462346	NA	rs1107972	NA	UKB-b-57	TRUE	reported	8KoqAI
17	43840681	C	T	0.024205	0.003626	2.50E-11	0.224732	462346	NA	rs6205589	NA	UKB-b-57	TRUE	reported	8KoqAI
17	43840864	G	A	0.024129	0.003626	2.80E-11	0.224666	462346	NA	rs7784934	NA	UKB-b-57	TRUE	reported	8KoqAI
17	43840899	G	C	0.024251	0.003626	2.30E-11	0.22461	462346	NA	rs7491868	NA	UKB-b-57	TRUE	reported	8KoqAI
17	43840935	T	C	0.024274	0.003626	2.20E-11	0.224649	462346	NA	rs7954514	NA	UKB-b-57	TRUE	reported	8KoqAI
17	43841571	T	A	0.024177	0.003625	2.60E-11	0.224668	462346	NA	rs5636903	NA	UKB-b-57	TRUE	reported	8KoqAI
17	43841729	T	C	0.024241	0.003626	2.30E-11	0.224575	462346	NA	rs1107972	NA	UKB-b-57	TRUE	reported	8KoqAI
17	43841912	C	T	0.024194	0.003625	2.50E-11	0.224704	462346	NA	rs1107972	NA	UKB-b-57	TRUE	reported	8KoqAI
17	43842462	T	C	0.024394	0.003629	1.80E-11	0.224466	462346	NA	rs5570733	NA	UKB-b-57	TRUE	reported	8KoqAI
17	43842494	A	G	0.024328	0.003629	2.00E-11	0.224469	462346	NA	rs6205589	NA	UKB-b-57	TRUE	reported	8KoqAI
17	43843395	C	T	0.02426	0.00363	2.30E-11	0.224365	462346	NA	rs7525700	NA	UKB-b-57	TRUE	reported	8KoqAI
17	43843943	G	A	0.024116	0.003625	2.90E-11	0.224666	462346	NA	rs6205589	NA	UKB-b-57	TRUE	reported	8KoqAI
17	43844044	A	G	0.024119	0.003625	2.90E-11	0.224668	462346	NA	rs6205589	NA	UKB-b-57	TRUE	reported	8KoqAI
17	43844201	C	T	0.024118	0.003625	2.90E-11	0.224669	462346	NA	rs6205589	NA	UKB-b-57	TRUE	reported	8KoqAI
17	43844486	T	A	0.024119	0.003625	2.90E-11	0.224668	462346	NA	rs5572584	NA	UKB-b-57	TRUE	reported	8KoqAI
17	43844559	T	G	0.024382	0.003658	2.70E-11	0.221525	462346	NA	rs5619450	NA	UKB-b-57	TRUE	reported	8KoqAI
17	43844560	T	G	0.02438	0.003659	2.70E-11	0.221522	462346	NA	rs5565791	NA	UKB-b-57	TRUE	reported	8KoqAI
17	43844798	G	A	0.023995	0.003625	3.60E-11	0.224769	462346	NA	rs5608231	NA	UKB-b-57	TRUE	reported	8KoqAI
17	43844859	A	G	0.024119	0.003625	2.90E-11	0.224668	462346	NA	rs5610964	NA	UKB-b-57	TRUE	reported	8KoqAI
17	43844977	T	A	0.024145	0.003626	2.80E-11	0.224597	462346	NA	rs6205589	NA	UKB-b-57	TRUE	reported	8KoqAI
17	43845002	C	G	0.024154	0.003626	2.70E-11	0.224608	462346	NA	rs6205590	NA	UKB-b-57	TRUE	reported	8KoqAI
17	43845041	G	A	0.024081	0.003625	3.10E-11	0.224692	462346	NA	rs6205590	NA	UKB-b-57	TRUE	reported	8KoqAI
17	43845480	T	C	-0.02324	0.003407	9.10E-12	0.728269	462346	NA	rs5808904	NA	UKB-b-57	TRUE	reported	8KoqAI
17	43846668	C	A	0.024119	0.003625	2.90E-11	0.224674	462346	NA	rs6205590	NA	UKB-b-57	TRUE	reported	8KoqAI
17	43846820	G	A	0.02412	0.003625	2.90E-11	0.224674	462346	NA	rs1113740	NA	UKB-b-57	TRUE	reported	8KoqAI
17	43847039	C	T	0.02412	0.003625	2.90E-11	0.224673	462346	NA	rs1139341	NA	UKB-b-57	TRUE	reported	8KoqAI
17	43847374	G	A	0.024124	0.003625	2.80E-11	0.224665	462346	NA	rs6205592	NA	UKB-b-57	TRUE	reported	8KoqAI
17	43847741	T	C	0.02412	0.003625	2.90E-11	0.224673	462346	NA	rs5626832	NA	UKB-b-57	TRUE	reported	8KoqAI
17	43847868	T	C	0.024114	0.003625	2.90E-11	0.224669	462346	NA	rs5607024	NA	UKB-b-57	TRUE	reported	8KoqAI
17	43847912	T	C	0.024119	0.003625	2.90E-11	0.224672	462346	NA	rs5638726	NA	UKB-b-57	TRUE	reported	8KoqAI
17	43848181	C	T	0.024118	0.003625	2.90E-11	0.224671	462346	NA	rs3430348	NA	UKB-b-57	TRUE	reported	8KoqAI
17	43848412	A	G	0.024143	0.003626	2.80E-11	0.224654	462346	NA	rs6205593	NA	UKB-b-57	TRUE	reported	8KoqAI
17	43848461	G	A	0.024098	0.003629	3.10E-11	0.22435	462346	NA	rs6205593	NA	UKB-b-57	TRUE	reported	8KoqAI
17	43848750	T	C	0.024266	0.003649	2.90E-11	0.22263	462346	NA	rs6205593	NA	UKB-b-57	TRUE	reported	8KoqAI
17	43848761	T	A	0.024299	0.003648	2.70E-11	0.222632	462346	NA	rs6205593	NA	UKB-b-57	TRUE	reported	8KoqAI
17	43848968	G	T	0.024212	0.003634	2.70E-11	0.224151	462346	NA	rs6205593	NA	UKB-b-57	TRUE	reported	8KoqAI
17	43849327	G	A	0.024184	0.003625	2.50E-11	0.224666	462346	NA	rs7629480	NA	UKB-b-57	TRUE	reported	8KoqAI
17	43849366	T	C	0.024243	0.003626	2.30E-11	0.224757	462346	NA	rs7591667	NA	UKB-b-57	TRUE	reported	8KoqAI
17	43849415	T	C	0.024083	0.003608	2.50E-11	0.229271	462346	NA	rs7973087	NA	UKB-b-57	TRUE	reported	8KoqAI
17	43849656	C	A	0.024179	0.003625	2.60E-11	0.224634	462346	NA	rs6205593	NA	UKB-b-57	TRUE	reported	8KoqAI
17	43849787	A	T	0.024187	0.003625	2.50E-11	0.224641	462346	NA	rs6205593	NA	UKB-b-57	TRUE	reported	8KoqAI
17	43849896	A	G	0.024188	0.003625	2.50E-11	0.224641	462346	NA	rs6205594	NA	UKB-b-57	TRUE	reported	8KoqAI
17	43850519	C	T	0.024188	0.003625	2.50E-11	0.22464	462346	NA	rs7688572	NA	UKB-b-57	TRUE	reported	8KoqAI
17	43850645	C	T	0.024331	0.003616	1.70E-11	0.226436	462346	NA	rs6081441	NA	UKB-b-57	TRUE	reported	8KoqAI
17	43850966	A	G	0.02419	0.003625	2.50E-11	0.224639	462346	NA	rs6205594	NA	UKB-b-57	TRUE	reported	8KoqAI
17	43851018	T	C	0.024187	0.003625	2.50E-11	0.22464	462346	NA	rs6205594	NA	UKB-b-57	TRUE	reported	8KoqAI
17	43851851	G	A	0.024163	0.003625	2.60E-11	0.224619	462346	NA	rs6205594	NA	UKB-b-57	TRUE	reported	8KoqAI
17	43851971	C	A	0.024162	0.003625	2.70E-11	0.224622	462346	NA	rs6205594	NA	UKB-b-57	TRUE	reported	8KoqAI
17	43852605	C	T	0.024081	0.003635	3.50E-11	0.223735	462346	NA	rs6205594	NA	UKB-b-57	TRUE	reported	8KoqAI
17	43852621	T	C	0.02407	0.003635	3.60E-11	0.223731	462346	NA	rs6205594	NA	UKB-b-57	TRUE	reported	8KoqAI
17	43852733	G	A	0.024199	0.003635	2.80E-11	0.223904	462346	NA	rs1138711	NA	UKB-b-57	TRUE	reported	8KoqAI
17	43852742	T	C	0.024196	0.003635	2.80E-11	0.223982	462346	NA	rs1113709	NA	UKB-b-57	TRUE	reported	8KoqAI
17	43853109	G	A	0.024101	0.003626	3.00E-11	0.224561	462346	NA	rs5578710	NA	UKB-b-57	TRUE	reported	8KoqAI
17	43853133	T	C	0.024141	0.003625	2.80E-11	0.224616	462346	NA	rs6205595	NA	UKB-b-57	TRUE	reported	8KoqAI
17	43853235	T	G	0.024161	0.003625	2.70E-11	0.224619	462346	NA	rs5604307	NA	UKB-b-57	TRUE	reported	8KoqAI
17	43853457	A	G	0.024161	0.003625	2.70E-11	0.224619	462346	NA	rs7796565	NA	UKB-b-57	TRUE	reported	8KoqAI
17	43853526	A	G	0.024161	0.003625	2.70E-11	0.224619	462346	NA	rs5584994	NA	UKB-b-57	TRUE	reported	8KoqAI
17	43853922	G	A	0.024238	0.003625	2.30E-11	0.224714	462346	NA	rs5630303	NA	UKB-b-57	TRUE	reported	8KoqAI
17	43854267	C	T	0.024162	0.003625	2.60E-11	0.224618	462346	NA	rs5599191	NA	UKB-b-57	TRUE	reported	8KoqAI

17	43854340	A	G	0.024149	0.003625	2.70E-11	0.224639	462346	NA	rs6205595	NA	UKB-b-57	TRUE	reported	8KoqAI
17	43854449	C	T	0.023947	0.003625	3.90E-11	0.225087	462346	NA	rs6205595	NA	UKB-b-57	TRUE	reported	8KoqAI
17	43854536	C	A	0.02417	0.003625	2.60E-11	0.224654	462346	NA	rs6205595	NA	UKB-b-57	TRUE	reported	8KoqAI
17	43854655	G	C	0.018272	0.003137	5.70E-09	0.370282	462346	NA	rs3418614	NA	UKB-b-57	TRUE	reported	8KoqAI
17	43855228	G	T	0.02416	0.003625	2.60E-11	0.224784	462346	NA	rs4074462	NA	UKB-b-57	TRUE	reported	8KoqAI
17	43856458	G	A	0.018262	0.003137	5.80E-09	0.369833	462346	NA	rs3507662	NA	UKB-b-57	TRUE	reported	8KoqAI
17	43856639	C	G	0.023998	0.003626	3.60E-11	0.224167	462346	NA	rs6205706	NA	UKB-b-57	TRUE	reported	8KoqAI
17	43856710	G	A	0.024159	0.003626	2.70E-11	0.224536	462346	NA	rs6205706	NA	UKB-b-57	TRUE	reported	8KoqAI
17	43856730	C	T	0.024144	0.003629	2.90E-11	0.224312	462346	NA	rs6205706	NA	UKB-b-57	TRUE	reported	8KoqAI
17	43857033	C	T	0.024161	0.003626	2.70E-11	0.22454	462346	NA	rs6205706	NA	UKB-b-57	TRUE	reported	8KoqAI
17	43857129	A	C	0.024172	0.003625	2.60E-11	0.224641	462346	NA	rs7683009	NA	UKB-b-57	TRUE	reported	8KoqAI
17	43858187	G	T	0.024268	0.003626	2.20E-11	0.22456	462346	NA	rs6205706	NA	UKB-b-57	TRUE	reported	8KoqAI
17	43858326	C	T	0.024174	0.003625	2.60E-11	0.224641	462346	NA	rs6205706	NA	UKB-b-57	TRUE	reported	8KoqAI
17	43858482	C	T	0.024137	0.003625	2.80E-11	0.224724	462346	NA	rs7891749	NA	UKB-b-57	TRUE	reported	8KoqAI
17	43858629	A	G	0.024175	0.003625	2.60E-11	0.224645	462346	NA	rs6205706	NA	UKB-b-57	TRUE	reported	8KoqAI
17	43859065	T	C	0.024178	0.003625	2.60E-11	0.224652	462346	NA	rs6205706	NA	UKB-b-57	TRUE	reported	8KoqAI
17	43859640	A	G	0.024182	0.003625	2.50E-11	0.224655	462346	NA	rs6205707	NA	UKB-b-57	TRUE	reported	8KoqAI
17	43859691	C	G	0.024182	0.003625	2.50E-11	0.224655	462346	NA	rs6205707	NA	UKB-b-57	TRUE	reported	8KoqAI
17	43861117	C	T	0.024049	0.003628	3.40E-11	0.224493	462346	NA	rs6205707	NA	UKB-b-57	TRUE	reported	8KoqAI
17	43871982	C	T	0.024102	0.003629	3.10E-11	0.224216	462346	NA	rs5631990	NA	UKB-b-57	TRUE	reported	8KoqAI
17	43872228	G	A	0.024236	0.003629	2.40E-11	0.224143	462346	NA	rs5594304	NA	UKB-b-57	TRUE	reported	8KoqAI
17	43879308	A	G	0.024181	0.003623	2.50E-11	0.224849	462346	NA	rs8018415	NA	UKB-b-57	TRUE	reported	8KoqAI
17	43881790	C	T	0.024138	0.003623	2.70E-11	0.2248	462346	NA	rs1768937	NA	UKB-b-57	TRUE	reported	8KoqAI
17	43885291	G	A	0.024155	0.003623	2.60E-11	0.224845	462346	NA	rs6205710	NA	UKB-b-57	TRUE	reported	8KoqAI
17	43887480	T	C	0.024344	0.003625	1.90E-11	0.225477	462346	NA	rs6205710	NA	UKB-b-57	TRUE	reported	8KoqAI
17	43892784	T	G	0.023909	0.003658	6.30E-11	0.222428	462346	NA	rs5591591	NA	UKB-b-57	TRUE	reported	8KoqAI
17	43892788	G	A	0.023892	0.00366	6.60E-11	0.22192	462346	NA	rs5566836	NA	UKB-b-57	TRUE	reported	8KoqAI
17	43892973	T	C	0.024168	0.003623	2.60E-11	0.224898	462346	NA	rs1768947	NA	UKB-b-57	TRUE	reported	8KoqAI
17	43893259	G	A	0.024107	0.003631	3.20E-11	0.224229	462346	NA	rs1173659	NA	UKB-b-57	TRUE	reported	8KoqAI
17	43893260	T	C	0.02416	0.003623	2.60E-11	0.224863	462346	NA	rs1176465	NA	UKB-b-57	TRUE	reported	8KoqAI
17	43893403	G	A	0.024139	0.003623	2.70E-11	0.224861	462346	NA	rs1776276	NA	UKB-b-57	TRUE	reported	8KoqAI
17	43893716	C	T	0.024048	0.003633	3.60E-11	0.224224	462346	NA	rs8072451	NA	UKB-b-57	TRUE	reported	8KoqAI
17	43893751	A	G	0.024082	0.003635	3.50E-11	0.224049	462346	NA	rs8073146	NA	UKB-b-57	TRUE	reported	8KoqAI
17	43894102	T	C	0.024147	0.003624	2.70E-11	0.224844	462346	NA	rs2836402	NA	UKB-b-57	TRUE	reported	8KoqAI
17	43894159	C	T	0.024171	0.003624	2.60E-11	0.224816	462346	NA	rs2836402	NA	UKB-b-57	TRUE	reported	8KoqAI
17	43894510	G	A	0.024133	0.003623	2.70E-11	0.224848	462346	NA	rs5577914	NA	UKB-b-57	TRUE	reported	8KoqAI
17	43894547	C	T	0.024046	0.003625	3.30E-11	0.224648	462346	NA	rs5635754	NA	UKB-b-57	TRUE	reported	8KoqAI
17	43894609	A	G	0.024046	0.003625	3.30E-11	0.224647	462346	NA	rs5609954	NA	UKB-b-57	TRUE	reported	8KoqAI
17	43894990	T	G	0.024137	0.003624	2.70E-11	0.22484	462346	NA	rs739645	NA	UKB-b-57	TRUE	reported	8KoqAI
17	43895008	C	G	0.024131	0.003623	2.70E-11	0.224846	462346	NA	rs739644	NA	UKB-b-57	TRUE	reported	8KoqAI
17	43895501	G	C	0.024169	0.003624	2.60E-11	0.224822	462346	NA	rs4564621	NA	UKB-b-57	TRUE	reported	8KoqAI
17	43895530	C	T	0.02419	0.003624	2.50E-11	0.224791	462346	NA	rs2316763	NA	UKB-b-57	TRUE	reported	8KoqAI
17	43895602	T	G	0.024286	0.003625	2.10E-11	0.224712	462346	NA	rs2316764	NA	UKB-b-57	TRUE	reported	8KoqAI
17	43895653	A	G	0.024263	0.003625	2.20E-11	0.224743	462346	NA	rs4277389	NA	UKB-b-57	TRUE	reported	8KoqAI
17	43895696	G	A	0.024185	0.003624	2.50E-11	0.22481	462346	NA	rs4566211	NA	UKB-b-57	TRUE	reported	8KoqAI
17	43895751	G	A	0.02419	0.003624	2.50E-11	0.224808	462346	NA	rs4566212	NA	UKB-b-57	TRUE	reported	8KoqAI
17	43895797	T	C	0.024267	0.003625	2.20E-11	0.224732	462346	NA	rs4309444	NA	UKB-b-57	TRUE	reported	8KoqAI
17	43896032	C	T	0.024153	0.003624	2.70E-11	0.224808	462346	NA	rs6205710	NA	UKB-b-57	TRUE	reported	8KoqAI
17	43896228	T	C	0.024126	0.003623	2.80E-11	0.224839	462346	NA	rs1215039	NA	UKB-b-57	TRUE	reported	8KoqAI
17	43896528	C	G	0.02422	0.003624	2.30E-11	0.22476	462346	NA	rs1768960	NA	UKB-b-57	TRUE	reported	8KoqAI
17	43896616	T	C	0.024223	0.003624	2.30E-11	0.224761	462346	NA	rs6205710	NA	UKB-b-57	TRUE	reported	8KoqAI
17	43896637	T	C	0.024223	0.003624	2.30E-11	0.224761	462346	NA	rs6205710	NA	UKB-b-57	TRUE	reported	8KoqAI
17	43896690	T	C	0.02432	0.003624	1.90E-11	0.224914	462346	NA	rs7807412	NA	UKB-b-57	TRUE	reported	8KoqAI
17	43896734	T	C	0.02422	0.003624	2.30E-11	0.22476	462346	NA	rs6205711	NA	UKB-b-57	TRUE	reported	8KoqAI
17	43897130	A	G	0.024091	0.003623	3.00E-11	0.224913	462346	NA	rs6205711	NA	UKB-b-57	TRUE	reported	8KoqAI
17	43897202	T	A	0.024148	0.003624	2.70E-11	0.224828	462346	NA	rs6205711	NA	UKB-b-57	TRUE	reported	8KoqAI
17	43897246	A	G	0.024157	0.003624	2.60E-11	0.224825	462346	NA	rs7858710	NA	UKB-b-57	TRUE	reported	8KoqAI
17	43897449	A	T	0.024223	0.003624	2.30E-11	0.22476	462346	NA	rs6205711	NA	UKB-b-57	TRUE	reported	8KoqAI
17	43897480	A	G	0.024145	0.003623	2.70E-11	0.224897	462346	NA	rs7850618	NA	UKB-b-57	TRUE	reported	8KoqAI
17	43897722	T	C	0.024202	0.003624	2.40E-11	0.224714	462346	NA	rs7960014	NA	UKB-b-57	TRUE	reported	8KoqAI
17	43898459	C	T	0.024297	0.003625	2.10E-11	0.224633	462346	NA	rs1117396	NA	UKB-b-57	TRUE	reported	8KoqAI
17	43898887	T	C	0.024199	0.003624	2.40E-11	0.224782	462346	NA	rs1776288	NA	UKB-b-57	TRUE	reported	8KoqAI
17	43898963	A	T	0.024196	0.003624	2.50E-11	0.224783	462346	NA	rs1768965	NA	UKB-b-57	TRUE	reported	8KoqAI
17	43899161	A	C	0.024169	0.003626	2.60E-11	0.224616	462346	NA	rs1776291	NA	UKB-b-57	TRUE	reported	8KoqAI
17	43899401	C	T	0.024177	0.003625	2.60E-11	0.224713	462346	NA	rs6205711	NA	UKB-b-57	TRUE	reported	8KoqAI
17	43899417	G	C	0.024177	0.003625	2.60E-11	0.224712	462346	NA	rs6205711	NA	UKB-b-57	TRUE	reported	8KoqAI
17	43899611	C	T	0.024178	0.003625	2.60E-11	0.224713	462346	NA	rs7891747	NA	UKB-b-57	TRUE	reported	8KoqAI
17	43899655	T	C	0.02416	0.003626	2.70E-11	0.224663	462346	NA	rs6205711	NA	UKB-b-57	TRUE	reported	8KoqAI
17	43899657	C	G	0.024158	0.003626	2.70E-11	0.22465	462346	NA	rs6205711	NA	UKB-b-57	TRUE	reported	8KoqAI
17	43899727	A	G	0.024312	0.003627	2.00E-11	0.224502	462346	NA	rs6205711	NA	UKB-b-57	TRUE	reported	8KoqAI
17	43899736	G	A	0.024357	0.003626	1.90E-11	0.224543	462346	NA	rs6205711	NA	UKB-b-57	TRUE	reported	8KoqAI
17	43899786	C	T	0.024349	0.003626	1.90E-11	0.224584	462346	NA	rs1776295	NA	UKB-b-57	TRUE	reported	8KoqAI
17	43900434	A	G	0.024193	0.003625	2.50E-11	0.224695	462346	NA	rs5563841	NA	UKB-b-57	TRUE	reported	8KoqAI
17	43900697	C	T	0.024213	0.003624	2.40E-11	0.224762	462346	NA	rs7950114	NA	UKB-b-57	TRUE	reported	8KoqAI
17	43900817	A	G	0.024216	0.003624	2.40E-11	0.224771	462346	NA	rs1121371	NA	UKB-b-57	TRUE	reported	8KoqAI
17	43901001	C	T	0.024135	0.003625	2.80E-11	0.22479	462346	NA	rs6205712	NA	UKB-b-57	TRUE	reported	8KoqAI
17	43901238	A	G	0.024212	0.003624	2.40E-11	0.224765	462346	NA	rs6205712	NA	UKB-b-57	TRUE	reported	8KoqAI
17	43901528	T	C	0.024117	0.003623	2.80E-11	0.224838	462346	NA	rs6205714	NA	UKB-b-57	TRUE	reported	8KoqAI
17	43901558	A	G	0.024143	0.003623	2.70E-11	0.22486	462346	NA	rs6205714	NA	UKB-b-57	TRUE	reported	8KoqAI
17	43902216	C	T	0.024116	0.003623	2.80E-11	0.224837	462346	NA	rs4335809	NA	UKB-b-57	TRUE	reported	8KoqAI
17	43902505	C	T	0.024115	0.003623	2.80E-11	0.224831	462346	NA	rs4341787	NA	UKB-b-57	TRUE	reported	8KoqAI
17	43902522	G	A	0.024115	0.003623	2.80E-11	0.224831	462346	NA	rs4523962	NA	UKB-b-57	TRUE	reported	8KoqAI
17	43902541	A	G	0.025411	0.003846	3.90E-11	0.197209	462346	NA	rs4327090	NA	UKB-b-57	TRUE	reported	8KoqAI
17	43902738	C	A	0.024124	0.003624	2.80E-11	0.224832	462346	NA	rs3885074	NA	UKB-b-57	TRUE	reported	8KoqAI
17	43902799	A	G	0.024118	0.003624	2.80E-11	0.224832	462346	NA	rs3885075	NA	UKB-b-57	TRUE	reported	8KoqAI
17	43902842	C	T	0.024117	0.003624	2.80E-11	0.224832	462346	NA	rs4128011	NA	UKB-b-57	TRUE	reported	8KoqAI
17	43902861	C	A	0.024117	0.003624	2.80E-11	0.224831	462346	NA	rs3428325	NA	UKB-b-57	TRUE	reported	8KoqAI
17	43902944	C	T	0.024108	0.003624	2.90E-11	0.22484	462346	NA	rs1912151	NA	UKB-b-57	TRUE	reported	8KoqAI
17	43902997	G	A	0.024102	0.003624	2.90E-11	0.224833	462346	NA	rs1396862	NA	UKB-b-57	TRUE	reported	8KoqAI
17	43903089	C	T	0.024107	0.003624	2.90E-11	0.224835	462346	NA	rs4128011	NA	UKB-b-57	TRUE	reported	8KoqAI
17	43903106	A	G	0.024111	0.003624	2.90E-11	0.224827	462346	NA	rs6205714	NA	UKB-b-57	TRUE	reported	8KoqAI
17	43903298	G	A	0.024184	0.003623	2.50E-11	0.225302	462346	NA	rs6205714	NA	UKB-b-57	TRUE	reported	8KoqAI
17	43903336	G	A	0.024318	0.003632	2.20E-11	0.223977	462346	NA	rs1776305	NA	UKB-b-57	TRUE	reported	8KoqAI
17	43903485	G	C	0.024117	0.003624	2.80E-11	0.224838	462346	NA	rs6205714	NA	UKB-b-57	TRUE	reported	8KoqAI
17	43903546	A	G	0.024118	0.003624	2.80E-11	0.224836	462346	NA	rs6205714	NA	UKB-b-57	TRUE	reported	8KoqAI
17	43903548	C	T	0.024118	0.003624	2.80E-11	0.224836	462346	NA	rs6205715	NA	UKB-b-57	TRUE	reported	8KoqAI
17	43903842	C	T	0.023711	0.00359	4.00E-11	0.23224	462346	NA	rs6205715	NA	UKB-b-57	TRUE	reported	8KoqAI
17	43904110	G	C	0.024115	0.003624	2.80E-11	0.22483	462346	NA	rs6205715	NA	UKB-b-57	TRUE	reported	8KoqAI
17	43904397	C	T	0.024115	0.003624	2.80E-11	0.22483	462346	NA	rs1768982	NA	UKB-b-57	TRUE	reported	8KoqAI
17	43904528	T	C	0.024118	0.003624	2.80E-11	0.22483	462346	NA	rs6205715	NA	UKB-b-57	TRUE	reported	8KoqAI
17	43904610	C	T	0.024121	0.003624	2.80E-11	0.224832	462346	NA	rs6205715	NA	UKB-b-57	TRUE	reported	8KoqAI
17	43904673	C	T	0.024117	0.003624	2.80E-11	0.224831	462346	NA	rs6205715	NA	UKB-b-57	TRUE	reported	8KoqAI
17	43904948	G	C	0.024116	0.003624	2.80E-11	0.22483	462346	NA	rs6205715	NA	UKB-b-57	TRUE	reported	8KoqAI
17	43905134	G	A	0.02412	0.003624	2.80E-11	0.22483	462346	NA	rs7887265	NA	UKB-b-57	TRUE	reported	8KoqAI
17	43905313	G	C	0.024115	0.003624	2.80E-11	0.224829	462346	NA	rs6205715	NA	UKB-b-57	TRUE	reported	8KoqAI
17	43905481	T	G	0.024098	0.003624	2.90E-11	0.224831	462346	NA	rs1776308	NA	UKB-b-57	TRUE	reported	8KoqAI
17	43906726	A	C	0.024161	0.003626	2.70E-11	0.224771	462346	NA	rs1742575	NA	UKB-b-57	TRUE	reported	8KoqAI

17	43906828	G	A	0.024034	0.003626	3.40E-11	0.22449	462346	NA	rs1768988	NA	UKB-b-57	TRUE	reported	8KoqAI
17	43907143	G	A	0.024091	0.003624	3.00E-11	0.224819	462346	NA	rs6205715	NA	UKB-b-57	TRUE	reported	8KoqAI
17	43907745	C	T	0.024107	0.003624	2.90E-11	0.224828	462346	NA	rs1876831	NA	UKB-b-57	TRUE	reported	8KoqAI
17	43907896	T	C	0.024106	0.003624	2.90E-11	0.224828	462346	NA	rs1694066	NA	UKB-b-57	TRUE	reported	8KoqAI
17	43907966	G	A	0.024099	0.003624	2.90E-11	0.224816	462346	NA	rs1694066	NA	UKB-b-57	TRUE	reported	8KoqAI
17	43908151	C	T	0.023984	0.003632	4.00E-11	0.223917	462346	NA	rs1694067	NA	UKB-b-57	TRUE	reported	8KoqAI
17	43908152	C	T	0.023958	0.003632	4.20E-11	0.22398	462346	NA	rs1694067	NA	UKB-b-57	TRUE	reported	8KoqAI
17	43908773	G	C	0.024108	0.003624	2.90E-11	0.224826	462346	NA	rs5576379	NA	UKB-b-57	TRUE	reported	8KoqAI
17	43908826	T	G	0.024109	0.003624	2.90E-11	0.224825	462346	NA	rs5586570	NA	UKB-b-57	TRUE	reported	8KoqAI
17	43908989	C	T	0.024174	0.003642	3.20E-11	0.223251	462346	NA	rs6205476	NA	UKB-b-57	TRUE	reported	8KoqAI
17	43909008	C	T	0.02417	0.003642	3.20E-11	0.223251	462346	NA	rs6205476	NA	UKB-b-57	TRUE	reported	8KoqAI
17	43909022	G	T	0.024168	0.003643	3.30E-11	0.223251	462346	NA	rs6205476	NA	UKB-b-57	TRUE	reported	8KoqAI
17	43910088	G	A	0.024112	0.003626	2.90E-11	0.224664	462346	NA	rs1768991	NA	UKB-b-57	TRUE	reported	8KoqAI
17	43910183	G	A	0.024046	0.003624	3.30E-11	0.224762	462346	NA	rs1776319	NA	UKB-b-57	TRUE	reported	8KoqAI
17	43910262	C	G	0.024103	0.003624	2.90E-11	0.224826	462346	NA	rs6205476	NA	UKB-b-57	TRUE	reported	8KoqAI
17	43910507	C	T	0.024103	0.003624	2.90E-11	0.224827	462346	NA	rs1694067	NA	UKB-b-57	TRUE	reported	8KoqAI
17	43911036	G	A	0.024123	0.003624	2.80E-11	0.224816	462346	NA	rs1694067	NA	UKB-b-57	TRUE	reported	8KoqAI
17	43911352	C	T	0.024102	0.003624	2.90E-11	0.224827	462346	NA	rs1876830	NA	UKB-b-57	TRUE	reported	8KoqAI
17	43911424	C	T	0.024092	0.003624	3.00E-11	0.224809	462346	NA	rs4145704	NA	UKB-b-57	TRUE	reported	8KoqAI
17	43911443	T	C	0.024102	0.003624	2.90E-11	0.224827	462346	NA	rs1876829	NA	UKB-b-57	TRUE	reported	8KoqAI
17	43911525	C	T	0.024102	0.003624	2.90E-11	0.224827	462346	NA	rs1876828	NA	UKB-b-57	TRUE	reported	8KoqAI
17	43911832	T	C	0.024101	0.003624	2.90E-11	0.224828	462346	NA	rs1876827	NA	UKB-b-57	TRUE	reported	8KoqAI
17	43911898	C	T	0.024146	0.003624	2.70E-11	0.224778	462346	NA	rs1694067	NA	UKB-b-57	TRUE	reported	8KoqAI
17	43912159	G	C	0.024138	0.003625	2.80E-11	0.224729	462346	NA	rs1694068	NA	UKB-b-57	TRUE	reported	8KoqAI
17	43912282	C	T	0.024074	0.003624	3.10E-11	0.224825	462346	NA	rs2836402	NA	UKB-b-57	TRUE	reported	8KoqAI
17	43912454	T	C	0.024102	0.003624	2.90E-11	0.224824	462346	NA	rs2316765	NA	UKB-b-57	TRUE	reported	8KoqAI
17	43912490	C	G	0.024105	0.003624	2.90E-11	0.224827	462346	NA	rs878886	NA	UKB-b-57	TRUE	reported	8KoqAI
17	43912582	C	T	0.024146	0.003624	2.70E-11	0.224807	462346	NA	rs878887	NA	UKB-b-57	TRUE	reported	8KoqAI
17	43912635	A	G	0.024144	0.003624	2.70E-11	0.224802	462346	NA	rs878888	NA	UKB-b-57	TRUE	reported	8KoqAI
17	43912723	T	C	0.02414	0.003624	2.70E-11	0.224806	462346	NA	rs4525537	NA	UKB-b-57	TRUE	reported	8KoqAI
17	43912786	G	C	0.024144	0.003624	2.70E-11	0.224802	462346	NA	rs4640231	NA	UKB-b-57	TRUE	reported	8KoqAI
17	43912830	T	C	0.024144	0.003624	2.70E-11	0.224802	462346	NA	rs4482334	NA	UKB-b-57	TRUE	reported	8KoqAI
17	43913315	C	T	0.024101	0.003624	2.90E-11	0.22483	462346	NA	rs5612711	NA	UKB-b-57	TRUE	reported	8KoqAI
17	43913544	G	T	-0.01687	0.00307	3.90E-08	0.566457	462346	NA	rs242948	NA	UKB-b-57	TRUE	reported	8KoqAI
17	43913557	T	G	0.024571	0.003666	2.10E-11	0.221463	462346	NA	rs7510459	NA	UKB-b-57	TRUE	reported	8KoqAI
17	43913558	T	G	0.024572	0.003667	2.10E-11	0.221458	462346	NA	rs7499828	NA	UKB-b-57	TRUE	reported	8KoqAI
17	43914554	C	A	0.024109	0.003624	2.90E-11	0.224835	462346	NA	rs1044536	NA	UKB-b-57	TRUE	reported	8KoqAI
17	43914558	G	A	0.024109	0.003624	2.90E-11	0.224835	462346	NA	rs1044536	NA	UKB-b-57	TRUE	reported	8KoqAI
17	43914598	G	C	0.024109	0.003624	2.90E-11	0.224834	462346	NA	rs6205480	NA	UKB-b-57	TRUE	reported	8KoqAI
17	43914728	G	T	0.024092	0.003624	3.00E-11	0.224827	462346	NA	rs6205480	NA	UKB-b-57	TRUE	reported	8KoqAI
17	43914809	C	T	0.024194	0.003623	2.40E-11	0.225091	462346	NA	rs6205480	NA	UKB-b-57	TRUE	reported	8KoqAI
17	43915054	A	G	0.024111	0.003624	2.90E-11	0.224835	462346	NA	rs6205480	NA	UKB-b-57	TRUE	reported	8KoqAI
17	43915312	C	T	0.024111	0.003624	2.90E-11	0.224835	462346	NA	rs6205480	NA	UKB-b-57	TRUE	reported	8KoqAI
17	43916356	G	A	0.024091	0.003624	3.00E-11	0.224834	462346	NA	rs1044536	NA	UKB-b-57	TRUE	reported	8KoqAI
17	43916509	A	G	0.024107	0.003624	2.90E-11	0.224832	462346	NA	rs1044533	NA	UKB-b-57	TRUE	reported	8KoqAI
17	43916773	A	C	0.024003	0.003624	3.50E-11	0.224916	462346	NA	rs1769017	NA	UKB-b-57	TRUE	reported	8KoqAI
17	43916932	G	A	0.024081	0.003624	3.00E-11	0.224797	462346	NA	rs7832842	NA	UKB-b-57	TRUE	reported	8KoqAI
17	43917086	A	G	0.024119	0.003624	2.80E-11	0.224833	462346	NA	rs7769226	NA	UKB-b-57	TRUE	reported	8KoqAI
17	43917136	G	A	-0.01672	0.003056	4.40E-08	0.563754	462346	NA	rs242947	NA	UKB-b-57	TRUE	reported	8KoqAI
17	43917776	G	T	0.024112	0.003624	2.90E-11	0.224827	462346	NA	rs5602397	NA	UKB-b-57	TRUE	reported	8KoqAI
17	43917818	G	A	0.024115	0.003624	2.80E-11	0.224832	462346	NA	rs1776351	NA	UKB-b-57	TRUE	reported	8KoqAI
17	43918190	T	C	0.024107	0.003624	2.90E-11	0.224836	462346	NA	rs1776353	NA	UKB-b-57	TRUE	reported	8KoqAI
17	43918239	C	T	0.024149	0.003624	2.70E-11	0.224799	462346	NA	rs6205480	NA	UKB-b-57	TRUE	reported	8KoqAI
17	43918418	A	G	0.024083	0.003624	3.00E-11	0.224832	462346	NA	rs1125837	NA	UKB-b-57	TRUE	reported	8KoqAI
17	43918524	A	G	0.024107	0.003624	2.90E-11	0.224838	462346	NA	rs7492228	NA	UKB-b-57	TRUE	reported	8KoqAI
17	43918613	T	C	0.024119	0.003624	2.80E-11	0.224907	462346	NA	rs5697166	NA	UKB-b-57	TRUE	reported	8KoqAI
17	43918651	C	G	0.024105	0.003624	2.90E-11	0.224807	462346	NA	rs6205481	NA	UKB-b-57	TRUE	reported	8KoqAI
17	43919105	C	T	0.024312	0.003657	3.00E-11	0.221898	462346	NA	rs2106785	NA	UKB-b-57	TRUE	reported	8KoqAI
17	43919301	T	C	0.024101	0.003624	2.90E-11	0.224838	462346	NA	rs5615080	NA	UKB-b-57	TRUE	reported	8KoqAI
17	43919884	T	G	0.024103	0.003624	2.90E-11	0.224842	462346	NA	rs1769031	NA	UKB-b-57	TRUE	reported	8KoqAI
17	43920300	T	C	0.024104	0.003624	2.90E-11	0.224843	462346	NA	rs5623367	NA	UKB-b-57	TRUE	reported	8KoqAI
17	43920378	C	T	0.024103	0.003624	2.90E-11	0.224843	462346	NA	rs5635480	NA	UKB-b-57	TRUE	reported	8KoqAI
17	43920411	T	C	0.0241	0.003624	2.90E-11	0.224838	462346	NA	rs5571821	NA	UKB-b-57	TRUE	reported	8KoqAI
17	43920974	T	C	0.024095	0.003624	2.90E-11	0.224848	462346	NA	rs1769032	NA	UKB-b-57	TRUE	reported	8KoqAI
17	43921210	G	T	0.024075	0.003624	3.10E-11	0.224854	462346	NA	rs1776359	NA	UKB-b-57	TRUE	reported	8KoqAI
17	43921572	T	A	0.024094	0.003624	2.90E-11	0.224848	462346	NA	rs6205481	NA	UKB-b-57	TRUE	reported	8KoqAI
17	43921754	T	C	0.024189	0.003624	2.50E-11	0.22477	462346	NA	rs1776363	NA	UKB-b-57	TRUE	reported	8KoqAI
17	43922942	T	C	0.023729	0.003623	5.70E-11	0.225583	462346	NA	rs6262125	NA	UKB-b-57	TRUE	reported	8KoqAI
17	43923180	G	A	-0.0168	0.003054	3.80E-08	0.564015	462346	NA	rs242944	NA	UKB-b-57	TRUE	reported	8KoqAI
17	43923266	G	A	0.024049	0.003624	3.20E-11	0.224776	462346	NA	rs6205481	NA	UKB-b-57	TRUE	reported	8KoqAI
17	43923654	G	C	0.024429	0.003624	1.60E-11	0.225566	462346	NA	rs1218523	NA	UKB-b-57	TRUE	reported	8KoqAI
17	43923683	A	G	0.023988	0.003623	3.60E-11	0.224914	462346	NA	rs1218526	NA	UKB-b-57	TRUE	reported	8KoqAI
17	43923703	C	T	0.024194	0.003625	2.50E-11	0.224706	462346	NA	rs1218523	NA	UKB-b-57	TRUE	reported	8KoqAI
17	43923934	T	C	0.024077	0.003623	3.00E-11	0.224838	462346	NA	rs1107972	NA	UKB-b-57	TRUE	reported	8KoqAI
17	43924073	T	C	0.023961	0.003623	3.80E-11	0.224852	462346	NA	rs1237312	NA	UKB-b-57	TRUE	reported	8KoqAI
17	43924130	G	A	0.02408	0.003623	3.00E-11	0.224834	462346	NA	rs1237313	NA	UKB-b-57	TRUE	reported	8KoqAI
17	43924200	C	G	0.024574	0.003641	1.50E-11	0.223274	462346	NA	rs1237314	NA	UKB-b-57	TRUE	reported	8KoqAI
17	43924219	T	C	0.024564	0.003643	1.50E-11	0.223184	462346	NA	rs1237312	NA	UKB-b-57	TRUE	reported	8KoqAI
17	43924231	G	A	0.024584	0.003643	1.50E-11	0.223183	462346	NA	rs1237314	NA	UKB-b-57	TRUE	reported	8KoqAI
17	43924337	A	C	0.024028	0.003617	3.10E-11	0.226209	462346	NA	rs1237316	NA	UKB-b-57	TRUE	reported	8KoqAI
17	43924521	G	A	0.024079	0.003623	3.00E-11	0.224844	462346	NA	rs1769066	NA	UKB-b-57	TRUE	reported	8KoqAI
17	43924776	C	T	0.024078	0.003623	3.00E-11	0.22484	462346	NA	rs5594382	NA	UKB-b-57	TRUE	reported	8KoqAI
17	43924803	A	G	0.024068	0.003624	3.10E-11	0.224814	462346	NA	rs1769067	NA	UKB-b-57	TRUE	reported	8KoqAI
17	43925297	C	T	0.025034	0.00343	2.90E-13	0.262687	462346	NA	rs1769070	NA	UKB-b-57	TRUE	reported	8KoqAI
17	43925605	G	A	0.023899	0.003613	3.70E-11	0.225398	462346	NA	rs1776949	NA	UKB-b-57	TRUE	reported	8KoqAI
17	43925729	A	G	0.023897	0.003613	3.70E-11	0.225401	462346	NA	rs7706376	NA	UKB-b-57	TRUE	reported	8KoqAI
17	43925966	G	A	0.023868	0.003613	4.00E-11	0.225464	462346	NA	rs6205481	NA	UKB-b-57	TRUE	reported	8KoqAI
17	43926056	A	C	0.023909	0.003613	3.70E-11	0.225401	462346	NA	rs6205481	NA	UKB-b-57	TRUE	reported	8KoqAI
17	43926149	A	G	0.023936	0.003614	3.50E-11	0.22526	462346	NA	rs6205481	NA	UKB-b-57	TRUE	reported	8KoqAI
17	43926755	A	G	0.023865	0.003614	4.00E-11	0.225377	462346	NA	rs6205482	NA	UKB-b-57	TRUE	reported	8KoqAI
17	43926948	A	G	0.023931	0.003617	3.70E-11	0.225078	462346	NA	rs5611321	NA	UKB-b-57	TRUE	reported	8KoqAI
17	43926992	C	T	0.023958	0.003616	3.50E-11	0.225188	462346	NA	rs5626913	NA	UKB-b-57	TRUE	reported	8KoqAI
17	43927255	G	T	0.023883	0.003613	3.80E-11	0.225464	462346	NA	rs5638575	NA	UKB-b-57	TRUE	reported	8KoqAI
17	43927290	G	A	0.024427	0.003641	2.00E-11	0.222043	462346	NA	rs1776955	NA	UKB-b-57	TRUE	reported	8KoqAI
17	43927708	A	G	0.023791	0.003613	4.60E-11	0.225543	462346	NA	rs6205482	NA	UKB-b-57	TRUE	reported	8KoqAI
17	43928614	T	G	0.023884	0.003613	3.80E-11	0.225714	462346	NA	rs885639	NA	UKB-b-57	TRUE	reported	8KoqAI
17	43929029	A	G	0.023929	0.003617	3.70E-11	0.225085	462346	NA	rs6205482	NA	UKB-b-57	TRUE	reported	8KoqAI
17	43929992	C	T	0.024011	0.003624	3.50E-11	0.224903	462346	NA	rs6205482	NA	UKB-b-57	TRUE	reported	8KoqAI
17	43930033	G	A	0.024019	0.003624	3.40E-11	0.224935	462346	NA	rs6205482	NA	UKB-b-57	TRUE	reported	8KoqAI
17	43930238	C	A	0.024053	0.003627	3.30E-11	0.224723	462346	NA	rs7958986	NA	UKB-b-57	TRUE	reported	8KoqAI
17	43930798	A	G	0.024059	0.003624	3.10E-11	0.224949	462346	NA	rs2316769	NA	UKB-b-57	TRUE	reported	8KoqAI
17	43930955	G	A	0.024146	0.003625	2.70E-11	0.224781	462346	NA	rs2873268	NA	UKB-b-57	TRUE	reported	8KoqAI
17	43930980	C	T	0.024241	0.003626	2.30E-11	0.22471	462346	NA	rs4340365	NA	UKB-b-57	TRUE	reported	8KoqAI
17	43931122	C	T	0.024049	0.003624	3.20E-11	0.224928	462346	NA	rs2873269	NA	UKB-b-57	TRUE	reported	8KoqAI
17	43931244	G	C	0.024224	0.003625	2.40E-11	0.224761	462346	NA	rs5602612	NA	UKB-b-57	TRUE	reported	8KoqAI
17	43931245	G	A	0.024097	0.003624	3.00E-11	0.224907	462346	NA	rs5632974	NA	UKB-b-57	TRUE	reported	8KoqAI
17	43931380	A	G	0.024067	0.003624	3.10E-11	0.224976	462346	NA	rs5628860	NA	UKB-b-57	TRUE	reported	8KoqAI

17	43931626	T	C	0.024104	0.003624	2.90E-11	0.224956	462346	NA	rs1769098	NA	UKB-b-57	TRUE	reported	8KoqAI
17	43931907	C	A	0.024065	0.003625	3.20E-11	0.22482	462346	NA	rs5614626	NA	UKB-b-57	TRUE	reported	8KoqAI
17	43932028	A	G	0.0242	0.003625	2.50E-11	0.225101	462346	NA	rs5594252	NA	UKB-b-57	TRUE	reported	8KoqAI
17	43932049	C	T	0.024219	0.003625	2.40E-11	0.225058	462346	NA	rs5625473	NA	UKB-b-57	TRUE	reported	8KoqAI
17	43932129	C	T	0.02406	0.003625	3.20E-11	0.225217	462346	NA	rs5632330	NA	UKB-b-57	TRUE	reported	8KoqAI
17	43932155	A	G	0.024102	0.003627	3.00E-11	0.224879	462346	NA	rs5575192	NA	UKB-b-57	TRUE	reported	8KoqAI
17	43932173	A	G	0.023845	0.003634	5.30E-11	0.22454	462346	NA	rs5566020	NA	UKB-b-57	TRUE	reported	8KoqAI
17	43932277	G	A	0.024025	0.003624	3.40E-11	0.224925	462346	NA	rs1138566	NA	UKB-b-57	TRUE	reported	8KoqAI
17	43932381	C	T	0.023994	0.003626	3.60E-11	0.224731	462346	NA	rs6205483	NA	UKB-b-57	TRUE	reported	8KoqAI
17	43932741	C	T	0.024997	0.003667	9.30E-12	0.221211	462346	NA	rs4627402	NA	UKB-b-57	TRUE	reported	8KoqAI
17	43932798	G	T	0.025099	0.00408	7.70E-10	0.177962	462346	NA	rs1044536	NA	UKB-b-57	TRUE	reported	8KoqAI
17	43933579	C	A	0.024189	0.003626	2.50E-11	0.224899	462346	NA	rs6205483	NA	UKB-b-57	TRUE	reported	8KoqAI
17	43933673	T	C	0.024202	0.003624	2.40E-11	0.224957	462346	NA	rs6205483	NA	UKB-b-57	TRUE	reported	8KoqAI
17	43933879	C	G	0.024153	0.003624	2.60E-11	0.224967	462346	NA	rs7656357	NA	UKB-b-57	TRUE	reported	8KoqAI
17	43934016	G	C	0.024108	0.003623	2.80E-11	0.225061	462346	NA	rs1119622	NA	UKB-b-57	TRUE	reported	8KoqAI
17	43934314	T	C	0.024107	0.003623	2.80E-11	0.225064	462346	NA	rs6205483	NA	UKB-b-57	TRUE	reported	8KoqAI
17	43934672	A	C	0.024188	0.003622	2.40E-11	0.22507	462346	NA	rs6205483	NA	UKB-b-57	TRUE	reported	8KoqAI
17	43934776	A	G	0.024192	0.003623	2.40E-11	0.225062	462346	NA	rs1044536	NA	UKB-b-57	TRUE	reported	8KoqAI
17	43934826	T	G	0.02426	0.003622	2.10E-11	0.225099	462346	NA	rs1044533	NA	UKB-b-57	TRUE	reported	8KoqAI
17	43934896	T	A	0.024283	0.003622	2.00E-11	0.225099	462346	NA	rs1044533	NA	UKB-b-57	TRUE	reported	8KoqAI
17	43934948	A	G	0.024297	0.003622	2.00E-11	0.225107	462346	NA	rs1044533	NA	UKB-b-57	TRUE	reported	8KoqAI
17	43935118	G	A	0.024166	0.003622	2.50E-11	0.225055	462346	NA	rs1044537	NA	UKB-b-57	TRUE	reported	8KoqAI
17	43935888	C	T	0.024174	0.003623	2.50E-11	0.225059	462346	NA	rs5599663	NA	UKB-b-57	TRUE	reported	8KoqAI
17	43936011	A	G	0.024152	0.003622	2.60E-11	0.22507	462346	NA	rs5588630	NA	UKB-b-57	TRUE	reported	8KoqAI
17	43936298	C	A	0.024152	0.003622	2.60E-11	0.22507	462346	NA	rs5613607	NA	UKB-b-57	TRUE	reported	8KoqAI
17	43937104	G	A	0.024179	0.003622	2.50E-11	0.225054	462346	NA	rs6205484	NA	UKB-b-57	TRUE	reported	8KoqAI
17	43937162	C	T	0.024179	0.003622	2.50E-11	0.225054	462346	NA	rs6205484	NA	UKB-b-57	TRUE	reported	8KoqAI
17	43937184	T	C	0.024118	0.003622	2.80E-11	0.225072	462346	NA	rs6205484	NA	UKB-b-57	TRUE	reported	8KoqAI
17	43937333	T	G	0.024155	0.003622	2.60E-11	0.22507	462346	NA	rs6205484	NA	UKB-b-57	TRUE	reported	8KoqAI
17	43937460	G	A	0.02418	0.003622	2.50E-11	0.225087	462346	NA	rs7662734	NA	UKB-b-57	TRUE	reported	8KoqAI
17	43937505	C	T	0.024186	0.003622	2.40E-11	0.225157	462346	NA	rs7594562	NA	UKB-b-57	TRUE	reported	8KoqAI
17	43937633	A	G	0.024181	0.003622	2.50E-11	0.225087	462346	NA	rs7988983	NA	UKB-b-57	TRUE	reported	8KoqAI
17	43937671	C	T	0.024181	0.003622	2.50E-11	0.225087	462346	NA	rs7762533	NA	UKB-b-57	TRUE	reported	8KoqAI
17	43937736	C	T	0.024179	0.003622	2.50E-11	0.225088	462346	NA	rs7748922	NA	UKB-b-57	TRUE	reported	8KoqAI
17	43938107	A	G	0.024158	0.003622	2.60E-11	0.225069	462346	NA	rs5588839	NA	UKB-b-57	TRUE	reported	8KoqAI
17	43938499	G	A	0.024159	0.003622	2.60E-11	0.225069	462346	NA	rs7632921	NA	UKB-b-57	TRUE	reported	8KoqAI
17	43938634	A	G	0.02416	0.003622	2.60E-11	0.225069	462346	NA	rs6205484	NA	UKB-b-57	TRUE	reported	8KoqAI
17	43938796	A	G	0.02416	0.003622	2.60E-11	0.225069	462346	NA	rs5564697	NA	UKB-b-57	TRUE	reported	8KoqAI
17	43938882	G	A	0.024157	0.003622	2.60E-11	0.225069	462346	NA	rs5567309	NA	UKB-b-57	TRUE	reported	8KoqAI
17	43939255	T	C	0.024196	0.003622	2.40E-11	0.225079	462346	NA	rs5634760	NA	UKB-b-57	TRUE	reported	8KoqAI
17	43939432	G	T	0.024165	0.003622	2.50E-11	0.225083	462346	NA	rs5624908	NA	UKB-b-57	TRUE	reported	8KoqAI
17	43939609	A	T	0.024141	0.003624	2.70E-11	0.224907	462346	NA	rs5622706	NA	UKB-b-57	TRUE	reported	8KoqAI
17	43939638	G	A	0.024142	0.003624	2.70E-11	0.224915	462346	NA	rs5571971	NA	UKB-b-57	TRUE	reported	8KoqAI
17	43939659	G	A	0.024137	0.003624	2.70E-11	0.224913	462346	NA	rs5629985	NA	UKB-b-57	TRUE	reported	8KoqAI
17	43939936	G	A	0.02416	0.003622	2.60E-11	0.225067	462346	NA	rs6205485	NA	UKB-b-57	TRUE	reported	8KoqAI
17	43940012	T	C	0.02434	0.003678	3.70E-11	0.22039	462346	NA	rs7521030	NA	UKB-b-57	TRUE	reported	8KoqAI
17	43940020	T	C	0.024339	0.003678	3.70E-11	0.220403	462346	NA	rs7494464	NA	UKB-b-57	TRUE	reported	8KoqAI
17	43940021	G	A	0.024387	0.003679	3.40E-11	0.220268	462346	NA	rs7941243	NA	UKB-b-57	TRUE	reported	8KoqAI
17	43940188	T	C	0.024162	0.003622	2.50E-11	0.225068	462346	NA	rs6205485	NA	UKB-b-57	TRUE	reported	8KoqAI
17	43940497	C	T	0.023961	0.003625	3.80E-11	0.224637	462346	NA	rs7940673	NA	UKB-b-57	TRUE	reported	8KoqAI
17	43940685	G	A	0.024163	0.003622	2.50E-11	0.225068	462346	NA	rs6205485	NA	UKB-b-57	TRUE	reported	8KoqAI
17	43940825	G	C	0.024163	0.003622	2.50E-11	0.225068	462346	NA	rs6205546	NA	UKB-b-57	TRUE	reported	8KoqAI
17	43941350	C	T	0.024165	0.003622	2.50E-11	0.225068	462346	NA	rs6205546	NA	UKB-b-57	TRUE	reported	8KoqAI
17	43941476	A	C	0.024165	0.003622	2.50E-11	0.225069	462346	NA	rs6205546	NA	UKB-b-57	TRUE	reported	8KoqAI
17	43941491	A	G	0.024164	0.003622	2.50E-11	0.22507	462346	NA	rs6205546	NA	UKB-b-57	TRUE	reported	8KoqAI
17	43941608	C	G	0.024165	0.003622	2.50E-11	0.225069	462346	NA	rs7765296	NA	UKB-b-57	TRUE	reported	8KoqAI
17	43941710	C	T	0.024174	0.003622	2.50E-11	0.225073	462346	NA	rs7569023	NA	UKB-b-57	TRUE	reported	8KoqAI
17	43941858	T	G	0.024166	0.003622	2.50E-11	0.225068	462346	NA	rs6205546	NA	UKB-b-57	TRUE	reported	8KoqAI
17	43942046	T	A	0.024166	0.003622	2.50E-11	0.225067	462346	NA	rs6205546	NA	UKB-b-57	TRUE	reported	8KoqAI
17	43942656	G	A	0.024167	0.003622	2.50E-11	0.225067	462346	NA	rs6205546	NA	UKB-b-57	TRUE	reported	8KoqAI
17	43942820	T	C	0.024167	0.003622	2.50E-11	0.225067	462346	NA	rs6205546	NA	UKB-b-57	TRUE	reported	8KoqAI
17	43943000	A	C	0.024176	0.003622	2.50E-11	0.225078	462346	NA	rs6205546	NA	UKB-b-57	TRUE	reported	8KoqAI
17	43943655	A	C	0.024219	0.003622	2.30E-11	0.2251	462346	NA	rs6205546	NA	UKB-b-57	TRUE	reported	8KoqAI
17	43943657	A	G	0.024214	0.003622	2.30E-11	0.225101	462346	NA	rs6205547	NA	UKB-b-57	TRUE	reported	8KoqAI
17	43943755	T	C	0.024215	0.003622	2.30E-11	0.225101	462346	NA	rs6205547	NA	UKB-b-57	TRUE	reported	8KoqAI
17	43943957	G	C	0.024204	0.003622	2.40E-11	0.225089	462346	NA	rs5632705	NA	UKB-b-57	TRUE	reported	8KoqAI
17	43943974	C	T	0.024168	0.003622	2.50E-11	0.225066	462346	NA	rs5603671	NA	UKB-b-57	TRUE	reported	8KoqAI
17	43944158	C	T	0.024169	0.003622	2.50E-11	0.225065	462346	NA	rs5578117	NA	UKB-b-57	TRUE	reported	8KoqAI
17	43944204	T	C	0.024178	0.003622	2.50E-11	0.225061	462346	NA	rs1215022	NA	UKB-b-57	TRUE	reported	8KoqAI
17	43944519	C	T	0.024165	0.003622	2.50E-11	0.225056	462346	NA	rs1215051	NA	UKB-b-57	TRUE	reported	8KoqAI
17	43945106	T	C	0.024161	0.003622	2.60E-11	0.225055	462346	NA	rs6205547	NA	UKB-b-57	TRUE	reported	8KoqAI
17	43945495	A	G	0.024168	0.003622	2.50E-11	0.225062	462346	NA	rs6205547	NA	UKB-b-57	TRUE	reported	8KoqAI
17	43945687	C	A	0.024165	0.003622	2.50E-11	0.225061	462346	NA	rs6205547	NA	UKB-b-57	TRUE	reported	8KoqAI
17	43945855	G	A	0.024166	0.003622	2.50E-11	0.22506	462346	NA	rs7863743	NA	UKB-b-57	TRUE	reported	8KoqAI
17	43945931	T	C	0.024166	0.003622	2.50E-11	0.22506	462346	NA	rs2316771	NA	UKB-b-57	TRUE	reported	8KoqAI
17	43945938	C	T	0.024166	0.003622	2.50E-11	0.22506	462346	NA	rs6205547	NA	UKB-b-57	TRUE	reported	8KoqAI
17	43946112	T	C	0.024179	0.003622	2.50E-11	0.22508	462346	NA	rs1078830	NA	UKB-b-57	TRUE	reported	8KoqAI
17	43946223	G	A	0.023988	0.003618	3.30E-11	0.226264	462346	NA	rs753235	NA	UKB-b-57	TRUE	reported	8KoqAI
17	43946291	A	G	0.024279	0.003821	2.10E-10	0.203163	462346	NA	rs886249	NA	UKB-b-57	TRUE	reported	8KoqAI
17	43946318	T	C	0.023656	0.00363	7.10E-11	0.22703	462346	NA	rs753236	NA	UKB-b-57	TRUE	reported	8KoqAI
17	43946423	A	C	0.024138	0.003624	2.70E-11	0.225089	462346	NA	rs2316772	NA	UKB-b-57	TRUE	reported	8KoqAI
17	43946536	C	T	0.024165	0.003621	2.50E-11	0.225208	462346	NA	rs2316773	NA	UKB-b-57	TRUE	reported	8KoqAI
17	43946642	T	C	0.024179	0.003622	2.50E-11	0.225104	462346	NA	rs2316774	NA	UKB-b-57	TRUE	reported	8KoqAI
17	43946707	T	C	0.024203	0.003622	2.40E-11	0.225096	462346	NA	rs739643	NA	UKB-b-57	TRUE	reported	8KoqAI
17	43946838	C	A	0.024186	0.003622	2.40E-11	0.225059	462346	NA	rs739642	NA	UKB-b-57	TRUE	reported	8KoqAI
17	43946875	C	T	0.024189	0.003623	2.40E-11	0.225014	462346	NA	rs757055	NA	UKB-b-57	TRUE	reported	8KoqAI
17	43947488	A	G	0.024191	0.003622	2.40E-11	0.225064	462346	NA	rs5639850	NA	UKB-b-57	TRUE	reported	8KoqAI
17	43947928	A	G	0.024176	0.003622	2.50E-11	0.225048	462346	NA	rs5628936	NA	UKB-b-57	TRUE	reported	8KoqAI
17	43948040	C	T	0.024193	0.003622	2.40E-11	0.225064	462346	NA	rs6205548	NA	UKB-b-57	TRUE	reported	8KoqAI
17	43948159	G	A	0.024193	0.003622	2.40E-11	0.225064	462346	NA	rs7823804	NA	UKB-b-57	TRUE	reported	8KoqAI
17	43948733	C	T	0.024176	0.003622	2.50E-11	0.225048	462346	NA	rs5567334	NA	UKB-b-57	TRUE	reported	8KoqAI
17	43949008	C	T	0.02418	0.003622	2.50E-11	0.225047	462346	NA	rs7709248	NA	UKB-b-57	TRUE	reported	8KoqAI
17	43949092	T	G	0.024135	0.003622	2.70E-11	0.225062	462346	NA	rs6205548	NA	UKB-b-57	TRUE	reported	8KoqAI
17	43949342	G	A	0.02349	0.003633	1.00E-10	0.225247	462346	NA	rs3473955	NA	UKB-b-57	TRUE	reported	8KoqAI
17	43949448	C	A	0.024453	0.003644	1.90E-11	0.22337	462346	NA	rs3409734	NA	UKB-b-57	TRUE	reported	8KoqAI
17	43949892	C	T	0.02418	0.003622	2.50E-11	0.22505	462346	NA	rs2106784	NA	UKB-b-57	TRUE	reported	8KoqAI
17	43950195	T	C	0.024185	0.003622	2.40E-11	0.225046	462346	NA	rs2158072	NA	UKB-b-57	TRUE	reported	8KoqAI
17	43950394	C	T	0.024203	0.003622	2.40E-11	0.225062	462346	NA	rs6205548	NA	UKB-b-57	TRUE	reported	8KoqAI
17	43950533	A	G	0.024205	0.003622	2.30E-11	0.225061	462346	NA	rs6205549	NA	UKB-b-57	TRUE	reported	8KoqAI
17	43951120	T	C	0.024206	0.003622	2.30E-11	0.225061	462346	NA	rs6205549	NA	UKB-b-57	TRUE	reported	8KoqAI
17	43951220	T	G	0.024208	0.003622	2.30E-11	0.225061	462346	NA	rs3441605	NA	UKB-b-57	TRUE	reported	8KoqAI
17	43951345	C	A	0.0242	0.003622	2.40E-11	0.225068	462346	NA	rs6205549	NA	UKB-b-57	TRUE	reported	8KoqAI
17	43951717	G	A	0.024305	0.003623	2.00E-11	0.224984	462346	NA	rs2055794	NA	UKB-b-57	TRUE	reported	8KoqAI
17	43951995	C	T	0.024306	0.003623	2.00E-11	0.224984	462346	NA	rs2055795	NA	UKB-b-57	TRUE	reported	8KoqAI
17	43952191	A	T	0.024307	0.003623	2.00E-11	0.224983	462346	NA	rs2055796	NA	UKB-b-57	TRUE	reported	8KoqAI
17	43952634	A	T	0.024213	0.003622	2.30E-11	0.225059	462346	NA	rs8034838	NA	UKB-b-57	TRUE	reported	8KoqAI
17	43952944	T	C	0.024215	0.003622	2.30E-11	0.225054	462346	NA	rs6205549	NA	UKB-b-57	TRUE	reported	8KoqAI

17	43953016	C	T	0.024311	0.003623	1.90E-11	0.224977	462346	NA	rs6205549	NA	UKB-b-57	TRUE	reported	8KoqAI
17	43953043	A	C	0.024216	0.003622	2.30E-11	0.225054	462346	NA	rs6205549	NA	UKB-b-57	TRUE	reported	8KoqAI
17	43953307	C	T	0.024309	0.003623	1.90E-11	0.224983	462346	NA	rs6205549	NA	UKB-b-57	TRUE	reported	8KoqAI
17	43953529	A	G	0.02431	0.003623	1.90E-11	0.224982	462346	NA	rs6205549	NA	UKB-b-57	TRUE	reported	8KoqAI
17	43953695	T	C	0.024347	0.003641	2.30E-11	0.223534	462346	NA	rs2188888	NA	UKB-b-57	TRUE	reported	8KoqAI
17	43953707	C	T	0.024346	0.003641	2.30E-11	0.223523	462346	NA	rs3919455	NA	UKB-b-57	TRUE	reported	8KoqAI
17	43953719	G	C	0.02436	0.003641	2.20E-11	0.223529	462346	NA	rs2214258	NA	UKB-b-57	TRUE	reported	8KoqAI
17	43953814	G	A	0.024217	0.003622	2.30E-11	0.225059	462346	NA	rs6205549	NA	UKB-b-57	TRUE	reported	8KoqAI
17	43953991	C	G	0.024217	0.003622	2.30E-11	0.225058	462346	NA	rs6205549	NA	UKB-b-57	TRUE	reported	8KoqAI
17	43954134	G	A	0.024217	0.003622	2.30E-11	0.225058	462346	NA	rs6205550	NA	UKB-b-57	TRUE	reported	8KoqAI
17	43954227	G	A	0.024217	0.003622	2.30E-11	0.225058	462346	NA	rs6205550	NA	UKB-b-57	TRUE	reported	8KoqAI
17	43954284	G	A	0.024221	0.003622	2.30E-11	0.22506	462346	NA	rs6205550	NA	UKB-b-57	TRUE	reported	8KoqAI
17	43954686	G	A	0.024217	0.003622	2.30E-11	0.225058	462346	NA	rs916793	NA	UKB-b-57	TRUE	reported	8KoqAI
17	43955030	A	G	0.024217	0.003622	2.30E-11	0.225058	462346	NA	rs4441322	NA	UKB-b-57	TRUE	reported	8KoqAI
17	43955093	T	C	0.024217	0.003622	2.30E-11	0.225058	462346	NA	rs1134140	NA	UKB-b-57	TRUE	reported	8KoqAI
17	43955365	C	A	0.024217	0.003622	2.30E-11	0.225058	462346	NA	rs5590525	NA	UKB-b-57	TRUE	reported	8KoqAI
17	43955487	C	T	0.024233	0.003621	2.20E-11	0.225266	462346	NA	rs1769132	NA	UKB-b-57	TRUE	reported	8KoqAI
17	43956370	G	T	0.024204	0.003624	2.40E-11	0.224916	462346	NA	rs6205551	NA	UKB-b-57	TRUE	reported	8KoqAI
17	43956390	T	C	0.024205	0.003624	2.40E-11	0.224916	462346	NA	rs6205551	NA	UKB-b-57	TRUE	reported	8KoqAI
17	43956425	C	T	0.024204	0.003624	2.40E-11	0.224916	462346	NA	rs6205551	NA	UKB-b-57	TRUE	reported	8KoqAI
17	43956505	A	C	0.024207	0.003624	2.40E-11	0.22492	462346	NA	rs6205551	NA	UKB-b-57	TRUE	reported	8KoqAI
17	43956642	T	C	0.024207	0.003624	2.40E-11	0.22492	462346	NA	rs5636463	NA	UKB-b-57	TRUE	reported	8KoqAI
17	43956734	C	A	0.024201	0.003623	2.40E-11	0.224972	462346	NA	rs5576860	NA	UKB-b-57	TRUE	reported	8KoqAI
17	43956778	A	G	0.024218	0.003623	2.30E-11	0.224955	462346	NA	rs5565365	NA	UKB-b-57	TRUE	reported	8KoqAI
17	43957221	C	T	0.024221	0.003622	2.30E-11	0.225061	462346	NA	rs5589410	NA	UKB-b-57	TRUE	reported	8KoqAI
17	43957269	T	C	0.024248	0.003622	2.20E-11	0.225077	462346	NA	rs5610177	NA	UKB-b-57	TRUE	reported	8KoqAI
17	43957328	C	T	0.024222	0.003622	2.30E-11	0.225061	462346	NA	rs5624978	NA	UKB-b-57	TRUE	reported	8KoqAI
17	43958079	C	T	0.02414	0.00362	2.60E-11	0.225571	462346	NA	rs5604041	NA	UKB-b-57	TRUE	reported	8KoqAI
17	43958099	T	C	0.024226	0.003625	2.30E-11	0.224837	462346	NA	rs5572125	NA	UKB-b-57	TRUE	reported	8KoqAI
17	43958139	A	C	0.024183	0.003625	2.50E-11	0.224804	462346	NA	rs5587681	NA	UKB-b-57	TRUE	reported	8KoqAI
17	43958172	A	G	0.024133	0.003625	2.80E-11	0.224826	462346	NA	rs5594473	NA	UKB-b-57	TRUE	reported	8KoqAI
17	43958736	G	A	0.024214	0.003625	2.40E-11	0.224767	462346	NA	rs6205552	NA	UKB-b-57	TRUE	reported	8KoqAI
17	43958744	A	G	0.024178	0.003625	2.60E-11	0.224743	462346	NA	rs1129154	NA	UKB-b-57	TRUE	reported	8KoqAI
17	43959092	G	T	0.024313	0.003623	1.90E-11	0.22498	462346	NA	rs5634355	NA	UKB-b-57	TRUE	reported	8KoqAI
17	43959113	A	G	0.024313	0.003623	1.90E-11	0.22498	462346	NA	rs5597567	NA	UKB-b-57	TRUE	reported	8KoqAI
17	43959261	G	A	0.024219	0.003622	2.30E-11	0.225057	462346	NA	rs5638739	NA	UKB-b-57	TRUE	reported	8KoqAI
17	43959661	G	A	0.024218	0.003622	2.30E-11	0.225057	462346	NA	rs2019816	NA	UKB-b-57	TRUE	reported	8KoqAI
17	43959752	A	T	0.024218	0.003622	2.30E-11	0.225057	462346	NA	rs2006305	NA	UKB-b-57	TRUE	reported	8KoqAI
17	43959918	T	A	0.024219	0.003622	2.30E-11	0.225057	462346	NA	rs2019822	NA	UKB-b-57	TRUE	reported	8KoqAI
17	43960020	A	G	0.024219	0.003622	2.30E-11	0.225057	462346	NA	rs6205552	NA	UKB-b-57	TRUE	reported	8KoqAI
17	43960172	C	T	0.024219	0.003622	2.30E-11	0.225059	462346	NA	rs7958036	NA	UKB-b-57	TRUE	reported	8KoqAI
17	43960283	A	G	0.024217	0.003622	2.30E-11	0.225057	462346	NA	rs5593761	NA	UKB-b-57	TRUE	reported	8KoqAI
17	43960323	A	G	0.023872	0.003651	6.20E-11	0.222687	462346	NA	rs5636590	NA	UKB-b-57	TRUE	reported	8KoqAI
17	43960325	T	C	0.023908	0.003651	5.80E-11	0.222709	462346	NA	rs5576726	NA	UKB-b-57	TRUE	reported	8KoqAI
17	43960341	G	A	0.0239	0.003651	5.90E-11	0.222729	462346	NA	rs5612730	NA	UKB-b-57	TRUE	reported	8KoqAI
17	43960600	T	C	0.024224	0.003622	2.30E-11	0.225065	462346	NA	rs5619441	NA	UKB-b-57	TRUE	reported	8KoqAI
17	43960812	A	T	0.024224	0.003622	2.30E-11	0.225061	462346	NA	rs5633848	NA	UKB-b-57	TRUE	reported	8KoqAI
17	43960911	A	G	0.024145	0.003622	2.60E-11	0.225247	462346	NA	rs5618021	NA	UKB-b-57	TRUE	reported	8KoqAI
17	43960918	T	C	0.02419	0.003623	2.40E-11	0.224986	462346	NA	rs5630144	NA	UKB-b-57	TRUE	reported	8KoqAI
17	43960997	T	C	0.024224	0.003622	2.30E-11	0.225061	462346	NA	rs6205553	NA	UKB-b-57	TRUE	reported	8KoqAI
17	43961044	A	G	0.024229	0.003622	2.20E-11	0.225063	462346	NA	rs6205553	NA	UKB-b-57	TRUE	reported	8KoqAI
17	43961142	T	C	0.024227	0.003622	2.20E-11	0.225058	462346	NA	rs6205554	NA	UKB-b-57	TRUE	reported	8KoqAI
17	43961292	G	T	0.024219	0.003622	2.30E-11	0.225056	462346	NA	rs1777010	NA	UKB-b-57	TRUE	reported	8KoqAI
17	43962017	G	C	0.024226	0.003622	2.20E-11	0.225061	462346	NA	rs6205554	NA	UKB-b-57	TRUE	reported	8KoqAI
17	43962982	G	A	0.02416	0.003622	2.50E-11	0.225088	462346	NA	rs7742652	NA	UKB-b-57	TRUE	reported	8KoqAI
17	43963046	G	A	0.02422	0.003622	2.30E-11	0.225056	462346	NA	rs7712918	NA	UKB-b-57	TRUE	reported	8KoqAI
17	43963120	C	T	0.024315	0.003623	1.90E-11	0.224979	462346	NA	rs7486382	NA	UKB-b-57	TRUE	reported	8KoqAI
17	43963282	C	G	0.02422	0.003622	2.30E-11	0.225056	462346	NA	rs8025431	NA	UKB-b-57	TRUE	reported	8KoqAI
17	43963416	C	G	0.02422	0.003622	2.30E-11	0.225056	462346	NA	rs1124254	NA	UKB-b-57	TRUE	reported	8KoqAI
17	43963844	C	T	0.024221	0.003622	2.30E-11	0.225056	462346	NA	rs6205554	NA	UKB-b-57	TRUE	reported	8KoqAI
17	43963943	C	A	0.024221	0.003622	2.30E-11	0.225056	462346	NA	rs1777012	NA	UKB-b-57	TRUE	reported	8KoqAI
17	43964163	G	A	0.024219	0.003622	2.30E-11	0.225056	462346	NA	rs1777015	NA	UKB-b-57	TRUE	reported	8KoqAI
17	43964191	C	A	0.024196	0.003622	2.40E-11	0.225029	462346	NA	rs1777018	NA	UKB-b-57	TRUE	reported	8KoqAI
17	43964283	G	C	0.024221	0.003622	2.30E-11	0.225056	462346	NA	rs1769144	NA	UKB-b-57	TRUE	reported	8KoqAI
17	43964539	A	G	0.024048	0.003645	4.20E-11	0.224867	462346	NA	rs6205554	NA	UKB-b-57	TRUE	reported	8KoqAI
17	43964561	C	T	0.02445	0.003655	2.20E-11	0.222823	462346	NA	rs6205554	NA	UKB-b-57	TRUE	reported	8KoqAI
17	43964567	A	C	0.024526	0.003655	1.90E-11	0.22223	462346	NA	rs6205554	NA	UKB-b-57	TRUE	reported	8KoqAI
17	43964755	C	T	0.024169	0.003622	2.50E-11	0.225074	462346	NA	rs6205554	NA	UKB-b-57	TRUE	reported	8KoqAI
17	43964899	A	G	0.024222	0.003622	2.30E-11	0.225056	462346	NA	rs6205554	NA	UKB-b-57	TRUE	reported	8KoqAI
17	43965063	T	C	0.024222	0.003622	2.30E-11	0.225056	462346	NA	rs1133272	NA	UKB-b-57	TRUE	reported	8KoqAI
17	43965595	G	C	0.024223	0.003622	2.30E-11	0.225056	462346	NA	rs6205554	NA	UKB-b-57	TRUE	reported	8KoqAI
17	43965680	G	A	0.02403	0.003619	3.10E-11	0.225627	462346	NA	rs6205555	NA	UKB-b-57	TRUE	reported	8KoqAI
17	43965687	C	G	0.02403	0.003619	3.10E-11	0.225626	462346	NA	rs6205555	NA	UKB-b-57	TRUE	reported	8KoqAI
17	43965955	A	G	0.024221	0.003622	2.30E-11	0.225056	462346	NA	rs6205555	NA	UKB-b-57	TRUE	reported	8KoqAI
17	43966178	C	A	0.024224	0.003622	2.30E-11	0.225056	462346	NA	rs6205555	NA	UKB-b-57	TRUE	reported	8KoqAI
17	43966260	A	G	0.024224	0.003622	2.30E-11	0.225056	462346	NA	rs6205555	NA	UKB-b-57	TRUE	reported	8KoqAI
17	43966282	G	T	0.024224	0.003622	2.30E-11	0.225056	462346	NA	rs6205555	NA	UKB-b-57	TRUE	reported	8KoqAI
17	43966608	A	T	0.024224	0.003622	2.30E-11	0.225056	462346	NA	rs1119416	NA	UKB-b-57	TRUE	reported	8KoqAI
17	43966749	T	C	0.024224	0.003622	2.30E-11	0.225056	462346	NA	rs6205555	NA	UKB-b-57	TRUE	reported	8KoqAI
17	43966795	C	A	0.024224	0.003622	2.30E-11	0.225056	462346	NA	rs6205555	NA	UKB-b-57	TRUE	reported	8KoqAI
17	43966955	A	G	0.024224	0.003622	2.30E-11	0.225056	462346	NA	rs6205555	NA	UKB-b-57	TRUE	reported	8KoqAI
17	43967138	T	G	0.024224	0.003622	2.30E-11	0.225055	462346	NA	rs6205555	NA	UKB-b-57	TRUE	reported	8KoqAI
17	43967562	T	C	0.024175	0.003622	2.50E-11	0.224993	462346	NA	rs7919304	NA	UKB-b-57	TRUE	reported	8KoqAI
17	43968219	A	G	0.024226	0.003622	2.20E-11	0.225056	462346	NA	rs1769146	NA	UKB-b-57	TRUE	reported	8KoqAI
17	43968463	C	G	0.024226	0.003622	2.20E-11	0.225056	462346	NA	rs1769150	NA	UKB-b-57	TRUE	reported	8KoqAI
17	43968572	G	A	0.024237	0.003622	2.20E-11	0.225123	462346	NA	rs6205676	NA	UKB-b-57	TRUE	reported	8KoqAI
17	43968874	A	G	0.024226	0.003622	2.20E-11	0.225056	462346	NA	rs5594360	NA	UKB-b-57	TRUE	reported	8KoqAI
17	43968937	T	C	0.024241	0.003622	2.20E-11	0.225036	462346	NA	rs5589573	NA	UKB-b-57	TRUE	reported	8KoqAI
17	43968994	T	C	0.024338	0.003623	1.80E-11	0.224965	462346	NA	rs5596052	NA	UKB-b-57	TRUE	reported	8KoqAI
17	43969019	T	C	0.024338	0.003623	1.80E-11	0.224965	462346	NA	rs5587724	NA	UKB-b-57	TRUE	reported	8KoqAI
17	43969136	C	T	0.024238	0.003622	2.20E-11	0.225047	462346	NA	rs1777029	NA	UKB-b-57	TRUE	reported	8KoqAI
17	43969168	T	A	0.024223	0.003622	2.30E-11	0.225066	462346	NA	rs1769155	NA	UKB-b-57	TRUE	reported	8KoqAI
17	43969686	T	A	0.024319	0.003623	1.90E-11	0.224979	462346	NA	rs6205676	NA	UKB-b-57	TRUE	reported	8KoqAI
17	43969779	G	T	0.024319	0.003623	1.90E-11	0.224979	462346	NA	rs1777033	NA	UKB-b-57	TRUE	reported	8KoqAI
17	43969833	A	G	0.024319	0.003623	1.90E-11	0.224979	462346	NA	rs6205677	NA	UKB-b-57	TRUE	reported	8KoqAI
17	43969901	G	A	0.024319	0.003623	1.90E-11	0.224979	462346	NA	rs6205677	NA	UKB-b-57	TRUE	reported	8KoqAI
17	43969952	T	C	0.024303	0.003623	2.00E-11	0.224963	462346	NA	rs6205677	NA	UKB-b-57	TRUE	reported	8KoqAI
17	43969995	C	T	0.024303	0.003623	2.00E-11	0.224963	462346	NA	rs6205677	NA	UKB-b-57	TRUE	reported	8KoqAI
17	43970108	C	A	0.024328	0.003623	1.90E-11	0.225073	462346	NA	rs7872912	NA	UKB-b-57	TRUE	reported	8KoqAI
17	43970154	T	C	0.024303	0.003623	2.00E-11	0.224963	462346	NA	rs1777034	NA	UKB-b-57	TRUE	reported	8KoqAI
17	43970303	G	A	0.02432	0.003623	1.90E-11	0.224979	462346	NA	rs6205677	NA	UKB-b-57	TRUE	reported	8KoqAI
17	43970662	G	T	0.024225	0.003622	2.30E-11	0.225055	462346	NA	rs1769161	NA	UKB-b-57	TRUE	reported	8KoqAI
17	43971457	G	C	0.024223	0.003622	2.30E-11	0.225056	462346	NA	rs7659440	NA	UKB-b-57	TRUE	reported	8KoqAI
17	43971481	T	C	0.024223	0.003622	2.30E-11	0.225056	462346	NA	rs8023320	NA	UKB-b-57	TRUE	reported	8KoqAI
17	43971604	C	G	0.024226	0.003622	2.20E-11	0.225063	462346	NA	rs6205677	NA	UKB-b-57	TRUE	reported	8KoqAI
17	43971785	A	G	0.024226	0.003622	2.30E-11	0.225056	462346	NA	rs1157589	NA	UKB-b-57	TRUE	reported	8KoqAI
17	43971937	C	A	0.024226	0.003622	2.20E-11	0.225055	462346	NA	rs6205677	NA	UKB-b-57	TRUE	reported	8KoqAI

17	43972142	G	A	0.024226	0.003622	2.20E-11	0.225055	462346	NA	rs1157589	NA	UKB-b-57	TRUE	reported	8KoqAI
17	43972581	C	T	0.024318	0.003622	1.90E-11	0.225046	462346	NA	rs7445722	NA	UKB-b-57	TRUE	reported	8KoqAI
17	43973080	A	G	0.024233	0.003622	2.20E-11	0.225073	462346	NA	rs7454832	NA	UKB-b-57	TRUE	reported	8KoqAI
17	43973121	G	C	0.024226	0.003622	2.20E-11	0.225054	462346	NA	rs1119721	NA	UKB-b-57	TRUE	reported	8KoqAI
17	43973233	C	T	0.024225	0.003622	2.30E-11	0.225056	462346	NA	rs7632415	NA	UKB-b-57	TRUE	reported	8KoqAI
17	43973408	C	T	0.024225	0.003622	2.30E-11	0.225044	462346	NA	rs1133477	NA	UKB-b-57	TRUE	reported	8KoqAI
17	43973413	A	G	0.024225	0.003622	2.30E-11	0.225044	462346	NA	rs7872078	NA	UKB-b-57	TRUE	reported	8KoqAI
17	43973652	C	T	0.024224	0.003622	2.30E-11	0.225056	462346	NA	rs6205678	NA	UKB-b-57	TRUE	reported	8KoqAI
17	43973899	T	C	0.024223	0.003622	2.30E-11	0.225056	462346	NA	rs6205678	NA	UKB-b-57	TRUE	reported	8KoqAI
17	43974065	C	A	0.024224	0.003622	2.30E-11	0.225056	462346	NA	rs7449658	NA	UKB-b-57	TRUE	reported	8KoqAI
17	43974230	C	T	0.02422	0.003622	2.30E-11	0.225053	462346	NA	rs6205678	NA	UKB-b-57	TRUE	reported	8KoqAI
17	43974476	G	T	0.024219	0.003622	2.30E-11	0.225053	462346	NA	rs8034621	NA	UKB-b-57	TRUE	reported	8KoqAI
17	43974477	A	T	0.024219	0.003622	2.30E-11	0.225053	462346	NA	rs7663268	NA	UKB-b-57	TRUE	reported	8KoqAI
17	43974549	G	C	0.024219	0.003622	2.30E-11	0.225053	462346	NA	rs6205678	NA	UKB-b-57	TRUE	reported	8KoqAI
17	43974889	C	G	0.024217	0.003622	2.30E-11	0.225052	462346	NA	rs6205678	NA	UKB-b-57	TRUE	reported	8KoqAI
17	43975263	T	C	0.024208	0.003622	2.30E-11	0.225058	462346	NA	rs6205678	NA	UKB-b-57	TRUE	reported	8KoqAI
17	43975285	T	C	0.024208	0.003622	2.30E-11	0.225058	462346	NA	rs6205678	NA	UKB-b-57	TRUE	reported	8KoqAI
17	43975415	A	C	0.0242	0.003622	2.40E-11	0.225049	462346	NA	rs6205678	NA	UKB-b-57	TRUE	reported	8KoqAI
17	43975417	G	A	0.0242	0.003622	2.40E-11	0.225049	462346	NA	rs6205679	NA	UKB-b-57	TRUE	reported	8KoqAI
17	43976064	T	C	0.024216	0.003622	2.30E-11	0.22504	462346	NA	rs6205679	NA	UKB-b-57	TRUE	reported	8KoqAI
17	43976627	G	T	0.024233	0.003622	2.20E-11	0.22505	462346	NA	rs7756167	NA	UKB-b-57	TRUE	reported	8KoqAI
17	43976644	G	A	0.024236	0.003622	2.20E-11	0.225054	462346	NA	rs6205679	NA	UKB-b-57	TRUE	reported	8KoqAI
17	43977018	A	G	0.024233	0.003622	2.20E-11	0.225054	462346	NA	rs6205679	NA	UKB-b-57	TRUE	reported	8KoqAI
17	43977049	A	G	0.024233	0.003622	2.20E-11	0.225054	462346	NA	rs6205679	NA	UKB-b-57	TRUE	reported	8KoqAI
17	43977702	C	T	0.02424	0.003622	2.20E-11	0.225073	462346	NA	rs1864324	NA	UKB-b-57	TRUE	reported	8KoqAI
17	43977827	C	T	0.024228	0.003622	2.20E-11	0.225053	462346	NA	rs1864325	NA	UKB-b-57	TRUE	reported	8KoqAI
17	43978041	C	T	0.024228	0.003622	2.20E-11	0.225053	462346	NA	rs6205679	NA	UKB-b-57	TRUE	reported	8KoqAI
17	43978120	A	G	0.024229	0.003622	2.20E-11	0.225055	462346	NA	rs6205679	NA	UKB-b-57	TRUE	reported	8KoqAI
17	43978295	G	C	0.02422	0.003622	2.30E-11	0.225051	462346	NA	rs5569130	NA	UKB-b-57	TRUE	reported	8KoqAI
17	43978320	G	A	0.024257	0.003622	2.10E-11	0.22505	462346	NA	rs5582384	NA	UKB-b-57	TRUE	reported	8KoqAI
17	43978534	G	A	0.024212	0.003622	2.30E-11	0.225062	462346	NA	rs1560310	NA	UKB-b-57	TRUE	reported	8KoqAI
17	43978604	A	G	0.02423	0.003622	2.20E-11	0.225054	462346	NA	rs1467962	NA	UKB-b-57	TRUE	reported	8KoqAI
17	43978625	T	C	0.02423	0.003622	2.20E-11	0.225054	462346	NA	rs1560311	NA	UKB-b-57	TRUE	reported	8KoqAI
17	43978934	T	G	0.024228	0.003622	2.20E-11	0.225055	462346	NA	rs1467963	NA	UKB-b-57	TRUE	reported	8KoqAI
17	43979468	T	A	0.024228	0.003622	2.20E-11	0.225054	462346	NA	rs6205680	NA	UKB-b-57	TRUE	reported	8KoqAI
17	43979504	G	A	0.024233	0.003622	2.20E-11	0.22506	462346	NA	rs6205680	NA	UKB-b-57	TRUE	reported	8KoqAI
17	43979622	G	A	0.024227	0.003622	2.20E-11	0.225054	462346	NA	rs1138342	NA	UKB-b-57	TRUE	reported	8KoqAI
17	43979822	A	T	0.024226	0.003622	2.20E-11	0.225054	462346	NA	rs2099151	NA	UKB-b-57	TRUE	reported	8KoqAI
17	43979971	C	T	0.024227	0.003622	2.20E-11	0.225051	462346	NA	rs2082068	NA	UKB-b-57	TRUE	reported	8KoqAI
17	43980530	A	C	0.024219	0.003622	2.30E-11	0.225055	462346	NA	rs1984937	NA	UKB-b-57	TRUE	reported	8KoqAI
17	43980762	A	G	0.024223	0.003622	2.30E-11	0.225055	462346	NA	rs6205680	NA	UKB-b-57	TRUE	reported	8KoqAI
17	43981795	G	A	0.02421	0.003622	2.30E-11	0.22505	462346	NA	rs1135892	NA	UKB-b-57	TRUE	reported	8KoqAI
17	43981831	A	T	0.02421	0.003622	2.30E-11	0.22505	462346	NA	rs1130299	NA	UKB-b-57	TRUE	reported	8KoqAI
17	43981958	C	T	0.024212	0.003622	2.30E-11	0.225059	462346	NA	rs1122752	NA	UKB-b-57	TRUE	reported	8KoqAI
17	43982159	A	G	0.024253	0.003622	2.10E-11	0.225058	462346	NA	rs6205680	NA	UKB-b-57	TRUE	reported	8KoqAI
17	43982286	C	T	0.024216	0.003622	2.30E-11	0.225055	462346	NA	rs6205680	NA	UKB-b-57	TRUE	reported	8KoqAI
17	43982903	A	G	0.024209	0.003622	2.30E-11	0.225036	462346	NA	rs7708486	NA	UKB-b-57	TRUE	reported	8KoqAI
17	43982961	T	C	0.024212	0.003622	2.30E-11	0.225034	462346	NA	rs7457362	NA	UKB-b-57	TRUE	reported	8KoqAI
17	43983159	T	C	0.024227	0.003622	2.20E-11	0.225055	462346	NA	rs7561797	NA	UKB-b-57	TRUE	reported	8KoqAI
17	43983363	G	A	0.024212	0.003622	2.30E-11	0.225035	462346	NA	rs6205680	NA	UKB-b-57	TRUE	reported	8KoqAI
17	43983801	A	G	0.024231	0.003622	2.20E-11	0.225054	462346	NA	rs6205680	NA	UKB-b-57	TRUE	reported	8KoqAI
17	43984253	G	C	0.024234	0.003622	2.20E-11	0.225054	462346	NA	rs6205680	NA	UKB-b-57	TRUE	reported	8KoqAI
17	43984399	C	T	0.024236	0.003622	2.20E-11	0.225054	462346	NA	rs1467966	NA	UKB-b-57	TRUE	reported	8KoqAI
17	43984646	T	C	0.023892	0.003635	5.00E-11	0.223984	462346	NA	rs6205680	NA	UKB-b-57	TRUE	reported	8KoqAI
17	43984669	A	G	0.023901	0.003635	4.90E-11	0.223971	462346	NA	rs6205680	NA	UKB-b-57	TRUE	reported	8KoqAI
17	43984672	T	C	0.023924	0.003635	4.70E-11	0.223998	462346	NA	rs6205681	NA	UKB-b-57	TRUE	reported	8KoqAI
17	43984864	C	T	0.024239	0.003622	2.20E-11	0.225054	462346	NA	rs7949290	NA	UKB-b-57	TRUE	reported	8KoqAI
17	43985247	C	T	0.024241	0.003622	2.20E-11	0.225054	462346	NA	rs6205681	NA	UKB-b-57	TRUE	reported	8KoqAI
17	43985597	C	T	0.024242	0.003622	2.20E-11	0.225056	462346	NA	rs7450962	NA	UKB-b-57	TRUE	reported	8KoqAI
17	43985662	T	C	0.024243	0.003622	2.20E-11	0.225056	462346	NA	rs7929098	NA	UKB-b-57	TRUE	reported	8KoqAI
17	43986396	C	T	0.024184	0.003623	2.50E-11	0.224927	462346	NA	rs1467968	NA	UKB-b-57	TRUE	reported	8KoqAI
17	43986420	A	G	0.024197	0.003623	2.40E-11	0.22492	462346	NA	rs4371195	NA	UKB-b-57	TRUE	reported	8KoqAI
17	43986432	G	A	0.024142	0.003624	2.70E-11	0.2252	462346	NA	rs1215012	NA	UKB-b-57	TRUE	reported	8KoqAI
17	43986462	A	G	0.02426	0.003625	2.20E-11	0.224741	462346	NA	rs1215052	NA	UKB-b-57	TRUE	reported	8KoqAI
17	43986992	G	C	0.024248	0.003622	2.20E-11	0.225066	462346	NA	rs7502888	NA	UKB-b-57	TRUE	reported	8KoqAI
17	43987154	G	A	0.024139	0.003625	2.70E-11	0.224861	462346	NA	rs6205681	NA	UKB-b-57	TRUE	reported	8KoqAI
17	43987184	T	C	0.024163	0.003622	2.50E-11	0.225138	462346	NA	rs1114478	NA	UKB-b-57	TRUE	reported	8KoqAI
17	43987723	G	A	0.024529	0.003625	1.30E-11	0.224396	462346	NA	rs2843914	NA	UKB-b-57	TRUE	reported	8KoqAI
17	43987801	T	C	0.02422	0.003626	2.40E-11	0.224658	462346	NA	rs5618970	NA	UKB-b-57	TRUE	reported	8KoqAI
17	43988205	A	G	0.023048	0.003591	1.40E-10	0.232069	462346	NA	rs2435204	NA	UKB-b-57	TRUE	reported	8KoqAI
17	43988623	G	A	0.024258	0.003622	2.10E-11	0.225061	462346	NA	rs6205683	NA	UKB-b-57	TRUE	reported	8KoqAI
17	43988884	T	C	0.024167	0.003622	2.50E-11	0.225033	462346	NA	rs6205683	NA	UKB-b-57	TRUE	reported	8KoqAI
17	43989087	A	G	0.024262	0.003622	2.10E-11	0.225055	462346	NA	rs6205683	NA	UKB-b-57	TRUE	reported	8KoqAI
17	43989221	T	C	0.024262	0.003622	2.10E-11	0.225055	462346	NA	rs6205683	NA	UKB-b-57	TRUE	reported	8KoqAI
17	43989689	T	C	0.0243	0.003622	2.00E-11	0.225078	462346	NA	rs6205684	NA	UKB-b-57	TRUE	reported	8KoqAI
17	43989898	T	G	0.024314	0.003622	1.90E-11	0.225056	462346	NA	rs1121163	NA	UKB-b-57	TRUE	reported	8KoqAI
17	43989909	A	G	0.024315	0.003622	1.90E-11	0.225055	462346	NA	rs7820953	NA	UKB-b-57	TRUE	reported	8KoqAI
17	43990041	A	C	0.024225	0.003624	2.30E-11	0.224963	462346	NA	rs6205684	NA	UKB-b-57	TRUE	reported	8KoqAI
17	43990072	A	G	0.02427	0.003622	2.10E-11	0.225058	462346	NA	rs7986012	NA	UKB-b-57	TRUE	reported	8KoqAI
17	43990662	G	A	0.024317	0.003622	1.90E-11	0.225171	462346	NA	rs9944484	NA	UKB-b-57	TRUE	reported	8KoqAI
17	43990919	A	G	0.024262	0.003622	2.10E-11	0.22506	462346	NA	rs1756396	NA	UKB-b-57	TRUE	reported	8KoqAI
17	43991272	A	G	0.024256	0.003622	2.10E-11	0.225053	462346	NA	rs1756398	NA	UKB-b-57	TRUE	reported	8KoqAI
17	43991781	G	T	0.024342	0.003622	1.80E-11	0.225141	462346	NA	rs1756402	NA	UKB-b-57	TRUE	reported	8KoqAI
17	43991888	G	A	0.024259	0.003622	2.10E-11	0.225058	462346	NA	rs5568237	NA	UKB-b-57	TRUE	reported	8KoqAI
17	43992233	C	T	0.02416	0.003635	3.00E-11	0.224019	462346	NA	rs5578867	NA	UKB-b-57	TRUE	reported	8KoqAI
17	43992244	G	A	0.024253	0.00363	2.40E-11	0.224366	462346	NA	rs5604295	NA	UKB-b-57	TRUE	reported	8KoqAI
17	43992283	A	G	0.024251	0.00363	2.40E-11	0.224368	462346	NA	rs5581079	NA	UKB-b-57	TRUE	reported	8KoqAI
17	43992305	G	A	0.024284	0.003629	2.20E-11	0.224465	462346	NA	rs5593330	NA	UKB-b-57	TRUE	reported	8KoqAI
17	43992456	G	A	0.024278	0.003622	2.00E-11	0.225043	462346	NA	rs6205684	NA	UKB-b-57	TRUE	reported	8KoqAI
17	43992463	G	C	0.024278	0.003622	2.00E-11	0.225043	462346	NA	rs6205684	NA	UKB-b-57	TRUE	reported	8KoqAI
17	43992581	C	T	0.024178	0.003619	2.40E-11	0.225612	462346	NA	rs6205685	NA	UKB-b-57	TRUE	reported	8KoqAI
17	43992806	A	G	0.024269	0.003622	2.10E-11	0.225053	462346	NA	rs6205685	NA	UKB-b-57	TRUE	reported	8KoqAI
17	43994252	T	C	0.024184	0.003622	2.40E-11	0.225359	462346	NA	rs7792690	NA	UKB-b-57	TRUE	reported	8KoqAI
17	43994358	C	T	0.024366	0.003625	1.80E-11	0.224831	462346	NA	rs1115419	NA	UKB-b-57	TRUE	reported	8KoqAI
17	43994386	C	A	0.024276	0.003624	2.10E-11	0.224895	462346	NA	rs1764951	NA	UKB-b-57	TRUE	reported	8KoqAI
17	43994435	A	G	0.024306	0.003623	2.00E-11	0.225051	462346	NA	rs6205685	NA	UKB-b-57	TRUE	reported	8KoqAI
17	43994623	G	A	0.024214	0.003622	2.30E-11	0.225068	462346	NA	rs1126471	NA	UKB-b-57	TRUE	reported	8KoqAI
17	43994648	C	T	0.02406	0.003621	3.10E-11	0.225327	462346	NA	rs1764955	NA	UKB-b-57	TRUE	reported	8KoqAI
17	43994952	A	G	0.024223	0.003622	2.30E-11	0.225046	462346	NA	rs1764957	NA	UKB-b-57	TRUE	reported	8KoqAI
17	43995098	G	A	0.024295	0.003623	2.00E-11	0.224998	462346	NA	rs1756415	NA	UKB-b-57	TRUE	reported	8KoqAI
17	43995990	A	G	0.024194	0.003622	2.40E-11	0.225076	462346	NA	rs1764963	NA	UKB-b-57	TRUE	reported	8KoqAI
17	43996908	T	G	0.024291	0.003622	2.00E-11	0.225086	462346	NA	rs5576080	NA	UKB-b-57	TRUE	reported	8KoqAI
17	43996960	A	G	0.024291	0.003622	2.00E-11	0.225086	462346	NA	rs5630367	NA	UKB-b-57	TRUE	reported	8KoqAI
17	43997372	T	C	0.024504	0.003622	1.30E-11	0.225116	462346	NA	rs1764964	NA	UKB-b-57	TRUE	reported	8KoqAI
17	43997499	A	T	0.024254	0.003622	2.10E-11	0.225059	462346	NA	rs7504647	NA	UKB-b-57	TRUE	reported	8KoqAI
17	43997520	C	T	0.024262	0.003622	2.10E-11	0.225065	462346	NA	rs1756422	NA	UKB-b-57	TRUE	reported	8KoqAI
17	43997901	G	C	0.02435	0.003623	1.80E-11	0.224988	462346	NA	rs1764970	NA	UKB-b-57	TRUE	reported	8KoqAI

17	43998151	C	T	0.02435	0.003623	1.80E-11	0.224985	462346	NA	rs6205896	NA	UKB-b-57	TRUE	reported	8KoqAI
17	43998328	C	T	0.024351	0.003623	1.80E-11	0.224977	462346	NA	rs1467969	NA	UKB-b-57	TRUE	reported	8KoqAI
17	43998574	T	G	0.024342	0.003623	1.80E-11	0.224987	462346	NA	rs1467970	NA	UKB-b-57	TRUE	reported	8KoqAI
17	43998767	T	C	0.024338	0.003622	1.80E-11	0.225209	462346	NA	rs767058	NA	UKB-b-57	TRUE	reported	8KoqAI
17	43998822	A	G	0.024343	0.003623	1.80E-11	0.224985	462346	NA	rs767057	NA	UKB-b-57	TRUE	reported	8KoqAI
17	43998910	C	T	0.024248	0.003622	2.20E-11	0.225062	462346	NA	rs767056	NA	UKB-b-57	TRUE	reported	8KoqAI
17	43999100	G	C	0.024255	0.003622	2.10E-11	0.225062	462346	NA	rs767059	NA	UKB-b-57	TRUE	reported	8KoqAI
17	43999406	A	G	0.024237	0.003622	2.20E-11	0.225065	462346	NA	rs6205896	NA	UKB-b-57	TRUE	reported	8KoqAI
17	43999529	G	C	0.024246	0.003622	2.20E-11	0.225063	462346	NA	rs6205896	NA	UKB-b-57	TRUE	reported	8KoqAI
17	43999785	C	G	0.024246	0.003622	2.20E-11	0.225063	462346	NA	rs1967981	NA	UKB-b-57	TRUE	reported	8KoqAI
17	43999793	G	A	0.024246	0.003622	2.20E-11	0.225063	462346	NA	rs1476127	NA	UKB-b-57	TRUE	reported	8KoqAI
17	44000202	G	A	0.02419	0.003622	2.40E-11	0.2251	462346	NA	rs5592943	NA	UKB-b-57	TRUE	reported	8KoqAI
17	44000234	A	T	0.024245	0.003622	2.20E-11	0.225063	462346	NA	rs8079501	NA	UKB-b-57	TRUE	reported	8KoqAI
17	44000505	C	T	0.024245	0.003622	2.20E-11	0.225063	462346	NA	rs5600270	NA	UKB-b-57	TRUE	reported	8KoqAI
17	44000610	G	A	0.024245	0.003622	2.20E-11	0.225063	462346	NA	rs8078495	NA	UKB-b-57	TRUE	reported	8KoqAI
17	44000914	T	C	0.024245	0.003622	2.20E-11	0.225063	462346	NA	rs1764986	NA	UKB-b-57	TRUE	reported	8KoqAI
17	44001228	G	A	0.024235	0.003622	2.20E-11	0.225067	462346	NA	rs6205896	NA	UKB-b-57	TRUE	reported	8KoqAI
17	44001379	C	T	0.024245	0.003622	2.20E-11	0.225063	462346	NA	rs1756449	NA	UKB-b-57	TRUE	reported	8KoqAI
17	44001621	A	G	0.024245	0.003622	2.20E-11	0.225063	462346	NA	rs1764991	NA	UKB-b-57	TRUE	reported	8KoqAI
17	44001661	A	G	0.024245	0.003622	2.20E-11	0.225063	462346	NA	rs1764995	NA	UKB-b-57	TRUE	reported	8KoqAI
17	44001957	T	G	0.024252	0.003622	2.10E-11	0.225062	462346	NA	rs5568545	NA	UKB-b-57	TRUE	reported	8KoqAI
17	44002062	A	C	0.024253	0.003622	2.10E-11	0.225062	462346	NA	rs1756459	NA	UKB-b-57	TRUE	reported	8KoqAI
17	44002138	A	G	0.024261	0.003622	2.10E-11	0.22506	462346	NA	rs1756461	NA	UKB-b-57	TRUE	reported	8KoqAI
17	44002431	C	T	0.024245	0.003622	2.20E-11	0.225063	462346	NA	rs6205900	NA	UKB-b-57	TRUE	reported	8KoqAI
17	44002555	A	G	0.024245	0.003622	2.20E-11	0.225063	462346	NA	rs1765006	NA	UKB-b-57	TRUE	reported	8KoqAI
17	44002595	C	T	0.024245	0.003622	2.20E-11	0.225063	462346	NA	rs1756470	NA	UKB-b-57	TRUE	reported	8KoqAI
17	44002969	A	C	0.024252	0.003622	2.10E-11	0.225064	462346	NA	rs7609025	NA	UKB-b-57	TRUE	reported	8KoqAI
17	44003397	T	A	0.024251	0.003622	2.10E-11	0.225065	462346	NA	rs5578859	NA	UKB-b-57	TRUE	reported	8KoqAI
17	44003446	A	G	0.024251	0.003622	2.10E-11	0.225064	462346	NA	rs5619711	NA	UKB-b-57	TRUE	reported	8KoqAI
17	44003599	C	T	0.024251	0.003622	2.10E-11	0.225064	462346	NA	rs5624931	NA	UKB-b-57	TRUE	reported	8KoqAI
17	44003893	A	G	0.024251	0.003622	2.10E-11	0.225064	462346	NA	rs6205900	NA	UKB-b-57	TRUE	reported	8KoqAI
17	44004076	T	C	0.024247	0.003622	2.20E-11	0.22503	462346	NA	rs7650007	NA	UKB-b-57	TRUE	reported	8KoqAI
17	44004472	A	G	0.024182	0.003622	2.40E-11	0.225075	462346	NA	rs6205900	NA	UKB-b-57	TRUE	reported	8KoqAI
17	44005254	T	A	0.024167	0.003622	2.50E-11	0.225072	462346	NA	rs1137961	NA	UKB-b-57	TRUE	reported	8KoqAI
17	44005329	G	A	0.024175	0.003622	2.50E-11	0.225074	462346	NA	rs1124542	NA	UKB-b-57	TRUE	reported	8KoqAI
17	44005413	A	G	0.024189	0.003622	2.40E-11	0.225077	462346	NA	rs1756478	NA	UKB-b-57	TRUE	reported	8KoqAI
17	44005615	A	G	0.024227	0.003622	2.20E-11	0.225056	462346	NA	rs6205900	NA	UKB-b-57	TRUE	reported	8KoqAI
17	44006085	A	G	0.02419	0.003622	2.40E-11	0.225074	462346	NA	rs6205900	NA	UKB-b-57	TRUE	reported	8KoqAI
17	44006130	T	C	0.024189	0.003622	2.40E-11	0.225073	462346	NA	rs6205900	NA	UKB-b-57	TRUE	reported	8KoqAI
17	44006252	T	C	0.024186	0.003622	2.40E-11	0.225073	462346	NA	rs6206170	NA	UKB-b-57	TRUE	reported	8KoqAI
17	44006601	T	C	0.024186	0.003622	2.40E-11	0.225071	462346	NA	rs1756482	NA	UKB-b-57	TRUE	reported	8KoqAI
17	44006896	T	C	0.024258	0.003623	2.10E-11	0.224985	462346	NA	rs6206170	NA	UKB-b-57	TRUE	reported	8KoqAI
17	44006954	T	C	0.024949	0.003641	7.30E-12	0.223311	462346	NA	rs6206170	NA	UKB-b-57	TRUE	reported	8KoqAI
17	44006956	A	G	0.024969	0.003641	7.00E-12	0.22335	462346	NA	rs6206170	NA	UKB-b-57	TRUE	reported	8KoqAI
17	44007493	C	T	0.024187	0.003622	2.40E-11	0.225076	462346	NA	rs6206170	NA	UKB-b-57	TRUE	reported	8KoqAI
17	44009876	C	A	0.02424	0.003622	2.20E-11	0.225061	462346	NA	rs6206171	NA	UKB-b-57	TRUE	reported	8KoqAI
17	44009921	A	G	0.02424	0.003622	2.20E-11	0.225061	462346	NA	rs7501759	NA	UKB-b-57	TRUE	reported	8KoqAI
17	44010040	A	G	0.024241	0.003622	2.20E-11	0.225067	462346	NA	rs2316782	NA	UKB-b-57	TRUE	reported	8KoqAI
17	44010118	G	C	0.024234	0.003622	2.20E-11	0.225059	462346	NA	rs2316783	NA	UKB-b-57	TRUE	reported	8KoqAI
17	44010364	C	G	0.024221	0.003622	2.30E-11	0.225054	462346	NA	rs4255816	NA	UKB-b-57	TRUE	reported	8KoqAI
17	44010405	A	G	0.024225	0.003622	2.30E-11	0.225039	462346	NA	rs6206171	NA	UKB-b-57	TRUE	reported	8KoqAI
17	44010452	C	A	0.024225	0.003622	2.30E-11	0.225038	462346	NA	rs6206171	NA	UKB-b-57	TRUE	reported	8KoqAI
17	44010661	A	G	0.024323	0.003623	1.90E-11	0.224962	462346	NA	rs1139525	NA	UKB-b-57	TRUE	reported	8KoqAI
17	44010984	G	A	0.024247	0.003622	2.20E-11	0.225061	462346	NA	rs7807751	NA	UKB-b-57	TRUE	reported	8KoqAI
17	44011018	A	G	0.024343	0.003623	1.80E-11	0.224984	462346	NA	rs1756487	NA	UKB-b-57	TRUE	reported	8KoqAI
17	44011073	G	A	0.024241	0.003622	2.20E-11	0.225058	462346	NA	rs1765025	NA	UKB-b-57	TRUE	reported	8KoqAI
17	44011526	A	G	0.024248	0.003622	2.20E-11	0.22506	462346	NA	rs6206171	NA	UKB-b-57	TRUE	reported	8KoqAI
17	44011749	A	G	0.024248	0.003622	2.20E-11	0.22506	462346	NA	rs1756494	NA	UKB-b-57	TRUE	reported	8KoqAI
17	44011825	A	C	0.024235	0.003622	2.20E-11	0.225075	462346	NA	rs1756498	NA	UKB-b-57	TRUE	reported	8KoqAI
17	44012096	A	G	0.024247	0.003622	2.20E-11	0.22506	462346	NA	rs6206171	NA	UKB-b-57	TRUE	reported	8KoqAI
17	44012343	G	A	0.024247	0.003622	2.20E-11	0.22506	462346	NA	rs1765033	NA	UKB-b-57	TRUE	reported	8KoqAI
17	44012463	T	C	0.024312	0.003621	1.90E-11	0.225386	462346	NA	rs1756502	NA	UKB-b-57	TRUE	reported	8KoqAI
17	44012915	T	C	0.024313	0.003622	1.90E-11	0.2251	462346	NA	rs7792442	NA	UKB-b-57	TRUE	reported	8KoqAI
17	44012989	C	T	0.024284	0.003622	2.00E-11	0.225083	462346	NA	rs6206171	NA	UKB-b-57	TRUE	reported	8KoqAI
17	44013024	T	C	0.024284	0.003622	2.00E-11	0.225082	462346	NA	rs1765038	NA	UKB-b-57	TRUE	reported	8KoqAI
17	44013103	G	T	0.024321	0.003622	1.90E-11	0.225076	462346	NA	rs1765041	NA	UKB-b-57	TRUE	reported	8KoqAI
17	44013189	C	T	0.024246	0.003622	2.20E-11	0.225061	462346	NA	rs6206172	NA	UKB-b-57	TRUE	reported	8KoqAI
17	44013346	T	C	0.024232	0.003622	2.20E-11	0.225065	462346	NA	rs6206172	NA	UKB-b-57	TRUE	reported	8KoqAI
17	44013475	G	A	0.024432	0.003637	1.90E-11	0.223798	462346	NA	rs7985765	NA	UKB-b-57	TRUE	reported	8KoqAI
17	44013483	A	G	0.024435	0.003637	1.80E-11	0.223799	462346	NA	rs1137563	NA	UKB-b-57	TRUE	reported	8KoqAI
17	44013938	A	G	0.024247	0.003622	2.20E-11	0.22506	462346	NA	rs1215011	NA	UKB-b-57	TRUE	reported	8KoqAI
17	44014137	G	T	0.024255	0.003622	2.10E-11	0.225064	462346	NA	rs1215046	NA	UKB-b-57	TRUE	reported	8KoqAI
17	44014263	T	C	0.024255	0.003622	2.10E-11	0.225067	462346	NA	rs1215019	NA	UKB-b-57	TRUE	reported	8KoqAI
17	44014384	G	A	0.024245	0.003622	2.20E-11	0.225059	462346	NA	rs5587416	NA	UKB-b-57	TRUE	reported	8KoqAI
17	44015446	A	G	0.024502	0.003634	1.60E-11	0.223931	462346	NA	rs1215022	NA	UKB-b-57	TRUE	reported	8KoqAI
17	44015464	A	G	0.024503	0.003633	1.50E-11	0.224021	462346	NA	rs1215023	NA	UKB-b-57	TRUE	reported	8KoqAI
17	44015624	G	A	0.024243	0.003622	2.20E-11	0.225056	462346	NA	rs1215057	NA	UKB-b-57	TRUE	reported	8KoqAI
17	44015745	A	G	0.024261	0.003622	2.10E-11	0.225037	462346	NA	rs1215023	NA	UKB-b-57	TRUE	reported	8KoqAI
17	44015816	A	G	0.024249	0.003622	2.20E-11	0.225032	462346	NA	rs1215024	NA	UKB-b-57	TRUE	reported	8KoqAI
17	44015929	G	A	0.024242	0.003622	2.20E-11	0.225056	462346	NA	rs1215010	NA	UKB-b-57	TRUE	reported	8KoqAI
17	44016157	G	C	0.024244	0.003622	2.20E-11	0.225056	462346	NA	rs6206172	NA	UKB-b-57	TRUE	reported	8KoqAI
17	44016341	G	A	0.024229	0.003622	2.20E-11	0.225109	462346	NA	rs6206172	NA	UKB-b-57	TRUE	reported	8KoqAI
17	44016462	A	G	0.024244	0.003622	2.20E-11	0.225056	462346	NA	rs1135371	NA	UKB-b-57	TRUE	reported	8KoqAI
17	44017124	G	T	0.02419	0.003622	2.40E-11	0.225064	462346	NA	rs6206172	NA	UKB-b-57	TRUE	reported	8KoqAI
17	44017180	T	G	0.024242	0.003622	2.20E-11	0.225055	462346	NA	rs6206172	NA	UKB-b-57	TRUE	reported	8KoqAI
17	44017283	A	G	0.024226	0.003622	2.20E-11	0.225059	462346	NA	rs6206172	NA	UKB-b-57	TRUE	reported	8KoqAI
17	44017342	A	G	0.024257	0.003622	2.10E-11	0.225093	462346	NA	rs6206173	NA	UKB-b-57	TRUE	reported	8KoqAI
17	44017666	C	A	0.02424	0.003622	2.20E-11	0.225054	462346	NA	rs6206173	NA	UKB-b-57	TRUE	reported	8KoqAI
17	44017941	G	T	0.024233	0.003622	2.20E-11	0.225043	462346	NA	rs6206173	NA	UKB-b-57	TRUE	reported	8KoqAI
17	44018399	A	G	0.024084	0.003613	2.60E-11	0.228351	462346	NA	rs6206173	NA	UKB-b-57	TRUE	reported	8KoqAI
17	44018488	T	C	0.024662	0.003665	1.70E-11	0.222584	462346	NA	rs6206173	NA	UKB-b-57	TRUE	reported	8KoqAI
17	44018519	T	C	0.023434	0.003691	2.20E-10	0.224173	462346	NA	rs7210219	NA	UKB-b-57	TRUE	reported	8KoqAI
17	44019479	C	G	0.024223	0.003622	2.30E-11	0.225058	462346	NA	rs6206276	NA	UKB-b-57	TRUE	reported	8KoqAI
17	44019643	G	A	0.02434	0.003622	1.80E-11	0.225087	462346	NA	rs6206276	NA	UKB-b-57	TRUE	reported	8KoqAI
17	44019680	T	C	0.02434	0.003622	1.80E-11	0.225087	462346	NA	rs6206277	NA	UKB-b-57	TRUE	reported	8KoqAI
17	44020282	G	A	0.024281	0.003622	2.00E-11	0.225076	462346	NA	rs6206277	NA	UKB-b-57	TRUE	reported	8KoqAI
17	44020314	C	T	0.024228	0.003622	2.20E-11	0.225054	462346	NA	rs6206277	NA	UKB-b-57	TRUE	reported	8KoqAI
17	44020545	A	G	0.024224	0.003622	2.30E-11	0.225045	462346	NA	rs6206277	NA	UKB-b-57	TRUE	reported	8KoqAI
17	44021061	C	T	0.024238	0.003622	2.20E-11	0.225047	462346	NA	rs7911576	NA	UKB-b-57	TRUE	reported	8KoqAI
17	44021218	C	T	0.024265	0.003622	2.10E-11	0.225085	462346	NA	rs2841680	NA	UKB-b-57	TRUE	reported	8KoqAI
17	44021463	T	C	0.024218	0.003622	2.30E-11	0.225044	462346	NA	rs6206277	NA	UKB-b-57	TRUE	reported	8KoqAI
17	44021699	G	T	0.023793	0.003642	6.50E-11	0.223343	462346	NA	rs2316784	NA	UKB-b-57	TRUE	reported	8KoqAI
17	44021717	G	A	0.02379	0.003642	6.50E-11	0.223343	462346	NA	rs4327091	NA	UKB-b-57	TRUE	reported	8KoqAI
17	44021744	T	C	0.023831	0.003638	5.70E-11	0.223738	462346	NA	rs4479290	NA	UKB-b-57	TRUE	reported	8KoqAI
17	44022043	A	C	0.024223	0.003623	2.30E-11	0.225057	462346	NA	rs6206277	NA	UKB-b-57	TRUE	reported	8KoqAI
17	44022068	G	A	0.024216	0.003622	2.30E-11	0.225071	462346	NA	rs6206277	NA	UKB-b-57	TRUE	reported	8KoqAI
17	44022319	G	A	0.024282	0.003623	2.10E-11	0.225016	462346	NA	rs7583950	NA	UKB-b-57	TRUE	reported	8KoqAI

17	44022376	C	T	0.024371	0.003624	1.70E-11	0.224965	462346	NA	rs7568610	NA	UKB-b-57	TRUE	reported	8KoqAI
17	44022433	T	C	0.024413	0.003637	1.90E-11	0.223816	462346	NA	rs7976541	NA	UKB-b-57	TRUE	reported	8KoqAI
17	44022434	G	A	0.02442	0.003637	1.90E-11	0.223783	462346	NA	rs7883473	NA	UKB-b-57	TRUE	reported	8KoqAI
17	44022528	A	G	0.024368	0.003625	1.80E-11	0.224861	462346	NA	rs6206277	NA	UKB-b-57	TRUE	reported	8KoqAI
17	44022577	A	C	0.024282	0.00363	2.20E-11	0.224417	462346	NA	rs6206277	NA	UKB-b-57	TRUE	reported	8KoqAI
17	44022593	C	T	0.024287	0.00363	2.20E-11	0.224405	462346	NA	rs6206278	NA	UKB-b-57	TRUE	reported	8KoqAI
17	44022632	G	A	0.024336	0.003625	1.90E-11	0.224806	462346	NA	rs6206278	NA	UKB-b-57	TRUE	reported	8KoqAI
17	44023087	G	A	0.024217	0.003622	2.30E-11	0.22503	462346	NA	rs6206278	NA	UKB-b-57	TRUE	reported	8KoqAI
17	44023520	T	G	0.024242	0.003622	2.20E-11	0.225025	462346	NA	rs6206278	NA	UKB-b-57	TRUE	reported	8KoqAI
17	44024552	C	G	0.024193	0.003623	2.40E-11	0.224999	462346	NA	rs6206278	NA	UKB-b-57	TRUE	reported	8KoqAI
17	44024562	G	T	0.024195	0.003623	2.40E-11	0.224999	462346	NA	rs6206278	NA	UKB-b-57	TRUE	reported	8KoqAI
17	44024660	G	C	0.024193	0.003622	2.40E-11	0.22504	462346	NA	rs6206278	NA	UKB-b-57	TRUE	reported	8KoqAI
17	44024865	T	C	0.024219	0.003622	2.30E-11	0.225037	462346	NA	rs6206278	NA	UKB-b-57	TRUE	reported	8KoqAI
17	44024906	A	G	0.024182	0.003622	2.50E-11	0.22504	462346	NA	rs6206278	NA	UKB-b-57	TRUE	reported	8KoqAI
17	44025033	T	C	0.02427	0.00364	2.60E-11	0.2236	462346	NA	rs6206278	NA	UKB-b-57	TRUE	reported	8KoqAI
17	44025045	C	T	0.02427	0.00364	2.60E-11	0.2236	462346	NA	rs6206279	NA	UKB-b-57	TRUE	reported	8KoqAI
17	44025273	G	A	0.024191	0.003623	2.40E-11	0.225018	462346	NA	rs6206279	NA	UKB-b-57	TRUE	reported	8KoqAI
17	44025347	T	C	0.024201	0.003623	2.40E-11	0.22502	462346	NA	rs6206279	NA	UKB-b-57	TRUE	reported	8KoqAI
17	44025592	G	C	0.024205	0.003623	2.40E-11	0.225021	462346	NA	rs7802698	NA	UKB-b-57	TRUE	reported	8KoqAI
17	44025650	T	C	0.024204	0.003623	2.40E-11	0.225021	462346	NA	rs1133953	NA	UKB-b-57	TRUE	reported	8KoqAI
17	44025888	C	A	-0.02415	0.003593	1.80E-11	0.767122	462346	NA	rs242559	NA	UKB-b-57	TRUE	reported	8KoqAI
17	44026321	T	C	0.02419	0.003623	2.40E-11	0.225015	462346	NA	rs6206279	NA	UKB-b-57	TRUE	reported	8KoqAI
17	44026394	A	G	0.02419	0.003623	2.40E-11	0.225015	462346	NA	rs6206279	NA	UKB-b-57	TRUE	reported	8KoqAI
17	44026478	A	G	0.023955	0.003643	4.90E-11	0.223144	462346	NA	rs7855622	NA	UKB-b-57	TRUE	reported	8KoqAI
17	44026548	T	C	-0.02412	0.003623	2.80E-11	0.774833	462346	NA	rs242561	NA	UKB-b-57	TRUE	reported	8KoqAI
17	44027090	C	T	0.024192	0.003623	2.40E-11	0.225022	462346	NA	rs6206279	NA	UKB-b-57	TRUE	reported	8KoqAI
17	44027316	G	A	0.024039	0.003625	3.30E-11	0.22479	462346	NA	rs6206279	NA	UKB-b-57	TRUE	reported	8KoqAI
17	44027366	T	G	0.024199	0.003643	3.10E-11	0.223166	462346	NA	rs6206279	NA	UKB-b-57	TRUE	reported	8KoqAI
17	44027383	A	G	0.024184	0.003644	3.20E-11	0.223109	462346	NA	rs6206279	NA	UKB-b-57	TRUE	reported	8KoqAI
17	44027521	G	T	0.024188	0.003623	2.50E-11	0.225031	462346	NA	rs6206279	NA	UKB-b-57	TRUE	reported	8KoqAI
17	44027753	A	G	0.02419	0.003623	2.50E-11	0.225026	462346	NA	rs6206280	NA	UKB-b-57	TRUE	reported	8KoqAI
17	44028011	T	G	0.024188	0.003623	2.50E-11	0.225024	462346	NA	rs6206280	NA	UKB-b-57	TRUE	reported	8KoqAI
17	44028045	G	T	0.024178	0.003623	2.50E-11	0.225046	462346	NA	rs6206280	NA	UKB-b-57	TRUE	reported	8KoqAI
17	44028183	T	C	0.024219	0.003624	2.30E-11	0.225171	462346	NA	rs6206280	NA	UKB-b-57	TRUE	reported	8KoqAI
17	44028707	G	A	0.024204	0.003623	2.40E-11	0.225045	462346	NA	rs7490370	NA	UKB-b-57	TRUE	reported	8KoqAI
17	44028796	T	C	0.024284	0.003625	2.10E-11	0.224905	462346	NA	rs7701744	NA	UKB-b-57	TRUE	reported	8KoqAI
17	44029178	G	C	0.024304	0.003624	2.00E-11	0.224947	462346	NA	rs7683928	NA	UKB-b-57	TRUE	reported	8KoqAI
17	44030115	C	T	0.024323	0.003624	1.90E-11	0.224979	462346	NA	rs1765057	NA	UKB-b-57	TRUE	reported	8KoqAI
17	44030195	A	T	0.024334	0.003624	1.90E-11	0.224959	462346	NA	rs1765059	NA	UKB-b-57	TRUE	reported	8KoqAI
17	44030231	T	C	0.024331	0.003624	1.90E-11	0.224963	462346	NA	rs1765063	NA	UKB-b-57	TRUE	reported	8KoqAI
17	44030311	T	G	0.024337	0.003624	1.90E-11	0.224955	462346	NA	rs1765065	NA	UKB-b-57	TRUE	reported	8KoqAI
17	44030773	A	G	0.024279	0.003626	2.10E-11	0.224835	462346	NA	rs6206280	NA	UKB-b-57	TRUE	reported	8KoqAI
17	44031420	G	A	0.024158	0.003624	2.60E-11	0.225064	462346	NA	rs6206280	NA	UKB-b-57	TRUE	reported	8KoqAI
17	44031540	G	T	0.024165	0.003624	2.60E-11	0.225066	462346	NA	rs5597800	NA	UKB-b-57	TRUE	reported	8KoqAI
17	44031946	A	G	0.024203	0.003624	2.40E-11	0.225051	462346	NA	rs5594632	NA	UKB-b-57	TRUE	reported	8KoqAI
17	44032073	A	G	0.024177	0.003624	2.50E-11	0.225069	462346	NA	rs5631773	NA	UKB-b-57	TRUE	reported	8KoqAI
17	44032470	C	G	0.024205	0.003624	2.40E-11	0.225075	462346	NA	rs5582115	NA	UKB-b-57	TRUE	reported	8KoqAI
17	44032597	G	A	0.024193	0.003624	2.50E-11	0.225079	462346	NA	rs5608048	NA	UKB-b-57	TRUE	reported	8KoqAI
17	44032768	T	C	0.024135	0.003625	2.80E-11	0.224953	462346	NA	rs1757171	NA	UKB-b-57	TRUE	reported	8KoqAI
17	44032879	A	G	0.024015	0.003625	3.50E-11	0.224892	462346	NA	rs7855535	NA	UKB-b-57	TRUE	reported	8KoqAI
17	44032915	T	C	0.024147	0.003632	3.00E-11	0.224353	462346	NA	rs1757173	NA	UKB-b-57	TRUE	reported	8KoqAI
17	44032956	G	A	0.024166	0.003632	2.90E-11	0.224338	462346	NA	rs6206281	NA	UKB-b-57	TRUE	reported	8KoqAI
17	44033002	T	C	0.024105	0.003626	3.00E-11	0.224919	462346	NA	rs6206326	NA	UKB-b-57	TRUE	reported	8KoqAI
17	44033132	C	T	0.023149	0.00368	3.20E-10	0.22006	462346	NA	rs1135202	NA	UKB-b-57	TRUE	reported	8KoqAI
17	44033169	T	C	0.023133	0.003679	3.20E-10	0.220156	462346	NA	rs1132774	NA	UKB-b-57	TRUE	reported	8KoqAI
17	44033188	G	A	0.023169	0.00368	3.00E-10	0.2201	462346	NA	rs1129503	NA	UKB-b-57	TRUE	reported	8KoqAI
17	44033394	G	C	0.023669	0.003644	8.30E-11	0.223379	462346	NA	rs6206326	NA	UKB-b-57	TRUE	reported	8KoqAI
17	44033401	C	T	0.023746	0.003644	7.20E-11	0.223352	462346	NA	rs6206326	NA	UKB-b-57	TRUE	reported	8KoqAI
17	44033468	G	A	0.024135	0.003628	2.90E-11	0.224768	462346	NA	rs6206326	NA	UKB-b-57	TRUE	reported	8KoqAI
17	44033488	C	T	0.024086	0.003627	3.10E-11	0.22484	462346	NA	rs6206326	NA	UKB-b-57	TRUE	reported	8KoqAI
17	44033802	T	C	0.024122	0.003626	2.90E-11	0.224984	462346	NA	rs1757178	NA	UKB-b-57	TRUE	reported	8KoqAI
17	44034833	G	A	0.024138	0.003626	2.80E-11	0.224926	462346	NA	rs6206326	NA	UKB-b-57	TRUE	reported	8KoqAI
17	44034859	A	G	0.024128	0.003626	2.90E-11	0.224892	462346	NA	rs1757180	NA	UKB-b-57	TRUE	reported	8KoqAI
17	44035367	A	G	0.024117	0.003624	2.90E-11	0.225068	462346	NA	rs1765077	NA	UKB-b-57	TRUE	reported	8KoqAI
17	44035516	G	A	0.02416	0.003625	2.60E-11	0.225056	462346	NA	rs7755545	NA	UKB-b-57	TRUE	reported	8KoqAI
17	44035706	A	G	0.024138	0.003625	2.70E-11	0.225077	462346	NA	rs1757185	NA	UKB-b-57	TRUE	reported	8KoqAI
17	44036047	G	A	0.024183	0.003624	2.50E-11	0.225115	462346	NA	rs6206327	NA	UKB-b-57	TRUE	reported	8KoqAI
17	44036129	C	T	0.024118	0.003624	2.80E-11	0.225071	462346	NA	rs1129168	NA	UKB-b-57	TRUE	reported	8KoqAI
17	44036287	A	G	0.024112	0.003624	2.90E-11	0.225078	462346	NA	rs6206327	NA	UKB-b-57	TRUE	reported	8KoqAI
17	44036408	T	G	0.024256	0.003624	2.20E-11	0.225315	462346	NA	rs6206327	NA	UKB-b-57	TRUE	reported	8KoqAI
17	44036462	T	A	0.024128	0.003624	2.80E-11	0.22515	462346	NA	rs6206327	NA	UKB-b-57	TRUE	reported	8KoqAI
17	44036787	T	C	0.024093	0.003624	3.00E-11	0.225129	462346	NA	rs1765081	NA	UKB-b-57	TRUE	reported	8KoqAI
17	44036914	C	T	0.024071	0.003637	3.70E-11	0.22397	462346	NA	rs6206327	NA	UKB-b-57	TRUE	reported	8KoqAI
17	44036936	G	A	0.024097	0.003638	3.50E-11	0.223986	462346	NA	rs6206327	NA	UKB-b-57	TRUE	reported	8KoqAI
17	44037079	G	A	0.024087	0.003624	3.00E-11	0.225066	462346	NA	rs1137931	NA	UKB-b-57	TRUE	reported	8KoqAI
17	44037299	G	C	0.024069	0.003629	3.30E-11	0.224573	462346	NA	rs6206328	NA	UKB-b-57	TRUE	reported	8KoqAI
17	44037491	A	G	0.024038	0.003627	3.40E-11	0.224834	462346	NA	rs1765084	NA	UKB-b-57	TRUE	reported	8KoqAI
17	44038785	A	G	0.024205	0.003654	3.50E-11	0.220165	462346	NA	rs6206328	NA	UKB-b-57	TRUE	reported	8KoqAI
17	44039008	G	A	0.024181	0.003634	2.90E-11	0.224065	462346	NA	rs1765086	NA	UKB-b-57	TRUE	reported	8KoqAI
17	44039043	G	A	0.024234	0.003625	2.30E-11	0.224924	462346	NA	rs6206328	NA	UKB-b-57	TRUE	reported	8KoqAI
17	44039410	A	G	0.024247	0.003624	2.20E-11	0.225024	462346	NA	rs1115200	NA	UKB-b-57	TRUE	reported	8KoqAI
17	44039516	G	T	0.024268	0.003624	2.10E-11	0.224971	462346	NA	rs1765087	NA	UKB-b-57	TRUE	reported	8KoqAI
17	44039691	A	G	0.024255	0.003623	2.20E-11	0.225015	462346	NA	rs1765090	NA	UKB-b-57	TRUE	reported	8KoqAI
17	44040120	C	T	0.024278	0.003623	2.10E-11	0.225003	462346	NA	rs5578094	NA	UKB-b-57	TRUE	reported	8KoqAI
17	44040823	C	T	0.024308	0.003623	1.90E-11	0.225006	462346	NA	rs5571194	NA	UKB-b-57	TRUE	reported	8KoqAI
17	44041101	T	C	0.024307	0.00365	2.70E-11	0.222475	462346	NA	rs5570924	NA	UKB-b-57	TRUE	reported	8KoqAI
17	44041107	G	A	0.024307	0.00365	2.70E-11	0.222474	462346	NA	rs5628095	NA	UKB-b-57	TRUE	reported	8KoqAI
17	44041562	G	T	0.024779	0.003674	1.50E-11	0.220375	462346	NA	rs1120581	NA	UKB-b-57	TRUE	reported	8KoqAI
17	44041817	A	G	0.024313	0.003623	1.90E-11	0.225034	462346	NA	rs1122068	NA	UKB-b-57	TRUE	reported	8KoqAI
17	44042221	A	G	0.02435	0.003622	1.80E-11	0.225034	462346	NA	rs5616649	NA	UKB-b-57	TRUE	reported	8KoqAI
17	44042361	T	C	0.0247	0.003637	1.10E-11	0.223767	462346	NA	rs5622643	NA	UKB-b-57	TRUE	reported	8KoqAI
17	44042366	G	A	0.024717	0.003637	1.10E-11	0.223777	462346	NA	rs5590703	NA	UKB-b-57	TRUE	reported	8KoqAI
17	44042783	G	A	0.02428	0.003623	2.00E-11	0.224981	462346	NA	rs5629411	NA	UKB-b-57	TRUE	reported	8KoqAI
17	44042868	G	A	0.024283	0.003623	2.00E-11	0.224982	462346	NA	rs1124158	NA	UKB-b-57	TRUE	reported	8KoqAI
17	44043378	T	C	0.024386	0.003622	1.70E-11	0.225051	462346	NA	rs6206329	NA	UKB-b-57	TRUE	reported	8KoqAI
17	44043636	C	T	0.024303	0.003622	2.00E-11	0.224986	462346	NA	rs6206329	NA	UKB-b-57	TRUE	reported	8KoqAI
17	44043819	G	A	0.024303	0.003622	2.00E-11	0.224987	462346	NA	rs6206329	NA	UKB-b-57	TRUE	reported	8KoqAI
17	44044157	G	A	0.024296	0.003623	2.00E-11	0.224982	462346	NA	rs6206329	NA	UKB-b-57	TRUE	reported	8KoqAI
17	44044220	A	T	0.024299	0.003623	2.00E-11	0.224982	462346	NA	rs1765097	NA	UKB-b-57	TRUE	reported	8KoqAI
17	44044431	C	T	0.024298	0.003623	2.00E-11	0.224982	462346	NA	rs6206329	NA	UKB-b-57	TRUE	reported	8KoqAI
17	44044508	A	C	0.024303	0.003622	2.00E-11	0.224987	462346	NA	rs1765099	NA	UKB-b-57	TRUE	reported	8KoqAI
17	44044823	T	A	0.024302	0.003622	2.00E-11	0.224987	462346	NA	rs7514509	NA	UKB-b-57	TRUE	reported	8KoqAI
17	44044940	T	C	0.024302	0.003622	2.00E-11	0.224987	462346	NA	rs1138573	NA	UKB-b-57	TRUE	reported	8KoqAI
17	44044985	A	G	0.02434	0.003623	1.80E-11	0.224919	462346	NA	rs1757214	NA	UKB-b-57	TRUE	reported	8KoqAI
17	44045474	A	G	0.024299	0.003623	2.00E-11	0.224983	462346	NA	rs7751349	NA	UKB-b-57	TRUE	reported	8KoqAI
17	44045974	C	T	0.024302	0.003622	2.00E-11	0.224988	462346	NA	rs1757216	NA	UKB-b-57	TRUE	reported	8KoqAI
17	44046202	C	T	0.024298	0.003622	2.00E-11	0.224992	462346	NA	rs6206329	NA	UKB-b-57	TRUE	reported	8KoqAI

17	44047216	T	G	0.024296	0.003623	2.00E-11	0.224968	462346	NA	rs6264196	NA	UKB-b-57	TRUE	reported	8KoqAI
17	44047609	C	T	0.024301	0.003622	2.00E-11	0.224989	462346	NA	rs7437341	NA	UKB-b-57	TRUE	reported	8KoqAI
17	44047802	A	T	0.024301	0.003622	2.00E-11	0.224989	462346	NA	rs6206329	NA	UKB-b-57	TRUE	reported	8KoqAI
17	44048323	G	T	0.024452	0.003648	2.00E-11	0.222771	462346	NA	rs6206329	NA	UKB-b-57	TRUE	reported	8KoqAI
17	44048336	T	C	0.024439	0.003648	2.10E-11	0.222759	462346	NA	rs6206329	NA	UKB-b-57	TRUE	reported	8KoqAI
17	44048350	T	C	0.024438	0.003648	2.10E-11	0.222792	462346	NA	rs6206330	NA	UKB-b-57	TRUE	reported	8KoqAI
17	44049133	T	C	0.024296	0.003623	2.00E-11	0.224986	462346	NA	rs6206330	NA	UKB-b-57	TRUE	reported	8KoqAI
17	44049329	C	T	0.024285	0.003622	2.00E-11	0.224998	462346	NA	rs7524240	NA	UKB-b-57	TRUE	reported	8KoqAI
17	44049550	G	A	0.024144	0.003622	2.60E-11	0.225109	462346	NA	rs1765109	NA	UKB-b-57	TRUE	reported	8KoqAI
17	44049867	A	G	0.024272	0.003622	2.10E-11	0.225004	462346	NA	rs1757224	NA	UKB-b-57	TRUE	reported	8KoqAI
17	44049900	A	G	0.024305	0.003623	2.00E-11	0.224978	462346	NA	rs6206330	NA	UKB-b-57	TRUE	reported	8KoqAI
17	44050282	G	A	0.024277	0.003622	2.10E-11	0.225001	462346	NA	rs6206330	NA	UKB-b-57	TRUE	reported	8KoqAI
17	44050340	G	A	0.02427	0.003622	2.10E-11	0.225003	462346	NA	rs1765113	NA	UKB-b-57	TRUE	reported	8KoqAI
17	44050395	C	A	0.02427	0.003622	2.10E-11	0.225003	462346	NA	rs6206330	NA	UKB-b-57	TRUE	reported	8KoqAI
17	44050463	T	C	0.02427	0.003622	2.10E-11	0.225003	462346	NA	rs6206377	NA	UKB-b-57	TRUE	reported	8KoqAI
17	44050823	G	T	0.02427	0.003622	2.10E-11	0.225004	462346	NA	rs6206377	NA	UKB-b-57	TRUE	reported	8KoqAI
17	44050850	A	G	0.02427	0.003622	2.10E-11	0.225004	462346	NA	rs6206377	NA	UKB-b-57	TRUE	reported	8KoqAI
17	44051588	C	G	0.024298	0.003693	4.70E-11	0.218722	462346	NA	rs1171249	NA	UKB-b-57	TRUE	reported	8KoqAI
17	44051589	T	G	0.024326	0.003693	4.50E-11	0.218771	462346	NA	rs1180874	NA	UKB-b-57	TRUE	reported	8KoqAI
17	44051612	A	G	0.024886	0.00367	1.20E-11	0.221225	462346	NA	rs7787579	NA	UKB-b-57	TRUE	reported	8KoqAI
17	44051846	A	G	0.024266	0.003622	2.10E-11	0.225006	462346	NA	rs1800547	NA	UKB-b-57	TRUE	reported	8KoqAI
17	44051924	G	A	0.02427	0.003622	2.10E-11	0.225005	462346	NA	rs1765121	NA	UKB-b-57	TRUE	reported	8KoqAI
17	44052009	T	C	0.02427	0.003622	2.10E-11	0.225005	462346	NA	rs1757236	NA	UKB-b-57	TRUE	reported	8KoqAI
17	44052284	G	A	0.024269	0.003622	2.10E-11	0.225004	462346	NA	rs1765124	NA	UKB-b-57	TRUE	reported	8KoqAI
17	44052733	A	G	0.024199	0.003622	2.40E-11	0.225083	462346	NA	rs7810401	NA	UKB-b-57	TRUE	reported	8KoqAI
17	44053171	A	G	0.024269	0.003622	2.10E-11	0.225004	462346	NA	rs6206377	NA	UKB-b-57	TRUE	reported	8KoqAI
17	44053448	A	G	0.024269	0.003622	2.10E-11	0.225004	462346	NA	rs2217394	NA	UKB-b-57	TRUE	reported	8KoqAI
17	44053989	G	A	0.024269	0.003622	2.10E-11	0.225004	462346	NA	rs6206377	NA	UKB-b-57	TRUE	reported	8KoqAI
17	44054237	G	A	0.024268	0.003622	2.10E-11	0.225004	462346	NA	rs1765128	NA	UKB-b-57	TRUE	reported	8KoqAI
17	44054388	T	C	0.024267	0.003622	2.10E-11	0.225005	462346	NA	rs1757246	NA	UKB-b-57	TRUE	reported	8KoqAI
17	44054596	T	G	0.024264	0.003622	2.10E-11	0.225006	462346	NA	rs1757249	NA	UKB-b-57	TRUE	reported	8KoqAI
17	44054671	C	T	0.024262	0.003622	2.10E-11	0.225007	462346	NA	rs6206377	NA	UKB-b-57	TRUE	reported	8KoqAI
17	44055638	T	A	0.024268	0.003624	2.10E-11	0.224868	462346	NA	rs754513	NA	UKB-b-57	TRUE	reported	8KoqAI
17	44055647	A	T	0.024212	0.003623	2.30E-11	0.224945	462346	NA	rs754512	NA	UKB-b-57	TRUE	reported	8KoqAI
17	44056238	A	G	0.024249	0.003622	2.20E-11	0.225005	462346	NA	rs6206378	NA	UKB-b-57	TRUE	reported	8KoqAI
17	44056433	T	C	0.024269	0.003622	2.10E-11	0.225007	462346	NA	rs2163129	NA	UKB-b-57	TRUE	reported	8KoqAI
17	44056434	G	A	0.024284	0.003622	2.00E-11	0.225009	462346	NA	rs2163130	NA	UKB-b-57	TRUE	reported	8KoqAI
17	44056767	G	A	0.024264	0.003622	2.10E-11	0.225008	462346	NA	rs1981997	NA	UKB-b-57	TRUE	reported	8KoqAI
17	44056833	G	A	0.024264	0.003622	2.10E-11	0.225008	462346	NA	rs1981998	NA	UKB-b-57	TRUE	reported	8KoqAI
17	44057595	G	A	0.023008	0.003602	1.70E-10	0.229047	462346	NA	rs3785884	NA	UKB-b-57	TRUE	reported	8KoqAI
17	44058004	A	G	0.024259	0.003622	2.10E-11	0.224997	462346	NA	rs1757261	NA	UKB-b-57	TRUE	reported	8KoqAI
17	44058017	G	T	0.024245	0.003622	2.20E-11	0.224998	462346	NA	rs7752734	NA	UKB-b-57	TRUE	reported	8KoqAI
17	44058629	T	A	0.024265	0.003622	2.10E-11	0.225006	462346	NA	rs1757262	NA	UKB-b-57	TRUE	reported	8KoqAI
17	44058861	C	A	0.024279	0.003622	2.00E-11	0.225009	462346	NA	rs1765148	NA	UKB-b-57	TRUE	reported	8KoqAI
17	44059215	C	T	0.024279	0.003622	2.00E-11	0.225024	462346	NA	rs6206378	NA	UKB-b-57	TRUE	reported	8KoqAI
17	44059775	A	G	0.024269	0.003622	2.10E-11	0.22501	462346	NA	rs6206378	NA	UKB-b-57	TRUE	reported	8KoqAI
17	44060248	G	A	0.024263	0.003622	2.10E-11	0.225015	462346	NA	rs5623485	NA	UKB-b-57	TRUE	reported	8KoqAI
17	44060775	C	T	0.024284	0.003622	2.00E-11	0.224998	462346	NA	rs6375041	NA	UKB-b-57	TRUE	reported	8KoqAI
17	44061023	G	A	0.02414	0.003623	2.70E-11	0.225288	462346	NA	rs6206378	NA	UKB-b-57	TRUE	reported	8KoqAI
17	44061036	T	C	0.024269	0.003622	2.10E-11	0.22501	462346	NA	rs6206378	NA	UKB-b-57	TRUE	reported	8KoqAI
17	44061278	C	T	0.024177	0.003622	2.50E-11	0.224986	462346	NA	rs1765154	NA	UKB-b-57	TRUE	reported	8KoqAI
17	44061696	C	T	0.024266	0.003622	2.10E-11	0.225006	462346	NA	rs6206378	NA	UKB-b-57	TRUE	reported	8KoqAI
17	44061898	T	C	0.02432	0.003622	1.90E-11	0.225217	462346	NA	rs6206378	NA	UKB-b-57	TRUE	reported	8KoqAI
17	44062337	G	A	0.024269	0.003622	2.10E-11	0.22501	462346	NA	rs5636373	NA	UKB-b-57	TRUE	reported	8KoqAI
17	44062689	A	C	0.024244	0.003631	2.40E-11	0.224445	462346	NA	rs5624056	NA	UKB-b-57	TRUE	reported	8KoqAI
17	44062915	G	T	0.024269	0.003622	2.10E-11	0.224998	462346	NA	rs6206379	NA	UKB-b-57	TRUE	reported	8KoqAI
17	44062993	A	G	0.024241	0.003622	2.20E-11	0.225003	462346	NA	rs6206379	NA	UKB-b-57	TRUE	reported	8KoqAI
17	44063140	A	G	0.024245	0.003622	2.20E-11	0.225006	462346	NA	rs1529534	NA	UKB-b-57	TRUE	reported	8KoqAI
17	44063393	G	A	0.024267	0.003622	2.10E-11	0.22501	462346	NA	rs1757279	NA	UKB-b-57	TRUE	reported	8KoqAI
17	44063563	T	C	0.024267	0.003622	2.10E-11	0.22501	462346	NA	rs1757282	NA	UKB-b-57	TRUE	reported	8KoqAI
17	44063723	A	C	0.024303	0.003622	2.00E-11	0.225011	462346	NA	rs9901937	NA	UKB-b-57	TRUE	reported	8KoqAI
17	44063766	A	G	0.024267	0.003622	2.10E-11	0.22501	462346	NA	rs1757285	NA	UKB-b-57	TRUE	reported	8KoqAI
17	44063787	A	G	0.024267	0.003622	2.10E-11	0.22501	462346	NA	rs6206379	NA	UKB-b-57	TRUE	reported	8KoqAI
17	44064208	G	A	0.024256	0.003622	2.10E-11	0.225035	462346	NA	rs1757289	NA	UKB-b-57	TRUE	reported	8KoqAI
17	44064334	T	C	0.024267	0.003622	2.10E-11	0.22501	462346	NA	rs6206379	NA	UKB-b-57	TRUE	reported	8KoqAI
17	44064759	A	C	0.024267	0.003622	2.10E-11	0.22501	462346	NA	rs1529535	NA	UKB-b-57	TRUE	reported	8KoqAI
17	44065410	G	A	0.024285	0.003622	2.00E-11	0.225003	462346	NA	rs1044537	NA	UKB-b-57	TRUE	reported	8KoqAI
17	44065669	A	T	0.024282	0.003622	2.00E-11	0.225002	462346	NA	rs919461	NA	UKB-b-57	TRUE	reported	8KoqAI
17	44065740	C	T	0.024282	0.003622	2.00E-11	0.225003	462346	NA	rs919462	NA	UKB-b-57	TRUE	reported	8KoqAI
17	44065901	C	T	0.024282	0.003622	2.00E-11	0.225003	462346	NA	rs919464	NA	UKB-b-57	TRUE	reported	8KoqAI
17	44066088	T	A	0.024284	0.003622	2.00E-11	0.225003	462346	NA	rs6206379	NA	UKB-b-57	TRUE	reported	8KoqAI
17	44066172	A	G	0.024284	0.003622	2.00E-11	0.225003	462346	NA	rs1123855	NA	UKB-b-57	TRUE	reported	8KoqAI
17	44066634	C	G	0.024284	0.003622	2.00E-11	0.225003	462346	NA	rs1765170	NA	UKB-b-57	TRUE	reported	8KoqAI
17	44066995	C	T	0.024283	0.003622	2.00E-11	0.225005	462346	NA	rs1131340	NA	UKB-b-57	TRUE	reported	8KoqAI
17	44067046	G	A	0.024284	0.003622	2.00E-11	0.225003	462346	NA	rs7635706	NA	UKB-b-57	TRUE	reported	8KoqAI
17	44067400	T	C	0.023966	0.003623	3.70E-11	0.225488	462346	NA	rs1044533	NA	UKB-b-57	TRUE	reported	8KoqAI
17	44067508	A	G	0.024284	0.003622	2.00E-11	0.225003	462346	NA	rs7944716	NA	UKB-b-57	TRUE	reported	8KoqAI
17	44067546	T	C	0.024284	0.003622	2.00E-11	0.225003	462346	NA	rs1765175	NA	UKB-b-57	TRUE	reported	8KoqAI
17	44067682	G	A	0.02427	0.003622	2.10E-11	0.225007	462346	NA	rs1044533	NA	UKB-b-57	TRUE	reported	8KoqAI
17	44067831	T	C	0.024284	0.003622	2.00E-11	0.225003	462346	NA	rs1116526	NA	UKB-b-57	TRUE	reported	8KoqAI
17	44068112	C	G	0.024284	0.003622	2.00E-11	0.225003	462346	NA	rs7574306	NA	UKB-b-57	TRUE	reported	8KoqAI
17	44068238	C	T	0.024284	0.003622	2.00E-11	0.225003	462346	NA	rs6206379	NA	UKB-b-57	TRUE	reported	8KoqAI
17	44068289	G	A	0.024284	0.003622	2.00E-11	0.225003	462346	NA	rs6206379	NA	UKB-b-57	TRUE	reported	8KoqAI
17	44068294	G	C	0.024284	0.003622	2.00E-11	0.225003	462346	NA	rs6206379	NA	UKB-b-57	TRUE	reported	8KoqAI
17	44068480	T	C	0.024284	0.003622	2.00E-11	0.225003	462346	NA	rs7729064	NA	UKB-b-57	TRUE	reported	8KoqAI
17	44068481	G	A	0.024191	0.003623	2.50E-11	0.224912	462346	NA	rs7661856	NA	UKB-b-57	TRUE	reported	8KoqAI
17	44068543	G	A	0.024284	0.003622	2.00E-11	0.225003	462346	NA	rs6206379	NA	UKB-b-57	TRUE	reported	8KoqAI
17	44068924	G	A	0.024236	0.003622	2.20E-11	0.224996	462346	NA	rs1052551	NA	UKB-b-57	TRUE	reported	8KoqAI
17	44069180	C	G	0.024275	0.003622	2.10E-11	0.225005	462346	NA	rs6206379	NA	UKB-b-57	TRUE	reported	8KoqAI
17	44069198	C	T	0.024277	0.003622	2.10E-11	0.225005	462346	NA	rs6206380	NA	UKB-b-57	TRUE	reported	8KoqAI
17	44069687	A	G	0.024276	0.003622	2.10E-11	0.225005	462346	NA	rs1765185	NA	UKB-b-57	TRUE	reported	8KoqAI
17	44069768	A	G	0.024261	0.003622	2.10E-11	0.225002	462346	NA	rs7475927	NA	UKB-b-57	TRUE	reported	8KoqAI
17	44069840	G	A	0.024261	0.003622	2.10E-11	0.225002	462346	NA	rs1765188	NA	UKB-b-57	TRUE	reported	8KoqAI
17	44069970	C	G	0.024261	0.003622	2.10E-11	0.225002	462346	NA	rs7859919	NA	UKB-b-57	TRUE	reported	8KoqAI
17	44070111	G	C	0.024261	0.003622	2.10E-11	0.225002	462346	NA	rs6206384	NA	UKB-b-57	TRUE	reported	8KoqAI
17	44070545	G	A	0.024249	0.003622	2.20E-11	0.225	462346	NA	rs5566234	NA	UKB-b-57	TRUE	reported	8KoqAI
17	44070956	T	A	0.024252	0.003622	2.20E-11	0.225005	462346	NA	rs5591364	NA	UKB-b-57	TRUE	reported	8KoqAI
17	44071089	C	G	0.024252	0.003622	2.20E-11	0.225004	462346	NA	rs1757317	NA	UKB-b-57	TRUE	reported	8KoqAI
17	44071294	T	C	0.024252	0.003622	2.20E-11	0.225004	462346	NA	rs6206384	NA	UKB-b-57	TRUE	reported	8KoqAI
17	44071674	A	G	0.024253	0.003622	2.20E-11	0.225006	462346	NA	rs6206384	NA	UKB-b-57	TRUE	reported	8KoqAI
17	44072017	C	A	0.024253	0.003622	2.20E-11	0.225005	462346	NA	rs5573602	NA	UKB-b-57	TRUE	reported	8KoqAI
17	44072041	T	A	0.024252	0.003622	2.20E-11	0.225004	462346	NA	rs5588608	NA	UKB-b-57	TRUE	reported	8KoqAI
17	44072423	A	C	0.024245	0.003622	2.20E-11	0.225078	462346	NA	rs7594493	NA	UKB-b-57	TRUE	reported	8KoqAI
17	44072854	T	C	0.024267	0.003622	2.10E-11	0.225041	462346	NA	rs1757324	NA	UKB-b-57	TRUE	reported	8KoqAI
17	44072881	C	G	0.024313	0.003622	1.90E-11	0.225092	462346	NA	rs1757326	NA	UKB-b-57	TRUE	reported	8KoqAI
17	44072984	A	G	0.024294	0.003617	1.90E-11	0.22672	462346	NA	rs1125728	NA	UKB-b-57	TRUE	reported	8KoqAI
17	44073027	G	A	0.024418	0.003624	1.60E-11	0.224966	462346	NA	rs1765203	NA	UKB-b-57	TRUE	reported	8KoqAI

17	44073062	T	G	0.024208	0.003622	2.30E-11	0.225334	462346	NA	rs6206384	NA	UKB-b-57	TRUE	reported	8KoqAI
17	44073145	G	C	0.024372	0.003624	1.80E-11	0.224834	462346	NA	rs1765206	NA	UKB-b-57	TRUE	reported	8KoqAI
17	44073739	G	A	0.024253	0.003622	2.20E-11	0.225004	462346	NA	rs6206385	NA	UKB-b-57	TRUE	reported	8KoqAI
17	44073889	A	G	0.024299	0.003622	2.00E-11	0.225313	462346	NA	rs1052553	NA	UKB-b-57	TRUE	reported	8KoqAI
17	44073973	T	C	0.024252	0.003622	2.20E-11	0.225003	462346	NA	rs1765212	NA	UKB-b-57	TRUE	reported	8KoqAI
17	44074435	C	G	0.024285	0.003624	2.10E-11	0.22504	462346	NA	rs6206385	NA	UKB-b-57	TRUE	reported	8KoqAI
17	44074581	A	G	0.024149	0.003626	2.70E-11	0.224979	462346	NA	rs6206385	NA	UKB-b-57	TRUE	reported	8KoqAI
17	44074613	A	G	0.024081	0.003638	3.60E-11	0.223874	462346	NA	rs6206385	NA	UKB-b-57	TRUE	reported	8KoqAI
17	44074619	G	A	0.024108	0.003638	3.40E-11	0.223831	462346	NA	rs6206385	NA	UKB-b-57	TRUE	reported	8KoqAI
17	44075099	T	C	0.024397	0.003632	1.90E-11	0.224667	462346	NA	rs2004673	NA	UKB-b-57	TRUE	reported	8KoqAI
17	44075110	A	T	0.024355	0.003633	2.00E-11	0.224634	462346	NA	rs2004674	NA	UKB-b-57	TRUE	reported	8KoqAI
17	44075535	T	C	0.024246	0.003626	2.30E-11	0.225086	462346	NA	rs6206385	NA	UKB-b-57	TRUE	reported	8KoqAI
17	44075837	T	C	0.024218	0.003626	2.40E-11	0.225098	462346	NA	rs1078269	NA	UKB-b-57	TRUE	reported	8KoqAI
17	44075901	A	G	0.024228	0.003626	2.30E-11	0.225089	462346	NA	rs1078268	NA	UKB-b-57	TRUE	reported	8KoqAI
17	44076466	A	G	0.024245	0.003624	2.20E-11	0.224995	462346	NA	rs6206385	NA	UKB-b-57	TRUE	reported	8KoqAI
17	44076665	A	G	0.024263	0.003622	2.10E-11	0.224949	462346	NA	rs6206385	NA	UKB-b-57	TRUE	reported	8KoqAI
17	44077708	G	T	0.024246	0.003623	2.20E-11	0.22511	462346	NA	rs7995925	NA	UKB-b-57	TRUE	reported	8KoqAI
17	44077850	G	A	0.023679	0.003655	9.20E-11	0.222515	462346	NA	rs5630163	NA	UKB-b-57	TRUE	reported	8KoqAI
17	44077851	T	A	0.023691	0.003655	9.00E-11	0.222521	462346	NA	rs5610830	NA	UKB-b-57	TRUE	reported	8KoqAI
17	44078616	G	A	0.024251	0.003622	2.20E-11	0.224992	462346	NA	rs6206385	NA	UKB-b-57	TRUE	reported	8KoqAI
17	44078832	G	A	0.02426	0.003622	2.10E-11	0.224992	462346	NA	rs6206466	NA	UKB-b-57	TRUE	reported	8KoqAI
17	44078892	G	A	0.024255	0.003622	2.10E-11	0.224986	462346	NA	rs6206466	NA	UKB-b-57	TRUE	reported	8KoqAI
17	44079567	C	T	0.024242	0.003622	2.20E-11	0.225071	462346	NA	rs1757344	NA	UKB-b-57	TRUE	reported	8KoqAI
17	44079721	A	G	0.024304	0.003622	2.00E-11	0.224999	462346	NA	rs6206466	NA	UKB-b-57	TRUE	reported	8KoqAI
17	44080039	T	G	0.024245	0.003621	2.10E-11	0.225454	462346	NA	rs6206466	NA	UKB-b-57	TRUE	reported	8KoqAI
17	44080408	G	C	0.024343	0.003621	1.80E-11	0.225197	462346	NA	rs1138949	NA	UKB-b-57	TRUE	reported	8KoqAI
17	44081064	A	G	0.024294	0.003618	1.90E-11	0.22576	462346	NA	rs8070723	NA	UKB-b-57	TRUE	reported	8KoqAI
17	44081224	G	A	0.026405	0.004087	1.00E-10	0.188865	462346	NA	rs1757350	NA	UKB-b-57	TRUE	reported	8KoqAI
17	44081462	T	C	0.024339	0.003623	1.80E-11	0.225022	462346	NA	rs6206466	NA	UKB-b-57	TRUE	reported	8KoqAI
17	44081527	T	C	0.024356	0.003623	1.80E-11	0.225016	462346	NA	rs6206466	NA	UKB-b-57	TRUE	reported	8KoqAI
17	44082181	A	G	0.024369	0.003623	1.70E-11	0.225205	462346	NA	rs1113245	NA	UKB-b-57	TRUE	reported	8KoqAI
17	44082249	A	T	0.024399	0.003623	1.60E-11	0.225075	462346	NA	rs1132011	NA	UKB-b-57	TRUE	reported	8KoqAI
17	44082514	T	C	0.024293	0.003629	2.20E-11	0.224663	462346	NA	rs6206466	NA	UKB-b-57	TRUE	reported	8KoqAI
17	44082528	A	T	0.02387	0.003634	5.10E-11	0.225403	462346	NA	rs6206466	NA	UKB-b-57	TRUE	reported	8KoqAI
17	44082603	A	C	0.02436	0.003623	1.80E-11	0.225012	462346	NA	rs1757359	NA	UKB-b-57	TRUE	reported	8KoqAI
17	44082981	G	A	0.02434	0.003623	1.80E-11	0.225213	462346	NA	rs6206466	NA	UKB-b-57	TRUE	reported	8KoqAI
17	44083081	G	A	0.024447	0.003625	1.50E-11	0.224898	462346	NA	rs1757360	NA	UKB-b-57	TRUE	reported	8KoqAI
17	44083323	C	T	0.024209	0.003624	2.40E-11	0.2264	462346	NA	rs1765233	NA	UKB-b-57	TRUE	reported	8KoqAI
17	44083402	G	A	0.023595	0.003641	9.20E-11	0.226358	462346	NA	rs1991556	NA	UKB-b-57	TRUE	reported	8KoqAI
17	44084533	T	C	0.024328	0.003622	1.90E-11	0.224998	462346	NA	rs7482936	NA	UKB-b-57	TRUE	reported	8KoqAI
17	44084676	C	T	0.024327	0.003622	1.90E-11	0.224998	462346	NA	rs6206466	NA	UKB-b-57	TRUE	reported	8KoqAI
17	44084844	G	C	0.024348	0.003623	1.80E-11	0.22499	462346	NA	rs7896288	NA	UKB-b-57	TRUE	reported	8KoqAI
17	44085743	T	C	0.024325	0.003622	1.90E-11	0.224998	462346	NA	rs7813686	NA	UKB-b-57	TRUE	reported	8KoqAI
17	44085852	T	A	0.024324	0.003622	1.90E-11	0.224998	462346	NA	rs6206467	NA	UKB-b-57	TRUE	reported	8KoqAI
17	44086100	T	C	0.024312	0.003622	1.90E-11	0.225001	462346	NA	rs6206467	NA	UKB-b-57	TRUE	reported	8KoqAI
17	44086521	G	A	0.024316	0.003622	1.90E-11	0.224998	462346	NA	rs6206467	NA	UKB-b-57	TRUE	reported	8KoqAI
17	44088772	C	A	0.024304	0.003622	2.00E-11	0.224999	462346	NA	rs6206467	NA	UKB-b-57	TRUE	reported	8KoqAI
17	44088926	C	T	0.024301	0.003622	2.00E-11	0.225059	462346	NA	rs6206467	NA	UKB-b-57	TRUE	reported	8KoqAI
17	44088937	G	C	0.024273	0.003622	2.10E-11	0.224971	462346	NA	rs1765244	NA	UKB-b-57	TRUE	reported	8KoqAI
17	44089108	T	C	0.024302	0.003622	2.00E-11	0.224999	462346	NA	rs7672322	NA	UKB-b-57	TRUE	reported	8KoqAI
17	44089563	C	T	0.024335	0.003622	1.80E-11	0.225027	462346	NA	rs733966	NA	UKB-b-57	TRUE	reported	8KoqAI
17	44089690	T	C	0.024291	0.003622	2.00E-11	0.225002	462346	NA	rs733967	NA	UKB-b-57	TRUE	reported	8KoqAI
17	44089715	C	T	0.024299	0.003622	2.00E-11	0.224998	462346	NA	rs733968	NA	UKB-b-57	TRUE	reported	8KoqAI
17	44089727	G	A	0.02428	0.003622	2.00E-11	0.225005	462346	NA	rs733969	NA	UKB-b-57	TRUE	reported	8KoqAI
17	44089921	C	T	0.024299	0.003622	2.00E-11	0.224998	462346	NA	rs4283261	NA	UKB-b-57	TRUE	reported	8KoqAI
17	44090035	C	G	0.02428	0.003622	2.00E-11	0.224992	462346	NA	rs6206226	NA	UKB-b-57	TRUE	reported	8KoqAI
17	44090148	A	C	0.024299	0.003622	2.00E-11	0.224998	462346	NA	rs7566675	NA	UKB-b-57	TRUE	reported	8KoqAI
17	44090236	T	C	0.024299	0.003622	2.00E-11	0.224997	462346	NA	rs4306559	NA	UKB-b-57	TRUE	reported	8KoqAI
17	44090339	T	C	0.024299	0.003622	2.00E-11	0.224998	462346	NA	rs747152	NA	UKB-b-57	TRUE	reported	8KoqAI
17	44090455	T	C	0.024265	0.003622	2.10E-11	0.224974	462346	NA	rs1215025	NA	UKB-b-57	TRUE	reported	8KoqAI
17	44090536	G	A	0.024318	0.003622	1.90E-11	0.225012	462346	NA	rs1215050	NA	UKB-b-57	TRUE	reported	8KoqAI
17	44090646	A	G	0.024294	0.003622	2.00E-11	0.225002	462346	NA	rs1215017	NA	UKB-b-57	TRUE	reported	8KoqAI
17	44090685	G	T	0.024304	0.003622	2.00E-11	0.225005	462346	NA	rs1215051	NA	UKB-b-57	TRUE	reported	8KoqAI
17	44090704	A	G	0.024312	0.003622	1.90E-11	0.225	462346	NA	rs6206226	NA	UKB-b-57	TRUE	reported	8KoqAI
17	44090796	T	C	0.024316	0.003622	1.90E-11	0.224996	462346	NA	rs1757385	NA	UKB-b-57	TRUE	reported	8KoqAI
17	44090854	C	T	0.024313	0.003622	1.90E-11	0.224996	462346	NA	rs6206226	NA	UKB-b-57	TRUE	reported	8KoqAI
17	44090858	C	T	0.024314	0.003622	1.90E-11	0.224997	462346	NA	rs6206226	NA	UKB-b-57	TRUE	reported	8KoqAI
17	44091092	G	C	0.024315	0.003622	1.90E-11	0.225001	462346	NA	rs6206226	NA	UKB-b-57	TRUE	reported	8KoqAI
17	44091195	T	C	0.024309	0.003622	1.90E-11	0.225006	462346	NA	rs6206227	NA	UKB-b-57	TRUE	reported	8KoqAI
17	44091724	G	A	0.024305	0.003622	1.90E-11	0.225	462346	NA	rs7553419	NA	UKB-b-57	TRUE	reported	8KoqAI
17	44091886	C	T	0.024318	0.003599	1.40E-11	0.229686	462346	NA	rs9891103	NA	UKB-b-57	TRUE	reported	8KoqAI
17	44092497	T	C	0.024312	0.003622	1.90E-11	0.224977	462346	NA	rs1215046	NA	UKB-b-57	TRUE	reported	8KoqAI
17	44092801	G	A	0.02425	0.003621	2.10E-11	0.225204	462346	NA	rs1214999	NA	UKB-b-57	TRUE	reported	8KoqAI
17	44093319	G	A	0.024185	0.003622	2.40E-11	0.225008	462346	NA	rs6206227	NA	UKB-b-57	TRUE	reported	8KoqAI
17	44093496	A	G	0.024301	0.003622	2.00E-11	0.224997	462346	NA	rs6206227	NA	UKB-b-57	TRUE	reported	8KoqAI
17	44093627	A	G	0.0243	0.003622	2.00E-11	0.224998	462346	NA	rs6206227	NA	UKB-b-57	TRUE	reported	8KoqAI
17	44093860	A	G	0.0243	0.003622	2.00E-11	0.224998	462346	NA	rs6206227	NA	UKB-b-57	TRUE	reported	8KoqAI
17	44094112	A	G	0.024299	0.003622	2.00E-11	0.224998	462346	NA	rs6206228	NA	UKB-b-57	TRUE	reported	8KoqAI
17	44094374	A	G	0.024298	0.003622	2.00E-11	0.224997	462346	NA	rs6206228	NA	UKB-b-57	TRUE	reported	8KoqAI
17	44094471	G	A	0.024301	0.003622	2.00E-11	0.225	462346	NA	rs1765250	NA	UKB-b-57	TRUE	reported	8KoqAI
17	44094667	C	G	0.024297	0.003622	2.00E-11	0.224997	462346	NA	rs2316948	NA	UKB-b-57	TRUE	reported	8KoqAI
17	44095055	C	A	0.024296	0.003622	2.00E-11	0.224997	462346	NA	rs6206228	NA	UKB-b-57	TRUE	reported	8KoqAI
17	44095335	C	A	0.024296	0.003622	2.00E-11	0.224998	462346	NA	rs1119763	NA	UKB-b-57	TRUE	reported	8KoqAI
17	44095467	T	C	0.023524	0.003595	6.00E-11	0.230127	462346	NA	rs5887955	NA	UKB-b-57	TRUE	reported	8KoqAI
17	44095570	G	A	0.024295	0.003622	2.00E-11	0.224997	462346	NA	rs6206228	NA	UKB-b-57	TRUE	reported	8KoqAI
17	44095661	T	G	0.024294	0.003622	2.00E-11	0.224997	462346	NA	rs6206228	NA	UKB-b-57	TRUE	reported	8KoqAI
17	44096486	C	T	0.024144	0.003622	2.60E-11	0.225318	462346	NA	rs6206228	NA	UKB-b-57	TRUE	reported	8KoqAI
17	44096553	G	A	0.025509	0.00369	4.80E-12	0.219513	462346	NA	rs6206228	NA	UKB-b-57	TRUE	reported	8KoqAI
17	44097235	G	C	0.024316	0.003622	1.90E-11	0.224978	462346	NA	rs6206228	NA	UKB-b-57	TRUE	reported	8KoqAI
17	44098862	A	G	0.024258	0.003623	2.10E-11	0.224942	462346	NA	rs7859020	NA	UKB-b-57	TRUE	reported	8KoqAI
17	44098967	G	A	0.024486	0.003607	1.10E-11	0.229957	462346	NA	rs1765252	NA	UKB-b-57	TRUE	reported	8KoqAI
17	44099082	C	T	0.024321	0.003623	1.90E-11	0.224988	462346	NA	rs1757390	NA	UKB-b-57	TRUE	reported	8KoqAI
17	44099129	C	A	0.024278	0.003623	2.10E-11	0.224927	462346	NA	rs6206229	NA	UKB-b-57	TRUE	reported	8KoqAI
17	44099669	G	A	0.024304	0.003622	1.90E-11	0.224999	462346	NA	rs5572676	NA	UKB-b-57	TRUE	reported	8KoqAI
17	44099809	A	C	0.024304	0.003622	1.90E-11	0.224997	462346	NA	rs6206229	NA	UKB-b-57	TRUE	reported	8KoqAI
17	44099827	C	G	0.024304	0.003622	1.90E-11	0.224997	462346	NA	rs7774765	NA	UKB-b-57	TRUE	reported	8KoqAI
17	44100553	C	A	0.02426	0.003623	2.10E-11	0.224973	462346	NA	rs6206229	NA	UKB-b-57	TRUE	reported	8KoqAI
17	44100666	A	C	0.024285	0.003622	2.00E-11	0.224989	462346	NA	rs6206229	NA	UKB-b-57	TRUE	reported	8KoqAI
17	44100906	A	G	0.024285	0.003622	2.00E-11	0.224997	462346	NA	rs6206229	NA	UKB-b-57	TRUE	reported	8KoqAI
17	44100916	C	T	0.024286	0.003622	2.00E-11	0.224995	462346	NA	rs6206229	NA	UKB-b-57	TRUE	reported	8KoqAI
17	44101004	A	G	0.024285	0.003622	2.00E-11	0.224997	462346	NA	rs6206229	NA	UKB-b-57	TRUE	reported	8KoqAI
17	44101563	T	C	0.023945	0.003621	3.80E-11	0.225694	462346	NA	rs9468	NA	UKB-b-57	TRUE	reported	8KoqAI
17	44101871	A	G	0.023994	0.003622	3.50E-11	0.22567	462346	NA	rs8712	NA	UKB-b-57	TRUE	reported	8KoqAI
17	44102604	T	C	0.024319	0.003622	1.90E-11	0.224978	462346	NA	rs1052587	NA	UKB-b-57	TRUE	reported	8KoqAI
17	44102638	A	G	0.024327	0.003623	1.90E-11	0.224922	462346	NA	rs1052590	NA	UKB-b-57	TRUE	reported	8KoqAI
17	44102689	G	C	0.024319	0.003622	1.90E-11	0.224978	462346	NA	rs1052594	NA	UKB-b-57	TRUE	reported	8KoqAI
17	44102865	A	C	0.024303	0.003622	2.00E-11	0.224975	462346	NA	rs1757404	NA	UKB-b-57	TRUE	reported	8KoqAI

17	44102933	T	C	0.02433	0.003623	1.90E-11	0.224956	462346	NA	rs1694079	NA	UKB-b-57	TRUE	reported	8KoqAI
17	44103296	T	C	0.024296	0.003622	2.00E-11	0.224999	462346	NA	rs7687	NA	UKB-b-57	TRUE	reported	8KoqAI
17	44103616	C	T	0.024296	0.003622	2.00E-11	0.224999	462346	NA	rs1765274	NA	UKB-b-57	TRUE	reported	8KoqAI
17	44103825	T	C	0.024779	0.00366	1.30E-11	0.221779	462346	NA	rs7501048	NA	UKB-b-57	TRUE	reported	8KoqAI
17	44103826	G	A	0.024835	0.003661	1.20E-11	0.221712	462346	NA	rs1694080	NA	UKB-b-57	TRUE	reported	8KoqAI
17	44104343	A	C	0.024281	0.003622	2.00E-11	0.224997	462346	NA	rs2158257	NA	UKB-b-57	TRUE	reported	8KoqAI
17	44104509	T	C	0.024294	0.003622	2.00E-11	0.224999	462346	NA	rs1757422	NA	UKB-b-57	TRUE	reported	8KoqAI
17	44106219	C	T	0.024342	0.003623	1.80E-11	0.224939	462346	NA	rs7868197	NA	UKB-b-57	TRUE	reported	8KoqAI
17	44106220	A	G	0.024737	0.003622	8.50E-12	0.225687	462346	NA	rs7977257	NA	UKB-b-57	TRUE	reported	8KoqAI
17	44106806	G	A	0.024268	0.003622	2.10E-11	0.225038	462346	NA	rs7822968	NA	UKB-b-57	TRUE	reported	8KoqAI
17	44108100	C	T	0.024267	0.003622	2.10E-11	0.225007	462346	NA	rs7350928	NA	UKB-b-57	TRUE	reported	8KoqAI
17	44108202	A	G	0.024295	0.003622	2.00E-11	0.225002	462346	NA	rs1757436	NA	UKB-b-57	TRUE	reported	8KoqAI
17	44108355	G	A	0.024297	0.003622	2.00E-11	0.225004	462346	NA	rs1765296	NA	UKB-b-57	TRUE	reported	8KoqAI
17	44108906	A	G	0.02453	0.003621	1.20E-11	0.226174	462346	NA	rs3457953	NA	UKB-b-57	TRUE	reported	8KoqAI
17	44109188	C	G	0.024294	0.003622	2.00E-11	0.22499	462346	NA	rs1757442	NA	UKB-b-57	TRUE	reported	8KoqAI
17	44109248	G	A	0.024296	0.003622	2.00E-11	0.225	462346	NA	rs5588113	NA	UKB-b-57	TRUE	reported	8KoqAI
17	44109769	C	G	0.024309	0.003622	1.90E-11	0.22498	462346	NA	rs1076222	NA	UKB-b-57	TRUE	reported	8KoqAI
17	44110271	G	A	0.024472	0.003605	1.10E-11	0.228407	462346	NA	rs7350980	NA	UKB-b-57	TRUE	reported	8KoqAI
17	44110532	G	A	0.024293	0.003622	2.00E-11	0.225	462346	NA	rs3607672	NA	UKB-b-57	TRUE	reported	8KoqAI
17	44110541	G	A	0.024293	0.003622	2.00E-11	0.225	462346	NA	rs3583391	NA	UKB-b-57	TRUE	reported	8KoqAI
17	44110670	C	G	0.024481	0.003605	1.10E-11	0.228413	462346	NA	rs4597358	NA	UKB-b-57	TRUE	reported	8KoqAI
17	44110888	A	T	0.024291	0.003622	2.00E-11	0.225	462346	NA	rs7700986	NA	UKB-b-57	TRUE	reported	8KoqAI
17	44111109	A	G	0.024293	0.003622	2.00E-11	0.225	462346	NA	rs6206232	NA	UKB-b-57	TRUE	reported	8KoqAI
17	44111613	A	G	0.024293	0.003622	2.00E-11	0.225	462346	NA	rs1757460	NA	UKB-b-57	TRUE	reported	8KoqAI
17	44111827	C	A	0.024291	0.003622	2.00E-11	0.225	462346	NA	rs1765316	NA	UKB-b-57	TRUE	reported	8KoqAI
17	44112385	A	G	0.024291	0.003622	2.00E-11	0.225	462346	NA	rs6206232	NA	UKB-b-57	TRUE	reported	8KoqAI
17	44112441	G	A	0.024293	0.003622	2.00E-11	0.225	462346	NA	rs1765319	NA	UKB-b-57	TRUE	reported	8KoqAI
17	44112638	T	C	0.024293	0.003622	2.00E-11	0.225	462346	NA	rs1765321	NA	UKB-b-57	TRUE	reported	8KoqAI
17	44112743	C	A	0.024291	0.003622	2.00E-11	0.225	462346	NA	rs1765325	NA	UKB-b-57	TRUE	reported	8KoqAI
17	44113359	T	C	0.024293	0.003622	2.00E-11	0.225	462346	NA	rs4608377	NA	UKB-b-57	TRUE	reported	8KoqAI
17	44113491	T	C	0.024293	0.003622	2.00E-11	0.225	462346	NA	rs1218522	NA	UKB-b-57	TRUE	reported	8KoqAI
17	44113963	C	T	0.024293	0.003622	2.00E-11	0.225	462346	NA	rs1215043	NA	UKB-b-57	TRUE	reported	8KoqAI
17	44114218	T	C	0.02429	0.003622	2.00E-11	0.225001	462346	NA	rs1215016	NA	UKB-b-57	TRUE	reported	8KoqAI
17	44114432	A	C	0.024292	0.003622	2.00E-11	0.225	462346	NA	rs6206213	NA	UKB-b-57	TRUE	reported	8KoqAI
17	44115074	T	C	0.02429	0.003622	2.00E-11	0.225001	462346	NA	rs1757479	NA	UKB-b-57	TRUE	reported	8KoqAI
17	44115107	C	T	0.02429	0.003622	2.00E-11	0.225001	462346	NA	rs1757482	NA	UKB-b-57	TRUE	reported	8KoqAI
17	44115351	T	C	0.024292	0.003622	2.00E-11	0.225	462346	NA	rs1218524	NA	UKB-b-57	TRUE	reported	8KoqAI
17	44115440	T	C	0.024343	0.003604	1.40E-11	0.228499	462346	NA	rs8077487	NA	UKB-b-57	TRUE	reported	8KoqAI
17	44115569	C	A	0.024291	0.003622	2.00E-11	0.225	462346	NA	rs1107972	NA	UKB-b-57	TRUE	reported	8KoqAI
17	44115730	G	A	0.02429	0.003622	2.00E-11	0.225001	462346	NA	rs1215054	NA	UKB-b-57	TRUE	reported	8KoqAI
17	44115886	C	T	0.02429	0.003622	2.00E-11	0.225001	462346	NA	rs1215009	NA	UKB-b-57	TRUE	reported	8KoqAI
17	44116238	G	A	0.02429	0.003622	2.00E-11	0.225001	462346	NA	rs4143744	NA	UKB-b-57	TRUE	reported	8KoqAI
17	44116312	T	C	0.02429	0.003622	2.00E-11	0.225001	462346	NA	rs6206213	NA	UKB-b-57	TRUE	reported	8KoqAI
17	44116950	T	G	0.024363	0.003604	1.40E-11	0.228474	462346	NA	rs7221390	NA	UKB-b-57	TRUE	reported	8KoqAI
17	44117119	A	G	0.024288	0.003622	2.00E-11	0.225005	462346	NA	rs3404328	NA	UKB-b-57	TRUE	reported	8KoqAI
17	44117397	A	G	0.02429	0.003622	2.00E-11	0.225	462346	NA	rs1051489	NA	UKB-b-57	TRUE	reported	8KoqAI
17	44118848	G	A	0.024232	0.003632	2.50E-11	0.224668	462346	NA	rs6206213	NA	UKB-b-57	TRUE	reported	8KoqAI
17	44118999	A	G	0.024296	0.003622	2.00E-11	0.225026	462346	NA	rs4138255	NA	UKB-b-57	TRUE	reported	8KoqAI
17	44119320	C	T	0.024268	0.003622	2.10E-11	0.224992	462346	NA	rs5589371	NA	UKB-b-57	TRUE	reported	8KoqAI
17	44119636	A	C	0.024266	0.003622	2.10E-11	0.224992	462346	NA	rs1051489	NA	UKB-b-57	TRUE	reported	8KoqAI
17	44120442	A	G	0.024256	0.003622	2.10E-11	0.224996	462346	NA	rs4137424	NA	UKB-b-57	TRUE	reported	8KoqAI
17	44120608	A	G	0.024245	0.003622	2.20E-11	0.225001	462346	NA	rs7925234	NA	UKB-b-57	TRUE	reported	8KoqAI
17	44121469	C	T	0.024257	0.003622	2.10E-11	0.224996	462346	NA	rs4139944	NA	UKB-b-57	TRUE	reported	8KoqAI
17	44121917	C	T	0.024288	0.003622	2.00E-11	0.225025	462346	NA	rs7776886	NA	UKB-b-57	TRUE	reported	8KoqAI
17	44122484	C	T	0.024242	0.003622	2.20E-11	0.224993	462346	NA	rs6206366	NA	UKB-b-57	TRUE	reported	8KoqAI
17	44123248	T	C	0.024265	0.003622	2.10E-11	0.224991	462346	NA	rs6206367	NA	UKB-b-57	TRUE	reported	8KoqAI
17	44123685	G	A	0.024266	0.003622	2.10E-11	0.224991	462346	NA	rs7457169	NA	UKB-b-57	TRUE	reported	8KoqAI
17	44123717	C	T	0.024266	0.003622	2.10E-11	0.224991	462346	NA	rs7969803	NA	UKB-b-57	TRUE	reported	8KoqAI
17	44124535	T	C	0.023999	0.003622	3.50E-11	0.225435	462346	NA	rs7906501	NA	UKB-b-57	TRUE	reported	8KoqAI
17	44124837	A	G	0.0243	0.003622	2.00E-11	0.225021	462346	NA	rs6206367	NA	UKB-b-57	TRUE	reported	8KoqAI
17	44125066	C	T	0.024308	0.003622	1.90E-11	0.225021	462346	NA	rs1125784	NA	UKB-b-57	TRUE	reported	8KoqAI
17	44125484	T	G	0.024307	0.003622	1.90E-11	0.225026	462346	NA	rs6206367	NA	UKB-b-57	TRUE	reported	8KoqAI
17	44125704	C	T	0.024336	0.003623	1.80E-11	0.225019	462346	NA	rs1215033	NA	UKB-b-57	TRUE	reported	8KoqAI
17	44126218	G	A	0.024326	0.003622	1.90E-11	0.225027	462346	NA	rs1215057	NA	UKB-b-57	TRUE	reported	8KoqAI
17	44126365	C	T	0.024357	0.003604	1.40E-11	0.228398	462346	NA	rs7218319	NA	UKB-b-57	TRUE	reported	8KoqAI
17	44126650	C	T	0.024307	0.003622	1.90E-11	0.224987	462346	NA	rs1127460	NA	UKB-b-57	TRUE	reported	8KoqAI
17	44126673	A	G	0.024556	0.003645	1.60E-11	0.223164	462346	NA	rs1123333	NA	UKB-b-57	TRUE	reported	8KoqAI
17	44126691	G	A	0.024572	0.003623	1.20E-11	0.225198	462346	NA	rs1113279	NA	UKB-b-57	TRUE	reported	8KoqAI
17	44126765	C	A	0.025124	0.003826	5.20E-11	0.199574	462346	NA	rs1134346	NA	UKB-b-57	TRUE	reported	8KoqAI
17	44126977	G	C	0.024311	0.003622	1.90E-11	0.224996	462346	NA	rs7497773	NA	UKB-b-57	TRUE	reported	8KoqAI
17	44128125	A	C	0.024314	0.003622	1.90E-11	0.224995	462346	NA	rs1215044	NA	UKB-b-57	TRUE	reported	8KoqAI
17	44128407	C	A	0.024307	0.003622	1.90E-11	0.224997	462346	NA	rs1215006	NA	UKB-b-57	TRUE	reported	8KoqAI
17	44128876	T	A	0.024306	0.003622	1.90E-11	0.224999	462346	NA	rs1765383	NA	UKB-b-57	TRUE	reported	8KoqAI
17	44129253	T	C	0.024309	0.003622	1.90E-11	0.225001	462346	NA	rs1757542	NA	UKB-b-57	TRUE	reported	8KoqAI
17	44129800	G	A	0.024312	0.003622	1.90E-11	0.224996	462346	NA	rs6206367	NA	UKB-b-57	TRUE	reported	8KoqAI
17	44129885	C	T	0.024306	0.003622	1.90E-11	0.224998	462346	NA	rs6206367	NA	UKB-b-57	TRUE	reported	8KoqAI
17	44129949	C	G	0.024306	0.003622	1.90E-11	0.224998	462346	NA	rs6206367	NA	UKB-b-57	TRUE	reported	8KoqAI
17	44130587	T	G	0.024304	0.003622	2.00E-11	0.224999	462346	NA	rs7964986	NA	UKB-b-57	TRUE	reported	8KoqAI
17	44130878	A	G	0.024306	0.003622	1.90E-11	0.224998	462346	NA	rs1118539	NA	UKB-b-57	TRUE	reported	8KoqAI
17	44130924	A	T	0.024306	0.003622	1.90E-11	0.224998	462346	NA	rs1126747	NA	UKB-b-57	TRUE	reported	8KoqAI
17	44131022	T	C	0.024311	0.003622	1.90E-11	0.224997	462346	NA	rs1120743	NA	UKB-b-57	TRUE	reported	8KoqAI
17	44131559	C	A	0.024296	0.003622	2.00E-11	0.225001	462346	NA	rs6206368	NA	UKB-b-57	TRUE	reported	8KoqAI
17	44131592	A	G	0.0243	0.003622	2.00E-11	0.225006	462346	NA	rs6206368	NA	UKB-b-57	TRUE	reported	8KoqAI
17	44131622	G	A	0.0243	0.003622	2.00E-11	0.225006	462346	NA	rs6206368	NA	UKB-b-57	TRUE	reported	8KoqAI
17	44131717	G	A	0.024297	0.003622	2.00E-11	0.225005	462346	NA	rs6206368	NA	UKB-b-57	TRUE	reported	8KoqAI
17	44131798	C	T	0.02426	0.003622	2.10E-11	0.225021	462346	NA	rs7918086	NA	UKB-b-57	TRUE	reported	8KoqAI
17	44132269	T	C	0.024312	0.003622	1.90E-11	0.225001	462346	NA	rs6206368	NA	UKB-b-57	TRUE	reported	8KoqAI
17	44132379	A	G	0.024312	0.003622	1.90E-11	0.225001	462346	NA	rs6206368	NA	UKB-b-57	TRUE	reported	8KoqAI
17	44132592	A	G	0.024312	0.003622	1.90E-11	0.225001	462346	NA	rs6206368	NA	UKB-b-57	TRUE	reported	8KoqAI
17	44132887	C	T	0.024308	0.003622	1.90E-11	0.224998	462346	NA	rs6206076	NA	UKB-b-57	TRUE	reported	8KoqAI
17	44133031	T	A	0.024314	0.003622	1.90E-11	0.224996	462346	NA	rs1757543	NA	UKB-b-57	TRUE	reported	8KoqAI
17	44133070	A	T	0.024314	0.003622	1.90E-11	0.224996	462346	NA	rs1765388	NA	UKB-b-57	TRUE	reported	8KoqAI
17	44133142	G	A	0.02431	0.003622	1.90E-11	0.225022	462346	NA	rs1765390	NA	UKB-b-57	TRUE	reported	8KoqAI
17	44133162	A	C	0.024314	0.003622	1.90E-11	0.224996	462346	NA	rs6206076	NA	UKB-b-57	TRUE	reported	8KoqAI
17	44133396	G	A	0.024258	0.003623	2.10E-11	0.224942	462346	NA	rs6206076	NA	UKB-b-57	TRUE	reported	8KoqAI
17	44133403	T	C	0.024258	0.003623	2.10E-11	0.224941	462346	NA	rs6206076	NA	UKB-b-57	TRUE	reported	8KoqAI
17	44133534	C	T	0.024308	0.003622	1.90E-11	0.224998	462346	NA	rs1112591	NA	UKB-b-57	TRUE	reported	8KoqAI
17	44133633	A	G	0.024308	0.003622	1.90E-11	0.224998	462346	NA	rs6206076	NA	UKB-b-57	TRUE	reported	8KoqAI
17	44133648	A	C	0.024308	0.003622	1.90E-11	0.224998	462346	NA	rs6206076	NA	UKB-b-57	TRUE	reported	8KoqAI
17	44134095	A	G	0.024309	0.003622	1.90E-11	0.224998	462346	NA	rs1757550	NA	UKB-b-57	TRUE	reported	8KoqAI
17	44134391	G	T	0.024309	0.003622	1.90E-11	0.224998	462346	NA	rs876944	NA	UKB-b-57	TRUE	reported	8KoqAI
17	44134910	G	A	0.02431	0.003622	1.90E-11	0.224998	462346	NA	rs6206076	NA	UKB-b-57	TRUE	reported	8KoqAI
17	44134984	G	A	0.024282	0.003622	2.00E-11	0.225045	462346	NA	rs6206076	NA	UKB-b-57	TRUE	reported	8KoqAI
17	44135026	G	C	0.02431	0.003622	1.90E-11	0.224998	462346	NA	rs6206076	NA	UKB-b-57	TRUE	reported	8KoqAI
17	44135359	T	C	0.024311	0.003622	1.90E-11	0.224998	462346	NA	rs1107820	NA	UKB-b-57	TRUE	reported	8KoqAI
17	44135827	G	A	0.024319	0.003622	1.90E-11	0.224995	462346	NA	rs1757555	NA	UKB-b-57	TRUE	reported	8KoqAI
17	44135985	A	G	0.02432	0.003622	1.90E-11	0.224995	462346	NA	rs1765399	NA	UKB-b-57	TRUE	reported	8KoqAI

17	44136577	A	C	0.024409	0.003623	1.60E-11	0.224922	462346	NA	rs1113720	NA	UKB-b-57	TRUE	reported	8KoqAI
17	44136634	G	A	0.024315	0.003622	1.90E-11	0.224996	462346	NA	rs6206078	NA	UKB-b-57	TRUE	reported	8KoqAI
17	44136763	C	T	0.02431	0.003622	1.90E-11	0.224985	462346	NA	rs6206078	NA	UKB-b-57	TRUE	reported	8KoqAI
17	44136803	G	T	0.02431	0.003622	1.90E-11	0.224985	462346	NA	rs6206078	NA	UKB-b-57	TRUE	reported	8KoqAI
17	44137070	A	G	0.024319	0.003622	1.90E-11	0.224995	462346	NA	rs4138474	NA	UKB-b-57	TRUE	reported	8KoqAI
17	44137189	A	T	0.024311	0.003622	1.90E-11	0.224985	462346	NA	rs5571429	NA	UKB-b-57	TRUE	reported	8KoqAI
17	44137311	G	A	0.024381	0.003622	1.70E-11	0.225085	462346	NA	rs5569078	NA	UKB-b-57	TRUE	reported	8KoqAI
17	44137925	T	C	0.024316	0.003622	1.90E-11	0.224996	462346	NA	rs1765401	NA	UKB-b-57	TRUE	reported	8KoqAI
17	44138201	T	C	0.024379	0.003604	1.30E-11	0.228414	462346	NA	rs7220752	NA	UKB-b-57	TRUE	reported	8KoqAI
17	44139083	G	C	0.024318	0.003622	1.90E-11	0.224996	462346	NA	rs2316951	NA	UKB-b-57	TRUE	reported	8KoqAI
17	44139190	G	C	0.02432	0.003622	1.90E-11	0.224995	462346	NA	rs2316952	NA	UKB-b-57	TRUE	reported	8KoqAI
17	44139662	C	T	0.024318	0.003622	1.90E-11	0.224996	462346	NA	rs1757568	NA	UKB-b-57	TRUE	reported	8KoqAI
17	44139762	C	G	0.024318	0.003622	1.90E-11	0.224996	462346	NA	rs1124119	NA	UKB-b-57	TRUE	reported	8KoqAI
17	44140341	G	A	0.024319	0.003622	1.90E-11	0.224998	462346	NA	rs2316953	NA	UKB-b-57	TRUE	reported	8KoqAI
17	44141347	A	G	0.024321	0.003622	1.90E-11	0.224995	462346	NA	rs2838	NA	UKB-b-57	TRUE	reported	8KoqAI
17	44141732	G	A	0.02432	0.003622	1.90E-11	0.224993	462346	NA	rs1215012	NA	UKB-b-57	TRUE	reported	8KoqAI
17	44141955	T	A	0.02432	0.003622	1.90E-11	0.224993	462346	NA	rs6206079	NA	UKB-b-57	TRUE	reported	8KoqAI
17	44142121	G	A	0.024321	0.003622	1.90E-11	0.224992	462346	NA	rs7630718	NA	UKB-b-57	TRUE	reported	8KoqAI
17	44142427	A	G	0.024316	0.003622	1.90E-11	0.224994	462346	NA	rs6206079	NA	UKB-b-57	TRUE	reported	8KoqAI
17	44142566	C	T	0.024315	0.003622	1.90E-11	0.225003	462346	NA	rs6206079	NA	UKB-b-57	TRUE	reported	8KoqAI
17	44142663	C	T	0.02432	0.003622	1.90E-11	0.224995	462346	NA	rs6206079	NA	UKB-b-57	TRUE	reported	8KoqAI
17	44142879	C	T	0.02432	0.003622	1.90E-11	0.224995	462346	NA	rs1757577	NA	UKB-b-57	TRUE	reported	8KoqAI
17	44143029	G	T	0.024326	0.003622	1.90E-11	0.225002	462346	NA	rs6206079	NA	UKB-b-57	TRUE	reported	8KoqAI
17	44143344	G	A	0.024319	0.003622	1.90E-11	0.224995	462346	NA	rs6206079	NA	UKB-b-57	TRUE	reported	8KoqAI
17	44143484	G	A	0.024319	0.003622	1.90E-11	0.224995	462346	NA	rs1215034	NA	UKB-b-57	TRUE	reported	8KoqAI
17	44144152	T	C	0.024319	0.003622	1.90E-11	0.224995	462346	NA	rs6206079	NA	UKB-b-57	TRUE	reported	8KoqAI
17	44144251	G	T	0.024318	0.003622	1.90E-11	0.224995	462346	NA	rs6206079	NA	UKB-b-57	TRUE	reported	8KoqAI
17	44144345	A	G	0.024412	0.003623	1.60E-11	0.224921	462346	NA	rs1757582	NA	UKB-b-57	TRUE	reported	8KoqAI
17	44144387	C	A	0.024412	0.003623	1.60E-11	0.224921	462346	NA	rs1757585	NA	UKB-b-57	TRUE	reported	8KoqAI
17	44144634	G	A	0.024411	0.003623	1.60E-11	0.224921	462346	NA	rs1765973	NA	UKB-b-57	TRUE	reported	8KoqAI
17	44145373	T	C	0.024411	0.003623	1.60E-11	0.224922	462346	NA	rs7564122	NA	UKB-b-57	TRUE	reported	8KoqAI
17	44145592	A	G	0.024408	0.003623	1.60E-11	0.224922	462346	NA	rs6206080	NA	UKB-b-57	TRUE	reported	8KoqAI
17	44145860	A	G	0.02441	0.003623	1.60E-11	0.224922	462346	NA	rs6206080	NA	UKB-b-57	TRUE	reported	8KoqAI
17	44146165	C	G	0.02441	0.003623	1.60E-11	0.224922	462346	NA	rs1765974	NA	UKB-b-57	TRUE	reported	8KoqAI
17	44146336	A	C	0.024408	0.003623	1.60E-11	0.224922	462346	NA	rs6206080	NA	UKB-b-57	TRUE	reported	8KoqAI
17	44146357	T	C	0.024395	0.003623	1.70E-11	0.224928	462346	NA	rs7597508	NA	UKB-b-57	TRUE	reported	8KoqAI
17	44146451	T	C	0.024408	0.003623	1.60E-11	0.224922	462346	NA	rs7795091	NA	UKB-b-57	TRUE	reported	8KoqAI
17	44146567	T	C	0.024408	0.003623	1.60E-11	0.224922	462346	NA	rs7978234	NA	UKB-b-57	TRUE	reported	8KoqAI
17	44147411	G	A	0.024314	0.003622	1.90E-11	0.224996	462346	NA	rs5631886	NA	UKB-b-57	TRUE	reported	8KoqAI
17	44147511	G	C	0.024314	0.003622	1.90E-11	0.224996	462346	NA	rs5604177	NA	UKB-b-57	TRUE	reported	8KoqAI
17	44147574	G	A	0.024314	0.003622	1.90E-11	0.224996	462346	NA	rs5594702	NA	UKB-b-57	TRUE	reported	8KoqAI
17	44147599	G	C	0.024314	0.003622	1.90E-11	0.224996	462346	NA	rs5592919	NA	UKB-b-57	TRUE	reported	8KoqAI
17	44147721	C	T	0.024314	0.003622	1.90E-11	0.224996	462346	NA	rs5632340	NA	UKB-b-57	TRUE	reported	8KoqAI
17	44148267	C	T	0.024306	0.003622	1.90E-11	0.225001	462346	NA	rs5583151	NA	UKB-b-57	TRUE	reported	8KoqAI
17	44148613	G	A	0.024307	0.003622	1.90E-11	0.225	462346	NA	rs6206080	NA	UKB-b-57	TRUE	reported	8KoqAI
17	44148674	A	G	0.024307	0.003622	1.90E-11	0.225	462346	NA	rs6206081	NA	UKB-b-57	TRUE	reported	8KoqAI
17	44149297	A	C	0.024307	0.003622	1.90E-11	0.225004	462346	NA	rs1117253	NA	UKB-b-57	TRUE	reported	8KoqAI
17	44149448	C	G	0.024306	0.003622	1.90E-11	0.225001	462346	NA	rs1215062	NA	UKB-b-57	TRUE	reported	8KoqAI
17	44149537	C	G	0.024293	0.003622	2.00E-11	0.224994	462346	NA	rs1215062	NA	UKB-b-57	TRUE	reported	8KoqAI
17	44149581	C	T	0.024307	0.003622	1.90E-11	0.225	462346	NA	rs1215062	NA	UKB-b-57	TRUE	reported	8KoqAI
17	44149741	G	A	0.024307	0.003622	1.90E-11	0.225	462346	NA	rs1215061	NA	UKB-b-57	TRUE	reported	8KoqAI
17	44150042	T	C	0.024306	0.003622	1.90E-11	0.225001	462346	NA	rs7485077	NA	UKB-b-57	TRUE	reported	8KoqAI
17	44150152	A	T	0.024306	0.003622	1.90E-11	0.225001	462346	NA	rs974295	NA	UKB-b-57	TRUE	reported	8KoqAI
17	44150233	G	A	0.024306	0.003622	1.90E-11	0.225001	462346	NA	rs974293	NA	UKB-b-57	TRUE	reported	8KoqAI
17	44150464	T	C	0.024306	0.003622	1.90E-11	0.224999	462346	NA	rs974292	NA	UKB-b-57	TRUE	reported	8KoqAI
17	44150480	A	T	0.024306	0.003622	1.90E-11	0.225001	462346	NA	rs974291	NA	UKB-b-57	TRUE	reported	8KoqAI
17	44150920	T	C	0.024301	0.003622	2.00E-11	0.224995	462346	NA	rs1215055	NA	UKB-b-57	TRUE	reported	8KoqAI
17	44151156	T	C	0.024301	0.003622	2.00E-11	0.224995	462346	NA	rs1215055	NA	UKB-b-57	TRUE	reported	8KoqAI
17	44151546	T	G	0.024317	0.003604	1.50E-11	0.228437	462346	NA	rs9907738	NA	UKB-b-57	TRUE	reported	8KoqAI
17	44151667	A	G	0.0243	0.003622	2.00E-11	0.224995	462346	NA	rs6206081	NA	UKB-b-57	TRUE	reported	8KoqAI
17	44151697	A	T	0.024298	0.003622	2.00E-11	0.224996	462346	NA	rs6206083	NA	UKB-b-57	TRUE	reported	8KoqAI
17	44152390	C	T	0.024301	0.003622	2.00E-11	0.225	462346	NA	rs6206083	NA	UKB-b-57	TRUE	reported	8KoqAI
17	44152447	G	A	0.024301	0.003622	2.00E-11	0.225	462346	NA	rs6206083	NA	UKB-b-57	TRUE	reported	8KoqAI
17	44153144	A	G	0.0243	0.003622	2.00E-11	0.225	462346	NA	rs6206083	NA	UKB-b-57	TRUE	reported	8KoqAI
17	44153227	A	G	0.024301	0.003622	2.00E-11	0.225	462346	NA	rs6206083	NA	UKB-b-57	TRUE	reported	8KoqAI
17	44153258	A	G	0.024214	0.003622	2.30E-11	0.225061	462346	NA	rs6206084	NA	UKB-b-57	TRUE	reported	8KoqAI
17	44153386	A	T	0.0243	0.003622	2.00E-11	0.225	462346	NA	rs6206084	NA	UKB-b-57	TRUE	reported	8KoqAI
17	44153607	T	A	0.024299	0.003622	2.00E-11	0.225	462346	NA	rs6206084	NA	UKB-b-57	TRUE	reported	8KoqAI
17	44153626	C	T	0.024301	0.003622	2.00E-11	0.225	462346	NA	rs6206084	NA	UKB-b-57	TRUE	reported	8KoqAI
17	44154105	A	G	0.0243	0.003622	2.00E-11	0.225	462346	NA	rs1121977	NA	UKB-b-57	TRUE	reported	8KoqAI
17	44154393	A	G	0.0243	0.003622	2.00E-11	0.225	462346	NA	rs6206084	NA	UKB-b-57	TRUE	reported	8KoqAI
17	44154461	A	C	0.0243	0.003622	2.00E-11	0.225	462346	NA	rs6206084	NA	UKB-b-57	TRUE	reported	8KoqAI
17	44154607	T	C	0.0243	0.003622	2.00E-11	0.225	462346	NA	rs7448138	NA	UKB-b-57	TRUE	reported	8KoqAI
17	44154946	C	T	0.024299	0.003622	2.00E-11	0.225	462346	NA	rs6206084	NA	UKB-b-57	TRUE	reported	8KoqAI
17	44155157	C	T	0.024299	0.003622	2.00E-11	0.225	462346	NA	rs5610003	NA	UKB-b-57	TRUE	reported	8KoqAI
17	44155707	C	T	0.024285	0.003622	2.00E-11	0.225003	462346	NA	rs5597273	NA	UKB-b-57	TRUE	reported	8KoqAI
17	44155732	C	T	0.024318	0.003604	1.50E-11	0.22842	462346	NA	rs2066899	NA	UKB-b-57	TRUE	reported	8KoqAI
17	44157597	A	G	0.024361	0.003622	1.70E-11	0.225053	462346	NA	rs1765988	NA	UKB-b-57	TRUE	reported	8KoqAI
17	44157622	T	G	0.024292	0.003622	2.00E-11	0.225	462346	NA	rs7893813	NA	UKB-b-57	TRUE	reported	8KoqAI
17	44157982	A	T	0.024293	0.003622	2.00E-11	0.224998	462346	NA	rs6206085	NA	UKB-b-57	TRUE	reported	8KoqAI
17	44157992	T	C	0.024291	0.003622	2.00E-11	0.225009	462346	NA	rs6206085	NA	UKB-b-57	TRUE	reported	8KoqAI
17	44159102	C	T	0.02429	0.003622	2.00E-11	0.224993	462346	NA	rs1476554	NA	UKB-b-57	TRUE	reported	8KoqAI
17	44159555	C	A	0.024299	0.003622	2.00E-11	0.224998	462346	NA	rs7713843	NA	UKB-b-57	TRUE	reported	8KoqAI
17	44159631	T	G	0.024299	0.003622	2.00E-11	0.224998	462346	NA	rs1119137	NA	UKB-b-57	TRUE	reported	8KoqAI
17	44159672	A	G	0.024299	0.003622	2.00E-11	0.224998	462346	NA	rs1115190	NA	UKB-b-57	TRUE	reported	8KoqAI
17	44159695	C	T	0.024299	0.003622	2.00E-11	0.224998	462346	NA	rs7792583	NA	UKB-b-57	TRUE	reported	8KoqAI
17	44159725	C	T	0.024299	0.003622	2.00E-11	0.224998	462346	NA	rs1765995	NA	UKB-b-57	TRUE	reported	8KoqAI
17	44159849	T	C	0.024299	0.003622	2.00E-11	0.224998	462346	NA	rs1757616	NA	UKB-b-57	TRUE	reported	8KoqAI
17	44159988	G	A	0.024303	0.003622	2.00E-11	0.225	462346	NA	rs1757620	NA	UKB-b-57	TRUE	reported	8KoqAI
17	44160050	G	A	0.024273	0.003622	2.10E-11	0.224997	462346	NA	rs6206085	NA	UKB-b-57	TRUE	reported	8KoqAI
17	44160592	G	A	0.024314	0.003622	1.90E-11	0.224999	462346	NA	rs6206085	NA	UKB-b-57	TRUE	reported	8KoqAI
17	44160674	G	C	0.024315	0.003622	1.90E-11	0.224999	462346	NA	rs1766001	NA	UKB-b-57	TRUE	reported	8KoqAI
17	44161302	A	G	0.024323	0.003622	1.90E-11	0.224999	462346	NA	rs1137881	NA	UKB-b-57	TRUE	reported	8KoqAI
17	44161360	A	T	0.024323	0.003622	1.90E-11	0.224999	462346	NA	rs1123649	NA	UKB-b-57	TRUE	reported	8KoqAI
17	44161470	A	C	0.02546	0.003855	4.00E-11	0.205802	462346	NA	rs8002833	NA	UKB-b-57	TRUE	reported	8KoqAI
17	44161860	C	T	0.024332	0.003622	1.90E-11	0.224999	462346	NA	rs6206085	NA	UKB-b-57	TRUE	reported	8KoqAI
17	44161875	A	C	0.024332	0.003622	1.90E-11	0.224999	462346	NA	rs6206085	NA	UKB-b-57	TRUE	reported	8KoqAI
17	44161985	T	G	0.024332	0.003622	1.90E-11	0.224999	462346	NA	rs2316955	NA	UKB-b-57	TRUE	reported	8KoqAI
17	44162284	T	C	0.024333	0.003622	1.80E-11	0.224998	462346	NA	rs1766006	NA	UKB-b-57	TRUE	reported	8KoqAI
17	44162597	C	A	0.024345	0.003604	1.40E-11	0.228408	462346	NA	rs8080583	NA	UKB-b-57	TRUE	reported	8KoqAI
17	44163411	T	C	0.024332	0.003622	1.80E-11	0.224997	462346	NA	rs5586413	NA	UKB-b-57	TRUE	reported	8KoqAI
17	44164090	G	C	0.024359	0.003622	1.80E-11	0.225021	462346	NA	rs6206085	NA	UKB-b-57	TRUE	reported	8KoqAI
17	44164133	G	A	0.024354	0.003622	1.80E-11	0.225025	462346	NA	rs6206085	NA	UKB-b-57	TRUE	reported	8KoqAI
17	44164323	G	A	0.024359	0.003622	1.80E-11	0.225021	462346	NA	rs5569223	NA	UKB-b-57	TRUE	reported	8KoqAI
17	44165049	A	G	0.024478	0.003631	1.60E-11	0.22431	462346	NA	rs3912063	NA	UKB-b-57	TRUE	reported	8KoqAI
17	44165069	A	G	0.024476	0.003631	1.60E-11	0.224316	462346	NA	rs3912062	NA	UKB-b-57	TRUE	reported	8KoqAI
17	44165098	C	T	0.024359	0.003622	1.80E-11	0.225024	462346	NA	rs3912061	NA	UKB-b-57	TRUE	reported	8KoqAI

17	44165435	C	T	0.024404	0.003623	1.60E-11	0.224964	462346	NA	rs3912060	NA	UKB-b-57	TRUE	reported 8KoqAI
17	44165803	T	C	0.024403	0.003623	1.60E-11	0.224962	462346	NA	rs1766013	NA	UKB-b-57	TRUE	reported 8KoqAI
17	44165852	A	G	0.024356	0.003622	1.80E-11	0.225024	462346	NA	rs7434823	NA	UKB-b-57	TRUE	reported 8KoqAI
17	44166197	A	G	0.024404	0.003623	1.60E-11	0.224964	462346	NA	rs6206176	NA	UKB-b-57	TRUE	reported 8KoqAI
17	44166311	C	T	0.02436	0.003622	1.80E-11	0.225024	462346	NA	rs1766016	NA	UKB-b-57	TRUE	reported 8KoqAI
17	44166500	G	A	0.024541	0.003621	1.20E-11	0.225661	462346	NA	rs1766022	NA	UKB-b-57	TRUE	reported 8KoqAI
17	44166646	C	T	0.024454	0.003623	1.50E-11	0.224951	462346	NA	rs1766025	NA	UKB-b-57	TRUE	reported 8KoqAI
17	44167101	T	C	0.024368	0.003622	1.70E-11	0.225022	462346	NA	rs1766029	NA	UKB-b-57	TRUE	reported 8KoqAI
17	44167464	G	A	0.024369	0.003622	1.70E-11	0.225022	462346	NA	rs6206176	NA	UKB-b-57	TRUE	reported 8KoqAI
17	44167661	G	T	0.02437	0.003622	1.70E-11	0.225022	462346	NA	rs7992363	NA	UKB-b-57	TRUE	reported 8KoqAI
17	44167729	A	G	0.02437	0.003622	1.70E-11	0.225022	462346	NA	rs6206176	NA	UKB-b-57	TRUE	reported 8KoqAI
17	44168508	C	T	0.024376	0.003623	1.70E-11	0.22501	462346	NA	rs1113643	NA	UKB-b-57	TRUE	reported 8KoqAI
17	44168677	A	G	0.024389	0.003604	1.30E-11	0.228434	462346	NA	rs2097760	NA	UKB-b-57	TRUE	reported 8KoqAI
17	44169377	G	C	0.024372	0.003622	1.70E-11	0.225024	462346	NA	rs6206177	NA	UKB-b-57	TRUE	reported 8KoqAI
17	44169406	G	A	0.024399	0.003627	1.70E-11	0.224692	462346	NA	rs6206177	NA	UKB-b-57	TRUE	reported 8KoqAI
17	44169437	T	C	0.024429	0.003627	1.60E-11	0.224677	462346	NA	rs6206177	NA	UKB-b-57	TRUE	reported 8KoqAI
17	44169581	T	C	0.024369	0.003622	1.70E-11	0.225024	462346	NA	rs1119706	NA	UKB-b-57	TRUE	reported 8KoqAI
17	44169605	T	C	0.024364	0.003622	1.70E-11	0.225021	462346	NA	rs1766033	NA	UKB-b-57	TRUE	reported 8KoqAI
17	44169759	G	A	0.024367	0.003622	1.70E-11	0.225019	462346	NA	rs6206178	NA	UKB-b-57	TRUE	reported 8KoqAI
17	44169769	C	T	0.024367	0.003622	1.70E-11	0.225018	462346	NA	rs6206178	NA	UKB-b-57	TRUE	reported 8KoqAI
17	44170018	G	C	0.024258	0.003622	2.10E-11	0.225465	462346	NA	rs1757663	NA	UKB-b-57	TRUE	reported 8KoqAI
17	44170238	G	A	0.024261	0.003622	2.10E-11	0.225461	462346	NA	rs1125963	NA	UKB-b-57	TRUE	reported 8KoqAI
17	44170414	G	A	0.024261	0.003622	2.10E-11	0.22546	462346	NA	rs6206179	NA	UKB-b-57	TRUE	reported 8KoqAI
17	44170612	C	T	0.024375	0.003622	1.70E-11	0.22502	462346	NA	rs1766039	NA	UKB-b-57	TRUE	reported 8KoqAI
17	44171024	T	C	0.024253	0.003622	2.20E-11	0.225463	462346	NA	rs6206179	NA	UKB-b-57	TRUE	reported 8KoqAI
17	44171554	G	A	0.024501	0.003631	1.50E-11	0.224814	462346	NA	rs6206179	NA	UKB-b-57	TRUE	reported 8KoqAI
17	44171565	C	A	0.024479	0.003631	1.60E-11	0.224782	462346	NA	rs6206179	NA	UKB-b-57	TRUE	reported 8KoqAI
17	44172327	T	A	0.024226	0.003623	2.30E-11	0.225448	462346	NA	rs1757669	NA	UKB-b-57	TRUE	reported 8KoqAI
17	44172391	C	T	0.024246	0.003623	2.20E-11	0.225417	462346	NA	rs8002080	NA	UKB-b-57	TRUE	reported 8KoqAI
17	44172408	C	G	0.024204	0.003623	2.40E-11	0.225394	462346	NA	rs7704430	NA	UKB-b-57	TRUE	reported 8KoqAI
17	44172698	T	C	0.024259	0.003623	2.10E-11	0.225448	462346	NA	rs7934828	NA	UKB-b-57	TRUE	reported 8KoqAI
17	44172773	C	T	0.024258	0.003623	2.10E-11	0.225447	462346	NA	rs7541275	NA	UKB-b-57	TRUE	reported 8KoqAI
17	44173179	C	A	0.024258	0.003623	2.10E-11	0.225448	462346	NA	rs6206179	NA	UKB-b-57	TRUE	reported 8KoqAI
17	44173356	C	G	0.02426	0.003623	2.10E-11	0.225451	462346	NA	rs1757670	NA	UKB-b-57	TRUE	reported 8KoqAI
17	44173408	G	A	0.02426	0.003623	2.10E-11	0.225451	462346	NA	rs6206179	NA	UKB-b-57	TRUE	reported 8KoqAI
17	44174351	T	A	0.024264	0.003623	2.10E-11	0.225444	462346	NA	rs6206180	NA	UKB-b-57	TRUE	reported 8KoqAI
17	44174513	C	A	0.024262	0.003623	2.10E-11	0.225451	462346	NA	rs6206180	NA	UKB-b-57	TRUE	reported 8KoqAI
17	44176092	G	A	0.024209	0.003623	2.40E-11	0.225405	462346	NA	rs5590034	NA	UKB-b-57	TRUE	reported 8KoqAI
17	44176170	C	G	0.024226	0.003623	2.30E-11	0.225451	462346	NA	rs5624067	NA	UKB-b-57	TRUE	reported 8KoqAI
17	44177337	C	T	0.024226	0.003623	2.30E-11	0.225451	462346	NA	rs6206180	NA	UKB-b-57	TRUE	reported 8KoqAI
17	44177669	C	T	0.024238	0.003623	2.20E-11	0.225454	462346	NA	rs7760420	NA	UKB-b-57	TRUE	reported 8KoqAI
17	44177755	C	G	0.02421	0.003623	2.40E-11	0.225407	462346	NA	rs8012091	NA	UKB-b-57	TRUE	reported 8KoqAI
17	44177993	C	A	0.024225	0.003623	2.30E-11	0.225452	462346	NA	rs1766046	NA	UKB-b-57	TRUE	reported 8KoqAI
17	44178272	C	T	0.024226	0.003623	2.30E-11	0.225451	462346	NA	rs1757677	NA	UKB-b-57	TRUE	reported 8KoqAI
17	44178337	T	C	0.024225	0.003623	2.30E-11	0.225452	462346	NA	rs1766048	NA	UKB-b-57	TRUE	reported 8KoqAI
17	44178839	T	C	0.024225	0.003623	2.30E-11	0.225453	462346	NA	rs6206180	NA	UKB-b-57	TRUE	reported 8KoqAI
17	44178884	G	A	0.024222	0.003623	2.30E-11	0.225479	462346	NA	rs6206181	NA	UKB-b-57	TRUE	reported 8KoqAI
17	44179749	G	A	0.02423	0.003623	2.30E-11	0.225454	462346	NA	rs1121028	NA	UKB-b-57	TRUE	reported 8KoqAI
17	44179969	G	A	0.024222	0.003623	2.30E-11	0.225454	462346	NA	rs1128934	NA	UKB-b-57	TRUE	reported 8KoqAI
17	44179992	A	G	0.024219	0.003623	2.30E-11	0.225456	462346	NA	rs1757684	NA	UKB-b-57	TRUE	reported 8KoqAI
17	44180173	T	G	0.024223	0.003623	2.30E-11	0.22545	462346	NA	rs6206181	NA	UKB-b-57	TRUE	reported 8KoqAI
17	44180442	A	G	0.02423	0.003623	2.30E-11	0.225448	462346	NA	rs1757687	NA	UKB-b-57	TRUE	reported 8KoqAI
17	44180954	A	G	0.024222	0.003623	2.30E-11	0.22545	462346	NA	rs1766059	NA	UKB-b-57	TRUE	reported 8KoqAI
17	44180997	C	T	0.024223	0.003623	2.30E-11	0.22545	462346	NA	rs6206181	NA	UKB-b-57	TRUE	reported 8KoqAI
17	44181118	G	A	0.024248	0.003625	2.20E-11	0.225271	462346	NA	rs6206181	NA	UKB-b-57	TRUE	reported 8KoqAI
17	44181454	C	T	0.02425	0.003625	2.20E-11	0.225272	462346	NA	rs1119523	NA	UKB-b-57	TRUE	reported 8KoqAI
17	44181508	T	G	0.024244	0.003625	2.30E-11	0.225271	462346	NA	rs6206181	NA	UKB-b-57	TRUE	reported 8KoqAI
17	44181681	A	G	0.024229	0.003623	2.30E-11	0.225449	462346	NA	rs6206181	NA	UKB-b-57	TRUE	reported 8KoqAI
17	44181933	T	C	0.024229	0.003623	2.30E-11	0.225449	462346	NA	rs1757695	NA	UKB-b-57	TRUE	reported 8KoqAI
17	44182181	T	C	0.024228	0.003623	2.30E-11	0.225448	462346	NA	rs7792436	NA	UKB-b-57	TRUE	reported 8KoqAI
17	44182336	C	A	0.024317	0.003623	1.90E-11	0.225378	462346	NA	rs6206181	NA	UKB-b-57	TRUE	reported 8KoqAI
17	44182706	A	G	0.024225	0.003625	2.30E-11	0.225259	462346	NA	rs6206181	NA	UKB-b-57	TRUE	reported 8KoqAI
17	44182917	C	G	0.024231	0.003623	2.20E-11	0.22545	462346	NA	rs6206181	NA	UKB-b-57	TRUE	reported 8KoqAI
17	44183403	A	G	0.024231	0.003623	2.20E-11	0.22545	462346	NA	rs1116763	NA	UKB-b-57	TRUE	reported 8KoqAI
17	44183616	C	A	0.024231	0.003623	2.30E-11	0.22545	462346	NA	rs1757698	NA	UKB-b-57	TRUE	reported 8KoqAI
17	44183790	G	A	0.024222	0.003623	2.30E-11	0.225436	462346	NA	rs7992370	NA	UKB-b-57	TRUE	reported 8KoqAI
17	44184375	A	G	0.024298	0.003626	2.10E-11	0.225174	462346	NA	rs7934621	NA	UKB-b-57	TRUE	reported 8KoqAI
17	44184404	G	A	0.024367	0.003627	1.80E-11	0.225192	462346	NA	rs7398468	NA	UKB-b-57	TRUE	reported 8KoqAI
17	44184428	T	C	0.024294	0.003627	2.10E-11	0.22513	462346	NA	rs1112956	NA	UKB-b-57	TRUE	reported 8KoqAI
17	44184828	G	T	0.017067	0.00312	4.50E-08	0.402808	462346	NA	rs4510068	NA	UKB-b-57	TRUE	reported 8KoqAI
17	44185245	C	A	0.024226	0.003623	2.30E-11	0.225448	462346	NA	rs7676151	NA	UKB-b-57	TRUE	reported 8KoqAI
17	44186055	G	A	0.024195	0.003631	2.70E-11	0.224787	462346	NA	rs6206182	NA	UKB-b-57	TRUE	reported 8KoqAI
17	44186063	C	T	0.02432	0.003619	1.80E-11	0.227625	462346	NA	rs1694090	NA	UKB-b-57	TRUE	reported 8KoqAI
17	44186252	G	A	0.024217	0.003623	2.30E-11	0.225448	462346	NA	rs1757702	NA	UKB-b-57	TRUE	reported 8KoqAI
17	44186301	T	C	0.024218	0.003623	2.30E-11	0.225448	462346	NA	rs1757705	NA	UKB-b-57	TRUE	reported 8KoqAI
17	44186593	T	C	0.024311	0.003624	2.00E-11	0.225374	462346	NA	rs7763546	NA	UKB-b-57	TRUE	reported 8KoqAI
17	44186894	A	C	0.024217	0.003623	2.30E-11	0.225451	462346	NA	rs6206182	NA	UKB-b-57	TRUE	reported 8KoqAI
17	44187257	A	G	0.024214	0.003605	1.90E-11	0.228839	462346	NA	rs9303525	NA	UKB-b-57	TRUE	reported 8KoqAI
17	44187492	A	G	0.024218	0.003623	2.30E-11	0.225448	462346	NA	rs1757709	NA	UKB-b-57	TRUE	reported 8KoqAI
17	44188477	T	G	0.02424	0.003623	2.20E-11	0.225426	462346	NA	rs1757715	NA	UKB-b-57	TRUE	reported 8KoqAI
17	44188645	C	T	0.024222	0.003623	2.30E-11	0.225455	462346	NA	rs6206182	NA	UKB-b-57	TRUE	reported 8KoqAI
17	44188755	T	C	0.024219	0.003623	2.30E-11	0.225448	462346	NA	rs6206182	NA	UKB-b-57	TRUE	reported 8KoqAI
17	44188764	T	C	0.024219	0.003623	2.30E-11	0.225448	462346	NA	rs5579406	NA	UKB-b-57	TRUE	reported 8KoqAI
17	44189067	A	G	0.017485	0.003079	1.40E-08	0.414409	462346	NA	rs7225002	NA	UKB-b-57	TRUE	reported 8KoqAI
17	44189373	C	T	0.024223	0.003623	2.30E-11	0.225444	462346	NA	rs1766084	NA	UKB-b-57	TRUE	reported 8KoqAI
17	44189408	T	C	0.024222	0.003623	2.30E-11	0.225445	462346	NA	rs1766086	NA	UKB-b-57	TRUE	reported 8KoqAI
17	44189855	G	A	0.024017	0.003646	4.50E-11	0.223761	462346	NA	rs7977278	NA	UKB-b-57	TRUE	reported 8KoqAI
17	44189858	G	A	0.024098	0.003638	3.50E-11	0.224155	462346	NA	rs7664033	NA	UKB-b-57	TRUE	reported 8KoqAI
17	44189910	A	G	0.024291	0.003636	2.40E-11	0.224431	462346	NA	rs7892933	NA	UKB-b-57	TRUE	reported 8KoqAI
17	44191085	G	A	0.024199	0.003623	2.40E-11	0.225443	462346	NA	rs1766090	NA	UKB-b-57	TRUE	reported 8KoqAI
17	44191159	C	T	0.024214	0.003623	2.30E-11	0.22545	462346	NA	rs1766093	NA	UKB-b-57	TRUE	reported 8KoqAI
17	44191682	G	A	0.024213	0.003623	2.30E-11	0.225462	462346	NA	rs6206184	NA	UKB-b-57	TRUE	reported 8KoqAI
17	44191835	A	G	0.024248	0.003624	2.20E-11	0.225343	462346	NA	rs1757731	NA	UKB-b-57	TRUE	reported 8KoqAI
17	44192395	G	T	0.024214	0.003623	2.30E-11	0.225449	462346	NA	rs4548919	NA	UKB-b-57	TRUE	reported 8KoqAI
17	44192568	C	T	0.024927	0.003775	4.00E-11	0.206099	462346	NA	rs4630591	NA	UKB-b-57	TRUE	reported 8KoqAI
17	44192618	A	G	0.024205	0.003623	2.40E-11	0.22545	462346	NA	rs4606752	NA	UKB-b-57	TRUE	reported 8KoqAI
17	44192923	A	G	0.023935	0.003651	5.50E-11	0.223447	462346	NA	rs1757736	NA	UKB-b-57	TRUE	reported 8KoqAI
17	44192946	C	T	0.023876	0.003651	6.20E-11	0.223132	462346	NA	rs6206184	NA	UKB-b-57	TRUE	reported 8KoqAI
17	44192957	T	C	0.023564	0.003654	1.10E-10	0.223325	462346	NA	rs1766101	NA	UKB-b-57	TRUE	reported 8KoqAI
17	44193097	C	A	0.024073	0.003625	3.10E-11	0.225281	462346	NA	rs1766102	NA	UKB-b-57	TRUE	reported 8KoqAI
17	44193882	C	T	0.024161	0.003624	2.60E-11	0.225423	462346	NA	rs6206184	NA	UKB-b-57	TRUE	reported 8KoqAI
17	44194152	T	A	0.024162	0.003624	2.60E-11	0.225434	462346	NA	rs1766104	NA	UKB-b-57	TRUE	reported 8KoqAI
17	44194381	T	A	0.024159	0.003624	2.60E-11	0.225433	462346	NA	rs1757744	NA	UKB-b-57	TRUE	reported 8KoqAI
17	44194519	T	C	0.024162	0.003624	2.60E-11	0.225434	462346	NA	rs6206185	NA	UKB-b-57	TRUE	reported 8KoqAI
17	44194621	C	G	0.024198	0.003624	2.40E-11	0.225408	462346	NA	rs1118801	NA	UKB-b-57	TRUE	reported 8KoqAI
17	44194735	G	T	0.024138	0.003624	2.70E-11	0.22542	462346	NA	rs1051490	NA	UKB-b-57	TRUE	reported 8KoqAI
17	44194835	T	C	0.02415	0.003624	2.70E-11	0.225406	462346	NA	rs1757749	NA	UKB-b-57	TRUE	reported 8KoqAI

17	44194891	C	T	0.02414	0.003624	2.70E-11	0.22542	462346	NA	rs6206185	NA	UKB-b-57	TRUE	reported	8KoqAI
17	44195424	A	C	0.024162	0.003624	2.60E-11	0.225434	462346	NA	rs6206185	NA	UKB-b-57	TRUE	reported	8KoqAI
17	44195495	G	A	0.024162	0.003624	2.60E-11	0.225434	462346	NA	rs727425	NA	UKB-b-57	TRUE	reported	8KoqAI
17	44196015	C	G	0.024159	0.003624	2.60E-11	0.225432	462346	NA	rs1766114	NA	UKB-b-57	TRUE	reported	8KoqAI
17	44196125	T	C	0.024166	0.003624	2.60E-11	0.225393	462346	NA	rs6206185	NA	UKB-b-57	TRUE	reported	8KoqAI
17	44196153	A	G	0.024259	0.003625	2.20E-11	0.225317	462346	NA	rs1468241	NA	UKB-b-57	TRUE	reported	8KoqAI
17	44196447	T	A	0.024151	0.003624	2.60E-11	0.225429	462346	NA	rs1468240	NA	UKB-b-57	TRUE	reported	8KoqAI
17	44196653	G	A	0.024141	0.003624	2.70E-11	0.225415	462346	NA	rs7522928	NA	UKB-b-57	TRUE	reported	8KoqAI
17	44196799	G	A	0.024151	0.003624	2.60E-11	0.22543	462346	NA	rs1128138	NA	UKB-b-57	TRUE	reported	8KoqAI
17	44196822	G	A	0.024137	0.003624	2.70E-11	0.2254	462346	NA	rs1139094	NA	UKB-b-57	TRUE	reported	8KoqAI
17	44197032	T	C	0.024143	0.003624	2.70E-11	0.225428	462346	NA	rs1116786	NA	UKB-b-57	TRUE	reported	8KoqAI
17	44197603	T	C	0.024211	0.003624	2.40E-11	0.225576	462346	NA	rs4383188	NA	UKB-b-57	TRUE	reported	8KoqAI
17	44197806	T	C	0.024162	0.003624	2.60E-11	0.225433	462346	NA	rs1966345	NA	UKB-b-57	TRUE	reported	8KoqAI
17	44198080	C	T	0.024162	0.003624	2.60E-11	0.225433	462346	NA	rs6206185	NA	UKB-b-57	TRUE	reported	8KoqAI
17	44198806	T	C	0.024154	0.003624	2.60E-11	0.225457	462346	NA	rs3087534	NA	UKB-b-57	TRUE	reported	8KoqAI
17	44199029	T	C	0.024179	0.003624	2.50E-11	0.225864	462346	NA	rs7692447	NA	UKB-b-57	TRUE	reported	8KoqAI
17	44200078	A	T	0.024169	0.003624	2.60E-11	0.22543	462346	NA	rs1125601	NA	UKB-b-57	TRUE	reported	8KoqAI
17	44200510	C	A	0.024169	0.003624	2.60E-11	0.22543	462346	NA	rs7565250	NA	UKB-b-57	TRUE	reported	8KoqAI
17	44201109	C	G	0.024168	0.003624	2.60E-11	0.225431	462346	NA	rs6206185	NA	UKB-b-57	TRUE	reported	8KoqAI
17	44201344	G	C	0.024173	0.003624	2.50E-11	0.225428	462346	NA	rs5580259	NA	UKB-b-57	TRUE	reported	8KoqAI
17	44201667	G	A	0.024269	0.003625	2.10E-11	0.225346	462346	NA	rs5625709	NA	UKB-b-57	TRUE	reported	8KoqAI
17	44201680	A	C	0.024269	0.003625	2.10E-11	0.225346	462346	NA	rs5567251	NA	UKB-b-57	TRUE	reported	8KoqAI
17	44201791	G	C	0.024253	0.003625	2.20E-11	0.225324	462346	NA	rs1120732	NA	UKB-b-57	TRUE	reported	8KoqAI
17	44201865	C	T	0.024246	0.003625	2.30E-11	0.225327	462346	NA	rs6206316	NA	UKB-b-57	TRUE	reported	8KoqAI
17	44201887	C	T	0.024246	0.003625	2.30E-11	0.225327	462346	NA	rs6206316	NA	UKB-b-57	TRUE	reported	8KoqAI
17	44202147	A	G	0.024246	0.003625	2.20E-11	0.225327	462346	NA	rs6206316	NA	UKB-b-57	TRUE	reported	8KoqAI
17	44202231	A	G	0.024251	0.003625	2.20E-11	0.22533	462346	NA	rs6206316	NA	UKB-b-57	TRUE	reported	8KoqAI
17	44202564	G	A	0.02427	0.003625	2.10E-11	0.225351	462346	NA	rs5566950	NA	UKB-b-57	TRUE	reported	8KoqAI
17	44202608	C	T	0.024176	0.003625	2.60E-11	0.225298	462346	NA	rs5568610	NA	UKB-b-57	TRUE	reported	8KoqAI
17	44202952	C	G	0.024296	0.003625	2.10E-11	0.225309	462346	NA	rs5588506	NA	UKB-b-57	TRUE	reported	8KoqAI
17	44203131	C	G	0.024296	0.003625	2.10E-11	0.22531	462346	NA	rs5627358	NA	UKB-b-57	TRUE	reported	8KoqAI
17	44203311	C	T	0.024264	0.003625	2.20E-11	0.225353	462346	NA	rs6206317	NA	UKB-b-57	TRUE	reported	8KoqAI
17	44203366	T	C	0.024289	0.003625	2.10E-11	0.225311	462346	NA	rs6206317	NA	UKB-b-57	TRUE	reported	8KoqAI
17	44203413	C	T	0.02428	0.003625	2.10E-11	0.225303	462346	NA	rs7638049	NA	UKB-b-57	TRUE	reported	8KoqAI
17	44203520	C	T	0.024259	0.003625	2.20E-11	0.225354	462346	NA	rs1131208	NA	UKB-b-57	TRUE	reported	8KoqAI
17	44204304	T	G	0.024275	0.003625	2.10E-11	0.225369	462346	NA	rs7709512	NA	UKB-b-57	TRUE	reported	8KoqAI
17	44204739	T	C	0.024199	0.003625	2.50E-11	0.225269	462346	NA	rs4581739	NA	UKB-b-57	TRUE	reported	8KoqAI
17	44204749	A	G	0.024199	0.003625	2.50E-11	0.225269	462346	NA	rs1136385	NA	UKB-b-57	TRUE	reported	8KoqAI
17	44205122	C	G	0.024076	0.003624	3.10E-11	0.225378	462346	NA	rs6206317	NA	UKB-b-57	TRUE	reported	8KoqAI
17	44205500	A	G	0.024069	0.003625	3.10E-11	0.225352	462346	NA	rs1757765	NA	UKB-b-57	TRUE	reported	8KoqAI
17	44205690	C	T	0.024032	0.003613	2.90E-11	0.227616	462346	NA	rs4471723	NA	UKB-b-57	TRUE	reported	8KoqAI
17	44205839	A	G	0.024064	0.003625	3.20E-11	0.225346	462346	NA	rs1122381	NA	UKB-b-57	TRUE	reported	8KoqAI
17	44206079	C	T	0.024062	0.003625	3.20E-11	0.225362	462346	NA	rs1122380	NA	UKB-b-57	TRUE	reported	8KoqAI
17	44206482	C	T	0.024069	0.003625	3.10E-11	0.225352	462346	NA	rs6206320	NA	UKB-b-57	TRUE	reported	8KoqAI
17	44206646	C	G	0.024071	0.003625	3.10E-11	0.225351	462346	NA	rs1051490	NA	UKB-b-57	TRUE	reported	8KoqAI
17	44207066	A	G	0.024076	0.003624	3.10E-11	0.225376	462346	NA	rs1766134	NA	UKB-b-57	TRUE	reported	8KoqAI
17	44207778	A	G	0.023985	0.003625	3.70E-11	0.225361	462346	NA	rs1766138	NA	UKB-b-57	TRUE	reported	8KoqAI
17	44207887	C	T	0.024207	0.003623	2.40E-11	0.226022	462346	NA	rs1051490	NA	UKB-b-57	TRUE	reported	8KoqAI
17	44207932	A	G	0.02408	0.003626	3.10E-11	0.225303	462346	NA	rs7930152	NA	UKB-b-57	TRUE	reported	8KoqAI
17	44208144	C	G	0.024061	0.003625	3.20E-11	0.225349	462346	NA	rs1766142	NA	UKB-b-57	TRUE	reported	8KoqAI
17	44208218	A	G	0.024061	0.003625	3.20E-11	0.22535	462346	NA	rs1757787	NA	UKB-b-57	TRUE	reported	8KoqAI
17	44208312	G	T	0.023994	0.003606	2.90E-11	0.228775	462346	NA	rs6503457	NA	UKB-b-57	TRUE	reported	8KoqAI
17	44208674	G	T	0.024	0.003606	2.80E-11	0.228766	462346	NA	rs8070942	NA	UKB-b-57	TRUE	reported	8KoqAI
17	44208945	C	T	0.02408	0.003624	3.10E-11	0.225376	462346	NA	rs6206320	NA	UKB-b-57	TRUE	reported	8KoqAI
17	44209313	T	C	0.024083	0.003624	3.00E-11	0.225375	462346	NA	rs6206320	NA	UKB-b-57	TRUE	reported	8KoqAI
17	44209364	G	A	0.024081	0.003624	3.00E-11	0.225376	462346	NA	rs1117243	NA	UKB-b-57	TRUE	reported	8KoqAI
17	44209474	A	T	0.024081	0.003624	3.00E-11	0.225376	462346	NA	rs1124016	NA	UKB-b-57	TRUE	reported	8KoqAI
17	44209628	T	C	0.0241	0.003625	2.90E-11	0.225374	462346	NA	rs1757795	NA	UKB-b-57	TRUE	reported	8KoqAI
17	44209875	T	C	0.024104	0.003625	3.00E-11	0.225267	462346	NA	rs5578078	NA	UKB-b-57	TRUE	reported	8KoqAI
17	44209919	C	G	0.024102	0.003626	3.00E-11	0.225265	462346	NA	rs3089	NA	UKB-b-57	TRUE	reported	8KoqAI
17	44209957	A	G	0.024126	0.003625	2.80E-11	0.225342	462346	NA	rs5590904	NA	UKB-b-57	TRUE	reported	8KoqAI
17	44209985	T	C	0.024112	0.003625	2.90E-11	0.225329	462346	NA	rs5611970	NA	UKB-b-57	TRUE	reported	8KoqAI
17	44210442	C	T	0.024186	0.003629	2.60E-11	0.224869	462346	NA	rs1757797	NA	UKB-b-57	TRUE	reported	8KoqAI
17	44210933	A	G	0.024062	0.003608	2.60E-11	0.228665	462346	NA	rs7207582	NA	UKB-b-57	TRUE	reported	8KoqAI
17	44210988	C	G	0.024149	0.003625	2.70E-11	0.225287	462346	NA	rs1215008	NA	UKB-b-57	TRUE	reported	8KoqAI
17	44211502	G	A	0.024079	0.003624	3.10E-11	0.225378	462346	NA	rs1114249	NA	UKB-b-57	TRUE	reported	8KoqAI
17	44211902	A	G	0.024079	0.003624	3.10E-11	0.225377	462346	NA	rs6206320	NA	UKB-b-57	TRUE	reported	8KoqAI
17	44212032	C	A	0.02408	0.003624	3.10E-11	0.225377	462346	NA	rs6206320	NA	UKB-b-57	TRUE	reported	8KoqAI
17	44212310	G	A	0.024097	0.003607	2.40E-11	0.228696	462346	NA	rs1022124	NA	UKB-b-57	TRUE	reported	8KoqAI
17	44212782	T	C	0.0242	0.003605	1.90E-11	0.229152	462346	NA	17_442127	NA	UKB-b-57	TRUE	reported	8KoqAI
17	44213204	C	T	0.024163	0.003624	2.60E-11	0.225586	462346	NA	rs1215032	NA	UKB-b-57	TRUE	reported	8KoqAI
17	44213712	G	A	0.024136	0.003624	2.80E-11	0.225614	462346	NA	rs2532316	NA	UKB-b-57	TRUE	reported	8KoqAI
17	44214094	A	G	0.024172	0.003624	2.60E-11	0.225601	462346	NA	rs2696604	NA	UKB-b-57	TRUE	reported	8KoqAI
17	44214815	C	G	0.024126	0.003624	2.80E-11	0.225596	462346	NA	rs740710	NA	UKB-b-57	TRUE	reported	8KoqAI
17	44214888	T	C	0.025356	0.003905	8.40E-11	0.202364	462346	NA	rs2077551	NA	UKB-b-57	TRUE	reported	8KoqAI
17	44215045	T	C	0.024135	0.003624	2.80E-11	0.225594	462346	NA	rs1918802	NA	UKB-b-57	TRUE	reported	8KoqAI
17	44215823	A	G	0.024134	0.003624	2.80E-11	0.225596	462346	NA	rs2696603	NA	UKB-b-57	TRUE	reported	8KoqAI
17	44215896	C	T	0.023606	0.00391	1.60E-09	0.202698	462346	NA	rs2696602	NA	UKB-b-57	TRUE	reported	8KoqAI
17	44216226	A	G	0.024138	0.003624	2.70E-11	0.225595	462346	NA	rs2696600	NA	UKB-b-57	TRUE	reported	8KoqAI
17	44217112	G	T	0.024138	0.003625	2.70E-11	0.225577	462346	NA	rs2532314	NA	UKB-b-57	TRUE	reported	8KoqAI
17	44217226	A	G	0.02415	0.003625	2.70E-11	0.225592	462346	NA	rs2696599	NA	UKB-b-57	TRUE	reported	8KoqAI
17	44217293	G	A	0.024108	0.003624	2.90E-11	0.225592	462346	NA	rs2532313	NA	UKB-b-57	TRUE	reported	8KoqAI
17	44218242	C	T	0.024125	0.003625	2.80E-11	0.225576	462346	NA	rs1918799	NA	UKB-b-57	TRUE	reported	8KoqAI
17	44219831	T	A	0.023818	0.003619	4.70E-11	0.226562	462346	NA	17_442198	NA	UKB-b-57	TRUE	reported	8KoqAI
17	44220454	T	G	0.024093	0.003625	3.00E-11	0.225577	462346	NA	rs1528075	NA	UKB-b-57	TRUE	reported	8KoqAI
17	44220942	G	A	0.024123	0.003625	2.80E-11	0.225589	462346	NA	rs2532308	NA	UKB-b-57	TRUE	reported	8KoqAI
17	44221350	G	A	0.024109	0.003624	2.90E-11	0.225585	462346	NA	17_442213	NA	UKB-b-57	TRUE	reported	8KoqAI
17	44221602	G	C	0.024094	0.003624	3.00E-11	0.225577	462346	NA	rs2696590	NA	UKB-b-57	TRUE	reported	8KoqAI
17	44221836	C	T	0.024165	0.00391	6.40E-10	0.202768	462346	NA	rs2696589	NA	UKB-b-57	TRUE	reported	8KoqAI
17	44222019	G	T	0.023561	0.003912	1.70E-09	0.202517	462346	NA	rs2222746	NA	UKB-b-57	TRUE	reported	8KoqAI
17	44222067	T	G	0.024117	0.003625	2.90E-11	0.225589	462346	NA	rs1406074	NA	UKB-b-57	TRUE	reported	8KoqAI
17	44222335	G	A	0.024146	0.003624	2.70E-11	0.225607	462346	NA	rs1918798	NA	UKB-b-57	TRUE	reported	8KoqAI
17	44222460	T	G	0.02414	0.003624	2.70E-11	0.225584	462346	NA	rs2696587	NA	UKB-b-57	TRUE	reported	8KoqAI
17	44223060	G	A	0.02411	0.003625	2.90E-11	0.225594	462346	NA	rs2532305	NA	UKB-b-57	TRUE	reported	8KoqAI
17	44223243	G	A	0.024132	0.003625	2.80E-11	0.225594	462346	NA	rs1918797	NA	UKB-b-57	TRUE	reported	8KoqAI
17	44223387	T	C	0.024141	0.003625	2.70E-11	0.225603	462346	NA	rs1918796	NA	UKB-b-57	TRUE	reported	8KoqAI
17	44224686	A	T	0.024133	0.003625	2.80E-11	0.225592	462346	NA	rs1918795	NA	UKB-b-57	TRUE	reported	8KoqAI
17	44225485	T	C	0.024132	0.003625	2.80E-11	0.225592	462346	NA	rs1406071	NA	UKB-b-57	TRUE	reported	8KoqAI
17	44225802	C	T	0.024132	0.003625	2.80E-11	0.225591	462346	NA	rs1406070	NA	UKB-b-57	TRUE	reported	8KoqAI
17	44225886	G	A	0.023979	0.00393	1.00E-09	0.201495	462346	NA	rs1406069	NA	UKB-b-57	TRUE	reported	8KoqAI
17	44226030	G	A	0.024131	0.003625	2.80E-11	0.225591	462346	NA	rs2696585	NA	UKB-b-57	TRUE	reported	8KoqAI
17	44226126	A	C	0.024131	0.003625	2.80E-11	0.225591	462346	NA	rs2532304	NA	UKB-b-57	TRUE	reported	8KoqAI
17	44226980	A	G	0.024029	0.003905	7.60E-10	0.203157	462346	NA	rs6206323	NA	UKB-b-57	TRUE	reported	8KoqAI
17	44227623	T	C	0.024072	0.003607	2.50E-11	0.229055	462346	NA	rs2696578	NA	UKB-b-57	TRUE	reported	8KoqAI
17	44228169	C	T	0.024397	0.003608	1.40E-11	0.229407	462346	NA	rs1918793	NA	UKB-b-57	TRUE	reported	8KoqAI
17	44228529	G	A	0.024172	0.003629	2.70E-11	0.225562	462346	NA	rs1918792	NA	UKB-b-57	TRUE	reported	8KoqAI
17	44228609	C	G	0.024172	0.003629	2.70E-11	0.225562	462346	NA	rs1918791	NA	UKB-b-57	TRUE	reported	8KoqAI

17	44228770	A	G	0.024173	0.003629	2.70E-11	0.225561	462346	NA	rs2696576	NA	UKB-b-57	TRUE	reported	8KoqAI
17	44228824	A	G	0.024171	0.003629	2.70E-11	0.225555	462346	NA	rs2696575	NA	UKB-b-57	TRUE	reported	8KoqAI
17	44229986	T	C	0.024153	0.003629	2.80E-11	0.225549	462346	NA	rs2532300	NA	UKB-b-57	TRUE	reported	8KoqAI
17	44230647	C	T	0.02418	0.003629	2.70E-11	0.225552	462346	NA	rs2696573	NA	UKB-b-57	TRUE	reported	8KoqAI
17	44231117	T	C	0.024184	0.003629	2.70E-11	0.225556	462346	NA	rs2532299	NA	UKB-b-57	TRUE	reported	8KoqAI
17	44231295	G	A	0.024185	0.003629	2.60E-11	0.225556	462346	NA	rs2532298	NA	UKB-b-57	TRUE	reported	8KoqAI
17	44231326	G	A	0.024183	0.003629	2.70E-11	0.225556	462346	NA	rs2532297	NA	UKB-b-57	TRUE	reported	8KoqAI
17	44231617	A	T	0.024182	0.003629	2.70E-11	0.225556	462346	NA	rs2696572	NA	UKB-b-57	TRUE	reported	8KoqAI
17	44232408	G	A	0.025848	0.0039	3.40E-11	0.202806	462346	NA	rs2532295	NA	UKB-b-57	TRUE	reported	8KoqAI
17	44232959	G	A	0.024183	0.003629	2.70E-11	0.225558	462346	NA	rs2109092	NA	UKB-b-57	TRUE	reported	8KoqAI
17	44233433	G	C	0.024179	0.003629	2.70E-11	0.22556	462346	NA	rs2532294	NA	UKB-b-57	TRUE	reported	8KoqAI
17	44233589	T	C	0.024182	0.003629	2.70E-11	0.225557	462346	NA	rs2532293	NA	UKB-b-57	TRUE	reported	8KoqAI
17	44233776	T	C	0.024177	0.003629	2.70E-11	0.225557	462346	NA	rs1766223	NA	UKB-b-57	TRUE	reported	8KoqAI
17	44233811	C	T	0.024177	0.003629	2.70E-11	0.225557	462346	NA	rs1758521	NA	UKB-b-57	TRUE	reported	8KoqAI
17	44234060	A	G	0.024205	0.003629	2.50E-11	0.225616	462346	NA	rs1528074	NA	UKB-b-57	TRUE	reported	8KoqAI
17	44234526	C	T	0.024153	0.003629	2.80E-11	0.225536	462346	NA	rs1406068	NA	UKB-b-57	TRUE	reported	8KoqAI
17	44235893	C	T	0.024175	0.003629	2.70E-11	0.225549	462346	NA	rs6206086	NA	UKB-b-57	TRUE	reported	8KoqAI
17	44236725	C	A	0.024159	0.003629	2.80E-11	0.225558	462346	NA	rs1528072	NA	UKB-b-57	TRUE	reported	8KoqAI
17	44237068	A	T	0.024186	0.003629	2.60E-11	0.22555	462346	NA	rs2532292	NA	UKB-b-57	TRUE	reported	8KoqAI
17	44237372	G	C	0.024156	0.003629	2.80E-11	0.225559	462346	NA	rs2696571	NA	UKB-b-57	TRUE	reported	8KoqAI
17	44237790	G	A	0.024155	0.003629	2.80E-11	0.22556	462346	NA	rs2696570	NA	UKB-b-57	TRUE	reported	8KoqAI
17	44238424	G	A	0.024134	0.003629	2.90E-11	0.225542	462346	NA	rs2532291	NA	UKB-b-57	TRUE	reported	8KoqAI
17	44238571	C	G	0.024199	0.003612	2.10E-11	0.228822	462346	NA	rs2532289	NA	UKB-b-57	TRUE	reported	8KoqAI
17	44238938	T	C	0.024134	0.003629	2.90E-11	0.225542	462346	NA	rs1766240	NA	UKB-b-57	TRUE	reported	8KoqAI
17	44238966	T	C	0.024135	0.003629	2.90E-11	0.225542	462346	NA	rs1758542	NA	UKB-b-57	TRUE	reported	8KoqAI
17	44239958	C	T	0.024133	0.003629	2.90E-11	0.225538	462346	NA	rs2532288	NA	UKB-b-57	TRUE	reported	8KoqAI
17	44240107	T	C	0.024133	0.003629	2.90E-11	0.225538	462346	NA	rs1918789	NA	UKB-b-57	TRUE	reported	8KoqAI
17	44240986	C	A	0.024143	0.003629	2.90E-11	0.225532	462346	NA	rs2141299	NA	UKB-b-57	TRUE	reported	8KoqAI
17	44241304	C	G	0.024143	0.003629	2.90E-11	0.225532	462346	NA	rs2696567	NA	UKB-b-57	TRUE	reported	8KoqAI
17	44241664	T	C	0.024172	0.003629	2.70E-11	0.225528	462346	NA	rs2532286	NA	UKB-b-57	TRUE	reported	8KoqAI
17	44242788	C	T	0.024126	0.003629	3.00E-11	0.225582	462346	NA	rs4792845	NA	UKB-b-57	TRUE	reported	8KoqAI
17	44242969	T	C	0.024141	0.003629	2.90E-11	0.225512	462346	NA	rs4792844	NA	UKB-b-57	TRUE	reported	8KoqAI
17	44243179	A	G	0.024143	0.003629	2.90E-11	0.22551	462346	NA	rs4792843	NA	UKB-b-57	TRUE	reported	8KoqAI
17	44243979	C	T	0.024142	0.003629	2.90E-11	0.225512	462346	NA	rs1758560	NA	UKB-b-57	TRUE	reported	8KoqAI
17	44244397	G	A	0.024187	0.003629	2.60E-11	0.225634	462346	NA	rs2696684	NA	UKB-b-57	TRUE	reported	8KoqAI
17	44244581	T	C	0.024142	0.003629	2.90E-11	0.225512	462346	NA	rs1758564	NA	UKB-b-57	TRUE	reported	8KoqAI
17	44244926	T	G	0.024127	0.003629	3.00E-11	0.225509	462346	NA	rs2696657	NA	UKB-b-57	TRUE	reported	8KoqAI
17	44245171	G	T	0.023977	0.003905	8.20E-10	0.203442	462346	NA	rs2532281	NA	UKB-b-57	TRUE	reported	8KoqAI
17	44245876	A	G	0.024142	0.003629	2.90E-11	0.225512	462346	NA	rs2696659	NA	UKB-b-57	TRUE	reported	8KoqAI
17	44246211	A	G	0.024143	0.003629	2.90E-11	0.225512	462346	NA	rs2696660	NA	UKB-b-57	TRUE	reported	8KoqAI
17	44246405	C	A	0.024142	0.003629	2.90E-11	0.225512	462346	NA	rs2532278	NA	UKB-b-57	TRUE	reported	8KoqAI
17	44246624	C	A	0.024142	0.003629	2.90E-11	0.225511	462346	NA	17_442466	NA	UKB-b-57	TRUE	reported	8KoqAI
17	44246997	G	A	0.024142	0.003629	2.90E-11	0.225512	462346	NA	rs2532275	NA	UKB-b-57	TRUE	reported	8KoqAI
17	44247164	A	G	0.024208	0.003612	2.00E-11	0.228785	462346	NA	rs2532274	NA	UKB-b-57	TRUE	reported	8KoqAI
17	44247314	C	T	0.024146	0.003629	2.90E-11	0.225513	462346	NA	rs2532273	NA	UKB-b-57	TRUE	reported	8KoqAI
17	44247673	G	A	0.024134	0.003629	2.90E-11	0.225507	462346	NA	rs2532272	NA	UKB-b-57	TRUE	reported	8KoqAI
17	44248042	G	A	0.024135	0.003629	2.90E-11	0.225504	462346	NA	rs2532271	NA	UKB-b-57	TRUE	reported	8KoqAI
17	44248769	T	C	0.024151	0.003629	2.80E-11	0.225562	462346	NA	rs1881193	NA	UKB-b-57	TRUE	reported	8KoqAI
17	44248814	G	A	0.023549	0.003643	1.00E-10	0.224438	462346	NA	rs1881194	NA	UKB-b-57	TRUE	reported	8KoqAI
17	44248837	T	C	0.022497	0.004035	2.50E-08	0.192068	462346	NA	rs3564321	NA	UKB-b-57	TRUE	reported	8KoqAI
17	44249096	A	C	0.023823	0.004043	3.80E-09	0.190015	462346	NA	rs1766288	NA	UKB-b-57	TRUE	reported	8KoqAI
17	44249199	T	G	0.022261	0.004048	3.80E-08	0.190849	462346	NA	rs1758597	NA	UKB-b-57	TRUE	reported	8KoqAI
17	44249621	A	G	0.022929	0.00367	4.20E-10	0.223704	462346	NA	rs2696662	NA	UKB-b-57	TRUE	reported	8KoqAI
17	44249800	G	A	0.022948	0.00367	4.00E-10	0.223694	462346	NA	rs2532270	NA	UKB-b-57	TRUE	reported	8KoqAI
17	44250108	T	C	0.023056	0.003668	3.30E-10	0.224277	462346	NA	rs2532269	NA	UKB-b-57	TRUE	reported	8KoqAI
17	44250616	G	A	0.022944	0.00367	4.00E-10	0.223708	462346	NA	rs2532267	NA	UKB-b-57	TRUE	reported	8KoqAI
17	44251230	T	C	0.022941	0.00367	4.10E-10	0.223689	462346	NA	rs956329	NA	UKB-b-57	TRUE	reported	8KoqAI
17	44251582	T	C	0.022946	0.00367	4.00E-10	0.223692	462346	NA	rs6206091	NA	UKB-b-57	TRUE	reported	8KoqAI
17	44251769	A	G	0.022946	0.00367	4.00E-10	0.223692	462346	NA	rs2696665	NA	UKB-b-57	TRUE	reported	8KoqAI
17	44251972	G	A	0.02294	0.00367	4.10E-10	0.223689	462346	NA	rs2696666	NA	UKB-b-57	TRUE	reported	8KoqAI
17	44252098	C	T	0.022943	0.00367	4.10E-10	0.223695	462346	NA	rs2732644	NA	UKB-b-57	TRUE	reported	8KoqAI
17	44252197	G	T	0.022946	0.00367	4.00E-10	0.223691	462346	NA	rs2532265	NA	UKB-b-57	TRUE	reported	8KoqAI
17	44252416	G	C	0.02295	0.00367	4.00E-10	0.223689	462346	NA	rs2532264	NA	UKB-b-57	TRUE	reported	8KoqAI
17	44252468	G	A	0.022419	0.004039	2.90E-08	0.195019	462346	NA	rs2532263	NA	UKB-b-57	TRUE	reported	8KoqAI
17	44253364	G	A	0.022949	0.00367	4.00E-10	0.223689	462346	NA	rs2532259	NA	UKB-b-57	TRUE	reported	8KoqAI
17	44254379	C	T	0.022963	0.00367	3.90E-10	0.223689	462346	NA	rs2732646	NA	UKB-b-57	TRUE	reported	8KoqAI
17	44254413	A	G	0.022963	0.00367	3.90E-10	0.223689	462346	NA	rs2732647	NA	UKB-b-57	TRUE	reported	8KoqAI
17	44254494	T	C	0.022963	0.00367	3.90E-10	0.22369	462346	NA	rs2532257	NA	UKB-b-57	TRUE	reported	8KoqAI
17	44255532	T	C	0.022943	0.00367	4.10E-10	0.22369	462346	NA	rs2532255	NA	UKB-b-57	TRUE	reported	8KoqAI
17	44255550	A	G	0.02412	0.004022	2.00E-09	0.19221	462346	NA	rs5589143	NA	UKB-b-57	TRUE	reported	8KoqAI
17	44255777	G	A	0.022941	0.00367	4.10E-10	0.223692	462346	NA	rs2696696	NA	UKB-b-57	TRUE	reported	8KoqAI
17	44256296	G	A	0.022383	0.004008	2.30E-08	0.197049	462346	NA	rs2532253	NA	UKB-b-57	TRUE	reported	8KoqAI
17	44256655	G	A	0.024174	0.003998	1.50E-09	0.196767	462346	NA	rs740708	NA	UKB-b-57	TRUE	reported	8KoqAI
17	44258231	A	G	0.02294	0.00367	4.10E-10	0.223685	462346	NA	rs740707	NA	UKB-b-57	TRUE	reported	8KoqAI
17	44258354	G	C	0.022943	0.00367	4.10E-10	0.223689	462346	NA	rs740706	NA	UKB-b-57	TRUE	reported	8KoqAI
17	44258422	G	T	0.022943	0.00367	4.10E-10	0.223689	462346	NA	rs758523	NA	UKB-b-57	TRUE	reported	8KoqAI
17	44258954	G	C	0.022945	0.00367	4.00E-10	0.223688	462346	NA	rs2141301	NA	UKB-b-57	TRUE	reported	8KoqAI
17	44259040	G	C	0.022941	0.00367	4.10E-10	0.223691	462346	NA	rs2141302	NA	UKB-b-57	TRUE	reported	8KoqAI
17	44259792	G	A	0.022942	0.00367	4.10E-10	0.223689	462346	NA	rs2696700	NA	UKB-b-57	TRUE	reported	8KoqAI
17	44259919	A	G	0.022719	0.003961	9.70E-09	0.201166	462346	NA	rs2696701	NA	UKB-b-57	TRUE	reported	8KoqAI
17	44260416	T	C	0.022942	0.00367	4.10E-10	0.223688	462346	NA	rs2696702	NA	UKB-b-57	TRUE	reported	8KoqAI
17	44261137	C	T	0.022949	0.00367	4.00E-10	0.223693	462346	NA	rs2732587	NA	UKB-b-57	TRUE	reported	8KoqAI
17	44261753	T	C	0.02294	0.00367	4.10E-10	0.223687	462346	NA	rs2696704	NA	UKB-b-57	TRUE	reported	8KoqAI
17	44262758	C	T	0.023442	0.004071	8.50E-09	0.188538	462346	NA	rs6206092	NA	UKB-b-57	TRUE	reported	8KoqAI
17	44263479	T	C	0.022961	0.00367	4.00E-10	0.223703	462346	NA	rs6206092	NA	UKB-b-57	TRUE	reported	8KoqAI
17	44263729	C	G	0.02296	0.00367	4.00E-10	0.223704	462346	NA	rs6206092	NA	UKB-b-57	TRUE	reported	8KoqAI
17	44263923	T	C	0.022762	0.003959	9.00E-09	0.200858	462346	NA	rs1766335	NA	UKB-b-57	TRUE	reported	8KoqAI
17	44263965	T	C	0.023075	0.00367	3.20E-10	0.223268	462346	NA	rs2696705	NA	UKB-b-57	TRUE	reported	8KoqAI
17	44264045	T	C	0.023077	0.00367	3.20E-10	0.223264	462346	NA	rs2696706	NA	UKB-b-57	TRUE	reported	8KoqAI
17	44264717	A	C	0.022961	0.00367	4.00E-10	0.223702	462346	NA	rs2696707	NA	UKB-b-57	TRUE	reported	8KoqAI
17	44264943	T	C	0.02296	0.00367	4.00E-10	0.223702	462346	NA	rs2696708	NA	UKB-b-57	TRUE	reported	8KoqAI
17	44265328	A	G	0.02296	0.00367	4.00E-10	0.223702	462346	NA	rs2532243	NA	UKB-b-57	TRUE	reported	8KoqAI
17	44265702	A	T	0.022959	0.00367	4.00E-10	0.223703	462346	NA	rs2532241	NA	UKB-b-57	TRUE	reported	8KoqAI
17	44266022	A	G	0.02296	0.00367	4.00E-10	0.223702	462346	NA	rs2732589	NA	UKB-b-57	TRUE	reported	8KoqAI
17	44266531	G	A	0.022959	0.00367	4.00E-10	0.223701	462346	NA	17_442665	NA	UKB-b-57	TRUE	reported	8KoqAI
17	44267227	A	G	0.02292	0.003671	4.30E-10	0.22376	462346	NA	rs2732595	NA	UKB-b-57	TRUE	reported	8KoqAI
17	44267617	A	G	0.022952	0.003671	4.00E-10	0.223713	462346	NA	rs1918788	NA	UKB-b-57	TRUE	reported	8KoqAI
17	44267863	G	A	0.022965	0.00367	3.90E-10	0.223716	462346	NA	rs2696526	NA	UKB-b-57	TRUE	reported	8KoqAI
17	44267904	T	C	0.022973	0.00367	3.90E-10	0.223729	462346	NA	rs2696523	NA	UKB-b-57	TRUE	reported	8KoqAI
17	44268488	C	T	0.022956	0.003671	4.00E-10	0.2237	462346	NA	17_442684	NA	UKB-b-57	TRUE	reported	8KoqAI
17	44269546	C	T	0.023018	0.003671	3.60E-10	0.223745	462346	NA	rs2732596	NA	UKB-b-57	TRUE	reported	8KoqAI
17	44269922	T	C	0.023932	0.00402	2.60E-09	0.192439	462346	NA	17_442699	NA	UKB-b-57	TRUE	reported	8KoqAI
17	44269964	T	A	0.023075	0.003671	3.30E-10	0.223261	462346	NA	17_442699	NA	UKB-b-57	TRUE	reported	8KoqAI
17	44270059	A	T	0.022959	0.003671	4.00E-10	0.223698	462346	NA	17_442700	NA	UKB-b-57	TRUE	reported	8KoqAI
17	44271152	G	A	0.023	0.003671	3.70E-10	0.223732	462346	NA	rs2732658	NA	UKB-b-57	TRUE	reported	8KoqAI
17	44272000	G	A	0.022956	0.003671	4.00E-10	0.223697	462346	NA	rs2532235	NA	UKB-b-57	TRUE	reported	8KoqAI
17	44272266	A	G	0.022862	0.003671	4.70E-10	0.223728	462346	NA	rs2532234	NA	UKB-b-57	TRUE	reported	8KoqAI

17	44272552	C	T	0.022929	0.003671	4.20E-10	0.223707	462346	NA	rs1766379	NA	UKB-b-57	TRUE	reported	8KoqAI
17	44272679	G	C	0.022929	0.003671	4.20E-10	0.223707	462346	NA	rs2732660	NA	UKB-b-57	TRUE	reported	8KoqAI
17	44272928	C	A	0.022929	0.003671	4.20E-10	0.223706	462346	NA	rs2696438	NA	UKB-b-57	TRUE	reported	8KoqAI
17	44273218	C	T	0.022862	0.003671	4.70E-10	0.223725	462346	NA	17_442732	NA	UKB-b-57	TRUE	reported	8KoqAI
17	44273264	G	A	0.022861	0.003671	4.70E-10	0.223725	462346	NA	rs2696439	NA	UKB-b-57	TRUE	reported	8KoqAI
17	44274560	T	C	0.022908	0.003671	4.40E-10	0.223649	462346	NA	rs2696440	NA	UKB-b-57	TRUE	reported	8KoqAI
17	44275035	T	G	0.022927	0.003671	4.20E-10	0.223675	462346	NA	rs2696442	NA	UKB-b-57	TRUE	reported	8KoqAI
17	44276013	G	A	0.022971	0.003671	3.90E-10	0.223705	462346	NA	rs1918784	NA	UKB-b-57	TRUE	reported	8KoqAI
17	44276330	T	C	0.022916	0.003671	4.30E-10	0.223671	462346	NA	rs1918785	NA	UKB-b-57	TRUE	reported	8KoqAI
17	44276821	G	A	0.022913	0.003671	4.30E-10	0.223672	462346	NA	rs2696443	NA	UKB-b-57	TRUE	reported	8KoqAI
17	44277265	A	C	0.022925	0.003671	4.20E-10	0.223678	462346	NA	17_442772	NA	UKB-b-57	TRUE	reported	8KoqAI
17	44277691	T	C	0.022936	0.003671	4.20E-10	0.223682	462346	NA	rs2732665	NA	UKB-b-57	TRUE	reported	8KoqAI
17	44277818	A	G	0.022953	0.003671	4.00E-10	0.223682	462346	NA	rs2532228	NA	UKB-b-57	TRUE	reported	8KoqAI
17	44277867	G	A	0.022952	0.003671	4.00E-10	0.223683	462346	NA	rs2532226	NA	UKB-b-57	TRUE	reported	8KoqAI
17	44277923	G	A	0.022897	0.003671	4.50E-10	0.223729	462346	NA	rs2532225	NA	UKB-b-57	TRUE	reported	8KoqAI
17	44278742	A	G	0.022878	0.003671	4.60E-10	0.223687	462346	NA	17_442787	NA	UKB-b-57	TRUE	reported	8KoqAI
17	44279101	A	G	0.022924	0.003671	4.30E-10	0.223673	462346	NA	rs1918786	NA	UKB-b-57	TRUE	reported	8KoqAI
17	44279252	A	G	0.022915	0.003671	4.30E-10	0.223668	462346	NA	rs1918787	NA	UKB-b-57	TRUE	reported	8KoqAI
17	44279760	G	A	0.022928	0.003671	4.20E-10	0.223672	462346	NA	rs1358438	NA	UKB-b-57	TRUE	reported	8KoqAI
17	44279803	T	G	0.024677	0.003963	4.70E-10	0.20007	462346	NA	rs1358437	NA	UKB-b-57	TRUE	reported	8KoqAI
17	44280188	T	A	0.02293	0.003671	4.20E-10	0.223669	462346	NA	rs2732675	NA	UKB-b-57	TRUE	reported	8KoqAI
17	44280523	A	G	0.022937	0.003671	4.20E-10	0.22367	462346	NA	rs2668660	NA	UKB-b-57	TRUE	reported	8KoqAI
17	44280697	T	C	0.022684	0.00395	9.30E-09	0.201919	462346	NA	rs2732674	NA	UKB-b-57	TRUE	reported	8KoqAI
17	44280902	G	C	0.022932	0.003671	4.20E-10	0.223669	462346	NA	rs2732673	NA	UKB-b-57	TRUE	reported	8KoqAI
17	44281452	A	G	0.022816	0.003671	5.10E-10	0.224469	462346	NA	rs2668659	NA	UKB-b-57	TRUE	reported	8KoqAI
17	44281588	T	C	0.022924	0.003671	4.30E-10	0.223671	462346	NA	rs2696451	NA	UKB-b-57	TRUE	reported	8KoqAI
17	44281722	T	C	0.022962	0.003671	4.00E-10	0.223686	462346	NA	rs2696453	NA	UKB-b-57	TRUE	reported	8KoqAI
17	44283022	A	G	0.022961	0.003671	4.00E-10	0.223687	462346	NA	rs2668656	NA	UKB-b-57	TRUE	reported	8KoqAI
17	44283139	C	A	0.022962	0.003671	4.00E-10	0.223742	462346	NA	rs2732671	NA	UKB-b-57	TRUE	reported	8KoqAI
17	44283571	C	T	0.023936	0.00401	2.40E-09	0.196392	462346	NA	rs2696455	NA	UKB-b-57	TRUE	reported	8KoqAI
17	44284057	C	T	0.023	0.003672	3.70E-10	0.223757	462346	NA	rs2696457	NA	UKB-b-57	TRUE	reported	8KoqAI
17	44285531	G	A	0.022962	0.003672	4.00E-10	0.223701	462346	NA	rs2469933	NA	UKB-b-57	TRUE	reported	8KoqAI
17	44286128	A	G	0.022943	0.003672	4.10E-10	0.223688	462346	NA	rs2696463	NA	UKB-b-57	TRUE	reported	8KoqAI
17	44287310	A	G	0.022942	0.003672	4.10E-10	0.223687	462346	NA	rs2696464	NA	UKB-b-57	TRUE	reported	8KoqAI
17	44287373	G	C	0.023763	0.004025	3.50E-09	0.192224	462346	NA	rs2668690	NA	UKB-b-57	TRUE	reported	8KoqAI
17	44288281	C	T	0.024066	0.004017	2.10E-09	0.192696	462346	NA	17_442882	NA	UKB-b-57	TRUE	reported	8KoqAI
17	44288284	G	A	0.022566	0.003953	1.10E-08	0.201832	462346	NA	17_442882	NA	UKB-b-57	TRUE	reported	8KoqAI
17	44288579	T	C	0.022934	0.003672	4.20E-10	0.223684	462346	NA	17_442885	NA	UKB-b-57	TRUE	reported	8KoqAI
17	44289101	A	G	0.022937	0.003672	4.20E-10	0.223683	462346	NA	rs2732629	NA	UKB-b-57	TRUE	reported	8KoqAI
17	44289628	A	C	0.022937	0.003672	4.20E-10	0.223683	462346	NA	rs2668670	NA	UKB-b-57	TRUE	reported	8KoqAI
17	44290047	G	A	0.022935	0.003672	4.20E-10	0.223682	462346	NA	rs2668665	NA	UKB-b-57	TRUE	reported	8KoqAI
17	44290759	T	C	0.022927	0.003672	4.30E-10	0.223679	462346	NA	rs2668662	NA	UKB-b-57	TRUE	reported	8KoqAI
17	44291365	C	T	0.022925	0.003672	4.30E-10	0.223684	462346	NA	17_442913	NA	UKB-b-57	TRUE	reported	8KoqAI
17	44291381	A	G	0.022931	0.003672	4.20E-10	0.223683	462346	NA	17_442913	NA	UKB-b-57	TRUE	reported	8KoqAI
17	44291479	G	A	0.022931	0.003672	4.20E-10	0.223682	462346	NA	rs2732601	NA	UKB-b-57	TRUE	reported	8KoqAI
17	44291820	A	G	0.022929	0.003672	4.30E-10	0.223682	462346	NA	rs2696610	NA	UKB-b-57	TRUE	reported	8KoqAI
17	44292126	T	C	0.022936	0.003672	4.20E-10	0.223689	462346	NA	17_442921	NA	UKB-b-57	TRUE	reported	8KoqAI
17	44292319	T	C	0.02292	0.003673	4.40E-10	0.223681	462346	NA	rs2668694	NA	UKB-b-57	TRUE	reported	8KoqAI
17	44293020	G	A	0.022886	0.003675	4.80E-10	0.223662	462346	NA	rs2668692	NA	UKB-b-57	TRUE	reported	8KoqAI
17	44293205	A	G	0.022865	0.003657	4.00E-10	0.227056	462346	NA	17_442932	NA	UKB-b-57	TRUE	reported	8KoqAI
17	44293671	A	G	0.024029	0.00397	1.40E-09	0.199884	462346	NA	17_442936	NA	UKB-b-57	TRUE	reported	8KoqAI
17	44297148	G	A	0.023221	0.003672	2.60E-10	0.22511	462346	NA	rs2696689	NA	UKB-b-57	TRUE	reported	8KoqAI
17	44302881	C	T	0.022801	0.003676	5.50E-10	0.223696	462346	NA	rs2532404	NA	UKB-b-57	TRUE	reported	8KoqAI
17	44313757	G	A	0.022858	0.003677	5.10E-10	0.2238	462346	NA	rs2532386	NA	UKB-b-57	TRUE	reported	8KoqAI
17	44316076	G	T	0.022965	0.003676	4.20E-10	0.224072	462346	NA	rs5565393	NA	UKB-b-57	TRUE	reported	8KoqAI
17	44316449	A	C	0.022993	0.003676	4.00E-10	0.224094	462346	NA	rs6207309	NA	UKB-b-57	TRUE	reported	8KoqAI
17	44318184	A	G	0.023003	0.003676	3.90E-10	0.224045	462346	NA	rs2696668	NA	UKB-b-57	TRUE	reported	8KoqAI
17	44319366	C	T	0.022817	0.003678	5.50E-10	0.223745	462346	NA	rs2668632	NA	UKB-b-57	TRUE	reported	8KoqAI
17	44319369	T	C	0.022816	0.003678	5.50E-10	0.223745	462346	NA	rs2532382	NA	UKB-b-57	TRUE	reported	8KoqAI
17	44322960	A	G	0.03956	0.006962	1.30E-08	0.082644	462346	NA	rs2668637	NA	UKB-b-57	TRUE	reported	8KoqAI
17	44326245	A	G	0.024259	0.003955	8.60E-10	0.201184	462346	NA	rs2463524	NA	UKB-b-57	TRUE	reported	8KoqAI
17	44331214	T	C	0.023833	0.003907	1.10E-09	0.205189	462346	NA	rs2957316	NA	UKB-b-57	TRUE	reported	8KoqAI
17	44333423	G	T	0.023037	0.003679	3.80E-10	0.224041	462346	NA	rs2532363	NA	UKB-b-57	TRUE	reported	8KoqAI
17	44334052	G	A	0.022957	0.003677	4.30E-10	0.224148	462346	NA	rs6207315	NA	UKB-b-57	TRUE	reported	8KoqAI
17	44338735	C	T	0.022871	0.003679	5.10E-10	0.224225	462346	NA	rs2732686	NA	UKB-b-57	TRUE	reported	8KoqAI
17	44340253	A	G	0.022994	0.003678	4.00E-10	0.224322	462346	NA	rs2532348	NA	UKB-b-57	TRUE	reported	8KoqAI
17	44345063	C	T	0.023748	0.004018	3.40E-09	0.192877	462346	NA	rs2732651	NA	UKB-b-57	TRUE	reported	8KoqAI
17	44347218	C	A	0.023063	0.003701	4.60E-10	0.222294	462346	NA	rs2696557	NA	UKB-b-57	TRUE	reported	8KoqAI
17	44347727	A	T	0.023745	0.004021	3.50E-09	0.192794	462346	NA	rs2532332	NA	UKB-b-57	TRUE	reported	8KoqAI
17	44348298	A	G	0.023747	0.004021	3.50E-09	0.192778	462346	NA	rs2950694	NA	UKB-b-57	TRUE	reported	8KoqAI
17	44349079	C	A	0.023811	0.004019	3.10E-09	0.192955	462346	NA	rs7525741	NA	UKB-b-57	TRUE	reported	8KoqAI
17	44350090	G	A	0.022973	0.003702	5.40E-10	0.222127	462346	NA	rs2532329	NA	UKB-b-57	TRUE	reported	8KoqAI
17	44350293	A	G	0.023885	0.003965	1.70E-09	0.201079	462346	NA	rs2732711	NA	UKB-b-57	TRUE	reported	8KoqAI
17	44351285	A	G	0.023132	0.003703	4.20E-10	0.222253	462346	NA	rs2668617	NA	UKB-b-57	TRUE	reported	8KoqAI
17	44351298	G	A	0.024046	0.003967	1.30E-09	0.200855	462346	NA	rs2696544	NA	UKB-b-57	TRUE	reported	8KoqAI
17	44351387	C	T	0.022966	0.003702	5.50E-10	0.222117	462346	NA	rs2732708	NA	UKB-b-57	TRUE	reported	8KoqAI
17	44351686	C	T	0.023186	0.003701	3.70E-10	0.222344	462346	NA	rs2732706	NA	UKB-b-57	TRUE	reported	8KoqAI
17	44351743	G	A	0.024465	0.003979	7.80E-10	0.200102	462346	NA	rs2696541	NA	UKB-b-57	TRUE	reported	8KoqAI
17	44351929	T	G	0.022953	0.003702	5.70E-10	0.222169	462346	NA	rs2732705	NA	UKB-b-57	TRUE	reported	8KoqAI
17	44352131	G	T	0.022891	0.003702	6.30E-10	0.222326	462346	NA	rs2668622	NA	UKB-b-57	TRUE	reported	8KoqAI
17	44352872	A	G	0.02292	0.003704	6.10E-10	0.222039	462346	NA	rs2668624	NA	UKB-b-57	TRUE	reported	8KoqAI
17	44353222	T	G	0.024049	0.003969	1.40E-09	0.200912	462346	NA	rs2732703	NA	UKB-b-57	TRUE	reported	8KoqAI
17	44353885	G	A	0.022858	0.003704	6.80E-10	0.222108	462346	NA	rs2668628	NA	UKB-b-57	TRUE	reported	8KoqAI
17	44354549	C	G	0.0229	0.003701	6.10E-10	0.222726	462346	NA	rs2261201	NA	UKB-b-57	TRUE	reported	8KoqAI
17	44355602	A	G	0.022996	0.003726	6.80E-10	0.220161	462346	NA	rs2696532	NA	UKB-b-57	TRUE	reported	8KoqAI
17	44355634	C	A	0.022954	0.003756	9.90E-10	0.21225	462346	NA	rs2696531	NA	UKB-b-57	TRUE	reported	8KoqAI
17	44356793	A	G	0.022871	0.003704	6.60E-10	0.222075	462346	NA	rs2696524	NA	UKB-b-57	TRUE	reported	8KoqAI
17	44356949	T	C	0.022895	0.003704	6.40E-10	0.222034	462346	NA	rs7856971	NA	UKB-b-57	TRUE	reported	8KoqAI
17	44357351	T	C	0.022888	0.003704	6.50E-10	0.222019	462346	NA	rs1766518	NA	UKB-b-57	TRUE	reported	8KoqAI
17	44359663	G	T	0.024277	0.003939	7.10E-10	0.20327	462346	NA	rs3612399	NA	UKB-b-57	TRUE	reported	8KoqAI
17	44360941	T	C	0.024836	0.004081	1.20E-09	0.192469	462346	NA	rs2532317	NA	UKB-b-57	TRUE	reported	8KoqAI
17	44363929	C	T	0.022938	0.003705	6.00E-10	0.222121	462346	NA	rs2696500	NA	UKB-b-57	TRUE	reported	8KoqAI
17	44364056	T	C	0.02376	0.004022	3.50E-09	0.192838	462346	NA	rs2732615	NA	UKB-b-57	TRUE	reported	8KoqAI
17	44364209	C	T	0.023632	0.004024	4.30E-09	0.192657	462346	NA	rs2732614	NA	UKB-b-57	TRUE	reported	8KoqAI
17	44364573	G	A	0.024167	0.004178	7.30E-09	0.182153	462346	NA	rs1133015	NA	UKB-b-57	TRUE	reported	8KoqAI
17	44364584	C	T	0.024686	0.004089	1.60E-09	0.192378	462346	NA	rs2696498	NA	UKB-b-57	TRUE	reported	8KoqAI
17	44364649	C	T	0.023081	0.003706	4.70E-10	0.221907	462346	NA	rs2696497	NA	UKB-b-57	TRUE	reported	8KoqAI
17	44366572	A	G	0.023835	0.004066	4.60E-09	0.189967	462346	NA	rs2732607	NA	UKB-b-57	TRUE	reported	8KoqAI
17	44368212	C	A	0.023655	0.004024	4.20E-09	0.192639	462346	NA	rs2957297	NA	UKB-b-57	TRUE	reported	8KoqAI
17	44368215	C	G	0.024732	0.00416	2.80E-09	0.18743	462346	NA	rs2732600	NA	UKB-b-57	TRUE	reported	8KoqAI
17	44368216	G	A	0.024373	0.004168	5.00E-09	0.187014	462346	NA	rs2732599	NA	UKB-b-57	TRUE	reported	8KoqAI
17	44515243	C	T	0.022739	0.003649	4.60E-10	0.245704	462346	NA	rs199491	NA	UKB-b-57	TRUE	reported	8KoqAI
17	44753025	C	T	-0.02021	0.003614	2.20E-08	0.767143	462346	NA	rs199470	NA	UKB-b-57	TRUE	reported	8KoqAI
17	44764775	A	C	0.023214	0.003936	3.70E-09	0.198784	462346	NA	rs199460	NA	UKB-b-57	TRUE	reported	8KoqAI
17	44773783	A	G	-0.02354	0.003678	1.60E-10	0.783353	462346	NA	rs199441	NA	UKB-b-57	TRUE	reported	8KoqAI
17	44787312	C	T	0.022784	0.003686	6.30E-10	0.21426	462346	NA	rs1378358	NA	UKB-b-57	TRUE	reported	8KoqAI
17	44787313	G	C	0.023109	0.003686	3.60E-10	0.214	462346	NA	rs538628	NA	UKB-b-57	TRUE	reported	8KoqAI

17	44790203	A	G	0.023041	0.003686	4.10E-10	0.213979	462346	NA	rs169201	NA	UKB-b-57	TRUE	reported	8KoqAI
17	44793503	A	G	0.023096	0.003686	3.70E-10	0.214009	462346	NA	rs199439	NA	UKB-b-57	TRUE	reported	8KoqAI
17	44795469	C	T	0.022978	0.003686	4.60E-10	0.213943	462346	NA	rs199457	NA	UKB-b-57	TRUE	reported	8KoqAI
17	44797919	C	T	0.022913	0.003688	5.20E-10	0.213731	462346	NA	rs199456	NA	UKB-b-57	TRUE	reported	8KoqAI
17	44801784	G	A	0.023073	0.003687	3.90E-10	0.213923	462346	NA	rs199451	NA	UKB-b-57	TRUE	reported	8KoqAI
17	44809001	A	G	0.02301	0.00369	4.50E-10	0.213632	462346	NA	rs199448	NA	UKB-b-57	TRUE	reported	8KoqAI
17	44812188	C	T	0.023051	0.003691	4.20E-10	0.213618	462346	NA	rs199447	NA	UKB-b-57	TRUE	reported	8KoqAI
17	44817408	C	T	0.023303	0.00369	2.70E-10	0.213733	462346	NA	rs199445	NA	UKB-b-57	TRUE	reported	8KoqAI
17	44819565	C	T	0.023296	0.003689	2.70E-10	0.213935	462346	NA	rs199443	NA	UKB-b-57	TRUE	reported	8KoqAI
17	44822662	A	G	0.023293	0.003689	2.70E-10	0.213908	462346	NA	rs199535	NA	UKB-b-57	TRUE	reported	8KoqAI
17	44824213	T	G	0.023243	0.00369	3.00E-10	0.213773	462346	NA	rs199534	NA	UKB-b-57	TRUE	reported	8KoqAI
17	44826056	C	G	0.023604	0.003682	1.50E-10	0.215209	462346	NA	rs9896243	NA	UKB-b-57	TRUE	reported	8KoqAI
17	44828931	G	A	0.023067	0.003679	3.60E-10	0.215091	462346	NA	rs199533	NA	UKB-b-57	TRUE	reported	8KoqAI
17	44843136	C	T	0.022043	0.003689	2.30E-09	0.214769	462346	NA	rs199528	NA	UKB-b-57	TRUE	reported	8KoqAI
17	44847707	C	G	-0.02133	0.00364	4.70E-09	0.776921	462346	NA	rs199526	NA	UKB-b-57	TRUE	reported	8KoqAI
17	44847834	T	G	0.022051	0.003685	2.20E-09	0.215429	462346	NA	rs199525	NA	UKB-b-57	TRUE	reported	8KoqAI
17	44856485	C	T	-0.02227	0.003674	1.40E-09	0.783942	462346	NA	rs199516	NA	UKB-b-57	TRUE	reported	8KoqAI
17	44856641	G	C	-0.02213	0.003674	1.70E-09	0.783939	462346	NA	rs199515	NA	UKB-b-57	TRUE	reported	8KoqAI
17	44856881	G	A	-0.02228	0.003674	1.30E-09	0.783843	462346	NA	rs199514	NA	UKB-b-57	TRUE	reported	8KoqAI
17	44856932	A	G	-0.02227	0.003674	1.30E-09	0.783843	462346	NA	rs199513	NA	UKB-b-57	TRUE	reported	8KoqAI
17	44857352	T	C	-0.02227	0.003675	1.40E-09	0.78397	462346	NA	rs199512	NA	UKB-b-57	TRUE	reported	8KoqAI
17	44858728	G	A	-0.02223	0.003675	1.50E-09	0.783949	462346	NA	rs199509	NA	UKB-b-57	TRUE	reported	8KoqAI
17	44858855	A	G	-0.02222	0.003675	1.50E-09	0.783951	462346	NA	rs199507	NA	UKB-b-57	TRUE	reported	8KoqAI
17	44859031	A	G	-0.02236	0.003676	1.20E-09	0.784038	462346	NA	rs199506	NA	UKB-b-57	TRUE	reported	8KoqAI
17	44859144	C	T	-0.02217	0.003675	1.60E-09	0.783967	462346	NA	rs415430	NA	UKB-b-57	TRUE	reported	8KoqAI
17	44859148	T	C	-0.02217	0.003675	1.60E-09	0.783967	462346	NA	rs430685	NA	UKB-b-57	TRUE	reported	8KoqAI
17	44859410	A	G	-0.02222	0.003678	1.50E-09	0.784142	462346	NA	rs199505	NA	UKB-b-57	TRUE	reported	8KoqAI
17	44859715	T	C	-0.02211	0.003678	1.90E-09	0.784197	462346	NA	rs70602	NA	UKB-b-57	TRUE	reported	8KoqAI
17	44860021	C	T	0.021927	0.003681	2.60E-09	0.215328	462346	NA	rs70600	NA	UKB-b-57	TRUE	reported	8KoqAI
17	44861003	C	T	-0.02226	0.003673	1.40E-09	0.782752	462346	NA	rs199504	NA	UKB-b-57	TRUE	reported	8KoqAI
17	44862347	G	A	0.020682	0.003705	2.40E-08	0.215384	462346	NA	rs199502	NA	UKB-b-57	TRUE	reported	8KoqAI
17	44865498	C	T	0.02339	0.003986	4.40E-09	0.205688	462346	NA	rs199499	NA	UKB-b-57	TRUE	reported	8KoqAI
17	44865603	T	C	0.022377	0.003855	6.50E-09	0.221037	462346	NA	rs199498	NA	UKB-b-57	TRUE	reported	8KoqAI
17	57608214	A	G	0.020483	0.003688	2.80E-08	0.213952	462346	NA	rs7210832	NA	UKB-b-57	TRUE	reported	8KoqAI
17	57611219	G	C	0.020673	0.003688	2.10E-08	0.21408	462346	NA	rs9906665	NA	UKB-b-57	TRUE	reported	8KoqAI
17	57615392	C	T	0.020716	0.003706	2.30E-08	0.211798	462346	NA	rs7210881	NA	UKB-b-57	TRUE	reported	8KoqAI
17	57616940	G	T	0.020576	0.003711	2.90E-08	0.210473	462346	NA	rs9911670	NA	UKB-b-57	TRUE	reported	8KoqAI
17	57618151	T	C	0.020663	0.003708	2.50E-08	0.210823	462346	NA	rs9893638	NA	UKB-b-57	TRUE	reported	8KoqAI
17	57620880	G	A	0.020771	0.003686	1.70E-08	0.214357	462346	NA	rs7210390	NA	UKB-b-57	TRUE	reported	8KoqAI
17	57622689	C	T	0.020633	0.003709	2.60E-08	0.210804	462346	NA	rs9896884	NA	UKB-b-57	TRUE	reported	8KoqAI
17	57623037	C	T	0.020652	0.003709	2.60E-08	0.210808	462346	NA	rs2014756	NA	UKB-b-57	TRUE	reported	8KoqAI
17	57623664	A	T	0.020663	0.003709	2.50E-08	0.210819	462346	NA	rs6675966	NA	UKB-b-57	TRUE	reported	8KoqAI
17	57625123	G	A	0.020772	0.003686	1.70E-08	0.214435	462346	NA	rs7217180	NA	UKB-b-57	TRUE	reported	8KoqAI
17	57625758	A	C	0.020688	0.003685	2.00E-08	0.214497	462346	NA	rs1919181	NA	UKB-b-57	TRUE	reported	8KoqAI
17	57627786	T	C	0.018793	0.003332	1.70E-08	0.292633	462346	NA	rs1232587	NA	UKB-b-57	TRUE	reported	8KoqAI
17	57636129	C	A	0.020721	0.003686	1.90E-08	0.214413	462346	NA	rs8066493	NA	UKB-b-57	TRUE	reported	8KoqAI
17	57637645	G	A	0.020703	0.003686	1.90E-08	0.214376	462346	NA	rs2014295	NA	UKB-b-57	TRUE	reported	8KoqAI
17	57637803	T	C	0.02064	0.003709	2.60E-08	0.210803	462346	NA	rs4968384	NA	UKB-b-57	TRUE	reported	8KoqAI
17	57639056	C	T	0.020724	0.003709	2.30E-08	0.210741	462346	NA	rs5615508	NA	UKB-b-57	TRUE	reported	8KoqAI
17	57640524	G	A	0.020755	0.003709	2.20E-08	0.21082	462346	NA	rs9899390	NA	UKB-b-57	TRUE	reported	8KoqAI
17	57642075	C	T	0.020844	0.003686	1.60E-08	0.214369	462346	NA	rs6503923	NA	UKB-b-57	TRUE	reported	8KoqAI
17	57647317	G	A	0.020848	0.003686	1.50E-08	0.214371	462346	NA	rs2333551	NA	UKB-b-57	TRUE	reported	8KoqAI
17	57648337	G	A	0.020843	0.003686	1.60E-08	0.214315	462346	NA	rs9909087	NA	UKB-b-57	TRUE	reported	8KoqAI
17	57665668	G	A	0.020993	0.003715	1.60E-08	0.212149	462346	NA	rs2697388	NA	UKB-b-57	TRUE	reported	8KoqAI
17	57680480	G	A	0.020829	0.003686	1.60E-08	0.214158	462346	NA	rs7209369	NA	UKB-b-57	TRUE	reported	8KoqAI
17	57680872	C	T	0.020687	0.003707	2.40E-08	0.210897	462346	NA	rs8066828	NA	UKB-b-57	TRUE	reported	8KoqAI
17	57681125	T	C	0.020872	0.003685	1.50E-08	0.214321	462346	NA	rs6503929	NA	UKB-b-57	TRUE	reported	8KoqAI
17	57691502	A	G	0.020993	0.003686	1.20E-08	0.214389	462346	NA	rs9913684	NA	UKB-b-57	TRUE	reported	8KoqAI
17	57691607	G	T	0.018994	0.003339	1.30E-08	0.291961	462346	NA	rs9914526	NA	UKB-b-57	TRUE	reported	8KoqAI
17	57692689	A	G	0.020704	0.003708	2.40E-08	0.210783	462346	NA	rs2856458	NA	UKB-b-57	TRUE	reported	8KoqAI
17	57696827	A	G	-0.02051	0.003537	6.60E-09	0.757916	462346	NA	rs3803862	NA	UKB-b-57	TRUE	reported	8KoqAI
17	57699229	C	G	-0.0192	0.003238	3.00E-09	0.672311	462346	NA	rs1528491	NA	UKB-b-57	TRUE	reported	8KoqAI
17	57702853	T	G	-0.02061	0.00353	5.30E-09	0.758209	462346	NA	rs9905730	NA	UKB-b-57	TRUE	reported	8KoqAI
17	57713280	A	G	-0.02029	0.003529	8.90E-09	0.757825	462346	NA	rs9891822	NA	UKB-b-57	TRUE	reported	8KoqAI
17	57713872	G	A	-0.02038	0.003528	7.60E-09	0.75772	462346	NA	rs8074079	NA	UKB-b-57	TRUE	reported	8KoqAI
17	57715911	C	T	-0.02037	0.003528	7.70E-09	0.757743	462346	NA	rs1534550	NA	UKB-b-57	TRUE	reported	8KoqAI
17	57717911	C	T	-0.0204	0.003528	7.30E-09	0.757749	462346	NA	rs1085301	NA	UKB-b-57	TRUE	reported	8KoqAI
17	57728344	C	T	-0.02033	0.003528	8.30E-09	0.7577	462346	NA	rs4968389	NA	UKB-b-57	TRUE	reported	8KoqAI
17	57730189	A	G	-0.01972	0.003581	3.70E-08	0.767191	462346	NA	rs8069123	NA	UKB-b-57	TRUE	reported	8KoqAI
17	57730565	T	A	-0.02023	0.003529	1.00E-08	0.757858	462346	NA	rs8071030	NA	UKB-b-57	TRUE	reported	8KoqAI
17	57733798	A	G	-0.02026	0.003529	9.50E-09	0.757834	462346	NA	rs1107938	NA	UKB-b-57	TRUE	reported	8KoqAI
17	57734066	G	A	-0.02014	0.003529	1.20E-08	0.75797	462346	NA	rs8071796	NA	UKB-b-57	TRUE	reported	8KoqAI
17	57735565	A	G	-0.02011	0.003529	1.20E-08	0.75793	462346	NA	rs9898835	NA	UKB-b-57	TRUE	reported	8KoqAI
17	57739267	C	T	-0.02028	0.003528	9.10E-09	0.757694	462346	NA	rs1187200	NA	UKB-b-57	TRUE	reported	8KoqAI
17	57740075	C	T	-0.02013	0.003529	1.20E-08	0.758006	462346	NA	rs907066	NA	UKB-b-57	TRUE	reported	8KoqAI
17	57756318	G	A	-0.02011	0.003529	1.20E-08	0.757921	462346	NA	rs9913835	NA	UKB-b-57	TRUE	reported	8KoqAI
17	57757689	T	A	-0.02043	0.003528	7.00E-09	0.757557	462346	NA	rs1165800	NA	UKB-b-57	TRUE	reported	8KoqAI
17	57766975	T	C	-0.02015	0.00353	1.10E-08	0.757793	462346	NA	rs1554846	NA	UKB-b-57	TRUE	reported	8KoqAI
17	57769998	A	G	-0.01898	0.003233	4.30E-09	0.677165	462346	NA	rs3539126	NA	UKB-b-57	TRUE	reported	8KoqAI
17	57772814	C	T	-0.02016	0.003528	1.10E-08	0.757639	462346	NA	rs1085301	NA	UKB-b-57	TRUE	reported	8KoqAI
17	57776597	T	G	-0.02046	0.003528	6.60E-09	0.757592	462346	NA	rs9652857	NA	UKB-b-57	TRUE	reported	8KoqAI
17	57780943	G	T	-0.01927	0.003227	2.30E-09	0.674892	462346	NA	rs4968391	NA	UKB-b-57	TRUE	reported	8KoqAI
17	57791844	A	G	-0.01915	0.003225	2.90E-09	0.674753	462346	NA	rs1294723	NA	UKB-b-57	TRUE	reported	8KoqAI
18	21022448	G	T	-0.01854	0.003177	5.30E-09	0.349788	462346	NA	rs9962848	NA	UKB-b-57	TRUE	reported	8KoqAI
18	21023463	G	A	-0.01854	0.003176	5.30E-09	0.349792	462346	NA	rs2337067	NA	UKB-b-57	TRUE	reported	8KoqAI
18	21024298	G	C	-0.01855	0.003176	5.20E-09	0.3498	462346	NA	rs2878881	NA	UKB-b-57	TRUE	reported	8KoqAI
18	21035598	G	A	-0.01765	0.003177	2.70E-08	0.349646	462346	NA	rs1260796	NA	UKB-b-57	TRUE	reported	8KoqAI
18	21043466	C	T	-0.01788	0.003178	1.80E-08	0.348771	462346	NA	rs1875410	NA	UKB-b-57	TRUE	reported	8KoqAI
18	21044607	G	A	-0.01786	0.003178	1.90E-08	0.349045	462346	NA	rs2291993	NA	UKB-b-57	TRUE	reported	8KoqAI
18	21045557	G	A	-0.01792	0.003177	1.70E-08	0.348984	462346	NA	rs9957219	NA	UKB-b-57	TRUE	reported	8KoqAI
18	21049202	A	C	-0.01784	0.003176	1.90E-08	0.349149	462346	NA	rs1166336	NA	UKB-b-57	TRUE	reported	8KoqAI
18	21049512	C	T	-0.01784	0.003176	1.90E-08	0.349149	462346	NA	rs1295848	NA	UKB-b-57	TRUE	reported	8KoqAI
18	21049872	A	G	-0.01785	0.003176	1.90E-08	0.34915	462346	NA	rs8088717	NA	UKB-b-57	TRUE	reported	8KoqAI
18	21053204	A	T	-0.0175	0.003161	3.10E-08	0.356322	462346	NA	rs7244132	NA	UKB-b-57	TRUE	reported	8KoqAI
18	21056245	T	A	-0.01737	0.003156	3.70E-08	0.358259	462346	NA	rs9967555	NA	UKB-b-57	TRUE	reported	8KoqAI
18	21057450	A	G	-0.0174	0.003156	3.50E-08	0.358525	462346	NA	rs7227951	NA	UKB-b-57	TRUE	reported	8KoqAI
18	21067076	A	G	-0.01944	0.003195	1.20E-09	0.349229	462346	NA	rs1166364	NA	UKB-b-57	TRUE	reported	8KoqAI
18	21068118	A	G	-0.01963	0.003196	8.20E-10	0.348562	462346	NA	rs1187714	NA	UKB-b-57	TRUE	reported	8KoqAI
18	21068987	C	T	-0.01956	0.003197	9.40E-10	0.348419	462346	NA	rs6209588	NA	UKB-b-57	TRUE	reported	8KoqAI
18	21069068	G	A	-0.01957	0.003198	9.30E-10	0.348299	462346	NA	rs6209588	NA	UKB-b-57	TRUE	reported	8KoqAI
18	21069137	G	T	-0.0196	0.003198	8.90E-10	0.348311	462346	NA	rs6209589	NA	UKB-b-57	TRUE	reported	8KoqAI
18	21071440	A	C	-0.01962	0.003215	1.00E-09	0.3476	462346	NA	rs1295410	NA	UKB-b-57	TRUE	reported	8KoqAI
18	21071895	G	A	-0.01965	0.00322	1.10E-09	0.347086	462346	NA	rs1187814	NA	UKB-b-57	TRUE	reported	8KoqAI
18	21071925	A	G	-0.01963	0.00322	1.10E-09	0.347184	462346	NA	rs1187596	NA	UKB-b-57	TRUE	reported	8KoqAI
18	21074255	G	A	0.021071	0.003124	1.50E-11	0.408407	462346	NA	rs303752	NA	UKB-b-57	TRUE	reported	8KoqAI
18	21074922	G	A	0.028621	0.003209	4.70E-19	0.346398	462346	NA	rs303753	NA	UKB-b-57	TRUE	reported	8KoqAI
18	21075441	C	T	0.028696	0.003058	6.40E-21	0.541515	462346	NA	rs167336	NA	UKB-b-57	TRUE	reported	8KoqAI

18	21075938	A	G	-0.029	0.003058	2.50E-21	0.457953	462346	NA	rs3483314	NA	UKB-b-57	TRUE	reported	8KoqAI
18	21076446	A	G	-0.02901	0.003057	2.40E-21	0.457924	462346	NA	rs1187350	NA	UKB-b-57	TRUE	reported	8KoqAI
18	21076764	G	T	0.028437	0.003054	1.30E-20	0.534273	462346	NA	rs303754	NA	UKB-b-57	TRUE	reported	8KoqAI
18	21077259	A	C	-0.02902	0.003062	2.60E-21	0.456996	462346	NA	rs3438579	NA	UKB-b-57	TRUE	reported	8KoqAI
18	21077269	G	A	-0.02901	0.00306	2.50E-21	0.455881	462346	NA	rs3486551	NA	UKB-b-57	TRUE	reported	8KoqAI
18	21078716	T	G	0.028707	0.003198	2.80E-19	0.34565	462346	NA	rs303757	NA	UKB-b-57	TRUE	reported	8KoqAI
18	21079363	T	C	0.028852	0.003052	3.30E-21	0.542828	462346	NA	rs182498	NA	UKB-b-57	TRUE	reported	8KoqAI
18	21079654	T	C	-0.02804	0.003082	9.00E-20	0.422279	462346	NA	rs1367085	NA	UKB-b-57	TRUE	reported	8KoqAI
18	21080859	C	A	-0.02933	0.003053	7.60E-22	0.459939	462346	NA	rs1893659	NA	UKB-b-57	TRUE	reported	8KoqAI
18	21082317	T	C	0.028472	0.003193	4.80E-19	0.345836	462346	NA	rs303762	NA	UKB-b-57	TRUE	reported	8KoqAI
18	21082381	T	C	0.028816	0.003049	3.40E-21	0.543196	462346	NA	rs303761	NA	UKB-b-57	TRUE	reported	8KoqAI
18	21083275	A	G	-0.02894	0.003047	2.20E-21	0.456873	462346	NA	rs4800487	NA	UKB-b-57	TRUE	reported	8KoqAI
18	21083738	C	T	0.028591	0.003189	3.10E-19	0.345697	462346	NA	rs303760	NA	UKB-b-57	TRUE	reported	8KoqAI
18	21085085	G	A	-0.02783	0.003034	4.60E-20	0.48696	462346	NA	rs7233671	NA	UKB-b-57	TRUE	reported	8KoqAI
18	21085355	T	G	-0.02797	0.003074	9.20E-20	0.421858	462346	NA	rs1296611	NA	UKB-b-57	TRUE	reported	8KoqAI
18	21085857	G	A	-0.02783	0.003034	4.70E-20	0.486984	462346	NA	rs7234958	NA	UKB-b-57	TRUE	reported	8KoqAI
18	21086216	A	C	-0.0278	0.003034	5.00E-20	0.486982	462346	NA	rs1429935	NA	UKB-b-57	TRUE	reported	8KoqAI
18	21087531	C	T	0.028639	0.003187	2.60E-19	0.345856	462346	NA	rs891389	NA	UKB-b-57	TRUE	reported	8KoqAI
18	21090023	A	G	-0.02755	0.003032	1.00E-19	0.494727	462346	NA	rs1788808	NA	UKB-b-57	TRUE	reported	8KoqAI
18	21091121	C	T	-0.02765	0.003033	7.90E-20	0.486895	462346	NA	rs6507705	NA	UKB-b-57	TRUE	reported	8KoqAI
18	21091212	A	T	-0.02847	0.003186	4.00E-19	0.653991	462346	NA	rs8094509	NA	UKB-b-57	TRUE	reported	8KoqAI
18	21094207	C	T	-0.0287	0.003042	4.00E-21	0.456716	462346	NA	rs1166407	NA	UKB-b-57	TRUE	reported	8KoqAI
18	21097460	A	G	-0.02759	0.003031	8.90E-20	0.494287	462346	NA	rs1788817	NA	UKB-b-57	TRUE	reported	8KoqAI
18	21099583	C	T	-0.02846	0.003183	3.90E-19	0.65407	462346	NA	rs1652342	NA	UKB-b-57	TRUE	reported	8KoqAI
18	21099831	A	C	-0.02851	0.003183	3.40E-19	0.654307	462346	NA	rs1652340	NA	UKB-b-57	TRUE	reported	8KoqAI
18	21100240	C	T	-0.02754	0.003029	9.60E-20	0.494429	462346	NA	rs1367083	NA	UKB-b-57	TRUE	reported	8KoqAI
18	21100504	G	A	-0.02851	0.003182	3.30E-19	0.654318	462346	NA	rs1788819	NA	UKB-b-57	TRUE	reported	8KoqAI
18	21101011	G	A	-0.0277	0.003029	5.90E-20	0.487186	462346	NA	rs2356683	NA	UKB-b-57	TRUE	reported	8KoqAI
18	21101456	C	A	-0.02771	0.003029	5.90E-20	0.487051	462346	NA	rs6507708	NA	UKB-b-57	TRUE	reported	8KoqAI
18	21101944	A	G	-0.02848	0.00318	3.40E-19	0.654175	462346	NA	rs1788820	NA	UKB-b-57	TRUE	reported	8KoqAI
18	21103909	T	C	-0.02751	0.003028	1.10E-19	0.494911	462346	NA	rs891387	NA	UKB-b-57	TRUE	reported	8KoqAI
18	21103971	T	G	-0.02882	0.003039	2.40E-21	0.457184	462346	NA	rs891386	NA	UKB-b-57	TRUE	reported	8KoqAI
18	21104888	C	T	-0.02739	0.003032	1.70E-19	0.481935	462346	NA	rs1808579	NA	UKB-b-57	TRUE	reported	8KoqAI
18	21108613	T	C	-0.02735	0.003038	2.20E-19	0.484517	462346	NA	rs5860974	NA	UKB-b-57	TRUE	reported	8KoqAI
18	21108614	G	A	-0.02729	0.003037	2.60E-19	0.484472	462346	NA	rs5744042	NA	UKB-b-57	TRUE	reported	8KoqAI
18	21109250	T	C	-0.02778	0.003185	2.70E-18	0.653366	462346	NA	rs1788825	NA	UKB-b-57	TRUE	reported	8KoqAI
18	21109269	T	C	-0.02822	0.003186	8.20E-19	0.654368	462346	NA	rs1652375	NA	UKB-b-57	TRUE	reported	8KoqAI
18	21109466	G	T	-0.02581	0.003037	1.90E-17	0.462274	462346	NA	rs1652376	NA	UKB-b-57	TRUE	reported	8KoqAI
18	21113285	T	C	-0.02746	0.00303	1.30E-19	0.489708	462346	NA	rs2510344	NA	UKB-b-57	TRUE	reported	8KoqAI
18	21114917	A	G	-0.02457	0.003192	1.40E-14	0.660984	462346	NA	rs1788823	NA	UKB-b-57	TRUE	reported	8KoqAI
18	21114997	C	T	-0.02702	0.003051	8.30E-19	0.46237	462346	NA	rs2236707	NA	UKB-b-57	TRUE	reported	8KoqAI
18	21115060	A	G	-0.02785	0.003034	4.40E-20	0.488359	462346	NA	rs6507716	NA	UKB-b-57	TRUE	reported	8KoqAI
18	21116998	A	G	-0.02746	0.003028	1.20E-19	0.489921	462346	NA	rs1296468	NA	UKB-b-57	TRUE	reported	8KoqAI
18	21117419	G	T	-0.02747	0.003028	1.20E-19	0.489838	462346	NA	rs4800162	NA	UKB-b-57	TRUE	reported	8KoqAI
18	21117571	C	A	-0.02746	0.003028	1.20E-19	0.489927	462346	NA	rs4800488	NA	UKB-b-57	TRUE	reported	8KoqAI
18	21118025	G	C	-0.02739	0.003028	1.50E-19	0.490451	462346	NA	rs1166509	NA	UKB-b-57	TRUE	reported	8KoqAI
18	21119291	A	G	-0.02754	0.003029	9.50E-20	0.49045	462346	NA	rs6507720	NA	UKB-b-57	TRUE	reported	8KoqAI
18	21119777	G	A	-0.02752	0.003028	1.00E-19	0.489455	462346	NA	rs1140458	NA	UKB-b-57	TRUE	reported	8KoqAI
18	21120035	T	C	-0.02746	0.003028	1.20E-19	0.490541	462346	NA	rs7239575	NA	UKB-b-57	TRUE	reported	8KoqAI
18	21120444	T	C	-0.02754	0.003028	9.40E-20	0.490014	462346	NA	rs1805082	NA	UKB-b-57	TRUE	reported	8KoqAI
18	21120869	A	G	-0.02823	0.00318	6.70E-19	0.653741	462346	NA	rs1788821	NA	UKB-b-57	TRUE	reported	8KoqAI
18	21124945	C	G	-0.0282	0.003179	7.30E-19	0.653493	462346	NA	rs1788799	NA	UKB-b-57	TRUE	reported	8KoqAI
18	21125942	A	G	-0.0283	0.00318	5.50E-19	0.653766	462346	NA	rs4800489	NA	UKB-b-57	TRUE	reported	8KoqAI
18	21126081	A	C	-0.02754	0.003028	9.40E-20	0.495212	462346	NA	rs4800490	NA	UKB-b-57	TRUE	reported	8KoqAI
18	21126952	C	T	-0.02759	0.003029	8.30E-20	0.493877	462346	NA	rs1618725	NA	UKB-b-57	TRUE	reported	8KoqAI
18	21127910	C	T	-0.0276	0.003032	8.80E-20	0.486265	462346	NA	rs2435307	NA	UKB-b-57	TRUE	reported	8KoqAI
18	21128857	T	C	-0.0275	0.003031	1.20E-19	0.496618	462346	NA	rs1652344	NA	UKB-b-57	TRUE	reported	8KoqAI
18	21130634	C	T	-0.02834	0.003181	5.20E-19	0.653987	462346	NA	rs1788763	NA	UKB-b-57	TRUE	reported	8KoqAI
18	21131929	T	C	-0.02735	0.003028	1.70E-19	0.495489	462346	NA	rs1652343	NA	UKB-b-57	TRUE	reported	8KoqAI
18	21132571	G	C	-0.02833	0.003181	5.30E-19	0.654045	462346	NA	rs1788762	NA	UKB-b-57	TRUE	reported	8KoqAI
18	21133084	A	G	-0.02744	0.003028	1.30E-19	0.495737	462346	NA	rs1788761	NA	UKB-b-57	TRUE	reported	8KoqAI
18	21133937	G	A	-0.02879	0.003038	2.60E-21	0.457464	462346	NA	rs1166355	NA	UKB-b-57	TRUE	reported	8KoqAI
18	21134239	C	T	-0.02744	0.003028	1.30E-19	0.495317	462346	NA	rs1631685	NA	UKB-b-57	TRUE	reported	8KoqAI
18	21137442	A	G	-0.02778	0.00322	6.30E-18	0.66722	462346	NA	rs1788760	NA	UKB-b-57	TRUE	reported	8KoqAI
18	21138865	T	C	-0.02838	0.003181	4.60E-19	0.653934	462346	NA	rs2472610	NA	UKB-b-57	TRUE	reported	8KoqAI
18	21140432	T	C	-0.02806	0.003069	6.10E-20	0.422917	462346	NA	rs1805081	NA	UKB-b-57	TRUE	reported	8KoqAI
18	21142340	T	C	-0.02834	0.003182	5.40E-19	0.654114	462346	NA	rs1788785	NA	UKB-b-57	TRUE	reported	8KoqAI
18	21142438	C	G	-0.02869	0.003039	3.70E-21	0.458397	462346	NA	rs2510340	NA	UKB-b-57	TRUE	reported	8KoqAI
18	21142734	A	T	-0.02834	0.003182	5.40E-19	0.654108	462346	NA	rs1652349	NA	UKB-b-57	TRUE	reported	8KoqAI
18	21142992	A	T	-0.02825	0.003183	6.90E-19	0.654203	462346	NA	rs1624695	NA	UKB-b-57	TRUE	reported	8KoqAI
18	21143183	C	T	-0.02741	0.003029	1.50E-19	0.49591	462346	NA	rs1623060	NA	UKB-b-57	TRUE	reported	8KoqAI
18	21143739	T	G	-0.02736	0.003029	1.70E-19	0.496193	462346	NA	rs2960578	NA	UKB-b-57	TRUE	reported	8KoqAI
18	21143932	T	C	-0.02856	0.003039	5.60E-21	0.458411	462346	NA	rs4800493	NA	UKB-b-57	TRUE	reported	8KoqAI
18	21146085	C	T	-0.02751	0.003031	1.10E-19	0.487651	462346	NA	rs1245726	NA	UKB-b-57	TRUE	reported	8KoqAI
18	21147509	C	T	-0.02731	0.00303	2.00E-19	0.494836	462346	NA	rs1652348	NA	UKB-b-57	TRUE	reported	8KoqAI
18	21154024	G	A	-0.02847	0.003183	3.70E-19	0.653951	462346	NA	rs1788826	NA	UKB-b-57	TRUE	reported	8KoqAI
18	21155861	G	A	-0.02863	0.00304	4.70E-21	0.457698	462346	NA	rs6145202	NA	UKB-b-57	TRUE	reported	8KoqAI
18	21156593	G	A	-0.02879	0.00304	2.80E-21	0.457576	462346	NA	rs8093440	NA	UKB-b-57	TRUE	reported	8KoqAI
18	21159630	A	G	-0.02854	0.003192	3.90E-19	0.65571	462346	NA	rs1788784	NA	UKB-b-57	TRUE	reported	8KoqAI
18	21161134	C	T	-0.02735	0.003035	2.10E-19	0.495276	462346	NA	rs1788783	NA	UKB-b-57	TRUE	reported	8KoqAI
18	21162288	C	T	-0.0269	0.00321	5.30E-17	0.662357	462346	NA	rs1429934	NA	UKB-b-57	TRUE	reported	8KoqAI
18	21164994	G	C	-0.02705	0.003047	6.80E-19	0.468862	462346	NA	rs1367084	NA	UKB-b-57	TRUE	reported	8KoqAI
18	21165080	C	A	-0.02623	0.003041	6.40E-18	0.498549	462346	NA	rs1788781	NA	UKB-b-57	TRUE	reported	8KoqAI
18	21165163	C	T	-0.02706	0.00322	4.40E-17	0.663811	462346	NA	rs1623003	NA	UKB-b-57	TRUE	reported	8KoqAI
18	21165409	T	C	-0.02702	0.003047	7.50E-19	0.468761	462346	NA	rs4800495	NA	UKB-b-57	TRUE	reported	8KoqAI
18	21167194	G	A	-0.01769	0.003129	1.60E-08	0.376716	462346	NA	rs2981422	NA	UKB-b-57	TRUE	reported	8KoqAI
18	21167742	T	A	-0.01725	0.003142	4.00E-08	0.369046	462346	NA	rs1788772	NA	UKB-b-57	TRUE	reported	8KoqAI
18	21171980	A	G	-0.01823	0.00327	2.50E-08	0.315258	462346	NA	rs9304355	NA	UKB-b-57	TRUE	reported	8KoqAI
18	21172017	T	C	-0.01877	0.003286	1.10E-08	0.310091	462346	NA	rs7234270	NA	UKB-b-57	TRUE	reported	8KoqAI
18	21173667	G	C	-0.01724	0.003163	5.00E-08	0.363202	462346	NA	rs1652358	NA	UKB-b-57	TRUE	reported	8KoqAI
18	22639237	C	T	0.020264	0.00357	1.40E-08	0.40703	462346	NA	rs5022348	NA	UKB-b-57	TRUE	reported	8KoqAI
18	22647270	C	T	0.016731	0.003065	4.80E-08	0.57026	462346	NA	rs4800204	NA	UKB-b-57	TRUE	reported	8KoqAI
18	38180077	T	C	0.023613	0.004248	2.70E-08	0.149702	462346	NA	rs2839121	NA	UKB-b-57	TRUE	reported	8KoqAI
18	38204609	T	C	0.024006	0.004244	1.50E-08	0.149866	462346	NA	rs9958066	NA	UKB-b-57	TRUE	reported	8KoqAI
18	38237592	C	T	0.024526	0.004291	1.10E-08	0.145946	462346	NA	rs9962501	NA	UKB-b-57	TRUE	reported	8KoqAI
18	38245134	A	G	0.024496	0.004291	1.10E-08	0.14597	462346	NA	rs7231635	NA	UKB-b-57	TRUE	reported	8KoqAI
18	38273129	A	G	0.024623	0.004293	9.70E-09	0.145876	462346	NA	rs2869401	NA	UKB-b-57	TRUE	reported	8KoqAI
18	38276860	T	C	0.024715	0.004295	8.70E-09	0.145899	462346	NA	rs1166461	NA	UKB-b-57	TRUE	reported	8KoqAI
18	38287433	A	C	0.02535	0.004292	3.50E-09	0.147123	462346	NA	rs9946719	NA	UKB-b-57	TRUE	reported	8KoqAI
18	38291210	G	A	0.025594	0.004284	2.30E-09	0.147471	462346	NA	rs1008408	NA	UKB-b-57	TRUE	reported	8KoqAI
18	38291689	C	T	0.025635	0.004285	2.20E-09	0.147389	462346	NA	rs9965910	NA	UKB-b-57	TRUE	reported	8KoqAI
18	38296736	C	T	0.025802	0.004285	1.70E-09	0.147383	462346	NA	rs9959195	NA	UKB-b-57	TRUE	reported	8KoqAI
18	38299067	G	A	0.025658	0.004285	2.10E-09	0.147401	462346	NA	rs2849826	NA	UKB-b-57	TRUE	reported	8KoqAI
18	38310893	A	T	0.025654	0.004284	2.10E-09	0.147424	462346	NA	rs1774905	NA	UKB-b-57	TRUE	reported	8KoqAI
18	38313195	C	T	0.026113	0.004327	1.60E-09	0.145599	462346	NA	rs2043677	NA	UKB-b-57	TRUE	reported	8KoqAI
18	38315529	C	G	0.025638	0.004284	2.20E-09	0.147507	462346	NA	rs1232622	NA	UKB-b-57	TRUE	reported	8KoqAI
18	38321055	T	G	0.025686	0.004284	2.00E-09	0.14744	462346	NA	rs4386211	NA	UKB-b-57	TRUE	reported	8KoqAI

18	38323994	C	T	0.0253	0.004289	3.70E-09	0.146949	462346	NA	rs1137177	NA	UKB-b-57	TRUE	reported	8KoqAI
18	38324847	G	T	0.025259	0.00429	3.90E-09	0.146888	462346	NA	rs1166346	NA	UKB-b-57	TRUE	reported	8KoqAI
18	38328207	G	A	0.025224	0.004289	4.10E-09	0.146934	462346	NA	rs2163161	NA	UKB-b-57	TRUE	reported	8KoqAI
18	38335298	A	G	0.024324	0.004254	1.10E-08	0.151099	462346	NA	rs8097675	NA	UKB-b-57	TRUE	reported	8KoqAI
18	40703580	C	T	-0.01681	0.003078	4.70E-08	0.418707	462346	NA	rs8083110	NA	UKB-b-57	TRUE	reported	8KoqAI
18	50630791	C	G	0.018262	0.00314	6.00E-09	0.375088	462346	NA	rs5632730	NA	UKB-b-57	TRUE	reported	8KoqAI
18	50635119	G	A	0.018108	0.003103	5.40E-09	0.39667	462346	NA	rs1165980	NA	UKB-b-57	TRUE	reported	8KoqAI
18	50697120	C	T	0.019146	0.003097	6.30E-10	0.397525	462346	NA	rs1748630	NA	UKB-b-57	TRUE	reported	8KoqAI
18	50709981	A	G	0.018527	0.003089	2.00E-09	0.403354	462346	NA	rs1740839	NA	UKB-b-57	TRUE	reported	8KoqAI
18	50718314	C	T	0.018635	0.003096	1.80E-09	0.397213	462346	NA	rs1748725	NA	UKB-b-57	TRUE	reported	8KoqAI
18	50718757	A	G	0.017411	0.003058	1.20E-08	0.432729	462346	NA	rs7232543	NA	UKB-b-57	TRUE	reported	8KoqAI
18	50718880	G	A	0.016688	0.003055	4.70E-08	0.43766	462346	NA	rs7233123	NA	UKB-b-57	TRUE	reported	8KoqAI
18	50718944	C	G	0.018231	0.003089	3.60E-09	0.403001	462346	NA	rs1748727	NA	UKB-b-57	TRUE	reported	8KoqAI
18	50721712	G	A	0.018244	0.003088	3.50E-09	0.403058	462346	NA	rs6209923	NA	UKB-b-57	TRUE	reported	8KoqAI
18	50723080	G	A	0.017768	0.003027	4.40E-09	0.505769	462346	NA	rs7230285	NA	UKB-b-57	TRUE	reported	8KoqAI
18	50723179	G	A	0.017787	0.003027	4.20E-09	0.505741	462346	NA	rs7230446	NA	UKB-b-57	TRUE	reported	8KoqAI
18	50723283	T	G	0.017661	0.003028	5.50E-09	0.505884	462346	NA	rs1748748	NA	UKB-b-57	TRUE	reported	8KoqAI
18	50723460	T	C	0.017108	0.003028	1.60E-08	0.510671	462346	NA	rs2069191	NA	UKB-b-57	TRUE	reported	8KoqAI
18	50724899	G	A	0.017141	0.003028	1.50E-08	0.510654	462346	NA	rs6209923	NA	UKB-b-57	TRUE	reported	8KoqAI
18	50725943	T	C	0.017418	0.003029	8.90E-09	0.511983	462346	NA	rs8084239	NA	UKB-b-57	TRUE	reported	8KoqAI
18	50726294	C	A	0.017965	0.003063	4.50E-09	0.426976	462346	NA	rs1022137	NA	UKB-b-57	TRUE	reported	8KoqAI
18	50726559	G	A	0.018074	0.003027	2.40E-09	0.507089	462346	NA	rs8084351	NA	UKB-b-57	TRUE	reported	8KoqAI
18	50726749	A	T	0.018092	0.003028	2.30E-09	0.507111	462346	NA	rs8084280	NA	UKB-b-57	TRUE	reported	8KoqAI
18	50727518	C	T	0.018825	0.003095	1.20E-09	0.397481	462346	NA	rs3794923	NA	UKB-b-57	TRUE	reported	8KoqAI
18	50727691	C	T	0.01881	0.003095	1.20E-09	0.397449	462346	NA	rs6209923	NA	UKB-b-57	TRUE	reported	8KoqAI
18	50729267	T	C	0.018353	0.003059	2.00E-09	0.428883	462346	NA	rs6093982	NA	UKB-b-57	TRUE	reported	8KoqAI
18	50730524	C	T	0.018202	0.003062	2.80E-09	0.428277	462346	NA	rs6209923	NA	UKB-b-57	TRUE	reported	8KoqAI
18	50730603	G	A	0.01821	0.003062	2.70E-09	0.428271	462346	NA	rs6209923	NA	UKB-b-57	TRUE	reported	8KoqAI
18	50731055	G	T	0.018023	0.003062	3.90E-09	0.428129	462346	NA	rs6209923	NA	UKB-b-57	TRUE	reported	8KoqAI
18	50731802	G	A	0.018211	0.003062	2.70E-09	0.428461	462346	NA	rs7227069	NA	UKB-b-57	TRUE	reported	8KoqAI
18	50735418	C	T	0.01887	0.003096	1.10E-09	0.398054	462346	NA	rs6210076	NA	UKB-b-57	TRUE	reported	8KoqAI
18	50737358	G	T	0.018631	0.003097	1.80E-09	0.39678	462346	NA	rs6210076	NA	UKB-b-57	TRUE	reported	8KoqAI
18	50738039	A	G	0.018639	0.003095	1.70E-09	0.397284	462346	NA	rs6210076	NA	UKB-b-57	TRUE	reported	8KoqAI
18	50739344	C	G	0.017798	0.003063	6.20E-09	0.426991	462346	NA	rs8086812	NA	UKB-b-57	TRUE	reported	8KoqAI
18	50739860	G	A	0.017552	0.003063	1.00E-08	0.426773	462346	NA	rs6210076	NA	UKB-b-57	TRUE	reported	8KoqAI
18	50740163	A	G	0.017548	0.003064	1.00E-08	0.42679	462346	NA	rs5590605	NA	UKB-b-57	TRUE	reported	8KoqAI
18	50741130	A	G	0.018464	0.003096	2.50E-09	0.396836	462346	NA	rs6210077	NA	UKB-b-57	TRUE	reported	8KoqAI
18	50741297	G	A	0.017558	0.003064	1.00E-08	0.426801	462346	NA	rs6210077	NA	UKB-b-57	TRUE	reported	8KoqAI
18	50745236	C	A	0.018945	0.003097	9.50E-10	0.397626	462346	NA	rs1166382	NA	UKB-b-57	TRUE	reported	8KoqAI
18	50746748	C	T	0.017016	0.003031	2.00E-08	0.512156	462346	NA	rs4632195	NA	UKB-b-57	TRUE	reported	8KoqAI
18	50747956	G	A	0.01695	0.00305	2.70E-08	0.446902	462346	NA	rs1367633	NA	UKB-b-57	TRUE	reported	8KoqAI
18	50748499	A	G	0.017872	0.003074	6.10E-09	0.417749	462346	NA	rs1050296	NA	UKB-b-57	TRUE	reported	8KoqAI
18	50754093	A	G	0.017272	0.003078	2.00E-08	0.414813	462346	NA	rs6210077	NA	UKB-b-57	TRUE	reported	8KoqAI
18	50757261	G	T	0.01775	0.003074	7.80E-09	0.41837	462346	NA	rs1748858	NA	UKB-b-57	TRUE	reported	8KoqAI
18	50760933	A	C	0.017872	0.003074	6.10E-09	0.418506	462346	NA	rs1748872	NA	UKB-b-57	TRUE	reported	8KoqAI
18	50762855	C	G	0.01791	0.003075	5.70E-09	0.418358	462346	NA	rs4456576	NA	UKB-b-57	TRUE	reported	8KoqAI
18	50776391	T	C	0.018921	0.003113	1.20E-09	0.38843	462346	NA	rs1741055	NA	UKB-b-57	TRUE	reported	8KoqAI
18	50778737	C	T	0.018245	0.003077	3.00E-09	0.418856	462346	NA	rs1750182	NA	UKB-b-57	TRUE	reported	8KoqAI
18	50779412	G	A	0.018073	0.003078	4.30E-09	0.418349	462346	NA	rs1431181	NA	UKB-b-57	TRUE	reported	8KoqAI
18	50788149	G	A	0.018412	0.003146	4.80E-09	0.404215	462346	NA	rs5710660	NA	UKB-b-57	TRUE	reported	8KoqAI
18	50792476	C	G	0.018152	0.003078	3.70E-09	0.419038	462346	NA	rs1741102	NA	UKB-b-57	TRUE	reported	8KoqAI
18	50795113	C	T	0.018178	0.003078	3.50E-09	0.419556	462346	NA	rs1741106	NA	UKB-b-57	TRUE	reported	8KoqAI
18	50796002	G	A	0.018235	0.003078	3.10E-09	0.418701	462346	NA	rs6209798	NA	UKB-b-57	TRUE	reported	8KoqAI
18	50796817	A	G	0.018363	0.003077	2.40E-09	0.419854	462346	NA	rs1431188	NA	UKB-b-57	TRUE	reported	8KoqAI
18	50801646	C	A	0.018305	0.00308	2.80E-09	0.417621	462346	NA	rs1166018	NA	UKB-b-57	TRUE	reported	8KoqAI
18	50807090	T	A	0.017674	0.003147	2.00E-08	0.373398	462346	NA	rs5602765	NA	UKB-b-57	TRUE	reported	8KoqAI
18	50807359	A	G	0.019338	0.003067	2.90E-10	0.428329	462346	NA	rs1741133	NA	UKB-b-57	TRUE	reported	8KoqAI
18	50810675	C	T	0.019071	0.003081	6.00E-10	0.418284	462346	NA	rs6209798	NA	UKB-b-57	TRUE	reported	8KoqAI
18	50812736	G	T	0.018475	0.003082	2.00E-09	0.416864	462346	NA	rs1166093	NA	UKB-b-57	TRUE	reported	8KoqAI
18	50818827	C	A	0.018574	0.003083	1.70E-09	0.416524	462346	NA	rs6209798	NA	UKB-b-57	TRUE	reported	8KoqAI
18	50819236	T	G	0.018592	0.003081	1.60E-09	0.416742	462346	NA	rs6209798	NA	UKB-b-57	TRUE	reported	8KoqAI
18	50822943	G	T	0.019796	0.003068	1.10E-10	0.425616	462346	NA	rs1741154	NA	UKB-b-57	TRUE	reported	8KoqAI
18	50824885	G	C	0.019686	0.003069	1.40E-10	0.425481	462346	NA	rs1108297	NA	UKB-b-57	TRUE	reported	8KoqAI
18	50826478	A	G	0.018758	0.00308	1.10E-09	0.416984	462346	NA	rs6209798	NA	UKB-b-57	TRUE	reported	8KoqAI
18	50827476	C	T	0.019863	0.003068	9.60E-11	0.425749	462346	NA	rs7292534	NA	UKB-b-57	TRUE	reported	8KoqAI
18	50829023	A	G	0.018646	0.00308	1.40E-09	0.416739	462346	NA	rs1187750	NA	UKB-b-57	TRUE	reported	8KoqAI
18	50832102	A	G	0.019857	0.003069	9.70E-11	0.426136	462346	NA	rs1431196	NA	UKB-b-57	TRUE	reported	8KoqAI
18	50835863	T	C	0.019955	0.003068	7.80E-11	0.426294	462346	NA	rs6209799	NA	UKB-b-57	TRUE	reported	8KoqAI
18	50836414	A	G	0.019965	0.003067	7.60E-11	0.426267	462346	NA	rs1187302	NA	UKB-b-57	TRUE	reported	8KoqAI
18	50840962	T	A	0.019898	0.003067	8.70E-11	0.423303	462346	NA	rs6209799	NA	UKB-b-57	TRUE	reported	8KoqAI
18	50843233	T	A	0.020002	0.003067	6.90E-11	0.423591	462346	NA	rs6209799	NA	UKB-b-57	TRUE	reported	8KoqAI
18	50843269	C	A	0.020001	0.003067	7.00E-11	0.423589	462346	NA	rs6209799	NA	UKB-b-57	TRUE	reported	8KoqAI
18	50845830	G	T	0.019989	0.003067	7.10E-11	0.423464	462346	NA	rs1346914	NA	UKB-b-57	TRUE	reported	8KoqAI
18	50847784	T	G	0.020002	0.003066	6.90E-11	0.423581	462346	NA	rs1166241	NA	UKB-b-57	TRUE	reported	8KoqAI
18	50850307	C	T	0.019918	0.003066	8.20E-11	0.423269	462346	NA	rs2047914	NA	UKB-b-57	TRUE	reported	8KoqAI
18	50851107	A	G	0.01996	0.003066	7.50E-11	0.423333	462346	NA	rs2043145	NA	UKB-b-57	TRUE	reported	8KoqAI
18	50854411	G	A	0.01979	0.003066	1.10E-10	0.423104	462346	NA	rs1979914	NA	UKB-b-57	TRUE	reported	8KoqAI
18	50857663	T	C	0.019922	0.003065	8.10E-11	0.423451	462346	NA	rs1750344	NA	UKB-b-57	TRUE	reported	8KoqAI
18	50858019	T	C	0.019902	0.003066	8.50E-11	0.423492	462346	NA	rs1504746	NA	UKB-b-57	TRUE	reported	8KoqAI
18	50862311	C	T	0.019625	0.003066	1.50E-10	0.423196	462346	NA	rs6209801	NA	UKB-b-57	TRUE	reported	8KoqAI
18	50863861	G	A	0.017983	0.003183	1.60E-08	0.360122	462346	NA	rs6209801	NA	UKB-b-57	TRUE	reported	8KoqAI
18	50869085	T	C	0.019743	0.003066	1.20E-10	0.423148	462346	NA	rs6209801	NA	UKB-b-57	TRUE	reported	8KoqAI
18	50872623	T	C	0.019633	0.003065	1.50E-10	0.423523	462346	NA	rs1166432	NA	UKB-b-57	TRUE	reported	8KoqAI
18	50875225	A	C	0.019559	0.003066	1.80E-10	0.422994	462346	NA	rs6209801	NA	UKB-b-57	TRUE	reported	8KoqAI
18	50882781	C	T	0.019589	0.003065	1.60E-10	0.423252	462346	NA	rs6209802	NA	UKB-b-57	TRUE	reported	8KoqAI
18	50884529	C	A	0.019568	0.003065	1.70E-10	0.423136	462346	NA	rs4405620	NA	UKB-b-57	TRUE	reported	8KoqAI
18	50886142	A	C	0.019636	0.003065	1.50E-10	0.423238	462346	NA	rs1741263	NA	UKB-b-57	TRUE	reported	8KoqAI
18	50886590	A	T	0.019605	0.003065	1.60E-10	0.42326	462346	NA	rs5603011	NA	UKB-b-57	TRUE	reported	8KoqAI
18	50887961	G	A	0.018879	0.003061	6.90E-10	0.428644	462346	NA	rs1050297	NA	UKB-b-57	TRUE	reported	8KoqAI
18	50888351	T	C	0.01965	0.003065	1.40E-10	0.423339	462346	NA	rs1741278	NA	UKB-b-57	TRUE	reported	8KoqAI
18	50888891	T	C	0.01964	0.003065	1.50E-10	0.423327	462346	NA	rs6209802	NA	UKB-b-57	TRUE	reported	8KoqAI
18	50889558	G	A	0.01964	0.003065	1.50E-10	0.423338	462346	NA	rs1504744	NA	UKB-b-57	TRUE	reported	8KoqAI
18	50894002	A	C	0.019622	0.003065	1.50E-10	0.423362	462346	NA	rs6209802	NA	UKB-b-57	TRUE	reported	8KoqAI
18	50895766	G	T	0.019632	0.003066	1.50E-10	0.423426	462346	NA	rs7227402	NA	UKB-b-57	TRUE	reported	8KoqAI
18	50902560	G	A	0.019864	0.003071	9.90E-11	0.422115	462346	NA	rs6209804	NA	UKB-b-57	TRUE	reported	8KoqAI
18	50902578	A	G	0.019866	0.003071	9.80E-11	0.422114	462346	NA	rs6209804	NA	UKB-b-57	TRUE	reported	8KoqAI
18	50902747	G	C	0.019774	0.003065	1.10E-10	0.423223	462346	NA	rs6209804	NA	UKB-b-57	TRUE	reported	8KoqAI
18	50902773	T	C	0.019774	0.003065	1.10E-10	0.423223	462346	NA	rs6209804	NA	UKB-b-57	TRUE	reported	8KoqAI
18	50903050	C	T	0.019729	0.003065	1.20E-10	0.423155	462346	NA	rs1750437	NA	UKB-b-57	TRUE	reported	8KoqAI
18	50904361	G	A	0.019732	0.003065	1.20E-10	0.423081	462346	NA	rs6209804	NA	UKB-b-57	TRUE	reported	8KoqAI
18	50904562	C	G	0.019735	0.003065	1.20E-10	0.423069	462346	NA	rs6209804	NA	UKB-b-57	TRUE	reported	8KoqAI
18	50904666	G	A	0.019801	0.003065	1.00E-10	0.423127	462346	NA	rs6209804	NA	UKB-b-57	TRUE	reported	8KoqAI
18	50907127	A	G	0.019455	0.00307	2.30E-10	0.422686	462346	NA	rs1166524	NA	UKB-b-57	TRUE	reported	8KoqAI
18	52858610	T	C	0.01724	0.003041	1.40E-08	0.499953	462346	NA	rs1787253	NA	UKB-b-57	TRUE	reported	8KoqAI
18	52859261	A	C	0.017105	0.00304	1.80E-08	0.499821	462346	NA	rs1125313	NA	UKB-b-57	TRUE	reported	8KoqAI
18	52903085	A	G	0.02981	0.005413	3.60E-08	0.914369	462346	NA	rs1261070	NA	UKB-b-57	TRUE	reported	8KoqAI
18	52982458	C	T	0.018497	0.00319	6.70E-09	0.352687	462346	NA	rs893946	NA	UKB-b-57	TRUE	reported	8KoqAI

18	53013947	C	T	0.018224	0.003087	3.60E-09	0.416986	462346	NA	rs1660237	NA	UKB-b-57	TRUE	reported	8KoqAI
18	53038676	A	T	0.018197	0.003092	4.00E-09	0.415108	462346	NA	rs1660242	NA	UKB-b-57	TRUE	reported	8KoqAI
18	53041903	A	G	0.017923	0.003251	3.50E-08	0.328159	462346	NA	rs1011392	NA	UKB-b-57	TRUE	reported	8KoqAI
18	53045198	C	T	0.019482	0.003062	2.00E-10	0.459949	462346	NA	rs1788030	NA	UKB-b-57	TRUE	reported	8KoqAI
18	53048678	A	C	0.017701	0.003106	1.20E-08	0.404511	462346	NA	rs1788025	NA	UKB-b-57	TRUE	reported	8KoqAI
18	53052169	C	T	0.017457	0.003113	2.10E-08	0.399115	462346	NA	rs9950000	NA	UKB-b-57	TRUE	reported	8KoqAI
18	53053897	A	G	0.017314	0.003112	2.60E-08	0.399372	462346	NA	rs9320010	NA	UKB-b-57	TRUE	reported	8KoqAI
18	53064491	G	A	0.017838	0.003064	5.80E-09	0.460496	462346	NA	rs2872041	NA	UKB-b-57	TRUE	reported	8KoqAI
18	53079067	A	G	0.017039	0.003109	4.20E-08	0.402767	462346	NA	rs2919446	NA	UKB-b-57	TRUE	reported	8KoqAI
18	53085412	T	C	0.018135	0.003063	3.20E-09	0.46162	462346	NA	rs2958163	NA	UKB-b-57	TRUE	reported	8KoqAI
18	53095471	T	C	-0.01723	0.00311	3.00E-08	0.597993	462346	NA	rs9320016	NA	UKB-b-57	TRUE	reported	8KoqAI
18	53103739	G	A	-0.01721	0.003113	3.30E-08	0.616513	462346	NA	rs4341827	NA	UKB-b-57	TRUE	reported	8KoqAI
18	53104253	A	T	-0.01855	0.00319	6.10E-09	0.656578	462346	NA	rs7228159	NA	UKB-b-57	TRUE	reported	8KoqAI
18	53107115	G	A	-0.01717	0.003114	3.50E-08	0.616231	462346	NA	rs7236656	NA	UKB-b-57	TRUE	reported	8KoqAI
18	53125435	G	A	-0.01951	0.00305	1.60E-10	0.539592	462346	NA	rs2924321	NA	UKB-b-57	TRUE	reported	8KoqAI
18	53198836	A	G	0.029493	0.00523	1.70E-08	0.906597	462346	NA	rs619466	NA	UKB-b-57	TRUE	reported	8KoqAI
18	53204864	C	T	0.030488	0.005442	2.10E-08	0.914781	462346	NA	rs611439	NA	UKB-b-57	TRUE	reported	8KoqAI
18	53210302	G	T	0.022328	0.003996	2.30E-08	0.825865	462346	NA	rs613872	NA	UKB-b-57	TRUE	reported	8KoqAI
18	53227648	A	G	0.030237	0.00546	3.10E-08	0.915198	462346	NA	rs584564	NA	UKB-b-57	TRUE	reported	8KoqAI
18	53273614	G	A	0.03051	0.005421	1.80E-08	0.913845	462346	NA	rs635538	NA	UKB-b-57	TRUE	reported	8KoqAI
18	53314500	T	A	-0.03115	0.005434	9.90E-09	0.085688	462346	NA	rs652749	NA	UKB-b-57	TRUE	reported	8KoqAI
18	53351484	G	A	0.030173	0.005418	2.60E-08	0.913434	462346	NA	rs784219	NA	UKB-b-57	TRUE	reported	8KoqAI
18	53397199	T	C	0.021274	0.0039	4.90E-08	0.812558	462346	NA	rs784257	NA	UKB-b-57	TRUE	reported	8KoqAI
18	53398626	G	A	0.021284	0.003894	4.60E-08	0.811752	462346	NA	rs784256	NA	UKB-b-57	TRUE	reported	8KoqAI
18	57732418	T	C	-0.0191	0.003441	2.80E-08	0.735045	462346	NA	rs4940926	NA	UKB-b-57	TRUE	reported	8KoqAI
18	57732689	A	G	-0.01893	0.003434	3.50E-08	0.733518	462346	NA	rs4940927	NA	UKB-b-57	TRUE	reported	8KoqAI
18	57733386	T	C	0.018822	0.003435	4.30E-08	0.266091	462346	NA	rs6819124	NA	UKB-b-57	TRUE	reported	8KoqAI
18	57733925	G	A	0.018801	0.003434	4.40E-08	0.266222	462346	NA	rs7228430	NA	UKB-b-57	TRUE	reported	8KoqAI
18	57734101	G	A	0.018806	0.003434	4.30E-08	0.266185	462346	NA	rs7228753	NA	UKB-b-57	TRUE	reported	8KoqAI
18	57734857	C	T	0.018776	0.003433	4.50E-08	0.266188	462346	NA	rs7234864	NA	UKB-b-57	TRUE	reported	8KoqAI
18	57735552	G	T	0.018865	0.003421	3.50E-08	0.268617	462346	NA	rs7238484	NA	UKB-b-57	TRUE	reported	8KoqAI
18	57735675	C	G	0.018795	0.003421	3.90E-08	0.268606	462346	NA	rs1296611	NA	UKB-b-57	TRUE	reported	8KoqAI
18	57735792	C	T	0.018799	0.00342	3.90E-08	0.268598	462346	NA	rs4479345	NA	UKB-b-57	TRUE	reported	8KoqAI
18	57735945	T	C	0.018654	0.00342	4.90E-08	0.268812	462346	NA	rs3603066	NA	UKB-b-57	TRUE	reported	8KoqAI
18	57736270	G	A	0.018803	0.00342	3.90E-08	0.268587	462346	NA	rs1942867	NA	UKB-b-57	TRUE	reported	8KoqAI
18	57738602	T	G	0.018789	0.003421	4.00E-08	0.268592	462346	NA	rs7241535	NA	UKB-b-57	TRUE	reported	8KoqAI
18	57739072	C	T	0.018824	0.003428	4.00E-08	0.267251	462346	NA	rs1166436	NA	UKB-b-57	TRUE	reported	8KoqAI
18	57739840	G	T	0.019026	0.003427	2.80E-08	0.267222	462346	NA	rs7231987	NA	UKB-b-57	TRUE	reported	8KoqAI
18	57742493	A	G	0.020781	0.003506	3.10E-09	0.248678	462346	NA	rs7298293	NA	UKB-b-57	TRUE	reported	8KoqAI
18	57744189	A	G	0.020696	0.003506	3.60E-09	0.248497	462346	NA	rs7240566	NA	UKB-b-57	TRUE	reported	8KoqAI
18	57744576	T	C	0.020749	0.003506	3.30E-09	0.248553	462346	NA	rs1152044	NA	UKB-b-57	TRUE	reported	8KoqAI
18	57745302	C	T	0.020716	0.003507	3.50E-09	0.248438	462346	NA	rs5564578	NA	UKB-b-57	TRUE	reported	8KoqAI
18	57747215	G	T	0.020679	0.003508	3.70E-09	0.248334	462346	NA	rs7298293	NA	UKB-b-57	TRUE	reported	8KoqAI
18	57747974	G	A	0.02073	0.003506	3.40E-09	0.248721	462346	NA	rs8092496	NA	UKB-b-57	TRUE	reported	8KoqAI
18	57749028	A	G	0.020836	0.003506	2.80E-09	0.24865	462346	NA	rs1706656	NA	UKB-b-57	TRUE	reported	8KoqAI
18	57749186	T	C	0.020845	0.003506	2.80E-09	0.248653	462346	NA	rs3557893	NA	UKB-b-57	TRUE	reported	8KoqAI
18	57750897	T	C	0.020816	0.003507	2.90E-09	0.248523	462346	NA	rs1706656	NA	UKB-b-57	TRUE	reported	8KoqAI
18	57751014	T	C	0.020813	0.003507	2.90E-09	0.248522	462346	NA	rs1295734	NA	UKB-b-57	TRUE	reported	8KoqAI
18	57751185	A	T	0.020817	0.003506	2.90E-09	0.248808	462346	NA	rs4299252	NA	UKB-b-57	TRUE	reported	8KoqAI
18	57751960	T	C	0.020994	0.003512	2.30E-09	0.247603	462346	NA	rs8091524	NA	UKB-b-57	TRUE	reported	8KoqAI
18	57752803	A	C	0.020726	0.003507	3.40E-09	0.248572	462346	NA	rs7237076	NA	UKB-b-57	TRUE	reported	8KoqAI
18	57754018	T	C	0.020875	0.003506	2.60E-09	0.248643	462346	NA	rs8084085	NA	UKB-b-57	TRUE	reported	8KoqAI
18	57754901	C	T	0.020915	0.003506	2.40E-09	0.248906	462346	NA	rs6567154	NA	UKB-b-57	TRUE	reported	8KoqAI
18	57755117	C	T	0.020966	0.003507	2.20E-09	0.248628	462346	NA	rs6567155	NA	UKB-b-57	TRUE	reported	8KoqAI
18	57756055	C	T	0.021047	0.003507	2.00E-09	0.248482	462346	NA	rs1942860	NA	UKB-b-57	TRUE	reported	8KoqAI
18	57757978	A	G	0.020588	0.003516	4.80E-09	0.246964	462346	NA	rs1166236	NA	UKB-b-57	TRUE	reported	8KoqAI
18	57761524	C	T	0.020937	0.003516	2.60E-09	0.247759	462346	NA	rs3591865	NA	UKB-b-57	TRUE	reported	8KoqAI
18	57766512	A	G	0.020676	0.003522	4.30E-09	0.24821	462346	NA	rs1539952	NA	UKB-b-57	TRUE	reported	8KoqAI
18	57780253	T	C	0.02077	0.003524	3.80E-09	0.24826	462346	NA	rs2856480	NA	UKB-b-57	TRUE	reported	8KoqAI
18	57780326	A	G	0.020563	0.003525	5.40E-09	0.247961	462346	NA	rs3411052	NA	UKB-b-57	TRUE	reported	8KoqAI
18	57783111	A	G	0.020819	0.003524	3.50E-09	0.248016	462346	NA	rs8092668	NA	UKB-b-57	TRUE	reported	8KoqAI
18	57797485	T	C	0.020852	0.00353	3.50E-09	0.247194	462346	NA	rs953442	NA	UKB-b-57	TRUE	reported	8KoqAI
18	57802714	G	A	0.020885	0.003615	7.60E-09	0.233016	462346	NA	rs7298298	NA	UKB-b-57	TRUE	reported	8KoqAI
18	57808978	C	A	0.021719	0.00361	1.80E-09	0.230034	462346	NA	rs6672316	NA	UKB-b-57	TRUE	reported	8KoqAI
18	57824038	C	G	0.022701	0.003617	3.50E-10	0.227841	462346	NA	rs7240682	NA	UKB-b-57	TRUE	reported	8KoqAI
18	57826570	T	C	0.022979	0.003619	2.20E-10	0.227505	462346	NA	rs1296787	NA	UKB-b-57	TRUE	reported	8KoqAI
18	57829135	T	C	0.02314	0.003582	1.10E-10	0.232738	462346	NA	rs6567160	NA	UKB-b-57	TRUE	reported	8KoqAI
18	57838401	G	A	0.022846	0.003577	1.70E-10	0.233413	462346	NA	rs663129	NA	UKB-b-57	TRUE	reported	8KoqAI
18	57839769	C	A	0.02304	0.003576	1.20E-10	0.233504	462346	NA	rs571312	NA	UKB-b-57	TRUE	reported	8KoqAI
18	57848369	A	T	0.022503	0.003573	3.00E-10	0.23423	462346	NA	rs523288	NA	UKB-b-57	TRUE	reported	8KoqAI
18	57848531	T	C	0.022478	0.003578	3.30E-10	0.233101	462346	NA	rs2168711	NA	UKB-b-57	TRUE	reported	8KoqAI
18	57848651	A	G	0.022456	0.003579	3.50E-10	0.232995	462346	NA	rs6692241	NA	UKB-b-57	TRUE	reported	8KoqAI
18	57849023	G	A	0.022483	0.003579	3.30E-10	0.233001	462346	NA	rs1296713	NA	UKB-b-57	TRUE	reported	8KoqAI
18	57849429	C	T	0.022534	0.003579	3.00E-10	0.233099	462346	NA	rs5808460	NA	UKB-b-57	TRUE	reported	8KoqAI
18	57850422	G	T	0.022767	0.003574	1.90E-10	0.233907	462346	NA	rs538656	NA	UKB-b-57	TRUE	reported	8KoqAI
18	57850583	C	A	0.02255	0.003578	2.90E-10	0.233065	462346	NA	rs1717551	NA	UKB-b-57	TRUE	reported	8KoqAI
18	57851097	T	C	0.022524	0.003578	3.10E-10	0.233128	462346	NA	rs1778231	NA	UKB-b-57	TRUE	reported	8KoqAI
18	57851763	A	G	0.021663	0.003563	1.20E-09	0.236085	462346	NA	rs1087177	NA	UKB-b-57	TRUE	reported	8KoqAI
18	57852587	T	C	0.021677	0.003558	1.10E-09	0.237246	462346	NA	rs476828	NA	UKB-b-57	TRUE	reported	8KoqAI
18	57852948	A	C	0.021779	0.003564	9.90E-10	0.236099	462346	NA	rs1115221	NA	UKB-b-57	TRUE	reported	8KoqAI
18	57854694	C	T	0.021751	0.003568	1.10E-09	0.235848	462346	NA	rs3463341	NA	UKB-b-57	TRUE	reported	8KoqAI
18	57858802	A	G	0.018864	0.003424	3.60E-08	0.268407	462346	NA	rs492443	NA	UKB-b-57	TRUE	reported	8KoqAI
18	57906500	G	A	0.018772	0.003435	4.60E-08	0.266464	462346	NA	rs656384	NA	UKB-b-57	TRUE	reported	8KoqAI
19	49103447	C	T	-0.01897	0.003098	9.10E-10	0.414147	462346	NA	rs411482	NA	UKB-b-57	TRUE	reported	8KoqAI
19	49104308	C	T	-0.01892	0.003099	1.00E-09	0.414	462346	NA	rs1236093	NA	UKB-b-57	TRUE	reported	8KoqAI
19	49114236	T	G	0.018695	0.003088	1.40E-09	0.582778	462346	NA	rs454715	NA	UKB-b-57	TRUE	reported	8KoqAI
19	49116076	C	G	0.018799	0.003075	9.80E-10	0.582414	462346	NA	rs2617801	NA	UKB-b-57	TRUE	reported	8KoqAI
19	49117247	A	G	0.018699	0.003073	1.20E-09	0.583282	462346	NA	rs374886	NA	UKB-b-57	TRUE	reported	8KoqAI
19	49168182	T	C	-0.01897	0.003226	4.10E-09	0.655329	462346	NA	rs373002	NA	UKB-b-57	TRUE	reported	8KoqAI
19	49168942	G	A	-0.02036	0.003079	3.80E-11	0.463961	462346	NA	rs8111874	NA	UKB-b-57	TRUE	reported	8KoqAI
19	49170068	G	A	-0.01887	0.003237	5.60E-09	0.661387	462346	NA	rs6782418	NA	UKB-b-57	TRUE	reported	8KoqAI
19	49171306	C	T	-0.02022	0.003076	4.90E-11	0.464549	462346	NA	rs1246211	NA	UKB-b-57	TRUE	reported	8KoqAI
19	49187669	G	A	-0.01912	0.003205	2.40E-09	0.642053	462346	NA	rs2098709	NA	UKB-b-57	TRUE	reported	8KoqAI
19	49201217	C	T	0.017246	0.003084	2.20E-08	0.43452	462346	NA	rs368565	NA	UKB-b-57	TRUE	reported	8KoqAI
19	49203829	C	T	-0.0184	0.003119	3.70E-09	0.462017	462346	NA	rs3510624	NA	UKB-b-57	TRUE	reported	8KoqAI
19	49206108	C	G	-0.01986	0.003024	5.10E-11	0.507885	462346	NA	rs679574	NA	UKB-b-57	TRUE	reported	8KoqAI
19	49206145	G	C	-0.01984	0.003024	5.30E-11	0.50789	462346	NA	rs516316	NA	UKB-b-57	TRUE	reported	8KoqAI
19	49206172	C	T	-0.0198	0.003024	5.80E-11	0.507883	462346	NA	rs516246	NA	UKB-b-57	TRUE	reported	8KoqAI
19	49206417	A	G	-0.01991	0.003024	4.50E-11	0.507801	462346	NA	rs492602	NA	UKB-b-57	TRUE	reported	8KoqAI
19	49206462	C	T	-0.01965	0.003023	8.10E-11	0.507877	462346	NA	rs681343	NA	UKB-b-57	TRUE	reported	8KoqAI
19	49206603	C	T	0.017238	0.003056	1.70E-08	0.430387	462346	NA	rs281377	NA	UKB-b-57	TRUE	reported	8KoqAI
19	49206674	G	A	-0.01964	0.003024	8.30E-11	0.507862	462346	NA	rs601338	NA	UKB-b-57	TRUE	reported	8KoqAI
19	49206985	G	A	-0.01942	0.003033	1.50E-10	0.53507	462346	NA	rs602662	NA	UKB-b-57	TRUE	reported	8KoqAI
19	49207206	A	G	-0.01959	0.003034	1.10E-10	0.536233	462346	NA	rs485186	NA	UKB-b-57	TRUE	reported	8KoqAI
19	49207255	A	G	-0.01985	0.00304	6.60E-11	0.534342	462346	NA	rs485073	NA	UKB-b-57	TRUE	reported	8KoqAI
19	49207257	T	C	-0.01989	0.00304	6.00E-11	0.534276	462346	NA	rs603985	NA	UKB-b-57	TRUE	reported	8KoqAI
19	49207554	C	T	-0.01922	0.003033	2.30E-10	0.534007	462346	NA	rs571689	NA	UKB-b-57	TRUE	reported	8KoqAI

19	49207651	T	C	-0.01924	0.003033	2.30E-10	0.534005	462346	NA	rs570794	NA	UKB-b-57	TRUE	reported	8KoqAI
19	49207718	T	C	-0.01924	0.003033	2.20E-10	0.534001	462346	NA	rs569970	NA	UKB-b-57	TRUE	reported	8KoqAI
19	49207792	G	A	-0.01945	0.003033	1.40E-10	0.53328	462346	NA	rs2251034	NA	UKB-b-57	TRUE	reported	8KoqAI
19	49208501	A	G	-0.01923	0.003037	2.40E-10	0.532721	462346	NA	rs507855	NA	UKB-b-57	TRUE	reported	8KoqAI
19	49208543	T	C	-0.01918	0.003037	2.70E-10	0.532668	462346	NA	rs507766	NA	UKB-b-57	TRUE	reported	8KoqAI
19	49208564	C	T	-0.01919	0.003037	2.60E-10	0.532693	462346	NA	rs507711	NA	UKB-b-57	TRUE	reported	8KoqAI
19	49208629	G	C	-0.01918	0.003033	2.50E-10	0.533861	462346	NA	rs506897	NA	UKB-b-57	TRUE	reported	8KoqAI
19	49208865	G	A	-0.01911	0.003033	3.00E-10	0.53365	462346	NA	rs504963	NA	UKB-b-57	TRUE	reported	8KoqAI
19	49208978	A	G	-0.0193	0.003033	2.00E-10	0.532925	462346	NA	rs632111	NA	UKB-b-57	TRUE	reported	8KoqAI
19	49209010	T	C	-0.01909	0.003033	3.10E-10	0.533865	462346	NA	rs503279	NA	UKB-b-57	TRUE	reported	8KoqAI
19	49209226	G	A	-0.01943	0.003033	1.50E-10	0.533186	462346	NA	rs633372	NA	UKB-b-57	TRUE	reported	8KoqAI
19	49209339	T	C	-0.01932	0.003034	1.90E-10	0.532893	462346	NA	rs2548459	NA	UKB-b-57	TRUE	reported	8KoqAI
19	49209464	C	A	-0.01874	0.003033	6.50E-10	0.532191	462346	NA	rs692854	NA	UKB-b-57	TRUE	reported	8KoqAI
19	49209566	A	G	-0.01903	0.003033	3.50E-10	0.531537	462346	NA	rs1704773	NA	UKB-b-57	TRUE	reported	8KoqAI
19	49209851	A	G	-0.01881	0.003034	5.70E-10	0.528193	462346	NA	rs646327	NA	UKB-b-57	TRUE	reported	8KoqAI
19	49211969	T	C	-0.01847	0.003032	1.10E-09	0.533218	462346	NA	rs676388	NA	UKB-b-57	TRUE	reported	8KoqAI
19	49213284	G	A	-0.01873	0.003035	6.80E-10	0.531315	462346	NA	rs584768	NA	UKB-b-57	TRUE	reported	8KoqAI
19	49213504	G	A	-0.01871	0.003035	7.10E-10	0.531231	462346	NA	rs2452170	NA	UKB-b-57	TRUE	reported	8KoqAI
19	49213833	G	A	-0.01871	0.003035	7.00E-10	0.531227	462346	NA	rs2638282	NA	UKB-b-57	TRUE	reported	8KoqAI
19	49214274	G	A	-0.01894	0.003031	4.10E-10	0.52323	462346	NA	rs281379	NA	UKB-b-57	TRUE	reported	8KoqAI
19	49215095	C	T	-0.01694	0.003047	2.70E-08	0.547379	462346	NA	rs4002471	NA	UKB-b-57	TRUE	reported	8KoqAI
19	49218060	C	T	-0.01953	0.003039	1.30E-10	0.497861	462346	NA	rs3586662	NA	UKB-b-57	TRUE	reported	8KoqAI
19	49218111	G	T	-0.01682	0.003049	3.40E-08	0.546956	462346	NA	rs3398810	NA	UKB-b-57	TRUE	reported	8KoqAI
19	49218430	A	G	-0.01685	0.003049	3.30E-08	0.546958	462346	NA	rs1297875	NA	UKB-b-57	TRUE	reported	8KoqAI
19	49218437	A	C	-0.01684	0.003049	3.30E-08	0.546969	462346	NA	rs1297875	NA	UKB-b-57	TRUE	reported	8KoqAI
19	49218602	C	T	-0.01726	0.003048	1.50E-08	0.545774	462346	NA	rs1297927	NA	UKB-b-57	TRUE	reported	8KoqAI
19	49219459	T	C	-0.01863	0.003045	9.40E-10	0.544574	462346	NA	rs660745	NA	UKB-b-57	TRUE	reported	8KoqAI
19	49220323	A	G	-0.0185	0.003045	1.20E-09	0.543663	462346	NA	rs1167290	NA	UKB-b-57	TRUE	reported	8KoqAI
19	49225766	A	T	-0.01901	0.003039	4.00E-10	0.541309	462346	NA	rs1188279	NA	UKB-b-57	TRUE	reported	8KoqAI
19	49227043	G	A	-0.01892	0.003034	4.50E-10	0.53614	462346	NA	rs1166679	NA	UKB-b-57	TRUE	reported	8KoqAI
19	49227256	C	T	-0.01891	0.003034	4.60E-10	0.536143	462346	NA	rs1297989	NA	UKB-b-57	TRUE	reported	8KoqAI
19	49227443	C	T	-0.019	0.003034	3.80E-10	0.536355	462346	NA	rs1187890	NA	UKB-b-57	TRUE	reported	8KoqAI
19	49228272	T	C	-0.01906	0.003033	3.30E-10	0.53628	462346	NA	rs2287921	NA	UKB-b-57	TRUE	reported	8KoqAI
19	49229323	G	A	-0.01877	0.003043	6.90E-10	0.553145	462346	NA	rs479486	NA	UKB-b-57	TRUE	reported	8KoqAI
19	49229525	T	C	-0.01914	0.003033	2.80E-10	0.536392	462346	NA	rs8112983	NA	UKB-b-57	TRUE	reported	8KoqAI
19	49232226	G	A	-0.01921	0.003034	2.40E-10	0.53527	462346	NA	rs2287922	NA	UKB-b-57	TRUE	reported	8KoqAI
19	49233406	C	A	0.017114	0.00307	2.50E-08	0.414602	462346	NA	rs281408	NA	UKB-b-57	TRUE	reported	8KoqAI
19	49234299	A	T	0.017036	0.003071	2.90E-08	0.414753	462346	NA	rs412712	NA	UKB-b-57	TRUE	reported	8KoqAI
19	49236102	T	C	-0.01872	0.003033	6.80E-10	0.530188	462346	NA	rs439523	NA	UKB-b-57	TRUE	reported	8KoqAI
19	49237547	G	A	0.017357	0.003073	1.60E-08	0.414844	462346	NA	rs1297914	NA	UKB-b-57	TRUE	reported	8KoqAI
19	49237552	A	C	0.017354	0.003073	1.60E-08	0.414837	462346	NA	rs1297849	NA	UKB-b-57	TRUE	reported	8KoqAI
19	49239026	T	C	-0.0198	0.003027	6.10E-11	0.499468	462346	NA	rs418464	NA	UKB-b-57	TRUE	reported	8KoqAI
19	49239053	T	C	-0.01995	0.003039	5.20E-11	0.496364	462346	NA	rs452270	NA	UKB-b-57	TRUE	reported	8KoqAI
19	49239200	G	A	-0.01968	0.003027	8.00E-11	0.499162	462346	NA	rs1231281	NA	UKB-b-57	TRUE	reported	8KoqAI
19	49241006	C	G	0.017319	0.003068	1.70E-08	0.435044	462346	NA	rs1298107	NA	UKB-b-57	TRUE	reported	8KoqAI
19	49241014	G	A	-0.01952	0.003044	1.40E-10	0.493497	462346	NA	rs380743	NA	UKB-b-57	TRUE	reported	8KoqAI
19	49241015	C	T	-0.01956	0.003043	1.30E-10	0.493472	462346	NA	rs427248	NA	UKB-b-57	TRUE	reported	8KoqAI
19	49241400	C	T	0.017412	0.003056	1.20E-08	0.429631	462346	NA	rs5606650	NA	UKB-b-57	TRUE	reported	8KoqAI
19	49241976	A	G	-0.01983	0.003026	5.60E-11	0.499463	462346	NA	rs973579	NA	UKB-b-57	TRUE	reported	8KoqAI
19	49244219	C	A	0.017991	0.003056	3.90E-09	0.427818	462346	NA	rs2307018	NA	UKB-b-57	TRUE	reported	8KoqAI
19	49244220	G	A	0.017928	0.003056	4.50E-09	0.427847	462346	NA	rs2307019	NA	UKB-b-57	TRUE	reported	8KoqAI
19	49246866	A	G	0.020434	0.003028	1.50E-11	0.499612	462346	NA	rs838147	NA	UKB-b-57	TRUE	reported	8KoqAI
19	49247086	A	G	0.018298	0.00306	2.20E-09	0.427333	462346	NA	rs1246232	NA	UKB-b-57	TRUE	reported	8KoqAI
19	49247124	T	G	0.0183	0.00306	2.20E-09	0.427338	462346	NA	rs1246029	NA	UKB-b-57	TRUE	reported	8KoqAI
19	49247601	C	T	0.018279	0.003061	2.40E-09	0.427432	462346	NA	rs1167204	NA	UKB-b-57	TRUE	reported	8KoqAI
19	49247693	C	T	0.018287	0.003061	2.30E-09	0.427435	462346	NA	rs8108136	NA	UKB-b-57	TRUE	reported	8KoqAI
19	49247786	G	A	0.01829	0.003061	2.30E-09	0.427428	462346	NA	rs8108468	NA	UKB-b-57	TRUE	reported	8KoqAI
19	49247900	G	A	0.018293	0.003062	2.30E-09	0.427412	462346	NA	rs8111288	NA	UKB-b-57	TRUE	reported	8KoqAI
19	49247963	G	T	0.018306	0.003062	2.20E-09	0.427416	462346	NA	rs8111399	NA	UKB-b-57	TRUE	reported	8KoqAI
19	49248022	A	C	0.018309	0.003062	2.20E-09	0.427411	462346	NA	rs8111208	NA	UKB-b-57	TRUE	reported	8KoqAI
19	49248120	A	G	0.018305	0.003062	2.30E-09	0.427392	462346	NA	rs1108394	NA	UKB-b-57	TRUE	reported	8KoqAI
19	49248255	G	A	0.018313	0.003062	2.20E-09	0.427391	462346	NA	rs1261120	NA	UKB-b-57	TRUE	reported	8KoqAI
19	49248295	G	A	0.018324	0.003063	2.20E-09	0.427399	462346	NA	rs1261120	NA	UKB-b-57	TRUE	reported	8KoqAI
19	49248331	G	A	0.01827	0.003063	2.40E-09	0.427362	462346	NA	rs1261121	NA	UKB-b-57	TRUE	reported	8KoqAI
19	49248640	A	G	0.018211	0.003063	2.80E-09	0.427285	462346	NA	rs1298211	NA	UKB-b-57	TRUE	reported	8KoqAI
19	49248730	G	A	0.021955	0.003055	6.70E-13	0.542982	462346	NA	rs838145	NA	UKB-b-57	TRUE	reported	8KoqAI
19	49249443	A	T	0.017815	0.003065	6.20E-09	0.428061	462346	NA	rs1297503	NA	UKB-b-57	TRUE	reported	8KoqAI
19	49249795	C	T	0.017997	0.003066	4.40E-09	0.427563	462346	NA	rs8102032	NA	UKB-b-57	TRUE	reported	8KoqAI
19	49249814	C	A	0.018005	0.003066	4.30E-09	0.427574	462346	NA	rs8104897	NA	UKB-b-57	TRUE	reported	8KoqAI
19	49249888	G	A	0.018015	0.003066	4.20E-09	0.427561	462346	NA	rs8105137	NA	UKB-b-57	TRUE	reported	8KoqAI
19	49250197	T	C	0.018043	0.003054	3.50E-09	0.443587	462346	NA	rs8106205	NA	UKB-b-57	TRUE	reported	8KoqAI
19	49250239	C	T	0.020106	0.003035	3.50E-11	0.499982	462346	NA	rs838144	NA	UKB-b-57	TRUE	reported	8KoqAI
19	49250538	A	G	0.018079	0.003055	3.20E-09	0.443648	462346	NA	rs1166732	NA	UKB-b-57	TRUE	reported	8KoqAI
19	49250633	G	A	0.018015	0.003068	4.30E-09	0.427444	462346	NA	rs2840001	NA	UKB-b-57	TRUE	reported	8KoqAI
19	49250657	C	G	0.01799	0.003055	3.90E-09	0.444174	462346	NA	rs2840001	NA	UKB-b-57	TRUE	reported	8KoqAI
19	49254955	C	T	0.01856	0.003062	1.40E-09	0.467575	462346	NA	rs8103840	NA	UKB-b-57	TRUE	reported	8KoqAI
19	49259529	A	G	0.018932	0.003132	1.50E-09	0.549383	462346	NA	rs838133	NA	UKB-b-57	TRUE	reported	8KoqAI
19	49261368	T	C	0.019456	0.003165	7.90E-10	0.605976	462346	NA	rs739320	NA	UKB-b-57	TRUE	reported	8KoqAI
19	49263477	G	C	0.020181	0.003167	1.90E-10	0.600835	462346	NA	rs2081194	NA	UKB-b-57	TRUE	reported	8KoqAI
19	49264547	A	G	0.019226	0.003226	2.50E-09	0.640255	462346	NA	rs9749209	NA	UKB-b-57	TRUE	reported	8KoqAI
20	35508347	C	G	0.017014	0.003075	3.10E-08	0.587618	462346	NA	rs6065410	NA	UKB-b-57	TRUE	reported	8KoqAI
20	35508708	A	G	0.016847	0.003073	4.20E-08	0.587727	462346	NA	rs1291124	NA	UKB-b-57	TRUE	reported	8KoqAI
20	35508959	T	C	0.016794	0.003073	4.60E-08	0.587769	462346	NA	rs1291125	NA	UKB-b-57	TRUE	reported	8KoqAI
20	35510689	T	C	0.017009	0.003075	3.20E-08	0.587961	462346	NA	rs1291128	NA	UKB-b-57	TRUE	reported	8KoqAI
20	35511066	G	C	0.016942	0.003075	3.60E-08	0.58791	462346	NA	rs1291130	NA	UKB-b-57	TRUE	reported	8KoqAI
20	35512648	A	G	0.018857	0.003226	5.10E-09	0.670706	462346	NA	rs1291133	NA	UKB-b-57	TRUE	reported	8KoqAI
20	35525362	A	G	0.018037	0.003229	2.30E-08	0.671794	462346	NA	rs1291142	NA	UKB-b-57	TRUE	reported	8KoqAI
20	35528475	T	C	0.019418	0.003266	2.70E-09	0.685915	462346	NA	rs1291145	NA	UKB-b-57	TRUE	reported	8KoqAI
20	35538424	G	A	-0.01932	0.003268	3.40E-09	0.313631	462346	NA	rs6130083	NA	UKB-b-57	TRUE	reported	8KoqAI
20	35538558	A	G	-0.01935	0.003274	3.40E-09	0.313128	462346	NA	rs6016724	NA	UKB-b-57	TRUE	reported	8KoqAI
20	35543207	C	T	-0.01942	0.003268	2.80E-09	0.313668	462346	NA	rs6130096	NA	UKB-b-57	TRUE	reported	8KoqAI
20	35554045	G	A	-0.01752	0.003102	1.60E-08	0.393487	462346	NA	rs6030195	NA	UKB-b-57	TRUE	reported	8KoqAI
20	35554361	G	A	-0.01953	0.003271	2.40E-09	0.31415	462346	NA	rs6030200	NA	UKB-b-57	TRUE	reported	8KoqAI
20	35554528	C	T	-0.01746	0.003102	1.80E-08	0.393609	462346	NA	rs6130119	NA	UKB-b-57	TRUE	reported	8KoqAI
20	35555911	A	G	-0.0195	0.003281	2.80E-09	0.31313	462346	NA	rs6016780	NA	UKB-b-57	TRUE	reported	8KoqAI
20	35555921	C	T	-0.01749	0.003109	1.90E-08	0.392547	462346	NA	rs6016781	NA	UKB-b-57	TRUE	reported	8KoqAI
20	35556192	T	G	-0.01748	0.003103	1.80E-08	0.393611	462346	NA	rs6016783	NA	UKB-b-57	TRUE	reported	8KoqAI
20	35567830	T	G	-0.01946	0.003271	2.70E-09	0.314317	462346	NA	rs4239694	NA	UKB-b-57	TRUE	reported	8KoqAI
20	35569619	G	T	-0.01953	0.003271	2.40E-09	0.314251	462346	NA	rs6030312	NA	UKB-b-57	TRUE	reported	8KoqAI
20	35569824	C	A	-0.01758	0.003101	1.40E-08	0.394355	462346	NA	rs6030315	NA	UKB-b-57	TRUE	reported	8KoqAI
20	35570011	G	A	-0.01758	0.003101	1.40E-08	0.394353	462346	NA	rs1891643	NA	UKB-b-57	TRUE	reported	8KoqAI
21	34270051	A	G	-0.0298	0.005233	1.20E-08	0.093465	462346	NA	rs1170085	NA	UKB-b-57	TRUE	reported	8KoqAI
22	24821154	G	T	-0.07032	0.012812	4.00E-08	0.014184	462346	NA	rs1380198	NA	UKB-b-57	TRUE	reported	8KoqAI
22	24827571	G	A	-0.0706	0.012839	3.80E-08	0.014103	462346	NA	rs1507228	NA	UKB-b-57	TRUE	reported	8KoqAI
22	24828853	C	T	-0.07046	0.012791	3.60E-08	0.0142	462346	NA	rs7165168	NA	UKB-b-57	TRUE	reported	8KoqAI
22	24830400	C	A	-0.07058	0.012839	3.90E-08	0.014104	462346	NA	rs7572753	NA	UKB-b-57	TRUE	reported	8KoqAI
22	24830401	C	A	-0.07058	0.012839	3.90E-08	0.014104	462346	NA	rs8035589	NA	UKB-b-57	TRUE	reported	8KoqAI

22	24842031	C	T	-0.06993	0.012796	4.60E-08	0.014188	462346	NA	rs7481373	NA	UKB-b-57	TRUE	reported	8KoqAI
22	24842494	A	G	-0.07042	0.012838	4.10E-08	0.014107	462346	NA	rs7976022	NA	UKB-b-57	TRUE	reported	8KoqAI
22	24842684	C	T	-0.06993	0.012796	4.60E-08	0.014189	462346	NA	rs7862592	NA	UKB-b-57	TRUE	reported	8KoqAI
22	24843219	A	C	-0.07031	0.012836	4.30E-08	0.014112	462346	NA	rs7866303	NA	UKB-b-57	TRUE	reported	8KoqAI
22	24844948	G	A	-0.07002	0.012796	4.40E-08	0.014193	462346	NA	rs1700492	NA	UKB-b-57	TRUE	reported	8KoqAI
22	24848603	T	C	-0.07005	0.012797	4.40E-08	0.014192	462346	NA	rs4257477	NA	UKB-b-57	TRUE	reported	8KoqAI
22	48874412	G	T	0.016981	0.003039	2.30E-08	0.456775	462346	NA	rs1085485	NA	UKB-b-57	TRUE	reported	8KoqAI
22	48874685	A	G	0.016906	0.003039	2.70E-08	0.456981	462346	NA	rs1109084	NA	UKB-b-57	TRUE	reported	8KoqAI
22	48875699	C	T	0.017021	0.00304	2.20E-08	0.45671	462346	NA	rs9615905	NA	UKB-b-57	TRUE	reported	8KoqAI
22	48875958	T	C	0.016964	0.003063	3.10E-08	0.564102	462346	NA	rs1074227	NA	UKB-b-57	TRUE	reported	8KoqAI
22	48881562	G	C	0.017393	0.003046	1.10E-08	0.454379	462346	NA	rs1894544	NA	UKB-b-57	TRUE	reported	8KoqAI

Major depression SNPs

chr.exposu	pos.exposu	other_allel	effect_allel	beta.expos	se.exposur	pval.expos	eaf.exposu	samplesize	ncase.expo	SNP	ncontrol.e	exposure	mr_keep.e	pval_origir	id.exposure
1	18122009	T	C	-0.0264	0.0046	9.69E-09	0.326	NA	170756	rs4141983	329443	ieu-b-102	TRUE	reported	WYslv6
1	49528403	C	A	-0.0409	0.0073	2.35E-08	0.096	NA	170756	rs1710547	329443	ieu-b-102	TRUE	reported	WYslv6
1	49554476	C	T	-0.0409	0.0073	2.33E-08	0.096	NA	170756	rs589436	329443	ieu-b-102	TRUE	reported	WYslv6
1	49563581	C	T	-0.0412	0.0073	1.71E-08	0.0963	NA	170756	rs655065	329443	ieu-b-102	TRUE	reported	WYslv6
1	49581006	C	T	0.0416	0.0073	1.32E-08	0.9037	NA	170756	rs590503	329443	ieu-b-102	TRUE	reported	WYslv6
1	49675276	G	C	-0.0449	0.0075	1.75E-09	0.0923	NA	170756	rs354155	329443	ieu-b-102	TRUE	reported	WYslv6
1	49692886	T	C	-0.0449	0.0082	4.79E-08	0.0772	NA	170756	rs7485880	329443	ieu-b-102	TRUE	reported	WYslv6
1	49695296	T	C	-0.0447	0.0075	2.16E-09	0.0919	NA	170756	rs8020462	329443	ieu-b-102	TRUE	reported	WYslv6
1	49699638	C	T	-0.044	0.0075	3.75E-09	0.0925	NA	170756	rs3511764	329443	ieu-b-102	TRUE	reported	WYslv6
1	49746671	C	T	-0.0448	0.0075	2.08E-09	0.0916	NA	170756	rs7164773	329443	ieu-b-102	TRUE	reported	WYslv6
1	49754798	A	G	-0.0453	0.0074	1.10E-09	0.0927	NA	170756	rs3420129	329443	ieu-b-102	TRUE	reported	WYslv6
1	49758840	G	A	-0.0453	0.0074	1.12E-09	0.0927	NA	170756	rs3400238	329443	ieu-b-102	TRUE	reported	WYslv6
1	49799321	T	C	-0.0455	0.0075	1.13E-09	0.0919	NA	170756	rs1710562	329443	ieu-b-102	TRUE	reported	WYslv6
1	49803103	A	G	-0.0461	0.0075	7.06E-10	0.0919	NA	170756	rs3546686	329443	ieu-b-102	TRUE	reported	WYslv6
1	49814015	G	C	-0.0449	0.0074	1.46E-09	0.0937	NA	170756	rs1307320	329443	ieu-b-102	TRUE	reported	WYslv6
1	49887243	A	G	-0.0452	0.0081	2.71E-08	0.0776	NA	170756	rs7729949	329443	ieu-b-102	TRUE	reported	WYslv6
1	49890533	T	A	-0.0458	0.0075	8.35E-10	0.0922	NA	170756	rs1534390	329443	ieu-b-102	TRUE	reported	WYslv6
1	49927396	G	A	-0.0447	0.0074	1.87E-09	0.0936	NA	170756	rs3533809	329443	ieu-b-102	TRUE	reported	WYslv6
1	49995988	G	A	-0.0449	0.0074	1.61E-09	0.0934	NA	170756	rs1272710	329443	ieu-b-102	TRUE	reported	WYslv6
1	50021569	G	A	-0.046	0.0075	9.45E-10	0.093	NA	170756	rs1385363	329443	ieu-b-102	TRUE	reported	WYslv6
1	50028625	C	A	-0.0436	0.0075	6.48E-09	0.0925	NA	170756	rs3616378	329443	ieu-b-102	TRUE	reported	WYslv6
1	50053885	C	T	-0.0455	0.0075	1.01E-09	0.0922	NA	170756	rs1158757	329443	ieu-b-102	TRUE	reported	WYslv6
1	50101228	A	T	-0.0463	0.0076	9.30E-10	0.0921	NA	170756	rs1272383	329443	ieu-b-102	TRUE	reported	WYslv6
1	50114228	T	A	-0.0458	0.0074	7.67E-10	0.0923	NA	170756	rs7917211	329443	ieu-b-102	TRUE	reported	WYslv6
1	50115798	C	T	-0.0462	0.0076	1.09E-09	0.0922	NA	170756	rs1027146	329443	ieu-b-102	TRUE	reported	WYslv6
1	50218323	G	A	0.046	0.0076	1.25E-09	0.9074	NA	170756	rs4926828	329443	ieu-b-102	TRUE	reported	WYslv6
1	50232651	T	A	0.0457	0.0074	7.75E-10	0.9076	NA	170756	rs6671257	329443	ieu-b-102	TRUE	reported	WYslv6
1	50243627	T	C	0.0458	0.0074	6.88E-10	0.9072	NA	170756	rs1923007	329443	ieu-b-102	TRUE	reported	WYslv6
1	50331725	T	C	0.0462	0.0075	6.02E-10	0.9079	NA	170756	rs1078892	329443	ieu-b-102	TRUE	reported	WYslv6
1	50415802	A	G	-0.0458	0.0075	7.86E-10	0.0927	NA	170756	rs1557612	329443	ieu-b-102	TRUE	reported	WYslv6
1	50445126	C	T	-0.0483	0.0075	8.99E-11	0.0935	NA	170756	rs3565431	329443	ieu-b-102	TRUE	reported	WYslv6
1	50450860	G	A	-0.0459	0.0074	7.06E-10	0.0924	NA	170756	rs3406872	329443	ieu-b-102	TRUE	reported	WYslv6
1	50456837	C	T	-0.0462	0.0074	5.61E-10	0.0925	NA	170756	rs1157694	329443	ieu-b-102	TRUE	reported	WYslv6
1	50482476	A	G	-0.0461	0.0074	5.99E-10	0.0924	NA	170756	rs1274701	329443	ieu-b-102	TRUE	reported	WYslv6
1	50504100	A	G	-0.0475	0.0075	2.17E-10	0.0918	NA	170756	rs3559661	329443	ieu-b-102	TRUE	reported	WYslv6
1	50559162	A	G	-0.0491	0.0075	6.00E-11	0.0914	NA	170756	rs1157924	329443	ieu-b-102	TRUE	reported	WYslv6
1	50577654	G	T	-0.0349	0.0064	4.34E-08	0.1316	NA	170756	rs2480677	329443	ieu-b-102	TRUE	reported	WYslv6
1	50584682	A	G	-0.0364	0.0064	1.54E-08	0.1302	NA	170756	rs7517535	329443	ieu-b-102	TRUE	reported	WYslv6
1	50588093	A	G	-0.0359	0.0064	2.22E-08	0.1295	NA	170756	rs2494883	329443	ieu-b-102	TRUE	reported	WYslv6
1	50588843	G	T	-0.035	0.0063	3.36E-08	0.1322	NA	170756	rs2480692	329443	ieu-b-102	TRUE	reported	WYslv6
1	50591405	G	T	-0.035	0.0063	3.39E-08	0.1322	NA	170756	rs2494884	329443	ieu-b-102	TRUE	reported	WYslv6
1	50591743	C	T	-0.0354	0.0064	2.91E-08	0.1315	NA	170756	rs2494885	329443	ieu-b-102	TRUE	reported	WYslv6
1	52266242	G	A	0.0242	0.0043	1.69E-08	0.5057	NA	170756	rs8375	329443	ieu-b-102	TRUE	reported	WYslv6
1	52269046	C	A	0.0271	0.0043	3.53E-10	0.5299	NA	170756	rs6685190	329443	ieu-b-102	TRUE	reported	WYslv6
1	52274078	T	G	0.0283	0.0043	5.11E-11	0.5329	NA	170756	rs7551758	329443	ieu-b-102	TRUE	reported	WYslv6
1	52279650	T	G	0.0251	0.0045	3.20E-08	0.6585	NA	170756	rs2982846	329443	ieu-b-102	TRUE	reported	WYslv6
1	52290984	T	G	0.0237	0.0043	3.82E-08	0.5045	NA	170756	rs1120589	329443	ieu-b-102	TRUE	reported	WYslv6
1	52294515	G	A	0.0268	0.0043	6.46E-10	0.528	NA	170756	rs2405861	329443	ieu-b-102	TRUE	reported	WYslv6
1	52297961	C	T	0.0277	0.0043	1.34E-10	0.5299	NA	170756	rs1213532	329443	ieu-b-102	TRUE	reported	WYslv6
1	52298151	C	T	0.0249	0.0043	7.44E-09	0.5074	NA	170756	rs1120589	329443	ieu-b-102	TRUE	reported	WYslv6
1	52305664	G	A	0.0269	0.0043	6.01E-10	0.5284	NA	170756	rs7520502	329443	ieu-b-102	TRUE	reported	WYslv6
1	52310756	A	G	0.0268	0.0043	6.70E-10	0.528	NA	170756	rs6702037	329443	ieu-b-102	TRUE	reported	WYslv6
1	52310882	C	T	0.0258	0.0043	2.30E-09	0.5365	NA	170756	rs4422953	329443	ieu-b-102	TRUE	reported	WYslv6
1	52311115	T	C	-0.0248	0.0045	4.63E-08	0.3411	NA	170756	rs6793266	329443	ieu-b-102	TRUE	reported	WYslv6
1	52316785	T	C	0.0269	0.0043	6.09E-10	0.5282	NA	170756	rs6676789	329443	ieu-b-102	TRUE	reported	WYslv6
1	52317231	T	A	-0.0249	0.0045	3.08E-08	0.377	NA	170756	rs1557931	329443	ieu-b-102	TRUE	reported	WYslv6
1	52320559	T	C	0.0248	0.0043	7.53E-09	0.5078	NA	170756	rs7526552	329443	ieu-b-102	TRUE	reported	WYslv6
1	52322661	C	T	-0.0248	0.0045	4.29E-08	0.3408	NA	170756	rs1120589	329443	ieu-b-102	TRUE	reported	WYslv6
1	52323772	G	C	0.0278	0.0043	1.17E-10	0.5302	NA	170756	rs6691091	329443	ieu-b-102	TRUE	reported	WYslv6
1	52331695	C	A	-0.0249	0.0045	4.23E-08	0.3408	NA	170756	rs3525625	329443	ieu-b-102	TRUE	reported	WYslv6
1	52334960	G	A	-0.0252	0.0045	2.12E-08	0.3498	NA	170756	rs3417264	329443	ieu-b-102	TRUE	reported	WYslv6
1	52335096	G	A	-0.0249	0.0045	4.25E-08	0.3409	NA	170756	rs3474356	329443	ieu-b-102	TRUE	reported	WYslv6
1	52337039	T	C	0.028	0.0043	8.37E-11	0.5305	NA	170756	rs1538881	329443	ieu-b-102	TRUE	reported	WYslv6
1	52339759	T	C	0.0279	0.0043	9.47E-11	0.5298	NA	170756	rs7413471	329443	ieu-b-102	TRUE	reported	WYslv6
1	52341507	G	A	-0.0251	0.0045	3.08E-08	0.3414	NA	170756	rs3572856	329443	ieu-b-102	TRUE	reported	WYslv6
1	52342427	T	C	0.0282	0.0043	5.89E-11	0.5298	NA	170756	rs1890946	329443	ieu-b-102	TRUE	reported	WYslv6
1	52344414	A	C	0.0255	0.0043	3.04E-09	0.5069	NA	170756	rs736756	329443	ieu-b-102	TRUE	reported	WYslv6
1	52345760	C	T	0.0263	0.0045	4.64E-09	0.6378	NA	170756	rs2842586	329443	ieu-b-102	TRUE	reported	WYslv6
1	67052164	C	T	0.0238	0.0043	3.50E-08	0.5639	NA	170756	rs1445575	329443	ieu-b-102	TRUE	reported	WYslv6
1	67052609	T	C	0.0238	0.0043	3.69E-08	0.5639	NA	170756	rs4655628	329443	ieu-b-102	TRUE	reported	WYslv6
1	67054080	T	C	0.0245	0.0043	1.52E-08	0.5626	NA	170756	rs1424817	329443	ieu-b-102	TRUE	reported	WYslv6
1	67054081	T	A	0.0239	0.0044	4.72E-08	0.5626	NA	170756	rs8796493	329443	ieu-b-102	TRUE	reported	WYslv6
1	67054907	C	G	0.025	0.0044	1.12E-08	0.5653	NA	170756	rs1291308	329443	ieu-b-102	TRUE	reported	WYslv6
1	67058023	T	C	0.0241	0.0043	2.77E-08	0.5626	NA	170756	rs4655633	329443	ieu-b-102	TRUE	reported	WYslv6
1	67062126	G	A	0.0241	0.0043	2.45E-08	0.5639	NA	170756	rs1074976	329443	ieu-b-102	TRUE	reported	WYslv6
1	67062885	T	C	0.024	0.0043	2.90E-08	0.5637	NA	170756	rs964574	329443	ieu-b-102	TRUE	reported	WYslv6
1	67065911	G	A	0.0239	0.0043	3.35E-08	0.5632	NA	170756	rs1088963	329443	ieu-b-102	TRUE	reported	WYslv6
1	67066030	G	A	0.024	0.0043	2.89E-08	0.5633	NA	170756	rs1078921	329443	ieu-b-102	TRUE	reported	WYslv6
1	67066555	A	G	0.0241	0.0043	2.50E-08	0.5632	NA	170756	rs6588207	329443	ieu-b-102	TRUE	reported	WYslv6
1	67067369	C	T	0.024	0.0043	3.14E-08	0.4336	NA	170756	rs7528182	329443	ieu-b-102	TRUE	reported	WYslv6
1	67068112	T	C	0.0243	0.0043	2.07E-08	0.5635	NA	170756	rs6689864	329443	ieu-b-102	TRUE	reported	WYslv6
1	67068240	T	G	0.0239	0.0043	3.39E-08	0.5634	NA	170756	rs4655635	329443	ieu-b-102	TRUE	reported	WYslv6
1	67068537	A	G	0.0242	0.0043	2.30E-08	0.5632	NA	170756	rs4655636	329443	ieu-b-102	TRUE	reported	WYslv6
1	67070381	T	C	0.0242	0.0043	2.15E-08	0.5638	NA	170756	rs6696927	329443	ieu-b-102	TRUE	reported	WYslv6
1	67072031	C	T	0.0242	0.0043	2.31E-08	0.5633	NA	170756	rs7524818	329443	ieu-b-102	TRUE	reported	WYslv6
1	67072976	A	G	0.0236	0.0043	4.95E-08	0.433	NA	170756	rs2312154	329443	ieu-b-102	TRUE	reported	WYslv6
1	67073314	T	C	0.024	0.0043	3.12E-08	0.4336	NA	170756	rs4488004	329443	ieu-b-102	TRUE	reported	WYslv6
1	67073566	T	C	0.0241	0.0043	2.35E-08	0.5633	NA	170756	rs1562217	329443	ieu-b-102	TRUE	reported	WYslv6
1	67074177	C	T	0.0237	0.0043	4.41E-08	0.433	NA	170756	rs6679568	329443	ieu-b-102	TRUE	reported	WYslv6
1	67075575	G	A	0.0241	0.0043	2.65E-08	0.5638	NA	170756	rs1088963	329443	ieu-b-102	TRUE	reported	WYslv6
1	67083671	T	C	0.0252	0.0043	6.50E-09	0.5727	NA	170756	rs1248904	329443	ieu-b-102	TRUE	reported	WYslv6
1	67086111	A	G	0.0244	0.0043	1.77E-08	0.562	NA	170756	rs1558094	329443	ieu-b-102	TRUE	reported	WYslv6
1	67089220	T	C	0.0241	0.0043	2.51E-08	0.565	NA	170756	rs4655640	329443	ieu-b-102	TRUE	reported	WYslv6
1	67093018	T	C	0.0241	0.0043	2.60E-08	0.5652	NA	170756	rs7524851	329443	ieu-b-102	TRUE	reported	WYslv6
1	67094336	G	A	0.0241	0.0043	2.41E-08	0.5652	NA	170756	rs4655646	329443	ieu-b-102	TRUE	reported	WYslv6
1	67097853	T	C	0.0241	0.0043	2.54E-08	0.5652	NA	170756	rs1120893	329443	ieu-b-102	TRUE	reported	WYslv6
1	67106077	C	G	0.0245	0.0043	1.63E-08	0.5658	NA	170756	rs4655647	329443	ieu-b-102	TRUE	reported	WYslv6
1	67110516	G	A	0.0246	0.0043	1.19E-08	0.5645	NA	170756	rs1325261	329443	ieu-b-102	TRUE	reported	WYslv6
1	67114309	C	T	0.0244	0.0043	1.59E-08	0.5651	NA	170756	rs4655648	329443	ieu-b-102	TRUE	reported	WYslv6
1	67116231	C	T	0.0246	0.0043	1.40E-08	0.5655	NA	170756	rs1329978	329443	ieu-b-102	TRUE	reported	WYslv6
1	67121001	A	T	0.0243	0.0043	1.97E-08	0.5646	NA	170756	rs1536115	329443	ieu-b-102	TRUE	reported	WYslv6
1	67121132	C	T	0.0243	0.0043	2.00E-08	0.5654	NA	170756	rs9662943	329443	ieu-b-102	TRUE	reported	WYslv6
1	67121364	C	T	0.0242	0.0043	2.07E-08	0.5621	NA	170756	rs9662962	329443	ieu-b-102	TRUE	reported	WYslv6

1	67121434	T	C	0.0243	0.0043	2.06E-08	0.5621	NA	170756	rs9659879	329443	ieu-b-102	TRUE	reported	WYslv6
1	67121634	T	C	0.0242	0.0043	2.11E-08	0.5621	NA	170756	rs7521111	329443	ieu-b-102	TRUE	reported	WYslv6
1	67121663	T	C	0.0243	0.0043	1.99E-08	0.5621	NA	170756	rs7521123	329443	ieu-b-102	TRUE	reported	WYslv6
1	67122552	C	T	0.0243	0.0043	1.87E-08	0.5628	NA	170756	rs6656550	329443	ieu-b-102	TRUE	reported	WYslv6
1	67122565	A	T	0.0243	0.0043	1.94E-08	0.5621	NA	170756	rs6680944	329443	ieu-b-102	TRUE	reported	WYslv6
1	67123057	A	G	0.0243	0.0043	1.89E-08	0.5621	NA	170756	rs6681460	329443	ieu-b-102	TRUE	reported	WYslv6
1	67123459	C	T	0.0243	0.0043	2.01E-08	0.5621	NA	170756	rs6659968	329443	ieu-b-102	TRUE	reported	WYslv6
1	67123742	C	A	0.0243	0.0043	1.87E-08	0.5622	NA	170756	rs2031478	329443	ieu-b-102	TRUE	reported	WYslv6
1	67126585	C	T	0.0245	0.0043	1.57E-08	0.5625	NA	170756	rs6670378	329443	ieu-b-102	TRUE	reported	WYslv6
1	67127367	C	A	0.0244	0.0043	1.73E-08	0.5632	NA	170756	rs2872078	329443	ieu-b-102	TRUE	reported	WYslv6
1	67129254	A	T	0.0244	0.0043	1.73E-08	0.5626	NA	170756	rs2147777	329443	ieu-b-102	TRUE	reported	WYslv6
1	67129482	G	A	0.0244	0.0043	1.64E-08	0.5624	NA	170756	rs6680876	329443	ieu-b-102	TRUE	reported	WYslv6
1	67129791	C	A	0.0244	0.0043	1.77E-08	0.5632	NA	170756	rs1325263	329443	ieu-b-102	TRUE	reported	WYslv6
1	67132262	T	C	0.0251	0.0043	7.29E-09	0.5599	NA	170756	rs7538938	329443	ieu-b-102	TRUE	reported	WYslv6
1	67138367	C	T	0.0245	0.0043	1.66E-08	0.5631	NA	170756	rs1088964	329443	ieu-b-102	TRUE	reported	WYslv6
1	67139667	C	T	0.0238	0.0044	4.80E-08	0.5642	NA	170756	rs1570815	329443	ieu-b-102	TRUE	reported	WYslv6
1	67140723	T	C	0.0241	0.0043	2.87E-08	0.563	NA	170756	rs7519747	329443	ieu-b-102	TRUE	reported	WYslv6
1	67141949	A	G	0.0242	0.0043	2.61E-08	0.5628	NA	170756	rs6588215	329443	ieu-b-102	TRUE	reported	WYslv6
1	67142286	T	C	0.0245	0.0044	1.83E-08	0.5636	NA	170756	rs4655650	329443	ieu-b-102	TRUE	reported	WYslv6
1	67143055	G	A	0.0242	0.0043	2.60E-08	0.5626	NA	170756	rs7542924	329443	ieu-b-102	TRUE	reported	WYslv6
1	67143334	T	G	0.0243	0.0043	2.29E-08	0.5622	NA	170756	rs1088964	329443	ieu-b-102	TRUE	reported	WYslv6
1	67143368	G	C	0.0246	0.0044	1.47E-08	0.5627	NA	170756	rs1088964	329443	ieu-b-102	TRUE	reported	WYslv6
1	67145322	C	A	0.0242	0.0043	2.50E-08	0.563	NA	170756	rs1088964	329443	ieu-b-102	TRUE	reported	WYslv6
1	67146817	C	T	0.025	0.0044	9.04E-09	0.5629	NA	170756	rs1078921	329443	ieu-b-102	TRUE	reported	WYslv6
1	67150280	C	T	0.0244	0.0043	1.91E-08	0.5626	NA	170756	rs1074976	329443	ieu-b-102	TRUE	reported	WYslv6
1	67150811	G	A	0.0243	0.0043	2.06E-08	0.5619	NA	170756	rs4655506	329443	ieu-b-102	TRUE	reported	WYslv6
1	67151185	T	C	0.0244	0.0043	1.83E-08	0.5626	NA	170756	rs1078921	329443	ieu-b-102	TRUE	reported	WYslv6
1	67152499	G	A	0.0243	0.0043	2.21E-08	0.5626	NA	170756	rs2025595	329443	ieu-b-102	TRUE	reported	WYslv6
1	72556393	C	T	-0.0247	0.0044	1.47E-08	0.5144	NA	170756	rs973526	329443	ieu-b-102	TRUE	reported	WYslv6
1	72558138	T	C	0.0273	0.0043	2.95E-10	0.4564	NA	170756	rs1214009	329443	ieu-b-102	TRUE	reported	WYslv6
1	72559740	C	T	-0.0267	0.0043	6.70E-10	0.5218	NA	170756	rs727726	329443	ieu-b-102	TRUE	reported	WYslv6
1	72565460	C	T	-0.0272	0.0043	2.96E-10	0.525	NA	170756	rs2220253	329443	ieu-b-102	TRUE	reported	WYslv6
1	72569613	T	A	-0.0267	0.0043	5.96E-10	0.5236	NA	170756	rs2630392	329443	ieu-b-102	TRUE	reported	WYslv6
1	72571364	C	T	-0.0266	0.0043	6.34E-10	0.5243	NA	170756	rs1486091	329443	ieu-b-102	TRUE	reported	WYslv6
1	72571656	C	A	0.0271	0.0043	3.69E-10	0.4568	NA	170756	rs1039296	329443	ieu-b-102	TRUE	reported	WYslv6
1	72573363	T	C	0.0271	0.0043	3.37E-10	0.4568	NA	170756	rs1486093	329443	ieu-b-102	TRUE	reported	WYslv6
1	72573432	T	A	-0.0269	0.0043	4.95E-10	0.5256	NA	170756	rs1486094	329443	ieu-b-102	TRUE	reported	WYslv6
1	72573798	T	C	0.027	0.0043	3.75E-10	0.4567	NA	170756	rs1564970	329443	ieu-b-102	TRUE	reported	WYslv6
1	72577177	G	A	-0.0268	0.0043	4.85E-10	0.5221	NA	170756	rs2630395	329443	ieu-b-102	TRUE	reported	WYslv6
1	72577179	T	A	-0.0269	0.0043	4.71E-10	0.5221	NA	170756	rs7157547	329443	ieu-b-102	TRUE	reported	WYslv6
1	72578709	T	C	0.0277	0.0043	1.30E-10	0.4574	NA	170756	rs1240262	329443	ieu-b-102	TRUE	reported	WYslv6
1	72583375	T	A	-0.0276	0.0043	1.54E-10	0.5224	NA	170756	rs1385912	329443	ieu-b-102	TRUE	reported	WYslv6
1	72583763	G	A	-0.0276	0.0043	1.36E-10	0.5223	NA	170756	rs2630400	329443	ieu-b-102	TRUE	reported	WYslv6
1	72584184	G	A	0.0278	0.0043	1.05E-10	0.458	NA	170756	rs1486096	329443	ieu-b-102	TRUE	reported	WYslv6
1	72586208	C	T	0.0278	0.0043	1.16E-10	0.4569	NA	170756	rs8799319	329443	ieu-b-102	TRUE	reported	WYslv6
1	72587186	G	C	0.0278	0.0043	1.18E-10	0.4579	NA	170756	rs7556006	329443	ieu-b-102	TRUE	reported	WYslv6
1	72590193	G	A	-0.0275	0.0043	1.73E-10	0.5225	NA	170756	rs2821239	329443	ieu-b-102	TRUE	reported	WYslv6
1	72590865	G	A	-0.0275	0.0043	1.74E-10	0.5225	NA	170756	rs1049937	329443	ieu-b-102	TRUE	reported	WYslv6
1	72593174	G	T	0.027	0.0044	7.13E-10	0.454	NA	170756	rs7650597	329443	ieu-b-102	TRUE	reported	WYslv6
1	72593767	C	T	0.0277	0.0043	1.38E-10	0.4575	NA	170756	rs1214162	329443	ieu-b-102	TRUE	reported	WYslv6
1	72597051	A	G	0.0276	0.0043	1.43E-10	0.4584	NA	170756	rs1213260	329443	ieu-b-102	TRUE	reported	WYslv6
1	72597654	T	A	0.0278	0.0043	1.16E-10	0.4564	NA	170756	rs1275063	329443	ieu-b-102	TRUE	reported	WYslv6
1	72603400	C	G	-0.0276	0.0043	1.32E-10	0.5214	NA	170756	rs2630390	329443	ieu-b-102	TRUE	reported	WYslv6
1	72609955	G	C	0.0277	0.0043	1.32E-10	0.4578	NA	170756	rs5731690	329443	ieu-b-102	TRUE	reported	WYslv6
1	72610802	A	T	0.0277	0.0043	1.33E-10	0.4578	NA	170756	rs1120992	329443	ieu-b-102	TRUE	reported	WYslv6
1	72610879	G	T	0.0277	0.0043	1.25E-10	0.4578	NA	170756	rs1213360	329443	ieu-b-102	TRUE	reported	WYslv6
1	72612371	C	T	0.0278	0.0043	1.08E-10	0.4589	NA	170756	rs1120992	329443	ieu-b-102	TRUE	reported	WYslv6
1	72613608	G	T	0.0277	0.0043	1.43E-10	0.4577	NA	170756	rs7515320	329443	ieu-b-102	TRUE	reported	WYslv6
1	72613917	C	T	0.0275	0.0043	1.72E-10	0.4577	NA	170756	rs7541218	329443	ieu-b-102	TRUE	reported	WYslv6
1	72614077	G	T	0.0275	0.0043	1.74E-10	0.4576	NA	170756	rs7517923	329443	ieu-b-102	TRUE	reported	WYslv6
1	72618544	C	A	0.0285	0.0043	3.65E-11	0.498	NA	170756	rs2422136	329443	ieu-b-102	TRUE	reported	WYslv6
1	72619961	C	A	0.028	0.0043	1.05E-10	0.4528	NA	170756	rs2422137	329443	ieu-b-102	TRUE	reported	WYslv6
1	72627031	G	A	0.0284	0.0043	3.81E-11	0.4974	NA	170756	rs1120992	329443	ieu-b-102	TRUE	reported	WYslv6
1	72629795	T	C	0.0284	0.0043	3.99E-11	0.4974	NA	170756	rs2821296	329443	ieu-b-102	TRUE	reported	WYslv6
1	72634986	T	C	0.0284	0.0043	3.96E-11	0.4974	NA	170756	rs9948947	329443	ieu-b-102	TRUE	reported	WYslv6
1	72637154	G	T	-0.0291	0.0053	4.98E-08	0.2036	NA	170756	rs6176561	329443	ieu-b-102	TRUE	reported	WYslv6
1	72639381	T	C	-0.0292	0.0053	4.74E-08	0.2035	NA	170756	rs6176561	329443	ieu-b-102	TRUE	reported	WYslv6
1	72639856	G	A	0.0285	0.0043	3.38E-11	0.4973	NA	170756	rs2821295	329443	ieu-b-102	TRUE	reported	WYslv6
1	72640442	A	C	-0.0291	0.0053	4.82E-08	0.2036	NA	170756	rs7990865	329443	ieu-b-102	TRUE	reported	WYslv6
1	72642650	G	A	0.0285	0.0043	3.46E-11	0.4974	NA	170756	rs2821293	329443	ieu-b-102	TRUE	reported	WYslv6
1	72642877	A	G	0.0284	0.0043	3.63E-11	0.4985	NA	170756	rs1120993	329443	ieu-b-102	TRUE	reported	WYslv6
1	72644116	C	T	0.0282	0.0043	5.42E-11	0.4977	NA	170756	rs1203658	329443	ieu-b-102	TRUE	reported	WYslv6
1	72645734	G	A	0.0284	0.0043	4.11E-11	0.4976	NA	170756	rs6699731	329443	ieu-b-102	TRUE	reported	WYslv6
1	72645850	G	A	0.0278	0.0043	1.14E-10	0.4982	NA	170756	rs6699841	329443	ieu-b-102	TRUE	reported	WYslv6
1	72647443	A	G	0.0284	0.0043	3.84E-11	0.4974	NA	170756	rs1120993	329443	ieu-b-102	TRUE	reported	WYslv6
1	72649154	C	T	-0.0294	0.0054	4.04E-08	0.2022	NA	170756	rs1272608	329443	ieu-b-102	TRUE	reported	WYslv6
1	72650925	G	T	0.0285	0.0043	3.31E-11	0.4973	NA	170756	rs6668604	329443	ieu-b-102	TRUE	reported	WYslv6
1	72660048	A	C	-0.0292	0.0053	4.51E-08	0.2037	NA	170756	rs3428970	329443	ieu-b-102	TRUE	reported	WYslv6
1	72661104	C	T	-0.0286	0.0043	3.00E-11	0.503	NA	170756	rs2821288	329443	ieu-b-102	TRUE	reported	WYslv6
1	72662344	A	T	-0.0293	0.0044	3.80E-11	0.4741	NA	170756	rs3866323	329443	ieu-b-102	TRUE	reported	WYslv6
1	72662959	C	T	-0.0291	0.0053	4.91E-08	0.2038	NA	170756	rs3484070	329443	ieu-b-102	TRUE	reported	WYslv6
1	72667196	C	T	-0.0285	0.0043	3.08E-11	0.5024	NA	170756	rs2821285	329443	ieu-b-102	TRUE	reported	WYslv6
1	72673194	G	A	-0.0286	0.0043	2.97E-11	0.5024	NA	170756	rs2797540	329443	ieu-b-102	TRUE	reported	WYslv6
1	72674489	C	T	-0.0285	0.0043	3.24E-11	0.5017	NA	170756	rs1858600	329443	ieu-b-102	TRUE	reported	WYslv6
1	72681358	A	G	-0.0285	0.0043	3.36E-11	0.5025	NA	170756	rs1194259	329443	ieu-b-102	TRUE	reported	WYslv6
1	72682718	T	A	-0.0292	0.0054	4.87E-08	0.2026	NA	170756	rs1272508	329443	ieu-b-102	TRUE	reported	WYslv6
1	72683226	C	T	-0.0284	0.0043	3.75E-11	0.5028	NA	170756	rs1675356	329443	ieu-b-102	TRUE	reported	WYslv6
1	72688503	G	A	-0.0284	0.0043	3.86E-11	0.5031	NA	170756	rs1194286	329443	ieu-b-102	TRUE	reported	WYslv6
1	72695547	G	T	-0.0283	0.0043	4.34E-11	0.5028	NA	170756	rs1205484	329443	ieu-b-102	TRUE	reported	WYslv6
1	72696927	C	T	-0.0285	0.0043	3.25E-11	0.5027	NA	170756	rs1194273	329443	ieu-b-102	TRUE	reported	WYslv6
1	72701100	G	A	-0.0286	0.0043	2.87E-11	0.5027	NA	170756	rs2797543	329443	ieu-b-102	TRUE	reported	WYslv6
1	72701965	A	G	-0.0282	0.0043	5.17E-11	0.5026	NA	170756	rs1194271	329443	ieu-b-102	TRUE	reported	WYslv6
1	72701991	C	G	-0.0282	0.0043	5.66E-11	0.5026	NA	170756	rs1209251	329443	ieu-b-102	TRUE	reported	WYslv6
1	72702313	T	C	-0.0284	0.0043	4.33E-11	0.5025	NA	170756	rs1194270	329443	ieu-b-102	TRUE	reported	WYslv6
1	72703860	G	A	-0.0288	0.0043	2.28E-11	0.5027	NA	170756	rs1194269	329443	ieu-b-102	TRUE	reported	WYslv6
1	72704526	A	G	-0.0286	0.0043	2.89E-11	0.5028	NA	170756	rs1194267	329443	ieu-b-102	TRUE	reported	WYslv6
1	72707226	A	G	-0.0287	0.0043	2.79E-11	0.5028	NA	170756	rs1194265	329443	ieu-b-102	TRUE	reported	WYslv6
1	72708154	G	C	-0.0287	0.0043	2.76E-11	0.5027	NA	170756	rs1194263	329443	ieu-b-102	TRUE	reported	WYslv6
1	72708584	A	G	-0.0287	0.0043	2.76E-11	0.5028	NA	170756	rs1026567	329443	ieu-b-102	TRUE	reported	WYslv6
1	72709042	G	A	-0.0292	0.0043	1.15E-11	0.5027	NA	170756	rs1026566	329443	ieu-b-102	TRUE	reported	WYslv6
1	72712529	A	C	-0.0288	0.0043	2.37E-11	0.5026	NA	170756	rs1194284	329443	ieu-b-102	TRUE	reported	WYslv6
1	72712607	A	T	-0.0288	0.0043	2.29E-11	0.4826	NA	170756	rs2126077	329443	ieu-b-102	TRUE	reported	WYslv6
1	72713037	C	T	-0.0289	0.0043	2.06E-11	0.5025	NA	170756	rs1194283	329443	ieu-b-102	TRUE	reported	WYslv6
1	72713559	T	C	-0.029	0.0043	2.03E-11	0.5029	NA	170756	rs1194282	329443	ieu-b-102	TRUE	reported	WYslv6
1	72714003	T	C	-0.0291	0.0043	1.71E-11	0.5027	NA	170756	rs1194281	329443	ieu-b-102	TRUE	reported	WYslv6
1	72714220	C	T	-0.0289	0.0043	2.99E-11	0.501	NA	170756	rs1194278	329443	ieu-b-102	TRUE	reported	WYslv6
1	72714331	C	G	-0.0293	0.0043	1.46E-11	0.5029	NA	170756	rs1194277	329443	ieu-b-102	TRUE	reported	WYslv6

1	72717743	A	G	-0.0244	0.0043	1.42E-08	0.513	NA	170756	rs1776012	329443	ieu-b-102	TRUE	reported	WYslv6
1	72723211	T	A	-0.0321	0.0054	3.44E-09	0.1967	NA	170756	rs6176564	329443	ieu-b-102	TRUE	reported	WYslv6
1	72724409	A	C	-0.0319	0.0054	4.16E-09	0.1967	NA	170756	rs3472345	329443	ieu-b-102	TRUE	reported	WYslv6
1	72725344	T	C	-0.0244	0.0043	1.41E-08	0.5124	NA	170756	rs9424977	329443	ieu-b-102	TRUE	reported	WYslv6
1	72733841	A	T	-0.0243	0.0043	1.59E-08	0.5118	NA	170756	rs1380995	329443	ieu-b-102	TRUE	reported	WYslv6
1	72735458	T	C	-0.0246	0.0043	1.14E-08	0.5113	NA	170756	rs1213723	329443	ieu-b-102	TRUE	reported	WYslv6
1	72738699	T	C	-0.0319	0.0054	4.06E-09	0.1967	NA	170756	rs3437616	329443	ieu-b-102	TRUE	reported	WYslv6
1	72739527	A	C	-0.032	0.0054	3.89E-09	0.1967	NA	170756	rs1273756	329443	ieu-b-102	TRUE	reported	WYslv6
1	72740634	G	A	-0.0244	0.0043	1.49E-08	0.5124	NA	170756	rs7549792	329443	ieu-b-102	TRUE	reported	WYslv6
1	72741829	T	C	-0.0245	0.0043	1.25E-08	0.5124	NA	170756	rs1158461	329443	ieu-b-102	TRUE	reported	WYslv6
1	72742235	G	T	-0.0245	0.0043	1.27E-08	0.5124	NA	170756	rs9425081	329443	ieu-b-102	TRUE	reported	WYslv6
1	72742387	C	T	-0.0319	0.0054	4.07E-09	0.1966	NA	170756	rs3402095	329443	ieu-b-102	TRUE	reported	WYslv6
1	72744144	G	A	-0.0248	0.0043	8.84E-09	0.5123	NA	170756	rs3101340	329443	ieu-b-102	TRUE	reported	WYslv6
1	72744950	C	G	-0.0325	0.0054	2.19E-09	0.1962	NA	170756	rs3487424	329443	ieu-b-102	TRUE	reported	WYslv6
1	72744996	T	C	0.025	0.0043	6.75E-09	0.4876	NA	170756	rs1870676	329443	ieu-b-102	TRUE	reported	WYslv6
1	72745962	A	G	-0.0325	0.0054	2.22E-09	0.1963	NA	170756	rs3457934	329443	ieu-b-102	TRUE	reported	WYslv6
1	72747844	C	T	0.0252	0.0043	4.62E-09	0.4873	NA	170756	rs3101341	329443	ieu-b-102	TRUE	reported	WYslv6
1	72748669	A	C	0.0377	0.0044	1.04E-17	0.6039	NA	170756	rs1470369	329443	ieu-b-102	TRUE	reported	WYslv6
1	72750470	G	A	-0.0348	0.0055	2.26E-10	0.189	NA	170756	rs3436114	329443	ieu-b-102	TRUE	reported	WYslv6
1	72750500	A	G	0.0371	0.0044	4.04E-17	0.6042	NA	170756	rs1120994	329443	ieu-b-102	TRUE	reported	WYslv6
1	72751134	C	T	0.038	0.0044	4.68E-18	0.6044	NA	170756	rs3101337	329443	ieu-b-102	TRUE	reported	WYslv6
1	72751185	T	C	0.0379	0.0044	6.24E-18	0.6043	NA	170756	rs3101336	329443	ieu-b-102	TRUE	reported	WYslv6
1	72751552	A	G	-0.0348	0.0055	2.30E-10	0.189	NA	170756	rs2613499	329443	ieu-b-102	TRUE	reported	WYslv6
1	72752073	G	A	-0.035	0.0055	1.86E-10	0.1889	NA	170756	rs1274078	329443	ieu-b-102	TRUE	reported	WYslv6
1	72752230	T	C	0.0376	0.0044	1.15E-17	0.6044	NA	170756	rs2815765	329443	ieu-b-102	TRUE	reported	WYslv6
1	72752939	C	T	-0.0348	0.0055	2.29E-10	0.189	NA	170756	rs2613498	329443	ieu-b-102	TRUE	reported	WYslv6
1	72753112	A	G	-0.0343	0.0055	4.79E-10	0.1883	NA	170756	rs6176565	329443	ieu-b-102	TRUE	reported	WYslv6
1	72753825	A	T	0.0348	0.0055	2.40E-10	0.8107	NA	170756	rs1218908	329443	ieu-b-102	TRUE	reported	WYslv6
1	72753956	T	C	0.0349	0.0055	2.13E-10	0.811	NA	170756	rs2568953	329443	ieu-b-102	TRUE	reported	WYslv6
1	72754123	A	G	0.0349	0.0055	2.09E-10	0.811	NA	170756	rs2815764	329443	ieu-b-102	TRUE	reported	WYslv6
1	72754314	C	T	-0.0349	0.0055	2.28E-10	0.1889	NA	170756	rs6176565	329443	ieu-b-102	TRUE	reported	WYslv6
1	72754788	T	C	0.0348	0.0055	2.22E-10	0.811	NA	170756	rs2613496	329443	ieu-b-102	TRUE	reported	WYslv6
1	72755105	C	G	0.038	0.0044	4.43E-18	0.6044	NA	170756	rs2568952	329443	ieu-b-102	TRUE	reported	WYslv6
1	72764289	C	T	0.035	0.0055	1.92E-10	0.8109	NA	170756	rs2815757	329443	ieu-b-102	TRUE	reported	WYslv6
1	72764376	T	C	0.0381	0.0044	3.57E-18	0.6043	NA	170756	rs2568956	329443	ieu-b-102	TRUE	reported	WYslv6
1	72764430	A	G	0.0349	0.0055	2.16E-10	0.811	NA	170756	rs2568957	329443	ieu-b-102	TRUE	reported	WYslv6
1	72765116	G	A	0.0382	0.0044	2.90E-18	0.6042	NA	170756	rs2568958	329443	ieu-b-102	TRUE	reported	WYslv6
1	72811904	G	T	0.0373	0.0044	2.72E-17	0.6044	NA	170756	rs1120994	329443	ieu-b-102	TRUE	reported	WYslv6
1	72812249	A	G	0.0381	0.0044	3.75E-18	0.6051	NA	170756	rs2568960	329443	ieu-b-102	TRUE	reported	WYslv6
1	72812324	G	A	0.038	0.0044	4.83E-18	0.6037	NA	170756	rs2815753	329443	ieu-b-102	TRUE	reported	WYslv6
1	72812440	G	A	0.0374	0.0044	1.66E-17	0.6041	NA	170756	rs2815752	329443	ieu-b-102	TRUE	reported	WYslv6
1	72812657	A	G	0.038	0.0044	4.49E-18	0.6044	NA	170756	rs2613494	329443	ieu-b-102	TRUE	reported	WYslv6
1	72812747	C	T	0.0382	0.0044	3.48E-18	0.6042	NA	170756	rs2568961	329443	ieu-b-102	TRUE	reported	WYslv6
1	72813129	C	T	0.0381	0.0044	3.82E-18	0.6043	NA	170756	rs1460943	329443	ieu-b-102	TRUE	reported	WYslv6
1	72813218	C	A	0.038	0.0044	4.48E-18	0.6038	NA	170756	rs1432639	329443	ieu-b-102	TRUE	reported	WYslv6
1	72813256	A	T	0.0381	0.0044	4.33E-18	0.6044	NA	170756	rs1460942	329443	ieu-b-102	TRUE	reported	WYslv6
1	72814617	G	A	0.0325	0.0055	2.56E-09	0.8054	NA	170756	rs1460940	329443	ieu-b-102	TRUE	reported	WYslv6
1	72814783	A	G	0.033	0.0054	1.19E-09	0.8053	NA	170756	rs2815749	329443	ieu-b-102	TRUE	reported	WYslv6
1	72816147	G	A	0.0333	0.0054	9.69E-10	0.8053	NA	170756	rs2815748	329443	ieu-b-102	TRUE	reported	WYslv6
1	72819612	T	C	0.0369	0.0044	4.62E-17	0.5995	NA	170756	rs2012697	329443	ieu-b-102	TRUE	reported	WYslv6
1	72819884	T	C	0.0326	0.0055	2.39E-09	0.8058	NA	170756	rs2613500	329443	ieu-b-102	TRUE	reported	WYslv6
1	72821126	G	T	0.0327	0.0055	1.98E-09	0.8057	NA	170756	rs2815747	329443	ieu-b-102	TRUE	reported	WYslv6
1	72823713	C	T	0.0369	0.0044	4.42E-17	0.5991	NA	170756	rs990871	329443	ieu-b-102	TRUE	reported	WYslv6
1	72824855	G	A	0.0367	0.0044	7.31E-17	0.5994	NA	170756	rs6661921	329443	ieu-b-102	TRUE	reported	WYslv6
1	72824860	C	T	0.0367	0.0044	7.87E-17	0.5994	NA	170756	rs6687024	329443	ieu-b-102	TRUE	reported	WYslv6
1	72825144	A	T	0.0392	0.0045	1.50E-18	0.6154	NA	170756	rs9944027	329443	ieu-b-102	TRUE	reported	WYslv6
1	72828221	T	C	0.0372	0.0044	1.93E-17	0.5988	NA	170756	rs1088994	329443	ieu-b-102	TRUE	reported	WYslv6
1	72829680	A	T	0.0373	0.0044	1.73E-17	0.5988	NA	170756	rs1120995	329443	ieu-b-102	TRUE	reported	WYslv6
1	72833582	C	T	0.0332	0.0054	1.09E-09	0.8053	NA	170756	rs2613508	329443	ieu-b-102	TRUE	reported	WYslv6
1	72834503	A	G	0.0332	0.0054	9.95E-10	0.8053	NA	170756	rs2613507	329443	ieu-b-102	TRUE	reported	WYslv6
1	72835410	C	T	0.0333	0.0054	8.84E-10	0.8049	NA	170756	rs2613505	329443	ieu-b-102	TRUE	reported	WYslv6
1	72835740	G	A	0.0333	0.0054	9.90E-10	0.8052	NA	170756	rs2613504	329443	ieu-b-102	TRUE	reported	WYslv6
1	72836456	A	T	0.0374	0.0044	1.44E-17	0.599	NA	170756	rs1841499	329443	ieu-b-102	TRUE	reported	WYslv6
1	72837239	T	C	0.037	0.0043	1.59E-17	0.5294	NA	170756	rs1403039	329443	ieu-b-102	TRUE	reported	WYslv6
1	72837490	C	T	0.0375	0.0044	1.27E-17	0.5989	NA	170756	rs1120995	329443	ieu-b-102	TRUE	reported	WYslv6
1	72837500	C	T	0.0375	0.0044	1.21E-17	0.5984	NA	170756	rs1120995	329443	ieu-b-102	TRUE	reported	WYslv6
1	72838406	G	A	0.0373	0.0044	1.66E-17	0.5992	NA	170756	rs9130285	329443	ieu-b-102	TRUE	reported	WYslv6
1	72838529	A	G	0.0332	0.0054	1.12E-09	0.8053	NA	170756	rs1993709	329443	ieu-b-102	TRUE	reported	WYslv6
1	72839774	C	A	0.0332	0.0054	1.11E-09	0.8053	NA	170756	rs2613503	329443	ieu-b-102	TRUE	reported	WYslv6
1	72846190	A	G	0.0325	0.0054	2.14E-09	0.8011	NA	170756	rs1599339	329443	ieu-b-102	TRUE	reported	WYslv6
1	72846460	C	G	0.0308	0.0054	1.42E-08	0.7998	NA	170756	rs1599337	329443	ieu-b-102	TRUE	reported	WYslv6
1	72847620	A	C	0.0309	0.0044	1.76E-12	0.5848	NA	170756	rs1599335	329443	ieu-b-102	TRUE	reported	WYslv6
1	72848682	A	T	0.0307	0.0044	3.72E-12	0.6044	NA	170756	rs2815781	329443	ieu-b-102	TRUE	reported	WYslv6
1	72849086	G	A	0.0307	0.0044	2.36E-12	0.5868	NA	170756	rs1841493	329443	ieu-b-102	TRUE	reported	WYslv6
1	72850392	T	C	0.0307	0.0044	3.87E-12	0.6038	NA	170756	rs2613480	329443	ieu-b-102	TRUE	reported	WYslv6
1	72850425	G	A	0.0307	0.0044	3.87E-12	0.6038	NA	170756	rs2815778	329443	ieu-b-102	TRUE	reported	WYslv6
1	72852773	G	A	0.0305	0.0044	3.21E-12	0.5837	NA	170756	rs1961767	329443	ieu-b-102	TRUE	reported	WYslv6
1	72852995	G	A	0.0305	0.0044	3.90E-12	0.5848	NA	170756	rs1072882	329443	ieu-b-102	TRUE	reported	WYslv6
1	72853409	C	T	0.0321	0.0054	2.83E-09	0.8013	NA	170756	rs2815775	329443	ieu-b-102	TRUE	reported	WYslv6
1	72856428	G	A	0.0285	0.0044	1.25E-10	0.6095	NA	170756	rs4366274	329443	ieu-b-102	TRUE	reported	WYslv6
1	72856991	A	G	0.0298	0.0054	2.76E-08	0.7849	NA	170756	rs2613476	329443	ieu-b-102	TRUE	reported	WYslv6
1	72861567	A	T	0.0319	0.0054	3.48E-09	0.8012	NA	170756	rs1460939	329443	ieu-b-102	TRUE	reported	WYslv6
1	72863102	C	T	0.0303	0.0044	4.44E-12	0.5858	NA	170756	rs1824530	329443	ieu-b-102	TRUE	reported	WYslv6
1	72863906	G	A	0.0306	0.0044	2.15E-12	0.5853	NA	170756	rs4650133	329443	ieu-b-102	TRUE	reported	WYslv6
1	72865045	A	G	0.0312	0.0044	1.17E-12	0.604	NA	170756	rs1903372	329443	ieu-b-102	TRUE	reported	WYslv6
1	72866574	T	G	0.0302	0.0044	5.94E-12	0.5844	NA	170756	rs1599334	329443	ieu-b-102	TRUE	reported	WYslv6
1	72866847	A	G	0.0312	0.0044	1.20E-12	0.6039	NA	170756	rs2815767	329443	ieu-b-102	TRUE	reported	WYslv6
1	72866971	G	A	0.0312	0.0044	1.18E-12	0.604	NA	170756	rs2613473	329443	ieu-b-102	TRUE	reported	WYslv6
1	72870881	G	T	0.0291	0.0045	7.77E-11	0.6376	NA	170756	rs290697	329443	ieu-b-102	TRUE	reported	WYslv6
1	72871160	C	T	0.0303	0.0044	4.78E-12	0.585	NA	170756	rs1120996	329443	ieu-b-102	TRUE	reported	WYslv6
1	72874257	C	T	0.0299	0.0044	8.64E-12	0.5851	NA	170756	rs1838662	329443	ieu-b-102	TRUE	reported	WYslv6
1	72874456	A	G	0.0323	0.0054	2.11E-09	0.801	NA	170756	rs2590943	329443	ieu-b-102	TRUE	reported	WYslv6
1	72875201	A	T	0.0299	0.0044	9.75E-12	0.5851	NA	170756	rs1078933	329443	ieu-b-102	TRUE	reported	WYslv6
1	72875646	T	A	0.0299	0.0044	8.66E-12	0.5851	NA	170756	rs1120996	329443	ieu-b-102	TRUE	reported	WYslv6
1	72876286	G	A	0.0297	0.0044	2.30E-11	0.6292	NA	170756	rs290703	329443	ieu-b-102	TRUE	reported	WYslv6
1	72876346	A	G	0.0299	0.0044	1.70E-11	0.6291	NA	170756	rs290702	329443	ieu-b-102	TRUE	reported	WYslv6
1	72882343	C	G	0.0308	0.0044	1.75E-12	0.5851	NA	170756	rs7532281	329443	ieu-b-102	TRUE	reported	WYslv6
1	72883303	A	G	0.0327	0.0054	1.40E-09	0.801	NA	170756	rs1120996	329443	ieu-b-102	TRUE	reported	WYslv6
1	72884712	G	A	0.0309	0.0044	1.50E-12	0.5853	NA	170756	rs1078933	329443	ieu-b-102	TRUE	reported	WYslv6
1	72885087	C	T	0.031	0.0044	1.18E-12	0.5849	NA	170756	rs1120996	329443	ieu-b-102	TRUE	reported	WYslv6
1	72885281	T	G	-0.0326	0.0054	1.52E-09	0.1991	NA	170756	rs2590942	329443	ieu-b-102	TRUE	reported	WYslv6
1	72885969	G	C	0.0307	0.0044	2.11E-12	0.5852	NA	170756	rs7549534	329443	ieu-b-102	TRUE	reported	WYslv6
1	72886559	C	G	0.031	0.0044	1.16E-12	0.5853	NA	170756	rs6673910	329443	ieu-b-102	TRUE	reported	WYslv6
1	72887188	C	T	-0.0328	0.0054	1.19E-09	0.199	NA	170756	rs2492971	329443	ieu-b-102	TRUE	reported	WYslv6
1	72887750	C	T	0.0311	0.0044	1.06E-12	0.5851	NA	170756	rs1088995	329443	ieu-b-102	TRUE	reported	WYslv6
1	72887886	G	A	0.0311	0.0044	1.06E-12	0.5851	NA	170756	rs1088995	329443	ieu-b-102	TRUE	reported	WYslv6
1	72888654	C	T	-0.0327	0.0054	1.35E-09	0.1988	NA	170756	rs4649955	329443	ieu-b-102	TRUE	reported	WYslv6

1	72889878	T	C	-0.0327	0.0054	1.35E-09	0.1988	NA	170756	rs2815766	329443	ieu-b-102	TRUE	reported	WYslv6
1	72890548	T	A	0.0309	0.0044	1.39E-12	0.5854	NA	170756	rs1088995	329443	ieu-b-102	TRUE	reported	WYslv6
1	72891435	G	A	-0.0327	0.0054	1.34E-09	0.1988	NA	170756	rs2797104	329443	ieu-b-102	TRUE	reported	WYslv6
1	72893648	C	A	0.0312	0.0044	1.08E-12	0.5829	NA	170756	rs6424469	329443	ieu-b-102	TRUE	reported	WYslv6
1	72893683	T	G	0.0311	0.0044	1.21E-12	0.5829	NA	170756	rs6424470	329443	ieu-b-102	TRUE	reported	WYslv6
1	72895551	C	T	0.0309	0.0044	1.45E-12	0.5853	NA	170756	rs2166314	329443	ieu-b-102	TRUE	reported	WYslv6
1	72896654	C	T	0.0309	0.0044	1.37E-12	0.5853	NA	170756	rs2422221	329443	ieu-b-102	TRUE	reported	WYslv6
1	72897106	A	G	0.031	0.0044	1.26E-12	0.5853	NA	170756	rs980243	329443	ieu-b-102	TRUE	reported	WYslv6
1	72897985	C	A	0.0309	0.0044	1.41E-12	0.5853	NA	170756	rs1445582	329443	ieu-b-102	TRUE	reported	WYslv6
1	72899059	G	A	-0.0327	0.0054	1.41E-09	0.1988	NA	170756	rs782257	329443	ieu-b-102	TRUE	reported	WYslv6
1	72899359	A	G	0.031	0.0044	1.18E-12	0.5851	NA	170756	rs1445581	329443	ieu-b-102	TRUE	reported	WYslv6
1	72901141	T	G	-0.0327	0.0054	1.36E-09	0.1988	NA	170756	rs992956	329443	ieu-b-102	TRUE	reported	WYslv6
1	72901515	C	T	-0.0326	0.0054	1.51E-09	0.1989	NA	170756	rs1373914	329443	ieu-b-102	TRUE	reported	WYslv6
1	72901573	T	C	0.031	0.0044	1.30E-12	0.5858	NA	170756	rs1373913	329443	ieu-b-102	TRUE	reported	WYslv6
1	72901752	T	G	0.031	0.0044	1.34E-12	0.5858	NA	170756	rs6424471	329443	ieu-b-102	TRUE	reported	WYslv6
1	72902623	G	A	-0.0315	0.0044	7.60E-13	0.3956	NA	170756	rs1084441	329443	ieu-b-102	TRUE	reported	WYslv6
1	72902776	T	A	-0.0315	0.0044	7.59E-13	0.3956	NA	170756	rs1084440	329443	ieu-b-102	TRUE	reported	WYslv6
1	72902919	T	G	-0.0315	0.0044	7.45E-13	0.3955	NA	170756	rs1200955	329443	ieu-b-102	TRUE	reported	WYslv6
1	72902974	T	C	0.0309	0.0044	1.35E-12	0.5858	NA	170756	rs1596762	329443	ieu-b-102	TRUE	reported	WYslv6
1	72903118	A	G	0.031	0.0044	1.26E-12	0.5858	NA	170756	rs7531797	329443	ieu-b-102	TRUE	reported	WYslv6
1	72904244	C	G	0.031	0.0044	1.34E-12	0.5857	NA	170756	rs1203594	329443	ieu-b-102	TRUE	reported	WYslv6
1	72906385	G	A	-0.0326	0.0054	1.65E-09	0.1988	NA	170756	rs782240	329443	ieu-b-102	TRUE	reported	WYslv6
1	72906443	A	G	0.0304	0.0044	3.88E-12	0.586	NA	170756	rs1822977	329443	ieu-b-102	TRUE	reported	WYslv6
1	72906786	A	G	0.0312	0.0044	1.06E-12	0.5862	NA	170756	rs2121056	329443	ieu-b-102	TRUE	reported	WYslv6
1	72908137	T	C	-0.0315	0.0044	8.47E-13	0.3956	NA	170756	rs782239	329443	ieu-b-102	TRUE	reported	WYslv6
1	72909011	A	G	0.0303	0.0044	4.68E-12	0.5859	NA	170756	rs7534013	329443	ieu-b-102	TRUE	reported	WYslv6
1	72909435	C	T	0.0309	0.0044	1.36E-12	0.5857	NA	170756	rs4649956	329443	ieu-b-102	TRUE	reported	WYslv6
1	72910098	T	C	0.03	0.0044	8.18E-12	0.5859	NA	170756	rs1202613	329443	ieu-b-102	TRUE	reported	WYslv6
1	72910182	T	G	0.0308	0.0044	1.85E-12	0.5862	NA	170756	rs1088995	329443	ieu-b-102	TRUE	reported	WYslv6
1	72911317	T	A	0.0304	0.0044	4.14E-12	0.5859	NA	170756	rs1405566	329443	ieu-b-102	TRUE	reported	WYslv6
1	72911829	C	T	-0.0314	0.0044	9.22E-13	0.3959	NA	170756	rs782252	329443	ieu-b-102	TRUE	reported	WYslv6
1	72912745	G	A	0.0304	0.0044	4.10E-12	0.5862	NA	170756	rs2422222	329443	ieu-b-102	TRUE	reported	WYslv6
1	72913499	C	A	0.031	0.0044	1.31E-12	0.5858	NA	170756	rs7535539	329443	ieu-b-102	TRUE	reported	WYslv6
1	72915804	G	A	-0.0291	0.0045	6.95E-11	0.3619	NA	170756	rs782246	329443	ieu-b-102	TRUE	reported	WYslv6
1	72915844	G	A	-0.0325	0.0054	1.79E-09	0.1987	NA	170756	rs782245	329443	ieu-b-102	TRUE	reported	WYslv6
1	72916124	G	A	0.031	0.0044	1.30E-12	0.5857	NA	170756	rs1120996	329443	ieu-b-102	TRUE	reported	WYslv6
1	72916451	G	A	-0.0291	0.0045	6.99E-11	0.3619	NA	170756	rs782244	329443	ieu-b-102	TRUE	reported	WYslv6
1	72916538	C	T	-0.0315	0.0044	8.72E-13	0.3955	NA	170756	rs782243	329443	ieu-b-102	TRUE	reported	WYslv6
1	72916574	C	T	-0.0324	0.0054	1.91E-09	0.1987	NA	170756	rs782242	329443	ieu-b-102	TRUE	reported	WYslv6
1	72920283	A	G	0.0303	0.0044	4.69E-12	0.586	NA	170756	rs2121055	329443	ieu-b-102	TRUE	reported	WYslv6
1	72921381	G	A	0.0305	0.0044	3.33E-12	0.5854	NA	170756	rs1275939	329443	ieu-b-102	TRUE	reported	WYslv6
1	72924057	C	T	-0.0327	0.0054	1.41E-09	0.1987	NA	170756	rs782221	329443	ieu-b-102	TRUE	reported	WYslv6
1	72924689	C	A	0.0305	0.0044	3.79E-12	0.5859	NA	170756	rs4650138	329443	ieu-b-102	TRUE	reported	WYslv6
1	72924810	A	G	-0.03	0.0045	1.72E-11	0.3703	NA	170756	rs782219	329443	ieu-b-102	TRUE	reported	WYslv6
1	72924848	C	T	0.0311	0.0044	1.10E-12	0.5858	NA	170756	rs4650139	329443	ieu-b-102	TRUE	reported	WYslv6
1	72925397	G	C	-0.0327	0.0054	1.39E-09	0.1989	NA	170756	rs782218	329443	ieu-b-102	TRUE	reported	WYslv6
1	72926855	A	G	-0.0326	0.0054	1.51E-09	0.1985	NA	170756	rs6177177	329443	ieu-b-102	TRUE	reported	WYslv6
1	72927346	G	A	0.0311	0.0044	1.02E-12	0.5858	NA	170756	rs7523080	329443	ieu-b-102	TRUE	reported	WYslv6
1	72928467	T	C	-0.0292	0.0045	6.30E-11	0.3619	NA	170756	rs1838658	329443	ieu-b-102	TRUE	reported	WYslv6
1	72930635	T	C	0.0313	0.0044	7.91E-13	0.5857	NA	170756	rs1203576	329443	ieu-b-102	TRUE	reported	WYslv6
1	72932316	G	T	-0.0327	0.0054	1.49E-09	0.1983	NA	170756	rs1664410	329443	ieu-b-102	TRUE	reported	WYslv6
1	72934077	G	C	-0.0292	0.0045	6.09E-11	0.3615	NA	170756	rs782237	329443	ieu-b-102	TRUE	reported	WYslv6
1	72934826	A	G	-0.0316	0.0044	6.98E-13	0.3953	NA	170756	rs782236	329443	ieu-b-102	TRUE	reported	WYslv6
1	72936345	C	T	0.0285	0.0044	1.22E-10	0.6189	NA	170756	rs1088995	329443	ieu-b-102	TRUE	reported	WYslv6
1	72936795	C	T	0.0296	0.0044	1.82E-11	0.611	NA	170756	rs1822981	329443	ieu-b-102	TRUE	reported	WYslv6
1	72937078	C	G	0.0301	0.0045	1.31E-11	0.6298	NA	170756	rs949773	329443	ieu-b-102	TRUE	reported	WYslv6
1	72937523	C	T	0.0301	0.0045	1.32E-11	0.6298	NA	170756	rs1807758	329443	ieu-b-102	TRUE	reported	WYslv6
1	72937842	A	G	0.0312	0.0044	8.57E-13	0.586	NA	170756	rs7530496	329443	ieu-b-102	TRUE	reported	WYslv6
1	72938027	G	A	0.0302	0.0045	1.22E-11	0.63	NA	170756	rs1088995	329443	ieu-b-102	TRUE	reported	WYslv6
1	72938249	A	C	0.0289	0.0044	7.84E-11	0.6197	NA	170756	rs1049349	329443	ieu-b-102	TRUE	reported	WYslv6
1	72938495	C	T	-0.0328	0.0054	1.24E-09	0.1984	NA	170756	rs796698	329443	ieu-b-102	TRUE	reported	WYslv6
1	72939855	A	C	0.0294	0.0045	4.60E-11	0.6385	NA	170756	rs7536869	329443	ieu-b-102	TRUE	reported	WYslv6
1	72940273	A	G	0.0376	0.0045	4.24E-17	0.6299	NA	170756	rs1078934	329443	ieu-b-102	TRUE	reported	WYslv6
1	72944617	A	T	0.0298	0.0044	1.50E-11	0.6111	NA	170756	rs7550173	329443	ieu-b-102	TRUE	reported	WYslv6
1	72944714	A	G	0.0313	0.0044	7.51E-13	0.5861	NA	170756	rs1120997	329443	ieu-b-102	TRUE	reported	WYslv6
1	72945128	A	G	-0.033	0.0054	1.05E-09	0.1982	NA	170756	rs782214	329443	ieu-b-102	TRUE	reported	WYslv6
1	72945666	C	T	-0.0297	0.0045	3.65E-11	0.3616	NA	170756	rs782212	329443	ieu-b-102	TRUE	reported	WYslv6
1	72946328	G	A	-0.033	0.0054	1.05E-09	0.1982	NA	170756	rs782211	329443	ieu-b-102	TRUE	reported	WYslv6
1	72947342	G	A	0.0296	0.0044	1.99E-11	0.6114	NA	170756	rs1203346	329443	ieu-b-102	TRUE	reported	WYslv6
1	72947430	T	C	-0.0303	0.0045	9.70E-12	0.3698	NA	170756	rs782210	329443	ieu-b-102	TRUE	reported	WYslv6
1	72949290	G	A	-0.0329	0.0054	1.09E-09	0.1982	NA	170756	rs707444	329443	ieu-b-102	TRUE	reported	WYslv6
1	72949748	A	T	0.0314	0.0044	7.07E-13	0.5861	NA	170756	rs4649957	329443	ieu-b-102	TRUE	reported	WYslv6
1	72951344	C	T	0.0314	0.0044	6.83E-13	0.586	NA	170756	rs1822979	329443	ieu-b-102	TRUE	reported	WYslv6
1	72953022	T	C	-0.0303	0.0045	9.60E-12	0.3699	NA	170756	rs782208	329443	ieu-b-102	TRUE	reported	WYslv6
1	72953251	G	A	-0.0303	0.0045	1.05E-11	0.3699	NA	170756	rs782207	329443	ieu-b-102	TRUE	reported	WYslv6
1	72954611	A	C	-0.0303	0.0045	1.05E-11	0.3695	NA	170756	rs813004	329443	ieu-b-102	TRUE	reported	WYslv6
1	72956535	A	T	0.028	0.0044	2.49E-10	0.6104	NA	170756	rs1088995	329443	ieu-b-102	TRUE	reported	WYslv6
1	73281986	G	A	-0.0241	0.0044	3.73E-08	0.5125	NA	170756	rs1256602	329443	ieu-b-102	TRUE	reported	WYslv6
1	73288601	A	T	-0.0237	0.0043	4.66E-08	0.5115	NA	170756	rs1088999	329443	ieu-b-102	TRUE	reported	WYslv6
1	73305782	G	A	0.0255	0.0044	6.10E-09	0.4533	NA	170756	rs662655	329443	ieu-b-102	TRUE	reported	WYslv6
1	73307627	C	T	-0.0269	0.0043	4.75E-10	0.4788	NA	170756	rs2841192	329443	ieu-b-102	TRUE	reported	WYslv6
1	73309082	C	T	0.0272	0.0043	3.24E-10	0.5176	NA	170756	rs1212994	329443	ieu-b-102	TRUE	reported	WYslv6
1	73310138	C	T	-0.0269	0.0043	5.04E-10	0.479	NA	170756	rs940917	329443	ieu-b-102	TRUE	reported	WYslv6
1	73311542	G	A	-0.0265	0.0043	8.61E-10	0.4791	NA	170756	rs7546928	329443	ieu-b-102	TRUE	reported	WYslv6
1	73313762	G	A	-0.0264	0.0043	9.19E-10	0.4788	NA	170756	rs9999123	329443	ieu-b-102	TRUE	reported	WYslv6
1	73314473	C	T	-0.0265	0.0043	7.83E-10	0.4784	NA	170756	rs2901713	329443	ieu-b-102	TRUE	reported	WYslv6
1	73314547	C	T	-0.0264	0.0043	9.12E-10	0.4788	NA	170756	rs1525979	329443	ieu-b-102	TRUE	reported	WYslv6
1	73314894	T	A	-0.0265	0.0043	8.62E-10	0.4788	NA	170756	rs1525978	329443	ieu-b-102	TRUE	reported	WYslv6
1	73318565	C	T	-0.0265	0.0043	6.95E-10	0.4781	NA	170756	rs1088999	329443	ieu-b-102	TRUE	reported	WYslv6
1	73318667	T	C	-0.0265	0.0043	7.36E-10	0.4785	NA	170756	rs1206649	329443	ieu-b-102	TRUE	reported	WYslv6
1	73321797	G	A	-0.0265	0.0043	7.33E-10	0.4783	NA	170756	rs7519251	329443	ieu-b-102	TRUE	reported	WYslv6
1	73331948	A	G	-0.0265	0.0043	6.87E-10	0.4826	NA	170756	rs6424496	329443	ieu-b-102	TRUE	reported	WYslv6
1	73332211	C	A	-0.0263	0.0043	9.62E-10	0.4774	NA	170756	rs1010292	329443	ieu-b-102	TRUE	reported	WYslv6
1	73333163	A	G	-0.0262	0.0043	1.15E-09	0.4814	NA	170756	rs1121007	329443	ieu-b-102	TRUE	reported	WYslv6
1	73334735	G	A	-0.0263	0.0043	1.02E-09	0.4775	NA	170756	rs1525996	329443	ieu-b-102	TRUE	reported	WYslv6
1	73336263	A	C	-0.0262	0.0043	1.13E-09	0.4776	NA	170756	rs1121008	329443	ieu-b-102	TRUE	reported	WYslv6
1	73338414	T	A	-0.026	0.0043	1.43E-09	0.4792	NA	170756	rs1185133	329443	ieu-b-102	TRUE	reported	WYslv6
1	73343966	G	A	-0.0262	0.0043	1.12E-09	0.4794	NA	170756	rs1121008	329443	ieu-b-102	TRUE	reported	WYslv6
1	73344155	G	C	-0.0266	0.0043	6.70E-10	0.4801	NA	170756	rs1256525	329443	ieu-b-102	TRUE	reported	WYslv6
1	73348484	G	A	-0.0268	0.0043	5.40E-10	0.4789	NA	170756	rs1405051	329443	ieu-b-102	TRUE	reported	WYslv6
1	73348889	C	A	-0.0266	0.0043	6.63E-10	0.4802	NA	170756	rs6679116	329443	ieu-b-102	TRUE	reported	WYslv6
1	73352029	C	A	-0.0265	0.0043	7.31E-10	0.4802	NA	170756	rs6689491	329443	ieu-b-102	TRUE	reported	WYslv6
1	73353328	A	T	-0.0265	0.0043	8.38E-10	0.4789	NA	170756	rs6657082	329443	ieu-b-102	TRUE	reported	WYslv6
1	73361777	G	C	-0.0266	0.0043	6.86E-10	0.4801	NA	170756	rs1121008	329443	ieu-b-102	TRUE	reported	WYslv6
1	73362466	T	G	-0.0266	0.0043	6.86E-10	0.4801	NA	170756	rs1121008	329443	ieu-b-102	TRUE	reported	WYslv6
1	73362999	A	C	-0.0266	0.0043	6.86E-10	0.4801	NA	170756	rs1464952	329443	ieu-b-102	TRUE	reported	WYslv6
1	73366368	G	A	-0.0274	0.0043	2.75E-10	0.4763	NA	170756	rs1088999	329443	ieu-b-102	TRUE	reported	WYslv6

1	73369708	A	G	-0.0265	0.0043	9.11E-10	0.4746	NA	170756	rs1272736	329443	ieu-b-102	TRUE	reported	WYslv6
1	73371702	T	C	-0.0264	0.0043	8.73E-10	0.4782	NA	170756	rs7523396	329443	ieu-b-102	TRUE	reported	WYslv6
1	73371816	T	C	-0.0264	0.0043	8.96E-10	0.4782	NA	170756	rs7525762	329443	ieu-b-102	TRUE	reported	WYslv6
1	73376127	G	A	-0.0263	0.0043	9.45E-10	0.4782	NA	170756	rs7529752	329443	ieu-b-102	TRUE	reported	WYslv6
1	73382356	C	T	-0.0263	0.0043	9.61E-10	0.4782	NA	170756	rs9970090	329443	ieu-b-102	TRUE	reported	WYslv6
1	73388521	A	G	-0.0257	0.0043	2.29E-09	0.4764	NA	170756	rs4330880	329443	ieu-b-102	TRUE	reported	WYslv6
1	73396527	C	T	-0.0258	0.0043	2.09E-09	0.4781	NA	170756	rs4579707	329443	ieu-b-102	TRUE	reported	WYslv6
1	73402002	G	A	-0.0257	0.0043	2.40E-09	0.4784	NA	170756	rs4526565	329443	ieu-b-102	TRUE	reported	WYslv6
1	73402010	C	G	-0.0257	0.0043	2.31E-09	0.4784	NA	170756	rs4271155	329443	ieu-b-102	TRUE	reported	WYslv6
1	73402421	C	G	-0.0258	0.0043	2.10E-09	0.4783	NA	170756	rs4272573	329443	ieu-b-102	TRUE	reported	WYslv6
1	73408437	G	A	-0.0257	0.0043	2.19E-09	0.4783	NA	170756	rs7536364	329443	ieu-b-102	TRUE	reported	WYslv6
1	73408983	T	C	-0.0259	0.0043	1.75E-09	0.478	NA	170756	rs6424507	329443	ieu-b-102	TRUE	reported	WYslv6
1	73412289	G	T	0.0275	0.0043	2.43E-10	0.5176	NA	170756	rs4589057	329443	ieu-b-102	TRUE	reported	WYslv6
1	73419657	C	T	-0.0259	0.0043	1.77E-09	0.4779	NA	170756	rs4584349	329443	ieu-b-102	TRUE	reported	WYslv6
1	73426984	C	T	0.0276	0.0043	1.34E-10	0.5165	NA	170756	rs1212237	329443	ieu-b-102	TRUE	reported	WYslv6
1	73428581	A	G	-0.0276	0.0043	1.49E-10	0.4721	NA	170756	rs4269731	329443	ieu-b-102	TRUE	reported	WYslv6
1	73429567	T	C	-0.0278	0.0043	9.89E-11	0.4738	NA	170756	rs1121009	329443	ieu-b-102	TRUE	reported	WYslv6
1	73429853	A	G	-0.0278	0.0043	1.01E-10	0.474	NA	170756	rs7516673	329443	ieu-b-102	TRUE	reported	WYslv6
1	73435629	A	G	-0.0276	0.0043	1.42E-10	0.4726	NA	170756	rs1121010	329443	ieu-b-102	TRUE	reported	WYslv6
1	73436249	T	C	0.0271	0.0043	3.09E-10	0.5195	NA	170756	rs6667006	329443	ieu-b-102	TRUE	reported	WYslv6
1	73445967	C	G	-0.0277	0.0043	1.25E-10	0.4729	NA	170756	rs6667756	329443	ieu-b-102	TRUE	reported	WYslv6
1	73446245	G	A	0.027	0.0043	4.24E-10	0.5186	NA	170756	rs4233093	329443	ieu-b-102	TRUE	reported	WYslv6
1	73446704	G	A	0.0264	0.0043	7.86E-10	0.511	NA	170756	rs1272453	329443	ieu-b-102	TRUE	reported	WYslv6
1	73446790	C	T	-0.0274	0.0043	2.08E-10	0.4713	NA	170756	rs1204286	329443	ieu-b-102	TRUE	reported	WYslv6
1	73447142	G	T	0.0241	0.0043	2.46E-08	0.4841	NA	170756	rs1231211	329443	ieu-b-102	TRUE	reported	WYslv6
1	73447821	C	T	0.028	0.0043	9.08E-11	0.517	NA	170756	rs7528791	329443	ieu-b-102	TRUE	reported	WYslv6
1	73447895	A	T	0.0278	0.0043	1.27E-10	0.516	NA	170756	rs7540420	329443	ieu-b-102	TRUE	reported	WYslv6
1	73448345	G	C	-0.0272	0.0043	2.47E-10	0.4731	NA	170756	rs1472156	329443	ieu-b-102	TRUE	reported	WYslv6
1	73449159	T	G	-0.0281	0.0043	8.80E-11	0.4721	NA	170756	rs1180326	329443	ieu-b-102	TRUE	reported	WYslv6
1	73450187	C	A	-0.0275	0.0043	1.72E-10	0.4729	NA	170756	rs6682035	329443	ieu-b-102	TRUE	reported	WYslv6
1	73450470	A	C	-0.0276	0.0043	1.71E-10	0.4707	NA	170756	rs6694433	329443	ieu-b-102	TRUE	reported	WYslv6
1	73450706	G	A	-0.0274	0.0043	2.29E-10	0.4714	NA	170756	rs6657069	329443	ieu-b-102	TRUE	reported	WYslv6
1	73458846	C	T	-0.0286	0.0043	3.43E-11	0.4576	NA	170756	rs1356054	329443	ieu-b-102	TRUE	reported	WYslv6
1	73458994	T	G	0.0241	0.0043	2.28E-08	0.504	NA	170756	rs1240599	329443	ieu-b-102	TRUE	reported	WYslv6
1	73459777	T	A	-0.0285	0.0043	3.78E-11	0.4573	NA	170756	rs1121010	329443	ieu-b-102	TRUE	reported	WYslv6
1	73460425	T	C	0.0241	0.0043	2.07E-08	0.5033	NA	170756	rs4233096	329443	ieu-b-102	TRUE	reported	WYslv6
1	73466001	T	G	0.0244	0.0043	1.35E-08	0.5029	NA	170756	rs1213774	329443	ieu-b-102	TRUE	reported	WYslv6
1	73466004	C	A	0.0245	0.0043	1.29E-08	0.5029	NA	170756	rs1211898	329443	ieu-b-102	TRUE	reported	WYslv6
1	73466797	G	A	-0.0286	0.0043	2.92E-11	0.4576	NA	170756	rs1553143	329443	ieu-b-102	TRUE	reported	WYslv6
1	73467516	A	T	0.0243	0.0043	1.50E-08	0.5033	NA	170756	rs4298682	329443	ieu-b-102	TRUE	reported	WYslv6
1	73470800	C	T	0.0241	0.0043	1.90E-08	0.5032	NA	170756	rs3447743	329443	ieu-b-102	TRUE	reported	WYslv6
1	73471633	C	A	0.0241	0.0043	2.07E-08	0.5037	NA	170756	rs4344273	329443	ieu-b-102	TRUE	reported	WYslv6
1	73483214	A	C	0.0242	0.0043	1.80E-08	0.5033	NA	170756	rs1211835	329443	ieu-b-102	TRUE	reported	WYslv6
1	73484026	T	G	-0.0289	0.0043	1.82E-11	0.4575	NA	170756	rs1209744	329443	ieu-b-102	TRUE	reported	WYslv6
1	73489405	C	T	0.0243	0.0043	1.48E-08	0.5031	NA	170756	rs1212661	329443	ieu-b-102	TRUE	reported	WYslv6
1	73489496	A	G	0.0243	0.0043	1.50E-08	0.5031	NA	170756	rs1211916	329443	ieu-b-102	TRUE	reported	WYslv6
1	73497953	T	C	0.0242	0.0043	1.66E-08	0.5031	NA	170756	rs1121010	329443	ieu-b-102	TRUE	reported	WYslv6
1	73498942	C	G	0.0243	0.0043	1.44E-08	0.5035	NA	170756	rs4592217	329443	ieu-b-102	TRUE	reported	WYslv6
1	73507348	C	G	-0.0293	0.0043	1.38E-11	0.4578	NA	170756	rs4559446	329443	ieu-b-102	TRUE	reported	WYslv6
1	73507701	A	G	-0.0294	0.0043	1.32E-11	0.4578	NA	170756	rs6424508	329443	ieu-b-102	TRUE	reported	WYslv6
1	73509067	G	A	-0.0289	0.0043	2.71E-11	0.4578	NA	170756	rs1088999	329443	ieu-b-102	TRUE	reported	WYslv6
1	73513786	C	T	-0.0288	0.0043	3.27E-11	0.458	NA	170756	rs1121010	329443	ieu-b-102	TRUE	reported	WYslv6
1	73514129	A	G	0.0242	0.0043	1.78E-08	0.5031	NA	170756	rs1213423	329443	ieu-b-102	TRUE	reported	WYslv6
1	73515194	G	A	0.024	0.0043	2.21E-08	0.5024	NA	170756	rs1212793	329443	ieu-b-102	TRUE	reported	WYslv6
1	73515337	T	A	0.0248	0.0043	9.42E-09	0.5015	NA	170756	rs9669717	329443	ieu-b-102	TRUE	reported	WYslv6
1	73516461	C	T	0.0284	0.0043	4.33E-11	0.533	NA	170756	rs1214382	329443	ieu-b-102	TRUE	reported	WYslv6
1	73516572	C	A	-0.0289	0.0043	2.57E-11	0.4585	NA	170756	rs1121011	329443	ieu-b-102	TRUE	reported	WYslv6
1	73518904	G	A	0.0283	0.0043	4.55E-11	0.533	NA	170756	rs4604650	329443	ieu-b-102	TRUE	reported	WYslv6
1	73518965	A	C	0.0241	0.0043	1.98E-08	0.5031	NA	170756	rs3758567	329443	ieu-b-102	TRUE	reported	WYslv6
1	73520188	A	G	0.0242	0.0043	1.84E-08	0.5031	NA	170756	rs1121011	329443	ieu-b-102	TRUE	reported	WYslv6
1	73522589	C	T	0.0284	0.0043	4.13E-11	0.533	NA	170756	rs1241078	329443	ieu-b-102	TRUE	reported	WYslv6
1	73522868	C	A	-0.029	0.0044	2.81E-11	0.4574	NA	170756	rs6690611	329443	ieu-b-102	TRUE	reported	WYslv6
1	73523256	T	A	-0.029	0.0043	2.47E-11	0.4578	NA	170756	rs6657470	329443	ieu-b-102	TRUE	reported	WYslv6
1	73524225	C	A	-0.029	0.0043	2.31E-11	0.4578	NA	170756	rs1202195	329443	ieu-b-102	TRUE	reported	WYslv6
1	73524351	C	A	-0.029	0.0043	2.47E-11	0.4578	NA	170756	rs1202200	329443	ieu-b-102	TRUE	reported	WYslv6
1	73525109	A	C	0.0247	0.0043	1.03E-08	0.5025	NA	170756	rs1240922	329443	ieu-b-102	TRUE	reported	WYslv6
1	73525436	G	A	-0.0296	0.0044	1.15E-11	0.4576	NA	170756	rs1203722	329443	ieu-b-102	TRUE	reported	WYslv6
1	73525922	A	C	0.0243	0.0043	1.62E-08	0.504	NA	170756	rs3463124	329443	ieu-b-102	TRUE	reported	WYslv6
1	73527633	G	T	-0.0286	0.0043	3.30E-11	0.4572	NA	170756	rs1121011	329443	ieu-b-102	TRUE	reported	WYslv6
1	73527750	A	G	-0.0288	0.0044	3.63E-11	0.457	NA	170756	rs7554291	329443	ieu-b-102	TRUE	reported	WYslv6
1	73527852	A	G	0.0242	0.0043	1.78E-08	0.5031	NA	170756	rs1121011	329443	ieu-b-102	TRUE	reported	WYslv6
1	73528252	G	C	-0.029	0.0043	2.51E-11	0.458	NA	170756	rs7519995	329443	ieu-b-102	TRUE	reported	WYslv6
1	73529275	C	T	-0.0247	0.0043	9.46E-09	0.4917	NA	170756	rs6693460	329443	ieu-b-102	TRUE	reported	WYslv6
1	73530246	G	C	-0.029	0.0043	2.49E-11	0.4578	NA	170756	rs7526372	329443	ieu-b-102	TRUE	reported	WYslv6
1	73530815	C	T	-0.0286	0.0043	3.06E-11	0.4572	NA	170756	rs7552256	329443	ieu-b-102	TRUE	reported	WYslv6
1	73531160	C	G	-0.0286	0.0043	3.23E-11	0.457	NA	170756	rs9919151	329443	ieu-b-102	TRUE	reported	WYslv6
1	73531198	C	T	0.0242	0.0043	1.87E-08	0.5031	NA	170756	rs1121011	329443	ieu-b-102	TRUE	reported	WYslv6
1	73531286	C	T	0.0242	0.0043	1.88E-08	0.5031	NA	170756	rs1327078	329443	ieu-b-102	TRUE	reported	WYslv6
1	73532151	T	C	0.0283	0.0043	4.51E-11	0.533	NA	170756	rs4649975	329443	ieu-b-102	TRUE	reported	WYslv6
1	73532694	G	A	0.0283	0.0043	4.64E-11	0.533	NA	170756	rs1121011	329443	ieu-b-102	TRUE	reported	WYslv6
1	73533199	A	C	0.0283	0.0043	5.32E-11	0.4997	NA	170756	rs1557544	329443	ieu-b-102	TRUE	reported	WYslv6
1	73533314	T	C	0.0241	0.0043	1.92E-08	0.5031	NA	170756	rs7528197	329443	ieu-b-102	TRUE	reported	WYslv6
1	73533483	T	C	0.0284	0.0043	4.31E-11	0.5326	NA	170756	rs7528312	329443	ieu-b-102	TRUE	reported	WYslv6
1	73533746	C	A	0.0242	0.0043	1.86E-08	0.5029	NA	170756	rs1257901	329443	ieu-b-102	TRUE	reported	WYslv6
1	73533878	C	T	0.0242	0.0043	1.85E-08	0.5029	NA	170756	rs4649976	329443	ieu-b-102	TRUE	reported	WYslv6
1	73537454	A	C	0.0282	0.0043	5.26E-11	0.533	NA	170756	rs4614208	329443	ieu-b-102	TRUE	reported	WYslv6
1	73538089	G	A	0.0236	0.0043	4.08E-08	0.4999	NA	170756	rs1275512	329443	ieu-b-102	TRUE	reported	WYslv6
1	73538351	T	G	0.0282	0.0043	5.35E-11	0.533	NA	170756	rs1213419	329443	ieu-b-102	TRUE	reported	WYslv6
1	73540630	T	C	-0.0291	0.0043	2.03E-11	0.4555	NA	170756	rs6054941	329443	ieu-b-102	TRUE	reported	WYslv6
1	73540800	A	T	0.0273	0.0043	2.09E-10	0.5265	NA	170756	rs1240911	329443	ieu-b-102	TRUE	reported	WYslv6
1	73541071	T	C	0.0282	0.0043	5.83E-11	0.533	NA	170756	rs1121012	329443	ieu-b-102	TRUE	reported	WYslv6
1	73542285	T	C	0.024	0.0043	2.27E-08	0.5031	NA	170756	rs3549351	329443	ieu-b-102	TRUE	reported	WYslv6
1	73542409	C	T	0.024	0.0043	2.21E-08	0.5031	NA	170756	rs3596756	329443	ieu-b-102	TRUE	reported	WYslv6
1	73542540	A	G	0.024	0.0043	2.27E-08	0.5031	NA	170756	rs3544938	329443	ieu-b-102	TRUE	reported	WYslv6
1	73542697	T	C	0.0282	0.0043	5.66E-11	0.533	NA	170756	rs1275955	329443	ieu-b-102	TRUE	reported	WYslv6
1	73542900	A	G	0.0282	0.0043	5.33E-11	0.533	NA	170756	rs4650177	329443	ieu-b-102	TRUE	reported	WYslv6
1	73543028	G	A	0.0282	0.0043	5.53E-11	0.533	NA	170756	rs4650178	329443	ieu-b-102	TRUE	reported	WYslv6
1	73544070	C	T	0.0282	0.0043	5.57E-11	0.533	NA	170756	rs4650180	329443	ieu-b-102	TRUE	reported	WYslv6
1	73544556	A	G	0.0282	0.0043	5.28E-11	0.533	NA	170756	rs4650181	329443	ieu-b-102	TRUE	reported	WYslv6
1	73545913	G	A	0.0282	0.0043	5.29E-11	0.533	NA	170756	rs6177360	329443	ieu-b-102	TRUE	reported	WYslv6
1	73546252	G	T	0.024	0.0043	2.31E-08	0.5031	NA	170756	rs3531254	329443	ieu-b-102	TRUE	reported	WYslv6
1	73546362	T	C	0.0282	0.0043	5.63E-11	0.533	NA	170756	rs1158939	329443	ieu-b-102	TRUE	reported	WYslv6
1	73547051	A	G	0.024	0.0043	2.42E-08	0.5031	NA	170756	rs3453592	329443	ieu-b-102	TRUE	reported	WYslv6
1	73547271	T	A	0.024	0.0043	2.29E-08	0.5031	NA	170756	rs5585945	329443	ieu-b-102	TRUE	reported	WYslv6
1	73547563	T	A	0.0281	0.0043	6.36E-11	0.5319	NA	170756	rs5619339	329443	ieu-b-102	TRUE	reported	WYslv6
1	73547634	G	A	0.028	0.0043	9.06E-11	0.532	NA	170756	rs5595463	329443	ieu-b-102	TRUE	reported	WYslv6
1	73547669	G	A	0.0244	0.0043	1.53E-08	0.4995	NA	170756	rs5622483	329443	ieu-b-102	TRUE	reported	WYslv6

1	73547769	G	A	0.0237	0.0043	3.83E-08	0.5011	NA	170756	rs5583492	329443	ieu-b-102	TRUE	reported	WYslv6
1	73548232	T	C	0.0279	0.0043	9.59E-11	0.5314	NA	170756	rs7542203	329443	ieu-b-102	TRUE	reported	WYslv6
1	73548236	G	A	0.0238	0.0043	3.19E-08	0.5017	NA	170756	rs7549892	329443	ieu-b-102	TRUE	reported	WYslv6
1	73548406	G	T	0.0282	0.0043	5.33E-11	0.533	NA	170756	rs7549985	329443	ieu-b-102	TRUE	reported	WYslv6
1	73548607	T	A	0.0282	0.0043	5.33E-11	0.533	NA	170756	rs1213909	329443	ieu-b-102	TRUE	reported	WYslv6
1	73548861	T	C	0.024	0.0043	2.33E-08	0.503	NA	170756	rs1397056	329443	ieu-b-102	TRUE	reported	WYslv6
1	73549063	C	T	0.0282	0.0043	6.01E-11	0.5332	NA	170756	rs7976723	329443	ieu-b-102	TRUE	reported	WYslv6
1	73549290	T	C	0.0282	0.0043	5.67E-11	0.533	NA	170756	rs1379485	329443	ieu-b-102	TRUE	reported	WYslv6
1	73549374	G	C	0.0282	0.0043	5.65E-11	0.533	NA	170756	rs7658840	329443	ieu-b-102	TRUE	reported	WYslv6
1	73549529	G	T	0.029	0.0043	2.07E-11	0.531	NA	170756	rs1240793	329443	ieu-b-102	TRUE	reported	WYslv6
1	73549581	T	C	0.0282	0.0043	5.65E-11	0.533	NA	170756	rs1240923	329443	ieu-b-102	TRUE	reported	WYslv6
1	73549671	C	T	0.0282	0.0043	5.66E-11	0.533	NA	170756	rs1274835	329443	ieu-b-102	TRUE	reported	WYslv6
1	73549773	T	C	0.0282	0.0043	5.65E-11	0.533	NA	170756	rs1240926	329443	ieu-b-102	TRUE	reported	WYslv6
1	73549779	C	A	0.0282	0.0043	5.66E-11	0.533	NA	170756	rs1240299	329443	ieu-b-102	TRUE	reported	WYslv6
1	73549987	A	G	0.0282	0.0043	5.66E-11	0.533	NA	170756	rs1181140	329443	ieu-b-102	TRUE	reported	WYslv6
1	73550364	T	C	0.0282	0.0043	5.38E-11	0.533	NA	170756	rs4623647	329443	ieu-b-102	TRUE	reported	WYslv6
1	73550543	C	T	-0.0281	0.0043	7.56E-11	0.4543	NA	170756	rs6681060	329443	ieu-b-102	TRUE	reported	WYslv6
1	73551194	T	G	0.0284	0.0043	4.33E-11	0.533	NA	170756	rs1214218	329443	ieu-b-102	TRUE	reported	WYslv6
1	73551261	G	A	0.0241	0.0043	2.04E-08	0.5029	NA	170756	rs1213826	329443	ieu-b-102	TRUE	reported	WYslv6
1	73551310	C	T	0.0278	0.0043	1.31E-10	0.5283	NA	170756	rs1212339	329443	ieu-b-102	TRUE	reported	WYslv6
1	73551385	A	G	0.0283	0.0043	5.00E-11	0.5321	NA	170756	rs1214568	329443	ieu-b-102	TRUE	reported	WYslv6
1	73551390	T	C	0.0242	0.0043	1.91E-08	0.5021	NA	170756	rs1214225	329443	ieu-b-102	TRUE	reported	WYslv6
1	73551426	G	A	0.0283	0.0043	4.53E-11	0.5322	NA	170756	rs1213830	329443	ieu-b-102	TRUE	reported	WYslv6
1	73551507	G	A	0.0242	0.0043	1.83E-08	0.5029	NA	170756	rs1213833	329443	ieu-b-102	TRUE	reported	WYslv6
1	73551749	A	G	0.0241	0.0043	2.18E-08	0.5028	NA	170756	rs1214580	329443	ieu-b-102	TRUE	reported	WYslv6
1	73551763	T	A	0.0282	0.0043	5.39E-11	0.5328	NA	170756	rs1214237	329443	ieu-b-102	TRUE	reported	WYslv6
1	73551845	T	C	0.0282	0.0043	5.54E-11	0.533	NA	170756	rs1214241	329443	ieu-b-102	TRUE	reported	WYslv6
1	73552005	G	A	0.0291	0.0043	1.90E-11	0.5306	NA	170756	rs1213921	329443	ieu-b-102	TRUE	reported	WYslv6
1	73552100	G	A	0.0283	0.0043	5.22E-11	0.533	NA	170756	rs4650182	329443	ieu-b-102	TRUE	reported	WYslv6
1	73552107	T	C	-0.0281	0.0043	7.06E-11	0.4545	NA	170756	rs1204684	329443	ieu-b-102	TRUE	reported	WYslv6
1	73552234	A	T	0.024	0.0043	2.28E-08	0.5031	NA	170756	rs2001964	329443	ieu-b-102	TRUE	reported	WYslv6
1	73552835	C	T	0.0283	0.0043	4.52E-11	0.533	NA	170756	rs7543704	329443	ieu-b-102	TRUE	reported	WYslv6
1	73552927	T	C	0.0283	0.0043	4.96E-11	0.533	NA	170756	rs7512691	329443	ieu-b-102	TRUE	reported	WYslv6
1	73552990	C	T	0.0285	0.0043	4.74E-11	0.5332	NA	170756	rs7543826	329443	ieu-b-102	TRUE	reported	WYslv6
1	73553029	A	T	0.0241	0.0043	2.03E-08	0.503	NA	170756	rs7555449	329443	ieu-b-102	TRUE	reported	WYslv6
1	73553688	C	T	0.0281	0.0043	7.07E-11	0.5343	NA	170756	rs1273160	329443	ieu-b-102	TRUE	reported	WYslv6
1	73553855	T	C	0.0282	0.0043	5.42E-11	0.533	NA	170756	rs1275240	329443	ieu-b-102	TRUE	reported	WYslv6
1	73554179	G	C	0.0283	0.0043	5.22E-11	0.5329	NA	170756	rs1214145	329443	ieu-b-102	TRUE	reported	WYslv6
1	73554520	C	T	0.0283	0.0043	4.96E-11	0.5332	NA	170756	rs1212665	329443	ieu-b-102	TRUE	reported	WYslv6
1	73554714	G	T	0.0242	0.0043	1.89E-08	0.502	NA	170756	rs6424510	329443	ieu-b-102	TRUE	reported	WYslv6
1	73554722	G	C	0.0286	0.0043	4.05E-11	0.5322	NA	170756	rs6424511	329443	ieu-b-102	TRUE	reported	WYslv6
1	73554755	G	A	0.0242	0.0043	1.98E-08	0.5021	NA	170756	rs6424512	329443	ieu-b-102	TRUE	reported	WYslv6
1	73554931	T	C	0.0282	0.0043	5.73E-11	0.533	NA	170756	rs6424513	329443	ieu-b-102	TRUE	reported	WYslv6
1	73555023	A	G	0.0282	0.0043	5.86E-11	0.533	NA	170756	rs7550849	329443	ieu-b-102	TRUE	reported	WYslv6
1	73555040	T	C	0.0282	0.0043	5.84E-11	0.533	NA	170756	rs6424514	329443	ieu-b-102	TRUE	reported	WYslv6
1	73555153	G	C	0.024	0.0043	2.35E-08	0.5031	NA	170756	rs7516218	329443	ieu-b-102	TRUE	reported	WYslv6
1	73555433	C	T	0.0282	0.0043	6.08E-11	0.533	NA	170756	rs4649977	329443	ieu-b-102	TRUE	reported	WYslv6
1	73555495	C	A	0.0282	0.0043	5.93E-11	0.533	NA	170756	rs4649978	329443	ieu-b-102	TRUE	reported	WYslv6
1	73555556	A	T	0.0282	0.0043	5.86E-11	0.533	NA	170756	rs1212019	329443	ieu-b-102	TRUE	reported	WYslv6
1	73555866	C	A	0.0282	0.0043	5.81E-11	0.533	NA	170756	rs1212782	329443	ieu-b-102	TRUE	reported	WYslv6
1	73555874	A	T	0.0282	0.0043	5.82E-11	0.533	NA	170756	rs1468208	329443	ieu-b-102	TRUE	reported	WYslv6
1	73556293	A	G	0.0281	0.0043	6.71E-11	0.5331	NA	170756	rs4649979	329443	ieu-b-102	TRUE	reported	WYslv6
1	73556698	T	A	0.0286	0.0043	3.91E-11	0.5324	NA	170756	rs4233097	329443	ieu-b-102	TRUE	reported	WYslv6
1	73556703	G	A	0.0286	0.0043	3.91E-11	0.5324	NA	170756	rs4233098	329443	ieu-b-102	TRUE	reported	WYslv6
1	73556846	C	T	0.0284	0.0043	4.77E-11	0.5313	NA	170756	rs4495635	329443	ieu-b-102	TRUE	reported	WYslv6
1	73556856	A	C	0.0283	0.0043	4.92E-11	0.5311	NA	170756	rs4562575	329443	ieu-b-102	TRUE	reported	WYslv6
1	73557256	C	T	0.0281	0.0043	7.08E-11	0.5331	NA	170756	rs4498756	329443	ieu-b-102	TRUE	reported	WYslv6
1	73557271	G	C	0.0239	0.0043	2.79E-08	0.5032	NA	170756	rs4295841	329443	ieu-b-102	TRUE	reported	WYslv6
1	73557484	G	A	0.024	0.0043	2.24E-08	0.5034	NA	170756	rs4297242	329443	ieu-b-102	TRUE	reported	WYslv6
1	73557617	C	T	0.0284	0.0043	7.00E-11	0.5284	NA	170756	rs4500267	329443	ieu-b-102	TRUE	reported	WYslv6
1	73557656	C	T	0.0279	0.0043	1.21E-10	0.5287	NA	170756	rs7961671	329443	ieu-b-102	TRUE	reported	WYslv6
1	73557976	G	T	0.028	0.0043	7.48E-11	0.5328	NA	170756	rs1121012	329443	ieu-b-102	TRUE	reported	WYslv6
1	73557990	C	T	0.028	0.0043	7.23E-11	0.5326	NA	170756	rs1089000	329443	ieu-b-102	TRUE	reported	WYslv6
1	73558100	A	T	0.028	0.0043	7.28E-11	0.5326	NA	170756	rs1089000	329443	ieu-b-102	TRUE	reported	WYslv6
1	73558176	T	A	0.0239	0.0043	2.71E-08	0.5032	NA	170756	rs7517842	329443	ieu-b-102	TRUE	reported	WYslv6
1	73558512	A	C	0.0281	0.0043	6.81E-11	0.5331	NA	170756	rs4358996	329443	ieu-b-102	TRUE	reported	WYslv6
1	73558708	C	T	0.0255	0.0043	4.26E-09	0.5024	NA	170756	rs2010513	329443	ieu-b-102	TRUE	reported	WYslv6
1	73559093	G	A	0.0281	0.0043	6.69E-11	0.5331	NA	170756	rs4310400	329443	ieu-b-102	TRUE	reported	WYslv6
1	73559174	T	C	0.0281	0.0043	6.94E-11	0.5331	NA	170756	rs4292907	329443	ieu-b-102	TRUE	reported	WYslv6
1	73559787	C	T	0.0281	0.0043	6.66E-11	0.5331	NA	170756	rs1121012	329443	ieu-b-102	TRUE	reported	WYslv6
1	73559856	C	T	0.0281	0.0043	6.57E-11	0.5331	NA	170756	rs1121012	329443	ieu-b-102	TRUE	reported	WYslv6
1	73559867	G	A	0.0239	0.0043	2.62E-08	0.5032	NA	170756	rs1214135	329443	ieu-b-102	TRUE	reported	WYslv6
1	73559968	C	T	0.0239	0.0043	2.78E-08	0.5032	NA	170756	rs1212723	329443	ieu-b-102	TRUE	reported	WYslv6
1	73559972	G	A	-0.028	0.0043	9.46E-11	0.4542	NA	170756	rs7532052	329443	ieu-b-102	TRUE	reported	WYslv6
1	73560154	C	T	0.0239	0.0043	2.86E-08	0.5032	NA	170756	rs1212730	329443	ieu-b-102	TRUE	reported	WYslv6
1	73560297	C	T	0.0281	0.0043	6.82E-11	0.5338	NA	170756	rs1453834	329443	ieu-b-102	TRUE	reported	WYslv6
1	73560811	G	T	0.0281	0.0043	6.80E-11	0.5331	NA	170756	rs4128552	329443	ieu-b-102	TRUE	reported	WYslv6
1	73560895	C	T	0.0281	0.0043	7.03E-11	0.5331	NA	170756	rs4128551	329443	ieu-b-102	TRUE	reported	WYslv6
1	73561040	T	A	0.0281	0.0043	6.92E-11	0.5331	NA	170756	rs4307522	329443	ieu-b-102	TRUE	reported	WYslv6
1	73561598	A	G	0.024	0.0043	2.36E-08	0.5034	NA	170756	rs1212093	329443	ieu-b-102	TRUE	reported	WYslv6
1	73561603	T	C	0.024	0.0043	2.42E-08	0.5034	NA	170756	rs1121012	329443	ieu-b-102	TRUE	reported	WYslv6
1	73561690	T	C	0.028	0.0043	7.78E-11	0.5331	NA	170756	rs1399725	329443	ieu-b-102	TRUE	reported	WYslv6
1	73562776	A	G	0.0239	0.0043	2.63E-08	0.5032	NA	170756	rs1212205	329443	ieu-b-102	TRUE	reported	WYslv6
1	73573941	A	G	-0.0277	0.0043	1.39E-10	0.4544	NA	170756	rs1203335	329443	ieu-b-102	TRUE	reported	WYslv6
1	73575075	C	T	0.0237	0.0043	3.46E-08	0.5023	NA	170756	rs4130885	329443	ieu-b-102	TRUE	reported	WYslv6
1	73576140	G	T	-0.0278	0.0043	1.22E-10	0.4545	NA	170756	rs6696689	329443	ieu-b-102	TRUE	reported	WYslv6
1	73578629	T	C	0.0237	0.0043	3.42E-08	0.5023	NA	170756	rs4425940	329443	ieu-b-102	TRUE	reported	WYslv6
1	73579237	G	A	0.0236	0.0043	4.06E-08	0.5029	NA	170756	rs4603080	329443	ieu-b-102	TRUE	reported	WYslv6
1	73579255	G	A	0.0235	0.0043	4.44E-08	0.5029	NA	170756	rs4233101	329443	ieu-b-102	TRUE	reported	WYslv6
1	73579642	G	A	-0.0277	0.0043	1.42E-10	0.4548	NA	170756	rs1121012	329443	ieu-b-102	TRUE	reported	WYslv6
1	73579799	T	G	0.0237	0.0043	3.58E-08	0.5024	NA	170756	rs4233103	329443	ieu-b-102	TRUE	reported	WYslv6
1	73580090	G	A	0.024	0.0043	2.32E-08	0.503	NA	170756	rs4650188	329443	ieu-b-102	TRUE	reported	WYslv6
1	73580187	T	C	0.0237	0.0043	3.56E-08	0.5023	NA	170756	rs1121012	329443	ieu-b-102	TRUE	reported	WYslv6
1	73580447	G	T	0.0237	0.0043	3.48E-08	0.5024	NA	170756	rs6662171	329443	ieu-b-102	TRUE	reported	WYslv6
1	73580964	C	T	0.0238	0.0043	3.19E-08	0.5024	NA	170756	rs7124277	329443	ieu-b-102	TRUE	reported	WYslv6
1	73581434	C	G	0.0277	0.0043	1.15E-10	0.5322	NA	170756	rs4622012	329443	ieu-b-102	TRUE	reported	WYslv6
1	73581923	G	A	0.0238	0.0043	3.23E-08	0.5024	NA	170756	rs4469676	329443	ieu-b-102	TRUE	reported	WYslv6
1	73584532	G	A	0.0277	0.0043	1.20E-10	0.5323	NA	170756	rs4477222	329443	ieu-b-102	TRUE	reported	WYslv6
1	73584790	T	C	0.0238	0.0043	3.10E-08	0.5024	NA	170756	rs4355999	329443	ieu-b-102	TRUE	reported	WYslv6
1	73584838	C	T	0.0238	0.0043	2.95E-08	0.5024	NA	170756	rs4263954	329443	ieu-b-102	TRUE	reported	WYslv6
1	73585925	T	G	0.0278	0.0043	1.02E-10	0.5322	NA	170756	rs1272478	329443	ieu-b-102	TRUE	reported	WYslv6
1	73588040	T	G	0.0238	0.0043	3.04E-08	0.5024	NA	170756	rs4543746	329443	ieu-b-102	TRUE	reported	WYslv6
1	73589164	T	A	0.0238	0.0043	2.97E-08	0.5023	NA	170756	rs6162823	329443	ieu-b-102	TRUE	reported	WYslv6
1	73589551	T	C	0.0238	0.0043	2.93E-08	0.5024	NA	170756	rs1213028	329443	ieu-b-102	TRUE	reported	WYslv6
1	73590062	G	A	0.0239	0.0043	2.89E-08	0.5021	NA	170756	rs4622015	329443	ieu-b-102	TRUE	reported	WYslv6
1	73592758	G	T	-0.0277	0.0043	1.51E-10	0.4559	NA	170756	rs4433341	329443	ieu-b-102	TRUE	reported	WYslv6
1	73593313	T	A	-0.0279	0.0043	1.11E-10	0.4551	NA	170756	rs4306085	329443	ieu-b-102	TRUE	reported	WYslv6

1	73594693	T	A	0.0278	0.0043	1.13E-10	0.5321	NA	170756	rs7511505	329443	ieu-b-102	TRUE	reported	WYslv6
1	73596365	A	G	0.0239	0.0043	2.85E-08	0.5035	NA	170756	rs7524355	329443	ieu-b-102	TRUE	reported	WYslv6
1	73596491	G	A	-0.0278	0.0043	1.17E-10	0.4546	NA	170756	rs1121013	329443	ieu-b-102	TRUE	reported	WYslv6
1	73596619	T	C	0.0238	0.0043	2.95E-08	0.5023	NA	170756	rs7526981	329443	ieu-b-102	TRUE	reported	WYslv6
1	73597329	C	T	0.0239	0.0043	2.89E-08	0.5022	NA	170756	rs4520373	329443	ieu-b-102	TRUE	reported	WYslv6
1	73598083	A	G	-0.0238	0.0043	3.07E-08	0.4852	NA	170756	rs6676166	329443	ieu-b-102	TRUE	reported	WYslv6
1	73598422	C	A	0.0238	0.0043	2.95E-08	0.502	NA	170756	rs6664103	329443	ieu-b-102	TRUE	reported	WYslv6
1	73598783	T	C	-0.0239	0.0043	2.98E-08	0.4851	NA	170756	rs1465752	329443	ieu-b-102	TRUE	reported	WYslv6
1	73599228	G	T	0.0239	0.0043	2.82E-08	0.5025	NA	170756	rs1213120	329443	ieu-b-102	TRUE	reported	WYslv6
1	73599279	G	T	0.0239	0.0043	2.74E-08	0.5022	NA	170756	rs4427361	329443	ieu-b-102	TRUE	reported	WYslv6
1	73599480	G	A	0.0239	0.0043	2.87E-08	0.5022	NA	170756	rs4428835	329443	ieu-b-102	TRUE	reported	WYslv6
1	73600264	G	T	0.0239	0.0043	2.86E-08	0.5022	NA	170756	rs7547148	329443	ieu-b-102	TRUE	reported	WYslv6
1	73600989	T	C	0.0239	0.0043	2.82E-08	0.5022	NA	170756	rs1213704	329443	ieu-b-102	TRUE	reported	WYslv6
1	73601062	C	T	0.0239	0.0043	2.64E-08	0.5018	NA	170756	rs9308110	329443	ieu-b-102	TRUE	reported	WYslv6
1	73601299	T	C	0.0238	0.0043	2.98E-08	0.5025	NA	170756	rs1213715	329443	ieu-b-102	TRUE	reported	WYslv6
1	73601419	A	G	0.0239	0.0043	2.84E-08	0.5022	NA	170756	rs2858983	329443	ieu-b-102	TRUE	reported	WYslv6
1	73601472	A	C	0.0239	0.0043	2.81E-08	0.5022	NA	170756	rs3445992	329443	ieu-b-102	TRUE	reported	WYslv6
1	73602245	T	G	-0.0238	0.0043	3.17E-08	0.4852	NA	170756	rs1204208	329443	ieu-b-102	TRUE	reported	WYslv6
1	73602381	C	T	0.0239	0.0043	2.89E-08	0.5022	NA	170756	rs4582739	329443	ieu-b-102	TRUE	reported	WYslv6
1	73602865	T	C	0.0239	0.0043	2.89E-08	0.5022	NA	170756	rs1213839	329443	ieu-b-102	TRUE	reported	WYslv6
1	73602873	C	T	0.0239	0.0043	2.89E-08	0.5022	NA	170756	rs1211963	329443	ieu-b-102	TRUE	reported	WYslv6
1	73603515	T	G	0.0239	0.0043	2.72E-08	0.5022	NA	170756	rs1272588	329443	ieu-b-102	TRUE	reported	WYslv6
1	73605887	G	A	0.0239	0.0043	2.77E-08	0.5022	NA	170756	rs1121014	329443	ieu-b-102	TRUE	reported	WYslv6
1	73606306	T	A	0.0239	0.0043	2.77E-08	0.5022	NA	170756	rs1214247	329443	ieu-b-102	TRUE	reported	WYslv6
1	73606971	G	T	0.0239	0.0043	2.84E-08	0.5022	NA	170756	rs1240716	329443	ieu-b-102	TRUE	reported	WYslv6
1	73607592	T	G	-0.0278	0.0043	1.31E-10	0.455	NA	170756	rs4366276	329443	ieu-b-102	TRUE	reported	WYslv6
1	73607966	C	G	-0.0278	0.0043	1.10E-10	0.4549	NA	170756	rs4634854	329443	ieu-b-102	TRUE	reported	WYslv6
1	73608924	G	A	-0.0238	0.0043	3.03E-08	0.4852	NA	170756	rs4564089	329443	ieu-b-102	TRUE	reported	WYslv6
1	73609004	C	T	0.0239	0.0043	2.79E-08	0.5022	NA	170756	rs1212108	329443	ieu-b-102	TRUE	reported	WYslv6
1	73609533	A	G	0.0239	0.0043	2.74E-08	0.5022	NA	170756	rs1214358	329443	ieu-b-102	TRUE	reported	WYslv6
1	73610030	T	C	0.0239	0.0043	2.62E-08	0.5022	NA	170756	rs1121014	329443	ieu-b-102	TRUE	reported	WYslv6
1	73610214	G	A	0.0239	0.0043	2.62E-08	0.5022	NA	170756	rs4391625	329443	ieu-b-102	TRUE	reported	WYslv6
1	73611050	A	C	0.0239	0.0043	2.59E-08	0.5022	NA	170756	rs1159093	329443	ieu-b-102	TRUE	reported	WYslv6
1	73611675	A	G	0.024	0.0043	2.31E-08	0.5021	NA	170756	rs6027378	329443	ieu-b-102	TRUE	reported	WYslv6
1	73611688	C	T	0.024	0.0043	2.44E-08	0.5026	NA	170756	rs5785235	329443	ieu-b-102	TRUE	reported	WYslv6
1	73611750	A	T	-0.0239	0.0043	2.94E-08	0.4852	NA	170756	rs4268306	329443	ieu-b-102	TRUE	reported	WYslv6
1	73611960	A	C	0.024	0.0043	2.57E-08	0.5022	NA	170756	rs1157626	329443	ieu-b-102	TRUE	reported	WYslv6
1	73612985	T	C	0.0239	0.0043	2.63E-08	0.5026	NA	170756	rs4570373	329443	ieu-b-102	TRUE	reported	WYslv6
1	73613006	C	T	-0.0239	0.0043	2.94E-08	0.4847	NA	170756	rs1035724	329443	ieu-b-102	TRUE	reported	WYslv6
1	73613322	C	G	0.0239	0.0043	2.77E-08	0.5029	NA	170756	rs4593761	329443	ieu-b-102	TRUE	reported	WYslv6
1	73613426	T	C	0.024	0.0043	2.56E-08	0.5029	NA	170756	rs4571921	329443	ieu-b-102	TRUE	reported	WYslv6
1	73613560	C	T	0.0239	0.0043	2.73E-08	0.5022	NA	170756	rs4452995	329443	ieu-b-102	TRUE	reported	WYslv6
1	73614081	C	T	-0.0239	0.0043	2.90E-08	0.4852	NA	170756	rs7552761	329443	ieu-b-102	TRUE	reported	WYslv6
1	73614389	C	T	-0.0242	0.0043	1.94E-08	0.4839	NA	170756	rs6424521	329443	ieu-b-102	TRUE	reported	WYslv6
1	73615026	T	G	0.024	0.0043	2.46E-08	0.5022	NA	170756	rs3419322	329443	ieu-b-102	TRUE	reported	WYslv6
1	73615127	C	T	-0.0244	0.0043	1.95E-08	0.4858	NA	170756	rs1078935	329443	ieu-b-102	TRUE	reported	WYslv6
1	73616467	T	C	0.024	0.0043	2.42E-08	0.5022	NA	170756	rs3998	329443	ieu-b-102	TRUE	reported	WYslv6
1	73616611	C	T	0.024	0.0043	2.28E-08	0.5023	NA	170756	rs4341323	329443	ieu-b-102	TRUE	reported	WYslv6
1	73617423	C	T	0.0241	0.0043	2.24E-08	0.5023	NA	170756	rs6176760	329443	ieu-b-102	TRUE	reported	WYslv6
1	73617681	A	T	0.0256	0.0043	3.45E-09	0.4982	NA	170756	rs4449980	329443	ieu-b-102	TRUE	reported	WYslv6
1	73617682	A	C	0.0256	0.0043	3.58E-09	0.4984	NA	170756	rs3866323	329443	ieu-b-102	TRUE	reported	WYslv6
1	73618080	C	T	0.024	0.0043	2.37E-08	0.5022	NA	170756	rs4353041	329443	ieu-b-102	TRUE	reported	WYslv6
1	73618176	G	A	-0.024	0.0043	2.63E-08	0.4852	NA	170756	rs1121014	329443	ieu-b-102	TRUE	reported	WYslv6
1	73618327	C	G	0.0242	0.0043	2.20E-08	0.5018	NA	170756	rs1272907	329443	ieu-b-102	TRUE	reported	WYslv6
1	73618348	A	G	0.0242	0.0043	2.27E-08	0.5018	NA	170756	rs4454487	329443	ieu-b-102	TRUE	reported	WYslv6
1	73618456	C	T	0.0241	0.0043	2.24E-08	0.5023	NA	170756	rs7417820	329443	ieu-b-102	TRUE	reported	WYslv6
1	73618622	G	C	0.0242	0.0043	2.28E-08	0.5018	NA	170756	rs4633240	329443	ieu-b-102	TRUE	reported	WYslv6
1	73618629	A	G	0.0242	0.0043	2.22E-08	0.5018	NA	170756	rs4454488	329443	ieu-b-102	TRUE	reported	WYslv6
1	73618752	A	G	0.024	0.0043	2.41E-08	0.5022	NA	170756	rs4369180	329443	ieu-b-102	TRUE	reported	WYslv6
1	73618845	G	T	0.024	0.0043	2.50E-08	0.5022	NA	170756	rs4633241	329443	ieu-b-102	TRUE	reported	WYslv6
1	73619229	T	A	-0.024	0.0043	2.43E-08	0.4852	NA	170756	rs1039993	329443	ieu-b-102	TRUE	reported	WYslv6
1	73619315	T	C	0.0239	0.0043	2.80E-08	0.502	NA	170756	rs1214498	329443	ieu-b-102	TRUE	reported	WYslv6
1	73619432	G	T	-0.0239	0.0043	2.95E-08	0.4857	NA	170756	rs1039982	329443	ieu-b-102	TRUE	reported	WYslv6
1	73619434	A	C	0.0241	0.0043	2.18E-08	0.502	NA	170756	rs1553153	329443	ieu-b-102	TRUE	reported	WYslv6
1	73620166	A	G	0.024	0.0043	2.38E-08	0.5022	NA	170756	rs4465150	329443	ieu-b-102	TRUE	reported	WYslv6
1	73620456	G	T	0.024	0.0043	2.30E-08	0.5022	NA	170756	rs3421997	329443	ieu-b-102	TRUE	reported	WYslv6
1	73620628	A	G	0.0241	0.0043	2.28E-08	0.5022	NA	170756	rs6176637	329443	ieu-b-102	TRUE	reported	WYslv6
1	73620687	G	C	0.0241	0.0043	2.28E-08	0.5022	NA	170756	rs6812208	329443	ieu-b-102	TRUE	reported	WYslv6
1	73620770	T	C	0.0245	0.0043	1.28E-08	0.5011	NA	170756	rs1137408	329443	ieu-b-102	TRUE	reported	WYslv6
1	73620791	A	G	0.0239	0.0043	2.72E-08	0.5013	NA	170756	rs1129594	329443	ieu-b-102	TRUE	reported	WYslv6
1	73620861	G	A	0.0241	0.0043	2.17E-08	0.5024	NA	170756	rs1131701	329443	ieu-b-102	TRUE	reported	WYslv6
1	73620907	G	A	-0.0278	0.0043	1.30E-10	0.4545	NA	170756	rs1224010	329443	ieu-b-102	TRUE	reported	WYslv6
1	73621082	C	T	0.0244	0.0043	1.81E-08	0.4972	NA	170756	rs1135500	329443	ieu-b-102	TRUE	reported	WYslv6
1	73621176	G	A	0.0243	0.0043	1.54E-08	0.5016	NA	170756	rs6176637	329443	ieu-b-102	TRUE	reported	WYslv6
1	73621202	C	T	0.0242	0.0043	1.90E-08	0.502	NA	170756	rs7617114	329443	ieu-b-102	TRUE	reported	WYslv6
1	73622007	A	G	-0.0272	0.0043	2.74E-10	0.4632	NA	170756	rs3740477	329443	ieu-b-102	TRUE	reported	WYslv6
1	73622248	A	G	0.024	0.0043	2.28E-08	0.5022	NA	170756	rs7528238	329443	ieu-b-102	TRUE	reported	WYslv6
1	73623390	A	T	-0.0238	0.0043	3.66E-08	0.493	NA	170756	rs1121014	329443	ieu-b-102	TRUE	reported	WYslv6
1	73625762	G	A	0.0239	0.0043	3.42E-08	0.4943	NA	170756	rs1121015	329443	ieu-b-102	TRUE	reported	WYslv6
1	73625793	G	A	0.0245	0.0043	1.62E-08	0.4946	NA	170756	rs1121015	329443	ieu-b-102	TRUE	reported	WYslv6
1	73625813	A	T	-0.0241	0.0043	2.77E-08	0.4936	NA	170756	rs3866323	329443	ieu-b-102	TRUE	reported	WYslv6
1	73626418	G	A	0.0235	0.0043	4.98E-08	0.4938	NA	170756	rs3544670	329443	ieu-b-102	TRUE	reported	WYslv6
1	73626527	T	C	-0.0273	0.0043	2.40E-10	0.4631	NA	170756	rs6672279	329443	ieu-b-102	TRUE	reported	WYslv6
1	73627224	C	G	0.0238	0.0043	3.62E-08	0.489	NA	170756	rs1121015	329443	ieu-b-102	TRUE	reported	WYslv6
1	73627225	C	A	0.0239	0.0043	3.42E-08	0.489	NA	170756	rs3541329	329443	ieu-b-102	TRUE	reported	WYslv6
1	73627804	A	T	0.0235	0.0043	4.66E-08	0.4939	NA	170756	rs4579710	329443	ieu-b-102	TRUE	reported	WYslv6
1	73628283	G	T	0.0235	0.0043	4.66E-08	0.4939	NA	170756	rs4294371	329443	ieu-b-102	TRUE	reported	WYslv6
1	73628556	C	T	0.0244	0.0043	1.73E-08	0.4902	NA	170756	rs6664120	329443	ieu-b-102	TRUE	reported	WYslv6
1	73628557	G	A	0.0244	0.0043	1.78E-08	0.4903	NA	170756	rs7158699	329443	ieu-b-102	TRUE	reported	WYslv6
1	73628722	C	T	0.0235	0.0043	4.64E-08	0.4939	NA	170756	rs6666765	329443	ieu-b-102	TRUE	reported	WYslv6
1	73629044	A	G	0.0235	0.0043	4.63E-08	0.4939	NA	170756	rs1121016	329443	ieu-b-102	TRUE	reported	WYslv6
1	73629113	C	T	0.0235	0.0043	4.64E-08	0.4939	NA	170756	rs1121016	329443	ieu-b-102	TRUE	reported	WYslv6
1	73629341	A	G	0.0236	0.0043	4.49E-08	0.4939	NA	170756	rs1121016	329443	ieu-b-102	TRUE	reported	WYslv6
1	73629507	G	T	0.0235	0.0043	4.64E-08	0.4939	NA	170756	rs1121016	329443	ieu-b-102	TRUE	reported	WYslv6
1	73629676	T	A	0.0236	0.0043	4.21E-08	0.4937	NA	170756	rs1274269	329443	ieu-b-102	TRUE	reported	WYslv6
1	73630073	C	A	0.0235	0.0043	4.63E-08	0.4939	NA	170756	rs1121016	329443	ieu-b-102	TRUE	reported	WYslv6
1	73630650	T	A	0.0236	0.0043	4.30E-08	0.4938	NA	170756	rs6686255	329443	ieu-b-102	TRUE	reported	WYslv6
1	73630701	C	T	0.0235	0.0043	4.82E-08	0.4938	NA	170756	rs6671028	329443	ieu-b-102	TRUE	reported	WYslv6
1	73630707	G	A	0.0235	0.0043	4.78E-08	0.4938	NA	170756	rs6694634	329443	ieu-b-102	TRUE	reported	WYslv6
1	73630771	G	A	0.0236	0.0043	4.49E-08	0.4939	NA	170756	rs6697098	329443	ieu-b-102	TRUE	reported	WYslv6
1	73631558	C	A	0.024	0.0043	2.70E-08	0.4942	NA	170756	rs6674333	329443	ieu-b-102	TRUE	reported	WYslv6
1	73631632	C	T	0.0236	0.0043	4.52E-08	0.4939	NA	170756	rs6674404	329443	ieu-b-102	TRUE	reported	WYslv6
1	73631812	G	C	0.0236	0.0043	4.34E-08	0.4939	NA	170756	rs6700625	329443	ieu-b-102	TRUE	reported	WYslv6
1	73631994	C	T	0.0236	0.0043	4.31E-08	0.4939	NA	170756	rs4537504	329443	ieu-b-102	TRUE	reported	WYslv6
1	73633408	G	A	0.0238	0.0043	3.39E-08	0.4938	NA	170756	rs1121016	329443	ieu-b-102	TRUE	reported	WYslv6
1	73634013	A	G	0.0244	0.0043	1.47E-08	0.5023	NA	170756	rs1212885	329443	ieu-b-102	TRUE	reported	WYslv6
1	73634128	C	T	0.0238	0.0043	3.39E-08	0.4938	NA	170756	rs6684331	329443	ieu-b-102	TRUE	reported	WYslv6

1	73634621	T	C	0.0246	0.0043	1.19E-08	0.5021	NA	170756	rs6699861	329443	ieu-b-102	TRUE	reported	WYslv6
1	73634781	C	T	0.0244	0.0043	1.43E-08	0.5023	NA	170756	rs1213649	329443	ieu-b-102	TRUE	reported	WYslv6
1	73635177	G	A	0.0244	0.0043	1.48E-08	0.5023	NA	170756	rs1212252	329443	ieu-b-102	TRUE	reported	WYslv6
1	73636118	A	T	0.0244	0.0043	1.48E-08	0.5023	NA	170756	rs4593762	329443	ieu-b-102	TRUE	reported	WYslv6
1	73636172	C	A	0.0244	0.0043	1.49E-08	0.5023	NA	170756	rs4492565	329443	ieu-b-102	TRUE	reported	WYslv6
1	73636502	G	A	0.0244	0.0043	1.44E-08	0.5023	NA	170756	rs4650193	329443	ieu-b-102	TRUE	reported	WYslv6
1	73636583	T	C	-0.0245	0.0043	1.51E-08	0.4843	NA	170756	rs1121016	329443	ieu-b-102	TRUE	reported	WYslv6
1	73636606	A	G	0.0244	0.0043	1.54E-08	0.5023	NA	170756	rs4650194	329443	ieu-b-102	TRUE	reported	WYslv6
1	73636689	C	T	-0.0243	0.0043	1.63E-08	0.4851	NA	170756	rs1121016	329443	ieu-b-102	TRUE	reported	WYslv6
1	73637003	G	C	0.0244	0.0043	1.45E-08	0.5023	NA	170756	rs1158989	329443	ieu-b-102	TRUE	reported	WYslv6
1	73637083	C	T	0.0244	0.0043	1.48E-08	0.5023	NA	170756	rs1158246	329443	ieu-b-102	TRUE	reported	WYslv6
1	73637170	T	C	0.0244	0.0043	1.48E-08	0.5023	NA	170756	rs1121016	329443	ieu-b-102	TRUE	reported	WYslv6
1	73637446	A	G	-0.0237	0.0043	3.88E-08	0.4935	NA	170756	rs1121017	329443	ieu-b-102	TRUE	reported	WYslv6
1	73637641	G	A	0.0238	0.0043	3.22E-08	0.4938	NA	170756	rs4650195	329443	ieu-b-102	TRUE	reported	WYslv6
1	73637694	C	G	-0.0237	0.0043	3.94E-08	0.4935	NA	170756	rs1089001	329443	ieu-b-102	TRUE	reported	WYslv6
1	73637800	C	A	0.0244	0.0043	1.42E-08	0.5023	NA	170756	rs4650196	329443	ieu-b-102	TRUE	reported	WYslv6
1	73638323	A	G	0.0238	0.0043	3.33E-08	0.4939	NA	170756	rs3513393	329443	ieu-b-102	TRUE	reported	WYslv6
1	73638834	A	C	0.0238	0.0043	3.37E-08	0.4939	NA	170756	rs6695455	329443	ieu-b-102	TRUE	reported	WYslv6
1	73638840	A	G	0.0238	0.0043	3.37E-08	0.4939	NA	170756	rs6695460	329443	ieu-b-102	TRUE	reported	WYslv6
1	73639010	G	A	0.0238	0.0043	3.15E-08	0.4936	NA	170756	rs6658012	329443	ieu-b-102	TRUE	reported	WYslv6
1	73639192	C	G	0.0238	0.0043	3.38E-08	0.4939	NA	170756	rs6683380	329443	ieu-b-102	TRUE	reported	WYslv6
1	73639316	T	C	0.0238	0.0043	3.47E-08	0.4939	NA	170756	rs6698500	329443	ieu-b-102	TRUE	reported	WYslv6
1	73639581	G	T	0.0238	0.0043	3.44E-08	0.4939	NA	170756	rs1089001	329443	ieu-b-102	TRUE	reported	WYslv6
1	73639751	C	T	0.0238	0.0043	3.39E-08	0.4939	NA	170756	rs1089001	329443	ieu-b-102	TRUE	reported	WYslv6
1	73639819	G	A	0.0238	0.0043	3.28E-08	0.4939	NA	170756	rs1089001	329443	ieu-b-102	TRUE	reported	WYslv6
1	73639926	C	T	0.0238	0.0043	3.34E-08	0.4939	NA	170756	rs1089001	329443	ieu-b-102	TRUE	reported	WYslv6
1	73640129	A	G	-0.0276	0.0043	1.75E-10	0.4635	NA	170756	rs1074981	329443	ieu-b-102	TRUE	reported	WYslv6
1	73640527	A	G	0.0243	0.0043	1.63E-08	0.5023	NA	170756	rs1212455	329443	ieu-b-102	TRUE	reported	WYslv6
1	73640680	A	G	0.0237	0.0043	3.76E-08	0.4939	NA	170756	rs3471923	329443	ieu-b-102	TRUE	reported	WYslv6
1	73640914	A	T	0.0237	0.0043	3.63E-08	0.4938	NA	170756	rs3486820	329443	ieu-b-102	TRUE	reported	WYslv6
1	73640940	A	T	0.0237	0.0043	3.67E-08	0.4938	NA	170756	rs3495609	329443	ieu-b-102	TRUE	reported	WYslv6
1	73641001	C	T	0.0237	0.0043	3.68E-08	0.4938	NA	170756	rs3605165	329443	ieu-b-102	TRUE	reported	WYslv6
1	73641024	T	A	0.0237	0.0043	3.68E-08	0.4938	NA	170756	rs3605372	329443	ieu-b-102	TRUE	reported	WYslv6
1	73641059	A	T	0.0238	0.0043	3.36E-08	0.4938	NA	170756	rs3528665	329443	ieu-b-102	TRUE	reported	WYslv6
1	73641086	G	A	-0.0237	0.0043	3.94E-08	0.4935	NA	170756	rs1205955	329443	ieu-b-102	TRUE	reported	WYslv6
1	73641529	C	A	-0.0237	0.0043	3.74E-08	0.4935	NA	170756	rs1120694	329443	ieu-b-102	TRUE	reported	WYslv6
1	73642486	A	G	0.0238	0.0043	3.14E-08	0.4938	NA	170756	rs1212668	329443	ieu-b-102	TRUE	reported	WYslv6
1	73642627	A	G	0.0237	0.0043	4.12E-08	0.4932	NA	170756	rs6657881	329443	ieu-b-102	TRUE	reported	WYslv6
1	73642630	A	C	0.0236	0.0043	4.40E-08	0.4932	NA	170756	rs6657885	329443	ieu-b-102	TRUE	reported	WYslv6
1	73642992	G	T	0.0238	0.0043	3.29E-08	0.4938	NA	170756	rs4650197	329443	ieu-b-102	TRUE	reported	WYslv6
1	73643834	A	C	-0.0237	0.0043	4.17E-08	0.4933	NA	170756	rs1078935	329443	ieu-b-102	TRUE	reported	WYslv6
1	73643869	A	G	0.0238	0.0043	3.80E-08	0.4933	NA	170756	rs6176559	329443	ieu-b-102	TRUE	reported	WYslv6
1	73643871	T	A	0.0237	0.0043	4.50E-08	0.4938	NA	170756	rs3866323	329443	ieu-b-102	TRUE	reported	WYslv6
1	73644010	T	G	0.0247	0.0043	1.02E-08	0.503	NA	170756	rs6743763	329443	ieu-b-102	TRUE	reported	WYslv6
1	73644670	C	T	0.0242	0.0043	2.20E-08	0.4937	NA	170756	rs4592219	329443	ieu-b-102	TRUE	reported	WYslv6
1	73644787	C	A	0.0241	0.0043	2.28E-08	0.4937	NA	170756	rs4503305	329443	ieu-b-102	TRUE	reported	WYslv6
1	73644897	T	C	-0.024	0.0043	2.83E-08	0.4936	NA	170756	rs1089001	329443	ieu-b-102	TRUE	reported	WYslv6
1	73645150	C	G	0.0241	0.0043	2.40E-08	0.4937	NA	170756	rs3557848	329443	ieu-b-102	TRUE	reported	WYslv6
1	73645238	C	T	0.0241	0.0043	2.49E-08	0.4938	NA	170756	rs3520928	329443	ieu-b-102	TRUE	reported	WYslv6
1	73645418	G	T	0.024	0.0043	2.65E-08	0.4938	NA	170756	rs3454806	329443	ieu-b-102	TRUE	reported	WYslv6
1	73645739	C	A	0.0241	0.0043	2.48E-08	0.4939	NA	170756	rs1213729	329443	ieu-b-102	TRUE	reported	WYslv6
1	73645820	G	A	0.0271	0.0044	5.88E-10	0.5065	NA	170756	rs3535032	329443	ieu-b-102	TRUE	reported	WYslv6
1	73645866	T	C	-0.0239	0.0043	3.00E-08	0.4935	NA	170756	rs6703953	329443	ieu-b-102	TRUE	reported	WYslv6
1	73646085	C	T	0.0241	0.0043	2.51E-08	0.4938	NA	170756	rs1121017	329443	ieu-b-102	TRUE	reported	WYslv6
1	73646317	T	C	0.024	0.0043	2.62E-08	0.4938	NA	170756	rs4601539	329443	ieu-b-102	TRUE	reported	WYslv6
1	73646705	C	T	-0.024	0.0043	2.90E-08	0.4933	NA	170756	rs1208761	329443	ieu-b-102	TRUE	reported	WYslv6
1	73646931	C	T	0.0245	0.0043	1.78E-08	0.4734	NA	170756	rs4641264	329443	ieu-b-102	TRUE	reported	WYslv6
1	73646944	G	A	0.0239	0.0043	3.10E-08	0.4935	NA	170756	rs5689008	329443	ieu-b-102	TRUE	reported	WYslv6
1	73646999	G	A	0.0239	0.0043	3.16E-08	0.4935	NA	170756	rs4578169	329443	ieu-b-102	TRUE	reported	WYslv6
1	73647048	T	C	0.0239	0.0043	3.21E-08	0.4935	NA	170756	rs7514409	329443	ieu-b-102	TRUE	reported	WYslv6
1	73647072	G	C	-0.0239	0.0043	3.33E-08	0.4936	NA	170756	rs1121017	329443	ieu-b-102	TRUE	reported	WYslv6
1	73647117	C	A	0.0239	0.0043	3.26E-08	0.4935	NA	170756	rs7545548	329443	ieu-b-102	TRUE	reported	WYslv6
1	73647221	G	A	-0.0238	0.0043	3.65E-08	0.4929	NA	170756	rs1074981	329443	ieu-b-102	TRUE	reported	WYslv6
1	73647387	G	A	-0.024	0.0043	2.64E-08	0.4928	NA	170756	rs1078935	329443	ieu-b-102	TRUE	reported	WYslv6
1	73647800	C	A	0.0242	0.0043	2.44E-08	0.4605	NA	170756	rs1213393	329443	ieu-b-102	TRUE	reported	WYslv6
1	73651204	C	A	0.0285	0.0043	3.33E-11	0.5003	NA	170756	rs4274014	329443	ieu-b-102	TRUE	reported	WYslv6
1	73651519	A	T	-0.0276	0.0043	1.36E-10	0.4927	NA	170756	rs1174308	329443	ieu-b-102	TRUE	reported	WYslv6
1	73652122	T	C	0.0285	0.0043	3.37E-11	0.5003	NA	170756	rs4650199	329443	ieu-b-102	TRUE	reported	WYslv6
1	73652197	G	A	0.0254	0.0043	4.06E-09	0.4701	NA	170756	rs4650200	329443	ieu-b-102	TRUE	reported	WYslv6
1	73652349	G	A	0.0253	0.0043	4.32E-09	0.4701	NA	170756	rs4649981	329443	ieu-b-102	TRUE	reported	WYslv6
1	73654543	G	A	0.0255	0.0043	3.34E-09	0.471	NA	170756	rs1121017	329443	ieu-b-102	TRUE	reported	WYslv6
1	73655313	C	A	0.0254	0.0043	3.91E-09	0.4708	NA	170756	rs1214215	329443	ieu-b-102	TRUE	reported	WYslv6
1	73655821	A	G	0.0285	0.0043	3.37E-11	0.5053	NA	170756	rs1121017	329443	ieu-b-102	TRUE	reported	WYslv6
1	73658085	G	C	0.0289	0.0043	1.84E-11	0.4896	NA	170756	rs1206903	329443	ieu-b-102	TRUE	reported	WYslv6
1	73658307	T	G	0.0289	0.0043	1.74E-11	0.4896	NA	170756	rs1078936	329443	ieu-b-102	TRUE	reported	WYslv6
1	73658397	G	A	0.0289	0.0043	1.68E-11	0.4895	NA	170756	rs1078936	329443	ieu-b-102	TRUE	reported	WYslv6
1	73658507	A	T	0.0289	0.0043	1.70E-11	0.4895	NA	170756	rs7514832	329443	ieu-b-102	TRUE	reported	WYslv6
1	73658562	T	C	0.0289	0.0043	1.69E-11	0.4895	NA	170756	rs1089001	329443	ieu-b-102	TRUE	reported	WYslv6
1	73661205	C	T	0.0274	0.0043	2.18E-10	0.4821	NA	170756	rs4272574	329443	ieu-b-102	TRUE	reported	WYslv6
1	73661616	C	T	0.0289	0.0043	1.83E-11	0.4894	NA	170756	rs1214298	329443	ieu-b-102	TRUE	reported	WYslv6
1	73661661	T	C	0.0288	0.0043	2.26E-11	0.4892	NA	170756	rs1213209	329443	ieu-b-102	TRUE	reported	WYslv6
1	73662169	A	G	0.0289	0.0043	1.68E-11	0.4895	NA	170756	rs1089001	329443	ieu-b-102	TRUE	reported	WYslv6
1	73663180	G	A	0.0289	0.0043	1.77E-11	0.4894	NA	170756	rs1121017	329443	ieu-b-102	TRUE	reported	WYslv6
1	73663455	G	A	0.0288	0.0043	1.97E-11	0.4895	NA	170756	rs1089001	329443	ieu-b-102	TRUE	reported	WYslv6
1	73664022	G	A	0.0292	0.0043	1.10E-11	0.4893	NA	170756	rs6424532	329443	ieu-b-102	TRUE	reported	WYslv6
1	73664064	G	C	0.0289	0.0043	1.81E-11	0.4895	NA	170756	rs6424533	329443	ieu-b-102	TRUE	reported	WYslv6
1	73664320	A	G	0.0282	0.0043	5.88E-11	0.4826	NA	170756	rs6424535	329443	ieu-b-102	TRUE	reported	WYslv6
1	73664395	C	G	0.0289	0.0043	1.70E-11	0.4896	NA	170756	rs6424536	329443	ieu-b-102	TRUE	reported	WYslv6
1	73664563	C	T	0.0289	0.0043	1.76E-11	0.4895	NA	170756	rs1451062	329443	ieu-b-102	TRUE	reported	WYslv6
1	73664609	T	C	0.0289	0.0043	1.91E-11	0.4896	NA	170756	rs1121018	329443	ieu-b-102	TRUE	reported	WYslv6
1	73665514	G	C	0.0288	0.0043	2.17E-11	0.4894	NA	170756	rs1121018	329443	ieu-b-102	TRUE	reported	WYslv6
1	73665844	A	G	0.0288	0.0043	2.03E-11	0.4896	NA	170756	rs1450103	329443	ieu-b-102	TRUE	reported	WYslv6
1	73666227	A	T	0.028	0.0043	7.81E-11	0.4823	NA	170756	rs9529154	329443	ieu-b-102	TRUE	reported	WYslv6
1	73666614	A	G	-0.0272	0.0043	2.47E-10	0.4936	NA	170756	rs1204444	329443	ieu-b-102	TRUE	reported	WYslv6
1	73666825	G	A	0.0279	0.0043	8.40E-11	0.4825	NA	170756	rs1121018	329443	ieu-b-102	TRUE	reported	WYslv6
1	73666929	A	G	-0.0272	0.0043	2.39E-10	0.4937	NA	170756	rs2858557	329443	ieu-b-102	TRUE	reported	WYslv6
1	73668519	A	G	0.0279	0.0043	8.97E-11	0.4826	NA	170756	rs1078936	329443	ieu-b-102	TRUE	reported	WYslv6
1	73668836	A	G	0.0277	0.0043	1.38E-10	0.481	NA	170756	rs8893878	329443	ieu-b-102	TRUE	reported	WYslv6
1	73668882	T	A	0.0275	0.0043	1.56E-10	0.4819	NA	170756	rs1089002	329443	ieu-b-102	TRUE	reported	WYslv6
1	73669845	G	A	-0.0272	0.0043	2.70E-10	0.4927	NA	170756	rs6692884	329443	ieu-b-102	TRUE	reported	WYslv6
1	73674126	A	G	-0.0271	0.0043	4.10E-10	0.4908	NA	170756	rs1337645	329443	ieu-b-102	TRUE	reported	WYslv6
1	73677003	G	A	0.0285	0.0043	3.38E-11	0.4894	NA	170756	rs1180672	329443	ieu-b-102	TRUE	reported	WYslv6
1	73678454	A	T	0.0284	0.0043	6.19E-11	0.4845	NA	170756	rs1209285	329443	ieu-b-102	TRUE	reported	WYslv6
1	73680774	G	A	-0.0273	0.0043	2.27E-10	0.4931	NA	170756	rs4288548	329443	ieu-b-102	TRUE	reported	WYslv6
1	73684273	G	A	-0.0274	0.0043	1.92E-10	0.4936	NA	170756	rs4475699	329443	ieu-b-102	TRUE	reported	WYslv6
1	73685994	A	T	0.028	0.0043	8.24E-11	0.4826	NA	170756	rs4261072	329443	ieu-b-102	TRUE	reported	WYslv6
1	73686005	G	A	-0.0275	0.0043	2.20E-10	0.4933	NA	170756	rs7518008	329443	ieu-b-102	TRUE	reported	WYslv6

1	73686480	C	T	-0.0273	0.0043	2.03E-10	0.4936	NA	170756	rs1121018	329443	ieu-b-102	TRUE	reported	WYslv6
1	73694600	T	C	-0.0273	0.0043	2.35E-10	0.4938	NA	170756	rs4129350	329443	ieu-b-102	TRUE	reported	WYslv6
1	73696114	G	A	0.0279	0.0043	9.42E-11	0.4823	NA	170756	rs7533366	329443	ieu-b-102	TRUE	reported	WYslv6
1	73700690	T	G	-0.0273	0.0043	2.10E-10	0.4937	NA	170756	rs8179409	329443	ieu-b-102	TRUE	reported	WYslv6
1	73701671	G	T	0.0279	0.0043	9.38E-11	0.4823	NA	170756	rs6697023	329443	ieu-b-102	TRUE	reported	WYslv6
1	73701723	C	T	0.0279	0.0043	9.26E-11	0.4823	NA	170756	rs6675954	329443	ieu-b-102	TRUE	reported	WYslv6
1	73706169	A	C	0.0278	0.0043	9.70E-11	0.4826	NA	170756	rs6684541	329443	ieu-b-102	TRUE	reported	WYslv6
1	73706449	A	G	-0.0273	0.0043	2.29E-10	0.4937	NA	170756	rs6684841	329443	ieu-b-102	TRUE	reported	WYslv6
1	73706712	T	A	0.0279	0.0043	9.21E-11	0.4824	NA	170756	rs1442083	329443	ieu-b-102	TRUE	reported	WYslv6
1	73708637	A	G	-0.0272	0.0043	2.73E-10	0.4935	NA	170756	rs1240497	329443	ieu-b-102	TRUE	reported	WYslv6
1	73709391	C	T	-0.0273	0.0043	2.34E-10	0.4937	NA	170756	rs6682579	329443	ieu-b-102	TRUE	reported	WYslv6
1	73711369	A	T	0.0279	0.0043	9.40E-11	0.4823	NA	170756	rs1121018	329443	ieu-b-102	TRUE	reported	WYslv6
1	73711493	A	G	-0.0273	0.0043	2.22E-10	0.4938	NA	170756	rs5757810	329443	ieu-b-102	TRUE	reported	WYslv6
1	73711811	C	T	0.0279	0.0043	8.46E-11	0.4823	NA	170756	rs1121018	329443	ieu-b-102	TRUE	reported	WYslv6
1	73715233	T	C	0.0281	0.0043	6.93E-11	0.4827	NA	170756	rs1121018	329443	ieu-b-102	TRUE	reported	WYslv6
1	73720577	T	C	0.028	0.0043	7.44E-11	0.4824	NA	170756	rs4650206	329443	ieu-b-102	TRUE	reported	WYslv6
1	73722460	C	G	0.0284	0.0043	4.47E-11	0.4819	NA	170756	rs4074990	329443	ieu-b-102	TRUE	reported	WYslv6
1	73725998	T	G	0.0292	0.0043	1.43E-11	0.4892	NA	170756	rs1213698	329443	ieu-b-102	TRUE	reported	WYslv6
1	73729188	A	G	0.0261	0.0043	1.38E-09	0.4905	NA	170756	rs1121018	329443	ieu-b-102	TRUE	reported	WYslv6
1	73731145	C	T	0.0263	0.0043	1.11E-09	0.4909	NA	170756	rs6672818	329443	ieu-b-102	TRUE	reported	WYslv6
1	73735403	G	C	-0.0269	0.0043	4.24E-10	0.5028	NA	170756	rs6176563	329443	ieu-b-102	TRUE	reported	WYslv6
1	73736562	G	A	0.028	0.0043	9.16E-11	0.4715	NA	170756	rs4571923	329443	ieu-b-102	TRUE	reported	WYslv6
1	73736889	C	T	0.0263	0.0043	1.05E-09	0.4925	NA	170756	rs1275903	329443	ieu-b-102	TRUE	reported	WYslv6
1	73738163	A	G	0.0263	0.0043	1.06E-09	0.4909	NA	170756	rs1089002	329443	ieu-b-102	TRUE	reported	WYslv6
1	73739641	G	C	0.0263	0.0043	1.03E-09	0.4909	NA	170756	rs1207704	329443	ieu-b-102	TRUE	reported	WYslv6
1	73742618	T	G	0.0263	0.0043	1.09E-09	0.4909	NA	170756	rs1121019	329443	ieu-b-102	TRUE	reported	WYslv6
1	73743873	A	G	-0.0269	0.0043	4.04E-10	0.503	NA	170756	rs1121019	329443	ieu-b-102	TRUE	reported	WYslv6
1	73744843	C	T	-0.0263	0.0043	1.03E-09	0.5089	NA	170756	rs6684341	329443	ieu-b-102	TRUE	reported	WYslv6
1	73747237	T	C	0.0264	0.0043	8.91E-10	0.491	NA	170756	rs7513593	329443	ieu-b-102	TRUE	reported	WYslv6
1	73749283	C	T	-0.027	0.0043	3.58E-10	0.5026	NA	170756	rs1121019	329443	ieu-b-102	TRUE	reported	WYslv6
1	73750422	T	C	-0.0269	0.0043	4.09E-10	0.5026	NA	170756	rs1204421	329443	ieu-b-102	TRUE	reported	WYslv6
1	73750567	C	T	-0.0263	0.0043	1.10E-09	0.5087	NA	170756	rs1121019	329443	ieu-b-102	TRUE	reported	WYslv6
1	73751701	C	A	0.0264	0.0043	9.52E-10	0.4911	NA	170756	rs1089002	329443	ieu-b-102	TRUE	reported	WYslv6
1	73752009	T	A	0.0264	0.0043	9.08E-10	0.491	NA	170756	rs1214251	329443	ieu-b-102	TRUE	reported	WYslv6
1	73755529	A	G	0.0264	0.0043	8.72E-10	0.491	NA	170756	rs7549372	329443	ieu-b-102	TRUE	reported	WYslv6
1	73755783	C	T	-0.0271	0.0043	3.18E-10	0.5026	NA	170756	rs1089003	329443	ieu-b-102	TRUE	reported	WYslv6
1	73757456	G	A	0.0267	0.0043	6.71E-10	0.4909	NA	170756	rs4463622	329443	ieu-b-102	TRUE	reported	WYslv6
1	73757712	C	T	0.0267	0.0043	6.67E-10	0.4913	NA	170756	rs6424544	329443	ieu-b-102	TRUE	reported	WYslv6
1	73758014	G	A	0.0268	0.0043	6.43E-10	0.4905	NA	170756	rs7523734	329443	ieu-b-102	TRUE	reported	WYslv6
1	73758117	G	A	0.028	0.0043	8.36E-11	0.4715	NA	170756	rs7523829	329443	ieu-b-102	TRUE	reported	WYslv6
1	73760104	T	C	-0.0271	0.0043	3.35E-10	0.5026	NA	170756	rs3564155	329443	ieu-b-102	TRUE	reported	WYslv6
1	73764712	G	A	-0.026	0.0043	1.50E-09	0.5075	NA	170756	rs1204937	329443	ieu-b-102	TRUE	reported	WYslv6
1	73766037	C	T	-0.0265	0.0043	7.76E-10	0.5099	NA	170756	rs7555507	329443	ieu-b-102	TRUE	reported	WYslv6
1	73766120	A	G	-0.0263	0.0043	9.51E-10	0.5086	NA	170756	rs7522217	329443	ieu-b-102	TRUE	reported	WYslv6
1	73766162	G	T	-0.0262	0.0043	1.15E-09	0.5082	NA	170756	rs7532462	329443	ieu-b-102	TRUE	reported	WYslv6
1	73768022	T	C	-0.0266	0.0043	6.85E-10	0.5023	NA	170756	rs1202334	329443	ieu-b-102	TRUE	reported	WYslv6
1	73768366	C	A	0.0252	0.0045	2.07E-08	0.3526	NA	170756	rs1212957	329443	ieu-b-102	TRUE	reported	WYslv6
1	73771345	A	G	-0.0269	0.0043	4.18E-10	0.5025	NA	170756	rs1923240	329443	ieu-b-102	TRUE	reported	WYslv6
1	73772693	T	C	-0.0267	0.0043	5.57E-10	0.5022	NA	170756	rs2180945	329443	ieu-b-102	TRUE	reported	WYslv6
1	73780291	A	T	-0.0269	0.0043	4.45E-10	0.5026	NA	170756	rs3585140	329443	ieu-b-102	TRUE	reported	WYslv6
1	73782148	T	G	-0.0267	0.0043	5.43E-10	0.5021	NA	170756	rs2224494	329443	ieu-b-102	TRUE	reported	WYslv6
1	73787158	G	A	-0.0268	0.0043	4.93E-10	0.5025	NA	170756	rs1089003	329443	ieu-b-102	TRUE	reported	WYslv6
1	73789324	T	C	-0.0251	0.0045	2.10E-08	0.6477	NA	170756	rs1089003	329443	ieu-b-102	TRUE	reported	WYslv6
1	73790407	G	A	-0.0269	0.0043	4.52E-10	0.5025	NA	170756	rs1273708	329443	ieu-b-102	TRUE	reported	WYslv6
1	73790683	G	T	-0.0268	0.0043	5.10E-10	0.5025	NA	170756	rs1256442	329443	ieu-b-102	TRUE	reported	WYslv6
1	73791614	T	G	-0.0269	0.0043	4.01E-10	0.5027	NA	170756	rs1923204	329443	ieu-b-102	TRUE	reported	WYslv6
1	73796949	T	G	-0.0268	0.0043	5.05E-10	0.5027	NA	170756	rs1203655	329443	ieu-b-102	TRUE	reported	WYslv6
1	73797306	C	T	-0.0268	0.0043	4.93E-10	0.5025	NA	170756	rs3845343	329443	ieu-b-102	TRUE	reported	WYslv6
1	73801632	A	G	-0.0248	0.0045	3.42E-08	0.6469	NA	170756	rs1369586	329443	ieu-b-102	TRUE	reported	WYslv6
1	73805104	A	T	-0.0267	0.0043	5.36E-10	0.5022	NA	170756	rs1120980	329443	ieu-b-102	TRUE	reported	WYslv6
1	73814159	A	G	-0.0268	0.0043	5.08E-10	0.5024	NA	170756	rs1078936	329443	ieu-b-102	TRUE	reported	WYslv6
1	73815631	T	G	-0.0268	0.0043	4.82E-10	0.5024	NA	170756	rs1049351	329443	ieu-b-102	TRUE	reported	WYslv6
1	73816564	C	T	-0.0253	0.0045	1.83E-08	0.6478	NA	170756	rs1160682	329443	ieu-b-102	TRUE	reported	WYslv6
1	73822289	C	T	-0.0266	0.0043	5.81E-10	0.5034	NA	170756	rs1203314	329443	ieu-b-102	TRUE	reported	WYslv6
1	73829223	C	T	-0.0268	0.0043	4.21E-10	0.5011	NA	170756	rs1923217	329443	ieu-b-102	TRUE	reported	WYslv6
1	73834650	T	G	-0.027	0.0043	3.29E-10	0.5029	NA	170756	rs911493	329443	ieu-b-102	TRUE	reported	WYslv6
1	73835741	G	A	-0.027	0.0043	3.59E-10	0.5026	NA	170756	rs2340402	329443	ieu-b-102	TRUE	reported	WYslv6
1	73836801	G	T	-0.027	0.0043	5.29E-10	0.4954	NA	170756	rs1202793	329443	ieu-b-102	TRUE	reported	WYslv6
1	73840341	C	T	-0.0269	0.0043	3.92E-10	0.5027	NA	170756	rs1240625	329443	ieu-b-102	TRUE	reported	WYslv6
1	73843468	T	C	-0.0268	0.0043	4.48E-10	0.503	NA	170756	rs1923225	329443	ieu-b-102	TRUE	reported	WYslv6
1	73846894	C	T	-0.0268	0.0043	4.31E-10	0.503	NA	170756	rs1923226	329443	ieu-b-102	TRUE	reported	WYslv6
1	73847138	G	A	-0.0268	0.0043	4.37E-10	0.5029	NA	170756	rs1121021	329443	ieu-b-102	TRUE	reported	WYslv6
1	73847802	C	T	-0.0268	0.0043	4.61E-10	0.503	NA	170756	rs7546305	329443	ieu-b-102	TRUE	reported	WYslv6
1	73851213	A	G	-0.0261	0.0043	1.28E-09	0.4934	NA	170756	rs1271843	329443	ieu-b-102	TRUE	reported	WYslv6
1	73858351	G	A	-0.026	0.0043	1.35E-09	0.4935	NA	170756	rs1049351	329443	ieu-b-102	TRUE	reported	WYslv6
1	73860631	G	A	-0.0258	0.0043	1.95E-09	0.4933	NA	170756	rs1049351	329443	ieu-b-102	TRUE	reported	WYslv6
1	73862004	G	T	-0.0258	0.0043	2.03E-09	0.4932	NA	170756	rs1338650	329443	ieu-b-102	TRUE	reported	WYslv6
1	73864251	C	T	-0.0258	0.0043	2.03E-09	0.4933	NA	170756	rs1121023	329443	ieu-b-102	TRUE	reported	WYslv6
1	73865790	G	A	-0.0256	0.0043	2.45E-09	0.493	NA	170756	rs1885250	329443	ieu-b-102	TRUE	reported	WYslv6
1	73866020	G	T	-0.0257	0.0043	2.11E-09	0.4933	NA	170756	rs1885251	329443	ieu-b-102	TRUE	reported	WYslv6
1	73868386	A	C	-0.0256	0.0043	2.54E-09	0.4928	NA	170756	rs1258376	329443	ieu-b-102	TRUE	reported	WYslv6
1	73874321	G	A	-0.0257	0.0043	2.40E-09	0.4933	NA	170756	rs1316718	329443	ieu-b-102	TRUE	reported	WYslv6
1	73878194	T	C	-0.0256	0.0043	2.26E-09	0.493	NA	170756	rs1121023	329443	ieu-b-102	TRUE	reported	WYslv6
1	73879821	C	T	-0.0256	0.0043	2.25E-09	0.493	NA	170756	rs1121023	329443	ieu-b-102	TRUE	reported	WYslv6
1	73880654	A	G	-0.0256	0.0043	2.72E-09	0.4931	NA	170756	rs1046586	329443	ieu-b-102	TRUE	reported	WYslv6
1	73880724	C	A	-0.0256	0.0043	2.34E-09	0.493	NA	170756	rs1046586	329443	ieu-b-102	TRUE	reported	WYslv6
1	73884929	G	C	-0.0256	0.0043	2.38E-09	0.493	NA	170756	rs1204431	329443	ieu-b-102	TRUE	reported	WYslv6
1	73886699	T	C	-0.0254	0.0043	3.37E-09	0.4926	NA	170756	rs7553998	329443	ieu-b-102	TRUE	reported	WYslv6
1	73887712	T	A	-0.0256	0.0043	2.63E-09	0.4931	NA	170756	rs4557913	329443	ieu-b-102	TRUE	reported	WYslv6
1	73887871	G	A	-0.0256	0.0043	2.26E-09	0.4928	NA	170756	rs1361244	329443	ieu-b-102	TRUE	reported	WYslv6
1	73888982	T	C	-0.0257	0.0043	2.43E-09	0.4929	NA	170756	rs1338656	329443	ieu-b-102	TRUE	reported	WYslv6
1	73889110	T	C	-0.0256	0.0043	2.64E-09	0.493	NA	170756	rs1424521	329443	ieu-b-102	TRUE	reported	WYslv6
1	73889436	C	T	-0.0256	0.0043	2.61E-09	0.493	NA	170756	rs4354497	329443	ieu-b-102	TRUE	reported	WYslv6
1	73889941	T	C	-0.0255	0.0043	2.80E-09	0.4929	NA	170756	rs1416274	329443	ieu-b-102	TRUE	reported	WYslv6
1	73890390	T	C	-0.0256	0.0043	2.68E-09	0.4929	NA	170756	rs7521446	329443	ieu-b-102	TRUE	reported	WYslv6
1	73890473	A	T	-0.0256	0.0043	2.67E-09	0.4929	NA	170756	rs7519030	329443	ieu-b-102	TRUE	reported	WYslv6
1	73890737	C	G	-0.0256	0.0043	2.70E-09	0.4929	NA	170756	rs6176551	329443	ieu-b-102	TRUE	reported	WYslv6
1	73890886	C	T	-0.0255	0.0043	2.91E-09	0.4929	NA	170756	rs6176551	329443	ieu-b-102	TRUE	reported	WYslv6
1	73890900	G	T	-0.0257	0.0043	2.43E-09	0.493	NA	170756	rs6176551	329443	ieu-b-102	TRUE	reported	WYslv6
1	73891887	G	A	-0.026	0.0043	1.44E-09	0.4937	NA	170756	rs7157547	329443	ieu-b-102	TRUE	reported	WYslv6
1	73892420	G	T	-0.026	0.0043	1.88E-09	0.4936	NA	170756	rs4636406	329443	ieu-b-102	TRUE	reported	WYslv6
1	73892579	A	G	-0.0256	0.0043	2.74E-09	0.4932	NA	170756	rs1538375	329443	ieu-b-102	TRUE	reported	WYslv6
1	73892613	A	C	-0.0255	0.0043	2.88E-09	0.4932	NA	170756	rs1310232	329443	ieu-b-102	TRUE	reported	WYslv6
1	73892824	C	A	-0.0256	0.0043	2.63E-09	0.4932	NA	170756	rs1416266	329443	ieu-b-102	TRUE	reported	WYslv6
1	73893148	T	C	-0.0256	0.0043	2.49E-09	0.4935	NA	170756	rs4406595	329443	ieu-b-102	TRUE	reported	WYslv6
1	73893579	G	A	-0.0255	0.0043	2.89E-09	0.493	NA	170756	rs1338647	329443	ieu-b-102	TRUE	reported	WYslv6
1	73893609	G	C	-0.0255	0.0043	2.88E-09	0.4928	NA	170756	rs7124276	329443	ieu-b-102	TRUE	reported	WYslv6

1	73893632	T	C	-0.0259	0.0043	2.08E-09	0.4935	NA	170756	rs1338649	329443	ieu-b-102	TRUE	reported	WYslv6
1	73894065	G	A	-0.0258	0.0043	2.56E-09	0.4945	NA	170756	rs1256911	329443	ieu-b-102	TRUE	reported	WYslv6
1	73894150	T	C	-0.0259	0.0043	2.19E-09	0.4936	NA	170756	rs1256253	329443	ieu-b-102	TRUE	reported	WYslv6
1	73894459	C	T	-0.026	0.0043	1.87E-09	0.4936	NA	170756	rs1256538	329443	ieu-b-102	TRUE	reported	WYslv6
1	73894768	G	A	-0.0253	0.0043	4.27E-09	0.4954	NA	170756	rs5606008	329443	ieu-b-102	TRUE	reported	WYslv6
1	73895051	A	G	-0.0251	0.0043	5.79E-09	0.4951	NA	170756	rs1202872	329443	ieu-b-102	TRUE	reported	WYslv6
1	73895169	G	A	-0.025	0.0043	7.15E-09	0.4946	NA	170756	rs7528128	329443	ieu-b-102	TRUE	reported	WYslv6
1	73895982	C	G	-0.025	0.0043	6.26E-09	0.495	NA	170756	rs1121023	329443	ieu-b-102	TRUE	reported	WYslv6
1	73896105	A	G	-0.0248	0.0043	9.12E-09	0.4936	NA	170756	rs1121024	329443	ieu-b-102	TRUE	reported	WYslv6
1	73897246	T	A	-0.0252	0.0043	4.30E-09	0.495	NA	170756	rs6672225	329443	ieu-b-102	TRUE	reported	WYslv6
1	73897381	A	G	-0.0252	0.0043	4.92E-09	0.495	NA	170756	rs6669843	329443	ieu-b-102	TRUE	reported	WYslv6
1	73897809	C	T	-0.0251	0.0043	4.96E-09	0.4945	NA	170756	rs1121024	329443	ieu-b-102	TRUE	reported	WYslv6
1	73898030	C	G	-0.025	0.0043	5.76E-09	0.4952	NA	170756	rs1121024	329443	ieu-b-102	TRUE	reported	WYslv6
1	73898258	G	A	-0.0251	0.0043	4.82E-09	0.495	NA	170756	rs1121024	329443	ieu-b-102	TRUE	reported	WYslv6
1	73899742	A	G	-0.0252	0.0043	4.86E-09	0.4948	NA	170756	rs1572668	329443	ieu-b-102	TRUE	reported	WYslv6
1	73900932	T	C	-0.025	0.0043	6.62E-09	0.4974	NA	170756	rs1121024	329443	ieu-b-102	TRUE	reported	WYslv6
1	73902953	A	G	-0.0253	0.0043	5.46E-09	0.4951	NA	170756	rs3510187	329443	ieu-b-102	TRUE	reported	WYslv6
1	73904211	G	A	-0.0251	0.0043	4.81E-09	0.4949	NA	170756	rs4369181	329443	ieu-b-102	TRUE	reported	WYslv6
1	73904937	A	G	-0.0254	0.0043	4.70E-09	0.4949	NA	170756	rs1407320	329443	ieu-b-102	TRUE	reported	WYslv6
1	73905414	A	C	-0.0251	0.0043	4.67E-09	0.495	NA	170756	rs1203350	329443	ieu-b-102	TRUE	reported	WYslv6
1	73905524	A	T	-0.0251	0.0043	5.07E-09	0.495	NA	170756	rs6679389	329443	ieu-b-102	TRUE	reported	WYslv6
1	73908975	T	C	-0.0256	0.0043	3.40E-09	0.4955	NA	170756	rs4287126	329443	ieu-b-102	TRUE	reported	WYslv6
1	73909964	A	G	-0.0256	0.0043	3.22E-09	0.4954	NA	170756	rs4113049	329443	ieu-b-102	TRUE	reported	WYslv6
1	73910055	T	C	-0.0256	0.0043	3.21E-09	0.4954	NA	170756	rs4113050	329443	ieu-b-102	TRUE	reported	WYslv6
1	73910157	G	T	-0.0254	0.0043	4.33E-09	0.4957	NA	170756	rs1274772	329443	ieu-b-102	TRUE	reported	WYslv6
1	73912799	C	G	-0.0252	0.0043	4.36E-09	0.4954	NA	170756	rs1121025	329443	ieu-b-102	TRUE	reported	WYslv6
1	73914157	A	G	-0.025	0.0043	6.57E-09	0.497	NA	170756	rs1203716	329443	ieu-b-102	TRUE	reported	WYslv6
1	73926994	A	T	-0.0249	0.0043	7.77E-09	0.5096	NA	170756	rs1203943	329443	ieu-b-102	TRUE	reported	WYslv6
1	73934633	A	C	-0.0251	0.0043	4.76E-09	0.5077	NA	170756	rs1121025	329443	ieu-b-102	TRUE	reported	WYslv6
1	73935251	G	A	-0.025	0.0043	5.19E-09	0.5075	NA	170756	rs1121025	329443	ieu-b-102	TRUE	reported	WYslv6
1	73935978	C	G	-0.025	0.0043	5.44E-09	0.5073	NA	170756	rs4361942	329443	ieu-b-102	TRUE	reported	WYslv6
1	73939898	C	T	-0.0249	0.0043	7.26E-09	0.5076	NA	170756	rs1275469	329443	ieu-b-102	TRUE	reported	WYslv6
1	73941881	C	A	-0.0249	0.0043	6.57E-09	0.5075	NA	170756	rs1121026	329443	ieu-b-102	TRUE	reported	WYslv6
1	73942894	C	T	-0.0248	0.0043	9.07E-09	0.5074	NA	170756	rs1121026	329443	ieu-b-102	TRUE	reported	WYslv6
1	73944904	T	A	-0.0248	0.0043	1.00E-08	0.5066	NA	170756	rs1223995	329443	ieu-b-102	TRUE	reported	WYslv6
1	73952123	C	A	-0.0241	0.0043	2.14E-08	0.5094	NA	170756	rs7513375	329443	ieu-b-102	TRUE	reported	WYslv6
1	73958086	G	T	-0.0246	0.0043	1.02E-08	0.5073	NA	170756	rs1021756	329443	ieu-b-102	TRUE	reported	WYslv6
1	73964312	G	T	-0.0246	0.0043	1.01E-08	0.5069	NA	170756	rs1121026	329443	ieu-b-102	TRUE	reported	WYslv6
1	73969936	G	A	-0.0249	0.0043	6.10E-09	0.5075	NA	170756	rs7416650	329443	ieu-b-102	TRUE	reported	WYslv6
1	73971450	T	C	-0.025	0.0043	5.29E-09	0.5074	NA	170756	rs2173735	329443	ieu-b-102	TRUE	reported	WYslv6
1	73974388	C	T	-0.025	0.0043	5.92E-09	0.507	NA	170756	rs1274240	329443	ieu-b-102	TRUE	reported	WYslv6
1	73977107	T	G	-0.025	0.0043	5.30E-09	0.5075	NA	170756	rs1202648	329443	ieu-b-102	TRUE	reported	WYslv6
1	73985364	A	G	-0.025	0.0043	6.27E-09	0.5075	NA	170756	rs6160598	329443	ieu-b-102	TRUE	reported	WYslv6
1	73986097	G	A	-0.0255	0.0043	3.88E-09	0.5081	NA	170756	rs2341392	329443	ieu-b-102	TRUE	reported	WYslv6
1	73987608	T	C	-0.025	0.0043	6.09E-09	0.5075	NA	170756	rs1338653	329443	ieu-b-102	TRUE	reported	WYslv6
1	73991792	T	G	-0.025	0.0043	5.99E-09	0.5076	NA	170756	rs1121027	329443	ieu-b-102	TRUE	reported	WYslv6
1	73999568	C	T	-0.0245	0.0043	1.44E-08	0.5589	NA	170756	rs1204833	329443	ieu-b-102	TRUE	reported	WYslv6
1	74005206	C	T	-0.0245	0.0043	1.45E-08	0.5593	NA	170756	rs7520239	329443	ieu-b-102	TRUE	reported	WYslv6
1	74007768	G	T	-0.0237	0.0043	4.19E-08	0.5519	NA	170756	rs7551153	329443	ieu-b-102	TRUE	reported	WYslv6
1	74011492	T	A	-0.0241	0.0043	2.52E-08	0.5526	NA	170756	rs1089004	329443	ieu-b-102	TRUE	reported	WYslv6
1	74011554	T	G	-0.0238	0.0043	3.76E-08	0.5524	NA	170756	rs1089004	329443	ieu-b-102	TRUE	reported	WYslv6
1	74016652	G	T	-0.0238	0.0043	3.55E-08	0.5522	NA	170756	rs1604348	329443	ieu-b-102	TRUE	reported	WYslv6
1	74018481	T	C	-0.0239	0.0043	3.33E-08	0.5516	NA	170756	rs1489208	329443	ieu-b-102	TRUE	reported	WYslv6
1	74028580	C	T	-0.0236	0.0043	4.99E-08	0.5514	NA	170756	rs1203328	329443	ieu-b-102	TRUE	reported	WYslv6
1	74030787	G	T	-0.0236	0.0043	4.95E-08	0.5514	NA	170756	rs1360243	329443	ieu-b-102	TRUE	reported	WYslv6
1	74032534	T	G	-0.0236	0.0043	4.98E-08	0.5514	NA	170756	rs1202691	329443	ieu-b-102	TRUE	reported	WYslv6
1	74033340	A	C	-0.0237	0.0043	4.40E-08	0.5516	NA	170756	rs7542499	329443	ieu-b-102	TRUE	reported	WYslv6
1	74035041	T	C	-0.0245	0.0044	1.94E-08	0.5654	NA	170756	rs978433	329443	ieu-b-102	TRUE	reported	WYslv6
1	74036009	A	G	-0.0241	0.0043	2.96E-08	0.5649	NA	170756	rs1854643	329443	ieu-b-102	TRUE	reported	WYslv6
1	74038928	C	A	-0.0242	0.0044	2.63E-08	0.5669	NA	170756	rs1854644	329443	ieu-b-102	TRUE	reported	WYslv6
1	74039267	T	G	-0.0246	0.0044	1.65E-08	0.5657	NA	170756	rs6694107	329443	ieu-b-102	TRUE	reported	WYslv6
1	74041576	T	C	-0.0245	0.0043	1.50E-08	0.5656	NA	170756	rs1202560	329443	ieu-b-102	TRUE	reported	WYslv6
1	74041715	A	G	-0.0246	0.0043	1.43E-08	0.5657	NA	170756	rs1312232	329443	ieu-b-102	TRUE	reported	WYslv6
1	74042455	C	T	-0.0247	0.0043	1.24E-08	0.5654	NA	170756	rs7542794	329443	ieu-b-102	TRUE	reported	WYslv6
1	74061909	T	A	-0.0246	0.0043	1.39E-08	0.5656	NA	170756	rs1411282	329443	ieu-b-102	TRUE	reported	WYslv6
1	74067414	A	G	-0.0245	0.0043	1.50E-08	0.5655	NA	170756	rs1121030	329443	ieu-b-102	TRUE	reported	WYslv6
1	74067804	G	C	-0.0247	0.0043	1.24E-08	0.5656	NA	170756	rs1089005	329443	ieu-b-102	TRUE	reported	WYslv6
1	74077588	T	C	-0.0255	0.0043	3.81E-09	0.5636	NA	170756	rs7540134	329443	ieu-b-102	TRUE	reported	WYslv6
1	74078828	C	T	-0.0243	0.0044	2.70E-08	0.5907	NA	170756	rs2134094	329443	ieu-b-102	TRUE	reported	WYslv6
1	74081123	C	T	-0.0242	0.0044	3.02E-08	0.5908	NA	170756	rs1604352	329443	ieu-b-102	TRUE	reported	WYslv6
1	74081154	T	C	-0.0243	0.0044	2.85E-08	0.5908	NA	170756	rs1576437	329443	ieu-b-102	TRUE	reported	WYslv6
1	74081912	T	C	-0.0244	0.0044	2.49E-08	0.5912	NA	170756	rs6424554	329443	ieu-b-102	TRUE	reported	WYslv6
1	74083804	G	T	-0.0243	0.0044	2.63E-08	0.5908	NA	170756	rs1576436	329443	ieu-b-102	TRUE	reported	WYslv6
1	74084595	A	G	-0.0243	0.0044	2.83E-08	0.5908	NA	170756	rs1015719	329443	ieu-b-102	TRUE	reported	WYslv6
1	74091104	T	C	-0.0241	0.0044	3.80E-08	0.5914	NA	170756	rs1952645	329443	ieu-b-102	TRUE	reported	WYslv6
1	74093259	C	T	-0.0246	0.0044	1.93E-08	0.5916	NA	170756	rs1576428	329443	ieu-b-102	TRUE	reported	WYslv6
1	74093417	G	A	-0.0246	0.0044	1.88E-08	0.5916	NA	170756	rs1576427	329443	ieu-b-102	TRUE	reported	WYslv6
1	74094854	T	C	-0.0244	0.0044	2.50E-08	0.5918	NA	170756	rs1089006	329443	ieu-b-102	TRUE	reported	WYslv6
1	74096615	G	A	-0.0244	0.0044	2.52E-08	0.5918	NA	170756	rs1952643	329443	ieu-b-102	TRUE	reported	WYslv6
1	74097006	T	C	-0.0246	0.0044	1.87E-08	0.5916	NA	170756	rs7524524	329443	ieu-b-102	TRUE	reported	WYslv6
1	74097497	G	A	-0.0244	0.0044	2.52E-08	0.5917	NA	170756	rs1204606	329443	ieu-b-102	TRUE	reported	WYslv6
1	74098633	C	T	-0.0244	0.0044	2.62E-08	0.5918	NA	170756	rs1576426	329443	ieu-b-102	TRUE	reported	WYslv6
1	74098899	C	T	-0.0243	0.0044	2.75E-08	0.5918	NA	170756	rs1329981	329443	ieu-b-102	TRUE	reported	WYslv6
1	1.76E+08	C	T	-0.0262	0.0045	4.53E-09	0.378	NA	170756	rs1091311	329443	ieu-b-102	TRUE	reported	WYslv6
1	1.76E+08	T	C	-0.026	0.0045	5.61E-09	0.3836	NA	170756	rs1203063	329443	ieu-b-102	TRUE	reported	WYslv6
1	1.76E+08	C	A	-0.0258	0.0045	7.84E-09	0.3774	NA	170756	rs1202397	329443	ieu-b-102	TRUE	reported	WYslv6
1	1.76E+08	G	A	-0.026	0.0045	5.26E-09	0.3784	NA	170756	rs3584637	329443	ieu-b-102	TRUE	reported	WYslv6
1	1.76E+08	T	C	-0.0259	0.0044	5.29E-09	0.3806	NA	170756	rs1204024	329443	ieu-b-102	TRUE	reported	WYslv6
1	1.76E+08	A	C	-0.0257	0.0044	7.55E-09	0.379	NA	170756	rs1091312	329443	ieu-b-102	TRUE	reported	WYslv6
1	1.76E+08	T	A	-0.0257	0.0044	7.66E-09	0.3782	NA	170756	rs1049449	329443	ieu-b-102	TRUE	reported	WYslv6
1	1.76E+08	G	A	-0.0255	0.0044	8.31E-09	0.3784	NA	170756	rs6673760	329443	ieu-b-102	TRUE	reported	WYslv6
1	1.76E+08	G	A	-0.0256	0.0044	7.62E-09	0.3782	NA	170756	rs3421516	329443	ieu-b-102	TRUE	reported	WYslv6
1	1.76E+08	A	T	-0.0256	0.0044	7.49E-09	0.3785	NA	170756	rs1204729	329443	ieu-b-102	TRUE	reported	WYslv6
1	1.76E+08	G	A	-0.0254	0.0044	1.12E-08	0.3778	NA	170756	rs3748618	329443	ieu-b-102	TRUE	reported	WYslv6
1	1.76E+08	A	G	-0.0253	0.0044	1.09E-08	0.3783	NA	170756	rs1203974	329443	ieu-b-102	TRUE	reported	WYslv6
1	1.76E+08	G	A	-0.0253	0.0044	1.09E-08	0.3783	NA	170756	rs1204291	329443	ieu-b-102	TRUE	reported	WYslv6
1	1.76E+08	A	T	-0.0255	0.0044	9.69E-09	0.3778	NA	170756	rs1240910	329443	ieu-b-102	TRUE	reported	WYslv6
1	1.76E+08	T	C	-0.0256	0.0044	8.80E-09	0.3777	NA	170756	rs1275550	329443	ieu-b-102	TRUE	reported	WYslv6
1	1.76E+08	G	A	-0.0255	0.0044	9.03E-09	0.3775	NA	170756	rs4526571	329443	ieu-b-102	TRUE	reported	WYslv6
1	1.76E+08	G	C	-0.0255	0.0044	7.63E-09	0.3954	NA	170756	rs1202429	329443	ieu-b-102	TRUE	reported	WYslv6
1	1.76E+08	G	A	-0.0255	0.0044	6.91E-09	0.395	NA	170756	rs1075312	329443	ieu-b-102	TRUE	reported	WYslv6
1	1.76E+08	T	A	-0.0256	0.0044	6.44E-09	0.3953	NA	170756	rs1091316	329443	ieu-b-102	TRUE	reported	WYslv6
1	1.76E+08	A	T	-0.0257	0.0044	5.65E-09	0.3954	NA	170756	rs1203173	329443	ieu-b-102	TRUE	reported	WYslv6
1	1.97E+08	C	G	-0.0285	0.0052	4.29E-08	0.2209	NA	170756	rs2759663	329443	ieu-b-102	TRUE	reported	WYslv6
1	1.98E+08	T	A	-0.0298	0.0053	1.70E-08	0.2143	NA	170756	rs1211851	329443	ieu-b-102	TRUE	reported	WYslv6
1	1.98E+08	A	G	-0.0279	0.0051	3.75E-08	0.2364	NA	170756	rs1158331	329443	ieu-b-102	TRUE	reported	WYslv6
1	1.98E+08	C	T	-0.0279	0.0051	3.65E-08	0.2362	NA	170756	rs6702421	329443	ieu-b-102	TRUE	reported	WYslv6

1	1.98E+08	C	T	-0.0279	0.0051	3.58E-08	0.2362	NA	170756	rs3593983	329443	ieu-b-102	TRUE	reported	WYslv6
1	1.98E+08	G	A	-0.0282	0.0051	2.72E-08	0.2361	NA	170756	rs1214102	329443	ieu-b-102	TRUE	reported	WYslv6
1	1.98E+08	A	T	0.0287	0.0052	3.43E-08	0.7472	NA	170756	rs4512681	329443	ieu-b-102	TRUE	reported	WYslv6
1	1.98E+08	T	C	0.0288	0.0052	3.67E-08	0.7816	NA	170756	rs1073769	329443	ieu-b-102	TRUE	reported	WYslv6
1	1.98E+08	C	T	-0.0292	0.0053	2.94E-08	0.2151	NA	170756	rs6682968	329443	ieu-b-102	TRUE	reported	WYslv6
1	1.98E+08	C	A	-0.0294	0.0052	1.79E-08	0.2176	NA	170756	rs1684184	329443	ieu-b-102	TRUE	reported	WYslv6
1	1.98E+08	G	A	-0.0295	0.0052	1.63E-08	0.2179	NA	170756	rs2488389	329443	ieu-b-102	TRUE	reported	WYslv6
1	1.98E+08	G	A	-0.0295	0.0052	1.54E-08	0.218	NA	170756	rs1211704	329443	ieu-b-102	TRUE	reported	WYslv6
1	1.98E+08	G	A	-0.0294	0.0052	1.70E-08	0.2176	NA	170756	rs1213158	329443	ieu-b-102	TRUE	reported	WYslv6
1	1.98E+08	C	T	-0.0294	0.0052	1.67E-08	0.218	NA	170756	rs2477077	329443	ieu-b-102	TRUE	reported	WYslv6
1	1.98E+08	G	C	-0.0297	0.0053	1.86E-08	0.2131	NA	170756	rs2488397	329443	ieu-b-102	TRUE	reported	WYslv6
1	1.98E+08	G	C	-0.0297	0.0053	1.79E-08	0.2132	NA	170756	rs2488398	329443	ieu-b-102	TRUE	reported	WYslv6
1	1.98E+08	C	T	-0.0298	0.0053	1.65E-08	0.2126	NA	170756	rs1684190	329443	ieu-b-102	TRUE	reported	WYslv6
1	1.98E+08	C	T	-0.0303	0.0054	1.62E-08	0.2072	NA	170756	rs1558456	329443	ieu-b-102	TRUE	reported	WYslv6
1	1.98E+08	G	C	-0.0297	0.0053	1.85E-08	0.2127	NA	170756	rs1684191	329443	ieu-b-102	TRUE	reported	WYslv6
1	1.98E+08	C	T	-0.03	0.0053	1.50E-08	0.2101	NA	170756	rs1764152	329443	ieu-b-102	TRUE	reported	WYslv6
1	1.98E+08	G	A	-0.0296	0.0053	2.16E-08	0.2121	NA	170756	rs1539414	329443	ieu-b-102	TRUE	reported	WYslv6
1	1.98E+08	T	A	-0.0292	0.0053	3.59E-08	0.2089	NA	170756	rs1212316	329443	ieu-b-102	TRUE	reported	WYslv6
1	1.98E+08	C	T	-0.0294	0.0053	3.03E-08	0.2079	NA	170756	rs1213427	329443	ieu-b-102	TRUE	reported	WYslv6
2	2.08E+08	C	A	0.0262	0.0046	1.57E-08	0.3133	NA	170756	rs5577285	329443	ieu-b-102	TRUE	reported	WYslv6
2	2.08E+08	T	C	0.0254	0.0046	4.46E-08	0.3119	NA	170756	rs7592120	329443	ieu-b-102	TRUE	reported	WYslv6
2	2.08E+08	G	A	0.0262	0.0047	1.85E-08	0.312	NA	170756	rs6218862	329443	ieu-b-102	TRUE	reported	WYslv6
2	2.08E+08	G	A	-0.0237	0.0043	4.17E-08	0.4821	NA	170756	rs6732724	329443	ieu-b-102	TRUE	reported	WYslv6
2	2.08E+08	G	A	0.0262	0.0046	1.53E-08	0.3134	NA	170756	rs7586277	329443	ieu-b-102	TRUE	reported	WYslv6
2	2.08E+08	G	A	0.0261	0.0046	1.65E-08	0.3139	NA	170756	rs6218863	329443	ieu-b-102	TRUE	reported	WYslv6
2	2.08E+08	G	A	0.0263	0.0046	1.35E-08	0.3141	NA	170756	rs2111592	329443	ieu-b-102	TRUE	reported	WYslv6
2	2.08E+08	C	T	0.0258	0.0046	2.31E-08	0.3139	NA	170756	rs1263686	329443	ieu-b-102	TRUE	reported	WYslv6
2	2.08E+08	T	C	0.026	0.0046	1.87E-08	0.3141	NA	170756	rs1263674	329443	ieu-b-102	TRUE	reported	WYslv6
2	2.08E+08	T	A	0.026	0.0046	1.83E-08	0.3135	NA	170756	rs7296822	329443	ieu-b-102	TRUE	reported	WYslv6
2	2.08E+08	T	C	0.026	0.0046	1.99E-08	0.3139	NA	170756	rs1268864	329443	ieu-b-102	TRUE	reported	WYslv6
2	2.08E+08	A	G	0.0257	0.0046	2.78E-08	0.3134	NA	170756	rs1721951	329443	ieu-b-102	TRUE	reported	WYslv6
2	2.08E+08	T	C	-0.0246	0.0045	3.23E-08	0.6281	NA	170756	rs1920049	329443	ieu-b-102	TRUE	reported	WYslv6
2	2.08E+08	T	C	-0.0247	0.0045	2.78E-08	0.6286	NA	170756	rs1022171	329443	ieu-b-102	TRUE	reported	WYslv6
2	2.08E+08	G	A	0.0256	0.0046	3.11E-08	0.3131	NA	170756	rs723305	329443	ieu-b-102	TRUE	reported	WYslv6
2	2.08E+08	T	C	0.026	0.0046	2.04E-08	0.3131	NA	170756	rs5640465	329443	ieu-b-102	TRUE	reported	WYslv6
2	2.08E+08	A	G	-0.0257	0.0046	2.74E-08	0.687	NA	170756	rs1021104	329443	ieu-b-102	TRUE	reported	WYslv6
2	2.08E+08	G	A	-0.0258	0.0046	2.64E-08	0.687	NA	170756	rs1018782	329443	ieu-b-102	TRUE	reported	WYslv6
2	2.08E+08	G	A	-0.0256	0.0046	3.29E-08	0.6866	NA	170756	rs1261789	329443	ieu-b-102	TRUE	reported	WYslv6
2	2.08E+08	T	A	0.0258	0.0046	2.62E-08	0.3134	NA	170756	rs7557813	329443	ieu-b-102	TRUE	reported	WYslv6
2	2.08E+08	T	G	0.0258	0.0046	2.46E-08	0.3134	NA	170756	rs1990777	329443	ieu-b-102	TRUE	reported	WYslv6
2	2.08E+08	T	C	-0.0246	0.0045	3.31E-08	0.6287	NA	170756	rs9865142	329443	ieu-b-102	TRUE	reported	WYslv6
2	2.08E+08	T	C	0.0256	0.0046	3.30E-08	0.3141	NA	170756	rs2216375	329443	ieu-b-102	TRUE	reported	WYslv6
2	2.08E+08	G	A	-0.0257	0.0046	2.97E-08	0.6871	NA	170756	rs759663	329443	ieu-b-102	TRUE	reported	WYslv6
2	2.13E+08	C	A	0.0255	0.0046	3.85E-08	0.3153	NA	170756	rs5589771	329443	ieu-b-102	TRUE	reported	WYslv6
2	2.13E+08	T	C	0.0254	0.0047	4.97E-08	0.3067	NA	170756	rs6218299	329443	ieu-b-102	TRUE	reported	WYslv6
2	2.13E+08	C	T	0.0257	0.0047	3.34E-08	0.3076	NA	170756	rs3544437	329443	ieu-b-102	TRUE	reported	WYslv6
2	2.13E+08	G	A	0.0262	0.0047	2.78E-08	0.2956	NA	170756	rs6218302	329443	ieu-b-102	TRUE	reported	WYslv6
2	2.13E+08	G	A	0.0261	0.0047	3.01E-08	0.2957	NA	170756	rs7601502	329443	ieu-b-102	TRUE	reported	WYslv6
2	2.13E+08	G	A	0.0265	0.0047	1.71E-08	0.2975	NA	170756	rs7294850	329443	ieu-b-102	TRUE	reported	WYslv6
2	2.13E+08	C	T	0.0263	0.0047	2.35E-08	0.2969	NA	170756	rs6218302	329443	ieu-b-102	TRUE	reported	WYslv6
2	2.13E+08	A	T	0.0265	0.0047	1.85E-08	0.2957	NA	170756	rs6107960	329443	ieu-b-102	TRUE	reported	WYslv6
2	2.13E+08	C	T	0.0257	0.0047	3.92E-08	0.3029	NA	170756	rs1486423	329443	ieu-b-102	TRUE	reported	WYslv6
2	2.13E+08	A	C	0.0262	0.0047	3.05E-08	0.2963	NA	170756	rs6218303	329443	ieu-b-102	TRUE	reported	WYslv6
3	49125063	C	T	0.0578	0.0106	4.83E-08	0.0439	NA	170756	rs1489239	329443	ieu-b-102	TRUE	reported	WYslv6
3	49210732	A	C	-0.029	0.0051	1.68E-08	0.7739	NA	170756	rs7617480	329443	ieu-b-102	TRUE	reported	WYslv6
3	49214066	G	A	-0.0291	0.0052	1.72E-08	0.7743	NA	170756	rs1308403	329443	ieu-b-102	TRUE	reported	WYslv6
3	49214303	G	T	-0.0292	0.0052	1.59E-08	0.7739	NA	170756	rs9831648	329443	ieu-b-102	TRUE	reported	WYslv6
3	49619493	C	T	-0.032	0.0059	4.81E-08	0.8395	NA	170756	rs3489079	329443	ieu-b-102	TRUE	reported	WYslv6
3	49621994	C	T	-0.032	0.0059	4.71E-08	0.8397	NA	170756	rs7629322	329443	ieu-b-102	TRUE	reported	WYslv6
3	49624065	T	C	-0.032	0.0059	4.98E-08	0.8398	NA	170756	rs9869120	329443	ieu-b-102	TRUE	reported	WYslv6
3	49639803	G	T	-0.0321	0.0059	4.43E-08	0.8397	NA	170756	rs1491983	329443	ieu-b-102	TRUE	reported	WYslv6
3	49642027	C	T	-0.0321	0.0058	3.98E-08	0.8395	NA	170756	rs4241405	329443	ieu-b-102	TRUE	reported	WYslv6
3	49645209	C	T	-0.0319	0.0058	4.95E-08	0.8397	NA	170756	rs1113021	329443	ieu-b-102	TRUE	reported	WYslv6
3	49645458	A	G	-0.032	0.0058	4.36E-08	0.8399	NA	170756	rs1263731	329443	ieu-b-102	TRUE	reported	WYslv6
3	49646981	T	C	-0.0321	0.0058	4.09E-08	0.8399	NA	170756	rs2029591	329443	ieu-b-102	TRUE	reported	WYslv6
3	49647404	T	A	-0.0319	0.0058	4.79E-08	0.8397	NA	170756	rs1463728	329443	ieu-b-102	TRUE	reported	WYslv6
3	49798864	G	A	0.0311	0.0057	3.75E-08	0.1747	NA	170756	rs7621797	329443	ieu-b-102	TRUE	reported	WYslv6
3	49825369	G	A	0.0309	0.0056	4.25E-08	0.1754	NA	170756	rs9851930	329443	ieu-b-102	TRUE	reported	WYslv6
3	49829653	A	G	0.0307	0.0056	4.97E-08	0.1759	NA	170756	rs7637711	329443	ieu-b-102	TRUE	reported	WYslv6
3	61143922	C	T	0.0247	0.0045	3.66E-08	0.3584	NA	170756	rs1113082	329443	ieu-b-102	TRUE	reported	WYslv6
3	61144081	C	A	0.0247	0.0045	3.53E-08	0.359	NA	170756	rs9816087	329443	ieu-b-102	TRUE	reported	WYslv6
3	61185652	G	C	0.024	0.0044	4.47E-08	0.4057	NA	170756	rs1113082	329443	ieu-b-102	TRUE	reported	WYslv6
3	61186550	T	G	0.0239	0.0044	4.99E-08	0.406	NA	170756	rs9846073	329443	ieu-b-102	TRUE	reported	WYslv6
3	61188985	T	G	0.024	0.0044	4.43E-08	0.4053	NA	170756	rs1980311	329443	ieu-b-102	TRUE	reported	WYslv6
3	61189043	T	G	0.0242	0.0044	4.21E-08	0.4049	NA	170756	rs1980310	329443	ieu-b-102	TRUE	reported	WYslv6
3	61189121	T	C	0.0239	0.0044	4.66E-08	0.406	NA	170756	rs1980309	329443	ieu-b-102	TRUE	reported	WYslv6
3	61189235	A	G	0.024	0.0044	4.37E-08	0.406	NA	170756	rs1980308	329443	ieu-b-102	TRUE	reported	WYslv6
3	61189741	A	G	0.024	0.0044	4.34E-08	0.406	NA	170756	rs1554599	329443	ieu-b-102	TRUE	reported	WYslv6
3	61192911	G	A	0.0247	0.0045	2.96E-08	0.3957	NA	170756	rs1419548	329443	ieu-b-102	TRUE	reported	WYslv6
3	61193647	T	C	0.0243	0.0044	3.77E-08	0.4018	NA	170756	rs7432943	329443	ieu-b-102	TRUE	reported	WYslv6
3	61195001	G	C	0.0239	0.0044	4.87E-08	0.4051	NA	170756	rs4688354	329443	ieu-b-102	TRUE	reported	WYslv6
3	61195302	T	A	0.0239	0.0044	5.00E-08	0.406	NA	170756	rs4605537	329443	ieu-b-102	TRUE	reported	WYslv6
3	61195894	C	G	0.0242	0.0044	3.84E-08	0.4048	NA	170756	rs9850114	329443	ieu-b-102	TRUE	reported	WYslv6
3	61196535	G	A	0.0239	0.0044	4.94E-08	0.4048	NA	170756	rs1249255	329443	ieu-b-102	TRUE	reported	WYslv6
3	61225849	T	C	0.024	0.0044	4.15E-08	0.4128	NA	170756	rs6809031	329443	ieu-b-102	TRUE	reported	WYslv6
3	61240533	A	G	0.0247	0.0044	1.41E-08	0.4174	NA	170756	rs9831249	329443	ieu-b-102	TRUE	reported	WYslv6
3	61241048	T	C	0.0246	0.0044	1.50E-08	0.4176	NA	170756	rs6445200	329443	ieu-b-102	TRUE	reported	WYslv6
3	61243611	T	C	0.0238	0.0044	4.61E-08	0.4155	NA	170756	rs6785875	329443	ieu-b-102	TRUE	reported	WYslv6
3	61254420	G	C	0.0246	0.0044	1.71E-08	0.4165	NA	170756	rs815717	329443	ieu-b-102	TRUE	reported	WYslv6
3	61255413	G	A	0.0248	0.0044	1.41E-08	0.4117	NA	170756	rs843812	329443	ieu-b-102	TRUE	reported	WYslv6
3	61262007	T	C	0.0239	0.0044	4.54E-08	0.416	NA	170756	rs815709	329443	ieu-b-102	TRUE	reported	WYslv6
3	61262149	A	G	0.024	0.0044	3.65E-08	0.4153	NA	170756	rs815710	329443	ieu-b-102	TRUE	reported	WYslv6
3	61262414	A	G	0.0239	0.0044	4.54E-08	0.4151	NA	170756	rs863635	329443	ieu-b-102	TRUE	reported	WYslv6
3	61264084	C	G	0.024	0.0044	3.60E-08	0.4267	NA	170756	rs815715	329443	ieu-b-102	TRUE	reported	WYslv6
3	1.16E+08	T	A	0.0412	0.0074	2.41E-08	0.0931	NA	170756	rs7695401	329443	ieu-b-102	TRUE	reported	WYslv6
3	1.18E+08	C	T	0.0295	0.0048	7.07E-10	0.285	NA	170756	rs6783233	329443	ieu-b-102	TRUE	reported	WYslv6
3	1.18E+08	T	C	0.0297	0.0048	6.03E-10	0.284	NA	170756	rs6651164	329443	ieu-b-102	TRUE	reported	WYslv6
3	1.18E+08	T	G	0.0282	0.0048	4.39E-09	0.2783	NA	170756	rs1171112	329443	ieu-b-102	TRUE	reported	WYslv6
3	1.18E+08	A	G	0.0285	0.0048	3.04E-09	0.278	NA	170756	rs7318246	329443	ieu-b-102	TRUE	reported	WYslv6
3	1.18E+08	A	G	0.0285	0.0048	3.12E-09	0.2781	NA	170756	rs7318246	329443	ieu-b-102	TRUE	reported	WYslv6
3	1.18E+08	A	G	0.0277	0.0048	8.07E-09	0.2784	NA	170756	rs7316370	329443	ieu-b-102	TRUE	reported	WYslv6
3	1.18E+08	G	A	0.0278	0.0051	3.77E-08	0.2653	NA	170756	rs1228177	329443	ieu-b-102	TRUE	reported	WYslv6
3	1.18E+08	A	G	0.0278	0.0048	6.32E-09	0.2782	NA	170756	rs5570751	329443	ieu-b-102	TRUE	reported	WYslv6
3	1.18E+08	T	A	0.0281	0.0048	4.66E-09	0.2777	NA	170756	rs7316371	329443	ieu-b-102	TRUE	reported	WYslv6
3	1.18E+08	A	G	0.028	0.0048	5.32E-09	0.278	NA	170756	rs7316371	329443	ieu-b-102	TRUE	reported	WYslv6
3	1.18E+08	A	C	0.028	0.0048	5.16E-09	0.278	NA	170756	rs7316371	329443	ieu-b-102	TRUE	reported	WYslv6
3	1.18E+08	C	T	0.028	0.0048	5.08E-09	0.2776	NA	170756	rs7316371	329443	ieu-b-102	TRUE	reported	WYslv6

3	1.18E+08	C	T	0.0279	0.0048	5.68E-09	0.2776	NA	170756	rs4688064	329443	ieu-b-102	TRUE	reported	WYslv6
3	1.18E+08	C	A	0.0281	0.0048	4.98E-09	0.2783	NA	170756	rs6792102	329443	ieu-b-102	TRUE	reported	WYslv6
3	1.18E+08	C	T	0.028	0.0048	5.27E-09	0.2777	NA	170756	rs7316372	329443	ieu-b-102	TRUE	reported	WYslv6
3	1.18E+08	T	C	0.0277	0.0048	7.62E-09	0.2781	NA	170756	rs7316373	329443	ieu-b-102	TRUE	reported	WYslv6
3	1.18E+08	A	T	0.0278	0.0048	6.63E-09	0.278	NA	170756	rs7316373	329443	ieu-b-102	TRUE	reported	WYslv6
3	1.18E+08	A	G	0.028	0.0048	4.94E-09	0.2781	NA	170756	rs7432631	329443	ieu-b-102	TRUE	reported	WYslv6
3	1.18E+08	C	G	0.0281	0.0048	4.34E-09	0.2779	NA	170756	rs3488995	329443	ieu-b-102	TRUE	reported	WYslv6
3	1.18E+08	G	T	0.0288	0.0048	2.10E-09	0.2741	NA	170756	rs7543989	329443	ieu-b-102	TRUE	reported	WYslv6
3	1.18E+08	T	C	0.0278	0.0048	7.06E-09	0.2767	NA	170756	rs7427162	329443	ieu-b-102	TRUE	reported	WYslv6
3	1.18E+08	C	T	0.0273	0.0048	1.19E-08	0.2761	NA	170756	rs7316377	329443	ieu-b-102	TRUE	reported	WYslv6
3	1.18E+08	A	G	0.0277	0.0048	8.20E-09	0.2756	NA	170756	rs1171060	329443	ieu-b-102	TRUE	reported	WYslv6
3	1.18E+08	T	C	0.0277	0.0048	7.83E-09	0.2756	NA	170756	rs7316377	329443	ieu-b-102	TRUE	reported	WYslv6
3	1.18E+08	G	A	0.0277	0.0048	7.72E-09	0.2756	NA	170756	rs7316377	329443	ieu-b-102	TRUE	reported	WYslv6
3	1.18E+08	G	C	0.0277	0.0048	7.43E-09	0.2755	NA	170756	rs7316378	329443	ieu-b-102	TRUE	reported	WYslv6
3	1.18E+08	G	A	0.0273	0.0048	1.44E-08	0.2777	NA	170756	rs1396362	329443	ieu-b-102	TRUE	reported	WYslv6
3	1.18E+08	G	A	0.0268	0.0049	3.42E-08	0.2722	NA	170756	rs1439147	329443	ieu-b-102	TRUE	reported	WYslv6
3	1.18E+08	T	C	0.0275	0.0048	1.09E-08	0.2757	NA	170756	rs7316378	329443	ieu-b-102	TRUE	reported	WYslv6
3	1.18E+08	T	G	0.0274	0.0048	1.17E-08	0.2753	NA	170756	rs7316378	329443	ieu-b-102	TRUE	reported	WYslv6
3	1.18E+08	T	C	0.0275	0.0048	1.18E-08	0.2749	NA	170756	rs6791692	329443	ieu-b-102	TRUE	reported	WYslv6
3	1.18E+08	G	T	0.0274	0.0048	1.24E-08	0.2756	NA	170756	rs6778857	329443	ieu-b-102	TRUE	reported	WYslv6
3	1.18E+08	T	C	0.0276	0.0048	9.47E-09	0.2754	NA	170756	rs1766581	329443	ieu-b-102	TRUE	reported	WYslv6
3	1.18E+08	G	C	0.0276	0.0048	9.58E-09	0.2755	NA	170756	rs4687901	329443	ieu-b-102	TRUE	reported	WYslv6
3	1.18E+08	G	A	0.0275	0.0048	1.01E-08	0.2755	NA	170756	rs7316379	329443	ieu-b-102	TRUE	reported	WYslv6
3	1.18E+08	T	C	0.0275	0.0048	1.04E-08	0.2756	NA	170756	rs1171696	329443	ieu-b-102	TRUE	reported	WYslv6
3	1.18E+08	A	G	0.0274	0.0048	1.17E-08	0.2755	NA	170756	rs7617580	329443	ieu-b-102	TRUE	reported	WYslv6
3	1.18E+08	C	T	0.0277	0.0048	8.35E-09	0.2754	NA	170756	rs7318303	329443	ieu-b-102	TRUE	reported	WYslv6
3	1.18E+08	T	C	0.0277	0.0048	8.58E-09	0.2754	NA	170756	rs6766743	329443	ieu-b-102	TRUE	reported	WYslv6
3	1.18E+08	G	T	0.0276	0.0048	9.47E-09	0.2752	NA	170756	rs6791342	329443	ieu-b-102	TRUE	reported	WYslv6
3	1.18E+08	G	A	0.0277	0.0048	8.24E-09	0.2752	NA	170756	rs7316830	329443	ieu-b-102	TRUE	reported	WYslv6
3	1.18E+08	T	C	0.0278	0.0048	7.61E-09	0.2753	NA	170756	rs7316830	329443	ieu-b-102	TRUE	reported	WYslv6
3	1.18E+08	C	T	0.028	0.0048	6.21E-09	0.2744	NA	170756	rs1766605	329443	ieu-b-102	TRUE	reported	WYslv6
3	1.18E+08	A	T	0.028	0.0048	5.73E-09	0.2741	NA	170756	rs1772262	329443	ieu-b-102	TRUE	reported	WYslv6
3	1.18E+08	A	C	0.028	0.0048	6.25E-09	0.2743	NA	170756	rs7316837	329443	ieu-b-102	TRUE	reported	WYslv6
3	1.18E+08	A	G	0.0282	0.0048	4.47E-09	0.2739	NA	170756	rs6765305	329443	ieu-b-102	TRUE	reported	WYslv6
3	1.18E+08	G	T	0.0286	0.0048	2.95E-09	0.274	NA	170756	rs4688071	329443	ieu-b-102	TRUE	reported	WYslv6
3	1.18E+08	C	T	0.0281	0.0048	5.20E-09	0.2747	NA	170756	rs1563438	329443	ieu-b-102	TRUE	reported	WYslv6
3	1.18E+08	G	C	0.0283	0.0048	3.93E-09	0.2746	NA	170756	rs1610148	329443	ieu-b-102	TRUE	reported	WYslv6
3	1.18E+08	T	C	0.027	0.0048	2.46E-08	0.271	NA	170756	rs1266941	329443	ieu-b-102	TRUE	reported	WYslv6
3	1.18E+08	G	A	0.0267	0.0048	3.38E-08	0.2707	NA	170756	rs7316839	329443	ieu-b-102	TRUE	reported	WYslv6
3	1.18E+08	T	C	0.0267	0.0048	3.40E-08	0.2707	NA	170756	rs7316840	329443	ieu-b-102	TRUE	reported	WYslv6
3	1.18E+08	G	A	0.0267	0.0048	3.23E-08	0.2712	NA	170756	rs6438430	329443	ieu-b-102	TRUE	reported	WYslv6
3	1.18E+08	T	C	0.0265	0.0048	4.32E-08	0.272	NA	170756	rs5595842	329443	ieu-b-102	TRUE	reported	WYslv6
3	1.18E+08	G	C	0.0275	0.0049	1.45E-08	0.2686	NA	170756	rs7316840	329443	ieu-b-102	TRUE	reported	WYslv6
3	1.18E+08	C	T	0.0272	0.0048	1.77E-08	0.2727	NA	170756	rs7643173	329443	ieu-b-102	TRUE	reported	WYslv6
3	1.18E+08	T	C	0.0271	0.0048	1.93E-08	0.2727	NA	170756	rs7317660	329443	ieu-b-102	TRUE	reported	WYslv6
3	1.18E+08	G	A	0.0271	0.0048	1.90E-08	0.2727	NA	170756	rs7317660	329443	ieu-b-102	TRUE	reported	WYslv6
3	1.18E+08	C	T	0.0277	0.0048	9.42E-09	0.2726	NA	170756	rs1772296	329443	ieu-b-102	TRUE	reported	WYslv6
3	1.18E+08	C	T	0.0277	0.0048	9.48E-09	0.2723	NA	170756	rs1772297	329443	ieu-b-102	TRUE	reported	WYslv6
3	1.18E+08	C	T	0.0277	0.0048	8.91E-09	0.2723	NA	170756	rs1171225	329443	ieu-b-102	TRUE	reported	WYslv6
3	1.18E+08	C	T	0.0277	0.0048	9.51E-09	0.2723	NA	170756	rs5640174	329443	ieu-b-102	TRUE	reported	WYslv6
3	1.18E+08	C	A	0.0278	0.0048	8.14E-09	0.2723	NA	170756	rs7317851	329443	ieu-b-102	TRUE	reported	WYslv6
3	1.18E+08	A	G	0.0276	0.0049	2.01E-08	0.2572	NA	170756	rs7627393	329443	ieu-b-102	TRUE	reported	WYslv6
3	1.18E+08	G	A	0.0277	0.0049	1.82E-08	0.2575	NA	170756	rs1165808	329443	ieu-b-102	TRUE	reported	WYslv6
3	1.18E+08	T	G	0.0259	0.0047	4.15E-08	0.2926	NA	170756	rs7632354	329443	ieu-b-102	TRUE	reported	WYslv6
3	1.18E+08	A	G	0.0273	0.0049	2.78E-08	0.2575	NA	170756	rs6703823	329443	ieu-b-102	TRUE	reported	WYslv6
3	1.18E+08	A	G	0.0257	0.0047	4.30E-08	0.2959	NA	170756	rs1170911	329443	ieu-b-102	TRUE	reported	WYslv6
3	1.18E+08	A	G	0.0258	0.0047	3.94E-08	0.2958	NA	170756	rs1450978	329443	ieu-b-102	TRUE	reported	WYslv6
3	1.18E+08	G	A	0.0277	0.0049	1.62E-08	0.2569	NA	170756	rs1499969	329443	ieu-b-102	TRUE	reported	WYslv6
3	1.18E+08	C	A	0.0256	0.0047	4.89E-08	0.2958	NA	170756	rs1772330	329443	ieu-b-102	TRUE	reported	WYslv6
3	1.18E+08	A	G	0.0269	0.0049	4.53E-08	0.2567	NA	170756	rs1766708	329443	ieu-b-102	TRUE	reported	WYslv6
3	1.18E+08	C	A	0.0271	0.0049	3.53E-08	0.2566	NA	170756	rs7317852	329443	ieu-b-102	TRUE	reported	WYslv6
3	1.18E+08	G	A	0.0272	0.0049	3.87E-08	0.2581	NA	170756	rs1502860	329443	ieu-b-102	TRUE	reported	WYslv6
3	1.18E+08	G	A	0.0271	0.0049	3.70E-08	0.2563	NA	170756	rs5605752	329443	ieu-b-102	TRUE	reported	WYslv6
3	1.18E+08	G	A	0.027	0.0049	4.14E-08	0.2564	NA	170756	rs5862475	329443	ieu-b-102	TRUE	reported	WYslv6
3	1.18E+08	T	C	0.0272	0.0049	3.44E-08	0.2564	NA	170756	rs7318590	329443	ieu-b-102	TRUE	reported	WYslv6
3	1.18E+08	G	A	0.0277	0.0049	2.02E-08	0.2563	NA	170756	rs7318591	329443	ieu-b-102	TRUE	reported	WYslv6
3	1.18E+08	G	A	0.0274	0.005	4.37E-08	0.2497	NA	170756	rs7318592	329443	ieu-b-102	TRUE	reported	WYslv6
3	1.18E+08	A	C	0.0277	0.005	2.93E-08	0.2501	NA	170756	rs1772473	329443	ieu-b-102	TRUE	reported	WYslv6
3	1.18E+08	G	A	0.0276	0.005	3.56E-08	0.2493	NA	170756	rs7318592	329443	ieu-b-102	TRUE	reported	WYslv6
3	1.18E+08	A	G	0.0277	0.005	2.33E-08	0.2532	NA	170756	rs5598962	329443	ieu-b-102	TRUE	reported	WYslv6
3	1.18E+08	T	C	0.0277	0.005	2.75E-08	0.2533	NA	170756	rs7619072	329443	ieu-b-102	TRUE	reported	WYslv6
3	1.58E+08	T	G	0.024	0.0044	3.79E-08	0.4171	NA	170756	rs699927	329443	ieu-b-102	TRUE	reported	WYslv6
3	1.58E+08	C	G	0.0239	0.0044	3.99E-08	0.4158	NA	170756	rs7636974	329443	ieu-b-102	TRUE	reported	WYslv6
3	1.58E+08	A	T	0.0238	0.0044	4.92E-08	0.4166	NA	170756	rs1420698	329443	ieu-b-102	TRUE	reported	WYslv6
3	1.58E+08	G	A	-0.0238	0.0044	4.88E-08	0.583	NA	170756	rs7430565	329443	ieu-b-102	TRUE	reported	WYslv6
3	1.58E+08	T	C	-0.0239	0.0044	4.15E-08	0.583	NA	170756	rs2882092	329443	ieu-b-102	TRUE	reported	WYslv6
3	1.58E+08	A	G	-0.0241	0.0044	3.28E-08	0.5774	NA	170756	rs3546963	329443	ieu-b-102	TRUE	reported	WYslv6
5	87545318	C	T	-0.0241	0.0043	2.78E-08	0.5351	NA	170756	rs8798910	329443	ieu-b-102	TRUE	reported	WYslv6
5	87630769	A	G	0.0237	0.0043	4.71E-08	0.457	NA	170756	rs247910	329443	ieu-b-102	TRUE	reported	WYslv6
5	1.04E+08	A	G	0.0283	0.0045	2.56E-10	0.3709	NA	170756	rs349588	329443	ieu-b-102	TRUE	reported	WYslv6
5	1.04E+08	G	A	0.0277	0.0045	6.15E-10	0.3669	NA	170756	rs349586	329443	ieu-b-102	TRUE	reported	WYslv6
5	1.04E+08	C	A	0.027	0.0045	1.80E-09	0.3657	NA	170756	rs168388	329443	ieu-b-102	TRUE	reported	WYslv6
5	1.04E+08	G	A	0.0287	0.0045	1.92E-10	0.3544	NA	170756	rs1308622	329443	ieu-b-102	TRUE	reported	WYslv6
5	1.04E+08	A	G	-0.0239	0.0043	3.41E-08	0.5474	NA	170756	rs9753089	329443	ieu-b-102	TRUE	reported	WYslv6
5	1.04E+08	G	C	0.0298	0.0045	4.03E-11	0.3484	NA	170756	rs4703300	329443	ieu-b-102	TRUE	reported	WYslv6
5	1.04E+08	A	C	0.0301	0.0045	1.94E-11	0.3532	NA	170756	rs2896539	329443	ieu-b-102	TRUE	reported	WYslv6
5	1.04E+08	A	G	0.0299	0.0045	2.91E-11	0.3519	NA	170756	rs2963222	329443	ieu-b-102	TRUE	reported	WYslv6
5	1.04E+08	A	T	0.0307	0.0045	8.06E-12	0.3482	NA	170756	rs1372502	329443	ieu-b-102	TRUE	reported	WYslv6
5	1.04E+08	C	T	0.0306	0.0045	9.21E-12	0.3488	NA	170756	rs1899386	329443	ieu-b-102	TRUE	reported	WYslv6
5	1.04E+08	A	G	0.0301	0.0045	1.94E-11	0.3502	NA	170756	rs964040	329443	ieu-b-102	TRUE	reported	WYslv6
5	1.04E+08	G	A	0.0298	0.0045	3.31E-11	0.3494	NA	170756	rs2963227	329443	ieu-b-102	TRUE	reported	WYslv6
5	1.04E+08	A	C	0.0295	0.0045	3.62E-11	0.3694	NA	170756	rs1442114	329443	ieu-b-102	TRUE	reported	WYslv6
5	1.04E+08	C	T	0.0311	0.0045	5.28E-12	0.3447	NA	170756	rs1530303	329443	ieu-b-102	TRUE	reported	WYslv6
5	1.04E+08	C	T	0.0315	0.0045	2.40E-12	0.3464	NA	170756	rs4510551	329443	ieu-b-102	TRUE	reported	WYslv6
5	1.04E+08	T	C	0.0299	0.0044	1.63E-11	0.3725	NA	170756	rs2403304	329443	ieu-b-102	TRUE	reported	WYslv6
5	1.04E+08	C	T	0.0318	0.0045	1.67E-12	0.3469	NA	170756	rs3594960	329443	ieu-b-102	TRUE	reported	WYslv6
5	1.04E+08	T	C	0.0316	0.0045	2.55E-12	0.3466	NA	170756	rs1316292	329443	ieu-b-102	TRUE	reported	WYslv6
5	1.04E+08	G	A	0.0305	0.0045	1.26E-11	0.3506	NA	170756	rs1318167	329443	ieu-b-102	TRUE	reported	WYslv6
5	1.04E+08	C	T	0.0319	0.0045	1.81E-12	0.3449	NA	170756	rs1316640	329443	ieu-b-102	TRUE	reported	WYslv6
5	1.04E+08	C	T	0.0307	0.0044	5.25E-12	0.3706	NA	170756	rs5564912	329443	ieu-b-102	TRUE	reported	WYslv6
5	1.04E+08	A	G	0.0312	0.0045	4.57E-12	0.346	NA	170756	rs1317747	329443	ieu-b-102	TRUE	reported	WYslv6
5	1.04E+08	G	A	0.0301	0.0044	1.16E-11	0.375	NA	170756	rs1372504	329443	ieu-b-102	TRUE	reported	WYslv6
5	1.04E+08	C	T	0.0313	0.0045	3.94E-12	0.345	NA	170756	rs1583953	329443	ieu-b-102	TRUE	reported	WYslv6
5	1.04E+08	G	A	0.0314	0.0045	3.38E-12	0.3448	NA	170756	rs1450712	329443	ieu-b-102	TRUE	reported	WYslv6
5	1.04E+08	A	G	0.0315	0.0045	2.64E-12	0.347	NA	170756	rs768792	329443	ieu-b-102	TRUE	reported	WYslv6
5	1.04E+08	T	C	0.0319	0.0045	1.43E-12	0.3486	NA	170756	rs1372500	329443	ieu-b-102	TRUE	reported	WYslv6
5	1.04E+08	T	C	0.0315	0.0045	2.51E-12	0.3511	NA	170756	rs2028526	329443	ieu-b-102	TRUE	reported	WYslv6

5	1.04E+08	A	T	0.029	0.0046	3.52E-10	0.3804	NA	170756	rs1325506	329443	ieu-b-102	TRUE	reported	WYslv6
5	1.04E+08	C	T	0.0316	0.0045	2.33E-12	0.352	NA	170756	rs876392	329443	ieu-b-102	TRUE	reported	WYslv6
5	1.04E+08	G	T	0.0314	0.0045	3.83E-12	0.3437	NA	170756	rs1442111	329443	ieu-b-102	TRUE	reported	WYslv6
5	1.04E+08	G	A	0.0315	0.0045	2.71E-12	0.3495	NA	170756	rs2919955	329443	ieu-b-102	TRUE	reported	WYslv6
5	1.04E+08	G	A	0.0309	0.0045	8.68E-12	0.3447	NA	170756	rs920623	329443	ieu-b-102	TRUE	reported	WYslv6
5	1.04E+08	G	C	0.0338	0.0045	9.52E-14	0.3563	NA	170756	rs7602540	329443	ieu-b-102	TRUE	reported	WYslv6
5	1.04E+08	G	A	0.0247	0.0043	1.04E-08	0.4867	NA	170756	rs1316652	329443	ieu-b-102	TRUE	reported	WYslv6
5	1.04E+08	A	G	0.0305	0.0044	6.56E-12	0.379	NA	170756	rs4235642	329443	ieu-b-102	TRUE	reported	WYslv6
5	1.04E+08	G	A	0.0238	0.0043	3.53E-08	0.4843	NA	170756	rs3866907	329443	ieu-b-102	TRUE	reported	WYslv6
5	1.04E+08	A	G	0.0238	0.0043	3.21E-08	0.4853	NA	170756	rs6790992	329443	ieu-b-102	TRUE	reported	WYslv6
5	1.04E+08	T	A	0.0237	0.0043	3.73E-08	0.4853	NA	170756	rs1046400	329443	ieu-b-102	TRUE	reported	WYslv6
5	1.04E+08	G	A	0.0235	0.0043	4.49E-08	0.4853	NA	170756	rs7706513	329443	ieu-b-102	TRUE	reported	WYslv6
5	1.04E+08	T	C	0.0236	0.0043	4.35E-08	0.4843	NA	170756	rs1316528	329443	ieu-b-102	TRUE	reported	WYslv6
5	1.04E+08	T	G	0.0238	0.0043	2.93E-08	0.4851	NA	170756	rs1436523	329443	ieu-b-102	TRUE	reported	WYslv6
5	1.04E+08	T	G	0.0237	0.0043	3.37E-08	0.4859	NA	170756	rs1318710	329443	ieu-b-102	TRUE	reported	WYslv6
5	1.04E+08	C	T	0.0238	0.0043	3.14E-08	0.4851	NA	170756	rs1195822	329443	ieu-b-102	TRUE	reported	WYslv6
5	1.04E+08	C	T	0.0237	0.0043	3.51E-08	0.4853	NA	170756	rs1174712	329443	ieu-b-102	TRUE	reported	WYslv6
5	1.04E+08	C	A	0.0238	0.0043	2.92E-08	0.4855	NA	170756	rs4703307	329443	ieu-b-102	TRUE	reported	WYslv6
5	1.04E+08	T	A	0.0247	0.0043	1.02E-08	0.4848	NA	170756	rs7999786	329443	ieu-b-102	TRUE	reported	WYslv6
5	1.04E+08	A	C	0.0238	0.0043	3.31E-08	0.4991	NA	170756	rs1195537	329443	ieu-b-102	TRUE	reported	WYslv6
5	1.04E+08	G	A	0.0239	0.0043	2.51E-08	0.4857	NA	170756	rs969397	329443	ieu-b-102	TRUE	reported	WYslv6
5	1.04E+08	G	C	0.0239	0.0043	2.46E-08	0.4995	NA	170756	rs3531995	329443	ieu-b-102	TRUE	reported	WYslv6
5	1.04E+08	G	A	0.0242	0.0043	1.79E-08	0.4854	NA	170756	rs1363098	329443	ieu-b-102	TRUE	reported	WYslv6
5	1.04E+08	C	G	0.024	0.0043	2.33E-08	0.4853	NA	170756	rs6870957	329443	ieu-b-102	TRUE	reported	WYslv6
5	1.04E+08	T	C	0.024	0.0043	2.23E-08	0.4995	NA	170756	rs1582419	329443	ieu-b-102	TRUE	reported	WYslv6
5	1.04E+08	T	G	0.0237	0.0043	3.49E-08	0.486	NA	170756	rs4515268	329443	ieu-b-102	TRUE	reported	WYslv6
5	1.04E+08	C	T	0.0237	0.0043	3.11E-08	0.4858	NA	170756	rs1363097	329443	ieu-b-102	TRUE	reported	WYslv6
5	1.04E+08	G	A	0.0237	0.0043	3.31E-08	0.4848	NA	170756	rs1363096	329443	ieu-b-102	TRUE	reported	WYslv6
5	1.04E+08	G	C	0.0238	0.0043	3.07E-08	0.4849	NA	170756	rs7731266	329443	ieu-b-102	TRUE	reported	WYslv6
5	1.04E+08	A	C	0.0239	0.0043	2.62E-08	0.4849	NA	170756	rs4482879	329443	ieu-b-102	TRUE	reported	WYslv6
5	1.04E+08	G	T	0.0238	0.0043	2.71E-08	0.4849	NA	170756	rs4703040	329443	ieu-b-102	TRUE	reported	WYslv6
5	1.04E+08	T	G	0.0259	0.0043	2.27E-09	0.4783	NA	170756	rs6869862	329443	ieu-b-102	TRUE	reported	WYslv6
5	1.04E+08	G	C	0.0339	0.0045	4.06E-14	0.3557	NA	170756	rs1592757	329443	ieu-b-102	TRUE	reported	WYslv6
5	1.04E+08	C	T	0.0265	0.0043	7.66E-10	0.464	NA	170756	rs6867409	329443	ieu-b-102	TRUE	reported	WYslv6
5	1.04E+08	C	G	0.0265	0.0043	7.94E-10	0.4632	NA	170756	rs2193933	329443	ieu-b-102	TRUE	reported	WYslv6
5	1.04E+08	A	T	0.0262	0.0043	1.18E-09	0.4627	NA	170756	rs1592756	329443	ieu-b-102	TRUE	reported	WYslv6
5	1.04E+08	T	A	0.0266	0.0043	6.80E-10	0.4637	NA	170756	rs1592755	329443	ieu-b-102	TRUE	reported	WYslv6
5	1.04E+08	C	T	0.0274	0.0043	1.58E-10	0.4807	NA	170756	rs7706353	329443	ieu-b-102	TRUE	reported	WYslv6
5	1.04E+08	T	G	0.0266	0.0043	6.23E-10	0.4636	NA	170756	rs4438849	329443	ieu-b-102	TRUE	reported	WYslv6
5	1.04E+08	T	C	0.0274	0.0043	1.56E-10	0.4808	NA	170756	rs4320234	329443	ieu-b-102	TRUE	reported	WYslv6
5	1.04E+08	T	C	0.0274	0.0043	1.56E-10	0.481	NA	170756	rs1833514	329443	ieu-b-102	TRUE	reported	WYslv6
5	1.04E+08	T	C	0.0274	0.0043	1.69E-10	0.4807	NA	170756	rs4521446	329443	ieu-b-102	TRUE	reported	WYslv6
5	1.04E+08	A	G	0.0274	0.0043	1.57E-10	0.4807	NA	170756	rs6596578	329443	ieu-b-102	TRUE	reported	WYslv6
5	1.04E+08	T	C	-0.0267	0.0043	5.63E-10	0.5369	NA	170756	rs6874138	329443	ieu-b-102	TRUE	reported	WYslv6
5	1.04E+08	T	C	-0.0275	0.0043	1.48E-10	0.5193	NA	170756	rs1006442	329443	ieu-b-102	TRUE	reported	WYslv6
5	1.04E+08	C	T	-0.0266	0.0043	6.30E-10	0.5365	NA	170756	rs1005913	329443	ieu-b-102	TRUE	reported	WYslv6
5	1.04E+08	G	C	-0.0337	0.0045	9.29E-14	0.6442	NA	170756	rs1421669	329443	ieu-b-102	TRUE	reported	WYslv6
5	1.04E+08	C	T	-0.0273	0.0043	2.05E-10	0.5191	NA	170756	rs6865511	329443	ieu-b-102	TRUE	reported	WYslv6
5	1.04E+08	C	T	-0.0275	0.0043	1.49E-10	0.5193	NA	170756	rs1421668	329443	ieu-b-102	TRUE	reported	WYslv6
5	1.04E+08	A	G	-0.0275	0.0043	1.53E-10	0.5193	NA	170756	rs6891820	329443	ieu-b-102	TRUE	reported	WYslv6
5	1.04E+08	G	A	-0.0266	0.0043	6.18E-10	0.5365	NA	170756	rs1473144	329443	ieu-b-102	TRUE	reported	WYslv6
5	1.04E+08	A	G	-0.0272	0.0043	2.63E-10	0.5191	NA	170756	rs1265753	329443	ieu-b-102	TRUE	reported	WYslv6
5	1.04E+08	A	G	-0.0271	0.0043	3.18E-10	0.5153	NA	170756	rs1265800	329443	ieu-b-102	TRUE	reported	WYslv6
5	1.04E+08	T	C	-0.027	0.0043	4.00E-10	0.5145	NA	170756	rs1265845	329443	ieu-b-102	TRUE	reported	WYslv6
5	1.04E+08	A	G	-0.027	0.0043	4.06E-10	0.5477	NA	170756	rs1337525	329443	ieu-b-102	TRUE	reported	WYslv6
5	1.04E+08	A	G	-0.0344	0.0045	2.25E-14	0.6436	NA	170756	rs1265803	329443	ieu-b-102	TRUE	reported	WYslv6
5	1.04E+08	G	A	-0.0279	0.0044	1.47E-10	0.5088	NA	170756	rs1265996	329443	ieu-b-102	TRUE	reported	WYslv6
5	1.04E+08	T	C	-0.0273	0.0043	1.89E-10	0.5194	NA	170756	rs1124252	329443	ieu-b-102	TRUE	reported	WYslv6
5	1.04E+08	T	A	-0.0274	0.0043	1.69E-10	0.5194	NA	170756	rs1124252	329443	ieu-b-102	TRUE	reported	WYslv6
5	1.04E+08	C	T	-0.0273	0.0043	1.85E-10	0.5194	NA	170756	rs1251542	329443	ieu-b-102	TRUE	reported	WYslv6
5	1.04E+08	A	C	-0.0274	0.0043	1.79E-10	0.5195	NA	170756	rs1715667	329443	ieu-b-102	TRUE	reported	WYslv6
5	1.04E+08	C	T	-0.0274	0.0043	1.77E-10	0.5194	NA	170756	rs2403284	329443	ieu-b-102	TRUE	reported	WYslv6
5	1.04E+08	G	A	-0.0272	0.0043	2.60E-10	0.5322	NA	170756	rs7703746	329443	ieu-b-102	TRUE	reported	WYslv6
5	1.04E+08	G	A	-0.0273	0.0043	1.85E-10	0.5194	NA	170756	rs1421667	329443	ieu-b-102	TRUE	reported	WYslv6
5	1.04E+08	C	T	-0.0272	0.0043	2.45E-10	0.5193	NA	170756	rs2032790	329443	ieu-b-102	TRUE	reported	WYslv6
5	1.04E+08	A	G	-0.0272	0.0043	2.37E-10	0.5193	NA	170756	rs1421666	329443	ieu-b-102	TRUE	reported	WYslv6
5	1.04E+08	C	T	-0.0272	0.0043	2.35E-10	0.5193	NA	170756	rs4295362	329443	ieu-b-102	TRUE	reported	WYslv6
5	1.04E+08	C	T	-0.0273	0.0043	2.25E-10	0.5193	NA	170756	rs959857	329443	ieu-b-102	TRUE	reported	WYslv6
5	1.04E+08	T	C	-0.0273	0.0043	2.26E-10	0.5193	NA	170756	rs768905	329443	ieu-b-102	TRUE	reported	WYslv6
5	1.04E+08	C	T	-0.0284	0.0044	8.58E-11	0.5129	NA	170756	rs1203271	329443	ieu-b-102	TRUE	reported	WYslv6
5	1.04E+08	A	C	-0.0282	0.0043	6.32E-11	0.5143	NA	170756	rs6236244	329443	ieu-b-102	TRUE	reported	WYslv6
5	1.04E+08	C	A	-0.028	0.0043	7.90E-11	0.5102	NA	170756	rs1007111	329443	ieu-b-102	TRUE	reported	WYslv6
5	1.04E+08	A	G	-0.0289	0.0043	2.02E-11	0.5064	NA	170756	rs1007880	329443	ieu-b-102	TRUE	reported	WYslv6
5	1.04E+08	A	G	-0.0282	0.0043	5.80E-11	0.5115	NA	170756	rs1173819	329443	ieu-b-102	TRUE	reported	WYslv6
5	1.04E+08	A	C	-0.0281	0.0043	6.30E-11	0.5111	NA	170756	rs1173819	329443	ieu-b-102	TRUE	reported	WYslv6
5	1.04E+08	C	T	-0.0281	0.0043	6.40E-11	0.5111	NA	170756	rs1317261	329443	ieu-b-102	TRUE	reported	WYslv6
5	1.04E+08	T	A	-0.0281	0.0043	6.24E-11	0.5115	NA	170756	rs1315849	329443	ieu-b-102	TRUE	reported	WYslv6
5	1.04E+08	A	T	-0.0275	0.0043	1.61E-10	0.5104	NA	170756	rs1217716	329443	ieu-b-102	TRUE	reported	WYslv6
5	1.04E+08	C	T	-0.0282	0.0043	5.75E-11	0.5115	NA	170756	rs1421665	329443	ieu-b-102	TRUE	reported	WYslv6
5	1.04E+08	G	A	-0.0282	0.0043	6.10E-11	0.5116	NA	170756	rs1317736	329443	ieu-b-102	TRUE	reported	WYslv6
5	1.04E+08	A	G	-0.0282	0.0043	6.07E-11	0.5116	NA	170756	rs1005336	329443	ieu-b-102	TRUE	reported	WYslv6
5	1.04E+08	A	T	-0.0282	0.0043	5.30E-11	0.5122	NA	170756	rs1005337	329443	ieu-b-102	TRUE	reported	WYslv6
5	1.04E+08	T	G	-0.0281	0.0043	6.10E-11	0.5115	NA	170756	rs1005497	329443	ieu-b-102	TRUE	reported	WYslv6
5	1.04E+08	A	G	-0.0281	0.0043	6.14E-11	0.5115	NA	170756	rs1007392	329443	ieu-b-102	TRUE	reported	WYslv6
5	1.04E+08	A	G	-0.0281	0.0043	6.39E-11	0.5113	NA	170756	rs1265827	329443	ieu-b-102	TRUE	reported	WYslv6
5	1.04E+08	G	A	-0.0285	0.0043	3.81E-11	0.512	NA	170756	rs1047929	329443	ieu-b-102	TRUE	reported	WYslv6
5	1.04E+08	A	G	-0.0285	0.0043	3.52E-11	0.5121	NA	170756	rs1277549	329443	ieu-b-102	TRUE	reported	WYslv6
5	1.04E+08	T	C	-0.0281	0.0043	6.20E-11	0.5115	NA	170756	rs7966648	329443	ieu-b-102	TRUE	reported	WYslv6
5	1.04E+08	G	C	-0.0281	0.0043	6.78E-11	0.5118	NA	170756	rs7723509	329443	ieu-b-102	TRUE	reported	WYslv6
5	1.04E+08	A	G	-0.028	0.0043	7.28E-11	0.5115	NA	170756	rs6236245	329443	ieu-b-102	TRUE	reported	WYslv6
5	1.04E+08	A	G	-0.0285	0.0043	4.83E-11	0.5098	NA	170756	rs1491393	329443	ieu-b-102	TRUE	reported	WYslv6
5	1.04E+08	A	G	-0.0285	0.0043	5.17E-11	0.5101	NA	170756	rs9457197	329443	ieu-b-102	TRUE	reported	WYslv6
5	1.04E+08	A	C	-0.0281	0.0043	6.42E-11	0.5115	NA	170756	rs1047783	329443	ieu-b-102	TRUE	reported	WYslv6
5	1.04E+08	C	T	-0.0291	0.0043	1.60E-11	0.5123	NA	170756	rs1363105	329443	ieu-b-102	TRUE	reported	WYslv6
5	1.04E+08	C	G	-0.0291	0.0043	1.56E-11	0.5124	NA	170756	rs1363104	329443	ieu-b-102	TRUE	reported	WYslv6
5	1.04E+08	G	T	-0.0281	0.0043	6.69E-11	0.5115	NA	170756	rs1363102	329443	ieu-b-102	TRUE	reported	WYslv6
5	1.04E+08	C	T	-0.0281	0.0043	6.59E-11	0.5115	NA	170756	rs1007257	329443	ieu-b-102	TRUE	reported	WYslv6
5	1.04E+08	C	T	-0.0281	0.0043	6.40E-11	0.5113	NA	170756	rs3866907	329443	ieu-b-102	TRUE	reported	WYslv6
5	1.04E+08	G	A	-0.028	0.0043	7.39E-11	0.5115	NA	170756	rs1005745	329443	ieu-b-102	TRUE	reported	WYslv6
5	1.04E+08	G	T	-0.0281	0.0043	6.81E-11	0.5115	NA	170756	rs1005746	329443	ieu-b-102	TRUE	reported	WYslv6
5	1.04E+08	G	T	0.0345	0.006	1.02E-08	0.1582	NA	170756	rs6759760	329443	ieu-b-102	TRUE	reported	WYslv6
5	1.04E+08	A	C	-0.0281	0.0043	6.28E-11	0.5115	NA	170756	rs1005280	329443	ieu-b-102	TRUE	reported	WYslv6
5	1.04E+08	G	A	-0.0281	0.0043	6.47E-11	0.5115	NA	170756	rs1005964	329443	ieu-b-102	TRUE	reported	WYslv6
5	1.04E+08	C	A	0.0286	0.0043	3.69E-11	0.4495	NA	170756	rs254011	329443	ieu-b-102	TRUE	reported	WYslv6
5	1.04E+08	C	T	0.028	0.0043	1.20E-10	0.4509	NA	170756	rs254013	329443	ieu-b-102	TRUE	reported	WYslv6
5	1.04E+08	T	A	0.0282	0.0043	7.58E-11	0.4366	NA	170756	rs445578	329443	ieu-b-102	TRUE	reported	WYslv6
5	1.04E+08	C	T	0.0284	0.0043	5.20E-11	0.4365	NA	170756	rs2447838	329443	ieu-b-102	TRUE	reported	WYslv6

5	1.04E+08	G	A	0.0284	0.0043	5.93E-11	0.4373	NA	170756	rs2431112	329443	ieu-b-102	TRUE	reported	WYslv6
5	1.04E+08	C	T	0.0284	0.0043	4.79E-11	0.4368	NA	170756	rs2447832	329443	ieu-b-102	TRUE	reported	WYslv6
5	1.04E+08	T	A	0.0285	0.0043	4.57E-11	0.4361	NA	170756	rs254035	329443	ieu-b-102	TRUE	reported	WYslv6
5	1.04E+08	C	T	-0.0279	0.0043	8.42E-11	0.5118	NA	170756	rs1592754	329443	ieu-b-102	TRUE	reported	WYslv6
5	1.04E+08	C	T	-0.0272	0.0043	2.58E-10	0.5245	NA	170756	rs1218789	329443	ieu-b-102	TRUE	reported	WYslv6
5	1.04E+08	C	T	-0.0271	0.0043	2.83E-10	0.524	NA	170756	rs1218790	329443	ieu-b-102	TRUE	reported	WYslv6
5	1.04E+08	T	A	0.0289	0.0044	6.31E-11	0.4288	NA	170756	rs254027	329443	ieu-b-102	TRUE	reported	WYslv6
5	1.04E+08	T	G	-0.0271	0.0043	2.51E-10	0.5232	NA	170756	rs1363101	329443	ieu-b-102	TRUE	reported	WYslv6
5	1.04E+08	T	C	-0.027	0.0043	3.22E-10	0.5241	NA	170756	rs1045506	329443	ieu-b-102	TRUE	reported	WYslv6
5	1.04E+08	T	G	-0.027	0.0043	3.24E-10	0.5241	NA	170756	rs1045506	329443	ieu-b-102	TRUE	reported	WYslv6
5	1.04E+08	C	A	-0.0276	0.0043	1.23E-10	0.5241	NA	170756	rs2018142	329443	ieu-b-102	TRUE	reported	WYslv6
5	1.04E+08	C	G	0.0286	0.0043	3.76E-11	0.4353	NA	170756	rs254025	329443	ieu-b-102	TRUE	reported	WYslv6
5	1.04E+08	G	T	0.0285	0.0043	4.15E-11	0.4353	NA	170756	rs254024	329443	ieu-b-102	TRUE	reported	WYslv6
5	1.04E+08	A	G	0.0281	0.0043	8.01E-11	0.436	NA	170756	rs2431109	329443	ieu-b-102	TRUE	reported	WYslv6
5	1.04E+08	C	T	0.0286	0.0043	3.59E-11	0.4352	NA	170756	rs2161097	329443	ieu-b-102	TRUE	reported	WYslv6
5	1.04E+08	A	T	0.0283	0.0043	5.67E-11	0.4361	NA	170756	rs1363100	329443	ieu-b-102	TRUE	reported	WYslv6
5	1.04E+08	C	T	0.0278	0.0043	1.22E-10	0.437	NA	170756	rs2112163	329443	ieu-b-102	TRUE	reported	WYslv6
5	1.04E+08	C	T	0.0281	0.0043	7.50E-11	0.4362	NA	170756	rs2447828	329443	ieu-b-102	TRUE	reported	WYslv6
5	1.04E+08	T	C	0.0357	0.0046	6.03E-15	0.3274	NA	170756	rs2431108	329443	ieu-b-102	TRUE	reported	WYslv6
5	1.04E+08	C	T	0.0277	0.0043	1.54E-10	0.4354	NA	170756	rs2447827	329443	ieu-b-102	TRUE	reported	WYslv6
5	1.04E+08	C	T	0.0275	0.0043	1.90E-10	0.4362	NA	170756	rs254020	329443	ieu-b-102	TRUE	reported	WYslv6
5	1.04E+08	T	A	0.0361	0.0046	2.93E-15	0.3275	NA	170756	rs173823	329443	ieu-b-102	TRUE	reported	WYslv6
5	1.04E+08	T	C	0.0272	0.0043	3.17E-10	0.4366	NA	170756	rs254023	329443	ieu-b-102	TRUE	reported	WYslv6
5	1.04E+08	C	G	0.036	0.0046	3.53E-15	0.3271	NA	170756	rs171697	329443	ieu-b-102	TRUE	reported	WYslv6
5	1.04E+08	A	G	0.0276	0.0043	1.78E-10	0.4355	NA	170756	rs254045	329443	ieu-b-102	TRUE	reported	WYslv6
5	1.04E+08	G	A	0.0363	0.0046	2.36E-15	0.3269	NA	170756	rs1247771	329443	ieu-b-102	TRUE	reported	WYslv6
5	1.04E+08	A	C	0.028	0.0043	1.13E-10	0.4355	NA	170756	rs185260	329443	ieu-b-102	TRUE	reported	WYslv6
5	1.04E+08	G	A	0.0366	0.0046	1.43E-15	0.3271	NA	170756	rs30266	329443	ieu-b-102	TRUE	reported	WYslv6
5	1.04E+08	G	A	0.0283	0.0043	7.24E-11	0.4342	NA	170756	rs33817	329443	ieu-b-102	TRUE	reported	WYslv6
5	1.04E+08	T	G	0.0366	0.0046	1.40E-15	0.3297	NA	170756	rs40465	329443	ieu-b-102	TRUE	reported	WYslv6
5	1.04E+08	C	A	-0.0312	0.0044	1.03E-12	0.5892	NA	170756	rs416223	329443	ieu-b-102	TRUE	reported	WYslv6
5	1.04E+08	A	G	-0.0317	0.0044	7.14E-13	0.6055	NA	170756	rs325485	329443	ieu-b-102	TRUE	reported	WYslv6
5	1.04E+08	G	A	-0.0315	0.0044	6.69E-13	0.5901	NA	170756	rs325481	329443	ieu-b-102	TRUE	reported	WYslv6
5	1.04E+08	G	C	0.0364	0.0047	6.65E-15	0.3249	NA	170756	rs1561410	329443	ieu-b-102	TRUE	reported	WYslv6
5	1.04E+08	A	G	-0.0315	0.0044	6.65E-13	0.5907	NA	170756	rs7966409	329443	ieu-b-102	TRUE	reported	WYslv6
5	1.04E+08	G	C	-0.0302	0.0044	5.22E-12	0.5877	NA	170756	rs325501	329443	ieu-b-102	TRUE	reported	WYslv6
5	1.04E+08	G	A	-0.0286	0.0044	4.85E-11	0.5719	NA	170756	rs325502	329443	ieu-b-102	TRUE	reported	WYslv6
5	1.04E+08	C	G	-0.03	0.0043	4.71E-12	0.56	NA	170756	rs325506	329443	ieu-b-102	TRUE	reported	WYslv6
5	1.04E+08	G	A	-0.0287	0.0044	4.27E-11	0.5719	NA	170756	rs21126	329443	ieu-b-102	TRUE	reported	WYslv6
5	1.04E+08	C	G	-0.0298	0.0044	7.23E-12	0.5748	NA	170756	rs396755	329443	ieu-b-102	TRUE	reported	WYslv6
5	1.04E+08	C	T	-0.0276	0.0043	1.95E-10	0.5731	NA	170756	rs410915	329443	ieu-b-102	TRUE	reported	WYslv6
5	1.04E+08	G	A	0.0331	0.0046	5.34E-13	0.3268	NA	170756	rs1205523	329443	ieu-b-102	TRUE	reported	WYslv6
5	1.04E+08	C	T	-0.027	0.0043	4.77E-10	0.574	NA	170756	rs325521	329443	ieu-b-102	TRUE	reported	WYslv6
5	1.04E+08	C	A	-0.027	0.0043	5.18E-10	0.5733	NA	170756	rs325523	329443	ieu-b-102	TRUE	reported	WYslv6
5	1.04E+08	C	G	-0.0288	0.0044	3.71E-11	0.5815	NA	170756	rs325528	329443	ieu-b-102	TRUE	reported	WYslv6
5	1.04E+08	A	G	-0.0334	0.0046	3.29E-13	0.6783	NA	170756	rs161645	329443	ieu-b-102	TRUE	reported	WYslv6
5	1.04E+08	T	C	0.0324	0.0057	1.55E-08	0.1735	NA	170756	rs3584192	329443	ieu-b-102	TRUE	reported	WYslv6
5	1.04E+08	T	C	-0.0323	0.0046	2.04E-12	0.6761	NA	170756	rs6421926	329443	ieu-b-102	TRUE	reported	WYslv6
5	1.04E+08	A	C	-0.033	0.0046	6.45E-13	0.6789	NA	170756	rs60271	329443	ieu-b-102	TRUE	reported	WYslv6
5	1.04E+08	A	C	-0.0337	0.0046	2.32E-13	0.6791	NA	170756	rs323509	329443	ieu-b-102	TRUE	reported	WYslv6
5	1.64E+08	G	T	0.0281	0.0045	3.05E-10	0.4914	NA	170756	rs7927884	329443	ieu-b-102	TRUE	reported	WYslv6
5	1.64E+08	G	T	0.0274	0.0044	3.41E-10	0.5151	NA	170756	rs1317100	329443	ieu-b-102	TRUE	reported	WYslv6
5	1.64E+08	C	G	0.0275	0.0043	1.87E-10	0.5229	NA	170756	rs1317286	329443	ieu-b-102	TRUE	reported	WYslv6
5	1.64E+08	A	C	0.0278	0.0043	1.58E-10	0.5209	NA	170756	rs9314050	329443	ieu-b-102	TRUE	reported	WYslv6
5	1.64E+08	T	C	0.0276	0.0044	2.24E-10	0.5174	NA	170756	rs1047648	329443	ieu-b-102	TRUE	reported	WYslv6
5	1.64E+08	C	T	0.0275	0.0043	2.54E-10	0.5217	NA	170756	rs4441893	329443	ieu-b-102	TRUE	reported	WYslv6
5	1.64E+08	A	G	0.0272	0.0043	3.97E-10	0.5191	NA	170756	rs7446573	329443	ieu-b-102	TRUE	reported	WYslv6
5	1.64E+08	A	G	0.0274	0.0043	3.16E-10	0.5211	NA	170756	rs7446574	329443	ieu-b-102	TRUE	reported	WYslv6
5	1.64E+08	G	T	0.0278	0.0043	1.54E-10	0.5223	NA	170756	rs7442891	329443	ieu-b-102	TRUE	reported	WYslv6
5	1.64E+08	T	C	0.0278	0.0043	1.38E-10	0.5235	NA	170756	rs7447460	329443	ieu-b-102	TRUE	reported	WYslv6
5	1.64E+08	A	G	0.0289	0.0043	2.96E-11	0.5204	NA	170756	rs4532381	329443	ieu-b-102	TRUE	reported	WYslv6
5	1.64E+08	T	C	0.0275	0.0043	2.01E-10	0.5244	NA	170756	rs6892804	329443	ieu-b-102	TRUE	reported	WYslv6
5	1.64E+08	C	T	0.0278	0.0043	1.26E-10	0.5236	NA	170756	rs6868116	329443	ieu-b-102	TRUE	reported	WYslv6
5	1.64E+08	C	T	0.0278	0.0043	1.21E-10	0.5231	NA	170756	rs6868125	329443	ieu-b-102	TRUE	reported	WYslv6
5	1.64E+08	A	G	0.0278	0.0043	1.56E-10	0.5208	NA	170756	rs7732352	329443	ieu-b-102	TRUE	reported	WYslv6
5	1.64E+08	C	A	0.0277	0.0043	1.32E-10	0.5236	NA	170756	rs7713037	329443	ieu-b-102	TRUE	reported	WYslv6
5	1.64E+08	A	G	0.0275	0.0043	1.83E-10	0.5232	NA	170756	rs1335773	329443	ieu-b-102	TRUE	reported	WYslv6
5	1.64E+08	T	C	0.0274	0.0043	2.19E-10	0.523	NA	170756	rs1338644	329443	ieu-b-102	TRUE	reported	WYslv6
5	1.64E+08	G	C	0.0278	0.0043	1.11E-10	0.5236	NA	170756	rs7716948	329443	ieu-b-102	TRUE	reported	WYslv6
5	1.64E+08	C	A	0.0276	0.0043	1.68E-10	0.5225	NA	170756	rs7718204	329443	ieu-b-102	TRUE	reported	WYslv6
5	1.64E+08	T	G	0.0278	0.0043	1.13E-10	0.5236	NA	170756	rs7704022	329443	ieu-b-102	TRUE	reported	WYslv6
5	1.64E+08	G	A	0.0278	0.0043	1.12E-10	0.5236	NA	170756	rs7717252	329443	ieu-b-102	TRUE	reported	WYslv6
5	1.64E+08	C	A	0.0277	0.0043	1.28E-10	0.5234	NA	170756	rs1473997	329443	ieu-b-102	TRUE	reported	WYslv6
5	1.64E+08	T	A	0.0279	0.0043	9.87E-11	0.5237	NA	170756	rs6869136	329443	ieu-b-102	TRUE	reported	WYslv6
5	1.64E+08	T	A	0.0277	0.0043	1.25E-10	0.5236	NA	170756	rs6869299	329443	ieu-b-102	TRUE	reported	WYslv6
5	1.64E+08	G	A	0.0276	0.0043	1.38E-10	0.5237	NA	170756	rs6556800	329443	ieu-b-102	TRUE	reported	WYslv6
5	1.64E+08	A	G	0.0275	0.0043	1.81E-10	0.5228	NA	170756	rs6556801	329443	ieu-b-102	TRUE	reported	WYslv6
5	1.64E+08	T	C	0.0278	0.0043	1.07E-10	0.5229	NA	170756	rs7714851	329443	ieu-b-102	TRUE	reported	WYslv6
5	1.64E+08	T	G	0.0278	0.0043	1.14E-10	0.5229	NA	170756	rs6556802	329443	ieu-b-102	TRUE	reported	WYslv6
5	1.64E+08	G	A	0.028	0.0043	8.68E-11	0.5234	NA	170756	rs9791143	329443	ieu-b-102	TRUE	reported	WYslv6
5	1.64E+08	C	A	0.0278	0.0043	1.16E-10	0.5234	NA	170756	rs4563645	329443	ieu-b-102	TRUE	reported	WYslv6
5	1.64E+08	C	T	0.0277	0.0043	1.19E-10	0.5234	NA	170756	rs4566816	329443	ieu-b-102	TRUE	reported	WYslv6
5	1.64E+08	T	C	0.028	0.0043	7.94E-11	0.5233	NA	170756	rs1317319	329443	ieu-b-102	TRUE	reported	WYslv6
5	1.64E+08	C	G	0.0279	0.0043	9.71E-11	0.5232	NA	170756	rs6896348	329443	ieu-b-102	TRUE	reported	WYslv6
5	1.64E+08	A	G	0.028	0.0043	8.05E-11	0.5233	NA	170756	rs3517258	329443	ieu-b-102	TRUE	reported	WYslv6
5	1.64E+08	A	G	0.0279	0.0043	8.87E-11	0.5235	NA	170756	rs9885469	329443	ieu-b-102	TRUE	reported	WYslv6
5	1.64E+08	G	A	0.0276	0.0043	1.48E-10	0.5238	NA	170756	rs7711961	329443	ieu-b-102	TRUE	reported	WYslv6
5	1.64E+08	A	G	0.0275	0.0043	1.64E-10	0.5233	NA	170756	rs4596404	329443	ieu-b-102	TRUE	reported	WYslv6
5	1.64E+08	T	C	0.0275	0.0043	1.74E-10	0.5233	NA	170756	rs4286698	329443	ieu-b-102	TRUE	reported	WYslv6
5	1.64E+08	G	C	0.0275	0.0043	1.81E-10	0.5233	NA	170756	rs4434398	329443	ieu-b-102	TRUE	reported	WYslv6
5	1.64E+08	C	T	0.0275	0.0043	1.82E-10	0.5238	NA	170756	rs4077277	329443	ieu-b-102	TRUE	reported	WYslv6
5	1.64E+08	C	G	0.0275	0.0043	1.79E-10	0.5238	NA	170756	rs4077278	329443	ieu-b-102	TRUE	reported	WYslv6
5	1.64E+08	G	T	0.0273	0.0043	2.25E-10	0.5231	NA	170756	rs4339373	329443	ieu-b-102	TRUE	reported	WYslv6
5	1.64E+08	T	A	0.0274	0.0043	2.17E-10	0.5226	NA	170756	rs7725011	329443	ieu-b-102	TRUE	reported	WYslv6
5	1.64E+08	G	A	0.0275	0.0043	1.55E-10	0.5226	NA	170756	rs4346787	329443	ieu-b-102	TRUE	reported	WYslv6
5	1.64E+08	G	A	0.0284	0.0043	3.84E-11	0.534	NA	170756	rs7705171	329443	ieu-b-102	TRUE	reported	WYslv6
5	1.64E+08	T	G	0.0288	0.0043	2.17E-11	0.5343	NA	170756	rs4543289	329443	ieu-b-102	TRUE	reported	WYslv6
5	1.64E+08	G	A	0.0289	0.0043	1.98E-11	0.5343	NA	170756	rs4552634	329443	ieu-b-102	TRUE	reported	WYslv6
5	1.64E+08	T	C	0.0289	0.0043	1.89E-11	0.5342	NA	170756	rs1003544	329443	ieu-b-102	TRUE	reported	WYslv6
5	1.64E+08	G	A	0.029	0.0043	1.72E-11	0.5343	NA	170756	rs7721129	329443	ieu-b-102	TRUE	reported	WYslv6
5	1.64E+08	G	A	0.029	0.0043	1.61E-11	0.5343	NA	170756	rs7725715	329443	ieu-b-102	TRUE	reported	WYslv6
5	1.64E+08	C	T	0.0289	0.0043	1.74E-11	0.5343	NA	170756	rs7727318	329443	ieu-b-102	TRUE	reported	WYslv6
5	1.64E+08	T	C	0.0289	0.0043	2.11E-11	0.5325	NA	170756	rs1043470	329443	ieu-b-102	TRUE	reported	WYslv6
5	1.65E+08	T	C	0.0245	0.0044	3.80E-08	0.3826	NA	170756	rs4362959	329443	ieu-b-102	TRUE	reported	WYslv6
5	1.65E+08	G	A	0.0286	0.0043	3.01E-11	0.5338	NA	170756	rs9302967	329443	ieu-b-102	TRUE	reported	WYslv6
5	1.65E+08	T	C	0.0286	0.0043	3.14E-11	0.5336	NA	170756	rs6878588	329443	ieu-b-102	TRUE	reported	WYslv6
5	1.65E+08	A	G	0.0285	0.0043	3.75E-11	0.5335	NA	170756	rs6873557	329443	ieu-b-102	TRUE	reported	WYslv6

5	1.65E+08	A	G	0.0286	0.0043	3.19E-11	0.5335	NA	170756	rs1195763	329443	ieu-b-102	TRUE	reported	WYslv6
5	1.65E+08	A	C	0.029	0.0043	1.64E-11	0.5326	NA	170756	rs7607070	329443	ieu-b-102	TRUE	reported	WYslv6
5	1.65E+08	T	C	0.0282	0.0043	6.63E-11	0.5372	NA	170756	rs1004597	329443	ieu-b-102	TRUE	reported	WYslv6
5	1.65E+08	G	A	0.0247	0.0044	2.07E-08	0.6094	NA	170756	rs1003562	329443	ieu-b-102	TRUE	reported	WYslv6
5	1.65E+08	G	A	0.0247	0.0044	1.99E-08	0.6093	NA	170756	rs1047574	329443	ieu-b-102	TRUE	reported	WYslv6
5	1.65E+08	C	T	0.0251	0.0044	1.11E-08	0.6032	NA	170756	rs1363118	329443	ieu-b-102	TRUE	reported	WYslv6
5	1.65E+08	C	T	0.0282	0.0043	6.46E-11	0.5351	NA	170756	rs6556810	329443	ieu-b-102	TRUE	reported	WYslv6
5	1.65E+08	C	T	0.0243	0.0044	3.80E-08	0.3802	NA	170756	rs6556811	329443	ieu-b-102	TRUE	reported	WYslv6
5	1.65E+08	G	A	0.0251	0.0044	1.23E-08	0.604	NA	170756	rs1195205	329443	ieu-b-102	TRUE	reported	WYslv6
5	1.65E+08	G	T	0.0243	0.0044	3.83E-08	0.3813	NA	170756	rs1345077	329443	ieu-b-102	TRUE	reported	WYslv6
5	1.65E+08	T	C	0.028	0.0043	8.85E-11	0.536	NA	170756	rs9314056	329443	ieu-b-102	TRUE	reported	WYslv6
5	1.65E+08	T	C	0.0281	0.0043	8.23E-11	0.536	NA	170756	rs9314057	329443	ieu-b-102	TRUE	reported	WYslv6
5	1.65E+08	G	A	0.028	0.0043	9.89E-11	0.5362	NA	170756	rs1047649	329443	ieu-b-102	TRUE	reported	WYslv6
5	1.65E+08	C	T	0.0269	0.0043	6.28E-10	0.5614	NA	170756	rs1024996	329443	ieu-b-102	TRUE	reported	WYslv6
5	1.65E+08	C	T	0.0251	0.0044	1.55E-08	0.3769	NA	170756	rs1218783	329443	ieu-b-102	TRUE	reported	WYslv6
5	1.65E+08	T	C	0.0267	0.0043	8.37E-10	0.5616	NA	170756	rs2861139	329443	ieu-b-102	TRUE	reported	WYslv6
5	1.65E+08	C	T	0.0265	0.0043	1.01E-09	0.5615	NA	170756	rs3095954	329443	ieu-b-102	TRUE	reported	WYslv6
5	1.65E+08	A	G	0.0266	0.0043	9.40E-10	0.5615	NA	170756	rs3095955	329443	ieu-b-102	TRUE	reported	WYslv6
5	1.65E+08	G	C	0.0262	0.0043	1.61E-09	0.561	NA	170756	rs189843	329443	ieu-b-102	TRUE	reported	WYslv6
5	1.65E+08	A	C	0.0262	0.0043	1.74E-09	0.561	NA	170756	rs253537	329443	ieu-b-102	TRUE	reported	WYslv6
5	1.65E+08	G	A	0.0262	0.0043	1.54E-09	0.561	NA	170756	rs183704	329443	ieu-b-102	TRUE	reported	WYslv6
5	1.65E+08	C	T	0.0268	0.0044	7.73E-10	0.5599	NA	170756	rs253540	329443	ieu-b-102	TRUE	reported	WYslv6
5	1.65E+08	T	G	0.0246	0.0044	3.01E-08	0.3749	NA	170756	rs1195181	329443	ieu-b-102	TRUE	reported	WYslv6
5	1.65E+08	T	G	0.0248	0.0044	2.43E-08	0.3742	NA	170756	rs1318043	329443	ieu-b-102	TRUE	reported	WYslv6
5	1.65E+08	C	T	0.025	0.0044	1.89E-08	0.3737	NA	170756	rs1363117	329443	ieu-b-102	TRUE	reported	WYslv6
5	1.65E+08	G	A	0.0257	0.0043	3.12E-09	0.562	NA	170756	rs3095951	329443	ieu-b-102	TRUE	reported	WYslv6
5	1.65E+08	G	A	0.0253	0.0043	5.52E-09	0.5602	NA	170756	rs2052513	329443	ieu-b-102	TRUE	reported	WYslv6
5	1.65E+08	G	C	0.0252	0.0045	1.56E-08	0.3725	NA	170756	rs1315664	329443	ieu-b-102	TRUE	reported	WYslv6
5	1.65E+08	A	G	0.0253	0.0043	5.78E-09	0.5603	NA	170756	rs2052514	329443	ieu-b-102	TRUE	reported	WYslv6
5	1.65E+08	C	T	0.0252	0.0043	6.77E-09	0.5601	NA	170756	rs2544720	329443	ieu-b-102	TRUE	reported	WYslv6
5	1.65E+08	A	G	0.0253	0.0045	1.41E-08	0.3732	NA	170756	rs6876603	329443	ieu-b-102	TRUE	reported	WYslv6
5	1.65E+08	G	A	0.025	0.0044	1.95E-08	0.3737	NA	170756	rs6895218	329443	ieu-b-102	TRUE	reported	WYslv6
5	1.65E+08	C	A	0.0253	0.0043	5.85E-09	0.5571	NA	170756	rs1440983	329443	ieu-b-102	TRUE	reported	WYslv6
5	1.65E+08	C	T	0.0252	0.0043	6.13E-09	0.5599	NA	170756	rs2431612	329443	ieu-b-102	TRUE	reported	WYslv6
5	1.65E+08	C	T	0.0252	0.0043	6.65E-09	0.5602	NA	170756	rs3095952	329443	ieu-b-102	TRUE	reported	WYslv6
5	1.65E+08	T	G	0.0253	0.0043	5.70E-09	0.5604	NA	170756	rs6897738	329443	ieu-b-102	TRUE	reported	WYslv6
5	1.65E+08	A	G	0.0251	0.0044	1.63E-08	0.3744	NA	170756	rs1318615	329443	ieu-b-102	TRUE	reported	WYslv6
5	1.65E+08	T	C	0.0252	0.0043	6.13E-09	0.5603	NA	170756	rs2431131	329443	ieu-b-102	TRUE	reported	WYslv6
5	1.65E+08	G	A	0.0252	0.0043	6.57E-09	0.5603	NA	170756	rs1317514	329443	ieu-b-102	TRUE	reported	WYslv6
5	1.65E+08	G	A	0.0253	0.0043	5.64E-09	0.5587	NA	170756	rs253544	329443	ieu-b-102	TRUE	reported	WYslv6
5	1.65E+08	T	C	0.0255	0.0044	9.86E-09	0.3741	NA	170756	rs1113535	329443	ieu-b-102	TRUE	reported	WYslv6
5	1.65E+08	A	G	0.0255	0.0044	9.84E-09	0.3741	NA	170756	rs1113535	329443	ieu-b-102	TRUE	reported	WYslv6
5	1.65E+08	C	G	0.0255	0.0043	4.11E-09	0.56	NA	170756	rs1113535	329443	ieu-b-102	TRUE	reported	WYslv6
5	1.65E+08	A	G	0.0255	0.0043	4.25E-09	0.5602	NA	170756	rs1469062	329443	ieu-b-102	TRUE	reported	WYslv6
5	1.65E+08	C	G	-0.0269	0.0044	1.27E-09	0.38	NA	170756	rs1739188	329443	ieu-b-102	TRUE	reported	WYslv6
5	1.65E+08	A	G	-0.0255	0.0044	8.56E-09	0.3786	NA	170756	rs7731911	329443	ieu-b-102	TRUE	reported	WYslv6
5	1.65E+08	G	A	-0.0256	0.0044	7.77E-09	0.3785	NA	170756	rs7715290	329443	ieu-b-102	TRUE	reported	WYslv6
5	1.65E+08	A	G	-0.0255	0.0044	8.60E-09	0.3782	NA	170756	rs4291037	329443	ieu-b-102	TRUE	reported	WYslv6
5	1.65E+08	T	C	0.0244	0.0044	3.69E-08	0.3765	NA	170756	rs1113536	329443	ieu-b-102	TRUE	reported	WYslv6
5	1.65E+08	G	C	-0.0255	0.0044	8.52E-09	0.3787	NA	170756	rs7720784	329443	ieu-b-102	TRUE	reported	WYslv6
5	1.65E+08	C	T	0.0243	0.0044	4.52E-08	0.3767	NA	170756	rs6870178	329443	ieu-b-102	TRUE	reported	WYslv6
5	1.65E+08	C	T	0.0242	0.0044	4.66E-08	0.3767	NA	170756	rs6874498	329443	ieu-b-102	TRUE	reported	WYslv6
5	1.65E+08	T	C	-0.0261	0.0044	3.80E-09	0.3779	NA	170756	rs1174472	329443	ieu-b-102	TRUE	reported	WYslv6
5	1.65E+08	T	C	-0.0259	0.0044	4.61E-09	0.3784	NA	170756	rs1006767	329443	ieu-b-102	TRUE	reported	WYslv6
5	1.65E+08	T	C	-0.0259	0.0044	4.75E-09	0.3784	NA	170756	rs1006794	329443	ieu-b-102	TRUE	reported	WYslv6
5	1.65E+08	G	C	-0.0259	0.0044	4.71E-09	0.3784	NA	170756	rs3494575	329443	ieu-b-102	TRUE	reported	WYslv6
5	1.65E+08	G	A	-0.026	0.0044	4.44E-09	0.3784	NA	170756	rs3514512	329443	ieu-b-102	TRUE	reported	WYslv6
5	1.65E+08	T	C	-0.0261	0.0044	3.51E-09	0.3781	NA	170756	rs3591345	329443	ieu-b-102	TRUE	reported	WYslv6
5	1.65E+08	A	G	0.0243	0.0044	4.27E-08	0.3774	NA	170756	rs885477	329443	ieu-b-102	TRUE	reported	WYslv6
5	1.65E+08	A	T	0.0242	0.0044	4.59E-08	0.3775	NA	170756	rs2013312	329443	ieu-b-102	TRUE	reported	WYslv6
5	1.65E+08	G	A	-0.026	0.0044	4.58E-09	0.3782	NA	170756	rs3520334	329443	ieu-b-102	TRUE	reported	WYslv6
5	1.65E+08	T	C	-0.026	0.0044	4.43E-09	0.3782	NA	170756	rs4868918	329443	ieu-b-102	TRUE	reported	WYslv6
5	1.65E+08	A	G	-0.026	0.0044	4.57E-09	0.378	NA	170756	rs4868919	329443	ieu-b-102	TRUE	reported	WYslv6
5	1.65E+08	G	A	-0.026	0.0044	4.33E-09	0.3782	NA	170756	rs4868920	329443	ieu-b-102	TRUE	reported	WYslv6
5	1.65E+08	A	T	-0.0261	0.0045	4.38E-09	0.3774	NA	170756	rs1288044	329443	ieu-b-102	TRUE	reported	WYslv6
5	1.65E+08	A	G	-0.0257	0.0044	6.07E-09	0.3785	NA	170756	rs1174844	329443	ieu-b-102	TRUE	reported	WYslv6
5	1.65E+08	A	G	-0.0258	0.0044	5.88E-09	0.3785	NA	170756	rs6238568	329443	ieu-b-102	TRUE	reported	WYslv6
5	1.65E+08	A	T	-0.0258	0.0044	5.93E-09	0.3785	NA	170756	rs1007678	329443	ieu-b-102	TRUE	reported	WYslv6
5	1.65E+08	A	C	-0.0258	0.0044	5.76E-09	0.3781	NA	170756	rs1007682	329443	ieu-b-102	TRUE	reported	WYslv6
5	1.65E+08	T	C	-0.0258	0.0044	5.40E-09	0.3786	NA	170756	rs4868753	329443	ieu-b-102	TRUE	reported	WYslv6
5	1.65E+08	G	A	-0.0259	0.0044	4.73E-09	0.3786	NA	170756	rs1318605	329443	ieu-b-102	TRUE	reported	WYslv6
5	1.65E+08	A	G	-0.0259	0.0044	5.06E-09	0.3786	NA	170756	rs1174743	329443	ieu-b-102	TRUE	reported	WYslv6
5	1.65E+08	A	G	-0.0258	0.0044	5.47E-09	0.3786	NA	170756	rs1174750	329443	ieu-b-102	TRUE	reported	WYslv6
5	1.65E+08	T	A	-0.0258	0.0044	5.47E-09	0.3787	NA	170756	rs1174899	329443	ieu-b-102	TRUE	reported	WYslv6
5	1.65E+08	A	G	-0.026	0.0044	4.15E-09	0.3786	NA	170756	rs1174918	329443	ieu-b-102	TRUE	reported	WYslv6
5	1.65E+08	A	T	-0.0258	0.0044	5.66E-09	0.3785	NA	170756	rs1733344	329443	ieu-b-102	TRUE	reported	WYslv6
5	1.65E+08	T	C	-0.0243	0.0044	4.03E-08	0.3813	NA	170756	rs4130574	329443	ieu-b-102	TRUE	reported	WYslv6
5	1.65E+08	T	C	-0.0243	0.0044	4.23E-08	0.3814	NA	170756	rs4367315	329443	ieu-b-102	TRUE	reported	WYslv6
5	1.65E+08	T	C	-0.0244	0.0044	3.62E-08	0.3829	NA	170756	rs1422679	329443	ieu-b-102	TRUE	reported	WYslv6
5	1.65E+08	T	C	-0.0243	0.0044	4.18E-08	0.3823	NA	170756	rs1422680	329443	ieu-b-102	TRUE	reported	WYslv6
5	1.65E+08	C	T	-0.0244	0.0044	3.76E-08	0.382	NA	170756	rs3566530	329443	ieu-b-102	TRUE	reported	WYslv6
5	1.65E+08	G	A	-0.0245	0.0044	3.34E-08	0.3819	NA	170756	rs1290432	329443	ieu-b-102	TRUE	reported	WYslv6
6	25710571	G	A	-0.0428	0.0076	2.13E-08	0.0887	NA	170756	rs6800663	329443	ieu-b-102	TRUE	reported	WYslv6
6	25714959	A	G	-0.0425	0.0076	2.24E-08	0.0892	NA	170756	rs3449301	329443	ieu-b-102	TRUE	reported	WYslv6
6	25786226	T	C	-0.0416	0.0076	4.18E-08	0.0892	NA	170756	rs1321395	329443	ieu-b-102	TRUE	reported	WYslv6
6	25822661	C	T	-0.0413	0.0076	4.72E-08	0.0896	NA	170756	rs1320134	329443	ieu-b-102	TRUE	reported	WYslv6
6	25864818	C	T	-0.0416	0.0076	4.20E-08	0.089	NA	170756	rs1321194	329443	ieu-b-102	TRUE	reported	WYslv6
6	25866935	T	C	-0.0413	0.0076	4.66E-08	0.0893	NA	170756	rs5591263	329443	ieu-b-102	TRUE	reported	WYslv6
6	25874423	G	A	-0.0424	0.0076	2.93E-08	0.0872	NA	170756	rs1319847	329443	ieu-b-102	TRUE	reported	WYslv6
6	25884519	G	A	-0.0415	0.0076	3.84E-08	0.0896	NA	170756	rs3601412	329443	ieu-b-102	TRUE	reported	WYslv6
6	25918855	G	A	-0.0412	0.0075	4.98E-08	0.0896	NA	170756	rs1752672	329443	ieu-b-102	TRUE	reported	WYslv6
6	26021872	C	T	-0.0419	0.0076	2.83E-08	0.0896	NA	170756	rs4126677	329443	ieu-b-102	TRUE	reported	WYslv6
6	26037601	G	A	-0.0418	0.0075	2.91E-08	0.0897	NA	170756	rs1321004	329443	ieu-b-102	TRUE	reported	WYslv6
6	26044864	T	C	-0.0416	0.0075	3.36E-08	0.0898	NA	170756	rs3550651	329443	ieu-b-102	TRUE	reported	WYslv6
6	26067360	A	G	-0.0418	0.0075	2.79E-08	0.0899	NA	170756	rs3505060	329443	ieu-b-102	TRUE	reported	WYslv6
6	26073375	C	A	-0.0419	0.0075	2.88E-08	0.0897	NA	170756	rs3488848	329443	ieu-b-102	TRUE	reported	WYslv6
6	26083519	T	C	-0.0419	0.0075	2.70E-08	0.0898	NA	170756	rs3419761	329443	ieu-b-102	TRUE	reported	WYslv6
6	26133070	G	A	-0.0435	0.0075	8.14E-09	0.0902	NA	170756	rs3610988	329443	ieu-b-102	TRUE	reported	WYslv6
6	26175866	A	C	-0.0466	0.0074	2.87E-10	0.0944	NA	170756	rs1759865	329443	ieu-b-102	TRUE	reported	WYslv6
6	26189356	G	C	-0.0478	0.0074	1.01E-10	0.0944	NA	170756	rs1320457	329443	ieu-b-102	TRUE	reported	WYslv6
6	26199903	C	T	-0.048	0.0074	9.57E-11	0.0941	NA	170756	rs3496155	329443	ieu-b-102	TRUE	reported	WYslv6
6	26266311	A	C	-0.0487	0.0073	3.23E-11	0.0956	NA	170756	rs5570601	329443	ieu-b-102	TRUE	reported	WYslv6
6	26309908	G	A	-0.0475	0.0073	5.67E-11	0.098	NA	170756	rs1048443	329443	ieu-b-102	TRUE	reported	WYslv6
6	26312170	G	C	-0.0428	0.0068	2.86E-10	0.1133	NA	170756	rs9467701	329443	ieu-b-102	TRUE	reported	WYslv6
6	26313348	C	T	-0.0428	0.0068	3.05E-10	0.1133	NA	170756	rs6923139	329443	ieu-b-102	TRUE	reported	WYslv6
6	26314233	G	C	-0.0445	0.0069	1.02E-10	0.1105	NA	170756	rs1883215	329443	ieu-b-102	TRUE	reported	WYslv6
6	26316295	G	A	-0.0487	0.0073	3.15E-11	0.0955	NA	170756	rs1322052	329443	ieu-b-102	TRUE	reported	WYslv6

6	26316982	A	G	-0.0354	0.0062	1.28E-08	0.1397	NA	170756	rs1196636	329443	ieu-b-102	TRUE	reported	WYslv6
6	26318262	C	T	-0.0475	0.0072	5.57E-11	0.098	NA	170756	rs3516229	329443	ieu-b-102	TRUE	reported	WYslv6
6	26319486	C	T	-0.0429	0.0068	3.23E-10	0.1121	NA	170756	rs9467704	329443	ieu-b-102	TRUE	reported	WYslv6
6	26322115	C	T	-0.0428	0.0068	2.98E-10	0.1133	NA	170756	rs6902392	329443	ieu-b-102	TRUE	reported	WYslv6
6	26322153	A	G	-0.0428	0.0068	2.98E-10	0.1133	NA	170756	rs6902389	329443	ieu-b-102	TRUE	reported	WYslv6
6	26322526	A	G	-0.0427	0.0068	3.08E-10	0.1134	NA	170756	rs6903015	329443	ieu-b-102	TRUE	reported	WYslv6
6	26322861	C	T	-0.0426	0.0068	3.50E-10	0.1134	NA	170756	rs6908156	329443	ieu-b-102	TRUE	reported	WYslv6
6	26327905	G	C	-0.0429	0.0068	2.82E-10	0.1134	NA	170756	rs9467707	329443	ieu-b-102	TRUE	reported	WYslv6
6	26327956	T	C	-0.0428	0.0068	3.06E-10	0.1134	NA	170756	rs1450170	329443	ieu-b-102	TRUE	reported	WYslv6
6	26328353	T	C	-0.0486	0.0073	3.61E-11	0.0957	NA	170756	rs3410745	329443	ieu-b-102	TRUE	reported	WYslv6
6	26328366	G	A	-0.0437	0.0069	3.09E-10	0.1118	NA	170756	rs7154467	329443	ieu-b-102	TRUE	reported	WYslv6
6	26328462	G	C	-0.0439	0.0068	1.43E-10	0.1135	NA	170756	rs6939978	329443	ieu-b-102	TRUE	reported	WYslv6
6	26332605	A	C	-0.0433	0.0068	1.80E-10	0.1166	NA	170756	rs9467711	329443	ieu-b-102	TRUE	reported	WYslv6
6	26334306	A	G	-0.0439	0.0068	1.39E-10	0.1136	NA	170756	rs3456976	329443	ieu-b-102	TRUE	reported	WYslv6
6	26336572	G	A	-0.0486	0.0073	2.95E-11	0.0974	NA	170756	rs3415876	329443	ieu-b-102	TRUE	reported	WYslv6
6	26338128	T	C	-0.0418	0.0068	7.78E-10	0.1149	NA	170756	rs9467713	329443	ieu-b-102	TRUE	reported	WYslv6
6	26339131	A	G	-0.0479	0.0073	5.38E-11	0.0984	NA	170756	rs3594256	329443	ieu-b-102	TRUE	reported	WYslv6
6	26340785	G	A	-0.0377	0.0065	5.20E-09	0.1293	NA	170756	rs9467714	329443	ieu-b-102	TRUE	reported	WYslv6
6	26343057	C	G	-0.0323	0.0058	2.56E-08	0.1687	NA	170756	rs9379850	329443	ieu-b-102	TRUE	reported	WYslv6
6	26354100	G	T	-0.0461	0.0065	1.72E-12	0.1246	NA	170756	rs6750921	329443	ieu-b-102	TRUE	reported	WYslv6
6	26354219	C	G	-0.0275	0.0048	9.48E-09	0.2875	NA	170756	rs9366652	329443	ieu-b-102	TRUE	reported	WYslv6
6	26354247	G	A	-0.046	0.0065	1.81E-12	0.1246	NA	170756	rs9366653	329443	ieu-b-102	TRUE	reported	WYslv6
6	26354780	A	C	-0.0455	0.0065	2.84E-12	0.1253	NA	170756	rs9379851	329443	ieu-b-102	TRUE	reported	WYslv6
6	26355094	G	A	-0.046	0.0065	1.80E-12	0.1246	NA	170756	rs3593464	329443	ieu-b-102	TRUE	reported	WYslv6
6	26355283	A	C	-0.027	0.0048	1.28E-08	0.287	NA	170756	rs6933583	329443	ieu-b-102	TRUE	reported	WYslv6
6	26355758	T	G	-0.0271	0.0048	1.25E-08	0.2872	NA	170756	rs4712980	329443	ieu-b-102	TRUE	reported	WYslv6
6	26356347	C	A	-0.046	0.0065	1.81E-12	0.1246	NA	170756	rs3530497	329443	ieu-b-102	TRUE	reported	WYslv6
6	26356822	A	G	-0.0269	0.0048	1.46E-08	0.287	NA	170756	rs9467725	329443	ieu-b-102	TRUE	reported	WYslv6
6	26356853	C	T	-0.046	0.0065	1.84E-12	0.1249	NA	170756	rs7155733	329443	ieu-b-102	TRUE	reported	WYslv6
6	26356951	T	C	-0.046	0.0065	1.84E-12	0.1246	NA	170756	rs9393703	329443	ieu-b-102	TRUE	reported	WYslv6
6	26357011	C	T	-0.046	0.0065	1.79E-12	0.1246	NA	170756	rs9358930	329443	ieu-b-102	TRUE	reported	WYslv6
6	26357278	A	G	-0.046	0.0065	1.85E-12	0.1246	NA	170756	rs9379852	329443	ieu-b-102	TRUE	reported	WYslv6
6	26357635	A	G	-0.046	0.0065	1.85E-12	0.1246	NA	170756	rs9379853	329443	ieu-b-102	TRUE	reported	WYslv6
6	26358775	T	C	-0.046	0.0065	1.90E-12	0.1246	NA	170756	rs9393704	329443	ieu-b-102	TRUE	reported	WYslv6
6	26359241	A	T	-0.0459	0.0065	2.10E-12	0.1245	NA	170756	rs7283648	329443	ieu-b-102	TRUE	reported	WYslv6
6	26360311	G	A	-0.0459	0.0065	2.15E-12	0.1245	NA	170756	rs9348709	329443	ieu-b-102	TRUE	reported	WYslv6
6	26360597	A	G	-0.031	0.005	5.77E-10	0.2462	NA	170756	rs9348711	329443	ieu-b-102	TRUE	reported	WYslv6
6	26361011	G	A	-0.0455	0.0065	3.48E-12	0.1244	NA	170756	rs9393705	329443	ieu-b-102	TRUE	reported	WYslv6
6	26361430	C	T	-0.0273	0.0048	9.56E-09	0.2857	NA	170756	rs4712981	329443	ieu-b-102	TRUE	reported	WYslv6
6	26361500	G	A	-0.0459	0.0065	2.12E-12	0.1245	NA	170756	rs9393706	329443	ieu-b-102	TRUE	reported	WYslv6
6	26361946	C	G	-0.0272	0.0048	1.06E-08	0.2857	NA	170756	rs1094681	329443	ieu-b-102	TRUE	reported	WYslv6
6	26361985	A	G	-0.0459	0.0065	2.07E-12	0.1245	NA	170756	rs3455093	329443	ieu-b-102	TRUE	reported	WYslv6
6	26362119	A	T	-0.0459	0.0065	2.12E-12	0.1245	NA	170756	rs3462202	329443	ieu-b-102	TRUE	reported	WYslv6
6	26362486	T	C	-0.0459	0.0065	2.17E-12	0.1245	NA	170756	rs9393707	329443	ieu-b-102	TRUE	reported	WYslv6
6	26362643	T	C	-0.0459	0.0065	1.96E-12	0.1245	NA	170756	rs9393708	329443	ieu-b-102	TRUE	reported	WYslv6
6	26362705	C	T	-0.0458	0.0065	2.22E-12	0.1245	NA	170756	rs9358932	329443	ieu-b-102	TRUE	reported	WYslv6
6	26362854	T	C	-0.0459	0.0065	2.13E-12	0.1245	NA	170756	rs9379854	329443	ieu-b-102	TRUE	reported	WYslv6
6	26363688	C	T	-0.0272	0.0048	1.07E-08	0.2857	NA	170756	rs9358933	329443	ieu-b-102	TRUE	reported	WYslv6
6	26363755	G	C	-0.046	0.0065	1.93E-12	0.1245	NA	170756	rs9358934	329443	ieu-b-102	TRUE	reported	WYslv6
6	26364628	A	C	-0.0458	0.0065	2.21E-12	0.1245	NA	170756	rs9357006	329443	ieu-b-102	TRUE	reported	WYslv6
6	26364930	T	C	-0.046	0.0065	1.82E-12	0.1247	NA	170756	rs9379855	329443	ieu-b-102	TRUE	reported	WYslv6
6	26365586	T	C	-0.0457	0.0065	2.65E-12	0.1245	NA	170756	rs2836260	329443	ieu-b-102	TRUE	reported	WYslv6
6	26365679	T	G	-0.0472	0.0064	1.38E-13	0.1316	NA	170756	rs5882558	329443	ieu-b-102	TRUE	reported	WYslv6
6	26365759	T	C	-0.046	0.0067	6.71E-12	0.1219	NA	170756	rs1561795	329443	ieu-b-102	TRUE	reported	WYslv6
6	26365913	G	T	-0.0455	0.0065	3.18E-12	0.1245	NA	170756	rs7155733	329443	ieu-b-102	TRUE	reported	WYslv6
6	26366511	T	A	-0.0457	0.0065	2.56E-12	0.1245	NA	170756	rs9348712	329443	ieu-b-102	TRUE	reported	WYslv6
6	26366836	A	C	-0.0457	0.0065	2.44E-12	0.1245	NA	170756	rs9379856	329443	ieu-b-102	TRUE	reported	WYslv6
6	26367654	G	C	-0.0457	0.0065	2.50E-12	0.1245	NA	170756	rs9379857	329443	ieu-b-102	TRUE	reported	WYslv6
6	26367689	T	C	-0.0456	0.0065	2.78E-12	0.1245	NA	170756	rs9379858	329443	ieu-b-102	TRUE	reported	WYslv6
6	26367833	A	G	-0.0458	0.0065	2.33E-12	0.1245	NA	170756	rs9393710	329443	ieu-b-102	TRUE	reported	WYslv6
6	26369549	C	T	-0.0458	0.0065	2.26E-12	0.1245	NA	170756	rs9379859	329443	ieu-b-102	TRUE	reported	WYslv6
6	26369587	A	G	-0.0459	0.0065	2.18E-12	0.1245	NA	170756	rs9358935	329443	ieu-b-102	TRUE	reported	WYslv6
6	26370365	G	C	-0.0458	0.0065	2.18E-12	0.1245	NA	170756	rs3443653	329443	ieu-b-102	TRUE	reported	WYslv6
6	26370474	C	T	-0.0458	0.0065	2.18E-12	0.1245	NA	170756	rs3530732	329443	ieu-b-102	TRUE	reported	WYslv6
6	26370572	C	T	-0.0459	0.0065	2.07E-12	0.1245	NA	170756	rs3487849	329443	ieu-b-102	TRUE	reported	WYslv6
6	26370657	A	G	-0.0459	0.0065	2.13E-12	0.1245	NA	170756	rs9358936	329443	ieu-b-102	TRUE	reported	WYslv6
6	26370659	C	T	-0.0457	0.0065	2.74E-12	0.1248	NA	170756	rs7154429	329443	ieu-b-102	TRUE	reported	WYslv6
6	26370707	T	C	-0.0303	0.005	1.02E-09	0.2491	NA	170756	rs9379862	329443	ieu-b-102	TRUE	reported	WYslv6
6	26371000	G	C	-0.0459	0.0065	2.13E-12	0.1245	NA	170756	rs9393712	329443	ieu-b-102	TRUE	reported	WYslv6
6	26371679	A	G	-0.046	0.0065	1.95E-12	0.1245	NA	170756	rs1217385	329443	ieu-b-102	TRUE	reported	WYslv6
6	26372427	A	G	-0.0462	0.0065	1.77E-12	0.1237	NA	170756	rs9379863	329443	ieu-b-102	TRUE	reported	WYslv6
6	26372584	G	A	-0.0462	0.0065	1.77E-12	0.1237	NA	170756	rs9379864	329443	ieu-b-102	TRUE	reported	WYslv6
6	26372786	A	G	-0.0462	0.0065	1.64E-12	0.1237	NA	170756	rs1217631	329443	ieu-b-102	TRUE	reported	WYslv6
6	26372827	C	T	-0.0462	0.0065	1.77E-12	0.1237	NA	170756	rs1217460	329443	ieu-b-102	TRUE	reported	WYslv6
6	26373086	C	T	-0.0461	0.0065	1.79E-12	0.1238	NA	170756	rs1217462	329443	ieu-b-102	TRUE	reported	WYslv6
6	26373121	G	A	-0.0464	0.0065	1.40E-12	0.1236	NA	170756	rs1217463	329443	ieu-b-102	TRUE	reported	WYslv6
6	26373150	C	T	-0.0461	0.0065	1.81E-12	0.1237	NA	170756	rs1217463	329443	ieu-b-102	TRUE	reported	WYslv6
6	26373507	G	A	-0.0464	0.0065	1.40E-12	0.1237	NA	170756	rs1321682	329443	ieu-b-102	TRUE	reported	WYslv6
6	26373678	G	A	-0.046	0.0065	1.99E-12	0.1238	NA	170756	rs9393713	329443	ieu-b-102	TRUE	reported	WYslv6
6	26373740	G	T	-0.0458	0.0065	2.72E-12	0.1237	NA	170756	rs9393714	329443	ieu-b-102	TRUE	reported	WYslv6
6	26374274	C	G	-0.0462	0.0065	1.61E-12	0.1237	NA	170756	rs9358937	329443	ieu-b-102	TRUE	reported	WYslv6
6	26374903	A	G	-0.0269	0.0048	1.54E-08	0.2855	NA	170756	rs733528	329443	ieu-b-102	TRUE	reported	WYslv6
6	26374978	T	C	-0.0462	0.0065	1.65E-12	0.1237	NA	170756	rs2073527	329443	ieu-b-102	TRUE	reported	WYslv6
6	26375143	G	C	-0.0273	0.0048	1.20E-08	0.2863	NA	170756	rs2073528	329443	ieu-b-102	TRUE	reported	WYslv6
6	26375159	A	G	-0.0463	0.0065	1.53E-12	0.1237	NA	170756	rs2073529	329443	ieu-b-102	TRUE	reported	WYslv6
6	26375243	T	C	-0.0464	0.0066	1.52E-12	0.1236	NA	170756	rs2073530	329443	ieu-b-102	TRUE	reported	WYslv6
6	26375256	A	G	-0.0464	0.0066	1.45E-12	0.1234	NA	170756	rs2073531	329443	ieu-b-102	TRUE	reported	WYslv6
6	26375642	C	G	-0.0268	0.0048	2.08E-08	0.283	NA	170756	rs3770427	329443	ieu-b-102	TRUE	reported	WYslv6
6	26375645	G	A	-0.0462	0.0066	3.18E-12	0.1218	NA	170756	rs1357755	329443	ieu-b-102	TRUE	reported	WYslv6
6	26375658	G	A	-0.0465	0.0066	2.31E-12	0.1219	NA	170756	rs9348716	329443	ieu-b-102	TRUE	reported	WYslv6
6	26375854	C	G	-0.0461	0.0065	1.60E-12	0.1248	NA	170756	rs9379871	329443	ieu-b-102	TRUE	reported	WYslv6
6	26376103	A	G	-0.0462	0.0065	1.67E-12	0.1237	NA	170756	rs3757138	329443	ieu-b-102	TRUE	reported	WYslv6
6	26376161	A	G	-0.0292	0.0047	5.97E-10	0.2968	NA	170756	rs1985732	329443	ieu-b-102	TRUE	reported	WYslv6
6	26376368	A	G	-0.0459	0.0066	2.95E-12	0.123	NA	170756	rs2855115	329443	ieu-b-102	TRUE	reported	WYslv6
6	26376832	T	C	-0.027	0.0047	1.05E-08	0.2967	NA	170756	rs1321859	329443	ieu-b-102	TRUE	reported	WYslv6
6	26377028	G	A	-0.0464	0.0066	1.54E-12	0.1235	NA	170756	rs9366654	329443	ieu-b-102	TRUE	reported	WYslv6
6	26377385	C	G	-0.0461	0.0065	1.77E-12	0.1238	NA	170756	rs8798816	329443	ieu-b-102	TRUE	reported	WYslv6
6	26377546	A	G	-0.045	0.0065	4.80E-12	0.126	NA	170756	rs1977	329443	ieu-b-102	TRUE	reported	WYslv6
6	26377573	T	A	-0.046	0.0065	1.97E-12	0.1238	NA	170756	rs1978	329443	ieu-b-102	TRUE	reported	WYslv6
6	26377591	G	T	-0.0457	0.0066	2.93E-12	0.1238	NA	170756	rs1979	329443	ieu-b-102	TRUE	reported	WYslv6
6	26377927	T	C	-0.0461	0.0066	2.42E-12	0.1233	NA	170756	rs6807221	329443	ieu-b-102	TRUE	reported	WYslv6
6	26377939	G	A	-0.046	0.0066	2.71E-12	0.1233	NA	170756	rs6682310	329443	ieu-b-102	TRUE	reported	WYslv6
6	26378288	T	A	-0.0462	0.0066	1.94E-12	0.1238	NA	170756	rs7284153	329443	ieu-b-102	TRUE	reported	WYslv6
6	26385093	C	G	-0.0425	0.0065	6.71E-11	0.1254	NA	170756	rs2072806	329443	ieu-b-102	TRUE	reported	WYslv6
6	26387546	C	T	-0.0425	0.0065	6.50E-11	0.1254	NA	170756	rs9358938	329443	ieu-b-102	TRUE	reported	WYslv6
6	26392515	G	C	-0.0427	0.0065	5.08E-11	0.1256	NA	170756	rs2072803	329443	ieu-b-102	TRUE	reported	WYslv6
6	26402717	T	C	-0.0432	0.0066	4.48E-11	0.1231	NA	170756	rs9379873	329443	ieu-b-102	TRUE	reported	WYslv6

6	26404374	A	G	-0.0297	0.0051	7.59E-09	0.2286	NA	170756	rs3799378	329443	ieu-b-102	TRUE	reported	WYslv6
6	26407482	G	A	-0.0432	0.0066	4.45E-11	0.123	NA	170756	rs9393718	329443	ieu-b-102	TRUE	reported	WYslv6
6	26408551	T	G	-0.0532	0.0072	1.62E-13	0.0993	NA	170756	rs7578236	329443	ieu-b-102	TRUE	reported	WYslv6
6	26409890	G	C	-0.0528	0.0072	2.28E-13	0.0993	NA	170756	rs4126683	329443	ieu-b-102	TRUE	reported	WYslv6
6	26415409	T	A	-0.0428	0.0066	6.91E-11	0.1233	NA	170756	rs3427332	329443	ieu-b-102	TRUE	reported	WYslv6
6	26416787	C	G	-0.0428	0.0066	6.84E-11	0.1234	NA	170756	rs9358939	329443	ieu-b-102	TRUE	reported	WYslv6
6	26421345	T	C	-0.0429	0.0066	6.11E-11	0.1233	NA	170756	rs2393667	329443	ieu-b-102	TRUE	reported	WYslv6
6	26422627	C	A	-0.0427	0.0066	1.25E-10	0.1233	NA	170756	rs3734537	329443	ieu-b-102	TRUE	reported	WYslv6
6	26426856	G	A	-0.0429	0.0066	5.91E-11	0.1234	NA	170756	rs2076030	329443	ieu-b-102	TRUE	reported	WYslv6
6	26431982	C	T	-0.0426	0.0066	7.63E-11	0.1234	NA	170756	rs2235251	329443	ieu-b-102	TRUE	reported	WYslv6
6	26434630	T	C	-0.0428	0.0066	6.83E-11	0.1234	NA	170756	rs9366656	329443	ieu-b-102	TRUE	reported	WYslv6
6	26441640	C	T	-0.0531	0.0072	1.96E-13	0.0993	NA	170756	rs1322049	329443	ieu-b-102	TRUE	reported	WYslv6
6	26444732	C	T	-0.0429	0.0066	5.73E-11	0.1234	NA	170756	rs9379875	329443	ieu-b-102	TRUE	reported	WYslv6
6	26444938	T	A	-0.0429	0.0066	6.03E-11	0.1234	NA	170756	rs3594248	329443	ieu-b-102	TRUE	reported	WYslv6
6	26454363	C	T	-0.0482	0.0069	2.17E-12	0.1111	NA	170756	rs3460599	329443	ieu-b-102	TRUE	reported	WYslv6
6	26454956	C	T	-0.0484	0.0068	1.59E-12	0.1116	NA	170756	rs6675720	329443	ieu-b-102	TRUE	reported	WYslv6
6	26455814	T	C	-0.0478	0.0069	3.23E-12	0.111	NA	170756	rs3568081	329443	ieu-b-102	TRUE	reported	WYslv6
6	26456074	T	G	-0.0478	0.0069	3.22E-12	0.1109	NA	170756	rs3603362	329443	ieu-b-102	TRUE	reported	WYslv6
6	26462104	C	T	-0.048	0.0069	2.58E-12	0.111	NA	170756	rs3562749	329443	ieu-b-102	TRUE	reported	WYslv6
6	26463271	T	A	-0.048	0.0069	2.65E-12	0.111	NA	170756	rs3823158	329443	ieu-b-102	TRUE	reported	WYslv6
6	26463660	G	A	-0.0476	0.0069	3.65E-12	0.1111	NA	170756	rs1319550	329443	ieu-b-102	TRUE	reported	WYslv6
6	26466035	A	C	-0.0257	0.0047	3.57E-08	0.3067	NA	170756	rs2273558	329443	ieu-b-102	TRUE	reported	WYslv6
6	26467182	T	C	-0.0298	0.0054	4.60E-08	0.1939	NA	170756	rs3799380	329443	ieu-b-102	TRUE	reported	WYslv6
6	26468326	G	A	-0.0478	0.0069	3.20E-12	0.1112	NA	170756	rs3734542	329443	ieu-b-102	TRUE	reported	WYslv6
6	26468660	G	A	-0.0311	0.0056	2.18E-08	0.1838	NA	170756	rs3734544	329443	ieu-b-102	TRUE	reported	WYslv6
6	26469866	G	A	-0.0309	0.0056	2.68E-08	0.1837	NA	170756	rs9366658	329443	ieu-b-102	TRUE	reported	WYslv6
6	26469875	A	C	-0.031	0.0056	2.53E-08	0.1838	NA	170756	rs9358944	329443	ieu-b-102	TRUE	reported	WYslv6
6	26470032	T	C	-0.0311	0.0056	2.11E-08	0.1838	NA	170756	rs9348719	329443	ieu-b-102	TRUE	reported	WYslv6
6	26471155	T	C	-0.0297	0.0054	4.98E-08	0.1939	NA	170756	rs6456727	329443	ieu-b-102	TRUE	reported	WYslv6
6	26472114	A	G	-0.0313	0.0056	1.72E-08	0.1838	NA	170756	rs9358945	329443	ieu-b-102	TRUE	reported	WYslv6
6	26474044	C	T	-0.031	0.0056	2.36E-08	0.1838	NA	170756	rs7773938	329443	ieu-b-102	TRUE	reported	WYslv6
6	26474571	C	T	-0.0312	0.0056	2.05E-08	0.1838	NA	170756	rs5629696	329443	ieu-b-102	TRUE	reported	WYslv6
6	26476782	T	A	-0.0312	0.0056	2.06E-08	0.1838	NA	170756	rs3757150	329443	ieu-b-102	TRUE	reported	WYslv6
6	26477143	T	A	-0.0311	0.0056	2.10E-08	0.1838	NA	170756	rs1689171	329443	ieu-b-102	TRUE	reported	WYslv6
6	26478252	C	T	-0.0477	0.0069	3.53E-12	0.1112	NA	170756	rs3410439	329443	ieu-b-102	TRUE	reported	WYslv6
6	26478927	G	A	-0.0309	0.0056	2.63E-08	0.1838	NA	170756	rs9358946	329443	ieu-b-102	TRUE	reported	WYslv6
6	26479000	G	A	-0.0311	0.0056	2.25E-08	0.1837	NA	170756	rs9358947	329443	ieu-b-102	TRUE	reported	WYslv6
6	26479150	C	T	-0.0442	0.0065	1.11E-11	0.1251	NA	170756	rs1689172	329443	ieu-b-102	TRUE	reported	WYslv6
6	26481642	T	G	-0.031	0.0056	2.53E-08	0.1838	NA	170756	rs7756567	329443	ieu-b-102	TRUE	reported	WYslv6
6	26482468	G	A	-0.0308	0.0056	3.05E-08	0.1838	NA	170756	rs9348720	329443	ieu-b-102	TRUE	reported	WYslv6
6	26483806	G	A	-0.0311	0.0056	2.38E-08	0.1836	NA	170756	rs9358948	329443	ieu-b-102	TRUE	reported	WYslv6
6	26485573	A	G	-0.0453	0.0066	5.30E-12	0.1226	NA	170756	rs9348721	329443	ieu-b-102	TRUE	reported	WYslv6
6	26487169	C	T	-0.0456	0.0066	4.15E-12	0.1226	NA	170756	rs6900665	329443	ieu-b-102	TRUE	reported	WYslv6
6	26488860	C	A	-0.0456	0.0066	4.11E-12	0.1226	NA	170756	rs3738676	329443	ieu-b-102	TRUE	reported	WYslv6
6	26489607	C	T	-0.0317	0.0056	1.46E-08	0.1815	NA	170756	rs3431309	329443	ieu-b-102	TRUE	reported	WYslv6
6	26493384	G	T	-0.0315	0.0056	1.77E-08	0.1813	NA	170756	rs1319504	329443	ieu-b-102	TRUE	reported	WYslv6
6	26495099	C	T	-0.031	0.0056	2.51E-08	0.184	NA	170756	rs2093169	329443	ieu-b-102	TRUE	reported	WYslv6
6	26496916	G	A	-0.0313	0.0056	1.79E-08	0.1838	NA	170756	rs9379880	329443	ieu-b-102	TRUE	reported	WYslv6
6	26498758	G	T	-0.0537	0.0072	8.32E-14	0.0999	NA	170756	rs7284378	329443	ieu-b-102	TRUE	reported	WYslv6
6	26499185	C	T	-0.0479	0.0069	3.99E-12	0.1115	NA	170756	rs1322026	329443	ieu-b-102	TRUE	reported	WYslv6
6	26500011	C	G	-0.0443	0.0064	4.70E-12	0.1297	NA	170756	rs9393727	329443	ieu-b-102	TRUE	reported	WYslv6
6	26501768	C	T	-0.0442	0.0064	5.19E-12	0.1297	NA	170756	rs2295593	329443	ieu-b-102	TRUE	reported	WYslv6
6	26509382	C	T	-0.0479	0.0069	2.93E-12	0.1112	NA	170756	rs7575772	329443	ieu-b-102	TRUE	reported	WYslv6
6	26510748	C	T	-0.0443	0.0064	4.71E-12	0.1297	NA	170756	rs3799383	329443	ieu-b-102	TRUE	reported	WYslv6
6	26511076	C	T	-0.0443	0.0064	4.81E-12	0.1297	NA	170756	rs9379882	329443	ieu-b-102	TRUE	reported	WYslv6
6	26511744	G	C	-0.0481	0.0069	2.24E-12	0.1112	NA	170756	rs3414826	329443	ieu-b-102	TRUE	reported	WYslv6
6	26514940	C	T	-0.0443	0.0064	4.61E-12	0.1296	NA	170756	rs1217612	329443	ieu-b-102	TRUE	reported	WYslv6
6	26529890	G	A	-0.0482	0.0069	2.02E-12	0.1114	NA	170756	rs3543303	329443	ieu-b-102	TRUE	reported	WYslv6
6	26530376	C	T	-0.0481	0.0069	2.19E-12	0.1114	NA	170756	rs6694110	329443	ieu-b-102	TRUE	reported	WYslv6
6	26530419	A	G	-0.0443	0.0064	4.55E-12	0.1297	NA	170756	rs6723493	329443	ieu-b-102	TRUE	reported	WYslv6
6	26534024	G	T	-0.0379	0.0064	4.05E-09	0.1555	NA	170756	rs1021299	329443	ieu-b-102	TRUE	reported	WYslv6
6	26537801	G	A	-0.0454	0.0064	1.87E-12	0.1282	NA	170756	rs6920256	329443	ieu-b-102	TRUE	reported	WYslv6
6	26549212	G	A	-0.0485	0.0069	1.52E-12	0.1114	NA	170756	rs3424677	329443	ieu-b-102	TRUE	reported	WYslv6
6	26550144	C	T	-0.0485	0.0069	1.49E-12	0.1114	NA	170756	rs7284542	329443	ieu-b-102	TRUE	reported	WYslv6
6	26555433	C	A	-0.0446	0.0064	3.65E-12	0.1296	NA	170756	rs1690178	329443	ieu-b-102	TRUE	reported	WYslv6
6	26562269	G	T	-0.0488	0.0069	1.07E-12	0.1113	NA	170756	rs3527723	329443	ieu-b-102	TRUE	reported	WYslv6
6	26563864	A	C	-0.0486	0.0069	1.36E-12	0.1116	NA	170756	rs7284446	329443	ieu-b-102	TRUE	reported	WYslv6
6	26569135	T	C	-0.0493	0.0069	6.10E-13	0.1117	NA	170756	rs3616239	329443	ieu-b-102	TRUE	reported	WYslv6
6	26573631	C	G	-0.0431	0.0063	6.69E-12	0.1366	NA	170756	rs1321402	329443	ieu-b-102	TRUE	reported	WYslv6
6	26579560	C	T	-0.0427	0.0063	1.13E-11	0.1366	NA	170756	rs9379891	329443	ieu-b-102	TRUE	reported	WYslv6
6	26582035	C	T	-0.0542	0.0072	4.73E-14	0.1005	NA	170756	rs1319871	329443	ieu-b-102	TRUE	reported	WYslv6
6	26582327	A	G	-0.0438	0.0063	4.14E-12	0.1353	NA	170756	rs9393735	329443	ieu-b-102	TRUE	reported	WYslv6
6	26587373	G	T	-0.0501	0.0069	3.26E-13	0.111	NA	170756	rs1319073	329443	ieu-b-102	TRUE	reported	WYslv6
6	26588521	C	T	-0.0468	0.0065	8.58E-13	0.1237	NA	170756	rs3422956	329443	ieu-b-102	TRUE	reported	WYslv6
6	26590578	A	T	-0.0468	0.0065	8.05E-13	0.1237	NA	170756	rs1320335	329443	ieu-b-102	TRUE	reported	WYslv6
6	26593037	A	G	-0.05	0.0069	3.52E-13	0.1109	NA	170756	rs3478127	329443	ieu-b-102	TRUE	reported	WYslv6
6	26593275	C	T	-0.0544	0.0072	3.87E-14	0.1001	NA	170756	rs3540031	329443	ieu-b-102	TRUE	reported	WYslv6
6	26598004	A	G	-0.0467	0.0065	8.88E-13	0.1238	NA	170756	rs4634439	329443	ieu-b-102	TRUE	reported	WYslv6
6	26599509	A	G	-0.0545	0.0072	3.82E-14	0.0998	NA	170756	rs4552743	329443	ieu-b-102	TRUE	reported	WYslv6
6	26601526	T	C	-0.0472	0.0065	5.01E-13	0.124	NA	170756	rs9379897	329443	ieu-b-102	TRUE	reported	WYslv6
6	26601940	G	A	-0.0463	0.0065	1.36E-12	0.1242	NA	170756	rs1320891	329443	ieu-b-102	TRUE	reported	WYslv6
6	26602453	A	G	-0.0468	0.0065	8.23E-13	0.1238	NA	170756	rs2836051	329443	ieu-b-102	TRUE	reported	WYslv6
6	26603015	T	A	-0.0468	0.0065	7.85E-13	0.1238	NA	170756	rs9379899	329443	ieu-b-102	TRUE	reported	WYslv6
6	26603811	A	C	-0.0462	0.0066	2.05E-12	0.1249	NA	170756	rs1455828	329443	ieu-b-102	TRUE	reported	WYslv6
6	26603866	T	C	-0.0467	0.0065	8.79E-13	0.1238	NA	170756	rs9379901	329443	ieu-b-102	TRUE	reported	WYslv6
6	26603924	C	A	-0.0499	0.0069	3.74E-13	0.111	NA	170756	rs3507654	329443	ieu-b-102	TRUE	reported	WYslv6
6	26605206	G	A	-0.0467	0.0065	9.56E-13	0.1238	NA	170756	rs9348726	329443	ieu-b-102	TRUE	reported	WYslv6
6	26609989	G	C	-0.0497	0.0069	4.86E-13	0.1111	NA	170756	rs1321298	329443	ieu-b-102	TRUE	reported	WYslv6
6	26631568	G	T	-0.0542	0.0072	5.01E-14	0.1	NA	170756	rs6648831	329443	ieu-b-102	TRUE	reported	WYslv6
6	26633711	T	A	-0.0543	0.0072	4.76E-14	0.1001	NA	170756	rs6777715	329443	ieu-b-102	TRUE	reported	WYslv6
6	26651053	T	A	-0.0539	0.0072	6.53E-14	0.1002	NA	170756	rs1320178	329443	ieu-b-102	TRUE	reported	WYslv6
6	26668216	G	A	0.024	0.0043	3.15E-08	0.4595	NA	170756	rs6239620	329443	ieu-b-102	TRUE	reported	WYslv6
6	26670618	A	G	-0.046	0.0068	1.58E-11	0.1145	NA	170756	rs1491231	329443	ieu-b-102	TRUE	reported	WYslv6
6	26743531	G	C	-0.0489	0.0073	1.67E-11	0.1231	NA	170756	rs1321143	329443	ieu-b-102	TRUE	reported	WYslv6
6	26799828	C	G	-0.048	0.0072	3.08E-11	0.1113	NA	170756	rs7454868	329443	ieu-b-102	TRUE	reported	WYslv6
6	26824920	T	C	-0.0483	0.0069	2.36E-12	0.1118	NA	170756	rs5566482	329443	ieu-b-102	TRUE	reported	WYslv6
6	26828359	C	T	-0.048	0.0069	3.44E-12	0.1116	NA	170756	rs1909454	329443	ieu-b-102	TRUE	reported	WYslv6
6	26843517	A	C	-0.0532	0.0072	1.31E-13	0.1011	NA	170756	rs5744016	329443	ieu-b-102	TRUE	reported	WYslv6
6	26851415	G	A	-0.0482	0.0069	2.22E-12	0.1124	NA	170756	rs7766656	329443	ieu-b-102	TRUE	reported	WYslv6
6	26903379	C	T	-0.0543	0.0073	8.30E-14	0.0995	NA	170756	rs7120606	329443	ieu-b-102	TRUE	reported	WYslv6
6	26903585	T	G	-0.0517	0.0071	3.61E-13	0.1031	NA	170756	rs9125028	329443	ieu-b-102	TRUE	reported	WYslv6
6	26909096	C	G	-0.0512	0.0071	7.49E-13	0.1028	NA	170756	rs6088211	329443	ieu-b-102	TRUE	reported	WYslv6
6	26921528	G	A	-0.0493	0.0072	8.81E-12	0.1027	NA	170756	rs1561985	329443	ieu-b-102	TRUE	reported	WYslv6
6	26927602	G	A	-0.0515	0.0071	4.32E-13	0.1031	NA	170756	rs8026458	329443	ieu-b-102	TRUE	reported	WYslv6
6	26935379	C	T	-0.0517	0.0072	5.46E-13	0.1024	NA	170756	rs3517330	329443	ieu-b-102	TRUE	reported	WYslv6
6	26942146	A	C	-0.0526	0.0072	2.58E-13	0.1009	NA	170756	rs6697520	329443	ieu-b-102	TRUE	reported	WYslv6
6	26946687	C	T	-0.0515	0.0071	3.99E-13	0.103	NA	170756	rs3586952	329443	ieu-b-102	TRUE	reported	WYslv6

6	26949672	A	G	-0.0518	0.0071	3.39E-13	0.1028	NA	170756	rs3481637	329443	ieu-b-102	TRUE	reported	WYslv6
6	26961280	C	T	-0.0537	0.0073	1.67E-13	0.0995	NA	170756	rs3454649	329443	ieu-b-102	TRUE	reported	WYslv6
6	26970895	C	A	-0.0446	0.0063	2.06E-12	0.1329	NA	170756	rs3486499	329443	ieu-b-102	TRUE	reported	WYslv6
6	26985607	C	A	-0.0446	0.0063	2.05E-12	0.1329	NA	170756	rs5687466	329443	ieu-b-102	TRUE	reported	WYslv6
6	26995168	G	A	-0.0447	0.0063	1.92E-12	0.1329	NA	170756	rs1187699	329443	ieu-b-102	TRUE	reported	WYslv6
6	26998693	T	C	-0.0447	0.0063	1.90E-12	0.1329	NA	170756	rs3521279	329443	ieu-b-102	TRUE	reported	WYslv6
6	26999845	C	T	-0.0439	0.0065	1.48E-11	0.1253	NA	170756	rs1321728	329443	ieu-b-102	TRUE	reported	WYslv6
6	26999908	G	C	-0.0447	0.0063	1.79E-12	0.1329	NA	170756	rs3576928	329443	ieu-b-102	TRUE	reported	WYslv6
6	27004098	C	T	-0.0449	0.0063	1.43E-12	0.1329	NA	170756	rs5771359	329443	ieu-b-102	TRUE	reported	WYslv6
6	27007687	T	C	-0.0438	0.0064	6.73E-12	0.1311	NA	170756	rs3574136	329443	ieu-b-102	TRUE	reported	WYslv6
6	27021173	A	G	-0.0287	0.0052	3.51E-08	0.217	NA	170756	rs7283826	329443	ieu-b-102	TRUE	reported	WYslv6
6	27023081	G	A	-0.0286	0.0052	4.01E-08	0.2171	NA	170756	rs1221524	329443	ieu-b-102	TRUE	reported	WYslv6
6	27024687	C	T	-0.031	0.0051	1.74E-09	0.2257	NA	170756	rs3740981	329443	ieu-b-102	TRUE	reported	WYslv6
6	27025281	T	C	-0.031	0.0051	1.58E-09	0.2257	NA	170756	rs1219921	329443	ieu-b-102	TRUE	reported	WYslv6
6	27030924	A	G	-0.031	0.0051	1.63E-09	0.2257	NA	170756	rs3922717	329443	ieu-b-102	TRUE	reported	WYslv6
6	27030980	T	C	-0.0309	0.0051	1.84E-09	0.2255	NA	170756	rs1320736	329443	ieu-b-102	TRUE	reported	WYslv6
6	27031029	A	G	-0.0439	0.0065	1.39E-11	0.1254	NA	170756	rs1319132	329443	ieu-b-102	TRUE	reported	WYslv6
6	27031100	C	T	-0.031	0.0051	1.70E-09	0.2257	NA	170756	rs1220345	329443	ieu-b-102	TRUE	reported	WYslv6
6	27031567	C	T	0.092	0.0169	4.96E-08	0.0168	NA	170756	rs1465294	329443	ieu-b-102	TRUE	reported	WYslv6
6	27032711	T	G	-0.0311	0.0051	1.48E-09	0.2258	NA	170756	rs3604869	329443	ieu-b-102	TRUE	reported	WYslv6
6	27033104	T	C	-0.031	0.0051	1.67E-09	0.2257	NA	170756	rs1220428	329443	ieu-b-102	TRUE	reported	WYslv6
6	27039233	T	C	-0.0285	0.0052	4.36E-08	0.2171	NA	170756	rs1221577	329443	ieu-b-102	TRUE	reported	WYslv6
6	27040273	T	A	-0.0286	0.0052	4.20E-08	0.217	NA	170756	rs1219085	329443	ieu-b-102	TRUE	reported	WYslv6
6	27040402	G	C	-0.0441	0.0065	1.15E-11	0.1254	NA	170756	rs3560861	329443	ieu-b-102	TRUE	reported	WYslv6
6	27042287	C	A	-0.0446	0.0065	6.77E-12	0.1254	NA	170756	rs3552652	329443	ieu-b-102	TRUE	reported	WYslv6
6	27046250	A	G	-0.0286	0.0052	3.90E-08	0.2173	NA	170756	rs6940638	329443	ieu-b-102	TRUE	reported	WYslv6
6	27047256	G	A	-0.036	0.0056	1.44E-10	0.1789	NA	170756	rs6904071	329443	ieu-b-102	TRUE	reported	WYslv6
6	27050396	A	G	-0.0362	0.0056	1.19E-10	0.1788	NA	170756	rs3438870	329443	ieu-b-102	TRUE	reported	WYslv6
6	27054772	G	A	-0.0534	0.0072	8.78E-14	0.1012	NA	170756	rs6709207	329443	ieu-b-102	TRUE	reported	WYslv6
6	27059443	A	T	-0.0357	0.0056	1.99E-10	0.1798	NA	170756	rs926300	329443	ieu-b-102	TRUE	reported	WYslv6
6	27063547	T	C	0.0904	0.0162	2.51E-08	0.018	NA	170756	rs7518859	329443	ieu-b-102	TRUE	reported	WYslv6
6	27067083	C	T	-0.0359	0.0056	1.61E-10	0.1792	NA	170756	rs7284219	329443	ieu-b-102	TRUE	reported	WYslv6
6	27072542	A	G	-0.0542	0.0072	4.16E-14	0.1007	NA	170756	rs5583452	329443	ieu-b-102	TRUE	reported	WYslv6
6	27091425	C	A	-0.0357	0.0056	1.96E-10	0.1793	NA	170756	rs6913660	329443	ieu-b-102	TRUE	reported	WYslv6
6	27091661	T	C	-0.0537	0.0072	7.41E-14	0.1011	NA	170756	rs6646218	329443	ieu-b-102	TRUE	reported	WYslv6
6	27103654	G	A	-0.0356	0.0056	2.17E-10	0.1793	NA	170756	rs1180274	329443	ieu-b-102	TRUE	reported	WYslv6
6	27124904	G	C	-0.0357	0.0056	1.87E-10	0.1795	NA	170756	rs6124010	329443	ieu-b-102	TRUE	reported	WYslv6
6	27136225	A	G	-0.0354	0.0056	2.75E-10	0.1792	NA	170756	rs1321918	329443	ieu-b-102	TRUE	reported	WYslv6
6	27139048	C	T	-0.0353	0.0056	3.17E-10	0.1792	NA	170756	rs6914824	329443	ieu-b-102	TRUE	reported	WYslv6
6	27140866	T	A	-0.0353	0.0056	3.08E-10	0.1793	NA	170756	rs6754023	329443	ieu-b-102	TRUE	reported	WYslv6
6	27141904	C	T	-0.0535	0.0072	8.21E-14	0.1011	NA	170756	rs3576859	329443	ieu-b-102	TRUE	reported	WYslv6
6	27143883	T	C	-0.0353	0.0056	3.04E-10	0.1795	NA	170756	rs1319405	329443	ieu-b-102	TRUE	reported	WYslv6
6	27145341	C	T	-0.0531	0.0072	1.23E-13	0.1014	NA	170756	rs3556544	329443	ieu-b-102	TRUE	reported	WYslv6
6	27153984	A	C	-0.0356	0.0056	2.34E-10	0.1797	NA	170756	rs3456920	329443	ieu-b-102	TRUE	reported	WYslv6
6	27158033	A	G	-0.0351	0.0056	2.74E-10	0.1842	NA	170756	rs6934329	329443	ieu-b-102	TRUE	reported	WYslv6
6	27163456	G	A	-0.0434	0.0065	2.63E-11	0.1262	NA	170756	rs3590954	329443	ieu-b-102	TRUE	reported	WYslv6
6	27171700	T	C	-0.0339	0.0056	1.62E-09	0.1799	NA	170756	rs3478355	329443	ieu-b-102	TRUE	reported	WYslv6
6	27174557	A	G	0.0705	0.012	4.71E-09	0.0327	NA	170756	rs1773764	329443	ieu-b-102	TRUE	reported	WYslv6
6	27176628	G	A	-0.0426	0.0065	5.10E-11	0.1264	NA	170756	rs3481711	329443	ieu-b-102	TRUE	reported	WYslv6
6	27182377	A	C	0.0704	0.012	4.51E-09	0.0327	NA	170756	rs1501868	329443	ieu-b-102	TRUE	reported	WYslv6
6	27184949	C	T	0.0691	0.0121	1.04E-08	0.0328	NA	170756	rs1472178	329443	ieu-b-102	TRUE	reported	WYslv6
6	27185354	G	A	-0.0424	0.0065	6.13E-11	0.1265	NA	170756	rs5686231	329443	ieu-b-102	TRUE	reported	WYslv6
6	27185664	T	C	-0.0421	0.0065	8.06E-11	0.1265	NA	170756	rs1321935	329443	ieu-b-102	TRUE	reported	WYslv6
6	27191456	C	T	0.0662	0.0115	1.01E-08	0.0356	NA	170756	rs1405271	329443	ieu-b-102	TRUE	reported	WYslv6
6	27197931	A	G	0.0781	0.0132	3.13E-09	0.0271	NA	170756	rs5565885	329443	ieu-b-102	TRUE	reported	WYslv6
6	27198343	T	G	-0.0426	0.0065	4.93E-11	0.1264	NA	170756	rs6745745	329443	ieu-b-102	TRUE	reported	WYslv6
6	27203350	G	A	0.0661	0.0116	1.18E-08	0.0357	NA	170756	rs1164257	329443	ieu-b-102	TRUE	reported	WYslv6
6	27205422	C	T	-0.0426	0.0065	5.28E-11	0.1263	NA	170756	rs1321292	329443	ieu-b-102	TRUE	reported	WYslv6
6	27221047	C	T	0.0683	0.0119	1.10E-08	0.033	NA	170756	rs1148570	329443	ieu-b-102	TRUE	reported	WYslv6
6	27226775	C	T	0.0716	0.013	3.46E-08	0.0279	NA	170756	rs1502074	329443	ieu-b-102	TRUE	reported	WYslv6
6	27229265	G	A	-0.0422	0.0065	7.27E-11	0.1267	NA	170756	rs4452638	329443	ieu-b-102	TRUE	reported	WYslv6
6	27231666	G	A	0.0682	0.012	1.19E-08	0.033	NA	170756	rs5613831	329443	ieu-b-102	TRUE	reported	WYslv6
6	27245340	T	C	0.0678	0.0118	1.04E-08	0.0337	NA	170756	rs1150913	329443	ieu-b-102	TRUE	reported	WYslv6
6	27249686	G	A	-0.0538	0.0072	7.39E-14	0.1001	NA	170756	rs7283944	329443	ieu-b-102	TRUE	reported	WYslv6
6	27251379	A	G	-0.0526	0.0072	2.20E-13	0.1008	NA	170756	rs1320708	329443	ieu-b-102	TRUE	reported	WYslv6
6	27257143	A	C	0.0741	0.0131	1.63E-08	0.0274	NA	170756	rs7709390	329443	ieu-b-102	TRUE	reported	WYslv6
6	27259792	T	C	0.0737	0.0131	1.76E-08	0.0273	NA	170756	rs7673639	329443	ieu-b-102	TRUE	reported	WYslv6
6	27271590	A	G	0.0731	0.0131	2.22E-08	0.0273	NA	170756	rs1471210	329443	ieu-b-102	TRUE	reported	WYslv6
6	27274834	C	T	-0.0543	0.0072	4.14E-14	0.1003	NA	170756	rs5611437	329443	ieu-b-102	TRUE	reported	WYslv6
6	27296894	G	A	0.0725	0.0131	2.94E-08	0.0275	NA	170756	rs1447447	329443	ieu-b-102	TRUE	reported	WYslv6
6	27309779	G	C	-0.0565	0.0071	1.95E-15	0.1027	NA	170756	rs7153755	329443	ieu-b-102	TRUE	reported	WYslv6
6	27318848	G	A	-0.0246	0.0043	1.21E-08	0.5398	NA	170756	rs7772739	329443	ieu-b-102	TRUE	reported	WYslv6
6	27320744	G	C	-0.0248	0.0043	1.01E-08	0.539	NA	170756	rs2393926	329443	ieu-b-102	TRUE	reported	WYslv6
6	27326863	G	A	-0.0242	0.0043	2.14E-08	0.54	NA	170756	rs2235232	329443	ieu-b-102	TRUE	reported	WYslv6
6	27327000	C	T	-0.0572	0.0071	1.17E-15	0.1014	NA	170756	rs7283947	329443	ieu-b-102	TRUE	reported	WYslv6
6	27332891	T	C	-0.0565	0.0071	1.94E-15	0.1023	NA	170756	rs1310759	329443	ieu-b-102	TRUE	reported	WYslv6
6	27334573	G	A	-0.0241	0.0043	2.73E-08	0.5403	NA	170756	rs9357038	329443	ieu-b-102	TRUE	reported	WYslv6
6	27352750	G	A	-0.056	0.0072	5.14E-15	0.1022	NA	170756	rs7155902	329443	ieu-b-102	TRUE	reported	WYslv6
6	27357414	C	G	-0.056	0.0071	3.30E-15	0.103	NA	170756	rs3433255	329443	ieu-b-102	TRUE	reported	WYslv6
6	27357978	A	G	-0.0568	0.0071	1.97E-15	0.1014	NA	170756	rs6785963	329443	ieu-b-102	TRUE	reported	WYslv6
6	27358166	A	G	-0.0557	0.0071	4.30E-15	0.1028	NA	170756	rs3512005	329443	ieu-b-102	TRUE	reported	WYslv6
6	27361681	T	C	0.0246	0.0043	1.20E-08	0.4598	NA	170756	rs1252945	329443	ieu-b-102	TRUE	reported	WYslv6
6	27363644	A	C	0.0249	0.0043	8.12E-09	0.459	NA	170756	rs1266499	329443	ieu-b-102	TRUE	reported	WYslv6
6	27369704	C	G	-0.0563	0.0071	3.21E-15	0.1017	NA	170756	rs1320768	329443	ieu-b-102	TRUE	reported	WYslv6
6	27374818	C	G	0.0277	0.0045	9.52E-10	0.6568	NA	170756	rs4516940	329443	ieu-b-102	TRUE	reported	WYslv6
6	27375611	A	C	0.0274	0.0045	1.43E-09	0.6568	NA	170756	rs2076305	329443	ieu-b-102	TRUE	reported	WYslv6
6	27376001	G	A	0.0287	0.0045	1.67E-10	0.6439	NA	170756	rs2064219	329443	ieu-b-102	TRUE	reported	WYslv6
6	27376910	G	A	0.0283	0.0044	1.55E-10	0.6158	NA	170756	rs6938943	329443	ieu-b-102	TRUE	reported	WYslv6
6	27377235	A	G	-0.0556	0.0071	4.96E-15	0.1028	NA	170756	rs1321853	329443	ieu-b-102	TRUE	reported	WYslv6
6	27377668	T	C	0.0282	0.0044	1.69E-10	0.6158	NA	170756	rs1252525	329443	ieu-b-102	TRUE	reported	WYslv6
6	27377830	A	G	0.0237	0.0043	3.89E-08	0.4629	NA	170756	rs1094691	329443	ieu-b-102	TRUE	reported	WYslv6
6	27378373	C	T	0.0284	0.0044	1.40E-10	0.6136	NA	170756	rs4711150	329443	ieu-b-102	TRUE	reported	WYslv6
6	27379119	C	T	-0.0561	0.0071	3.80E-15	0.1018	NA	170756	rs1319669	329443	ieu-b-102	TRUE	reported	WYslv6
6	27379372	A	G	0.0282	0.0043	6.96E-11	0.5393	NA	170756	rs6908553	329443	ieu-b-102	TRUE	reported	WYslv6
6	27379409	G	A	0.0272	0.0044	9.56E-10	0.6285	NA	170756	rs4713096	329443	ieu-b-102	TRUE	reported	WYslv6
6	27379547	G	A	0.0268	0.0045	2.05E-09	0.6285	NA	170756	rs4713097	329443	ieu-b-102	TRUE	reported	WYslv6
6	27380101	A	G	0.0264	0.0045	3.31E-09	0.624	NA	170756	rs4711151	329443	ieu-b-102	TRUE	reported	WYslv6
6	27380202	T	C	0.0236	0.0043	4.88E-08	0.4592	NA	170756	rs4711152	329443	ieu-b-102	TRUE	reported	WYslv6
6	27380897	C	T	0.0288	0.0043	2.37E-11	0.5348	NA	170756	rs1252526	329443	ieu-b-102	TRUE	reported	WYslv6
6	27380978	C	T	0.0279	0.0044	2.61E-10	0.6107	NA	170756	rs1094691	329443	ieu-b-102	TRUE	reported	WYslv6
6	27380993	G	A	0.0268	0.0044	1.59E-09	0.624	NA	170756	rs1094691	329443	ieu-b-102	TRUE	reported	WYslv6
6	27381660	T	C	-0.0556	0.0071	5.41E-15	0.1028	NA	170756	rs3495337	329443	ieu-b-102	TRUE	reported	WYslv6
6	27382386	G	T	-0.0556	0.0071	5.44E-15	0.1027	NA	170756	rs6684163	329443	ieu-b-102	TRUE	reported	WYslv6
6	27384520	T	C	0.0274	0.0043	2.03E-10	0.5409	NA	170756	rs6929812	329443	ieu-b-102	TRUE	reported	WYslv6
6	27388027	C	G	0.0265	0.0044	2.21E-09	0.6247	NA	170756	rs2064220	329443	ieu-b-102	TRUE	reported	WYslv6
6	27388448	C	A	0.0287	0.0043	2.77E-11	0.5358	NA	170756	rs2064221	329443	ieu-b-102	TRUE	reported	WYslv6
6	27388739	G	A	0.0269	0.0044	1.25E-09	0.6247	NA	170756	rs6456782	329443	ieu-b-102	TRUE	reported	WYslv6

6	27388754	T	C	-0.0559	0.0071	4.74E-15	0.1017	NA	170756	rs3415072	329443	ieu-b-102	TRUE	reported	WYslv6
6	27389150	T	G	0.0263	0.0045	3.45E-09	0.6249	NA	170756	rs7753569	329443	ieu-b-102	TRUE	reported	WYslv6
6	27389258	A	C	0.0265	0.0045	2.89E-09	0.6249	NA	170756	rs7771598	329443	ieu-b-102	TRUE	reported	WYslv6
6	27389490	T	C	0.0265	0.0045	2.70E-09	0.6251	NA	170756	rs7754086	329443	ieu-b-102	TRUE	reported	WYslv6
6	27389892	C	A	0.026	0.0045	5.68E-09	0.6223	NA	170756	rs6240113	329443	ieu-b-102	TRUE	reported	WYslv6
6	27390021	A	T	0.0255	0.0045	1.29E-08	0.6244	NA	170756	rs6456784	329443	ieu-b-102	TRUE	reported	WYslv6
6	27390115	G	C	-0.0558	0.0071	5.24E-15	0.1017	NA	170756	rs1319122	329443	ieu-b-102	TRUE	reported	WYslv6
6	27390399	T	G	0.027	0.0043	3.63E-10	0.5483	NA	170756	rs6456785	329443	ieu-b-102	TRUE	reported	WYslv6
6	27390993	T	C	0.0264	0.0045	2.96E-09	0.6249	NA	170756	rs6928314	329443	ieu-b-102	TRUE	reported	WYslv6
6	27391243	T	A	0.0264	0.0045	2.99E-09	0.6249	NA	170756	rs1221228	329443	ieu-b-102	TRUE	reported	WYslv6
6	27391320	C	T	0.0263	0.0045	3.37E-09	0.6249	NA	170756	rs1220518	329443	ieu-b-102	TRUE	reported	WYslv6
6	27391802	C	T	-0.0554	0.0071	5.73E-15	0.1029	NA	170756	rs3407125	329443	ieu-b-102	TRUE	reported	WYslv6
6	27391935	G	A	0.0287	0.0043	2.58E-11	0.5358	NA	170756	rs3418546	329443	ieu-b-102	TRUE	reported	WYslv6
6	27392041	G	A	0.0264	0.0045	3.30E-09	0.6249	NA	170756	rs1252554	329443	ieu-b-102	TRUE	reported	WYslv6
6	27392110	A	G	0.0267	0.0044	1.69E-09	0.6248	NA	170756	rs1252890	329443	ieu-b-102	TRUE	reported	WYslv6
6	27392148	C	G	0.0264	0.0045	3.11E-09	0.6249	NA	170756	rs1320709	329443	ieu-b-102	TRUE	reported	WYslv6
6	27392955	C	T	0.0256	0.0045	9.39E-09	0.6232	NA	170756	rs1387306	329443	ieu-b-102	TRUE	reported	WYslv6
6	27393709	T	C	0.0264	0.0045	3.06E-09	0.6249	NA	170756	rs7747444	329443	ieu-b-102	TRUE	reported	WYslv6
6	27393871	G	A	0.0238	0.0043	3.48E-08	0.4589	NA	170756	rs1252649	329443	ieu-b-102	TRUE	reported	WYslv6
6	27393884	A	G	0.0264	0.0045	3.35E-09	0.6249	NA	170756	rs1252984	329443	ieu-b-102	TRUE	reported	WYslv6
6	27395764	T	C	0.0285	0.0043	3.47E-11	0.535	NA	170756	rs6926142	329443	ieu-b-102	TRUE	reported	WYslv6
6	27396066	T	C	0.0267	0.0044	1.77E-09	0.6248	NA	170756	rs2206253	329443	ieu-b-102	TRUE	reported	WYslv6
6	27396658	C	A	0.0286	0.0043	3.31E-11	0.535	NA	170756	rs2893916	329443	ieu-b-102	TRUE	reported	WYslv6
6	27397198	T	C	0.0267	0.0044	1.81E-09	0.6248	NA	170756	rs1048439	329443	ieu-b-102	TRUE	reported	WYslv6
6	27398133	G	A	0.0263	0.0045	3.67E-09	0.624	NA	170756	rs3866982	329443	ieu-b-102	TRUE	reported	WYslv6
6	27399631	C	A	0.0267	0.0044	1.77E-09	0.6243	NA	170756	rs2206254	329443	ieu-b-102	TRUE	reported	WYslv6
6	27400031	G	T	0.0266	0.0044	2.14E-09	0.6243	NA	170756	rs1569566	329443	ieu-b-102	TRUE	reported	WYslv6
6	27400938	G	C	0.0267	0.0044	1.80E-09	0.6249	NA	170756	rs979353	329443	ieu-b-102	TRUE	reported	WYslv6
6	27401196	C	T	0.0263	0.0045	3.61E-09	0.6244	NA	170756	rs979352	329443	ieu-b-102	TRUE	reported	WYslv6
6	27402023	A	G	0.0265	0.0044	2.20E-09	0.6243	NA	170756	rs6908086	329443	ieu-b-102	TRUE	reported	WYslv6
6	27403225	C	A	0.0266	0.0044	2.05E-09	0.6251	NA	170756	rs6914542	329443	ieu-b-102	TRUE	reported	WYslv6
6	27403345	A	T	0.0262	0.0045	3.99E-09	0.6243	NA	170756	rs6914585	329443	ieu-b-102	TRUE	reported	WYslv6
6	27403605	C	T	0.0236	0.0043	4.46E-08	0.459	NA	170756	rs2001262	329443	ieu-b-102	TRUE	reported	WYslv6
6	27404067	T	C	0.0261	0.0045	5.49E-09	0.6231	NA	170756	rs6900278	329443	ieu-b-102	TRUE	reported	WYslv6
6	27404771	A	G	0.0265	0.0044	2.22E-09	0.6243	NA	170756	rs1219785	329443	ieu-b-102	TRUE	reported	WYslv6
6	27405683	T	C	0.0266	0.0044	2.03E-09	0.6243	NA	170756	rs1048439	329443	ieu-b-102	TRUE	reported	WYslv6
6	27406156	A	G	0.0266	0.0044	2.00E-09	0.6243	NA	170756	rs1220001	329443	ieu-b-102	TRUE	reported	WYslv6
6	27406607	C	G	-0.0552	0.0071	7.86E-15	0.1027	NA	170756	rs3571647	329443	ieu-b-102	TRUE	reported	WYslv6
6	27407798	C	T	0.0264	0.0045	3.28E-09	0.6241	NA	170756	rs4713101	329443	ieu-b-102	TRUE	reported	WYslv6
6	27408181	G	T	0.0262	0.0044	4.10E-09	0.6317	NA	170756	rs760880	329443	ieu-b-102	TRUE	reported	WYslv6
6	27409164	T	C	0.0267	0.0044	1.84E-09	0.6249	NA	170756	rs5764704	329443	ieu-b-102	TRUE	reported	WYslv6
6	27409287	T	C	0.0266	0.0044	1.99E-09	0.6235	NA	170756	rs5570518	329443	ieu-b-102	TRUE	reported	WYslv6
6	27410220	G	A	0.0272	0.0044	8.17E-10	0.623	NA	170756	rs9016678	329443	ieu-b-102	TRUE	reported	WYslv6
6	27410422	G	A	-0.0546	0.0069	2.64E-15	0.1094	NA	170756	rs1195166	329443	ieu-b-102	TRUE	reported	WYslv6
6	27410679	A	G	0.0249	0.0045	4.47E-08	0.5975	NA	170756	rs1561831	329443	ieu-b-102	TRUE	reported	WYslv6
6	27412386	C	T	0.0289	0.0043	1.85E-11	0.5334	NA	170756	rs7772160	329443	ieu-b-102	TRUE	reported	WYslv6
6	27412730	T	C	0.0271	0.0044	1.01E-09	0.6232	NA	170756	rs7754678	329443	ieu-b-102	TRUE	reported	WYslv6
6	27412967	C	A	0.0269	0.0044	1.25E-09	0.6229	NA	170756	rs1094691	329443	ieu-b-102	TRUE	reported	WYslv6
6	27413345	C	T	-0.0554	0.0071	6.72E-15	0.1026	NA	170756	rs1319147	329443	ieu-b-102	TRUE	reported	WYslv6
6	27413831	A	T	0.0273	0.0043	2.85E-10	0.5451	NA	170756	rs3539445	329443	ieu-b-102	TRUE	reported	WYslv6
6	27413924	A	G	-0.0554	0.0071	6.94E-15	0.1026	NA	170756	rs1319504	329443	ieu-b-102	TRUE	reported	WYslv6
6	27415831	T	C	0.0268	0.0044	1.56E-09	0.6232	NA	170756	rs6456786	329443	ieu-b-102	TRUE	reported	WYslv6
6	27418847	T	G	0.0274	0.0043	2.23E-10	0.5452	NA	170756	rs7509	329443	ieu-b-102	TRUE	reported	WYslv6
6	27421304	C	T	0.0269	0.0044	1.31E-09	0.6231	NA	170756	rs7744110	329443	ieu-b-102	TRUE	reported	WYslv6
6	27422066	C	T	0.028	0.0045	5.85E-10	0.6571	NA	170756	rs1459522	329443	ieu-b-102	TRUE	reported	WYslv6
6	27422493	T	C	0.027	0.0044	1.17E-09	0.6231	NA	170756	rs2235252	329443	ieu-b-102	TRUE	reported	WYslv6
6	27422794	G	A	0.0269	0.0044	1.37E-09	0.623	NA	170756	rs2235254	329443	ieu-b-102	TRUE	reported	WYslv6
6	27423190	G	A	0.0271	0.0046	2.45E-09	0.6643	NA	170756	rs726830	329443	ieu-b-102	TRUE	reported	WYslv6
6	27423568	G	A	0.0269	0.0044	1.24E-09	0.6231	NA	170756	rs1219911	329443	ieu-b-102	TRUE	reported	WYslv6
6	27424184	T	C	0.0268	0.0044	1.54E-09	0.623	NA	170756	rs1187516	329443	ieu-b-102	TRUE	reported	WYslv6
6	27424311	A	G	0.0268	0.0044	1.46E-09	0.623	NA	170756	rs2092122	329443	ieu-b-102	TRUE	reported	WYslv6
6	27424374	G	A	0.0269	0.0044	1.28E-09	0.6231	NA	170756	rs764460	329443	ieu-b-102	TRUE	reported	WYslv6
6	27424443	T	A	0.0268	0.0044	1.47E-09	0.623	NA	170756	rs764461	329443	ieu-b-102	TRUE	reported	WYslv6
6	27424960	G	C	0.0268	0.0044	1.47E-09	0.623	NA	170756	rs1048439	329443	ieu-b-102	TRUE	reported	WYslv6
6	27425349	T	C	-0.0554	0.0071	6.53E-15	0.1025	NA	170756	rs4126926	329443	ieu-b-102	TRUE	reported	WYslv6
6	27425644	G	C	-0.0569	0.0073	4.85E-15	0.099	NA	170756	rs3419630	329443	ieu-b-102	TRUE	reported	WYslv6
6	27426498	G	A	0.0269	0.0044	1.31E-09	0.6231	NA	170756	rs6456789	329443	ieu-b-102	TRUE	reported	WYslv6
6	27426659	G	T	0.0269	0.0044	1.35E-09	0.6231	NA	170756	rs7743443	329443	ieu-b-102	TRUE	reported	WYslv6
6	27426717	G	T	0.0268	0.0044	1.41E-09	0.623	NA	170756	rs7743465	329443	ieu-b-102	TRUE	reported	WYslv6
6	27427542	C	T	0.0269	0.0044	1.36E-09	0.6231	NA	170756	rs7748366	329443	ieu-b-102	TRUE	reported	WYslv6
6	27428705	T	C	0.0268	0.0044	1.41E-09	0.6231	NA	170756	rs1080702	329443	ieu-b-102	TRUE	reported	WYslv6
6	27428991	C	T	0.027	0.0044	1.08E-09	0.6231	NA	170756	rs1094692	329443	ieu-b-102	TRUE	reported	WYslv6
6	27433029	C	T	-0.0547	0.0068	7.23E-16	0.1145	NA	170756	rs3461398	329443	ieu-b-102	TRUE	reported	WYslv6
6	27441723	T	A	-0.055	0.0068	4.98E-16	0.1146	NA	170756	rs1498661	329443	ieu-b-102	TRUE	reported	WYslv6
6	27442247	A	G	0.024	0.0043	2.59E-08	0.4557	NA	170756	rs2064093	329443	ieu-b-102	TRUE	reported	WYslv6
6	27443202	G	T	-0.0549	0.0068	5.48E-16	0.1145	NA	170756	rs1561846	329443	ieu-b-102	TRUE	reported	WYslv6
6	27445703	C	T	0.0241	0.0043	2.24E-08	0.4558	NA	170756	rs7761966	329443	ieu-b-102	TRUE	reported	WYslv6
6	27446446	C	A	0.0242	0.0043	2.10E-08	0.4558	NA	170756	rs4711155	329443	ieu-b-102	TRUE	reported	WYslv6
6	27446566	T	C	-0.0547	0.0068	5.40E-16	0.1149	NA	170756	rs7749305	329443	ieu-b-102	TRUE	reported	WYslv6
6	27451419	A	G	-0.0537	0.0068	3.05E-15	0.1142	NA	170756	rs6918792	329443	ieu-b-102	TRUE	reported	WYslv6
6	27456242	T	C	0.0238	0.0043	3.29E-08	0.4598	NA	170756	rs1252686	329443	ieu-b-102	TRUE	reported	WYslv6
6	27457317	A	C	0.0239	0.0043	3.06E-08	0.4598	NA	170756	rs7339536	329443	ieu-b-102	TRUE	reported	WYslv6
6	27459923	G	A	-0.055	0.0068	5.14E-16	0.1145	NA	170756	rs3486479	329443	ieu-b-102	TRUE	reported	WYslv6
6	27462824	T	C	0.0236	0.0043	4.28E-08	0.4608	NA	170756	rs2393962	329443	ieu-b-102	TRUE	reported	WYslv6
6	27474715	A	G	-0.0553	0.0071	4.48E-15	0.1047	NA	170756	rs5861663	329443	ieu-b-102	TRUE	reported	WYslv6
6	27475120	A	C	0.024	0.0043	3.35E-08	0.457	NA	170756	rs4413607	329443	ieu-b-102	TRUE	reported	WYslv6
6	27475144	A	C	0.024	0.0043	2.83E-08	0.4578	NA	170756	rs2393964	329443	ieu-b-102	TRUE	reported	WYslv6
6	27480526	C	T	-0.0551	0.007	5.21E-15	0.105	NA	170756	rs3457397	329443	ieu-b-102	TRUE	reported	WYslv6
6	27491299	G	A	-0.0545	0.0067	6.00E-16	0.1161	NA	170756	rs6904596	329443	ieu-b-102	TRUE	reported	WYslv6
6	27498217	G	C	0.0293	0.0045	4.87E-11	0.6244	NA	170756	rs1320141	329443	ieu-b-102	TRUE	reported	WYslv6
6	27498581	G	A	0.0293	0.0045	4.32E-11	0.6248	NA	170756	rs1220807	329443	ieu-b-102	TRUE	reported	WYslv6
6	27501345	T	C	0.0293	0.0044	3.92E-11	0.6244	NA	170756	rs1094693	329443	ieu-b-102	TRUE	reported	WYslv6
6	27502893	G	C	0.0287	0.0045	1.12E-10	0.6262	NA	170756	rs1094693	329443	ieu-b-102	TRUE	reported	WYslv6
6	27507494	C	G	0.0291	0.0045	7.36E-11	0.624	NA	170756	rs1221501	329443	ieu-b-102	TRUE	reported	WYslv6
6	27509493	A	C	-0.055	0.007	3.84E-15	0.1064	NA	170756	rs1319563	329443	ieu-b-102	TRUE	reported	WYslv6
6	27515519	A	C	0.0292	0.0044	5.07E-11	0.625	NA	170756	rs6910838	329443	ieu-b-102	TRUE	reported	WYslv6
6	27516833	G	T	0.0288	0.0045	1.10E-10	0.6243	NA	170756	rs4713112	329443	ieu-b-102	TRUE	reported	WYslv6
6	27520752	T	A	-0.0548	0.007	4.88E-15	0.1065	NA	170756	rs1320933	329443	ieu-b-102	TRUE	reported	WYslv6
6	27521096	C	T	-0.0564	0.0071	1.64E-15	0.1037	NA	170756	rs3584827	329443	ieu-b-102	TRUE	reported	WYslv6
6	27521856	A	G	-0.0548	0.007	4.71E-15	0.1064	NA	170756	rs3496529	329443	ieu-b-102	TRUE	reported	WYslv6
6	27523298	T	G	-0.0548	0.007	4.77E-15	0.1064	NA	170756	rs6678511	329443	ieu-b-102	TRUE	reported	WYslv6
6	27523869	G	A	-0.0548	0.007	4.63E-15	0.1065	NA	170756	rs1403650	329443	ieu-b-102	TRUE	reported	WYslv6
6	27528643	G	A	0.0285	0.0045	1.71E-10	0.6226	NA	170756	rs6915266	329443	ieu-b-102	TRUE	reported	WYslv6
6	27534528	A	G	-0.0544	0.007	6.75E-15	0.1066	NA	170756	rs1048439	329443	ieu-b-102	TRUE	reported	WYslv6
6	27556141	A	T	-0.0529	0.0067	2.76E-15	0.1214	NA	170756	rs1336201	329443	ieu-b-102	TRUE	reported	WYslv6
6	27560587	A	G	0.0284	0.0044	1.60E-10	0.6232	NA	170756	rs1094694	329443	ieu-b-102	TRUE	reported	WYslv6
6	27560805	C	T	-0.055	0.007	3.83E-15	0.1064	NA	170756	rs3410507	329443	ieu-b-102	TRUE	reported	WYslv6

6	27564547	C	T	0.0297	0.0045	2.81E-11	0.6143	NA	170756	rs5548816	329443	ieu-b-102	TRUE	reported	WYslv6
6	27564879	T	C	-0.055	0.007	3.82E-15	0.1063	NA	170756	rs1320277	329443	ieu-b-102	TRUE	reported	WYslv6
6	27573463	G	A	0.028	0.0045	3.18E-10	0.6227	NA	170756	rs1080702	329443	ieu-b-102	TRUE	reported	WYslv6
6	27573536	A	G	0.0283	0.0045	2.10E-10	0.6231	NA	170756	rs6456799	329443	ieu-b-102	TRUE	reported	WYslv6
6	27575268	T	A	-0.0549	0.007	4.32E-15	0.1063	NA	170756	rs7284504	329443	ieu-b-102	TRUE	reported	WYslv6
6	27582795	A	C	-0.0293	0.0044	4.03E-11	0.3744	NA	170756	rs9357042	329443	ieu-b-102	TRUE	reported	WYslv6
6	27586220	C	T	-0.0547	0.007	5.23E-15	0.1065	NA	170756	rs6765222	329443	ieu-b-102	TRUE	reported	WYslv6
6	27587027	C	T	-0.0281	0.0045	3.19E-10	0.3684	NA	170756	rs9393830	329443	ieu-b-102	TRUE	reported	WYslv6
6	27587518	C	T	-0.0289	0.0044	7.43E-11	0.3777	NA	170756	rs6921256	329443	ieu-b-102	TRUE	reported	WYslv6
6	27592003	C	T	-0.0576	0.0072	8.88E-16	0.1027	NA	170756	rs1212355	329443	ieu-b-102	TRUE	reported	WYslv6
6	27606716	C	T	-0.055	0.007	3.86E-15	0.1066	NA	170756	rs1321209	329443	ieu-b-102	TRUE	reported	WYslv6
6	27607660	C	A	-0.055	0.007	3.81E-15	0.1065	NA	170756	rs3403854	329443	ieu-b-102	TRUE	reported	WYslv6
6	27616843	C	T	-0.0555	0.0071	3.87E-15	0.1053	NA	170756	rs1449699	329443	ieu-b-102	TRUE	reported	WYslv6
6	27616887	T	C	-0.0293	0.0045	5.17E-11	0.375	NA	170756	rs6899903	329443	ieu-b-102	TRUE	reported	WYslv6
6	27622084	T	C	-0.0548	0.007	4.72E-15	0.1066	NA	170756	rs1413427	329443	ieu-b-102	TRUE	reported	WYslv6
6	27624640	A	G	-0.0551	0.007	3.45E-15	0.1066	NA	170756	rs3454393	329443	ieu-b-102	TRUE	reported	WYslv6
6	27625532	T	C	-0.0292	0.0044	4.49E-11	0.3768	NA	170756	rs6456801	329443	ieu-b-102	TRUE	reported	WYslv6
6	27626631	A	G	-0.0278	0.0048	9.28E-09	0.2822	NA	170756	rs1417381	329443	ieu-b-102	TRUE	reported	WYslv6
6	27633018	T	C	-0.0261	0.0045	7.22E-09	0.3519	NA	170756	rs9366697	329443	ieu-b-102	TRUE	reported	WYslv6
6	27640246	G	A	-0.0551	0.007	3.46E-15	0.1065	NA	170756	rs5640570	329443	ieu-b-102	TRUE	reported	WYslv6
6	27640989	A	G	-0.0556	0.007	2.62E-15	0.1062	NA	170756	rs7155903	329443	ieu-b-102	TRUE	reported	WYslv6
6	27646492	A	G	-0.0552	0.007	3.03E-15	0.1066	NA	170756	rs1321063	329443	ieu-b-102	TRUE	reported	WYslv6
6	27647509	G	T	-0.0552	0.007	2.91E-15	0.1065	NA	170756	rs1321527	329443	ieu-b-102	TRUE	reported	WYslv6
6	27653120	C	T	-0.0576	0.0073	2.20E-15	0.1003	NA	170756	rs1321762	329443	ieu-b-102	TRUE	reported	WYslv6
6	27665920	C	T	-0.0261	0.0045	9.20E-09	0.34	NA	170756	rs2179096	329443	ieu-b-102	TRUE	reported	WYslv6
6	27668300	A	C	-0.0563	0.0071	1.80E-15	0.1055	NA	170756	rs3592707	329443	ieu-b-102	TRUE	reported	WYslv6
6	27669976	A	G	-0.0552	0.007	2.77E-15	0.1067	NA	170756	rs1774992	329443	ieu-b-102	TRUE	reported	WYslv6
6	27671825	G	A	-0.0551	0.007	3.39E-15	0.1067	NA	170756	rs1319296	329443	ieu-b-102	TRUE	reported	WYslv6
6	27672035	C	G	-0.0303	0.0048	2.78E-10	0.2785	NA	170756	rs9366698	329443	ieu-b-102	TRUE	reported	WYslv6
6	27682914	G	T	-0.0334	0.005	2.02E-11	0.2493	NA	170756	rs7772027	329443	ieu-b-102	TRUE	reported	WYslv6
6	27683063	T	A	-0.0334	0.005	2.07E-11	0.2493	NA	170756	rs9468200	329443	ieu-b-102	TRUE	reported	WYslv6
6	27683244	A	T	-0.0335	0.005	1.78E-11	0.2493	NA	170756	rs3475287	329443	ieu-b-102	TRUE	reported	WYslv6
6	27683572	A	G	-0.0334	0.005	2.13E-11	0.2495	NA	170756	rs9380010	329443	ieu-b-102	TRUE	reported	WYslv6
6	27683798	G	A	-0.0334	0.005	2.03E-11	0.2495	NA	170756	rs9368528	329443	ieu-b-102	TRUE	reported	WYslv6
6	27683924	A	G	-0.0335	0.005	1.73E-11	0.2494	NA	170756	rs1283208	329443	ieu-b-102	TRUE	reported	WYslv6
6	27684631	G	A	-0.0334	0.005	1.97E-11	0.2495	NA	170756	rs9368529	329443	ieu-b-102	TRUE	reported	WYslv6
6	27684654	A	G	-0.0335	0.005	1.70E-11	0.2494	NA	170756	rs9380012	329443	ieu-b-102	TRUE	reported	WYslv6
6	27685348	A	G	-0.0555	0.007	2.04E-15	0.1067	NA	170756	rs3440991	329443	ieu-b-102	TRUE	reported	WYslv6
6	27685825	G	A	-0.0556	0.007	1.93E-15	0.1067	NA	170756	rs3443285	329443	ieu-b-102	TRUE	reported	WYslv6
6	27686991	A	G	-0.0336	0.005	1.61E-11	0.2494	NA	170756	rs1317038	329443	ieu-b-102	TRUE	reported	WYslv6
6	27687035	C	T	-0.0333	0.005	2.09E-11	0.2508	NA	170756	rs9468201	329443	ieu-b-102	TRUE	reported	WYslv6
6	27687973	G	A	-0.0336	0.005	1.59E-11	0.2493	NA	170756	rs9393847	329443	ieu-b-102	TRUE	reported	WYslv6
6	27688369	G	A	-0.0336	0.005	1.44E-11	0.2494	NA	170756	rs9393848	329443	ieu-b-102	TRUE	reported	WYslv6
6	27688625	G	T	-0.0555	0.007	2.07E-15	0.1066	NA	170756	rs3406484	329443	ieu-b-102	TRUE	reported	WYslv6
6	27688667	A	G	-0.0336	0.005	1.42E-11	0.2494	NA	170756	rs9468203	329443	ieu-b-102	TRUE	reported	WYslv6
6	27688809	C	T	-0.0338	0.005	1.21E-11	0.2495	NA	170756	rs9468204	329443	ieu-b-102	TRUE	reported	WYslv6
6	27688841	A	C	-0.0555	0.007	2.04E-15	0.1067	NA	170756	rs1321231	329443	ieu-b-102	TRUE	reported	WYslv6
6	27688927	G	A	-0.0376	0.0055	9.55E-12	0.1874	NA	170756	rs9357045	329443	ieu-b-102	TRUE	reported	WYslv6
6	27688930	C	T	-0.0377	0.0055	9.90E-12	0.1875	NA	170756	rs9348774	329443	ieu-b-102	TRUE	reported	WYslv6
6	27689502	G	A	-0.0371	0.0055	1.24E-11	0.1899	NA	170756	rs9295740	329443	ieu-b-102	TRUE	reported	WYslv6
6	27689939	T	C	-0.036	0.0057	2.85E-10	0.1711	NA	170756	rs2056923	329443	ieu-b-102	TRUE	reported	WYslv6
6	27690174	G	A	-0.0252	0.0045	2.79E-08	0.3397	NA	170756	rs2056924	329443	ieu-b-102	TRUE	reported	WYslv6
6	27690453	G	T	-0.0334	0.005	1.77E-11	0.2507	NA	170756	rs9468206	329443	ieu-b-102	TRUE	reported	WYslv6
6	27690905	A	G	-0.0331	0.005	2.38E-11	0.2543	NA	170756	rs2056925	329443	ieu-b-102	TRUE	reported	WYslv6
6	27693003	A	T	-0.0336	0.0049	1.12E-11	0.2537	NA	170756	rs6922574	329443	ieu-b-102	TRUE	reported	WYslv6
6	27693641	T	A	-0.028	0.0047	2.65E-09	0.3255	NA	170756	rs9842901	329443	ieu-b-102	TRUE	reported	WYslv6
6	27695329	C	T	-0.0337	0.0049	9.93E-12	0.2537	NA	170756	rs9348775	329443	ieu-b-102	TRUE	reported	WYslv6
6	27696951	C	T	-0.0341	0.0049	5.02E-12	0.2542	NA	170756	rs9366700	329443	ieu-b-102	TRUE	reported	WYslv6
6	27698355	A	G	-0.0342	0.0049	4.80E-12	0.2543	NA	170756	rs6456802	329443	ieu-b-102	TRUE	reported	WYslv6
6	27698837	C	T	-0.0573	0.0072	1.11E-15	0.1029	NA	170756	rs1320229	329443	ieu-b-102	TRUE	reported	WYslv6
6	27698857	A	G	-0.0554	0.007	2.08E-15	0.1067	NA	170756	rs1320229	329443	ieu-b-102	TRUE	reported	WYslv6
6	27699581	A	T	-0.0341	0.0049	4.99E-12	0.2543	NA	170756	rs9393851	329443	ieu-b-102	TRUE	reported	WYslv6
6	27700559	A	G	-0.0341	0.0049	5.14E-12	0.2543	NA	170756	rs9461400	329443	ieu-b-102	TRUE	reported	WYslv6
6	27701122	T	C	-0.0555	0.007	2.06E-15	0.1067	NA	170756	rs1469544	329443	ieu-b-102	TRUE	reported	WYslv6
6	27702425	G	T	-0.0555	0.007	2.07E-15	0.1066	NA	170756	rs1319354	329443	ieu-b-102	TRUE	reported	WYslv6
6	27702440	A	C	-0.0341	0.0049	5.35E-12	0.2542	NA	170756	rs1021444	329443	ieu-b-102	TRUE	reported	WYslv6
6	27702561	A	G	-0.0555	0.007	2.09E-15	0.1067	NA	170756	rs1319348	329443	ieu-b-102	TRUE	reported	WYslv6
6	27702832	G	A	-0.0342	0.0049	4.87E-12	0.2549	NA	170756	rs9295742	329443	ieu-b-102	TRUE	reported	WYslv6
6	27703291	G	A	-0.0342	0.0049	4.91E-12	0.2553	NA	170756	rs9461401	329443	ieu-b-102	TRUE	reported	WYslv6
6	27706180	G	A	-0.0258	0.0045	1.38E-08	0.3391	NA	170756	rs8888902	329443	ieu-b-102	TRUE	reported	WYslv6
6	27708732	A	G	-0.0378	0.0057	3.79E-11	0.1706	NA	170756	rs6940116	329443	ieu-b-102	TRUE	reported	WYslv6
6	27709441	C	T	-0.034	0.0049	5.71E-12	0.2546	NA	170756	rs6902689	329443	ieu-b-102	TRUE	reported	WYslv6
6	27710165	A	C	-0.0559	0.0068	2.31E-16	0.1128	NA	170756	rs1769396	329443	ieu-b-102	TRUE	reported	WYslv6
6	27711530	C	T	-0.0341	0.0049	5.13E-12	0.2548	NA	170756	rs6914924	329443	ieu-b-102	TRUE	reported	WYslv6
6	27712120	A	C	-0.034	0.0049	5.63E-12	0.2546	NA	170756	rs6930992	329443	ieu-b-102	TRUE	reported	WYslv6
6	27713299	G	C	-0.0342	0.0049	4.69E-12	0.2548	NA	170756	rs9468214	329443	ieu-b-102	TRUE	reported	WYslv6
6	27714575	G	T	-0.0342	0.0049	4.54E-12	0.255	NA	170756	rs6901520	329443	ieu-b-102	TRUE	reported	WYslv6
6	27715102	G	C	-0.0342	0.0049	4.27E-12	0.2555	NA	170756	rs9295743	329443	ieu-b-102	TRUE	reported	WYslv6
6	27715243	A	C	-0.0341	0.005	7.97E-12	0.2523	NA	170756	rs9283880	329443	ieu-b-102	TRUE	reported	WYslv6
6	27715255	A	T	-0.0341	0.005	8.59E-12	0.2523	NA	170756	rs9283881	329443	ieu-b-102	TRUE	reported	WYslv6
6	27715258	A	C	-0.0341	0.005	8.07E-12	0.2523	NA	170756	rs9283882	329443	ieu-b-102	TRUE	reported	WYslv6
6	27715470	T	A	-0.0338	0.0049	7.62E-12	0.2541	NA	170756	rs9283883	329443	ieu-b-102	TRUE	reported	WYslv6
6	27718109	A	G	-0.0341	0.0049	5.54E-12	0.2547	NA	170756	rs7760523	329443	ieu-b-102	TRUE	reported	WYslv6
6	27718272	T	C	-0.0341	0.0049	5.18E-12	0.2547	NA	170756	rs3595740	329443	ieu-b-102	TRUE	reported	WYslv6
6	27719375	G	T	-0.0341	0.0049	4.89E-12	0.2548	NA	170756	rs9461405	329443	ieu-b-102	TRUE	reported	WYslv6
6	27719764	T	C	-0.0334	0.005	3.44E-11	0.2404	NA	170756	rs9461406	329443	ieu-b-102	TRUE	reported	WYslv6
6	27720249	C	A	-0.0271	0.0046	3.00E-09	0.3309	NA	170756	rs5725218	329443	ieu-b-102	TRUE	reported	WYslv6
6	27720712	C	G	-0.0289	0.0048	1.88E-09	0.3134	NA	170756	rs3604229	329443	ieu-b-102	TRUE	reported	WYslv6
6	27722064	T	C	-0.0333	0.005	2.76E-11	0.2449	NA	170756	rs4713121	329443	ieu-b-102	TRUE	reported	WYslv6
6	27726467	G	A	-0.0336	0.005	2.40E-11	0.2406	NA	170756	rs9468217	329443	ieu-b-102	TRUE	reported	WYslv6
6	27726731	C	T	-0.0346	0.0049	2.57E-12	0.254	NA	170756	rs7776351	329443	ieu-b-102	TRUE	reported	WYslv6
6	27727513	G	A	-0.0331	0.005	3.54E-11	0.245	NA	170756	rs4711163	329443	ieu-b-102	TRUE	reported	WYslv6
6	27728827	T	C	-0.0343	0.0049	4.22E-12	0.2541	NA	170756	rs7773070	329443	ieu-b-102	TRUE	reported	WYslv6
6	27730064	T	C	-0.0337	0.005	2.21E-11	0.2406	NA	170756	rs9295746	329443	ieu-b-102	TRUE	reported	WYslv6
6	27730082	C	T	-0.0554	0.007	2.40E-15	0.1067	NA	170756	rs1358767	329443	ieu-b-102	TRUE	reported	WYslv6
6	27730334	T	C	-0.0553	0.007	2.68E-15	0.1068	NA	170756	rs1419035	329443	ieu-b-102	TRUE	reported	WYslv6
6	27730463	A	G	-0.0342	0.005	7.17E-12	0.2486	NA	170756	rs7759217	329443	ieu-b-102	TRUE	reported	WYslv6
6	27731755	C	A	-0.0343	0.005	6.17E-12	0.2483	NA	170756	rs1381715	329443	ieu-b-102	TRUE	reported	WYslv6
6	27732976	C	T	-0.0336	0.0051	3.54E-11	0.2342	NA	170756	rs9468220	329443	ieu-b-102	TRUE	reported	WYslv6
6	27734470	T	C	-0.0339	0.005	1.07E-11	0.2488	NA	170756	rs9366702	329443	ieu-b-102	TRUE	reported	WYslv6
6	27734954	C	G	-0.0332	0.0051	6.04E-11	0.2343	NA	170756	rs7756968	329443	ieu-b-102	TRUE	reported	WYslv6
6	27736993	T	G	-0.0274	0.0046	2.13E-09	0.3308	NA	170756	rs9295747	329443	ieu-b-102	TRUE	reported	WYslv6
6	27737605	A	G	-0.0338	0.005	1.19E-11	0.2478	NA	170756	rs9368530	329443	ieu-b-102	TRUE	reported	WYslv6
6	27738430	C	A	-0.0323	0.0051	2.77E-10	0.235	NA	170756	rs1999376	329443	ieu-b-102	TRUE	reported	WYslv6
6	27738732	A	T	-0.0331	0.0051	6.97E-11	0.2341	NA	170756	rs2893929	329443	ieu-b-102	TRUE	reported	WYslv6
6	27738801	G	A	-0.0334	0.0051	4.93E-11	0.2341	NA	170756	rs4140646	329443	ieu-b-102	TRUE	reported	WYslv6
6	27738806	T	C	-0.0334	0.0051	4.95E-11	0.2341	NA	170756	rs2893930	329443	ieu-b-102	TRUE	reported	WYslv6

6	27739254	T	C	-0.0337	0.005	1.31E-11	0.2478	NA	170756	rs742046	329443	ieu-b-102	TRUE	reported	WYslv6
6	27739380	A	G	-0.033	0.0051	8.11E-11	0.2343	NA	170756	rs742047	329443	ieu-b-102	TRUE	reported	WYslv6
6	27739566	T	A	-0.0553	0.007	2.43E-15	0.1068	NA	170756	rs3550103	329443	ieu-b-102	TRUE	reported	WYslv6
6	27740300	C	A	-0.0331	0.0051	7.40E-11	0.234	NA	170756	rs760587	329443	ieu-b-102	TRUE	reported	WYslv6
6	27740666	C	T	-0.0336	0.005	1.62E-11	0.2474	NA	170756	rs9468223	329443	ieu-b-102	TRUE	reported	WYslv6
6	27741825	T	C	-0.0336	0.005	1.58E-11	0.2481	NA	170756	rs2179094	329443	ieu-b-102	TRUE	reported	WYslv6
6	27742035	A	G	-0.0338	0.005	1.22E-11	0.2476	NA	170756	rs1985099	329443	ieu-b-102	TRUE	reported	WYslv6
6	27745719	T	G	-0.0334	0.005	2.44E-11	0.2468	NA	170756	rs9468225	329443	ieu-b-102	TRUE	reported	WYslv6
6	27745738	G	A	-0.0326	0.005	6.46E-11	0.2502	NA	170756	rs9461407	329443	ieu-b-102	TRUE	reported	WYslv6
6	27746590	A	T	-0.0328	0.0051	1.11E-10	0.2345	NA	170756	rs1048440	329443	ieu-b-102	TRUE	reported	WYslv6
6	27746692	T	C	-0.0327	0.0051	1.17E-10	0.2345	NA	170756	rs3506990	329443	ieu-b-102	TRUE	reported	WYslv6
6	27748010	G	A	-0.0333	0.005	2.37E-11	0.2478	NA	170756	rs2893931	329443	ieu-b-102	TRUE	reported	WYslv6
6	27748015	G	A	-0.033	0.005	3.77E-11	0.2484	NA	170756	rs2394002	329443	ieu-b-102	TRUE	reported	WYslv6
6	27749330	G	C	-0.0335	0.005	1.80E-11	0.2481	NA	170756	rs2092075	329443	ieu-b-102	TRUE	reported	WYslv6
6	27750079	T	C	-0.0334	0.005	1.95E-11	0.248	NA	170756	rs1321170	329443	ieu-b-102	TRUE	reported	WYslv6
6	27750086	G	A	-0.0335	0.005	1.92E-11	0.248	NA	170756	rs9468229	329443	ieu-b-102	TRUE	reported	WYslv6
6	27750858	C	T	-0.0334	0.005	2.15E-11	0.248	NA	170756	rs1273858	329443	ieu-b-102	TRUE	reported	WYslv6
6	27757654	T	A	-0.0337	0.005	1.58E-11	0.2473	NA	170756	rs200465	329443	ieu-b-102	TRUE	reported	WYslv6
6	27757855	C	G	-0.038	0.0055	5.80E-12	0.1878	NA	170756	rs200468	329443	ieu-b-102	TRUE	reported	WYslv6
6	27759115	G	C	-0.0563	0.0071	1.98E-15	0.1043	NA	170756	rs3503786	329443	ieu-b-102	TRUE	reported	WYslv6
6	27767640	G	A	-0.0376	0.0055	9.83E-12	0.1877	NA	170756	rs200474	329443	ieu-b-102	TRUE	reported	WYslv6
6	27768348	A	T	-0.0378	0.0055	7.00E-12	0.1881	NA	170756	rs200476	329443	ieu-b-102	TRUE	reported	WYslv6
6	27770890	C	T	-0.0379	0.0055	5.75E-12	0.1881	NA	170756	rs169262	329443	ieu-b-102	TRUE	reported	WYslv6
6	27771106	T	C	-0.0553	0.007	2.38E-15	0.1072	NA	170756	rs1319572	329443	ieu-b-102	TRUE	reported	WYslv6
6	27773832	T	A	-0.0543	0.0065	6.19E-17	0.1261	NA	170756	rs200481	329443	ieu-b-102	TRUE	reported	WYslv6
6	27773904	G	A	-0.0543	0.0065	6.26E-17	0.1261	NA	170756	rs200482	329443	ieu-b-102	TRUE	reported	WYslv6
6	27774824	G	A	-0.0547	0.0065	4.54E-17	0.1254	NA	170756	rs200483	329443	ieu-b-102	TRUE	reported	WYslv6
6	27775028	C	T	-0.0555	0.007	1.94E-15	0.1068	NA	170756	rs1775118	329443	ieu-b-102	TRUE	reported	WYslv6
6	27775674	A	G	-0.0549	0.0065	2.85E-17	0.1264	NA	170756	rs200484	329443	ieu-b-102	TRUE	reported	WYslv6
6	27775697	G	C	-0.0542	0.0065	7.55E-17	0.1261	NA	170756	rs200485	329443	ieu-b-102	TRUE	reported	WYslv6
6	27779506	C	T	-0.0378	0.0055	4.85E-12	0.1914	NA	170756	rs200486	329443	ieu-b-102	TRUE	reported	WYslv6
6	27781872	G	C	-0.0543	0.0065	7.10E-17	0.1254	NA	170756	rs9368531	329443	ieu-b-102	TRUE	reported	WYslv6
6	27782031	T	G	-0.0542	0.0065	7.48E-17	0.1262	NA	170756	rs370155	329443	ieu-b-102	TRUE	reported	WYslv6
6	27782528	T	C	-0.0562	0.0068	1.03E-16	0.1203	NA	170756	rs401763	329443	ieu-b-102	TRUE	reported	WYslv6
6	27782535	C	T	-0.0554	0.0067	1.66E-16	0.1214	NA	170756	rs390764	329443	ieu-b-102	TRUE	reported	WYslv6
6	27782538	T	A	-0.0559	0.0067	1.22E-16	0.1204	NA	170756	rs401754	329443	ieu-b-102	TRUE	reported	WYslv6
6	27783359	A	G	-0.0543	0.0065	7.22E-17	0.1254	NA	170756	rs2747054	329443	ieu-b-102	TRUE	reported	WYslv6
6	27783941	C	T	-0.0556	0.007	1.82E-15	0.1068	NA	170756	rs3471892	329443	ieu-b-102	TRUE	reported	WYslv6
6	27785820	T	C	-0.0378	0.0055	4.93E-12	0.1906	NA	170756	rs200504	329443	ieu-b-102	TRUE	reported	WYslv6
6	27785882	A	G	-0.0336	0.005	1.33E-11	0.2511	NA	170756	rs200503	329443	ieu-b-102	TRUE	reported	WYslv6
6	27788062	C	T	-0.0338	0.005	9.66E-12	0.2514	NA	170756	rs200502	329443	ieu-b-102	TRUE	reported	WYslv6
6	27788942	C	T	-0.0541	0.0065	8.91E-17	0.1257	NA	170756	rs200501	329443	ieu-b-102	TRUE	reported	WYslv6
6	27792640	C	T	-0.0542	0.0065	8.22E-17	0.1253	NA	170756	rs200497	329443	ieu-b-102	TRUE	reported	WYslv6
6	27795418	G	A	-0.0527	0.0065	5.24E-16	0.1269	NA	170756	rs200493	329443	ieu-b-102	TRUE	reported	WYslv6
6	27796935	G	T	-0.054	0.0065	1.16E-16	0.1255	NA	170756	rs200490	329443	ieu-b-102	TRUE	reported	WYslv6
6	27797416	C	A	-0.0554	0.007	2.26E-15	0.1068	NA	170756	rs7155905	329443	ieu-b-102	TRUE	reported	WYslv6
6	27798257	T	C	-0.0542	0.0065	8.98E-17	0.1253	NA	170756	rs200489	329443	ieu-b-102	TRUE	reported	WYslv6
6	27798887	C	G	-0.0555	0.007	2.07E-15	0.1068	NA	170756	rs1162734	329443	ieu-b-102	TRUE	reported	WYslv6
6	27799514	C	T	-0.0591	0.0075	2.76E-15	0.0986	NA	170756	rs3520226	329443	ieu-b-102	TRUE	reported	WYslv6
6	27804934	T	C	-0.0377	0.0055	5.27E-12	0.191	NA	170756	rs201004	329443	ieu-b-102	TRUE	reported	WYslv6
6	27805255	A	C	-0.0557	0.007	1.68E-15	0.1068	NA	170756	rs3470688	329443	ieu-b-102	TRUE	reported	WYslv6
6	27806985	A	G	-0.0555	0.007	1.82E-15	0.1068	NA	170756	rs1321265	329443	ieu-b-102	TRUE	reported	WYslv6
6	27808192	A	G	-0.0538	0.0065	1.36E-16	0.1258	NA	170756	rs201002	329443	ieu-b-102	TRUE	reported	WYslv6
6	27808899	T	C	-0.0376	0.0055	6.40E-12	0.1908	NA	170756	rs201001	329443	ieu-b-102	TRUE	reported	WYslv6
6	27809159	C	T	-0.0376	0.0055	6.31E-12	0.1908	NA	170756	rs201000	329443	ieu-b-102	TRUE	reported	WYslv6
6	27810569	A	G	-0.0555	0.007	1.80E-15	0.1068	NA	170756	rs3581975	329443	ieu-b-102	TRUE	reported	WYslv6
6	27810626	T	C	-0.0544	0.0065	5.97E-17	0.1255	NA	170756	rs175597	329443	ieu-b-102	TRUE	reported	WYslv6
6	27811815	G	A	-0.034	0.0047	5.83E-13	0.294	NA	170756	rs200997	329443	ieu-b-102	TRUE	reported	WYslv6
6	27811828	G	A	-0.0543	0.0065	7.22E-17	0.1253	NA	170756	rs200996	329443	ieu-b-102	TRUE	reported	WYslv6
6	27813694	T	C	-0.0543	0.0065	7.32E-17	0.1255	NA	170756	rs1468496	329443	ieu-b-102	TRUE	reported	WYslv6
6	27813813	G	T	-0.0353	0.0051	5.73E-12	0.2285	NA	170756	rs200994	329443	ieu-b-102	TRUE	reported	WYslv6
6	27814677	A	G	-0.0556	0.0072	1.00E-14	0.1158	NA	170756	rs200992	329443	ieu-b-102	TRUE	reported	WYslv6
6	27815494	C	A	-0.0423	0.0058	3.87E-13	0.1639	NA	170756	rs200991	329443	ieu-b-102	TRUE	reported	WYslv6
6	27815639	A	G	-0.0557	0.007	1.71E-15	0.1068	NA	170756	rs1319478	329443	ieu-b-102	TRUE	reported	WYslv6
6	27815823	T	G	-0.0545	0.0065	5.59E-17	0.1255	NA	170756	rs200990	329443	ieu-b-102	TRUE	reported	WYslv6
6	27816442	A	G	-0.0546	0.0065	5.20E-17	0.1254	NA	170756	rs200989	329443	ieu-b-102	TRUE	reported	WYslv6
6	27818482	C	G	-0.0556	0.007	1.78E-15	0.1068	NA	170756	rs3611676	329443	ieu-b-102	TRUE	reported	WYslv6
6	27818535	A	G	-0.0556	0.007	1.78E-15	0.1068	NA	170756	rs1394682	329443	ieu-b-102	TRUE	reported	WYslv6
6	27824766	G	C	-0.0387	0.0051	2.68E-14	0.2522	NA	170756	rs200986	329443	ieu-b-102	TRUE	reported	WYslv6
6	27828151	T	A	-0.0547	0.0065	4.29E-17	0.1253	NA	170756	rs3429513	329443	ieu-b-102	TRUE	reported	WYslv6
6	27830326	C	T	-0.0547	0.0065	4.34E-17	0.1253	NA	170756	rs200983	329443	ieu-b-102	TRUE	reported	WYslv6
6	27833174	A	G	-0.0544	0.0065	5.97E-17	0.1254	NA	170756	rs200981	329443	ieu-b-102	TRUE	reported	WYslv6
6	27834085	A	G	-0.0556	0.007	1.90E-15	0.1068	NA	170756	rs1027949	329443	ieu-b-102	TRUE	reported	WYslv6
6	27834139	C	G	-0.0556	0.007	1.94E-15	0.1067	NA	170756	rs1319990	329443	ieu-b-102	TRUE	reported	WYslv6
6	27835218	G	A	-0.0555	0.007	1.61E-15	0.1071	NA	170756	rs1776308	329443	ieu-b-102	TRUE	reported	WYslv6
6	27835272	T	C	-0.0545	0.0065	5.52E-17	0.1253	NA	170756	rs200948	329443	ieu-b-102	TRUE	reported	WYslv6
6	27835435	A	G	-0.0538	0.0064	3.61E-17	0.1319	NA	170756	rs200949	329443	ieu-b-102	TRUE	reported	WYslv6
6	27835772	A	G	-0.0546	0.0065	5.24E-17	0.1253	NA	170756	rs200950	329443	ieu-b-102	TRUE	reported	WYslv6
6	27835930	A	G	-0.0358	0.0049	1.98E-13	0.2662	NA	170756	rs200951	329443	ieu-b-102	TRUE	reported	WYslv6
6	27836976	C	T	-0.0546	0.0065	4.99E-17	0.1253	NA	170756	rs200952	329443	ieu-b-102	TRUE	reported	WYslv6
6	27837183	T	C	-0.0556	0.007	1.77E-15	0.1067	NA	170756	rs1769575	329443	ieu-b-102	TRUE	reported	WYslv6
6	27837267	T	C	-0.0545	0.0065	5.65E-17	0.1254	NA	170756	rs200953	329443	ieu-b-102	TRUE	reported	WYslv6
6	27838764	C	G	-0.0547	0.0065	4.37E-17	0.1253	NA	170756	rs200954	329443	ieu-b-102	TRUE	reported	WYslv6
6	27839746	T	C	-0.0424	0.0057	1.58E-13	0.1694	NA	170756	rs200956	329443	ieu-b-102	TRUE	reported	WYslv6
6	27840926	T	C	-0.0571	0.0071	9.24E-16	0.1029	NA	170756	rs4550959	329443	ieu-b-102	TRUE	reported	WYslv6
6	27843742	A	G	-0.0364	0.0055	3.83E-11	0.2529	NA	170756	rs9468248	329443	ieu-b-102	TRUE	reported	WYslv6
6	27845129	C	T	-0.0557	0.0066	3.40E-17	0.1215	NA	170756	rs557042	329443	ieu-b-102	TRUE	reported	WYslv6
6	27846744	G	C	-0.0552	0.0066	5.81E-17	0.1218	NA	170756	rs483143	329443	ieu-b-102	TRUE	reported	WYslv6
6	27847517	G	A	-0.0356	0.0049	3.75E-13	0.261	NA	170756	rs510987	329443	ieu-b-102	TRUE	reported	WYslv6
6	27849676	C	A	-0.0357	0.0052	5.36E-12	0.2268	NA	170756	rs573179	329443	ieu-b-102	TRUE	reported	WYslv6
6	27850714	A	T	-0.0557	0.0066	3.22E-17	0.1215	NA	170756	rs493161	329443	ieu-b-102	TRUE	reported	WYslv6
6	27850873	G	A	-0.0431	0.0056	1.18E-14	0.182	NA	170756	rs494887	329443	ieu-b-102	TRUE	reported	WYslv6
6	27852011	C	T	-0.0432	0.0056	1.56E-14	0.1817	NA	170756	rs200980	329443	ieu-b-102	TRUE	reported	WYslv6
6	27852357	G	A	-0.0432	0.0056	1.14E-14	0.182	NA	170756	rs200979	329443	ieu-b-102	TRUE	reported	WYslv6
6	27853168	C	G	-0.0365	0.0052	1.43E-12	0.2257	NA	170756	rs200978	329443	ieu-b-102	TRUE	reported	WYslv6
6	27854301	T	C	-0.0556	0.0066	3.53E-17	0.1215	NA	170756	rs200977	329443	ieu-b-102	TRUE	reported	WYslv6
6	27854760	C	A	-0.0431	0.0056	1.29E-14	0.1821	NA	170756	rs169287	329443	ieu-b-102	TRUE	reported	WYslv6
6	27854963	A	G	-0.0434	0.0056	8.80E-15	0.1814	NA	170756	rs200976	329443	ieu-b-102	TRUE	reported	WYslv6
6	27855625	G	T	-0.043	0.0056	1.45E-14	0.182	NA	170756	rs200975	329443	ieu-b-102	TRUE	reported	WYslv6
6	27855845	A	G	-0.0431	0.0056	1.26E-14	0.182	NA	170756	rs200974	329443	ieu-b-102	TRUE	reported	WYslv6
6	27858421	A	G	-0.0433	0.0056	9.39E-15	0.1822	NA	170756	rs200973	329443	ieu-b-102	TRUE	reported	WYslv6
6	27858904	C	T	-0.0357	0.0049	3.08E-13	0.2622	NA	170756	rs200971	329443	ieu-b-102	TRUE	reported	WYslv6
6	27859453	T	C	-0.0364	0.0052	1.64E-12	0.2257	NA	170756	rs200969	329443	ieu-b-102	TRUE	reported	WYslv6
6	27859568	T	C	-0.043	0.0056	1.35E-14	0.182	NA	170756	rs200968	329443	ieu-b-102	TRUE	reported	WYslv6
6	27862127	A	G	-0.0358	0.0049	2.98E-13	0.2612	NA	170756	rs200967	329443	ieu-b-102	TRUE	reported	WYslv6
6	27862152	T	C	-0.043	0.0056	1.36E-14	0.1821	NA	170756	rs200966	329443	ieu-b-102	TRUE	reported	WYslv6
6	27865902	A	T	-0.0571	0.0071	9.88E-16	0.1029	NA	170756	rs6686808	329443	ieu-b-102	TRUE	reported	WYslv6

6	27866384	G	A	-0.0434	0.0056	9.32E-15	0.1819	NA	170756	rs200965	329443	ieu-b-102	TRUE	reported	WYslv6
6	27866943	G	C	-0.0435	0.0056	8.09E-15	0.182	NA	170756	rs1398299	329443	ieu-b-102	TRUE	reported	WYslv6
6	27868792	C	T	-0.0579	0.0071	2.65E-16	0.1045	NA	170756	rs1319964	329443	ieu-b-102	TRUE	reported	WYslv6
6	27877446	T	A	-0.0504	0.0063	1.06E-15	0.1372	NA	170756	rs188015	329443	ieu-b-102	TRUE	reported	WYslv6
6	27877985	A	G	-0.0347	0.005	5.24E-12	0.2444	NA	170756	rs149855	329443	ieu-b-102	TRUE	reported	WYslv6
6	27879200	C	A	-0.0562	0.007	1.55E-15	0.1049	NA	170756	rs3478897	329443	ieu-b-102	TRUE	reported	WYslv6
6	27879982	A	G	-0.0568	0.007	6.58E-16	0.1053	NA	170756	rs6174209	329443	ieu-b-102	TRUE	reported	WYslv6
6	27884012	C	T	-0.0567	0.007	5.83E-16	0.1061	NA	170756	rs1321887	329443	ieu-b-102	TRUE	reported	WYslv6
6	27896799	A	C	-0.0583	0.0071	1.35E-16	0.1048	NA	170756	rs7155905	329443	ieu-b-102	TRUE	reported	WYslv6
6	27905509	T	C	-0.0584	0.0071	1.16E-16	0.1046	NA	170756	rs6704072	329443	ieu-b-102	TRUE	reported	WYslv6
6	27912437	G	A	-0.0594	0.0071	6.17E-17	0.1042	NA	170756	rs1495830	329443	ieu-b-102	TRUE	reported	WYslv6
6	27932301	G	A	-0.0585	0.0071	1.09E-16	0.1047	NA	170756	rs6766211	329443	ieu-b-102	TRUE	reported	WYslv6
6	27938484	A	G	-0.0585	0.0071	1.15E-16	0.1046	NA	170756	rs1321611	329443	ieu-b-102	TRUE	reported	WYslv6
6	27943369	C	T	-0.0585	0.0071	1.06E-16	0.1047	NA	170756	rs3610135	329443	ieu-b-102	TRUE	reported	WYslv6
6	27945396	G	A	-0.0585	0.0071	1.09E-16	0.1047	NA	170756	rs2836049	329443	ieu-b-102	TRUE	reported	WYslv6
6	27965503	C	T	-0.0356	0.005	1.22E-12	0.2449	NA	170756	rs1193944	329443	ieu-b-102	TRUE	reported	WYslv6
6	27967089	T	C	-0.0508	0.0063	5.91E-16	0.1373	NA	170756	rs156743	329443	ieu-b-102	TRUE	reported	WYslv6
6	27967274	G	A	-0.0351	0.0051	4.40E-12	0.2378	NA	170756	rs156744	329443	ieu-b-102	TRUE	reported	WYslv6
6	27967591	T	A	-0.035	0.005	3.02E-12	0.245	NA	170756	rs464312	329443	ieu-b-102	TRUE	reported	WYslv6
6	27970031	G	T	-0.0349	0.0051	5.45E-12	0.2378	NA	170756	rs149946	329443	ieu-b-102	TRUE	reported	WYslv6
6	27970704	A	G	-0.0348	0.005	5.21E-12	0.2436	NA	170756	rs469228	329443	ieu-b-102	TRUE	reported	WYslv6
6	27970705	A	C	-0.0347	0.005	5.53E-12	0.2436	NA	170756	rs3417174	329443	ieu-b-102	TRUE	reported	WYslv6
6	27970715	T	C	-0.0589	0.0071	1.52E-16	0.1034	NA	170756	rs7153757	329443	ieu-b-102	TRUE	reported	WYslv6
6	27972433	A	T	-0.0351	0.0051	4.12E-12	0.2379	NA	170756	rs149947	329443	ieu-b-102	TRUE	reported	WYslv6
6	27974817	C	G	-0.0352	0.005	2.32E-12	0.2452	NA	170756	rs149948	329443	ieu-b-102	TRUE	reported	WYslv6
6	27977495	T	C	-0.0353	0.0051	3.13E-12	0.2381	NA	170756	rs149968	329443	ieu-b-102	TRUE	reported	WYslv6
6	27977737	T	C	-0.0352	0.0051	3.60E-12	0.2381	NA	170756	rs149969	329443	ieu-b-102	TRUE	reported	WYslv6
6	27980220	G	A	-0.0351	0.005	2.41E-12	0.2452	NA	170756	rs149970	329443	ieu-b-102	TRUE	reported	WYslv6
6	27983227	C	T	-0.0325	0.0048	8.10E-12	0.288	NA	170756	rs149972	329443	ieu-b-102	TRUE	reported	WYslv6
6	27986340	G	A	-0.0325	0.0048	7.66E-12	0.288	NA	170756	rs149975	329443	ieu-b-102	TRUE	reported	WYslv6
6	27989174	A	G	-0.0325	0.0048	1.24E-11	0.2863	NA	170756	rs149977	329443	ieu-b-102	TRUE	reported	WYslv6
6	27989252	C	T	-0.0585	0.0071	1.31E-16	0.1047	NA	170756	rs1429653	329443	ieu-b-102	TRUE	reported	WYslv6
6	27989370	T	C	-0.0323	0.0048	1.32E-11	0.2879	NA	170756	rs149978	329443	ieu-b-102	TRUE	reported	WYslv6
6	27991007	G	A	-0.0355	0.0051	2.32E-12	0.2384	NA	170756	rs9351736	329443	ieu-b-102	TRUE	reported	WYslv6
6	27992883	C	T	-0.0328	0.0048	1.01E-11	0.2845	NA	170756	rs149982	329443	ieu-b-102	TRUE	reported	WYslv6
6	27992898	G	A	-0.058	0.0071	2.21E-16	0.1049	NA	170756	rs1382344	329443	ieu-b-102	TRUE	reported	WYslv6
6	27994416	A	T	-0.0583	0.0071	1.30E-16	0.1049	NA	170756	rs7155906	329443	ieu-b-102	TRUE	reported	WYslv6
6	27998184	T	A	-0.0324	0.0048	8.89E-12	0.288	NA	170756	rs149989	329443	ieu-b-102	TRUE	reported	WYslv6
6	28001033	T	C	-0.0324	0.0048	9.37E-12	0.288	NA	170756	rs149941	329443	ieu-b-102	TRUE	reported	WYslv6
6	28001610	T	C	-0.0324	0.0048	9.29E-12	0.288	NA	170756	rs149942	329443	ieu-b-102	TRUE	reported	WYslv6
6	28002388	G	A	-0.0511	0.0063	3.42E-16	0.1376	NA	170756	rs149943	329443	ieu-b-102	TRUE	reported	WYslv6
6	28002818	C	T	-0.0324	0.0048	9.46E-12	0.288	NA	170756	rs149944	329443	ieu-b-102	TRUE	reported	WYslv6
6	28002853	T	C	-0.0324	0.0048	9.38E-12	0.288	NA	170756	rs149945	329443	ieu-b-102	TRUE	reported	WYslv6
6	28004059	T	C	-0.036	0.0051	1.57E-12	0.2362	NA	170756	rs156736	329443	ieu-b-102	TRUE	reported	WYslv6
6	28006650	A	G	-0.0322	0.0048	1.29E-11	0.2875	NA	170756	rs149897	329443	ieu-b-102	TRUE	reported	WYslv6
6	28007357	A	C	-0.0323	0.0048	1.19E-11	0.2875	NA	170756	rs156734	329443	ieu-b-102	TRUE	reported	WYslv6
6	28010078	G	C	-0.0323	0.0048	1.43E-11	0.2857	NA	170756	rs169431	329443	ieu-b-102	TRUE	reported	WYslv6
6	28011516	T	C	-0.0508	0.0063	4.68E-16	0.1384	NA	170756	rs149949	329443	ieu-b-102	TRUE	reported	WYslv6
6	28011551	C	G	-0.0319	0.0048	2.31E-11	0.2883	NA	170756	rs202908	329443	ieu-b-102	TRUE	reported	WYslv6
6	28011585	A	G	-0.0349	0.0051	5.21E-12	0.2386	NA	170756	rs175954	329443	ieu-b-102	TRUE	reported	WYslv6
6	28013617	A	C	-0.0324	0.0048	9.86E-12	0.2875	NA	170756	rs149958	329443	ieu-b-102	TRUE	reported	WYslv6
6	28013854	T	C	-0.0321	0.0048	1.72E-11	0.2873	NA	170756	rs149959	329443	ieu-b-102	TRUE	reported	WYslv6
6	28014025	G	C	-0.0323	0.0048	1.27E-11	0.2875	NA	170756	rs156738	329443	ieu-b-102	TRUE	reported	WYslv6
6	28014597	C	T	-0.0375	0.0051	1.91E-13	0.2342	NA	170756	rs149900	329443	ieu-b-102	TRUE	reported	WYslv6
6	28015569	A	C	-0.0357	0.0051	1.87E-12	0.238	NA	170756	rs149961	329443	ieu-b-102	TRUE	reported	WYslv6
6	28015918	G	A	-0.0322	0.0048	1.42E-11	0.2875	NA	170756	rs149962	329443	ieu-b-102	TRUE	reported	WYslv6
6	28017250	C	T	-0.0581	0.0071	1.70E-16	0.1048	NA	170756	rs1320021	329443	ieu-b-102	TRUE	reported	WYslv6
6	28018689	T	A	-0.0321	0.0047	1.13E-11	0.289	NA	170756	rs1288590	329443	ieu-b-102	TRUE	reported	WYslv6
6	28022248	T	C	-0.0363	0.0051	7.57E-13	0.2359	NA	170756	rs203890	329443	ieu-b-102	TRUE	reported	WYslv6
6	28022723	C	A	-0.0325	0.0048	8.87E-12	0.2847	NA	170756	rs1217379	329443	ieu-b-102	TRUE	reported	WYslv6
6	28023008	T	A	-0.036	0.0051	2.72E-12	0.2346	NA	170756	rs6652937	329443	ieu-b-102	TRUE	reported	WYslv6
6	28023300	C	A	-0.036	0.0054	3.71E-11	0.2283	NA	170756	rs1217402	329443	ieu-b-102	TRUE	reported	WYslv6
6	28024486	C	G	-0.0355	0.0051	2.29E-12	0.2381	NA	170756	rs9357060	329443	ieu-b-102	TRUE	reported	WYslv6
6	28024570	A	G	-0.0355	0.0051	2.28E-12	0.238	NA	170756	rs9468271	329443	ieu-b-102	TRUE	reported	WYslv6
6	28025279	A	G	-0.0356	0.0051	2.16E-12	0.2379	NA	170756	rs9380045	329443	ieu-b-102	TRUE	reported	WYslv6
6	28025486	C	T	-0.0355	0.0051	2.20E-12	0.238	NA	170756	rs9348789	329443	ieu-b-102	TRUE	reported	WYslv6
6	28025768	G	A	-0.0358	0.0051	2.24E-12	0.237	NA	170756	rs9468272	329443	ieu-b-102	TRUE	reported	WYslv6
6	28025926	T	C	-0.0507	0.0063	6.08E-16	0.1378	NA	170756	rs3594910	329443	ieu-b-102	TRUE	reported	WYslv6
6	28026077	G	T	-0.0355	0.0051	2.30E-12	0.238	NA	170756	rs9468274	329443	ieu-b-102	TRUE	reported	WYslv6
6	28026136	G	T	-0.0356	0.0051	2.07E-12	0.238	NA	170756	rs1206554	329443	ieu-b-102	TRUE	reported	WYslv6
6	28027688	A	C	-0.0356	0.0051	2.12E-12	0.238	NA	170756	rs9468276	329443	ieu-b-102	TRUE	reported	WYslv6
6	28028067	A	G	-0.032	0.0048	1.96E-11	0.2873	NA	170756	rs9368547	329443	ieu-b-102	TRUE	reported	WYslv6
6	28028390	C	A	-0.0355	0.0051	2.23E-12	0.238	NA	170756	rs9468277	329443	ieu-b-102	TRUE	reported	WYslv6
6	28028482	C	T	-0.0355	0.0051	2.26E-12	0.238	NA	170756	rs9468278	329443	ieu-b-102	TRUE	reported	WYslv6
6	28029837	T	C	-0.0355	0.0051	2.30E-12	0.238	NA	170756	rs1321843	329443	ieu-b-102	TRUE	reported	WYslv6
6	28030499	T	C	-0.0352	0.0051	3.45E-12	0.2378	NA	170756	rs9380046	329443	ieu-b-102	TRUE	reported	WYslv6
6	28031838	C	A	-0.032	0.0048	1.71E-11	0.2874	NA	170756	rs183244	329443	ieu-b-102	TRUE	reported	WYslv6
6	28033039	T	G	-0.0319	0.0048	1.88E-11	0.2874	NA	170756	rs149950	329443	ieu-b-102	TRUE	reported	WYslv6
6	28033087	T	C	-0.032	0.0048	1.78E-11	0.2874	NA	170756	rs149951	329443	ieu-b-102	TRUE	reported	WYslv6
6	28033174	A	G	-0.0353	0.0051	5.60E-12	0.2301	NA	170756	rs9295755	329443	ieu-b-102	TRUE	reported	WYslv6
6	28033396	C	A	-0.0351	0.0051	6.86E-12	0.2302	NA	170756	rs9295756	329443	ieu-b-102	TRUE	reported	WYslv6
6	28033523	A	G	-0.0352	0.0051	6.39E-12	0.2302	NA	170756	rs1048440	329443	ieu-b-102	TRUE	reported	WYslv6
6	28034035	A	G	-0.0351	0.0051	7.23E-12	0.2298	NA	170756	rs4713132	329443	ieu-b-102	TRUE	reported	WYslv6
6	28034041	T	C	-0.0351	0.0051	7.75E-12	0.2298	NA	170756	rs4713133	329443	ieu-b-102	TRUE	reported	WYslv6
6	28034121	T	C	-0.0351	0.0051	6.75E-12	0.2302	NA	170756	rs4713134	329443	ieu-b-102	TRUE	reported	WYslv6
6	28034737	A	G	-0.0351	0.0051	7.15E-12	0.2302	NA	170756	rs9368548	329443	ieu-b-102	TRUE	reported	WYslv6
6	28035246	G	A	-0.0319	0.0048	2.29E-11	0.2872	NA	170756	rs149954	329443	ieu-b-102	TRUE	reported	WYslv6
6	28036225	G	C	-0.0318	0.0048	2.59E-11	0.2874	NA	170756	rs149955	329443	ieu-b-102	TRUE	reported	WYslv6
6	28037032	T	A	-0.0344	0.0051	1.24E-11	0.2358	NA	170756	rs6147114	329443	ieu-b-102	TRUE	reported	WYslv6
6	28037893	G	T	-0.0354	0.0051	3.92E-12	0.2327	NA	170756	rs9380047	329443	ieu-b-102	TRUE	reported	WYslv6
6	28038929	G	A	-0.059	0.007	5.90E-17	0.1048	NA	170756	rs7155907	329443	ieu-b-102	TRUE	reported	WYslv6
6	28039015	T	C	-0.0355	0.0051	3.21E-12	0.2328	NA	170756	rs7755442	329443	ieu-b-102	TRUE	reported	WYslv6
6	28039586	G	A	-0.036	0.0051	2.08E-12	0.2296	NA	170756	rs4713135	329443	ieu-b-102	TRUE	reported	WYslv6
6	28042465	C	G	-0.0353	0.0051	4.51E-12	0.2317	NA	170756	rs1319413	329443	ieu-b-102	TRUE	reported	WYslv6
6	28043828	A	G	-0.0471	0.0062	2.21E-14	0.1425	NA	170756	rs3471208	329443	ieu-b-102	TRUE	reported	WYslv6
6	28044337	T	C	-0.0355	0.0051	3.52E-12	0.2325	NA	170756	rs5574792	329443	ieu-b-102	TRUE	reported	WYslv6
6	28044482	A	G	-0.0355	0.0051	3.52E-12	0.2325	NA	170756	rs5631087	329443	ieu-b-102	TRUE	reported	WYslv6
6	28046169	C	A	-0.0356	0.0051	3.33E-12	0.2319	NA	170756	rs3471681	329443	ieu-b-102	TRUE	reported	WYslv6
6	28046673	T	C	-0.0323	0.0048	1.29E-11	0.2845	NA	170756	rs203876	329443	ieu-b-102	TRUE	reported	WYslv6
6	28046789	A	G	-0.0355	0.0051	3.14E-12	0.2326	NA	170756	rs9393884	329443	ieu-b-102	TRUE	reported	WYslv6
6	28048535	A	G	-0.0354	0.0051	3.82E-12	0.2325	NA	170756	rs9380049	329443	ieu-b-102	TRUE	reported	WYslv6
6	28048538	A	G	-0.0354	0.0051	4.31E-12	0.2316	NA	170756	rs9380050	329443	ieu-b-102	TRUE	reported	WYslv6
6	28048624	T	C	-0.0324	0.0048	1.22E-11	0.2841	NA	170756	rs203877	329443	ieu-b-102	TRUE	reported	WYslv6
6	28050009	A	T	-0.0354	0.0051	3.68E-12	0.2325	NA	170756	rs1561795	329443	ieu-b-102	TRUE	reported	WYslv6
6	28050762	T	G	-0.0354	0.0051	3.68E-12	0.2325	NA	170756	rs5636434	329443	ieu-b-102	TRUE	reported	WYslv6
6	28051772	A	G	-0.0354	0.0051	3.75E-12	0.2323	NA	170756	rs9357061	329443	ieu-b-102	TRUE	reported	WYslv6
6	28051803	T	A	-0.0354	0.0051	3.69E-12	0.2323	NA	170756	rs9368550	329443	ieu-b-102	TRUE	reported	WYslv6

6	28052823	A	C	-0.0471	0.0062	2.34E-14	0.1425	NA	170756	rs1321505	329443	ieu-b-102	TRUE	reported	WYslv6
6	28053097	G	A	-0.0354	0.0051	3.69E-12	0.2323	NA	170756	rs2295594	329443	ieu-b-102	TRUE	reported	WYslv6
6	28054404	G	A	-0.0354	0.0051	3.59E-12	0.2323	NA	170756	rs9357062	329443	ieu-b-102	TRUE	reported	WYslv6
6	28054707	T	C	-0.0354	0.0051	3.58E-12	0.2323	NA	170756	rs1689366	329443	ieu-b-102	TRUE	reported	WYslv6
6	28057594	A	T	-0.0354	0.0051	3.98E-12	0.2323	NA	170756	rs2273564	329443	ieu-b-102	TRUE	reported	WYslv6
6	28058635	A	G	-0.0355	0.0051	3.21E-12	0.2324	NA	170756	rs1853097	329443	ieu-b-102	TRUE	reported	WYslv6
6	28058949	G	C	-0.059	0.007	5.65E-17	0.1048	NA	170756	rs3590287	329443	ieu-b-102	TRUE	reported	WYslv6
6	28059020	G	A	-0.0354	0.0051	3.99E-12	0.2326	NA	170756	rs9393887	329443	ieu-b-102	TRUE	reported	WYslv6
6	28059217	G	C	-0.0353	0.0051	4.18E-12	0.2323	NA	170756	rs9393888	329443	ieu-b-102	TRUE	reported	WYslv6
6	28059437	T	C	-0.0353	0.0051	4.17E-12	0.2323	NA	170756	rs3734573	329443	ieu-b-102	TRUE	reported	WYslv6
6	28060005	G	A	-0.0353	0.0051	4.12E-12	0.2323	NA	170756	rs9357063	329443	ieu-b-102	TRUE	reported	WYslv6
6	28060239	T	C	-0.0593	0.007	3.88E-17	0.1048	NA	170756	rs1319757	329443	ieu-b-102	TRUE	reported	WYslv6
6	28061744	G	A	-0.0353	0.0051	4.14E-12	0.2323	NA	170756	rs3823180	329443	ieu-b-102	TRUE	reported	WYslv6
6	28061792	A	G	-0.0354	0.0051	3.99E-12	0.2323	NA	170756	rs9368551	329443	ieu-b-102	TRUE	reported	WYslv6
6	28062066	C	A	-0.0321	0.0048	1.64E-11	0.2842	NA	170756	rs203893	329443	ieu-b-102	TRUE	reported	WYslv6
6	28063855	C	T	-0.0353	0.0051	4.12E-12	0.2323	NA	170756	rs9393890	329443	ieu-b-102	TRUE	reported	WYslv6
6	28064623	T	C	-0.0353	0.0051	4.51E-12	0.2323	NA	170756	rs9380052	329443	ieu-b-102	TRUE	reported	WYslv6
6	28064633	A	G	-0.0353	0.0051	4.38E-12	0.2323	NA	170756	rs9366715	329443	ieu-b-102	TRUE	reported	WYslv6
6	28065801	T	C	-0.0591	0.007	4.88E-17	0.1047	NA	170756	rs3416605	329443	ieu-b-102	TRUE	reported	WYslv6
6	28065888	T	C	-0.032	0.0048	2.02E-11	0.2849	NA	170756	rs476167	329443	ieu-b-102	TRUE	reported	WYslv6
6	28065993	T	C	-0.0356	0.0051	3.24E-12	0.2323	NA	170756	rs3446681	329443	ieu-b-102	TRUE	reported	WYslv6
6	28067196	T	C	-0.0321	0.0048	1.75E-11	0.2843	NA	170756	rs203892	329443	ieu-b-102	TRUE	reported	WYslv6
6	28067537	G	A	-0.0353	0.0051	4.28E-12	0.2323	NA	170756	rs9380054	329443	ieu-b-102	TRUE	reported	WYslv6
6	28067951	T	G	-0.0349	0.0051	7.53E-12	0.2327	NA	170756	rs2116981	329443	ieu-b-102	TRUE	reported	WYslv6
6	28068426	A	G	-0.0353	0.0051	4.98E-12	0.2317	NA	170756	rs9368552	329443	ieu-b-102	TRUE	reported	WYslv6
6	28070998	C	T	-0.0398	0.0053	8.43E-14	0.2052	NA	170756	rs1343744	329443	ieu-b-102	TRUE	reported	WYslv6
6	28071393	C	T	-0.0323	0.0048	1.27E-11	0.2842	NA	170756	rs188105	329443	ieu-b-102	TRUE	reported	WYslv6
6	28072602	G	A	-0.0352	0.0051	5.50E-12	0.2321	NA	170756	rs1181396	329443	ieu-b-102	TRUE	reported	WYslv6
6	28073881	G	A	-0.0577	0.007	1.89E-16	0.1067	NA	170756	rs3466224	329443	ieu-b-102	TRUE	reported	WYslv6
6	28074519	A	G	-0.0364	0.0053	5.73E-12	0.2252	NA	170756	rs1506148	329443	ieu-b-102	TRUE	reported	WYslv6
6	28074901	C	T	-0.0591	0.007	4.52E-17	0.1065	NA	170756	rs3595243	329443	ieu-b-102	TRUE	reported	WYslv6
6	28074925	C	A	-0.0352	0.0051	4.46E-12	0.2387	NA	170756	rs3557651	329443	ieu-b-102	TRUE	reported	WYslv6
6	28075000	A	C	-0.0353	0.0051	4.30E-12	0.2323	NA	170756	rs3413176	329443	ieu-b-102	TRUE	reported	WYslv6
6	28075911	G	A	-0.0369	0.0054	5.34E-12	0.2202	NA	170756	rs1126156	329443	ieu-b-102	TRUE	reported	WYslv6
6	28076270	A	G	-0.0353	0.0051	4.30E-12	0.2323	NA	170756	rs1339874	329443	ieu-b-102	TRUE	reported	WYslv6
6	28077374	C	A	-0.0323	0.0048	1.36E-11	0.2841	NA	170756	rs203884	329443	ieu-b-102	TRUE	reported	WYslv6
6	28077602	G	A	-0.0354	0.0051	3.95E-12	0.2323	NA	170756	rs1771134	329443	ieu-b-102	TRUE	reported	WYslv6
6	28078032	A	C	-0.0353	0.0051	4.45E-12	0.2323	NA	170756	rs3607860	329443	ieu-b-102	TRUE	reported	WYslv6
6	28078303	G	A	-0.0354	0.0051	3.70E-12	0.2327	NA	170756	rs6932109	329443	ieu-b-102	TRUE	reported	WYslv6
6	28078356	A	G	-0.0322	0.0048	1.59E-11	0.2843	NA	170756	rs203883	329443	ieu-b-102	TRUE	reported	WYslv6
6	28078411	A	T	-0.0355	0.0051	3.29E-12	0.2323	NA	170756	rs6931858	329443	ieu-b-102	TRUE	reported	WYslv6
6	28078502	G	A	-0.0322	0.0048	1.60E-11	0.2843	NA	170756	rs203882	329443	ieu-b-102	TRUE	reported	WYslv6
6	28079160	C	T	-0.0354	0.0051	4.36E-12	0.2323	NA	170756	rs9393891	329443	ieu-b-102	TRUE	reported	WYslv6
6	28079428	C	T	-0.0354	0.0051	4.24E-12	0.2323	NA	170756	rs9468286	329443	ieu-b-102	TRUE	reported	WYslv6
6	28079598	T	C	-0.0592	0.007	4.78E-17	0.1047	NA	170756	rs1320381	329443	ieu-b-102	TRUE	reported	WYslv6
6	28079946	G	T	-0.0353	0.0051	4.33E-12	0.2323	NA	170756	rs7739216	329443	ieu-b-102	TRUE	reported	WYslv6
6	28079953	T	C	-0.0353	0.0051	4.45E-12	0.2323	NA	170756	rs3551224	329443	ieu-b-102	TRUE	reported	WYslv6
6	28080628	C	A	-0.0592	0.007	4.47E-17	0.1048	NA	170756	rs3458811	329443	ieu-b-102	TRUE	reported	WYslv6
6	28080777	T	C	-0.0613	0.0073	5.26E-17	0.1029	NA	170756	rs5766405	329443	ieu-b-102	TRUE	reported	WYslv6
6	28081394	A	G	-0.0353	0.0051	4.19E-12	0.2323	NA	170756	rs9393892	329443	ieu-b-102	TRUE	reported	WYslv6
6	28081629	A	G	-0.0353	0.0051	4.15E-12	0.2323	NA	170756	rs9380055	329443	ieu-b-102	TRUE	reported	WYslv6
6	28081758	T	C	-0.0592	0.007	4.47E-17	0.1047	NA	170756	rs3437150	329443	ieu-b-102	TRUE	reported	WYslv6
6	28082265	T	C	-0.0353	0.0051	4.13E-12	0.2323	NA	170756	rs9368553	329443	ieu-b-102	TRUE	reported	WYslv6
6	28082711	T	C	-0.0354	0.0051	3.89E-12	0.2323	NA	170756	rs9368554	329443	ieu-b-102	TRUE	reported	WYslv6
6	28083521	C	G	-0.0353	0.0051	4.60E-12	0.2323	NA	170756	rs4713137	329443	ieu-b-102	TRUE	reported	WYslv6
6	28084189	G	A	-0.0353	0.0051	4.27E-12	0.2323	NA	170756	rs9348793	329443	ieu-b-102	TRUE	reported	WYslv6
6	28086413	C	T	-0.0323	0.0048	1.44E-11	0.2841	NA	170756	rs1770131	329443	ieu-b-102	TRUE	reported	WYslv6
6	28086478	A	G	-0.0353	0.0051	6.49E-12	0.2301	NA	170756	rs6905516	329443	ieu-b-102	TRUE	reported	WYslv6
6	28086479	A	C	-0.0353	0.0051	6.13E-12	0.2301	NA	170756	rs7154660	329443	ieu-b-102	TRUE	reported	WYslv6
6	28086883	T	C	-0.0465	0.0061	2.14E-14	0.1466	NA	170756	rs9461432	329443	ieu-b-102	TRUE	reported	WYslv6
6	28087674	C	T	-0.0354	0.0051	3.93E-12	0.2323	NA	170756	rs9468290	329443	ieu-b-102	TRUE	reported	WYslv6
6	28089481	C	T	-0.0591	0.007	5.12E-17	0.1048	NA	170756	rs7155907	329443	ieu-b-102	TRUE	reported	WYslv6
6	28089699	C	A	-0.0324	0.0048	1.26E-11	0.2841	NA	170756	rs1631552	329443	ieu-b-102	TRUE	reported	WYslv6
6	28089731	C	T	-0.0591	0.007	4.96E-17	0.1048	NA	170756	rs6688649	329443	ieu-b-102	TRUE	reported	WYslv6
6	28090931	A	G	-0.0353	0.0051	4.54E-12	0.2324	NA	170756	rs6934769	329443	ieu-b-102	TRUE	reported	WYslv6
6	28091580	A	G	-0.0591	0.007	4.96E-17	0.1047	NA	170756	rs3534522	329443	ieu-b-102	TRUE	reported	WYslv6
6	28092307	C	G	-0.0354	0.0051	3.89E-12	0.2323	NA	170756	rs1771180	329443	ieu-b-102	TRUE	reported	WYslv6
6	28092603	T	G	-0.0329	0.0048	6.43E-12	0.2825	NA	170756	rs1561808	329443	ieu-b-102	TRUE	reported	WYslv6
6	28092685	C	T	-0.0464	0.0061	2.45E-14	0.1466	NA	170756	rs4713139	329443	ieu-b-102	TRUE	reported	WYslv6
6	28094366	G	A	-0.0354	0.0051	3.75E-12	0.2327	NA	170756	rs6922063	329443	ieu-b-102	TRUE	reported	WYslv6
6	28094731	T	C	-0.0354	0.0051	3.84E-12	0.2327	NA	170756	rs2275508	329443	ieu-b-102	TRUE	reported	WYslv6
6	28095172	C	T	-0.0354	0.0051	3.85E-12	0.2323	NA	170756	rs9461433	329443	ieu-b-102	TRUE	reported	WYslv6
6	28095355	G	A	-0.0354	0.0051	3.85E-12	0.2323	NA	170756	rs9468292	329443	ieu-b-102	TRUE	reported	WYslv6
6	28097193	G	A	-0.0465	0.0061	2.11E-14	0.1467	NA	170756	rs1274847	329443	ieu-b-102	TRUE	reported	WYslv6
6	28099344	G	A	-0.0354	0.0051	3.54E-12	0.2325	NA	170756	rs1947863	329443	ieu-b-102	TRUE	reported	WYslv6
6	28101678	G	C	-0.0355	0.0051	3.51E-12	0.2323	NA	170756	rs4713141	329443	ieu-b-102	TRUE	reported	WYslv6
6	28102378	C	T	-0.0467	0.0061	2.01E-14	0.1467	NA	170756	rs3413021	329443	ieu-b-102	TRUE	reported	WYslv6
6	28102465	A	C	-0.0369	0.0053	5.28E-12	0.2204	NA	170756	rs9461434	329443	ieu-b-102	TRUE	reported	WYslv6
6	28103691	C	T	-0.0353	0.0051	4.14E-12	0.2326	NA	170756	rs1340004	329443	ieu-b-102	TRUE	reported	WYslv6
6	28104476	C	T	-0.0353	0.0051	4.38E-12	0.2328	NA	170756	rs9380056	329443	ieu-b-102	TRUE	reported	WYslv6
6	28104634	G	T	-0.0352	0.0051	4.94E-12	0.2328	NA	170756	rs9380057	329443	ieu-b-102	TRUE	reported	WYslv6
6	28105196	C	T	-0.0353	0.0051	4.45E-12	0.2326	NA	170756	rs1947862	329443	ieu-b-102	TRUE	reported	WYslv6
6	28106141	T	C	-0.0351	0.0051	5.32E-12	0.2329	NA	170756	rs6941992	329443	ieu-b-102	TRUE	reported	WYslv6
6	28106347	T	G	-0.0352	0.0051	5.12E-12	0.2328	NA	170756	rs4713142	329443	ieu-b-102	TRUE	reported	WYslv6
6	28106759	A	C	-0.0348	0.0051	8.52E-12	0.2324	NA	170756	rs4713143	329443	ieu-b-102	TRUE	reported	WYslv6
6	28106790	C	G	-0.0351	0.0051	5.34E-12	0.2328	NA	170756	rs4713144	329443	ieu-b-102	TRUE	reported	WYslv6
6	28106827	T	C	-0.0353	0.0051	4.36E-12	0.2331	NA	170756	rs4713145	329443	ieu-b-102	TRUE	reported	WYslv6
6	28107654	T	C	-0.0351	0.0051	5.67E-12	0.2326	NA	170756	rs3757187	329443	ieu-b-102	TRUE	reported	WYslv6
6	28107776	T	A	-0.0351	0.0051	6.12E-12	0.2324	NA	170756	rs1161362	329443	ieu-b-102	TRUE	reported	WYslv6
6	28108085	C	T	-0.0345	0.0051	1.33E-11	0.2317	NA	170756	rs1904841	329443	ieu-b-102	TRUE	reported	WYslv6
6	28108232	C	T	-0.0346	0.0051	1.11E-11	0.2316	NA	170756	rs1904840	329443	ieu-b-102	TRUE	reported	WYslv6
6	28108788	C	T	-0.0315	0.0048	4.30E-11	0.2834	NA	170756	rs2791332	329443	ieu-b-102	TRUE	reported	WYslv6
6	28108967	T	C	-0.0346	0.0051	1.18E-11	0.2315	NA	170756	rs9368555	329443	ieu-b-102	TRUE	reported	WYslv6
6	28109262	T	C	-0.0347	0.0051	1.09E-11	0.2315	NA	170756	rs9393893	329443	ieu-b-102	TRUE	reported	WYslv6
6	28110148	C	T	-0.0348	0.0051	9.27E-12	0.2315	NA	170756	rs868987	329443	ieu-b-102	TRUE	reported	WYslv6
6	28111536	A	T	-0.0348	0.0051	9.45E-12	0.2315	NA	170756	rs4713146	329443	ieu-b-102	TRUE	reported	WYslv6
6	28112562	A	G	-0.0347	0.0051	9.82E-12	0.2315	NA	170756	rs9393894	329443	ieu-b-102	TRUE	reported	WYslv6
6	28112713	T	C	-0.0348	0.0051	9.59E-12	0.2316	NA	170756	rs9468295	329443	ieu-b-102	TRUE	reported	WYslv6
6	28113730	A	G	-0.0347	0.0051	1.07E-11	0.2316	NA	170756	rs9468296	329443	ieu-b-102	TRUE	reported	WYslv6
6	28113746	A	C	-0.0316	0.0048	3.67E-11	0.2834	NA	170756	rs1225716	329443	ieu-b-102	TRUE	reported	WYslv6
6	28114818	G	T	-0.059	0.007	5.81E-17	0.1048	NA	170756	rs3574957	329443	ieu-b-102	TRUE	reported	WYslv6
6	28115156	C	T	-0.0348	0.0051	9.21E-12	0.2316	NA	170756	rs1424460	329443	ieu-b-102	TRUE	reported	WYslv6
6	28115921	G	A	-0.0347	0.0051	9.70E-12	0.2315	NA	170756	rs4713148	329443	ieu-b-102	TRUE	reported	WYslv6
6	28117757	T	A	-0.0348	0.0051	8.96E-12	0.2315	NA	170756	rs9348794	329443	ieu-b-102	TRUE	reported	WYslv6
6	28118874	G	T	-0.0348	0.0051	9.19E-12	0.2315	NA	170756	rs9468297	329443	ieu-b-102	TRUE	reported	WYslv6
6	28119055	T	C	-0.059	0.007	5.60E-17	0.1048	NA	170756	rs7284678	329443	ieu-b-102	TRUE	reported	WYslv6
6	28120401	G	C	-0.0317	0.0048	3.61E-11	0.2834	NA	170756	rs2622319	329443	ieu-b-102	TRUE	reported	WYslv6

6	28120663	C	G	-0.0348	0.0051	8.84E-12	0.2315	NA	170756	rs9295758	329443	ieu-b-102	TRUE	reported	WYslv6
6	28120898	G	A	-0.0349	0.0051	8.32E-12	0.2315	NA	170756	rs1777466	329443	ieu-b-102	TRUE	reported	WYslv6
6	28122345	A	G	-0.0349	0.0051	8.34E-12	0.2315	NA	170756	rs9468298	329443	ieu-b-102	TRUE	reported	WYslv6
6	28123928	T	C	-0.0314	0.0048	5.26E-11	0.2834	NA	170756	rs8895775	329443	ieu-b-102	TRUE	reported	WYslv6
6	28124114	C	T	-0.059	0.0071	5.90E-17	0.1047	NA	170756	rs1320591	329443	ieu-b-102	TRUE	reported	WYslv6
6	28124469	C	T	-0.0348	0.0051	9.45E-12	0.2316	NA	170756	rs9295759	329443	ieu-b-102	TRUE	reported	WYslv6
6	28126202	A	G	-0.0348	0.0051	8.93E-12	0.2315	NA	170756	rs9348796	329443	ieu-b-102	TRUE	reported	WYslv6
6	28126834	C	T	-0.0348	0.0051	9.32E-12	0.2316	NA	170756	rs1155221	329443	ieu-b-102	TRUE	reported	WYslv6
6	28126840	C	T	-0.0466	0.0061	1.97E-14	0.1466	NA	170756	rs9468300	329443	ieu-b-102	TRUE	reported	WYslv6
6	28127444	T	C	-0.0351	0.0051	6.36E-12	0.233	NA	170756	rs9380058	329443	ieu-b-102	TRUE	reported	WYslv6
6	28127621	T	C	-0.0348	0.0051	8.93E-12	0.2315	NA	170756	rs9393895	329443	ieu-b-102	TRUE	reported	WYslv6
6	28127703	C	T	-0.035	0.0051	7.14E-12	0.2327	NA	170756	rs9393896	329443	ieu-b-102	TRUE	reported	WYslv6
6	28127710	G	T	-0.0339	0.0051	4.23E-11	0.2332	NA	170756	rs9393897	329443	ieu-b-102	TRUE	reported	WYslv6
6	28129232	C	T	-0.0596	0.0071	5.28E-17	0.1038	NA	170756	rs3700348	329443	ieu-b-102	TRUE	reported	WYslv6
6	28129580	T	C	-0.0348	0.0051	8.85E-12	0.2315	NA	170756	rs9357065	329443	ieu-b-102	TRUE	reported	WYslv6
6	28129831	T	C	-0.0348	0.0051	9.10E-12	0.2315	NA	170756	rs9357066	329443	ieu-b-102	TRUE	reported	WYslv6
6	28130089	C	T	-0.0589	0.007	6.73E-17	0.1048	NA	170756	rs1320130	329443	ieu-b-102	TRUE	reported	WYslv6
6	28130376	G	T	-0.0348	0.0051	9.41E-12	0.2316	NA	170756	rs9393898	329443	ieu-b-102	TRUE	reported	WYslv6
6	28130450	G	A	-0.0589	0.007	6.42E-17	0.1048	NA	170756	rs3476515	329443	ieu-b-102	TRUE	reported	WYslv6
6	28131153	T	C	-0.0348	0.0051	1.04E-11	0.2316	NA	170756	rs9368556	329443	ieu-b-102	TRUE	reported	WYslv6
6	28131537	T	C	-0.0349	0.0051	8.74E-12	0.2315	NA	170756	rs9368557	329443	ieu-b-102	TRUE	reported	WYslv6
6	28132358	A	C	-0.0347	0.0051	1.03E-11	0.2315	NA	170756	rs9380059	329443	ieu-b-102	TRUE	reported	WYslv6
6	28132603	G	A	-0.0348	0.0051	9.17E-12	0.2315	NA	170756	rs9380060	329443	ieu-b-102	TRUE	reported	WYslv6
6	28132726	A	G	-0.0348	0.0051	8.97E-12	0.2315	NA	170756	rs1183304	329443	ieu-b-102	TRUE	reported	WYslv6
6	28132803	T	C	-0.0348	0.0051	9.18E-12	0.2315	NA	170756	rs9380061	329443	ieu-b-102	TRUE	reported	WYslv6
6	28133239	A	T	-0.0589	0.007	6.83E-17	0.1048	NA	170756	rs3450582	329443	ieu-b-102	TRUE	reported	WYslv6
6	28133306	A	G	-0.0348	0.0051	8.89E-12	0.2315	NA	170756	rs9368558	329443	ieu-b-102	TRUE	reported	WYslv6
6	28133528	G	C	-0.0348	0.0051	9.17E-12	0.2315	NA	170756	rs9393899	329443	ieu-b-102	TRUE	reported	WYslv6
6	28134221	T	C	-0.0588	0.007	6.92E-17	0.1048	NA	170756	rs3509843	329443	ieu-b-102	TRUE	reported	WYslv6
6	28135660	G	C	-0.0348	0.0051	8.59E-12	0.2315	NA	170756	rs9283884	329443	ieu-b-102	TRUE	reported	WYslv6
6	28136212	G	A	-0.0347	0.0051	9.71E-12	0.2315	NA	170756	rs4713150	329443	ieu-b-102	TRUE	reported	WYslv6
6	28136356	G	A	-0.0348	0.0051	9.13E-12	0.2315	NA	170756	rs4713151	329443	ieu-b-102	TRUE	reported	WYslv6
6	28136797	A	G	-0.0348	0.0051	8.81E-12	0.2315	NA	170756	rs9393901	329443	ieu-b-102	TRUE	reported	WYslv6
6	28137027	T	G	-0.0347	0.0051	9.63E-12	0.2315	NA	170756	rs3173443	329443	ieu-b-102	TRUE	reported	WYslv6
6	28137454	G	A	-0.0349	0.0051	7.41E-12	0.2315	NA	170756	rs4713152	329443	ieu-b-102	TRUE	reported	WYslv6
6	28137499	G	A	-0.0592	0.0071	6.46E-17	0.1046	NA	170756	rs7284679	329443	ieu-b-102	TRUE	reported	WYslv6
6	28137533	G	C	-0.0348	0.0051	8.65E-12	0.2315	NA	170756	rs9348797	329443	ieu-b-102	TRUE	reported	WYslv6
6	28137569	G	A	-0.0348	0.0051	8.45E-12	0.2315	NA	170756	rs9380062	329443	ieu-b-102	TRUE	reported	WYslv6
6	28137853	A	G	-0.0348	0.0051	9.96E-12	0.2315	NA	170756	rs9380063	329443	ieu-b-102	TRUE	reported	WYslv6
6	28139710	A	T	-0.0589	0.007	6.49E-17	0.1048	NA	170756	rs1321798	329443	ieu-b-102	TRUE	reported	WYslv6
6	28141253	G	T	-0.0589	0.007	6.28E-17	0.1049	NA	170756	rs6729753	329443	ieu-b-102	TRUE	reported	WYslv6
6	28141460	G	A	-0.0467	0.0061	1.82E-14	0.1467	NA	170756	rs8180562	329443	ieu-b-102	TRUE	reported	WYslv6
6	28141548	T	G	-0.0348	0.0051	9.60E-12	0.2315	NA	170756	rs1233297	329443	ieu-b-102	TRUE	reported	WYslv6
6	28142276	C	T	-0.0319	0.0048	2.40E-11	0.2874	NA	170756	rs1150678	329443	ieu-b-102	TRUE	reported	WYslv6
6	28142587	G	C	-0.0348	0.0051	9.96E-12	0.2315	NA	170756	rs1205605	329443	ieu-b-102	TRUE	reported	WYslv6
6	28143011	G	A	-0.035	0.0051	7.86E-12	0.2313	NA	170756	rs3866983	329443	ieu-b-102	TRUE	reported	WYslv6
6	28143118	A	G	-0.0465	0.0061	2.12E-14	0.1466	NA	170756	rs9380064	329443	ieu-b-102	TRUE	reported	WYslv6
6	28144751	A	G	-0.0349	0.0051	9.02E-12	0.2315	NA	170756	rs9380065	329443	ieu-b-102	TRUE	reported	WYslv6
6	28144832	T	C	-0.0589	0.007	6.50E-17	0.1047	NA	170756	rs7568953	329443	ieu-b-102	TRUE	reported	WYslv6
6	28147385	G	A	-0.0316	0.0048	3.89E-11	0.2834	NA	170756	rs9295760	329443	ieu-b-102	TRUE	reported	WYslv6
6	28147987	A	G	-0.0346	0.0051	1.28E-11	0.2317	NA	170756	rs9295761	329443	ieu-b-102	TRUE	reported	WYslv6
6	28150878	C	T	-0.0589	0.0071	7.18E-17	0.1045	NA	170756	rs3483258	329443	ieu-b-102	TRUE	reported	WYslv6
6	28150998	G	A	-0.0341	0.0051	2.17E-11	0.2363	NA	170756	rs9380067	329443	ieu-b-102	TRUE	reported	WYslv6
6	28152583	T	C	-0.0348	0.0051	7.49E-12	0.2359	NA	170756	rs6901017	329443	ieu-b-102	TRUE	reported	WYslv6
6	28155418	T	C	-0.0348	0.0051	7.71E-12	0.2357	NA	170756	rs9295762	329443	ieu-b-102	TRUE	reported	WYslv6
6	28159960	T	C	-0.0376	0.0052	4.18E-13	0.2233	NA	170756	rs9627911	329443	ieu-b-102	TRUE	reported	WYslv6
6	28160799	T	G	-0.0348	0.0051	7.06E-12	0.2363	NA	170756	rs1225598	329443	ieu-b-102	TRUE	reported	WYslv6
6	28162087	C	T	-0.0318	0.0048	2.19E-11	0.2879	NA	170756	rs1225597	329443	ieu-b-102	TRUE	reported	WYslv6
6	28162620	T	C	-0.0348	0.0051	7.52E-12	0.2357	NA	170756	rs1225596	329443	ieu-b-102	TRUE	reported	WYslv6
6	28165099	T	C	-0.035	0.0051	5.55E-12	0.2361	NA	170756	rs1150689	329443	ieu-b-102	TRUE	reported	WYslv6
6	28165190	C	T	-0.0348	0.0051	6.89E-12	0.2361	NA	170756	rs1225599	329443	ieu-b-102	TRUE	reported	WYslv6
6	28166447	C	G	-0.0349	0.0051	6.58E-12	0.2361	NA	170756	rs1233705	329443	ieu-b-102	TRUE	reported	WYslv6
6	28167635	G	C	-0.032	0.0048	1.73E-11	0.2879	NA	170756	rs1071893	329443	ieu-b-102	TRUE	reported	WYslv6
6	28168343	C	T	-0.0473	0.0062	1.82E-14	0.1425	NA	170756	rs9368561	329443	ieu-b-102	TRUE	reported	WYslv6
6	28168726	C	G	-0.035	0.0051	4.90E-12	0.236	NA	170756	rs1233701	329443	ieu-b-102	TRUE	reported	WYslv6
6	28169158	T	A	-0.0349	0.0051	6.56E-12	0.2361	NA	170756	rs1233699	329443	ieu-b-102	TRUE	reported	WYslv6
6	28169241	G	A	-0.0592	0.007	4.45E-17	0.1049	NA	170756	rs1319529	329443	ieu-b-102	TRUE	reported	WYslv6
6	28170297	G	A	-0.0445	0.006	1.82E-13	0.1497	NA	170756	rs735765	329443	ieu-b-102	TRUE	reported	WYslv6
6	28172263	G	A	-0.0348	0.0051	7.20E-12	0.2361	NA	170756	rs1233697	329443	ieu-b-102	TRUE	reported	WYslv6
6	28172953	G	A	-0.0348	0.0051	6.98E-12	0.2361	NA	170756	rs1233707	329443	ieu-b-102	TRUE	reported	WYslv6
6	28173010	C	T	-0.035	0.0051	5.46E-12	0.2361	NA	170756	rs1237875	329443	ieu-b-102	TRUE	reported	WYslv6
6	28173219	G	A	-0.0318	0.0048	2.42E-11	0.2874	NA	170756	rs1233708	329443	ieu-b-102	TRUE	reported	WYslv6
6	28173957	A	G	-0.0351	0.0051	4.66E-12	0.236	NA	170756	rs1150692	329443	ieu-b-102	TRUE	reported	WYslv6
6	28174590	T	C	-0.0348	0.0051	6.89E-12	0.2366	NA	170756	rs1150693	329443	ieu-b-102	TRUE	reported	WYslv6
6	28174757	G	A	-0.0595	0.0071	3.61E-17	0.1046	NA	170756	rs1319763	329443	ieu-b-102	TRUE	reported	WYslv6
6	28175549	G	C	-0.0376	0.0051	1.57E-13	0.2427	NA	170756	rs1186640	329443	ieu-b-102	TRUE	reported	WYslv6
6	28187632	T	G	-0.0598	0.0071	2.38E-17	0.1048	NA	170756	rs3500116	329443	ieu-b-102	TRUE	reported	WYslv6
6	28190830	T	A	-0.0488	0.0062	2.78E-15	0.1416	NA	170756	rs9393908	329443	ieu-b-102	TRUE	reported	WYslv6
6	28191057	C	T	-0.0488	0.0062	3.10E-15	0.1416	NA	170756	rs9366717	329443	ieu-b-102	TRUE	reported	WYslv6
6	28191288	G	T	-0.0596	0.007	2.50E-17	0.1051	NA	170756	rs3565693	329443	ieu-b-102	TRUE	reported	WYslv6
6	28193102	C	A	-0.0595	0.007	2.88E-17	0.1051	NA	170756	rs3424344	329443	ieu-b-102	TRUE	reported	WYslv6
6	28194629	A	T	-0.0344	0.005	7.75E-12	0.2429	NA	170756	rs9461443	329443	ieu-b-102	TRUE	reported	WYslv6
6	28197186	C	T	-0.0341	0.005	1.06E-11	0.2432	NA	170756	rs3447709	329443	ieu-b-102	TRUE	reported	WYslv6
6	28198831	G	A	-0.037	0.0055	2.07E-11	0.1865	NA	170756	rs2299029	329443	ieu-b-102	TRUE	reported	WYslv6
6	28199764	A	G	-0.0369	0.0055	2.49E-11	0.1866	NA	170756	rs1196713	329443	ieu-b-102	TRUE	reported	WYslv6
6	28199937	G	C	-0.0371	0.0055	1.86E-11	0.1868	NA	170756	rs1370796	329443	ieu-b-102	TRUE	reported	WYslv6
6	28201531	G	A	-0.0595	0.007	2.86E-17	0.105	NA	170756	rs1320401	329443	ieu-b-102	TRUE	reported	WYslv6
6	28203056	T	G	-0.0595	0.007	2.77E-17	0.105	NA	170756	rs1320521	329443	ieu-b-102	TRUE	reported	WYslv6
6	28205502	A	T	-0.0368	0.0055	2.83E-11	0.1868	NA	170756	rs9366718	329443	ieu-b-102	TRUE	reported	WYslv6
6	28207200	C	T	-0.0372	0.0055	1.62E-11	0.1864	NA	170756	rs9393909	329443	ieu-b-102	TRUE	reported	WYslv6
6	28208192	C	G	-0.0371	0.0055	1.96E-11	0.1865	NA	170756	rs9393910	329443	ieu-b-102	TRUE	reported	WYslv6
6	28208558	A	G	-0.037	0.0055	2.03E-11	0.1865	NA	170756	rs9368563	329443	ieu-b-102	TRUE	reported	WYslv6
6	28209102	G	A	-0.0372	0.0055	1.68E-11	0.1865	NA	170756	rs9295768	329443	ieu-b-102	TRUE	reported	WYslv6
6	28209531	T	A	-0.037	0.0055	2.04E-11	0.1865	NA	170756	rs9468318	329443	ieu-b-102	TRUE	reported	WYslv6
6	28210293	C	G	-0.037	0.0055	2.29E-11	0.1864	NA	170756	rs9357067	329443	ieu-b-102	TRUE	reported	WYslv6
6	28214698	C	T	-0.0521	0.0065	1.56E-15	0.1244	NA	170756	rs1772029	329443	ieu-b-102	TRUE	reported	WYslv6
6	28216486	C	G	-0.0467	0.006	7.42E-15	0.1522	NA	170756	rs7740487	329443	ieu-b-102	TRUE	reported	WYslv6
6	28217797	G	T	-0.0459	0.006	2.59E-14	0.1533	NA	170756	rs6814101	329443	ieu-b-102	TRUE	reported	WYslv6
6	28218199	A	C	-0.0467	0.006	7.13E-15	0.1522	NA	170756	rs7511343	329443	ieu-b-102	TRUE	reported	WYslv6
6	28219661	G	A	-0.0466	0.006	7.93E-15	0.1522	NA	170756	rs9986596	329443	ieu-b-102	TRUE	reported	WYslv6
6	28221264	G	A	0.0475	0.006	1.90E-15	0.8467	NA	170756	rs1679732	329443	ieu-b-102	TRUE	reported	WYslv6
6	28221816	A	G	0.047	0.006	5.02E-15	0.8479	NA	170756	rs1045636	329443	ieu-b-102	TRUE	reported	WYslv6
6	28225311	C	G	-0.06	0.007	1.59E-17	0.105	NA	170756	rs1320809	329443	ieu-b-102	TRUE	reported	WYslv6
6	28228342	A	G	0.0475	0.006	1.83E-15	0.8465	NA	170756	rs1467481	329443	ieu-b-102	TRUE	reported	WYslv6
6	28229411	G	A	0.0474	0.006	2.25E-15	0.8465	NA	170756	rs1778511	329443	ieu-b-102	TRUE	reported	WYslv6
6	28229881	C	T	0.0474	0.006	2.05E-15	0.8465	NA	170756	rs1778508	329443	ieu-b-102	TRUE	reported	WYslv6
6	28234597	C	T	-0.0465	0.006	6.65E-15	0.1542	NA	170756	rs2799077	329443	ieu-b-102	TRUE	reported	WYslv6

6	28235176	A	C	-0.0472	0.006	2.79E-15	0.1536	NA	170756	rs2799079	329443	ieu-b-102	TRUE	reported	WYslv6
6	28238059	T	C	-0.0606	0.007	4.83E-18	0.1066	NA	170756	rs6799822	329443	ieu-b-102	TRUE	reported	WYslv6
6	28239915	G	A	-0.0475	0.006	1.93E-15	0.1536	NA	170756	rs1196553	329443	ieu-b-102	TRUE	reported	WYslv6
6	28241081	G	A	-0.0474	0.006	2.06E-15	0.1535	NA	170756	rs2743555	329443	ieu-b-102	TRUE	reported	WYslv6
6	28250179	T	C	-0.0607	0.007	4.26E-18	0.1065	NA	170756	rs3487880	329443	ieu-b-102	TRUE	reported	WYslv6
6	28250955	A	C	-0.0608	0.007	3.84E-18	0.1064	NA	170756	rs3439684	329443	ieu-b-102	TRUE	reported	WYslv6
6	28250984	G	A	-0.0486	0.006	5.10E-16	0.153	NA	170756	rs6901575	329443	ieu-b-102	TRUE	reported	WYslv6
6	28254988	T	C	0.0417	0.0074	1.81E-08	0.0934	NA	170756	rs1690184	329443	ieu-b-102	TRUE	reported	WYslv6
6	28257720	A	G	0.0415	0.0074	2.08E-08	0.0934	NA	170756	rs7744788	329443	ieu-b-102	TRUE	reported	WYslv6
6	28258208	C	A	-0.0608	0.007	3.97E-18	0.1065	NA	170756	rs3469122	329443	ieu-b-102	TRUE	reported	WYslv6
6	28262686	G	A	-0.0483	0.006	6.62E-16	0.1543	NA	170756	rs6905391	329443	ieu-b-102	TRUE	reported	WYslv6
6	28265218	A	G	0.0416	0.0074	1.95E-08	0.0934	NA	170756	rs7562130	329443	ieu-b-102	TRUE	reported	WYslv6
6	28265572	C	T	-0.0604	0.007	7.91E-18	0.1052	NA	170756	rs1320734	329443	ieu-b-102	TRUE	reported	WYslv6
6	28265954	A	T	-0.0604	0.007	7.98E-18	0.1053	NA	170756	rs1274871	329443	ieu-b-102	TRUE	reported	WYslv6
6	28268452	G	C	-0.0484	0.006	5.26E-16	0.1545	NA	170756	rs1936365	329443	ieu-b-102	TRUE	reported	WYslv6
6	28268824	A	G	-0.0598	0.007	1.51E-17	0.106	NA	170756	rs3393208	329443	ieu-b-102	TRUE	reported	WYslv6
6	28271973	C	A	-0.0604	0.007	8.51E-18	0.1052	NA	170756	rs5618911	329443	ieu-b-102	TRUE	reported	WYslv6
6	28277671	T	G	-0.0603	0.007	9.10E-18	0.1051	NA	170756	rs1319178	329443	ieu-b-102	TRUE	reported	WYslv6
6	28278688	G	A	-0.0608	0.007	3.43E-18	0.1066	NA	170756	rs3495048	329443	ieu-b-102	TRUE	reported	WYslv6
6	28279100	A	T	-0.0483	0.006	5.72E-16	0.1545	NA	170756	rs853694	329443	ieu-b-102	TRUE	reported	WYslv6
6	28281541	C	T	-0.0593	0.0071	4.45E-17	0.1059	NA	170756	rs3507289	329443	ieu-b-102	TRUE	reported	WYslv6
6	28281894	G	A	-0.0608	0.007	3.60E-18	0.1066	NA	170756	rs3466112	329443	ieu-b-102	TRUE	reported	WYslv6
6	28285985	G	A	-0.0605	0.007	4.50E-18	0.1084	NA	170756	rs1319088	329443	ieu-b-102	TRUE	reported	WYslv6
6	28288785	C	T	-0.0484	0.006	4.96E-16	0.1548	NA	170756	rs853685	329443	ieu-b-102	TRUE	reported	WYslv6
6	28290328	T	G	-0.0603	0.007	9.81E-18	0.1053	NA	170756	rs5607569	329443	ieu-b-102	TRUE	reported	WYslv6
6	28290647	T	C	-0.0603	0.007	1.00E-17	0.1053	NA	170756	rs3421884	329443	ieu-b-102	TRUE	reported	WYslv6
6	28295875	T	C	-0.0485	0.006	4.16E-16	0.1547	NA	170756	rs853682	329443	ieu-b-102	TRUE	reported	WYslv6
6	28296650	C	A	-0.0484	0.006	4.84E-16	0.1547	NA	170756	rs853681	329443	ieu-b-102	TRUE	reported	WYslv6
6	28296863	C	A	-0.0485	0.006	4.27E-16	0.1547	NA	170756	rs853679	329443	ieu-b-102	TRUE	reported	WYslv6
6	28297313	T	A	-0.0482	0.006	6.46E-16	0.1549	NA	170756	rs853678	329443	ieu-b-102	TRUE	reported	WYslv6
6	28299687	C	T	-0.0496	0.006	9.17E-17	0.1554	NA	170756	rs853676	329443	ieu-b-102	TRUE	reported	WYslv6
6	28301099	A	G	-0.0553	0.0067	1.70E-16	0.1172	NA	170756	rs7752448	329443	ieu-b-102	TRUE	reported	WYslv6
6	28301195	A	G	-0.0572	0.0069	7.71E-17	0.1141	NA	170756	rs7752608	329443	ieu-b-102	TRUE	reported	WYslv6
6	28304384	A	C	-0.061	0.007	2.59E-18	0.1069	NA	170756	rs6734077	329443	ieu-b-102	TRUE	reported	WYslv6
6	28305508	G	A	-0.06	0.007	1.31E-17	0.1055	NA	170756	rs1285775	329443	ieu-b-102	TRUE	reported	WYslv6
6	28306671	T	C	-0.0582	0.007	9.88E-17	0.1064	NA	170756	rs1321761	329443	ieu-b-102	TRUE	reported	WYslv6
6	28314880	C	T	-0.0615	0.007	2.44E-18	0.1047	NA	170756	rs3501603	329443	ieu-b-102	TRUE	reported	WYslv6
6	28318331	G	T	-0.0613	0.0071	3.73E-18	0.1047	NA	170756	rs1159490	329443	ieu-b-102	TRUE	reported	WYslv6
6	28323702	G	A	-0.0611	0.007	3.73E-18	0.1052	NA	170756	rs1319880	329443	ieu-b-102	TRUE	reported	WYslv6
6	28324378	C	T	-0.0613	0.007	2.88E-18	0.1052	NA	170756	rs3535335	329443	ieu-b-102	TRUE	reported	WYslv6
6	28326431	G	A	0.043	0.0074	7.87E-09	0.0929	NA	170756	rs1164067	329443	ieu-b-102	TRUE	reported	WYslv6
6	28332141	G	A	-0.0609	0.007	4.57E-18	0.1054	NA	170756	rs1321402	329443	ieu-b-102	TRUE	reported	WYslv6
6	28339478	A	G	0.0588	0.0103	1.10E-08	0.0457	NA	170756	rs1165373	329443	ieu-b-102	TRUE	reported	WYslv6
6	28345282	C	T	-0.0617	0.007	1.51E-18	0.1055	NA	170756	rs3501720	329443	ieu-b-102	TRUE	reported	WYslv6
6	28349698	A	G	-0.0611	0.007	3.38E-18	0.1055	NA	170756	rs1172006	329443	ieu-b-102	TRUE	reported	WYslv6
6	28354835	C	G	-0.062	0.007	1.14E-18	0.1056	NA	170756	rs6798181	329443	ieu-b-102	TRUE	reported	WYslv6
6	28355671	C	T	0.0422	0.0074	1.17E-08	0.0933	NA	170756	rs7452546	329443	ieu-b-102	TRUE	reported	WYslv6
6	28357807	C	T	-0.0619	0.007	1.18E-18	0.1056	NA	170756	rs3609217	329443	ieu-b-102	TRUE	reported	WYslv6
6	28358009	T	A	-0.0613	0.007	2.87E-18	0.1054	NA	170756	rs1321398	329443	ieu-b-102	TRUE	reported	WYslv6
6	28359632	T	G	-0.0619	0.007	1.20E-18	0.1056	NA	170756	rs2232429	329443	ieu-b-102	TRUE	reported	WYslv6
6	28360659	G	C	-0.0619	0.007	1.26E-18	0.1056	NA	170756	rs2232426	329443	ieu-b-102	TRUE	reported	WYslv6
6	28362309	G	A	-0.0613	0.007	2.76E-18	0.1054	NA	170756	rs3454698	329443	ieu-b-102	TRUE	reported	WYslv6
6	28364232	G	A	-0.0613	0.007	2.78E-18	0.1054	NA	170756	rs3487126	329443	ieu-b-102	TRUE	reported	WYslv6
6	28366151	A	G	-0.062	0.007	1.14E-18	0.1056	NA	170756	rs2232423	329443	ieu-b-102	TRUE	reported	WYslv6
6	28366262	G	A	0.0424	0.0074	1.05E-08	0.0933	NA	170756	rs2232422	329443	ieu-b-102	TRUE	reported	WYslv6
6	28368106	T	C	0.042	0.0074	1.41E-08	0.0932	NA	170756	rs7826490	329443	ieu-b-102	TRUE	reported	WYslv6
6	28368508	G	C	-0.0614	0.007	2.32E-18	0.1054	NA	170756	rs3588347	329443	ieu-b-102	TRUE	reported	WYslv6
6	28380931	C	T	0.042	0.0074	1.62E-08	0.093	NA	170756	rs1165110	329443	ieu-b-102	TRUE	reported	WYslv6
6	28394680	C	T	-0.0608	0.007	5.13E-18	0.1055	NA	170756	rs1320168	329443	ieu-b-102	TRUE	reported	WYslv6
6	28411941	G	C	-0.0607	0.007	5.64E-18	0.1054	NA	170756	rs6738117	329443	ieu-b-102	TRUE	reported	WYslv6
6	28415601	T	C	0.0593	0.0103	8.63E-09	0.0457	NA	170756	rs1404272	329443	ieu-b-102	TRUE	reported	WYslv6
6	28423599	T	C	0.0421	0.0074	1.49E-08	0.093	NA	170756	rs7766749	329443	ieu-b-102	TRUE	reported	WYslv6
6	28490915	T	C	0.0616	0.0104	3.77E-09	0.0449	NA	170756	rs1490454	329443	ieu-b-102	TRUE	reported	WYslv6
6	28534833	C	G	0.0629	0.0108	4.97E-09	0.0426	NA	170756	rs1395820	329443	ieu-b-102	TRUE	reported	WYslv6
6	28537908	T	C	0.0591	0.0102	7.25E-09	0.0465	NA	170756	rs1562027	329443	ieu-b-102	TRUE	reported	WYslv6
6	28548674	G	A	0.0626	0.0107	5.34E-09	0.0427	NA	170756	rs1469186	329443	ieu-b-102	TRUE	reported	WYslv6
6	28678607	T	C	0.0631	0.0099	1.57E-10	0.0499	NA	170756	rs1400418	329443	ieu-b-102	TRUE	reported	WYslv6
6	28682406	G	C	0.0628	0.01	3.87E-10	0.0482	NA	170756	rs1450975	329443	ieu-b-102	TRUE	reported	WYslv6
6	28684506	G	A	0.0632	0.0099	1.52E-10	0.0498	NA	170756	rs1140514	329443	ieu-b-102	TRUE	reported	WYslv6
6	28686006	C	G	0.0631	0.01	3.19E-10	0.0483	NA	170756	rs1154995	329443	ieu-b-102	TRUE	reported	WYslv6
6	28696772	T	G	0.0642	0.0099	9.18E-11	0.0494	NA	170756	rs1508146	329443	ieu-b-102	TRUE	reported	WYslv6
6	28696816	C	A	0.0641	0.0101	2.07E-10	0.0479	NA	170756	rs1148076	329443	ieu-b-102	TRUE	reported	WYslv6
6	28700457	G	C	0.0645	0.0101	1.59E-10	0.0479	NA	170756	rs1485841	329443	ieu-b-102	TRUE	reported	WYslv6
6	28720609	A	G	0.0628	0.01	3.65E-10	0.0481	NA	170756	rs1167406	329443	ieu-b-102	TRUE	reported	WYslv6
6	28725240	C	T	0.0646	0.01	9.46E-11	0.0492	NA	170756	rs1485455	329443	ieu-b-102	TRUE	reported	WYslv6
6	28745638	C	T	0.0614	0.01	8.02E-10	0.0494	NA	170756	rs1480923	329443	ieu-b-102	TRUE	reported	WYslv6
6	28755650	C	T	0.0519	0.0089	5.42E-09	0.0621	NA	170756	rs1163589	329443	ieu-b-102	TRUE	reported	WYslv6
6	28755896	G	A	0.0519	0.0089	5.27E-09	0.062	NA	170756	rs1151744	329443	ieu-b-102	TRUE	reported	WYslv6
6	28756136	G	A	0.0519	0.0089	5.13E-09	0.062	NA	170756	rs1150927	329443	ieu-b-102	TRUE	reported	WYslv6
6	28757581	C	T	0.0624	0.0102	9.06E-10	0.0476	NA	170756	rs1423322	329443	ieu-b-102	TRUE	reported	WYslv6
6	28761844	T	C	0.0624	0.01	4.91E-10	0.0481	NA	170756	rs1505308	329443	ieu-b-102	TRUE	reported	WYslv6
6	28763730	A	G	0.0625	0.01	4.54E-10	0.0482	NA	170756	rs3752809	329443	ieu-b-102	TRUE	reported	WYslv6
6	28763796	C	T	0.06	0.0098	7.84E-10	0.0511	NA	170756	rs5388398	329443	ieu-b-102	TRUE	reported	WYslv6
6	28763825	G	A	0.0505	0.0089	1.69E-08	0.0624	NA	170756	rs1163039	329443	ieu-b-102	TRUE	reported	WYslv6
6	28764097	G	C	0.0527	0.0089	2.67E-09	0.0628	NA	170756	rs1406198	329443	ieu-b-102	TRUE	reported	WYslv6
6	28764668	C	G	0.0526	0.0089	3.35E-09	0.062	NA	170756	rs1147412	329443	ieu-b-102	TRUE	reported	WYslv6
6	28765033	G	T	0.0525	0.0089	3.58E-09	0.0621	NA	170756	rs1148471	329443	ieu-b-102	TRUE	reported	WYslv6
6	28766845	G	A	0.0531	0.0089	2.28E-09	0.0622	NA	170756	rs1466749	329443	ieu-b-102	TRUE	reported	WYslv6
6	28771300	G	A	0.0629	0.0099	1.71E-10	0.0499	NA	170756	rs1423956	329443	ieu-b-102	TRUE	reported	WYslv6
6	28772128	G	C	0.0535	0.0089	1.67E-09	0.0621	NA	170756	rs1140600	329443	ieu-b-102	TRUE	reported	WYslv6
6	28774130	A	T	0.0535	0.0089	1.69E-09	0.0621	NA	170756	rs1159670	329443	ieu-b-102	TRUE	reported	WYslv6
6	28774422	C	T	0.0535	0.0089	1.69E-09	0.0621	NA	170756	rs1153859	329443	ieu-b-102	TRUE	reported	WYslv6
6	28775155	A	G	0.0535	0.0089	1.69E-09	0.0621	NA	170756	rs1153073	329443	ieu-b-102	TRUE	reported	WYslv6
6	28777991	G	A	0.0629	0.0099	1.81E-10	0.0499	NA	170756	rs1147418	329443	ieu-b-102	TRUE	reported	WYslv6
6	28783772	T	C	0.0633	0.01	2.61E-10	0.0483	NA	170756	rs1473743	329443	ieu-b-102	TRUE	reported	WYslv6
6	28791481	G	A	0.063	0.0099	1.62E-10	0.0499	NA	170756	rs1483976	329443	ieu-b-102	TRUE	reported	WYslv6
6	28797097	A	G	0.0635	0.01	2.37E-10	0.0482	NA	170756	rs1141467	329443	ieu-b-102	TRUE	reported	WYslv6
6	28814098	A	G	0.0633	0.0098	1.28E-10	0.05	NA	170756	rs1155318	329443	ieu-b-102	TRUE	reported	WYslv6
6	28818951	A	G	0.0634	0.0098	1.24E-10	0.05	NA	170756	rs1474068	329443	ieu-b-102	TRUE	reported	WYslv6
6	28821169	C	A	0.0615	0.0099	6.00E-10	0.0501	NA	170756	rs1380923	329443	ieu-b-102	TRUE	reported	WYslv6
6	28828010	T	C	0.0638	0.0098	8.86E-11	0.0501	NA	170756	rs1163803	329443	ieu-b-102	TRUE	reported	WYslv6
6	28834598	C	T	0.0645	0.0102	2.65E-10	0.0474	NA	170756	rs1492360	329443	ieu-b-102	TRUE	reported	WYslv6
6	28837658	A	C	0.0631	0.0098	1.29E-10	0.0501	NA	170756	rs1502980	329443	ieu-b-102	TRUE	reported	WYslv6
6	28839632	G	C	0.0623	0.0098	2.15E-10	0.0504	NA	170756	rs1427358	329443	ieu-b-102	TRUE	reported	WYslv6
6	28884159	G	A	0.0637	0.01	2.05E-10	0.0486	NA	170756	rs1502920	329443	ieu-b-102	TRUE	reported	WYslv6
6	28894755	C	G	0.0645	0.0098	3.95E-11	0.0533	NA	170756	rs1414255	329443	ieu-b-102	TRUE	reported	WYslv6
6	28898526	A	G	0.0632	0.01	2.79E-10	0.0483	NA	170756	rs1146275	329443	ieu-b-102	TRUE	reported	WYslv6

6	28904710	G	C	0.0633	0.01	2.27E-10	0.0487	NA	170756	rs1449658	329443	ieu-b-102	TRUE	reported	WYslv6
6	28921061	A	T	0.0625	0.01	4.25E-10	0.0483	NA	170756	rs1428808	329443	ieu-b-102	TRUE	reported	WYslv6
6	28926891	C	T	0.0625	0.01	4.31E-10	0.0484	NA	170756	rs1389107	329443	ieu-b-102	TRUE	reported	WYslv6
6	28941134	C	T	0.0681	0.0106	1.44E-10	0.0443	NA	170756	rs1491421	329443	ieu-b-102	TRUE	reported	WYslv6
6	28941628	C	T	0.061	0.0098	5.72E-10	0.0502	NA	170756	rs1167081	329443	ieu-b-102	TRUE	reported	WYslv6
6	28945166	C	G	0.0599	0.01	2.01E-09	0.049	NA	170756	rs5718292	329443	ieu-b-102	TRUE	reported	WYslv6
6	28965512	G	A	0.0596	0.01	2.20E-09	0.0489	NA	170756	rs1143570	329443	ieu-b-102	TRUE	reported	WYslv6
6	28983572	A	T	0.0601	0.0103	5.84E-09	0.0475	NA	170756	rs1562378	329443	ieu-b-102	TRUE	reported	WYslv6
6	28983905	C	T	0.0587	0.01	3.79E-09	0.0489	NA	170756	rs1390595	329443	ieu-b-102	TRUE	reported	WYslv6
6	28984481	G	A	0.0585	0.01	4.52E-09	0.0489	NA	170756	rs1409552	329443	ieu-b-102	TRUE	reported	WYslv6
6	29003630	C	T	0.0593	0.0098	1.38E-09	0.0507	NA	170756	rs1403854	329443	ieu-b-102	TRUE	reported	WYslv6
6	29003820	A	C	0.0593	0.0098	1.39E-09	0.0506	NA	170756	rs1485462	329443	ieu-b-102	TRUE	reported	WYslv6
6	29062551	A	G	0.0591	0.0099	2.47E-09	0.0498	NA	170756	rs1151661	329443	ieu-b-102	TRUE	reported	WYslv6
6	29071904	A	C	0.0597	0.0098	1.02E-09	0.0506	NA	170756	rs1425351	329443	ieu-b-102	TRUE	reported	WYslv6
6	29090028	C	T	0.0585	0.0099	3.72E-09	0.0492	NA	170756	rs1158176	329443	ieu-b-102	TRUE	reported	WYslv6
6	29105249	C	T	0.0584	0.0099	3.75E-09	0.0494	NA	170756	rs1378768	329443	ieu-b-102	TRUE	reported	WYslv6
6	29111742	T	C	0.0587	0.0099	3.13E-09	0.0494	NA	170756	rs1462523	329443	ieu-b-102	TRUE	reported	WYslv6
6	29120756	G	A	0.0589	0.0099	2.83E-09	0.0494	NA	170756	rs1419172	329443	ieu-b-102	TRUE	reported	WYslv6
6	29165037	C	A	0.069	0.0107	1.00E-10	0.0441	NA	170756	rs1142245	329443	ieu-b-102	TRUE	reported	WYslv6
6	29169377	G	A	0.0585	0.0097	1.67E-09	0.0516	NA	170756	rs1562689	329443	ieu-b-102	TRUE	reported	WYslv6
6	29169515	A	G	0.0583	0.0097	1.85E-09	0.0517	NA	170756	rs1153748	329443	ieu-b-102	TRUE	reported	WYslv6
6	29196418	G	A	0.0606	0.0099	1.06E-09	0.0493	NA	170756	rs1153834	329443	ieu-b-102	TRUE	reported	WYslv6
6	29218513	C	T	0.0592	0.0097	9.48E-10	0.0519	NA	170756	rs1497540	329443	ieu-b-102	TRUE	reported	WYslv6
6	29222229	A	G	0.0592	0.0097	9.50E-10	0.0519	NA	170756	rs1158963	329443	ieu-b-102	TRUE	reported	WYslv6
6	29225025	T	G	0.0593	0.0097	9.01E-10	0.0519	NA	170756	rs1497023	329443	ieu-b-102	TRUE	reported	WYslv6
6	29226953	A	G	0.059	0.0097	1.04E-09	0.0519	NA	170756	rs1165081	329443	ieu-b-102	TRUE	reported	WYslv6
6	29245503	C	G	0.0579	0.0097	2.55E-09	0.0517	NA	170756	rs1489504	329443	ieu-b-102	TRUE	reported	WYslv6
6	29259509	G	A	0.0613	0.0097	3.06E-10	0.0512	NA	170756	rs1451082	329443	ieu-b-102	TRUE	reported	WYslv6
6	29262341	C	T	0.0612	0.0098	3.43E-10	0.0511	NA	170756	rs1165676	329443	ieu-b-102	TRUE	reported	WYslv6
6	29288001	G	A	0.0599	0.0099	1.31E-09	0.05	NA	170756	rs1486829	329443	ieu-b-102	TRUE	reported	WYslv6
6	29317177	C	T	0.0693	0.0106	5.96E-11	0.0443	NA	170756	rs1484668	329443	ieu-b-102	TRUE	reported	WYslv6
6	29339635	T	C	0.0565	0.0097	6.24E-09	0.0518	NA	170756	rs1153253	329443	ieu-b-102	TRUE	reported	WYslv6
6	29341041	T	C	0.0543	0.0096	1.69E-08	0.0527	NA	170756	rs1416150	329443	ieu-b-102	TRUE	reported	WYslv6
6	29341967	C	T	0.0561	0.0097	7.55E-09	0.0518	NA	170756	rs1438572	329443	ieu-b-102	TRUE	reported	WYslv6
6	29448128	A	G	0.0589	0.0093	2.80E-10	0.0563	NA	170756	rs1144061	329443	ieu-b-102	TRUE	reported	WYslv6
6	29659591	C	T	0.0648	0.0094	5.25E-12	0.058	NA	170756	rs1148820	329443	ieu-b-102	TRUE	reported	WYslv6
6	29725013	A	G	0.0641	0.0092	3.00E-12	0.0599	NA	170756	rs1159382	329443	ieu-b-102	TRUE	reported	WYslv6
6	29867140	C	T	0.0805	0.0141	1.08E-08	0.0349	NA	170756	rs1152674	329443	ieu-b-102	TRUE	reported	WYslv6
6	30216186	C	T	0.0364	0.0059	7.28E-10	0.1628	NA	170756	rs1397470	329443	ieu-b-102	TRUE	reported	WYslv6
6	31102618	C	G	-0.0446	0.0061	3.71E-13	0.1461	NA	170756	rs3130566	329443	ieu-b-102	TRUE	reported	WYslv6
6	31102790	G	T	-0.0445	0.0061	4.50E-13	0.1462	NA	170756	rs3132566	329443	ieu-b-102	TRUE	reported	WYslv6
6	31103195	C	G	-0.0445	0.0061	4.65E-13	0.1462	NA	170756	rs3094669	329443	ieu-b-102	TRUE	reported	WYslv6
6	31296388	G	C	-0.0509	0.0069	1.94E-13	0.1189	NA	170756	rs1140463	329443	ieu-b-102	TRUE	reported	WYslv6
6	31321267	A	C	-0.0453	0.0064	1.15E-12	0.1335	NA	170756	rs2596500	329443	ieu-b-102	TRUE	reported	WYslv6
6	31323953	C	G	0.0262	0.0046	9.83E-09	0.6372	NA	170756	rs1131285	329443	ieu-b-102	TRUE	reported	WYslv6
6	31323960	G	C	0.0265	0.0046	7.20E-09	0.6366	NA	170756	rs7970049	329443	ieu-b-102	TRUE	reported	WYslv6
6	31324892	G	C	0.0273	0.0044	6.33E-10	0.5466	NA	170756	rs7658759	329443	ieu-b-102	TRUE	reported	WYslv6
6	31324953	C	T	0.0246	0.0045	4.79E-08	0.6269	NA	170756	rs9266207	329443	ieu-b-102	TRUE	reported	WYslv6
6	31325341	G	C	0.0252	0.0046	3.25E-08	0.6526	NA	170756	rs6736697	329443	ieu-b-102	TRUE	reported	WYslv6
6	31325380	C	T	0.025	0.0045	3.75E-08	0.6506	NA	170756	rs9266231	329443	ieu-b-102	TRUE	reported	WYslv6
6	31325494	T	C	0.0255	0.0045	1.90E-08	0.6455	NA	170756	rs9266234	329443	ieu-b-102	TRUE	reported	WYslv6
6	31325565	G	A	0.0253	0.0045	2.63E-08	0.6447	NA	170756	rs9266241	329443	ieu-b-102	TRUE	reported	WYslv6
6	31325620	T	C	0.0247	0.0045	4.92E-08	0.6508	NA	170756	rs9266242	329443	ieu-b-102	TRUE	reported	WYslv6
6	31325692	G	A	0.0284	0.0048	3.92E-09	0.7183	NA	170756	rs9266244	329443	ieu-b-102	TRUE	reported	WYslv6
6	31325702	A	G	0.0284	0.0048	3.92E-09	0.7183	NA	170756	rs9266245	329443	ieu-b-102	TRUE	reported	WYslv6
6	31326794	A	T	0.0253	0.0043	5.45E-09	0.5372	NA	170756	rs1894743	329443	ieu-b-102	TRUE	reported	WYslv6
6	31328092	A	G	0.0281	0.0048	5.09E-09	0.7186	NA	170756	rs2523582	329443	ieu-b-102	TRUE	reported	WYslv6
6	31328542	G	A	0.0281	0.0048	4.71E-09	0.7186	NA	170756	rs2523578	329443	ieu-b-102	TRUE	reported	WYslv6
6	31329921	T	G	-0.0315	0.0052	1.09E-09	0.2254	NA	170756	rs2523569	329443	ieu-b-102	TRUE	reported	WYslv6
6	31329932	G	A	-0.0314	0.0052	1.16E-09	0.2252	NA	170756	rs2523568	329443	ieu-b-102	TRUE	reported	WYslv6
6	31546850	G	A	-0.043	0.0065	3.24E-11	0.1282	NA	170756	rs1800628	329443	ieu-b-102	TRUE	reported	WYslv6
6	31558702	C	T	-0.0425	0.0064	3.98E-11	0.13	NA	170756	rs3130063	329443	ieu-b-102	TRUE	reported	WYslv6
6	31783507	G	C	-0.025	0.0045	2.07E-08	0.3754	NA	170756	rs7613120	329443	ieu-b-102	TRUE	reported	WYslv6
6	31840477	C	T	-0.0405	0.0066	1.03E-09	0.1233	NA	170756	rs501942	329443	ieu-b-102	TRUE	reported	WYslv6
6	31916400	A	G	0.0255	0.0045	1.39E-08	0.6452	NA	170756	rs537160	329443	ieu-b-102	TRUE	reported	WYslv6
6	32577633	G	C	-0.0373	0.0065	7.50E-09	0.1345	NA	170756	rs535777	329443	ieu-b-102	TRUE	reported	WYslv6
6	66534041	G	T	-0.0286	0.005	1.17E-08	0.2466	NA	170756	rs6817005	329443	ieu-b-102	TRUE	reported	WYslv6
6	66624249	C	A	-0.0248	0.0045	4.48E-08	0.3419	NA	170756	rs9345731	329443	ieu-b-102	TRUE	reported	WYslv6
6	67000001	A	G	-0.0261	0.0045	8.56E-09	0.6466	NA	170756	rs2214123	329443	ieu-b-102	TRUE	reported	WYslv6
6	67001525	T	C	0.0276	0.005	4.15E-08	0.2432	NA	170756	rs1220403	329443	ieu-b-102	TRUE	reported	WYslv6
6	67001573	A	G	0.0276	0.005	3.89E-08	0.2434	NA	170756	rs1220259	329443	ieu-b-102	TRUE	reported	WYslv6
6	67004973	C	T	0.0281	0.0051	2.81E-08	0.243	NA	170756	rs1764407	329443	ieu-b-102	TRUE	reported	WYslv6
6	67005130	G	T	0.028	0.0051	3.05E-08	0.2431	NA	170756	rs1221617	329443	ieu-b-102	TRUE	reported	WYslv6
6	67008262	G	A	0.0279	0.005	2.91E-08	0.2488	NA	170756	rs9345808	329443	ieu-b-102	TRUE	reported	WYslv6
6	1.43E+08	G	A	0.0239	0.0043	2.73E-08	0.4875	NA	170756	rs7740440	329443	ieu-b-102	TRUE	reported	WYslv6
6	1.43E+08	C	T	0.0238	0.0043	3.68E-08	0.4624	NA	170756	rs2175165	329443	ieu-b-102	TRUE	reported	WYslv6
6	1.43E+08	A	C	0.0238	0.0043	3.03E-08	0.4896	NA	170756	rs5437418	329443	ieu-b-102	TRUE	reported	WYslv6
6	1.43E+08	T	G	0.0244	0.0043	1.40E-08	0.484	NA	170756	rs1848750	329443	ieu-b-102	TRUE	reported	WYslv6
6	1.43E+08	A	G	0.0238	0.0043	3.66E-08	0.4603	NA	170756	rs1396898	329443	ieu-b-102	TRUE	reported	WYslv6
6	1.43E+08	A	G	0.0237	0.0043	3.45E-08	0.4884	NA	170756	rs7753052	329443	ieu-b-102	TRUE	reported	WYslv6
6	1.43E+08	C	G	0.026	0.0043	2.24E-09	0.4688	NA	170756	rs2876520	329443	ieu-b-102	TRUE	reported	WYslv6
6	1.65E+08	C	T	0.028	0.0051	4.47E-08	0.2265	NA	170756	rs9365753	329443	ieu-b-102	TRUE	reported	WYslv6
6	1.65E+08	T	C	0.0281	0.0051	4.35E-08	0.2265	NA	170756	rs7724742	329443	ieu-b-102	TRUE	reported	WYslv6
6	1.65E+08	C	T	0.0282	0.0051	3.73E-08	0.226	NA	170756	rs9347903	329443	ieu-b-102	TRUE	reported	WYslv6
6	1.65E+08	A	G	0.0283	0.0051	3.49E-08	0.2262	NA	170756	rs9364755	329443	ieu-b-102	TRUE	reported	WYslv6
6	1.65E+08	C	T	0.0281	0.0051	4.12E-08	0.2262	NA	170756	rs9356244	329443	ieu-b-102	TRUE	reported	WYslv6
6	1.65E+08	T	C	0.0261	0.0048	4.19E-08	0.2839	NA	170756	rs9347028	329443	ieu-b-102	TRUE	reported	WYslv6
7	2085165	T	C	-0.027	0.0049	4.90E-08	0.2533	NA	170756	rs7797112	329443	ieu-b-102	TRUE	reported	WYslv6
7	2086814	T	C	-0.027	0.0049	4.68E-08	0.2529	NA	170756	rs1023566	329443	ieu-b-102	TRUE	reported	WYslv6
7	12247330	T	C	0.0271	0.0045	2.47E-09	0.3369	NA	170756	rs6460894	329443	ieu-b-102	TRUE	reported	WYslv6
7	12250378	G	A	0.0307	0.0044	1.89E-12	0.4093	NA	170756	rs1234864	329443	ieu-b-102	TRUE	reported	WYslv6
7	12250402	G	A	0.031	0.0044	1.09E-12	0.4105	NA	170756	rs3807865	329443	ieu-b-102	TRUE	reported	WYslv6
7	12251560	C	G	0.0306	0.0044	2.14E-12	0.4145	NA	170756	rs7781670	329443	ieu-b-102	TRUE	reported	WYslv6
7	12251671	T	A	0.0305	0.0044	2.37E-12	0.4145	NA	170756	rs3867103	329443	ieu-b-102	TRUE	reported	WYslv6
7	12251790	G	C	0.0305	0.0044	2.29E-12	0.4148	NA	170756	rs1019307	329443	ieu-b-102	TRUE	reported	WYslv6
7	12252540	C	G	0.0297	0.0043	8.80E-12	0.4184	NA	170756	rs6460895	329443	ieu-b-102	TRUE	reported	WYslv6
7	12252659	G	A	0.0298	0.0043	7.63E-12	0.4185	NA	170756	rs6460896	329443	ieu-b-102	TRUE	reported	WYslv6
7	12252741	T	G	0.0298	0.0043	7.72E-12	0.4185	NA	170756	rs6460897	329443	ieu-b-102	TRUE	reported	WYslv6
7	12252893	A	G	0.0297	0.0043	8.04E-12	0.4185	NA	170756	rs6460898	329443	ieu-b-102	TRUE	reported	WYslv6
7	12253050	A	C	0.0298	0.0043	7.35E-12	0.4185	NA	170756	rs6460899	329443	ieu-b-102	TRUE	reported	WYslv6
7	12253088	A	G	0.0297	0.0043	7.97E-12	0.4184	NA	170756	rs6460900	329443	ieu-b-102	TRUE	reported	WYslv6
7	12253362	T	C	0.0297	0.0043	7.87E-12	0.4185	NA	170756	rs1269932	329443	ieu-b-102	TRUE	reported	WYslv6
7	12253372	T	C	0.0297	0.0043	8.14E-12	0.4185	NA	170756	rs1353660	329443	ieu-b-102	TRUE	reported	WYslv6
7	12253543	T	C	0.0297	0.0043	6.97E-12	0.4184	NA	170756	rs1023480	329443	ieu-b-102	TRUE	reported	WYslv6
7	12253563	C	T	0.0298	0.0043	7.10E-12	0.4184	NA	170756	rs1027825	329443	ieu-b-102	TRUE	reported	WYslv6
7	12253880	G	A	0.0299	0.0043	5.42E-12	0.4179	NA	170756	rs2043539	329443	ieu-b-102	TRUE	reported	WYslv6
7	12253893	T	C	0.0272	0.0046	3.48E-09	0.3174	NA	170756	rs2043538	329443	ieu-b-102	TRUE	reported	WYslv6

7	12254939	C	T	0.0267	0.0046	6.56E-09	0.3162	NA	170756	rs1714989	329443	ieu-b-102	TRUE	reported	WYslv6
7	12255434	C	T	0.0295	0.0043	1.02E-11	0.4175	NA	170756	rs6460901	329443	ieu-b-102	TRUE	reported	WYslv6
7	12255511	G	A	0.0294	0.0043	1.23E-11	0.4177	NA	170756	rs6460902	329443	ieu-b-102	TRUE	reported	WYslv6
7	12255664	G	A	0.0295	0.0043	1.14E-11	0.4175	NA	170756	rs1020006	329443	ieu-b-102	TRUE	reported	WYslv6
7	12255778	T	C	0.0268	0.0046	6.29E-09	0.3149	NA	170756	rs1020004	329443	ieu-b-102	TRUE	reported	WYslv6
7	12256540	A	G	0.0269	0.0046	5.41E-09	0.3159	NA	170756	rs1197438	329443	ieu-b-102	TRUE	reported	WYslv6
7	12256548	T	C	0.0295	0.0043	1.06E-11	0.4175	NA	170756	rs1197113	329443	ieu-b-102	TRUE	reported	WYslv6
7	12257527	C	T	0.0295	0.0043	1.24E-11	0.4191	NA	170756	rs7785189	329443	ieu-b-102	TRUE	reported	WYslv6
7	12257549	A	G	0.0294	0.0043	1.17E-11	0.4176	NA	170756	rs1375874	329443	ieu-b-102	TRUE	reported	WYslv6
7	12258755	G	C	0.0293	0.0043	1.68E-11	0.4169	NA	170756	rs3823612	329443	ieu-b-102	TRUE	reported	WYslv6
7	12260546	C	T	0.0269	0.0046	6.65E-09	0.3166	NA	170756	rs1266624	329443	ieu-b-102	TRUE	reported	WYslv6
7	12261829	A	G	0.0293	0.0043	1.51E-11	0.4176	NA	170756	rs1095039	329443	ieu-b-102	TRUE	reported	WYslv6
7	12262242	G	A	0.0292	0.0043	1.76E-11	0.4177	NA	170756	rs3800847	329443	ieu-b-102	TRUE	reported	WYslv6
7	12262571	C	T	0.0291	0.0043	1.96E-11	0.4176	NA	170756	rs1435527	329443	ieu-b-102	TRUE	reported	WYslv6
7	12262717	T	C	0.0291	0.0043	1.98E-11	0.4177	NA	170756	rs1435525	329443	ieu-b-102	TRUE	reported	WYslv6
7	12262801	G	A	0.0289	0.0043	2.80E-11	0.4174	NA	170756	rs1435524	329443	ieu-b-102	TRUE	reported	WYslv6
7	12263378	G	T	0.0291	0.0044	2.79E-11	0.4177	NA	170756	rs1197433	329443	ieu-b-102	TRUE	reported	WYslv6
7	12263437	C	T	0.0292	0.0043	1.76E-11	0.4172	NA	170756	rs1197430	329443	ieu-b-102	TRUE	reported	WYslv6
7	12263538	T	C	0.0291	0.0043	2.38E-11	0.4169	NA	170756	rs1095039	329443	ieu-b-102	TRUE	reported	WYslv6
7	12263546	T	C	0.0292	0.0043	1.74E-11	0.4166	NA	170756	rs1095039	329443	ieu-b-102	TRUE	reported	WYslv6
7	12263587	C	T	0.029	0.0043	2.50E-11	0.4172	NA	170756	rs1095039	329443	ieu-b-102	TRUE	reported	WYslv6
7	12263799	T	C	0.029	0.0044	5.57E-11	0.4042	NA	170756	rs8798235	329443	ieu-b-102	TRUE	reported	WYslv6
7	12264297	G	A	0.029	0.0043	2.40E-11	0.4177	NA	170756	rs3800843	329443	ieu-b-102	TRUE	reported	WYslv6
7	12264467	T	C	0.029	0.0043	2.41E-11	0.4177	NA	170756	rs1095039	329443	ieu-b-102	TRUE	reported	WYslv6
7	12264666	T	C	0.0289	0.0043	2.53E-11	0.4177	NA	170756	rs1095039	329443	ieu-b-102	TRUE	reported	WYslv6
7	12264737	C	T	0.0293	0.0044	2.14E-11	0.4114	NA	170756	rs1095039	329443	ieu-b-102	TRUE	reported	WYslv6
7	12264871	G	A	0.0296	0.0044	1.25E-11	0.4143	NA	170756	rs1095039	329443	ieu-b-102	TRUE	reported	WYslv6
7	12265059	A	G	0.0299	0.0044	8.97E-12	0.4151	NA	170756	rs7685415	329443	ieu-b-102	TRUE	reported	WYslv6
7	12265192	T	C	0.0294	0.0044	1.42E-11	0.4174	NA	170756	rs1504650	329443	ieu-b-102	TRUE	reported	WYslv6
7	12265739	C	G	0.0267	0.0046	7.39E-09	0.3148	NA	170756	rs6966602	329443	ieu-b-102	TRUE	reported	WYslv6
7	12265848	T	C	0.0292	0.0043	1.70E-11	0.4172	NA	170756	rs6948844	329443	ieu-b-102	TRUE	reported	WYslv6
7	12265952	A	T	0.029	0.0043	2.22E-11	0.4174	NA	170756	rs6966757	329443	ieu-b-102	TRUE	reported	WYslv6
7	12265988	C	T	0.0291	0.0044	2.37E-11	0.4143	NA	170756	rs6966915	329443	ieu-b-102	TRUE	reported	WYslv6
7	12266706	G	A	0.0289	0.0043	2.51E-11	0.4179	NA	170756	rs7804433	329443	ieu-b-102	TRUE	reported	WYslv6
7	12266814	A	G	0.029	0.0043	2.30E-11	0.4175	NA	170756	rs7804234	329443	ieu-b-102	TRUE	reported	WYslv6
7	12266867	G	T	0.0289	0.0043	2.90E-11	0.415	NA	170756	rs7804736	329443	ieu-b-102	TRUE	reported	WYslv6
7	12267221	A	G	0.0291	0.0043	1.97E-11	0.4177	NA	170756	rs4721057	329443	ieu-b-102	TRUE	reported	WYslv6
7	12267256	C	T	0.0291	0.0043	2.30E-11	0.415	NA	170756	rs4721058	329443	ieu-b-102	TRUE	reported	WYslv6
7	12267495	G	C	0.0288	0.0043	3.01E-11	0.415	NA	170756	rs4721059	329443	ieu-b-102	TRUE	reported	WYslv6
7	12267552	G	A	0.029	0.0044	2.70E-11	0.4139	NA	170756	rs4721060	329443	ieu-b-102	TRUE	reported	WYslv6
7	12267559	G	C	0.0288	0.0044	3.48E-11	0.4138	NA	170756	rs4721061	329443	ieu-b-102	TRUE	reported	WYslv6
7	12267614	T	C	0.029	0.0043	2.29E-11	0.4143	NA	170756	rs7792410	329443	ieu-b-102	TRUE	reported	WYslv6
7	12267734	G	A	0.0291	0.0044	2.30E-11	0.4143	NA	170756	rs7809700	329443	ieu-b-102	TRUE	reported	WYslv6
7	12267837	A	G	0.029	0.0044	2.54E-11	0.4145	NA	170756	rs1322998	329443	ieu-b-102	TRUE	reported	WYslv6
7	12268117	C	T	0.0291	0.0043	2.06E-11	0.4143	NA	170756	rs2854983	329443	ieu-b-102	TRUE	reported	WYslv6
7	12268243	C	A	0.0291	0.0043	1.96E-11	0.4143	NA	170756	rs1323051	329443	ieu-b-102	TRUE	reported	WYslv6
7	12268468	G	T	0.0291	0.0043	2.03E-11	0.4143	NA	170756	rs2022275	329443	ieu-b-102	TRUE	reported	WYslv6
7	12268551	G	A	0.0291	0.0044	2.28E-11	0.4143	NA	170756	rs5011431	329443	ieu-b-102	TRUE	reported	WYslv6
7	12268668	A	C	0.0299	0.0044	6.94E-12	0.4103	NA	170756	rs5011432	329443	ieu-b-102	TRUE	reported	WYslv6
7	12268717	C	T	0.0291	0.0044	2.29E-11	0.4132	NA	170756	rs5011434	329443	ieu-b-102	TRUE	reported	WYslv6
7	12268758	A	C	0.029	0.0043	2.67E-11	0.4154	NA	170756	rs5011436	329443	ieu-b-102	TRUE	reported	WYslv6
7	12268792	A	T	0.0297	0.0044	1.19E-11	0.4123	NA	170756	rs5011437	329443	ieu-b-102	TRUE	reported	WYslv6
7	12268798	C	T	0.0296	0.0044	1.26E-11	0.4123	NA	170756	rs3867103	329443	ieu-b-102	TRUE	reported	WYslv6
7	12269417	C	G	0.029	0.0043	2.18E-11	0.4144	NA	170756	rs3173615	329443	ieu-b-102	TRUE	reported	WYslv6
7	12269575	G	C	0.0293	0.0044	1.61E-11	0.4141	NA	170756	rs1323771	329443	ieu-b-102	TRUE	reported	WYslv6
7	12269593	C	A	0.0293	0.0044	2.09E-11	0.4147	NA	170756	rs1323751	329443	ieu-b-102	TRUE	reported	WYslv6
7	12269762	G	T	0.0291	0.0044	2.18E-11	0.4143	NA	170756	rs1269933	329443	ieu-b-102	TRUE	reported	WYslv6
7	12269804	C	T	0.0291	0.0043	2.12E-11	0.4143	NA	170756	rs1269933	329443	ieu-b-102	TRUE	reported	WYslv6
7	12269817	G	A	0.0291	0.0044	2.21E-11	0.4144	NA	170756	rs1266862	329443	ieu-b-102	TRUE	reported	WYslv6
7	12270519	G	A	0.0292	0.0044	2.00E-11	0.4143	NA	170756	rs3815535	329443	ieu-b-102	TRUE	reported	WYslv6
7	12270625	G	A	0.0291	0.0043	2.12E-11	0.4143	NA	170756	rs2302635	329443	ieu-b-102	TRUE	reported	WYslv6
7	12270770	T	A	0.0291	0.0044	2.18E-11	0.4143	NA	170756	rs2302634	329443	ieu-b-102	TRUE	reported	WYslv6
7	12270815	C	T	0.0291	0.0044	2.11E-11	0.4143	NA	170756	rs2302633	329443	ieu-b-102	TRUE	reported	WYslv6
7	12270967	A	G	0.0265	0.0046	8.96E-09	0.315	NA	170756	rs2302632	329443	ieu-b-102	TRUE	reported	WYslv6
7	12271252	T	C	0.029	0.0043	2.28E-11	0.4175	NA	170756	rs7808568	329443	ieu-b-102	TRUE	reported	WYslv6
7	12271997	G	A	0.0291	0.0044	2.14E-11	0.4143	NA	170756	rs1266991	329443	ieu-b-102	TRUE	reported	WYslv6
7	12272116	C	G	0.0292	0.0044	2.01E-11	0.4144	NA	170756	rs1042949	329443	ieu-b-102	TRUE	reported	WYslv6
7	12272568	A	T	0.0291	0.0043	1.93E-11	0.4143	NA	170756	rs3800841	329443	ieu-b-102	TRUE	reported	WYslv6
7	12273152	A	G	0.0265	0.0046	8.98E-09	0.3161	NA	170756	rs14978	329443	ieu-b-102	TRUE	reported	WYslv6
7	12273413	C	T	0.0292	0.0043	1.87E-11	0.4143	NA	170756	rs1054168	329443	ieu-b-102	TRUE	reported	WYslv6
7	12273496	G	A	0.0292	0.0043	1.81E-11	0.4142	NA	170756	rs1054169	329443	ieu-b-102	TRUE	reported	WYslv6
7	12275508	T	C	0.0291	0.0043	1.91E-11	0.4177	NA	170756	rs1468804	329443	ieu-b-102	TRUE	reported	WYslv6
7	12275675	A	C	0.029	0.0043	2.21E-11	0.4178	NA	170756	rs1198538	329443	ieu-b-102	TRUE	reported	WYslv6
7	12275818	A	G	0.0291	0.0043	1.92E-11	0.4178	NA	170756	rs1060700	329443	ieu-b-102	TRUE	reported	WYslv6
7	12276011	G	A	0.0292	0.0043	1.81E-11	0.4178	NA	170756	rs1468802	329443	ieu-b-102	TRUE	reported	WYslv6
7	12276045	G	C	0.0292	0.0043	1.97E-11	0.4173	NA	170756	rs1468801	329443	ieu-b-102	TRUE	reported	WYslv6
7	12276885	T	C	0.0292	0.0044	1.93E-11	0.4143	NA	170756	rs2356065	329443	ieu-b-102	TRUE	reported	WYslv6
7	12277020	T	C	0.0265	0.0046	9.13E-09	0.3151	NA	170756	rs1269933	329443	ieu-b-102	TRUE	reported	WYslv6
7	12277773	G	A	0.0293	0.0044	1.77E-11	0.414	NA	170756	rs7797705	329443	ieu-b-102	TRUE	reported	WYslv6
7	12278437	G	A	0.0264	0.0046	9.73E-09	0.3155	NA	170756	rs6969722	329443	ieu-b-102	TRUE	reported	WYslv6
7	12279559	G	C	0.0265	0.0046	9.02E-09	0.315	NA	170756	rs1548885	329443	ieu-b-102	TRUE	reported	WYslv6
7	12279761	A	C	0.0292	0.0044	1.94E-11	0.4145	NA	170756	rs1562714	329443	ieu-b-102	TRUE	reported	WYslv6
7	12280730	T	A	0.0292	0.0044	2.16E-11	0.4144	NA	170756	rs1028142	329443	ieu-b-102	TRUE	reported	WYslv6
7	12280838	A	G	0.0266	0.0046	8.16E-09	0.3153	NA	170756	rs1023636	329443	ieu-b-102	TRUE	reported	WYslv6
7	12282451	T	C	0.0294	0.0044	1.47E-11	0.4132	NA	170756	rs7805419	329443	ieu-b-102	TRUE	reported	WYslv6
7	12283227	A	G	0.0292	0.0044	2.02E-11	0.4144	NA	170756	rs6460905	329443	ieu-b-102	TRUE	reported	WYslv6
7	12283250	T	A	0.0264	0.0046	1.07E-08	0.3152	NA	170756	rs1396845	329443	ieu-b-102	TRUE	reported	WYslv6
7	12283276	T	C	0.0292	0.0044	2.08E-11	0.4144	NA	170756	rs6460906	329443	ieu-b-102	TRUE	reported	WYslv6
7	12283329	G	C	0.0292	0.0044	2.07E-11	0.4145	NA	170756	rs7791726	329443	ieu-b-102	TRUE	reported	WYslv6
7	12283787	A	G	0.0291	0.0044	2.27E-11	0.4144	NA	170756	rs1990622	329443	ieu-b-102	TRUE	reported	WYslv6
7	12283873	C	G	0.0285	0.0044	6.24E-11	0.4138	NA	170756	rs1990621	329443	ieu-b-102	TRUE	reported	WYslv6
7	12284008	A	G	0.0291	0.0044	2.32E-11	0.4144	NA	170756	rs1990620	329443	ieu-b-102	TRUE	reported	WYslv6
7	12284378	G	A	0.0279	0.0044	1.92E-10	0.4012	NA	170756	rs4721064	329443	ieu-b-102	TRUE	reported	WYslv6
7	12285140	C	T	0.0303	0.0044	5.56E-12	0.4128	NA	170756	rs1269933	329443	ieu-b-102	TRUE	reported	WYslv6
7	12286050	A	G	0.0269	0.0046	6.29E-09	0.3149	NA	170756	rs1023539	329443	ieu-b-102	TRUE	reported	WYslv6
7	38724868	C	T	0.0363	0.0066	3.07E-08	0.1342	NA	170756	rs5908293	329443	ieu-b-102	TRUE	reported	WYslv6
7	82448100	T	C	0.024	0.0043	2.11E-08	0.4739	NA	170756	rs2522831	329443	ieu-b-102	TRUE	reported	WYslv6
7	82450364	A	G	0.024	0.0043	2.38E-08	0.4739	NA	170756	rs2522832	329443	ieu-b-102	TRUE	reported	WYslv6
7	82454404	C	G	0.0243	0.0043	1.71E-08	0.47	NA	170756	rs1304113	329443	ieu-b-102	TRUE	reported	WYslv6
7	82454672	T	C	0.0238	0.0043	3.02E-08	0.4736	NA	170756	rs2371214	329443	ieu-b-102	TRUE	reported	WYslv6
7	82473053	C	A	0.0236	0.0043	4.52E-08	0.4717	NA	170756	rs2522836	329443	ieu-b-102	TRUE	reported	WYslv6
7	1.09E+08	G	A	0.0237	0.0043	4.71E-08	0.4668	NA	170756	rs5810418	329443	ieu-b-102	TRUE	reported	WYslv6
7	1.09E+08	T	A	0.0238	0.0043	4.12E-08	0.4659	NA	170756	rs4730387	329443	ieu-b-102	TRUE	reported	WYslv6
7	1.09E+08	G	T	-0.0237	0.0043	4.49E-08	0.5506	NA	170756	rs1283070	329443	ieu-b-102	TRUE	reported	WYslv6
7	1.17E+08	A	G	0.0281	0.0045	4.22E-10	0.6349	NA	170756	rs2402267	329443	ieu-b-102	TRUE	reported	WYslv6
7	1.17E+08	C	T	0.0277	0.0045	5.49E-10	0.6349	NA	170756	rs1024503	329443	ieu-b-102	TRUE	reported	WYslv6
7	1.18E+08	C	T	0.0274	0.0043	2.38E-10	0.5483	NA	170756	rs7807677	329443	ieu-b-102	TRUE	reported	WYslv6

7	1.18E+08	G	A	0.027	0.0043	4.44E-10	0.5471	NA	170756	rs7796203	329443	ieu-b-102	TRUE	reported	WYslv6
7	1.18E+08	C	G	0.027	0.0043	4.33E-10	0.5497	NA	170756	rs3589563	329443	ieu-b-102	TRUE	reported	WYslv6
7	1.18E+08	C	T	0.027	0.0045	2.25E-09	0.6509	NA	170756	rs7801539	329443	ieu-b-102	TRUE	reported	WYslv6
7	1.18E+08	G	C	0.027	0.0045	2.40E-09	0.6509	NA	170756	rs7801931	329443	ieu-b-102	TRUE	reported	WYslv6
7	1.18E+08	T	C	0.027	0.0045	2.24E-09	0.6509	NA	170756	rs7794364	329443	ieu-b-102	TRUE	reported	WYslv6
7	1.18E+08	A	G	0.0271	0.0045	2.01E-09	0.6508	NA	170756	rs7795627	329443	ieu-b-102	TRUE	reported	WYslv6
7	1.18E+08	C	T	0.0271	0.0045	2.07E-09	0.6509	NA	170756	rs7795873	329443	ieu-b-102	TRUE	reported	WYslv6
7	1.18E+08	A	G	0.027	0.0043	4.44E-10	0.5502	NA	170756	rs1215487	329443	ieu-b-102	TRUE	reported	WYslv6
7	1.18E+08	G	T	0.0271	0.0045	1.98E-09	0.6509	NA	170756	rs7796616	329443	ieu-b-102	TRUE	reported	WYslv6
7	1.18E+08	C	G	0.0272	0.0044	4.02E-10	0.5521	NA	170756	rs7809993	329443	ieu-b-102	TRUE	reported	WYslv6
7	1.18E+08	T	G	0.027	0.0043	4.42E-10	0.5498	NA	170756	rs6965740	329443	ieu-b-102	TRUE	reported	WYslv6
7	1.18E+08	T	C	0.0273	0.0045	1.51E-09	0.6493	NA	170756	rs6466628	329443	ieu-b-102	TRUE	reported	WYslv6
7	1.18E+08	A	C	0.0276	0.0043	1.71E-10	0.5459	NA	170756	rs2193255	329443	ieu-b-102	TRUE	reported	WYslv6
7	1.18E+08	C	T	0.0273	0.0045	1.38E-09	0.651	NA	170756	rs1024254	329443	ieu-b-102	TRUE	reported	WYslv6
7	1.18E+08	G	T	0.0276	0.0043	1.69E-10	0.5459	NA	170756	rs1024275	329443	ieu-b-102	TRUE	reported	WYslv6
7	1.18E+08	A	G	0.026	0.0043	1.44E-09	0.5094	NA	170756	rs1023301	329443	ieu-b-102	TRUE	reported	WYslv6
7	1.18E+08	C	G	0.0273	0.0045	1.49E-09	0.6511	NA	170756	rs1027096	329443	ieu-b-102	TRUE	reported	WYslv6
7	1.18E+08	C	T	0.0273	0.0045	1.49E-09	0.6511	NA	170756	rs7788766	329443	ieu-b-102	TRUE	reported	WYslv6
7	1.18E+08	A	G	0.0272	0.0045	1.69E-09	0.6509	NA	170756	rs1012084	329443	ieu-b-102	TRUE	reported	WYslv6
7	1.18E+08	A	T	0.0271	0.0045	1.86E-09	0.6509	NA	170756	rs1861637	329443	ieu-b-102	TRUE	reported	WYslv6
7	1.18E+08	C	T	0.0243	0.0043	1.70E-08	0.462	NA	170756	rs1748872	329443	ieu-b-102	TRUE	reported	WYslv6
7	1.18E+08	C	A	0.0242	0.0043	1.90E-08	0.462	NA	170756	rs1025085	329443	ieu-b-102	TRUE	reported	WYslv6
7	1.18E+08	T	C	0.0241	0.0043	2.18E-08	0.4621	NA	170756	rs1026699	329443	ieu-b-102	TRUE	reported	WYslv6
7	1.18E+08	C	T	0.0242	0.0043	2.05E-08	0.4621	NA	170756	rs1477114	329443	ieu-b-102	TRUE	reported	WYslv6
7	1.18E+08	T	G	0.0243	0.0043	1.65E-08	0.4619	NA	170756	rs7789130	329443	ieu-b-102	TRUE	reported	WYslv6
7	1.18E+08	A	C	0.0243	0.0043	1.79E-08	0.4619	NA	170756	rs1270615	329443	ieu-b-102	TRUE	reported	WYslv6
7	1.18E+08	A	G	0.0242	0.0043	1.89E-08	0.4618	NA	170756	rs2193257	329443	ieu-b-102	TRUE	reported	WYslv6
7	1.18E+08	A	G	0.0241	0.0043	2.13E-08	0.4621	NA	170756	rs6466630	329443	ieu-b-102	TRUE	reported	WYslv6
7	1.18E+08	C	A	0.027	0.0043	3.45E-10	0.4998	NA	170756	rs9013229	329443	ieu-b-102	TRUE	reported	WYslv6
7	1.18E+08	A	G	0.0241	0.0043	2.14E-08	0.4621	NA	170756	rs6466632	329443	ieu-b-102	TRUE	reported	WYslv6
7	1.18E+08	T	C	0.0242	0.0043	1.89E-08	0.4621	NA	170756	rs6964051	329443	ieu-b-102	TRUE	reported	WYslv6
7	1.18E+08	C	T	0.0242	0.0043	1.83E-08	0.4621	NA	170756	rs1270616	329443	ieu-b-102	TRUE	reported	WYslv6
7	1.18E+08	C	G	0.0243	0.0043	1.69E-08	0.4622	NA	170756	rs1266851	329443	ieu-b-102	TRUE	reported	WYslv6
7	1.18E+08	C	T	0.0243	0.0043	1.59E-08	0.4622	NA	170756	rs1548460	329443	ieu-b-102	TRUE	reported	WYslv6
7	1.18E+08	T	C	0.0272	0.0043	2.64E-10	0.4998	NA	170756	rs1548461	329443	ieu-b-102	TRUE	reported	WYslv6
7	1.18E+08	A	T	0.0243	0.0043	1.51E-08	0.4623	NA	170756	rs6466636	329443	ieu-b-102	TRUE	reported	WYslv6
7	1.18E+08	T	C	0.0241	0.0043	1.94E-08	0.4623	NA	170756	rs1027292	329443	ieu-b-102	TRUE	reported	WYslv6
7	1.18E+08	C	T	0.0242	0.0043	1.74E-08	0.4623	NA	170756	rs6950622	329443	ieu-b-102	TRUE	reported	WYslv6
7	1.18E+08	C	A	0.0241	0.0043	1.94E-08	0.4619	NA	170756	rs1197805	329443	ieu-b-102	TRUE	reported	WYslv6
7	1.18E+08	C	T	0.0242	0.0043	1.85E-08	0.4619	NA	170756	rs6971964	329443	ieu-b-102	TRUE	reported	WYslv6
7	1.18E+08	C	G	0.0241	0.0043	1.97E-08	0.4621	NA	170756	rs2158050	329443	ieu-b-102	TRUE	reported	WYslv6
7	1.18E+08	G	T	0.0243	0.0043	1.80E-08	0.461	NA	170756	rs1024432	329443	ieu-b-102	TRUE	reported	WYslv6
7	1.18E+08	T	C	0.0241	0.0043	2.18E-08	0.4617	NA	170756	rs1024433	329443	ieu-b-102	TRUE	reported	WYslv6
7	1.18E+08	C	A	0.0242	0.0043	1.79E-08	0.4619	NA	170756	rs1024434	329443	ieu-b-102	TRUE	reported	WYslv6
7	1.18E+08	A	T	0.0242	0.0043	1.77E-08	0.462	NA	170756	rs6950716	329443	ieu-b-102	TRUE	reported	WYslv6
7	1.18E+08	G	A	0.0242	0.0043	1.88E-08	0.462	NA	170756	rs6247181	329443	ieu-b-102	TRUE	reported	WYslv6
7	1.18E+08	A	G	0.0242	0.0043	1.81E-08	0.462	NA	170756	rs6959314	329443	ieu-b-102	TRUE	reported	WYslv6
7	1.18E+08	A	G	0.0242	0.0043	1.97E-08	0.462	NA	170756	rs1322130	329443	ieu-b-102	TRUE	reported	WYslv6
7	1.18E+08	G	A	0.0242	0.0043	1.71E-08	0.4622	NA	170756	rs6966339	329443	ieu-b-102	TRUE	reported	WYslv6
7	1.18E+08	A	G	0.0269	0.0044	6.69E-10	0.4183	NA	170756	rs916784	329443	ieu-b-102	TRUE	reported	WYslv6
7	1.18E+08	T	C	0.0272	0.0044	5.02E-10	0.4155	NA	170756	rs2402273	329443	ieu-b-102	TRUE	reported	WYslv6
7	1.18E+08	A	G	0.0267	0.0044	9.21E-10	0.4187	NA	170756	rs1176835	329443	ieu-b-102	TRUE	reported	WYslv6
7	1.18E+08	G	C	0.0269	0.0044	7.09E-10	0.4148	NA	170756	rs1013278	329443	ieu-b-102	TRUE	reported	WYslv6
7	1.18E+08	G	A	0.0268	0.0044	8.62E-10	0.4147	NA	170756	rs1468183	329443	ieu-b-102	TRUE	reported	WYslv6
7	1.18E+08	G	C	0.0267	0.0044	9.65E-10	0.4147	NA	170756	rs2843869	329443	ieu-b-102	TRUE	reported	WYslv6
7	1.18E+08	C	G	0.0266	0.0044	1.02E-09	0.4187	NA	170756	rs1343857	329443	ieu-b-102	TRUE	reported	WYslv6
7	1.18E+08	A	G	0.0265	0.0044	1.14E-09	0.4185	NA	170756	rs7784849	329443	ieu-b-102	TRUE	reported	WYslv6
7	1.18E+08	C	G	0.0265	0.0044	1.22E-09	0.4146	NA	170756	rs1211391	329443	ieu-b-102	TRUE	reported	WYslv6
7	1.18E+08	C	T	0.0266	0.0044	1.06E-09	0.4142	NA	170756	rs1028070	329443	ieu-b-102	TRUE	reported	WYslv6
7	1.18E+08	A	C	0.0265	0.0044	1.20E-09	0.4141	NA	170756	rs1023723	329443	ieu-b-102	TRUE	reported	WYslv6
7	1.18E+08	C	T	0.0265	0.0044	1.16E-09	0.4144	NA	170756	rs2402295	329443	ieu-b-102	TRUE	reported	WYslv6
7	1.18E+08	T	C	0.0265	0.0044	1.16E-09	0.4144	NA	170756	rs1022699	329443	ieu-b-102	TRUE	reported	WYslv6
7	1.18E+08	T	A	0.0265	0.0044	1.21E-09	0.4144	NA	170756	rs2871427	329443	ieu-b-102	TRUE	reported	WYslv6
7	1.18E+08	C	T	0.0265	0.0044	1.21E-09	0.4144	NA	170756	rs7793280	329443	ieu-b-102	TRUE	reported	WYslv6
7	1.18E+08	G	C	0.0264	0.0044	1.29E-09	0.4143	NA	170756	rs1211180	329443	ieu-b-102	TRUE	reported	WYslv6
7	1.18E+08	A	G	0.0275	0.0044	2.63E-10	0.4167	NA	170756	rs1027082	329443	ieu-b-102	TRUE	reported	WYslv6
7	1.18E+08	C	T	0.0276	0.0044	2.62E-10	0.4129	NA	170756	rs1026498	329443	ieu-b-102	TRUE	reported	WYslv6
7	1.18E+08	C	T	0.0283	0.0044	1.16E-10	0.4118	NA	170756	rs1503469	329443	ieu-b-102	TRUE	reported	WYslv6
7	1.18E+08	C	G	0.0275	0.0044	3.06E-10	0.4128	NA	170756	rs1756851	329443	ieu-b-102	TRUE	reported	WYslv6
7	1.18E+08	G	T	0.0262	0.0046	8.53E-09	0.3366	NA	170756	rs6170243	329443	ieu-b-102	TRUE	reported	WYslv6
7	1.18E+08	T	A	0.0276	0.0044	2.83E-10	0.4129	NA	170756	rs970185	329443	ieu-b-102	TRUE	reported	WYslv6
7	1.18E+08	C	T	0.0274	0.0044	3.61E-10	0.4129	NA	170756	rs1028203	329443	ieu-b-102	TRUE	reported	WYslv6
7	1.18E+08	C	T	0.0238	0.0044	4.64E-08	0.4231	NA	170756	rs2188836	329443	ieu-b-102	TRUE	reported	WYslv6
9	11131371	A	T	-0.0292	0.0053	3.28E-08	0.2105	NA	170756	rs1095955	329443	ieu-b-102	TRUE	reported	WYslv6
9	11136403	G	A	-0.0315	0.0054	5.72E-09	0.1966	NA	170756	rs2151719	329443	ieu-b-102	TRUE	reported	WYslv6
9	11139180	A	T	-0.0319	0.0054	4.38E-09	0.1943	NA	170756	rs1095956	329443	ieu-b-102	TRUE	reported	WYslv6
9	11140428	G	C	-0.0318	0.0054	4.60E-09	0.1943	NA	170756	rs7865888	329443	ieu-b-102	TRUE	reported	WYslv6
9	11140883	A	G	-0.032	0.0054	3.89E-09	0.195	NA	170756	rs7866090	329443	ieu-b-102	TRUE	reported	WYslv6
9	11141119	G	A	-0.0319	0.0054	4.53E-09	0.1943	NA	170756	rs7869639	329443	ieu-b-102	TRUE	reported	WYslv6
9	11142482	A	G	-0.0318	0.0054	4.65E-09	0.1942	NA	170756	rs1237912	329443	ieu-b-102	TRUE	reported	WYslv6
9	11142732	C	A	-0.0318	0.0054	4.99E-09	0.1944	NA	170756	rs1237644	329443	ieu-b-102	TRUE	reported	WYslv6
9	11142835	C	G	-0.0319	0.0054	4.14E-09	0.1942	NA	170756	rs1237646	329443	ieu-b-102	TRUE	reported	WYslv6
9	11144237	C	T	-0.032	0.0054	4.06E-09	0.1942	NA	170756	rs5898205	329443	ieu-b-102	TRUE	reported	WYslv6
9	11145582	C	T	-0.0321	0.0054	3.35E-09	0.1944	NA	170756	rs1237757	329443	ieu-b-102	TRUE	reported	WYslv6
9	11145717	T	C	-0.0321	0.0054	3.65E-09	0.1944	NA	170756	rs1080935	329443	ieu-b-102	TRUE	reported	WYslv6
9	11150555	G	C	0.0262	0.0044	1.78E-09	0.5812	NA	170756	rs1075619	329443	ieu-b-102	TRUE	reported	WYslv6
9	11152340	A	G	0.0259	0.0044	3.02E-09	0.5815	NA	170756	rs1316909	329443	ieu-b-102	TRUE	reported	WYslv6
9	11153277	C	T	0.0259	0.0044	3.00E-09	0.5803	NA	170756	rs1931415	329443	ieu-b-102	TRUE	reported	WYslv6
9	11154844	A	T	0.0259	0.0044	3.08E-09	0.5812	NA	170756	rs1002756	329443	ieu-b-102	TRUE	reported	WYslv6
9	11155055	G	T	-0.0442	0.0071	4.31E-10	0.1072	NA	170756	rs7269426	329443	ieu-b-102	TRUE	reported	WYslv6
9	11155468	T	C	0.0258	0.0044	3.40E-09	0.581	NA	170756	rs1095957	329443	ieu-b-102	TRUE	reported	WYslv6
9	11156999	A	C	0.0255	0.0044	5.51E-09	0.5819	NA	170756	rs1011744	329443	ieu-b-102	TRUE	reported	WYslv6
9	11158137	T	A	0.0258	0.0044	3.20E-09	0.5818	NA	170756	rs7048081	329443	ieu-b-102	TRUE	reported	WYslv6
9	11158345	T	C	0.0258	0.0044	3.29E-09	0.5818	NA	170756	rs1443485	329443	ieu-b-102	TRUE	reported	WYslv6
9	11158480	G	C	0.0258	0.0044	3.56E-09	0.5819	NA	170756	rs1931425	329443	ieu-b-102	TRUE	reported	WYslv6
9	11160620	A	G	0.0257	0.0044	3.71E-09	0.5817	NA	170756	rs945001	329443	ieu-b-102	TRUE	reported	WYslv6
9	11165729	C	G	-0.0307	0.0055	2.26E-08	0.1911	NA	170756	rs1095957	329443	ieu-b-102	TRUE	reported	WYslv6
9	11168641	C	G	-0.0312	0.0055	1.09E-08	0.1939	NA	170756	rs1095957	329443	ieu-b-102	TRUE	reported	WYslv6
9	11170509	C	T	-0.031	0.0055	1.54E-08	0.1934	NA	170756	rs1178779	329443	ieu-b-102	TRUE	reported	WYslv6
9	11170765	A	T	-0.031	0.0055	1.45E-08	0.1937	NA	170756	rs1290856	329443	ieu-b-102	TRUE	reported	WYslv6
9	11171013	T	C	-0.0314	0.0055	1.00E-08	0.1938	NA	170756	rs5586022	329443	ieu-b-102	TRUE	reported	WYslv6
9	11174784	T	C	-0.0302	0.0055	3.08E-08	0.1946	NA	170756	rs1095958	329443	ieu-b-102	TRUE	reported	WYslv6
9	11174994	A	G	-0.0301	0.0055	3.61E-08	0.1944	NA	170756	rs1095958	329443	ieu-b-102	TRUE	reported	WYslv6
9	11179005	C	T	-0.0309	0.0054	7.76E-09	0.2081	NA	170756	rs5607065	329443	ieu-b-102	TRUE	reported	WYslv6
9	11179452	G	T	-0.0307	0.0053	9.00E-09	0.2079	NA	170756	rs1095958	329443	ieu-b-102	TRUE	reported	WYslv6
9	11180739	A	G	-0.0309	0.0053	6.04E-09	0.2089	NA	170756	rs1238019	329443	ieu-b-102	TRUE	reported	WYslv6
9	11180908	A	G	-0.0314	0.0053	3.60E-09	0.2077	NA	170756	rs7856376	329443	ieu-b-102	TRUE	reported	WYslv6

9	11181046	C	T	-0.031	0.0053	5.15E-09	0.2088	NA	170756	rs7859480	329443	ieu-b-102	TRUE	reported	WYslv6
9	11185487	A	G	-0.0313	0.0053	3.69E-09	0.209	NA	170756	rs1237666	329443	ieu-b-102	TRUE	reported	WYslv6
9	11186820	C	A	-0.0312	0.0053	4.02E-09	0.2101	NA	170756	rs7269428	329443	ieu-b-102	TRUE	reported	WYslv6
9	11186828	A	C	-0.0312	0.0053	4.14E-09	0.21	NA	170756	rs7269428	329443	ieu-b-102	TRUE	reported	WYslv6
9	11187013	G	A	-0.031	0.0053	5.07E-09	0.209	NA	170756	rs1238010	329443	ieu-b-102	TRUE	reported	WYslv6
9	11188613	G	T	-0.031	0.0053	4.94E-09	0.209	NA	170756	rs7269429	329443	ieu-b-102	TRUE	reported	WYslv6
9	11189845	T	C	-0.0305	0.0053	1.01E-08	0.209	NA	170756	rs1095960	329443	ieu-b-102	TRUE	reported	WYslv6
9	11193902	A	T	-0.031	0.0053	5.11E-09	0.2092	NA	170756	rs7269429	329443	ieu-b-102	TRUE	reported	WYslv6
9	11195988	G	C	-0.0307	0.0053	6.92E-09	0.209	NA	170756	rs7269430	329443	ieu-b-102	TRUE	reported	WYslv6
9	11196733	C	T	-0.0268	0.0044	8.05E-10	0.428	NA	170756	rs2130121	329443	ieu-b-102	TRUE	reported	WYslv6
9	11202702	C	T	-0.0294	0.0044	1.93E-11	0.4035	NA	170756	rs1178891	329443	ieu-b-102	TRUE	reported	WYslv6
9	11203149	A	G	-0.0295	0.0044	1.68E-11	0.4042	NA	170756	rs1931388	329443	ieu-b-102	TRUE	reported	WYslv6
9	11203611	T	G	-0.03	0.0052	8.33E-09	0.2195	NA	170756	rs1237821	329443	ieu-b-102	TRUE	reported	WYslv6
9	11203919	G	A	0.0237	0.0043	3.59E-08	0.4982	NA	170756	rs1011691	329443	ieu-b-102	TRUE	reported	WYslv6
9	11204004	C	T	0.0238	0.0043	3.34E-08	0.4982	NA	170756	rs7852252	329443	ieu-b-102	TRUE	reported	WYslv6
9	11205022	G	C	-0.03	0.0052	9.31E-09	0.2183	NA	170756	rs1095961	329443	ieu-b-102	TRUE	reported	WYslv6
9	11207039	C	G	-0.0302	0.0052	6.56E-09	0.2195	NA	170756	rs1095961	329443	ieu-b-102	TRUE	reported	WYslv6
9	11208711	A	G	-0.0304	0.0052	5.73E-09	0.2193	NA	170756	rs1095962	329443	ieu-b-102	TRUE	reported	WYslv6
9	11208995	C	A	-0.0293	0.0044	2.62E-11	0.4036	NA	170756	rs1080938	329443	ieu-b-102	TRUE	reported	WYslv6
9	11210897	A	G	-0.0305	0.0052	4.74E-09	0.2191	NA	170756	rs4457429	329443	ieu-b-102	TRUE	reported	WYslv6
9	11211186	T	C	-0.0303	0.0052	6.22E-09	0.2195	NA	170756	rs7269621	329443	ieu-b-102	TRUE	reported	WYslv6
9	11211722	G	C	-0.0301	0.0052	7.59E-09	0.219	NA	170756	rs1095962	329443	ieu-b-102	TRUE	reported	WYslv6
9	11214620	A	G	-0.0296	0.0053	2.21E-08	0.2159	NA	170756	rs1095962	329443	ieu-b-102	TRUE	reported	WYslv6
9	11217308	C	G	-0.029	0.0052	2.30E-08	0.2212	NA	170756	rs1179145	329443	ieu-b-102	TRUE	reported	WYslv6
9	11218170	A	G	-0.0287	0.0052	3.10E-08	0.2212	NA	170756	rs1178808	329443	ieu-b-102	TRUE	reported	WYslv6
9	11219740	C	T	-0.0301	0.0052	6.78E-09	0.2195	NA	170756	rs7269622	329443	ieu-b-102	TRUE	reported	WYslv6
9	11221407	G	A	-0.0302	0.0052	6.29E-09	0.2196	NA	170756	rs1095963	329443	ieu-b-102	TRUE	reported	WYslv6
9	11221604	G	T	-0.0302	0.0052	6.42E-09	0.2196	NA	170756	rs1095963	329443	ieu-b-102	TRUE	reported	WYslv6
9	11221999	A	G	-0.029	0.0052	2.11E-08	0.2212	NA	170756	rs1489185	329443	ieu-b-102	TRUE	reported	WYslv6
9	11222778	A	G	-0.0299	0.0052	8.49E-09	0.2197	NA	170756	rs1095963	329443	ieu-b-102	TRUE	reported	WYslv6
9	11223385	G	C	0.0236	0.0043	3.91E-08	0.491	NA	170756	rs1906746	329443	ieu-b-102	TRUE	reported	WYslv6
9	11224603	T	C	0.0236	0.0043	4.09E-08	0.4949	NA	170756	rs1080939	329443	ieu-b-102	TRUE	reported	WYslv6
9	11225712	T	C	-0.0292	0.0052	2.19E-08	0.2174	NA	170756	rs8796037	329443	ieu-b-102	TRUE	reported	WYslv6
9	11227070	G	T	0.0238	0.0043	3.05E-08	0.4943	NA	170756	rs1044565	329443	ieu-b-102	TRUE	reported	WYslv6
9	11228608	A	G	-0.0235	0.0043	4.66E-08	0.5055	NA	170756	rs9298667	329443	ieu-b-102	TRUE	reported	WYslv6
9	11229140	A	T	0.0236	0.0043	4.32E-08	0.4944	NA	170756	rs2225670	329443	ieu-b-102	TRUE	reported	WYslv6
9	11229986	C	T	-0.0397	0.0068	5.97E-09	0.1159	NA	170756	rs5599350	329443	ieu-b-102	TRUE	reported	WYslv6
9	11230551	T	C	0.0235	0.0043	4.79E-08	0.4947	NA	170756	rs1075620	329443	ieu-b-102	TRUE	reported	WYslv6
9	11230856	A	C	-0.0297	0.0052	1.12E-08	0.2192	NA	170756	rs7269623	329443	ieu-b-102	TRUE	reported	WYslv6
9	11232061	T	C	0.0237	0.0043	3.77E-08	0.4945	NA	170756	rs7041114	329443	ieu-b-102	TRUE	reported	WYslv6
9	11233609	T	C	0.0244	0.0043	1.43E-08	0.4931	NA	170756	rs1075620	329443	ieu-b-102	TRUE	reported	WYslv6
9	11233634	C	T	0.0238	0.0043	3.18E-08	0.4931	NA	170756	rs1075621	329443	ieu-b-102	TRUE	reported	WYslv6
9	11233740	T	G	-0.0297	0.0052	1.20E-08	0.2193	NA	170756	rs1080939	329443	ieu-b-102	TRUE	reported	WYslv6
9	11234635	A	G	-0.0296	0.0052	1.25E-08	0.2192	NA	170756	rs7034246	329443	ieu-b-102	TRUE	reported	WYslv6
9	11234802	C	T	-0.0298	0.0052	1.01E-08	0.2188	NA	170756	rs901988	329443	ieu-b-102	TRUE	reported	WYslv6
9	11235049	C	G	0.0239	0.0043	2.63E-08	0.4945	NA	170756	rs1398388	329443	ieu-b-102	TRUE	reported	WYslv6
9	11235823	T	A	-0.0298	0.0052	1.03E-08	0.2189	NA	170756	rs1237711	329443	ieu-b-102	TRUE	reported	WYslv6
9	11236474	T	G	-0.0303	0.0052	4.54E-09	0.2244	NA	170756	rs1011833	329443	ieu-b-102	TRUE	reported	WYslv6
9	11237297	A	G	-0.0297	0.0052	1.18E-08	0.2181	NA	170756	rs7046663	329443	ieu-b-102	TRUE	reported	WYslv6
9	11237650	G	A	-0.0297	0.0052	1.16E-08	0.2181	NA	170756	rs1095964	329443	ieu-b-102	TRUE	reported	WYslv6
9	11238710	C	T	-0.0298	0.0052	1.10E-08	0.2174	NA	170756	rs7269624	329443	ieu-b-102	TRUE	reported	WYslv6
9	11239009	C	T	0.0239	0.0043	2.85E-08	0.4954	NA	170756	rs1489184	329443	ieu-b-102	TRUE	reported	WYslv6
9	11240173	T	C	0.0238	0.0043	3.07E-08	0.4953	NA	170756	rs1095965	329443	ieu-b-102	TRUE	reported	WYslv6
9	11240827	C	G	0.0239	0.0043	2.83E-08	0.4959	NA	170756	rs1095965	329443	ieu-b-102	TRUE	reported	WYslv6
9	11241807	A	G	-0.03	0.0052	6.37E-09	0.2231	NA	170756	rs3694754	329443	ieu-b-102	TRUE	reported	WYslv6
9	11241909	A	C	-0.0297	0.0052	1.16E-08	0.2181	NA	170756	rs745485	329443	ieu-b-102	TRUE	reported	WYslv6
9	11242515	C	G	-0.0299	0.0052	7.62E-09	0.2231	NA	170756	rs1080939	329443	ieu-b-102	TRUE	reported	WYslv6
9	11243270	C	T	0.024	0.0043	2.63E-08	0.4956	NA	170756	rs1075621	329443	ieu-b-102	TRUE	reported	WYslv6
9	11243667	G	A	0.0238	0.0043	3.15E-08	0.4952	NA	170756	rs1080939	329443	ieu-b-102	TRUE	reported	WYslv6
9	11244554	A	G	-0.0237	0.0043	3.70E-08	0.5046	NA	170756	rs1012354	329443	ieu-b-102	TRUE	reported	WYslv6
9	11245124	C	T	0.0238	0.0043	3.17E-08	0.4951	NA	170756	rs9644846	329443	ieu-b-102	TRUE	reported	WYslv6
9	11245272	T	C	-0.0299	0.0052	7.28E-09	0.2233	NA	170756	rs7871118	329443	ieu-b-102	TRUE	reported	WYslv6
9	11245459	G	C	-0.0297	0.0052	1.28E-08	0.2182	NA	170756	rs5616841	329443	ieu-b-102	TRUE	reported	WYslv6
9	11245779	T	C	-0.0297	0.0052	1.27E-08	0.2177	NA	170756	rs1095965	329443	ieu-b-102	TRUE	reported	WYslv6
9	11245830	A	G	-0.0298	0.0052	7.96E-09	0.2235	NA	170756	rs1012446	329443	ieu-b-102	TRUE	reported	WYslv6
9	11247241	C	T	0.0237	0.0043	3.59E-08	0.495	NA	170756	rs1080940	329443	ieu-b-102	TRUE	reported	WYslv6
9	11248964	C	T	0.0242	0.0043	2.39E-08	0.4956	NA	170756	rs1190082	329443	ieu-b-102	TRUE	reported	WYslv6
9	11250216	G	A	-0.0293	0.0052	1.89E-08	0.2182	NA	170756	rs7269625	329443	ieu-b-102	TRUE	reported	WYslv6
9	11250841	A	G	-0.0297	0.0052	1.41E-08	0.2178	NA	170756	rs7027175	329443	ieu-b-102	TRUE	reported	WYslv6
9	11251670	G	C	-0.0239	0.0043	3.54E-08	0.525	NA	170756	rs4741135	329443	ieu-b-102	TRUE	reported	WYslv6
9	11251849	T	C	-0.0301	0.0053	9.68E-09	0.2177	NA	170756	rs1151517	329443	ieu-b-102	TRUE	reported	WYslv6
9	11252959	T	G	0.0244	0.0043	1.78E-08	0.4748	NA	170756	rs1586578	329443	ieu-b-102	TRUE	reported	WYslv6
9	11254070	T	C	0.0242	0.0044	2.71E-08	0.4848	NA	170756	rs1387292	329443	ieu-b-102	TRUE	reported	WYslv6
9	11256041	T	C	-0.0297	0.0051	7.95E-09	0.2332	NA	170756	rs1151517	329443	ieu-b-102	TRUE	reported	WYslv6
9	11256275	C	T	-0.0299	0.0051	6.61E-09	0.2323	NA	170756	rs1305213	329443	ieu-b-102	TRUE	reported	WYslv6
9	11256918	A	G	-0.0292	0.0051	1.26E-08	0.235	NA	170756	rs1011718	329443	ieu-b-102	TRUE	reported	WYslv6
9	11257041	C	G	-0.0295	0.0052	1.19E-08	0.2305	NA	170756	rs1149629	329443	ieu-b-102	TRUE	reported	WYslv6
9	11276005	C	T	0.0245	0.0044	2.26E-08	0.5871	NA	170756	rs1075621	329443	ieu-b-102	TRUE	reported	WYslv6
9	11306553	G	A	-0.0286	0.0052	4.60E-08	0.217	NA	170756	rs1095971	329443	ieu-b-102	TRUE	reported	WYslv6
9	11321119	A	G	-0.0252	0.0044	8.23E-09	0.4114	NA	170756	rs1012464	329443	ieu-b-102	TRUE	reported	WYslv6
9	11321327	C	T	-0.0252	0.0044	8.31E-09	0.4098	NA	170756	rs1011862	329443	ieu-b-102	TRUE	reported	WYslv6
9	11321937	T	A	-0.0253	0.0044	7.48E-09	0.41	NA	170756	rs1080942	329443	ieu-b-102	TRUE	reported	WYslv6
9	11327345	T	G	-0.0286	0.0052	3.91E-08	0.222	NA	170756	rs1095972	329443	ieu-b-102	TRUE	reported	WYslv6
9	11327556	T	A	-0.0286	0.0052	3.78E-08	0.2223	NA	170756	rs1095972	329443	ieu-b-102	TRUE	reported	WYslv6
9	11339378	A	T	-0.0286	0.0052	4.68E-08	0.2155	NA	170756	rs1095973	329443	ieu-b-102	TRUE	reported	WYslv6
9	11373866	T	G	-0.0295	0.0053	2.13E-08	0.2134	NA	170756	rs1718520	329443	ieu-b-102	TRUE	reported	WYslv6
9	11379022	T	G	-0.0293	0.0052	2.01E-08	0.2181	NA	170756	rs1095975	329443	ieu-b-102	TRUE	reported	WYslv6
9	11379262	A	G	-0.0296	0.0053	1.86E-08	0.2131	NA	170756	rs1095975	329443	ieu-b-102	TRUE	reported	WYslv6
9	11379630	C	T	-0.0298	0.0053	1.64E-08	0.2131	NA	170756	rs1095975	329443	ieu-b-102	TRUE	reported	WYslv6
9	11380203	T	A	-0.0297	0.0053	1.81E-08	0.2131	NA	170756	rs1179208	329443	ieu-b-102	TRUE	reported	WYslv6
9	11381697	C	A	-0.0298	0.0053	1.57E-08	0.2131	NA	170756	rs7269813	329443	ieu-b-102	TRUE	reported	WYslv6
9	11381746	A	C	-0.0296	0.0053	1.84E-08	0.2132	NA	170756	rs6726992	329443	ieu-b-102	TRUE	reported	WYslv6
9	11382792	G	A	-0.0288	0.0052	3.27E-08	0.2198	NA	170756	rs1011956	329443	ieu-b-102	TRUE	reported	WYslv6
9	11384213	A	T	-0.0241	0.0044	4.48E-08	0.4009	NA	170756	rs1591444	329443	ieu-b-102	TRUE	reported	WYslv6
9	11384256	G	A	-0.0293	0.0052	2.24E-08	0.2149	NA	170756	rs1080944	329443	ieu-b-102	TRUE	reported	WYslv6
9	11386135	G	A	-0.0293	0.0052	2.18E-08	0.2149	NA	170756	rs1132921	329443	ieu-b-102	TRUE	reported	WYslv6
9	11390523	T	A	-0.0294	0.0052	1.77E-08	0.2178	NA	170756	rs1095975	329443	ieu-b-102	TRUE	reported	WYslv6
9	11391810	G	T	0.0287	0.0052	3.34E-08	0.7792	NA	170756	rs4645638	329443	ieu-b-102	TRUE	reported	WYslv6
9	11393305	G	A	0.0284	0.0052	4.65E-08	0.7792	NA	170756	rs1015663	329443	ieu-b-102	TRUE	reported	WYslv6
9	11394962	C	A	-0.0296	0.0052	1.74E-08	0.2145	NA	170756	rs1080945	329443	ieu-b-102	TRUE	reported	WYslv6
9	11396014	G	T	-0.0297	0.0052	1.49E-08	0.2143	NA	170756	rs1095976	329443	ieu-b-102	TRUE	reported	WYslv6
9	11396033	T	C	0.0293	0.0052	2.31E-08	0.7829	NA	170756	rs1154692	329443	ieu-b-102	TRUE	reported	WYslv6
9	11400078	A	G	-0.0301	0.0052	7.37E-09	0.2185	NA	170756	rs1095976	329443	ieu-b-102	TRUE	reported	WYslv6
9	11400254	C	G	-0.0296	0.0052	1.31E-08	0.2197	NA	170756	rs1012579	329443	ieu-b-102	TRUE	reported	WYslv6
9	11400376	T	G	-0.0296	0.0052	1.26E-08	0.2197	NA	170756	rs1011683	329443	ieu-b-102	TRUE	reported	WYslv6
9	11400557	T	C	-0.0286	0.0052	4.60E-08	0.2164	NA	170756	rs3734621	329443	ieu-b-102	TRUE	reported	WYslv6
9	11400661	T	C	-0.0296	0.0052	1.33E-08	0.219	NA	170756	rs1414215	329443	ieu-b-102	TRUE	reported	WYslv6

9	11401425	C	T	-0.0295	0.0052	1.40E-08	0.2188	NA	170756	rs2382296	329443	ieu-b-102	TRUE	reported	WYslv6
9	11401471	C	T	-0.0295	0.0052	1.38E-08	0.2188	NA	170756	rs1095976	329443	ieu-b-102	TRUE	reported	WYslv6
9	11402081	T	C	-0.0295	0.0052	1.50E-08	0.2189	NA	170756	rs1304172	329443	ieu-b-102	TRUE	reported	WYslv6
9	11402453	A	G	-0.0293	0.0052	1.66E-08	0.22	NA	170756	rs1012149	329443	ieu-b-102	TRUE	reported	WYslv6
9	11403179	G	T	-0.0245	0.0044	2.25E-08	0.4059	NA	170756	rs6252779	329443	ieu-b-102	TRUE	reported	WYslv6
9	11407277	T	C	-0.03	0.0052	8.04E-09	0.2186	NA	170756	rs1095977	329443	ieu-b-102	TRUE	reported	WYslv6
9	11408052	C	T	-0.024	0.0044	4.89E-08	0.3976	NA	170756	rs1095977	329443	ieu-b-102	TRUE	reported	WYslv6
9	11408315	T	C	-0.0301	0.0052	7.09E-09	0.2186	NA	170756	rs7269816	329443	ieu-b-102	TRUE	reported	WYslv6
9	11411676	T	G	-0.03	0.0052	8.44E-09	0.2188	NA	170756	rs5634638	329443	ieu-b-102	TRUE	reported	WYslv6
9	11413520	C	T	-0.0299	0.0052	8.55E-09	0.2188	NA	170756	rs2095486	329443	ieu-b-102	TRUE	reported	WYslv6
9	11415999	G	A	-0.0306	0.0052	4.23E-09	0.2174	NA	170756	rs7807731	329443	ieu-b-102	TRUE	reported	WYslv6
9	11416492	G	C	-0.0306	0.0052	4.21E-09	0.2177	NA	170756	rs2184546	329443	ieu-b-102	TRUE	reported	WYslv6
9	11417276	T	G	-0.0307	0.0052	3.71E-09	0.2174	NA	170756	rs1095978	329443	ieu-b-102	TRUE	reported	WYslv6
9	11417639	G	T	-0.0245	0.0044	2.12E-08	0.4053	NA	170756	rs6252780	329443	ieu-b-102	TRUE	reported	WYslv6
9	11417702	G	C	-0.0245	0.0044	2.07E-08	0.4053	NA	170756	rs1405608	329443	ieu-b-102	TRUE	reported	WYslv6
9	11419389	C	T	-0.0244	0.0044	2.52E-08	0.4051	NA	170756	rs6252781	329443	ieu-b-102	TRUE	reported	WYslv6
9	11421758	C	T	-0.0304	0.0052	6.13E-09	0.2168	NA	170756	rs1095978	329443	ieu-b-102	TRUE	reported	WYslv6
9	11421771	T	C	-0.0244	0.0044	2.63E-08	0.4048	NA	170756	rs7045135	329443	ieu-b-102	TRUE	reported	WYslv6
9	11422521	A	G	-0.0307	0.0052	4.01E-09	0.2172	NA	170756	rs1095978	329443	ieu-b-102	TRUE	reported	WYslv6
9	11423966	T	C	-0.0308	0.0052	3.68E-09	0.2173	NA	170756	rs5607203	329443	ieu-b-102	TRUE	reported	WYslv6
9	11425008	A	G	-0.0243	0.0044	2.70E-08	0.4052	NA	170756	rs4639602	329443	ieu-b-102	TRUE	reported	WYslv6
9	11425356	A	G	-0.0307	0.0052	4.06E-09	0.2173	NA	170756	rs4563977	329443	ieu-b-102	TRUE	reported	WYslv6
9	11426915	C	G	-0.0306	0.0052	4.42E-09	0.2173	NA	170756	rs1095979	329443	ieu-b-102	TRUE	reported	WYslv6
9	11428153	C	A	-0.0305	0.0052	4.75E-09	0.2173	NA	170756	rs1095979	329443	ieu-b-102	TRUE	reported	WYslv6
9	11429566	T	C	-0.0306	0.0052	4.41E-09	0.2172	NA	170756	rs1237658	329443	ieu-b-102	TRUE	reported	WYslv6
9	11431990	G	A	-0.0307	0.0052	4.21E-09	0.2173	NA	170756	rs1095979	329443	ieu-b-102	TRUE	reported	WYslv6
9	11435129	C	A	-0.0296	0.0052	1.29E-08	0.2187	NA	170756	rs1179525	329443	ieu-b-102	TRUE	reported	WYslv6
9	11438192	T	C	-0.0295	0.0052	1.38E-08	0.22	NA	170756	rs9407483	329443	ieu-b-102	TRUE	reported	WYslv6
9	11439306	C	T	-0.0243	0.0044	3.06E-08	0.4076	NA	170756	rs1114797	329443	ieu-b-102	TRUE	reported	WYslv6
9	11440848	A	C	-0.0295	0.0052	1.42E-08	0.2196	NA	170756	rs1080947	329443	ieu-b-102	TRUE	reported	WYslv6
9	11445540	G	A	-0.0307	0.0052	4.20E-09	0.218	NA	170756	rs1095981	329443	ieu-b-102	TRUE	reported	WYslv6
9	11445598	G	A	-0.0304	0.0052	5.35E-09	0.2184	NA	170756	rs1095981	329443	ieu-b-102	TRUE	reported	WYslv6
9	11445637	C	A	-0.0299	0.0052	8.66E-09	0.2197	NA	170756	rs1095981	329443	ieu-b-102	TRUE	reported	WYslv6
9	11445802	G	A	-0.0303	0.0052	5.74E-09	0.2187	NA	170756	rs1095981	329443	ieu-b-102	TRUE	reported	WYslv6
9	11447799	A	C	-0.0298	0.0052	9.00E-09	0.2202	NA	170756	rs1095982	329443	ieu-b-102	TRUE	reported	WYslv6
9	11448547	T	C	-0.0299	0.0052	8.63E-09	0.2203	NA	170756	rs1179385	329443	ieu-b-102	TRUE	reported	WYslv6
9	11449346	C	T	-0.0298	0.0052	9.02E-09	0.221	NA	170756	rs6255048	329443	ieu-b-102	TRUE	reported	WYslv6
9	11452034	A	G	-0.0301	0.0052	7.14E-09	0.2188	NA	170756	rs5605632	329443	ieu-b-102	TRUE	reported	WYslv6
9	11453149	A	G	-0.0301	0.0052	8.74E-09	0.2197	NA	170756	rs5611603	329443	ieu-b-102	TRUE	reported	WYslv6
9	11455272	G	A	-0.0301	0.0052	6.98E-09	0.2188	NA	170756	rs6255054	329443	ieu-b-102	TRUE	reported	WYslv6
9	11455955	C	T	-0.0301	0.0052	7.29E-09	0.2188	NA	170756	rs1049195	329443	ieu-b-102	TRUE	reported	WYslv6
9	11459140	A	G	-0.0297	0.0052	1.02E-08	0.2206	NA	170756	rs1095982	329443	ieu-b-102	TRUE	reported	WYslv6
9	11459410	G	A	-0.0301	0.0052	7.26E-09	0.2185	NA	170756	rs1095982	329443	ieu-b-102	TRUE	reported	WYslv6
9	11461549	G	A	-0.0297	0.0052	1.07E-08	0.2207	NA	170756	rs1095983	329443	ieu-b-102	TRUE	reported	WYslv6
9	11461669	C	A	-0.0297	0.0052	1.08E-08	0.2206	NA	170756	rs1095983	329443	ieu-b-102	TRUE	reported	WYslv6
9	11465508	A	T	-0.0297	0.0052	9.92E-09	0.2205	NA	170756	rs1095983	329443	ieu-b-102	TRUE	reported	WYslv6
9	11471297	G	A	-0.0301	0.0052	1.02E-08	0.2151	NA	170756	rs5640218	329443	ieu-b-102	TRUE	reported	WYslv6
9	11475063	T	G	-0.0292	0.0052	2.00E-08	0.2202	NA	170756	rs5606715	329443	ieu-b-102	TRUE	reported	WYslv6
9	11482326	G	C	-0.0297	0.0052	1.13E-08	0.2208	NA	170756	rs1126920	329443	ieu-b-102	TRUE	reported	WYslv6
9	11483986	T	C	-0.0294	0.0052	1.38E-08	0.2207	NA	170756	rs1151517	329443	ieu-b-102	TRUE	reported	WYslv6
9	11484286	A	G	-0.0296	0.0052	1.22E-08	0.2207	NA	170756	rs1152123	329443	ieu-b-102	TRUE	reported	WYslv6
9	11484364	C	G	-0.0295	0.0052	1.27E-08	0.2206	NA	170756	rs1095985	329443	ieu-b-102	TRUE	reported	WYslv6
9	11484979	T	C	-0.0295	0.0052	1.35E-08	0.2206	NA	170756	rs1095985	329443	ieu-b-102	TRUE	reported	WYslv6
9	11485612	C	T	-0.0293	0.0052	1.58E-08	0.2206	NA	170756	rs1095985	329443	ieu-b-102	TRUE	reported	WYslv6
9	11487280	T	C	-0.0293	0.0052	1.67E-08	0.2206	NA	170756	rs1095986	329443	ieu-b-102	TRUE	reported	WYslv6
9	11487789	G	A	-0.0293	0.0052	1.64E-08	0.2205	NA	170756	rs1080949	329443	ieu-b-102	TRUE	reported	WYslv6
9	11490048	A	T	-0.0292	0.0052	1.90E-08	0.2205	NA	170756	rs6255253	329443	ieu-b-102	TRUE	reported	WYslv6
9	11490521	T	C	-0.0291	0.0052	1.99E-08	0.2205	NA	170756	rs1718863	329443	ieu-b-102	TRUE	reported	WYslv6
9	11491500	C	T	-0.0292	0.0052	1.82E-08	0.2205	NA	170756	rs1049195	329443	ieu-b-102	TRUE	reported	WYslv6
9	11493421	A	T	-0.0292	0.0052	1.79E-08	0.2203	NA	170756	rs1718891	329443	ieu-b-102	TRUE	reported	WYslv6
9	11493575	G	A	-0.0292	0.0052	1.76E-08	0.2203	NA	170756	rs1718894	329443	ieu-b-102	TRUE	reported	WYslv6
9	11495720	A	T	-0.0287	0.0052	3.05E-08	0.2204	NA	170756	rs1718917	329443	ieu-b-102	TRUE	reported	WYslv6
9	11497863	G	A	-0.0295	0.0052	1.43E-08	0.2195	NA	170756	rs5604471	329443	ieu-b-102	TRUE	reported	WYslv6
9	11500424	A	G	-0.0287	0.0052	3.14E-08	0.2202	NA	170756	rs1095986	329443	ieu-b-102	TRUE	reported	WYslv6
9	11500942	T	G	-0.0286	0.0052	3.73E-08	0.2204	NA	170756	rs2083816	329443	ieu-b-102	TRUE	reported	WYslv6
9	11503956	G	A	-0.0288	0.0052	2.96E-08	0.2204	NA	170756	rs5639913	329443	ieu-b-102	TRUE	reported	WYslv6
9	11506222	C	A	-0.029	0.0052	2.28E-08	0.2202	NA	170756	rs6255254	329443	ieu-b-102	TRUE	reported	WYslv6
9	11506471	A	G	-0.029	0.0052	2.38E-08	0.2202	NA	170756	rs1718928	329443	ieu-b-102	TRUE	reported	WYslv6
9	11506504	A	G	-0.029	0.0052	2.37E-08	0.2202	NA	170756	rs6255254	329443	ieu-b-102	TRUE	reported	WYslv6
9	11508875	G	C	-0.0288	0.0052	2.80E-08	0.2202	NA	170756	rs1036906	329443	ieu-b-102	TRUE	reported	WYslv6
9	11509772	T	A	-0.0303	0.0053	7.91E-09	0.2141	NA	170756	rs1080950	329443	ieu-b-102	TRUE	reported	WYslv6
9	11510934	A	G	-0.0303	0.0053	8.35E-09	0.2143	NA	170756	rs6255372	329443	ieu-b-102	TRUE	reported	WYslv6
9	11513019	T	C	-0.0296	0.0051	5.35E-09	0.2427	NA	170756	rs1982277	329443	ieu-b-102	TRUE	reported	WYslv6
9	11513617	T	G	-0.0306	0.0053	5.80E-09	0.214	NA	170756	rs1718948	329443	ieu-b-102	TRUE	reported	WYslv6
9	11514847	G	A	-0.0299	0.0052	1.12E-08	0.2155	NA	170756	rs1080950	329443	ieu-b-102	TRUE	reported	WYslv6
9	11519984	T	C	-0.0305	0.0053	6.99E-09	0.2133	NA	170756	rs1095988	329443	ieu-b-102	TRUE	reported	WYslv6
9	11521429	T	C	-0.0303	0.0053	8.03E-09	0.2134	NA	170756	rs6255372	329443	ieu-b-102	TRUE	reported	WYslv6
9	11522013	C	T	-0.0281	0.005	1.44E-08	0.252	NA	170756	rs1421110	329443	ieu-b-102	TRUE	reported	WYslv6
9	11522951	T	C	-0.0288	0.005	8.01E-09	0.2487	NA	170756	rs958538	329443	ieu-b-102	TRUE	reported	WYslv6
9	11525751	T	G	-0.0305	0.0053	6.47E-09	0.2133	NA	170756	rs1238036	329443	ieu-b-102	TRUE	reported	WYslv6
9	11526198	T	C	-0.0305	0.0053	6.44E-09	0.2132	NA	170756	rs1095989	329443	ieu-b-102	TRUE	reported	WYslv6
9	11527978	G	A	-0.0305	0.0053	6.92E-09	0.2132	NA	170756	rs1154740	329443	ieu-b-102	TRUE	reported	WYslv6
9	11529596	C	A	-0.0302	0.0053	9.90E-09	0.2131	NA	170756	rs1179478	329443	ieu-b-102	TRUE	reported	WYslv6
9	11530473	C	T	-0.0279	0.005	2.17E-08	0.2506	NA	170756	rs1080951	329443	ieu-b-102	TRUE	reported	WYslv6
9	11532261	C	G	-0.0301	0.0053	1.01E-08	0.214	NA	170756	rs1237952	329443	ieu-b-102	TRUE	reported	WYslv6
9	11535004	T	A	-0.0298	0.0053	1.43E-08	0.214	NA	170756	rs7270020	329443	ieu-b-102	TRUE	reported	WYslv6
9	11536677	A	G	-0.0302	0.0053	9.73E-09	0.2138	NA	170756	rs1779383	329443	ieu-b-102	TRUE	reported	WYslv6
9	11539295	A	G	-0.0297	0.0053	1.63E-08	0.2138	NA	170756	rs1095990	329443	ieu-b-102	TRUE	reported	WYslv6
9	11542604	A	G	-0.0301	0.0053	1.07E-08	0.2137	NA	170756	rs6255373	329443	ieu-b-102	TRUE	reported	WYslv6
9	11544925	C	T	-0.0299	0.0053	1.83E-08	0.2114	NA	170756	rs6255373	329443	ieu-b-102	TRUE	reported	WYslv6
9	11544964	T	G	-0.0278	0.0051	4.30E-08	0.2445	NA	170756	rs1095991	329443	ieu-b-102	TRUE	reported	WYslv6
9	11545347	G	C	-0.0298	0.0053	1.51E-08	0.2137	NA	170756	rs6255374	329443	ieu-b-102	TRUE	reported	WYslv6
9	11546400	G	C	-0.0298	0.0053	1.40E-08	0.2137	NA	170756	rs1095991	329443	ieu-b-102	TRUE	reported	WYslv6
9	11547243	C	T	-0.0291	0.0052	2.65E-08	0.2176	NA	170756	rs1779413	329443	ieu-b-102	TRUE	reported	WYslv6
9	11548730	G	A	-0.0286	0.0052	3.46E-08	0.2199	NA	170756	rs7270020	329443	ieu-b-102	TRUE	reported	WYslv6
9	11549994	G	A	-0.0284	0.0052	4.73E-08	0.22	NA	170756	rs1095991	329443	ieu-b-102	TRUE	reported	WYslv6
9	11551242	C	T	-0.029	0.0052	2.54E-08	0.2193	NA	170756	rs1240331	329443	ieu-b-102	TRUE	reported	WYslv6
9	11553208	A	G	-0.0279	0.005	1.83E-08	0.2535	NA	170756	rs1080951	329443	ieu-b-102	TRUE	reported	WYslv6
9	11553381	G	A	-0.0293	0.0053	2.91E-08	0.2147	NA	170756	rs1095992	329443	ieu-b-102	TRUE	reported	WYslv6
9	11553382	T	C	-0.0294	0.0053	2.62E-08	0.2147	NA	170756	rs3867326	329443	ieu-b-102	TRUE	reported	WYslv6
9	11557159	T	C	-0.0289	0.0052	2.81E-08	0.2188	NA	170756	rs1095992	329443	ieu-b-102	TRUE	reported	WYslv6
9	11557242	A	C	-0.0289	0.0052	2.75E-08	0.2187	NA	170756	rs1095992	329443	ieu-b-102	TRUE	reported	WYslv6
9	11557876	G	A	-0.0287	0.0052	3.42E-08	0.2196	NA	170756	rs1095993	329443	ieu-b-102	TRUE	reported	WYslv6
9	11559938	G	A	-0.0286	0.0052	3.55E-08	0.2196	NA	170756	rs1348385	329443	ieu-b-102	TRUE	reported	WYslv6
9	11560184	C	T	-0.0272	0.0049	3.34E-08	0.2579	NA	170756	rs1348384	329443	ieu-b-102	TRUE	reported	WYslv6
9	11561105	T	G	-0.0286	0.0052	3.60E-08	0.2196	NA	170756	rs7270021	329443	ieu-b-102	TRUE	reported	WYslv6
9	11561150	G	T	-0.0271	0.0049	3.65E-08	0.2563	NA	170756	rs1073824	329443	ieu-b-102	TRUE	reported	WYslv6

9	11561385	C	T	-0.0289	0.0052	2.86E-08	0.2191	NA	170756	rs1080952	329443	ieu-b-102	TRUE	reported	WYslv6
9	11562051	G	A	-0.0287	0.0052	3.36E-08	0.2196	NA	170756	rs1095993	329443	ieu-b-102	TRUE	reported	WYslv6
9	11562615	T	G	-0.0288	0.0052	3.04E-08	0.2198	NA	170756	rs1138909	329443	ieu-b-102	TRUE	reported	WYslv6
9	11564041	T	G	-0.0286	0.0052	3.74E-08	0.2201	NA	170756	rs1095993	329443	ieu-b-102	TRUE	reported	WYslv6
9	11566803	C	T	-0.0288	0.0052	2.84E-08	0.2201	NA	170756	rs1095994	329443	ieu-b-102	TRUE	reported	WYslv6
9	11568387	G	A	-0.0288	0.0052	3.04E-08	0.22	NA	170756	rs1095994	329443	ieu-b-102	TRUE	reported	WYslv6
9	11578540	C	T	-0.0286	0.0052	3.64E-08	0.2199	NA	170756	rs1095995	329443	ieu-b-102	TRUE	reported	WYslv6
9	11580309	G	A	-0.0282	0.0052	4.97E-08	0.2214	NA	170756	rs1377808	329443	ieu-b-102	TRUE	reported	WYslv6
9	11587493	C	T	-0.0283	0.0052	4.25E-08	0.2225	NA	170756	rs1095997	329443	ieu-b-102	TRUE	reported	WYslv6
9	11588186	T	G	-0.0283	0.0052	4.38E-08	0.2224	NA	170756	rs1095997	329443	ieu-b-102	TRUE	reported	WYslv6
9	11595926	C	G	-0.0282	0.0052	4.82E-08	0.2229	NA	170756	rs1095999	329443	ieu-b-102	TRUE	reported	WYslv6
9	11598192	C	T	-0.0288	0.0052	2.73E-08	0.2209	NA	170756	rs1095999	329443	ieu-b-102	TRUE	reported	WYslv6
9	11598287	T	C	-0.0292	0.0052	1.60E-08	0.2225	NA	170756	rs1095999	329443	ieu-b-102	TRUE	reported	WYslv6
9	11598443	G	C	-0.0287	0.0052	3.16E-08	0.2202	NA	170756	rs1095999	329443	ieu-b-102	TRUE	reported	WYslv6
9	11598511	A	C	-0.0287	0.0052	3.22E-08	0.2201	NA	170756	rs1095999	329443	ieu-b-102	TRUE	reported	WYslv6
9	11598539	T	C	-0.0287	0.0052	3.08E-08	0.2201	NA	170756	rs1095999	329443	ieu-b-102	TRUE	reported	WYslv6
9	11598811	T	G	-0.0287	0.0052	3.25E-08	0.2203	NA	170756	rs4618798	329443	ieu-b-102	TRUE	reported	WYslv6
9	11599164	G	C	-0.0287	0.0052	3.06E-08	0.2203	NA	170756	rs1337808	329443	ieu-b-102	TRUE	reported	WYslv6
9	11599324	T	C	-0.0287	0.0052	2.98E-08	0.2203	NA	170756	rs7270023	329443	ieu-b-102	TRUE	reported	WYslv6
9	11599997	C	G	-0.0286	0.0052	3.43E-08	0.2202	NA	170756	rs4617256	329443	ieu-b-102	TRUE	reported	WYslv6
9	11600065	C	G	-0.0292	0.0052	2.21E-08	0.2184	NA	170756	rs4617257	329443	ieu-b-102	TRUE	reported	WYslv6
9	11600067	G	A	-0.0292	0.0052	2.13E-08	0.2184	NA	170756	rs3867326	329443	ieu-b-102	TRUE	reported	WYslv6
9	11600463	A	G	-0.0285	0.0052	3.75E-08	0.2205	NA	170756	rs1080953	329443	ieu-b-102	TRUE	reported	WYslv6
9	11600564	A	T	-0.0286	0.0052	3.61E-08	0.2205	NA	170756	rs1095999	329443	ieu-b-102	TRUE	reported	WYslv6
9	11600635	A	C	-0.0285	0.0052	3.80E-08	0.2206	NA	170756	rs1096000	329443	ieu-b-102	TRUE	reported	WYslv6
9	11600783	T	C	-0.0286	0.0052	3.64E-08	0.2203	NA	170756	rs1096000	329443	ieu-b-102	TRUE	reported	WYslv6
9	11600875	G	A	-0.0285	0.0052	3.70E-08	0.2205	NA	170756	rs1096000	329443	ieu-b-102	TRUE	reported	WYslv6
9	11601305	C	T	-0.0308	0.0054	1.41E-08	0.2096	NA	170756	rs1237548	329443	ieu-b-102	TRUE	reported	WYslv6
9	11601309	A	G	-0.03	0.0054	2.42E-08	0.2111	NA	170756	rs1237818	329443	ieu-b-102	TRUE	reported	WYslv6
9	11601919	C	T	-0.0291	0.0052	2.36E-08	0.2184	NA	170756	rs7270024	329443	ieu-b-102	TRUE	reported	WYslv6
9	11601932	C	G	-0.0291	0.0052	2.45E-08	0.2184	NA	170756	rs7270024	329443	ieu-b-102	TRUE	reported	WYslv6
9	11602052	C	A	-0.0286	0.0052	3.66E-08	0.2205	NA	170756	rs5594557	329443	ieu-b-102	TRUE	reported	WYslv6
9	11602138	C	T	-0.0289	0.0052	3.28E-08	0.2181	NA	170756	rs5638101	329443	ieu-b-102	TRUE	reported	WYslv6
9	11602144	A	G	-0.0289	0.0052	3.31E-08	0.2182	NA	170756	rs5573420	329443	ieu-b-102	TRUE	reported	WYslv6
9	11602189	C	T	-0.0292	0.0052	2.24E-08	0.2187	NA	170756	rs5616997	329443	ieu-b-102	TRUE	reported	WYslv6
9	11602658	C	T	-0.0285	0.0052	3.81E-08	0.2205	NA	170756	rs4621919	329443	ieu-b-102	TRUE	reported	WYslv6
9	11602719	C	T	-0.0285	0.0052	3.80E-08	0.2205	NA	170756	rs4562429	329443	ieu-b-102	TRUE	reported	WYslv6
9	11602782	G	A	-0.0286	0.0052	3.63E-08	0.2205	NA	170756	rs4382565	329443	ieu-b-102	TRUE	reported	WYslv6
9	11602884	G	C	-0.0285	0.0052	3.74E-08	0.2205	NA	170756	rs4413878	329443	ieu-b-102	TRUE	reported	WYslv6
9	11602902	T	C	-0.0285	0.0052	3.84E-08	0.2203	NA	170756	rs4524892	329443	ieu-b-102	TRUE	reported	WYslv6
9	11602915	A	C	-0.0285	0.0052	3.75E-08	0.2203	NA	170756	rs4506321	329443	ieu-b-102	TRUE	reported	WYslv6
9	11603298	C	T	-0.0285	0.0052	3.74E-08	0.2204	NA	170756	rs1237622	329443	ieu-b-102	TRUE	reported	WYslv6
9	11603333	C	A	-0.0285	0.0052	3.77E-08	0.2206	NA	170756	rs1237623	329443	ieu-b-102	TRUE	reported	WYslv6
9	11603385	A	C	-0.0285	0.0052	3.81E-08	0.2206	NA	170756	rs1237898	329443	ieu-b-102	TRUE	reported	WYslv6
9	11604194	C	T	-0.0286	0.0052	3.52E-08	0.2206	NA	170756	rs6255157	329443	ieu-b-102	TRUE	reported	WYslv6
9	11604620	T	A	-0.0286	0.0052	3.55E-08	0.2205	NA	170756	rs6255157	329443	ieu-b-102	TRUE	reported	WYslv6
9	11604724	C	A	-0.0286	0.0052	3.55E-08	0.2205	NA	170756	rs998426	329443	ieu-b-102	TRUE	reported	WYslv6
9	11604929	C	T	-0.0286	0.0052	3.65E-08	0.2205	NA	170756	rs6255157	329443	ieu-b-102	TRUE	reported	WYslv6
9	11605821	C	T	-0.0283	0.0052	4.86E-08	0.2204	NA	170756	rs1096000	329443	ieu-b-102	TRUE	reported	WYslv6
9	11606293	A	T	-0.0284	0.0052	4.57E-08	0.2206	NA	170756	rs1781733	329443	ieu-b-102	TRUE	reported	WYslv6
9	11606402	C	T	-0.0284	0.0052	4.58E-08	0.2206	NA	170756	rs7270025	329443	ieu-b-102	TRUE	reported	WYslv6
9	11606727	C	T	-0.0284	0.0052	4.58E-08	0.2206	NA	170756	rs1096000	329443	ieu-b-102	TRUE	reported	WYslv6
9	11606971	T	C	-0.0286	0.0052	3.91E-08	0.2197	NA	170756	rs1096000	329443	ieu-b-102	TRUE	reported	WYslv6
9	11607188	G	C	-0.0268	0.0049	4.99E-08	0.2584	NA	170756	rs1080953	329443	ieu-b-102	TRUE	reported	WYslv6
9	11607207	T	C	-0.0285	0.0052	4.29E-08	0.22	NA	170756	rs3867326	329443	ieu-b-102	TRUE	reported	WYslv6
9	11607294	C	T	-0.0283	0.0052	4.83E-08	0.2205	NA	170756	rs1096000	329443	ieu-b-102	TRUE	reported	WYslv6
9	11607516	G	A	-0.0283	0.0052	4.69E-08	0.2206	NA	170756	rs1096001	329443	ieu-b-102	TRUE	reported	WYslv6
9	11607738	T	C	-0.0283	0.0052	4.80E-08	0.2206	NA	170756	rs1096001	329443	ieu-b-102	TRUE	reported	WYslv6
9	11607822	G	A	-0.0283	0.0052	4.74E-08	0.2206	NA	170756	rs1096001	329443	ieu-b-102	TRUE	reported	WYslv6
9	11607929	T	C	-0.0283	0.0052	4.70E-08	0.2206	NA	170756	rs1096001	329443	ieu-b-102	TRUE	reported	WYslv6
9	11608040	C	A	-0.0283	0.0052	4.69E-08	0.2206	NA	170756	rs1096001	329443	ieu-b-102	TRUE	reported	WYslv6
9	11608310	T	C	-0.0283	0.0052	4.70E-08	0.2206	NA	170756	rs1096001	329443	ieu-b-102	TRUE	reported	WYslv6
9	11608722	C	A	-0.0283	0.0052	4.69E-08	0.2206	NA	170756	rs1096001	329443	ieu-b-102	TRUE	reported	WYslv6
9	11608954	G	A	-0.0283	0.0052	4.86E-08	0.2206	NA	170756	rs1096002	329443	ieu-b-102	TRUE	reported	WYslv6
9	11629615	G	A	-0.0286	0.0052	3.03E-08	0.2224	NA	170756	rs1096003	329443	ieu-b-102	TRUE	reported	WYslv6
9	11630621	A	G	-0.028	0.0051	4.95E-08	0.2278	NA	170756	rs1096003	329443	ieu-b-102	TRUE	reported	WYslv6
9	11636127	A	G	-0.0285	0.0052	3.61E-08	0.2226	NA	170756	rs1151505	329443	ieu-b-102	TRUE	reported	WYslv6
9	11651144	A	G	-0.0293	0.0052	1.39E-08	0.2231	NA	170756	rs1096006	329443	ieu-b-102	TRUE	reported	WYslv6
9	11669225	G	A	-0.0282	0.0052	4.38E-08	0.2254	NA	170756	rs5296195	329443	ieu-b-102	TRUE	reported	WYslv6
9	11670414	T	G	-0.0287	0.0051	2.29E-08	0.2266	NA	170756	rs1178896	329443	ieu-b-102	TRUE	reported	WYslv6
9	11673648	G	A	-0.0281	0.0051	4.95E-08	0.2257	NA	170756	rs6255338	329443	ieu-b-102	TRUE	reported	WYslv6
9	11676128	A	G	-0.0282	0.0052	4.59E-08	0.2256	NA	170756	rs6255339	329443	ieu-b-102	TRUE	reported	WYslv6
9	11699270	C	G	-0.0289	0.0051	1.95E-08	0.2256	NA	170756	rs1096010	329443	ieu-b-102	TRUE	reported	WYslv6
9	11701686	C	A	-0.0283	0.0051	3.72E-08	0.2265	NA	170756	rs1080955	329443	ieu-b-102	TRUE	reported	WYslv6
9	11703866	C	A	-0.0292	0.0052	1.68E-08	0.2234	NA	170756	rs5583344	329443	ieu-b-102	TRUE	reported	WYslv6
9	11703892	C	T	-0.0287	0.0052	2.83E-08	0.2236	NA	170756	rs5631838	329443	ieu-b-102	TRUE	reported	WYslv6
9	11724099	C	A	-0.0289	0.0052	2.57E-08	0.2198	NA	170756	rs7548976	329443	ieu-b-102	TRUE	reported	WYslv6
9	17012877	G	A	-0.0241	0.0043	2.74E-08	0.4336	NA	170756	rs1487500	329443	ieu-b-102	TRUE	reported	WYslv6
9	17016503	A	T	-0.0251	0.0043	6.36E-09	0.4591	NA	170756	rs263645	329443	ieu-b-102	TRUE	reported	WYslv6
9	17023857	A	G	-0.0244	0.0043	1.50E-08	0.4906	NA	170756	rs2632542	329443	ieu-b-102	TRUE	reported	WYslv6
9	17024177	T	G	-0.0243	0.0043	1.66E-08	0.4901	NA	170756	rs387621	329443	ieu-b-102	TRUE	reported	WYslv6
9	17025267	T	G	-0.0246	0.0043	1.12E-08	0.4902	NA	170756	rs431528	329443	ieu-b-102	TRUE	reported	WYslv6
9	17025503	G	A	-0.0246	0.0043	1.11E-08	0.4898	NA	170756	rs1231521	329443	ieu-b-102	TRUE	reported	WYslv6
9	17029204	C	G	-0.0239	0.0043	2.75E-08	0.4856	NA	170756	rs2815186	329443	ieu-b-102	TRUE	reported	WYslv6
9	17029756	T	G	-0.0243	0.0043	1.61E-08	0.4848	NA	170756	rs263568	329443	ieu-b-102	TRUE	reported	WYslv6
9	17029942	C	T	-0.0247	0.0043	9.86E-09	0.4843	NA	170756	rs166418	329443	ieu-b-102	TRUE	reported	WYslv6
9	17033119	G	C	-0.0245	0.0043	1.30E-08	0.4615	NA	170756	rs263573	329443	ieu-b-102	TRUE	reported	WYslv6
9	17033840	G	A	-0.025	0.0043	6.67E-09	0.4605	NA	170756	rs263575	329443	ieu-b-102	TRUE	reported	WYslv6
9	17034019	T	G	-0.0244	0.0043	1.41E-08	0.4617	NA	170756	rs263576	329443	ieu-b-102	TRUE	reported	WYslv6
9	17038715	G	C	-0.0246	0.0043	1.12E-08	0.4616	NA	170756	rs263579	329443	ieu-b-102	TRUE	reported	WYslv6
9	17039312	T	G	-0.0239	0.0043	3.14E-08	0.4563	NA	170756	rs263580	329443	ieu-b-102	TRUE	reported	WYslv6
9	17040154	A	G	-0.0242	0.0043	1.96E-08	0.4628	NA	170756	rs263583	329443	ieu-b-102	TRUE	reported	WYslv6
9	17041110	T	C	-0.0237	0.0043	3.68E-08	0.4622	NA	170756	rs263584	329443	ieu-b-102	TRUE	reported	WYslv6
9	17042544	G	C	-0.0243	0.0043	1.80E-08	0.4606	NA	170756	rs2694058	329443	ieu-b-102	TRUE	reported	WYslv6
9	17042893	A	G	-0.0238	0.0043	3.40E-08	0.462	NA	170756	rs1482143	329443	ieu-b-102	TRUE	reported	WYslv6
9	17044971	T	A	-0.0238	0.0043	3.79E-08	0.468	NA	170756	rs393488	329443	ieu-b-102	TRUE	reported	WYslv6
9	25219171	G	T	-0.0375	0.0069	4.80E-08	0.11	NA	170756	rs4626698	329443	ieu-b-102	TRUE	reported	WYslv6
9	25232978	G	A	-0.039	0.007	2.41E-08	0.1081	NA	170756	rs5928317	329443	ieu-b-102	TRUE	reported	WYslv6
9	25235063	C	T	-0.0382	0.007	4.08E-08	0.1067	NA	170756	rs1205703	329443	ieu-b-102	TRUE	reported	WYslv6
9	25236859	T	C	-0.0377	0.0069	4.92E-08	0.1083	NA	170756	rs7347414	329443	ieu-b-102	TRUE	reported	WYslv6
9	25273673	G	A	-0.0387	0.0071	4.42E-08	0.11	NA	170756	rs5973650	329443	ieu-b-102	TRUE	reported	WYslv6
9	36999369	C	T	0.0247	0.0045	3.45E-08	0.3756	NA	170756	rs7030813	329443	ieu-b-102	TRUE	reported	WYslv6
9	37000687	C	T	0.0245	0.0045	4.16E-08	0.3753	NA	170756	rs1396733	329443	ieu-b-102	TRUE	reported	WYslv6
9	37001471	T	A	0.0246	0.0045	3.79E-08	0.3762	NA	170756	rs1329572	329443	ieu-b-102	TRUE	reported	WYslv6
9	37076618	A	T	0.0317	0.0058	4.13E-08	0.164	NA	170756	rs6253374	329443	ieu-b-102	TRUE	reported	WYslv6
9	37078909	C	T	0.0317	0.0058	4.52E-08	0.1641	NA	170756	rs6253374	329443	ieu-b-102	TRUE	reported	WYslv6
9	37080941	G	A	0.0323	0.0059	4.96E-08	0.1597	NA	170756	rs1097319	329443	ieu-b-102	TRUE	reported	WYslv6

9	37082333	A	G	0.0323	0.0058	2.68E-08	0.1622	NA	170756	rs1097319	329443	ieu-b-102	TRUE	reported	WYslv6
9	37082403	A	G	0.0324	0.0058	2.62E-08	0.1622	NA	170756	rs1097319	329443	ieu-b-102	TRUE	reported	WYslv6
9	37082726	G	A	0.0323	0.0058	2.68E-08	0.1622	NA	170756	rs1128372	329443	ieu-b-102	TRUE	reported	WYslv6
9	37091018	C	T	0.0323	0.0058	2.81E-08	0.1622	NA	170756	rs1237706	329443	ieu-b-102	TRUE	reported	WYslv6
9	37095504	G	A	0.0322	0.0058	2.99E-08	0.1623	NA	170756	rs6777757	329443	ieu-b-102	TRUE	reported	WYslv6
9	37098306	C	G	0.0323	0.0058	2.76E-08	0.1622	NA	170756	rs6253566	329443	ieu-b-102	TRUE	reported	WYslv6
9	37100525	G	T	0.0327	0.006	4.01E-08	0.157	NA	170756	rs1097320	329443	ieu-b-102	TRUE	reported	WYslv6
9	37104234	A	G	0.0324	0.0058	2.19E-08	0.1624	NA	170756	rs1097320	329443	ieu-b-102	TRUE	reported	WYslv6
9	37106370	T	A	0.0324	0.0058	2.55E-08	0.1622	NA	170756	rs6253567	329443	ieu-b-102	TRUE	reported	WYslv6
9	37106442	T	C	0.0325	0.0058	2.15E-08	0.1623	NA	170756	rs6253567	329443	ieu-b-102	TRUE	reported	WYslv6
9	37106754	C	T	0.0324	0.0058	2.62E-08	0.1618	NA	170756	rs1097321	329443	ieu-b-102	TRUE	reported	WYslv6
9	37113815	G	C	0.0325	0.0058	2.20E-08	0.1628	NA	170756	rs1097321	329443	ieu-b-102	TRUE	reported	WYslv6
9	37121745	G	A	0.0325	0.0059	2.99E-08	0.1612	NA	170756	rs1097322	329443	ieu-b-102	TRUE	reported	WYslv6
9	37121785	A	T	0.0327	0.0058	2.09E-08	0.1625	NA	170756	rs1097322	329443	ieu-b-102	TRUE	reported	WYslv6
9	37121837	T	C	0.0324	0.0058	2.21E-08	0.1629	NA	170756	rs1219109	329443	ieu-b-102	TRUE	reported	WYslv6
9	37125911	C	T	0.0322	0.0058	2.70E-08	0.1636	NA	170756	rs7965292	329443	ieu-b-102	TRUE	reported	WYslv6
9	37127983	C	A	0.0324	0.0058	2.26E-08	0.163	NA	170756	rs1097322	329443	ieu-b-102	TRUE	reported	WYslv6
9	37132463	C	T	0.0323	0.0058	2.42E-08	0.163	NA	170756	rs6098223	329443	ieu-b-102	TRUE	reported	WYslv6
9	37132859	G	A	0.0323	0.0058	2.42E-08	0.163	NA	170756	rs5640153	329443	ieu-b-102	TRUE	reported	WYslv6
9	37134011	G	A	0.0325	0.0058	2.02E-08	0.163	NA	170756	rs5795165	329443	ieu-b-102	TRUE	reported	WYslv6
9	37134370	G	C	0.0323	0.0059	4.10E-08	0.1572	NA	170756	rs6253570	329443	ieu-b-102	TRUE	reported	WYslv6
9	37139650	G	A	0.0322	0.0058	2.80E-08	0.1637	NA	170756	rs1097322	329443	ieu-b-102	TRUE	reported	WYslv6
9	37139899	G	A	0.0325	0.0058	2.12E-08	0.1631	NA	170756	rs1097322	329443	ieu-b-102	TRUE	reported	WYslv6
9	37140170	G	A	0.0325	0.0058	2.11E-08	0.1631	NA	170756	rs2068020	329443	ieu-b-102	TRUE	reported	WYslv6
9	37150985	G	A	0.0327	0.0058	1.74E-08	0.1625	NA	170756	rs1368802	329443	ieu-b-102	TRUE	reported	WYslv6
9	37151252	C	T	0.0328	0.0058	1.59E-08	0.1635	NA	170756	rs1097323	329443	ieu-b-102	TRUE	reported	WYslv6
9	37151342	C	G	0.0329	0.0058	1.45E-08	0.163	NA	170756	rs1748696	329443	ieu-b-102	TRUE	reported	WYslv6
9	37151468	A	G	0.0329	0.0058	1.50E-08	0.1628	NA	170756	rs1748698	329443	ieu-b-102	TRUE	reported	WYslv6
9	37152389	G	A	0.0328	0.0058	1.32E-08	0.1662	NA	170756	rs1081451	329443	ieu-b-102	TRUE	reported	WYslv6
9	37166400	T	C	0.0329	0.0058	1.43E-08	0.1632	NA	170756	rs9006009	329443	ieu-b-102	TRUE	reported	WYslv6
9	37171808	T	C	0.0327	0.0058	1.74E-08	0.1632	NA	170756	rs1097324	329443	ieu-b-102	TRUE	reported	WYslv6
9	37175547	C	T	0.033	0.0058	1.19E-08	0.1628	NA	170756	rs6253571	329443	ieu-b-102	TRUE	reported	WYslv6
9	37175765	T	C	0.0329	0.0058	1.18E-08	0.1663	NA	170756	rs1081451	329443	ieu-b-102	TRUE	reported	WYslv6
9	37177924	A	G	0.0331	0.0058	1.18E-08	0.1631	NA	170756	rs1097324	329443	ieu-b-102	TRUE	reported	WYslv6
9	37182580	C	G	0.0331	0.0058	1.09E-08	0.1631	NA	170756	rs6253571	329443	ieu-b-102	TRUE	reported	WYslv6
9	37182655	G	A	0.0339	0.0058	4.69E-09	0.1639	NA	170756	rs6253571	329443	ieu-b-102	TRUE	reported	WYslv6
9	37184633	C	T	0.0332	0.0058	1.07E-08	0.1631	NA	170756	rs5691615	329443	ieu-b-102	TRUE	reported	WYslv6
9	37184780	A	G	0.0331	0.0058	1.08E-08	0.1631	NA	170756	rs5831984	329443	ieu-b-102	TRUE	reported	WYslv6
9	37192156	G	A	0.0331	0.0058	1.10E-08	0.1631	NA	170756	rs1097324	329443	ieu-b-102	TRUE	reported	WYslv6
9	37192988	A	C	0.0329	0.0058	1.40E-08	0.1634	NA	170756	rs1097324	329443	ieu-b-102	TRUE	reported	WYslv6
9	37198176	T	C	0.0324	0.0058	2.18E-08	0.1635	NA	170756	rs1097325	329443	ieu-b-102	TRUE	reported	WYslv6
9	37202969	C	T	0.033	0.0058	1.29E-08	0.1633	NA	170756	rs5608394	329443	ieu-b-102	TRUE	reported	WYslv6
9	37206109	G	T	0.0332	0.0058	1.09E-08	0.1634	NA	170756	rs1346204	329443	ieu-b-102	TRUE	reported	WYslv6
9	37207269	T	C	0.0332	0.0058	1.04E-08	0.1636	NA	170756	rs4526442	329443	ieu-b-102	TRUE	reported	WYslv6
9	37219050	A	T	0.033	0.0058	1.20E-08	0.1636	NA	170756	rs1097325	329443	ieu-b-102	TRUE	reported	WYslv6
9	37223862	A	G	0.0331	0.0058	8.63E-09	0.167	NA	170756	rs2029646	329443	ieu-b-102	TRUE	reported	WYslv6
9	37224460	T	C	0.033	0.0058	1.18E-08	0.1635	NA	170756	rs1097325	329443	ieu-b-102	TRUE	reported	WYslv6
9	37232221	G	A	0.0331	0.0058	1.05E-08	0.1633	NA	170756	rs1097326	329443	ieu-b-102	TRUE	reported	WYslv6
9	37237963	C	T	0.033	0.0058	1.29E-08	0.1633	NA	170756	rs6163741	329443	ieu-b-102	TRUE	reported	WYslv6
9	37240488	T	C	0.0331	0.0058	1.07E-08	0.1635	NA	170756	rs6253572	329443	ieu-b-102	TRUE	reported	WYslv6
9	37242504	T	C	0.0329	0.0058	1.26E-08	0.1635	NA	170756	rs1097326	329443	ieu-b-102	TRUE	reported	WYslv6
9	37242704	T	A	0.0329	0.0058	1.06E-08	0.1668	NA	170756	rs495304	329443	ieu-b-102	TRUE	reported	WYslv6
9	37249831	T	C	0.0331	0.0058	1.03E-08	0.1634	NA	170756	rs1097326	329443	ieu-b-102	TRUE	reported	WYslv6
9	37254694	T	G	0.0331	0.0058	1.08E-08	0.1634	NA	170756	rs1097326	329443	ieu-b-102	TRUE	reported	WYslv6
9	37255616	T	A	0.0331	0.0058	1.04E-08	0.1634	NA	170756	rs6714462	329443	ieu-b-102	TRUE	reported	WYslv6
9	37257282	T	C	0.0331	0.0058	1.14E-08	0.1634	NA	170756	rs1097327	329443	ieu-b-102	TRUE	reported	WYslv6
9	37259118	T	C	0.0329	0.0058	1.32E-08	0.164	NA	170756	rs1097327	329443	ieu-b-102	TRUE	reported	WYslv6
9	37259236	A	G	0.0331	0.0058	1.04E-08	0.1637	NA	170756	rs1097327	329443	ieu-b-102	TRUE	reported	WYslv6
9	37259678	A	G	0.0331	0.0058	1.11E-08	0.1636	NA	170756	rs4304407	329443	ieu-b-102	TRUE	reported	WYslv6
9	37268221	C	T	0.0327	0.0058	1.70E-08	0.1628	NA	170756	rs1097328	329443	ieu-b-102	TRUE	reported	WYslv6
9	37271781	G	A	0.0332	0.0058	1.10E-08	0.1628	NA	170756	rs6253379	329443	ieu-b-102	TRUE	reported	WYslv6
9	37277902	A	C	0.0329	0.0058	1.28E-08	0.1636	NA	170756	rs1097328	329443	ieu-b-102	TRUE	reported	WYslv6
9	37280173	G	A	0.0329	0.0058	1.43E-08	0.1633	NA	170756	rs1097328	329443	ieu-b-102	TRUE	reported	WYslv6
9	37282830	G	A	0.033	0.0059	1.76E-08	0.1615	NA	170756	rs1097328	329443	ieu-b-102	TRUE	reported	WYslv6
9	37283343	T	C	0.0329	0.0058	1.39E-08	0.1633	NA	170756	rs6253379	329443	ieu-b-102	TRUE	reported	WYslv6
9	37288938	C	A	0.0327	0.0058	1.76E-08	0.1633	NA	170756	rs5826524	329443	ieu-b-102	TRUE	reported	WYslv6
9	37291034	T	C	0.0328	0.0058	1.63E-08	0.1634	NA	170756	rs6253380	329443	ieu-b-102	TRUE	reported	WYslv6
9	37293079	A	G	0.0329	0.0058	1.50E-08	0.1636	NA	170756	rs6253380	329443	ieu-b-102	TRUE	reported	WYslv6
9	37293627	T	C	0.0329	0.0058	1.49E-08	0.1636	NA	170756	rs3802420	329443	ieu-b-102	TRUE	reported	WYslv6
9	37295658	G	A	0.0328	0.0058	1.53E-08	0.1634	NA	170756	rs1097329	329443	ieu-b-102	TRUE	reported	WYslv6
9	37303730	G	A	0.033	0.0058	1.26E-08	0.1635	NA	170756	rs1133800	329443	ieu-b-102	TRUE	reported	WYslv6
9	37331037	G	A	0.0328	0.0058	1.53E-08	0.1636	NA	170756	rs1097330	329443	ieu-b-102	TRUE	reported	WYslv6
9	37331605	T	G	0.0329	0.0058	1.49E-08	0.1636	NA	170756	rs1180592	329443	ieu-b-102	TRUE	reported	WYslv6
9	37333910	C	T	0.0327	0.0058	1.72E-08	0.1635	NA	170756	rs1170225	329443	ieu-b-102	TRUE	reported	WYslv6
9	37344529	C	T	0.0332	0.006	3.56E-08	0.151	NA	170756	rs1097330	329443	ieu-b-102	TRUE	reported	WYslv6
9	37345800	G	A	0.0327	0.0058	1.76E-08	0.1633	NA	170756	rs1375645	329443	ieu-b-102	TRUE	reported	WYslv6
9	37360811	G	T	0.0328	0.0058	1.74E-08	0.1635	NA	170756	rs5612171	329443	ieu-b-102	TRUE	reported	WYslv6
9	37361314	C	T	0.0323	0.0058	2.68E-08	0.1639	NA	170756	rs1097330	329443	ieu-b-102	TRUE	reported	WYslv6
9	37361785	A	G	0.0324	0.0058	2.69E-08	0.1634	NA	170756	rs7273779	329443	ieu-b-102	TRUE	reported	WYslv6
9	1.2E+08	T	C	-0.0272	0.0048	1.12E-08	0.284	NA	170756	rs1888286	329443	ieu-b-102	TRUE	reported	WYslv6
9	1.2E+08	A	G	-0.0272	0.0049	3.34E-08	0.2568	NA	170756	rs1075987	329443	ieu-b-102	TRUE	reported	WYslv6
9	1.2E+08	T	G	-0.028	0.0048	4.71E-09	0.2806	NA	170756	rs1081796	329443	ieu-b-102	TRUE	reported	WYslv6
9	1.2E+08	T	C	-0.0281	0.0048	4.25E-09	0.281	NA	170756	rs2418449	329443	ieu-b-102	TRUE	reported	WYslv6
9	1.2E+08	C	T	-0.028	0.0048	4.84E-09	0.2805	NA	170756	rs4629949	329443	ieu-b-102	TRUE	reported	WYslv6
9	1.2E+08	A	G	-0.0277	0.0048	7.75E-09	0.2781	NA	170756	rs1075987	329443	ieu-b-102	TRUE	reported	WYslv6
9	1.2E+08	G	A	-0.0271	0.0048	1.93E-08	0.2783	NA	170756	rs1075988	329443	ieu-b-102	TRUE	reported	WYslv6
9	1.2E+08	A	C	-0.0277	0.0048	7.22E-09	0.2779	NA	170756	rs1075988	329443	ieu-b-102	TRUE	reported	WYslv6
9	1.2E+08	C	T	-0.0266	0.0048	2.51E-08	0.2843	NA	170756	rs7856424	329443	ieu-b-102	TRUE	reported	WYslv6
9	1.2E+08	T	C	-0.0276	0.0048	8.76E-09	0.2781	NA	170756	rs1075988	329443	ieu-b-102	TRUE	reported	WYslv6
9	1.2E+08	C	G	-0.0275	0.0048	9.04E-09	0.2781	NA	170756	rs1928984	329443	ieu-b-102	TRUE	reported	WYslv6
9	1.2E+08	T	C	-0.0275	0.0048	9.71E-09	0.2781	NA	170756	rs1928987	329443	ieu-b-102	TRUE	reported	WYslv6
10	1.06E+08	G	A	-0.0267	0.0044	1.44E-09	0.4081	NA	170756	rs1159621	329443	ieu-b-102	TRUE	reported	WYslv6
10	1.06E+08	T	C	-0.0272	0.0044	6.49E-10	0.4124	NA	170756	rs1159923	329443	ieu-b-102	TRUE	reported	WYslv6
10	1.06E+08	T	C	-0.027	0.0044	7.73E-10	0.4137	NA	170756	rs9787523	329443	ieu-b-102	TRUE	reported	WYslv6
10	1.06E+08	A	G	-0.0268	0.0044	1.29E-09	0.4167	NA	170756	rs3896224	329443	ieu-b-102	TRUE	reported	WYslv6
10	1.07E+08	G	A	0.0286	0.0052	3.51E-08	0.2206	NA	170756	rs731163	329443	ieu-b-102	TRUE	reported	WYslv6
10	1.07E+08	G	A	0.0286	0.0052	3.72E-08	0.2205	NA	170756	rs1223700	329443	ieu-b-102	TRUE	reported	WYslv6
10	1.07E+08	C	T	0.029	0.0052	2.51E-08	0.2193	NA	170756	rs971527	329443	ieu-b-102	TRUE	reported	WYslv6
10	1.07E+08	T	G	0.0287	0.0052	3.21E-08	0.2201	NA	170756	rs1119219	329443	ieu-b-102	TRUE	reported	WYslv6
10	1.07E+08	C	T	0.0287	0.0052	3.08E-08	0.2206	NA	170756	rs2451462	329443	ieu-b-102	TRUE	reported	WYslv6
10	1.07E+08	A	G	0.0284	0.0052	4.76E-08	0.2199	NA	170756	rs2496028	329443	ieu-b-102	TRUE	reported	WYslv6
10	1.07E+08	A	G	-0.0269	0.0045	1.70E-09	0.6307	NA	170756	rs1088405	329443	ieu-b-102	TRUE	reported	WYslv6
10	1.07E+08	T	C	-0.0279	0.0045	3.94E-10	0.6351	NA	170756	rs2496014	329443	ieu-b-102	TRUE	reported	WYslv6
10	1.07E+08	T	C	-0.0271	0.0044	1.09E-09	0.6316	NA	170756	rs7900775	329443	ieu-b-102	TRUE	reported	WYslv6
10	1.07E+08	T	G	-0.0279	0.0045	4.08E-10	0.6351	NA	170756	rs1027190	329443	ieu-b-102	TRUE	reported	WYslv6
10	1.07E+08	A	G	-0.03	0.0045	2.29E-11	0.6434	NA	170756	rs1021363	329443	ieu-b-102	TRUE	reported	WYslv6
10	1.07E+08	G	A	-0.0293	0.0045	6.85E-11	0.6445	NA	170756	rs1021362	329443	ieu-b-102	TRUE	reported	WYslv6

10	1.07E+08	A	C	-0.0273	0.0045	1.70E-09	0.6598	NA	170756	rs2491381	329443	ieu-b-102	TRUE	reported	WYslv6
10	1.07E+08	G	T	-0.0277	0.0045	8.48E-10	0.6575	NA	170756	rs2491376	329443	ieu-b-102	TRUE	reported	WYslv6
10	1.07E+08	T	C	-0.0278	0.0045	8.05E-10	0.6575	NA	170756	rs1477228	329443	ieu-b-102	TRUE	reported	WYslv6
10	1.07E+08	T	C	-0.0278	0.0045	7.78E-10	0.6575	NA	170756	rs2496022	329443	ieu-b-102	TRUE	reported	WYslv6
10	1.07E+08	T	C	-0.0277	0.0045	9.09E-10	0.6575	NA	170756	rs2451499	329443	ieu-b-102	TRUE	reported	WYslv6
10	1.07E+08	C	T	-0.0279	0.0045	7.14E-10	0.6573	NA	170756	rs2451500	329443	ieu-b-102	TRUE	reported	WYslv6
10	1.07E+08	T	C	-0.0273	0.0045	1.73E-09	0.6595	NA	170756	rs728423	329443	ieu-b-102	TRUE	reported	WYslv6
10	1.07E+08	G	A	-0.0277	0.0045	9.65E-10	0.6575	NA	170756	rs1961639	329443	ieu-b-102	TRUE	reported	WYslv6
10	1.07E+08	G	A	-0.0272	0.0045	1.86E-09	0.6596	NA	170756	rs1352653	329443	ieu-b-102	TRUE	reported	WYslv6
10	1.07E+08	C	T	-0.0279	0.0045	6.98E-10	0.6568	NA	170756	rs2496024	329443	ieu-b-102	TRUE	reported	WYslv6
10	1.07E+08	G	A	-0.0272	0.0045	1.92E-09	0.6599	NA	170756	rs2496023	329443	ieu-b-102	TRUE	reported	WYslv6
10	1.07E+08	T	A	-0.0276	0.0045	9.99E-10	0.6575	NA	170756	rs2932558	329443	ieu-b-102	TRUE	reported	WYslv6
10	1.07E+08	T	C	-0.0274	0.0045	1.44E-09	0.6589	NA	170756	rs2491373	329443	ieu-b-102	TRUE	reported	WYslv6
10	1.07E+08	C	T	-0.0276	0.0045	1.00E-09	0.6578	NA	170756	rs923546	329443	ieu-b-102	TRUE	reported	WYslv6
10	1.07E+08	G	A	-0.0277	0.0045	9.61E-10	0.6578	NA	170756	rs2932559	329443	ieu-b-102	TRUE	reported	WYslv6
10	1.07E+08	T	C	-0.0294	0.005	3.84E-09	0.2469	NA	170756	rs7906067	329443	ieu-b-102	TRUE	reported	WYslv6
10	1.07E+08	T	C	-0.0294	0.005	3.68E-09	0.2481	NA	170756	rs1826169	329443	ieu-b-102	TRUE	reported	WYslv6
10	1.07E+08	C	G	-0.0303	0.005	1.34E-09	0.2458	NA	170756	rs7068414	329443	ieu-b-102	TRUE	reported	WYslv6
10	1.07E+08	T	C	-0.0301	0.005	1.74E-09	0.2458	NA	170756	rs7914784	329443	ieu-b-102	TRUE	reported	WYslv6
10	1.07E+08	T	C	-0.0293	0.005	4.24E-09	0.2481	NA	170756	rs1404785	329443	ieu-b-102	TRUE	reported	WYslv6
10	1.07E+08	A	G	-0.0293	0.005	3.85E-09	0.2481	NA	170756	rs7907899	329443	ieu-b-102	TRUE	reported	WYslv6
10	1.07E+08	G	A	-0.0294	0.005	3.39E-09	0.2481	NA	170756	rs7912102	329443	ieu-b-102	TRUE	reported	WYslv6
10	1.07E+08	T	C	-0.0294	0.005	3.78E-09	0.2481	NA	170756	rs7897270	329443	ieu-b-102	TRUE	reported	WYslv6
10	1.07E+08	C	G	-0.0294	0.005	3.77E-09	0.2481	NA	170756	rs7915656	329443	ieu-b-102	TRUE	reported	WYslv6
10	1.07E+08	T	A	-0.0294	0.005	3.68E-09	0.2474	NA	170756	rs6186732	329443	ieu-b-102	TRUE	reported	WYslv6
10	1.07E+08	G	A	-0.0304	0.005	1.33E-09	0.2451	NA	170756	rs3867472	329443	ieu-b-102	TRUE	reported	WYslv6
10	1.07E+08	C	A	-0.0301	0.005	1.69E-09	0.245	NA	170756	rs1050977	329443	ieu-b-102	TRUE	reported	WYslv6
10	1.07E+08	G	T	-0.0302	0.005	1.54E-09	0.2459	NA	170756	rs7068171	329443	ieu-b-102	TRUE	reported	WYslv6
10	1.07E+08	G	A	-0.03	0.005	1.81E-09	0.2459	NA	170756	rs7068586	329443	ieu-b-102	TRUE	reported	WYslv6
10	1.07E+08	T	C	-0.0292	0.005	4.70E-09	0.248	NA	170756	rs1586662	329443	ieu-b-102	TRUE	reported	WYslv6
10	1.07E+08	G	A	-0.0292	0.005	4.37E-09	0.248	NA	170756	rs7073438	329443	ieu-b-102	TRUE	reported	WYslv6
10	1.07E+08	C	T	-0.0301	0.005	1.72E-09	0.2459	NA	170756	rs6584630	329443	ieu-b-102	TRUE	reported	WYslv6
10	1.07E+08	A	G	-0.0292	0.005	4.51E-09	0.2481	NA	170756	rs1565425	329443	ieu-b-102	TRUE	reported	WYslv6
10	1.07E+08	T	C	-0.0302	0.005	1.55E-09	0.2458	NA	170756	rs1565424	329443	ieu-b-102	TRUE	reported	WYslv6
10	1.07E+08	C	T	-0.0304	0.005	1.19E-09	0.2456	NA	170756	rs6584631	329443	ieu-b-102	TRUE	reported	WYslv6
10	1.07E+08	C	T	-0.0294	0.005	3.80E-09	0.2477	NA	170756	rs7901022	329443	ieu-b-102	TRUE	reported	WYslv6
10	1.07E+08	C	T	-0.0294	0.005	3.41E-09	0.2477	NA	170756	rs5669351	329443	ieu-b-102	TRUE	reported	WYslv6
10	1.07E+08	G	A	-0.0291	0.005	5.44E-09	0.2476	NA	170756	rs2007698	329443	ieu-b-102	TRUE	reported	WYslv6
10	1.07E+08	G	A	-0.029	0.005	5.76E-09	0.2476	NA	170756	rs868009	329443	ieu-b-102	TRUE	reported	WYslv6
10	1.07E+08	G	A	-0.0299	0.005	2.15E-09	0.2451	NA	170756	rs1777502	329443	ieu-b-102	TRUE	reported	WYslv6
10	1.07E+08	C	T	-0.03	0.005	2.02E-09	0.2451	NA	170756	rs1263692	329443	ieu-b-102	TRUE	reported	WYslv6
10	1.07E+08	C	T	-0.0299	0.005	2.31E-09	0.2451	NA	170756	rs1777510	329443	ieu-b-102	TRUE	reported	WYslv6
10	1.07E+08	T	C	-0.03	0.005	1.93E-09	0.245	NA	170756	rs7912437	329443	ieu-b-102	TRUE	reported	WYslv6
10	1.07E+08	C	T	-0.03	0.005	1.96E-09	0.245	NA	170756	rs7896186	329443	ieu-b-102	TRUE	reported	WYslv6
10	1.07E+08	C	T	-0.0292	0.005	4.71E-09	0.2473	NA	170756	rs7896463	329443	ieu-b-102	TRUE	reported	WYslv6
10	1.07E+08	G	A	-0.03	0.005	1.93E-09	0.245	NA	170756	rs7901032	329443	ieu-b-102	TRUE	reported	WYslv6
10	1.07E+08	C	T	-0.0292	0.005	4.91E-09	0.2473	NA	170756	rs6584633	329443	ieu-b-102	TRUE	reported	WYslv6
10	1.07E+08	G	A	-0.03	0.005	1.83E-09	0.245	NA	170756	rs1525388	329443	ieu-b-102	TRUE	reported	WYslv6
10	1.07E+08	T	C	-0.0292	0.005	4.51E-09	0.2484	NA	170756	rs3430366	329443	ieu-b-102	TRUE	reported	WYslv6
10	1.07E+08	G	A	-0.0301	0.005	1.67E-09	0.2454	NA	170756	rs6186734	329443	ieu-b-102	TRUE	reported	WYslv6
10	1.07E+08	A	C	-0.0301	0.005	1.65E-09	0.2448	NA	170756	rs1159660	329443	ieu-b-102	TRUE	reported	WYslv6
10	1.07E+08	G	A	-0.0297	0.005	2.37E-09	0.2487	NA	170756	rs1224661	329443	ieu-b-102	TRUE	reported	WYslv6
10	1.07E+08	C	T	-0.0297	0.005	2.22E-09	0.2488	NA	170756	rs1224656	329443	ieu-b-102	TRUE	reported	WYslv6
10	1.07E+08	A	T	-0.0306	0.005	8.27E-10	0.2467	NA	170756	rs1119223	329443	ieu-b-102	TRUE	reported	WYslv6
10	1.07E+08	A	G	-0.0306	0.005	8.41E-10	0.2467	NA	170756	rs6055115	329443	ieu-b-102	TRUE	reported	WYslv6
10	1.07E+08	C	T	-0.0306	0.005	8.36E-10	0.2467	NA	170756	rs6584635	329443	ieu-b-102	TRUE	reported	WYslv6
10	1.07E+08	T	C	-0.0306	0.005	8.49E-10	0.2467	NA	170756	rs1711792	329443	ieu-b-102	TRUE	reported	WYslv6
10	1.07E+08	T	C	-0.0295	0.005	3.02E-09	0.2488	NA	170756	rs7086782	329443	ieu-b-102	TRUE	reported	WYslv6
10	1.07E+08	A	C	-0.0297	0.005	2.24E-09	0.2487	NA	170756	rs7068754	329443	ieu-b-102	TRUE	reported	WYslv6
10	1.07E+08	A	G	-0.0297	0.005	2.23E-09	0.2487	NA	170756	rs2177741	329443	ieu-b-102	TRUE	reported	WYslv6
10	1.07E+08	A	C	-0.0298	0.005	2.73E-09	0.2482	NA	170756	rs9606409	329443	ieu-b-102	TRUE	reported	WYslv6
10	1.07E+08	A	G	-0.029	0.005	5.05E-09	0.2496	NA	170756	rs7903869	329443	ieu-b-102	TRUE	reported	WYslv6
10	1.07E+08	G	A	-0.0295	0.005	2.73E-09	0.2505	NA	170756	rs7085496	329443	ieu-b-102	TRUE	reported	WYslv6
11	61471678	C	T	-0.0315	0.0056	1.90E-08	0.1886	NA	170756	rs198457	329443	ieu-b-102	TRUE	reported	WYslv6
11	88472034	T	C	-0.0264	0.0046	9.38E-09	0.3246	NA	170756	rs1102113	329443	ieu-b-102	TRUE	reported	WYslv6
11	88472070	G	A	-0.0265	0.0044	2.60E-09	0.3812	NA	170756	rs1102113	329443	ieu-b-102	TRUE	reported	WYslv6
11	88472271	T	A	-0.0264	0.0046	9.90E-09	0.3247	NA	170756	rs1227176	329443	ieu-b-102	TRUE	reported	WYslv6
11	88472369	T	C	-0.0282	0.0045	4.16E-10	0.3551	NA	170756	rs1903844	329443	ieu-b-102	TRUE	reported	WYslv6
11	88472482	C	T	-0.0285	0.0045	2.55E-10	0.3549	NA	170756	rs1903845	329443	ieu-b-102	TRUE	reported	WYslv6
11	88472856	G	A	-0.0266	0.0044	2.11E-09	0.3827	NA	170756	rs1083134	329443	ieu-b-102	TRUE	reported	WYslv6
11	88475777	G	A	0.0264	0.0044	2.36E-09	0.6186	NA	170756	rs1000061	329443	ieu-b-102	TRUE	reported	WYslv6
11	88475896	T	C	0.0275	0.0045	6.72E-10	0.6184	NA	170756	rs1000062	329443	ieu-b-102	TRUE	reported	WYslv6
11	88475938	A	G	0.0281	0.0045	4.49E-10	0.6485	NA	170756	rs1532547	329443	ieu-b-102	TRUE	reported	WYslv6
11	88476351	G	C	0.0273	0.0045	8.96E-10	0.6178	NA	170756	rs1903846	329443	ieu-b-102	TRUE	reported	WYslv6
11	88477214	T	C	0.0284	0.0045	2.75E-10	0.6464	NA	170756	rs1461826	329443	ieu-b-102	TRUE	reported	WYslv6
11	88477337	T	G	0.0278	0.0045	8.47E-10	0.6471	NA	170756	rs6483415	329443	ieu-b-102	TRUE	reported	WYslv6
11	88477340	G	T	0.0258	0.0045	7.17E-09	0.6192	NA	170756	rs6483416	329443	ieu-b-102	TRUE	reported	WYslv6
11	88477352	A	T	0.0278	0.0045	8.00E-10	0.6469	NA	170756	rs6483417	329443	ieu-b-102	TRUE	reported	WYslv6
11	88478226	G	A	-0.0279	0.0045	5.95E-10	0.3517	NA	170756	rs1279911	329443	ieu-b-102	TRUE	reported	WYslv6
11	88480235	C	A	-0.027	0.0046	5.67E-09	0.3184	NA	170756	rs3579040	329443	ieu-b-102	TRUE	reported	WYslv6
11	88485300	G	A	-0.0268	0.0046	5.25E-09	0.3261	NA	170756	rs4753182	329443	ieu-b-102	TRUE	reported	WYslv6
11	88493373	C	A	-0.0263	0.0046	9.30E-09	0.3256	NA	170756	rs4753184	329443	ieu-b-102	TRUE	reported	WYslv6
11	88505080	A	G	-0.0264	0.0046	8.69E-09	0.3259	NA	170756	rs1227559	329443	ieu-b-102	TRUE	reported	WYslv6
11	88506594	C	A	-0.0263	0.0046	1.07E-08	0.3252	NA	170756	rs1102128	329443	ieu-b-102	TRUE	reported	WYslv6
11	88515738	G	T	-0.0263	0.0046	9.58E-09	0.3261	NA	170756	rs1499189	329443	ieu-b-102	TRUE	reported	WYslv6
11	88520835	A	G	-0.0261	0.0046	1.24E-08	0.3266	NA	170756	rs1102133	329443	ieu-b-102	TRUE	reported	WYslv6
11	88529456	C	T	-0.0265	0.0047	1.42E-08	0.3096	NA	170756	rs3862367	329443	ieu-b-102	TRUE	reported	WYslv6
11	88529474	C	A	-0.0259	0.0046	1.76E-08	0.3237	NA	170756	rs3862368	329443	ieu-b-102	TRUE	reported	WYslv6
11	88530555	G	A	-0.0262	0.0046	1.31E-08	0.3228	NA	170756	rs1279779	329443	ieu-b-102	TRUE	reported	WYslv6
11	88545014	C	T	0.0256	0.0044	4.97E-09	0.4102	NA	170756	rs1279512	329443	ieu-b-102	TRUE	reported	WYslv6
11	88548272	A	G	-0.026	0.0046	1.28E-08	0.3273	NA	170756	rs1280158	329443	ieu-b-102	TRUE	reported	WYslv6
11	88549978	T	C	-0.026	0.0046	1.28E-08	0.3268	NA	170756	rs1102143	329443	ieu-b-102	TRUE	reported	WYslv6
11	88551984	T	C	-0.026	0.0046	1.31E-08	0.3272	NA	170756	rs1227992	329443	ieu-b-102	TRUE	reported	WYslv6
11	88574834	G	A	-0.0256	0.0046	2.10E-08	0.327	NA	170756	rs1083151	329443	ieu-b-102	TRUE	reported	WYslv6
11	88575037	A	G	0.0265	0.0046	6.12E-09	0.6647	NA	170756	rs643566	329443	ieu-b-102	TRUE	reported	WYslv6
11	88575159	T	A	0.0261	0.0046	1.19E-08	0.6723	NA	170756	rs1332490	329443	ieu-b-102	TRUE	reported	WYslv6
11	88578230	C	G	0.024	0.0044	4.94E-08	0.3873	NA	170756	rs1603897	329443	ieu-b-102	TRUE	reported	WYslv6
11	88581339	T	C	0.0267	0.0046	5.24E-09	0.6711	NA	170756	rs643304	329443	ieu-b-102	TRUE	reported	WYslv6
11	88583978	A	T	0.0262	0.0046	1.00E-08	0.6719	NA	170756	rs316087	329443	ieu-b-102	TRUE	reported	WYslv6
11	88584160	A	C	0.0268	0.0046	4.57E-09	0.6717	NA	170756	rs316086	329443	ieu-b-102	TRUE	reported	WYslv6
11	88586850	G	A	0.0263	0.0046	8.47E-09	0.6716	NA	170756	rs614116	329443	ieu-b-102	TRUE	reported	WYslv6
11	88588786	C	T	0.027	0.0046	3.36E-09	0.6689	NA	170756	rs645327	329443	ieu-b-102	TRUE	reported	WYslv6
11	88591918	T	C	-0.0268	0.0046	4.37E-09	0.3289	NA	170756	rs6190299	329443	ieu-b-102	TRUE	reported	WYslv6
11	88593326	C	G	0.0273	0.0046	2.69E-09	0.6722	NA	170756	rs316085	329443	ieu-b-102	TRUE	reported	WYslv6
11	88599826	A	G	0.0266	0.0046	7.19E-09	0.6729	NA	170756	rs652659	329443	ieu-b-102	TRUE	reported	WYslv6
11	88599914	T	A	0.0265	0.0046	7.84E-09	0.6729	NA	170756	rs568109	329443	ieu-b-102	TRUE	reported	WYslv6
11	88604170	G	A	0.0264	0.0046	7.90E-09	0.672	NA	170756	rs316083	329443	ieu-b-102	TRUE	reported	WYslv6

11	88606887	C	T	0.0262	0.0046	1.13E-08	0.6731	NA	170756	rs653421	329443	ieu-b-102	TRUE	reported	WYslv6
11	88608787	G	C	-0.0258	0.0046	1.89E-08	0.3259	NA	170756	rs9258790	329443	ieu-b-102	TRUE	reported	WYslv6
11	88614324	A	T	-0.0276	0.0046	2.79E-09	0.3198	NA	170756	rs1016051	329443	ieu-b-102	TRUE	reported	WYslv6
11	88615165	C	T	0.0257	0.0046	1.69E-08	0.6634	NA	170756	rs632604	329443	ieu-b-102	TRUE	reported	WYslv6
11	88615547	A	G	0.0255	0.0046	2.80E-08	0.6426	NA	170756	rs681820	329443	ieu-b-102	TRUE	reported	WYslv6
11	88615667	C	T	0.0256	0.0046	1.84E-08	0.6631	NA	170756	rs682290	329443	ieu-b-102	TRUE	reported	WYslv6
11	88616758	A	G	0.0257	0.0046	1.78E-08	0.6649	NA	170756	rs589675	329443	ieu-b-102	TRUE	reported	WYslv6
11	88616875	C	A	-0.0256	0.0046	1.83E-08	0.3348	NA	170756	rs1043758	329443	ieu-b-102	TRUE	reported	WYslv6
11	88617288	A	G	0.0258	0.0046	1.54E-08	0.6636	NA	170756	rs655869	329443	ieu-b-102	TRUE	reported	WYslv6
11	88617341	C	T	0.0258	0.0046	1.49E-08	0.6634	NA	170756	rs591893	329443	ieu-b-102	TRUE	reported	WYslv6
11	88628297	G	A	-0.0242	0.0043	2.54E-08	0.5816	NA	170756	rs316097	329443	ieu-b-102	TRUE	reported	WYslv6
11	88629072	G	A	0.0247	0.0044	1.76E-08	0.3988	NA	170756	rs584412	329443	ieu-b-102	TRUE	reported	WYslv6
11	88629801	G	A	0.0249	0.0044	1.34E-08	0.3993	NA	170756	rs604179	329443	ieu-b-102	TRUE	reported	WYslv6
11	88630395	T	G	-0.0242	0.0043	2.61E-08	0.5823	NA	170756	rs316092	329443	ieu-b-102	TRUE	reported	WYslv6
11	88630581	C	T	-0.0239	0.0043	3.79E-08	0.5809	NA	170756	rs514641	329443	ieu-b-102	TRUE	reported	WYslv6
11	88631922	C	T	-0.0237	0.0043	4.77E-08	0.5809	NA	170756	rs1139341	329443	ieu-b-102	TRUE	reported	WYslv6
11	88632972	C	T	-0.0237	0.0043	4.44E-08	0.5637	NA	170756	rs594561	329443	ieu-b-102	TRUE	reported	WYslv6
11	88633985	G	A	-0.0256	0.0044	5.07E-09	0.5947	NA	170756	rs582994	329443	ieu-b-102	TRUE	reported	WYslv6
11	88633993	G	T	0.026	0.0045	6.11E-09	0.363	NA	170756	rs585473	329443	ieu-b-102	TRUE	reported	WYslv6
11	88634036	G	C	0.0268	0.0044	1.33E-09	0.3847	NA	170756	rs585423	329443	ieu-b-102	TRUE	reported	WYslv6
11	88634197	G	T	-0.0264	0.0044	1.67E-09	0.5961	NA	170756	rs504183	329443	ieu-b-102	TRUE	reported	WYslv6
11	88635135	T	C	0.026	0.0044	4.77E-09	0.3666	NA	170756	rs598758	329443	ieu-b-102	TRUE	reported	WYslv6
11	88635823	T	C	-0.0241	0.0043	2.53E-08	0.5641	NA	170756	rs601882	329443	ieu-b-102	TRUE	reported	WYslv6
11	88636910	G	A	0.0255	0.0045	1.13E-08	0.3627	NA	170756	rs6812928	329443	ieu-b-102	TRUE	reported	WYslv6
11	88639670	T	C	-0.0242	0.0043	2.14E-08	0.5639	NA	170756	rs488006	329443	ieu-b-102	TRUE	reported	WYslv6
11	88640415	C	T	-0.0236	0.0043	4.91E-08	0.563	NA	170756	rs316090	329443	ieu-b-102	TRUE	reported	WYslv6
11	88640570	T	C	0.0258	0.0044	6.82E-09	0.3672	NA	170756	rs650222	329443	ieu-b-102	TRUE	reported	WYslv6
11	88643077	T	A	0.0239	0.0044	4.86E-08	0.3971	NA	170756	rs584552	329443	ieu-b-102	TRUE	reported	WYslv6
11	88649077	T	C	0.0259	0.0044	5.55E-09	0.3677	NA	170756	rs658708	329443	ieu-b-102	TRUE	reported	WYslv6
11	88650187	T	A	-0.0241	0.0043	2.48E-08	0.5636	NA	170756	rs643340	329443	ieu-b-102	TRUE	reported	WYslv6
11	88650460	C	A	-0.0242	0.0043	2.05E-08	0.5636	NA	170756	rs642078	329443	ieu-b-102	TRUE	reported	WYslv6
11	88650737	A	C	-0.0245	0.0043	1.45E-08	0.5645	NA	170756	rs630573	329443	ieu-b-102	TRUE	reported	WYslv6
11	88650997	A	T	-0.0241	0.0044	3.41E-08	0.5818	NA	170756	rs629600	329443	ieu-b-102	TRUE	reported	WYslv6
11	88651047	T	C	0.0266	0.0044	2.18E-09	0.3645	NA	170756	rs640211	329443	ieu-b-102	TRUE	reported	WYslv6
11	88651367	A	C	-0.0238	0.0044	4.76E-08	0.5817	NA	170756	rs664731	329443	ieu-b-102	TRUE	reported	WYslv6
11	88652346	A	G	0.0261	0.0044	4.30E-09	0.3681	NA	170756	rs7927616	329443	ieu-b-102	TRUE	reported	WYslv6
11	88652506	C	G	-0.0243	0.0043	2.34E-08	0.5632	NA	170756	rs3016248	329443	ieu-b-102	TRUE	reported	WYslv6
11	88653700	G	A	0.0267	0.0044	1.97E-09	0.3646	NA	170756	rs1279874	329443	ieu-b-102	TRUE	reported	WYslv6
11	88654192	T	C	-0.0241	0.0043	2.55E-08	0.5638	NA	170756	rs621230	329443	ieu-b-102	TRUE	reported	WYslv6
11	88654815	C	T	0.0262	0.0044	3.95E-09	0.3664	NA	170756	rs573912	329443	ieu-b-102	TRUE	reported	WYslv6
11	88655165	T	C	-0.0261	0.0044	2.50E-09	0.5938	NA	170756	rs687767	329443	ieu-b-102	TRUE	reported	WYslv6
11	88655388	A	G	0.0262	0.0044	3.74E-09	0.3665	NA	170756	rs686845	329443	ieu-b-102	TRUE	reported	WYslv6
11	88656762	A	G	-0.0262	0.0044	2.24E-09	0.5942	NA	170756	rs647669	329443	ieu-b-102	TRUE	reported	WYslv6
11	88657026	A	G	0.0265	0.0044	1.72E-09	0.3886	NA	170756	rs1555042	329443	ieu-b-102	TRUE	reported	WYslv6
11	88657674	A	G	-0.0245	0.0043	1.54E-08	0.5635	NA	170756	rs614868	329443	ieu-b-102	TRUE	reported	WYslv6
11	88658836	T	G	0.0265	0.0044	2.60E-09	0.3663	NA	170756	rs528637	329443	ieu-b-102	TRUE	reported	WYslv6
11	88659892	A	G	-0.0245	0.0043	1.60E-08	0.5637	NA	170756	rs584784	329443	ieu-b-102	TRUE	reported	WYslv6
11	88660220	C	G	0.0264	0.0045	3.08E-09	0.366	NA	170756	rs613460	329443	ieu-b-102	TRUE	reported	WYslv6
11	88663199	T	G	0.0253	0.0045	1.48E-08	0.3616	NA	170756	rs493306	329443	ieu-b-102	TRUE	reported	WYslv6
11	88665692	A	G	-0.0239	0.0043	3.47E-08	0.5628	NA	170756	rs538601	329443	ieu-b-102	TRUE	reported	WYslv6
11	88672449	C	G	0.0265	0.0045	3.91E-09	0.3585	NA	170756	rs573060	329443	ieu-b-102	TRUE	reported	WYslv6
11	88672564	T	C	0.0265	0.0045	4.14E-09	0.3586	NA	170756	rs633748	329443	ieu-b-102	TRUE	reported	WYslv6
11	88672976	T	C	0.0264	0.0045	4.23E-09	0.3589	NA	170756	rs643216	329443	ieu-b-102	TRUE	reported	WYslv6
11	88676854	G	T	0.0248	0.0045	3.02E-08	0.3691	NA	170756	rs655683	329443	ieu-b-102	TRUE	reported	WYslv6
11	88677314	T	C	-0.025	0.0044	1.22E-08	0.5856	NA	170756	rs557505	329443	ieu-b-102	TRUE	reported	WYslv6
11	88677349	C	T	0.0266	0.0044	2.00E-09	0.3889	NA	170756	rs558249	329443	ieu-b-102	TRUE	reported	WYslv6
11	88677665	C	T	-0.0265	0.0044	2.15E-09	0.6064	NA	170756	rs669480	329443	ieu-b-102	TRUE	reported	WYslv6
11	88679449	G	A	-0.0254	0.0044	7.34E-09	0.5851	NA	170756	rs602040	329443	ieu-b-102	TRUE	reported	WYslv6
11	88679798	A	G	-0.0249	0.0044	1.29E-08	0.5876	NA	170756	rs7805573	329443	ieu-b-102	TRUE	reported	WYslv6
11	88681359	C	T	0.025	0.0044	1.19E-08	0.412	NA	170756	rs1338388	329443	ieu-b-102	TRUE	reported	WYslv6
11	88686164	T	C	-0.0249	0.0044	1.50E-08	0.5876	NA	170756	rs561940	329443	ieu-b-102	TRUE	reported	WYslv6
11	88686594	T	A	-0.025	0.0044	1.23E-08	0.5875	NA	170756	rs612781	329443	ieu-b-102	TRUE	reported	WYslv6
11	88686678	T	G	-0.0249	0.0044	1.30E-08	0.5875	NA	170756	rs624913	329443	ieu-b-102	TRUE	reported	WYslv6
11	88687580	A	G	0.0269	0.0044	1.13E-09	0.3902	NA	170756	rs598134	329443	ieu-b-102	TRUE	reported	WYslv6
11	88688233	T	C	-0.0277	0.0043	1.37E-10	0.5171	NA	170756	rs597462	329443	ieu-b-102	TRUE	reported	WYslv6
11	88688830	T	C	-0.0275	0.0043	1.85E-10	0.5163	NA	170756	rs538955	329443	ieu-b-102	TRUE	reported	WYslv6
11	88689953	G	A	-0.0278	0.0043	1.14E-10	0.5177	NA	170756	rs2012923	329443	ieu-b-102	TRUE	reported	WYslv6
11	88690174	T	G	0.0275	0.0043	1.75E-10	0.479	NA	170756	rs572735	329443	ieu-b-102	TRUE	reported	WYslv6
11	88690814	T	A	0.0275	0.0043	1.90E-10	0.4794	NA	170756	rs546980	329443	ieu-b-102	TRUE	reported	WYslv6
11	88693073	T	C	0.0265	0.0043	7.91E-10	0.4603	NA	170756	rs604551	329443	ieu-b-102	TRUE	reported	WYslv6
11	88694072	T	C	-0.0277	0.0043	1.24E-10	0.5166	NA	170756	rs627387	329443	ieu-b-102	TRUE	reported	WYslv6
11	88694470	A	G	0.0267	0.0043	6.39E-10	0.4603	NA	170756	rs496939	329443	ieu-b-102	TRUE	reported	WYslv6
11	88694594	T	C	0.0278	0.0043	1.16E-10	0.4792	NA	170756	rs538945	329443	ieu-b-102	TRUE	reported	WYslv6
11	88695383	C	T	0.0279	0.0043	1.18E-10	0.4395	NA	170756	rs651890	329443	ieu-b-102	TRUE	reported	WYslv6
11	88695846	A	G	-0.0278	0.0043	1.15E-10	0.5176	NA	170756	rs1150313	329443	ieu-b-102	TRUE	reported	WYslv6
11	88695883	C	T	0.028	0.0043	1.08E-10	0.4396	NA	170756	rs564567	329443	ieu-b-102	TRUE	reported	WYslv6
11	88698066	T	A	-0.0277	0.0043	1.23E-10	0.5177	NA	170756	rs516476	329443	ieu-b-102	TRUE	reported	WYslv6
11	88698680	T	G	-0.0276	0.0043	1.41E-10	0.5175	NA	170756	rs604846	329443	ieu-b-102	TRUE	reported	WYslv6
11	88701202	A	G	-0.0277	0.0043	1.25E-10	0.5214	NA	170756	rs677483	329443	ieu-b-102	TRUE	reported	WYslv6
11	88701329	A	C	-0.0279	0.0043	9.58E-11	0.5173	NA	170756	rs677976	329443	ieu-b-102	TRUE	reported	WYslv6
11	88703390	T	C	-0.0273	0.0043	2.30E-10	0.5226	NA	170756	rs561512	329443	ieu-b-102	TRUE	reported	WYslv6
11	88704092	T	C	-0.0274	0.0043	2.14E-10	0.5183	NA	170756	rs567990	329443	ieu-b-102	TRUE	reported	WYslv6
11	88704918	T	C	-0.028	0.0043	8.13E-11	0.5168	NA	170756	rs534815	329443	ieu-b-102	TRUE	reported	WYslv6
11	88705235	T	C	-0.0279	0.0043	9.13E-11	0.5177	NA	170756	rs537635	329443	ieu-b-102	TRUE	reported	WYslv6
11	88712383	A	G	-0.0281	0.0043	7.12E-11	0.5223	NA	170756	rs524874	329443	ieu-b-102	TRUE	reported	WYslv6
11	88716140	T	A	-0.0278	0.0043	1.09E-10	0.5207	NA	170756	rs513762	329443	ieu-b-102	TRUE	reported	WYslv6
11	88719052	T	C	-0.0278	0.0043	9.84E-11	0.5202	NA	170756	rs492612	329443	ieu-b-102	TRUE	reported	WYslv6
11	88719347	T	C	-0.0278	0.0043	1.23E-10	0.516	NA	170756	rs1283430	329443	ieu-b-102	TRUE	reported	WYslv6
11	88720235	C	T	-0.0278	0.0043	1.43E-10	0.5167	NA	170756	rs648826	329443	ieu-b-102	TRUE	reported	WYslv6
11	88720486	A	G	-0.0281	0.0044	1.13E-10	0.5186	NA	170756	rs631861	329443	ieu-b-102	TRUE	reported	WYslv6
11	88720594	T	C	-0.0289	0.0045	1.34E-10	0.4947	NA	170756	rs7482675	329443	ieu-b-102	TRUE	reported	WYslv6
11	88720813	G	C	-0.0266	0.0043	8.27E-10	0.5049	NA	170756	rs7478753	329443	ieu-b-102	TRUE	reported	WYslv6
11	88721458	C	T	-0.0291	0.0044	3.59E-11	0.5069	NA	170756	rs7117646	329443	ieu-b-102	TRUE	reported	WYslv6
11	88726033	C	T	-0.0279	0.0043	8.66E-11	0.5165	NA	170756	rs1229118	329443	ieu-b-102	TRUE	reported	WYslv6
11	88726224	A	T	-0.0282	0.0043	5.53E-11	0.5164	NA	170756	rs1174832	329443	ieu-b-102	TRUE	reported	WYslv6
11	88726465	T	A	-0.0284	0.0043	6.30E-11	0.5148	NA	170756	rs1227059	329443	ieu-b-102	TRUE	reported	WYslv6
11	88726515	G	A	-0.0282	0.0043	6.44E-11	0.5215	NA	170756	rs7358491	329443	ieu-b-102	TRUE	reported	WYslv6
11	88731228	G	A	-0.0266	0.0043	6.18E-10	0.5041	NA	170756	rs1076581	329443	ieu-b-102	TRUE	reported	WYslv6
11	88732591	T	C	-0.0283	0.0043	6.83E-11	0.5211	NA	170756	rs4517513	329443	ieu-b-102	TRUE	reported	WYslv6
11	88732956	T	C	-0.0294	0.0044	3.35E-11	0.4952	NA	170756	rs1083154	329443	ieu-b-102	TRUE	reported	WYslv6
11	88734123	A	G	-0.0277	0.0043	1.17E-10	0.516	NA	170756	rs1076582	329443	ieu-b-102	TRUE	reported	WYslv6
11	88734948	A	T	-0.028	0.0043	7.69E-11	0.5189	NA	170756	rs1076582	329443	ieu-b-102	TRUE	reported	WYslv6
11	88739932	A	G	-0.0274	0.0044	4.02E-10	0.5458	NA	170756	rs1228376	329443	ieu-b-102	TRUE	reported	WYslv6
11	88740608	A	G	-0.0276	0.0043	1.97E-10	0.5549	NA	170756	rs1074129	329443	ieu-b-102	TRUE	reported	WYslv6
11	88740827	T	G	-0.0286	0.0043	3.84E-11	0.5199	NA	170756	rs1076518	329443	ieu-b-102	TRUE	reported	WYslv6
11	88740896	A	G	-0.0276	0.0043	2.02E-10	0.5542	NA	170756	rs1073411	329443	ieu-b-102	TRUE	reported	WYslv6
11	88741390	T	C	-0.0276	0.0043	2.19E-10	0.5542	NA	170756	rs7127754	329443	ieu-b-102	TRUE	reported	WYslv6

11	88742518	A	G	-0.027	0.0044	6.90E-10	0.5557	NA	170756	rs1076518	329443	ieu-b-102	TRUE	reported	WYslv6
11	88742578	A	C	-0.0276	0.0043	2.08E-10	0.5545	NA	170756	rs1076518	329443	ieu-b-102	TRUE	reported	WYslv6
11	88743208	T	A	-0.0276	0.0043	2.01E-10	0.5545	NA	170756	rs4753209	329443	ieu-b-102	TRUE	reported	WYslv6
11	88743265	C	G	-0.0271	0.0043	4.02E-10	0.5544	NA	170756	rs4753210	329443	ieu-b-102	TRUE	reported	WYslv6
11	88743356	C	G	-0.0277	0.0043	1.86E-10	0.5543	NA	170756	rs4753211	329443	ieu-b-102	TRUE	reported	WYslv6
11	88744035	A	G	-0.0275	0.0043	2.52E-10	0.554	NA	170756	rs1083019	329443	ieu-b-102	TRUE	reported	WYslv6
11	88744416	A	G	-0.0281	0.0044	1.09E-10	0.5528	NA	170756	rs7929619	329443	ieu-b-102	TRUE	reported	WYslv6
11	88744425	T	C	-0.0281	0.0044	9.82E-11	0.553	NA	170756	rs7932640	329443	ieu-b-102	TRUE	reported	WYslv6
11	88745439	T	C	-0.0282	0.0043	7.55E-11	0.5189	NA	170756	rs1083020	329443	ieu-b-102	TRUE	reported	WYslv6
11	88745739	C	G	-0.0273	0.0044	4.05E-10	0.554	NA	170756	rs1076518	329443	ieu-b-102	TRUE	reported	WYslv6
11	88748162	A	G	-0.0295	0.0044	2.89E-11	0.4948	NA	170756	rs1555061	329443	ieu-b-102	TRUE	reported	WYslv6
11	88749072	C	T	-0.0273	0.0043	3.63E-10	0.556	NA	170756	rs2226563	329443	ieu-b-102	TRUE	reported	WYslv6
11	88752128	A	T	-0.025	0.0043	8.07E-09	0.5225	NA	170756	rs1208560	329443	ieu-b-102	TRUE	reported	WYslv6
11	88752567	G	C	-0.0265	0.0043	8.80E-10	0.5046	NA	170756	rs4370938	329443	ieu-b-102	TRUE	reported	WYslv6
11	88753845	T	C	-0.0271	0.0044	5.03E-10	0.5532	NA	170756	rs9270596	329443	ieu-b-102	TRUE	reported	WYslv6
11	88755348	G	A	-0.0274	0.0044	3.43E-10	0.5567	NA	170756	rs7967935	329443	ieu-b-102	TRUE	reported	WYslv6
11	88756713	G	A	0.0287	0.0044	8.27E-11	0.4076	NA	170756	rs3600642	329443	ieu-b-102	TRUE	reported	WYslv6
11	88756779	T	C	0.0291	0.0044	2.93E-11	0.44	NA	170756	rs4497414	329443	ieu-b-102	TRUE	reported	WYslv6
11	88757597	C	T	0.0286	0.0044	8.99E-11	0.4078	NA	170756	rs1043081	329443	ieu-b-102	TRUE	reported	WYslv6
11	88757950	C	A	0.0282	0.0044	1.08E-10	0.4565	NA	170756	rs7125164	329443	ieu-b-102	TRUE	reported	WYslv6
11	88758721	T	G	0.0288	0.0044	7.48E-11	0.4102	NA	170756	rs1043081	329443	ieu-b-102	TRUE	reported	WYslv6
11	88760806	G	A	0.0285	0.0044	1.08E-10	0.4071	NA	170756	rs1222411	329443	ieu-b-102	TRUE	reported	WYslv6
11	88760872	G	A	0.0287	0.0044	5.48E-11	0.4392	NA	170756	rs7114254	329443	ieu-b-102	TRUE	reported	WYslv6
11	88762042	G	A	0.0283	0.0045	2.74E-10	0.4004	NA	170756	rs1083020	329443	ieu-b-102	TRUE	reported	WYslv6
11	88762060	C	T	0.0281	0.0044	2.08E-10	0.4352	NA	170756	rs1074129	329443	ieu-b-102	TRUE	reported	WYslv6
11	88762668	A	G	0.0284	0.0044	9.42E-11	0.4432	NA	170756	rs7108722	329443	ieu-b-102	TRUE	reported	WYslv6
11	88765779	G	A	-0.0244	0.0043	1.57E-08	0.486	NA	170756	rs1101843	329443	ieu-b-102	TRUE	reported	WYslv6
11	88767496	G	A	-0.0243	0.0044	3.46E-08	0.57	NA	170756	rs4611203	329443	ieu-b-102	TRUE	reported	WYslv6
11	88768842	G	A	0.0269	0.0046	4.25E-09	0.3329	NA	170756	rs1779197	329443	ieu-b-102	TRUE	reported	WYslv6
11	88779776	T	C	0.0278	0.0046	1.02E-09	0.3369	NA	170756	rs1228478	329443	ieu-b-102	TRUE	reported	WYslv6
11	88787070	G	A	0.0272	0.0046	2.48E-09	0.3345	NA	170756	rs1101844	329443	ieu-b-102	TRUE	reported	WYslv6
11	88787299	G	C	0.0269	0.0046	3.89E-09	0.3321	NA	170756	rs1101844	329443	ieu-b-102	TRUE	reported	WYslv6
11	88787544	G	A	-0.0248	0.0043	9.54E-09	0.4872	NA	170756	rs1225430	329443	ieu-b-102	TRUE	reported	WYslv6
11	88788880	C	G	0.0271	0.0046	3.12E-09	0.3321	NA	170756	rs1101844	329443	ieu-b-102	TRUE	reported	WYslv6
11	88793485	T	C	0.0267	0.0046	4.83E-09	0.3335	NA	170756	rs3595057	329443	ieu-b-102	TRUE	reported	WYslv6
11	88797386	C	T	0.0268	0.0046	5.96E-09	0.3333	NA	170756	rs1101844	329443	ieu-b-102	TRUE	reported	WYslv6
11	88798185	G	A	0.0267	0.0046	4.83E-09	0.3334	NA	170756	rs6734621	329443	ieu-b-102	TRUE	reported	WYslv6
11	88799517	G	C	0.0271	0.0046	2.70E-09	0.3361	NA	170756	rs1257701	329443	ieu-b-102	TRUE	reported	WYslv6
11	88799731	T	C	0.027	0.0049	4.11E-08	0.2663	NA	170756	rs3474969	329443	ieu-b-102	TRUE	reported	WYslv6
11	88804684	A	C	-0.0249	0.0043	7.29E-09	0.4914	NA	170756	rs7929744	329443	ieu-b-102	TRUE	reported	WYslv6
11	88805066	T	C	0.0277	0.0046	1.32E-09	0.336	NA	170756	rs1236590	329443	ieu-b-102	TRUE	reported	WYslv6
11	88814870	A	G	0.0259	0.0045	6.78E-09	0.3697	NA	170756	rs7127487	329443	ieu-b-102	TRUE	reported	WYslv6
11	88815760	A	G	0.0274	0.0046	1.86E-09	0.3369	NA	170756	rs1101845	329443	ieu-b-102	TRUE	reported	WYslv6
11	88816137	T	A	0.0257	0.0045	8.56E-09	0.3693	NA	170756	rs1083020	329443	ieu-b-102	TRUE	reported	WYslv6
11	88816538	C	T	0.026	0.0045	6.16E-09	0.366	NA	170756	rs4121738	329443	ieu-b-102	TRUE	reported	WYslv6
11	88819742	G	C	0.0276	0.0046	1.73E-09	0.3365	NA	170756	rs1101846	329443	ieu-b-102	TRUE	reported	WYslv6
11	88821114	G	C	0.0276	0.0046	1.42E-09	0.3355	NA	170756	rs1101846	329443	ieu-b-102	TRUE	reported	WYslv6
11	88822199	T	C	0.0258	0.0045	1.24E-08	0.3613	NA	170756	rs1101846	329443	ieu-b-102	TRUE	reported	WYslv6
11	88822368	G	A	0.0254	0.0045	1.87E-08	0.3455	NA	170756	rs1101846	329443	ieu-b-102	TRUE	reported	WYslv6
11	88824176	C	T	0.0259	0.0045	6.53E-09	0.3662	NA	170756	rs1942490	329443	ieu-b-102	TRUE	reported	WYslv6
11	88825663	G	T	0.0257	0.0045	8.62E-09	0.3651	NA	170756	rs1101846	329443	ieu-b-102	TRUE	reported	WYslv6
11	88826343	G	A	0.0257	0.0045	8.35E-09	0.3655	NA	170756	rs3921012	329443	ieu-b-102	TRUE	reported	WYslv6
11	88826626	A	C	0.0277	0.0046	1.64E-09	0.3335	NA	170756	rs1101846	329443	ieu-b-102	TRUE	reported	WYslv6
11	88826813	T	C	0.0256	0.0045	1.01E-08	0.3674	NA	170756	rs1076518	329443	ieu-b-102	TRUE	reported	WYslv6
11	88828356	T	C	-0.0261	0.0043	1.46E-09	0.5049	NA	170756	rs1083020	329443	ieu-b-102	TRUE	reported	WYslv6
11	88828429	T	A	-0.0259	0.0046	2.14E-08	0.3552	NA	170756	rs6190338	329443	ieu-b-102	TRUE	reported	WYslv6
11	88830495	T	C	0.0256	0.0045	9.97E-09	0.3697	NA	170756	rs7944714	329443	ieu-b-102	TRUE	reported	WYslv6
11	88830751	A	G	0.0257	0.0045	9.14E-09	0.3699	NA	170756	rs7941957	329443	ieu-b-102	TRUE	reported	WYslv6
11	88831103	G	A	0.0252	0.0045	2.33E-08	0.3493	NA	170756	rs1101847	329443	ieu-b-102	TRUE	reported	WYslv6
11	88831337	A	T	-0.0261	0.0043	1.54E-09	0.5022	NA	170756	rs1076518	329443	ieu-b-102	TRUE	reported	WYslv6
11	88834157	G	T	0.026	0.0045	6.04E-09	0.3658	NA	170756	rs9665831	329443	ieu-b-102	TRUE	reported	WYslv6
11	88834258	G	A	0.028	0.0046	1.15E-09	0.3323	NA	170756	rs9665832	329443	ieu-b-102	TRUE	reported	WYslv6
11	88836328	C	T	0.0276	0.0046	1.81E-09	0.3337	NA	170756	rs4121733	329443	ieu-b-102	TRUE	reported	WYslv6
11	88838984	A	G	-0.0263	0.0043	1.09E-09	0.5018	NA	170756	rs1083020	329443	ieu-b-102	TRUE	reported	WYslv6
11	88840925	G	A	-0.0263	0.0043	1.39E-09	0.4952	NA	170756	rs1942496	329443	ieu-b-102	TRUE	reported	WYslv6
11	88841459	T	C	0.0257	0.0045	1.49E-08	0.3499	NA	170756	rs1942497	329443	ieu-b-102	TRUE	reported	WYslv6
11	88842024	T	C	0.0273	0.0046	1.96E-09	0.3376	NA	170756	rs1280480	329443	ieu-b-102	TRUE	reported	WYslv6
11	88844060	A	G	0.0257	0.0047	3.34E-08	0.3548	NA	170756	rs7925102	329443	ieu-b-102	TRUE	reported	WYslv6
11	88844343	C	A	0.0255	0.0045	1.64E-08	0.3499	NA	170756	rs1076518	329443	ieu-b-102	TRUE	reported	WYslv6
11	88845435	C	A	0.0256	0.0045	9.99E-09	0.3672	NA	170756	rs1101847	329443	ieu-b-102	TRUE	reported	WYslv6
11	88845892	T	C	-0.026	0.0043	1.96E-09	0.5037	NA	170756	rs1565368	329443	ieu-b-102	TRUE	reported	WYslv6
11	88848859	G	A	0.025	0.0045	2.10E-08	0.3666	NA	170756	rs2156123	329443	ieu-b-102	TRUE	reported	WYslv6
11	88850382	A	T	0.0253	0.0045	2.73E-08	0.3487	NA	170756	rs4420272	329443	ieu-b-102	TRUE	reported	WYslv6
11	88850924	G	A	0.0264	0.0046	7.35E-09	0.3366	NA	170756	rs4623905	329443	ieu-b-102	TRUE	reported	WYslv6
11	88851105	G	T	0.0253	0.0045	2.70E-08	0.3484	NA	170756	rs1083021	329443	ieu-b-102	TRUE	reported	WYslv6
11	88851967	A	G	0.0252	0.0045	2.84E-08	0.3486	NA	170756	rs7480884	329443	ieu-b-102	TRUE	reported	WYslv6
11	88854535	A	G	0.0252	0.0045	1.72E-08	0.3688	NA	170756	rs1083021	329443	ieu-b-102	TRUE	reported	WYslv6
11	88855628	G	A	0.0249	0.0045	3.75E-08	0.3454	NA	170756	rs1942484	329443	ieu-b-102	TRUE	reported	WYslv6
11	88856292	G	A	0.0266	0.0045	4.56E-09	0.3392	NA	170756	rs1236332	329443	ieu-b-102	TRUE	reported	WYslv6
11	88856423	G	C	0.0264	0.0045	6.04E-09	0.3406	NA	170756	rs1236364	329443	ieu-b-102	TRUE	reported	WYslv6
11	88856782	T	G	0.0264	0.0045	6.02E-09	0.3406	NA	170756	rs1942486	329443	ieu-b-102	TRUE	reported	WYslv6
11	88857653	G	A	0.0252	0.0045	2.73E-08	0.3453	NA	170756	rs1101848	329443	ieu-b-102	TRUE	reported	WYslv6
11	88858397	G	A	0.025	0.0045	2.55E-08	0.3646	NA	170756	rs1083021	329443	ieu-b-102	TRUE	reported	WYslv6
11	88860927	T	C	0.0252	0.0045	1.56E-08	0.3696	NA	170756	rs7925346	329443	ieu-b-102	TRUE	reported	WYslv6
11	88862822	A	T	0.0264	0.0045	6.28E-09	0.3408	NA	170756	rs1779291	329443	ieu-b-102	TRUE	reported	WYslv6
11	88863140	G	T	0.0252	0.0045	1.59E-08	0.3697	NA	170756	rs4121729	329443	ieu-b-102	TRUE	reported	WYslv6
11	88865114	A	G	0.0264	0.0045	6.75E-09	0.3407	NA	170756	rs1236351	329443	ieu-b-102	TRUE	reported	WYslv6
11	88866026	C	T	0.025	0.0045	3.31E-08	0.3465	NA	170756	rs5620150	329443	ieu-b-102	TRUE	reported	WYslv6
11	88868611	G	A	0.0251	0.0045	1.95E-08	0.3682	NA	170756	rs2843749	329443	ieu-b-102	TRUE	reported	WYslv6
11	88869323	C	G	0.026	0.0046	1.18E-08	0.3393	NA	170756	rs1083021	329443	ieu-b-102	TRUE	reported	WYslv6
11	88871260	G	C	0.0266	0.0046	5.89E-09	0.3368	NA	170756	rs7103137	329443	ieu-b-102	TRUE	reported	WYslv6
11	88872164	G	A	0.0267	0.0046	5.33E-09	0.3366	NA	170756	rs1083021	329443	ieu-b-102	TRUE	reported	WYslv6
11	88872509	A	T	0.0266	0.0046	5.70E-09	0.3369	NA	170756	rs1083021	329443	ieu-b-102	TRUE	reported	WYslv6
11	88873294	C	G	0.0271	0.0046	3.15E-09	0.3363	NA	170756	rs3548667	329443	ieu-b-102	TRUE	reported	WYslv6
11	88873421	T	C	0.0273	0.0046	3.51E-09	0.3356	NA	170756	rs1101850	329443	ieu-b-102	TRUE	reported	WYslv6
11	88874152	G	C	-0.0275	0.0043	2.08E-10	0.512	NA	170756	rs1083022	329443	ieu-b-102	TRUE	reported	WYslv6
11	88874969	T	C	-0.0267	0.0043	6.07E-10	0.5188	NA	170756	rs4121744	329443	ieu-b-102	TRUE	reported	WYslv6
11	88876845	A	G	-0.0266	0.0043	6.63E-10	0.5156	NA	170756	rs7342208	329443	ieu-b-102	TRUE	reported	WYslv6
11	88877463	A	G	-0.0268	0.0043	5.20E-10	0.5172	NA	170756	rs1083022	329443	ieu-b-102	TRUE	reported	WYslv6
11	88878052	A	T	-0.0271	0.0043	3.27E-10	0.5164	NA	170756	rs4315072	329443	ieu-b-102	TRUE	reported	WYslv6
11	88880402	G	A	-0.0267	0.0043	5.71E-10	0.5162	NA	170756	rs1365696	329443	ieu-b-102	TRUE	reported	WYslv6
11	88882550	G	A	-0.0267	0.0043	5.72E-10	0.5157	NA	170756	rs1076519	329443	ieu-b-102	TRUE	reported	WYslv6
11	88883499	A	G	-0.0264	0.0043	7.99E-10	0.5152	NA	170756	rs1101851	329443	ieu-b-102	TRUE	reported	WYslv6
11	88883648	C	A	-0.0267	0.0043	5.65E-10	0.5149	NA	170756	rs1101851	329443	ieu-b-102	TRUE	reported	WYslv6
11	88883885	A	G	-0.0263	0.0043	1.03E-09	0.5151	NA	170756	rs1083022	329443	ieu-b-102	TRUE	reported	WYslv6
11	88884079	T	C	-0.0276	0.0043	1.58E-10	0.5228	NA	170756	rs1083022	329443	ieu-b-102	TRUE	reported	WYslv6
11	88885187	C	T	-0.0278	0.0043	1.21E-10	0.5231	NA	170756	rs4255542	329443	ieu-b-102	TRUE	reported	WYslv6

11	88885480	C	T	-0.0267	0.0043	5.33E-10	0.5141	NA	170756	rs4397857	329443	ieu-b-102	TRUE	reported	WYslv6
11	88885945	G	A	-0.0278	0.0043	1.02E-10	0.5248	NA	170756	rs1083022	329443	ieu-b-102	TRUE	reported	WYslv6
11	88886767	A	T	-0.0271	0.0043	4.16E-10	0.5229	NA	170756	rs4309163	329443	ieu-b-102	TRUE	reported	WYslv6
11	88888262	C	T	-0.025	0.0044	1.18E-08	0.5715	NA	170756	rs1182116	329443	ieu-b-102	TRUE	reported	WYslv6
11	88889868	C	T	-0.0254	0.0045	1.08E-08	0.5801	NA	170756	rs1266568	329443	ieu-b-102	TRUE	reported	WYslv6
11	88889886	C	T	-0.0251	0.0044	8.72E-09	0.5744	NA	170756	rs3501632	329443	ieu-b-102	TRUE	reported	WYslv6
11	88889893	G	T	-0.0252	0.0045	1.44E-08	0.5802	NA	170756	rs3514812	329443	ieu-b-102	TRUE	reported	WYslv6
11	88890451	G	A	-0.0277	0.0043	1.08E-10	0.5225	NA	170756	rs6190341	329443	ieu-b-102	TRUE	reported	WYslv6
11	88890822	G	A	-0.0254	0.0043	4.74E-09	0.5754	NA	170756	rs1083022	329443	ieu-b-102	TRUE	reported	WYslv6
11	88891114	A	G	-0.0251	0.0043	7.58E-09	0.5737	NA	170756	rs1083022	329443	ieu-b-102	TRUE	reported	WYslv6
11	88891543	C	T	-0.0252	0.0043	6.47E-09	0.5762	NA	170756	rs4081455	329443	ieu-b-102	TRUE	reported	WYslv6
11	88891876	T	A	-0.0249	0.0044	1.05E-08	0.5735	NA	170756	rs4998767	329443	ieu-b-102	TRUE	reported	WYslv6
11	88893001	C	T	0.0274	0.0048	1.49E-08	0.278	NA	170756	rs6727907	329443	ieu-b-102	TRUE	reported	WYslv6
11	88894537	C	T	-0.0249	0.0043	9.55E-09	0.5778	NA	170756	rs1083023	329443	ieu-b-102	TRUE	reported	WYslv6
11	88894768	A	G	-0.0249	0.0043	9.76E-09	0.5778	NA	170756	rs1101851	329443	ieu-b-102	TRUE	reported	WYslv6
11	88894922	A	G	-0.0248	0.0043	1.18E-08	0.5779	NA	170756	rs2212751	329443	ieu-b-102	TRUE	reported	WYslv6
11	88895388	C	T	-0.0248	0.0043	1.21E-08	0.5776	NA	170756	rs9808089	329443	ieu-b-102	TRUE	reported	WYslv6
11	88895963	A	C	-0.0246	0.0044	1.76E-08	0.5764	NA	170756	rs7960986	329443	ieu-b-102	TRUE	reported	WYslv6
11	88896425	C	T	-0.0247	0.0043	1.36E-08	0.5776	NA	170756	rs1431826	329443	ieu-b-102	TRUE	reported	WYslv6
11	88896609	T	G	-0.0248	0.0043	1.06E-08	0.575	NA	170756	rs7127661	329443	ieu-b-102	TRUE	reported	WYslv6
11	88896664	A	G	-0.0248	0.0043	1.18E-08	0.5749	NA	170756	rs7124483	329443	ieu-b-102	TRUE	reported	WYslv6
11	88897444	A	G	-0.0256	0.0044	7.40E-09	0.545	NA	170756	rs1083023	329443	ieu-b-102	TRUE	reported	WYslv6
11	88899362	C	T	-0.0248	0.0043	1.20E-08	0.5774	NA	170756	rs7947287	329443	ieu-b-102	TRUE	reported	WYslv6
11	88899605	C	T	-0.0274	0.0043	1.90E-10	0.5227	NA	170756	rs7950415	329443	ieu-b-102	TRUE	reported	WYslv6
11	88899728	G	A	-0.0248	0.0043	1.19E-08	0.5774	NA	170756	rs1942481	329443	ieu-b-102	TRUE	reported	WYslv6
11	88901170	T	C	-0.026	0.0043	1.29E-09	0.514	NA	170756	rs1076519	329443	ieu-b-102	TRUE	reported	WYslv6
11	88901570	G	A	-0.0261	0.0043	1.15E-09	0.5139	NA	170756	rs1101851	329443	ieu-b-102	TRUE	reported	WYslv6
11	88902144	C	T	-0.0247	0.0043	1.23E-08	0.5751	NA	170756	rs1083023	329443	ieu-b-102	TRUE	reported	WYslv6
11	88902724	A	T	-0.0246	0.0043	1.54E-08	0.5781	NA	170756	rs949539	329443	ieu-b-102	TRUE	reported	WYslv6
11	88902830	T	C	-0.0247	0.0043	1.32E-08	0.5753	NA	170756	rs949537	329443	ieu-b-102	TRUE	reported	WYslv6
11	88903317	G	A	-0.0248	0.0043	1.12E-08	0.5772	NA	170756	rs1942498	329443	ieu-b-102	TRUE	reported	WYslv6
11	88904971	T	A	-0.0249	0.0043	1.09E-08	0.5773	NA	170756	rs1242172	329443	ieu-b-102	TRUE	reported	WYslv6
11	88905261	C	T	-0.0248	0.0043	1.13E-08	0.5772	NA	170756	rs1279527	329443	ieu-b-102	TRUE	reported	WYslv6
11	88907162	G	A	-0.0249	0.0043	1.09E-08	0.5768	NA	170756	rs1165075	329443	ieu-b-102	TRUE	reported	WYslv6
11	88911696	C	A	-0.026	0.0045	5.91E-09	0.3682	NA	170756	rs1042602	329443	ieu-b-102	TRUE	reported	WYslv6
11	88912190	T	C	0.0265	0.0047	1.76E-08	0.2998	NA	170756	rs1227071	329443	ieu-b-102	TRUE	reported	WYslv6
11	88913663	A	G	0.0275	0.0043	1.90E-10	0.4888	NA	170756	rs621313	329443	ieu-b-102	TRUE	reported	WYslv6
11	88914415	C	T	0.0263	0.0047	2.18E-08	0.2995	NA	170756	rs1101852	329443	ieu-b-102	TRUE	reported	WYslv6
11	88921524	C	T	0.026	0.0047	3.53E-08	0.2979	NA	170756	rs1779367	329443	ieu-b-102	TRUE	reported	WYslv6
11	88921557	C	T	0.0252	0.0044	6.84E-09	0.4365	NA	170756	rs1027212	329443	ieu-b-102	TRUE	reported	WYslv6
11	88921572	C	A	0.0263	0.0048	4.56E-08	0.2805	NA	170756	rs7296313	329443	ieu-b-102	TRUE	reported	WYslv6
11	88933250	C	T	0.0266	0.0043	6.18E-10	0.4815	NA	170756	rs574028	329443	ieu-b-102	TRUE	reported	WYslv6
11	88935088	C	T	-0.0252	0.0045	2.01E-08	0.3659	NA	170756	rs1228558	329443	ieu-b-102	TRUE	reported	WYslv6
11	88937602	T	C	0.0266	0.0043	5.66E-10	0.4825	NA	170756	rs1083023	329443	ieu-b-102	TRUE	reported	WYslv6
11	88938356	A	G	0.0259	0.0047	3.43E-08	0.2985	NA	170756	rs1083024	329443	ieu-b-102	TRUE	reported	WYslv6
11	88938933	G	C	0.0259	0.0047	3.44E-08	0.2983	NA	170756	rs1228661	329443	ieu-b-102	TRUE	reported	WYslv6
11	88943630	G	A	0.026	0.0047	3.26E-08	0.299	NA	170756	rs1101853	329443	ieu-b-102	TRUE	reported	WYslv6
11	88953819	C	G	0.0269	0.0043	5.64E-10	0.4834	NA	170756	rs1939252	329443	ieu-b-102	TRUE	reported	WYslv6
11	88953846	T	C	0.026	0.0047	3.21E-08	0.2984	NA	170756	rs1101853	329443	ieu-b-102	TRUE	reported	WYslv6
11	88969066	C	T	0.027	0.0043	3.62E-10	0.484	NA	170756	rs1412785	329443	ieu-b-102	TRUE	reported	WYslv6
11	88969774	T	C	0.0259	0.0047	3.33E-08	0.3013	NA	170756	rs1076519	329443	ieu-b-102	TRUE	reported	WYslv6
11	88970006	A	T	0.0269	0.0043	4.02E-10	0.4841	NA	170756	rs1101854	329443	ieu-b-102	TRUE	reported	WYslv6
11	88970138	T	G	0.0256	0.0047	3.67E-08	0.3105	NA	170756	rs7358418	329443	ieu-b-102	TRUE	reported	WYslv6
11	88971035	A	G	0.0256	0.0047	4.04E-08	0.3094	NA	170756	rs7939929	329443	ieu-b-102	TRUE	reported	WYslv6
11	88971134	G	A	0.0258	0.0047	4.12E-08	0.2997	NA	170756	rs1236504	329443	ieu-b-102	TRUE	reported	WYslv6
11	88971269	C	T	0.0258	0.0047	4.09E-08	0.2997	NA	170756	rs1101854	329443	ieu-b-102	TRUE	reported	WYslv6
11	88971373	A	G	0.0256	0.0047	3.77E-08	0.3096	NA	170756	rs7940368	329443	ieu-b-102	TRUE	reported	WYslv6
11	88971638	T	A	0.0256	0.0047	4.21E-08	0.3079	NA	170756	rs1076519	329443	ieu-b-102	TRUE	reported	WYslv6
11	88971684	C	T	0.0255	0.0047	4.65E-08	0.3088	NA	170756	rs1076520	329443	ieu-b-102	TRUE	reported	WYslv6
11	88971704	G	C	0.0256	0.0047	3.99E-08	0.3085	NA	170756	rs1076520	329443	ieu-b-102	TRUE	reported	WYslv6
11	88972118	C	A	0.0257	0.0047	4.45E-08	0.2996	NA	170756	rs7358330	329443	ieu-b-102	TRUE	reported	WYslv6
11	88973201	C	T	0.0256	0.0047	4.09E-08	0.3095	NA	170756	rs7933594	329443	ieu-b-102	TRUE	reported	WYslv6
11	88973361	G	A	0.0258	0.0047	4.24E-08	0.2996	NA	170756	rs1101854	329443	ieu-b-102	TRUE	reported	WYslv6
11	88976157	T	C	-0.0261	0.0045	8.08E-09	0.3757	NA	170756	rs1229516	329443	ieu-b-102	TRUE	reported	WYslv6
11	88978545	A	G	0.0255	0.0043	3.95E-09	0.4915	NA	170756	rs4601783	329443	ieu-b-102	TRUE	reported	WYslv6
11	1.13E+08	G	A	-0.0235	0.0043	4.80E-08	0.5495	NA	170756	rs1121460	329443	ieu-b-102	TRUE	reported	WYslv6
11	1.13E+08	G	A	-0.0237	0.0043	4.13E-08	0.5496	NA	170756	rs6277	329443	ieu-b-102	TRUE	reported	WYslv6
11	1.13E+08	C	T	-0.0244	0.0044	2.91E-08	0.6083	NA	170756	rs2734839	329443	ieu-b-102	TRUE	reported	WYslv6
11	1.13E+08	A	G	-0.0242	0.0044	3.86E-08	0.6081	NA	170756	rs2734838	329443	ieu-b-102	TRUE	reported	WYslv6
11	1.13E+08	C	T	-0.0245	0.0044	2.49E-08	0.6084	NA	170756	rs2734837	329443	ieu-b-102	TRUE	reported	WYslv6
11	1.13E+08	G	A	-0.0243	0.0044	3.29E-08	0.6081	NA	170756	rs1107162	329443	ieu-b-102	TRUE	reported	WYslv6
11	1.13E+08	G	A	-0.0241	0.0044	4.66E-08	0.6084	NA	170756	rs1800498	329443	ieu-b-102	TRUE	reported	WYslv6
11	1.13E+08	T	A	-0.0241	0.0044	4.42E-08	0.6085	NA	170756	rs2234690	329443	ieu-b-102	TRUE	reported	WYslv6
11	1.13E+08	G	C	-0.0241	0.0044	4.66E-08	0.6085	NA	170756	rs2587548	329443	ieu-b-102	TRUE	reported	WYslv6
11	1.13E+08	G	A	-0.0242	0.0044	3.78E-08	0.6085	NA	170756	rs2734833	329443	ieu-b-102	TRUE	reported	WYslv6
11	1.13E+08	T	C	-0.0241	0.0044	4.41E-08	0.6084	NA	170756	rs1160818	329443	ieu-b-102	TRUE	reported	WYslv6
11	1.13E+08	T	A	-0.024	0.0044	4.88E-08	0.6084	NA	170756	rs1280848	329443	ieu-b-102	TRUE	reported	WYslv6
11	1.13E+08	A	C	-0.0242	0.0044	3.82E-08	0.6082	NA	170756	rs1076563	329443	ieu-b-102	TRUE	reported	WYslv6
11	1.13E+08	A	G	-0.0242	0.0044	3.85E-08	0.6082	NA	170756	rs1116313	329443	ieu-b-102	TRUE	reported	WYslv6
11	1.13E+08	G	T	-0.0242	0.0044	3.45E-08	0.6034	NA	170756	rs1279890	329443	ieu-b-102	TRUE	reported	WYslv6
11	1.13E+08	G	A	-0.024	0.0044	4.50E-08	0.6034	NA	170756	rs7131627	329443	ieu-b-102	TRUE	reported	WYslv6
11	1.13E+08	C	T	-0.024	0.0044	4.69E-08	0.6031	NA	170756	rs7131440	329443	ieu-b-102	TRUE	reported	WYslv6
11	1.13E+08	A	C	-0.024	0.0044	4.94E-08	0.603	NA	170756	rs7131465	329443	ieu-b-102	TRUE	reported	WYslv6
11	1.13E+08	C	T	-0.024	0.0044	4.74E-08	0.603	NA	170756	rs1121460	329443	ieu-b-102	TRUE	reported	WYslv6
11	1.13E+08	G	C	-0.0275	0.0045	7.01E-10	0.3837	NA	170756	rs1760161	329443	ieu-b-102	TRUE	reported	WYslv6
11	1.13E+08	T	C	0.0255	0.0043	3.82E-09	0.5164	NA	170756	rs4245146	329443	ieu-b-102	TRUE	reported	WYslv6
11	1.13E+08	C	T	0.0256	0.0043	3.54E-09	0.5162	NA	170756	rs4245147	329443	ieu-b-102	TRUE	reported	WYslv6
11	1.13E+08	T	C	0.0255	0.0043	4.03E-09	0.5164	NA	170756	rs4936271	329443	ieu-b-102	TRUE	reported	WYslv6
11	1.13E+08	T	C	0.0255	0.0043	3.96E-09	0.5165	NA	170756	rs4936272	329443	ieu-b-102	TRUE	reported	WYslv6
11	1.13E+08	G	A	0.0256	0.0043	3.21E-09	0.5158	NA	170756	rs4274224	329443	ieu-b-102	TRUE	reported	WYslv6
11	1.13E+08	A	C	-0.0272	0.0044	9.51E-10	0.3782	NA	170756	rs1555168	329443	ieu-b-102	TRUE	reported	WYslv6
11	1.13E+08	C	T	-0.0271	0.0044	1.04E-09	0.3783	NA	170756	rs4337071	329443	ieu-b-102	TRUE	reported	WYslv6
11	1.13E+08	G	A	-0.0269	0.0044	1.42E-09	0.3777	NA	170756	rs4630328	329443	ieu-b-102	TRUE	reported	WYslv6
11	1.13E+08	G	A	-0.0273	0.0044	7.02E-10	0.3784	NA	170756	rs1160105	329443	ieu-b-102	TRUE	reported	WYslv6
11	1.13E+08	C	A	-0.0273	0.0044	7.90E-10	0.3784	NA	170756	rs6190278	329443	ieu-b-102	TRUE	reported	WYslv6
11	1.13E+08	T	C	-0.0273	0.0044	6.84E-10	0.3784	NA	170756	rs6190280	329443	ieu-b-102	TRUE	reported	WYslv6
11	1.13E+08	C	A	-0.0275	0.0044	5.59E-10	0.3785	NA	170756	rs7110440	329443	ieu-b-102	TRUE	reported	WYslv6
11	1.13E+08	C	G	-0.0275	0.0044	5.35E-10	0.3785	NA	170756	rs3527707	329443	ieu-b-102	TRUE	reported	WYslv6
11	1.13E+08	T	C	0.0276	0.0044	4.33E-10	0.6218	NA	170756	rs7121986	329443	ieu-b-102	TRUE	reported	WYslv6
11	1.13E+08	G	A	0.0277	0.0044	3.75E-10	0.6218	NA	170756	rs6589377	329443	ieu-b-102	TRUE	reported	WYslv6
11	1.13E+08	C	T	0.0271	0.0044	9.68E-10	0.619	NA	170756	rs7929453	329443	ieu-b-102	TRUE	reported	WYslv6
11	1.13E+08	G	T	0.0277	0.0044	3.67E-10	0.6207	NA	170756	rs1483771	329443	ieu-b-102	TRUE	reported	WYslv6
11	1.13E+08	T	C	-0.0277	0.0044	4.04E-10	0.378	NA	170756	rs1760203	329443	ieu-b-102	TRUE	reported	WYslv6
11	1.13E+08	C	T	0.0278	0.0044	3.69E-10	0.622	NA	170756	rs4938021	329443	ieu-b-102	TRUE	reported	WYslv6
11	1.13E+08	C	T	0.0278	0.0044	3.35E-10	0.622	NA	170756	rs1344309	329443	ieu-b-102	TRUE	reported	WYslv6
11	1.13E+08	G	C	0.0278	0.0044	3.57E-10	0.622	NA	170756	rs4936276	329443	ieu-b-102	TRUE	reported	WYslv6
11	1.13E+08	G	A	-0.0277	0.0044	5.00E-10	0.3678	NA	170756	rs6190281	329443	ieu-b-102	TRUE	reported	WYslv6

11	1.13E+08	C	T	0.0277	0.0045	4.85E-10	0.6322	NA	170756	rs7934649	329443	ieu-b-102	TRUE	reported	WYslv6
11	1.13E+08	C	A	-0.0275	0.0045	1.06E-09	0.3639	NA	170756	rs1260122	329443	ieu-b-102	TRUE	reported	WYslv6
11	1.13E+08	G	T	0.0275	0.0045	7.69E-10	0.6342	NA	170756	rs6589379	329443	ieu-b-102	TRUE	reported	WYslv6
11	1.13E+08	T	G	0.0277	0.0045	4.96E-10	0.6323	NA	170756	rs4938023	329443	ieu-b-102	TRUE	reported	WYslv6
11	1.13E+08	C	A	0.0276	0.0045	6.02E-10	0.6323	NA	170756	rs7111031	329443	ieu-b-102	TRUE	reported	WYslv6
11	1.13E+08	C	A	-0.0241	0.0043	2.79E-08	0.433	NA	170756	rs6589381	329443	ieu-b-102	TRUE	reported	WYslv6
11	1.13E+08	C	T	-0.024	0.0043	3.14E-08	0.4329	NA	170756	rs7949802	329443	ieu-b-102	TRUE	reported	WYslv6
11	1.13E+08	G	A	-0.0238	0.0043	4.31E-08	0.4327	NA	170756	rs4479021	329443	ieu-b-102	TRUE	reported	WYslv6
11	1.13E+08	G	A	-0.0237	0.0043	4.72E-08	0.433	NA	170756	rs7107293	329443	ieu-b-102	TRUE	reported	WYslv6
11	1.13E+08	A	C	-0.0238	0.0043	4.40E-08	0.433	NA	170756	rs4534613	329443	ieu-b-102	TRUE	reported	WYslv6
11	1.13E+08	T	G	-0.0237	0.0043	4.75E-08	0.4331	NA	170756	rs3573858	329443	ieu-b-102	TRUE	reported	WYslv6
11	1.13E+08	C	T	-0.0237	0.0043	4.78E-08	0.4331	NA	170756	rs3421635	329443	ieu-b-102	TRUE	reported	WYslv6
11	1.13E+08	A	G	-0.0238	0.0043	4.45E-08	0.4332	NA	170756	rs4245154	329443	ieu-b-102	TRUE	reported	WYslv6
11	1.13E+08	C	T	-0.0269	0.0045	2.48E-09	0.356	NA	170756	rs2514218	329443	ieu-b-102	TRUE	reported	WYslv6
11	1.13E+08	A	C	0.0256	0.0046	3.13E-08	0.6821	NA	170756	rs4309187	329443	ieu-b-102	TRUE	reported	WYslv6
12	52346983	G	A	0.0305	0.0054	1.26E-08	0.203	NA	170756	rs4761839	329443	ieu-b-102	TRUE	reported	WYslv6
12	52349071	C	G	0.0306	0.0054	1.11E-08	0.2037	NA	170756	rs1161014	329443	ieu-b-102	TRUE	reported	WYslv6
12	52349088	T	C	0.0309	0.0054	8.03E-09	0.2035	NA	170756	rs1161231	329443	ieu-b-102	TRUE	reported	WYslv6
12	52352301	G	A	0.0309	0.0054	7.96E-09	0.2034	NA	170756	rs6191404	329443	ieu-b-102	TRUE	reported	WYslv6
13	31713542	A	T	-0.0345	0.0059	6.47E-09	0.16	NA	170756	rs1050739	329443	ieu-b-102	TRUE	reported	WYslv6
13	31733057	G	A	-0.0347	0.0059	4.66E-09	0.1594	NA	170756	rs1218209	329443	ieu-b-102	TRUE	reported	WYslv6
13	31763090	C	A	-0.028	0.0051	4.80E-08	0.2312	NA	170756	rs1606182	329443	ieu-b-102	TRUE	reported	WYslv6
13	31763867	C	T	-0.0296	0.0053	2.15E-08	0.212	NA	170756	rs4943195	329443	ieu-b-102	TRUE	reported	WYslv6
13	31765220	C	T	-0.0297	0.0053	2.58E-08	0.2083	NA	170756	rs9571287	329443	ieu-b-102	TRUE	reported	WYslv6
13	31774731	C	T	-0.031	0.0053	6.52E-09	0.2066	NA	170756	rs1484710	329443	ieu-b-102	TRUE	reported	WYslv6
13	31776835	C	A	-0.0303	0.0052	4.76E-09	0.2262	NA	170756	rs7317283	329443	ieu-b-102	TRUE	reported	WYslv6
13	31778163	A	T	-0.0304	0.0052	4.12E-09	0.2266	NA	170756	rs9540653	329443	ieu-b-102	TRUE	reported	WYslv6
13	31780988	G	A	-0.0297	0.0052	9.91E-09	0.2263	NA	170756	rs9540737	329443	ieu-b-102	TRUE	reported	WYslv6
13	31783977	C	G	0.0305	0.0052	4.64E-09	0.7782	NA	170756	rs9529119	329443	ieu-b-102	TRUE	reported	WYslv6
13	31788335	T	G	0.0281	0.0051	4.78E-08	0.772	NA	170756	rs912609	329443	ieu-b-102	TRUE	reported	WYslv6
13	31789836	C	T	-0.0333	0.0054	5.65E-10	0.202	NA	170756	rs1137620	329443	ieu-b-102	TRUE	reported	WYslv6
13	31790053	C	T	-0.034	0.0054	2.23E-10	0.2031	NA	170756	rs9529218	329443	ieu-b-102	TRUE	reported	WYslv6
13	31793571	T	C	-0.0331	0.0054	6.88E-10	0.202	NA	170756	rs9541200	329443	ieu-b-102	TRUE	reported	WYslv6
13	31798075	T	C	-0.027	0.0049	4.64E-08	0.2555	NA	170756	rs9541291	329443	ieu-b-102	TRUE	reported	WYslv6
13	31799646	G	A	-0.0335	0.0053	3.72E-10	0.2028	NA	170756	rs9529314	329443	ieu-b-102	TRUE	reported	WYslv6
13	31800257	C	A	-0.0327	0.0054	1.01E-09	0.2017	NA	170756	rs1212952	329443	ieu-b-102	TRUE	reported	WYslv6
13	31802631	A	G	-0.0269	0.0049	4.72E-08	0.2559	NA	170756	rs7317285	329443	ieu-b-102	TRUE	reported	WYslv6
13	31804992	A	G	-0.0269	0.0049	4.82E-08	0.2561	NA	170756	rs4245373	329443	ieu-b-102	TRUE	reported	WYslv6
13	31814318	T	C	-0.0335	0.0054	4.51E-10	0.2008	NA	170756	rs1242888	329443	ieu-b-102	TRUE	reported	WYslv6
13	31814464	G	A	-0.0333	0.0054	5.44E-10	0.2004	NA	170756	rs1242785	329443	ieu-b-102	TRUE	reported	WYslv6
13	31814723	C	T	-0.0332	0.0054	6.67E-10	0.2004	NA	170756	rs3479108	329443	ieu-b-102	TRUE	reported	WYslv6
13	31816524	A	G	-0.0277	0.0049	2.06E-08	0.255	NA	170756	rs1511575	329443	ieu-b-102	TRUE	reported	WYslv6
13	31819951	C	A	-0.0328	0.0054	1.04E-09	0.2013	NA	170756	rs7317287	329443	ieu-b-102	TRUE	reported	WYslv6
13	31819996	A	G	-0.0326	0.0054	1.26E-09	0.2015	NA	170756	rs7317287	329443	ieu-b-102	TRUE	reported	WYslv6
13	31821093	T	C	-0.0328	0.0055	2.01E-09	0.2075	NA	170756	rs1424290	329443	ieu-b-102	TRUE	reported	WYslv6
13	31822463	A	T	-0.0328	0.0054	1.02E-09	0.2014	NA	170756	rs7986671	329443	ieu-b-102	TRUE	reported	WYslv6
13	31822667	C	G	0.0302	0.0052	5.89E-09	0.7784	NA	170756	rs7982806	329443	ieu-b-102	TRUE	reported	WYslv6
13	31825170	T	G	-0.0326	0.0054	1.34E-09	0.2016	NA	170756	rs4943272	329443	ieu-b-102	TRUE	reported	WYslv6
13	31833578	C	G	-0.0325	0.0054	1.60E-09	0.2016	NA	170756	rs9542729	329443	ieu-b-102	TRUE	reported	WYslv6
13	31836770	C	G	-0.0302	0.0053	1.24E-08	0.2116	NA	170756	rs7332089	329443	ieu-b-102	TRUE	reported	WYslv6
13	31842146	A	T	-0.0275	0.005	4.87E-08	0.2475	NA	170756	rs9530024	329443	ieu-b-102	TRUE	reported	WYslv6
13	31843598	A	G	0.0294	0.0051	8.82E-09	0.7688	NA	170756	rs1924817	329443	ieu-b-102	TRUE	reported	WYslv6
13	31843739	G	A	-0.0294	0.0052	2.05E-08	0.2211	NA	170756	rs7336582	329443	ieu-b-102	TRUE	reported	WYslv6
13	31843782	C	A	0.029	0.0051	1.79E-08	0.7621	NA	170756	rs3867696	329443	ieu-b-102	TRUE	reported	WYslv6
13	31844120	C	T	-0.0294	0.0052	1.58E-08	0.2216	NA	170756	rs7332406	329443	ieu-b-102	TRUE	reported	WYslv6
13	31847324	C	T	-0.033	0.0055	1.37E-09	0.195	NA	170756	rs9530139	329443	ieu-b-102	TRUE	reported	WYslv6
13	31848383	A	G	-0.0321	0.0054	3.08E-09	0.1979	NA	170756	rs9543234	329443	ieu-b-102	TRUE	reported	WYslv6
13	31848823	A	G	-0.0311	0.0054	7.55E-09	0.201	NA	170756	rs728930	329443	ieu-b-102	TRUE	reported	WYslv6
13	31849531	C	T	0.0278	0.0051	4.42E-08	0.7654	NA	170756	rs877103	329443	ieu-b-102	TRUE	reported	WYslv6
13	31852385	T	C	-0.0287	0.0052	2.63E-08	0.2248	NA	170756	rs9543390	329443	ieu-b-102	TRUE	reported	WYslv6
13	31854693	G	A	-0.0287	0.0052	2.46E-08	0.2245	NA	170756	rs7990820	329443	ieu-b-102	TRUE	reported	WYslv6
13	31856548	G	A	-0.0304	0.0052	5.82E-09	0.2188	NA	170756	rs9530264	329443	ieu-b-102	TRUE	reported	WYslv6
13	31857425	G	A	-0.031	0.0052	2.57E-09	0.2199	NA	170756	rs1028750	329443	ieu-b-102	TRUE	reported	WYslv6
13	31859039	T	A	-0.0297	0.0052	8.58E-09	0.2259	NA	170756	rs7993050	329443	ieu-b-102	TRUE	reported	WYslv6
13	31860258	A	G	-0.0288	0.0052	2.25E-08	0.2247	NA	170756	rs9315121	329443	ieu-b-102	TRUE	reported	WYslv6
13	31860769	G	C	-0.0292	0.0051	1.33E-08	0.2259	NA	170756	rs9543606	329443	ieu-b-102	TRUE	reported	WYslv6
13	31861059	G	A	-0.0286	0.0052	2.86E-08	0.2247	NA	170756	rs1326820	329443	ieu-b-102	TRUE	reported	WYslv6
13	31861970	A	G	-0.0287	0.0052	2.58E-08	0.2247	NA	170756	rs7984170	329443	ieu-b-102	TRUE	reported	WYslv6
13	31862334	C	A	0.0281	0.0051	2.99E-08	0.7659	NA	170756	rs7998271	329443	ieu-b-102	TRUE	reported	WYslv6
13	31862891	C	T	0.0281	0.0051	3.29E-08	0.766	NA	170756	rs1606180	329443	ieu-b-102	TRUE	reported	WYslv6
13	31863241	T	C	0.0281	0.0051	2.92E-08	0.7659	NA	170756	rs1849167	329443	ieu-b-102	TRUE	reported	WYslv6
13	31867030	G	C	0.0282	0.0051	3.20E-08	0.7663	NA	170756	rs2203090	329443	ieu-b-102	TRUE	reported	WYslv6
13	31870394	G	A	-0.0319	0.0054	3.10E-09	0.1998	NA	170756	rs1432586	329443	ieu-b-102	TRUE	reported	WYslv6
13	31872514	T	G	0.028	0.0051	3.35E-08	0.7661	NA	170756	rs9565173	329443	ieu-b-102	TRUE	reported	WYslv6
13	31872819	C	T	0.0281	0.0051	2.87E-08	0.7659	NA	170756	rs9543985	329443	ieu-b-102	TRUE	reported	WYslv6
13	31873816	C	T	0.028	0.0051	3.53E-08	0.7661	NA	170756	rs7994976	329443	ieu-b-102	TRUE	reported	WYslv6
13	31874223	C	G	-0.0288	0.0052	2.49E-08	0.2241	NA	170756	rs9544036	329443	ieu-b-102	TRUE	reported	WYslv6
13	31874916	T	A	0.0277	0.0051	4.55E-08	0.766	NA	170756	rs4941810	329443	ieu-b-102	TRUE	reported	WYslv6
13	31874993	G	A	0.0279	0.0051	3.84E-08	0.7658	NA	170756	rs4941811	329443	ieu-b-102	TRUE	reported	WYslv6
13	31884319	G	A	0.028	0.0051	3.57E-08	0.7658	NA	170756	rs2313819	329443	ieu-b-102	TRUE	reported	WYslv6
13	31885154	G	A	0.0278	0.0051	4.17E-08	0.766	NA	170756	rs1443344	329443	ieu-b-102	TRUE	reported	WYslv6
13	31885907	A	G	-0.0286	0.0052	2.82E-08	0.2245	NA	170756	rs9544399	329443	ieu-b-102	TRUE	reported	WYslv6
13	31886850	A	G	-0.0286	0.0052	2.74E-08	0.2245	NA	170756	rs1087065	329443	ieu-b-102	TRUE	reported	WYslv6
13	31888767	G	A	-0.0286	0.0052	2.79E-08	0.2245	NA	170756	rs9544429	329443	ieu-b-102	TRUE	reported	WYslv6
13	31889040	G	A	-0.0317	0.0054	3.78E-09	0.2	NA	170756	rs9544436	329443	ieu-b-102	TRUE	reported	WYslv6
13	31893030	T	C	-0.0289	0.0052	2.07E-08	0.2245	NA	170756	rs9315123	329443	ieu-b-102	TRUE	reported	WYslv6
13	31893501	G	C	-0.0288	0.0052	2.35E-08	0.2245	NA	170756	rs9544560	329443	ieu-b-102	TRUE	reported	WYslv6
13	31894515	C	T	0.0281	0.0051	3.16E-08	0.7662	NA	170756	rs4943306	329443	ieu-b-102	TRUE	reported	WYslv6
13	31898381	A	G	-0.0291	0.0052	1.75E-08	0.224	NA	170756	rs3761871	329443	ieu-b-102	TRUE	reported	WYslv6
13	31899602	C	G	0.029	0.0051	1.15E-08	0.7646	NA	170756	rs7991258	329443	ieu-b-102	TRUE	reported	WYslv6
13	31900245	G	A	0.0314	0.0053	3.84E-09	0.7911	NA	170756	rs7334101	329443	ieu-b-102	TRUE	reported	WYslv6
13	31901210	C	A	0.0313	0.0053	3.86E-09	0.7914	NA	170756	rs1028748	329443	ieu-b-102	TRUE	reported	WYslv6
13	31901241	C	A	0.0312	0.0053	3.99E-09	0.791	NA	170756	rs1028747	329443	ieu-b-102	TRUE	reported	WYslv6
13	31901858	A	G	0.032	0.0053	1.58E-09	0.7901	NA	170756	rs1028746	329443	ieu-b-102	TRUE	reported	WYslv6
13	31902535	T	G	0.0322	0.0053	1.02E-09	0.7891	NA	170756	rs7339288	329443	ieu-b-102	TRUE	reported	WYslv6
13	31903834	G	T	0.0283	0.0049	6.71E-09	0.7382	NA	170756	rs1060709	329443	ieu-b-102	TRUE	reported	WYslv6
13	31905606	A	G	0.0302	0.0051	2.58E-09	0.7656	NA	170756	rs912604	329443	ieu-b-102	TRUE	reported	WYslv6
13	31905641	G	A	0.0303	0.0051	2.71E-09	0.7653	NA	170756	rs912603	329443	ieu-b-102	TRUE	reported	WYslv6
13	31905872	A	G	0.0305	0.0051	1.55E-09	0.7639	NA	170756	rs2056445	329443	ieu-b-102	TRUE	reported	WYslv6
13	31905887	G	T	0.0282	0.0049	8.31E-09	0.7382	NA	170756	rs2056444	329443	ieu-b-102	TRUE	reported	WYslv6
13	31905942	T	C	0.0305	0.0051	1.59E-09	0.7639	NA	170756	rs2056443	329443	ieu-b-102	TRUE	reported	WYslv6
13	31906141	A	C	0.0305	0.0051	1.70E-09	0.7639	NA	170756	rs13080	329443	ieu-b-102	TRUE	reported	WYslv6
13	31906291	T	C	0.0305	0.0051	1.61E-09	0.7639	NA	170756	rs1438355	329443	ieu-b-102	TRUE	reported	WYslv6
13	31907741	C	T	0.0306	0.0051	1.41E-09	0.7638	NA	170756	rs1409379	329443	ieu-b-102	TRUE	reported	WYslv6
13	31908295	T	C	0.0319	0.0053	2.03E-09	0.7907	NA	170756	rs4943314	329443	ieu-b-102	TRUE	reported	WYslv6
13	31909786	G	A	0.0304	0.0051	1.71E-09	0.7632	NA	170756	rs1397891	329443	ieu-b-102	TRUE	reported	WYslv6
13	31909791	G	A	0.0307	0.0051	1.43E-09	0.764	NA	170756	rs967516	329443	ieu-b-102	TRUE	reported	WYslv6

13	31911822	C	T	0.0278	0.005	3.07E-08	0.7576	NA	170756	rs9545125	329443	ieu-b-102	TRUE	reported	WYslv6
13	31912323	A	G	0.0282	0.005	1.68E-08	0.7562	NA	170756	rs7999260	329443	ieu-b-102	TRUE	reported	WYslv6
13	31912705	C	G	0.028	0.005	2.29E-08	0.7559	NA	170756	rs4943321	329443	ieu-b-102	TRUE	reported	WYslv6
13	31912892	A	G	0.0278	0.005	3.25E-08	0.7574	NA	170756	rs4943322	329443	ieu-b-102	TRUE	reported	WYslv6
13	31913070	A	G	0.0298	0.0052	1.18E-08	0.7797	NA	170756	rs4943323	329443	ieu-b-102	TRUE	reported	WYslv6
13	31913122	T	A	0.0287	0.005	1.31E-08	0.7512	NA	170756	rs4943324	329443	ieu-b-102	TRUE	reported	WYslv6
13	31914030	C	T	0.0282	0.005	2.08E-08	0.7514	NA	170756	rs9530946	329443	ieu-b-102	TRUE	reported	WYslv6
13	31914068	A	G	0.0277	0.005	3.67E-08	0.7569	NA	170756	rs7989071	329443	ieu-b-102	TRUE	reported	WYslv6
13	31914145	A	G	0.0277	0.005	3.88E-08	0.7535	NA	170756	rs7989252	329443	ieu-b-102	TRUE	reported	WYslv6
13	31914158	G	A	0.0268	0.0049	4.12E-08	0.728	NA	170756	rs2313820	329443	ieu-b-102	TRUE	reported	WYslv6
13	31915078	G	A	0.0276	0.005	3.50E-08	0.7552	NA	170756	rs2102562	329443	ieu-b-102	TRUE	reported	WYslv6
13	31921717	A	G	0.0278	0.005	3.30E-08	0.7579	NA	170756	rs7983430	329443	ieu-b-102	TRUE	reported	WYslv6
13	31924013	A	T	0.0276	0.005	3.94E-08	0.7572	NA	170756	rs4943341	329443	ieu-b-102	TRUE	reported	WYslv6
13	31924054	C	A	0.0278	0.005	2.90E-08	0.7559	NA	170756	rs4941819	329443	ieu-b-102	TRUE	reported	WYslv6
13	31924231	C	A	0.0276	0.005	3.51E-08	0.7556	NA	170756	rs9601445	329443	ieu-b-102	TRUE	reported	WYslv6
13	31924412	G	A	0.0278	0.005	2.75E-08	0.7557	NA	170756	rs7329543	329443	ieu-b-102	TRUE	reported	WYslv6
13	31924973	T	A	0.029	0.005	8.45E-09	0.7517	NA	170756	rs8797190	329443	ieu-b-102	TRUE	reported	WYslv6
13	31928963	T	A	0.0274	0.005	4.53E-08	0.7564	NA	170756	rs4943351	329443	ieu-b-102	TRUE	reported	WYslv6
13	31933957	G	A	0.0277	0.005	3.18E-08	0.7545	NA	170756	rs1590571	329443	ieu-b-102	TRUE	reported	WYslv6
13	31934115	C	T	0.0276	0.005	3.37E-08	0.7545	NA	170756	rs1590570	329443	ieu-b-102	TRUE	reported	WYslv6
13	31935371	G	A	0.0275	0.005	4.61E-08	0.7568	NA	170756	rs1326309	329443	ieu-b-102	TRUE	reported	WYslv6
13	31935616	G	C	0.0275	0.005	4.62E-08	0.7568	NA	170756	rs1326308	329443	ieu-b-102	TRUE	reported	WYslv6
13	31938186	A	C	0.0279	0.005	2.44E-08	0.7558	NA	170756	rs1326306	329443	ieu-b-102	TRUE	reported	WYslv6
13	31938487	G	C	0.0286	0.0052	4.50E-08	0.7819	NA	170756	rs9593648	329443	ieu-b-102	TRUE	reported	WYslv6
13	31938663	A	G	0.0281	0.005	2.16E-08	0.7536	NA	170756	rs7328774	329443	ieu-b-102	TRUE	reported	WYslv6
13	31941945	A	G	0.0308	0.0056	3.19E-08	0.803	NA	170756	rs1409380	329443	ieu-b-102	TRUE	reported	WYslv6
13	53858586	C	T	0.0255	0.0046	2.63E-08	0.3261	NA	170756	rs9596774	329443	ieu-b-102	TRUE	reported	WYslv6
13	53859017	C	T	0.0255	0.0046	2.66E-08	0.3261	NA	170756	rs9596775	329443	ieu-b-102	TRUE	reported	WYslv6
13	53860655	C	T	0.0255	0.0046	2.62E-08	0.3259	NA	170756	rs9536381	329443	ieu-b-102	TRUE	reported	WYslv6
13	53862411	T	C	0.0254	0.0046	3.16E-08	0.3258	NA	170756	rs9527059	329443	ieu-b-102	TRUE	reported	WYslv6
13	53863604	C	T	0.0253	0.0046	3.60E-08	0.3255	NA	170756	rs9805634	329443	ieu-b-102	TRUE	reported	WYslv6
13	53865563	C	T	0.0255	0.0046	3.88E-08	0.3115	NA	170756	rs9536383	329443	ieu-b-102	TRUE	reported	WYslv6
13	53879428	G	A	0.0252	0.0046	4.95E-08	0.3145	NA	170756	rs9536386	329443	ieu-b-102	TRUE	reported	WYslv6
13	80921519	C	T	-0.0264	0.0048	3.56E-08	0.2992	NA	170756	rs508502	329443	ieu-b-102	TRUE	reported	WYslv6
14	42036322	A	T	-0.0288	0.0043	2.96E-11	0.5096	NA	170756	rs5465504	329443	ieu-b-102	TRUE	reported	WYslv6
14	42045106	C	T	-0.0268	0.0044	8.24E-10	0.5893	NA	170756	rs8011321	329443	ieu-b-102	TRUE	reported	WYslv6
14	42051747	A	G	-0.027	0.0043	5.01E-10	0.5212	NA	170756	rs1566575	329443	ieu-b-102	TRUE	reported	WYslv6
14	42051771	T	C	-0.029	0.0043	2.20E-11	0.5042	NA	170756	rs1115724	329443	ieu-b-102	TRUE	reported	WYslv6
14	42052115	T	C	-0.0292	0.0043	1.42E-11	0.5105	NA	170756	rs4356390	329443	ieu-b-102	TRUE	reported	WYslv6
14	42066464	A	G	-0.0291	0.0043	1.25E-11	0.5117	NA	170756	rs7148091	329443	ieu-b-102	TRUE	reported	WYslv6
14	42068689	T	C	-0.0292	0.0043	1.11E-11	0.5117	NA	170756	rs1243144	329443	ieu-b-102	TRUE	reported	WYslv6
14	42069176	C	T	-0.0293	0.0043	9.49E-12	0.5101	NA	170756	rs6199028	329443	ieu-b-102	TRUE	reported	WYslv6
14	42069215	A	C	-0.027	0.0043	4.53E-10	0.5239	NA	170756	rs1258904	329443	ieu-b-102	TRUE	reported	WYslv6
14	42072408	C	G	-0.0293	0.0043	8.84E-12	0.5102	NA	170756	rs1778128	329443	ieu-b-102	TRUE	reported	WYslv6
14	42072610	T	C	-0.0293	0.0043	9.03E-12	0.5115	NA	170756	rs1609735	329443	ieu-b-102	TRUE	reported	WYslv6
14	42074524	A	G	-0.0292	0.0043	1.16E-11	0.5118	NA	170756	rs1950834	329443	ieu-b-102	TRUE	reported	WYslv6
14	42074726	G	A	-0.0295	0.0043	6.91E-12	0.5102	NA	170756	rs6199028	329443	ieu-b-102	TRUE	reported	WYslv6
14	42075952	G	T	-0.0291	0.0043	1.37E-11	0.5121	NA	170756	rs1111179	329443	ieu-b-102	TRUE	reported	WYslv6
14	42076337	A	G	-0.0292	0.0043	1.14E-11	0.513	NA	170756	rs1159354	329443	ieu-b-102	TRUE	reported	WYslv6
14	42076434	C	T	-0.029	0.0043	1.52E-11	0.5135	NA	170756	rs6199028	329443	ieu-b-102	TRUE	reported	WYslv6
14	42076640	G	T	-0.0292	0.0043	1.15E-11	0.5128	NA	170756	rs1950835	329443	ieu-b-102	TRUE	reported	WYslv6
14	42076745	C	T	-0.0288	0.0043	2.59E-11	0.5319	NA	170756	rs1156483	329443	ieu-b-102	TRUE	reported	WYslv6
14	42077288	A	G	-0.0271	0.0043	2.91E-10	0.5268	NA	170756	rs1137567	329443	ieu-b-102	TRUE	reported	WYslv6
14	42081882	T	C	-0.0293	0.0043	8.30E-12	0.5171	NA	170756	rs1956450	329443	ieu-b-102	TRUE	reported	WYslv6
14	42084030	A	T	-0.0275	0.0043	1.84E-10	0.5325	NA	170756	rs1950838	329443	ieu-b-102	TRUE	reported	WYslv6
14	42086798	T	G	-0.0293	0.0043	8.70E-12	0.5171	NA	170756	rs1048352	329443	ieu-b-102	TRUE	reported	WYslv6
14	42088506	T	A	-0.0275	0.0043	1.89E-10	0.5333	NA	170756	rs8007172	329443	ieu-b-102	TRUE	reported	WYslv6
14	42089908	T	C	-0.0276	0.0043	1.52E-10	0.5327	NA	170756	rs8013655	329443	ieu-b-102	TRUE	reported	WYslv6
14	42090036	A	C	-0.0274	0.0043	2.11E-10	0.5334	NA	170756	rs6572099	329443	ieu-b-102	TRUE	reported	WYslv6
14	42092565	C	T	-0.0275	0.0043	1.69E-10	0.5299	NA	170756	rs7268456	329443	ieu-b-102	TRUE	reported	WYslv6
14	42093431	A	G	-0.027	0.0043	3.81E-10	0.5314	NA	170756	rs8003822	329443	ieu-b-102	TRUE	reported	WYslv6
14	42095232	A	T	-0.0294	0.0043	7.22E-12	0.5171	NA	170756	rs8015100	329443	ieu-b-102	TRUE	reported	WYslv6
14	42096100	C	T	-0.0276	0.0043	1.56E-10	0.5299	NA	170756	rs8020581	329443	ieu-b-102	TRUE	reported	WYslv6
14	42097937	A	G	-0.0297	0.0043	4.74E-12	0.5173	NA	170756	rs1950829	329443	ieu-b-102	TRUE	reported	WYslv6
14	42100553	A	G	-0.0277	0.0044	2.55E-10	0.5957	NA	170756	rs1983711	329443	ieu-b-102	TRUE	reported	WYslv6
14	42102437	T	G	-0.0272	0.0044	5.42E-10	0.6038	NA	170756	rs5628073	329443	ieu-b-102	TRUE	reported	WYslv6
14	42102855	C	G	-0.0281	0.0043	7.31E-11	0.5331	NA	170756	rs1950831	329443	ieu-b-102	TRUE	reported	WYslv6
14	42114227	G	A	-0.0269	0.0048	1.90E-08	0.7175	NA	170756	rs712405	329443	ieu-b-102	TRUE	reported	WYslv6
14	42114318	G	A	-0.0265	0.0048	2.93E-08	0.7171	NA	170756	rs1320555	329443	ieu-b-102	TRUE	reported	WYslv6
14	42117374	C	A	-0.0269	0.0047	1.36E-08	0.7099	NA	170756	rs1272225	329443	ieu-b-102	TRUE	reported	WYslv6
14	42128837	C	T	0.0269	0.0048	1.75E-08	0.2822	NA	170756	rs1180649	329443	ieu-b-102	TRUE	reported	WYslv6
14	42141201	G	A	0.0268	0.0048	2.04E-08	0.2819	NA	170756	rs1298666	329443	ieu-b-102	TRUE	reported	WYslv6
14	42143061	T	C	0.028	0.0044	1.25E-10	0.4617	NA	170756	rs1014905	329443	ieu-b-102	TRUE	reported	WYslv6
14	42143231	A	G	0.0282	0.0043	8.21E-11	0.4709	NA	170756	rs6750544	329443	ieu-b-102	TRUE	reported	WYslv6
14	42145815	G	C	0.024	0.0044	3.77E-08	0.4146	NA	170756	rs8009571	329443	ieu-b-102	TRUE	reported	WYslv6
14	42146572	A	G	0.0294	0.0043	8.12E-12	0.4829	NA	170756	rs2121708	329443	ieu-b-102	TRUE	reported	WYslv6
14	42147544	C	T	0.0257	0.0044	3.62E-09	0.4198	NA	170756	rs1015035	329443	ieu-b-102	TRUE	reported	WYslv6
14	42148209	G	A	0.0258	0.0044	3.24E-09	0.4196	NA	170756	rs1597053	329443	ieu-b-102	TRUE	reported	WYslv6
14	42150128	C	T	0.0255	0.0044	4.73E-09	0.4209	NA	170756	rs8006276	329443	ieu-b-102	TRUE	reported	WYslv6
14	42150494	T	C	0.0254	0.0044	5.53E-09	0.4209	NA	170756	rs1267273	329443	ieu-b-102	TRUE	reported	WYslv6
14	42150804	G	A	0.0238	0.0044	4.87E-08	0.4076	NA	170756	rs8007820	329443	ieu-b-102	TRUE	reported	WYslv6
14	42152124	G	A	0.0259	0.0043	2.25E-09	0.4278	NA	170756	rs1162829	329443	ieu-b-102	TRUE	reported	WYslv6
14	42154542	C	T	0.029	0.0043	1.48E-11	0.4833	NA	170756	rs8009740	329443	ieu-b-102	TRUE	reported	WYslv6
14	42154546	T	C	0.029	0.0043	1.50E-11	0.4833	NA	170756	rs8010939	329443	ieu-b-102	TRUE	reported	WYslv6
14	42156235	A	T	0.0289	0.0043	2.52E-11	0.4829	NA	170756	rs1194129	329443	ieu-b-102	TRUE	reported	WYslv6
14	42157545	G	C	0.0238	0.0044	4.85E-08	0.4068	NA	170756	rs6572106	329443	ieu-b-102	TRUE	reported	WYslv6
14	42161283	A	C	0.0256	0.0043	3.34E-09	0.4287	NA	170756	rs1013810	329443	ieu-b-102	TRUE	reported	WYslv6
14	42163202	A	T	0.0288	0.0043	2.34E-11	0.4843	NA	170756	rs1597052	329443	ieu-b-102	TRUE	reported	WYslv6
14	42164112	G	C	0.0258	0.0043	2.52E-09	0.428	NA	170756	rs2415688	329443	ieu-b-102	TRUE	reported	WYslv6
14	42165449	T	G	0.0288	0.0043	2.08E-11	0.484	NA	170756	rs1014052	329443	ieu-b-102	TRUE	reported	WYslv6
14	42166111	A	G	0.0285	0.0043	3.10E-11	0.4842	NA	170756	rs1013118	329443	ieu-b-102	TRUE	reported	WYslv6
14	42168893	G	A	0.0246	0.0043	1.33E-08	0.4296	NA	170756	rs1115724	329443	ieu-b-102	TRUE	reported	WYslv6
14	42179732	T	C	0.0264	0.0044	1.25E-09	0.4308	NA	170756	rs4904738	329443	ieu-b-102	TRUE	reported	WYslv6
14	42181334	C	G	0.0275	0.0043	1.83E-10	0.4648	NA	170756	rs1597044	329443	ieu-b-102	TRUE	reported	WYslv6
14	42182103	A	G	0.0238	0.0043	3.62E-08	0.4651	NA	170756	rs1013271	329443	ieu-b-102	TRUE	reported	WYslv6
14	42183025	G	A	0.0242	0.0043	2.01E-08	0.4652	NA	170756	rs1184655	329443	ieu-b-102	TRUE	reported	WYslv6
14	42233384	G	T	0.0243	0.0043	1.69E-08	0.5251	NA	170756	rs7146404	329443	ieu-b-102	TRUE	reported	WYslv6
14	42234257	A	G	0.0242	0.0043	1.82E-08	0.5245	NA	170756	rs2038362	329443	ieu-b-102	TRUE	reported	WYslv6
14	42237790	G	T	0.0243	0.0043	1.66E-08	0.5246	NA	170756	rs4904787	329443	ieu-b-102	TRUE	reported	WYslv6
14	42243211	T	G	0.0243	0.0043	1.53E-08	0.525	NA	170756	rs7154241	329443	ieu-b-102	TRUE	reported	WYslv6
14	42243597	A	G	0.0242	0.0043	1.89E-08	0.5247	NA	170756	rs1374199	329443	ieu-b-102	TRUE	reported	WYslv6
14	42243781	A	G	0.0243	0.0043	1.58E-08	0.5248	NA	170756	rs1992851	329443	ieu-b-102	TRUE	reported	WYslv6
14	42249328	G	A	0.024	0.0043	2.37E-08	0.5247	NA	170756	rs7146838	329443	ieu-b-102	TRUE	reported	WYslv6
14	75120628	C	T	0.0252	0.0043	4.86E-09	0.4999	NA	170756	rs1162118	329443	ieu-b-102	TRUE	reported	WYslv6
14	75123374	C	A	0.0253	0.0043	4.05E-09	0.5193	NA	170756	rs1243435	329443	ieu-b-102	TRUE	reported	WYslv6
14	75125540	T	C	0.0258	0.0043	1.87E-09	0.5196	NA	170756	rs7152906	329443	ieu-b-102	TRUE	reported	WYslv6
14	75128425	G	A	0.0245	0.0043	1.15E-08	0.5005	NA	170756	rs9441	329443	ieu-b-102	TRUE	reported	WYslv6

14	75130235	T	G	0.0257	0.0043	2.21E-09	0.5194	NA	170756	rs1045430	329443	ieu-b-102	TRUE	reported	WYslv6
14	75137664	T	C	0.0254	0.0043	3.26E-09	0.5196	NA	170756	rs2302832	329443	ieu-b-102	TRUE	reported	WYslv6
14	75145528	T	A	0.0243	0.0043	1.59E-08	0.5009	NA	170756	rs7145575	329443	ieu-b-102	TRUE	reported	WYslv6
14	75157713	G	T	0.0253	0.0043	3.83E-09	0.5198	NA	170756	rs3559139	329443	ieu-b-102	TRUE	reported	WYslv6
14	75162021	G	A	0.0242	0.0043	1.84E-08	0.5018	NA	170756	rs1162936	329443	ieu-b-102	TRUE	reported	WYslv6
14	75164141	C	T	0.0244	0.0043	1.32E-08	0.5002	NA	170756	rs1258745	329443	ieu-b-102	TRUE	reported	WYslv6
14	75167706	A	G	0.0252	0.0043	4.19E-09	0.5186	NA	170756	rs1778268	329443	ieu-b-102	TRUE	reported	WYslv6
14	75172999	G	A	0.024	0.0043	2.18E-08	0.5027	NA	170756	rs1288995	329443	ieu-b-102	TRUE	reported	WYslv6
14	75178693	C	T	0.0242	0.0043	1.54E-08	0.5007	NA	170756	rs1243464	329443	ieu-b-102	TRUE	reported	WYslv6
14	75186010	A	G	0.0259	0.0044	2.79E-09	0.4595	NA	170756	rs1395994	329443	ieu-b-102	TRUE	reported	WYslv6
14	75190527	G	T	0.0249	0.0043	9.81E-09	0.4619	NA	170756	rs7141254	329443	ieu-b-102	TRUE	reported	WYslv6
14	75207263	G	A	0.0251	0.0043	5.69E-09	0.4629	NA	170756	rs3564144	329443	ieu-b-102	TRUE	reported	WYslv6
14	75207692	G	A	0.0243	0.0043	1.95E-08	0.4431	NA	170756	rs1243558	329443	ieu-b-102	TRUE	reported	WYslv6
14	75212114	G	C	0.0252	0.0043	5.21E-09	0.5444	NA	170756	rs1014349	329443	ieu-b-102	TRUE	reported	WYslv6
14	75220095	G	A	0.0235	0.0043	4.81E-08	0.4463	NA	170756	rs1243141	329443	ieu-b-102	TRUE	reported	WYslv6
14	75234830	T	G	0.0243	0.0043	1.60E-08	0.5395	NA	170756	rs1288966	329443	ieu-b-102	TRUE	reported	WYslv6
14	75235397	A	G	0.024	0.0043	2.31E-08	0.4647	NA	170756	rs1115909	329443	ieu-b-102	TRUE	reported	WYslv6
14	75239863	C	T	0.0239	0.0043	2.68E-08	0.4645	NA	170756	rs1289686	329443	ieu-b-102	TRUE	reported	WYslv6
14	75240090	A	G	0.0246	0.0043	1.17E-08	0.5389	NA	170756	rs1289113	329443	ieu-b-102	TRUE	reported	WYslv6
14	75245002	G	A	0.0245	0.0043	1.29E-08	0.5387	NA	170756	rs2359237	329443	ieu-b-102	TRUE	reported	WYslv6
14	75245756	G	C	0.0247	0.0043	1.13E-08	0.5398	NA	170756	rs6574194	329443	ieu-b-102	TRUE	reported	WYslv6
14	75254073	G	A	0.0254	0.0043	5.07E-09	0.5375	NA	170756	rs1258887	329443	ieu-b-102	TRUE	reported	WYslv6
14	75254167	G	A	0.0239	0.0043	2.78E-08	0.4649	NA	170756	rs3495681	329443	ieu-b-102	TRUE	reported	WYslv6
14	75267396	C	T	0.0237	0.0043	3.69E-08	0.4646	NA	170756	rs888413	329443	ieu-b-102	TRUE	reported	WYslv6
14	75268544	G	A	0.0237	0.0043	3.62E-08	0.4646	NA	170756	rs7153700	329443	ieu-b-102	TRUE	reported	WYslv6
14	75274292	T	C	0.0245	0.0043	1.32E-08	0.5393	NA	170756	rs1013901	329443	ieu-b-102	TRUE	reported	WYslv6
14	75276079	C	G	0.0244	0.0043	1.44E-08	0.539	NA	170756	rs957345	329443	ieu-b-102	TRUE	reported	WYslv6
14	75278211	G	A	0.0236	0.0043	3.82E-08	0.4639	NA	170756	rs1258841	329443	ieu-b-102	TRUE	reported	WYslv6
14	75281576	T	A	0.0243	0.0043	1.54E-08	0.5392	NA	170756	rs2884668	329443	ieu-b-102	TRUE	reported	WYslv6
14	75282116	T	G	0.0245	0.0043	1.26E-08	0.5395	NA	170756	rs1243202	329443	ieu-b-102	TRUE	reported	WYslv6
14	75287554	T	C	0.0242	0.0043	1.85E-08	0.5392	NA	170756	rs8016389	329443	ieu-b-102	TRUE	reported	WYslv6
14	75290018	C	G	0.0235	0.0043	4.78E-08	0.4644	NA	170756	rs8021280	329443	ieu-b-102	TRUE	reported	WYslv6
14	75291531	T	C	0.0241	0.0043	2.16E-08	0.539	NA	170756	rs1014402	329443	ieu-b-102	TRUE	reported	WYslv6
14	75293386	G	A	0.024	0.0043	2.29E-08	0.5391	NA	170756	rs1013836	329443	ieu-b-102	TRUE	reported	WYslv6
14	75296831	A	G	0.0243	0.0043	1.72E-08	0.5394	NA	170756	rs1014829	329443	ieu-b-102	TRUE	reported	WYslv6
14	75297727	T	C	0.0242	0.0043	1.86E-08	0.5392	NA	170756	rs7624577	329443	ieu-b-102	TRUE	reported	WYslv6
14	75298052	C	T	0.0236	0.0043	4.08E-08	0.4651	NA	170756	rs1115910	329443	ieu-b-102	TRUE	reported	WYslv6
14	75298637	A	G	0.0242	0.0043	1.90E-08	0.5392	NA	170756	rs7144654	329443	ieu-b-102	TRUE	reported	WYslv6
14	75299101	G	A	0.0235	0.0043	4.45E-08	0.4647	NA	170756	rs7150254	329443	ieu-b-102	TRUE	reported	WYslv6
14	75316614	T	C	-0.0235	0.0043	4.55E-08	0.5366	NA	170756	rs4899536	329443	ieu-b-102	TRUE	reported	WYslv6
14	1.04E+08	A	G	-0.0287	0.0052	2.97E-08	0.2218	NA	170756	rs3759575	329443	ieu-b-102	TRUE	reported	WYslv6
14	1.04E+08	C	T	-0.0288	0.0052	2.84E-08	0.2218	NA	170756	rs3759577	329443	ieu-b-102	TRUE	reported	WYslv6
14	1.04E+08	G	T	-0.0276	0.0048	8.07E-09	0.2827	NA	170756	rs1243695	329443	ieu-b-102	TRUE	reported	WYslv6
14	1.04E+08	C	T	-0.0279	0.0048	5.15E-09	0.2835	NA	170756	rs1243675	329443	ieu-b-102	TRUE	reported	WYslv6
14	1.04E+08	A	G	-0.0279	0.0048	5.20E-09	0.2832	NA	170756	rs1401047	329443	ieu-b-102	TRUE	reported	WYslv6
14	1.04E+08	G	A	-0.0279	0.0048	5.32E-09	0.2831	NA	170756	rs6575982	329443	ieu-b-102	TRUE	reported	WYslv6
14	1.04E+08	G	A	-0.0279	0.0048	5.31E-09	0.2832	NA	170756	rs7149767	329443	ieu-b-102	TRUE	reported	WYslv6
14	1.04E+08	G	A	-0.0278	0.0048	5.81E-09	0.2839	NA	170756	rs7145753	329443	ieu-b-102	TRUE	reported	WYslv6
14	1.04E+08	G	A	-0.0278	0.0048	5.82E-09	0.2839	NA	170756	rs7144010	329443	ieu-b-102	TRUE	reported	WYslv6
14	1.04E+08	G	A	-0.0278	0.0048	5.77E-09	0.284	NA	170756	rs4243738	329443	ieu-b-102	TRUE	reported	WYslv6
14	1.04E+08	G	A	-0.0278	0.0048	6.04E-09	0.2839	NA	170756	rs4243739	329443	ieu-b-102	TRUE	reported	WYslv6
14	1.04E+08	C	T	-0.0278	0.0048	5.82E-09	0.2834	NA	170756	rs7156471	329443	ieu-b-102	TRUE	reported	WYslv6
14	1.04E+08	T	C	-0.0279	0.0048	5.14E-09	0.2834	NA	170756	rs1243161	329443	ieu-b-102	TRUE	reported	WYslv6
14	1.04E+08	A	T	-0.0284	0.0049	6.24E-09	0.2778	NA	170756	rs7147640	329443	ieu-b-102	TRUE	reported	WYslv6
14	1.04E+08	T	C	-0.0279	0.0048	5.26E-09	0.2834	NA	170756	rs4906320	329443	ieu-b-102	TRUE	reported	WYslv6
14	1.04E+08	G	A	-0.0278	0.0048	5.52E-09	0.2837	NA	170756	rs7159668	329443	ieu-b-102	TRUE	reported	WYslv6
14	1.04E+08	A	G	-0.0266	0.0048	2.34E-08	0.2856	NA	170756	rs4906322	329443	ieu-b-102	TRUE	reported	WYslv6
14	1.04E+08	C	T	0.0268	0.0048	1.72E-08	0.7123	NA	170756	rs7148567	329443	ieu-b-102	TRUE	reported	WYslv6
14	1.04E+08	C	T	-0.0279	0.0048	5.14E-09	0.2829	NA	170756	rs6575985	329443	ieu-b-102	TRUE	reported	WYslv6
14	1.04E+08	A	T	-0.028	0.0048	4.87E-09	0.2833	NA	170756	rs975892	329443	ieu-b-102	TRUE	reported	WYslv6
14	1.04E+08	C	T	0.0279	0.0048	4.86E-09	0.7118	NA	170756	rs3551255	329443	ieu-b-102	TRUE	reported	WYslv6
14	1.04E+08	A	G	-0.0279	0.0048	5.26E-09	0.2834	NA	170756	rs3523330	329443	ieu-b-102	TRUE	reported	WYslv6
14	1.04E+08	A	C	0.0268	0.0048	1.65E-08	0.7124	NA	170756	rs1014405	329443	ieu-b-102	TRUE	reported	WYslv6
14	1.04E+08	G	A	0.0267	0.0048	1.94E-08	0.7124	NA	170756	rs6575986	329443	ieu-b-102	TRUE	reported	WYslv6
14	1.04E+08	G	A	-0.028	0.0048	4.68E-09	0.2829	NA	170756	rs3541043	329443	ieu-b-102	TRUE	reported	WYslv6
14	1.04E+08	A	G	0.0269	0.0048	1.63E-08	0.7126	NA	170756	rs7144271	329443	ieu-b-102	TRUE	reported	WYslv6
14	1.04E+08	G	A	0.0267	0.0048	1.95E-08	0.7124	NA	170756	rs1014897	329443	ieu-b-102	TRUE	reported	WYslv6
14	1.04E+08	A	G	-0.0278	0.0048	5.36E-09	0.2839	NA	170756	rs7140647	329443	ieu-b-102	TRUE	reported	WYslv6
14	1.04E+08	C	T	-0.0279	0.0048	4.93E-09	0.2827	NA	170756	rs1784106	329443	ieu-b-102	TRUE	reported	WYslv6
14	1.04E+08	G	A	-0.0279	0.0048	4.79E-09	0.2826	NA	170756	rs3441178	329443	ieu-b-102	TRUE	reported	WYslv6
14	1.04E+08	A	C	0.0268	0.0048	1.79E-08	0.713	NA	170756	rs6575988	329443	ieu-b-102	TRUE	reported	WYslv6
14	1.04E+08	A	G	-0.0279	0.0048	4.95E-09	0.2827	NA	170756	rs8018400	329443	ieu-b-102	TRUE	reported	WYslv6
14	1.04E+08	C	T	-0.0277	0.0048	7.04E-09	0.283	NA	170756	rs8003740	329443	ieu-b-102	TRUE	reported	WYslv6
14	1.04E+08	T	C	-0.0273	0.0048	1.11E-08	0.2822	NA	170756	rs6023542	329443	ieu-b-102	TRUE	reported	WYslv6
14	1.04E+08	C	A	-0.0275	0.0048	7.77E-09	0.2826	NA	170756	rs8016676	329443	ieu-b-102	TRUE	reported	WYslv6
14	1.04E+08	A	G	-0.028	0.0048	4.53E-09	0.2827	NA	170756	rs7156036	329443	ieu-b-102	TRUE	reported	WYslv6
14	1.04E+08	C	T	-0.0278	0.0048	5.70E-09	0.2827	NA	170756	rs6575991	329443	ieu-b-102	TRUE	reported	WYslv6
14	1.04E+08	A	G	-0.028	0.0048	4.89E-09	0.282	NA	170756	rs7146215	329443	ieu-b-102	TRUE	reported	WYslv6
14	1.04E+08	G	C	0.0265	0.0048	2.61E-08	0.7133	NA	170756	rs2273702	329443	ieu-b-102	TRUE	reported	WYslv6
14	1.04E+08	A	G	-0.0277	0.0048	6.22E-09	0.2826	NA	170756	rs2403174	329443	ieu-b-102	TRUE	reported	WYslv6
14	1.04E+08	A	G	-0.0277	0.0048	6.56E-09	0.2826	NA	170756	rs4906324	329443	ieu-b-102	TRUE	reported	WYslv6
14	1.04E+08	G	T	-0.0277	0.0048	6.55E-09	0.2826	NA	170756	rs2273701	329443	ieu-b-102	TRUE	reported	WYslv6
14	1.04E+08	T	C	0.0265	0.0048	2.54E-08	0.7131	NA	170756	rs1276690	329443	ieu-b-102	TRUE	reported	WYslv6
14	1.04E+08	A	G	-0.0274	0.0048	1.28E-08	0.2813	NA	170756	rs1368021	329443	ieu-b-102	TRUE	reported	WYslv6
14	1.04E+08	C	T	-0.0276	0.0048	7.91E-09	0.2826	NA	170756	rs1258827	329443	ieu-b-102	TRUE	reported	WYslv6
14	1.04E+08	A	G	0.0263	0.0048	3.32E-08	0.7129	NA	170756	rs7148614	329443	ieu-b-102	TRUE	reported	WYslv6
14	1.04E+08	G	C	-0.0278	0.0048	6.13E-09	0.2823	NA	170756	rs1243672	329443	ieu-b-102	TRUE	reported	WYslv6
14	1.04E+08	C	T	-0.0277	0.0048	6.72E-09	0.2823	NA	170756	rs1243280	329443	ieu-b-102	TRUE	reported	WYslv6
14	1.04E+08	C	T	0.0265	0.0048	2.43E-08	0.7134	NA	170756	rs1016242	329443	ieu-b-102	TRUE	reported	WYslv6
14	1.04E+08	A	G	-0.0276	0.0048	7.40E-09	0.2826	NA	170756	rs1116095	329443	ieu-b-102	TRUE	reported	WYslv6
14	1.04E+08	A	G	-0.0276	0.0048	7.40E-09	0.2826	NA	170756	rs8010247	329443	ieu-b-102	TRUE	reported	WYslv6
14	1.04E+08	G	A	-0.028	0.0048	4.76E-09	0.2819	NA	170756	rs8015429	329443	ieu-b-102	TRUE	reported	WYslv6
14	1.04E+08	T	C	-0.0278	0.0048	6.96E-09	0.2817	NA	170756	rs8014013	329443	ieu-b-102	TRUE	reported	WYslv6
14	1.04E+08	A	G	-0.0277	0.0048	7.39E-09	0.2817	NA	170756	rs2015638	329443	ieu-b-102	TRUE	reported	WYslv6
14	1.04E+08	A	G	-0.0277	0.0048	7.36E-09	0.2817	NA	170756	rs5574228	329443	ieu-b-102	TRUE	reported	WYslv6
14	1.04E+08	T	G	0.0275	0.0048	8.27E-09	0.7178	NA	170756	rs1162089	329443	ieu-b-102	TRUE	reported	WYslv6
14	1.04E+08	C	T	-0.0276	0.0048	8.16E-09	0.2814	NA	170756	rs5734412	329443	ieu-b-102	TRUE	reported	WYslv6
14	1.04E+08	T	C	-0.0276	0.0048	7.94E-09	0.2817	NA	170756	rs8014800	329443	ieu-b-102	TRUE	reported	WYslv6
14	1.04E+08	G	C	0.0275	0.0048	8.38E-09	0.7178	NA	170756	rs1162744	329443	ieu-b-102	TRUE	reported	WYslv6
14	1.04E+08	A	G	0.0278	0.0048	5.98E-09	0.718	NA	170756	rs5565807	329443	ieu-b-102	TRUE	reported	WYslv6
14	1.04E+08	G	C	-0.0278	0.0048	6.57E-09	0.2815	NA	170756	rs3483123	329443	ieu-b-102	TRUE	reported	WYslv6
14	1.04E+08	G	T	-0.0288	0.0048	2.37E-09	0.2821	NA	170756	rs1287854	329443	ieu-b-102	TRUE	reported	WYslv6
14	1.04E+08	A	G	0.0277	0.0048	7.45E-09	0.7175	NA	170756	rs3783399	329443	ieu-b-102	TRUE	reported	WYslv6
14	1.04E+08	T	C	-0.0277	0.0048	7.16E-09	0.2815	NA	170756	rs1243551	329443	ieu-b-102	TRUE	reported	WYslv6
14	1.04E+08	T	C	0.0276	0.0048	7.96E-09	0.7179	NA	170756	rs4143998	329443	ieu-b-102	TRUE	reported	WYslv6
14	1.04E+08	A	G	0.0276	0.0048	8.14E-09	0.718	NA	170756	rs1138400	329443	ieu-b-102	TRUE	reported	WYslv6
14	1.04E+08	C	T	-0.0274	0.0048	1.33E-08	0.2794	NA	170756	rs7802614	329443	ieu-b-102	TRUE	reported	WYslv6

14	1.04E+08	G	A	-0.0274	0.0048	1.11E-08	0.282	NA	170756	rs2756135	329443	ieu-b-102	TRUE	reported	WYslv6
14	1.04E+08	G	A	0.0274	0.0048	1.12E-08	0.7178	NA	170756	rs1243569	329443	ieu-b-102	TRUE	reported	WYslv6
14	1.04E+08	A	G	0.028	0.0048	6.19E-09	0.7173	NA	170756	rs9671414	329443	ieu-b-102	TRUE	reported	WYslv6
14	1.04E+08	G	C	-0.0276	0.0048	8.89E-09	0.2817	NA	170756	rs2756132	329443	ieu-b-102	TRUE	reported	WYslv6
14	1.04E+08	G	T	-0.0277	0.0048	7.32E-09	0.2816	NA	170756	rs1020851	329443	ieu-b-102	TRUE	reported	WYslv6
14	1.04E+08	T	C	-0.0271	0.0048	1.92E-08	0.282	NA	170756	rs1404849	329443	ieu-b-102	TRUE	reported	WYslv6
14	1.04E+08	C	T	0.0274	0.0048	1.04E-08	0.7176	NA	170756	rs2403192	329443	ieu-b-102	TRUE	reported	WYslv6
14	1.04E+08	C	T	-0.0274	0.0048	1.14E-08	0.282	NA	170756	rs2756128	329443	ieu-b-102	TRUE	reported	WYslv6
14	1.04E+08	G	A	-0.0273	0.0048	1.29E-08	0.282	NA	170756	rs2765049	329443	ieu-b-102	TRUE	reported	WYslv6
14	1.04E+08	T	C	0.0268	0.0048	2.40E-08	0.7145	NA	170756	rs1184879	329443	ieu-b-102	TRUE	reported	WYslv6
14	1.04E+08	G	A	-0.0277	0.0048	7.96E-09	0.2817	NA	170756	rs2765046	329443	ieu-b-102	TRUE	reported	WYslv6
14	1.04E+08	A	G	-0.0276	0.0048	9.43E-09	0.2817	NA	170756	rs2023395	329443	ieu-b-102	TRUE	reported	WYslv6
14	1.04E+08	G	C	-0.0276	0.0048	8.89E-09	0.2819	NA	170756	rs7770057	329443	ieu-b-102	TRUE	reported	WYslv6
14	1.04E+08	G	A	-0.0281	0.0045	4.74E-10	0.3629	NA	170756	rs2071408	329443	ieu-b-102	TRUE	reported	WYslv6
14	1.04E+08	T	C	0.0286	0.0045	2.16E-10	0.635	NA	170756	rs2071407	329443	ieu-b-102	TRUE	reported	WYslv6
14	1.04E+08	T	A	-0.0285	0.0045	2.86E-10	0.3624	NA	170756	rs2756121	329443	ieu-b-102	TRUE	reported	WYslv6
14	1.04E+08	G	A	-0.0283	0.0045	3.62E-10	0.3632	NA	170756	rs2765042	329443	ieu-b-102	TRUE	reported	WYslv6
14	1.04E+08	C	T	-0.0283	0.0045	3.62E-10	0.3635	NA	170756	rs2756118	329443	ieu-b-102	TRUE	reported	WYslv6
14	1.04E+08	T	A	-0.0289	0.0045	1.31E-10	0.3664	NA	170756	rs754287	329443	ieu-b-102	TRUE	reported	WYslv6
14	1.04E+08	G	C	-0.0286	0.0045	2.40E-10	0.3671	NA	170756	rs911555	329443	ieu-b-102	TRUE	reported	WYslv6
14	1.04E+08	G	T	-0.0287	0.0045	1.58E-10	0.376	NA	170756	rs911554	329443	ieu-b-102	TRUE	reported	WYslv6
14	1.04E+08	A	G	-0.0286	0.0045	2.16E-10	0.3675	NA	170756	rs911552	329443	ieu-b-102	TRUE	reported	WYslv6
14	1.04E+08	C	T	-0.0287	0.0045	2.15E-10	0.3675	NA	170756	rs2756127	329443	ieu-b-102	TRUE	reported	WYslv6
14	1.04E+08	G	A	-0.0281	0.0045	3.86E-10	0.3821	NA	170756	rs2756119	329443	ieu-b-102	TRUE	reported	WYslv6
14	1.04E+08	C	T	0.0288	0.0046	3.65E-10	0.659	NA	170756	rs2403193	329443	ieu-b-102	TRUE	reported	WYslv6
14	1.04E+08	G	A	-0.0299	0.0049	9.24E-10	0.2806	NA	170756	rs6199670	329443	ieu-b-102	TRUE	reported	WYslv6
14	1.04E+08	G	T	0.0277	0.0046	1.56E-09	0.6579	NA	170756	rs942866	329443	ieu-b-102	TRUE	reported	WYslv6
14	1.04E+08	A	G	0.0266	0.0043	7.44E-10	0.5131	NA	170756	rs1014947	329443	ieu-b-102	TRUE	reported	WYslv6
14	1.04E+08	T	C	0.0267	0.0043	6.69E-10	0.5127	NA	170756	rs1014115	329443	ieu-b-102	TRUE	reported	WYslv6
14	1.04E+08	G	A	0.0251	0.0044	8.37E-09	0.5404	NA	170756	rs1012942	329443	ieu-b-102	TRUE	reported	WYslv6
14	1.04E+08	T	C	0.0277	0.0045	9.37E-10	0.6459	NA	170756	rs752624	329443	ieu-b-102	TRUE	reported	WYslv6
14	1.04E+08	G	C	0.0269	0.0045	3.15E-09	0.6505	NA	170756	rs1234058	329443	ieu-b-102	TRUE	reported	WYslv6
14	1.04E+08	C	T	0.0276	0.0045	9.27E-10	0.6454	NA	170756	rs1162539	329443	ieu-b-102	TRUE	reported	WYslv6
14	1.04E+08	G	A	0.0267	0.0045	2.43E-09	0.6321	NA	170756	rs1334775	329443	ieu-b-102	TRUE	reported	WYslv6
14	1.04E+08	G	C	0.026	0.0045	7.37E-09	0.6377	NA	170756	rs9652403	329443	ieu-b-102	TRUE	reported	WYslv6
14	1.04E+08	C	T	0.0258	0.0045	1.38E-08	0.6444	NA	170756	rs1302825	329443	ieu-b-102	TRUE	reported	WYslv6
14	1.04E+08	T	C	0.0248	0.0045	3.73E-08	0.6382	NA	170756	rs1243829	329443	ieu-b-102	TRUE	reported	WYslv6
14	1.04E+08	C	T	0.0248	0.0045	3.71E-08	0.6362	NA	170756	rs8007489	329443	ieu-b-102	TRUE	reported	WYslv6
14	1.04E+08	G	C	-0.0285	0.0047	8.68E-10	0.3154	NA	170756	rs1243539	329443	ieu-b-102	TRUE	reported	WYslv6
14	1.04E+08	T	C	0.0268	0.0045	3.70E-09	0.6604	NA	170756	rs2879850	329443	ieu-b-102	TRUE	reported	WYslv6
14	1.04E+08	T	C	0.0268	0.0046	4.17E-09	0.6604	NA	170756	rs1289751	329443	ieu-b-102	TRUE	reported	WYslv6
14	1.04E+08	T	C	0.0268	0.0045	3.72E-09	0.6601	NA	170756	rs2882363	329443	ieu-b-102	TRUE	reported	WYslv6
14	1.04E+08	A	G	0.0271	0.0045	2.31E-09	0.6603	NA	170756	rs7161125	329443	ieu-b-102	TRUE	reported	WYslv6
14	1.04E+08	T	C	0.0267	0.0046	4.52E-09	0.6597	NA	170756	rs1162658	329443	ieu-b-102	TRUE	reported	WYslv6
14	1.04E+08	A	G	0.0271	0.0045	2.39E-09	0.6601	NA	170756	rs4383078	329443	ieu-b-102	TRUE	reported	WYslv6
14	1.04E+08	C	T	0.0254	0.0045	2.27E-08	0.6526	NA	170756	rs4464019	329443	ieu-b-102	TRUE	reported	WYslv6
14	1.04E+08	G	C	0.0271	0.0045	2.36E-09	0.6603	NA	170756	rs7710089	329443	ieu-b-102	TRUE	reported	WYslv6
14	1.04E+08	A	C	0.0271	0.0045	2.49E-09	0.6603	NA	170756	rs4374097	329443	ieu-b-102	TRUE	reported	WYslv6
14	1.04E+08	G	A	0.0271	0.0045	2.56E-09	0.6603	NA	170756	rs745079	329443	ieu-b-102	TRUE	reported	WYslv6
14	1.04E+08	G	A	0.0271	0.0045	2.60E-09	0.6602	NA	170756	rs1042513	329443	ieu-b-102	TRUE	reported	WYslv6
14	1.04E+08	T	C	0.0265	0.0046	6.09E-09	0.6577	NA	170756	rs8006526	329443	ieu-b-102	TRUE	reported	WYslv6
14	1.04E+08	G	A	0.0264	0.0046	6.82E-09	0.6578	NA	170756	rs3425812	329443	ieu-b-102	TRUE	reported	WYslv6
14	1.04E+08	G	A	0.0268	0.0045	3.80E-09	0.6601	NA	170756	rs1475902	329443	ieu-b-102	TRUE	reported	WYslv6
14	1.04E+08	T	C	0.027	0.0045	2.73E-09	0.6603	NA	170756	rs6199576	329443	ieu-b-102	TRUE	reported	WYslv6
14	1.04E+08	G	A	-0.0284	0.0046	1.06E-09	0.3156	NA	170756	rs4906340	329443	ieu-b-102	TRUE	reported	WYslv6
14	1.04E+08	G	C	0.0269	0.0046	4.58E-09	0.6611	NA	170756	rs4906341	329443	ieu-b-102	TRUE	reported	WYslv6
14	1.04E+08	A	G	0.0271	0.0045	2.39E-09	0.6603	NA	170756	rs7155511	329443	ieu-b-102	TRUE	reported	WYslv6
14	1.04E+08	T	C	0.0265	0.0045	5.64E-09	0.6597	NA	170756	rs7157116	329443	ieu-b-102	TRUE	reported	WYslv6
14	1.04E+08	A	G	0.0269	0.0045	3.26E-09	0.6608	NA	170756	rs4318144	329443	ieu-b-102	TRUE	reported	WYslv6
14	1.04E+08	T	C	0.0265	0.0046	5.76E-09	0.6594	NA	170756	rs1287900	329443	ieu-b-102	TRUE	reported	WYslv6
14	1.04E+08	A	G	0.0266	0.0046	5.20E-09	0.6596	NA	170756	rs4906342	329443	ieu-b-102	TRUE	reported	WYslv6
14	1.04E+08	A	G	0.0259	0.0045	1.29E-08	0.6582	NA	170756	rs9024624	329443	ieu-b-102	TRUE	reported	WYslv6
14	1.04E+08	G	C	0.027	0.0045	2.94E-09	0.6607	NA	170756	rs2403202	329443	ieu-b-102	TRUE	reported	WYslv6
14	1.04E+08	A	G	0.0268	0.0046	3.90E-09	0.6597	NA	170756	rs8020099	329443	ieu-b-102	TRUE	reported	WYslv6
14	1.04E+08	C	T	0.0282	0.0046	7.39E-10	0.6639	NA	170756	rs4906348	329443	ieu-b-102	TRUE	reported	WYslv6
14	1.04E+08	A	G	-0.0285	0.0047	9.30E-10	0.3119	NA	170756	rs4906349	329443	ieu-b-102	TRUE	reported	WYslv6
14	1.04E+08	A	G	0.0272	0.0046	2.39E-09	0.6626	NA	170756	rs3759586	329443	ieu-b-102	TRUE	reported	WYslv6
14	1.04E+08	C	T	0.0275	0.0045	1.52E-09	0.6626	NA	170756	rs729437	329443	ieu-b-102	TRUE	reported	WYslv6
14	1.04E+08	A	C	0.0262	0.0047	2.37E-08	0.6992	NA	170756	rs1054080	329443	ieu-b-102	TRUE	reported	WYslv6
14	1.04E+08	G	T	0.0268	0.0047	1.15E-08	0.6936	NA	170756	rs1394893	329443	ieu-b-102	TRUE	reported	WYslv6
14	1.04E+08	T	C	0.0268	0.0047	1.23E-08	0.699	NA	170756	rs3742465	329443	ieu-b-102	TRUE	reported	WYslv6
14	1.04E+08	T	C	0.027	0.0047	9.84E-09	0.6987	NA	170756	rs1185097	329443	ieu-b-102	TRUE	reported	WYslv6
14	1.04E+08	G	A	0.0272	0.0047	8.42E-09	0.6969	NA	170756	rs8021904	329443	ieu-b-102	TRUE	reported	WYslv6
14	1.04E+08	C	T	0.0271	0.0047	7.64E-09	0.6992	NA	170756	rs8007915	329443	ieu-b-102	TRUE	reported	WYslv6
14	1.04E+08	A	G	0.0271	0.0047	7.61E-09	0.6995	NA	170756	rs8008451	329443	ieu-b-102	TRUE	reported	WYslv6
14	1.04E+08	A	G	0.0282	0.0047	2.27E-09	0.6997	NA	170756	rs2896489	329443	ieu-b-102	TRUE	reported	WYslv6
14	1.04E+08	A	G	0.0269	0.0047	9.83E-09	0.6976	NA	170756	rs1243286	329443	ieu-b-102	TRUE	reported	WYslv6
14	1.04E+08	T	C	0.0271	0.0047	7.66E-09	0.6992	NA	170756	rs1567012	329443	ieu-b-102	TRUE	reported	WYslv6
14	1.04E+08	T	A	0.027	0.0047	8.67E-09	0.6992	NA	170756	rs7808392	329443	ieu-b-102	TRUE	reported	WYslv6
14	1.04E+08	T	C	0.0279	0.0047	3.38E-09	0.6991	NA	170756	rs2403203	329443	ieu-b-102	TRUE	reported	WYslv6
14	1.04E+08	C	G	0.027	0.0047	8.84E-09	0.6991	NA	170756	rs4900586	329443	ieu-b-102	TRUE	reported	WYslv6
14	1.04E+08	A	G	0.0281	0.0047	2.46E-09	0.6997	NA	170756	rs4906355	329443	ieu-b-102	TRUE	reported	WYslv6
14	1.04E+08	A	G	0.0264	0.0047	1.88E-08	0.6965	NA	170756	rs1013106	329443	ieu-b-102	TRUE	reported	WYslv6
14	1.04E+08	C	T	0.027	0.0047	7.97E-09	0.6991	NA	170756	rs4906358	329443	ieu-b-102	TRUE	reported	WYslv6
14	1.04E+08	G	A	0.0271	0.0047	7.17E-09	0.6991	NA	170756	rs8003289	329443	ieu-b-102	TRUE	reported	WYslv6
14	1.04E+08	A	G	0.0275	0.0047	6.32E-09	0.707	NA	170756	rs4900589	329443	ieu-b-102	TRUE	reported	WYslv6
14	1.04E+08	C	G	0.0271	0.0047	8.24E-09	0.6995	NA	170756	rs8702	329443	ieu-b-102	TRUE	reported	WYslv6
14	1.04E+08	C	T	0.0271	0.0047	7.25E-09	0.6991	NA	170756	rs1478573	329443	ieu-b-102	TRUE	reported	WYslv6
14	1.04E+08	A	G	0.0271	0.0047	7.31E-09	0.6991	NA	170756	rs861545	329443	ieu-b-102	TRUE	reported	WYslv6
14	1.04E+08	G	A	0.0269	0.0047	9.00E-09	0.6901	NA	170756	rs861544	329443	ieu-b-102	TRUE	reported	WYslv6
14	1.04E+08	C	T	0.027	0.0047	8.57E-09	0.6992	NA	170756	rs861537	329443	ieu-b-102	TRUE	reported	WYslv6
14	1.04E+08	G	A	0.0269	0.0047	9.01E-09	0.6914	NA	170756	rs709399	329443	ieu-b-102	TRUE	reported	WYslv6
14	1.04E+08	T	C	0.0273	0.0047	7.27E-09	0.7003	NA	170756	rs861530	329443	ieu-b-102	TRUE	reported	WYslv6
15	88943807	A	G	-0.0284	0.0051	3.07E-08	0.2367	NA	170756	rs3867861	329443	ieu-b-102	TRUE	reported	WYslv6
15	88945878	C	G	-0.0292	0.0052	1.76E-08	0.2308	NA	170756	rs2854141	329443	ieu-b-102	TRUE	reported	WYslv6
16	13764396	G	T	0.0247	0.0044	2.65E-08	0.3756	NA	170756	rs6498457	329443	ieu-b-102	TRUE	reported	WYslv6
16	13764456	C	A	0.0245	0.0044	3.39E-08	0.376	NA	170756	rs4997735	329443	ieu-b-102	TRUE	reported	WYslv6
16	13764680	T	C	0.0242	0.0044	4.88E-08	0.3752	NA	170756	rs2097287	329443	ieu-b-102	TRUE	reported	WYslv6
16	13764829	C	T	0.0245	0.0044	3.29E-08	0.3754	NA	170756	rs2105842	329443	ieu-b-102	TRUE	reported	WYslv6
16	13764965	A	C	0.0245	0.0044	3.40E-08	0.3758	NA	170756	rs7195634	329443	ieu-b-102	TRUE	reported	WYslv6
16	13765041	G	T	0.0242	0.0044	4.88E-08	0.3752	NA	170756	rs1292759	329443	ieu-b-102	TRUE	reported	WYslv6
16	13765063	T	G	0.0246	0.0044	2.91E-08	0.3756	NA	170756	rs7200727	329443	ieu-b-102	TRUE	reported	WYslv6
16	13765383	T	G	0.0245	0.0044	3.23E-08	0.3759	NA	170756	rs1293361	329443	ieu-b-102	TRUE	reported	WYslv6
16	13766292	G	A	0.0246	0.0044	3.18E-08	0.3753	NA	170756	rs7532205	329443	ieu-b-102	TRUE	reported	WYslv6
16	13766427	C	T	0.0307	0.0055	2.22E-08	0.188	NA	170756	rs4781529	329443	ieu-b-102	TRUE	reported	WYslv6
16	13767133	G	C	0.0244	0.0044	3.70E-08	0.3754	NA	170756	rs1291979	329443	ieu-b-102	TRUE	reported	WYslv6
16	13767712	A	G	0.0246	0.0044	2.94E-08	0.3757	NA	170756	rs9458936	329443	ieu-b-102	TRUE	reported	WYslv6

16	13768459	T	C	0.0246	0.0044	2.94E-08	0.3757	NA	170756	rs7186763	329443	ieu-b-102	TRUE	reported	WYslv6
16	13769573	T	G	0.0315	0.0055	9.97E-09	0.1884	NA	170756	rs2866725	329443	ieu-b-102	TRUE	reported	WYslv6
16	13771024	T	G	0.0248	0.0044	2.16E-08	0.3759	NA	170756	rs4780534	329443	ieu-b-102	TRUE	reported	WYslv6
16	13771735	T	C	0.0311	0.0055	1.50E-08	0.1883	NA	170756	rs6203509	329443	ieu-b-102	TRUE	reported	WYslv6
16	13772118	T	C	0.031	0.0056	2.41E-08	0.1832	NA	170756	rs3587116	329443	ieu-b-102	TRUE	reported	WYslv6
16	13772156	T	G	0.0248	0.0044	2.36E-08	0.3759	NA	170756	rs3574320	329443	ieu-b-102	TRUE	reported	WYslv6
16	13772258	A	G	0.0249	0.0044	2.14E-08	0.3759	NA	170756	rs3511320	329443	ieu-b-102	TRUE	reported	WYslv6
16	13772599	G	T	0.0247	0.0044	2.50E-08	0.3758	NA	170756	rs2051545	329443	ieu-b-102	TRUE	reported	WYslv6
16	13773131	A	G	0.0317	0.0055	7.26E-09	0.1891	NA	170756	rs1696262	329443	ieu-b-102	TRUE	reported	WYslv6
16	13774412	C	T	0.0249	0.0045	2.40E-08	0.377	NA	170756	rs3595134	329443	ieu-b-102	TRUE	reported	WYslv6
16	13774434	G	A	0.0249	0.0044	1.93E-08	0.3763	NA	170756	rs1217128	329443	ieu-b-102	TRUE	reported	WYslv6
16	13774810	G	A	0.0314	0.0056	1.52E-08	0.1846	NA	170756	rs1293314	329443	ieu-b-102	TRUE	reported	WYslv6
16	13777395	T	G	0.0246	0.0044	2.81E-08	0.376	NA	170756	rs1244862	329443	ieu-b-102	TRUE	reported	WYslv6
16	13777867	T	C	0.0246	0.0044	2.79E-08	0.376	NA	170756	rs1259698	329443	ieu-b-102	TRUE	reported	WYslv6
16	13778470	T	A	0.0319	0.0056	9.48E-09	0.1834	NA	170756	rs1244512	329443	ieu-b-102	TRUE	reported	WYslv6
16	13778853	C	G	0.0252	0.0044	1.38E-08	0.3766	NA	170756	rs4780537	329443	ieu-b-102	TRUE	reported	WYslv6
16	13779329	T	C	0.0402	0.0074	4.87E-08	0.0945	NA	170756	rs7672885	329443	ieu-b-102	TRUE	reported	WYslv6
16	13780276	C	T	0.0244	0.0044	4.09E-08	0.3713	NA	170756	rs1293339	329443	ieu-b-102	TRUE	reported	WYslv6
16	13780935	A	G	0.0252	0.0044	1.38E-08	0.3769	NA	170756	rs1201755	329443	ieu-b-102	TRUE	reported	WYslv6
16	13781491	A	C	0.0252	0.0044	1.42E-08	0.3771	NA	170756	rs2866726	329443	ieu-b-102	TRUE	reported	WYslv6
16	13781498	T	C	0.0252	0.0044	1.41E-08	0.3771	NA	170756	rs2866727	329443	ieu-b-102	TRUE	reported	WYslv6
16	13781527	T	C	0.0246	0.0044	3.26E-08	0.3717	NA	170756	rs2866728	329443	ieu-b-102	TRUE	reported	WYslv6
16	13782309	A	G	0.025	0.0044	1.78E-08	0.3771	NA	170756	rs4780539	329443	ieu-b-102	TRUE	reported	WYslv6
16	13783279	A	G	0.0248	0.0044	2.10E-08	0.3772	NA	170756	rs3557267	329443	ieu-b-102	TRUE	reported	WYslv6
16	13783390	T	C	0.0242	0.0044	4.83E-08	0.3718	NA	170756	rs1719942	329443	ieu-b-102	TRUE	reported	WYslv6
16	13784579	G	A	0.0248	0.0044	2.23E-08	0.3769	NA	170756	rs928975	329443	ieu-b-102	TRUE	reported	WYslv6
16	13785444	G	A	0.0243	0.0044	4.49E-08	0.3714	NA	170756	rs3455885	329443	ieu-b-102	TRUE	reported	WYslv6
16	13785959	G	A	0.0307	0.0053	8.14E-09	0.2077	NA	170756	rs1210224	329443	ieu-b-102	TRUE	reported	WYslv6
16	13786097	C	T	0.0315	0.0055	1.30E-08	0.1844	NA	170756	rs1292782	329443	ieu-b-102	TRUE	reported	WYslv6
16	13786757	T	G	0.0243	0.0045	4.74E-08	0.3757	NA	170756	rs3402041	329443	ieu-b-102	TRUE	reported	WYslv6
16	13790356	A	T	0.0308	0.0055	2.86E-08	0.1892	NA	170756	rs1293287	329443	ieu-b-102	TRUE	reported	WYslv6
16	13793837	A	T	0.0252	0.0045	1.66E-08	0.3768	NA	170756	rs1573634	329443	ieu-b-102	TRUE	reported	WYslv6
16	13800430	G	C	0.0327	0.0055	3.09E-09	0.1884	NA	170756	rs1291929	329443	ieu-b-102	TRUE	reported	WYslv6
18	35125161	C	T	0.0288	0.0052	2.45E-08	0.2283	NA	170756	rs948537	329443	ieu-b-102	TRUE	reported	WYslv6
18	35125314	A	G	0.0284	0.0051	3.40E-08	0.2289	NA	170756	rs948536	329443	ieu-b-102	TRUE	reported	WYslv6
18	35125862	C	T	0.0286	0.0051	2.35E-08	0.2314	NA	170756	rs7506216	329443	ieu-b-102	TRUE	reported	WYslv6
18	35126708	C	T	-0.029	0.005	7.22E-09	0.7502	NA	170756	rs7244667	329443	ieu-b-102	TRUE	reported	WYslv6
18	35127427	A	C	-0.0283	0.0046	6.65E-10	0.6676	NA	170756	rs1557341	329443	ieu-b-102	TRUE	reported	WYslv6
18	35129076	C	A	-0.0294	0.005	4.37E-09	0.7508	NA	170756	rs1557339	329443	ieu-b-102	TRUE	reported	WYslv6
18	35131444	A	G	-0.0291	0.005	5.63E-09	0.7517	NA	170756	rs1539853	329443	ieu-b-102	TRUE	reported	WYslv6
18	35134259	C	T	-0.0293	0.005	4.22E-09	0.7509	NA	170756	rs1260775	329443	ieu-b-102	TRUE	reported	WYslv6
18	35138110	T	G	0.0292	0.0047	3.37E-10	0.313	NA	170756	rs1187775	329443	ieu-b-102	TRUE	reported	WYslv6
18	35138245	A	G	-0.0294	0.0046	1.50E-10	0.6697	NA	170756	rs1296785	329443	ieu-b-102	TRUE	reported	WYslv6
18	35142133	C	T	0.0307	0.0051	2.11E-09	0.2281	NA	170756	rs6208146	329443	ieu-b-102	TRUE	reported	WYslv6
18	35145122	C	T	-0.029	0.0046	2.22E-10	0.6691	NA	170756	rs1108201	329443	ieu-b-102	TRUE	reported	WYslv6
18	35146366	T	A	0.0311	0.0051	1.51E-09	0.2254	NA	170756	rs8003506	329443	ieu-b-102	TRUE	reported	WYslv6
18	35147621	C	G	-0.0304	0.005	1.31E-09	0.7528	NA	170756	rs1296948	329443	ieu-b-102	TRUE	reported	WYslv6
18	35150526	G	A	0.0308	0.0051	1.90E-09	0.2284	NA	170756	rs9953231	329443	ieu-b-102	TRUE	reported	WYslv6
18	35152563	G	A	-0.0293	0.0046	1.41E-10	0.6684	NA	170756	rs1166507	329443	ieu-b-102	TRUE	reported	WYslv6
18	35154196	T	C	0.031	0.0051	1.47E-09	0.2252	NA	170756	rs6208146	329443	ieu-b-102	TRUE	reported	WYslv6
18	35154480	A	G	-0.0309	0.0051	1.72E-09	0.7713	NA	170756	rs1295964	329443	ieu-b-102	TRUE	reported	WYslv6
18	35154734	G	A	0.0309	0.0051	1.83E-09	0.2252	NA	170756	rs1757035	329443	ieu-b-102	TRUE	reported	WYslv6
18	35155910	C	T	-0.0292	0.0046	1.40E-10	0.6684	NA	170756	rs4799949	329443	ieu-b-102	TRUE	reported	WYslv6
18	35156177	A	G	0.0308	0.0051	1.87E-09	0.2254	NA	170756	rs7243428	329443	ieu-b-102	TRUE	reported	WYslv6
18	35156196	A	G	0.0308	0.0051	1.83E-09	0.2254	NA	170756	rs7243439	329443	ieu-b-102	TRUE	reported	WYslv6
18	35156537	C	A	-0.0308	0.0051	1.80E-09	0.7713	NA	170756	rs9955296	329443	ieu-b-102	TRUE	reported	WYslv6
18	35159124	C	T	-0.0279	0.0046	1.79E-09	0.6823	NA	170756	rs9964724	329443	ieu-b-102	TRUE	reported	WYslv6
18	35159172	A	T	-0.0276	0.0046	2.29E-09	0.6828	NA	170756	rs1557343	329443	ieu-b-102	TRUE	reported	WYslv6
18	35159596	A	C	-0.0271	0.0046	3.76E-09	0.6758	NA	170756	rs1941954	329443	ieu-b-102	TRUE	reported	WYslv6
18	35161285	A	G	0.0284	0.0051	1.79E-08	0.2378	NA	170756	rs8096815	329443	ieu-b-102	TRUE	reported	WYslv6
18	35167218	G	A	0.0282	0.0051	2.41E-08	0.2373	NA	170756	rs7289318	329443	ieu-b-102	TRUE	reported	WYslv6
18	35167222	T	C	-0.0246	0.0045	3.94E-08	0.645	NA	170756	rs7237475	329443	ieu-b-102	TRUE	reported	WYslv6
18	35168263	A	G	-0.0246	0.0045	4.08E-08	0.6449	NA	170756	rs1296774	329443	ieu-b-102	TRUE	reported	WYslv6
18	35169497	T	A	-0.0246	0.0045	4.16E-08	0.6441	NA	170756	rs6507210	329443	ieu-b-102	TRUE	reported	WYslv6
18	35171653	G	A	-0.0247	0.0045	3.26E-08	0.6438	NA	170756	rs1295694	329443	ieu-b-102	TRUE	reported	WYslv6
18	35172316	G	A	-0.0245	0.0045	4.31E-08	0.644	NA	170756	rs7505519	329443	ieu-b-102	TRUE	reported	WYslv6
18	35174815	A	G	0.0281	0.0051	2.70E-08	0.2369	NA	170756	rs7289319	329443	ieu-b-102	TRUE	reported	WYslv6
18	35174836	T	G	0.0281	0.0051	2.71E-08	0.2369	NA	170756	rs7289319	329443	ieu-b-102	TRUE	reported	WYslv6
18	35175765	G	A	-0.0264	0.0046	1.31E-08	0.5973	NA	170756	rs7232972	329443	ieu-b-102	TRUE	reported	WYslv6
18	35176161	A	G	-0.0275	0.0049	2.21E-08	0.7417	NA	170756	rs1166424	329443	ieu-b-102	TRUE	reported	WYslv6
18	35179808	G	A	-0.0246	0.0045	4.34E-08	0.6459	NA	170756	rs1296205	329443	ieu-b-102	TRUE	reported	WYslv6
18	35182708	T	C	0.0283	0.0051	3.97E-08	0.2265	NA	170756	rs7289319	329443	ieu-b-102	TRUE	reported	WYslv6
18	35183274	C	G	0.0286	0.0052	2.76E-08	0.226	NA	170756	rs5573123	329443	ieu-b-102	TRUE	reported	WYslv6
18	35185858	A	G	-0.0279	0.005	2.67E-08	0.7584	NA	170756	rs4799461	329443	ieu-b-102	TRUE	reported	WYslv6
18	50614732	G	A	0.0242	0.0043	2.23E-08	0.4614	NA	170756	rs1166339	329443	ieu-b-102	TRUE	reported	WYslv6
18	50617105	G	T	0.024	0.0044	4.07E-08	0.458	NA	170756	rs1221770	329443	ieu-b-102	TRUE	reported	WYslv6
18	50620708	T	C	0.0239	0.0043	2.87E-08	0.4634	NA	170756	rs4459619	329443	ieu-b-102	TRUE	reported	WYslv6
18	50622992	T	C	0.0239	0.0043	3.05E-08	0.464	NA	170756	rs6209918	329443	ieu-b-102	TRUE	reported	WYslv6
18	50630791	C	G	0.0259	0.0044	5.68E-09	0.3802	NA	170756	rs5632730	329443	ieu-b-102	TRUE	reported	WYslv6
18	50633449	T	G	0.0239	0.0043	2.93E-08	0.464	NA	170756	rs4494619	329443	ieu-b-102	TRUE	reported	WYslv6
18	50635119	G	A	0.0245	0.0044	2.42E-08	0.4013	NA	170756	rs1165980	329443	ieu-b-102	TRUE	reported	WYslv6
18	50641696	T	G	0.024	0.0043	2.52E-08	0.4629	NA	170756	rs5020052	329443	ieu-b-102	TRUE	reported	WYslv6
18	50697120	C	T	0.0248	0.0044	1.54E-08	0.4013	NA	170756	rs1748630	329443	ieu-b-102	TRUE	reported	WYslv6
18	50709981	A	G	0.0246	0.0044	1.90E-08	0.4074	NA	170756	rs1740839	329443	ieu-b-102	TRUE	reported	WYslv6
18	50718314	C	T	0.0245	0.0044	2.18E-08	0.4015	NA	170756	rs1748725	329443	ieu-b-102	TRUE	reported	WYslv6
18	50718757	A	G	0.0246	0.0043	1.28E-08	0.4361	NA	170756	rs7232543	329443	ieu-b-102	TRUE	reported	WYslv6
18	50718880	G	A	0.024	0.0043	3.04E-08	0.441	NA	170756	rs7233123	329443	ieu-b-102	TRUE	reported	WYslv6
18	50718944	C	G	0.0242	0.0044	3.32E-08	0.4071	NA	170756	rs1350711	329443	ieu-b-102	TRUE	reported	WYslv6
18	50721712	G	A	0.0241	0.0044	3.77E-08	0.4072	NA	170756	rs6209923	329443	ieu-b-102	TRUE	reported	WYslv6
18	50723080	G	A	0.0243	0.0043	1.61E-08	0.5092	NA	170756	rs7230285	329443	ieu-b-102	TRUE	reported	WYslv6
18	50723179	G	A	0.0243	0.0043	1.62E-08	0.5092	NA	170756	rs7230446	329443	ieu-b-102	TRUE	reported	WYslv6
18	50723283	T	G	0.0243	0.0043	1.65E-08	0.5094	NA	170756	rs1748748	329443	ieu-b-102	TRUE	reported	WYslv6
18	50723460	T	C	0.0237	0.0043	3.64E-08	0.5144	NA	170756	rs2069191	329443	ieu-b-102	TRUE	reported	WYslv6
18	50724899	G	A	0.0238	0.0043	3.12E-08	0.5144	NA	170756	rs6209923	329443	ieu-b-102	TRUE	reported	WYslv6
18	50726294	C	A	0.0251	0.0043	7.38E-09	0.4305	NA	170756	rs1022137	329443	ieu-b-102	TRUE	reported	WYslv6
18	50726559	G	A	0.0246	0.0043	1.05E-08	0.5105	NA	170756	rs8084351	329443	ieu-b-102	TRUE	reported	WYslv6
18	50726749	A	T	0.0245	0.0043	1.25E-08	0.5105	NA	170756	rs8084280	329443	ieu-b-102	TRUE	reported	WYslv6
18	50727518	C	T	0.0246	0.0044	1.87E-08	0.4017	NA	170756	rs3794923	329443	ieu-b-102	TRUE	reported	WYslv6
18	50727691	C	T	0.0246	0.0044	1.88E-08	0.4017	NA	170756	rs1164877	329443	ieu-b-102	TRUE	reported	WYslv6
18	50729267	T	C	0.0247	0.0043	1.14E-08	0.432	NA	170756	rs6093982	329443	ieu-b-102	TRUE	reported	WYslv6
18	50730524	C	T	0.0253	0.0043	5.09E-09	0.4316	NA	170756	rs1568363	329443	ieu-b-102	TRUE	reported	WYslv6
18	50730603	G	A	0.0253	0.0043	5.15E-09	0.4315	NA	170756	rs6209923	329443	ieu-b-102	TRUE	reported	WYslv6
18	50731055	G	T	0.0252	0.0043	6.05E-09	0.4314	NA	170756	rs6209923	329443	ieu-b-102	TRUE	reported	WYslv6
18	50731802	G	A	0.0254	0.0043	4.64E-09	0.4317	NA	170756	rs7227069	329443	ieu-b-102	TRUE	reported	WYslv6
18	50735418	C	T	0.0251	0.0044	9.76E-09	0.4022	NA	170756	rs6210076	329443	ieu-b-102	TRUE	reported	WYslv6
18	50737358	G	T	0.0251	0.0044	9.66E-09	0.4007	NA	170756	rs6210076	329443	ieu-b-102	TRUE	reported	WYslv6
18	50738039	A	G	0.0245	0.0044	2.10E-08	0.4009	NA	170756	rs6210076	329443	ieu-b-102	TRUE	reported	WYslv6

18	50739344	C	G	0.0251	0.0043	6.80E-09	0.4305	NA	170756	rs8086812	329443	ieu-b-102	TRUE	reported	WYslv6
18	50739860	G	A	0.0252	0.0043	6.52E-09	0.4302	NA	170756	rs1114577	329443	ieu-b-102	TRUE	reported	WYslv6
18	50740013	G	A	0.025	0.0044	1.19E-08	0.4332	NA	170756	rs1126334	329443	ieu-b-102	TRUE	reported	WYslv6
18	50740163	A	G	0.0251	0.0043	6.63E-09	0.4304	NA	170756	rs5590605	329443	ieu-b-102	TRUE	reported	WYslv6
18	50741130	A	G	0.0249	0.0044	1.38E-08	0.4012	NA	170756	rs6210077	329443	ieu-b-102	TRUE	reported	WYslv6
18	50741297	G	A	0.0252	0.0043	6.56E-09	0.4302	NA	170756	rs6210077	329443	ieu-b-102	TRUE	reported	WYslv6
18	50745236	C	A	0.0253	0.0044	7.92E-09	0.4018	NA	170756	rs1166382	329443	ieu-b-102	TRUE	reported	WYslv6
18	50746748	C	T	0.0248	0.0043	8.13E-09	0.5165	NA	170756	rs4632195	329443	ieu-b-102	TRUE	reported	WYslv6
18	50749935	A	T	0.0236	0.0043	4.67E-08	0.4488	NA	170756	rs1748831	329443	ieu-b-102	TRUE	reported	WYslv6
18	50750684	A	T	0.0237	0.0043	4.01E-08	0.4487	NA	170756	rs7243008	329443	ieu-b-102	TRUE	reported	WYslv6
18	50751425	G	C	0.0236	0.0043	4.97E-08	0.4483	NA	170756	rs1367638	329443	ieu-b-102	TRUE	reported	WYslv6
18	50753609	T	C	0.0237	0.0043	4.28E-08	0.4483	NA	170756	rs5784132	329443	ieu-b-102	TRUE	reported	WYslv6
18	50754996	G	A	0.0237	0.0043	4.31E-08	0.4483	NA	170756	rs1748853	329443	ieu-b-102	TRUE	reported	WYslv6
18	50776391	T	C	0.0267	0.0044	1.42E-09	0.3917	NA	170756	rs1741055	329443	ieu-b-102	TRUE	reported	WYslv6
18	50807090	T	A	0.0254	0.0045	1.17E-08	0.3775	NA	170756	rs5602765	329443	ieu-b-102	TRUE	reported	WYslv6
18	50807359	A	G	0.0243	0.0043	2.45E-08	0.4323	NA	170756	rs1741133	329443	ieu-b-102	TRUE	reported	WYslv6
18	50810675	C	T	0.0242	0.0044	3.13E-08	0.4222	NA	170756	rs6209798	329443	ieu-b-102	TRUE	reported	WYslv6
18	50818827	C	A	0.0241	0.0044	3.35E-08	0.42	NA	170756	rs6209798	329443	ieu-b-102	TRUE	reported	WYslv6
18	50819236	T	G	0.0239	0.0044	4.20E-08	0.4208	NA	170756	rs6209798	329443	ieu-b-102	TRUE	reported	WYslv6
18	50822943	G	T	0.0253	0.0044	6.19E-09	0.4302	NA	170756	rs1741154	329443	ieu-b-102	TRUE	reported	WYslv6
18	50824885	G	C	0.0251	0.0043	7.81E-09	0.4296	NA	170756	rs1108297	329443	ieu-b-102	TRUE	reported	WYslv6
18	50826478	A	G	0.0244	0.0044	2.20E-08	0.421	NA	170756	rs6209798	329443	ieu-b-102	TRUE	reported	WYslv6
18	50827476	C	T	0.025	0.0043	8.86E-09	0.4301	NA	170756	rs7292534	329443	ieu-b-102	TRUE	reported	WYslv6
18	50829023	A	G	0.0244	0.0044	2.17E-08	0.4208	NA	170756	rs1187750	329443	ieu-b-102	TRUE	reported	WYslv6
18	50832102	A	G	0.0248	0.0043	1.24E-08	0.4306	NA	170756	rs1431196	329443	ieu-b-102	TRUE	reported	WYslv6
18	50835863	T	C	0.0249	0.0043	1.09E-08	0.4307	NA	170756	rs6209799	329443	ieu-b-102	TRUE	reported	WYslv6
18	50836414	A	G	0.0249	0.0043	1.04E-08	0.4307	NA	170756	rs1187302	329443	ieu-b-102	TRUE	reported	WYslv6
18	50840962	T	A	0.0247	0.0043	1.30E-08	0.428	NA	170756	rs1568050	329443	ieu-b-102	TRUE	reported	WYslv6
18	50843233	T	A	0.0247	0.0043	1.22E-08	0.4282	NA	170756	rs6209799	329443	ieu-b-102	TRUE	reported	WYslv6
18	50843269	C	A	0.0247	0.0043	1.22E-08	0.4282	NA	170756	rs6209799	329443	ieu-b-102	TRUE	reported	WYslv6
18	50845830	G	T	0.0248	0.0043	1.11E-08	0.4281	NA	170756	rs1346914	329443	ieu-b-102	TRUE	reported	WYslv6
18	50847784	T	G	0.0247	0.0043	1.20E-08	0.4282	NA	170756	rs1166241	329443	ieu-b-102	TRUE	reported	WYslv6
18	50850307	C	T	0.0247	0.0043	1.33E-08	0.4281	NA	170756	rs2047914	329443	ieu-b-102	TRUE	reported	WYslv6
18	50851107	A	G	0.0248	0.0043	1.23E-08	0.4277	NA	170756	rs2043145	329443	ieu-b-102	TRUE	reported	WYslv6
18	50854411	G	A	0.0247	0.0043	1.19E-08	0.4276	NA	170756	rs1979914	329443	ieu-b-102	TRUE	reported	WYslv6
18	50857663	T	C	0.0247	0.0043	1.28E-08	0.4279	NA	170756	rs1750344	329443	ieu-b-102	TRUE	reported	WYslv6
18	50858019	T	C	0.0248	0.0043	1.11E-08	0.4279	NA	170756	rs1504746	329443	ieu-b-102	TRUE	reported	WYslv6
18	50861409	T	C	0.0253	0.0043	4.35E-09	0.5148	NA	170756	rs1367635	329443	ieu-b-102	TRUE	reported	WYslv6
18	50862311	C	T	0.0247	0.0043	1.22E-08	0.4277	NA	170756	rs6209801	329443	ieu-b-102	TRUE	reported	WYslv6
18	50863861	G	A	0.0251	0.0045	2.65E-08	0.3659	NA	170756	rs1296354	329443	ieu-b-102	TRUE	reported	WYslv6
18	50869085	T	C	0.0248	0.0043	1.13E-08	0.4277	NA	170756	rs6209801	329443	ieu-b-102	TRUE	reported	WYslv6
18	50870391	T	A	0.0249	0.0043	6.52E-09	0.5162	NA	170756	rs1322505	329443	ieu-b-102	TRUE	reported	WYslv6
18	50872623	T	C	0.0247	0.0043	1.19E-08	0.4281	NA	170756	rs1166432	329443	ieu-b-102	TRUE	reported	WYslv6
18	50875225	A	C	0.0244	0.0043	1.83E-08	0.4275	NA	170756	rs6209801	329443	ieu-b-102	TRUE	reported	WYslv6
18	50879700	T	A	0.0242	0.0043	1.93E-08	0.5203	NA	170756	rs7237127	329443	ieu-b-102	TRUE	reported	WYslv6
18	50882781	C	T	0.0244	0.0043	1.76E-08	0.4284	NA	170756	rs6209802	329443	ieu-b-102	TRUE	reported	WYslv6
18	50884529	C	A	0.0244	0.0043	1.87E-08	0.4276	NA	170756	rs4405620	329443	ieu-b-102	TRUE	reported	WYslv6
18	50886142	A	C	0.0245	0.0043	1.55E-08	0.4277	NA	170756	rs1741263	329443	ieu-b-102	TRUE	reported	WYslv6
18	50886590	A	T	0.0245	0.0043	1.65E-08	0.4277	NA	170756	rs1449890	329443	ieu-b-102	TRUE	reported	WYslv6
18	50887961	G	A	0.0239	0.0043	3.52E-08	0.4331	NA	170756	rs1050297	329443	ieu-b-102	TRUE	reported	WYslv6
18	50888351	T	C	0.0243	0.0043	1.99E-08	0.4278	NA	170756	rs1741278	329443	ieu-b-102	TRUE	reported	WYslv6
18	50888891	T	C	0.0244	0.0043	1.90E-08	0.4278	NA	170756	rs6209802	329443	ieu-b-102	TRUE	reported	WYslv6
18	50889558	G	A	0.0243	0.0043	2.10E-08	0.4278	NA	170756	rs1504744	329443	ieu-b-102	TRUE	reported	WYslv6
18	50894002	A	C	0.0243	0.0043	2.09E-08	0.4278	NA	170756	rs6209802	329443	ieu-b-102	TRUE	reported	WYslv6
18	50895766	G	T	0.0242	0.0043	2.58E-08	0.4278	NA	170756	rs7227402	329443	ieu-b-102	TRUE	reported	WYslv6
18	50902560	G	A	0.0243	0.0043	2.14E-08	0.4269	NA	170756	rs6209804	329443	ieu-b-102	TRUE	reported	WYslv6
18	50902578	A	G	0.0243	0.0043	2.15E-08	0.4269	NA	170756	rs6209804	329443	ieu-b-102	TRUE	reported	WYslv6
18	50902747	G	C	0.0243	0.0043	2.06E-08	0.4277	NA	170756	rs1240350	329443	ieu-b-102	TRUE	reported	WYslv6
18	50902773	T	C	0.0243	0.0043	2.06E-08	0.4277	NA	170756	rs6209804	329443	ieu-b-102	TRUE	reported	WYslv6
18	50903050	C	T	0.0243	0.0043	2.00E-08	0.4277	NA	170756	rs1750437	329443	ieu-b-102	TRUE	reported	WYslv6
18	50904361	G	A	0.0243	0.0043	2.05E-08	0.4276	NA	170756	rs6209804	329443	ieu-b-102	TRUE	reported	WYslv6
18	50904562	C	G	0.0243	0.0043	2.07E-08	0.4276	NA	170756	rs6209804	329443	ieu-b-102	TRUE	reported	WYslv6
18	50904666	G	A	0.0243	0.0043	1.99E-08	0.4276	NA	170756	rs6209804	329443	ieu-b-102	TRUE	reported	WYslv6
18	50907127	A	G	0.0247	0.0044	1.34E-08	0.4272	NA	170756	rs1166524	329443	ieu-b-102	TRUE	reported	WYslv6
18	52472408	A	G	-0.0286	0.0051	2.36E-08	0.2348	NA	170756	rs3550954	329443	ieu-b-102	TRUE	reported	WYslv6
18	52472982	G	A	-0.0285	0.0051	2.48E-08	0.2348	NA	170756	rs1297042	329443	ieu-b-102	TRUE	reported	WYslv6
18	52473167	G	A	-0.0285	0.0051	2.44E-08	0.2348	NA	170756	rs1263677	329443	ieu-b-102	TRUE	reported	WYslv6
18	52473793	A	G	-0.0287	0.0051	2.03E-08	0.2348	NA	170756	rs2311117	329443	ieu-b-102	TRUE	reported	WYslv6
18	52474170	A	G	-0.0291	0.0051	1.33E-08	0.2353	NA	170756	rs8093506	329443	ieu-b-102	TRUE	reported	WYslv6
18	52475051	G	A	-0.0286	0.0051	2.31E-08	0.2347	NA	170756	rs7236320	329443	ieu-b-102	TRUE	reported	WYslv6
18	52475073	G	A	-0.0286	0.0051	2.13E-08	0.2347	NA	170756	rs7802158	329443	ieu-b-102	TRUE	reported	WYslv6
18	52475162	A	G	-0.0285	0.0051	2.37E-08	0.2347	NA	170756	rs7243785	329443	ieu-b-102	TRUE	reported	WYslv6
18	52475860	C	G	0.028	0.0051	4.68E-08	0.7655	NA	170756	rs6566870	329443	ieu-b-102	TRUE	reported	WYslv6
18	52475904	T	C	0.028	0.0051	4.60E-08	0.7655	NA	170756	rs6566871	329443	ieu-b-102	TRUE	reported	WYslv6
18	52476469	A	G	-0.0287	0.0051	1.96E-08	0.2346	NA	170756	rs1115200	329443	ieu-b-102	TRUE	reported	WYslv6
18	52476797	A	G	-0.0287	0.0051	1.98E-08	0.2346	NA	170756	rs1245710	329443	ieu-b-102	TRUE	reported	WYslv6
18	52476870	T	A	-0.0287	0.0051	2.10E-08	0.2339	NA	170756	rs1245776	329443	ieu-b-102	TRUE	reported	WYslv6
18	52478970	A	G	-0.0287	0.0051	2.07E-08	0.2346	NA	170756	rs1296830	329443	ieu-b-102	TRUE	reported	WYslv6
18	52479039	G	A	-0.0285	0.0051	2.50E-08	0.2347	NA	170756	rs1296508	329443	ieu-b-102	TRUE	reported	WYslv6
18	52479487	A	G	-0.0285	0.0051	2.56E-08	0.2347	NA	170756	rs8092503	329443	ieu-b-102	TRUE	reported	WYslv6
18	52479764	G	A	-0.0285	0.0051	2.50E-08	0.2347	NA	170756	rs8089801	329443	ieu-b-102	TRUE	reported	WYslv6
18	52480578	G	A	-0.0285	0.0051	2.50E-08	0.2347	NA	170756	rs1115200	329443	ieu-b-102	TRUE	reported	WYslv6
18	52480795	A	G	-0.0282	0.0051	3.29E-08	0.2361	NA	170756	rs2009198	329443	ieu-b-102	TRUE	reported	WYslv6
18	52481402	C	G	-0.0285	0.0051	2.57E-08	0.2347	NA	170756	rs1296654	329443	ieu-b-102	TRUE	reported	WYslv6
18	52481438	T	G	-0.0284	0.0051	2.64E-08	0.2347	NA	170756	rs1296025	329443	ieu-b-102	TRUE	reported	WYslv6
18	52481583	A	G	0.028	0.0051	4.30E-08	0.7651	NA	170756	rs1033827	329443	ieu-b-102	TRUE	reported	WYslv6
18	52481635	G	A	-0.0284	0.0051	2.79E-08	0.2347	NA	170756	rs1297068	329443	ieu-b-102	TRUE	reported	WYslv6
18	52481750	A	T	-0.0285	0.0051	2.51E-08	0.2348	NA	170756	rs1291981	329443	ieu-b-102	TRUE	reported	WYslv6
18	52484299	T	A	-0.0284	0.0051	2.57E-08	0.2343	NA	170756	rs1295716	329443	ieu-b-102	TRUE	reported	WYslv6
18	52486080	C	T	-0.028	0.0051	3.51E-08	0.2347	NA	170756	rs1965662	329443	ieu-b-102	TRUE	reported	WYslv6
18	52488672	C	T	-0.028	0.0051	3.98E-08	0.2326	NA	170756	rs6209146	329443	ieu-b-102	TRUE	reported	WYslv6
18	52488698	T	C	-0.028	0.0051	3.86E-08	0.2343	NA	170756	rs6209146	329443	ieu-b-102	TRUE	reported	WYslv6
18	52489218	A	G	-0.028	0.0051	3.45E-08	0.2347	NA	170756	rs1296916	329443	ieu-b-102	TRUE	reported	WYslv6
18	52491427	T	G	-0.0281	0.0051	3.33E-08	0.2347	NA	170756	rs2871673	329443	ieu-b-102	TRUE	reported	WYslv6
18	52492252	T	C	-0.0279	0.0051	4.08E-08	0.2346	NA	170756	rs1260623	329443	ieu-b-102	TRUE	reported	WYslv6
18	52492470	T	C	-0.028	0.0051	3.41E-08	0.2347	NA	170756	rs1260626	329443	ieu-b-102	TRUE	reported	WYslv6
18	52492579	C	T	-0.028	0.0051	3.86E-08	0.234	NA	170756	rs1295983	329443	ieu-b-102	TRUE	reported	WYslv6
18	52492951	C	G	-0.0281	0.0051	3.42E-08	0.2339	NA	170756	rs1296046	329443	ieu-b-102	TRUE	reported	WYslv6
18	52493622	A	G	-0.028	0.0051	3.48E-08	0.2347	NA	170756	rs1819886	329443	ieu-b-102	TRUE	reported	WYslv6
18	52494397	G	T	-0.0279	0.0051	4.96E-08	0.2338	NA	170756	rs3764515	329443	ieu-b-102	TRUE	reported	WYslv6
18	52495089	A	G	-0.028	0.0051	3.56E-08	0.2347	NA	170756	rs3764513	329443	ieu-b-102	TRUE	reported	WYslv6
18	52495324	G	T	-0.0282	0.0051	2.84E-08	0.2339	NA	170756	rs3764512	329443	ieu-b-102	TRUE	reported	WYslv6
18	52495506	A	G	-0.0281	0.0051	3.20E-08	0.2348	NA	170756	rs1296411	329443	ieu-b-102	TRUE	reported	WYslv6
18	52497204	A	G	-0.028	0.0051	3.65E-08	0.2347	NA	170756	rs1245433	329443	ieu-b-102	TRUE	reported	WYslv6
18	52498290	C	G	-0.028	0.0051	3.43E-08	0.2347	NA	170756	rs3541316	329443	ieu-b-102	TRUE	reported	WYslv6
18	52498801	G	C	-0.028	0.0051	3.43E-08	0.2347	NA	170756	rs1833281	329443	ieu-b-102	TRUE	reported	WYslv6
18	52499160	C	T	-0.0281	0.0051	3.35E-08	0.2347	NA	170756	rs1245600	329443	ieu-b-102	TRUE	reported	WYslv6

18	52499448	C	T	-0.0281	0.0051	3.26E-08	0.2347	NA	170756	rs1245606	329443	ieu-b-102	TRUE	reported	WYslv6
18	52499626	T	C	-0.0281	0.0051	3.02E-08	0.2347	NA	170756	rs8796945	329443	ieu-b-102	TRUE	reported	WYslv6
18	52499801	T	G	-0.0281	0.0051	3.27E-08	0.2347	NA	170756	rs1245776	329443	ieu-b-102	TRUE	reported	WYslv6
18	52500011	G	T	-0.0281	0.0051	3.35E-08	0.2339	NA	170756	rs1245420	329443	ieu-b-102	TRUE	reported	WYslv6
18	52500292	C	T	-0.0281	0.0051	3.28E-08	0.2346	NA	170756	rs1245673	329443	ieu-b-102	TRUE	reported	WYslv6
18	52501012	A	G	-0.028	0.0051	3.87E-08	0.2347	NA	170756	rs1833282	329443	ieu-b-102	TRUE	reported	WYslv6
18	52579200	A	G	-0.0337	0.0061	2.63E-08	0.158	NA	170756	rs1296159	329443	ieu-b-102	TRUE	reported	WYslv6
18	52747689	T	C	-0.0268	0.0044	8.42E-10	0.4076	NA	170756	rs4128242	329443	ieu-b-102	TRUE	reported	WYslv6
18	52747871	C	T	-0.0269	0.0044	8.06E-10	0.4075	NA	170756	rs4131791	329443	ieu-b-102	TRUE	reported	WYslv6
18	52749067	A	G	-0.034	0.0056	9.94E-10	0.1817	NA	170756	rs4598994	329443	ieu-b-102	TRUE	reported	WYslv6
18	52750469	A	G	-0.0268	0.0044	9.54E-10	0.4074	NA	170756	rs4309482	329443	ieu-b-102	TRUE	reported	WYslv6
18	52750688	T	G	-0.0267	0.0044	9.80E-10	0.4074	NA	170756	rs1187471	329443	ieu-b-102	TRUE	reported	WYslv6
18	52751297	T	G	-0.0268	0.0044	9.36E-10	0.4074	NA	170756	rs3536090	329443	ieu-b-102	TRUE	reported	WYslv6
18	52751455	G	A	0.0241	0.0044	4.66E-08	0.4062	NA	170756	rs1245822	329443	ieu-b-102	TRUE	reported	WYslv6
18	52751578	G	T	0.0241	0.0044	4.81E-08	0.4063	NA	170756	rs9964176	329443	ieu-b-102	TRUE	reported	WYslv6
18	52751639	G	C	-0.0341	0.0056	9.71E-10	0.1817	NA	170756	rs1296605	329443	ieu-b-102	TRUE	reported	WYslv6
18	52751708	A	G	-0.0268	0.0044	8.50E-10	0.4073	NA	170756	rs1296945	329443	ieu-b-102	TRUE	reported	WYslv6
18	52752017	G	A	-0.0267	0.0044	9.75E-10	0.4073	NA	170756	rs1296654	329443	ieu-b-102	TRUE	reported	WYslv6
18	52752700	C	T	-0.0268	0.0044	9.33E-10	0.4072	NA	170756	rs4801131	329443	ieu-b-102	TRUE	reported	WYslv6
18	52753785	C	A	-0.0339	0.0056	1.18E-09	0.1821	NA	170756	rs1187534	329443	ieu-b-102	TRUE	reported	WYslv6
18	52756171	T	C	-0.0344	0.0055	5.11E-10	0.187	NA	170756	rs1296253	329443	ieu-b-102	TRUE	reported	WYslv6
18	52757346	T	C	-0.0345	0.0055	4.10E-10	0.187	NA	170756	rs9953930	329443	ieu-b-102	TRUE	reported	WYslv6
18	52837403	G	C	0.0242	0.0043	1.91E-08	0.4912	NA	170756	rs8093489	329443	ieu-b-102	TRUE	reported	WYslv6
18	52840925	T	C	0.0241	0.0043	2.15E-08	0.4915	NA	170756	rs4801146	329443	ieu-b-102	TRUE	reported	WYslv6
18	52842438	A	G	0.0241	0.0043	2.13E-08	0.4915	NA	170756	rs1391762	329443	ieu-b-102	TRUE	reported	WYslv6
18	52844074	G	A	0.0241	0.0043	2.22E-08	0.4911	NA	170756	rs9954765	329443	ieu-b-102	TRUE	reported	WYslv6
18	52844766	C	T	0.024	0.0043	2.40E-08	0.4914	NA	170756	rs1166545	329443	ieu-b-102	TRUE	reported	WYslv6
18	52845580	G	A	0.0241	0.0043	2.21E-08	0.4914	NA	170756	rs6209238	329443	ieu-b-102	TRUE	reported	WYslv6
18	52846725	T	G	0.024	0.0043	2.35E-08	0.4914	NA	170756	rs1942569	329443	ieu-b-102	TRUE	reported	WYslv6
18	52849324	A	T	0.024	0.0043	2.39E-08	0.4914	NA	170756	rs1215070	329443	ieu-b-102	TRUE	reported	WYslv6
18	52849561	T	C	0.0241	0.0043	2.33E-08	0.4914	NA	170756	rs1115227	329443	ieu-b-102	TRUE	reported	WYslv6
18	52850811	A	C	0.0239	0.0043	2.68E-08	0.4914	NA	170756	rs9950768	329443	ieu-b-102	TRUE	reported	WYslv6
18	53048678	A	C	0.0248	0.0044	1.69E-08	0.4029	NA	170756	rs1788025	329443	ieu-b-102	TRUE	reported	WYslv6
18	53050058	G	T	0.0294	0.0049	2.04E-09	0.2644	NA	170756	rs1348047	329443	ieu-b-102	TRUE	reported	WYslv6
18	53050523	G	A	0.0294	0.0049	1.89E-09	0.2646	NA	170756	rs1788019	329443	ieu-b-102	TRUE	reported	WYslv6
18	53055212	C	A	0.028	0.0049	1.23E-08	0.2592	NA	170756	rs3794891	329443	ieu-b-102	TRUE	reported	WYslv6
18	53057188	G	A	0.0312	0.0056	2.31E-08	0.1847	NA	170756	rs1115236	329443	ieu-b-102	TRUE	reported	WYslv6
18	53059748	T	C	0.0284	0.0049	8.10E-09	0.2591	NA	170756	rs1260767	329443	ieu-b-102	TRUE	reported	WYslv6
18	53061937	G	C	0.0281	0.0049	1.09E-08	0.2595	NA	170756	rs3794889	329443	ieu-b-102	TRUE	reported	WYslv6
18	53063229	G	C	0.0287	0.0049	5.59E-09	0.2581	NA	170756	rs4801149	329443	ieu-b-102	TRUE	reported	WYslv6
18	53065110	C	T	0.0287	0.0049	4.96E-09	0.2622	NA	170756	rs4801150	329443	ieu-b-102	TRUE	reported	WYslv6
18	53066079	T	A	0.0284	0.0049	7.04E-09	0.2613	NA	170756	rs1020169	329443	ieu-b-102	TRUE	reported	WYslv6
18	53067758	A	G	0.0283	0.0049	8.26E-09	0.2615	NA	170756	rs7238888	329443	ieu-b-102	TRUE	reported	WYslv6
18	53067954	G	A	0.0288	0.0047	7.57E-10	0.3094	NA	170756	rs7235757	329443	ieu-b-102	TRUE	reported	WYslv6
18	53069972	C	T	0.0242	0.0044	3.76E-08	0.3997	NA	170756	rs2958178	329443	ieu-b-102	TRUE	reported	WYslv6
18	53070914	G	A	0.0312	0.0056	1.98E-08	0.187	NA	170756	rs1245339	329443	ieu-b-102	TRUE	reported	WYslv6
18	53072535	G	A	0.0283	0.0049	8.13E-09	0.2616	NA	170756	rs2958165	329443	ieu-b-102	TRUE	reported	WYslv6
18	53074958	G	A	0.0284	0.0049	7.27E-09	0.2617	NA	170756	rs2958175	329443	ieu-b-102	TRUE	reported	WYslv6
18	53077795	C	T	0.0282	0.0048	3.68E-09	0.2822	NA	170756	rs2958186	329443	ieu-b-102	TRUE	reported	WYslv6
18	53079067	A	G	0.0243	0.0044	3.82E-08	0.3962	NA	170756	rs2919446	329443	ieu-b-102	TRUE	reported	WYslv6
18	53080391	T	C	0.0283	0.0049	8.48E-09	0.2617	NA	170756	rs2958161	329443	ieu-b-102	TRUE	reported	WYslv6
18	53083591	G	A	0.0283	0.0048	3.69E-09	0.2815	NA	170756	rs2860511	329443	ieu-b-102	TRUE	reported	WYslv6
18	53083865	T	C	0.0284	0.0047	1.01E-09	0.3106	NA	170756	rs2958162	329443	ieu-b-102	TRUE	reported	WYslv6
18	53084545	G	T	0.0283	0.0049	8.22E-09	0.2617	NA	170756	rs2919450	329443	ieu-b-102	TRUE	reported	WYslv6
18	53086455	C	T	0.0282	0.0049	9.24E-09	0.2616	NA	170756	rs1440477	329443	ieu-b-102	TRUE	reported	WYslv6
18	53087632	C	T	0.0281	0.0049	1.04E-08	0.2624	NA	170756	rs2958166	329443	ieu-b-102	TRUE	reported	WYslv6
18	53089138	C	T	0.0282	0.0048	4.27E-09	0.2814	NA	170756	rs2958169	329443	ieu-b-102	TRUE	reported	WYslv6
18	53089162	C	T	0.0283	0.0049	9.37E-09	0.2612	NA	170756	rs8098843	329443	ieu-b-102	TRUE	reported	WYslv6
18	53092547	T	C	-0.0307	0.0047	9.58E-11	0.7089	NA	170756	rs4374254	329443	ieu-b-102	TRUE	reported	WYslv6
18	53095471	T	C	-0.0241	0.0044	4.53E-08	0.6001	NA	170756	rs9320016	329443	ieu-b-102	TRUE	reported	WYslv6
18	53096708	T	C	0.0284	0.0049	7.94E-09	0.2621	NA	170756	rs4524013	329443	ieu-b-102	TRUE	reported	WYslv6
18	53097544	G	A	-0.0306	0.0047	1.02E-10	0.7081	NA	170756	rs4500831	329443	ieu-b-102	TRUE	reported	WYslv6
18	53098526	T	C	-0.0314	0.0048	4.04E-11	0.7094	NA	170756	rs7229456	329443	ieu-b-102	TRUE	reported	WYslv6
18	53098732	G	A	-0.0317	0.0048	2.48E-11	0.7027	NA	170756	rs7240481	329443	ieu-b-102	TRUE	reported	WYslv6
18	53099012	G	C	-0.0345	0.0047	2.53E-13	0.7012	NA	170756	rs1296714	329443	ieu-b-102	TRUE	reported	WYslv6
18	53099021	C	T	-0.0244	0.0045	4.97E-08	0.6037	NA	170756	rs1296333	329443	ieu-b-102	TRUE	reported	WYslv6
18	53099093	C	T	-0.0314	0.0048	3.93E-11	0.7013	NA	170756	rs1479636	329443	ieu-b-102	TRUE	reported	WYslv6
18	53101598	A	G	-0.0307	0.0046	1.65E-11	0.6675	NA	170756	rs1295804	329443	ieu-b-102	TRUE	reported	WYslv6
18	53101703	T	C	0.0283	0.0049	1.03E-08	0.2592	NA	170756	rs4801153	329443	ieu-b-102	TRUE	reported	WYslv6
18	53101860	C	G	-0.0296	0.0047	3.27E-10	0.6801	NA	170756	rs1296953	329443	ieu-b-102	TRUE	reported	WYslv6
18	53101904	C	T	-0.0272	0.0044	8.20E-10	0.6121	NA	170756	rs1296955	329443	ieu-b-102	TRUE	reported	WYslv6
18	53101984	C	T	0.0294	0.0048	7.41E-10	0.2821	NA	170756	rs4801154	329443	ieu-b-102	TRUE	reported	WYslv6
18	53103739	G	A	-0.0286	0.0044	1.20E-10	0.6189	NA	170756	rs4341827	329443	ieu-b-102	TRUE	reported	WYslv6
18	53104019	C	T	-0.0257	0.0044	7.53E-09	0.6275	NA	170756	rs4468713	329443	ieu-b-102	TRUE	reported	WYslv6
18	53104253	A	T	-0.0253	0.0045	2.52E-08	0.659	NA	170756	rs7228159	329443	ieu-b-102	TRUE	reported	WYslv6
18	53107115	G	A	-0.0285	0.0044	1.07E-10	0.6183	NA	170756	rs7236656	329443	ieu-b-102	TRUE	reported	WYslv6
18	53109035	A	G	0.0314	0.0047	2.14E-11	0.3007	NA	170756	rs7231748	329443	ieu-b-102	TRUE	reported	WYslv6
18	53109202	C	T	0.0314	0.0054	6.44E-09	0.1969	NA	170756	rs1050300	329443	ieu-b-102	TRUE	reported	WYslv6
18	53115879	T	A	-0.0259	0.0044	5.94E-09	0.6272	NA	170756	rs1452789	329443	ieu-b-102	TRUE	reported	WYslv6
18	53117304	A	G	0.0288	0.0048	2.51E-09	0.2732	NA	170756	rs1452788	329443	ieu-b-102	TRUE	reported	WYslv6
18	53121576	G	C	0.0286	0.0048	2.85E-09	0.273	NA	170756	rs1407981	329443	ieu-b-102	TRUE	reported	WYslv6
18	53122431	A	G	0.0276	0.0049	2.36E-08	0.267	NA	170756	rs1882258	329443	ieu-b-102	TRUE	reported	WYslv6
18	53123172	A	G	0.0311	0.0047	2.80E-11	0.3021	NA	170756	rs732779	329443	ieu-b-102	TRUE	reported	WYslv6
18	53125364	T	G	0.0309	0.0047	3.79E-11	0.302	NA	170756	rs9966430	329443	ieu-b-102	TRUE	reported	WYslv6
18	53125435	G	A	-0.0282	0.0043	7.15E-11	0.5426	NA	170756	rs2924321	329443	ieu-b-102	TRUE	reported	WYslv6
18	53131488	C	A	0.0251	0.0044	1.15E-08	0.5924	NA	170756	rs3760600	329443	ieu-b-102	TRUE	reported	WYslv6
18	53133740	T	C	-0.0252	0.0044	1.03E-08	0.4074	NA	170756	rs2924328	329443	ieu-b-102	TRUE	reported	WYslv6
18	53134652	T	C	0.0279	0.0044	2.19E-10	0.6014	NA	170756	rs1377243	329443	ieu-b-102	TRUE	reported	WYslv6
18	53135894	G	A	-0.0341	0.0048	1.23E-12	0.2875	NA	170756	rs2924329	329443	ieu-b-102	TRUE	reported	WYslv6
18	53143573	C	T	0.0236	0.0043	4.19E-08	0.4894	NA	170756	rs7233312	329443	ieu-b-102	TRUE	reported	WYslv6
18	53149021	A	T	0.0281	0.0046	7.77E-10	0.659	NA	170756	rs2958182	329443	ieu-b-102	TRUE	reported	WYslv6
18	53162930	G	A	0.0251	0.0044	8.96E-09	0.5766	NA	170756	rs1371728	329443	ieu-b-102	TRUE	reported	WYslv6
18	53165959	T	C	0.0251	0.0044	8.51E-09	0.5765	NA	170756	rs2958188	329443	ieu-b-102	TRUE	reported	WYslv6
18	53167520	T	C	0.0252	0.0044	7.12E-09	0.5768	NA	170756	rs2060886	329443	ieu-b-102	TRUE	reported	WYslv6
18	53168243	C	T	0.0282	0.0045	4.87E-10	0.6511	NA	170756	rs3017183	329443	ieu-b-102	TRUE	reported	WYslv6
18	53207207	A	G	0.0266	0.0048	3.00E-08	0.2755	NA	170756	rs1452787	329443	ieu-b-102	TRUE	reported	WYslv6
18	53239916	G	A	0.0343	0.0062	2.65E-08	0.1447	NA	170756	rs1321846	329443	ieu-b-102	TRUE	reported	WYslv6
18	77563334	G	A	0.0306	0.0053	8.79E-09	0.2058	NA	170756	rs1166556	329443	ieu-b-102	TRUE	reported	WYslv6
18	77566535	G	C	0.0265	0.0048	3.69E-08	0.2756	NA	170756	rs6650631	329443	ieu-b-102	TRUE	reported	WYslv6
18	77567761	T	G	0.0265	0.0049	4.87E-08	0.2715	NA	170756	rs1568241	329443	ieu-b-102	TRUE	reported	WYslv6
18	77574374	C	T	0.031	0.0053	5.30E-09	0.2065	NA	170756	rs7136754	329443	ieu-b-102	TRUE	reported	WYslv6
18	77575871	A	G	0.0266	0.0048	3.38E-08	0.2752	NA	170756	rs3494522	329443	ieu-b-102	TRUE	reported	WYslv6
18	77576337	G	A	0.031	0.0053	5.27E-09	0.2059	NA	170756	rs7136754	329443	ieu-b-102	TRUE	reported	WYslv6
18	77578191	C	A	0.0268	0.0048	2.72E-08	0.275	NA	170756	rs1166360	329443	ieu-b-102	TRUE	reported	WYslv6
18	77578986	G	A	0.0313	0.0053	4.60E-09	0.2058	NA	170756	rs1166429	329443	ieu-b-102	TRUE	reported	WYslv6
18	77579773	G	A	0.0286	0.0052	3.28E-08	0.2244	NA	170756	rs7236339	329443	ieu-b-102	TRUE	reported	WYslv6
18	77579812	A	G	0.0271	0.0048	2.14E-08	0.2743	NA	170756	rs7238071	329443	ieu-b-102	TRUE	reported	WYslv6

18	77580712	G	A	0.0323	0.0054	2.43E-09	0.2047	NA	170756	rs7241572	329443	ieu-b-102	TRUE	reported	WYslv6
20	44680412	C	T	0.0294	0.0053	2.99E-08	0.2172	NA	170756	rs3746522	329443	ieu-b-102	TRUE	reported	WYslv6
20	44680853	G	A	0.0313	0.005	2.84E-10	0.254	NA	170756	rs1262443	329443	ieu-b-102	TRUE	reported	WYslv6
20	44688665	G	A	0.031	0.0049	2.43E-10	0.2597	NA	170756	rs9074	329443	ieu-b-102	TRUE	reported	WYslv6
20	44692598	C	T	0.031	0.0049	2.40E-10	0.2597	NA	170756	rs1303732	329443	ieu-b-102	TRUE	reported	WYslv6
20	44700166	T	C	-0.0249	0.0043	7.81E-09	0.5244	NA	170756	rs6074012	329443	ieu-b-102	TRUE	reported	WYslv6
20	44702120	T	C	-0.0302	0.0049	6.37E-10	0.7396	NA	170756	rs2425752	329443	ieu-b-102	TRUE	reported	WYslv6
20	44721656	T	C	-0.0306	0.0049	4.19E-10	0.738	NA	170756	rs1555874	329443	ieu-b-102	TRUE	reported	WYslv6
20	44724305	A	G	-0.0309	0.0049	2.81E-10	0.739	NA	170756	rs6131010	329443	ieu-b-102	TRUE	reported	WYslv6
