# Supplementary material for: Risk of major depressive increases with increasing frequency of alcohol drinking: a bidirectional two-sample Mendelian randomization analysis
Source: Front Public Health. 2024 Jun 5;12:1372758. doi: 10.3389/fpubh.2024.1372758 (PMC11186411; doi:10.3389/fpubh.2024.1372758)
Supplement: Supplementary file 3 [file Data_Sheet_2.PDF]

Alcohol consumption SNPs

SNP	effect_allel	other_allel	effect_allel	other_allel	beta.expos	beta.outco	eaf.exposu	eaf.outcom	remove	palindromi	ambiguous	id.outcome	se.outcome	pval.outco	outcome	mr_keep.ou	pval_origin	data_sourc	se.exposure	exposure	mr_keep.ex	pval.exposu	pval_origin	id.exposure	data_sourc	action	mr_keep	samplesize.outcome
rs1194069	G	A	G	A	0.02694	-0.0126	0.6063	0.6002	FALSE	FALSE	FALSE	aW0umH	0.0044	0.004437	major depr	TRUE	reported	textfile	0.003042	alcohol cor	TRUE	8.29E-19	inferred	YCGTCY	textfile	2	TRUE	NA
rs1260326	C	T	C	T	0.02839	-0.0039	0.6066	0.6043	FALSE	FALSE	FALSE	aW0umH	0.0044	0.3688	major depr	TRUE	reported	textfile	0.002973	alcohol cor	TRUE	1.31E-21	inferred	YCGTCY	textfile	2	TRUE	NA
rs9841829	G	T	G	T	0.01869	-0.0122	0.2269	0.228	FALSE	FALSE	FALSE	aW0umH	0.0051	0.01725	major depr	TRUE	reported	textfile	0.002976	alcohol cor	TRUE	3.38E-10	inferred	YCGTCY	textfile	2	TRUE	NA

Alcohol intake frequency SNPs

SNP	effect_allel	other_allel	effect_allel	other_allel	beta.expos	beta.outco	eaf.expos	eaf.outcon	remove	palindrom	ambiguou	id.outcom	se.outcom	pval.outco	outcome	mr_keep.o	pval_origir	data_sourc	se.exposur	exposure	mr_keep.e	pval.expos	pval_origir	id.exposur	data_sourc	action	mr_keep	samplesize.outcome
rs1018831	T	C	T	C	-0.01979	-0.005	0.470852	0.4691	FALSE	FALSE	FALSE	t0TVbk	0.0043	0.2509	major dep	TRUE	reported	textfile	0.003036	alcohol int	TRUE	7.16E-11	inferred	O6xYKo	textfile	2	TRUE	NA
rs1079266	G	A	G	A	0.017432	-0.0019	0.505254	0.5034	FALSE	FALSE	FALSE	t0TVbk	0.0043	0.6638	major dep	TRUE	reported	textfile	0.003041	alcohol int	TRUE	9.86E-09	inferred	O6xYKo	textfile	2	TRUE	NA
rs1103942	T	C	T	C	-0.02356	0.0125	0.454624	0.4537	FALSE	FALSE	FALSE	t0TVbk	0.0043	0.003796	major dep	TRUE	reported	textfile	0.003037	alcohol int	TRUE	8.73E-15	inferred	O6xYKo	textfile	2	TRUE	NA
rs1122361	A	G	A	G	0.025091	8.00E-04	0.206155	0.2068	FALSE	FALSE	FALSE	t0TVbk	0.0053	0.8791	major dep	TRUE	reported	textfile	0.003754	alcohol int	TRUE	2.32E-11	inferred	O6xYKo	textfile	2	TRUE	NA
rs1170085	G	A	G	A	-0.0298	-0.0121	0.093465	0.093	FALSE	FALSE	FALSE	t0TVbk	0.0075	0.1037	major dep	TRUE	reported	textfile	0.005233	alcohol int	TRUE	1.24E-08	inferred	O6xYKo	textfile	2	TRUE	NA
rs1175077	A	G	A	G	-0.02049	-0.0053	0.209454	0.2062	FALSE	FALSE	FALSE	t0TVbk	0.0053	0.3241	major dep	TRUE	reported	textfile	0.003726	alcohol int	TRUE	3.80E-08	inferred	O6xYKo	textfile	2	TRUE	NA
rs1178721	T	C	T	C	0.024416	5.00E-04	0.369127	0.3652	FALSE	FALSE	FALSE	t0TVbk	0.0046	0.9071	major dep	TRUE	reported	textfile	0.003201	alcohol int	TRUE	2.38E-14	inferred	O6xYKo	textfile	2	TRUE	NA
rs1194069	G	A	G	A	-0.04371	-0.0126	0.604193	0.6002	FALSE	FALSE	FALSE	t0TVbk	0.0044	0.004437	major dep	TRUE	reported	textfile	0.003116	alcohol int	TRUE	1.04E-44	inferred	O6xYKo	textfile	2	TRUE	NA
rs1228589	A	G	A	G	0.02107	0.0061	0.246133	0.2453	FALSE	FALSE	FALSE	t0TVbk	0.005	0.2251	major dep	TRUE	reported	textfile	0.003528	alcohol int	TRUE	2.34E-09	inferred	O6xYKo	textfile	2	TRUE	NA
rs1310297	C	T	C	T	-0.01941	-0.0097	0.61881	0.6195	FALSE	FALSE	FALSE	t0TVbk	0.0044	0.02836	major dep	TRUE	reported	textfile	0.003119	alcohol int	TRUE	4.89E-10	inferred	O6xYKo	textfile	2	TRUE	NA
rs1317844	T	C	T	C	-0.01865	0.006	0.276349	0.2765	FALSE	FALSE	FALSE	t0TVbk	0.0048	0.2173	major dep	TRUE	reported	textfile	0.00339	alcohol int	TRUE	3.75E-08	inferred	O6xYKo	textfile	2	TRUE	NA
rs1339001	C	T	C	T	0.029612	0.021	0.134041	0.1315	FALSE	FALSE	FALSE	t0TVbk	0.0065	0.001317	major dep	TRUE	reported	textfile	0.004492	alcohol int	TRUE	4.33E-11	inferred	O6xYKo	textfile	2	TRUE	NA
rs1421085	C	T	C	T	0.019939	0.0102	0.403447	0.4057	FALSE	FALSE	FALSE	t0TVbk	0.0044	0.02009	major dep	TRUE	reported	textfile	0.003085	alcohol int	TRUE	1.02E-10	inferred	O6xYKo	textfile	2	TRUE	NA
rs1666658	C	T	C	T	0.017967	-0.0024	0.392206	0.3945	FALSE	FALSE	FALSE	t0TVbk	0.0044	0.589	major dep	TRUE	reported	textfile	0.003099	alcohol int	TRUE	6.69E-09	inferred	O6xYKo	textfile	2	TRUE	NA
rs1766275	C	T	C	T	0.030135	-0.003	0.089115	0.0865	FALSE	FALSE	FALSE	t0TVbk	0.008	0.7059	major dep	TRUE	reported	textfile	0.00546	alcohol int	TRUE	3.41E-08	inferred	O6xYKo	textfile	2	TRUE	NA
rs186347	T	G	T	G	0.017949	-0.0031	0.463343	0.4631	FALSE	FALSE	FALSE	t0TVbk	0.0043	0.4686	major dep	TRUE	reported	textfile	0.003051	alcohol int	TRUE	4.02E-09	inferred	O6xYKo	textfile	2	TRUE	NA
rs1937522	G	A	G	A	0.016898	0.004	0.528054	0.5275	FALSE	FALSE	FALSE	t0TVbk	0.0043	0.3532	major dep	TRUE	reported	textfile	0.003032	alcohol int	TRUE	2.50E-08	inferred	O6xYKo	textfile	2	TRUE	NA
rs1991083	T	C	T	C	-0.02239	0.003	0.679886	0.6823	FALSE	FALSE	FALSE	t0TVbk	0.0047	0.513	major dep	TRUE	reported	textfile	0.003258	alcohol int	TRUE	6.30E-12	inferred	O6xYKo	textfile	2	TRUE	NA
rs2043677	T	C	T	C	0.026113	0.0045	0.145599	0.1476	FALSE	FALSE	FALSE	t0TVbk	0.0061	0.4635	major dep	TRUE	reported	textfile	0.004327	alcohol int	TRUE	1.59E-09	inferred	O6xYKo	textfile	2	TRUE	NA
rs2159935	A	G	A	G	-0.01857	-0.0013	0.490369	0.4888	FALSE	FALSE	FALSE	t0TVbk	0.0043	0.7597	major dep	TRUE	reported	textfile	0.003026	alcohol int	TRUE	8.33E-10	inferred	O6xYKo	textfile	2	TRUE	NA
rs2160935	T	C	T	C	-0.01872	-0.0056	0.604293	0.6038	FALSE	FALSE	FALSE	t0TVbk	0.0044	0.2005	major dep	TRUE	reported	textfile	0.003091	alcohol int	TRUE	1.40E-09	inferred	O6xYKo	textfile	2	TRUE	NA
rs2244598	C	T	C	T	-0.01838	#######	0.605114	0.6087	FALSE	FALSE	FALSE	t0TVbk	0.0045	0.9296	major dep	TRUE	reported	textfile	0.003119	alcohol int	TRUE	3.81E-09	inferred	O6xYKo	textfile	2	TRUE	NA
rs2411453	G	T	G	T	-0.03508	3.00E-04	0.597353	0.5973	FALSE	FALSE	FALSE	t0TVbk	0.0044	0.952	major dep	TRUE	reported	textfile	0.00309	alcohol int	TRUE	7.32E-30	inferred	O6xYKo	textfile	2	TRUE	NA
rs2622167	A	G	A	G	-0.01912	-0.0045	0.428653	0.4261	FALSE	FALSE	FALSE	t0TVbk	0.0044	0.3045	major dep	TRUE	reported	textfile	0.003067	alcohol int	TRUE	4.61E-10	inferred	O6xYKo	textfile	2	TRUE	NA
rs262240	T	C	T	C	-0.01721	-0.0127	0.468553	0.4667	FALSE	FALSE	FALSE	t0TVbk	0.0043	0.003212	major dep	TRUE	reported	textfile	0.003035	alcohol int	TRUE	1.43E-08	inferred	O6xYKo	textfile	2	TRUE	NA
rs2717063	A	C	A	C	-0.02037	0.0111	0.585731	0.5863	FALSE	FALSE	FALSE	t0TVbk	0.0044	0.0118	major dep	TRUE	reported	textfile	0.003085	alcohol int	TRUE	4.00E-11	inferred	O6xYKo	textfile	2	TRUE	NA
rs3444085	T	C	T	C	-0.02268	-0.0087	0.157151	0.1572	FALSE	FALSE	FALSE	t0TVbk	0.0059	0.14	major dep	TRUE	reported	textfile	0.004151	alcohol int	TRUE	4.63E-08	inferred	O6xYKo	textfile	2	TRUE	NA
rs3447388	A	G	A	G	-0.02036	-0.0077	0.24819	0.2463	FALSE	FALSE	FALSE	t0TVbk	0.005	0.1239	major dep	TRUE	reported	textfile	0.003503	alcohol int	TRUE	6.18E-09	inferred	O6xYKo	textfile	2	TRUE	NA
rs3463102	T	C	T	C	-0.01691	0.0056	0.446061	0.4447	FALSE	FALSE	FALSE	t0TVbk	0.0043	0.1926	major dep	TRUE	reported	textfile	0.003048	alcohol int	TRUE	2.89E-08	inferred	O6xYKo	textfile	2	TRUE	NA
rs3510514	T	C	T	C	0.026345	-0.0135	0.401541	0.4002	FALSE	FALSE	FALSE	t0TVbk	0.0044	0.002164	major dep	TRUE	reported	textfile	0.003088	alcohol int	TRUE	1.44E-17	inferred	O6xYKo	textfile	2	TRUE	NA
rs362307	T	C	T	C	0.043305	0.0218	0.074582	0.0751	FALSE	FALSE	FALSE	t0TVbk	0.0086	0.01094	major dep	TRUE	reported	textfile	0.005802	alcohol int	TRUE	8.42E-14	inferred	O6xYKo	textfile	2	TRUE	NA
rs4241258	T	C	T	C	0.025064	0.0047	0.13763	0.1374	FALSE	FALSE	FALSE	t0TVbk	0.0063	0.4553	major dep	TRUE	reported	textfile	0.004403	alcohol int	TRUE	1.26E-08	inferred	O6xYKo	textfile	2	TRUE	NA
rs4242715	A	G	A	G	-0.01865	-0.0153	0.680585	0.6783	FALSE	FALSE	FALSE	t0TVbk	0.0047	0.001134	major dep	TRUE	reported	textfile	0.003248	alcohol int	TRUE	9.31E-09	inferred	O6xYKo	textfile	2	TRUE	NA
rs4417025	A	G	A	G	-0.01884	0.0066	0.361153	0.3579	FALSE	FALSE	FALSE	t0TVbk	0.0045	0.1428	major dep	TRUE	reported	textfile	0.003165	alcohol int	TRUE	2.65E-09	inferred	O6xYKo	textfile	2	TRUE	NA
rs4503294	T	C	T	C	0.018148	-0.0067	0.565333	0.5661	FALSE	FALSE	FALSE	t0TVbk	0.0045	0.1384	major dep	TRUE	reported	textfile	0.00307	alcohol int	TRUE	3.41E-09	inferred	O6xYKo	textfile	2	TRUE	NA
rs461599	C	A	C	A	-0.01919	-0.0121	0.462259	0.4627	FALSE	FALSE	FALSE	t0TVbk	0.0043	0.005163	major dep	TRUE	reported	textfile	0.00304	alcohol int	TRUE	2.74E-10	inferred	O6xYKo	textfile	2	TRUE	NA
rs4800487	G	A	G	A	-0.02894	0.0061	0.456873	0.4539	FALSE	FALSE	FALSE	t0TVbk	0.0043	0.1621	major dep	TRUE	reported	textfile	0.003047	alcohol int	TRUE	2.16E-21	inferred	O6xYKo	textfile	2	TRUE	NA
rs489062	A	G	A	G	0.01665	0.0018	0.437454	0.438	FALSE	FALSE	FALSE	t0TVbk	0.0043	0.674099	major dep	TRUE	reported	textfile	0.003053	alcohol int	TRUE	4.93E-08	inferred	O6xYKo	textfile	2	TRUE	NA
rs4968391	T	G	T	G	-0.01927	-0.0027	0.674892	0.6714	FALSE	FALSE	FALSE	t0TVbk	0.0046	0.5494	major dep	TRUE	reported	textfile	0.003227	alcohol int	TRUE	2.34E-09	inferred	O6xYKo	textfile	2	TRUE	NA
rs550942	T	C	T	C	0.022401	0.0189	0.823865	0.8222	FALSE	FALSE	FALSE	t0TVbk	0.0057	0.000906	major dep	TRUE	reported	textfile	0.003989	alcohol int	TRUE	1.96E-08	inferred	O6xYKo	textfile	2	TRUE	NA
rs5619443	T	C	T	C	0.02254	0.0107	0.16931	0.168	FALSE	FALSE	FALSE	t0TVbk	0.0058	0.06724	major dep	TRUE	reported	textfile	0.004071	alcohol int	TRUE	3.09E-08	inferred	O6xYKo	textfile	2	TRUE	NA
rs5890541	A	G	A	G	-0.02663	-0.002	0.410052	0.4071	FALSE	FALSE	FALSE	t0TVbk	0.0044	0.641201	major dep	TRUE	reported	textfile	0.003078	alcohol int	TRUE	5.07E-18	inferred	O6xYKo	textfile	2	TRUE	NA
rs6187351	T	G	T	G	0.020374	0.017	0.32785	0.3297	FALSE	FALSE	FALSE	t0TVbk	0.0047	0.000323	major dep	TRUE	reported	textfile	0.003303	alcohol int	TRUE	6.91E-10	inferred	O6xYKo	textfile	2	TRUE	NA
rs6230578	G	C	G	C	-0.04852	0.0068	0.102253	0.1037	FALSE	TRUE	FALSE	t0TVbk	0.0072	0.3483	major dep	TRUE	reported	textfile	0.005066	alcohol int	TRUE	9.88E-22	inferred	O6xYKo	textfile	2	TRUE	NA
rs6233967	A	C	A	C	0.018294	0.0014	0.626705	0.6274	FALSE	FALSE	FALSE	t0TVbk	0.0045	0.7613	major dep	TRUE	reported	textfile	0.003154	alcohol int	TRUE	6.62E-09	inferred	O6xYKo	textfile	2	TRUE	NA
rs6246631	T	C	T	C	-0.02549	0.0054	0.202827	0.2014	FALSE	FALSE	FALSE	t0TVbk	0.0054	0.3123	major dep	TRUE	reported	textfile	0.003774	alcohol int	TRUE	1.44E-11	inferred	O6xYKo	textfile	2	TRUE	NA
rs650558	T	C	T	C	0.020736	-0.001	0.247918	0.2459	FALSE	FALSE	FALSE	t0TVbk	0.005	0.834	major dep	TRUE	reported	textfile	0.003508	alcohol int	TRUE	3.39E-09	inferred	O6xYKo	textfile	2	TRUE	NA
rs6727281	T	C	T	C	-0.02432	0.0055	0.184023	0.186	FALSE	FALSE	FALSE	t0TVbk	0.0056	0.322	major dep	TRUE	reported	textfile	0.00392	alcohol int	TRUE	5.46E-10	inferred	O6xYKo	textfile	2	TRUE	NA
rs6943160	C	T	C	T	0.020627	0.001	0.208646	0.207	FALSE	FALSE	FALSE	t0TVbk	0.0053	0.8494	major dep	TRUE	reported	textfile	0.003728	alcohol int	TRUE	3.14E-08	inferred	O6xYKo	textfile	2	TRUE	NA
rs7165168	T	C	T	C	-0.07046	0.0123	0.0142	0.0143	FALSE	FALSE	FALSE	t0TVbk	0.0182	0.500299	major dep	TRUE	reported	textfile	0.012791	alcohol int	TRUE	3.62E-08	inferred	O6xYKo	textfile	2	TRUE	NA
rs7276922	T	A	T	A	-0.02314	-0.01	0.154942	0.1547	FALSE	TRUE	FALSE	t0TVbk	0.006	0.092259	major dep	TRUE	reported	textfile	0.004192	alcohol int	TRUE	3.40E-08	inferred	O6xYKo	textfile	2	TRUE	NA
rs7278706	A	G	A	G	-0.02819	0.0043	0.162767	0.1617	FALSE	FALSE	FALSE	t0TVbk	0.0058	0.4585	major dep	TRUE	reported	textfile	0.004103	alcohol int	TRUE	6.36E-12	inferred	O6xYKo	textfile	2	TRUE	NA
rs728538	G	T	G	T	0.022875	0.0169	0.168868	0.1694	FALSE	FALSE	FALSE	t0TVbk	0.0058	0.003687	major dep	TRUE	reported	textfile	0.004063	alcohol int	TRUE	1.79E-08	inferred	O6xYKo	textfile	2	TRUE	NA
rs7298932	G	A	G	A	-0.02372	0.0031	0.147849	0.148	FALSE	FALSE	FALSE	t0TVbk	0.0061	0.6128	major dep	TRUE	reported	textfile	0.004312	alcohol int	TRUE	3.76E-08	inferred	O6xYKo	textfile	2	TRUE	NA
rs7330939	T	C	T	C	-0.02133	-0.0028	0.720352	0.7186	FALSE	FALSE	FALSE	t0TVbk	0.0048	0.5583	major dep	TRUE	reported	textfile	0.003405	alcohol int	TRUE	3.73E-10	inferred	O6xYKo	textfile	2	TRUE	NA
rs7460106	C	T	C	T	-0.02171	-0.0078	0.23562	0.2391	FALSE	FALSE	FALSE	t0TVbk	0.0052	0.1351	major dep	TRUE	reported	textfile	0.003619	alcohol int	TRUE	1.99E-09	inferred	O6xYKo	textfile	2	TRUE	NA
rs7467914	C	T	C	T	-0.03207	0.0051	0.074515	0.0743	FALSE	FALSE	FALSE	t0TVbk	0.0082	0.5337	major dep	TRUE	reported	textfile	0.005758	alcohol int	TRUE	2.54E-08	inferred	O6xYKo	textfile	2	TRUE	NA
rs7514579	C	A	C	A	0.019667	-0.0052	0.232457	0.2313	FALSE	FALSE	FALSE	t0TVbk	0.0053	0.328	major dep	TRUE	reported	textfile	0.003598	alcohol int	TRUE	4.59E-08	inferred	O6xYKo	textfile	2	TRUE	NA
rs7608265	T	C	T	C	0.046427	-0.0129	0.054327	0.0526	FALSE	FALSE	FALSE	t0TVbk	0.0101	0.2008	major dep	TRUE	reported	textfile	0.006687	alcohol int	TRUE	3.83E-12	inferred	O6xYKo	textfile	2	TRUE	NA
rs780569	A	T	A	T	0.019803	0.0032	0.70882	0.7121	FALSE	TRUE	FALSE	t0TVbk	0.0048	0.499501	major dep	TRUE	reported	textfile	0.003365	alcohol int	TRUE	3.96E-09	inferred	O6xYKo	textfile	2	TRUE	NA
rs8029231	C	T	C	T	-0.03937	4.00E-04	0.057704	0.0591	FALSE	FALSE	FALSE	t0TVbk	0.0091	0.9679	major dep	TRUE	reported	textfile	0.006496	alcohol int	TRUE	1.35E-09	inferred	O6xYKo	textfile	2	TRUE	NA
rs8043563	C	G	C	G	0.023365	0.0058	0.737192	0.7391	FALSE	TRUE	FALSE	t0TVbk	0.005	0.245	major dep	TRUE	reported	textfile	0.003471	alcohol int	TRUE	1.68E-11	inferred	O6xYKo	textfile	2	TRUE	NA
rs8614	A	C	A	C	0.024781	#######	0.182509	0.1826	FALSE	FALSE	FALSE	t0TVbk	0.0056	0.9118	major dep	TRUE	reported	textfile	0.003925	alcohol int	TRUE	2.74E-10	inferred	O6xYKo	textfile	2	TRUE	NA
rs9403297	A	G	A	G	0.018823	0.0035	0.372967	0.3732	FALSE	FALSE	FALSE	t0TVbk	0.0044	0.4267	major dep	TRUE	reported	textfile	0.00313	alcohol int	TRUE	1.82E-09	inferred	O6xYKo	textfile	2	TRUE	NA
rs9829192	T	G	T	G	0.016932	0.0029	0.435133	0.4336	FALSE	FALSE	FALSE	t0TVbk	0.0043	0.5093	major dep	TRUE	reported	textfile	0.00305	alcohol int	TRUE	2.84E-08	inferred	O6xYKo	textfile	2	TRUE	NA
rs9906502	A	G	A	G	0.023788	-0.0125	0.176998	0.1772	FALSE	FALSE	FALSE	t0TVbk	0.0057	0.02692	major dep	TRUE	reported	textfile	0.003962	alcohol int	TRUE	1.92E-09	inferred	O6xYKo	textfile	2	TRUE	NA
