# Supplementary material for: Risk of major depressive increases with increasing frequency of alcohol drinking: a bidirectional two-sample Mendelian randomization analysis
Source: Front Public Health. 2024 Jun 5;12:1372758. doi: 10.3389/fpubh.2024.1372758 (PMC11186411; doi:10.3389/fpubh.2024.1372758)
Supplement: Supplementary file 4 [file Data_Sheet_3.PDF]

Cochran Q

id.exposur	id.outcom	outcome	exposure	method	Q	Q_df	Q_pval
YCGTCY	aW0umH	major dep	alcohol co	Inverse var	3.631084	2	0.16275

Egger

id.exposur	id.outcome	outcome	exposure	egger_inte	se	pval
YCGTCY	aW0umH	major dep	alcohol co	-0.02372	0.021518	0.469018
