# Supplementary material for: Risk of major depressive increases with increasing frequency of alcohol drinking: a bidirectional two-sample Mendelian randomization analysis
Source: Front Public Health. 2024 Jun 5;12:1372758. doi: 10.3389/fpubh.2024.1372758 (PMC11186411; doi:10.3389/fpubh.2024.1372758)
Supplement: Supplementary file 5 [file Data_Sheet_4.PDF]

Egger

id.exposur	id.outcom	outcome	exposure	egger_inte	se	pval
O6xYKo	t0TVbk	major dep	alcohol int	0.002672	0.003643	0.465943

Cochran Q

id.exposur	id.outcome	outcome	exposure	method	Q	Q_df	Q_pval
O6xYKo	t0TVbk	major dep	alcohol int	Inverse var	160.7895	65	4.41E-10
