# Supplementary material for: Risk of major depressive increases with increasing frequency of alcohol drinking: a bidirectional two-sample Mendelian randomization analysis
Source: Front Public Health. 2024 Jun 5;12:1372758. doi: 10.3389/fpubh.2024.1372758 (PMC11186411; doi:10.3389/fpubh.2024.1372758)
Supplement: Supplementary file 6 [file Data_Sheet_5.PDF]

White wine/champange intake SNPs

chr.exposu	pos.exposu	other_allel	effect_allel	beta.expos	se.exposur	pval.expos	eaf.exposu	samplesize	ncase.expo	SNP	ncontrol.e	exposure	mr_keep.e	pval_origir	id.exposure
1	19163656	G	A	0.043816	0.008848	7.30E-07	0.127848	64949	NA	rs4920553	NA	UKB-b-31	TRUE	reported	UkESpH
1	28383314	T	C	0.151763	0.033256	5.00E-06	0.008751	64949	NA	rs1489928	NA	UKB-b-31	TRUE	reported	UkESpH
1	96222052	T	C	0.03142	0.006819	4.10E-06	0.255658	64949	NA	rs1116548	NA	UKB-b-31	TRUE	reported	UkESpH
1	96228953	G	C	0.032448	0.006571	7.90E-07	0.286871	64949	NA	rs4551638	NA	UKB-b-31	TRUE	reported	UkESpH
1	2.07E+08	G	A	0.158207	0.031405	4.70E-07	0.010286	64949	NA	rs1921609	NA	UKB-b-31	TRUE	reported	UkESpH
2	54777857	C	G	0.054227	0.011731	3.80E-06	0.068966	64949	NA	rs7888581	NA	UKB-b-31	TRUE	reported	UkESpH
2	54792104	G	A	0.053701	0.011404	2.50E-06	0.072565	64949	NA	rs7947946	NA	UKB-b-31	TRUE	reported	UkESpH
2	54792729	G	A	0.054895	0.011624	2.30E-06	0.069755	64949	NA	rs7517672	NA	UKB-b-31	TRUE	reported	UkESpH
2	54817897	G	A	0.057326	0.011441	5.40E-07	0.07222	64949	NA	rs7825720	NA	UKB-b-31	TRUE	reported	UkESpH
2	54858214	A	G	0.055755	0.011385	9.70E-07	0.073227	64949	NA	rs6173012	NA	UKB-b-31	TRUE	reported	UkESpH
2	56675378	G	A	0.091186	0.018153	5.10E-07	0.027905	64949	NA	rs7280512	NA	UKB-b-31	TRUE	reported	UkESpH
3	4896170	T	G	0.029013	0.006335	4.70E-06	0.325375	64949	NA	rs9812852	NA	UKB-b-31	TRUE	reported	UkESpH
3	4897681	T	C	-0.02909	0.006334	4.40E-06	0.675151	64949	NA	rs4685838	NA	UKB-b-31	TRUE	reported	UkESpH
3	4897879	T	A	0.029033	0.006335	4.60E-06	0.324557	64949	NA	rs9819172	NA	UKB-b-31	TRUE	reported	UkESpH
3	4900301	G	A	0.029604	0.006336	3.00E-06	0.323663	64949	NA	rs1170787	NA	UKB-b-31	TRUE	reported	UkESpH
3	4900338	T	C	-0.02959	0.006336	3.00E-06	0.67595	64949	NA	rs9683311	NA	UKB-b-31	TRUE	reported	UkESpH
3	4905105	A	G	-0.0304	0.006305	1.40E-06	0.673713	64949	NA	rs7644783	NA	UKB-b-31	TRUE	reported	UkESpH
3	4905201	A	G	-0.03011	0.006306	1.80E-06	0.673752	64949	NA	rs2859808	NA	UKB-b-31	TRUE	reported	UkESpH
3	4905976	T	C	-0.02961	0.006308	2.70E-06	0.672788	64949	NA	rs1704161	NA	UKB-b-31	TRUE	reported	UkESpH
3	4906000	A	G	-0.02964	0.006309	2.60E-06	0.672947	64949	NA	rs6804059	NA	UKB-b-31	TRUE	reported	UkESpH
3	61401860	C	A	0.158581	0.034029	3.20E-06	0.008121	64949	NA	rs7745786	NA	UKB-b-31	TRUE	reported	UkESpH
3	1.15E+08	A	G	0.063456	0.013531	2.70E-06	0.050969	64949	NA	rs1164728	NA	UKB-b-31	TRUE	reported	UkESpH
3	1.15E+08	C	G	0.065371	0.013353	9.80E-07	0.051702	64949	NA	rs1543247	NA	UKB-b-31	TRUE	reported	UkESpH
3	1.72E+08	C	A	0.171651	0.035684	1.50E-06	0.007937	64949	NA	rs1168275	NA	UKB-b-31	TRUE	reported	UkESpH
4	1E+08	T	C	0.084117	0.017388	1.30E-06	0.970816	64949	NA	rs1229984	NA	UKB-b-31	TRUE	reported	UkESpH
4	1.67E+08	C	T	0.136439	0.028639	1.90E-06	0.0122	64949	NA	rs1154818	NA	UKB-b-31	TRUE	reported	UkESpH
5	1.58E+08	T	C	-0.04308	0.008589	5.30E-07	0.86278	64949	NA	rs2964331	NA	UKB-b-31	TRUE	reported	UkESpH
5	1.58E+08	T	C	-0.04354	0.008593	4.00E-07	0.862677	64949	NA	rs2988328	NA	UKB-b-31	TRUE	reported	UkESpH
5	1.58E+08	A	T	-0.04338	0.008593	4.40E-07	0.862668	64949	NA	rs2964333	NA	UKB-b-31	TRUE	reported	UkESpH
5	1.58E+08	C	T	-0.04331	0.008603	4.80E-07	0.862901	64949	NA	rs2988321	NA	UKB-b-31	TRUE	reported	UkESpH
5	1.58E+08	T	A	-0.04201	0.008473	7.10E-07	0.857727	64949	NA	rs1952657	NA	UKB-b-31	TRUE	reported	UkESpH
6	8002502	C	T	0.074054	0.016135	4.40E-06	0.034542	64949	NA	rs1164532	NA	UKB-b-31	TRUE	reported	UkESpH
11	16846313	C	T	0.029557	0.005916	5.80E-07	0.508795	64949	NA	rs1083268	NA	UKB-b-31	TRUE	reported	UkESpH
11	16849931	C	T	0.028294	0.005908	1.70E-06	0.511482	64949	NA	rs1016026	NA	UKB-b-31	TRUE	reported	UkESpH
11	16851317	G	A	0.028379	0.005908	1.60E-06	0.511393	64949	NA	rs1083269	NA	UKB-b-31	TRUE	reported	UkESpH
11	16851536	C	A	0.02837	0.005907	1.60E-06	0.511449	64949	NA	rs4757434	NA	UKB-b-31	TRUE	reported	UkESpH
11	16852672	C	T	0.028283	0.005908	1.70E-06	0.511484	64949	NA	rs740624	NA	UKB-b-31	TRUE	reported	UkESpH
11	16855622	G	A	0.02949	0.005906	6.00E-07	0.508433	64949	NA	rs1076635	NA	UKB-b-31	TRUE	reported	UkESpH
11	16855702	C	T	0.02949	0.005906	6.00E-07	0.508433	64949	NA	rs1074171	NA	UKB-b-31	TRUE	reported	UkESpH
11	16856344	T	C	0.028338	0.005908	1.60E-06	0.511448	64949	NA	rs1074171	NA	UKB-b-31	TRUE	reported	UkESpH
11	16857686	C	T	0.029458	0.005906	6.10E-07	0.508449	64949	NA	rs1076635	NA	UKB-b-31	TRUE	reported	UkESpH
11	16858496	G	A	0.029496	0.005906	5.90E-07	0.508414	64949	NA	rs9633895	NA	UKB-b-31	TRUE	reported	UkESpH
11	16859596	A	C	0.029636	0.005904	5.20E-07	0.508762	64949	NA	rs1083269	NA	UKB-b-31	TRUE	reported	UkESpH
11	16860002	G	A	0.02952	0.005905	5.80E-07	0.508399	64949	NA	rs1102405	NA	UKB-b-31	TRUE	reported	UkESpH
11	16860678	C	T	0.02952	0.005905	5.80E-07	0.508404	64949	NA	rs1083269	NA	UKB-b-31	TRUE	reported	UkESpH
11	16867980	T	C	0.028308	0.005904	1.60E-06	0.512288	64949	NA	rs2058161	NA	UKB-b-31	TRUE	reported	UkESpH
11	16873007	T	A	0.029384	0.006013	1.00E-06	0.418317	64949	NA	rs7928012	NA	UKB-b-31	TRUE	reported	UkESpH
11	16892477	C	G	-0.02828	0.005916	1.80E-06	0.542316	64949	NA	rs402973	NA	UKB-b-31	TRUE	reported	UkESpH
11	16894017	T	C	-0.02733	0.005913	3.80E-06	0.539252	64949	NA	rs450954	NA	UKB-b-31	TRUE	reported	UkESpH
11	16894413	G	A	-0.02724	0.005913	4.10E-06	0.539335	64949	NA	rs442389	NA	UKB-b-31	TRUE	reported	UkESpH
11	16894877	A	C	-0.02849	0.005913	1.40E-06	0.542462	64949	NA	rs370161	NA	UKB-b-31	TRUE	reported	UkESpH
11	16897496	C	T	0.034237	0.006137	2.40E-08	0.363045	64949	NA	rs1102406	NA	UKB-b-31	TRUE	reported	UkESpH
11	16905126	T	C	-0.02803	0.005915	2.10E-06	0.543556	64949	NA	rs384735	NA	UKB-b-31	TRUE	reported	UkESpH
11	16907500	C	T	0.033907	0.006142	3.40E-08	0.362073	64949	NA	rs1860271	NA	UKB-b-31	TRUE	reported	UkESpH
11	16909925	C	T	0.036226	0.006359	1.20E-08	0.341369	64949	NA	rs1076635	NA	UKB-b-31	TRUE	reported	UkESpH
11	16912251	A	C	-0.02802	0.005915	2.20E-06	0.543588	64949	NA	rs378755	NA	UKB-b-31	TRUE	reported	UkESpH
11	16913292	T	C	-0.0281	0.005915	2.00E-06	0.543517	64949	NA	rs1076635	NA	UKB-b-31	TRUE	reported	UkESpH
11	16913339	A	G	-0.02802	0.005915	2.20E-06	0.543586	64949	NA	rs1076635	NA	UKB-b-31	TRUE	reported	UkESpH
11	16916347	A	G	-0.02799	0.005914	2.20E-06	0.543374	64949	NA	rs1083269	NA	UKB-b-31	TRUE	reported	UkESpH
11	16916520	G	A	-0.028	0.005914	2.20E-06	0.543371	64949	NA	rs7128212	NA	UKB-b-31	TRUE	reported	UkESpH
11	16918993	T	C	0.031846	0.006095	1.70E-07	0.377841	64949	NA	rs1076635	NA	UKB-b-31	TRUE	reported	UkESpH
11	16924324	G	A	0.031795	0.0061	1.90E-07	0.3778	64949	NA	rs7109209	NA	UKB-b-31	TRUE	reported	UkESpH
11	16925684	G	A	0.028726	0.00593	1.30E-06	0.453301	64949	NA	rs20818	NA	UKB-b-31	TRUE	reported	UkESpH
11	16930106	A	G	-0.03059	0.005933	2.50E-07	0.464643	64949	NA	rs7483718	NA	UKB-b-31	TRUE	reported	UkESpH
11	16931046	A	G	-0.03057	0.005934	2.60E-07	0.464627	64949	NA	rs991606	NA	UKB-b-31	TRUE	reported	UkESpH
11	16933355	T	C	-0.03048	0.005935	2.80E-07	0.464585	64949	NA	rs7481075	NA	UKB-b-31	TRUE	reported	UkESpH
11	16934130	G	T	-0.02978	0.006045	8.40E-07	0.589999	64949	NA	rs7129155	NA	UKB-b-31	TRUE	reported	UkESpH
11	16934149	G	C	0.033368	0.00617	6.40E-08	0.366403	64949	NA	rs1076635	NA	UKB-b-31	TRUE	reported	UkESpH
11	16938450	G	A	0.031548	0.006154	2.90E-07	0.365402	64949	NA	rs1102407	NA	UKB-b-31	TRUE	reported	UkESpH
11	16947464	G	C	0.03117	0.006158	4.20E-07	0.366218	64949	NA	rs1083270	NA	UKB-b-31	TRUE	reported	UkESpH
11	62859026	C	A	0.183717	0.039172	2.70E-06	0.006098	64949	NA	rs1808139	NA	UKB-b-31	TRUE	reported	UkESpH
12	68110089	A	G	0.082777	0.017128	1.30E-06	0.030693	64949	NA	rs7313788	NA	UKB-b-31	TRUE	reported	UkESpH
13	22740581	T	C	0.108135	0.023311	3.50E-06	0.016505	64949	NA	rs5969639	NA	UKB-b-31	TRUE	reported	UkESpH
13	82956579	C	T	0.114973	0.024019	1.70E-06	0.016271	64949	NA	rs7323411	NA	UKB-b-31	TRUE	reported	UkESpH
14	1.04E+08	C	T	-0.0277	0.00601	4.00E-06	0.594443	64949	NA	rs1535099	NA	UKB-b-31	TRUE	reported	UkESpH
14	1.04E+08	T	C	0.028546	0.005945	1.60E-06	0.466276	64949	NA	rs2295148	NA	UKB-b-31	TRUE	reported	UkESpH
14	1.04E+08	C	T	0.028263	0.005959	2.10E-06	0.455444	64949	NA	rs2295147	NA	UKB-b-31	TRUE	reported	UkESpH
14	1.04E+08	C	G	0.03014	0.005932	3.80E-07	0.476488	64949	NA	rs941473	NA	UKB-b-31	TRUE	reported	UkESpH
14	1.04E+08	C	T	0.028264	0.005937	1.90E-06	0.464489	64949	NA	rs2295140	NA	UKB-b-31	TRUE	reported	UkESpH
14	1.04E+08	C	T	0.028202	0.005937	2.00E-06	0.464461	64949	NA	rs7141928	NA	UKB-b-31	TRUE	reported	UkESpH
14	1.04E+08	C	T	0.027688	0.005942	3.20E-06	0.460456	64949	NA	rs1955660	NA	UKB-b-31	TRUE	reported	UkESpH
14	1.04E+08	A	G	0.027843	0.005943	2.80E-06	0.464634	64949	NA	rs2368558	NA	UKB-b-31	TRUE	reported	UkESpH
14	1.04E+08	C	T	0.028094	0.005941	2.30E-06	0.464132	64949	NA	rs2368559	NA	UKB-b-31	TRUE	reported	UkESpH
14	1.04E+08	T	C	0.028141	0.005939	2.20E-06	0.463682	64949	NA	rs1162725	NA	UKB-b-31	TRUE	reported	UkESpH
14	1.04E+08	A	C	0.028137	0.005939	2.20E-06	0.463687	64949	NA	rs1243706	NA	UKB-b-31	TRUE	reported	UkESpH
14	1.04E+08	A	C	0.028134	0.005939	2.20E-06	0.463644	64949	NA	rs941475	NA	UKB-b-31	TRUE	reported	UkESpH
14	1.04E+08	A	C	0.028162	0.005939	2.10E-06	0.463589	64949	NA	rs4906368	NA	UKB-b-31	TRUE	reported	UkESpH
14	1.04E+08	A	G	0.028416	0.005948	1.80E-06	0.466292	64949	NA	rs6575998	NA	UKB-b-31	TRUE	reported	UkESpH
14	1.04E+08	A	C	0.028203	0.005941	2.10E-06	0.463744	64949	NA	rs2144080	NA	UKB-b-31	TRUE	reported	UkESpH
14	1.04E+08	C	T	0.028202	0.005941	2.10E-06	0.463748	64949	NA	rs2144079	NA	UKB-b-31	TRUE	reported	UkESpH
14	1.04E+08	A	T	0.028237	0.005941	2.00E-06	0.463867	64949	NA	rs1547604	NA	UKB-b-31	TRUE	reported	UkESpH
14	1.04E+08	G	C	0.027997	0.005942	2.50E-06	0.463993	64949	NA	rs2024669	NA	UKB-b-31	TRUE	reported	UkESpH
14	1.04E+08	G	C	0.0284	0.005942	1.80E-06	0.464346	64949	NA	rs8018363	NA	UKB-b-31	TRUE	reported	UkESpH
14	1.04E+08	C	A	0.028253	0.005942	2.00E-06	0.463624	64949	NA	rs6576003	NA	UKB-b-31	TRUE	reported	UkESpH
14	1.04E+08	C	T	0.02824	0.005942	2.00E-06	0.463632	64949	NA	rs6576005	NA	UKB-b-31	TRUE	reported	UkESpH
14	1.04E+08	T	C	0.028428	0.005944	1.70E-06	0.46361	64949	NA	rs8009868	NA	UKB-b-31	TRUE	reported	UkESpH
14	1.04E+08	A	G	0.028404	0.005944	1.80E-06	0.463656	64949	NA	rs1116076	NA	UKB-b-31	TRUE	reported	UkESpH
14	1.04E+08	C	T	-0.02848	0.005952	1.70E-06	0.536014	64949	NA	rs8014294	NA	UKB-b-31	TRUE	reported	UkESpH
14	1.04E+08	G	A	-0.0285	0.005956	1.70E-06	0.535964	64949	NA	rs1162878	NA	UKB-b-31	TRUE	reported	UkESpH
16	4362937	G	A	0.142264	0.030719	3.60E-06	0.011817	64949	NA	rs1432978	NA	UKB-b-31	TRUE	reported	UkESpH
16	22092319	A	G	0.147452	0.031442	2.70E-06	0.009369	64949	NA	rs1406602	NA	UKB-b-31	TRUE	reported	UkESpH
16	22187967	C	T	0.162885	0.033868	1.50E-06	0.00784	64949	NA	rs1448906	NA	UKB-b-31	TRUE	reported	UkESpH
16	22194785	G	T	0.162417	0.033842	1.60E-06	0.007847	64949	NA	rs1884926	NA	UKB-b-31	TRUE	reported	UkESpH

16	22196230	C	T	0.164054	0.033898	1.30E-06	0.007816	64949	NA	rs1457018	NA	UKB-b-31	TRUE	reported	UkESpH
17	26402980	C	T	0.12425	0.026959	4.10E-06	0.012763	64949	NA	rs3427949	NA	UKB-b-31	TRUE	reported	UkESpH
17	26428812	T	G	0.126741	0.027387	3.70E-06	0.012504	64949	NA	rs1170462	NA	UKB-b-31	TRUE	reported	UkESpH
17	68715837	T	G	-0.03325	0.007246	4.50E-06	0.215661	64949	NA	rs7286321	NA	UKB-b-31	TRUE	reported	UkESpH
17	68730618	C	T	-0.03324	0.007267	4.80E-06	0.215167	64949	NA	rs7286324	NA	UKB-b-31	TRUE	reported	UkESpH
18	22148121	G	A	-0.02923	0.0062	2.40E-06	0.350283	64949	NA	rs4800580	NA	UKB-b-31	TRUE	reported	UkESpH
18	22148755	T	G	-0.02927	0.006198	2.30E-06	0.350232	64949	NA	rs9304465	NA	UKB-b-31	TRUE	reported	UkESpH
18	22148973	C	G	-0.0296	0.00621	1.90E-06	0.346971	64949	NA	rs9304466	NA	UKB-b-31	TRUE	reported	UkESpH
18	22149164	G	A	-0.02913	0.006197	2.60E-06	0.350249	64949	NA	rs9304467	NA	UKB-b-31	TRUE	reported	UkESpH
18	22149741	T	C	-0.0297	0.006188	1.60E-06	0.350984	64949	NA	rs9950310	NA	UKB-b-31	TRUE	reported	UkESpH
18	22150403	A	G	-0.02987	0.006187	1.40E-06	0.351059	64949	NA	rs3502229	NA	UKB-b-31	TRUE	reported	UkESpH
18	22151982	C	T	-0.03003	0.006186	1.20E-06	0.350883	64949	NA	rs6762769	NA	UKB-b-31	TRUE	reported	UkESpH
18	22153886	A	T	-0.02925	0.006202	2.40E-06	0.346899	64949	NA	rs4800186	NA	UKB-b-31	TRUE	reported	UkESpH
18	22155018	C	A	-0.0295	0.006204	2.00E-06	0.346735	64949	NA	rs1218541	NA	UKB-b-31	TRUE	reported	UkESpH
18	22155136	C	T	-0.02935	0.006205	2.20E-06	0.346633	64949	NA	rs1296042	NA	UKB-b-31	TRUE	reported	UkESpH
18	22155843	A	G	-0.02942	0.006208	2.10E-06	0.346631	64949	NA	rs1945153	NA	UKB-b-31	TRUE	reported	UkESpH
18	22156798	G	A	-0.02923	0.00621	2.50E-06	0.346654	64949	NA	rs4800581	NA	UKB-b-31	TRUE	reported	UkESpH
18	22170630	G	A	-0.02957	0.006209	1.90E-06	0.351291	64949	NA	rs1894019	NA	UKB-b-31	TRUE	reported	UkESpH
18	22171896	G	A	-0.02958	0.006209	1.90E-06	0.351305	64949	NA	rs9955229	NA	UKB-b-31	TRUE	reported	UkESpH
18	22172695	G	A	-0.02973	0.006209	1.70E-06	0.351288	64949	NA	rs7230100	NA	UKB-b-31	TRUE	reported	UkESpH
18	22185861	A	T	-0.02967	0.006126	1.30E-06	0.375662	64949	NA	rs996242	NA	UKB-b-31	TRUE	reported	UkESpH
18	22186395	T	C	-0.02955	0.006151	1.60E-06	0.373451	64949	NA	rs4800583	NA	UKB-b-31	TRUE	reported	UkESpH
18	22186474	A	G	-0.02984	0.006143	1.20E-06	0.373952	64949	NA	rs4800585	NA	UKB-b-31	TRUE	reported	UkESpH
18	22186478	C	T	-0.02984	0.006143	1.20E-06	0.373945	64949	NA	rs4800586	NA	UKB-b-31	TRUE	reported	UkESpH
18	22186609	C	A	-0.02976	0.006125	1.20E-06	0.37577	64949	NA	rs4800587	NA	UKB-b-31	TRUE	reported	UkESpH
18	22186837	T	A	-0.02973	0.006125	1.20E-06	0.375768	64949	NA	rs2200064	NA	UKB-b-31	TRUE	reported	UkESpH
18	22187140	C	A	-0.02972	0.006124	1.20E-06	0.375771	64949	NA	rs1894021	NA	UKB-b-31	TRUE	reported	UkESpH
18	22187235	C	T	-0.0296	0.006126	1.40E-06	0.37548	64949	NA	rs1894022	NA	UKB-b-31	TRUE	reported	UkESpH
18	22205037	T	C	-0.03189	0.006008	1.10E-07	0.419944	64949	NA	rs339870	NA	UKB-b-31	TRUE	reported	UkESpH
18	22208601	G	A	-0.03149	0.006018	1.70E-07	0.417804	64949	NA	rs339866	NA	UKB-b-31	TRUE	reported	UkESpH

Beer/cider intake SNPs

chr.exposu	pos.exposu	other_allel	effect_allel	beta.expos	se.exposur	pval.expos	eaf.exposu	samplesize	ncase.expo	SNP	ncontrol.e	exposure	mr_keep.e	pval_origir	id.exposure
1	1.53E+08	T	C	0.182065	0.039727	4.60E-06	0.006256	64949	NA	rs1886730	NA	UKB-b-49	TRUE	reported	oWhqFI
1	1.53E+08	C	T	0.192767	0.040862	2.40E-06	0.005935	64949	NA	rs1408984	NA	UKB-b-49	TRUE	reported	oWhqFI
1	1.53E+08	C	T	0.209948	0.042384	7.30E-07	0.005577	64949	NA	rs5759808	NA	UKB-b-49	TRUE	reported	oWhqFI
2	27731212	T	C	0.037533	0.007655	9.40E-07	0.193605	64949	NA	rs3817588	NA	UKB-b-49	TRUE	reported	oWhqFI
2	1.48E+08	G	T	0.081221	0.017012	1.80E-06	0.032125	64949	NA	rs7286306	NA	UKB-b-49	TRUE	reported	oWhqFI
2	2.43E+08	G	A	-0.03162	0.006867	4.10E-06	0.263505	64949	NA	rs1247304	NA	UKB-b-49	TRUE	reported	oWhqFI
4	1E+08	T	C	0.108821	0.017656	7.10E-10	0.970816	64949	NA	rs1229984	NA	UKB-b-49	TRUE	reported	oWhqFI
4	1.01E+08	T	A	0.040612	0.008413	1.40E-06	0.152948	64949	NA	rs6839368	NA	UKB-b-49	TRUE	reported	oWhqFI
4	1.01E+08	C	T	0.039719	0.008622	4.10E-06	0.142822	64949	NA	rs1310392	NA	UKB-b-49	TRUE	reported	oWhqFI
4	1.01E+08	A	G	0.038109	0.008281	4.20E-06	0.155642	64949	NA	rs1134657	NA	UKB-b-49	TRUE	reported	oWhqFI
4	1.01E+08	T	C	0.047307	0.009138	2.30E-07	0.126599	64949	NA	rs6751017	NA	UKB-b-49	TRUE	reported	oWhqFI
4	1.07E+08	C	T	0.158177	0.034474	4.50E-06	0.007754	64949	NA	rs1389239	NA	UKB-b-49	TRUE	reported	oWhqFI
5	1.66E+08	C	A	-0.05894	0.01282	4.30E-06	0.942349	64949	NA	rs2964294	NA	UKB-b-49	TRUE	reported	oWhqFI
5	1.66E+08	A	T	-0.05867	0.012845	4.90E-06	0.94247	64949	NA	rs1450634	NA	UKB-b-49	TRUE	reported	oWhqFI
5	1.66E+08	C	T	-0.063	0.012583	5.60E-07	0.938916	64949	NA	rs2968372	NA	UKB-b-49	TRUE	reported	oWhqFI
5	1.66E+08	A	G	-0.06272	0.012584	6.20E-07	0.93893	64949	NA	rs2961855	NA	UKB-b-49	TRUE	reported	oWhqFI
5	1.66E+08	A	G	-0.06268	0.012586	6.40E-07	0.938863	64949	NA	rs1450628	NA	UKB-b-49	TRUE	reported	oWhqFI
5	1.66E+08	G	T	-0.06364	0.01256	4.00E-07	0.938593	64949	NA	rs1450627	NA	UKB-b-49	TRUE	reported	oWhqFI
5	1.66E+08	G	A	-0.06276	0.012587	6.20E-07	0.938916	64949	NA	rs2964297	NA	UKB-b-49	TRUE	reported	oWhqFI
5	1.66E+08	T	C	-0.06291	0.012602	6.00E-07	0.939006	64949	NA	rs2923175	NA	UKB-b-49	TRUE	reported	oWhqFI
5	1.66E+08	A	T	-0.06291	0.012601	6.00E-07	0.939011	64949	NA	rs2964298	NA	UKB-b-49	TRUE	reported	oWhqFI
5	1.66E+08	C	G	0.062189	0.013595	4.80E-06	0.05207	64949	NA	rs7282399	NA	UKB-b-49	TRUE	reported	oWhqFI
6	61923152	G	A	0.075364	0.015787	1.80E-06	0.039612	64949	NA	rs1401568	NA	UKB-b-49	TRUE	reported	oWhqFI
6	62698356	A	G	0.074869	0.01614	3.50E-06	0.037041	64949	NA	rs1221126	NA	UKB-b-49	TRUE	reported	oWhqFI
6	62792379	G	A	0.077793	0.015779	8.20E-07	0.037745	64949	NA	rs1219142	NA	UKB-b-49	TRUE	reported	oWhqFI
6	62793367	G	A	0.077161	0.015812	1.10E-06	0.037581	64949	NA	rs7288610	NA	UKB-b-49	TRUE	reported	oWhqFI
6	62855407	T	C	0.077229	0.015887	1.20E-06	0.037419	64949	NA	rs1220663	NA	UKB-b-49	TRUE	reported	oWhqFI
9	80479585	A	T	0.201257	0.042175	1.80E-06	0.00593	64949	NA	rs1912032	NA	UKB-b-49	TRUE	reported	oWhqFI
9	87643179	C	T	0.06633	0.01282	2.30E-07	0.058138	64949	NA	rs6256525	NA	UKB-b-49	TRUE	reported	oWhqFI
9	87656183	C	T	0.067558	0.014699	4.30E-06	0.043465	64949	NA	rs7778008	NA	UKB-b-49	TRUE	reported	oWhqFI
9	87684229	G	T	0.072009	0.014881	1.30E-06	0.043948	64949	NA	rs6256525	NA	UKB-b-49	TRUE	reported	oWhqFI
11	1.08E+08	C	G	-0.08888	0.019372	4.50E-06	0.024481	64949	NA	rs7299218	NA	UKB-b-49	TRUE	reported	oWhqFI
12	2875121	C	T	0.080896	0.01715	2.40E-06	0.046997	64949	NA	rs7551075	NA	UKB-b-49	TRUE	reported	oWhqFI
12	20413592	C	T	0.067013	0.012145	3.40E-08	0.071721	64949	NA	rs1842578	NA	UKB-b-49	TRUE	reported	oWhqFI
12	27614760	A	G	0.071773	0.01533	2.80E-06	0.040027	64949	NA	rs7308206	NA	UKB-b-49	TRUE	reported	oWhqFI
13	54677251	G	C	-0.03293	0.007042	2.90E-06	0.242752	64949	NA	rs2408991	NA	UKB-b-49	TRUE	reported	oWhqFI
17	33953109	G	A	0.084828	0.017695	1.60E-06	0.029616	64949	NA	rs225253	NA	UKB-b-49	TRUE	reported	oWhqFI
19	17951752	G	C	0.057026	0.011642	9.70E-07	0.074089	64949	NA	rs3212737	NA	UKB-b-49	TRUE	reported	oWhqFI
19	17952930	A	G	0.05518	0.01139	1.30E-06	0.075043	64949	NA	rs867174	NA	UKB-b-49	TRUE	reported	oWhqFI
19	49164952	A	G	0.029862	0.006446	3.60E-06	0.664689	64949	NA	rs281392	NA	UKB-b-49	TRUE	reported	oWhqFI
19	49168182	T	C	0.02953	0.006371	3.60E-06	0.651223	64949	NA	rs373002	NA	UKB-b-49	TRUE	reported	oWhqFI
19	49170068	G	A	0.029578	0.006394	3.70E-06	0.657804	64949	NA	rs6782418	NA	UKB-b-49	TRUE	reported	oWhqFI

Fortified wine intake SNPs

chr.exposu	pos.exposu	other_allel	effect_allel	beta.expos	se.exposur	pval.expos	eaf.exposu	samplesize	ncase.expo	SNP	ncontrol.e	exposure	mr_keep.e	pval_origir	id.exposure
1	12788509	G	T	0.035492	0.007355	1.40E-06	0.015715	64942	NA	rs1510348	NA	UKB-b-95	TRUE	reported	u6dHsG
1	2.22E+08	A	C	0.048791	0.010313	2.20E-06	0.005941	64942	NA	rs1472085	NA	UKB-b-95	TRUE	reported	u6dHsG
2	36448626	A	G	0.051071	0.01115	4.60E-06	0.005813	64942	NA	rs1505870	NA	UKB-b-95	TRUE	reported	u6dHsG
2	1.21E+08	G	A	0.042338	0.008264	3.00E-07	0.010322	64942	NA	rs1506689	NA	UKB-b-95	TRUE	reported	u6dHsG
3	4103328	C	T	0.02852	0.006192	4.10E-06	0.015599	64942	NA	rs7743980	NA	UKB-b-95	TRUE	reported	u6dHsG
3	84252694	T	C	-0.00715	0.00155	4.00E-06	0.441555	64942	NA	rs7430880	NA	UKB-b-95	TRUE	reported	u6dHsG
3	84253342	G	A	-0.00715	0.001551	4.00E-06	0.441608	64942	NA	rs6780104	NA	UKB-b-95	TRUE	reported	u6dHsG
3	84253609	T	C	-0.00716	0.001551	3.80E-06	0.441731	64942	NA	rs4279116	NA	UKB-b-95	TRUE	reported	u6dHsG
3	1.06E+08	G	T	0.047506	0.010165	3.00E-06	0.006599	64942	NA	rs1839943	NA	UKB-b-95	TRUE	reported	u6dHsG
3	1.4E+08	C	T	0.051802	0.010781	1.50E-06	0.005415	64942	NA	rs1497527	NA	UKB-b-95	TRUE	reported	u6dHsG
3	1.4E+08	T	A	0.045411	0.009177	7.50E-07	0.007076	64942	NA	rs4126545	NA	UKB-b-95	TRUE	reported	u6dHsG
3	1.4E+08	C	T	0.048063	0.009231	1.90E-07	0.007096	64942	NA	rs1494410	NA	UKB-b-95	TRUE	reported	u6dHsG
3	1.4E+08	C	A	0.048378	0.00925	1.70E-07	0.007044	64942	NA	rs1491915	NA	UKB-b-95	TRUE	reported	u6dHsG
3	1.4E+08	T	C	0.049984	0.009352	9.00E-08	0.007174	64942	NA	rs1473305	NA	UKB-b-95	TRUE	reported	u6dHsG
3	1.4E+08	G	A	0.053027	0.009942	9.60E-08	0.006581	64942	NA	rs1893606	NA	UKB-b-95	TRUE	reported	u6dHsG
3	1.4E+08	T	C	0.049437	0.009439	1.60E-07	0.007085	64942	NA	rs1896027	NA	UKB-b-95	TRUE	reported	u6dHsG
3	1.4E+08	G	A	0.043436	0.009302	3.00E-06	0.007228	64942	NA	rs1473750	NA	UKB-b-95	TRUE	reported	u6dHsG
3	1.41E+08	A	C	0.047529	0.009457	5.00E-07	0.007021	64942	NA	rs5614680	NA	UKB-b-95	TRUE	reported	u6dHsG
3	1.41E+08	C	A	0.049258	0.009698	3.80E-07	0.006734	64942	NA	rs1890454	NA	UKB-b-95	TRUE	reported	u6dHsG
3	1.41E+08	C	T	0.04989	0.009668	2.50E-07	0.006501	64942	NA	rs1438519	NA	UKB-b-95	TRUE	reported	u6dHsG
3	1.41E+08	T	C	0.049191	0.009656	3.50E-07	0.006502	64942	NA	rs1448728	NA	UKB-b-95	TRUE	reported	u6dHsG
3	1.41E+08	A	G	0.045553	0.009564	1.90E-06	0.006915	64942	NA	rs1463557	NA	UKB-b-95	TRUE	reported	u6dHsG
3	1.41E+08	C	T	0.052071	0.010366	5.10E-07	0.005918	64942	NA	rs1819691	NA	UKB-b-95	TRUE	reported	u6dHsG
4	96460507	C	A	0.042513	0.009083	2.90E-06	0.007273	64942	NA	rs7651653	NA	UKB-b-95	TRUE	reported	u6dHsG
4	1.11E+08	A	G	0.029213	0.006084	1.60E-06	0.018142	64942	NA	rs1507184	NA	UKB-b-95	TRUE	reported	u6dHsG
4	1.39E+08	T	C	0.044597	0.007963	2.10E-08	0.010176	64942	NA	rs1873492	NA	UKB-b-95	TRUE	reported	u6dHsG
4	1.77E+08	G	A	0.01599	0.003446	3.50E-06	0.056271	64942	NA	rs6234058	NA	UKB-b-95	TRUE	reported	u6dHsG
5	88936501	A	G	0.043302	0.009456	4.70E-06	0.006957	64942	NA	rs7277721	NA	UKB-b-95	TRUE	reported	u6dHsG
5	88964361	G	T	0.055268	0.010722	2.50E-07	0.005607	64942	NA	rs7277723	NA	UKB-b-95	TRUE	reported	u6dHsG
5	1.47E+08	T	C	0.054139	0.011159	1.20E-06	0.005687	64942	NA	rs1900197	NA	UKB-b-95	TRUE	reported	u6dHsG
5	1.73E+08	T	C	0.010144	0.002004	4.20E-07	0.180424	64942	NA	rs1776430	NA	UKB-b-95	TRUE	reported	u6dHsG
5	1.73E+08	G	A	0.010278	0.002061	6.10E-07	0.1684	64942	NA	rs7444016	NA	UKB-b-95	TRUE	reported	u6dHsG
5	1.73E+08	G	A	0.010922	0.002063	1.20E-07	0.168941	64942	NA	rs7281477	NA	UKB-b-95	TRUE	reported	u6dHsG
5	1.74E+08	C	T	0.010538	0.002057	3.00E-07	0.168466	64942	NA	rs1770875	NA	UKB-b-95	TRUE	reported	u6dHsG
6	9511324	C	A	0.050072	0.009619	1.90E-07	0.007413	64942	NA	rs1390172	NA	UKB-b-95	TRUE	reported	u6dHsG
6	17645949	T	C	0.041969	0.00801	1.60E-07	0.009857	64942	NA	rs1320897	NA	UKB-b-95	TRUE	reported	u6dHsG
6	43419646	C	A	0.052381	0.011103	2.40E-06	0.005458	64942	NA	rs1478083	NA	UKB-b-95	TRUE	reported	u6dHsG
6	74700880	C	G	0.04407	0.00954	3.90E-06	0.007716	64942	NA	rs1390924	NA	UKB-b-95	TRUE	reported	u6dHsG
7	8026627	C	T	0.046474	0.009337	6.40E-07	0.007049	64942	NA	rs1148942	NA	UKB-b-95	TRUE	reported	u6dHsG
7	8040514	T	C	0.044604	0.009318	1.70E-06	0.007049	64942	NA	rs1324019	NA	UKB-b-95	TRUE	reported	u6dHsG
7	41645710	A	G	0.050304	0.010636	2.20E-06	0.005958	64942	NA	rs5473763	NA	UKB-b-95	TRUE	reported	u6dHsG
7	87189458	C	T	0.026695	0.005758	3.50E-06	0.019498	64942	NA	rs7319839	NA	UKB-b-95	TRUE	reported	u6dHsG
7	1.39E+08	C	T	0.049661	0.010876	5.00E-06	0.005602	64942	NA	rs1506577	NA	UKB-b-95	TRUE	reported	u6dHsG
7	1.55E+08	C	T	0.019402	0.004042	1.60E-06	0.042095	64942	NA	rs1131465	NA	UKB-b-95	TRUE	reported	u6dHsG
8	448315	C	A	0.050804	0.010271	7.60E-07	0.005696	64942	NA	rs1327603	NA	UKB-b-95	TRUE	reported	u6dHsG
8	454864	G	C	0.0479	0.010348	3.70E-06	0.005591	64942	NA	rs3568070	NA	UKB-b-95	TRUE	reported	u6dHsG
8	1.42E+08	C	G	0.046684	0.010206	4.80E-06	0.006728	64942	NA	rs5654311	NA	UKB-b-95	TRUE	reported	u6dHsG
9	17238393	T	C	0.05187	0.010927	2.10E-06	0.005801	64942	NA	rs1179315	NA	UKB-b-95	TRUE	reported	u6dHsG
9	23545229	T	C	0.014006	0.00304	4.10E-06	0.071278	64942	NA	rs274958	NA	UKB-b-95	TRUE	reported	u6dHsG
9	23545445	A	G	0.013876	0.003039	5.00E-06	0.071531	64942	NA	rs9644868	NA	UKB-b-95	TRUE	reported	u6dHsG
9	84779502	A	G	0.040896	0.008791	3.30E-06	0.0083	64942	NA	rs1180765	NA	UKB-b-95	TRUE	reported	u6dHsG
9	91138241	A	G	0.012672	0.002722	3.20E-06	0.087206	64942	NA	rs2354555	NA	UKB-b-95	TRUE	reported	u6dHsG
9	91138753	C	G	0.012676	0.002722	3.20E-06	0.087222	64942	NA	rs2026837	NA	UKB-b-95	TRUE	reported	u6dHsG
9	91139780	C	T	0.012802	0.002738	2.90E-06	0.086181	64942	NA	rs4242606	NA	UKB-b-95	TRUE	reported	u6dHsG
9	91141073	T	C	0.012433	0.002715	4.70E-06	0.087936	64942	NA	rs2845556	NA	UKB-b-95	TRUE	reported	u6dHsG
9	91142251	C	T	0.012879	0.002728	2.30E-06	0.087132	64942	NA	rs1268670	NA	UKB-b-95	TRUE	reported	u6dHsG
9	91142376	C	A	0.012907	0.002734	2.40E-06	0.086604	64942	NA	rs1268672	NA	UKB-b-95	TRUE	reported	u6dHsG
9	91142852	A	G	0.01285	0.002739	2.70E-06	0.086318	64942	NA	rs1556264	NA	UKB-b-95	TRUE	reported	u6dHsG
9	1.34E+08	T	C	0.030273	0.006259	1.30E-06	0.016888	64942	NA	rs1413934	NA	UKB-b-95	TRUE	reported	u6dHsG
10	1.25E+08	G	C	0.019556	0.00408	1.60E-06	0.03935	64942	NA	rs1124842	NA	UKB-b-95	TRUE	reported	u6dHsG
11	18983155	A	G	0.023848	0.005028	2.10E-06	0.02497	64942	NA	rs1102491	NA	UKB-b-95	TRUE	reported	u6dHsG
11	19009516	G	T	0.02229	0.004829	3.90E-06	0.025977	64942	NA	rs7465333	NA	UKB-b-95	TRUE	reported	u6dHsG
11	24750595	A	G	0.054215	0.0106	3.10E-07	0.006283	64942	NA	rs1880284	NA	UKB-b-95	TRUE	reported	u6dHsG
11	1.06E+08	C	T	0.048241	0.00974	7.30E-07	0.007402	64942	NA	rs1885505	NA	UKB-b-95	TRUE	reported	u6dHsG
11	1.06E+08	A	C	0.03862	0.008428	4.60E-06	0.009266	64942	NA	rs1925414	NA	UKB-b-95	TRUE	reported	u6dHsG
11	1.31E+08	C	T	0.035691	0.006822	1.70E-07	0.013512	64942	NA	rs1422850	NA	UKB-b-95	TRUE	reported	u6dHsG
12	32388575	G	A	0.040268	0.008328	1.30E-06	0.008561	64942	NA	rs1178879	NA	UKB-b-95	TRUE	reported	u6dHsG
12	60068722	C	T	0.040364	0.008757	4.00E-06	0.008075	64942	NA	rs1494847	NA	UKB-b-95	TRUE	reported	u6dHsG
12	95494069	T	C	0.042491	0.009107	3.10E-06	0.007902	64942	NA	rs1822876	NA	UKB-b-95	TRUE	reported	u6dHsG
14	56030882	C	T	0.054603	0.010344	1.30E-07	0.005773	64942	NA	rs1883901	NA	UKB-b-95	TRUE	reported	u6dHsG
14	56157089	A	G	0.03098	0.006777	4.80E-06	0.013035	64942	NA	rs1171188	NA	UKB-b-95	TRUE	reported	u6dHsG
15	53891205	T	C	-0.0088	0.001821	1.40E-06	0.764833	64942	NA	rs1906445	NA	UKB-b-95	TRUE	reported	u6dHsG
15	53892200	T	G	-0.00881	0.001821	1.30E-06	0.765004	64942	NA	rs2899517	NA	UKB-b-95	TRUE	reported	u6dHsG
15	53897446	G	A	-0.00885	0.00182	1.20E-06	0.764717	64942	NA	rs7171089	NA	UKB-b-95	TRUE	reported	u6dHsG
15	53898355	G	C	-0.00884	0.00182	1.20E-06	0.76474	64942	NA	rs2171477	NA	UKB-b-95	TRUE	reported	u6dHsG
15	93085884	T	C	0.048681	0.009181	1.10E-07	0.008152	64942	NA	rs7694181	NA	UKB-b-95	TRUE	reported	u6dHsG
15	99095953	C	T	0.051888	0.010005	2.10E-07	0.006424	64942	NA	rs1423504	NA	UKB-b-95	TRUE	reported	u6dHsG
15	99097304	G	C	0.059145	0.010552	2.10E-08	0.005914	64942	NA	rs1497167	NA	UKB-b-95	TRUE	reported	u6dHsG
16	62910500	A	G	0.029267	0.006128	1.80E-06	0.016109	64942	NA	rs2914477	NA	UKB-b-95	TRUE	reported	u6dHsG
16	62914902	G	A	0.029499	0.006166	1.70E-06	0.015996	64942	NA	rs2911318	NA	UKB-b-95	TRUE	reported	u6dHsG
16	62931461	G	T	0.028626	0.006114	2.80E-06	0.016153	64942	NA	rs2962453	NA	UKB-b-95	TRUE	reported	u6dHsG
16	62934892	A	G	0.028376	0.006103	3.30E-06	0.016183	64942	NA	rs2962452	NA	UKB-b-95	TRUE	reported	u6dHsG
16	63190214	G	T	-0.02784	0.005248	1.10E-07	0.977811	64942	NA	rs1704584	NA	UKB-b-95	TRUE	reported	u6dHsG
16	63199313	G	C	0.028479	0.00527	6.50E-08	0.021983	64942	NA	rs5618770	NA	UKB-b-95	TRUE	reported	u6dHsG
16	63202485	G	A	0.028335	0.005263	7.30E-08	0.021997	64942	NA	rs7887085	NA	UKB-b-95	TRUE	reported	u6dHsG
16	63216783	T	C	0.028416	0.005282	7.50E-08	0.021817	64942	NA	rs2318935	NA	UKB-b-95	TRUE	reported	u6dHsG
16	63238344	A	C	0.043669	0.008572	3.50E-07	0.008981	64942	NA	rs7987705	NA	UKB-b-95	TRUE	reported	u6dHsG
16	63239848	G	A	0.028581	0.005285	6.40E-08	0.021768	64942	NA	rs7505349	NA	UKB-b-95	TRUE	reported	u6dHsG
16	63252532	T	C	0.028588	0.005285	6.30E-08	0.021772	64942	NA	rs7731795	NA	UKB-b-95	TRUE	reported	u6dHsG
16	63252611	T	C	0.028176	0.005269	8.90E-08	0.021917	64942	NA	rs2854799	NA	UKB-b-95	TRUE	reported	u6dHsG
16	63253442	T	G	0.028595	0.005285	6.30E-08	0.02177	64942	NA	rs2839143	NA	UKB-b-95	TRUE	reported	u6dHsG
16	63253494	C	T	0.028611	0.005285	6.20E-08	0.021764	64942	NA	rs2854846	NA	UKB-b-95	TRUE	reported	u6dHsG
16	63253733	A	G	0.028601	0.005285	6.20E-08	0.021767	64942	NA	rs2863216	NA	UKB-b-95	TRUE	reported	u6dHsG
16	63256296	C	T	0.02859	0.005284	6.30E-08	0.02178	64942	NA	rs4485394	NA	UKB-b-95	TRUE	reported	u6dHsG
16	63258044	A	G	0.026251	0.005147	3.40E-07	0.023012	64942	NA	rs1436406	NA	UKB-b-95	TRUE	reported	u6dHsG
16	63259304	T	C	0.026741	0.005167	2.30E-07	0.022826	64942	NA	rs1436409	NA	UKB-b-95	TRUE	reported	u6dHsG
16	63262025	A	G	0.026452	0.005155	2.90E-07	0.022929	64942	NA	rs2318849	NA	UKB-b-95	TRUE	reported	u6dHsG
16	63263231	G	A	0.028654	0.005286	5.90E-08	0.021756	64942	NA	rs7948251	NA	UKB-b-95	TRUE	reported	u6dHsG
16	63276563	G	A	0.02784	0.00524	1.10E-07	0.022178	64942	NA	rs1157454	NA	UKB-b-95	TRUE	reported	u6dHsG
16	63276918	T	C	0.027852	0.005241	1.10E-07	0.022174	64942	NA	rs1424324	NA	UKB-b-95	TRUE	reported	u6dHsG
16	63281776	G	A	0.026882	0.005176	2.10E-07	0.022837	64942	NA	rs7456622	NA	UKB-b-95	TRUE	reported	u6dHsG
16	63287215	A	G	0.024773	0.005063	1.00E-06	0.024082	64942	NA	rs2217912	NA	UKB-b-95	TRUE	reported	u6dHsG
16	63289540	A	G	0.02615	0.005145	3.70E-07	0.023129	64942	NA	rs7598286	NA	UKB-b-95	TRUE	reported	u6dHsG

16	63337329	G	C	0.027776	0.005232	1.10E-07	0.022367	64942	NA	rs160530	NA	UKB-b-95	TRUE	reported	u6dHsG
16	63349440	A	G	0.027994	0.005186	6.70E-08	0.022668	64942	NA	rs219570	NA	UKB-b-95	TRUE	reported	u6dHsG
16	63357406	T	C	0.027611	0.005186	1.00E-07	0.02268	64942	NA	rs160536	NA	UKB-b-95	TRUE	reported	u6dHsG
16	63368257	A	G	0.027714	0.005244	1.30E-07	0.022212	64942	NA	rs219600	NA	UKB-b-95	TRUE	reported	u6dHsG
16	63374584	G	A	0.027929	0.005255	1.10E-07	0.022073	64942	NA	rs219596	NA	UKB-b-95	TRUE	reported	u6dHsG
16	63377164	C	T	0.028165	0.005265	8.80E-08	0.021997	64942	NA	rs219589	NA	UKB-b-95	TRUE	reported	u6dHsG
16	63383374	G	A	0.025308	0.005075	6.10E-07	0.023744	64942	NA	rs403489	NA	UKB-b-95	TRUE	reported	u6dHsG
16	63387134	C	T	0.025221	0.005016	5.00E-07	0.024243	64942	NA	rs150736	NA	UKB-b-95	TRUE	reported	u6dHsG
16	63391295	A	C	0.025134	0.005006	5.10E-07	0.024314	64942	NA	rs150636	NA	UKB-b-95	TRUE	reported	u6dHsG
17	67405824	T	A	0.041205	0.008996	4.60E-06	0.007744	64942	NA	rs1853966	NA	UKB-b-95	TRUE	reported	u6dHsG
19	47805620	A	G	0.04586	0.010011	4.60E-06	0.006361	64942	NA	rs1446180	NA	UKB-b-95	TRUE	reported	u6dHsG
19	52331531	G	A	0.046666	0.008868	1.40E-07	0.007885	64942	NA	rs1457230	NA	UKB-b-95	TRUE	reported	u6dHsG
19	52360871	G	A	0.042762	0.008726	9.50E-07	0.008046	64942	NA	rs6210750	NA	UKB-b-95	TRUE	reported	u6dHsG
19	52378707	G	A	0.04088	0.008714	2.70E-06	0.008128	64942	NA	rs6210750	NA	UKB-b-95	TRUE	reported	u6dHsG
20	13467717	C	T	0.007577	0.001658	4.90E-06	0.685325	64942	NA	rs6042162	NA	UKB-b-95	TRUE	reported	u6dHsG
20	13493948	C	G	0.007562	0.001657	5.00E-06	0.684272	64942	NA	rs6042188	NA	UKB-b-95	TRUE	reported	u6dHsG
22	25793273	G	A	0.043006	0.009032	1.90E-06	0.007509	64942	NA	rs1179810	NA	UKB-b-95	TRUE	reported	u6dHsG

Red wine intake SNPs

chr.exposu	pos.exposu	other_allel	effect_allel	beta.expos	se.exposur	pval.expos	eaf.exposu	samplesize	ncase.expo	SNP	ncontrol.e:	exposure	mr_keep.e	pval_origir	id.exposure
1	1.76E+08	G	A	0.135479	0.027798	1.10E-06	0.016327	64949	NA	rs6180818	NA	UKB-b-13	TRUE	reported	HSB6ac
1	2.11E+08	A	C	0.074239	0.015888	3.00E-06	0.044234	64949	NA	rs6182865	NA	UKB-b-13	TRUE	reported	HSB6ac
2	2.33E+08	C	T	0.217623	0.045356	1.60E-06	0.00555	64949	NA	rs1507004	NA	UKB-b-13	TRUE	reported	HSB6ac
3	63440761	C	G	-0.20907	0.044351	2.40E-06	0.005974	64949	NA	rs1489983	NA	UKB-b-13	TRUE	reported	HSB6ac
3	86633182	G	A	0.21326	0.038881	4.10E-08	0.008215	64949	NA	rs1866822	NA	UKB-b-13	TRUE	reported	HSB6ac
3	86636645	G	A	0.211698	0.038718	4.60E-08	0.008223	64949	NA	rs3699079	NA	UKB-b-13	TRUE	reported	HSB6ac
3	86728792	T	G	0.228827	0.042476	7.20E-08	0.007179	64949	NA	rs1150766	NA	UKB-b-13	TRUE	reported	HSB6ac
3	86797676	G	C	0.175451	0.036854	1.90E-06	0.008719	64949	NA	rs1423650	NA	UKB-b-13	TRUE	reported	HSB6ac
3	1.61E+08	T	C	0.050723	0.010684	2.10E-06	0.103338	64949	NA	rs7316419	NA	UKB-b-13	TRUE	reported	HSB6ac
3	1.61E+08	G	A	0.050776	0.010684	2.00E-06	0.103343	64949	NA	rs1747866	NA	UKB-b-13	TRUE	reported	HSB6ac
3	1.61E+08	C	T	0.049987	0.010662	2.80E-06	0.103752	64949	NA	rs1747904	NA	UKB-b-13	TRUE	reported	HSB6ac
3	1.61E+08	T	C	0.048728	0.010644	4.70E-06	0.104213	64949	NA	rs2019095	NA	UKB-b-13	TRUE	reported	HSB6ac
3	1.61E+08	C	T	0.050028	0.010661	2.70E-06	0.103766	64949	NA	rs7316601	NA	UKB-b-13	TRUE	reported	HSB6ac
3	1.61E+08	G	A	0.050531	0.010661	2.10E-06	0.10377	64949	NA	rs7707777	NA	UKB-b-13	TRUE	reported	HSB6ac
3	1.61E+08	C	T	0.049679	0.010662	3.20E-06	0.103981	64949	NA	rs6788534	NA	UKB-b-13	TRUE	reported	HSB6ac
3	1.61E+08	C	T	0.050154	0.010674	2.60E-06	0.103741	64949	NA	rs7316603	NA	UKB-b-13	TRUE	reported	HSB6ac
3	1.61E+08	C	A	0.049072	0.010676	4.30E-06	0.103571	64949	NA	rs6686174	NA	UKB-b-13	TRUE	reported	HSB6ac
3	1.61E+08	G	A	0.049071	0.010676	4.30E-06	0.103572	64949	NA	rs6086126	NA	UKB-b-13	TRUE	reported	HSB6ac
3	1.61E+08	T	A	0.049071	0.010676	4.30E-06	0.103572	64949	NA	rs9828633	NA	UKB-b-13	TRUE	reported	HSB6ac
3	1.61E+08	G	A	0.049078	0.010677	4.30E-06	0.103572	64949	NA	rs7644500	NA	UKB-b-13	TRUE	reported	HSB6ac
3	1.61E+08	G	A	0.048779	0.010658	4.70E-06	0.104203	64949	NA	rs2048038	NA	UKB-b-13	TRUE	reported	HSB6ac
3	1.61E+08	G	T	0.048777	0.010658	4.70E-06	0.104203	64949	NA	rs2135449	NA	UKB-b-13	TRUE	reported	HSB6ac
3	1.61E+08	C	A	0.048755	0.010655	4.70E-06	0.104222	64949	NA	rs6800559	NA	UKB-b-13	TRUE	reported	HSB6ac
3	1.61E+08	T	C	0.048527	0.010595	4.70E-06	0.105515	64949	NA	rs6777722	NA	UKB-b-13	TRUE	reported	HSB6ac
3	1.61E+08	C	A	0.04853	0.010595	4.60E-06	0.105514	64949	NA	rs5911178	NA	UKB-b-13	TRUE	reported	HSB6ac
3	1.61E+08	C	T	0.048329	0.01059	5.00E-06	0.10557	64949	NA	rs6780945	NA	UKB-b-13	TRUE	reported	HSB6ac
3	1.61E+08	T	C	0.048528	0.010631	5.00E-06	0.104476	64949	NA	rs1742173	NA	UKB-b-13	TRUE	reported	HSB6ac
4	59979999	T	G	0.050776	0.010539	1.40E-06	0.892712	64949	NA	rs4860371	NA	UKB-b-13	TRUE	reported	HSB6ac
4	59980298	T	A	0.050864	0.010533	1.40E-06	0.892706	64949	NA	rs4860372	NA	UKB-b-13	TRUE	reported	HSB6ac
4	59982442	T	C	0.048948	0.01041	2.60E-06	0.887712	64949	NA	rs3942993	NA	UKB-b-13	TRUE	reported	HSB6ac
4	59987114	A	T	0.050686	0.010531	1.50E-06	0.892739	64949	NA	rs173356	NA	UKB-b-13	TRUE	reported	HSB6ac
4	59987608	G	A	0.049613	0.010538	2.50E-06	0.892838	64949	NA	rs357835	NA	UKB-b-13	TRUE	reported	HSB6ac
4	59987630	T	A	0.050716	0.010533	1.50E-06	0.892759	64949	NA	rs168574	NA	UKB-b-13	TRUE	reported	HSB6ac
4	59989165	T	C	0.050288	0.010534	1.80E-06	0.892826	64949	NA	rs357831	NA	UKB-b-13	TRUE	reported	HSB6ac
4	59989409	T	A	0.049665	0.010542	2.50E-06	0.893001	64949	NA	rs357830	NA	UKB-b-13	TRUE	reported	HSB6ac
4	59990496	A	G	0.050279	0.010534	1.80E-06	0.892829	64949	NA	rs357826	NA	UKB-b-13	TRUE	reported	HSB6ac
4	59990652	A	C	0.05029	0.010534	1.80E-06	0.892829	64949	NA	rs357825	NA	UKB-b-13	TRUE	reported	HSB6ac
4	59990722	C	T	0.050276	0.010534	1.80E-06	0.89283	64949	NA	rs357824	NA	UKB-b-13	TRUE	reported	HSB6ac
4	59995118	T	C	0.049849	0.010536	2.20E-06	0.892929	64949	NA	rs6551598	NA	UKB-b-13	TRUE	reported	HSB6ac
4	59995208	T	C	0.049874	0.01054	2.20E-06	0.892947	64949	NA	rs6551599	NA	UKB-b-13	TRUE	reported	HSB6ac
4	59995851	A	C	0.049864	0.010537	2.20E-06	0.892928	64949	NA	rs3114022	NA	UKB-b-13	TRUE	reported	HSB6ac
4	1E+08	T	C	0.116844	0.019127	1.00E-09	0.970816	64949	NA	rs1229984	NA	UKB-b-13	TRUE	reported	HSB6ac
4	1.68E+08	C	T	0.066382	0.014518	4.80E-06	0.053226	64949	NA	rs1164783	NA	UKB-b-13	TRUE	reported	HSB6ac
5	1.53E+08	T	C	0.057386	0.010779	1.00E-07	0.898057	64949	NA	rs514336	NA	UKB-b-13	TRUE	reported	HSB6ac
5	1.53E+08	C	G	0.060187	0.011314	1.00E-07	0.908853	64949	NA	rs475583	NA	UKB-b-13	TRUE	reported	HSB6ac
5	1.53E+08	C	T	0.058012	0.010746	6.70E-08	0.89771	64949	NA	rs566577	NA	UKB-b-13	TRUE	reported	HSB6ac
5	1.53E+08	A	G	-0.05754	0.010723	8.00E-08	0.102744	64949	NA	rs1493395	NA	UKB-b-13	TRUE	reported	HSB6ac
5	1.53E+08	A	T	-0.0577	0.01073	7.60E-08	0.102434	64949	NA	rs1908100	NA	UKB-b-13	TRUE	reported	HSB6ac
5	1.53E+08	G	C	-0.05119	0.010352	7.60E-07	0.11132	64949	NA	rs4958345	NA	UKB-b-13	TRUE	reported	HSB6ac
5	1.53E+08	G	A	-0.05019	0.010387	1.40E-06	0.110443	64949	NA	rs1265610	NA	UKB-b-13	TRUE	reported	HSB6ac
5	1.53E+08	G	A	-0.04991	0.010408	1.60E-06	0.110438	64949	NA	rs4958347	NA	UKB-b-13	TRUE	reported	HSB6ac
6	42764931	A	G	0.151991	0.033067	4.30E-06	0.01095	64949	NA	rs1149660	NA	UKB-b-13	TRUE	reported	HSB6ac
6	42804768	C	G	0.180399	0.039126	4.00E-06	0.008096	64949	NA	rs5601681	NA	UKB-b-13	TRUE	reported	HSB6ac
8	1.35E+08	C	T	0.04245	0.009113	3.20E-06	0.151749	64949	NA	rs7820063	NA	UKB-b-13	TRUE	reported	HSB6ac
13	28958955	C	A	0.031476	0.006533	1.50E-06	0.458991	64949	NA	rs3751395	NA	UKB-b-13	TRUE	reported	HSB6ac
22	21358522	C	T	0.041485	0.008942	3.50E-06	0.839938	64949	NA	rs430305	NA	UKB-b-13	TRUE	reported	HSB6ac
22	21359510	A	G	0.041844	0.00904	3.70E-06	0.844411	64949	NA	rs401639	NA	UKB-b-13	TRUE	reported	HSB6ac
22	21359706	A	G	0.042838	0.008973	1.80E-06	0.841475	64949	NA	rs402236	NA	UKB-b-13	TRUE	reported	HSB6ac
22	21360342	A	G	0.042002	0.009039	3.40E-06	0.844545	64949	NA	rs1210557	NA	UKB-b-13	TRUE	reported	HSB6ac
22	21360350	A	C	0.041976	0.009039	3.40E-06	0.844552	64949	NA	rs1210558	NA	UKB-b-13	TRUE	reported	HSB6ac
22	21361060	T	G	0.042378	0.009034	2.70E-06	0.844601	64949	NA	rs428214	NA	UKB-b-13	TRUE	reported	HSB6ac
22	21363452	A	G	0.0423	0.008971	2.40E-06	0.841532	64949	NA	rs378449	NA	UKB-b-13	TRUE	reported	HSB6ac
22	21364849	T	C	0.041804	0.009048	3.80E-06	0.844592	64949	NA	rs395938	NA	UKB-b-13	TRUE	reported	HSB6ac
22	21365668	A	G	0.041903	0.009044	3.60E-06	0.844013	64949	NA	rs1210564	NA	UKB-b-13	TRUE	reported	HSB6ac
22	21366110	T	C	0.041851	0.009045	3.70E-06	0.84411	64949	NA	rs412233	NA	UKB-b-13	TRUE	reported	HSB6ac
22	21367464	C	G	-0.04151	0.00904	4.40E-06	0.15636	64949	NA	rs3761411	NA	UKB-b-13	TRUE	reported	HSB6ac
22	21367713	G	C	-0.04294	0.009305	3.90E-06	0.146938	64949	NA	rs3761414	NA	UKB-b-13	TRUE	reported	HSB6ac
22	30592915	C	T	0.131867	0.028034	2.60E-06	0.01496	64949	NA	rs1498028	NA	UKB-b-13	TRUE	reported	HSB6ac

Spirits intake SNPs

chr.exposu	pos.exposu	other_allel	effect_allel	beta.expos	se.exposur	pval.expos	eaf.exposu	samplesize	ncase.expo	SNP	ncontrol.e	exposure	mr_keep.e	pval_origir	id.exposure
1	60233431	T	C	0.116515	0.023268	5.50E-07	0.00833	64949	NA	rs7487810	NA	UKB-b-37	TRUE	reported	yQBPeT
1	89528115	T	G	0.114512	0.024827	4.00E-06	0.007546	64949	NA	rs1489884	NA	UKB-b-37	TRUE	reported	yQBPeT
1	1.1E+08	A	T	0.06678	0.013098	3.40E-07	0.027872	64949	NA	rs7268952	NA	UKB-b-37	TRUE	reported	yQBPeT
1	2.15E+08	T	C	-0.07412	0.015979	3.50E-06	0.01933	64949	NA	rs1167565	NA	UKB-b-37	TRUE	reported	yQBPeT
2	669057	C	A	0.144751	0.029288	7.70E-07	0.005705	64949	NA	rs1851250	NA	UKB-b-37	TRUE	reported	yQBPeT
2	17371575	G	A	0.07475	0.016193	3.90E-06	0.017483	64949	NA	rs7800543	NA	UKB-b-37	TRUE	reported	yQBPeT
2	1.7E+08	T	G	0.121354	0.02507	1.30E-06	0.008906	64949	NA	rs1446265	NA	UKB-b-37	TRUE	reported	yQBPeT
2	1.71E+08	A	G	0.117562	0.025663	4.60E-06	0.008334	64949	NA	rs1477251	NA	UKB-b-37	TRUE	reported	yQBPeT
2	1.84E+08	G	A	0.1083	0.023559	4.30E-06	0.009298	64949	NA	rs1391643	NA	UKB-b-37	TRUE	reported	yQBPeT
2	1.84E+08	A	G	0.108612	0.023634	4.30E-06	0.009255	64949	NA	rs1735680	NA	UKB-b-37	TRUE	reported	yQBPeT
2	2.17E+08	G	A	0.086754	0.018842	4.10E-06	0.013207	64949	NA	rs1141122	NA	UKB-b-37	TRUE	reported	yQBPeT
3	69518546	T	C	-0.03103	0.006203	5.70E-07	0.135657	64949	NA	rs6797592	NA	UKB-b-37	TRUE	reported	yQBPeT
3	69518867	A	G	-0.03105	0.006238	6.40E-07	0.133888	64949	NA	rs5597674	NA	UKB-b-37	TRUE	reported	yQBPeT
3	69520479	T	C	-0.03119	0.006233	5.60E-07	0.134012	64949	NA	rs9828963	NA	UKB-b-37	TRUE	reported	yQBPeT
3	69521243	A	G	-0.03115	0.006231	5.80E-07	0.134007	64949	NA	rs9310158	NA	UKB-b-37	TRUE	reported	yQBPeT
3	69521568	T	C	-0.03115	0.006231	5.80E-07	0.134008	64949	NA	rs9834457	NA	UKB-b-37	TRUE	reported	yQBPeT
3	69523118	A	G	-0.03103	0.006239	6.60E-07	0.133582	64949	NA	rs1763373	NA	UKB-b-37	TRUE	reported	yQBPeT
3	69523254	G	A	-0.03087	0.006234	7.30E-07	0.133911	64949	NA	rs7293552	NA	UKB-b-37	TRUE	reported	yQBPeT
3	69523487	T	A	-0.03088	0.006234	7.30E-07	0.13391	64949	NA	rs9816753	NA	UKB-b-37	TRUE	reported	yQBPeT
3	69523979	C	T	-0.03141	0.006251	5.00E-07	0.134062	64949	NA	rs9869412	NA	UKB-b-37	TRUE	reported	yQBPeT
3	69524149	G	A	-0.0316	0.006253	4.30E-07	0.134039	64949	NA	rs9834677	NA	UKB-b-37	TRUE	reported	yQBPeT
3	69529609	T	C	-0.03138	0.006261	5.40E-07	0.133674	64949	NA	rs5911305	NA	UKB-b-37	TRUE	reported	yQBPeT
3	69529724	G	T	-0.03152	0.006264	4.90E-07	0.133412	64949	NA	rs5826831	NA	UKB-b-37	TRUE	reported	yQBPeT
3	69529778	G	A	-0.03154	0.006272	4.90E-07	0.133089	64949	NA	rs1436536	NA	UKB-b-37	TRUE	reported	yQBPeT
3	69529950	C	T	-0.03149	0.006259	4.90E-07	0.133823	64949	NA	rs9826150	NA	UKB-b-37	TRUE	reported	yQBPeT
3	69530268	C	A	-0.03138	0.006261	5.40E-07	0.133669	64949	NA	rs6162316	NA	UKB-b-37	TRUE	reported	yQBPeT
3	69530315	G	C	-0.03138	0.006261	5.40E-07	0.133669	64949	NA	rs5917648	NA	UKB-b-37	TRUE	reported	yQBPeT
3	69531499	G	A	-0.03305	0.006345	1.90E-07	0.130529	64949	NA	rs9310159	NA	UKB-b-37	TRUE	reported	yQBPeT
3	69531735	C	G	-0.03183	0.00627	3.80E-07	0.133491	64949	NA	rs1051097	NA	UKB-b-37	TRUE	reported	yQBPeT
3	69533040	T	G	-0.03176	0.00627	4.10E-07	0.133585	64949	NA	rs1051097	NA	UKB-b-37	TRUE	reported	yQBPeT
3	69533066	A	G	-0.03174	0.006271	4.20E-07	0.13356	64949	NA	rs7681738	NA	UKB-b-37	TRUE	reported	yQBPeT
3	69533292	G	A	-0.03162	0.006274	4.70E-07	0.133453	64949	NA	rs1768520	NA	UKB-b-37	TRUE	reported	yQBPeT
3	69533546	G	A	-0.03187	0.006269	3.70E-07	0.133669	64949	NA	rs7825994	NA	UKB-b-37	TRUE	reported	yQBPeT
3	69533961	C	T	-0.03144	0.006271	5.30E-07	0.133588	64949	NA	rs7672664	NA	UKB-b-37	TRUE	reported	yQBPeT
3	69539256	G	T	-0.02985	0.006411	3.20E-06	0.127818	64949	NA	rs7952638	NA	UKB-b-37	TRUE	reported	yQBPeT
4	2775411	G	A	0.06682	0.014503	4.10E-06	0.021925	64949	NA	rs7160823	NA	UKB-b-37	TRUE	reported	yQBPeT
4	13929278	A	G	0.09963	0.020518	1.20E-06	0.01175	64949	NA	rs1380080	NA	UKB-b-37	TRUE	reported	yQBPeT
4	13971017	C	T	0.085809	0.017791	1.40E-06	0.015112	64949	NA	rs1499907	NA	UKB-b-37	TRUE	reported	yQBPeT
4	14002768	C	G	0.084581	0.017862	2.20E-06	0.014523	64949	NA	rs1507493	NA	UKB-b-37	TRUE	reported	yQBPeT
4	69788637	T	A	0.069265	0.014464	1.70E-06	0.024502	64949	NA	rs1435394	NA	UKB-b-37	TRUE	reported	yQBPeT
5	94960359	C	G	0.119219	0.024642	1.30E-06	0.007909	64949	NA	rs1479303	NA	UKB-b-37	TRUE	reported	yQBPeT
5	1.61E+08	A	G	0.032178	0.007004	4.30E-06	0.102057	64949	NA	rs7733227	NA	UKB-b-37	TRUE	reported	yQBPeT
5	1.61E+08	C	T	0.032475	0.007012	3.60E-06	0.101776	64949	NA	rs4608967	NA	UKB-b-37	TRUE	reported	yQBPeT
6	44290731	G	A	0.118912	0.025757	3.90E-06	0.007592	64949	NA	rs1813139	NA	UKB-b-37	TRUE	reported	yQBPeT
6	45830188	A	T	0.062982	0.013482	3.00E-06	0.027293	64949	NA	rs1390454	NA	UKB-b-37	TRUE	reported	yQBPeT
6	1.12E+08	G	T	0.059035	0.012679	3.20E-06	0.030594	64949	NA	rs7899891	NA	UKB-b-37	TRUE	reported	yQBPeT
6	1.34E+08	A	C	-0.02061	0.004489	4.40E-06	0.662242	64949	NA	rs7746271	NA	UKB-b-37	TRUE	reported	yQBPeT
7	1.1E+08	T	C	0.100447	0.021281	2.40E-06	0.011055	64949	NA	rs1509814	NA	UKB-b-37	TRUE	reported	yQBPeT
8	1824441	G	C	0.107984	0.023583	4.70E-06	0.008547	64949	NA	rs3517186	NA	UKB-b-37	TRUE	reported	yQBPeT
8	3174335	G	T	0.137861	0.029635	3.30E-06	0.005473	64949	NA	rs1459461	NA	UKB-b-37	TRUE	reported	yQBPeT
9	1.32E+08	A	G	0.031595	0.006803	3.40E-06	0.110459	64949	NA	rs6258560	NA	UKB-b-37	TRUE	reported	yQBPeT
9	1.32E+08	C	G	0.032	0.006814	2.70E-06	0.110127	64949	NA	rs7887698	NA	UKB-b-37	TRUE	reported	yQBPeT
9	1.32E+08	G	A	0.031522	0.006838	4.00E-06	0.107415	64949	NA	rs1156410	NA	UKB-b-37	TRUE	reported	yQBPeT
12	57047425	G	A	0.068378	0.014489	2.40E-06	0.024674	64949	NA	rs1404806	NA	UKB-b-37	TRUE	reported	yQBPeT
13	37444072	G	C	0.097122	0.020338	1.80E-06	0.012655	64949	NA	rs7629659	NA	UKB-b-37	TRUE	reported	yQBPeT
14	46782056	A	G	0.023098	0.004676	7.80E-07	0.298063	64949	NA	rs6200119	NA	UKB-b-37	TRUE	reported	yQBPeT
15	61958774	C	A	0.084972	0.018273	3.30E-06	0.015414	64949	NA	rs1382423	NA	UKB-b-37	TRUE	reported	yQBPeT
16	17560713	T	A	0.070165	0.015282	4.40E-06	0.021871	64949	NA	rs1172035	NA	UKB-b-37	TRUE	reported	yQBPeT
17	77791361	T	C	0.087062	0.017916	1.20E-06	0.015616	64949	NA	rs1414153	NA	UKB-b-37	TRUE	reported	yQBPeT
19	3020228	T	C	-0.01997	0.004361	4.60E-06	0.401282	64949	NA	rs1041648	NA	UKB-b-37	TRUE	reported	yQBPeT
20	2223654	T	C	-0.03071	0.006544	2.70E-06	0.119489	64949	NA	rs3603809	NA	UKB-b-37	TRUE	reported	yQBPeT
20	2223831	T	C	-0.03057	0.006506	2.60E-06	0.120364	64949	NA	rs7264096	NA	UKB-b-37	TRUE	reported	yQBPeT
20	7169419	A	G	0.0329	0.006725	1.00E-06	0.111819	64949	NA	rs1248071	NA	UKB-b-37	TRUE	reported	yQBPeT
20	43311647	G	A	0.068658	0.014814	3.60E-06	0.022749	64949	NA	rs6220456	NA	UKB-b-37	TRUE	reported	yQBPeT

Other alcohol intake SNPs

chr.exposu	pos.exposu	other_allel	effect_allel	beta.expos	se.exposur	pval.expos	eaf.exposu	samplesize	ncase.expo	SNP	ncontrol.e	exposure	mr_keep.e	pval_origir	id.exposure
1	23234946	A	G	0.006934	0.001506	4.10E-06	0.531781	64944	NA	rs309499	NA	UKB-b-12	TRUE	reported	dNl64d
1	23236192	T	G	0.006934	0.001507	4.20E-06	0.531729	64944	NA	rs309498	NA	UKB-b-12	TRUE	reported	dNl64d
1	23242629	T	C	0.007181	0.001516	2.20E-06	0.525347	64944	NA	rs309494	NA	UKB-b-12	TRUE	reported	dNl64d
1	1.83E+08	C	T	0.023669	0.004513	1.60E-07	0.029641	64944	NA	rs1417236	NA	UKB-b-12	TRUE	reported	dNl64d
1	1.83E+08	C	T	0.02344	0.004485	1.70E-07	0.030048	64944	NA	rs1753679	NA	UKB-b-12	TRUE	reported	dNl64d
1	1.98E+08	A	G	0.03298	0.006793	1.20E-06	0.012388	64944	NA	rs7875283	NA	UKB-b-12	TRUE	reported	dNl64d
1	2.07E+08	G	A	0.045973	0.008758	1.50E-07	0.008728	64944	NA	rs4129990	NA	UKB-b-12	TRUE	reported	dNl64d
2	40323046	C	T	0.026815	0.005614	1.80E-06	0.018609	64944	NA	rs1153529	NA	UKB-b-12	TRUE	reported	dNl64d
2	40348333	C	G	0.029785	0.005766	2.40E-07	0.017519	64944	NA	rs1512249	NA	UKB-b-12	TRUE	reported	dNl64d
2	40405782	T	A	0.033203	0.006087	4.90E-08	0.015513	64944	NA	rs1153319	NA	UKB-b-12	TRUE	reported	dNl64d
2	57393357	A	G	0.042182	0.008903	2.20E-06	0.008007	64944	NA	rs1393460	NA	UKB-b-12	TRUE	reported	dNl64d
2	60154977	G	A	0.046885	0.009901	2.20E-06	0.006502	64944	NA	rs1855720	NA	UKB-b-12	TRUE	reported	dNl64d
2	62516445	C	T	0.05183	0.010656	1.20E-06	0.005911	64944	NA	rs1879434	NA	UKB-b-12	TRUE	reported	dNl64d
2	62701432	T	C	0.045725	0.00962	2.00E-06	0.006781	64944	NA	rs1489608	NA	UKB-b-12	TRUE	reported	dNl64d
2	62742451	A	G	0.056882	0.010748	1.20E-07	0.005547	64944	NA	rs1871198	NA	UKB-b-12	TRUE	reported	dNl64d
2	63016818	A	G	0.055913	0.010704	1.80E-07	0.005392	64944	NA	rs1923832	NA	UKB-b-12	TRUE	reported	dNl64d
2	63662288	C	T	0.057564	0.009854	5.20E-09	0.006169	64944	NA	rs1877304	NA	UKB-b-12	TRUE	reported	dNl64d
2	63669177	G	A	0.059384	0.009825	1.50E-09	0.006191	64944	NA	rs1486690	NA	UKB-b-12	TRUE	reported	dNl64d
2	63969281	C	T	0.045327	0.009349	1.20E-06	0.006926	64944	NA	rs5568680	NA	UKB-b-12	TRUE	reported	dNl64d
2	64260620	C	A	0.049039	0.009356	1.60E-07	0.00666	64944	NA	rs1910017	NA	UKB-b-12	TRUE	reported	dNl64d
2	64265562	G	A	0.049025	0.009356	1.60E-07	0.006661	64944	NA	rs1929339	NA	UKB-b-12	TRUE	reported	dNl64d
2	64321598	C	T	0.050079	0.009551	1.60E-07	0.006357	64944	NA	rs1406347	NA	UKB-b-12	TRUE	reported	dNl64d
2	1.31E+08	C	T	0.035186	0.006827	2.60E-07	0.012465	64944	NA	rs1105335	NA	UKB-b-12	TRUE	reported	dNl64d
2	1.31E+08	G	A	0.034216	0.006777	4.40E-07	0.012595	64944	NA	rs7298456	NA	UKB-b-12	TRUE	reported	dNl64d
2	1.31E+08	A	G	0.035095	0.007109	7.90E-07	0.011958	64944	NA	rs5846461	NA	UKB-b-12	TRUE	reported	dNl64d
2	1.42E+08	G	T	0.041516	0.008067	2.70E-07	0.009882	64944	NA	rs1443071	NA	UKB-b-12	TRUE	reported	dNl64d
2	1.72E+08	C	A	0.036413	0.007954	4.70E-06	0.011201	64944	NA	rs1500696	NA	UKB-b-12	TRUE	reported	dNl64d
2	1.8E+08	C	T	0.034677	0.007533	4.20E-06	0.01091	64944	NA	rs1138849	NA	UKB-b-12	TRUE	reported	dNl64d
2	2.07E+08	G	A	0.052156	0.010775	1.30E-06	0.005682	64944	NA	rs1812386	NA	UKB-b-12	TRUE	reported	dNl64d
3	23862494	T	G	0.04551	0.009825	3.60E-06	0.007197	64944	NA	rs1402663	NA	UKB-b-12	TRUE	reported	dNl64d
3	55062334	A	G	0.007032	0.001507	3.10E-06	0.481087	64944	NA	rs358054	NA	UKB-b-12	TRUE	reported	dNl64d
3	64013417	G	A	0.009305	0.002011	3.70E-06	0.168685	64944	NA	rs35839	NA	UKB-b-12	TRUE	reported	dNl64d
3	64130618	T	A	0.01393	0.002998	3.40E-06	0.068068	64944	NA	rs2008984	NA	UKB-b-12	TRUE	reported	dNl64d
3	1.47E+08	T	C	0.031808	0.006948	4.70E-06	0.01252	64944	NA	rs1155928	NA	UKB-b-12	TRUE	reported	dNl64d
3	1.47E+08	T	C	0.044734	0.009286	1.50E-06	0.007565	64944	NA	rs1810451	NA	UKB-b-12	TRUE	reported	dNl64d
3	1.47E+08	T	C	0.03874	0.008245	2.60E-06	0.008758	64944	NA	rs1822241	NA	UKB-b-12	TRUE	reported	dNl64d
3	1.56E+08	G	A	0.046762	0.009572	1.00E-06	0.006599	64944	NA	rs1453387	NA	UKB-b-12	TRUE	reported	dNl64d
3	1.59E+08	G	A	0.039633	0.00779	3.60E-07	0.010863	64944	NA	rs1146102	NA	UKB-b-12	TRUE	reported	dNl64d
4	6412659	G	A	0.040568	0.007871	2.50E-07	0.009613	64944	NA	rs1314810	NA	UKB-b-12	TRUE	reported	dNl64d
4	6414957	G	A	0.045081	0.007399	1.10E-09	0.010529	64944	NA	rs5635153	NA	UKB-b-12	TRUE	reported	dNl64d
4	6415241	G	A	0.04248	0.007357	7.70E-09	0.010614	64944	NA	rs1311239	NA	UKB-b-12	TRUE	reported	dNl64d
4	6415357	G	A	0.045262	0.007353	7.50E-10	0.010636	64944	NA	rs3453705	NA	UKB-b-12	TRUE	reported	dNl64d
4	6416086	G	A	0.044349	0.007458	2.70E-09	0.010282	64944	NA	rs3437352	NA	UKB-b-12	TRUE	reported	dNl64d
4	6417426	C	T	0.042613	0.007524	1.50E-08	0.010118	64944	NA	rs1312904	NA	UKB-b-12	TRUE	reported	dNl64d
4	6417739	C	A	0.043566	0.0075	6.30E-09	0.010194	64944	NA	rs8000761	NA	UKB-b-12	TRUE	reported	dNl64d
4	6418069	G	A	0.042393	0.007595	2.40E-08	0.009904	64944	NA	rs7159989	NA	UKB-b-12	TRUE	reported	dNl64d
4	6419370	G	A	0.043794	0.007684	1.20E-08	0.009706	64944	NA	rs1433304	NA	UKB-b-12	TRUE	reported	dNl64d
4	6419573	C	T	0.043711	0.007677	1.20E-08	0.00974	64944	NA	rs3415638	NA	UKB-b-12	TRUE	reported	dNl64d
4	6419945	G	A	0.04504	0.007668	4.30E-09	0.009779	64944	NA	rs3553852	NA	UKB-b-12	TRUE	reported	dNl64d
4	6422020	C	T	0.042298	0.00736	9.10E-09	0.01072	64944	NA	rs3569205	NA	UKB-b-12	TRUE	reported	dNl64d
4	6422457	G	A	-0.04354	0.007067	7.20E-10	0.988346	64944	NA	rs878282	NA	UKB-b-12	TRUE	reported	dNl64d
4	6423246	C	T	0.04016	0.007324	4.20E-08	0.01084	64944	NA	rs3576070	NA	UKB-b-12	TRUE	reported	dNl64d
4	6423895	G	A	0.045695	0.007601	1.80E-09	0.010049	64944	NA	rs1000670	NA	UKB-b-12	TRUE	reported	dNl64d
4	6424101	C	T	0.047006	0.007668	8.80E-10	0.009864	64944	NA	rs3497428	NA	UKB-b-12	TRUE	reported	dNl64d
4	59107387	G	A	0.027218	0.005949	4.80E-06	0.018556	64944	NA	rs1841443	NA	UKB-b-12	TRUE	reported	dNl64d
4	79441337	T	C	0.028484	0.006105	3.10E-06	0.015452	64944	NA	rs1747065	NA	UKB-b-12	TRUE	reported	dNl64d
4	79646819	G	A	0.029362	0.006404	4.50E-06	0.014549	64944	NA	rs1388119	NA	UKB-b-12	TRUE	reported	dNl64d
4	79877172	T	G	0.017176	0.003474	7.60E-07	0.051652	64944	NA	rs7745801	NA	UKB-b-12	TRUE	reported	dNl64d
4	79878068	G	T	0.017231	0.003477	7.20E-07	0.051608	64944	NA	rs7610583	NA	UKB-b-12	TRUE	reported	dNl64d
4	1.34E+08	G	A	0.007441	0.001534	1.20E-06	0.577499	64944	NA	rs1058267	NA	UKB-b-12	TRUE	reported	dNl64d
4	1.34E+08	C	A	0.0077	0.001514	3.70E-07	0.573094	64944	NA	rs1310374	NA	UKB-b-12	TRUE	reported	dNl64d
4	1.34E+08	C	T	0.007763	0.00152	3.30E-07	0.5774	64944	NA	rs6846610	NA	UKB-b-12	TRUE	reported	dNl64d
4	1.34E+08	A	T	0.007705	0.001516	3.70E-07	0.573449	64944	NA	rs1311006	NA	UKB-b-12	TRUE	reported	dNl64d
4	1.34E+08	C	A	0.007911	0.001521	2.00E-07	0.576342	64944	NA	rs7695589	NA	UKB-b-12	TRUE	reported	dNl64d
4	1.36E+08	T	C	0.026027	0.005674	4.50E-06	0.019152	64944	NA	rs2869089	NA	UKB-b-12	TRUE	reported	dNl64d
5	1888972	C	G	0.048798	0.009832	6.90E-07	0.006101	64944	NA	rs5476360	NA	UKB-b-12	TRUE	reported	dNl64d
5	57988014	T	C	0.045386	0.009075	5.70E-07	0.007339	64944	NA	rs1147836	NA	UKB-b-12	TRUE	reported	dNl64d
6	23421784	C	T	0.01685	0.003585	2.60E-06	0.046099	64944	NA	rs1157159	NA	UKB-b-12	TRUE	reported	dNl64d
6	23436016	A	C	0.017736	0.003591	7.80E-07	0.046571	64944	NA	rs9466782	NA	UKB-b-12	TRUE	reported	dNl64d
6	1.31E+08	A	T	0.067204	0.010371	9.20E-11	0.005642	64944	NA	rs1406005	NA	UKB-b-12	TRUE	reported	dNl64d
6	1.32E+08	G	A	0.049643	0.009934	5.80E-07	0.006523	64944	NA	rs1453449	NA	UKB-b-12	TRUE	reported	dNl64d
6	1.32E+08	T	C	0.059333	0.010297	8.30E-09	0.006247	64944	NA	rs3761500	NA	UKB-b-12	TRUE	reported	dNl64d
6	1.32E+08	C	T	0.066511	0.010427	1.80E-10	0.006195	64944	NA	rs3742778	NA	UKB-b-12	TRUE	reported	dNl64d
7	30269237	C	T	0.038028	0.008229	3.80E-06	0.008646	64944	NA	rs1137977	NA	UKB-b-12	TRUE	reported	dNl64d
7	30430070	T	C	0.039389	0.007755	3.80E-07	0.009462	64944	NA	rs7804909	NA	UKB-b-12	TRUE	reported	dNl64d
7	32149064	C	A	0.012219	0.002669	4.70E-06	0.086351	64944	NA	rs1176604	NA	UKB-b-12	TRUE	reported	dNl64d
7	32161314	G	T	0.01693	0.003553	1.90E-06	0.047889	64944	NA	rs1401980	NA	UKB-b-12	TRUE	reported	dNl64d
7	66209079	A	T	0.049352	0.010709	4.10E-06	0.006091	64944	NA	rs1497065	NA	UKB-b-12	TRUE	reported	dNl64d
7	66232244	C	G	0.049367	0.01071	4.00E-06	0.006089	64944	NA	rs5618030	NA	UKB-b-12	TRUE	reported	dNl64d
7	66232378	G	T	0.048931	0.010686	4.70E-06	0.006132	64944	NA	rs1504337	NA	UKB-b-12	TRUE	reported	dNl64d
7	75198539	A	T	0.049088	0.010531	3.10E-06	0.006127	64944	NA	rs5877181	NA	UKB-b-12	TRUE	reported	dNl64d
7	75287299	A	T	0.057669	0.010047	9.50E-09	0.00605	64944	NA	rs1476533	NA	UKB-b-12	TRUE	reported	dNl64d
7	75333248	G	A	0.056663	0.010199	2.80E-08	0.00584	64944	NA	rs1511777	NA	UKB-b-12	TRUE	reported	dNl64d
7	96111835	T	C	0.040493	0.007999	4.10E-07	0.009677	64944	NA	rs1868610	NA	UKB-b-12	TRUE	reported	dNl64d
7	1.47E+08	T	C	0.039787	0.008684	4.60E-06	0.008526	64944	NA	rs1174320	NA	UKB-b-12	TRUE	reported	dNl64d
8	5383932	G	C	0.032697	0.007096	4.10E-06	0.011526	64944	NA	rs1403347	NA	UKB-b-12	TRUE	reported	dNl64d
8	10927599	G	A	0.022813	0.004845	2.50E-06	0.024552	64944	NA	rs1173484	NA	UKB-b-12	TRUE	reported	dNl64d
8	10984445	C	T	0.02392	0.005049	2.20E-06	0.022596	64944	NA	rs1174812	NA	UKB-b-12	TRUE	reported	dNl64d
9	2943225	G	A	0.01651	0.003497	2.30E-06	0.049916	64944	NA	rs7435629	NA	UKB-b-12	TRUE	reported	dNl64d
9	93929150	G	C	0.047705	0.009927	1.50E-06	0.006286	64944	NA	rs1099179	NA	UKB-b-12	TRUE	reported	dNl64d
9	93955564	G	T	0.045683	0.009924	4.20E-06	0.006254	64944	NA	rs5676656	NA	UKB-b-12	TRUE	reported	dNl64d
9	94266315	T	C	0.015942	0.003448	3.80E-06	0.053307	64944	NA	rs7764117	NA	UKB-b-12	TRUE	reported	dNl64d
10	46218621	C	T	0.029082	0.006355	4.70E-06	0.014726	64944	NA	rs1383557	NA	UKB-b-12	TRUE	reported	dNl64d
10	89920474	C	T	0.032258	0.006908	3.00E-06	0.01198	64944	NA	rs8002446	NA	UKB-b-12	TRUE	reported	dNl64d
10	98974849	C	T	0.007384	0.001618	5.00E-06	0.323538	64944	NA	rs701810	NA	UKB-b-12	TRUE	reported	dNl64d
10	98975413	A	G	0.007398	0.001618	4.80E-06	0.323446	64944	NA	rs701812	NA	UKB-b-12	TRUE	reported	dNl64d
10	98975632	C	T	0.007473	0.001618	3.80E-06	0.323127	64944	NA	rs701813	NA	UKB-b-12	TRUE	reported	dNl64d
10	98975719	G	A	0.007428	0.001617	4.40E-06	0.323232	64944	NA	rs701814	NA	UKB-b-12	TRUE	reported	dNl64d
10	98975893	A	C	0.007544	0.001619	3.20E-06	0.321806	64944	NA	rs701815	NA	UKB-b-12	TRUE	reported	dNl64d
10	98976923	G	A	0.007516	0.001619	3.50E-06	0.320646	64944	NA	rs1687369	NA	UKB-b-12	TRUE	reported	dNl64d
10	98977250	C	T	0.007514	0.001619	3.50E-06	0.320688	64944	NA	rs701816	NA	UKB-b-12	TRUE	reported	dNl64d
10	98977329	G	C	0.007571	0.001618	2.90E-06	0.321656	64944	NA	rs701817	NA	UKB-b-12	TRUE	reported	dNl64d
10	98981643	T	C	0.007544	0.001618	3.10E-06	0.320576	64944	NA	rs1147600	NA	UKB-b-12	TRUE	reported	dNl64d

10	98986316	C	A	0.007582	0.001615	2.70E-06	0.3204	64944	NA	rs701819	NA	UKB-b-12	TRUE	reported	dNl64d
10	98987522	T	C	0.007587	0.001614	2.60E-06	0.32037	64944	NA	rs793517	NA	UKB-b-12	TRUE	reported	dNl64d
10	98987985	G	T	0.007576	0.001614	2.70E-06	0.320472	64944	NA	rs793516	NA	UKB-b-12	TRUE	reported	dNl64d
10	98995076	A	G	0.007588	0.001623	3.00E-06	0.312919	64944	NA	rs946981	NA	UKB-b-12	TRUE	reported	dNl64d
10	98998266	C	T	0.007419	0.001621	4.70E-06	0.314213	64944	NA	rs2484881	NA	UKB-b-12	TRUE	reported	dNl64d
10	98998355	T	C	0.007419	0.001622	4.80E-06	0.314227	64944	NA	rs2484880	NA	UKB-b-12	TRUE	reported	dNl64d
10	98999213	T	C	0.007425	0.001622	4.70E-06	0.314196	64944	NA	rs3478787	NA	UKB-b-12	TRUE	reported	dNl64d
10	99006083	G	A	0.00755	0.001623	3.30E-06	0.31331	64944	NA	rs13439	NA	UKB-b-12	TRUE	reported	dNl64d
10	99024154	G	A	0.007463	0.001623	4.20E-06	0.314088	64944	NA	rs796980	NA	UKB-b-12	TRUE	reported	dNl64d
10	99024572	A	G	0.007463	0.001623	4.30E-06	0.31409	64944	NA	rs793524	NA	UKB-b-12	TRUE	reported	dNl64d
10	99071242	G	T	0.007834	0.001642	1.80E-06	0.302975	64944	NA	rs7091162	NA	UKB-b-12	TRUE	reported	dNl64d
10	99071282	T	C	0.007441	0.001629	4.90E-06	0.308945	64944	NA	rs7074896	NA	UKB-b-12	TRUE	reported	dNl64d
10	1.35E+08	C	T	-0.007	0.001504	3.20E-06	0.511306	64944	NA	rs4880272	NA	UKB-b-12	TRUE	reported	dNl64d
10	1.35E+08	G	A	-0.00699	0.001499	3.20E-06	0.497613	64944	NA	rs4880425	NA	UKB-b-12	TRUE	reported	dNl64d
11	2430390	C	T	0.024788	0.005027	8.20E-07	0.022881	64944	NA	rs1228092	NA	UKB-b-12	TRUE	reported	dNl64d
11	4190902	C	A	0.041052	0.008085	3.80E-07	0.008839	64944	NA	rs1503354	NA	UKB-b-12	TRUE	reported	dNl64d
11	4309947	T	A	0.03867	0.008022	1.40E-06	0.009315	64944	NA	rs5709951	NA	UKB-b-12	TRUE	reported	dNl64d
11	4326252	T	G	0.038286	0.007995	1.70E-06	0.009413	64944	NA	rs1911893	NA	UKB-b-12	TRUE	reported	dNl64d
11	7914158	C	G	0.037921	0.007913	1.60E-06	0.010177	64944	NA	rs7959955	NA	UKB-b-12	TRUE	reported	dNl64d
11	10683151	C	A	0.040556	0.008772	3.80E-06	0.008499	64944	NA	rs1889508	NA	UKB-b-12	TRUE	reported	dNl64d
11	14031266	A	G	0.031534	0.006838	4.00E-06	0.01299	64944	NA	rs1171537	NA	UKB-b-12	TRUE	reported	dNl64d
11	23594770	T	C	0.037827	0.007894	1.70E-06	0.00935	64944	NA	rs1380804	NA	UKB-b-12	TRUE	reported	dNl64d
11	23629106	G	T	0.042657	0.008598	7.00E-07	0.007953	64944	NA	rs1406562	NA	UKB-b-12	TRUE	reported	dNl64d
11	37162800	C	G	0.041451	0.007832	1.20E-07	0.009868	64944	NA	rs1896556	NA	UKB-b-12	TRUE	reported	dNl64d
11	78547427	T	C	0.045701	0.009993	4.80E-06	0.00627	64944	NA	rs1381713	NA	UKB-b-12	TRUE	reported	dNl64d
11	87401749	C	G	0.031906	0.006905	3.80E-06	0.012181	64944	NA	rs4280024	NA	UKB-b-12	TRUE	reported	dNl64d
11	1.02E+08	T	G	0.043282	0.009411	4.20E-06	0.007375	64944	NA	rs1472618	NA	UKB-b-12	TRUE	reported	dNl64d
11	1.2E+08	G	A	0.030652	0.006439	1.90E-06	0.013977	64944	NA	rs1456372	NA	UKB-b-12	TRUE	reported	dNl64d
11	1.2E+08	C	T	0.030717	0.006496	2.30E-06	0.013774	64944	NA	rs1445024	NA	UKB-b-12	TRUE	reported	dNl64d
11	1.2E+08	G	A	0.033415	0.006484	2.60E-07	0.013938	64944	NA	rs1171144	NA	UKB-b-12	TRUE	reported	dNl64d
11	1.2E+08	G	A	0.049803	0.010543	2.30E-06	0.005457	64944	NA	rs1812725	NA	UKB-b-12	TRUE	reported	dNl64d
11	1.2E+08	A	G	0.045704	0.009143	5.80E-07	0.007161	64944	NA	rs1402433	NA	UKB-b-12	TRUE	reported	dNl64d
11	1.2E+08	G	A	0.057028	0.010437	4.70E-08	0.005623	64944	NA	rs1903541	NA	UKB-b-12	TRUE	reported	dNl64d
11	1.2E+08	G	A	0.04596	0.009173	5.40E-07	0.007134	64944	NA	rs1885392	NA	UKB-b-12	TRUE	reported	dNl64d
11	1.2E+08	G	A	0.045402	0.009165	7.30E-07	0.007169	64944	NA	rs1915563	NA	UKB-b-12	TRUE	reported	dNl64d
11	1.31E+08	C	T	0.048038	0.009811	9.80E-07	0.006686	64944	NA	rs1454395	NA	UKB-b-12	TRUE	reported	dNl64d
12	3950854	A	G	0.024477	0.005323	4.30E-06	0.024077	64944	NA	rs1439564	NA	UKB-b-12	TRUE	reported	dNl64d
12	4020016	C	G	0.037955	0.007934	1.70E-06	0.00939	64944	NA	rs1174766	NA	UKB-b-12	TRUE	reported	dNl64d
12	4021470	G	A	0.035969	0.007852	4.60E-06	0.009614	64944	NA	rs7759764	NA	UKB-b-12	TRUE	reported	dNl64d
12	66841989	C	A	0.041017	0.008657	2.20E-06	0.008218	64944	NA	rs7710500	NA	UKB-b-12	TRUE	reported	dNl64d
12	72559746	C	T	0.050295	0.010164	7.50E-07	0.005866	64944	NA	rs1481014	NA	UKB-b-12	TRUE	reported	dNl64d
12	1.23E+08	C	T	-0.03758	0.007761	1.30E-06	0.990391	64944	NA	rs1073497	NA	UKB-b-12	TRUE	reported	dNl64d
12	1.23E+08	C	T	0.040258	0.008128	7.30E-07	0.00881	64944	NA	rs7649558	NA	UKB-b-12	TRUE	reported	dNl64d
12	1.24E+08	G	C	0.039769	0.008099	9.10E-07	0.008783	64944	NA	rs1160810	NA	UKB-b-12	TRUE	reported	dNl64d
12	1.24E+08	G	A	0.036519	0.007681	2.00E-06	0.009698	64944	NA	rs7937592	NA	UKB-b-12	TRUE	reported	dNl64d
12	1.24E+08	G	A	0.036385	0.007673	2.10E-06	0.00972	64944	NA	rs1260315	NA	UKB-b-12	TRUE	reported	dNl64d
12	1.24E+08	T	C	0.03638	0.007673	2.10E-06	0.009713	64944	NA	rs1260319	NA	UKB-b-12	TRUE	reported	dNl64d
12	1.24E+08	G	C	0.036288	0.007668	2.20E-06	0.009737	64944	NA	rs2682428	NA	UKB-b-12	TRUE	reported	dNl64d
12	1.24E+08	G	T	0.035906	0.007799	4.20E-06	0.009478	64944	NA	rs1440451	NA	UKB-b-12	TRUE	reported	dNl64d
12	1.24E+08	G	A	0.035969	0.007796	3.90E-06	0.009468	64944	NA	rs1394733	NA	UKB-b-12	TRUE	reported	dNl64d
12	1.24E+08	C	T	0.034973	0.007662	5.00E-06	0.00994	64944	NA	rs2869472	NA	UKB-b-12	TRUE	reported	dNl64d
12	1.24E+08	G	T	0.037087	0.007794	2.00E-06	0.009535	64944	NA	rs1154603	NA	UKB-b-12	TRUE	reported	dNl64d
12	1.24E+08	C	T	0.039516	0.007779	3.80E-07	0.00989	64944	NA	rs2847073	NA	UKB-b-12	TRUE	reported	dNl64d
13	80511564	T	C	0.045617	0.009967	4.70E-06	0.007091	64944	NA	rs5618414	NA	UKB-b-12	TRUE	reported	dNl64d
13	82346982	A	G	0.047592	0.010022	2.00E-06	0.007067	64944	NA	rs1911585	NA	UKB-b-12	TRUE	reported	dNl64d
13	82956579	C	T	0.030037	0.006103	8.60E-07	0.016272	64944	NA	rs7323411	NA	UKB-b-12	TRUE	reported	dNl64d
13	83043472	G	A	0.031328	0.006432	1.10E-06	0.015418	64944	NA	rs7323417	NA	UKB-b-12	TRUE	reported	dNl64d
13	83117145	A	G	0.027353	0.005655	1.30E-06	0.018	64944	NA	rs7323641	NA	UKB-b-12	TRUE	reported	dNl64d
13	1.1E+08	C	T	0.026255	0.005552	2.30E-06	0.018971	64944	NA	rs7265829	NA	UKB-b-12	TRUE	reported	dNl64d
14	47208203	T	C	0.037947	0.008154	3.30E-06	0.009568	64944	NA	rs1808743	NA	UKB-b-12	TRUE	reported	dNl64d
14	52753593	C	T	0.030766	0.00636	1.30E-06	0.014043	64944	NA	rs1176939	NA	UKB-b-12	TRUE	reported	dNl64d
14	66376580	A	G	0.044221	0.008904	6.80E-07	0.008306	64944	NA	rs1130649	NA	UKB-b-12	TRUE	reported	dNl64d
15	71340688	C	G	0.028549	0.006113	3.00E-06	0.015665	64944	NA	rs1457728	NA	UKB-b-12	TRUE	reported	dNl64d
15	78711994	A	G	0.03566	0.007331	1.10E-06	0.01306	64944	NA	rs1431278	NA	UKB-b-12	TRUE	reported	dNl64d
15	88766520	G	A	0.024638	0.005196	2.10E-06	0.021434	64944	NA	rs7765815	NA	UKB-b-12	TRUE	reported	dNl64d
15	88781436	C	T	0.025075	0.005314	2.40E-06	0.02091	64944	NA	rs1181782	NA	UKB-b-12	TRUE	reported	dNl64d
15	97730071	C	T	0.050246	0.010381	1.30E-06	0.006222	64944	NA	rs1810442	NA	UKB-b-12	TRUE	reported	dNl64d
16	3223129	G	A	0.036707	0.007795	2.50E-06	0.010145	64944	NA	rs1814708	NA	UKB-b-12	TRUE	reported	dNl64d
16	78565164	G	C	0.048374	0.010448	3.70E-06	0.005996	64944	NA	rs1486877	NA	UKB-b-12	TRUE	reported	dNl64d
16	81183820	G	C	0.026528	0.00579	4.60E-06	0.020039	64944	NA	rs1402066	NA	UKB-b-12	TRUE	reported	dNl64d
17	54378029	C	T	0.01188	0.002588	4.40E-06	0.103383	64944	NA	rs1186933	NA	UKB-b-12	TRUE	reported	dNl64d
17	63519291	C	T	0.049825	0.010915	5.00E-06	0.005825	64944	NA	rs1449576	NA	UKB-b-12	TRUE	reported	dNl64d
18	25458758	G	C	0.031157	0.006784	4.40E-06	0.012381	64944	NA	rs7394506	NA	UKB-b-12	TRUE	reported	dNl64d
18	25459109	A	G	0.031119	0.006781	4.40E-06	0.012388	64944	NA	rs6129817	NA	UKB-b-12	TRUE	reported	dNl64d
18	25459858	A	C	0.03106	0.006774	4.50E-06	0.012395	64944	NA	rs7341521	NA	UKB-b-12	TRUE	reported	dNl64d
18	57646309	A	G	0.008692	0.001822	1.80E-06	0.226077	64944	NA	rs7234196	NA	UKB-b-12	TRUE	reported	dNl64d
19	857136	T	C	0.049305	0.009754	4.30E-07	0.007178	64944	NA	rs1883563	NA	UKB-b-12	TRUE	reported	dNl64d
19	13733953	C	T	0.02461	0.005305	3.50E-06	0.020187	64944	NA	rs1171975	NA	UKB-b-12	TRUE	reported	dNl64d
20	3200113	G	A	0.029938	0.006247	1.60E-06	0.014899	64944	NA	rs1480631	NA	UKB-b-12	TRUE	reported	dNl64d
20	55367285	C	T	0.00719	0.001567	4.50E-06	0.389404	64944	NA	rs2426687	NA	UKB-b-12	TRUE	reported	dNl64d
20	55369182	C	A	0.007258	0.001551	2.90E-06	0.401745	64944	NA	rs1321865	NA	UKB-b-12	TRUE	reported	dNl64d
20	55825431	C	A	0.007182	0.001573	5.00E-06	0.356333	64944	NA	rs4811830	NA	UKB-b-12	TRUE	reported	dNl64d
22	36470420	G	A	0.046997	0.010215	4.20E-06	0.006239	64944	NA	rs1419741	NA	UKB-b-12	TRUE	reported	dNl64d
22	40472202	C	T	0.015054	0.003185	2.30E-06	0.060487	64944	NA	rs1169951	NA	UKB-b-12	TRUE	reported	dNl64d
22	40488545	C	G	0.015213	0.003189	1.80E-06	0.060403	64944	NA	rs1177901	NA	UKB-b-12	TRUE	reported	dNl64d
22	40528393	C	T	0.017695	0.003297	8.00E-08	0.056866	64944	NA	rs1170939	NA	UKB-b-12	TRUE	reported	dNl64d
22	40607833	G	A	0.015608	0.003384	4.00E-06	0.053796	64944	NA	rs7840120	NA	UKB-b-12	TRUE	reported	dNl64d
