# Supplementary material for: Risk of major depressive increases with increasing frequency of alcohol drinking: a bidirectional two-sample Mendelian randomization analysis
Source: Front Public Health. 2024 Jun 5;12:1372758. doi: 10.3389/fpubh.2024.1372758 (PMC11186411; doi:10.3389/fpubh.2024.1372758)
Supplement: Supplementary file 7 [file Data_Sheet_6.PDF]

Beer/cider intake SNPs

SNP	effect_allel	other_allel	effect_allel	other_allel	beta.expos	beta.outco	eaf.exposu	eaf.outcom	remove	palindromi	ambiguous	id.outcome	se.outcome	pval.outco	outcome	mr_keep.ou	pval_origin	data_sourc	se.exposure	exposure	mr_keep.ex	pval.exposu	pval_origin	id.exposure	data_sourc	action	mr_keep	samplesize.outcome
rs1219142(	A	G	A	G	0.077793	0.0098	0.037745	0.0371	FALSE	FALSE	FALSE	MZoFAy	0.0117	0.401	major depr	TRUE	reported	textfile	0.015779	beer cider	TRUE	8.22E-07	inferred	k4LaVT	textfile	2	TRUE	NA
rs1450627	T	G	T	G	-0.06364	0.0089	0.938593	0.9381	FALSE	FALSE	FALSE	MZoFAy	0.009	0.3243	major depr	TRUE	reported	textfile	0.01256	beer cider	TRUE	4.03E-07	inferred	k4LaVT	textfile	2	TRUE	NA
rs1842578	T	C	T	C	0.067013	6.00E-04	0.071721	0.0746	FALSE	FALSE	FALSE	MZoFAy	0.0087	0.9424	major depr	TRUE	reported	textfile	0.012145	beer cider	TRUE	3.43E-08	inferred	k4LaVT	textfile	2	TRUE	NA
rs225253	A	G	A	G	0.084828	0.0064	0.029616	0.0301	FALSE	FALSE	FALSE	MZoFAy	0.0131	0.6231	major depr	TRUE	reported	textfile	0.017695	beer cider	TRUE	1.64E-06	inferred	k4LaVT	textfile	2	TRUE	NA
rs2408991	C	G	C	G	-0.03293	-0.004	0.242752	0.2407	FALSE	TRUE	FALSE	MZoFAy	0.005	0.4316	major depr	TRUE	reported	textfile	0.007042	beer cider	TRUE	2.92E-06	inferred	k4LaVT	textfile	2	TRUE	NA
rs3212737	C	G	C	G	0.057026	-0.0057	0.074089	0.0732	FALSE	TRUE	FALSE	MZoFAy	0.0087	0.5087	major depr	TRUE	reported	textfile	0.011642	beer cider	TRUE	9.67E-07	inferred	k4LaVT	textfile	2	TRUE	NA
rs373002	C	T	C	T	0.02953	-0.0034	0.651223	0.6488	FALSE	FALSE	FALSE	MZoFAy	0.0046	0.459	major depr	TRUE	reported	textfile	0.006371	beer cider	TRUE	3.57E-06	inferred	k4LaVT	textfile	2	TRUE	NA
rs3817588	C	T	C	T	0.037533	-0.0063	0.193605	0.1949	FALSE	FALSE	FALSE	MZoFAy	0.0055	0.2487	major depr	TRUE	reported	textfile	0.007655	beer cider	TRUE	9.45E-07	inferred	k4LaVT	textfile	2	TRUE	NA
rs6256525!	T	C	T	C	0.06633	0.0115	0.058138	0.0576	FALSE	FALSE	FALSE	MZoFAy	0.0096	0.2299	major depr	TRUE	reported	textfile	0.01282	beer cider	TRUE	2.29E-07	inferred	k4LaVT	textfile	2	TRUE	NA
rs6839368	A	T	A	T	0.040612	3.00E-04	0.152948	0.1531	FALSE	TRUE	FALSE	MZoFAy	0.006	0.9609	major depr	TRUE	reported	textfile	0.008413	beer cider	TRUE	1.39E-06	inferred	k4LaVT	textfile	2	TRUE	NA
rs7286306(	T	G	T	G	0.081221	-0.0014	0.032125	0.032	FALSE	FALSE	FALSE	MZoFAy	0.0126	0.9125	major depr	TRUE	reported	textfile	0.017012	beer cider	TRUE	1.80E-06	inferred	k4LaVT	textfile	2	TRUE	NA

Fortified wine intake SNPs

SNP	effect_allel	other_allel	effect_allel	other_allel	beta.expos	beta.outco	eaf.exposu	eaf.outcom	remove	palindromi	ambiguous	id.outcome	se.outcome	pval.outco	outcome	mr_keep.ou	pval_origin	data_sourc	se.exposure	exposure	mr_keep.ex	pval.exposu	pval_origin	id.exposure	data_sourc	action	mr_keep	samplesize.outcome
rs1102491	G	A	G	A	0.023848	0.0041	0.02497	0.0258	FALSE	FALSE	FALSE	Z6lLRk	0.0141	0.7701	major depr	TRUE	reported	textfile	0.005028	fortified wi	TRUE	2.11E-06	inferred	mOaHZ0	textfile	2	TRUE	NA
rs1124842	C	G	C	G	0.019556	-0.0041	0.03935	0.0383	FALSE	TRUE	FALSE	Z6lLRk	0.0118	0.730299	major depr	TRUE	reported	textfile	0.00408	fortified wi	TRUE	1.64E-06	inferred	mOaHZ0	textfile	2	TRUE	NA
rs1131465	T	C	T	C	0.019402	0.0119	0.042095	0.0411	FALSE	FALSE	FALSE	Z6lLRk	0.0118	0.3097	major depr	TRUE	reported	textfile	0.004042	fortified wi	TRUE	1.59E-06	inferred	mOaHZ0	textfile	2	TRUE	NA
rs1148942	T	C	T	C	0.046474	0.0202	0.007049	0.0067	FALSE	FALSE	FALSE	Z6lLRk	0.0273	0.4588	major depr	TRUE	reported	textfile	0.009337	fortified wi	TRUE	6.44E-07	inferred	mOaHZ0	textfile	2	TRUE	NA
rs1171188	G	A	G	A	0.03098	-0.0155	0.013035	0.0133	FALSE	FALSE	FALSE	Z6lLRk	0.0194	0.4241	major depr	TRUE	reported	textfile	0.006777	fortified wi	TRUE	4.84E-06	inferred	mOaHZ0	textfile	2	TRUE	NA
rs1178879	A	G	A	G	0.040268	0.0057	0.008561	0.0088	FALSE	FALSE	FALSE	Z6lLRk	0.0235	0.8081	major depr	TRUE	reported	textfile	0.008328	fortified wi	TRUE	1.33E-06	inferred	mOaHZ0	textfile	2	TRUE	NA
rs1268670	T	C	T	C	0.012879	-0.0068	0.087132	0.0885	FALSE	FALSE	FALSE	Z6lLRk	0.0077	0.3739	major depr	TRUE	reported	textfile	0.002728	fortified wi	TRUE	2.35E-06	inferred	mOaHZ0	textfile	2	TRUE	NA
rs1497167	C	G	C	G	0.059145	-0.0314	0.005914	0.0061	FALSE	TRUE	FALSE	Z6lLRk	0.0307	0.3061	major depr	TRUE	reported	textfile	0.010552	fortified wi	TRUE	2.08E-08	inferred	mOaHZ0	textfile	2	TRUE	NA
rs1507184	G	A	G	A	0.029213	0.0033	0.018142	0.0177	FALSE	FALSE	FALSE	Z6lLRk	0.0181	0.8538	major depr	TRUE	reported	textfile	0.006084	fortified wi	TRUE	1.57E-06	inferred	mOaHZ0	textfile	2	TRUE	NA
rs1822876	C	T	C	T	0.042491	0.0154	0.007902	0.0088	FALSE	FALSE	FALSE	Z6lLRk	0.0253	0.5418	major depr	TRUE	reported	textfile	0.009107	fortified wi	TRUE	3.07E-06	inferred	mOaHZ0	textfile	2	TRUE	NA
rs1883901	T	C	T	C	0.054603	-0.0104	0.005773	0.0063	FALSE	FALSE	FALSE	Z6lLRk	0.0283	0.7145	major depr	TRUE	reported	textfile	0.010344	fortified wi	TRUE	1.30E-07	inferred	mOaHZ0	textfile	2	TRUE	NA
rs274958	C	T	C	T	0.014006	-0.0023	0.071278	0.0704	FALSE	FALSE	FALSE	Z6lLRk	0.0086	0.786001	major depr	TRUE	reported	textfile	0.00304	fortified wi	TRUE	4.08E-06	inferred	mOaHZ0	textfile	2	TRUE	NA
rs4279116	C	T	C	T	-0.00716	-0.0053	0.441731	0.4409	FALSE	FALSE	FALSE	Z6lLRk	0.0043	0.2162	major depr	TRUE	reported	textfile	0.001551	fortified wi	TRUE	3.83E-06	inferred	mOaHZ0	textfile	2	TRUE	NA
rs6042162	T	C	T	C	0.007577	0	0.685325	0.6847	FALSE	FALSE	FALSE	Z6lLRk	0.0046	0.996	major depr	TRUE	reported	textfile	0.001658	fortified wi	TRUE	4.91E-06	inferred	mOaHZ0	textfile	2	TRUE	NA
rs6234058	A	G	A	G	0.01599	0.0046	0.056271	0.055	FALSE	FALSE	FALSE	Z6lLRk	0.0101	0.6498	major depr	TRUE	reported	textfile	0.003446	fortified wi	TRUE	3.47E-06	inferred	mOaHZ0	textfile	2	TRUE	NA
rs7171089	A	G	A	G	-0.00885	0.0039	0.764717	0.7671	FALSE	FALSE	FALSE	Z6lLRk	0.0051	0.4391	major depr	TRUE	reported	textfile	0.00182	fortified wi	TRUE	1.17E-06	inferred	mOaHZ0	textfile	2	TRUE	NA
rs7281477	A	G	A	G	0.010922	-0.0143	0.168941	0.1708	FALSE	FALSE	FALSE	Z6lLRk	0.0058	0.01291	major depr	TRUE	reported	textfile	0.002063	fortified wi	TRUE	1.19E-07	inferred	mOaHZ0	textfile	2	TRUE	NA
rs7319839	T	C	T	C	0.026695	0.0324	0.019498	0.0199	FALSE	FALSE	FALSE	Z6lLRk	0.0162	0.04591	major depr	TRUE	reported	textfile	0.005758	fortified wi	TRUE	3.54E-06	inferred	mOaHZ0	textfile	2	TRUE	NA
rs7743980	T	C	T	C	0.02852	-0.0184	0.015599	0.0157	FALSE	FALSE	FALSE	Z6lLRk	0.018	0.3071	major depr	TRUE	reported	textfile	0.006192	fortified wi	TRUE	4.11E-06	inferred	mOaHZ0	textfile	2	TRUE	NA
rs7948251	A	G	A	G	0.028654	0.0169	0.021756	0.0214	FALSE	FALSE	FALSE	Z6lLRk	0.0149	0.258	major depr	TRUE	reported	textfile	0.005286	fortified wi	TRUE	5.94E-08	inferred	mOaHZ0	textfile	2	TRUE	NA

Red wine intake SNPs

SNP	effect_allel	other_allele	effect_allel	other_allele	beta.expos	beta.outco	eaf.exposu	eaf.outcom	remove	palindromi	ambiguous	id.outcome	se.outcome	pval.outcor	outcome	mr_keep.ou	pval_origin	data_sourc	se.exposure	exposure	mr_keep.ex	pval.exposu	pval_origin	id.exposure	data_sourc	action	mr_keep	samplesize.outcome
rs1149660	G	A	G	A	0.151991	0.0163	0.01095	0.0108	FALSE	FALSE	FALSE	5R428f	0.0231	0.4825	major depr	TRUE	reported	textfile	0.033067	red wine in	TRUE	4.30E-06	inferred	xtluFE	textfile	2	TRUE	NA
rs1164783	T	C	T	C	0.066382	0.0115	0.053226	0.0516	FALSE	FALSE	FALSE	5R428f	0.0102	0.257	major depr	TRUE	reported	textfile	0.014518	red wine in	TRUE	4.82E-06	inferred	xtluFE	textfile	2	TRUE	NA
rs1747866	A	G	A	G	0.050776	-0.0027	0.103343	0.1049	FALSE	FALSE	FALSE	5R428f	0.007	0.6989	major depr	TRUE	reported	textfile	0.010684	red wine in	TRUE	2.01E-06	inferred	xtluFE	textfile	2	TRUE	NA
rs3751395	A	C	A	C	0.031476	0.0064	0.458991	0.46	FALSE	FALSE	FALSE	5R428f	0.0044	0.1441	major depr	TRUE	reported	textfile	0.006533	red wine in	TRUE	1.45E-06	inferred	xtluFE	textfile	2	TRUE	NA
rs402236	G	A	G	A	0.042838	-0.0111	0.841475	0.84	FALSE	FALSE	FALSE	5R428f	0.006	0.06612	major depr	TRUE	reported	textfile	0.008973	red wine in	TRUE	1.81E-06	inferred	xtluFE	textfile	2	TRUE	NA
rs4860372	A	T	A	T	0.050864	0.0033	0.892706	0.8922	FALSE	TRUE	FALSE	5R428f	0.0069	0.6294	major depr	TRUE	reported	textfile	0.010533	red wine in	TRUE	1.37E-06	inferred	xtluFE	textfile	2	TRUE	NA
rs566577	T	C	T	C	0.058012	-0.0058	0.89771	0.899	FALSE	FALSE	FALSE	5R428f	0.0072	0.415	major depr	TRUE	reported	textfile	0.010746	red wine in	TRUE	6.72E-08	inferred	xtluFE	textfile	2	TRUE	NA
rs7820063	T	C	T	C	0.04245	-0.0077	0.151749	0.1531	FALSE	FALSE	FALSE	5R428f	0.006	0.1976	major depr	TRUE	reported	textfile	0.009113	red wine in	TRUE	3.19E-06	inferred	xtluFE	textfile	2	TRUE	NA

Spirits intake SNPs

SNP	effect_allel	other_allel	effect_allel	other_allel	beta.expos	beta.outco	eaf.exposu	eaf.outcom	remove	palindromi	ambiguous	id.outcome	se.outcome	pval.outco	outcome	mr_keep.ou	pval_origin	data_sourc	se.exposure	exposure	mr_keep.ex	pval.expos	pval_origin	id.exposure	data_sourc	action	mr_keep	samplesize.outcome
rs1041648	C	T	C	T	-0.01997	-1.00E-04	0.401282	0.4038	FALSE	FALSE	FALSE	jXhDgG	0.0045	0.9901	major depr	TRUE	reported	textfile	0.004361	spirits	TRUE	4.64E-06	inferred	Aelm6l	textfile	2	TRUE	NA
rs1141122	A	G	A	G	0.086754	0.0135	0.013207	0.0127	FALSE	FALSE	FALSE	jXhDgG	0.0198	0.4954	major depr	TRUE	reported	textfile	0.018842	spirits	TRUE	4.14E-06	inferred	Aelm6l	textfile	2	TRUE	NA
rs1167565	C	T	C	T	-0.07412	-0.0174	0.01933	0.019	FALSE	FALSE	FALSE	jXhDgG	0.0167	0.2986	major depr	TRUE	reported	textfile	0.015979	spirits	TRUE	3.51E-06	inferred	Aelm6l	textfile	2	TRUE	NA
rs1172035	A	T	A	T	0.070165	0.0075	0.021871	0.0225	FALSE	TRUE	FALSE	jXhDgG	0.0156	0.6294	major depr	TRUE	reported	textfile	0.015282	spirits	TRUE	4.40E-06	inferred	Aelm6l	textfile	2	TRUE	NA
rs1248071	G	A	G	A	0.0329	-7.00E-04	0.111819	0.1107	FALSE	FALSE	FALSE	jXhDgG	0.0072	0.9274	major depr	TRUE	reported	textfile	0.006725	spirits	TRUE	9.98E-07	inferred	Aelm6l	textfile	2	TRUE	NA
rs1380080	G	A	G	A	0.09963	-0.0135	0.01175	0.0125	FALSE	FALSE	FALSE	jXhDgG	0.0204	0.5079	major depr	TRUE	reported	textfile	0.020518	spirits	TRUE	1.20E-06	inferred	Aelm6l	textfile	2	TRUE	NA
rs1390454	T	A	T	A	0.062982	0.002	0.027293	0.0284	FALSE	TRUE	FALSE	jXhDgG	0.0136	0.8831	major depr	TRUE	reported	textfile	0.013482	spirits	TRUE	2.99E-06	inferred	Aelm6l	textfile	2	TRUE	NA
rs1391643	A	G	A	G	0.1083	-0.0339	0.009298	0.0099	FALSE	FALSE	FALSE	jXhDgG	0.0247	0.1701	major depr	TRUE	reported	textfile	0.023559	spirits	TRUE	4.29E-06	inferred	Aelm6l	textfile	2	TRUE	NA
rs1435394	A	T	A	T	0.069265	0.0219	0.024502	0.0243	FALSE	TRUE	FALSE	jXhDgG	0.0151	0.1477	major depr	TRUE	reported	textfile	0.014464	spirits	TRUE	1.68E-06	inferred	Aelm6l	textfile	2	TRUE	NA
rs1509814	C	T	C	T	0.100447	0.03	0.011055	0.0121	FALSE	FALSE	FALSE	jXhDgG	0.0216	0.1655	major depr	TRUE	reported	textfile	0.021281	spirits	TRUE	2.36E-06	inferred	Aelm6l	textfile	2	TRUE	NA
rs4608967	T	C	T	C	0.032475	0.0209	0.101776	0.1034	FALSE	FALSE	FALSE	jXhDgG	0.007	0.002937	major depr	TRUE	reported	textfile	0.007012	spirits	TRUE	3.64E-06	inferred	Aelm6l	textfile	2	TRUE	NA
rs6200119	G	A	G	A	0.023098	0.0148	0.298063	0.2974	FALSE	FALSE	FALSE	jXhDgG	0.0048	0.001954	major depr	TRUE	reported	textfile	0.004676	spirits	TRUE	7.84E-07	inferred	Aelm6l	textfile	2	TRUE	NA
rs6220456	A	G	A	G	0.068658	-0.0219	0.022749	0.023	FALSE	FALSE	FALSE	jXhDgG	0.0153	0.1539	major depr	TRUE	reported	textfile	0.014814	spirits	TRUE	3.58E-06	inferred	Aelm6l	textfile	2	TRUE	NA
rs7268952	T	A	T	A	0.06678	0.0194	0.027872	0.0262	FALSE	TRUE	FALSE	jXhDgG	0.014	0.1669	major depr	TRUE	reported	textfile	0.013098	spirits	TRUE	3.43E-07	inferred	Aelm6l	textfile	2	TRUE	NA
rs7629659	C	G	C	G	0.097122	0.0128	0.012655	0.0118	FALSE	TRUE	FALSE	jXhDgG	0.0224	0.566601	major depr	TRUE	reported	textfile	0.020338	spirits	TRUE	1.79E-06	inferred	Aelm6l	textfile	2	TRUE	NA
rs7746271	C	A	C	A	-0.02061	-0.0029	0.662242	0.6643	FALSE	FALSE	FALSE	jXhDgG	0.0046	0.5276	major depr	TRUE	reported	textfile	0.004489	spirits	TRUE	4.39E-06	inferred	Aelm6l	textfile	2	TRUE	NA
rs7800543	A	G	A	G	0.07475	-0.0071	0.017483	0.0176	FALSE	FALSE	FALSE	jXhDgG	0.0168	0.674	major depr	TRUE	reported	textfile	0.016193	spirits	TRUE	3.91E-06	inferred	Aelm6l	textfile	2	TRUE	NA
rs7887698	G	C	G	C	0.032	-0.0145	0.110127	0.1081	FALSE	TRUE	FALSE	jXhDgG	0.0071	0.04215	major depr	TRUE	reported	textfile	0.006814	spirits	TRUE	2.65E-06	inferred	Aelm6l	textfile	2	TRUE	NA
rs7899891	T	G	T	G	0.059035	0.011	0.030594	0.0308	FALSE	FALSE	FALSE	jXhDgG	0.0129	0.3953	major depr	TRUE	reported	textfile	0.012679	spirits	TRUE	3.22E-06	inferred	Aelm6l	textfile	2	TRUE	NA
rs9310159	A	G	A	G	-0.03305	-0.015	0.130529	0.1311	FALSE	FALSE	FALSE	jXhDgG	0.0064	0.01945	major depr	TRUE	reported	textfile	0.006345	spirits	TRUE	1.90E-07	inferred	Aelm6l	textfile	2	TRUE	NA

White wine/champange intake SNPs

SNP	effect_allel	other_allel	effect_allel	other_allel	beta.expos	beta.outco	eaf.exposu	eaf.outcom	remove	palindromi	ambiguous	id.outcome	se.outcome	pval.outco	outcome	mr_keep.ou	pval_origin	data_sourc	se.exposure	exposure	mr_keep.ex	pval.expos	pval_origin	id.exposure	data_sourc	action	mr_keep	samplesize.outcome
rs1076635	T	C	T	C	0.036226	-0.0084	0.341369	0.3358	FALSE	FALSE	FALSE	daMlok	0.0047	0.070981	major depr	TRUE	reported	textfile	0.006359	white wine	TRUE	1.22E-08	inferred	tm5dNL	textfile	2	TRUE	NA
rs1164532	T	C	T	C	0.074054	0.0187	0.034542	0.0343	FALSE	FALSE	FALSE	daMlok	0.0121	0.1206	major depr	TRUE	reported	textfile	0.016135	white wine	TRUE	4.44E-06	inferred	tm5dNL	textfile	2	TRUE	NA
rs1543247	G	C	G	C	0.065371	0.0195	0.051702	0.0516	FALSE	TRUE	FALSE	daMlok	0.0098	0.04683	major depr	TRUE	reported	textfile	0.013353	white wine	TRUE	9.79E-07	inferred	tm5dNL	textfile	2	TRUE	NA
rs2988328	C	T	C	T	-0.04354	-8.00E-04	0.862677	0.8628	FALSE	FALSE	FALSE	daMlok	0.0063	0.9025	major depr	TRUE	reported	textfile	0.008593	white wine	TRUE	4.03E-07	inferred	tm5dNL	textfile	2	TRUE	NA
rs339866	A	G	A	G	-0.03149	0.0131	0.417804	0.4161	FALSE	FALSE	FALSE	daMlok	0.0044	0.002754	major depr	TRUE	reported	textfile	0.006018	white wine	TRUE	1.67E-07	inferred	tm5dNL	textfile	2	TRUE	NA
rs4551638	C	G	C	G	0.032448	-0.0069	0.286871	0.2853	FALSE	TRUE	FALSE	daMlok	0.0049	0.1623	major depr	TRUE	reported	textfile	0.006571	white wine	TRUE	7.88E-07	inferred	tm5dNL	textfile	2	TRUE	NA
rs4920553	A	G	A	G	0.043816	-0.0075	0.127848	0.1265	FALSE	FALSE	FALSE	daMlok	0.0065	0.2453	major depr	TRUE	reported	textfile	0.008848	white wine	TRUE	7.34E-07	inferred	tm5dNL	textfile	2	TRUE	NA
rs7280512	A	G	A	G	0.091186	0.0261	0.027905	0.0278	FALSE	FALSE	FALSE	daMlok	0.0133	0.050389	major depr	TRUE	reported	textfile	0.018153	white wine	TRUE	5.08E-07	inferred	tm5dNL	textfile	2	TRUE	NA
rs7286321	G	T	G	T	-0.03325	-0.0055	0.215661	0.2149	FALSE	FALSE	FALSE	daMlok	0.0053	0.3008	major depr	TRUE	reported	textfile	0.007246	white wine	TRUE	4.47E-06	inferred	tm5dNL	textfile	2	TRUE	NA
rs7313788	G	A	G	A	0.082777	-0.0044	0.030693	0.0309	FALSE	FALSE	FALSE	daMlok	0.0128	0.730099	major depr	TRUE	reported	textfile	0.017128	white wine	TRUE	1.35E-06	inferred	tm5dNL	textfile	2	TRUE	NA
rs7323411	T	C	T	C	0.114973	0.0026	0.016271	0.0156	FALSE	FALSE	FALSE	daMlok	0.0186	0.8905	major depr	TRUE	reported	textfile	0.024019	white wine	TRUE	1.70E-06	inferred	tm5dNL	textfile	2	TRUE	NA
rs7644783	G	A	G	A	-0.0304	0.0018	0.673713	0.6725	FALSE	FALSE	FALSE	daMlok	0.0046	0.6947	major depr	TRUE	reported	textfile	0.006305	white wine	TRUE	1.42E-06	inferred	tm5dNL	textfile	2	TRUE	NA

Other alcohol intake SNPs

SNP	effect_allel	other_allel	effect_allel	other_allel	beta.expos	beta.outcc	eaf.exposu	eaf.outcon	remove	palindrom	ambiguou	id.outcom	se.outcom	pval.outco	outcome	mr_keep.o	pval_origir	data_sourc	se.exposur	exposure	mr_keep.e	pval.expos	pval_origir	id.exposur	data_sourc	action	mr_keep	samplesize.outcome
rs1153319	A	T	A	T	0.033203	-0.0101	0.015513	0.0153	FALSE	TRUE	FALSE	wuktJL	0.0176	0.5651	major dep	TRUE	reported	textfile	0.006087	other alcol	TRUE	4.90E-08	inferred	UkHULc	textfile	2	TRUE	NA
rs1155928	C	T	C	T	0.031808	-0.0178	0.01252	0.0128	FALSE	FALSE	FALSE	wuktJL	0.0202	0.3773	major dep	TRUE	reported	textfile	0.006948	other alcol	TRUE	4.69E-06	inferred	UkHULc	textfile	2	TRUE	NA
rs1171144	A	G	A	G	0.033415	-0.0219	0.013938	0.0155	FALSE	FALSE	FALSE	wuktJL	0.0179	0.2202	major dep	TRUE	reported	textfile	0.006484	other alcol	TRUE	2.55E-07	inferred	UkHULc	textfile	2	TRUE	NA
rs1171537	G	A	G	A	0.031534	0.0011	0.01299	0.0132	FALSE	FALSE	FALSE	wuktJL	0.0199	0.9574	major dep	TRUE	reported	textfile	0.006838	other alcol	TRUE	3.99E-06	inferred	UkHULc	textfile	2	TRUE	NA
rs1174766	G	C	G	C	0.037955	0.0102	0.00939	0.0093	FALSE	TRUE	FALSE	wuktJL	0.0233	0.660601	major dep	TRUE	reported	textfile	0.007934	other alcol	TRUE	1.72E-06	inferred	UkHULc	textfile	2	TRUE	NA
rs1176939	T	C	T	C	0.030766	-0.014	0.014043	0.0137	FALSE	FALSE	FALSE	wuktJL	0.0194	0.472	major dep	TRUE	reported	textfile	0.00636	other alcol	TRUE	1.31E-06	inferred	UkHULc	textfile	2	TRUE	NA
rs1186933	T	C	T	C	0.01188	6.00E-04	0.103383	0.1043	FALSE	FALSE	FALSE	wuktJL	0.0075	0.9406	major dep	TRUE	reported	textfile	0.002588	other alcol	TRUE	4.44E-06	inferred	UkHULc	textfile	2	TRUE	NA
rs1321865	A	C	A	C	0.007258	0.0053	0.401745	0.4038	FALSE	FALSE	FALSE	wuktJL	0.0044	0.2334	major dep	TRUE	reported	textfile	0.001551	other alcol	TRUE	2.88E-06	inferred	UkHULc	textfile	2	TRUE	NA
rs1383557	T	C	T	C	0.029082	-0.0236	0.014726	0.0137	FALSE	FALSE	FALSE	wuktJL	0.0205	0.2503	major dep	TRUE	reported	textfile	0.006355	other alcol	TRUE	4.74E-06	inferred	UkHULc	textfile	2	TRUE	NA
rs1401980	T	G	T	G	0.01693	0.0059	0.047889	0.0478	FALSE	FALSE	FALSE	wuktJL	0.0102	0.5647	major dep	TRUE	reported	textfile	0.003553	other alcol	TRUE	1.89E-06	inferred	UkHULc	textfile	2	TRUE	NA
rs1402066	C	G	C	G	0.026528	0.0172	0.020039	0.0185	FALSE	TRUE	FALSE	wuktJL	0.0178	0.3351	major dep	TRUE	reported	textfile	0.00579	other alcol	TRUE	4.60E-06	inferred	UkHULc	textfile	2	TRUE	NA
rs1417236	T	C	T	C	0.023669	0.0085	0.029641	0.0307	FALSE	FALSE	FALSE	wuktJL	0.0128	0.508	major dep	TRUE	reported	textfile	0.004513	other alcol	TRUE	1.56E-07	inferred	UkHULc	textfile	2	TRUE	NA
rs1439564	G	A	G	A	0.024477	0.0066	0.024077	0.0233	FALSE	FALSE	FALSE	wuktJL	0.0156	0.671699	major dep	TRUE	reported	textfile	0.005323	other alcol	TRUE	4.25E-06	inferred	UkHULc	textfile	2	TRUE	NA
rs1454395	T	C	T	C	0.048038	0.0081	0.006686	0.0079	FALSE	FALSE	FALSE	wuktJL	0.0264	0.7579	major dep	TRUE	reported	textfile	0.009811	other alcol	TRUE	9.76E-07	inferred	UkHULc	textfile	2	TRUE	NA
rs1457728	G	C	G	C	0.028549	0.0138	0.015665	0.0151	FALSE	TRUE	FALSE	wuktJL	0.0182	0.4481	major dep	TRUE	reported	textfile	0.006113	other alcol	TRUE	3.01E-06	inferred	UkHULc	textfile	2	TRUE	NA
rs1480631	A	G	A	G	0.029938	-0.0172	0.014899	0.0152	FALSE	FALSE	FALSE	wuktJL	0.0179	0.3372	major dep	TRUE	reported	textfile	0.006247	other alcol	TRUE	1.65E-06	inferred	UkHULc	textfile	2	TRUE	NA
rs1808743	C	T	C	T	0.037947	0.0128	0.009568	0.0098	FALSE	FALSE	FALSE	wuktJL	0.0237	0.5911	major dep	TRUE	reported	textfile	0.008154	other alcol	TRUE	3.26E-06	inferred	UkHULc	textfile	2	TRUE	NA
rs1841443	A	G	A	G	0.027218	0.0052	0.018556	0.0185	FALSE	FALSE	FALSE	wuktJL	0.0176	0.765701	major dep	TRUE	reported	textfile	0.005949	other alcol	TRUE	4.75E-06	inferred	UkHULc	textfile	2	TRUE	NA
rs2008984	A	T	A	T	0.01393	0.0058	0.068068	0.0668	FALSE	TRUE	FALSE	wuktJL	0.0087	0.5037	major dep	TRUE	reported	textfile	0.002998	other alcol	TRUE	3.38E-06	inferred	UkHULc	textfile	2	TRUE	NA
rs309494	C	T	C	T	0.007181	-0.003	0.525347	0.5262	FALSE	FALSE	FALSE	wuktJL	0.0044	0.4902	major dep	TRUE	reported	textfile	0.001516	other alcol	TRUE	2.17E-06	inferred	UkHULc	textfile	2	TRUE	NA
rs358054	G	A	G	A	0.007032	7.00E-04	0.481087	0.4778	FALSE	FALSE	FALSE	wuktJL	0.0043	0.8707	major dep	TRUE	reported	textfile	0.001507	other alcol	TRUE	3.09E-06	inferred	UkHULc	textfile	2	TRUE	NA
rs4129990	A	G	A	G	0.045973	-0.0195	0.008728	0.0096	FALSE	FALSE	FALSE	wuktJL	0.025	0.4364	major dep	TRUE	reported	textfile	0.008758	other alcol	TRUE	1.53E-07	inferred	UkHULc	textfile	2	TRUE	NA
rs4280024	G	C	G	C	0.031906	0.0343	0.012181	0.0128	FALSE	TRUE	FALSE	wuktJL	0.0196	0.079631	major dep	TRUE	reported	textfile	0.006905	other alcol	TRUE	3.83E-06	inferred	UkHULc	textfile	2	TRUE	NA
rs4880425	A	G	A	G	-0.00699	0.0063	0.497613	0.5046	FALSE	FALSE	FALSE	wuktJL	0.0044	0.1573	major dep	TRUE	reported	textfile	0.001499	other alcol	TRUE	3.15E-06	inferred	UkHULc	textfile	2	TRUE	NA
rs7091162	T	G	T	G	0.007834	0.0163	0.302975	0.3049	FALSE	FALSE	FALSE	wuktJL	0.0047	0.000488	major dep	TRUE	reported	textfile	0.001642	other alcol	TRUE	1.83E-06	inferred	UkHULc	textfile	2	TRUE	NA
rs7234196	G	A	G	A	0.008692	0.0025	0.226077	0.2238	FALSE	FALSE	FALSE	wuktJL	0.0052	0.6256	major dep	TRUE	reported	textfile	0.001822	other alcol	TRUE	1.83E-06	inferred	UkHULc	textfile	2	TRUE	NA
rs7265829	T	C	T	C	0.026255	-0.0224	0.018971	0.0185	FALSE	FALSE	FALSE	wuktJL	0.0165	0.176	major dep	TRUE	reported	textfile	0.005552	other alcol	TRUE	2.26E-06	inferred	UkHULc	textfile	2	TRUE	NA
rs7323411	T	C	T	C	0.030037	0.0026	0.016272	0.0156	FALSE	FALSE	FALSE	wuktJL	0.0186	0.8905	major dep	TRUE	reported	textfile	0.006103	other alcol	TRUE	8.59E-07	inferred	UkHULc	textfile	2	TRUE	NA
rs7394506	C	G	C	G	0.031157	-0.0139	0.012381	0.0124	FALSE	TRUE	FALSE	wuktJL	0.0199	0.4854	major dep	TRUE	reported	textfile	0.006784	other alcol	TRUE	4.38E-06	inferred	UkHULc	textfile	2	TRUE	NA
rs7435629	A	G	A	G	0.01651	-0.0066	0.049916	0.0481	FALSE	FALSE	FALSE	wuktJL	0.0102	0.5156	major dep	TRUE	reported	textfile	0.003497	other alcol	TRUE	2.35E-06	inferred	UkHULc	textfile	2	TRUE	NA
rs7610583	T	G	T	G	0.017231	-0.013	0.051608	0.0512	FALSE	FALSE	FALSE	wuktJL	0.0103	0.2082	major dep	TRUE	reported	textfile	0.003477	other alcol	TRUE	7.21E-07	inferred	UkHULc	textfile	2	TRUE	NA
rs7695589	A	C	A	C	0.007911	0.0065	0.576342	0.5783	FALSE	FALSE	FALSE	wuktJL	0.0044	0.138	major dep	TRUE	reported	textfile	0.001521	other alcol	TRUE	1.99E-07	inferred	UkHULc	textfile	2	TRUE	NA
rs7764117	C	T	C	T	0.015942	-0.0024	0.053307	0.0522	FALSE	FALSE	FALSE	wuktJL	0.0102	0.8125	major dep	TRUE	reported	textfile	0.003448	other alcol	TRUE	3.77E-06	inferred	UkHULc	textfile	2	TRUE	NA
rs7765815	A	G	A	G	0.024638	-0.0107	0.021434	0.0216	FALSE	FALSE	FALSE	wuktJL	0.015	0.4764	major dep	TRUE	reported	textfile	0.005196	other alcol	TRUE	2.12E-06	inferred	UkHULc	textfile	2	TRUE	NA
rs7804909	C	T	C	T	0.039389	-0.0541	0.009462	0.0096	FALSE	FALSE	FALSE	wuktJL	0.0229	0.01829	major dep	TRUE	reported	textfile	0.007755	other alcol	TRUE	3.79E-07	inferred	UkHULc	textfile	2	TRUE	NA
rs8002446	T	C	T	C	0.032258	0.0072	0.01198	0.0121	FALSE	FALSE	FALSE	wuktJL	0.0206	0.7271	major dep	TRUE	reported	textfile	0.006908	other alcol	TRUE	3.02E-06	inferred	UkHULc	textfile	2	TRUE	NA
rs9466782	C	A	C	A	0.017736	0.0228	0.046571	0.0466	FALSE	FALSE	FALSE	wuktJL	0.0104	0.0276	major dep	TRUE	reported	textfile	0.003591	other alcol	TRUE	7.84E-07	inferred	UkHULc	textfile	2	TRUE	NA
