# Supplementary material for: Risk of major depressive increases with increasing frequency of alcohol drinking: a bidirectional two-sample Mendelian randomization analysis
Source: Front Public Health. 2024 Jun 5;12:1372758. doi: 10.3389/fpubh.2024.1372758 (PMC11186411; doi:10.3389/fpubh.2024.1372758)
Supplement: Supplementary file 8 [file Data_Sheet_7.PDF]

SNPs of MDD on alcohol consumption

SNP	effect_allele	other_allele	effect_allele	other_allele	beta.expos	beta.outco	eaf.exposu	eaf.outcom	remove	palindromi	ambiguous	id.outcome	se.outcome	pval.outco	outcome	mr_keep.o	pval_origin	data_sourc	se.exposure	exposure	mr_keep.e	pval.exposu	pval_origin	id.exposure	data_sourc	action	mr_keep	samplesize.outcome
rs1021363	G	A	G	A	-0.03	0.003619	0.6434	0.6418	FALSE	FALSE	FALSE	mdGfqh	0.002994	0.2268	alcohol cor	TRUE	reported	textfile	0.0045	major depr	TRUE	2.62E-11	inferred	I726FR	textfile	2	TRUE	NA
rs1083154	C	T	C	T	-0.0294	-0.00402	0.4952	0.4968	FALSE	FALSE	FALSE	mdGfqh	0.003108	0.1956	alcohol cor	TRUE	reported	textfile	0.0044	major depr	TRUE	2.36E-11	inferred	I726FR	textfile	2	TRUE	NA
rs1161231	C	T	C	T	0.0309	0.000374	0.2035	0.1974	FALSE	FALSE	FALSE	mdGfqh	0.003016	0.9013	alcohol cor	TRUE	reported	textfile	0.0054	major depr	TRUE	1.05E-08	inferred	I726FR	textfile	2	TRUE	NA
rs1204024	C	T	C	T	-0.0259	0.002732	0.3806	0.3783	FALSE	FALSE	FALSE	mdGfqh	0.002985	0.3601	alcohol cor	TRUE	reported	textfile	0.0044	major depr	TRUE	3.95E-09	inferred	I726FR	textfile	2	TRUE	NA
rs1291929	C	G	C	G	0.0327	0.003583	0.1884	0.1843	FALSE	TRUE	FALSE	mdGfqh	0.002991	0.231	alcohol cor	TRUE	reported	textfile	0.0055	major depr	TRUE	2.76E-09	inferred	I726FR	textfile	2	TRUE	NA
rs1296714	C	G	C	G	-0.0345	0.008357	0.7012	0.702	FALSE	TRUE	FALSE	mdGfqh	0.002974	0.004951	alcohol cor	TRUE	reported	textfile	0.0047	major depr	TRUE	2.13E-13	inferred	I726FR	textfile	2	TRUE	NA
rs1503469	T	C	T	C	0.0283	-0.00053	0.4118	0.4087	FALSE	FALSE	FALSE	mdGfqh	0.002989	0.8603	alcohol cor	TRUE	reported	textfile	0.0044	major depr	TRUE	1.26E-10	inferred	I726FR	textfile	2	TRUE	NA
rs1773764	G	A	G	A	0.0705	0.002828	0.0327	0.03345	FALSE	FALSE	FALSE	mdGfqh	0.002976	0.342	alcohol cor	TRUE	reported	textfile	0.012	major depr	TRUE	4.23E-09	inferred	I726FR	textfile	2	TRUE	NA
rs1931388	G	A	G	A	-0.0295	-0.00378	0.4042	0.4037	FALSE	FALSE	FALSE	mdGfqh	0.003007	0.2087	alcohol cor	TRUE	reported	textfile	0.0044	major depr	TRUE	2.02E-11	inferred	I726FR	textfile	2	TRUE	NA
rs1950829	G	A	G	A	-0.0297	0.003605	0.5173	0.5206	FALSE	FALSE	FALSE	mdGfqh	0.002987	0.2275	alcohol cor	TRUE	reported	textfile	0.0043	major depr	TRUE	4.95E-12	inferred	I726FR	textfile	2	TRUE	NA
rs198457	T	C	T	C	-0.0315	-0.00426	0.1886	0.1837	FALSE	FALSE	FALSE	mdGfqh	0.003011	0.1569	alcohol cor	TRUE	reported	textfile	0.0056	major depr	TRUE	1.86E-08	inferred	I726FR	textfile	2	TRUE	NA
rs2111592	A	G	A	G	0.0263	-0.00519	0.3141	0.3114	FALSE	FALSE	FALSE	mdGfqh	0.002982	0.0816	alcohol cor	TRUE	reported	textfile	0.0046	major depr	TRUE	1.08E-08	inferred	I726FR	textfile	2	TRUE	NA
rs2418449	C	T	C	T	-0.0281	0.002363	0.281	0.2799	FALSE	FALSE	FALSE	mdGfqh	0.002975	0.4271	alcohol cor	TRUE	reported	textfile	0.0048	major depr	TRUE	4.79E-09	inferred	I726FR	textfile	2	TRUE	NA
rs247910	G	A	G	A	0.0237	0.009909	0.457	0.4568	FALSE	FALSE	FALSE	mdGfqh	0.003018	0.001027	alcohol cor	TRUE	reported	textfile	0.0043	major depr	TRUE	3.56E-08	inferred	I726FR	textfile	2	TRUE	NA
rs2488389	A	G	A	G	-0.0295	-0.00475	0.2179	0.2161	FALSE	FALSE	FALSE	mdGfqh	0.002986	0.1119	alcohol cor	TRUE	reported	textfile	0.0052	major depr	TRUE	1.40E-08	inferred	I726FR	textfile	2	TRUE	NA
rs2522831	C	T	C	T	0.024	-0.00205	0.4739	0.4768	FALSE	FALSE	FALSE	mdGfqh	0.002987	0.4932	alcohol cor	TRUE	reported	textfile	0.0043	major depr	TRUE	2.39E-08	inferred	I726FR	textfile	2	TRUE	NA
rs2854141	G	C	G	C	-0.0292	0.002417	0.2308	0.2241	FALSE	TRUE	FALSE	mdGfqh	0.00304	0.4266	alcohol cor	TRUE	reported	textfile	0.0052	major depr	TRUE	1.96E-08	inferred	I726FR	textfile	2	TRUE	NA
rs30266	A	G	A	G	0.0366	-0.00161	0.3271	0.3267	FALSE	FALSE	FALSE	mdGfqh	0.003003	0.592899	alcohol cor	TRUE	reported	textfile	0.0046	major depr	TRUE	1.77E-15	inferred	I726FR	textfile	2	TRUE	NA
rs354155	C	G	C	G	-0.0449	-0.0044	0.0923	0.08926	FALSE	TRUE	FALSE	mdGfqh	0.002976	0.139	alcohol cor	TRUE	reported	textfile	0.0075	major depr	TRUE	2.14E-09	inferred	I726FR	textfile	2	TRUE	NA
rs3807865	A	G	A	G	0.031	-0.00688	0.4105	0.4128	FALSE	FALSE	FALSE	mdGfqh	0.002984	0.02114	alcohol cor	TRUE	reported	textfile	0.0044	major depr	TRUE	1.85E-12	inferred	I726FR	textfile	2	TRUE	NA
rs4141983	C	T	C	T	-0.0264	8.16E-05	0.326	0.3254	FALSE	FALSE	FALSE	mdGfqh	0.003011	0.9784	alcohol cor	TRUE	reported	textfile	0.0046	major depr	TRUE	9.52E-09	inferred	I726FR	textfile	2	TRUE	NA
rs508502	T	C	T	C	-0.0264	-0.0007	0.2992	0.2936	FALSE	FALSE	FALSE	mdGfqh	0.003051	0.8177	alcohol cor	TRUE	reported	textfile	0.0048	major depr	TRUE	3.80E-08	inferred	I726FR	textfile	2	TRUE	NA
rs5908293	T	C	T	C	0.0363	0.002025	0.1342	0.1203	FALSE	FALSE	FALSE	mdGfqh	0.003098	0.5134	alcohol cor	TRUE	reported	textfile	0.0066	major depr	TRUE	3.80E-08	inferred	I726FR	textfile	2	TRUE	NA
rs5928317	A	G	A	G	-0.039	0.003497	0.1081	0.1075	FALSE	FALSE	FALSE	mdGfqh	0.002982	0.2408	alcohol cor	TRUE	reported	textfile	0.007	major depr	TRUE	2.53E-08	inferred	I726FR	textfile	2	TRUE	NA
rs6253571	A	G	A	G	0.0339	0.00047	0.1639	0.1592	FALSE	FALSE	FALSE	mdGfqh	0.002985	0.8749	alcohol cor	TRUE	reported	textfile	0.0058	major depr	TRUE	5.07E-09	inferred	I726FR	textfile	2	TRUE	NA
rs6798181	G	C	G	C	-0.062	0.00344	0.1056	0.116	FALSE	TRUE	FALSE	mdGfqh	0.002975	0.2474	alcohol cor	TRUE	reported	textfile	0.007	major depr	TRUE	8.21E-19	inferred	I726FR	textfile	2	TRUE	NA
rs699927	G	T	G	T	0.024	0.006638	0.4171	0.4168	FALSE	FALSE	FALSE	mdGfqh	0.00298	0.02592	alcohol cor	TRUE	reported	textfile	0.0044	major depr	TRUE	4.91E-08	inferred	I726FR	textfile	2	TRUE	NA
rs7152906	C	T	C	T	0.0258	-0.00257	0.5196	0.5192	FALSE	FALSE	FALSE	mdGfqh	0.002978	0.3889	alcohol cor	TRUE	reported	textfile	0.0043	major depr	TRUE	1.97E-09	inferred	I726FR	textfile	2	TRUE	NA
rs7241572	A	G	A	G	0.0323	0.005044	0.2047	0.1991	FALSE	FALSE	FALSE	mdGfqh	0.003063	0.09958	alcohol cor	TRUE	reported	textfile	0.0054	major depr	TRUE	2.21E-09	inferred	I726FR	textfile	2	TRUE	NA
rs7294850	A	G	A	G	0.0265	-0.00041	0.2975	0.3007	FALSE	FALSE	FALSE	mdGfqh	0.002993	0.8906	alcohol cor	TRUE	reported	textfile	0.0047	major depr	TRUE	1.72E-08	inferred	I726FR	textfile	2	TRUE	NA
rs7538938	C	T	C	T	0.0251	-0.00385	0.5599	0.563	FALSE	FALSE	FALSE	mdGfqh	0.002998	0.1988	alcohol cor	TRUE	reported	textfile	0.0043	major depr	TRUE	5.31E-09	inferred	I726FR	textfile	2	TRUE	NA
rs754287	A	T	A	T	-0.0289	0.003239	0.3664	0.3711	FALSE	TRUE	FALSE	mdGfqh	0.003003	0.2808	alcohol cor	TRUE	reported	textfile	0.0045	major depr	TRUE	1.34E-10	inferred	I726FR	textfile	2	TRUE	NA
rs7551758	G	T	G	T	0.0283	-0.00144	0.5329	0.5338	FALSE	FALSE	FALSE	mdGfqh	0.002989	0.630999	alcohol cor	TRUE	reported	textfile	0.0043	major depr	TRUE	4.66E-11	inferred	I726FR	textfile	2	TRUE	NA
rs7617480	C	A	C	A	-0.029	-0.0053	0.7739	0.7744	FALSE	FALSE	FALSE	mdGfqh	0.002974	0.07471	alcohol cor	TRUE	reported	textfile	0.0051	major depr	TRUE	1.30E-08	inferred	I726FR	textfile	2	TRUE	NA
rs7695401	A	T	A	T	0.0412	0.006065	0.0931	0.0924	FALSE	TRUE	FALSE	mdGfqh	0.002985	0.04217	alcohol cor	TRUE	reported	textfile	0.0074	major depr	TRUE	2.58E-08	inferred	I726FR	textfile	2	TRUE	NA
rs7721129	A	G	A	G	0.029	-0.00303	0.5343	0.5361	FALSE	FALSE	FALSE	mdGfqh	0.002979	0.3092	alcohol cor	TRUE	reported	textfile	0.0043	major depr	TRUE	1.54E-11	inferred	I726FR	textfile	2	TRUE	NA
rs7797112	C	T	C	T	-0.027	0.002509	0.2533	0.2456	FALSE	FALSE	FALSE	mdGfqh	0.002988	0.4012	alcohol cor	TRUE	reported	textfile	0.0049	major depr	TRUE	3.58E-08	inferred	I726FR	textfile	2	TRUE	NA
rs843812	A	G	A	G	0.0248	-0.0037	0.4117	0.4069	FALSE	FALSE	FALSE	mdGfqh	0.003005	0.2181	alcohol cor	TRUE	reported	textfile	0.0044	major depr	TRUE	1.74E-08	inferred	I726FR	textfile	2	TRUE	NA
rs9074	A	G	A	G	0.031	0.001816	0.2597	0.2595	FALSE	FALSE	FALSE	mdGfqh	0.002974	0.5413	alcohol cor	TRUE	reported	textfile	0.0049	major depr	TRUE	2.51E-10	inferred	I726FR	textfile	2	TRUE	NA
rs9364755	G	A	G	A	0.0283	-0.00117	0.2262	0.224	FALSE	FALSE	FALSE	mdGfqh	0.002997	0.6968	alcohol cor	TRUE	reported	textfile	0.0051	major depr	TRUE	2.87E-08	inferred	I726FR	textfile	2	TRUE	NA
rs9529314	A	G	A	G	-0.0335	0.007425	0.2028	0.1997	FALSE	FALSE	FALSE	mdGfqh	0.003006	0.01352	alcohol cor	TRUE	reported	textfile	0.0053	major depr	TRUE	2.60E-10	inferred	I726FR	textfile	2	TRUE	NA
rs9596774	T	C	T	C	0.0255	0.001663	0.3261	0.3314	FALSE	FALSE	FALSE	mdGfqh	0.002979	0.5768	alcohol cor	TRUE	reported	textfile	0.0046	major depr	TRUE	2.97E-08	inferred	I726FR	textfile	2	TRUE	NA

SNPs of MDD on alcohol intake frequency

SNP	effect_allel	other_allel	effect_allel	other_allel	beta.expos	beta.outc	eaf.expos	eaf.outcom	remove	palindrom	ambiguou	id.outcom	se.outcom	pval.outco	outcome	mr_keep.o	pval_origir	data_sour	se.exposur	exposure	mr_keep.e	pval.expos	pval_origir	id.exposur	data_sour	action	mr_keep	samplesize.outcome
rs1021363	G	A	G	A	-0.03	-0.01486	0.6434	0.643608	FALSE	FALSE	FALSE	5jgP02	0.003165	2.70E-06	alcohol int	TRUE	reported	textfile	0.0045	major dep	TRUE	2.62E-11	inferred	cyAczG	textfile	2	TRUE	NA
rs1161231	C	T	C	T	0.0309	0.00362	0.2035	0.202631	FALSE	FALSE	FALSE	5jgP02	0.003784	0.34	alcohol int	TRUE	reported	textfile	0.0054	major dep	TRUE	1.05E-08	inferred	cyAczG	textfile	2	TRUE	NA
rs1204024	C	T	C	T	-0.0259	-0.00241	0.3806	0.379495	FALSE	FALSE	FALSE	5jgP02	0.003116	0.44	alcohol int	TRUE	reported	textfile	0.0044	major dep	TRUE	3.95E-09	inferred	cyAczG	textfile	2	TRUE	NA
rs1291929	C	G	C	G	0.0327	-0.00523	0.1884	0.189255	FALSE	TRUE	FALSE	5jgP02	0.003874	0.18	alcohol int	TRUE	reported	textfile	0.0055	major dep	TRUE	2.76E-09	inferred	cyAczG	textfile	2	TRUE	NA
rs1296714	C	G	C	G	-0.0345	-0.01277	0.7012	0.699464	FALSE	TRUE	FALSE	5jgP02	0.003322	0.00012	alcohol int	TRUE	reported	textfile	0.0047	major dep	TRUE	2.13E-13	inferred	cyAczG	textfile	2	TRUE	NA
rs1773764	G	A	G	A	0.0705	0.001843	0.0327	0.033463	FALSE	FALSE	FALSE	5jgP02	0.008426	0.83	alcohol int	TRUE	reported	textfile	0.012	major dep	TRUE	4.23E-09	inferred	cyAczG	textfile	2	TRUE	NA
rs1931388	G	A	G	A	-0.0295	0.001184	0.4042	0.403386	FALSE	FALSE	FALSE	5jgP02	0.003098	0.7	alcohol int	TRUE	reported	textfile	0.0044	major dep	TRUE	2.02E-11	inferred	cyAczG	textfile	2	TRUE	NA
rs1950829	G	A	G	A	-0.0297	-0.00335	0.5173	0.518605	FALSE	FALSE	FALSE	5jgP02	0.003034	0.27	alcohol int	TRUE	reported	textfile	0.0043	major dep	TRUE	4.95E-12	inferred	cyAczG	textfile	2	TRUE	NA
rs198457	T	C	T	C	-0.0315	0.012893	0.1886	0.188674	FALSE	FALSE	FALSE	5jgP02	0.003894	0.00093	alcohol int	TRUE	reported	textfile	0.0056	major dep	TRUE	1.86E-08	inferred	cyAczG	textfile	2	TRUE	NA
rs2111592	A	G	A	G	0.0263	0.005421	0.3141	0.311906	FALSE	FALSE	FALSE	5jgP02	0.003263	0.097	alcohol int	TRUE	reported	textfile	0.0046	major dep	TRUE	1.08E-08	inferred	cyAczG	textfile	2	TRUE	NA
rs2214123	G	A	G	A	-0.0261	-0.002	0.6466	0.647267	FALSE	FALSE	FALSE	5jgP02	0.003198	0.53	alcohol int	TRUE	reported	textfile	0.0045	major dep	TRUE	6.63E-09	inferred	cyAczG	textfile	2	TRUE	NA
rs2418449	C	T	C	T	-0.0281	-0.00438	0.281	0.280185	FALSE	FALSE	FALSE	5jgP02	0.003372	0.19	alcohol int	TRUE	reported	textfile	0.0048	major dep	TRUE	4.79E-09	inferred	cyAczG	textfile	2	TRUE	NA
rs2488389	A	G	A	G	-0.0295	0.017011	0.2179	0.218142	FALSE	FALSE	FALSE	5jgP02	0.003664	3.40E-06	alcohol int	TRUE	reported	textfile	0.0052	major dep	TRUE	1.40E-08	inferred	cyAczG	textfile	2	TRUE	NA
rs2522831	C	T	C	T	0.024	0.002403	0.4739	0.475527	FALSE	FALSE	FALSE	5jgP02	0.00303	0.43	alcohol int	TRUE	reported	textfile	0.0043	major dep	TRUE	2.39E-08	inferred	cyAczG	textfile	2	TRUE	NA
rs2854141	G	C	G	C	-0.0292	-0.00151	0.2308	0.23082	FALSE	TRUE	FALSE	5jgP02	0.003629	0.68	alcohol int	TRUE	reported	textfile	0.0052	major dep	TRUE	1.96E-08	inferred	cyAczG	textfile	2	TRUE	NA
rs30266	A	G	A	G	0.0366	0.008352	0.3271	0.328231	FALSE	FALSE	FALSE	5jgP02	0.003227	0.0096	alcohol int	TRUE	reported	textfile	0.0046	major dep	TRUE	1.77E-15	inferred	cyAczG	textfile	2	TRUE	NA
rs354155	C	G	C	G	-0.0449	0.013168	0.0923	0.089082	FALSE	TRUE	FALSE	5jgP02	0.005313	0.013	alcohol int	TRUE	reported	textfile	0.0075	major dep	TRUE	2.14E-09	inferred	cyAczG	textfile	2	TRUE	NA
rs3807865	A	G	A	G	0.031	0.005266	0.4105	0.41222	FALSE	FALSE	FALSE	5jgP02	0.00307	0.086	alcohol int	TRUE	reported	textfile	0.0044	major dep	TRUE	1.85E-12	inferred	cyAczG	textfile	2	TRUE	NA
rs4141983	C	T	C	T	-0.0264	0.007294	0.326	0.32533	FALSE	FALSE	FALSE	5jgP02	0.003242	0.024	alcohol int	TRUE	reported	textfile	0.0046	major dep	TRUE	9.52E-09	inferred	cyAczG	textfile	2	TRUE	NA
rs508502	T	C	T	C	-0.0264	0.001042	0.2992	0.300701	FALSE	FALSE	FALSE	5jgP02	0.003343	0.760001	alcohol int	TRUE	reported	textfile	0.0048	major dep	TRUE	3.80E-08	inferred	cyAczG	textfile	2	TRUE	NA
rs5908293	T	C	T	C	0.0363	-0.00391	0.1342	0.134166	FALSE	FALSE	FALSE	5jgP02	0.004564	0.39	alcohol int	TRUE	reported	textfile	0.0066	major dep	TRUE	3.80E-08	inferred	cyAczG	textfile	2	TRUE	NA
rs6253571	A	G	A	G	0.0339	0.008636	0.1639	0.161857	FALSE	FALSE	FALSE	5jgP02	0.004116	0.036	alcohol int	TRUE	reported	textfile	0.0058	major dep	TRUE	5.07E-09	inferred	cyAczG	textfile	2	TRUE	NA
rs6798181	G	C	G	C	-0.062	0.003602	0.1056	0.114102	FALSE	TRUE	FALSE	5jgP02	0.004753	0.450001	alcohol int	TRUE	reported	textfile	0.007	major dep	TRUE	8.21E-19	inferred	cyAczG	textfile	2	TRUE	NA
rs699927	G	T	G	T	0.024	-0.01144	0.4171	0.416377	FALSE	FALSE	FALSE	5jgP02	0.003072	0.0002	alcohol int	TRUE	reported	textfile	0.0044	major dep	TRUE	4.91E-08	inferred	cyAczG	textfile	2	TRUE	NA
rs7152906	C	T	C	T	0.0258	0.007495	0.5196	0.519501	FALSE	FALSE	FALSE	5jgP02	0.003027	0.013	alcohol int	TRUE	reported	textfile	0.0043	major dep	TRUE	1.97E-09	inferred	cyAczG	textfile	2	TRUE	NA
rs7241572	A	G	A	G	0.0323	0.008347	0.2047	0.208114	FALSE	FALSE	FALSE	5jgP02	0.003783	0.027	alcohol int	TRUE	reported	textfile	0.0054	major dep	TRUE	2.21E-09	inferred	cyAczG	textfile	2	TRUE	NA
rs7294850	A	G	A	G	0.0265	0.012644	0.2975	0.299596	FALSE	FALSE	FALSE	5jgP02	0.003305	0.00013	alcohol int	TRUE	reported	textfile	0.0047	major dep	TRUE	1.72E-08	inferred	cyAczG	textfile	2	TRUE	NA
rs7538938	C	T	C	T	0.0251	0.010339	0.5599	0.562721	FALSE	FALSE	FALSE	5jgP02	0.003054	0.00071	alcohol int	TRUE	reported	textfile	0.0043	major dep	TRUE	5.31E-09	inferred	cyAczG	textfile	2	TRUE	NA
rs754287	A	T	A	T	-0.0289	-0.00481	0.3664	0.369685	FALSE	TRUE	FALSE	5jgP02	0.003149	0.13	alcohol int	TRUE	reported	textfile	0.0045	major dep	TRUE	1.34E-10	inferred	cyAczG	textfile	2	TRUE	NA
rs7551758	G	T	G	T	0.0283	-0.00074	0.5329	0.533643	FALSE	FALSE	FALSE	5jgP02	0.003036	0.81	alcohol int	TRUE	reported	textfile	0.0043	major dep	TRUE	4.66E-11	inferred	cyAczG	textfile	2	TRUE	NA
rs7617480	C	A	C	A	-0.029	0.000727	0.7739	0.774103	FALSE	FALSE	FALSE	5jgP02	0.003621	0.84	alcohol int	TRUE	reported	textfile	0.0051	major dep	TRUE	1.30E-08	inferred	cyAczG	textfile	2	TRUE	NA
rs7695401	A	T	A	T	0.0412	-0.00674	0.0931	0.09355	FALSE	TRUE	FALSE	5jgP02	0.005203	0.2	alcohol int	TRUE	reported	textfile	0.0074	major dep	TRUE	2.58E-08	inferred	cyAczG	textfile	2	TRUE	NA
rs7721129	A	G	A	G	0.029	0.001695	0.5343	0.533285	FALSE	FALSE	FALSE	5jgP02	0.003032	0.58	alcohol int	TRUE	reported	textfile	0.0043	major dep	TRUE	1.54E-11	inferred	cyAczG	textfile	2	TRUE	NA
rs7797112	C	T	C	T	-0.027	-0.01311	0.2533	0.250218	FALSE	FALSE	FALSE	5jgP02	0.003498	0.00018	alcohol int	TRUE	reported	textfile	0.0049	major dep	TRUE	3.58E-08	inferred	cyAczG	textfile	2	TRUE	NA
rs843812	A	G	A	G	0.0248	-0.00565	0.4117	0.410747	FALSE	FALSE	FALSE	5jgP02	0.003089	0.066999	alcohol int	TRUE	reported	textfile	0.0044	major dep	TRUE	1.74E-08	inferred	cyAczG	textfile	2	TRUE	NA
rs9074	A	G	A	G	0.031	0.007044	0.2597	0.259567	FALSE	FALSE	FALSE	5jgP02	0.003455	0.041	alcohol int	TRUE	reported	textfile	0.0049	major dep	TRUE	2.51E-10	inferred	cyAczG	textfile	2	TRUE	NA
rs9364755	G	A	G	A	0.0283	0.005062	0.2262	0.22639	FALSE	FALSE	FALSE	5jgP02	0.003613	0.16	alcohol int	TRUE	reported	textfile	0.0051	major dep	TRUE	2.87E-08	inferred	cyAczG	textfile	2	TRUE	NA
rs9529314	A	G	A	G	-0.0335	-0.01374	0.2028	0.203597	FALSE	FALSE	FALSE	5jgP02	0.003759	0.00026	alcohol int	TRUE	reported	textfile	0.0053	major dep	TRUE	2.60E-10	inferred	cyAczG	textfile	2	TRUE	NA
rs9596774	T	C	T	C	0.0255	-0.00274	0.3261	0.327063	FALSE	FALSE	FALSE	5jgP02	0.003226	0.4	alcohol int	TRUE	reported	textfile	0.0046	major dep	TRUE	2.97E-08	inferred	cyAczG	textfile	2	TRUE	NA
