# Supplementary material for: Risk of major depressive increases with increasing frequency of alcohol drinking: a bidirectional two-sample Mendelian randomization analysis
Source: Front Public Health. 2024 Jun 5;12:1372758. doi: 10.3389/fpubh.2024.1372758 (PMC11186411; doi:10.3389/fpubh.2024.1372758)
Supplement: Supplementary file 9 [file Data_Sheet_8.PDF]

Cochran Q

id.exposur	id.outcome	outcome	exposure	method	Q	Q_df	Q_pval
cyAczG	5jgP02	alcohol int	major dep	Inverse var	183.0828	38	6.93E-21
