# Supplementary material for: Risk of major depressive increases with increasing frequency of alcohol drinking: a bidirectional two-sample Mendelian randomization analysis
Source: Front Public Health. 2024 Jun 5;12:1372758. doi: 10.3389/fpubh.2024.1372758 (PMC11186411; doi:10.3389/fpubh.2024.1372758)
Supplement: Supplementary file 10 [file Data_Sheet_9.PDF]

SNPs of alcohol consumption on Acid phosphodiesterase

SNP	effect_allele	other_allele	effect_allele	other_allele	beta.expos	beta.outco	eaf.exposu	eaf.outcom	remove	palindromi	ambiguous	id.outcome	se.outcome	pval.outcor	outcome	mr_keep.ou	pval_origin	data_sourc	se.exposure	exposure	mr_keep.ex	pval.exposu	pval_origin	id.exposure	data_sourc	action	mr_keep	samplesize.outcome
rs1194069	G	A	G	A	0.02694	0.0218	0.6063	0.59493	FALSE	FALSE	FALSE	DwLqdq	0.0253	0.389045	Acid sphin	TRUE	reported	textfile	0.003042	alcohol cor	TRUE	8.29E-19	inferred	vp8ESt	textfile	2	TRUE	NA
rs1260326	C	T	C	T	0.02839	-0.0371	0.6066	0.59849	FALSE	FALSE	FALSE	DwLqdq	0.0253	0.141254	Acid sphin	TRUE	reported	textfile	0.002973	alcohol cor	TRUE	1.31E-21	inferred	vp8ESt	textfile	2	TRUE	NA
rs1454527	C	G	C	G	-0.03427	0.1219	0.009985	0.01426	FALSE	TRUE	FALSE	DwLqdq	0.1046	0.245471	Acid sphin	TRUE	reported	textfile	0.002976	alcohol cor	TRUE	1.10E-30	inferred	vp8ESt	textfile	2	TRUE	NA
rs6232547	T	C	T	C	-0.01783	0.1721	0.007253	0.01085	FALSE	FALSE	FALSE	DwLqdq	0.1259	0.169824	Acid sphin	TRUE	reported	textfile	0.00298	alcohol cor	TRUE	2.19E-09	inferred	vp8ESt	textfile	2	TRUE	NA
rs9841829	G	T	G	T	0.01869	-0.0273	0.2269	0.22622	FALSE	FALSE	FALSE	DwLqdq	0.0295	0.354813	Acid sphin	TRUE	reported	textfile	0.002976	alcohol cor	TRUE	3.38E-10	inferred	vp8ESt	textfile	2	TRUE	NA

SNPs of alcohol consumption on BFP

SNP	effect_allel	other_allel	effect_allel	other_allel	beta.expos	beta.outco	eaf.exposu	eaf.outcom	remove	palindromi	ambiguous	id.outcome	se.outcome	pval.outco	outcome	mr_keep.ou	pval_origin	data_sourc	se.exposure	exposure	mr_keep.ex	pval.exposu	pval_origin	id.exposure	data_sourc	action	mr_keep	samplesize.outcome
rs1194069	G	A	G	A	0.02694	0.006045	0.6063	0.604237	FALSE	FALSE	FALSE	LWHhTs	0.001585	0.00014	body fat pe	TRUE	reported	textfile	0.003042	alcohol cor	TRUE	8.29E-19	inferred	Msb6Fj	textfile	2	TRUE	NA
rs1260326	C	T	C	T	0.02839	-0.00158	0.6066	0.604255	FALSE	FALSE	FALSE	LWHhTs	0.00157	0.31	body fat pe	TRUE	reported	textfile	0.002973	alcohol cor	TRUE	1.31E-21	inferred	Msb6Fj	textfile	2	TRUE	NA
rs9841829	G	T	G	T	0.01869	0.003704	0.2269	0.226867	FALSE	FALSE	FALSE	LWHhTs	0.001837	0.044	body fat pe	TRUE	reported	textfile	0.002976	alcohol cor	TRUE	3.38E-10	inferred	Msb6Fj	textfile	2	TRUE	NA

SNPs of alcohol consumption on BMI

SNP	effect_allel	other_allel	effect_allel	other_allel	beta.expos	beta.outco	eaf.exposu	eaf.outcom	remove	palindromi	ambiguous	id.outcome	se.outcome	pval.outcor	outcome	mr_keep.ou	pval_origin	data_sourc	se.exposure	exposure	mr_keep.ex	pval.expos	pval_origin	id.exposure	data_sourc	action	mr_keep	samplesize.outcome
rs1194069	G	A	G	A	0.02694	0.005098	0.6063	0.604181	FALSE	FALSE	FALSE	ubcOKS	0.002032	0.012	BMI	TRUE	reported	textfile	0.003042	alcohol cor	TRUE	8.29E-19	inferred	GSPf71	textfile	2	TRUE	NA
rs1260326	C	T	C	T	0.02839	0.009358	0.6066	0.604261	FALSE	FALSE	FALSE	ubcOKS	0.002012	3.30E-06	BMI	TRUE	reported	textfile	0.002973	alcohol cor	TRUE	1.31E-21	inferred	GSPf71	textfile	2	TRUE	NA
rs9841829	G	T	G	T	0.01869	0.014018	0.2269	0.226982	FALSE	FALSE	FALSE	ubcOKS	0.002355	2.60E-09	BMI	TRUE	reported	textfile	0.002976	alcohol cor	TRUE	3.38E-10	inferred	GSPf71	textfile	2	TRUE	NA

SNPs of alcohol consumption on CRP

SNP	effect_allel	other_allel	effect_allel	other_allel	beta.expos	beta.outco	eaf.exposu	eaf.outcom	remove	palindromi	ambiguous	id.outcome	se.outcom	pval.outco
rs1194069	G	A	G	A	0.02694	0.0082	0.6063	NA	FALSE	FALSE	FALSE	DJLyYP	0.0059	0.1624
rs1260326	C	T	C	T	0.02839	-0.062	0.6066	NA	FALSE	FALSE	FALSE	DJLyYP	0.0059	7.25E-26
rs9841829	G	T	G	T	0.01869	-0.0083	0.2269	NA	FALSE	FALSE	FALSE	DJLyYP	0.007	0.2386

SNP	outcome	mr_keep.ou	pval_origin	data_sourc	se.exposure	exposure	mr_keep.ex	pval.expos	pval_origin	id.exposure	data_sourc	action	mr_keep	samplesize.outcome
rs1194069	CRP	TRUE	reported	textfile	0.003042	alcohol cor	TRUE	8.29E-19	inferred	mI0NXX	textfile	2	TRUE	NA
rs1260326	CRP	TRUE	reported	textfile	0.002973	alcohol cor	TRUE	1.31E-21	inferred	mI0NXX	textfile	2	TRUE	NA
rs9841829	CRP	TRUE	reported	textfile	0.002976	alcohol cor	TRUE	3.38E-10	inferred	mI0NXX	textfile	2	TRUE	NA

SNPs of alcohol consumption on hydroxyhippurate

SNP	effect_allel	other_allel	effect_allel	other_allel	beta.expos	beta.outco	eaf.exposu	eaf.outcom	remove	palindromi	ambiguous	id.outcome	se.outcome	pval.outco	outcome	mr_keep.ou	pval_origin	data_sourc	se.exposure	exposure	mr_keep.ex	pval.expos	pval_origin	id.exposure	data_sourc	action	mr_keep	samplesize.outcome
rs1194069	G	A	G	A	0.02694	-0.008	0.6063	0.5743	FALSE	FALSE	FALSE	0h7RqK	0.0079	0.3083	4-hydroxyl	TRUE	reported	textfile	0.003042	alcohol co	TRUE	8.29E-19	inferred	cWL3WX	textfile	2	TRUE	NA
rs1260326	C	T	C	T	0.02839	0.0057	0.6066	0.5969	FALSE	FALSE	FALSE	0h7RqK	0.0077	0.4609	4-hydroxyl	TRUE	reported	textfile	0.002973	alcohol co	TRUE	1.31E-21	inferred	cWL3WX	textfile	2	TRUE	NA

SNPs of alcohol consumption on IL-6

SNP	effect_allel	other_allel	effect_allel	other_allel	beta.expos	beta.outco	eaf.exposu	eaf.outcom	remove	palindromi	ambiguous	id.outcome	se.outcome	pval.outco	outcome	mr_keep.ou	pval_origin	data_sourc	se.exposure	exposure	mr_keep.ex	pval.expos	pval_origin	id.exposure	data_sourc	action	mr_keep	samplesize.outcome
rs1194069	G	A	G	A	0.02694	0.0066	0.6063	NA	FALSE	FALSE	FALSE	yBiXmS	0.0163	0.683	interleukin-	TRUE	reported	textfile	0.003042	alcohol cor	TRUE	8.29E-19	inferred	HLGWC9	textfile	2	TRUE	NA
rs1260326	C	T	C	T	0.02839	-0.001	0.6066	NA	FALSE	FALSE	FALSE	yBiXmS	0.0164	0.9371	interleukin-	TRUE	reported	textfile	0.002973	alcohol cor	TRUE	1.31E-21	inferred	HLGWC9	textfile	2	TRUE	NA
rs9841829	G	T	G	T	0.01869	0.0152	0.2269	NA	FALSE	FALSE	FALSE	yBiXmS	0.0191	0.4383	interleukin-	TRUE	reported	textfile	0.002976	alcohol cor	TRUE	3.38E-10	inferred	HLGWC9	textfile	2	TRUE	NA

SNPs of alcohol consumption on minnitol

SNP	effect_allele	other_allele	effect_allele	other_allele	beta.expos	beta.outco	eaf.exposu	eaf.outcom	remove	palindromi	ambiguous	id.outcome	se.outcome	pval.outcor	outcome	mr_keep.ou	pval_origin	data_sourc	se.exposure	exposure	mr_keep.ex	pval.exposu	pval_origin	id.exposure	data_sourc	action	mr_keep	samplesize.outcome
rs1194069	G	A	G	A	0.02694	-0.0096	0.6063	0.5791	FALSE	FALSE	FALSE	WOP4CU	0.0099	0.3291	Minnitol	TRUE	reported	textfile	0.003042	alcohol cor	TRUE	8.29E-19	inferred	NxTtIr	textfile	2	TRUE	NA
rs1260326	C	T	C	T	0.02839	0.0069	0.6066	0.5933	FALSE	FALSE	FALSE	WOP4CU	0.0089	0.4384	Minnitol	TRUE	reported	textfile	0.002973	alcohol cor	TRUE	1.31E-21	inferred	NxTtIr	textfile	2	TRUE	NA

SNPs of alcohol consumption on Vitamin A

SNP	effect_allel	other_allel	effect_allel	other_allel	beta.expos	beta.outco	eaf.exposu	eaf.outcom	remove	palindromi	ambiguous	id.outcome	se.outcome	pval.outcor	outcome	mr_keep.ou	pval_origin	data_sourc	se.exposure	exposure	mr_keep.ex	pval.exposi	pval_origin	id.exposure	data_sourc	action	mr_keep	samplesize.outcome
rs1194069	G	A	G	A	0.02694	-0.00014	0.6063	0.60422	FALSE	FALSE	FALSE	cqOdRY	0.000295	0.630001	Vitamin A	TRUE	reported	textfile	0.003042	alcohol cor	TRUE	8.29E-19	inferred	msXTr5	textfile	2	TRUE	NA
rs1260326	C	T	C	T	0.02839	0.000251	0.6066	0.604342	FALSE	FALSE	FALSE	cqOdRY	0.000293	0.39	Vitamin A	TRUE	reported	textfile	0.002973	alcohol cor	TRUE	1.31E-21	inferred	msXTr5	textfile	2	TRUE	NA
rs9841829	G	T	G	T	0.01869	-5.79E-05	0.2269	0.227011	FALSE	FALSE	FALSE	cqOdRY	0.000342	0.87	Vitamin A	TRUE	reported	textfile	0.002976	alcohol cor	TRUE	3.38E-10	inferred	msXTr5	textfile	2	TRUE	NA

SNPs of alcohol intake frequency on Acid phosphodiesterase

SNP	effect_allel	other_allel	effect_allel	other_allel	beta.expos	beta.outco	eaf.exposu	eaf.outcom	remove	palindromi	ambiguous	id.outcome	se.outcome	pval.outcor	outcome	mr_keep.o	pval_origin	data_sourc	se.exposure	exposure	mr_keep.e	pval.expos	pval_origin	id.exposure	data_sourc	action	mr_keep	samplesize.outcome
rs1004787	A	G	A	G	-0.02223	-0.0696	0.532943	0.55009	FALSE	FALSE	FALSE	qg4VpY	0.0247	0.004786	Acid sphin	TRUE	reported	textfile	0.003045	alcohol inta	TRUE	2.90E-13	inferred	XqgiwA	textfile	2	TRUE	NA
rs1018831	T	C	T	C	-0.01979	-0.0485	0.470852	0.48103	FALSE	FALSE	FALSE	qg4VpY	0.0251	0.053703	Acid sphin	TRUE	reported	textfile	0.003036	alcohol inta	TRUE	7.16E-11	inferred	XqgiwA	textfile	2	TRUE	NA
rs1079266	G	A	G	A	0.017432	0.0195	0.505254	0.50456	FALSE	FALSE	FALSE	qg4VpY	0.0251	0.436516	Acid sphin	TRUE	reported	textfile	0.003041	alcohol inta	TRUE	9.86E-09	inferred	XqgiwA	textfile	2	TRUE	NA
rs1103942	T	C	T	C	-0.02356	0.0031	0.454624	0.44902	FALSE	FALSE	FALSE	qg4VpY	0.0247	0.891251	Acid sphin	TRUE	reported	textfile	0.003037	alcohol inta	TRUE	8.73E-15	inferred	XqgiwA	textfile	2	TRUE	NA
rs1122361	A	G	A	G	0.025091	0.0288	0.206155	0.21187	FALSE	FALSE	FALSE	qg4VpY	0.0302	0.338844	Acid sphin	TRUE	reported	textfile	0.003754	alcohol inta	TRUE	2.32E-11	inferred	XqgiwA	textfile	2	TRUE	NA
rs1170085	G	A	G	A	-0.0298	0.0559	0.093465	0.08538	FALSE	FALSE	FALSE	qg4VpY	0.0495	0.25704	Acid sphin	TRUE	reported	textfile	0.005233	alcohol inta	TRUE	1.24E-08	inferred	XqgiwA	textfile	2	TRUE	NA
rs1175077	A	G	A	G	-0.02049	-0.0279	0.209454	0.2063	FALSE	FALSE	FALSE	qg4VpY	0.0309	0.363078	Acid sphin	TRUE	reported	textfile	0.003726	alcohol inta	TRUE	3.80E-08	inferred	XqgiwA	textfile	2	TRUE	NA
rs1177994	C	G	C	G	-0.01967	0.0655	0.336989	0.36468	FALSE	TRUE	FALSE	qg4VpY	0.0284	0.02138	Acid sphin	TRUE	reported	textfile	0.00332	alcohol inta	TRUE	3.12E-09	inferred	XqgiwA	textfile	2	TRUE	NA
rs1178721	T	C	T	C	0.024416	-0.0347	0.369127	0.37224	FALSE	FALSE	FALSE	qg4VpY	0.0266	0.190546	Acid sphin	TRUE	reported	textfile	0.003201	alcohol inta	TRUE	2.38E-14	inferred	XqgiwA	textfile	2	TRUE	NA
rs1194069	G	A	G	A	-0.04371	0.0218	0.604193	0.59493	FALSE	FALSE	FALSE	qg4VpY	0.0253	0.389045	Acid sphin	TRUE	reported	textfile	0.003116	alcohol inta	TRUE	1.04E-44	inferred	XqgiwA	textfile	2	TRUE	NA
rs1215385	C	T	C	T	0.029444	0.0518	0.10497	0.10888	FALSE	FALSE	FALSE	qg4VpY	0.0396	0.190546	Acid sphin	TRUE	reported	textfile	0.004935	alcohol inta	TRUE	2.42E-09	inferred	XqgiwA	textfile	2	TRUE	NA
rs1228589	A	G	A	G	0.02107	0.0229	0.246133	0.2659	FALSE	FALSE	FALSE	qg4VpY	0.0286	0.426581	Acid sphin	TRUE	reported	textfile	0.003528	alcohol inta	TRUE	2.34E-09	inferred	XqgiwA	textfile	2	TRUE	NA
rs1229984	C	T	C	T	-0.26171	-0.0556	0.97277	0.96806	FALSE	FALSE	FALSE	qg4VpY	0.0702	0.426581	Acid sphin	TRUE	reported	textfile	0.009185	alcohol inta	TRUE	1.43E-178	inferred	XqgiwA	textfile	2	TRUE	NA
rs1310297	C	T	C	T	-0.01941	-0.0259	0.61881	0.62149	FALSE	FALSE	FALSE	qg4VpY	0.0256	0.30903	Acid sphin	TRUE	reported	textfile	0.003119	alcohol inta	TRUE	4.89E-10	inferred	XqgiwA	textfile	2	TRUE	NA
rs1313509	G	A	G	A	0.043834	0.0396	0.083483	0.08242	FALSE	FALSE	FALSE	qg4VpY	0.0458	0.389045	Acid sphin	TRUE	reported	textfile	0.005499	alcohol inta	TRUE	1.57E-15	inferred	XqgiwA	textfile	2	TRUE	NA
rs1317844	T	C	T	C	-0.01865	0.0313	0.276349	0.26758	FALSE	FALSE	FALSE	qg4VpY	0.0276	0.25704	Acid sphin	TRUE	reported	textfile	0.00339	alcohol inta	TRUE	3.75E-08	inferred	XqgiwA	textfile	2	TRUE	NA
rs1339001	C	T	C	T	0.029612	-0.0446	0.134041	0.14673	FALSE	FALSE	FALSE	qg4VpY	0.0354	0.20893	Acid sphin	TRUE	reported	textfile	0.004492	alcohol inta	TRUE	4.33E-11	inferred	XqgiwA	textfile	2	TRUE	NA
rs1421085	C	T	C	T	0.019939	0.0148	0.403447	0.41774	FALSE	FALSE	FALSE	qg4VpY	0.0251	0.562341	Acid sphin	TRUE	reported	textfile	0.003085	alcohol inta	TRUE	1.02E-10	inferred	XqgiwA	textfile	2	TRUE	NA
rs1515590	T	C	T	C	0.018245	0.034	0.383245	0.37335	FALSE	FALSE	FALSE	qg4VpY	0.026	0.190546	Acid sphin	TRUE	reported	textfile	0.003116	alcohol inta	TRUE	4.78E-09	inferred	XqgiwA	textfile	2	TRUE	NA
rs1666658	C	T	C	T	0.017967	-0.0022	0.392206	0.39086	FALSE	FALSE	FALSE	qg4VpY	0.0258	0.933254	Acid sphin	TRUE	reported	textfile	0.003099	alcohol inta	TRUE	6.69E-09	inferred	XqgiwA	textfile	2	TRUE	NA
rs1766275	C	T	C	T	0.030135	-0.0282	0.089115	0.09547	FALSE	FALSE	FALSE	qg4VpY	0.0448	0.524807	Acid sphin	TRUE	reported	textfile	0.00546	alcohol inta	TRUE	3.41E-08	inferred	XqgiwA	textfile	2	TRUE	NA
rs186347	T	G	T	G	0.017949	-0.0098	0.463343	0.46665	FALSE	FALSE	FALSE	qg4VpY	0.0242	0.691831	Acid sphin	TRUE	reported	textfile	0.003051	alcohol inta	TRUE	4.02E-09	inferred	XqgiwA	textfile	2	TRUE	NA
rs1937522	G	A	G	A	0.016898	-0.0193	0.528054	0.53017	FALSE	FALSE	FALSE	qg4VpY	0.0249	0.436516	Acid sphin	TRUE	reported	textfile	0.003032	alcohol inta	TRUE	2.50E-08	inferred	XqgiwA	textfile	2	TRUE	NA
rs1991083	T	C	T	C	-0.02239	0.0527	0.679886	0.67933	FALSE	FALSE	FALSE	qg4VpY	0.0268	0.050119	Acid sphin	TRUE	reported	textfile	0.003258	alcohol inta	TRUE	6.30E-12	inferred	XqgiwA	textfile	2	TRUE	NA
rs2043677	T	C	T	C	0.026113	-0.001	0.145599	0.14923	FALSE	FALSE	FALSE	qg4VpY	0.0347	0.977237	Acid sphin	TRUE	reported	textfile	0.004327	alcohol inta	TRUE	1.59E-09	inferred	XqgiwA	textfile	2	TRUE	NA
rs2159935	A	G	A	G	-0.01857	-0.0091	0.490369	0.49248	FALSE	FALSE	FALSE	qg4VpY	0.0246	0.707946	Acid sphin	TRUE	reported	textfile	0.003026	alcohol inta	TRUE	8.33E-10	inferred	XqgiwA	textfile	2	TRUE	NA
rs2160935	T	C	T	C	-0.01872	-0.0124	0.604293	0.6075	FALSE	FALSE	FALSE	qg4VpY	0.0257	0.630957	Acid sphin	TRUE	reported	textfile	0.003091	alcohol inta	TRUE	1.40E-09	inferred	XqgiwA	textfile	2	TRUE	NA
rs2244598	C	T	C	T	-0.01838	0.0082	0.605114	0.6081	FALSE	FALSE	FALSE	qg4VpY	0.0259	0.758578	Acid sphin	TRUE	reported	textfile	0.003119	alcohol inta	TRUE	3.81E-09	inferred	XqgiwA	textfile	2	TRUE	NA
rs2411453	G	T	G	T	-0.03508	0.0076	0.597353	0.59787	FALSE	FALSE	FALSE	qg4VpY	0.0254	0.758578	Acid sphin	TRUE	reported	textfile	0.00309	alcohol inta	TRUE	7.32E-30	inferred	XqgiwA	textfile	2	TRUE	NA
rs2535911	T	C	T	C	-0.01885	0.0062	0.354749	0.34473	FALSE	FALSE	FALSE	qg4VpY	0.0261	0.812831	Acid sphin	TRUE	reported	textfile	0.003168	alcohol inta	TRUE	2.71E-09	inferred	XqgiwA	textfile	2	TRUE	NA
rs2622167	A	G	A	G	-0.01912	0.0375	0.428653	0.43138	FALSE	FALSE	FALSE	qg4VpY	0.0251	0.134896	Acid sphin	TRUE	reported	textfile	0.003067	alcohol inta	TRUE	4.61E-10	inferred	XqgiwA	textfile	2	TRUE	NA
rs262240	T	C	T	C	-0.01721	-0.0109	0.468553	0.47551	FALSE	FALSE	FALSE	qg4VpY	0.0248	0.660693	Acid sphin	TRUE	reported	textfile	0.003035	alcohol inta	TRUE	1.43E-08	inferred	XqgiwA	textfile	2	TRUE	NA
rs2717063	A	C	A	C	-0.02037	-0.0031	0.585731	0.5742	FALSE	FALSE	FALSE	qg4VpY	0.0254	0.912011	Acid sphin	TRUE	reported	textfile	0.003085	alcohol inta	TRUE	4.00E-11	inferred	XqgiwA	textfile	2	TRUE	NA
rs2876812	C	T	C	T	0.0207	0.0021	0.759525	0.74909	FALSE	FALSE	FALSE	qg4VpY	0.0293	0.933254	Acid sphin	TRUE	reported	textfile	0.003552	alcohol inta	TRUE	5.62E-09	inferred	XqgiwA	textfile	2	TRUE	NA
rs2878710	A	G	A	G	0.017811	0.0109	0.40423	0.41093	FALSE	FALSE	FALSE	qg4VpY	0.0252	0.660693	Acid sphin	TRUE	reported	textfile	0.003085	alcohol inta	TRUE	7.74E-09	inferred	XqgiwA	textfile	2	TRUE	NA
rs324012	T	C	T	C	-0.01779	-0.0096	0.448569	0.45656	FALSE	FALSE	FALSE	qg4VpY	0.0248	0.691831	Acid sphin	TRUE	reported	textfile	0.003044	alcohol inta	TRUE	5.03E-09	inferred	XqgiwA	textfile	2	TRUE	NA
rs3444085	T	C	T	C	-0.02268	0.0122	0.157151	0.15642	FALSE	FALSE	FALSE	qg4VpY	0.0341	0.724436	Acid sphin	TRUE	reported	textfile	0.004151	alcohol inta	TRUE	4.63E-08	inferred	XqgiwA	textfile	2	TRUE	NA
rs3447388	A	G	A	G	-0.02036	-0.0302	0.24819	0.2489	FALSE	FALSE	FALSE	qg4VpY	0.0291	0.301995	Acid sphin	TRUE	reported	textfile	0.003503	alcohol inta	TRUE	6.18E-09	inferred	XqgiwA	textfile	2	TRUE	NA
rs3463102	T	C	T	C	-0.01691	0.0385	0.446061	0.44558	FALSE	FALSE	FALSE	qg4VpY	0.0249	0.123027	Acid sphin	TRUE	reported	textfile	0.003048	alcohol inta	TRUE	2.89E-08	inferred	XqgiwA	textfile	2	TRUE	NA
rs3481147	A	G	A	G	-0.02018	0.0507	0.230728	0.2259	FALSE	FALSE	FALSE	qg4VpY	0.0296	0.087096	Acid sphin	TRUE	reported	textfile	0.003593	alcohol inta	TRUE	1.95E-08	inferred	XqgiwA	textfile	2	TRUE	NA
rs3510514	T	C	T	C	0.026345	-0.04	0.401541	0.39715	FALSE	FALSE	FALSE	qg4VpY	0.0258	0.120226	Acid sphin	TRUE	reported	textfile	0.003088	alcohol inta	TRUE	1.44E-17	inferred	XqgiwA	textfile	2	TRUE	NA
rs362307	T	C	T	C	0.043305	0.0148	0.074582	0.07782	FALSE	FALSE	FALSE	qg4VpY	0.0469	0.758578	Acid sphin	TRUE	reported	textfile	0.005802	alcohol inta	TRUE	8.42E-14	inferred	XqgiwA	textfile	2	TRUE	NA
rs4241258	T	C	T	C	0.025064	0.0645	0.13763	0.13198	FALSE	FALSE	FALSE	qg4VpY	0.0369	0.081283	Acid sphin	TRUE	reported	textfile	0.004403	alcohol inta	TRUE	1.26E-08	inferred	XqgiwA	textfile	2	TRUE	NA
rs4242715	A	G	A	G	-0.01865	-0.0266	0.680585	0.67505	FALSE	FALSE	FALSE	qg4VpY	0.0264	0.316228	Acid sphin	TRUE	reported	textfile	0.003248	alcohol inta	TRUE	9.31E-09	inferred	XqgiwA	textfile	2	TRUE	NA
rs4417025	A	G	A	G	-0.01884	-0.042	0.361153	0.3639	FALSE	FALSE	FALSE	qg4VpY	0.0262	0.107152	Acid sphin	TRUE	reported	textfile	0.003165	alcohol inta	TRUE	2.65E-09	inferred	XqgiwA	textfile	2	TRUE	NA
rs4503294	T	C	T	C	0.018148	-0.031	0.565333	0.56804	FALSE	FALSE	FALSE	qg4VpY	0.025	0.213796	Acid sphin	TRUE	reported	textfile	0.00307	alcohol inta	TRUE	3.41E-09	inferred	XqgiwA	textfile	2	TRUE	NA
rs461599	C	A	C	A	-0.01919	0.0528	0.462259	0.45992	FALSE	FALSE	FALSE	qg4VpY	0.025	0.033884	Acid sphin	TRUE	reported	textfile	0.00304	alcohol inta	TRUE	2.74E-10	inferred	XqgiwA	textfile	2	TRUE	NA
rs4726481	T	G	T	G	0.021761	0.0332	0.400576	0.39697	FALSE	FALSE	FALSE	qg4VpY	0.0258	0.194984	Acid sphin	TRUE	reported	textfile	0.003102	alcohol inta	TRUE	2.29E-12	inferred	XqgiwA	textfile	2	TRUE	NA
rs4800487	G	A	G	A	-0.02894	-0.0128	0.456873	0.45307	FALSE	FALSE	FALSE	qg4VpY	0.0253	0.616595	Acid sphin	TRUE	reported	textfile	0.003047	alcohol inta	TRUE	2.16E-21	inferred	XqgiwA	textfile	2	TRUE	NA
rs489062	A	G	A	G	0.01665	-0.0046	0.437454	0.435	FALSE	FALSE	FALSE	qg4VpY	0.0251	0.851138	Acid sphin	TRUE	reported	textfile	0.003053	alcohol inta	TRUE	4.93E-08	inferred	XqgiwA	textfile	2	TRUE	NA
rs4916723	C	A	C	A	0.023948	0.0229	0.420617	0.41843	FALSE	FALSE	FALSE	qg4VpY	0.0257	0.371535	Acid sphin	TRUE	reported	textfile	0.0031	alcohol inta	TRUE	1.11E-14	inferred	XqgiwA	textfile	2	TRUE	NA
rs4968391	T	G	T	G	-0.01927	-0.0461	0.674892	0.67287	FALSE	FALSE	FALSE	qg4VpY	0.0264	0.081283	Acid sphin	TRUE	reported	textfile	0.003227	alcohol inta	TRUE	2.34E-09	inferred	XqgiwA	textfile	2	TRUE	NA
rs5022348	T	C	T	C	0.020264	0.0117	0.40703	0.57502	FALSE	FALSE	FALSE	qg4VpY	0.0249	0.630957	Acid sphin	TRUE	reported	textfile	0.00357	alcohol inta	TRUE	1.38E-08	inferred	XqgiwA	textfile	2	TRUE	NA
rs550942	T	C	T	C	0.022401	-0.0603	0.823865	0.82361	FALSE	FALSE	FALSE	qg4VpY	0.0333	0.070795	Acid sphin	TRUE	reported	textfile	0.003989	alcohol inta	TRUE	1.96E-08	inferred	XqgiwA	textfile	2	TRUE	NA
rs5619443	T	C	T	C	0.02254	0.0095	0.16931	0.17968	FALSE	FALSE	FALSE	qg4VpY	0.0324	0.776247	Acid sphin	TRUE	reported	textfile	0.004071	alcohol inta	TRUE	3.09E-08	inferred	XqgiwA	textfile	2	TRUE	NA
rs5890541	A	G	A	G	-0.02663	0.0017	0.410052	0.40589	FALSE	FALSE	FALSE	qg4VpY	0.0251	0.954993	Acid sphin	TRUE	reported	textfile	0.003078	alcohol inta	TRUE	5.07E-18	inferred	XqgiwA	textfile	2	TRUE	NA
rs6030312	T	G	T	G	-0.01953	-0.009	0.314251	0.30891	FALSE	FALSE	FALSE	qg4VpY	0.0269	0.74131	Acid sphin	TRUE	reported	textfile	0.003271	alcohol inta	TRUE	2.36E-09	inferred	XqgiwA	textfile	2	TRUE	NA
rs6187351	T	G	T	G	0.020374	-0.0463	0.32785	0.35352	FALSE	FALSE	FALSE	qg4VpY	0.027	0.087096	Acid sphin	TRUE	reported	textfile	0.003303	alcohol inta	TRUE	6.91E-10	inferred	XqgiwA	textfile	2	TRUE	NA
rs6230578	G	C	G	C	-0.04852	0.0027	0.102253	0.10629	FALSE	TRUE	FALSE	qg4VpY	0.0407	0.954993	Acid sphin	TRUE	reported	textfile	0.005066	alcohol inta	TRUE	9.88E-22	inferred	XqgiwA	textfile	2	TRUE	NA
rs6233967	A	C	A	C	0.018294	-0.0359	0.626705	0.61971	FALSE	FALSE	FALSE	qg4VpY	0.0257	0.162181	Acid sphin	TRUE	reported	textfile	0.003154	alcohol inta	TRUE	6.62E-09	inferred	XqgiwA	textfile	2	TRUE	NA
rs6246631	T	C	T	C	-0.02549	-0.0479	0.202827	0.19914	FALSE	FALSE	FALSE	qg4VpY	0.0309	0.120226	Acid sphin	TRUE	reported	textfile	0.003774	alcohol inta	TRUE	1.44E-11	inferred	XqgiwA	textfile	2	TRUE	NA
rs650558	T	C	T	C	0.020736	-0.0243	0.247918	0.2472	FALSE	FALSE	FALSE	qg4VpY	0.0285	0.389045	Acid sphin	TRUE	reported	textfile	0.003508	alcohol inta	TRUE	3.39E-09	inferred	XqgiwA	textfile	2	TRUE	NA
rs6727281	T	C	T	C	-0.02432	-0.0107	0.184023	0.18087	FALSE	FALSE	FALSE	qg4VpY	0.0323	0.74131	Acid sphin	TRUE	reported	textfile	0.00392	alcohol inta	TRUE	5.46E-10	inferred	XqgiwA	textfile	2	TRUE	NA
rs6943160	C	T	C	T	0.020627	-0.0308	0.208646	0.25369	FALSE	FALSE	FALSE	qg4VpY	0.0295	0.295121	Acid sphin	TRUE	reported	textfile	0.003728	alcohol inta	TRUE	3.14E-08	inferred	XqgiwA	textfile	2	TRUE	NA
rs7165168	T	C	T	C	-0.07046	0.0929	0.0142	0.01513	FALSE	FALSE	FALSE	qg4VpY	0.1006	0.354813	Acid sphin	TRUE	reported	textfile	0.012791	alcohol inta	TRUE	3.62E-08	inferred	XqgiwA	textfile	2	TRUE	NA
rs7231987	T	G	T	G	0.019026	0.0097	0.267222	0.26969	FALSE	FALSE	FALSE	qg4VpY	0.0273	0.724436	Acid sphin	TRUE	reported	textfile	0.003427	alcohol inta	TRUE	2.82E-08	inferred	XqgiwA	textfile	2	TRUE	NA
rs7276922	T	A	T	A	-0.02314	-0.0493	0.154942	0.15974	FALSE	TRUE	FALSE	qg4VpY	0.0352	0.162181	Acid sphin	TRUE	reported	textfile	0.004192	alcohol inta	TRUE	3.40E-08	inferred	XqgiwA	textfile	2	TRUE	NA
rs7278706	A	G	A	G	-0.02819	-0.0132	0.162767	0.16405	FALSE	FALSE	FALSE	qg4VpY	0.0335	0.691831	Acid sphin	TRUE	reported	textfile	0.004103	alcohol inta	TRUE	6.36E-12	inferred	XqgiwA	textfile	2	TRUE	NA
rs728538	G	T	G	T	0.022875	-0.0119	0.168868	0.17359	FALSE	FALSE	FALSE	qg4VpY	0.0333	0.724436	Acid sphin	TRUE	reported	textfile	0.004063	alcohol inta	TRUE	1.79E-08	inferred	XqgiwA	textfile	2	TRUE	NA
rs7298932	G	A	G	A	-0.02372	0.0051	0.147849	0.16161	FALSE	FALSE	FALSE	qg4VpY	0.0354	0.891251	Acid sphin	TRUE	reported	textfile	0.004312	alcohol inta	TRUE	3.76E-08	inferred	XqgiwA	textfile	2	TRUE	NA
rs7302200	A	G	A	G	-0.01842	-0.0043	0.339998	0.34545	FALSE	FALSE	FALSE	qg4VpY	0.026	0.870964	Acid sphin	TRUE	reported	textfile	0.003198	alcohol inta	TRUE	8.42E-09	inferred	XqgiwA	textfile	2	TRUE	NA
rs7305012	A	C	A	C	-0.026	-0.0246	0.164488	0.16044	FALSE	FALSE	FALSE	qg4VpY	0.0334	0.457088	Acid sphin	TRUE	reported	textfile	0.004091	alcohol inta	TRUE	2.06E-10	inferred	XqgiwA	textfile	2	TRUE	NA
rs7330939	T	C	T	C	-0.02133	5.00E-04	0.720352	0.63996	FALSE	FALSE	FALSE	qg4VpY	0.0281	0.977237	Acid sphin	TRUE	reported	textfile	0.003405	alcohol inta	TRUE	3.73E-10	inferred	XqgiwA	textfile	2	TRUE	NA
rs7460106	C	T	C	T	-0.02171	-0.0438	0.23562	0.22761	FALSE	FALSE	FALSE	qg4VpY	0.0299	0.144544	Acid sphin	TRUE	reported	textfile	0.003619	alcohol inta	TRUE	1.99E-09	inferred	XqgiwA	textfile	2	TRUE	NA
rs7467914	C	T	C	T	-0.03207	-0.0853	0.074515	0.07626	FALSE	FALSE	FALSE	qg4VpY	0.0465	0.067608	Acid sphin	TRUE	reported	textfile	0.005758	alcohol inta	TRUE	2.54E-08	inferred	XqgiwA	textfile	2	TRUE	NA
rs7514579	C	A	C	A	0.019667	0.0389	0.232457	0.2297	FALSE	FALSE	FALSE	qg4VpY	0.0297	0.190546	Acid sphin	TRUE	reported	textfile	0.003598	alcohol inta	TRUE	4.59E-08	inferred	XqgiwA	textfile	2	TRUE	NA
rs7608265	T	C	T	C	0.046427	-0.0254	0.054327	0.05566	FALSE	FALSE	FALSE	qg4VpY	0.0545	0.645654	Acid sphin	TRUE	reported	textfile	0.006687	alcohol inta	TRUE	3.83E-12	inferred	XqgiwA	textfile	2	TRUE	NA
rs7610856	A	C	A	C	-0.02386	0.0106	0.429053	0.43419	FALSE	FALSE	FALSE	qg4VpY	0.025	0.676083	Acid sphin	TRUE	reported	textfile	0.00307	alcohol inta	TRUE	7.68E-15	inferred	XqgiwA	textfile	2	TRUE	NA
rs780094	C	T	C	T	-0.05099	-0.0423	0.615206	0.60751	FALSE	FALSE	FALSE	qg4VpY	0.0253	0.093325	Acid sphin	TRUE	reported	textfile	0.003105	alcohol inta	TRUE	1.31E-60	inferred	XqgiwA	textfile	2	TRUE	NA
rs780569	A	T	A	T	0.019803	-0.0073	0.70882	0.71581	FALSE	TRUE	FALSE	qg4VpY	0.0273	0.794328	Acid sphin	TRUE	reported	textfile	0.003365	alcohol inta	TRUE	3.96E-09	inferred	XqgiwA	textfile	2	TRUE	NA
rs8029231	C	T	C	T	-0.03937	-0.0092	0.057704	0.05788	FALSE	FALSE	FALSE	qg4VpY	0.0536	0.870964	Acid sphin	TRUE	reported	textfile	0.006496	alcohol inta	TRUE	1.35E-09	inferred	XqgiwA	textfile	2	TRUE	NA
rs8043563	C	G	C	G	0.023365	-0.0099	0.737192	0.72086	FALSE	TRUE	FALSE	qg4VpY	0.0277	0.724436	Acid sphin	TRUE	reported	textfile	0.003471	alcohol inta	TRUE	1.68E-11	inferred	XqgiwA	textfile	2	TRUE	NA
rs8083110	T	C	T	C	-0.01681	-0.0016	0.418707	0.41183	FALSE	FALSE	FALSE	qg4VpY	0.0252	0.954993	Acid sphin	TRUE	reported	textfile	0.003078	alcohol inta	TRUE	4.72E-08	inferred	XqgiwA	textfile	2	TRUE	NA
rs838145	A	G	A	G	0.021955	0.0051	0.542982	0.54703	FALSE	FALSE	FALSE	qg4VpY	0.025	0.831764	Acid sphin	TRUE	reported	textfile	0.003055	alcohol inta	TRUE	6.70E-13	inferred	XqgiwA	textfile	2	TRUE	NA
rs8614	A	C	A	C	0.024781	0.0018	0.182509	0.1834	FALSE	FALSE	FALSE	qg4VpY	0.0321	0.954993	Acid sphin	TRUE	reported	textfile	0.003925	alcohol inta	TRUE	2.74E-10	inferred	XqgiwA	textfile	2	TRUE	NA
rs9349379	G	A	G	A	-0.01935	-0.0208	0.405493	0.40035	FALSE	FALSE	FALSE	qg4VpY	0.0247	0.398107	Acid sphin	TRUE	reported	textfile	0.003082	alcohol inta	TRUE	3.46E-10	inferred	XqgiwA	textfile	2	TRUE	NA
rs9372625	A	G	A	G	-0.02556	0.0106	0.381706	0.37772	FALSE	FALSE	FALSE	qg4VpY	0.0255	0.676083	Acid sphin	TRUE	reported	textfile	0.003125	alcohol inta	TRUE	2.85E-16	inferred	XqgiwA	textfile	2	TRUE	NA
rs9403297	A	G	A	G	0.018823	-0.0164	0.372967	0.37115	FALSE	FALSE	FALSE	qg4VpY	0.0254	0.512861	Acid sphin	TRUE	reported	textfile	0.00313	alcohol inta	TRUE	1.82E-09	inferred	XqgiwA	textfile	2	TRUE	NA
rs9648478	A	G	A	G	0.01686	0.0122	0.510245	0.50778	FALSE	FALSE	FALSE	qg4VpY	0.0248	0.616595	Acid sphin	TRUE	reported	textfile	0.003029	alcohol inta	TRUE	2.60E-08	inferred	XqgiwA	textfile	2	TRUE	NA
rs9814516	T	G	T	G	-0.02511	-0.025	0.237423	0.23603	FALSE	FALSE	FALSE	qg4VpY	0.0289	0.389045	Acid sphin	TRUE	reported	textfile	0.003556	alcohol inta	TRUE	1.64E-12	inferred	XqgiwA	textfile	2	TRUE	NA
rs9829192	T	G	T	G	0.016932	0.0454	0.435133	0.42836	FALSE	FALSE	FALSE	qg4VpY	0.0251	0.069183	Acid sphin	TRUE	reported	textfile	0.00305	alcohol inta	TRUE	2.84E-08	inferred	XqgiwA	textfile	2	TRUE	NA
rs9891943	C	T	C	T	0.019867	-0.023	0.232109	0.23634	FALSE	FALSE	FALSE	qg4VpY	0.0301	0.446684	Acid sphin	TRUE	reported	textfile	0.003641	alcohol inta	TRUE	4.88E-08	inferred	XqgiwA	textfile	2	TRUE	NA
rs9906502	A	G	A	G	0.023788	-0.0088	0.176998	0.17863	FALSE	FALSE	FALSE	qg4VpY	0.0325	0.794328	Acid sphin	TRUE	reported	textfile	0.003962	alcohol inta	TRUE	1.92E-09	inferred	XqgiwA	textfile	2	TRUE	NA

SNPs of alcohol intake frequency on BFP

SNP	effect_allel	other_allele	effect_allel	other_allele	beta.expos	beta.outco	eaf.exposu	eaf.outcom	remove	palindromi	ambiguous	id.outcome	se.outcome	pval.outco	outcome	mr_keep.o	pval_origin	data_sourc	se.exposure	exposure	mr_keep.e	pval.expos	pval_origin	id.exposure	data_sourc	action	mr_keep	samplesize.outcome
rs1018831	T	C	T	C	-0.01979	-0.00629	0.470852	0.470777	FALSE	FALSE	FALSE	rIZ4yc	0.001543	4.60E-05	body fat pe	TRUE	reported	textfile	0.003036	alcohol inta	TRUE	7.16E-11	inferred	JSsnZE	textfile	2	TRUE	NA
rs1079266	G	A	G	A	0.017432	0.004309	0.505254	0.505127	FALSE	FALSE	FALSE	rIZ4yc	0.001549	0.0054	body fat pe	TRUE	reported	textfile	0.003041	alcohol inta	TRUE	9.86E-09	inferred	JSsnZE	textfile	2	TRUE	NA
rs1103942	T	C	T	C	-0.02356	-0.01928	0.454624	0.454643	FALSE	FALSE	FALSE	rIZ4yc	0.001547	1.30E-35	body fat pe	TRUE	reported	textfile	0.003037	alcohol inta	TRUE	8.73E-15	inferred	JSsnZE	textfile	2	TRUE	NA
rs1122361	A	G	A	G	0.025091	0.005776	0.206155	0.206211	FALSE	FALSE	FALSE	rIZ4yc	0.001912	0.0025	body fat pe	TRUE	reported	textfile	0.003754	alcohol inta	TRUE	2.32E-11	inferred	JSsnZE	textfile	2	TRUE	NA
rs1170085	G	A	G	A	-0.0298	-0.0027	0.093465	0.093488	FALSE	FALSE	FALSE	rIZ4yc	0.002671	0.31	body fat pe	TRUE	reported	textfile	0.005233	alcohol inta	TRUE	1.24E-08	inferred	JSsnZE	textfile	2	TRUE	NA
rs1175077	A	G	A	G	-0.02049	-0.00321	0.209454	0.209499	FALSE	FALSE	FALSE	rIZ4yc	0.001896	0.089999	body fat pe	TRUE	reported	textfile	0.003726	alcohol inta	TRUE	3.80E-08	inferred	JSsnZE	textfile	2	TRUE	NA
rs1177994	C	G	C	G	-0.01967	-0.00577	0.336989	0.337032	FALSE	TRUE	FALSE	rIZ4yc	0.001692	0.00065	body fat pe	TRUE	reported	textfile	0.00332	alcohol inta	TRUE	3.12E-09	inferred	JSsnZE	textfile	2	TRUE	NA
rs1178721	T	C	T	C	0.024416	0.009891	0.369127	0.369084	FALSE	FALSE	FALSE	rIZ4yc	0.00163	1.30E-09	body fat pe	TRUE	reported	textfile	0.003201	alcohol inta	TRUE	2.38E-14	inferred	JSsnZE	textfile	2	TRUE	NA
rs1194069	G	A	G	A	-0.04371	0.006045	0.604193	0.604237	FALSE	FALSE	FALSE	rIZ4yc	0.001585	0.00014	body fat pe	TRUE	reported	textfile	0.003116	alcohol inta	TRUE	1.04E-44	inferred	JSsnZE	textfile	2	TRUE	NA
rs1228589	A	G	A	G	0.02107	0.011753	0.246133	0.246022	FALSE	FALSE	FALSE	rIZ4yc	0.001795	5.80E-11	body fat pe	TRUE	reported	textfile	0.003528	alcohol inta	TRUE	2.34E-09	inferred	JSsnZE	textfile	2	TRUE	NA
rs1229984	C	T	C	T	-0.26171	0.031006	0.97277	0.972781	FALSE	FALSE	FALSE	rIZ4yc	0.004675	3.30E-11	body fat pe	TRUE	reported	textfile	0.009185	alcohol inta	TRUE	1.43E-178	inferred	JSsnZE	textfile	2	TRUE	NA
rs1310297	C	T	C	T	-0.01941	-0.00105	0.61881	0.618789	FALSE	FALSE	FALSE	rIZ4yc	0.001587	0.51	body fat pe	TRUE	reported	textfile	0.003119	alcohol inta	TRUE	4.89E-10	inferred	JSsnZE	textfile	2	TRUE	NA
rs1317844	T	C	T	C	-0.01865	-0.00665	0.276349	0.276461	FALSE	FALSE	FALSE	rIZ4yc	0.001725	0.00012	body fat pe	TRUE	reported	textfile	0.00339	alcohol inta	TRUE	3.75E-08	inferred	JSsnZE	textfile	2	TRUE	NA
rs1339001	C	T	C	T	0.029612	0.004653	0.134041	0.133958	FALSE	FALSE	FALSE	rIZ4yc	0.002283	0.042	body fat pe	TRUE	reported	textfile	0.004492	alcohol inta	TRUE	4.33E-11	inferred	JSsnZE	textfile	2	TRUE	NA
rs1421085	C	T	C	T	0.019939	0.040951	0.403447	0.403467	FALSE	FALSE	FALSE	rIZ4yc	0.001572	1.20E-149	body fat pe	TRUE	reported	textfile	0.003085	alcohol inta	TRUE	1.02E-10	inferred	JSsnZE	textfile	2	TRUE	NA
rs1515590	T	C	T	C	0.018245	-3.24E-06	0.383245	0.383221	FALSE	FALSE	FALSE	rIZ4yc	0.001585	1	body fat pe	TRUE	reported	textfile	0.003116	alcohol inta	TRUE	4.78E-09	inferred	JSsnZE	textfile	2	TRUE	NA
rs1666658	C	T	C	T	0.017967	0.000545	0.392206	0.392247	FALSE	FALSE	FALSE	rIZ4yc	0.001578	0.73	body fat pe	TRUE	reported	textfile	0.003099	alcohol inta	TRUE	6.69E-09	inferred	JSsnZE	textfile	2	TRUE	NA
rs1766275	C	T	C	T	0.030135	0.006488	0.089115	0.089054	FALSE	FALSE	FALSE	rIZ4yc	0.002775	0.019	body fat pe	TRUE	reported	textfile	0.00546	alcohol inta	TRUE	3.41E-08	inferred	JSsnZE	textfile	2	TRUE	NA
rs186347	T	G	T	G	0.017949	0.003363	0.463343	0.463271	FALSE	FALSE	FALSE	rIZ4yc	0.001555	0.031	body fat pe	TRUE	reported	textfile	0.003051	alcohol inta	TRUE	4.02E-09	inferred	JSsnZE	textfile	2	TRUE	NA
rs1937522	G	A	G	A	0.016898	-0.00241	0.528054	0.52793	FALSE	FALSE	FALSE	rIZ4yc	0.001545	0.12	body fat pe	TRUE	reported	textfile	0.003032	alcohol inta	TRUE	2.50E-08	inferred	JSsnZE	textfile	2	TRUE	NA
rs1991083	T	C	T	C	-0.02239	-0.00456	0.679886	0.679821	FALSE	FALSE	FALSE	rIZ4yc	0.001656	0.0059	body fat pe	TRUE	reported	textfile	0.003258	alcohol inta	TRUE	6.30E-12	inferred	JSsnZE	textfile	2	TRUE	NA
rs2043677	T	C	T	C	0.026113	0.001878	0.145599	0.145566	FALSE	FALSE	FALSE	rIZ4yc	0.002206	0.39	body fat pe	TRUE	reported	textfile	0.004327	alcohol inta	TRUE	1.59E-09	inferred	JSsnZE	textfile	2	TRUE	NA
rs2159935	A	G	A	G	-0.01857	-0.00731	0.490369	0.490225	FALSE	FALSE	FALSE	rIZ4yc	0.001539	2.10E-06	body fat pe	TRUE	reported	textfile	0.003026	alcohol inta	TRUE	8.33E-10	inferred	JSsnZE	textfile	2	TRUE	NA
rs2160935	T	C	T	C	-0.01872	-0.00999	0.604293	0.604314	FALSE	FALSE	FALSE	rIZ4yc	0.001574	2.20E-10	body fat pe	TRUE	reported	textfile	0.003091	alcohol inta	TRUE	1.40E-09	inferred	JSsnZE	textfile	2	TRUE	NA
rs2244598	C	T	C	T	-0.01838	-0.00593	0.605114	0.605223	FALSE	FALSE	FALSE	rIZ4yc	0.001585	0.00018	body fat pe	TRUE	reported	textfile	0.003119	alcohol inta	TRUE	3.81E-09	inferred	JSsnZE	textfile	2	TRUE	NA
rs2411453	G	T	G	T	-0.03508	-0.02237	0.597353	0.597484	FALSE	FALSE	FALSE	rIZ4yc	0.001575	8.30E-46	body fat pe	TRUE	reported	textfile	0.00309	alcohol inta	TRUE	7.32E-30	inferred	JSsnZE	textfile	2	TRUE	NA
rs2535911	T	C	T	C	-0.01885	-0.00339	0.354749	0.354793	FALSE	FALSE	FALSE	rIZ4yc	0.001615	0.036	body fat pe	TRUE	reported	textfile	0.003168	alcohol inta	TRUE	2.71E-09	inferred	JSsnZE	textfile	2	TRUE	NA
rs2622167	A	G	A	G	-0.01912	0.005828	0.428653	0.428641	FALSE	FALSE	FALSE	rIZ4yc	0.001563	0.00019	body fat pe	TRUE	reported	textfile	0.003067	alcohol inta	TRUE	4.61E-10	inferred	JSsnZE	textfile	2	TRUE	NA
rs262240	T	C	T	C	-0.01721	0.001196	0.468553	0.468754	FALSE	FALSE	FALSE	rIZ4yc	0.001544	0.44	body fat pe	TRUE	reported	textfile	0.003035	alcohol inta	TRUE	1.43E-08	inferred	JSsnZE	textfile	2	TRUE	NA
rs2717063	A	C	A	C	-0.02037	0.00334	0.585731	0.585743	FALSE	FALSE	FALSE	rIZ4yc	0.001568	0.033	body fat pe	TRUE	reported	textfile	0.003085	alcohol inta	TRUE	4.00E-11	inferred	JSsnZE	textfile	2	TRUE	NA
rs2878710	A	G	A	G	0.017811	-0.00195	0.40423	0.404302	FALSE	FALSE	FALSE	rIZ4yc	0.001567	0.21	body fat pe	TRUE	reported	textfile	0.003085	alcohol inta	TRUE	7.74E-09	inferred	JSsnZE	textfile	2	TRUE	NA
rs3444085	T	C	T	C	-0.02268	-0.0037	0.157151	0.157161	FALSE	FALSE	FALSE	rIZ4yc	0.002114	0.08	body fat pe	TRUE	reported	textfile	0.004151	alcohol inta	TRUE	4.63E-08	inferred	JSsnZE	textfile	2	TRUE	NA
rs3447388	A	G	A	G	-0.02036	-0.00182	0.24819	0.248257	FALSE	FALSE	FALSE	rIZ4yc	0.001784	0.31	body fat pe	TRUE	reported	textfile	0.003503	alcohol inta	TRUE	6.18E-09	inferred	JSsnZE	textfile	2	TRUE	NA
rs3463102	T	C	T	C	-0.01691	0.003344	0.446061	0.44608	FALSE	FALSE	FALSE	rIZ4yc	0.001553	0.031	body fat pe	TRUE	reported	textfile	0.003048	alcohol inta	TRUE	2.89E-08	inferred	JSsnZE	textfile	2	TRUE	NA
rs3510514	T	C	T	C	0.026345	0.009837	0.401541	0.401394	FALSE	FALSE	FALSE	rIZ4yc	0.001573	4.00E-10	body fat pe	TRUE	reported	textfile	0.003088	alcohol inta	TRUE	1.44E-17	inferred	JSsnZE	textfile	2	TRUE	NA
rs362307	T	C	T	C	0.043305	0.014838	0.074582	0.074564	FALSE	FALSE	FALSE	rIZ4yc	0.002952	5.00E-07	body fat pe	TRUE	reported	textfile	0.005802	alcohol inta	TRUE	8.42E-14	inferred	JSsnZE	textfile	2	TRUE	NA
rs4241258	T	C	T	C	0.025064	0.002226	0.13763	0.137529	FALSE	FALSE	FALSE	rIZ4yc	0.002238	0.32	body fat pe	TRUE	reported	textfile	0.004403	alcohol inta	TRUE	1.26E-08	inferred	JSsnZE	textfile	2	TRUE	NA
rs4242715	A	G	A	G	-0.01865	-0.00965	0.680585	0.680409	FALSE	FALSE	FALSE	rIZ4yc	0.001654	5.30E-09	body fat pe	TRUE	reported	textfile	0.003248	alcohol inta	TRUE	9.31E-09	inferred	JSsnZE	textfile	2	TRUE	NA
rs4417025	A	G	A	G	-0.01884	-0.00098	0.361153	0.361215	FALSE	FALSE	FALSE	rIZ4yc	0.001609	0.54	body fat pe	TRUE	reported	textfile	0.003165	alcohol inta	TRUE	2.65E-09	inferred	JSsnZE	textfile	2	TRUE	NA
rs4503294	T	C	T	C	0.018148	0.002309	0.565333	0.565221	FALSE	FALSE	FALSE	rIZ4yc	0.00156	0.14	body fat pe	TRUE	reported	textfile	0.00307	alcohol inta	TRUE	3.41E-09	inferred	JSsnZE	textfile	2	TRUE	NA
rs461599	C	A	C	A	-0.01919	-0.00347	0.462259	0.46219	FALSE	FALSE	FALSE	rIZ4yc	0.001547	0.025	body fat pe	TRUE	reported	textfile	0.00304	alcohol inta	TRUE	2.74E-10	inferred	JSsnZE	textfile	2	TRUE	NA
rs4726481	T	G	T	G	0.021761	2.80E-05	0.400576	0.40063	FALSE	FALSE	FALSE	rIZ4yc	0.00158	0.99	body fat pe	TRUE	reported	textfile	0.003102	alcohol inta	TRUE	2.29E-12	inferred	JSsnZE	textfile	2	TRUE	NA
rs4800487	G	A	G	A	-0.02894	-0.01627	0.456873	0.457026	FALSE	FALSE	FALSE	rIZ4yc	0.001554	1.20E-25	body fat pe	TRUE	reported	textfile	0.003047	alcohol inta	TRUE	2.16E-21	inferred	JSsnZE	textfile	2	TRUE	NA
rs489062	A	G	A	G	0.01665	0.010861	0.437454	0.437458	FALSE	FALSE	FALSE	rIZ4yc	0.001555	2.80E-12	body fat pe	TRUE	reported	textfile	0.003053	alcohol inta	TRUE	4.93E-08	inferred	JSsnZE	textfile	2	TRUE	NA
rs4916723	C	A	C	A	0.023948	0.001557	0.420617	0.420655	FALSE	FALSE	FALSE	rIZ4yc	0.001577	0.32	body fat pe	TRUE	reported	textfile	0.0031	alcohol inta	TRUE	1.11E-14	inferred	JSsnZE	textfile	2	TRUE	NA
rs4968391	T	G	T	G	-0.01927	0.000173	0.674892	0.67478	FALSE	FALSE	FALSE	rIZ4yc	0.001645	0.92	body fat pe	TRUE	reported	textfile	0.003227	alcohol inta	TRUE	2.34E-09	inferred	JSsnZE	textfile	2	TRUE	NA
rs5022348	T	C	T	C	0.020264	0.006449	0.40703	0.406941	FALSE	FALSE	FALSE	rIZ4yc	0.00182	0.0004	body fat pe	TRUE	reported	textfile	0.00357	alcohol inta	TRUE	1.38E-08	inferred	JSsnZE	textfile	2	TRUE	NA
rs550942	T	C	T	C	0.022401	0.003694	0.823865	0.823943	FALSE	FALSE	FALSE	rIZ4yc	0.002032	0.069	body fat pe	TRUE	reported	textfile	0.003989	alcohol inta	TRUE	1.96E-08	inferred	JSsnZE	textfile	2	TRUE	NA
rs5619443	T	C	T	C	0.02254	0.006997	0.16931	0.169299	FALSE	FALSE	FALSE	rIZ4yc	0.002072	0.00073	body fat pe	TRUE	reported	textfile	0.004071	alcohol inta	TRUE	3.09E-08	inferred	JSsnZE	textfile	2	TRUE	NA
rs5890541	A	G	A	G	-0.02663	-0.0047	0.410052	0.41017	FALSE	FALSE	FALSE	rIZ4yc	0.001567	0.0027	body fat pe	TRUE	reported	textfile	0.003078	alcohol inta	TRUE	5.07E-18	inferred	JSsnZE	textfile	2	TRUE	NA
rs6030312	T	G	T	G	-0.01953	-0.00054	0.314251	0.314252	FALSE	FALSE	FALSE	rIZ4yc	0.001668	0.75	body fat pe	TRUE	reported	textfile	0.003271	alcohol inta	TRUE	2.36E-09	inferred	JSsnZE	textfile	2	TRUE	NA
rs6187351	T	G	T	G	0.020374	0.010208	0.32785	0.327772	FALSE	FALSE	FALSE	rIZ4yc	0.001682	1.30E-09	body fat pe	TRUE	reported	textfile	0.003303	alcohol inta	TRUE	6.91E-10	inferred	JSsnZE	textfile	2	TRUE	NA
rs6230578	G	C	G	C	-0.04852	0.00526	0.102253	0.102212	FALSE	TRUE	FALSE	rIZ4yc	0.002579	0.041	body fat pe	TRUE	reported	textfile	0.005066	alcohol inta	TRUE	9.88E-22	inferred	JSsnZE	textfile	2	TRUE	NA
rs6233967	A	C	A	C	0.018294	0.004044	0.626705	0.626523	FALSE	FALSE	FALSE	rIZ4yc	0.001605	0.012	body fat pe	TRUE	reported	textfile	0.003154	alcohol inta	TRUE	6.62E-09	inferred	JSsnZE	textfile	2	TRUE	NA
rs6246631	T	C	T	C	-0.02549	0.001666	0.202827	0.202803	FALSE	FALSE	FALSE	rIZ4yc	0.001923	0.39	body fat pe	TRUE	reported	textfile	0.003774	alcohol inta	TRUE	1.44E-11	inferred	JSsnZE	textfile	2	TRUE	NA
rs650558	T	C	T	C	0.020736	0.009779	0.247918	0.247943	FALSE	FALSE	FALSE	rIZ4yc	0.001788	4.50E-08	body fat pe	TRUE	reported	textfile	0.003508	alcohol inta	TRUE	3.39E-09	inferred	JSsnZE	textfile	2	TRUE	NA
rs6727281	T	C	T	C	-0.02432	-0.00138	0.184023	0.183993	FALSE	FALSE	FALSE	rIZ4yc	0.001992	0.49	body fat pe	TRUE	reported	textfile	0.00392	alcohol inta	TRUE	5.46E-10	inferred	JSsnZE	textfile	2	TRUE	NA
rs6943160	C	T	C	T	0.020627	-0.00191	0.208646	0.208586	FALSE	FALSE	FALSE	rIZ4yc	0.001899	0.31	body fat pe	TRUE	reported	textfile	0.003728	alcohol inta	TRUE	3.14E-08	inferred	JSsnZE	textfile	2	TRUE	NA
rs7165168	T	C	T	C	-0.07046	-0.00202	0.0142	0.014192	FALSE	FALSE	FALSE	rIZ4yc	0.006528	0.760001	body fat pe	TRUE	reported	textfile	0.012791	alcohol inta	TRUE	3.62E-08	inferred	JSsnZE	textfile	2	TRUE	NA
rs7231987	T	G	T	G	0.019026	0.021874	0.267222	0.267157	FALSE	FALSE	FALSE	rIZ4yc	0.001747	6.00E-36	body fat pe	TRUE	reported	textfile	0.003427	alcohol inta	TRUE	2.82E-08	inferred	JSsnZE	textfile	2	TRUE	NA
rs7276922	T	A	T	A	-0.02314	-0.00798	0.154942	0.154975	FALSE	TRUE	FALSE	rIZ4yc	0.00213	0.00018	body fat pe	TRUE	reported	textfile	0.004192	alcohol inta	TRUE	3.40E-08	inferred	JSsnZE	textfile	2	TRUE	NA
rs7278706	A	G	A	G	-0.02819	0.004029	0.162767	0.162749	FALSE	FALSE	FALSE	rIZ4yc	0.00209	0.054	body fat pe	TRUE	reported	textfile	0.004103	alcohol inta	TRUE	6.36E-12	inferred	JSsnZE	textfile	2	TRUE	NA
rs728538	G	T	G	T	0.022875	0.004481	0.168868	0.168894	FALSE	FALSE	FALSE	rIZ4yc	0.00207	0.03	body fat pe	TRUE	reported	textfile	0.004063	alcohol inta	TRUE	1.79E-08	inferred	JSsnZE	textfile	2	TRUE	NA
rs7298932	G	A	G	A	-0.02372	-0.00857	0.147849	0.147842	FALSE	FALSE	FALSE	rIZ4yc	0.002195	9.40E-05	body fat pe	TRUE	reported	textfile	0.004312	alcohol inta	TRUE	3.76E-08	inferred	JSsnZE	textfile	2	TRUE	NA
rs7330939	T	C	T	C	-0.02133	0.000836	0.720352	0.720423	FALSE	FALSE	FALSE	rIZ4yc	0.001735	0.630001	body fat pe	TRUE	reported	textfile	0.003405	alcohol inta	TRUE	3.73E-10	inferred	JSsnZE	textfile	2	TRUE	NA
rs7460106	C	T	C	T	-0.02171	-0.00492	0.23562	0.235708	FALSE	FALSE	FALSE	rIZ4yc	0.001843	0.0076	body fat pe	TRUE	reported	textfile	0.003619	alcohol inta	TRUE	1.99E-09	inferred	JSsnZE	textfile	2	TRUE	NA
rs7467914	C	T	C	T	-0.03207	-0.00204	0.074515	0.074479	FALSE	FALSE	FALSE	rIZ4yc	0.002933	0.49	body fat pe	TRUE	reported	textfile	0.005758	alcohol inta	TRUE	2.54E-08	inferred	JSsnZE	textfile	2	TRUE	NA
rs7514579	C	A	C	A	0.019667	0.003628	0.232457	0.232429	FALSE	FALSE	FALSE	rIZ4yc	0.001828	0.047	body fat pe	TRUE	reported	textfile	0.003598	alcohol inta	TRUE	4.59E-08	inferred	JSsnZE	textfile	2	TRUE	NA
rs7608265	T	C	T	C	0.046427	0.01109	0.054327	0.054327	FALSE	FALSE	FALSE	rIZ4yc	0.003402	0.0011	body fat pe	TRUE	reported	textfile	0.006687	alcohol inta	TRUE	3.83E-12	inferred	JSsnZE	textfile	2	TRUE	NA
rs780094	C	T	C	T	-0.05099	-0.00167	0.615206	0.615183	FALSE	FALSE	FALSE	rIZ4yc	0.001578	0.29	body fat pe	TRUE	reported	textfile	0.003105	alcohol inta	TRUE	1.31E-60	inferred	JSsnZE	textfile	2	TRUE	NA
rs780569	A	T	A	T	0.019803	0.002454	0.70882	0.708812	FALSE	TRUE	FALSE	rIZ4yc	0.00171	0.15	body fat pe	TRUE	reported	textfile	0.003365	alcohol inta	TRUE	3.96E-09	inferred	JSsnZE	textfile	2	TRUE	NA
rs8029231	C	T	C	T	-0.03937	-0.00406	0.057704	0.057751	FALSE	FALSE	FALSE	rIZ4yc	0.003309	0.22	body fat pe	TRUE	reported	textfile	0.006496	alcohol inta	TRUE	1.35E-09	inferred	JSsnZE	textfile	2	TRUE	NA
rs8043563	C	G	C	G	0.023365	-0.00539	0.737192	0.737208	FALSE	TRUE	FALSE	rIZ4yc	0.001768	0.0023	body fat pe	TRUE	reported	textfile	0.003471	alcohol inta	TRUE	1.68E-11	inferred	JSsnZE	textfile	2	TRUE	NA
rs8083110	T	C	T	C	-0.01681	-0.0117	0.418707	0.418628	FALSE	FALSE	FALSE	rIZ4yc	0.001569	9.10E-14	body fat pe	TRUE	reported	textfile	0.003078	alcohol inta	TRUE	4.72E-08	inferred	JSsnZE	textfile	2	TRUE	NA
rs838145	A	G	A	G	0.021955	0.00344	0.542982	0.543022	FALSE	FALSE	FALSE	rIZ4yc	0.001558	0.027	body fat pe	TRUE	reported	textfile	0.003055	alcohol inta	TRUE	6.70E-13	inferred	JSsnZE	textfile	2	TRUE	NA
rs8614	A	C	A	C	0.024781	-0.00037	0.182509	0.182443	FALSE	FALSE	FALSE	rIZ4yc	0.002001	0.85	body fat pe	TRUE	reported	textfile	0.003925	alcohol inta	TRUE	2.74E-10	inferred	JSsnZE	textfile	2	TRUE	NA
rs9403297	A	G	A	G	0.018823	0.006982	0.372967	0.372955	FALSE	FALSE	FALSE	rIZ4yc	0.001593	1.20E-05	body fat pe	TRUE	reported	textfile	0.00313	alcohol inta	TRUE	1.82E-09	inferred	JSsnZE	textfile	2	TRUE	NA
rs9648478	A	G	A	G	0.01686	0.009408	0.510245	0.510278	FALSE	FALSE	FALSE	rIZ4yc	0.001543	1.10E-09	body fat pe	TRUE	reported	textfile	0.003029	alcohol inta	TRUE	2.60E-08	inferred	JSsnZE	textfile	2	TRUE	NA
rs9829192	T	G	T	G	0.016932	0.002267	0.435133	0.435145	FALSE	FALSE	FALSE	rIZ4yc	0.001552	0.14	body fat pe	TRUE	reported	textfile	0.00305	alcohol inta	TRUE	2.84E-08	inferred	JSsnZE	textfile	2	TRUE	NA
rs9891943	C	T	C	T	0.019867	0.00048	0.232109	0.23202	FALSE	FALSE	FALSE	rIZ4yc	0.001856	0.8	body fat pe	TRUE	reported	textfile	0.003641	alcohol inta	TRUE	4.88E-08	inferred	JSsnZE	textfile	2	TRUE	NA
rs9906502	A	G	A	G	0.023788	0.00052	0.176998	0.17701	FALSE	FALSE	FALSE	rIZ4yc	0.002019	0.8	body fat pe	TRUE	reported	textfile	0.003962	alcohol inta	TRUE	1.92E-09	inferred	JSsnZE	textfile	2	TRUE	NA

SNPs of alcohol intake frequency on BMI

SNP	effect_allel	other_allel	effect_allel	other_allel	beta.expos	beta.outco	eaf.exposu	eaf.outcom	remove	palindromi	ambiguous	id.outcome	se.outcome	pval.outco	outcome	mr_keep.o	pval_origin	data_sourc	se.exposure	exposure	mr_keep.e	pval.expos	pval_origin	id.exposure	data_sourc	action	mr_keep	samplesize.outcome
rs1018831	T	C	T	C	-0.01979	-0.00907	0.470852	0.470831	FALSE	FALSE	FALSE	ww9T9Q	0.001977	4.50E-06	BMI	TRUE	reported	textfile	0.003036	alcohol int	TRUE	7.16E-11	inferred	IS8jAg	textfile	2	TRUE	NA
rs1079266	G	A	G	A	0.017432	0.007056	0.505254	0.505221	FALSE	FALSE	FALSE	ww9T9Q	0.001986	0.00038	BMI	TRUE	reported	textfile	0.003041	alcohol int	TRUE	9.86E-09	inferred	IS8jAg	textfile	2	TRUE	NA
rs1103942	T	C	T	C	-0.02356	-0.02159	0.454624	0.454633	FALSE	FALSE	FALSE	ww9T9Q	0.001984	1.40E-27	BMI	TRUE	reported	textfile	0.003037	alcohol int	TRUE	8.73E-15	inferred	IS8jAg	textfile	2	TRUE	NA
rs1122361	A	G	A	G	0.025091	0.009035	0.206155	0.206164	FALSE	FALSE	FALSE	ww9T9Q	0.002452	0.00023	BMI	TRUE	reported	textfile	0.003754	alcohol int	TRUE	2.32E-11	inferred	IS8jAg	textfile	2	TRUE	NA
rs1170085	G	A	G	A	-0.0298	-0.00493	0.093465	0.093482	FALSE	FALSE	FALSE	ww9T9Q	0.003426	0.15	BMI	TRUE	reported	textfile	0.005233	alcohol int	TRUE	1.24E-08	inferred	IS8jAg	textfile	2	TRUE	NA
rs1175077	A	G	A	G	-0.02049	-0.00475	0.209454	0.209504	FALSE	FALSE	FALSE	ww9T9Q	0.002431	0.051	BMI	TRUE	reported	textfile	0.003726	alcohol int	TRUE	3.80E-08	inferred	IS8jAg	textfile	2	TRUE	NA
rs1177994	C	G	C	G	-0.01967	-0.00826	0.336989	0.33695	FALSE	TRUE	FALSE	ww9T9Q	0.002171	0.00014	BMI	TRUE	reported	textfile	0.00332	alcohol int	TRUE	3.12E-09	inferred	IS8jAg	textfile	2	TRUE	NA
rs1178721	T	C	T	C	0.024416	0.013248	0.369127	0.369095	FALSE	FALSE	FALSE	ww9T9Q	0.002091	2.40E-10	BMI	TRUE	reported	textfile	0.003201	alcohol int	TRUE	2.38E-14	inferred	IS8jAg	textfile	2	TRUE	NA
rs1194069	G	A	G	A	-0.04371	0.005098	0.604193	0.604181	FALSE	FALSE	FALSE	ww9T9Q	0.002032	0.012	BMI	TRUE	reported	textfile	0.003116	alcohol int	TRUE	1.04E-44	inferred	IS8jAg	textfile	2	TRUE	NA
rs1228589	A	G	A	G	0.02107	0.020718	0.246133	0.246107	FALSE	FALSE	FALSE	ww9T9Q	0.0023	2.10E-19	BMI	TRUE	reported	textfile	0.003528	alcohol int	TRUE	2.34E-09	inferred	IS8jAg	textfile	2	TRUE	NA
rs1229984	C	T	C	T	-0.26171	0.037357	0.97277	0.972775	FALSE	FALSE	FALSE	ww9T9Q	0.005994	4.60E-10	BMI	TRUE	reported	textfile	0.009185	alcohol int	TRUE	1.43E-178	inferred	IS8jAg	textfile	2	TRUE	NA
rs1310297	C	T	C	T	-0.01941	-0.00144	0.61881	0.618847	FALSE	FALSE	FALSE	ww9T9Q	0.002035	0.48	BMI	TRUE	reported	textfile	0.003119	alcohol int	TRUE	4.89E-10	inferred	IS8jAg	textfile	2	TRUE	NA
rs1317844	T	C	T	C	-0.01865	-0.00695	0.276349	0.276301	FALSE	FALSE	FALSE	ww9T9Q	0.002212	0.0017	BMI	TRUE	reported	textfile	0.00339	alcohol int	TRUE	3.75E-08	inferred	IS8jAg	textfile	2	TRUE	NA
rs1339001	C	T	C	T	0.029612	0.014369	0.134041	0.134021	FALSE	FALSE	FALSE	ww9T9Q	0.002925	9.00E-07	BMI	TRUE	reported	textfile	0.004492	alcohol int	TRUE	4.33E-11	inferred	IS8jAg	textfile	2	TRUE	NA
rs1421085	C	T	C	T	0.019939	0.073461	0.403447	0.403381	FALSE	FALSE	FALSE	ww9T9Q	0.002015	1.00E-200	BMI	TRUE	reported	textfile	0.003085	alcohol int	TRUE	1.02E-10	inferred	IS8jAg	textfile	2	TRUE	NA
rs1515590	T	C	T	C	0.018245	0.003497	0.383245	0.383228	FALSE	FALSE	FALSE	ww9T9Q	0.002032	0.085	BMI	TRUE	reported	textfile	0.003116	alcohol int	TRUE	4.78E-09	inferred	IS8jAg	textfile	2	TRUE	NA
rs1666658	C	T	C	T	0.017967	0.006386	0.392206	0.392276	FALSE	FALSE	FALSE	ww9T9Q	0.002024	0.0016	BMI	TRUE	reported	textfile	0.003099	alcohol int	TRUE	6.69E-09	inferred	IS8jAg	textfile	2	TRUE	NA
rs1766275	C	T	C	T	0.030135	0.005722	0.089115	0.089065	FALSE	FALSE	FALSE	ww9T9Q	0.003557	0.11	BMI	TRUE	reported	textfile	0.00546	alcohol int	TRUE	3.41E-08	inferred	IS8jAg	textfile	2	TRUE	NA
rs186347	T	G	T	G	0.017949	0.003044	0.463343	0.463328	FALSE	FALSE	FALSE	ww9T9Q	0.001994	0.13	BMI	TRUE	reported	textfile	0.003051	alcohol int	TRUE	4.02E-09	inferred	IS8jAg	textfile	2	TRUE	NA
rs1937522	G	A	G	A	0.016898	-0.0026	0.528054	0.527974	FALSE	FALSE	FALSE	ww9T9Q	0.001982	0.19	BMI	TRUE	reported	textfile	0.003032	alcohol int	TRUE	2.50E-08	inferred	IS8jAg	textfile	2	TRUE	NA
rs1991083	T	C	T	C	-0.02239	-0.00152	0.679886	0.679897	FALSE	FALSE	FALSE	ww9T9Q	0.002122	0.47	BMI	TRUE	reported	textfile	0.003258	alcohol int	TRUE	6.30E-12	inferred	IS8jAg	textfile	2	TRUE	NA
rs2043677	T	C	T	C	0.026113	0.001543	0.145599	0.145595	FALSE	FALSE	FALSE	ww9T9Q	0.002829	0.59	BMI	TRUE	reported	textfile	0.004327	alcohol int	TRUE	1.59E-09	inferred	IS8jAg	textfile	2	TRUE	NA
rs2159935	A	G	A	G	-0.01857	-0.01337	0.490369	0.490374	FALSE	FALSE	FALSE	ww9T9Q	0.001973	1.20E-11	BMI	TRUE	reported	textfile	0.003026	alcohol int	TRUE	8.33E-10	inferred	IS8jAg	textfile	2	TRUE	NA
rs2160935	T	C	T	C	-0.01872	-0.01313	0.604293	0.604323	FALSE	FALSE	FALSE	ww9T9Q	0.002019	7.90E-11	BMI	TRUE	reported	textfile	0.003091	alcohol int	TRUE	1.40E-09	inferred	IS8jAg	textfile	2	TRUE	NA
rs2244598	C	T	C	T	-0.01838	-0.00887	0.605114	0.605133	FALSE	FALSE	FALSE	ww9T9Q	0.002032	1.30E-05	BMI	TRUE	reported	textfile	0.003119	alcohol int	TRUE	3.81E-09	inferred	IS8jAg	textfile	2	TRUE	NA
rs2411453	G	T	G	T	-0.03508	-0.0266	0.597353	0.597382	FALSE	FALSE	FALSE	ww9T9Q	0.002018	1.10E-39	BMI	TRUE	reported	textfile	0.00309	alcohol int	TRUE	7.32E-30	inferred	IS8jAg	textfile	2	TRUE	NA
rs2535911	T	C	T	C	-0.01885	-0.00986	0.354749	0.354763	FALSE	FALSE	FALSE	ww9T9Q	0.002071	1.90E-06	BMI	TRUE	reported	textfile	0.003168	alcohol int	TRUE	2.71E-09	inferred	IS8jAg	textfile	2	TRUE	NA
rs2622167	A	G	A	G	-0.01912	0.007085	0.428653	0.428777	FALSE	FALSE	FALSE	ww9T9Q	0.002003	0.00041	BMI	TRUE	reported	textfile	0.003067	alcohol int	TRUE	4.61E-10	inferred	IS8jAg	textfile	2	TRUE	NA
rs262240	T	C	T	C	-0.01721	0.001162	0.468553	0.468588	FALSE	FALSE	FALSE	ww9T9Q	0.001979	0.56	BMI	TRUE	reported	textfile	0.003035	alcohol int	TRUE	1.43E-08	inferred	IS8jAg	textfile	2	TRUE	NA
rs2717063	A	C	A	C	-0.02037	-0.00501	0.585731	0.585774	FALSE	FALSE	FALSE	ww9T9Q	0.002009	0.013	BMI	TRUE	reported	textfile	0.003085	alcohol int	TRUE	4.00E-11	inferred	IS8jAg	textfile	2	TRUE	NA
rs2878710	A	G	A	G	0.017811	-0.00416	0.40423	0.404232	FALSE	FALSE	FALSE	ww9T9Q	0.002009	0.038	BMI	TRUE	reported	textfile	0.003085	alcohol int	TRUE	7.74E-09	inferred	IS8jAg	textfile	2	TRUE	NA
rs3444085	T	C	T	C	-0.02268	-0.00446	0.157151	0.157121	FALSE	FALSE	FALSE	ww9T9Q	0.002712	0.1	BMI	TRUE	reported	textfile	0.004151	alcohol int	TRUE	4.63E-08	inferred	IS8jAg	textfile	2	TRUE	NA
rs3447388	A	G	A	G	-0.02036	-0.00073	0.24819	0.248126	FALSE	FALSE	FALSE	ww9T9Q	0.002288	0.75	BMI	TRUE	reported	textfile	0.003503	alcohol int	TRUE	6.18E-09	inferred	IS8jAg	textfile	2	TRUE	NA
rs3463102	T	C	T	C	-0.01691	0.002553	0.446061	0.446057	FALSE	FALSE	FALSE	ww9T9Q	0.001991	0.2	BMI	TRUE	reported	textfile	0.003048	alcohol int	TRUE	2.89E-08	inferred	IS8jAg	textfile	2	TRUE	NA
rs3510514	T	C	T	C	0.026345	0.022598	0.401541	0.401549	FALSE	FALSE	FALSE	ww9T9Q	0.002016	3.80E-29	BMI	TRUE	reported	textfile	0.003088	alcohol int	TRUE	1.44E-17	inferred	IS8jAg	textfile	2	TRUE	NA
rs362307	T	C	T	C	0.043305	0.024709	0.074582	0.074588	FALSE	FALSE	FALSE	ww9T9Q	0.003783	6.50E-11	BMI	TRUE	reported	textfile	0.005802	alcohol int	TRUE	8.42E-14	inferred	IS8jAg	textfile	2	TRUE	NA
rs4241258	T	C	T	C	0.025064	-0.0004	0.13763	0.137617	FALSE	FALSE	FALSE	ww9T9Q	0.002867	0.89	BMI	TRUE	reported	textfile	0.004403	alcohol int	TRUE	1.26E-08	inferred	IS8jAg	textfile	2	TRUE	NA
rs4242715	A	G	A	G	-0.01865	-0.01117	0.680585	0.680581	FALSE	FALSE	FALSE	ww9T9Q	0.002121	1.40E-07	BMI	TRUE	reported	textfile	0.003248	alcohol int	TRUE	9.31E-09	inferred	IS8jAg	textfile	2	TRUE	NA
rs4417025	A	G	A	G	-0.01884	-0.0013	0.361153	0.361164	FALSE	FALSE	FALSE	ww9T9Q	0.002062	0.53	BMI	TRUE	reported	textfile	0.003165	alcohol int	TRUE	2.65E-09	inferred	IS8jAg	textfile	2	TRUE	NA
rs4503294	T	C	T	C	0.018148	0.002246	0.565333	0.565305	FALSE	FALSE	FALSE	ww9T9Q	0.002	0.26	BMI	TRUE	reported	textfile	0.00307	alcohol int	TRUE	3.41E-09	inferred	IS8jAg	textfile	2	TRUE	NA
rs461599	C	A	C	A	-0.01919	-0.00491	0.462259	0.462243	FALSE	FALSE	FALSE	ww9T9Q	0.001984	0.013	BMI	TRUE	reported	textfile	0.00304	alcohol int	TRUE	2.74E-10	inferred	IS8jAg	textfile	2	TRUE	NA
rs4726481	T	G	T	G	0.021761	0.001441	0.400576	0.400583	FALSE	FALSE	FALSE	ww9T9Q	0.002026	0.48	BMI	TRUE	reported	textfile	0.003102	alcohol int	TRUE	2.29E-12	inferred	IS8jAg	textfile	2	TRUE	NA
rs4800487	G	A	G	A	-0.02894	-0.01935	0.456873	0.45693	FALSE	FALSE	FALSE	ww9T9Q	0.001992	2.60E-22	BMI	TRUE	reported	textfile	0.003047	alcohol int	TRUE	2.16E-21	inferred	IS8jAg	textfile	2	TRUE	NA
rs489062	A	G	A	G	0.01665	0.016502	0.437454	0.437431	FALSE	FALSE	FALSE	ww9T9Q	0.001994	1.30E-16	BMI	TRUE	reported	textfile	0.003053	alcohol int	TRUE	4.93E-08	inferred	IS8jAg	textfile	2	TRUE	NA
rs4916723	C	A	C	A	0.023948	0.004458	0.420617	0.420647	FALSE	FALSE	FALSE	ww9T9Q	0.002023	0.028	BMI	TRUE	reported	textfile	0.0031	alcohol int	TRUE	1.11E-14	inferred	IS8jAg	textfile	2	TRUE	NA
rs4968391	T	G	T	G	-0.01927	-0.0017	0.674892	0.674864	FALSE	FALSE	FALSE	ww9T9Q	0.00211	0.42	BMI	TRUE	reported	textfile	0.003227	alcohol int	TRUE	2.34E-09	inferred	IS8jAg	textfile	2	TRUE	NA
rs5022348	T	C	T	C	0.020264	0.008942	0.40703	0.406981	FALSE	FALSE	FALSE	ww9T9Q	0.002334	0.00013	BMI	TRUE	reported	textfile	0.00357	alcohol int	TRUE	1.38E-08	inferred	IS8jAg	textfile	2	TRUE	NA
rs550942	T	C	T	C	0.022401	0.004612	0.823865	0.823884	FALSE	FALSE	FALSE	ww9T9Q	0.002605	0.077	BMI	TRUE	reported	textfile	0.003989	alcohol int	TRUE	1.96E-08	inferred	IS8jAg	textfile	2	TRUE	NA
rs5619443	T	C	T	C	0.02254	0.006153	0.16931	0.169304	FALSE	FALSE	FALSE	ww9T9Q	0.002657	0.021	BMI	TRUE	reported	textfile	0.004071	alcohol int	TRUE	3.09E-08	inferred	IS8jAg	textfile	2	TRUE	NA
rs5890541	A	G	A	G	-0.02663	-0.01357	0.410052	0.410069	FALSE	FALSE	FALSE	ww9T9Q	0.002011	1.50E-11	BMI	TRUE	reported	textfile	0.003078	alcohol int	TRUE	5.07E-18	inferred	IS8jAg	textfile	2	TRUE	NA
rs6030312	T	G	T	G	-0.01953	-6.59E-05	0.314251	0.314231	FALSE	FALSE	FALSE	ww9T9Q	0.00214	0.98	BMI	TRUE	reported	textfile	0.003271	alcohol int	TRUE	2.36E-09	inferred	IS8jAg	textfile	2	TRUE	NA
rs6187351	T	G	T	G	0.020374	0.015949	0.32785	0.327833	FALSE	FALSE	FALSE	ww9T9Q	0.002157	1.40E-13	BMI	TRUE	reported	textfile	0.003303	alcohol int	TRUE	6.91E-10	inferred	IS8jAg	textfile	2	TRUE	NA
rs6230578	G	C	G	C	-0.04852	0.013098	0.102253	0.102277	FALSE	TRUE	FALSE	ww9T9Q	0.003306	7.40E-05	BMI	TRUE	reported	textfile	0.005066	alcohol int	TRUE	9.88E-22	inferred	IS8jAg	textfile	2	TRUE	NA
rs6233967	A	C	A	C	0.018294	0.003795	0.626705	0.626701	FALSE	FALSE	FALSE	ww9T9Q	0.002058	0.064999	BMI	TRUE	reported	textfile	0.003154	alcohol int	TRUE	6.62E-09	inferred	IS8jAg	textfile	2	TRUE	NA
rs6246631	T	C	T	C	-0.02549	0.009243	0.202827	0.202809	FALSE	FALSE	FALSE	ww9T9Q	0.002465	0.00018	BMI	TRUE	reported	textfile	0.003774	alcohol int	TRUE	1.44E-11	inferred	IS8jAg	textfile	2	TRUE	NA
rs650558	T	C	T	C	0.020736	0.011338	0.247918	0.247896	FALSE	FALSE	FALSE	ww9T9Q	0.002294	7.70E-07	BMI	TRUE	reported	textfile	0.003508	alcohol int	TRUE	3.39E-09	inferred	IS8jAg	textfile	2	TRUE	NA
rs6727281	T	C	T	C	-0.02432	-0.00157	0.184023	0.18398	FALSE	FALSE	FALSE	ww9T9Q	0.002553	0.54	BMI	TRUE	reported	textfile	0.00392	alcohol int	TRUE	5.46E-10	inferred	IS8jAg	textfile	2	TRUE	NA
rs6943160	C	T	C	T	0.020627	0.003761	0.208646	0.20864	FALSE	FALSE	FALSE	ww9T9Q	0.002435	0.12	BMI	TRUE	reported	textfile	0.003728	alcohol int	TRUE	3.14E-08	inferred	IS8jAg	textfile	2	TRUE	NA
rs7165168	T	C	T	C	-0.07046	-0.0026	0.0142	0.014196	FALSE	FALSE	FALSE	ww9T9Q	0.008374	0.760001	BMI	TRUE	reported	textfile	0.012791	alcohol int	TRUE	3.62E-08	inferred	IS8jAg	textfile	2	TRUE	NA
rs7231987	T	G	T	G	0.019026	0.046517	0.267222	0.26722	FALSE	FALSE	FALSE	ww9T9Q	0.002241	9.60E-96	BMI	TRUE	reported	textfile	0.003427	alcohol int	TRUE	2.82E-08	inferred	IS8jAg	textfile	2	TRUE	NA
rs7276922	T	A	T	A	-0.02314	-0.01364	0.154942	0.154914	FALSE	TRUE	FALSE	ww9T9Q	0.00273	5.90E-07	BMI	TRUE	reported	textfile	0.004192	alcohol int	TRUE	3.40E-08	inferred	IS8jAg	textfile	2	TRUE	NA
rs7278706	A	G	A	G	-0.02819	0.007469	0.162767	0.162734	FALSE	FALSE	FALSE	ww9T9Q	0.00268	0.0053	BMI	TRUE	reported	textfile	0.004103	alcohol int	TRUE	6.36E-12	inferred	IS8jAg	textfile	2	TRUE	NA
rs728538	G	T	G	T	0.022875	0.004598	0.168868	0.168857	FALSE	FALSE	FALSE	ww9T9Q	0.002653	0.083	BMI	TRUE	reported	textfile	0.004063	alcohol int	TRUE	1.79E-08	inferred	IS8jAg	textfile	2	TRUE	NA
rs7298932	G	A	G	A	-0.02372	-0.01143	0.147849	0.147838	FALSE	FALSE	FALSE	ww9T9Q	0.002816	4.90E-05	BMI	TRUE	reported	textfile	0.004312	alcohol int	TRUE	3.76E-08	inferred	IS8jAg	textfile	2	TRUE	NA
rs7330939	T	C	T	C	-0.02133	-0.00199	0.720352	0.720386	FALSE	FALSE	FALSE	ww9T9Q	0.002225	0.37	BMI	TRUE	reported	textfile	0.003405	alcohol int	TRUE	3.73E-10	inferred	IS8jAg	textfile	2	TRUE	NA
rs7460106	C	T	C	T	-0.02171	-0.00664	0.23562	0.235649	FALSE	FALSE	FALSE	ww9T9Q	0.002364	0.005	BMI	TRUE	reported	textfile	0.003619	alcohol int	TRUE	1.99E-09	inferred	IS8jAg	textfile	2	TRUE	NA
rs7467914	C	T	C	T	-0.03207	0.001757	0.074515	0.074498	FALSE	FALSE	FALSE	ww9T9Q	0.003761	0.64	BMI	TRUE	reported	textfile	0.005758	alcohol int	TRUE	2.54E-08	inferred	IS8jAg	textfile	2	TRUE	NA
rs7514579	C	A	C	A	0.019667	0.002387	0.232457	0.232439	FALSE	FALSE	FALSE	ww9T9Q	0.002343	0.31	BMI	TRUE	reported	textfile	0.003598	alcohol int	TRUE	4.59E-08	inferred	IS8jAg	textfile	2	TRUE	NA
rs7608265	T	C	T	C	0.046427	0.013596	0.054327	0.054336	FALSE	FALSE	FALSE	ww9T9Q	0.00436	0.0018	BMI	TRUE	reported	textfile	0.006687	alcohol int	TRUE	3.83E-12	inferred	IS8jAg	textfile	2	TRUE	NA
rs780094	C	T	C	T	-0.05099	0.008014	0.615206	0.61521	FALSE	FALSE	FALSE	ww9T9Q	0.002022	7.40E-05	BMI	TRUE	reported	textfile	0.003105	alcohol int	TRUE	1.31E-60	inferred	IS8jAg	textfile	2	TRUE	NA
rs780569	A	T	A	T	0.019803	0.000233	0.70882	0.708775	FALSE	TRUE	FALSE	ww9T9Q	0.002191	0.92	BMI	TRUE	reported	textfile	0.003365	alcohol int	TRUE	3.96E-09	inferred	IS8jAg	textfile	2	TRUE	NA
rs8029231	C	T	C	T	-0.03937	-0.01218	0.057704	0.057712	FALSE	FALSE	FALSE	ww9T9Q	0.004247	0.0041	BMI	TRUE	reported	textfile	0.006496	alcohol int	TRUE	1.35E-09	inferred	IS8jAg	textfile	2	TRUE	NA
rs8043563	C	G	C	G	0.023365	-0.01079	0.737192	0.737193	FALSE	TRUE	FALSE	ww9T9Q	0.002267	1.90E-06	BMI	TRUE	reported	textfile	0.003471	alcohol int	TRUE	1.68E-11	inferred	IS8jAg	textfile	2	TRUE	NA
rs8083110	T	C	T	C	-0.01681	-0.01231	0.418707	0.418703	FALSE	FALSE	FALSE	ww9T9Q	0.002012	9.50E-10	BMI	TRUE	reported	textfile	0.003078	alcohol int	TRUE	4.72E-08	inferred	IS8jAg	textfile	2	TRUE	NA
rs838145	A	G	A	G	0.021955	0.003687	0.542982	0.54302	FALSE	FALSE	FALSE	ww9T9Q	0.001998	0.064999	BMI	TRUE	reported	textfile	0.003055	alcohol int	TRUE	6.70E-13	inferred	IS8jAg	textfile	2	TRUE	NA
rs8614	A	C	A	C	0.024781	0.006348	0.182509	0.182488	FALSE	FALSE	FALSE	ww9T9Q	0.002567	0.013	BMI	TRUE	reported	textfile	0.003925	alcohol int	TRUE	2.74E-10	inferred	IS8jAg	textfile	2	TRUE	NA
rs9403297	A	G	A	G	0.018823	0.011382	0.372967	0.372916	FALSE	FALSE	FALSE	ww9T9Q	0.002042	2.50E-08	BMI	TRUE	reported	textfile	0.00313	alcohol int	TRUE	1.82E-09	inferred	IS8jAg	textfile	2	TRUE	NA
rs9648478	A	G	A	G	0.01686	0.005897	0.510245	0.510246	FALSE	FALSE	FALSE	ww9T9Q	0.001978	0.0029	BMI	TRUE	reported	textfile	0.003029	alcohol int	TRUE	2.60E-08	inferred	IS8jAg	textfile	2	TRUE	NA
rs9829192	T	G	T	G	0.016932	-0.00911	0.435133	0.435154	FALSE	FALSE	FALSE	ww9T9Q	0.001989	4.70E-06	BMI	TRUE	reported	textfile	0.00305	alcohol int	TRUE	2.84E-08	inferred	IS8jAg	textfile	2	TRUE	NA
rs9891943	C	T	C	T	0.019867	0.002841	0.232109	0.232064	FALSE	FALSE	FALSE	ww9T9Q	0.002381	0.23	BMI	TRUE	reported	textfile	0.003641	alcohol int	TRUE	4.88E-08	inferred	IS8jAg	textfile	2	TRUE	NA
rs9906502	A	G	A	G	0.023788	0.001491	0.176998	0.176969	FALSE	FALSE	FALSE	ww9T9Q	0.002591	0.56	BMI	TRUE	reported	textfile	0.003962	alcohol int	TRUE	1.92E-09	inferred	IS8jAg	textfile	2	TRUE	NA

SNPs of alcohol intake frequency on CRP

SNP	effect_allel	other_allel	effect_allel	other_allel	beta.expos	beta.outco	eaf.exposu	eaf.outcom	remove	palindromi	ambiguous	id.outcome	se.outcom	pval.outco	outcome
rs1018831	T	C	T	C	-0.01979	-0.0049	0.470852	NA	FALSE	FALSE	FALSE	YO3EtP	0.0057	0.3961	CRP
rs1079266	G	A	G	A	0.017432	0.0071	0.505254	NA	FALSE	FALSE	FALSE	YO3EtP	0.0057	0.2123	CRP
rs1103942	T	C	T	C	-0.02356	-0.009	0.454624	NA	FALSE	FALSE	FALSE	YO3EtP	0.0058	0.1178	CRP
rs1122361	A	G	A	G	0.025091	-0.0073	0.206155	NA	FALSE	FALSE	FALSE	YO3EtP	0.007	0.2992	CRP
rs1170085	G	A	G	A	-0.0298	0.0043	0.093465	NA	FALSE	FALSE	FALSE	YO3EtP	0.0095	0.6498	CRP
rs1175077	A	G	A	G	-0.02049	-0.007	0.209454	NA	FALSE	FALSE	FALSE	YO3EtP	0.0071	0.3237	CRP
rs1178721	T	C	T	C	0.024416	0.0053	0.369127	NA	FALSE	FALSE	FALSE	YO3EtP	0.0059	0.3664	CRP
rs1194069	G	A	G	A	-0.04371	0.0082	0.604193	NA	FALSE	FALSE	FALSE	YO3EtP	0.0059	0.1624	CRP
rs1228589	A	G	A	G	0.02107	0.0021	0.246133	NA	FALSE	FALSE	FALSE	YO3EtP	0.0067	0.7526	CRP
rs1229984	C	T	C	T	-0.26171	0.0583	0.97277	NA	FALSE	FALSE	FALSE	YO3EtP	0.0202	0.003968	CRP
rs1310297	C	T	C	T	-0.01941	-0.0012	0.61881	NA	FALSE	FALSE	FALSE	YO3EtP	0.0059	0.8355	CRP
rs1317844	T	C	T	C	-0.01865	-0.0131	0.276349	NA	FALSE	FALSE	FALSE	YO3EtP	0.0061	0.03069	CRP
rs1339001	C	T	C	T	0.029612	0.0051	0.134041	NA	FALSE	FALSE	FALSE	YO3EtP	0.0089	0.565501	CRP
rs1421085	C	T	C	T	0.019939	0.0124	0.403447	NA	FALSE	FALSE	FALSE	YO3EtP	0.0057	0.02848	CRP
rs1666658	C	T	C	T	0.017967	0.002	0.392206	NA	FALSE	FALSE	FALSE	YO3EtP	0.0057	0.723401	CRP
rs1766275	C	T	C	T	0.030135	0.0054	0.089115	NA	FALSE	FALSE	FALSE	YO3EtP	0.012	0.6556	CRP
rs186347	T	G	T	G	0.017949	0.0061	0.463343	NA	FALSE	FALSE	FALSE	YO3EtP	0.0057	0.2843	CRP
rs1937522	G	A	G	A	0.016898	0.0066	0.528054	NA	FALSE	FALSE	FALSE	YO3EtP	0.0056	0.2385	CRP
rs1991083	T	C	T	C	-0.02239	-2.00E-04	0.679886	NA	FALSE	FALSE	FALSE	YO3EtP	0.006	0.9767	CRP
rs2043677	T	C	T	C	0.026113	-0.0035	0.145599	NA	FALSE	FALSE	FALSE	YO3EtP	0.0083	0.672699	CRP
rs2159935	A	G	A	G	-0.01857	-0.0052	0.490369	NA	FALSE	FALSE	FALSE	YO3EtP	0.0055	0.3439	CRP
rs2160935	T	C	T	C	-0.01872	-0.0027	0.604293	NA	FALSE	FALSE	FALSE	YO3EtP	0.0057	0.6342	CRP
rs2244598	C	T	C	T	-0.01838	-0.006	0.605114	NA	FALSE	FALSE	FALSE	YO3EtP	0.006	0.3178	CRP
rs2411453	G	T	G	T	-0.03508	-0.0107	0.597353	NA	FALSE	FALSE	FALSE	YO3EtP	0.0058	0.063979	CRP
rs2622167	A	G	A	G	-0.01912	-0.0016	0.428653	NA	FALSE	FALSE	FALSE	YO3EtP	0.0057	0.778999	CRP
rs262240	T	C	T	C	-0.01721	-0.005	0.468553	NA	FALSE	FALSE	FALSE	YO3EtP	0.0058	0.3888	CRP
rs2717063	A	C	A	C	-0.02037	-0.0026	0.585731	NA	FALSE	FALSE	FALSE	YO3EtP	0.0057	0.643	CRP
rs3444085	T	C	T	C	-0.02268	0.0083	0.157151	NA	FALSE	FALSE	FALSE	YO3EtP	0.0079	0.2934	CRP
rs3447388	A	G	A	G	-0.02036	0.0011	0.24819	NA	FALSE	FALSE	FALSE	YO3EtP	0.0065	0.8652	CRP
rs3463102	T	C	T	C	-0.01691	0.0042	0.446061	NA	FALSE	FALSE	FALSE	YO3EtP	0.0057	0.4591	CRP
rs3510514	T	C	T	C	0.026345	0.0066	0.401541	NA	FALSE	FALSE	FALSE	YO3EtP	0.0056	0.239	CRP
rs362307	T	C	T	C	0.043305	-0.0034	0.074582	NA	FALSE	FALSE	FALSE	YO3EtP	0.0103	0.743	CRP
rs4241258	T	C	T	C	0.025064	-0.002	0.13763	NA	FALSE	FALSE	FALSE	YO3EtP	0.0083	0.8126	CRP
rs4242715	A	G	A	G	-0.01865	0	0.680585	NA	FALSE	FALSE	FALSE	YO3EtP	0.006	0.9943	CRP
rs4417025	A	G	A	G	-0.01884	-0.0033	0.361153	NA	FALSE	FALSE	FALSE	YO3EtP	0.0058	0.5727	CRP
rs4503294	T	C	T	C	0.018148	-0.0066	0.565333	NA	FALSE	FALSE	FALSE	YO3EtP	0.0058	0.2548	CRP
rs461599	C	A	C	A	-0.01919	-2.00E-04	0.462259	NA	FALSE	FALSE	FALSE	YO3EtP	0.0057	0.9648	CRP
rs4800487	G	A	G	A	-0.02894	-0.0176	0.456873	NA	FALSE	FALSE	FALSE	YO3EtP	0.0056	0.001804	CRP
rs489062	A	G	A	G	0.01665	0.0111	0.437454	NA	FALSE	FALSE	FALSE	YO3EtP	0.0057	0.05124	CRP
rs4968391	T	G	T	G	-0.01927	0.0117	0.674892	NA	FALSE	FALSE	FALSE	YO3EtP	0.0059	0.04825	CRP
rs550942	T	C	T	C	0.022401	0.0044	0.823865	NA	FALSE	FALSE	FALSE	YO3EtP	0.0075	0.557	CRP
rs5619443	T	C	T	C	0.02254	-0.0081	0.16931	NA	FALSE	FALSE	FALSE	YO3EtP	0.0075	0.2757	CRP
rs5890541	A	G	A	G	-0.02663	-6.00E-04	0.410052	NA	FALSE	FALSE	FALSE	YO3EtP	0.0058	0.914	CRP
rs6187351	T	G	T	G	0.020374	-0.0067	0.32785	NA	FALSE	FALSE	FALSE	YO3EtP	0.006	0.2673	CRP
rs6233967	A	C	A	C	0.018294	-0.0021	0.626705	NA	FALSE	FALSE	FALSE	YO3EtP	0.0059	0.722301	CRP
rs6246631	T	C	T	C	-0.02549	-0.0192	0.202827	NA	FALSE	FALSE	FALSE	YO3EtP	0.0069	0.005557	CRP
rs650558	T	C	T	C	0.020736	0.0138	0.247918	NA	FALSE	FALSE	FALSE	YO3EtP	0.0063	0.02943	CRP
rs6727281	T	C	T	C	-0.02432	-0.0074	0.184023	NA	FALSE	FALSE	FALSE	YO3EtP	0.0072	0.305	CRP
rs6943160	C	T	C	T	0.020627	0.0039	0.208646	NA	FALSE	FALSE	FALSE	YO3EtP	0.0069	0.5751	CRP
rs7165168	T	C	T	C	-0.07046	-0.0075	0.0142	NA	FALSE	FALSE	FALSE	YO3EtP	0.0237	0.75	CRP
rs7278706	A	G	A	G	-0.02819	0.015	0.162767	NA	FALSE	FALSE	FALSE	YO3EtP	0.0075	0.0463	CRP
rs728538	G	T	G	T	0.022875	0.029	0.168868	NA	FALSE	FALSE	FALSE	YO3EtP	0.0077	0.000179	CRP
rs7298932	G	A	G	A	-0.02372	-0.0035	0.147849	NA	FALSE	FALSE	FALSE	YO3EtP	0.0081	0.667599	CRP
rs7330939	T	C	T	C	-0.02133	-0.0056	0.720352	NA	FALSE	FALSE	FALSE	YO3EtP	0.0065	0.3858	CRP
rs7460106	C	T	C	T	-0.02171	0.0133	0.23562	NA	FALSE	FALSE	FALSE	YO3EtP	0.0069	0.053291	CRP
rs7467914	C	T	C	T	-0.03207	0.003	0.074515	NA	FALSE	FALSE	FALSE	YO3EtP	0.0112	0.7896	CRP
rs7514579	C	A	C	A	0.019667	-7.00E-04	0.232457	NA	FALSE	FALSE	FALSE	YO3EtP	0.0065	0.9118	CRP
rs7608265	T	C	T	C	0.046427	0.0187	0.054327	NA	FALSE	FALSE	FALSE	YO3EtP	0.0131	0.1526	CRP
rs8029231	C	T	C	T	-0.03937	-0.0189	0.057704	NA	FALSE	FALSE	FALSE	YO3EtP	0.0118	0.1095	CRP
rs8614	A	C	A	C	0.024781	0.0155	0.182509	NA	FALSE	FALSE	FALSE	YO3EtP	0.0075	0.03945	CRP
rs9403297	A	G	A	G	0.018823	-2.00E-04	0.372967	NA	FALSE	FALSE	FALSE	YO3EtP	0.006	0.9683	CRP
rs9829192	T	G	T	G	0.016932	-0.0044	0.435133	NA	FALSE	FALSE	FALSE	YO3EtP	0.0058	0.4507	CRP
rs9906502	A	G	A	G	0.023788	0.0137	0.176998	NA	FALSE	FALSE	FALSE	YO3EtP	0.0072	0.05707	CRP

SNP	mr_keep.ou	pval_origin	data_sourc	se.exposure	exposure	mr_keep.ex	pval.expos	pval_origin	id.exposure	data_sourc	action	mr_keep	samplesize.outcome
rs1018831	TRUE	reported	textfile	0.003036	alcohol inta	TRUE	7.16E-11	inferred	haGrSf	textfile	2	TRUE	NA
rs1079266	TRUE	reported	textfile	0.003041	alcohol inta	TRUE	9.86E-09	inferred	haGrSf	textfile	2	TRUE	NA
rs1103942	TRUE	reported	textfile	0.003037	alcohol inta	TRUE	8.73E-15	inferred	haGrSf	textfile	2	TRUE	NA
rs1122361	TRUE	reported	textfile	0.003754	alcohol inta	TRUE	2.32E-11	inferred	haGrSf	textfile	2	TRUE	NA
rs1170085	TRUE	reported	textfile	0.005233	alcohol inta	TRUE	1.24E-08	inferred	haGrSf	textfile	2	TRUE	NA
rs1175077	TRUE	reported	textfile	0.003726	alcohol inta	TRUE	3.80E-08	inferred	haGrSf	textfile	2	TRUE	NA
rs1178721	TRUE	reported	textfile	0.003201	alcohol inta	TRUE	2.38E-14	inferred	haGrSf	textfile	2	TRUE	NA
rs1194069	TRUE	reported	textfile	0.003116	alcohol inta	TRUE	1.04E-44	inferred	haGrSf	textfile	2	TRUE	NA
rs1228589	TRUE	reported	textfile	0.003528	alcohol inta	TRUE	2.34E-09	inferred	haGrSf	textfile	2	TRUE	NA
rs1229984	TRUE	reported	textfile	0.009185	alcohol inta	TRUE	1.43E-178	inferred	haGrSf	textfile	2	TRUE	NA
rs1310297	TRUE	reported	textfile	0.003119	alcohol inta	TRUE	4.89E-10	inferred	haGrSf	textfile	2	TRUE	NA
rs1317844	TRUE	reported	textfile	0.00339	alcohol inta	TRUE	3.75E-08	inferred	haGrSf	textfile	2	TRUE	NA
rs1339001	TRUE	reported	textfile	0.004492	alcohol inta	TRUE	4.33E-11	inferred	haGrSf	textfile	2	TRUE	NA
rs1421085	TRUE	reported	textfile	0.003085	alcohol inta	TRUE	1.02E-10	inferred	haGrSf	textfile	2	TRUE	NA
rs1666658	TRUE	reported	textfile	0.003099	alcohol inta	TRUE	6.69E-09	inferred	haGrSf	textfile	2	TRUE	NA
rs1766275	TRUE	reported	textfile	0.00546	alcohol inta	TRUE	3.41E-08	inferred	haGrSf	textfile	2	TRUE	NA
rs186347	TRUE	reported	textfile	0.003051	alcohol inta	TRUE	4.02E-09	inferred	haGrSf	textfile	2	TRUE	NA
rs1937522	TRUE	reported	textfile	0.003032	alcohol inta	TRUE	2.50E-08	inferred	haGrSf	textfile	2	TRUE	NA
rs1991083	TRUE	reported	textfile	0.003258	alcohol inta	TRUE	6.30E-12	inferred	haGrSf	textfile	2	TRUE	NA
rs2043677	TRUE	reported	textfile	0.004327	alcohol inta	TRUE	1.59E-09	inferred	haGrSf	textfile	2	TRUE	NA
rs2159935	TRUE	reported	textfile	0.003026	alcohol inta	TRUE	8.33E-10	inferred	haGrSf	textfile	2	TRUE	NA
rs2160935	TRUE	reported	textfile	0.003091	alcohol inta	TRUE	1.40E-09	inferred	haGrSf	textfile	2	TRUE	NA
rs2244598	TRUE	reported	textfile	0.003119	alcohol inta	TRUE	3.81E-09	inferred	haGrSf	textfile	2	TRUE	NA
rs2411453	TRUE	reported	textfile	0.00309	alcohol inta	TRUE	7.32E-30	inferred	haGrSf	textfile	2	TRUE	NA
rs2622167	TRUE	reported	textfile	0.003067	alcohol inta	TRUE	4.61E-10	inferred	haGrSf	textfile	2	TRUE	NA
rs262240	TRUE	reported	textfile	0.003035	alcohol inta	TRUE	1.43E-08	inferred	haGrSf	textfile	2	TRUE	NA
rs2717063	TRUE	reported	textfile	0.003085	alcohol inta	TRUE	4.00E-11	inferred	haGrSf	textfile	2	TRUE	NA
rs3444085	TRUE	reported	textfile	0.004151	alcohol inta	TRUE	4.63E-08	inferred	haGrSf	textfile	2	TRUE	NA
rs3447388	TRUE	reported	textfile	0.003503	alcohol inta	TRUE	6.18E-09	inferred	haGrSf	textfile	2	TRUE	NA
rs3463102	TRUE	reported	textfile	0.003048	alcohol inta	TRUE	2.89E-08	inferred	haGrSf	textfile	2	TRUE	NA
rs3510514	TRUE	reported	textfile	0.003088	alcohol inta	TRUE	1.44E-17	inferred	haGrSf	textfile	2	TRUE	NA
rs362307	TRUE	reported	textfile	0.005802	alcohol inta	TRUE	8.42E-14	inferred	haGrSf	textfile	2	TRUE	NA
rs4241258	TRUE	reported	textfile	0.004403	alcohol inta	TRUE	1.26E-08	inferred	haGrSf	textfile	2	TRUE	NA
rs4242715	TRUE	reported	textfile	0.003248	alcohol inta	TRUE	9.31E-09	inferred	haGrSf	textfile	2	TRUE	NA
rs4417025	TRUE	reported	textfile	0.003165	alcohol inta	TRUE	2.65E-09	inferred	haGrSf	textfile	2	TRUE	NA
rs4503294	TRUE	reported	textfile	0.00307	alcohol inta	TRUE	3.41E-09	inferred	haGrSf	textfile	2	TRUE	NA
rs461599	TRUE	reported	textfile	0.00304	alcohol inta	TRUE	2.74E-10	inferred	haGrSf	textfile	2	TRUE	NA
rs4800487	TRUE	reported	textfile	0.003047	alcohol inta	TRUE	2.16E-21	inferred	haGrSf	textfile	2	TRUE	NA
rs489062	TRUE	reported	textfile	0.003053	alcohol inta	TRUE	4.93E-08	inferred	haGrSf	textfile	2	TRUE	NA
rs4968391	TRUE	reported	textfile	0.003227	alcohol inta	TRUE	2.34E-09	inferred	haGrSf	textfile	2	TRUE	NA
rs550942	TRUE	reported	textfile	0.003989	alcohol inta	TRUE	1.96E-08	inferred	haGrSf	textfile	2	TRUE	NA
rs5619443	TRUE	reported	textfile	0.004071	alcohol inta	TRUE	3.09E-08	inferred	haGrSf	textfile	2	TRUE	NA
rs5890541	TRUE	reported	textfile	0.003078	alcohol inta	TRUE	5.07E-18	inferred	haGrSf	textfile	2	TRUE	NA
rs6187351	TRUE	reported	textfile	0.003303	alcohol inta	TRUE	6.91E-10	inferred	haGrSf	textfile	2	TRUE	NA
rs6233967	TRUE	reported	textfile	0.003154	alcohol inta	TRUE	6.62E-09	inferred	haGrSf	textfile	2	TRUE	NA
rs6246631	TRUE	reported	textfile	0.003774	alcohol inta	TRUE	1.44E-11	inferred	haGrSf	textfile	2	TRUE	NA
rs650558	TRUE	reported	textfile	0.003508	alcohol inta	TRUE	3.39E-09	inferred	haGrSf	textfile	2	TRUE	NA
rs6727281	TRUE	reported	textfile	0.00392	alcohol inta	TRUE	5.46E-10	inferred	haGrSf	textfile	2	TRUE	NA
rs6943160	TRUE	reported	textfile	0.003728	alcohol inta	TRUE	3.14E-08	inferred	haGrSf	textfile	2	TRUE	NA
rs7165168	TRUE	reported	textfile	0.012791	alcohol inta	TRUE	3.62E-08	inferred	haGrSf	textfile	2	TRUE	NA
rs7278706	TRUE	reported	textfile	0.004103	alcohol inta	TRUE	6.36E-12	inferred	haGrSf	textfile	2	TRUE	NA
rs728538	TRUE	reported	textfile	0.004063	alcohol inta	TRUE	1.79E-08	inferred	haGrSf	textfile	2	TRUE	NA
rs7298932	TRUE	reported	textfile	0.004312	alcohol inta	TRUE	3.76E-08	inferred	haGrSf	textfile	2	TRUE	NA
rs7330939	TRUE	reported	textfile	0.003405	alcohol inta	TRUE	3.73E-10	inferred	haGrSf	textfile	2	TRUE	NA
rs7460106	TRUE	reported	textfile	0.003619	alcohol inta	TRUE	1.99E-09	inferred	haGrSf	textfile	2	TRUE	NA
rs7467914	TRUE	reported	textfile	0.005758	alcohol inta	TRUE	2.54E-08	inferred	haGrSf	textfile	2	TRUE	NA
rs7514579	TRUE	reported	textfile	0.003598	alcohol inta	TRUE	4.59E-08	inferred	haGrSf	textfile	2	TRUE	NA
rs7608265	TRUE	reported	textfile	0.006687	alcohol inta	TRUE	3.83E-12	inferred	haGrSf	textfile	2	TRUE	NA
rs8029231	TRUE	reported	textfile	0.006496	alcohol inta	TRUE	1.35E-09	inferred	haGrSf	textfile	2	TRUE	NA
rs8614	TRUE	reported	textfile	0.003925	alcohol inta	TRUE	2.74E-10	inferred	haGrSf	textfile	2	TRUE	NA
rs9403297	TRUE	reported	textfile	0.00313	alcohol inta	TRUE	1.82E-09	inferred	haGrSf	textfile	2	TRUE	NA
rs9829192	TRUE	reported	textfile	0.00305	alcohol inta	TRUE	2.84E-08	inferred	haGrSf	textfile	2	TRUE	NA
rs9906502	TRUE	reported	textfile	0.003962	alcohol inta	TRUE	1.92E-09	inferred	haGrSf	textfile	2	TRUE	NA

SNPs of alcohol intake frequency on hydroxyhippurate

SNP	effect_allel	other_allel	effect_allel	other_allel	beta.expos	beta.outco	eaf.exposu	eaf.outcom	remove	palindromi	ambiguous	id.outcome	se.outcome	pval.outco	outcome	mr_keep.o	pval_origin	data_sourc	se.exposure	exposure	mr_keep.e	pval.expos	pval_origin	id.exposure	data_sourc	action	mr_keep	samplesize.outcome
rs1018831	T	C	T	C	-0.01979	0.0124	0.470852	0.4596	FALSE	FALSE	FALSE	5hZSMJ	0.0078	0.1095	4-hydroxyl	TRUE	reported	textfile	0.003036	alcohol inta	TRUE	7.16E-11	inferred	w07FrX	textfile	2	TRUE	NA
rs1079266	G	A	G	A	0.017432	-0.0056	0.505254	0.5095	FALSE	FALSE	FALSE	5hZSMJ	0.0078	0.4677	4-hydroxyl	TRUE	reported	textfile	0.003041	alcohol inta	TRUE	9.86E-09	inferred	w07FrX	textfile	2	TRUE	NA
rs1122361	A	G	A	G	0.025091	0.0106	0.206155	0.2075	FALSE	FALSE	FALSE	5hZSMJ	0.0095	0.2675	4-hydroxyl	TRUE	reported	textfile	0.003754	alcohol inta	TRUE	2.32E-11	inferred	w07FrX	textfile	2	TRUE	NA
rs1170085	G	A	G	A	-0.0298	-0.0059	0.093465	0.0922	FALSE	FALSE	FALSE	5hZSMJ	0.0135	0.663399	4-hydroxyl	TRUE	reported	textfile	0.005233	alcohol inta	TRUE	1.24E-08	inferred	w07FrX	textfile	2	TRUE	NA
rs1175077	A	G	A	G	-0.02049	0.0127	0.209454	0.1925	FALSE	FALSE	FALSE	5hZSMJ	0.0095	0.1803	4-hydroxyl	TRUE	reported	textfile	0.003726	alcohol inta	TRUE	3.80E-08	inferred	w07FrX	textfile	2	TRUE	NA
rs1178721	T	C	T	C	0.024416	2.00E-04	0.369127	0.3735	FALSE	FALSE	FALSE	5hZSMJ	0.0081	0.9754	4-hydroxyl	TRUE	reported	textfile	0.003201	alcohol inta	TRUE	2.38E-14	inferred	w07FrX	textfile	2	TRUE	NA
rs1194069	G	A	G	A	-0.04371	-0.008	0.604193	0.5743	FALSE	FALSE	FALSE	5hZSMJ	0.0079	0.3083	4-hydroxyl	TRUE	reported	textfile	0.003116	alcohol inta	TRUE	1.04E-44	inferred	w07FrX	textfile	2	TRUE	NA
rs1215385	C	T	C	T	0.029444	-0.0315	0.10497	0.1001	FALSE	FALSE	FALSE	5hZSMJ	0.0138	0.02227	4-hydroxyl	TRUE	reported	textfile	0.004935	alcohol inta	TRUE	2.42E-09	inferred	w07FrX	textfile	2	TRUE	NA
rs1228589	A	G	A	G	0.02107	0.0141	0.246133	0.2336	FALSE	FALSE	FALSE	5hZSMJ	0.0091	0.1235	4-hydroxyl	TRUE	reported	textfile	0.003528	alcohol inta	TRUE	2.34E-09	inferred	w07FrX	textfile	2	TRUE	NA
rs1310297	C	T	C	T	-0.01941	-0.0036	0.61881	0.6423	FALSE	FALSE	FALSE	5hZSMJ	0.0078	0.6414	4-hydroxyl	TRUE	reported	textfile	0.003119	alcohol inta	TRUE	4.89E-10	inferred	w07FrX	textfile	2	TRUE	NA
rs1421085	C	T	C	T	0.019939	0.0164	0.403447	0.4367	FALSE	FALSE	FALSE	5hZSMJ	0.0105	0.1187	4-hydroxyl	TRUE	reported	textfile	0.003085	alcohol inta	TRUE	1.02E-10	inferred	w07FrX	textfile	2	TRUE	NA
rs1766275	C	T	C	T	0.030135	-0.0544	0.089115	0.0944	FALSE	FALSE	FALSE	5hZSMJ	0.0206	0.008182	4-hydroxyl	TRUE	reported	textfile	0.00546	alcohol inta	TRUE	3.41E-08	inferred	w07FrX	textfile	2	TRUE	NA
rs186347	T	G	T	G	0.017949	0.003	0.463343	0.4785	FALSE	FALSE	FALSE	5hZSMJ	0.008	0.7112	4-hydroxyl	TRUE	reported	textfile	0.003051	alcohol inta	TRUE	4.02E-09	inferred	w07FrX	textfile	2	TRUE	NA
rs1937522	G	A	G	A	0.016898	-0.0025	0.528054	0.5231	FALSE	FALSE	FALSE	5hZSMJ	0.0072	0.7281	4-hydroxyl	TRUE	reported	textfile	0.003032	alcohol inta	TRUE	2.50E-08	inferred	w07FrX	textfile	2	TRUE	NA
rs1991083	T	C	T	C	-0.02239	-7.00E-04	0.679886	0.7087	FALSE	FALSE	FALSE	5hZSMJ	0.0088	0.9361	4-hydroxyl	TRUE	reported	textfile	0.003258	alcohol inta	TRUE	6.30E-12	inferred	w07FrX	textfile	2	TRUE	NA
rs2159935	A	G	A	G	-0.01857	-0.0058	0.490369	0.4817	FALSE	FALSE	FALSE	5hZSMJ	0.0078	0.4511	4-hydroxyl	TRUE	reported	textfile	0.003026	alcohol inta	TRUE	8.33E-10	inferred	w07FrX	textfile	2	TRUE	NA
rs2160935	T	C	T	C	-0.01872	8.00E-04	0.604293	0.5903	FALSE	FALSE	FALSE	5hZSMJ	0.0076	0.9172	4-hydroxyl	TRUE	reported	textfile	0.003091	alcohol inta	TRUE	1.40E-09	inferred	w07FrX	textfile	2	TRUE	NA
rs2411453	G	T	G	T	-0.03508	-0.001	0.597353	0.5827	FALSE	FALSE	FALSE	5hZSMJ	0.0081	0.9012	4-hydroxyl	TRUE	reported	textfile	0.00309	alcohol inta	TRUE	7.32E-30	inferred	w07FrX	textfile	2	TRUE	NA
rs2535911	T	C	T	C	-0.01885	0.0042	0.354749	0.3396	FALSE	FALSE	FALSE	5hZSMJ	0.0082	0.61	4-hydroxyl	TRUE	reported	textfile	0.003168	alcohol inta	TRUE	2.71E-09	inferred	w07FrX	textfile	2	TRUE	NA
rs2717063	A	C	A	C	-0.02037	0.0016	0.585731	0.5759	FALSE	FALSE	FALSE	5hZSMJ	0.0078	0.8422	4-hydroxyl	TRUE	reported	textfile	0.003085	alcohol inta	TRUE	4.00E-11	inferred	w07FrX	textfile	2	TRUE	NA
rs4241258	T	C	T	C	0.025064	0.0086	0.13763	0.1334	FALSE	FALSE	FALSE	5hZSMJ	0.0109	0.4293	4-hydroxyl	TRUE	reported	textfile	0.004403	alcohol inta	TRUE	1.26E-08	inferred	w07FrX	textfile	2	TRUE	NA
rs4417025	A	G	A	G	-0.01884	-0.01	0.361153	0.331	FALSE	FALSE	FALSE	5hZSMJ	0.012	0.4047	4-hydroxyl	TRUE	reported	textfile	0.003165	alcohol inta	TRUE	2.65E-09	inferred	w07FrX	textfile	2	TRUE	NA
rs461599	C	A	C	A	-0.01919	0.0147	0.462259	0.4783	FALSE	FALSE	FALSE	5hZSMJ	0.0075	0.05202	4-hydroxyl	TRUE	reported	textfile	0.00304	alcohol inta	TRUE	2.74E-10	inferred	w07FrX	textfile	2	TRUE	NA
rs489062	A	G	A	G	0.01665	0.0102	0.437454	0.4474	FALSE	FALSE	FALSE	5hZSMJ	0.0077	0.1833	4-hydroxyl	TRUE	reported	textfile	0.003053	alcohol inta	TRUE	4.93E-08	inferred	w07FrX	textfile	2	TRUE	NA
rs4916723	C	A	C	A	0.023948	0.0065	0.420617	0.4094	FALSE	FALSE	FALSE	5hZSMJ	0.0077	0.4001	4-hydroxyl	TRUE	reported	textfile	0.0031	alcohol inta	TRUE	1.11E-14	inferred	w07FrX	textfile	2	TRUE	NA
rs5022348	T	C	T	C	0.020264	-0.0153	0.40703	0.5615	FALSE	FALSE	FALSE	5hZSMJ	0.0078	0.04949	4-hydroxyl	TRUE	reported	textfile	0.00357	alcohol inta	TRUE	1.38E-08	inferred	w07FrX	textfile	2	TRUE	NA
rs550942	T	C	T	C	0.022401	-0.0035	0.823865	0.8353	FALSE	FALSE	FALSE	5hZSMJ	0.0103	0.732	4-hydroxyl	TRUE	reported	textfile	0.003989	alcohol inta	TRUE	1.96E-08	inferred	w07FrX	textfile	2	TRUE	NA
rs6727281	T	C	T	C	-0.02432	-0.0033	0.184023	0.1785	FALSE	FALSE	FALSE	5hZSMJ	0.0101	0.7461	4-hydroxyl	TRUE	reported	textfile	0.00392	alcohol inta	TRUE	5.46E-10	inferred	w07FrX	textfile	2	TRUE	NA
rs7610856	A	C	A	C	-0.02386	0.0158	0.429053	0.4096	FALSE	FALSE	FALSE	5hZSMJ	0.0077	0.04064	4-hydroxyl	TRUE	reported	textfile	0.00307	alcohol inta	TRUE	7.68E-15	inferred	w07FrX	textfile	2	TRUE	NA
rs780094	C	T	C	T	-0.05099	0.0045	0.615206	0.6075	FALSE	FALSE	FALSE	5hZSMJ	0.0077	0.5575	4-hydroxyl	TRUE	reported	textfile	0.003105	alcohol inta	TRUE	1.31E-60	inferred	w07FrX	textfile	2	TRUE	NA
rs780569	A	T	A	T	0.019803	7.00E-04	0.70882	0.7358	FALSE	TRUE	FALSE	5hZSMJ	0.0085	0.9377	4-hydroxyl	TRUE	reported	textfile	0.003365	alcohol inta	TRUE	3.96E-09	inferred	w07FrX	textfile	2	TRUE	NA
rs838145	A	G	A	G	0.021955	0.0019	0.542982	0.5692	FALSE	FALSE	FALSE	5hZSMJ	0.0078	0.807	4-hydroxyl	TRUE	reported	textfile	0.003055	alcohol inta	TRUE	6.70E-13	inferred	w07FrX	textfile	2	TRUE	NA
rs8614	A	C	A	C	0.024781	-0.0021	0.182509	0.1782	FALSE	FALSE	FALSE	5hZSMJ	0.0106	0.8394	4-hydroxyl	TRUE	reported	textfile	0.003925	alcohol inta	TRUE	2.74E-10	inferred	w07FrX	textfile	2	TRUE	NA
rs9349379	G	A	G	A	-0.01935	0.0093	0.405493	0.4026	FALSE	FALSE	FALSE	5hZSMJ	0.0079	0.2369	4-hydroxyl	TRUE	reported	textfile	0.003082	alcohol inta	TRUE	3.46E-10	inferred	w07FrX	textfile	2	TRUE	NA
rs9372625	A	G	A	G	-0.02556	-0.0186	0.381706	0.3803	FALSE	FALSE	FALSE	5hZSMJ	0.0077	0.01615	4-hydroxyl	TRUE	reported	textfile	0.003125	alcohol inta	TRUE	2.85E-16	inferred	w07FrX	textfile	2	TRUE	NA
rs9814516	T	G	T	G	-0.02511	-0.0102	0.237423	0.2356	FALSE	FALSE	FALSE	5hZSMJ	0.0086	0.2346	4-hydroxyl	TRUE	reported	textfile	0.003556	alcohol inta	TRUE	1.64E-12	inferred	w07FrX	textfile	2	TRUE	NA

SNPs of alcohol intake frequency on IL-6

SNP	effect_allel	other_allel	effect_allel	other_allel	beta.expos	beta.outco	eaf.exposu	eaf.outcom	remove	palindromi	ambiguous	id.outcome	se.outcome	pval.outcor	outcome	mr_keep.ou	pval_origin	data_sourc	se.exposure	exposure	mr_keep.ex	pval.expos	pval_origin	id.exposure	data_sourc	action	mr_keep	samplesize.outcome
rs1004787	A	G	A	G	-0.02223	0.0031	0.532943	NA	FALSE	FALSE	FALSE	NWehKT	0.0161	0.8487	interleukin-	TRUE	reported	textfile	0.003045	alcohol inta	TRUE	2.90E-13	inferred	hcTyg4	textfile	2	TRUE	NA
rs1018831	T	C	T	C	-0.01979	0.0011	0.470852	NA	FALSE	FALSE	FALSE	NWehKT	0.0158	0.9294	interleukin-	TRUE	reported	textfile	0.003036	alcohol inta	TRUE	7.16E-11	inferred	hcTyg4	textfile	2	TRUE	NA
rs1079266	G	A	G	A	0.017432	-0.0326	0.505254	NA	FALSE	FALSE	FALSE	NWehKT	0.0157	0.03842	interleukin-	TRUE	reported	textfile	0.003041	alcohol inta	TRUE	9.86E-09	inferred	hcTyg4	textfile	2	TRUE	NA
rs1103942	T	C	T	C	-0.02356	-0.0027	0.454624	NA	FALSE	FALSE	FALSE	NWehKT	0.0164	0.8789	interleukin-	TRUE	reported	textfile	0.003037	alcohol inta	TRUE	8.73E-15	inferred	hcTyg4	textfile	2	TRUE	NA
rs1122361	A	G	A	G	0.025091	-0.0229	0.206155	NA	FALSE	FALSE	FALSE	NWehKT	0.0174	0.1865	interleukin-	TRUE	reported	textfile	0.003754	alcohol inta	TRUE	2.32E-11	inferred	hcTyg4	textfile	2	TRUE	NA
rs1170085	G	A	G	A	-0.0298	0.0288	0.093465	NA	FALSE	FALSE	FALSE	NWehKT	0.0253	0.2549	interleukin-	TRUE	reported	textfile	0.005233	alcohol inta	TRUE	1.24E-08	inferred	hcTyg4	textfile	2	TRUE	NA
rs1175077	A	G	A	G	-0.02049	6.00E-04	0.209454	NA	FALSE	FALSE	FALSE	NWehKT	0.0222	0.9743	interleukin-	TRUE	reported	textfile	0.003726	alcohol inta	TRUE	3.80E-08	inferred	hcTyg4	textfile	2	TRUE	NA
rs1178721	T	C	T	C	0.024416	0.0029	0.369127	NA	FALSE	FALSE	FALSE	NWehKT	0.0166	0.8594	interleukin-	TRUE	reported	textfile	0.003201	alcohol inta	TRUE	2.38E-14	inferred	hcTyg4	textfile	2	TRUE	NA
rs1194069	G	A	G	A	-0.04371	0.0066	0.604193	NA	FALSE	FALSE	FALSE	NWehKT	0.0163	0.683	interleukin-	TRUE	reported	textfile	0.003116	alcohol inta	TRUE	1.04E-44	inferred	hcTyg4	textfile	2	TRUE	NA
rs1215385	C	T	C	T	0.029444	-0.0274	0.10497	NA	FALSE	FALSE	FALSE	NWehKT	0.0338	0.4202	interleukin-	TRUE	reported	textfile	0.004935	alcohol inta	TRUE	2.42E-09	inferred	hcTyg4	textfile	2	TRUE	NA
rs1228589	A	G	A	G	0.02107	0.0156	0.246133	NA	FALSE	FALSE	FALSE	NWehKT	0.0178	0.3839	interleukin-	TRUE	reported	textfile	0.003528	alcohol inta	TRUE	2.34E-09	inferred	hcTyg4	textfile	2	TRUE	NA
rs1310297	C	T	C	T	-0.01941	-0.0067	0.61881	NA	FALSE	FALSE	FALSE	NWehKT	0.0158	0.668299	interleukin-	TRUE	reported	textfile	0.003119	alcohol inta	TRUE	4.89E-10	inferred	hcTyg4	textfile	2	TRUE	NA
rs1313509	G	A	G	A	0.043834	-0.2223	0.083483	NA	FALSE	FALSE	FALSE	NWehKT	0.0658	0.000708	interleukin-	TRUE	reported	textfile	0.005499	alcohol inta	TRUE	1.57E-15	inferred	hcTyg4	textfile	2	TRUE	NA
rs1317844	T	C	T	C	-0.01865	-0.0192	0.276349	NA	FALSE	FALSE	FALSE	NWehKT	0.0171	0.2648	interleukin-	TRUE	reported	textfile	0.00339	alcohol inta	TRUE	3.75E-08	inferred	hcTyg4	textfile	2	TRUE	NA
rs1339001	C	T	C	T	0.029612	0.0059	0.134041	NA	FALSE	FALSE	FALSE	NWehKT	0.0359	0.8651	interleukin-	TRUE	reported	textfile	0.004492	alcohol inta	TRUE	4.33E-11	inferred	hcTyg4	textfile	2	TRUE	NA
rs1421085	C	T	C	T	0.019939	0.0196	0.403447	NA	FALSE	FALSE	FALSE	NWehKT	0.0158	0.2162	interleukin-	TRUE	reported	textfile	0.003085	alcohol inta	TRUE	1.02E-10	inferred	hcTyg4	textfile	2	TRUE	NA
rs1515590	T	C	T	C	0.018245	-0.026	0.383245	NA	FALSE	FALSE	FALSE	NWehKT	0.016	0.1042	interleukin-	TRUE	reported	textfile	0.003116	alcohol inta	TRUE	4.78E-09	inferred	hcTyg4	textfile	2	TRUE	NA
rs1666658	C	T	C	T	0.017967	0.0029	0.392206	NA	FALSE	FALSE	FALSE	NWehKT	0.0161	0.8544	interleukin-	TRUE	reported	textfile	0.003099	alcohol inta	TRUE	6.69E-09	inferred	hcTyg4	textfile	2	TRUE	NA
rs1766275	C	T	C	T	0.030135	-0.0429	0.089115	NA	FALSE	FALSE	FALSE	NWehKT	0.029	0.1401	interleukin-	TRUE	reported	textfile	0.00546	alcohol inta	TRUE	3.41E-08	inferred	hcTyg4	textfile	2	TRUE	NA
rs186347	T	G	T	G	0.017949	0.0132	0.463343	NA	FALSE	FALSE	FALSE	NWehKT	0.0159	0.4065	interleukin-	TRUE	reported	textfile	0.003051	alcohol inta	TRUE	4.02E-09	inferred	hcTyg4	textfile	2	TRUE	NA
rs1937522	G	A	G	A	0.016898	-0.0132	0.528054	NA	FALSE	FALSE	FALSE	NWehKT	0.0158	0.3999	interleukin-	TRUE	reported	textfile	0.003032	alcohol inta	TRUE	2.50E-08	inferred	hcTyg4	textfile	2	TRUE	NA
rs1991083	T	C	T	C	-0.02239	0.0254	0.679886	NA	FALSE	FALSE	FALSE	NWehKT	0.0182	0.1635	interleukin-	TRUE	reported	textfile	0.003258	alcohol inta	TRUE	6.30E-12	inferred	hcTyg4	textfile	2	TRUE	NA
rs2043677	T	C	T	C	0.026113	-0.037	0.145599	NA	FALSE	FALSE	FALSE	NWehKT	0.0216	0.08737	interleukin-	TRUE	reported	textfile	0.004327	alcohol inta	TRUE	1.59E-09	inferred	hcTyg4	textfile	2	TRUE	NA
rs2159935	A	G	A	G	-0.01857	-0.0219	0.490369	NA	FALSE	FALSE	FALSE	NWehKT	0.0156	0.1652	interleukin-	TRUE	reported	textfile	0.003026	alcohol inta	TRUE	8.33E-10	inferred	hcTyg4	textfile	2	TRUE	NA
rs2160935	T	C	T	C	-0.01872	-0.0339	0.604293	NA	FALSE	FALSE	FALSE	NWehKT	0.0167	0.04287	interleukin-	TRUE	reported	textfile	0.003091	alcohol inta	TRUE	1.40E-09	inferred	hcTyg4	textfile	2	TRUE	NA
rs2244598	C	T	C	T	-0.01838	0.0038	0.605114	NA	FALSE	FALSE	FALSE	NWehKT	0.0165	0.8226	interleukin-	TRUE	reported	textfile	0.003119	alcohol inta	TRUE	3.81E-09	inferred	hcTyg4	textfile	2	TRUE	NA
rs2411453	G	T	G	T	-0.03508	-0.02	0.597353	NA	FALSE	FALSE	FALSE	NWehKT	0.0158	0.21	interleukin-	TRUE	reported	textfile	0.00309	alcohol inta	TRUE	7.32E-30	inferred	hcTyg4	textfile	2	TRUE	NA
rs2535911	T	C	T	C	-0.01885	6.00E-04	0.354749	NA	FALSE	FALSE	FALSE	NWehKT	0.0157	0.9548	interleukin-	TRUE	reported	textfile	0.003168	alcohol inta	TRUE	2.71E-09	inferred	hcTyg4	textfile	2	TRUE	NA
rs2622167	A	G	A	G	-0.01912	0.0049	0.428653	NA	FALSE	FALSE	FALSE	NWehKT	0.017	0.7952	interleukin-	TRUE	reported	textfile	0.003067	alcohol inta	TRUE	4.61E-10	inferred	hcTyg4	textfile	2	TRUE	NA
rs262240	T	C	T	C	-0.01721	0.0139	0.468553	NA	FALSE	FALSE	FALSE	NWehKT	0.0162	0.3905	interleukin-	TRUE	reported	textfile	0.003035	alcohol inta	TRUE	1.43E-08	inferred	hcTyg4	textfile	2	TRUE	NA
rs2717063	A	C	A	C	-0.02037	0.0127	0.585731	NA	FALSE	FALSE	FALSE	NWehKT	0.0161	0.4319	interleukin-	TRUE	reported	textfile	0.003085	alcohol inta	TRUE	4.00E-11	inferred	hcTyg4	textfile	2	TRUE	NA
rs2876812	C	T	C	T	0.0207	0.0269	0.759525	NA	FALSE	FALSE	FALSE	NWehKT	0.0178	0.1316	interleukin-	TRUE	reported	textfile	0.003552	alcohol inta	TRUE	5.62E-09	inferred	hcTyg4	textfile	2	TRUE	NA
rs2878710	A	G	A	G	0.017811	0.0111	0.40423	NA	FALSE	FALSE	FALSE	NWehKT	0.0185	0.5427	interleukin-	TRUE	reported	textfile	0.003085	alcohol inta	TRUE	7.74E-09	inferred	hcTyg4	textfile	2	TRUE	NA
rs324012	T	C	T	C	-0.01779	0.0158	0.448569	NA	FALSE	FALSE	FALSE	NWehKT	0.0157	0.3154	interleukin-	TRUE	reported	textfile	0.003044	alcohol inta	TRUE	5.03E-09	inferred	hcTyg4	textfile	2	TRUE	NA
rs3444085	T	C	T	C	-0.02268	-0.0117	0.157151	NA	FALSE	FALSE	FALSE	NWehKT	0.0189	0.5497	interleukin-	TRUE	reported	textfile	0.004151	alcohol inta	TRUE	4.63E-08	inferred	hcTyg4	textfile	2	TRUE	NA
rs3447388	A	G	A	G	-0.02036	-0.0075	0.24819	NA	FALSE	FALSE	FALSE	NWehKT	0.0189	0.6991	interleukin-	TRUE	reported	textfile	0.003503	alcohol inta	TRUE	6.18E-09	inferred	hcTyg4	textfile	2	TRUE	NA
rs3463102	T	C	T	C	-0.01691	0.0112	0.446061	NA	FALSE	FALSE	FALSE	NWehKT	0.0168	0.513	interleukin-	TRUE	reported	textfile	0.003048	alcohol inta	TRUE	2.89E-08	inferred	hcTyg4	textfile	2	TRUE	NA
rs3481147	A	G	A	G	-0.02018	0.0061	0.230728	NA	FALSE	FALSE	FALSE	NWehKT	0.0197	0.778501	interleukin-	TRUE	reported	textfile	0.003593	alcohol inta	TRUE	1.95E-08	inferred	hcTyg4	textfile	2	TRUE	NA
rs3510514	T	C	T	C	0.026345	0.0245	0.401541	NA	FALSE	FALSE	FALSE	NWehKT	0.0162	0.1334	interleukin-	TRUE	reported	textfile	0.003088	alcohol inta	TRUE	1.44E-17	inferred	hcTyg4	textfile	2	TRUE	NA
rs362307	T	C	T	C	0.043305	0.0074	0.074582	NA	FALSE	FALSE	FALSE	NWehKT	0.0332	0.8322	interleukin-	TRUE	reported	textfile	0.005802	alcohol inta	TRUE	8.42E-14	inferred	hcTyg4	textfile	2	TRUE	NA
rs4241258	T	C	T	C	0.025064	-1.00E-04	0.13763	NA	FALSE	FALSE	FALSE	NWehKT	0.0263	0.9688	interleukin-	TRUE	reported	textfile	0.004403	alcohol inta	TRUE	1.26E-08	inferred	hcTyg4	textfile	2	TRUE	NA
rs4242715	A	G	A	G	-0.01865	3.00E-04	0.680585	NA	FALSE	FALSE	FALSE	NWehKT	0.0159	0.9928	interleukin-	TRUE	reported	textfile	0.003248	alcohol inta	TRUE	9.31E-09	inferred	hcTyg4	textfile	2	TRUE	NA
rs4417025	A	G	A	G	-0.01884	-0.0241	0.361153	NA	FALSE	FALSE	FALSE	NWehKT	0.0188	0.1906	interleukin-	TRUE	reported	textfile	0.003165	alcohol inta	TRUE	2.65E-09	inferred	hcTyg4	textfile	2	TRUE	NA
rs461599	C	A	C	A	-0.01919	-0.0161	0.462259	NA	FALSE	FALSE	FALSE	NWehKT	0.0156	0.3028	interleukin-	TRUE	reported	textfile	0.00304	alcohol inta	TRUE	2.74E-10	inferred	hcTyg4	textfile	2	TRUE	NA
rs4726481	T	G	T	G	0.021761	0.0203	0.400576	NA	FALSE	FALSE	FALSE	NWehKT	0.0168	0.2287	interleukin-	TRUE	reported	textfile	0.003102	alcohol inta	TRUE	2.29E-12	inferred	hcTyg4	textfile	2	TRUE	NA
rs4800487	G	A	G	A	-0.02894	-0.0047	0.456873	NA	FALSE	FALSE	FALSE	NWehKT	0.0157	0.7583	interleukin-	TRUE	reported	textfile	0.003047	alcohol inta	TRUE	2.16E-21	inferred	hcTyg4	textfile	2	TRUE	NA
rs489062	A	G	A	G	0.01665	-0.0176	0.437454	NA	FALSE	FALSE	FALSE	NWehKT	0.0161	0.266	interleukin-	TRUE	reported	textfile	0.003053	alcohol inta	TRUE	4.93E-08	inferred	hcTyg4	textfile	2	TRUE	NA
rs4916723	C	A	C	A	0.023948	0.0055	0.420617	NA	FALSE	FALSE	FALSE	NWehKT	0.0158	0.7274	interleukin-	TRUE	reported	textfile	0.0031	alcohol inta	TRUE	1.11E-14	inferred	hcTyg4	textfile	2	TRUE	NA
rs4968391	T	G	T	G	-0.01927	-0.0032	0.674892	NA	FALSE	FALSE	FALSE	NWehKT	0.0161	0.8444	interleukin-	TRUE	reported	textfile	0.003227	alcohol inta	TRUE	2.34E-09	inferred	hcTyg4	textfile	2	TRUE	NA
rs550942	T	C	T	C	0.022401	0.006	0.823865	NA	FALSE	FALSE	FALSE	NWehKT	0.0264	0.8178	interleukin-	TRUE	reported	textfile	0.003989	alcohol inta	TRUE	1.96E-08	inferred	hcTyg4	textfile	2	TRUE	NA
rs5619443	T	C	T	C	0.02254	0.0361	0.16931	NA	FALSE	FALSE	FALSE	NWehKT	0.0241	0.1331	interleukin-	TRUE	reported	textfile	0.004071	alcohol inta	TRUE	3.09E-08	inferred	hcTyg4	textfile	2	TRUE	NA
rs5890541	A	G	A	G	-0.02663	-0.0075	0.410052	NA	FALSE	FALSE	FALSE	NWehKT	0.0158	0.6356	interleukin-	TRUE	reported	textfile	0.003078	alcohol inta	TRUE	5.07E-18	inferred	hcTyg4	textfile	2	TRUE	NA
rs6030312	T	G	T	G	-0.01953	0.0092	0.314251	NA	FALSE	FALSE	FALSE	NWehKT	0.0162	0.57	interleukin-	TRUE	reported	textfile	0.003271	alcohol inta	TRUE	2.36E-09	inferred	hcTyg4	textfile	2	TRUE	NA
rs6187351	T	G	T	G	0.020374	-0.0023	0.32785	NA	FALSE	FALSE	FALSE	NWehKT	0.0179	0.8963	interleukin-	TRUE	reported	textfile	0.003303	alcohol inta	TRUE	6.91E-10	inferred	hcTyg4	textfile	2	TRUE	NA
rs6233967	A	C	A	C	0.018294	-0.0088	0.626705	NA	FALSE	FALSE	FALSE	NWehKT	0.0169	0.6052	interleukin-	TRUE	reported	textfile	0.003154	alcohol inta	TRUE	6.62E-09	inferred	hcTyg4	textfile	2	TRUE	NA
rs6246631	T	C	T	C	-0.02549	-0.0269	0.202827	NA	FALSE	FALSE	FALSE	NWehKT	0.0208	0.1975	interleukin-	TRUE	reported	textfile	0.003774	alcohol inta	TRUE	1.44E-11	inferred	hcTyg4	textfile	2	TRUE	NA
rs650558	T	C	T	C	0.020736	-0.002	0.247918	NA	FALSE	FALSE	FALSE	NWehKT	0.0195	0.9135	interleukin-	TRUE	reported	textfile	0.003508	alcohol inta	TRUE	3.39E-09	inferred	hcTyg4	textfile	2	TRUE	NA
rs6727281	T	C	T	C	-0.02432	0.0057	0.184023	NA	FALSE	FALSE	FALSE	NWehKT	0.0179	0.751799	interleukin-	TRUE	reported	textfile	0.00392	alcohol inta	TRUE	5.46E-10	inferred	hcTyg4	textfile	2	TRUE	NA
rs6943160	C	T	C	T	0.020627	0.0286	0.208646	NA	FALSE	FALSE	FALSE	NWehKT	0.0186	0.1304	interleukin-	TRUE	reported	textfile	0.003728	alcohol inta	TRUE	3.14E-08	inferred	hcTyg4	textfile	2	TRUE	NA
rs7165168	T	C	T	C	-0.07046	-0.0944	0.0142	NA	FALSE	FALSE	FALSE	NWehKT	0.0896	0.2978	interleukin-	TRUE	reported	textfile	0.012791	alcohol inta	TRUE	3.62E-08	inferred	hcTyg4	textfile	2	TRUE	NA
rs7231987	T	G	T	G	0.019026	0.0089	0.267222	NA	FALSE	FALSE	FALSE	NWehKT	0.0198	0.657201	interleukin-	TRUE	reported	textfile	0.003427	alcohol inta	TRUE	2.82E-08	inferred	hcTyg4	textfile	2	TRUE	NA
rs7278706	A	G	A	G	-0.02819	0.0349	0.162767	NA	FALSE	FALSE	FALSE	NWehKT	0.0215	0.1052	interleukin-	TRUE	reported	textfile	0.004103	alcohol inta	TRUE	6.36E-12	inferred	hcTyg4	textfile	2	TRUE	NA
rs728538	G	T	G	T	0.022875	-0.0203	0.168868	NA	FALSE	FALSE	FALSE	NWehKT	0.0203	0.3182	interleukin-	TRUE	reported	textfile	0.004063	alcohol inta	TRUE	1.79E-08	inferred	hcTyg4	textfile	2	TRUE	NA
rs7298932	G	A	G	A	-0.02372	0.0152	0.147849	NA	FALSE	FALSE	FALSE	NWehKT	0.0242	0.5361	interleukin-	TRUE	reported	textfile	0.004312	alcohol inta	TRUE	3.76E-08	inferred	hcTyg4	textfile	2	TRUE	NA
rs7302200	A	G	A	G	-0.01842	0.0185	0.339998	NA	FALSE	FALSE	FALSE	NWehKT	0.0176	0.2938	interleukin-	TRUE	reported	textfile	0.003198	alcohol inta	TRUE	8.42E-09	inferred	hcTyg4	textfile	2	TRUE	NA
rs7305012	A	C	A	C	-0.026	-0.0263	0.164488	NA	FALSE	FALSE	FALSE	NWehKT	0.0181	0.1454	interleukin-	TRUE	reported	textfile	0.004091	alcohol inta	TRUE	2.06E-10	inferred	hcTyg4	textfile	2	TRUE	NA
rs7330939	T	C	T	C	-0.02133	-0.0228	0.720352	NA	FALSE	FALSE	FALSE	NWehKT	0.0166	0.1678	interleukin-	TRUE	reported	textfile	0.003405	alcohol inta	TRUE	3.73E-10	inferred	hcTyg4	textfile	2	TRUE	NA
rs7460106	C	T	C	T	-0.02171	-0.0148	0.23562	NA	FALSE	FALSE	FALSE	NWehKT	0.0186	0.4268	interleukin-	TRUE	reported	textfile	0.003619	alcohol inta	TRUE	1.99E-09	inferred	hcTyg4	textfile	2	TRUE	NA
rs7467914	C	T	C	T	-0.03207	-0.0465	0.074515	NA	FALSE	FALSE	FALSE	NWehKT	0.0298	0.1205	interleukin-	TRUE	reported	textfile	0.005758	alcohol inta	TRUE	2.54E-08	inferred	hcTyg4	textfile	2	TRUE	NA
rs7514579	C	A	C	A	0.019667	-0.0016	0.232457	NA	FALSE	FALSE	FALSE	NWehKT	0.0195	0.9375	interleukin-	TRUE	reported	textfile	0.003598	alcohol inta	TRUE	4.59E-08	inferred	hcTyg4	textfile	2	TRUE	NA
rs7608265	T	C	T	C	0.046427	0.029	0.054327	NA	FALSE	FALSE	FALSE	NWehKT	0.0481	0.5164	interleukin-	TRUE	reported	textfile	0.006687	alcohol inta	TRUE	3.83E-12	inferred	hcTyg4	textfile	2	TRUE	NA
rs7610856	A	C	A	C	-0.02386	0.0048	0.429053	NA	FALSE	FALSE	FALSE	NWehKT	0.0162	0.7693	interleukin-	TRUE	reported	textfile	0.00307	alcohol inta	TRUE	7.68E-15	inferred	hcTyg4	textfile	2	TRUE	NA
rs780094	C	T	C	T	-0.05099	7.00E-04	0.615206	NA	FALSE	FALSE	FALSE	NWehKT	0.0163	0.9753	interleukin-	TRUE	reported	textfile	0.003105	alcohol inta	TRUE	1.31E-60	inferred	hcTyg4	textfile	2	TRUE	NA
rs8029231	C	T	C	T	-0.03937	-0.0335	0.057704	NA	FALSE	FALSE	FALSE	NWehKT	0.0288	0.2459	interleukin-	TRUE	reported	textfile	0.006496	alcohol inta	TRUE	1.35E-09	inferred	hcTyg4	textfile	2	TRUE	NA
rs8083110	T	C	T	C	-0.01681	0.0126	0.418707	NA	FALSE	FALSE	FALSE	NWehKT	0.0156	0.4188	interleukin-	TRUE	reported	textfile	0.003078	alcohol inta	TRUE	4.72E-08	inferred	hcTyg4	textfile	2	TRUE	NA
rs838145	A	G	A	G	0.021955	-0.0036	0.542982	NA	FALSE	FALSE	FALSE	NWehKT	0.0166	0.8217	interleukin-	TRUE	reported	textfile	0.003055	alcohol inta	TRUE	6.70E-13	inferred	hcTyg4	textfile	2	TRUE	NA
rs8614	A	C	A	C	0.024781	-0.0045	0.182509	NA	FALSE	FALSE	FALSE	NWehKT	0.0225	0.8473	interleukin-	TRUE	reported	textfile	0.003925	alcohol inta	TRUE	2.74E-10	inferred	hcTyg4	textfile	2	TRUE	NA
rs9349379	G	A	G	A	-0.01935	-0.0029	0.405493	NA	FALSE	FALSE	FALSE	NWehKT	0.0159	0.862	interleukin-	TRUE	reported	textfile	0.003082	alcohol inta	TRUE	3.46E-10	inferred	hcTyg4	textfile	2	TRUE	NA
rs9372625	A	G	A	G	-0.02556	-0.002	0.381706	NA	FALSE	FALSE	FALSE	NWehKT	0.0168	0.907	interleukin-	TRUE	reported	textfile	0.003125	alcohol inta	TRUE	2.85E-16	inferred	hcTyg4	textfile	2	TRUE	NA
rs9403297	A	G	A	G	0.018823	0.0256	0.372967	NA	FALSE	FALSE	FALSE	NWehKT	0.0173	0.1416	interleukin-	TRUE	reported	textfile	0.00313	alcohol inta	TRUE	1.82E-09	inferred	hcTyg4	textfile	2	TRUE	NA
rs9648478	A	G	A	G	0.01686	-0.0139	0.510245	NA	FALSE	FALSE	FALSE	NWehKT	0.0156	0.3768	interleukin-	TRUE	reported	textfile	0.003029	alcohol inta	TRUE	2.60E-08	inferred	hcTyg4	textfile	2	TRUE	NA
rs9814516	T	G	T	G	-0.02511	0.0144	0.237423	NA	FALSE	FALSE	FALSE	NWehKT	0.0188	0.4582	interleukin-	TRUE	reported	textfile	0.003556	alcohol inta	TRUE	1.64E-12	inferred	hcTyg4	textfile	2	TRUE	NA
rs9829192	T	G	T	G	0.016932	-0.012	0.435133	NA	FALSE	FALSE	FALSE	NWehKT	0.0158	0.4614	interleukin-	TRUE	reported	textfile	0.00305	alcohol inta	TRUE	2.84E-08	inferred	hcTyg4	textfile	2	TRUE	NA
rs9891943	C	T	C	T	0.019867	0.0177	0.232109	NA	FALSE	FALSE	FALSE	NWehKT	0.0219	0.4243	interleukin-	TRUE	reported	textfile	0.003641	alcohol inta	TRUE	4.88E-08	inferred	hcTyg4	textfile	2	TRUE	NA
rs9906502	A	G	A	G	0.023788	-0.0025	0.176998	NA	FALSE	FALSE	FALSE	NWehKT	0.02	0.8884	interleukin-	TRUE	reported	textfile	0.003962	alcohol inta	TRUE	1.92E-09	inferred	hcTyg4	textfile	2	TRUE	NA

SNPs of alcohol intake frequency on minnitol

SNP	effect_allel	other_allel	effect_allel	other_allel	beta.expos	beta.outco	eaf.exposu	eaf.outcom	remove	palindromi	ambiguous	id.outcome	se.outcome	pval.outco	outcome	mr_keep.ou	pval_origin	data_sourc	se.exposure	exposure	mr_keep.ex	pval.expos	pval_origin	id.exposure	data_sourc	action	mr_keep	samplesize.outcome
rs1018831	T	C	T	C	-0.01979	0.005	0.470852	0.4701	FALSE	FALSE	FALSE	ckGzPF	0.0096	0.6027	Minnitol	TRUE	reported	textfile	0.003036	alcohol inta	TRUE	7.16E-11	inferred	AfPWZt	textfile	2	TRUE	NA
rs1079266	G	A	G	A	0.017432	-0.0087	0.505254	0.502	FALSE	FALSE	FALSE	ckGzPF	0.009	0.334	Minnitol	TRUE	reported	textfile	0.003041	alcohol inta	TRUE	9.86E-09	inferred	AfPWZt	textfile	2	TRUE	NA
rs1122361	A	G	A	G	0.025091	0.0039	0.206155	0.2095	FALSE	FALSE	FALSE	ckGzPF	0.0111	0.726901	Minnitol	TRUE	reported	textfile	0.003754	alcohol inta	TRUE	2.32E-11	inferred	AfPWZt	textfile	2	TRUE	NA
rs1170085	G	A	G	A	-0.0298	0.0156	0.093465	0.0918	FALSE	FALSE	FALSE	ckGzPF	0.0157	0.3208	Minnitol	TRUE	reported	textfile	0.005233	alcohol inta	TRUE	1.24E-08	inferred	AfPWZt	textfile	2	TRUE	NA
rs1175077	A	G	A	G	-0.02049	-0.0172	0.209454	0.1965	FALSE	FALSE	FALSE	ckGzPF	0.0114	0.132	Minnitol	TRUE	reported	textfile	0.003726	alcohol inta	TRUE	3.80E-08	inferred	AfPWZt	textfile	2	TRUE	NA
rs1178721	T	C	T	C	0.024416	-0.0175	0.369127	0.3654	FALSE	FALSE	FALSE	ckGzPF	0.01	0.07972	Minnitol	TRUE	reported	textfile	0.003201	alcohol inta	TRUE	2.38E-14	inferred	AfPWZt	textfile	2	TRUE	NA
rs1194069	G	A	G	A	-0.04371	-0.0096	0.604193	0.5791	FALSE	FALSE	FALSE	ckGzPF	0.0099	0.3291	Minnitol	TRUE	reported	textfile	0.003116	alcohol inta	TRUE	1.04E-44	inferred	AfPWZt	textfile	2	TRUE	NA
rs1215385	C	T	C	T	0.029444	0.0277	0.10497	0.1022	FALSE	FALSE	FALSE	ckGzPF	0.0154	0.07152	Minnitol	TRUE	reported	textfile	0.004935	alcohol inta	TRUE	2.42E-09	inferred	AfPWZt	textfile	2	TRUE	NA
rs1228589	A	G	A	G	0.02107	0.0167	0.246133	0.2369	FALSE	FALSE	FALSE	ckGzPF	0.0106	0.1143	Minnitol	TRUE	reported	textfile	0.003528	alcohol inta	TRUE	2.34E-09	inferred	AfPWZt	textfile	2	TRUE	NA
rs1310297	C	T	C	T	-0.01941	-0.0017	0.61881	0.6431	FALSE	FALSE	FALSE	ckGzPF	0.0095	0.8553	Minnitol	TRUE	reported	textfile	0.003119	alcohol inta	TRUE	4.89E-10	inferred	AfPWZt	textfile	2	TRUE	NA
rs1421085	C	T	C	T	0.019939	-0.0044	0.403447	0.4305	FALSE	FALSE	FALSE	ckGzPF	0.0155	0.777	Minnitol	TRUE	reported	textfile	0.003085	alcohol inta	TRUE	1.02E-10	inferred	AfPWZt	textfile	2	TRUE	NA
rs1766275	C	T	C	T	0.030135	-0.0195	0.089115	0.0736	FALSE	FALSE	FALSE	ckGzPF	0.026	0.4526	Minnitol	TRUE	reported	textfile	0.00546	alcohol inta	TRUE	3.41E-08	inferred	AfPWZt	textfile	2	TRUE	NA
rs186347	T	G	T	G	0.017949	0.0077	0.463343	0.473	FALSE	FALSE	FALSE	ckGzPF	0.0094	0.4162	Minnitol	TRUE	reported	textfile	0.003051	alcohol inta	TRUE	4.02E-09	inferred	AfPWZt	textfile	2	TRUE	NA
rs1937522	G	A	G	A	0.016898	-0.0139	0.528054	0.5201	FALSE	FALSE	FALSE	ckGzPF	0.0089	0.1191	Minnitol	TRUE	reported	textfile	0.003032	alcohol inta	TRUE	2.50E-08	inferred	AfPWZt	textfile	2	TRUE	NA
rs1991083	T	C	T	C	-0.02239	-0.0047	0.679886	0.7079	FALSE	FALSE	FALSE	ckGzPF	0.0102	0.6487	Minnitol	TRUE	reported	textfile	0.003258	alcohol inta	TRUE	6.30E-12	inferred	AfPWZt	textfile	2	TRUE	NA
rs2159935	A	G	A	G	-0.01857	-0.0057	0.490369	0.4761	FALSE	FALSE	FALSE	ckGzPF	0.0094	0.544	Minnitol	TRUE	reported	textfile	0.003026	alcohol inta	TRUE	8.33E-10	inferred	AfPWZt	textfile	2	TRUE	NA
rs2160935	T	C	T	C	-0.01872	1.00E-04	0.604293	0.6031	FALSE	FALSE	FALSE	ckGzPF	0.009	0.9954	Minnitol	TRUE	reported	textfile	0.003091	alcohol inta	TRUE	1.40E-09	inferred	AfPWZt	textfile	2	TRUE	NA
rs2411453	G	T	G	T	-0.03508	0.0061	0.597353	0.5829	FALSE	FALSE	FALSE	ckGzPF	0.0098	0.533499	Minnitol	TRUE	reported	textfile	0.00309	alcohol inta	TRUE	7.32E-30	inferred	AfPWZt	textfile	2	TRUE	NA
rs2535911	T	C	T	C	-0.01885	-0.0031	0.354749	0.3463	FALSE	FALSE	FALSE	ckGzPF	0.0095	0.7449	Minnitol	TRUE	reported	textfile	0.003168	alcohol inta	TRUE	2.71E-09	inferred	AfPWZt	textfile	2	TRUE	NA
rs2717063	A	C	A	C	-0.02037	0.0059	0.585731	0.5789	FALSE	FALSE	FALSE	ckGzPF	0.0095	0.5385	Minnitol	TRUE	reported	textfile	0.003085	alcohol inta	TRUE	4.00E-11	inferred	AfPWZt	textfile	2	TRUE	NA
rs4241258	T	C	T	C	0.025064	0.0042	0.13763	0.13	FALSE	FALSE	FALSE	ckGzPF	0.0133	0.751	Minnitol	TRUE	reported	textfile	0.004403	alcohol inta	TRUE	1.26E-08	inferred	AfPWZt	textfile	2	TRUE	NA
rs4417025	A	G	A	G	-0.01884	-0.02	0.361153	0.331	FALSE	FALSE	FALSE	ckGzPF	0.012	0.095581	Minnitol	TRUE	reported	textfile	0.003165	alcohol inta	TRUE	2.65E-09	inferred	AfPWZt	textfile	2	TRUE	NA
rs461599	C	A	C	A	-0.01919	0.013	0.462259	0.4701	FALSE	FALSE	FALSE	ckGzPF	0.0089	0.1434	Minnitol	TRUE	reported	textfile	0.00304	alcohol inta	TRUE	2.74E-10	inferred	AfPWZt	textfile	2	TRUE	NA
rs489062	A	G	A	G	0.01665	-0.0087	0.437454	0.4425	FALSE	FALSE	FALSE	ckGzPF	0.0089	0.332	Minnitol	TRUE	reported	textfile	0.003053	alcohol inta	TRUE	4.93E-08	inferred	AfPWZt	textfile	2	TRUE	NA
rs4916723	C	A	C	A	0.023948	-0.0153	0.420617	0.4113	FALSE	FALSE	FALSE	ckGzPF	0.0095	0.1071	Minnitol	TRUE	reported	textfile	0.0031	alcohol inta	TRUE	1.11E-14	inferred	AfPWZt	textfile	2	TRUE	NA
rs5022348	T	C	T	C	0.020264	0.0207	0.40703	0.5673	FALSE	FALSE	FALSE	ckGzPF	0.009	0.02107	Minnitol	TRUE	reported	textfile	0.00357	alcohol inta	TRUE	1.38E-08	inferred	AfPWZt	textfile	2	TRUE	NA
rs550942	T	C	T	C	0.022401	-0.0148	0.823865	0.8245	FALSE	FALSE	FALSE	ckGzPF	0.0116	0.2007	Minnitol	TRUE	reported	textfile	0.003989	alcohol inta	TRUE	1.96E-08	inferred	AfPWZt	textfile	2	TRUE	NA
rs6727281	T	C	T	C	-0.02432	0.0165	0.184023	0.1723	FALSE	FALSE	FALSE	ckGzPF	0.0124	0.1837	Minnitol	TRUE	reported	textfile	0.00392	alcohol inta	TRUE	5.46E-10	inferred	AfPWZt	textfile	2	TRUE	NA
rs7610856	A	C	A	C	-0.02386	3.00E-04	0.429053	0.4224	FALSE	FALSE	FALSE	ckGzPF	0.0095	0.9756	Minnitol	TRUE	reported	textfile	0.00307	alcohol inta	TRUE	7.68E-15	inferred	AfPWZt	textfile	2	TRUE	NA
rs780094	C	T	C	T	-0.05099	0.0077	0.615206	0.6039	FALSE	FALSE	FALSE	ckGzPF	0.0089	0.3872	Minnitol	TRUE	reported	textfile	0.003105	alcohol inta	TRUE	1.31E-60	inferred	AfPWZt	textfile	2	TRUE	NA
rs780569	A	T	A	T	0.019803	-0.0122	0.70882	0.7323	FALSE	TRUE	FALSE	ckGzPF	0.0104	0.2378	Minnitol	TRUE	reported	textfile	0.003365	alcohol inta	TRUE	3.96E-09	inferred	AfPWZt	textfile	2	TRUE	NA
rs838145	A	G	A	G	0.021955	-0.0217	0.542982	0.5676	FALSE	FALSE	FALSE	ckGzPF	0.0097	0.02516	Minnitol	TRUE	reported	textfile	0.003055	alcohol inta	TRUE	6.70E-13	inferred	AfPWZt	textfile	2	TRUE	NA
rs8614	A	C	A	C	0.024781	-0.0204	0.182509	0.1812	FALSE	FALSE	FALSE	ckGzPF	0.0122	0.09489	Minnitol	TRUE	reported	textfile	0.003925	alcohol inta	TRUE	2.74E-10	inferred	AfPWZt	textfile	2	TRUE	NA
rs9349379	G	A	G	A	-0.01935	-0.0065	0.405493	0.402	FALSE	FALSE	FALSE	ckGzPF	0.0096	0.4999	Minnitol	TRUE	reported	textfile	0.003082	alcohol inta	TRUE	3.46E-10	inferred	AfPWZt	textfile	2	TRUE	NA
rs9372625	A	G	A	G	-0.02556	-0.0085	0.381706	0.3727	FALSE	FALSE	FALSE	ckGzPF	0.0095	0.3734	Minnitol	TRUE	reported	textfile	0.003125	alcohol inta	TRUE	2.85E-16	inferred	AfPWZt	textfile	2	TRUE	NA
rs9814516	T	G	T	G	-0.02511	0.0131	0.237423	0.2341	FALSE	FALSE	FALSE	ckGzPF	0.0106	0.2156	Minnitol	TRUE	reported	textfile	0.003556	alcohol inta	TRUE	1.64E-12	inferred	AfPWZt	textfile	2	TRUE	NA

SNPs of alcohol intake frequency on Vitamin A

SNP	effect_allel	other_allel	effect_allel	other_allel	beta.expos	beta.outco	eaf.expos	eaf.outcon	remove	palindrom	ambiguou	id.outcome	se.outcom	pval.outco	outcome	mr_keep.o	pval_origir	data_sourc	se.exposur	exposure	mr_keep.e	pval.expos	pval_origir	id.exposur	data_sourc	action	mr_keep	samplesize.outcome
rs1004787	A	G	A	G	-0.02223	-0.00017	0.532943	0.532921	FALSE	FALSE	FALSE	6wQelu	0.000289	0.56	Vitamin A	TRUE	reported	textfile	0.003045	alcohol int	TRUE	2.90E-13	inferred	jK8tw0	textfile	2	TRUE	NA
rs1018831	T	C	T	C	-0.01979	0.000386	0.470852	0.470835	FALSE	FALSE	FALSE	6wQelu	0.000288	0.18	Vitamin A	TRUE	reported	textfile	0.003036	alcohol int	TRUE	7.16E-11	inferred	jK8tw0	textfile	2	TRUE	NA
rs1079266	G	A	G	A	0.017432	#######	0.505254	0.505203	FALSE	FALSE	FALSE	6wQelu	0.000288	0.93	Vitamin A	TRUE	reported	textfile	0.003041	alcohol int	TRUE	9.86E-09	inferred	jK8tw0	textfile	2	TRUE	NA
rs1103942	T	C	T	C	-0.02356	4.49E-05	0.454624	0.454576	FALSE	FALSE	FALSE	6wQelu	0.000288	0.88	Vitamin A	TRUE	reported	textfile	0.003037	alcohol int	TRUE	8.73E-15	inferred	jK8tw0	textfile	2	TRUE	NA
rs1122361	A	G	A	G	0.025091	#######	0.206155	0.206161	FALSE	FALSE	FALSE	6wQelu	0.000355	0.99	Vitamin A	TRUE	reported	textfile	0.003754	alcohol int	TRUE	2.32E-11	inferred	jK8tw0	textfile	2	TRUE	NA
rs1170085	G	A	G	A	-0.0298	-0.00016	0.093465	0.093509	FALSE	FALSE	FALSE	6wQelu	0.000495	0.75	Vitamin A	TRUE	reported	textfile	0.005233	alcohol int	TRUE	1.24E-08	inferred	jK8tw0	textfile	2	TRUE	NA
rs1175077	A	G	A	G	-0.02049	#######	0.209454	0.209469	FALSE	FALSE	FALSE	6wQelu	0.000353	0.89	Vitamin A	TRUE	reported	textfile	0.003726	alcohol int	TRUE	3.80E-08	inferred	jK8tw0	textfile	2	TRUE	NA
rs1177994	C	G	C	G	-0.01967	-0.00019	0.336989	0.337032	FALSE	TRUE	FALSE	6wQelu	0.000314	0.54	Vitamin A	TRUE	reported	textfile	0.00332	alcohol int	TRUE	3.12E-09	inferred	jK8tw0	textfile	2	TRUE	NA
rs1178721	T	C	T	C	0.024416	4.16E-05	0.369127	0.369037	FALSE	FALSE	FALSE	6wQelu	0.000303	0.89	Vitamin A	TRUE	reported	textfile	0.003201	alcohol int	TRUE	2.38E-14	inferred	jK8tw0	textfile	2	TRUE	NA
rs1194069	G	A	G	A	-0.04371	-0.00014	0.604193	0.60422	FALSE	FALSE	FALSE	6wQelu	0.000295	0.630001	Vitamin A	TRUE	reported	textfile	0.003116	alcohol int	TRUE	1.04E-44	inferred	jK8tw0	textfile	2	TRUE	NA
rs1215385	C	T	C	T	0.029444	-0.00023	0.10497	0.10497	FALSE	FALSE	FALSE	6wQelu	0.000467	0.630001	Vitamin A	TRUE	reported	textfile	0.004935	alcohol int	TRUE	2.42E-09	inferred	jK8tw0	textfile	2	TRUE	NA
rs1228589	A	G	A	G	0.02107	0.000719	0.246133	0.246104	FALSE	FALSE	FALSE	6wQelu	0.000334	0.031	Vitamin A	TRUE	reported	textfile	0.003528	alcohol int	TRUE	2.34E-09	inferred	jK8tw0	textfile	2	TRUE	NA
rs1310297	C	T	C	T	-0.01941	7.51E-05	0.61881	0.618812	FALSE	FALSE	FALSE	6wQelu	0.000295	0.8	Vitamin A	TRUE	reported	textfile	0.003119	alcohol int	TRUE	4.89E-10	inferred	jK8tw0	textfile	2	TRUE	NA
rs1313509	G	A	G	A	0.043834	0.001091	0.083483	0.083393	FALSE	FALSE	FALSE	6wQelu	0.000521	0.036	Vitamin A	TRUE	reported	textfile	0.005499	alcohol int	TRUE	1.57E-15	inferred	jK8tw0	textfile	2	TRUE	NA
rs1317844	T	C	T	C	-0.01865	0.000208	0.276349	0.276375	FALSE	FALSE	FALSE	6wQelu	0.000321	0.52	Vitamin A	TRUE	reported	textfile	0.00339	alcohol int	TRUE	3.75E-08	inferred	jK8tw0	textfile	2	TRUE	NA
rs1339001	C	T	C	T	0.029612	-0.00063	0.134041	0.133964	FALSE	FALSE	FALSE	6wQelu	0.000426	0.14	Vitamin A	TRUE	reported	textfile	0.004492	alcohol int	TRUE	4.33E-11	inferred	jK8tw0	textfile	2	TRUE	NA
rs1421085	C	T	C	T	0.019939	-0.00017	0.403447	0.403469	FALSE	FALSE	FALSE	6wQelu	0.000292	0.55	Vitamin A	TRUE	reported	textfile	0.003085	alcohol int	TRUE	1.02E-10	inferred	jK8tw0	textfile	2	TRUE	NA
rs1515590	T	C	T	C	0.018245	-0.00059	0.383245	0.383196	FALSE	FALSE	FALSE	6wQelu	0.000295	0.045	Vitamin A	TRUE	reported	textfile	0.003116	alcohol int	TRUE	4.78E-09	inferred	jK8tw0	textfile	2	TRUE	NA
rs1666658	C	T	C	T	0.017967	#######	0.392206	0.39221	FALSE	FALSE	FALSE	6wQelu	0.000293	0.780001	Vitamin A	TRUE	reported	textfile	0.003099	alcohol int	TRUE	6.69E-09	inferred	jK8tw0	textfile	2	TRUE	NA
rs1766275	C	T	C	T	0.030135	-0.00022	0.089115	0.089126	FALSE	FALSE	FALSE	6wQelu	0.000517	0.67	Vitamin A	TRUE	reported	textfile	0.00546	alcohol int	TRUE	3.41E-08	inferred	jK8tw0	textfile	2	TRUE	NA
rs186347	T	G	T	G	0.017949	-0.00047	0.463343	0.463308	FALSE	FALSE	FALSE	6wQelu	0.000289	0.11	Vitamin A	TRUE	reported	textfile	0.003051	alcohol int	TRUE	4.02E-09	inferred	jK8tw0	textfile	2	TRUE	NA
rs1937522	G	A	G	A	0.016898	-0.00032	0.528054	0.528027	FALSE	FALSE	FALSE	6wQelu	0.000287	0.27	Vitamin A	TRUE	reported	textfile	0.003032	alcohol int	TRUE	2.50E-08	inferred	jK8tw0	textfile	2	TRUE	NA
rs1991083	T	C	T	C	-0.02239	0.000213	0.679886	0.679912	FALSE	FALSE	FALSE	6wQelu	0.000309	0.49	Vitamin A	TRUE	reported	textfile	0.003258	alcohol int	TRUE	6.30E-12	inferred	jK8tw0	textfile	2	TRUE	NA
rs2043677	T	C	T	C	0.026113	0.00054	0.145599	0.145616	FALSE	FALSE	FALSE	6wQelu	0.000409	0.19	Vitamin A	TRUE	reported	textfile	0.004327	alcohol int	TRUE	1.59E-09	inferred	jK8tw0	textfile	2	TRUE	NA
rs2159935	A	G	A	G	-0.01857	-0.00013	0.490369	0.490341	FALSE	FALSE	FALSE	6wQelu	0.000287	0.66	Vitamin A	TRUE	reported	textfile	0.003026	alcohol int	TRUE	8.33E-10	inferred	jK8tw0	textfile	2	TRUE	NA
rs2160935	T	C	T	C	-0.01872	-0.00012	0.604293	0.604314	FALSE	FALSE	FALSE	6wQelu	0.000293	0.68	Vitamin A	TRUE	reported	textfile	0.003091	alcohol int	TRUE	1.40E-09	inferred	jK8tw0	textfile	2	TRUE	NA
rs2244598	C	T	C	T	-0.01838	0.000202	0.605114	0.605094	FALSE	FALSE	FALSE	6wQelu	0.000295	0.49	Vitamin A	TRUE	reported	textfile	0.003119	alcohol int	TRUE	3.81E-09	inferred	jK8tw0	textfile	2	TRUE	NA
rs2411453	G	T	G	T	-0.03508	0.000118	0.597353	0.597338	FALSE	FALSE	FALSE	6wQelu	0.000293	0.69	Vitamin A	TRUE	reported	textfile	0.00309	alcohol int	TRUE	7.32E-30	inferred	jK8tw0	textfile	2	TRUE	NA
rs2535911	T	C	T	C	-0.01885	#######	0.354749	0.354794	FALSE	FALSE	FALSE	6wQelu	0.0003	0.75	Vitamin A	TRUE	reported	textfile	0.003168	alcohol int	TRUE	2.71E-09	inferred	jK8tw0	textfile	2	TRUE	NA
rs2622167	A	G	A	G	-0.01912	-0.0005	0.428653	0.428668	FALSE	FALSE	FALSE	6wQelu	0.00029	0.083	Vitamin A	TRUE	reported	textfile	0.003067	alcohol int	TRUE	4.61E-10	inferred	jK8tw0	textfile	2	TRUE	NA
rs262240	T	C	T	C	-0.01721	-0.00037	0.468553	0.468499	FALSE	FALSE	FALSE	6wQelu	0.000287	0.2	Vitamin A	TRUE	reported	textfile	0.003035	alcohol int	TRUE	1.43E-08	inferred	jK8tw0	textfile	2	TRUE	NA
rs2717063	A	C	A	C	-0.02037	-0.00028	0.585731	0.585679	FALSE	FALSE	FALSE	6wQelu	0.000292	0.34	Vitamin A	TRUE	reported	textfile	0.003085	alcohol int	TRUE	4.00E-11	inferred	jK8tw0	textfile	2	TRUE	NA
rs2876812	C	T	C	T	0.0207	0.000344	0.759525	0.759492	FALSE	FALSE	FALSE	6wQelu	0.000336	0.31	Vitamin A	TRUE	reported	textfile	0.003552	alcohol int	TRUE	5.62E-09	inferred	jK8tw0	textfile	2	TRUE	NA
rs2878710	A	G	A	G	0.017811	#######	0.40423	0.404228	FALSE	FALSE	FALSE	6wQelu	0.000292	0.81	Vitamin A	TRUE	reported	textfile	0.003085	alcohol int	TRUE	7.74E-09	inferred	jK8tw0	textfile	2	TRUE	NA
rs324012	T	C	T	C	-0.01779	-0.00037	0.448569	0.448564	FALSE	FALSE	FALSE	6wQelu	0.000288	0.19	Vitamin A	TRUE	reported	textfile	0.003044	alcohol int	TRUE	5.03E-09	inferred	jK8tw0	textfile	2	TRUE	NA
rs3444085	T	C	T	C	-0.02268	-0.00023	0.157151	0.157162	FALSE	FALSE	FALSE	6wQelu	0.000393	0.57	Vitamin A	TRUE	reported	textfile	0.004151	alcohol int	TRUE	4.63E-08	inferred	jK8tw0	textfile	2	TRUE	NA
rs3447388	A	G	A	G	-0.02036	#######	0.24819	0.248166	FALSE	FALSE	FALSE	6wQelu	0.000332	0.84	Vitamin A	TRUE	reported	textfile	0.003503	alcohol int	TRUE	6.18E-09	inferred	jK8tw0	textfile	2	TRUE	NA
rs3463102	T	C	T	C	-0.01691	-0.00018	0.446061	0.446078	FALSE	FALSE	FALSE	6wQelu	0.000289	0.54	Vitamin A	TRUE	reported	textfile	0.003048	alcohol int	TRUE	2.89E-08	inferred	jK8tw0	textfile	2	TRUE	NA
rs3481147	A	G	A	G	-0.02018	-0.00059	0.230728	0.230749	FALSE	FALSE	FALSE	6wQelu	0.00034	0.083	Vitamin A	TRUE	reported	textfile	0.003593	alcohol int	TRUE	1.95E-08	inferred	jK8tw0	textfile	2	TRUE	NA
rs3510514	T	C	T	C	0.026345	#######	0.401541	0.401648	FALSE	FALSE	FALSE	6wQelu	0.000292	0.92	Vitamin A	TRUE	reported	textfile	0.003088	alcohol int	TRUE	1.44E-17	inferred	jK8tw0	textfile	2	TRUE	NA
rs362307	T	C	T	C	0.043305	0.00056	0.074582	0.074569	FALSE	FALSE	FALSE	6wQelu	0.000549	0.31	Vitamin A	TRUE	reported	textfile	0.005802	alcohol int	TRUE	8.42E-14	inferred	jK8tw0	textfile	2	TRUE	NA
rs4241258	T	C	T	C	0.025064	0.000692	0.13763	0.137657	FALSE	FALSE	FALSE	6wQelu	0.000417	0.097	Vitamin A	TRUE	reported	textfile	0.004403	alcohol int	TRUE	1.26E-08	inferred	jK8tw0	textfile	2	TRUE	NA
rs4242715	A	G	A	G	-0.01865	0.000313	0.680585	0.680557	FALSE	FALSE	FALSE	6wQelu	0.000307	0.31	Vitamin A	TRUE	reported	textfile	0.003248	alcohol int	TRUE	9.31E-09	inferred	jK8tw0	textfile	2	TRUE	NA
rs4417025	A	G	A	G	-0.01884	-0.00011	0.361153	0.361139	FALSE	FALSE	FALSE	6wQelu	0.0003	0.709999	Vitamin A	TRUE	reported	textfile	0.003165	alcohol int	TRUE	2.65E-09	inferred	jK8tw0	textfile	2	TRUE	NA
rs4503294	T	C	T	C	0.018148	#######	0.565333	0.565363	FALSE	FALSE	FALSE	6wQelu	0.000291	0.95	Vitamin A	TRUE	reported	textfile	0.00307	alcohol int	TRUE	3.41E-09	inferred	jK8tw0	textfile	2	TRUE	NA
rs461599	C	A	C	A	-0.01919	0.00034	0.462259	0.462292	FALSE	FALSE	FALSE	6wQelu	0.000288	0.24	Vitamin A	TRUE	reported	textfile	0.00304	alcohol int	TRUE	2.74E-10	inferred	jK8tw0	textfile	2	TRUE	NA
rs4726481	T	G	T	G	0.021761	-0.0001	0.400576	0.400547	FALSE	FALSE	FALSE	6wQelu	0.000294	0.73	Vitamin A	TRUE	reported	textfile	0.003102	alcohol int	TRUE	2.29E-12	inferred	jK8tw0	textfile	2	TRUE	NA
rs4800487	G	A	G	A	-0.02894	0.000407	0.456873	0.456998	FALSE	FALSE	FALSE	6wQelu	0.000288	0.16	Vitamin A	TRUE	reported	textfile	0.003047	alcohol int	TRUE	2.16E-21	inferred	jK8tw0	textfile	2	TRUE	NA
rs489062	A	G	A	G	0.01665	#######	0.437454	0.437477	FALSE	FALSE	FALSE	6wQelu	0.000289	0.87	Vitamin A	TRUE	reported	textfile	0.003053	alcohol int	TRUE	4.93E-08	inferred	jK8tw0	textfile	2	TRUE	NA
rs4916723	C	A	C	A	0.023948	0.000298	0.420617	0.420583	FALSE	FALSE	FALSE	6wQelu	0.000293	0.31	Vitamin A	TRUE	reported	textfile	0.0031	alcohol int	TRUE	1.11E-14	inferred	jK8tw0	textfile	2	TRUE	NA
rs4968391	T	G	T	G	-0.01927	0.000523	0.674892	0.674943	FALSE	FALSE	FALSE	6wQelu	0.000305	0.087	Vitamin A	TRUE	reported	textfile	0.003227	alcohol int	TRUE	2.34E-09	inferred	jK8tw0	textfile	2	TRUE	NA
rs5022348	T	C	T	C	0.020264	0.000379	0.40703	0.406989	FALSE	FALSE	FALSE	6wQelu	0.000338	0.26	Vitamin A	TRUE	reported	textfile	0.00357	alcohol int	TRUE	1.38E-08	inferred	jK8tw0	textfile	2	TRUE	NA
rs550942	T	C	T	C	0.022401	0.000362	0.823865	0.823831	FALSE	FALSE	FALSE	6wQelu	0.000378	0.34	Vitamin A	TRUE	reported	textfile	0.003989	alcohol int	TRUE	1.96E-08	inferred	jK8tw0	textfile	2	TRUE	NA
rs5619443	T	C	T	C	0.02254	8.74E-05	0.16931	0.16922	FALSE	FALSE	FALSE	6wQelu	0.000386	0.82	Vitamin A	TRUE	reported	textfile	0.004071	alcohol int	TRUE	3.09E-08	inferred	jK8tw0	textfile	2	TRUE	NA
rs5890541	A	G	A	G	-0.02663	-0.00085	0.410052	0.410096	FALSE	FALSE	FALSE	6wQelu	0.000291	0.0037	Vitamin A	TRUE	reported	textfile	0.003078	alcohol int	TRUE	5.07E-18	inferred	jK8tw0	textfile	2	TRUE	NA
rs6030312	T	G	T	G	-0.01953	3.15E-05	0.314251	0.314262	FALSE	FALSE	FALSE	6wQelu	0.00031	0.92	Vitamin A	TRUE	reported	textfile	0.003271	alcohol int	TRUE	2.36E-09	inferred	jK8tw0	textfile	2	TRUE	NA
rs6187351	T	G	T	G	0.020374	#######	0.32785	0.327829	FALSE	FALSE	FALSE	6wQelu	0.000313	0.77	Vitamin A	TRUE	reported	textfile	0.003303	alcohol int	TRUE	6.91E-10	inferred	jK8tw0	textfile	2	TRUE	NA
rs6230578	G	C	G	C	-0.04852	-0.00058	0.102253	0.102263	FALSE	TRUE	FALSE	6wQelu	0.00048	0.22	Vitamin A	TRUE	reported	textfile	0.005066	alcohol int	TRUE	9.88E-22	inferred	jK8tw0	textfile	2	TRUE	NA
rs6233967	A	C	A	C	0.018294	0.000184	0.626705	0.626711	FALSE	FALSE	FALSE	6wQelu	0.000299	0.54	Vitamin A	TRUE	reported	textfile	0.003154	alcohol int	TRUE	6.62E-09	inferred	jK8tw0	textfile	2	TRUE	NA
rs6246631	T	C	T	C	-0.02549	-0.00032	0.202827	0.202856	FALSE	FALSE	FALSE	6wQelu	0.000357	0.38	Vitamin A	TRUE	reported	textfile	0.003774	alcohol int	TRUE	1.44E-11	inferred	jK8tw0	textfile	2	TRUE	NA
rs650558	T	C	T	C	0.020736	-0.00023	0.247918	0.247941	FALSE	FALSE	FALSE	6wQelu	0.000332	0.49	Vitamin A	TRUE	reported	textfile	0.003508	alcohol int	TRUE	3.39E-09	inferred	jK8tw0	textfile	2	TRUE	NA
rs6727281	T	C	T	C	-0.02432	0.000291	0.184023	0.184045	FALSE	FALSE	FALSE	6wQelu	0.000371	0.43	Vitamin A	TRUE	reported	textfile	0.00392	alcohol int	TRUE	5.46E-10	inferred	jK8tw0	textfile	2	TRUE	NA
rs6943160	C	T	C	T	0.020627	0.000214	0.208646	0.208631	FALSE	FALSE	FALSE	6wQelu	0.000353	0.54	Vitamin A	TRUE	reported	textfile	0.003728	alcohol int	TRUE	3.14E-08	inferred	jK8tw0	textfile	2	TRUE	NA
rs7231987	T	G	T	G	0.019026	-0.00013	0.267222	0.267211	FALSE	FALSE	FALSE	6wQelu	0.000324	0.69	Vitamin A	TRUE	reported	textfile	0.003427	alcohol int	TRUE	2.82E-08	inferred	jK8tw0	textfile	2	TRUE	NA
rs7276922	T	A	T	A	-0.02314	-0.00092	0.154942	0.154967	FALSE	TRUE	FALSE	6wQelu	0.000397	0.021	Vitamin A	TRUE	reported	textfile	0.004192	alcohol int	TRUE	3.40E-08	inferred	jK8tw0	textfile	2	TRUE	NA
rs7278706	A	G	A	G	-0.02819	0.00048	0.162767	0.16279	FALSE	FALSE	FALSE	6wQelu	0.000388	0.22	Vitamin A	TRUE	reported	textfile	0.004103	alcohol int	TRUE	6.36E-12	inferred	jK8tw0	textfile	2	TRUE	NA
rs728538	G	T	G	T	0.022875	0.00063	0.168868	0.168852	FALSE	FALSE	FALSE	6wQelu	0.000385	0.1	Vitamin A	TRUE	reported	textfile	0.004063	alcohol int	TRUE	1.79E-08	inferred	jK8tw0	textfile	2	TRUE	NA
rs7298932	G	A	G	A	-0.02372	0.000616	0.147849	0.147832	FALSE	FALSE	FALSE	6wQelu	0.000408	0.13	Vitamin A	TRUE	reported	textfile	0.004312	alcohol int	TRUE	3.76E-08	inferred	jK8tw0	textfile	2	TRUE	NA
rs7302200	A	G	A	G	-0.01842	3.91E-05	0.339998	0.340046	FALSE	FALSE	FALSE	6wQelu	0.000303	0.9	Vitamin A	TRUE	reported	textfile	0.003198	alcohol int	TRUE	8.42E-09	inferred	jK8tw0	textfile	2	TRUE	NA
rs7305012	A	C	A	C	-0.026	0.000874	0.164488	0.16443	FALSE	FALSE	FALSE	6wQelu	0.000387	0.024	Vitamin A	TRUE	reported	textfile	0.004091	alcohol int	TRUE	2.06E-10	inferred	jK8tw0	textfile	2	TRUE	NA
rs7330939	T	C	T	C	-0.02133	-0.00024	0.720352	0.720358	FALSE	FALSE	FALSE	6wQelu	0.000322	0.450001	Vitamin A	TRUE	reported	textfile	0.003405	alcohol int	TRUE	3.73E-10	inferred	jK8tw0	textfile	2	TRUE	NA
rs7460106	C	T	C	T	-0.02171	-0.00044	0.23562	0.235601	FALSE	FALSE	FALSE	6wQelu	0.000343	0.2	Vitamin A	TRUE	reported	textfile	0.003619	alcohol int	TRUE	1.99E-09	inferred	jK8tw0	textfile	2	TRUE	NA
rs7467914	C	T	C	T	-0.03207	2.79E-05	0.074515	0.074515	FALSE	FALSE	FALSE	6wQelu	0.000545	0.96	Vitamin A	TRUE	reported	textfile	0.005758	alcohol int	TRUE	2.54E-08	inferred	jK8tw0	textfile	2	TRUE	NA
rs7514579	C	A	C	A	0.019667	-0.0005	0.232457	0.23246	FALSE	FALSE	FALSE	6wQelu	0.000341	0.14	Vitamin A	TRUE	reported	textfile	0.003598	alcohol int	TRUE	4.59E-08	inferred	jK8tw0	textfile	2	TRUE	NA
rs7608265	T	C	T	C	0.046427	0.001375	0.054327	0.054294	FALSE	FALSE	FALSE	6wQelu	0.000633	0.03	Vitamin A	TRUE	reported	textfile	0.006687	alcohol int	TRUE	3.83E-12	inferred	jK8tw0	textfile	2	TRUE	NA
rs7610856	A	C	A	C	-0.02386	-0.00025	0.429053	0.429042	FALSE	FALSE	FALSE	6wQelu	0.000291	0.4	Vitamin A	TRUE	reported	textfile	0.00307	alcohol int	TRUE	7.68E-15	inferred	jK8tw0	textfile	2	TRUE	NA
rs780094	C	T	C	T	-0.05099	0.000112	0.615206	0.615279	FALSE	FALSE	FALSE	6wQelu	0.000294	0.7	Vitamin A	TRUE	reported	textfile	0.003105	alcohol int	TRUE	1.31E-60	inferred	jK8tw0	textfile	2	TRUE	NA
rs780569	A	T	A	T	0.019803	-0.00054	0.70882	0.708819	FALSE	TRUE	FALSE	6wQelu	0.000319	0.087999	Vitamin A	TRUE	reported	textfile	0.003365	alcohol int	TRUE	3.96E-09	inferred	jK8tw0	textfile	2	TRUE	NA
rs8029231	C	T	C	T	-0.03937	-0.00068	0.057704	0.057721	FALSE	FALSE	FALSE	6wQelu	0.000615	0.27	Vitamin A	TRUE	reported	textfile	0.006496	alcohol int	TRUE	1.35E-09	inferred	jK8tw0	textfile	2	TRUE	NA
rs8043563	C	G	C	G	0.023365	0.000215	0.737192	0.737181	FALSE	TRUE	FALSE	6wQelu	0.000329	0.51	Vitamin A	TRUE	reported	textfile	0.003471	alcohol int	TRUE	1.68E-11	inferred	jK8tw0	textfile	2	TRUE	NA
rs8083110	T	C	T	C	-0.01681	0.000416	0.418707	0.418687	FALSE	FALSE	FALSE	6wQelu	0.000291	0.15	Vitamin A	TRUE	reported	textfile	0.003078	alcohol int	TRUE	4.72E-08	inferred	jK8tw0	textfile	2	TRUE	NA
rs838145	A	G	A	G	0.021955	0.000175	0.542982	0.542995	FALSE	FALSE	FALSE	6wQelu	0.000289	0.54	Vitamin A	TRUE	reported	textfile	0.003055	alcohol int	TRUE	6.70E-13	inferred	jK8tw0	textfile	2	TRUE	NA
rs8614	A	C	A	C	0.024781	-0.00031	0.182509	0.182483	FALSE	FALSE	FALSE	6wQelu	0.000371	0.41	Vitamin A	TRUE	reported	textfile	0.003925	alcohol int	TRUE	2.74E-10	inferred	jK8tw0	textfile	2	TRUE	NA
rs9349379	G	A	G	A	-0.01935	-0.00071	0.405493	0.405505	FALSE	FALSE	FALSE	6wQelu	0.000292	0.014	Vitamin A	TRUE	reported	textfile	0.003082	alcohol int	TRUE	3.46E-10	inferred	jK8tw0	textfile	2	TRUE	NA
rs9372625	A	G	A	G	-0.02556	#######	0.381706	0.381751	FALSE	FALSE	FALSE	6wQelu	0.000296	0.780001	Vitamin A	TRUE	reported	textfile	0.003125	alcohol int	TRUE	2.85E-16	inferred	jK8tw0	textfile	2	TRUE	NA
rs9403297	A	G	A	G	0.018823	-0.00015	0.372967	0.372966	FALSE	FALSE	FALSE	6wQelu	0.000296	0.62	Vitamin A	TRUE	reported	textfile	0.00313	alcohol int	TRUE	1.82E-09	inferred	jK8tw0	textfile	2	TRUE	NA
rs9648478	A	G	A	G	0.01686	#######	0.510245	0.510226	FALSE	FALSE	FALSE	6wQelu	0.000287	0.98	Vitamin A	TRUE	reported	textfile	0.003029	alcohol int	TRUE	2.60E-08	inferred	jK8tw0	textfile	2	TRUE	NA
rs9814516	T	G	T	G	-0.02511	4.15E-05	0.237423	0.237421	FALSE	FALSE	FALSE	6wQelu	0.000337	0.9	Vitamin A	TRUE	reported	textfile	0.003556	alcohol int	TRUE	1.64E-12	inferred	jK8tw0	textfile	2	TRUE	NA
rs9829192	T	G	T	G	0.016932	-0.00059	0.435133	0.435146	FALSE	FALSE	FALSE	6wQelu	0.000289	0.042	Vitamin A	TRUE	reported	textfile	0.00305	alcohol int	TRUE	2.84E-08	inferred	jK8tw0	textfile	2	TRUE	NA
rs9891943	C	T	C	T	0.019867	-0.00048	0.232109	0.23211	FALSE	FALSE	FALSE	6wQelu	0.000345	0.17	Vitamin A	TRUE	reported	textfile	0.003641	alcohol int	TRUE	4.88E-08	inferred	jK8tw0	textfile	2	TRUE	NA
rs9906502	A	G	A	G	0.023788	0.00054	0.176998	0.17705	FALSE	FALSE	FALSE	6wQelu	0.000375	0.15	Vitamin A	TRUE	reported	textfile	0.003962	alcohol int	TRUE	1.92E-09	inferred	jK8tw0	textfile	2	TRUE	NA
