# Supplementary material for: Risk of major depressive increases with increasing frequency of alcohol drinking: a bidirectional two-sample Mendelian randomization analysis
Source: Front Public Health. 2024 Jun 5;12:1372758. doi: 10.3389/fpubh.2024.1372758 (PMC11186411; doi:10.3389/fpubh.2024.1372758)
Supplement: Supplementary file 11 [file Image_1.pdf]

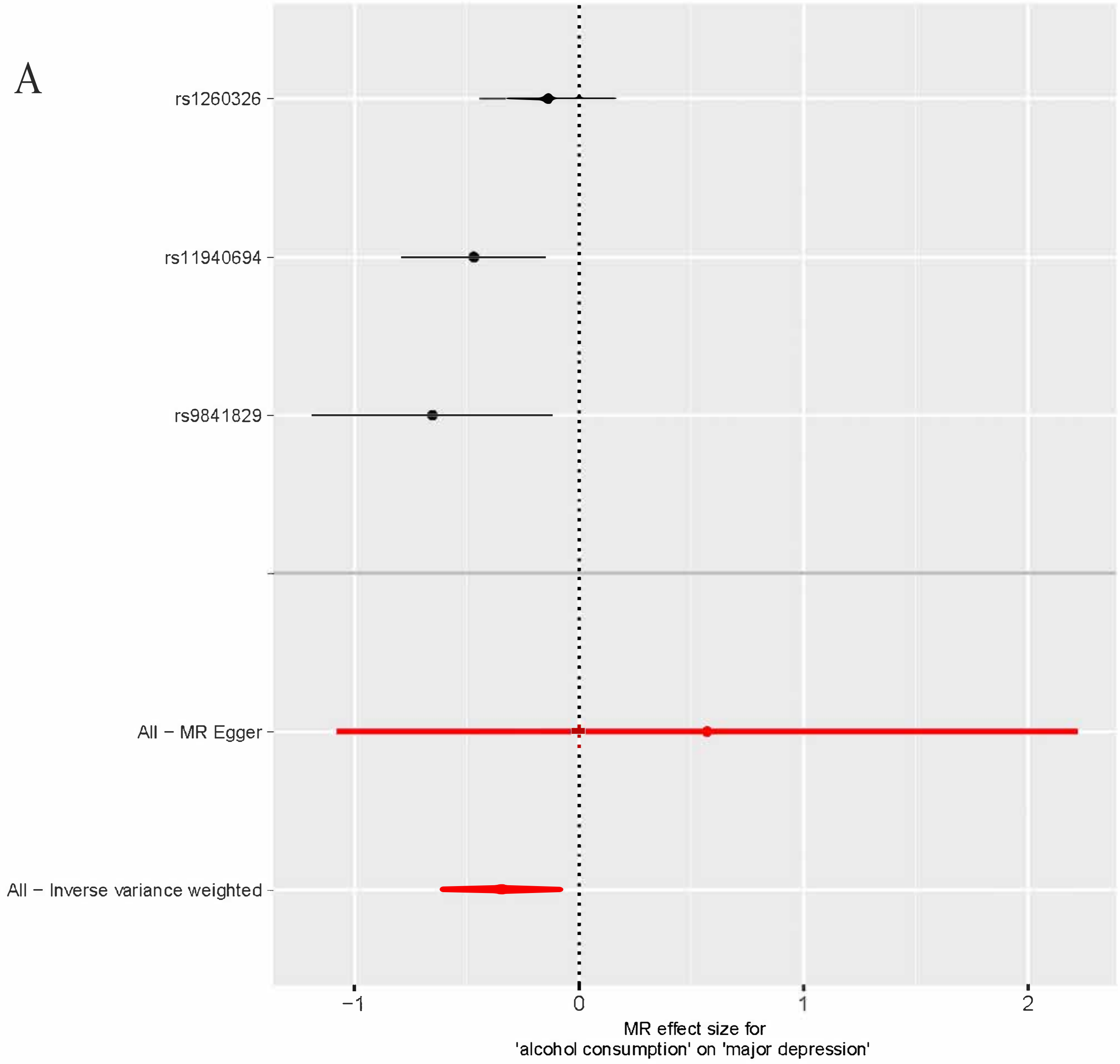
A
rs1260326
rs11940694
rs9841829
All − MR Egger
All − Inverse variance weighted
−1
0
1
2
MR effect size for
'alcohol consumption' on 'major depression'

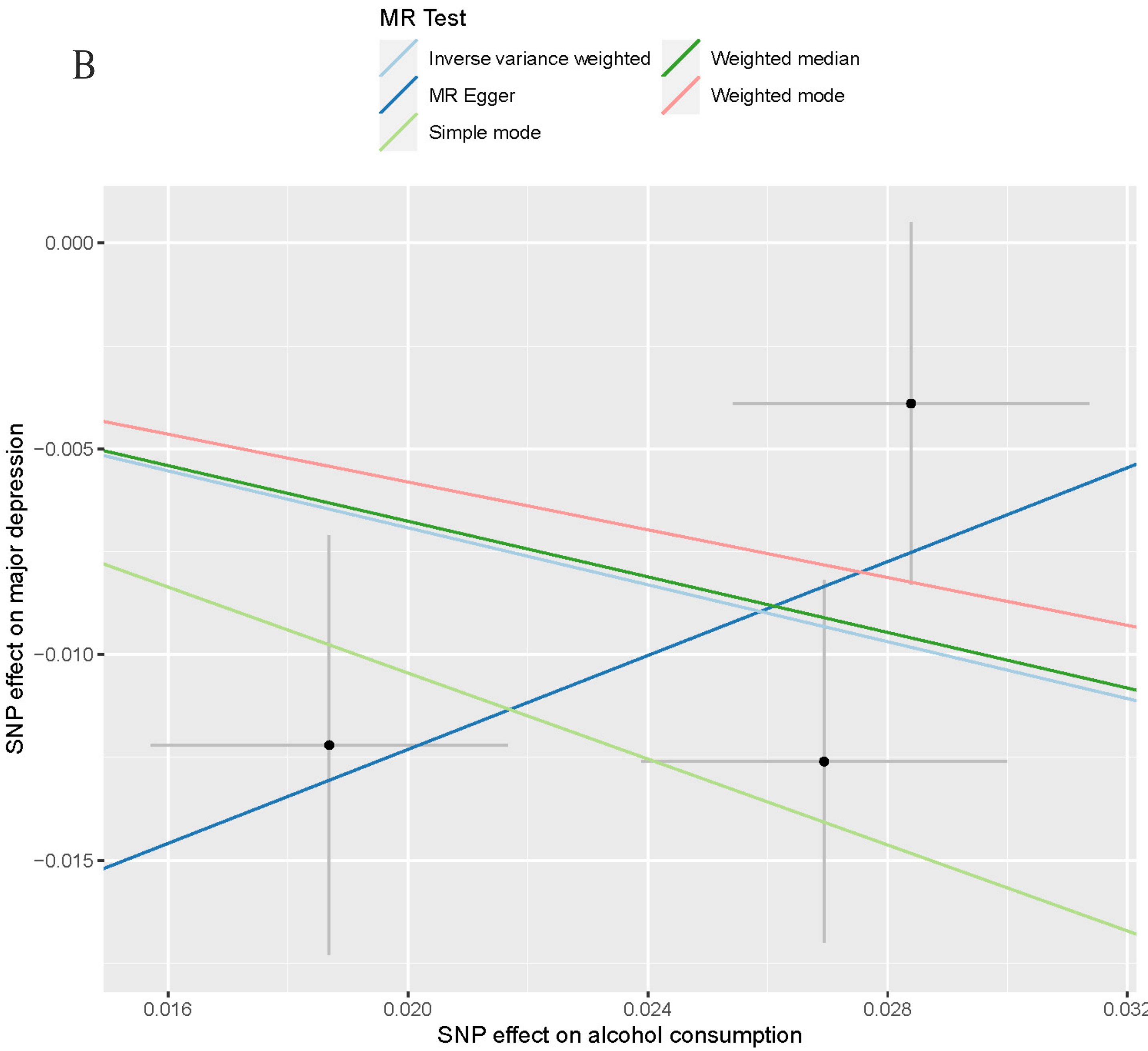
B
MR Test
Inverse variance weighted
MR Egger
Simple mode
Weighted median
Weighted mode
0.000
−0.005
−0.010
−0.015
SNP effect on major depression
0.016
0.020
0.024
0.028
0.032
SNP effect on alcohol consumption

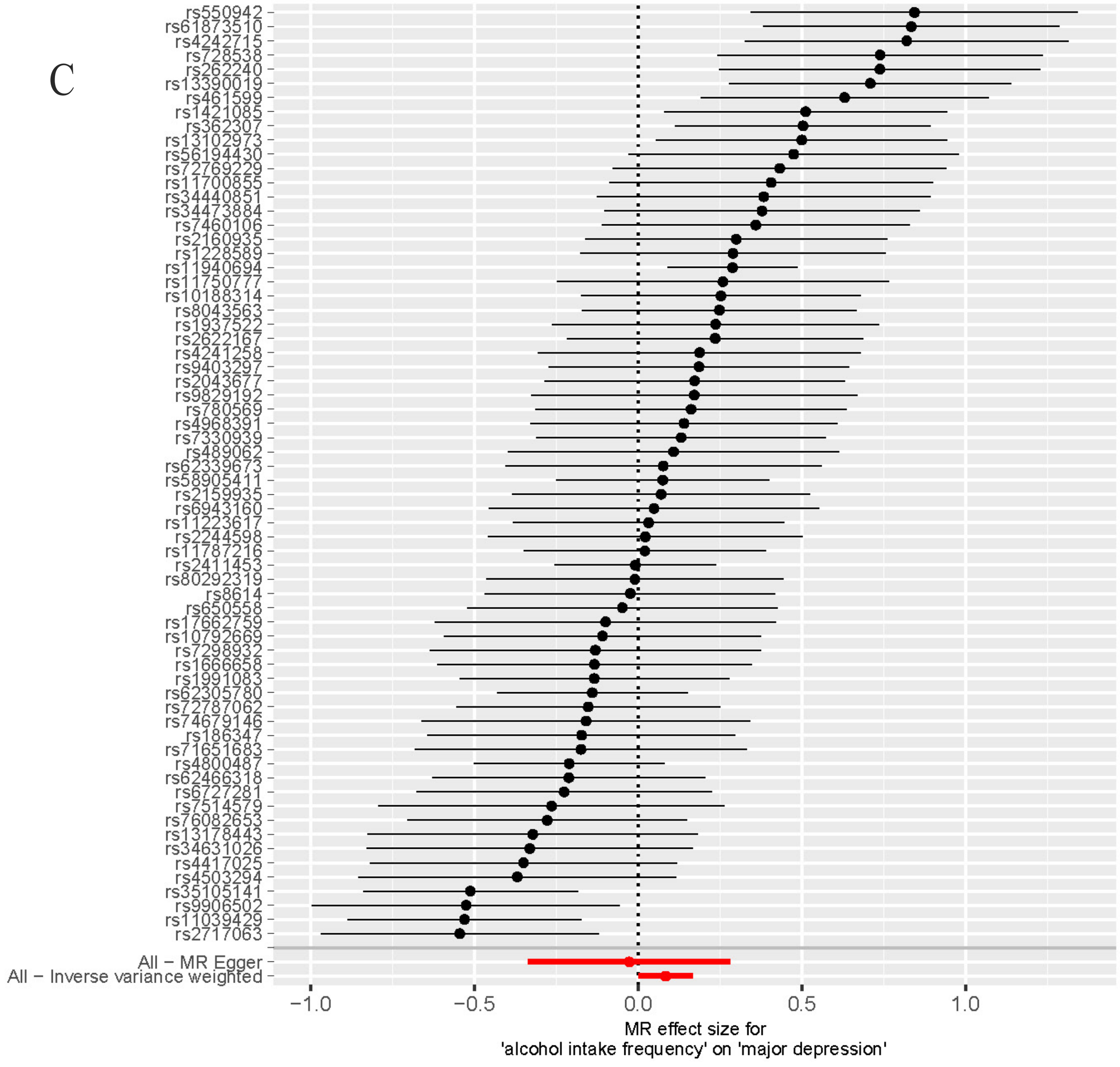
C
rs550942
rs61873510
rs4242715
rs728538
rs262240
rs13390019
rs461599
rs1421085
rs362307
rs13102973
rs56194430
rs72769229
rs11700855
rs34440851
rs34473884
rs7460106
rs2160935
rs1228589
rs11940694
rs11750777
rs10188314
rs8043563
rs1937522
rs2622167
rs4241258
rs9403297
rs2043677
rs9829192
rs780569
rs4968391
rs7330939
rs489062
rs62339673
rs58905411
rs2159935
rs6943160
rs11223617
rs2244598
rs11787216
rs2411453
rs80292319
rs8614
rs650558
rs17662759
rs10792669
rs7298932
rs1666658
rs1991083
rs62305780
rs72787062
rs74679146
rs186347
rs71651683
rs4800487
rs62466318
rs6727281
rs7514579
rs76082653
rs13178443
rs34631026
rs4417025
rs4503294
rs35105141
rs9906502
rs11039429
rs2717063
All − MR Egger
All − Inverse variance weighted
−1.0
−0.5
0.0
0.5
1.0
MR effect size for
'alcohol intake frequency' on 'major depression'

D

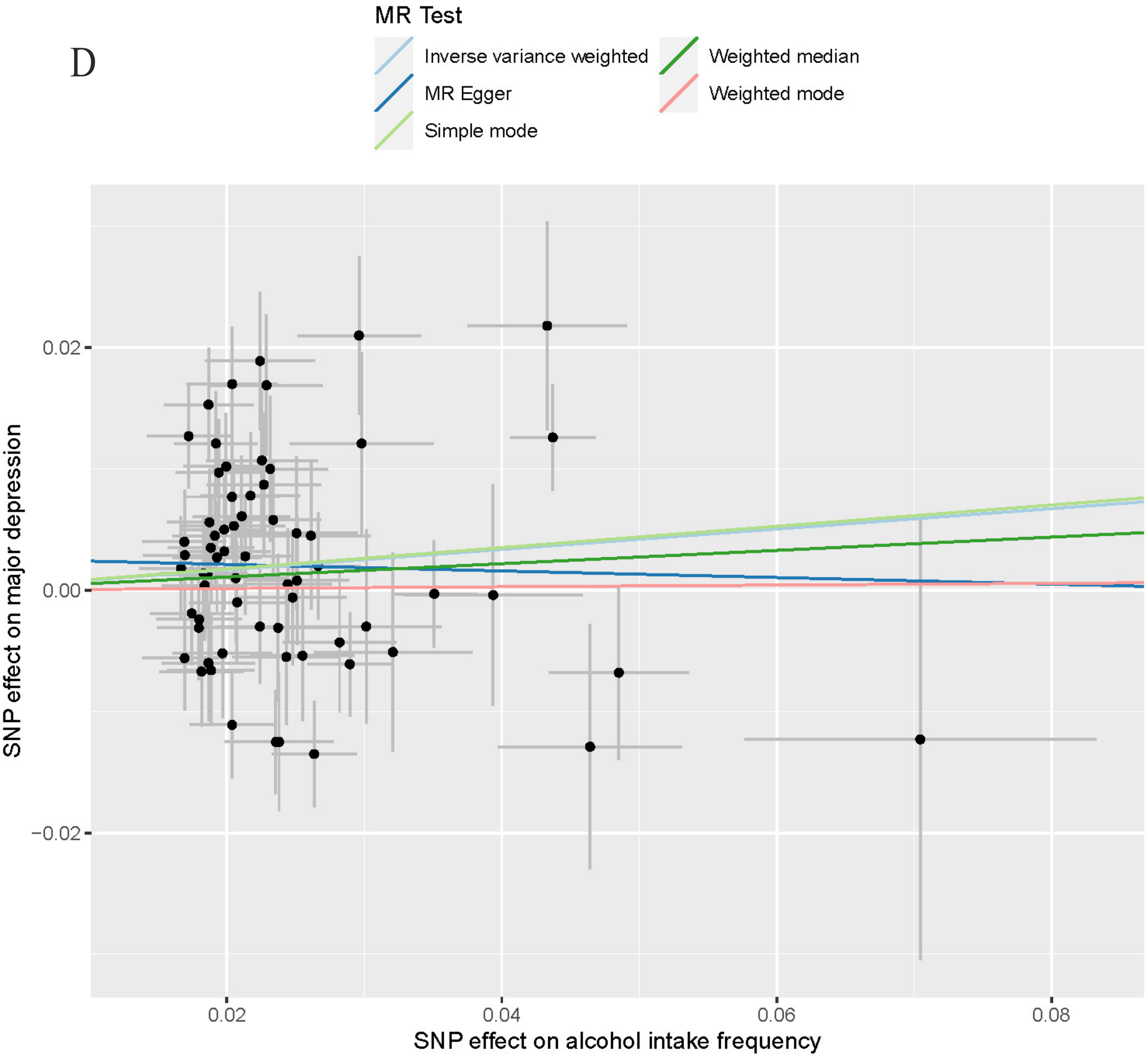
MR Test
Inverse variance weighted
MR Egger
Simple mode
Weighted median
Weighted mode
0.02
0.00
−0.02
SNP effect on major depression
0.02
0.04
0.06
0.08
SNP effect on alcohol intake frequency

Supplementary Fig 1.MR results for the relationship between alcohol and major depression. **A**, forest plot of individual and combined SNP MR-estimated effect sizes.The effect estimates represent the log odds for major depression increase in alcohol consumption, and the error bars represent 95% CIs. **B**, scatter plot of SNP effects on relative alcohol consumption versus major depression, with the slope of each line corresponding to the estimated MR effect per method. The data are expressed as raw β values with 95% CIs. **C**, forest plot of individual and combined SNP MR-estimated effect sizes ,that is alcohol intake frequency and major depression. **D**, scatter plot of SNP effects on relative alcohol intake frequency versus major depression.
